# Total Synthesis of the SJG‑2 Glycan: General Strategies for Constructing Sterically Congested Sialylated Glycans

**DOI:** 10.1021/jacs.6c00368

**Published:** 2026-03-17

**Authors:** Yung-Yu Su, Avijit K. Adak, Yan-Ting Kuo, Hong-Jyune Lin, Ya-Syuan Li, Hsin-Kai Tseng, Chun-Cheng Lin

**Affiliations:** † Department of Chemistry, 34881 National Tsing Hua University, Hsinchu 300044, Taiwan; ‡ Department of Medicinal and Applied Chemistry, Kaohsiung Medical University, Kaohsiung 807378, Taiwan

## Abstract

Gangliosides are a diverse class of glycosphingolipids whose sialylated glycans regulate processes ranging from neuronal repair to immune responses. Among them, SJG-2, an echinoderm-derived ganglioside with potent neuroregenerative activity, stands out for its rare and highly branched trisialylated glycan motif. The unusual architecture of the heptasaccharide SJG-2 glycan with two terminal sialic acids attached to galactose (Gal) via α­(2,3)- and α­(2,4)-sialylations and an additional internal β­(1,8)-sialylation to another sialic acid has rendered it synthetically inaccessible for over two decades. Here, we report the first total synthesis of the SJG-2 glycan. A conformational inversion strategy using a ^1^C_4_-configured Gal acceptor enabled efficient α­(2,4)- and α­(2,3)-sialylations. In the key convergent [4 + 3] glycosylation, selective *N*-acetyl,5-*N*,4-O-carbonyl protection of sialic acid suppressed competing C8 reactivity, while subsequent deacetylation proved essential for forming the β­(1,8)-glycosidic bond. This work achieves a long-standing synthetic goal and establishes broadly applicable strategies for constructing sterically congested sialylated glycans, opening new opportunities to access complex gangliosides and explore their neurobiological functions.

## Graphical abstract

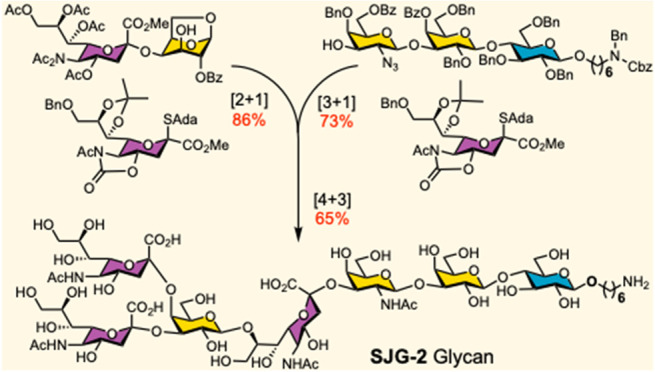

## Introduction

Glycosphingolipids are amphipathic biomolecules that predominantly reside on the extracellular surface of the plasma membrane and participate in diverse biological processes such as immune regulation, cell–cell communication, and neural development.^1,2^ Within this family, gangliosides are distinguished by their sialic acid residues and are directly implicated in both physiological and pathological contexts, including neurodegenerative diseases such as Parkinson’s disease and Alzheimer’s disease.^3–5^ Despite their importance, the stereoselectivity associated with sialylation, especially in building multiple, differently positioned sialic acid residues, have made the synthesis of structurally defined gangliosides a long-standing challenge in synthetic glycochemistry and a central goal for developing chemical probes and therapeutic agents.^6–9^

Among these, echinoderm-derived gangliosides (EGs) represent a structurally distinctive subgroup.^10^ Comparative studies have shown that several EGs, including LLG-5, LLG-3, GAA-7, and especially SJG-2, exhibit enhanced neuroregenerative activity in PC12 cells in the presence of nerve growth factor (NGF) compared to the classical GM1.^11^ These findings highlight their therapeutic potential as leads for neuroregenerative drug development and have motivated synthetic efforts toward representative EGs such as GAA-7,^12–14^ LLG-5,^15^ and LLG-3.^16–18^ However, despite its superior activity, SJG-2 has remained chemically inaccessible since its isolation and structural elucidation in 2003, largely due to its unusually complex architecture.^19^ The unique structure of SJG-2 (Figure 1A), which features two terminal sialic acids attached to a central galactose (Gal) core through α­(2,4)- and α­(2,3)-linkages and an internal β­(1,8)-linked sialic acid, represents one of the most sterically congested and stereochemically demanding motifs encountered in natural glycan synthesis.

**Figure 1:**
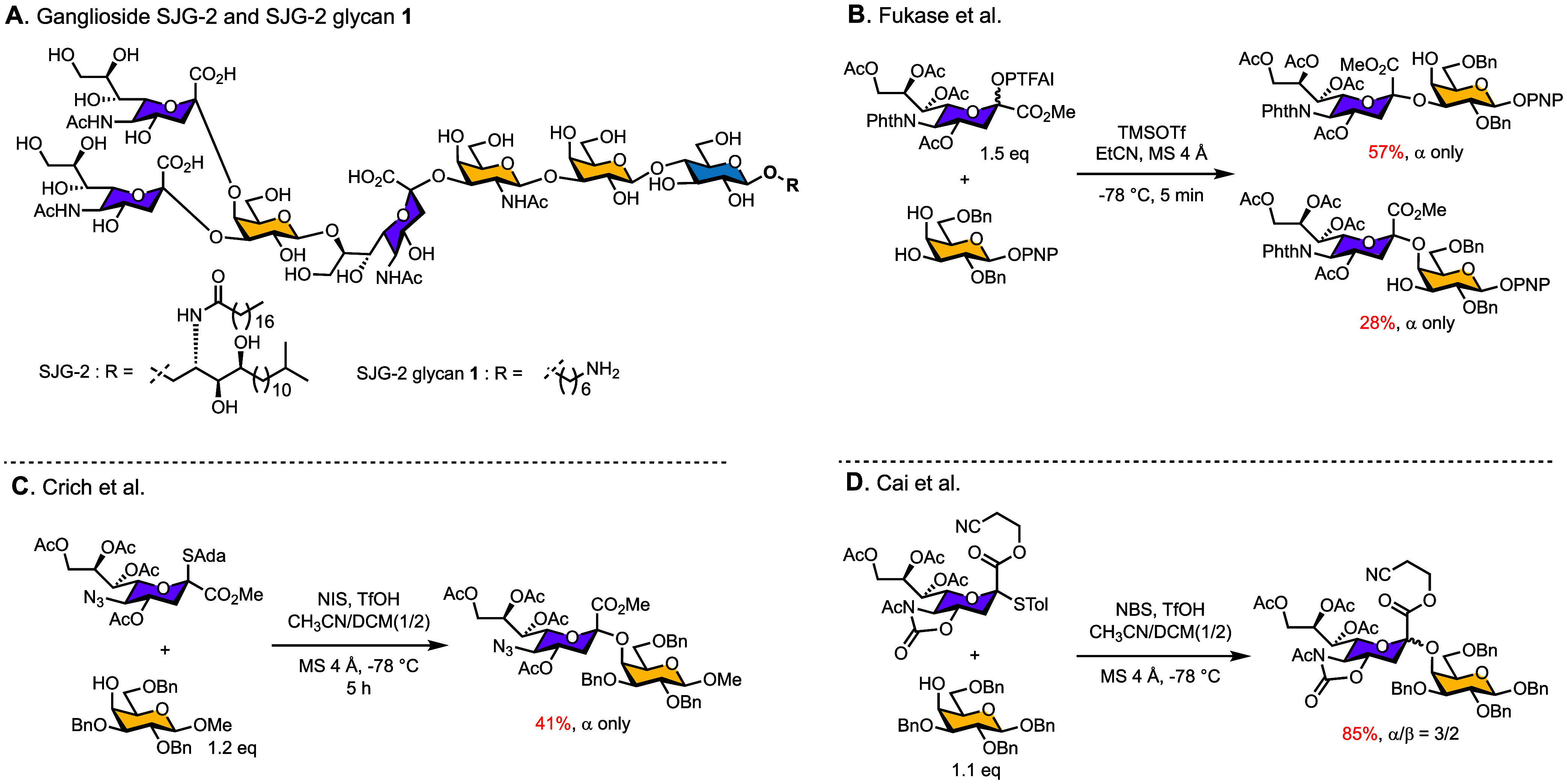
(A) Structure of the SJG-2 glycan **1**. (B–D) Literature strategies for the formation of Neu5Ac-α­(2,4)-Gal glycosidic bonds.

Sialic acid-containing glycans have long been pursued as synthetic targets in glycochemistry because of their central roles in glycobiology.^20–22^ Among the factors governing sialylation efficiency, the nature of the anomeric leaving group is particularly critical.^23^ Conventional halide and thioglycoside donors are widely used,^24^ while more reactive alternatives, including adamantyl thioethers (SAda),^25^ phosphate,^26^ and *N*-phenyltrifluoroacetimidates (PTFAI)^27^ have been developed to enhance glycosylation yields. Furthermore, protecting group effects on sialic acid donors have been systematically explored to improve both reactivity and stereoselectivity. Protecting groups, such as Troc,^28,29^ N_3_,^30^ and 5-*N*,4-*O*-oxazolidinone,^25,31,32^ have shown good performance, though their effectiveness often depends strongly on the structural features of the acceptors.

The synthetic challenges of SJG-2 are rooted in two rare and sterically demanding linkages. First, the α­(2,4)-sialoside, a notoriously difficult motif to construct due to the poor nucleophilicity of the axial C4-OH group of Gal.^33^ Previous studies illustrate this difficulty: Fukase and co-workers obtained only a minor disaccharide product in 28% yield using an *N*-phthaloyl-protected donor (Figure 1B);^34^ Crich’s group achieved 41% yield with an N_3_-protected donor carrying an SAda leaving group (Figure 1C);^35^ and more recently, Cai and co-workers employed a 5-*N*,4-*O*-oxazolidinone donor to promote C4 sialylation, albeit with the formation of mixed products (Figure 1D).^36^ These limited studies indicate both the inherent difficulty of forming this linkage and the importance of developing viable strategies for α­(2,4)-sialylation. Second, SJG-2 contains a rare Gal-β­(1,8)-Neu5Ac linkage. Unlike α­(2,8)^31,37–39^ or α­(2,9)^40,41^ sialic acid extensions, which have been extensively investigated, efficient strategies for constructing Gal-β­(1,8)-Neu5Ac linkage remain scarcely documented. This bond represents one of the most formidable obstacles in the total synthesis of SJG-2. Together, these barriers highlight the necessity of integrating donor design, conformational control, and protecting-group engineering to achieve the reactivity and selectivity required for the synthesis of SJG-2.

In this report, we present the first total synthesis of the SJG-2 glycan **1**, a long-elusive target that has remained inaccessible to chemical synthesis for over two decades. The synthesis was accomplished via a conformational inversion strategy that enabled efficient installation of the α­(2,4)- and α­(2,3)-sialyl linkages at the nonreducing end Gal (Scheme 1). A silyl ether-free protecting group design enhanced acid stability during assembly of the reducing-end tetrasaccharide (Scheme 4). Furthermore, *N*-acetylation of the 5-*N*,4-*O*-oxazolidinone-protected sialic acid unit attenuated the reactivity of the C8 hydroxyl group in the acceptor. Subsequent deacetylation restored C8-OH nucleophilicity and enabled the key convergent [4 + 3] glycosylation. Collectively, these design elements overcome the synthetic barriers associated with constructing Neu5Ac-α­(2,4)-Gal and Gal-β­(1,8)-Neu5Ac linkages, allowing access to this structurally rare trisialylated ganglioside. This achievement provides a practical route to sterically congested sialylated glycans and also establishes a general framework for synthesizing complex gangliosides for structure–activity investigations and biological studies.

**Scheme 1:**
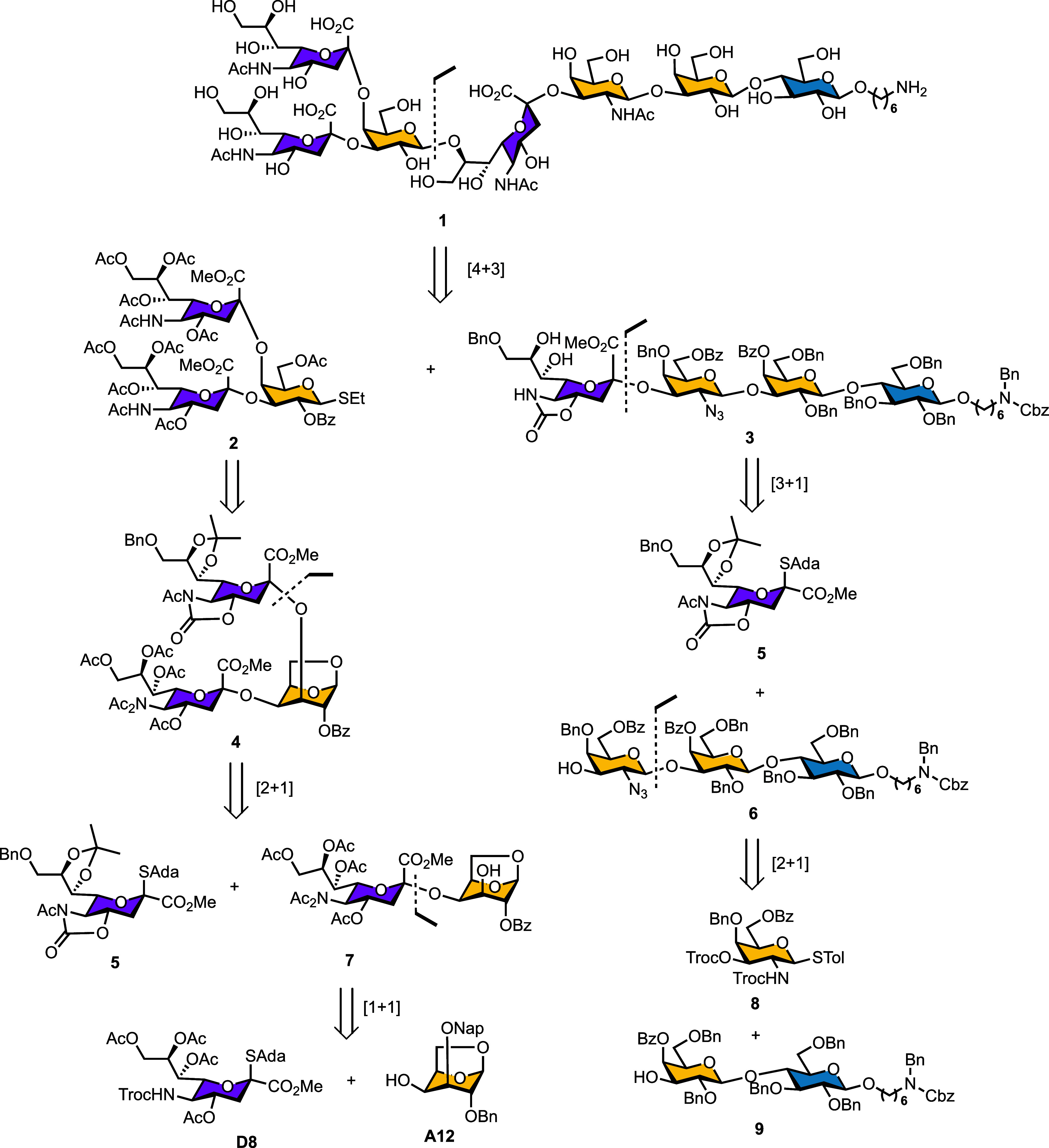
Retrosynthetic Analysis of SJG-2 **1**

## Results and Discussion

### Retrosynthetic Analysis of SJG-2 Glycan 1

To establish an efficient route toward SJG-2, we first analyzed its structure to identify strategic disconnections and feasible synthetic fragments. The target heptasaccharide SJG-2 (**1**) was retrosynthetically divided into a nonreducing-end trisaccharide (**2**) and a reducing-end tetrasaccharide (**3**) (Scheme 1). This convergent strategy allowed the key [4 + 3] glycosylation to construct the crucial β­(1,8)-linkage connecting the two fragments. The nonreducing end trisaccharide donor (**2**) was derived from a ^1^C_4_-configured anhydro-Gal building block, which facilitated stereocontrolled installation of two sialic acid residues. Sequential α­(2,4)- and α­(2,3)-sialylations were carried out using donors **D8** and **5**, respectively, to construct the branched sialylated Gal core. For the reducing-end tetrasaccharide (**3**), a stepwise assembly was adopted, beginning from galactosamine donor (**8**) and lactose acceptor (**9**) to afford trisaccharide intermediate (**6**) via [2 + 1] glycosylation. Subsequent sialylation of **6** with donor **5** completed the construction of fragment **3**. This design provided a convergent and flexible synthetic route to SJG-2, allowing modular access to both fragments and efficient assembly through the final [4 + 3] glycosylation.

### Synthesis of Nonreducing-End Disialyl Trisaccharide Donor Precursors: Investigation of ^4^C_1_–Gal Acceptors

To construct the nonreducing-end trisaccharide fragment **2** necessitates selective installation of two sialic acids onto a Gal acceptor. We first examined α­(2,4)-sialylation at the C4-OH of Gal acceptors (Tables 1 and S1). Using disaccharide acceptor **A1** (entries 1–2) exclusively produced β-anomers, regardless of the sialyl donor used. Notably, these sialyl donors carried a 5-*N*,4-*O*-oxazolidinone group, which typically promotes α-selectivity.^36^ Changing the leaving group improved the yield from 28% to 82% (entries 1–6) but did not affect stereoselectivity. The reduced α-selectivity observed for cyclic Gal acceptors is consistent with donor–acceptor mismatch in sialylation reactions, in which steric hindrance and diminished nucleophilicity of the axial C4-OH weaken the kinetic preference for α-attack even when strongly α-directing donors are employed. Similar behavior has been documented in related sialylation systems, including oxazolidinone-protected donors and sterically hindered acceptors.^25,34–36^ Accordingly, the axial C4-OH of the Gal acceptor likely limits the stereodirecting influence of the 5-*N*,4-*O*-oxazolidinone-protected donors under these conditions. These findings indicate that conventional α-directing donors cannot be reliably applied to sterically congested Gal acceptors, requiring alternative methods in constructing such linkages.

**Table 1:**
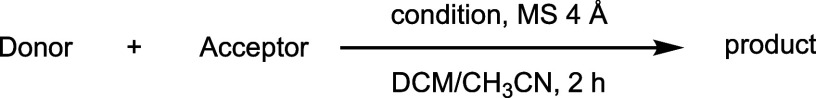

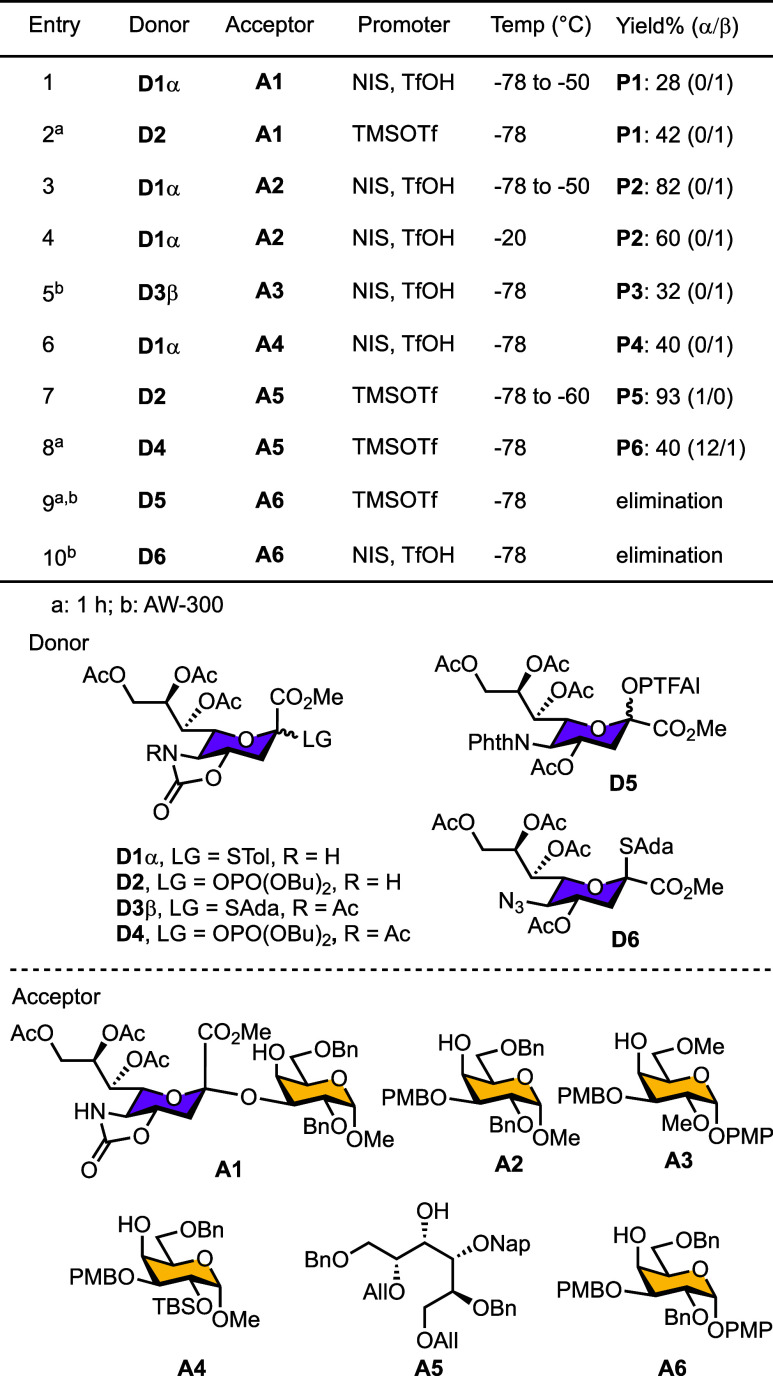
Evaluation of α­(2,4)-Sialylation between Various Sialyl Donors and ^4^C_1_–Gal Acceptors (The Structures of **P1–P6** are Shown in Figure S1)

To further identify the influences of protecting groups on the acceptor, we evaluated a series of monosaccharide acceptors, namely, **A2**, **A3**, and **A4**, which contain PMB/Bn, Me, and TBS substituents, respectively. Although prior studies reported increased reactivity for **A4**,^42,43^ only β-anomers were observed in all cases (entries 3–6). Interestingly, when the open-ring galactitol acceptor **A5** was employed, donor **D2** furnished the desired α­(2,4)-linked product **P5** in high yield and exclusive stereoselectivity (93%, α-only; entry 7). This outcome is probably due to increased conformational flexibility and reduced steric congestion around the reacting hydroxyl group, which likely facilitates nucleophilic approach and restores α-selectivity. In contrast, donor **D4** underwent rapid hydrolysis, reducing the yield to 40% (entry 8). Attempts to regenerate the cyclic Gal structure from **P5** by allyl deprotection followed by selective oxidation of the primary hydroxy group were unsuccessful, preventing its use in downstream synthesis.

We also tested previously reported sialyl donors known to produce α­(2,4)-linkages with galactosyl acceptors. However, neither Fukase’s donor **D5** ^34^ (entry 9) nor Crich’s donor **D6** ^35^ (entry 10) afforded the desired product under our reaction conditions, instead yielding elimination side products. Comprehensive screening data are summarized in the Supporting Information (Table S1), and the product structures (**P**) are shown in Figure S1. The anomeric configuration of the sialic acid residues was confirmed by analyzing heteronuclear ^3^ *J*_C1,H3ax_ coupling constants. These were measured using either ^13^C-coupled NMR or EXSIDE experiments, depending on the compounds.^44^ In selected cases, HMBC spectra were also used to confirm the C_1_–H_3ax_ corelation, providing additional support for the configuration assignment.^45^

### Synthesis of Disialyl Trisaccharide Donor Precursors Using ^1^C_4_–Gal Acceptor

The results summarized in Table 1 indicated that native ^4^C_1_-configured Gal acceptors were incompatible with α­(2,4)-sialylation. To overcome this limitation, we hypothesized that conformational inversion of the Gal acceptor into the ^1^C_4_ form, repositioning the C4-OH from axial to equatorial orientation, would increase nucleophilicity and thereby improve sialylation efficiency. Initial experiments using diol acceptors **A7** and **A8** (entries 1 and 2, Table 2) produced only monosialyted products when donor **D2** or **D5** were used, however, in both cases, the desired α­(2,4)-glycosidic linkage was obtained. These results confirmed that ^1^C_4_-configured Gal acceptors are viable substrates for stereoselective α-sialylation.

**Table 2:**
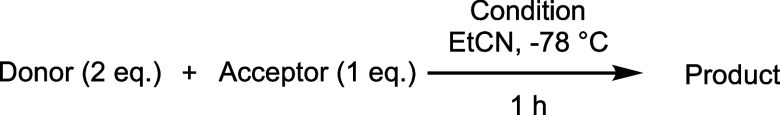

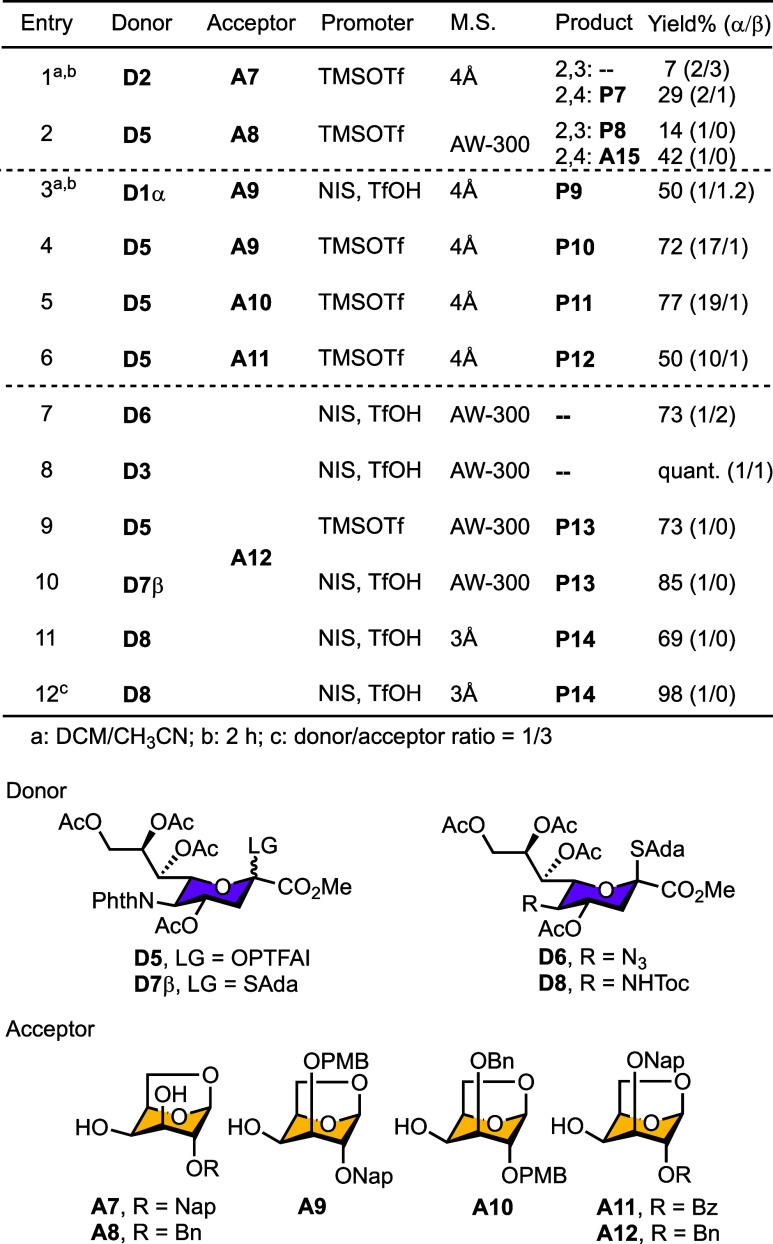
Evaluation of α­(2,4)-Sialylation with ^1^C_4_–Gal Acceptors (The Structures of **P7**–**P14** are Shown in Figure S1)

We next examined the role of protecting groups at the C3 and C2 positions of the Gal acceptor (**A9**-**A12**). Donor **D5** consistently gave higher α selectivity and yield than donor **D1** (entries 3 and 4). Moreover, the nature of the C2 protecting group strongly influenced reactivity: ether-type substituents enhanced yields, whereas benzoyl esters significantly decreased the yields (entries 5 and 9 vs 6). Notably, orthogonally protected **A12** was identified as a practical acceptor, suitable for downstream transformation.

A broader evaluation of sialyl donors (**D3** and **D5**-**D8**), differing in their C5-*N* protecting groups and anomeric leaving groups, further clarified donor performance. Donors bearing an *N*-phthalimido (Phth) protection at C5–N (**D5** and **D7**) performed better than those containing N_3_ (**D6**) or a 5-*N*,4-*O*-oxazolidinone (**D3**) (entries 7–9). Within this group, the SAda-leaving group (**D7**) outperformed its PTFAI counterparts (**D5**), while donor **D8** (*N*-Troc) consistently promoted efficient α­(2,4)-glycosylation with excellent α-selectivity across a range of donor-to-acceptor ratios (entries 11 and 12). Additional extended screening data are summarized in the Supporting Information (Table S2). Presumably, the superior performance of the N-Troc-protected donor (**D8**) is attributed to its strong electron-withdrawing character,^28^ which modulates oxocarbenium ion reactivity and promotes formation of a more ordered nitrilium-oxocarbenium ion pair in nitrile solvent,^46,47^ thereby favoring α-glycosylation. In contrast, C2-benzoylated Gal acceptor (**A11**) displayed diminished reactivity. The electron-withdrawing benzoyl substituent further decreases the nucleophilicity of the intrinsically weak axial C4-OH nucleophile, resulting in reduced conversion without improved stereocontrol. Collectively, these results demonstrate that conformational inversion to ^1^C_4_–Gal, in combination with judicious donor and protecting-group selection, enables efficient and stereoselective α­(2,4)-sialylation. This strategy ultimately provided the key disialyl trisaccharide donor precursors required for the assembly of SJG-2.

### Synthesis of Nonreducing-End Disialyl Trisaccharide Donor Precursors

After successfully establishing the α­(2,4)-glycosidic linkage, we next investigated the α­(2,3)-sialylation required to construct the trisaccharide donor (Table 3). To facilitate this transformation, the acceptor conformation was reverted to the ^4^C_1_ form (**A13**) to position the C3-OH group equatorial, thereby enhancing its reactivity. The syntheses of acceptors **A13** and **A14** (Table 3) are described in the Supporting Information.

**Table 3:**
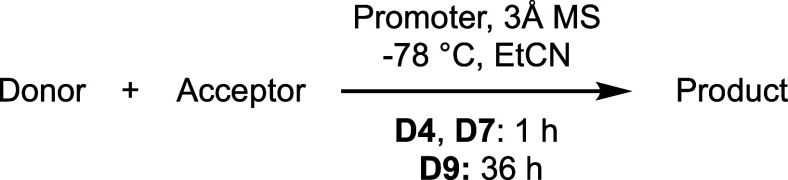

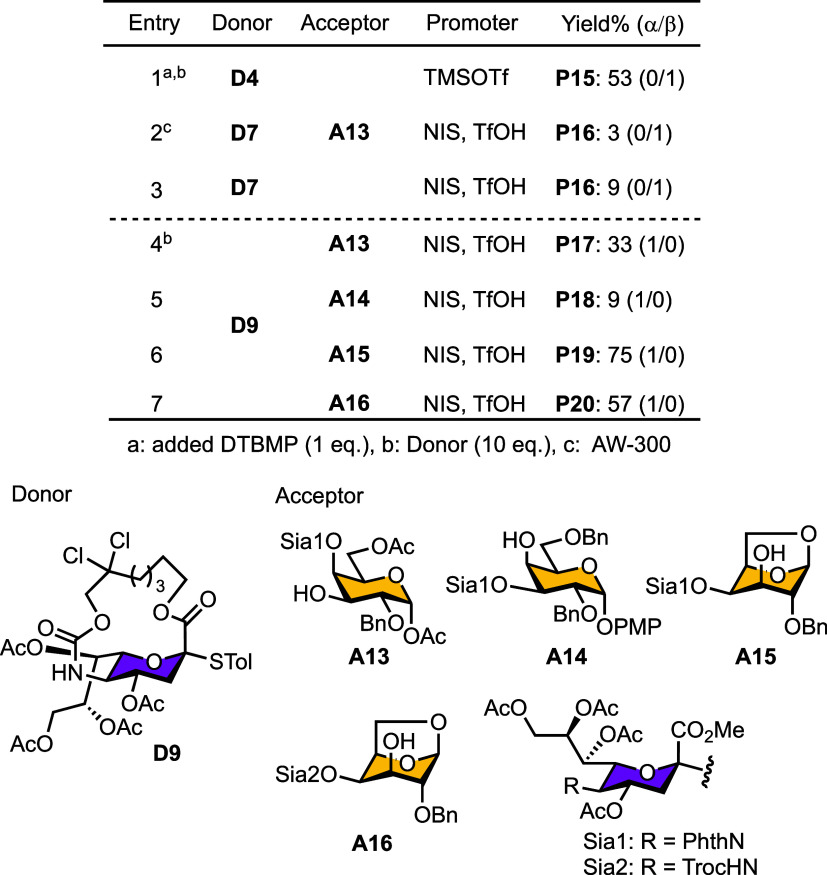
Evaluation of α­(2,3)-Sialylation with Various Donors and Acceptors (The Structures of **P15–P20** are Shown in Figure S1)

Sialylation of the ^4^C_1_-configured acceptor **A13** using either the highly α-selective donor **D7** or the commonly employed donor **D4** afforded trisaccharide products exclusively as β-anomers. Even with Ando’s donor **D9**,^48^ the desired α-configured disialylated trisaccharide was obtained only in modest yield (33%, **P17**, entry 4), and related ^4^C_1_-configured acceptor bearing neighboring sialyl substituent (entry 5) also gave poor sialylation yields.

Notably, the axial C3-OH of ^1^C_4_-configured acceptors **A15** and **A16** exhibited markedly higher reactivity in the second sialylation, producing the target trisaccharides **P19** and **P20** in 75% and 57% yields, respectively (entries 6 and 7). This comparison indicates that the ^1^C_4_ conformation is more favorable for C3 sialylation under these conditions. Additional donor screening (Table S3) (phthalimido donors with various leaving groups and oxazolidinone-protected donors with SAda or OPO­(OBu)_2_ leaving groups) primarily resulted in elimination byproducts rather than productive glycosylation. Furthermore, attempts to employ intramolecular glycosylation strategies were also unsuccessful (Table S4).

We next attempted to manipulate the protecting groups on the sialic acid moieties of compounds **P19** and **P20** (Scheme 2), which produced divergent outcomes. Despite extensive efforts, **P19** could not be converted into the desired compound **10**, whereas **P20** was successfully transformed into compound **11** after replacing the NHTroc and its constrained substituent with an NHAc group. This discrepancy was attributed to partial hydrolysis of the phthalimido group in **P19**, as evidenced by the disappearance of Phth signals in the ^1^H NMR spectrum and increased polarity during chromatography. To enhance β-selectivity in the forthcoming [4 + 3] glycosylation, a benzoyl group was introduced at the Gal C2-OH position. However, direct debenzylation of **11** followed by benzoylation proved unsuccessful: the liberated hydroxyl was poorly nucleophilic and failed to undergo benzoylation under standard conditions with either Bz_2_O or BzCl at room temperature. Heating the reaction mixture promoted benzoylation at Gal-C2, but concomitant acylation of the sialic acid C5-NHAc also occurred, yielding an undesired *N*-benzoylacetamide (NBzAc) byproduct (Scheme S1). To circumvent this issue, compound **11** was first diacetylated to produce **12** (Scheme 2). Subsequent hydrogenation followed by selective benzoylation at Gal C2 gave compound **13** in 74% yield over two steps. Further transformations involved ring opening of the anhydro-Gal unit under Lewis acid/acetic anhydride conditions, followed by selective C1 deprotection with hydrazine acetate to yield the desired glycosyl donor precursor **14** in 91% overall yield (two steps). Notably, the diacetylamino group (NAc_2_) exhibited limited stability under the final deprotection conditions, undergoing partial hydrolysis to regenerate the NHAc group. With the nonreducing-end fragment secured, we then focused on the synthesis of the reducing-end tetrasaccharide, which posed distinct stereoelectronic and protecting-group challenges.

**Scheme 2:**
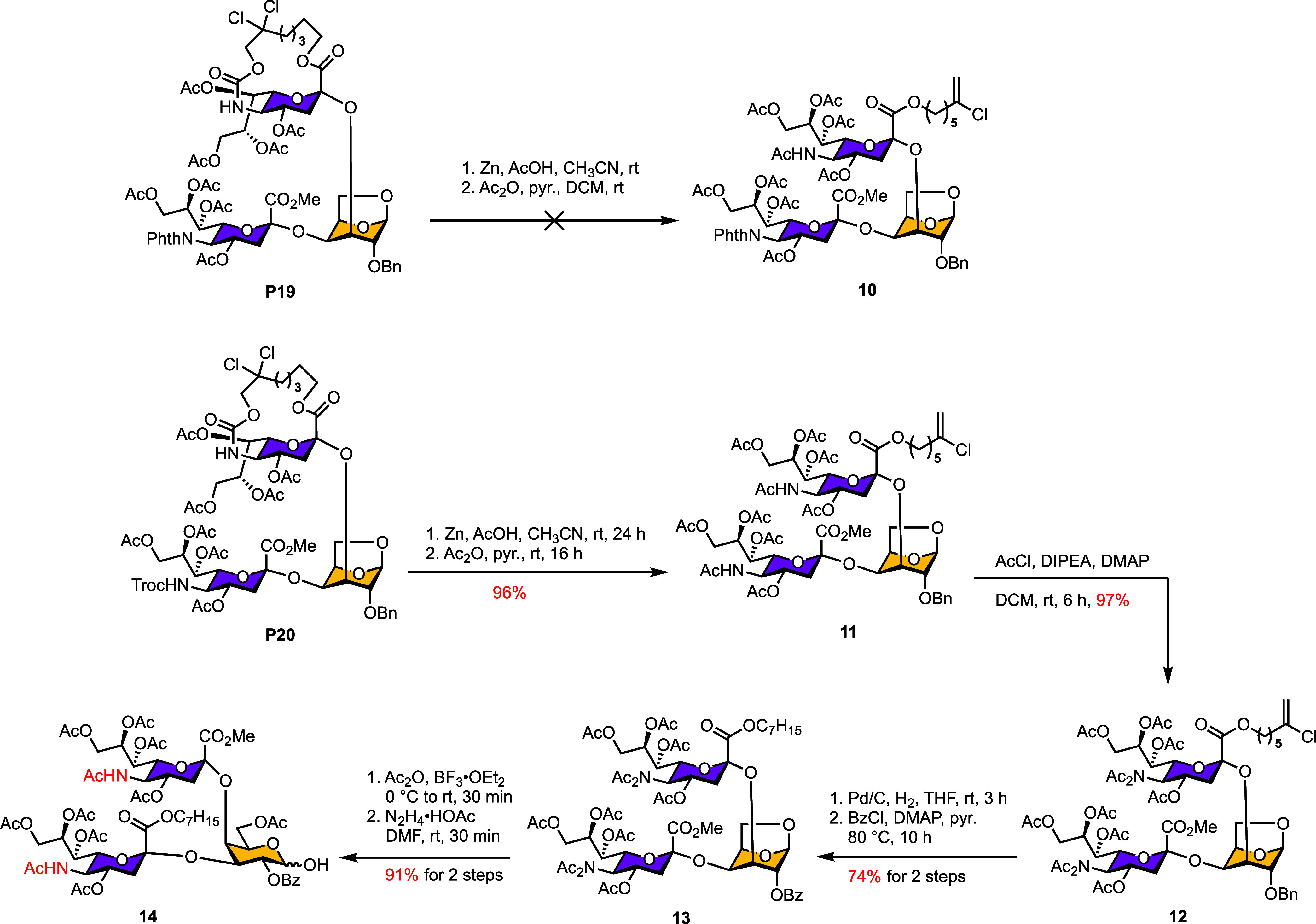
Strategic Modification of Trisaccharide **P20** to Access Glycosyl Donor Precursor **14** for [4 + 3] Glycosylation

### Synthesis of Reducing-End Tetrasaccharide Acceptors

The next key objective was to construct the rare Neu5Ac-α­(2,3)-GalNAc linkage. Because this motif is uncommon among natural glycans, it remains relatively underexplored, with few precedents available.^49–52^ We therefore evaluated how the protecting groups on galactosamine acceptor and the leaving groups of sialyl donors influence both the efficiency and stereoselectivity of α-sialylation. In addition, since the downstream chain extension required formation of a Gal-β­(1,8)-Neu5Ac bond, the C8 protecting group on the sialic acid donor needed to be orthogonally compatible, prompting us to carefully examine its impact on α-sialylation and subsequent transformations.

Initial efforts to build the reducing-end tetrasaccharide from the sialic acid terminus were unsuccessful (Table S5). Sialylation of a galactosamine acceptor bearing an NHTroc group led only to elimination byproducts (Table S5, entries 1–10). Substituting the Troc group with an azide (S14 and S15 in Table S5) enabled successful coupling with sialyl donor **D5** (entries 11 and 12 of Table S5), consistent with previous reports.^52^ However, α­(2,3)-sialylation using orthogonally protected donors (S7 and S10) failed under all tested conditions (entries 13 and 14 of Table S5). Inspired by the finding that reliable α­(2,3)-sialylation could be achieved only in a more favorable donor–acceptor combination, we shifted the synthetic strategy to construct the glycan sequence from the reducing end using lactose-derived acceptor.

To evaluate donor and acceptor effects, a series of trisaccharide acceptors were synthesized from lactose and galactosamine derivatives bearing varied protecting groups and subsequently converted to the corresponding azides **15**–**18** (see Scheme S2 for the detailed syntheses). When trisaccharide acceptor **15** (bearing a benzylidene acetal) was glycosylated with sialyl donor **19**, the α-anomer was obtained in 55% yield (Table 4, entry 1). By contrast, electron-withdrawing benzoyl protection on acceptor **16** significantly reduced reactivity and selectivity (entry 2). The electron-donating benzyl-protected acceptor **17** increased yield to 85%, though α-selectivity was lost (entries 3 vs 1). Switching to a chloroacetyl (ClAc)-protected donor **20** enhanced reactivity but gave only elimination products regardless of the acceptor used (entries 4–6).

**Table 4:**
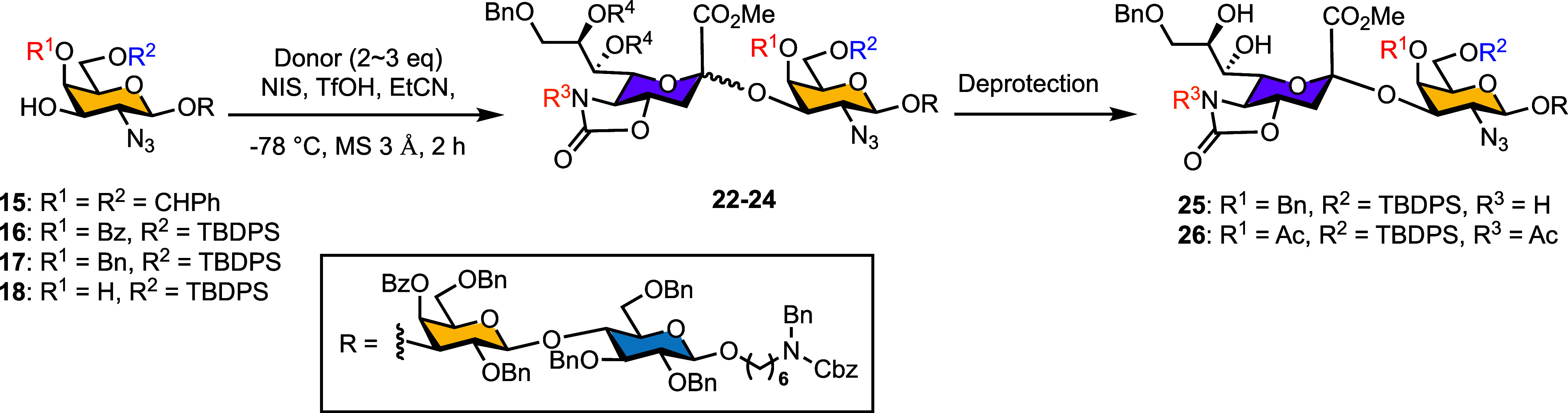

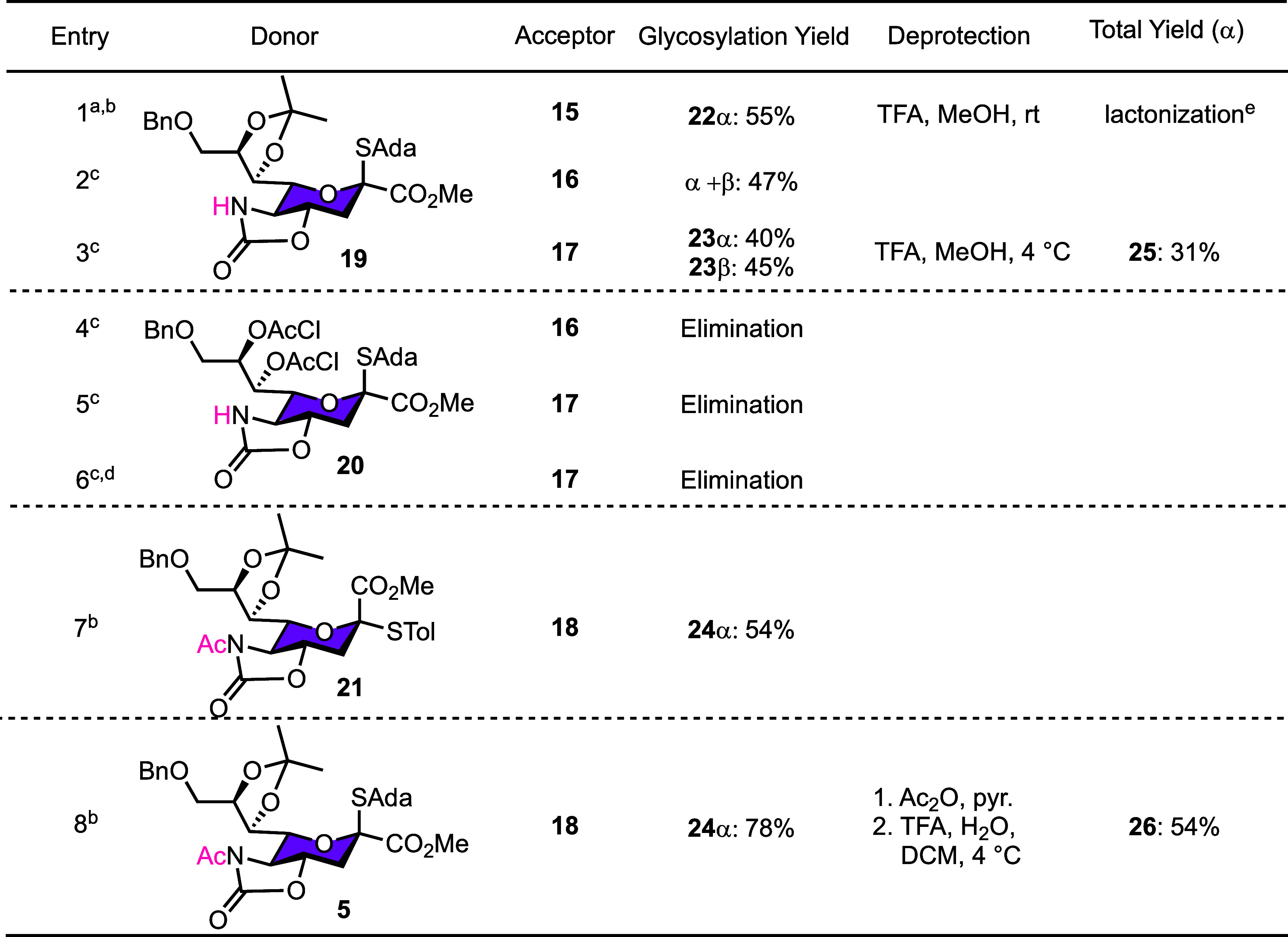
Effects of Sialyl Donor Reactivity and Acceptor-Protecting Groups on C3

a Gal OBz was deprotected to OH.

b Donor 2 eq.

c Donor 3 eq.

d TMSOTf.

e Between carboxylic acid of sialic acid and C4-OH of Gal.

To probe steric effects, we next employed acceptor **18**, lacking C4-OH protection. Glycosylation with sialyl donors **5** and **21** (both lacking a C5-NH amide, previously shown to enhance glycosylation reactivity)^53^ yielded α-anomers in 78% and 54%, respectively. The more reactive SAda donor **5** gave higher yield than STol donor **21**, indicating that both donor reactivity and steric hindrance at the acceptor C4-position critically affect α­(2,3)-sialylation efficiency.

Finally, isopropylidene deprotection of the resulting TBDPS-protected tetrasaccharides proved challenging. At room temperature, partial TBDPS cleavage occurred under mildly acidic conditions. Performing the reaction at 4 °C (see Table S6 for other deprotection conditions) successfully preserved the TBDPS, yielding the desired tetrasaccharides **25** and **26** in 31% (two steps) and 54% (three steps) yields, respectively. These results demonstrate that balancing donor reactivity and acceptor protection is essential for α­(2,3)-sialylation, providing robust access to stable reducing-end tetrasaccharide acceptors of SJG-2.

### First Generation of [4 + 3] Glycosylation

With donor precursor **14** and acceptors **25** and **26** in hand, we next attempted the key [4 + 3] glycosylation to assemble the full heptasaccharide framework of SJG-2. Compound **14** was transformed into either TCAI or PTFAI derivatives to compare the effects of different leaving groups on coupling efficiency with acceptors **25** or **26** (Table 5). The imidate donors, however, showed high susceptibility to hydrolysis; purification prior to glycosylation reduced the overall two-step yield from 36% to 31% (entries 1 vs 2). The best result was obtained using a 1:2 donor-to-acceptor ratio, where glycosylation of acceptor **25** with the imidate donor produced the desired β-linked heptasaccharide **27** in 48% yield over two steps (entry 3). Notably, no glycosylation occurred when acceptor **26** was employed, regardless of the donor–acceptor ratio (entries 4 and 5). Furthermore, the resulting heptasaccharide **27** showed highly acid lability, undergoing partial decomposition even under mild conditions such as silica gel chromatography.

**Table 5:**
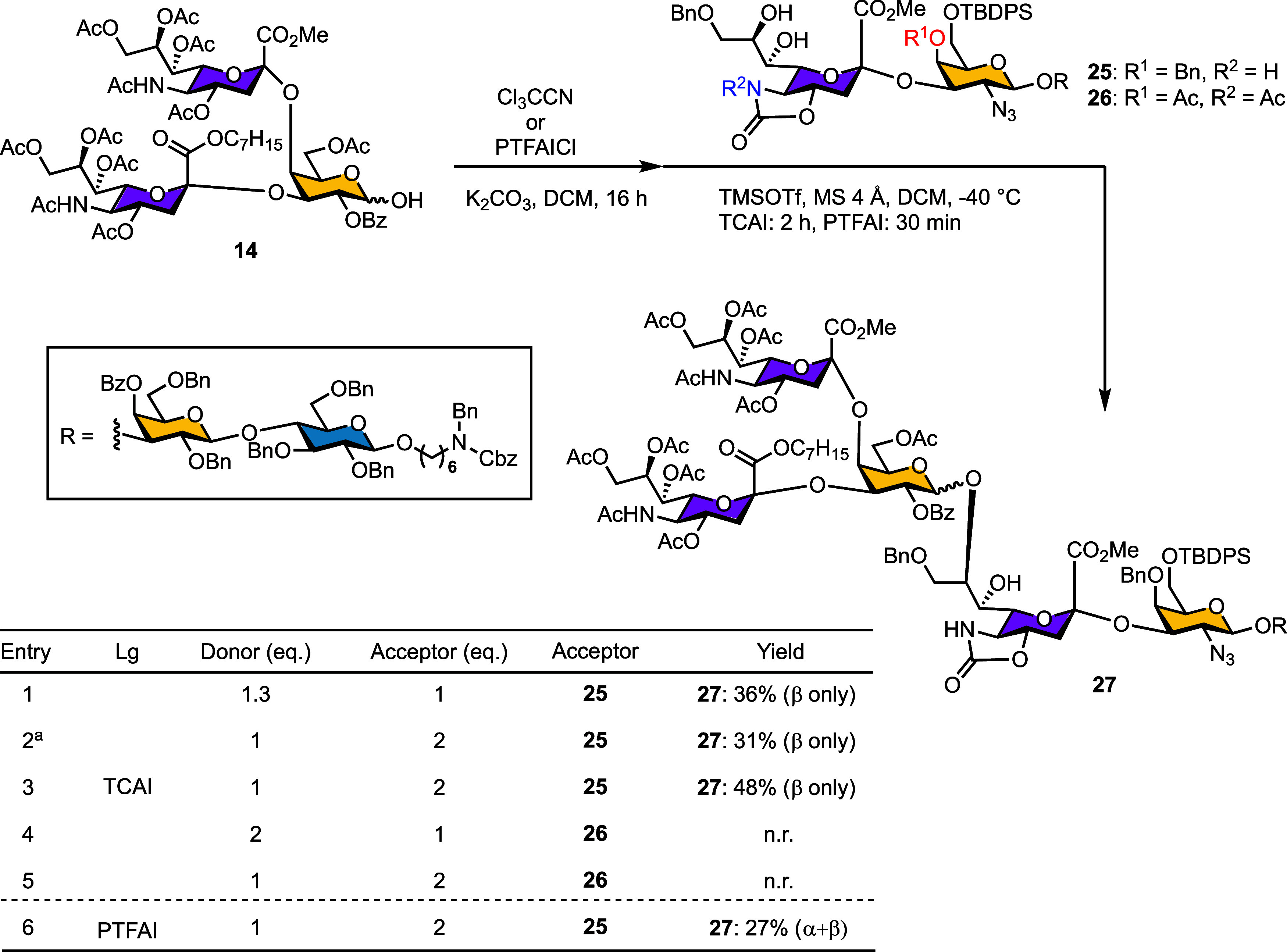
Evaluation of Leaving Groups, Donor–Acceptor Ratios, and Acceptor Structure in the [4 + 3] Glycosylation Step

a Purified at imidate.

Attempts at global deprotection of heptasaccharide **27** were unsuccessful (Scheme S3). Efforts to remove the TBDPS group either before or after ester hydrolysis led to degradation into unidentified fragments, while selective azide reduction using Lindlar’s catalyst also failed. Although this first-generation strategy successfully constructed the heptasaccharide framework, several limitations became evident. These included the limited availability of donor **D9**, which hindered scale-up; the modest yield (57%) of the key reducing-end disialyl intermediate **P20**; and the conformational rigidity of its constrained sialic acid residue, which complicated structural characterization and required variable-temperature NMR analysis. Collectively, these challenges highlight the need to redesign the synthetic route and refine donor–acceptor pairing to overcome the obstacles encountered in this first-generation approach.

### Second Generation of [4 + 3] Glycosylation

Encouraged by the excellent α-selectivity and efficiency of donor **5** (Table 4) in constructing the reducing-end tetrasaccharide acceptor, we next applied it in a [2 + 1] glycosylation with acceptor **A15** to generate the trisaccharide donor (Scheme 3). Compared with Ando’s donor **D9**, donor **5** delivered a significantly improved outcome, yielding α-trisaccharide **28** in 91% yield (**28** vs **P19** in Table 3). Attempts to modify the orthogonal protecting groups on **28** (Scheme S4), however, were inefficient, giving poor overall yields due to acetyl migration and the intrinsically low nucleophilicity of the sialic acid C7 and C8 hydroxyl groups. Preparative synthesis was further complicated by the requirement for HPLC purification.

**Scheme 3:**
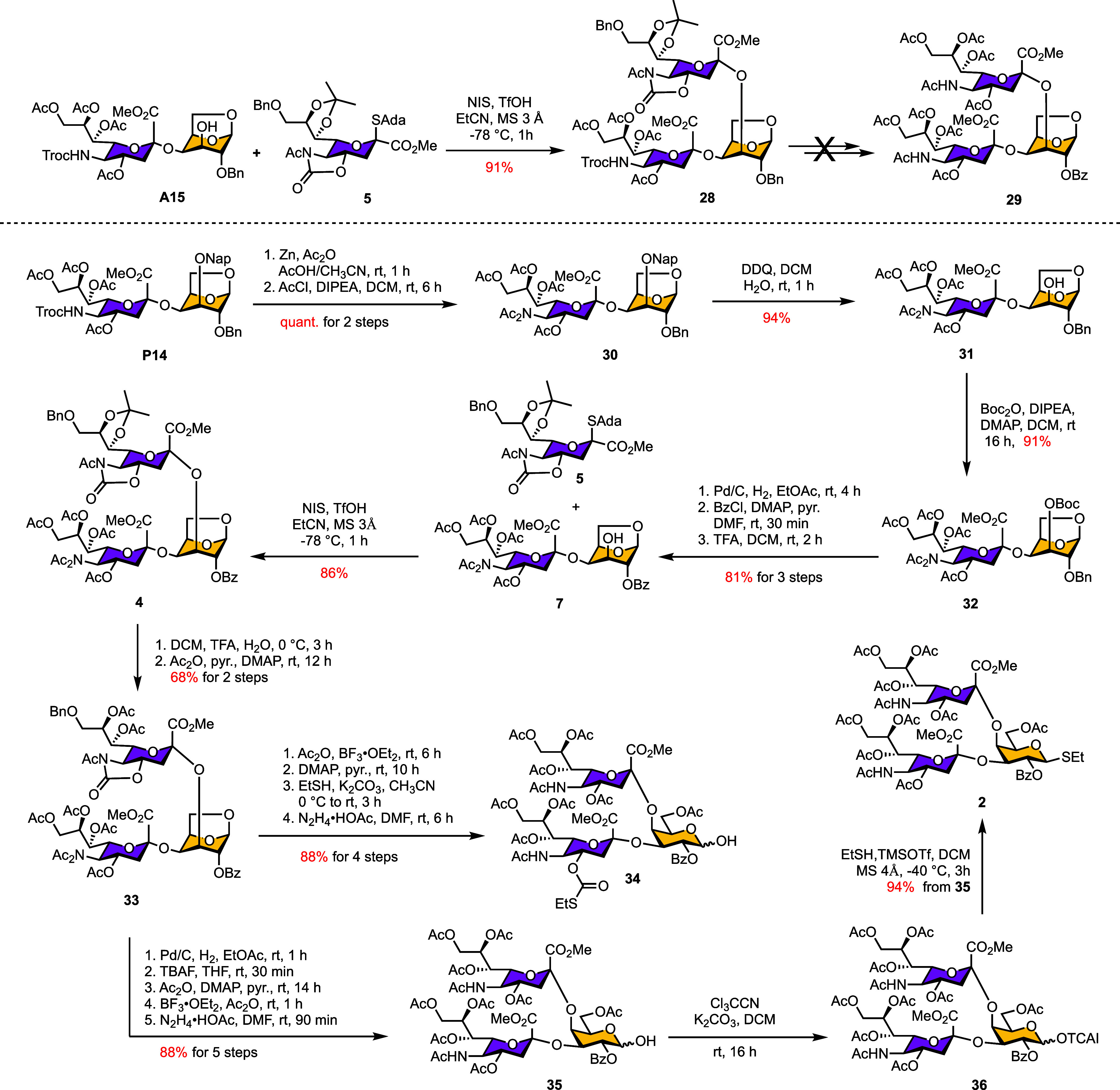
[2 + 1] Glycosylation Strategy Involving Disaccharide-Based Protection Group Remodeling^a^

a Synthesis of Trisaccharide Donors **2** and **36**.

We next evaluated both ^1^C_4_- and ^4^C_1_-configured Gal acceptors bearing free C3 and C4 hydroxyl groups as substrates for mono- or disialylation (Scheme S5). These efforts also produced unsatisfactory results, prompting a redesign of the synthetic sequence by remodeling the protecting groups at the disaccharide stage. As shown in Scheme 3, the NHTroc group on disaccharide **P14** (Table 2, entry 12) was transformed into an NAc_2_ group through Zn-mediated Troc deprotection followed by acetylation, affording compound **30** in quantitative yield. To preserve orthogonality at Gal-C3, the Nap group was converted into a Boc group to give **32** (91% yield). Subsequent hydrogenolysis of the Gal-C2 benzyl group, followed by benzoylation, and final Boc deprotection with TFA yielded compound **7** in 81% yield over three steps. Importantly, benzoylation proved far more efficient at the disaccharide stage than at the trisaccharide stage (compound **12**, Scheme 2), proceeding smoothly at room temperature.

Using this modified disaccharide **7**, a [2 + 1] glycosylation was then performed. Despite the presence of electron-withdrawing Bz group at the ^1^C_4_ Gal-C2 position, donor **5** remained highly effective, producing the desired α-trisaccharide **4** in 86% yield. Subsequent deprotection-protection manipulations of the hydroxyl groups, including TBAF- or ethanethiol-mediated^54^ cleavage of the 5-*N*,4-*O*-oxazolidinone motif, afforded the synthesis of trisaccharides **34**, **36**, and **2** in good overall yields.

In our earlier [4 + 3] glycosylation studies, the TBDPS groups on tetrasaccharide **23** and heptasaccharide **27** proved highly acid-labile. To improve acid stability and streamline global deprotection, we replaced the TBDPS group of compound **17** with a benzoyl group, generating trisaccharide **6** (Scheme S2) as the revised acceptor scaffold (Scheme 4). In the subsequent [3 + 1] glycosylation of acceptor **6** with donor **5** to form the reducing-end tetrasaccharide **37**, installation of a benzyl group at GalN_3_–C4 slightly reduced α-selectivity (from 78% to 73%, with 16% β anomer) compared to the free C4-OH acceptor (Table 4, Entry 8). Nevertheless, eliminating TBDPS-related decomposition improved the overall two-step yield (from **6**), giving acceptor **38** in 64% yield.

**Scheme 4:**
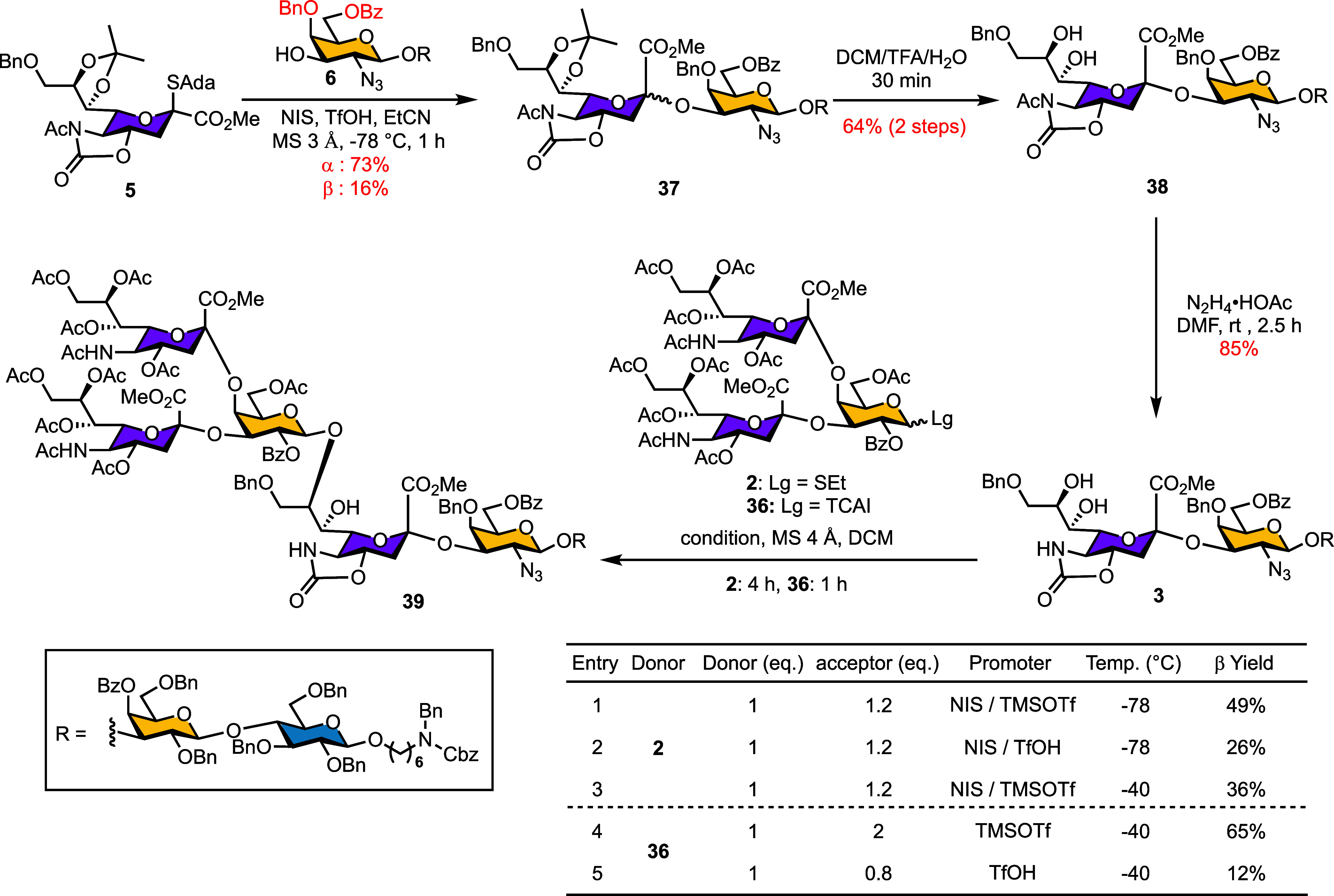
Reactivity Recovery via Protecting Group Remodeling and Conformational Optimization for [4 + 3] Glycosylation

However, tetrasaccharide **38** failed to undergo glycosylation under either [4 + 3] (using TCAI or PTFAI derivatives of **34**) or [4 + 1] conditions (Scheme S6). To rationalize this poor reactivity, we performed ChemDraw conformational analysis of reactive (**25**) versus unreactive (**26** and **38**) acceptors. The comparison revealed that the *N*-acetyl group in the unreactive substrates, which appeared to introduce steric hindrance near the sialic acid C8-OH site, thereby reducing nucleophilicity. This hypothesis was experimentally validated: selective hydrazine acetate treatment of compound **38** effectively removed the oxazolidinone acetyl group, yielding tetrasaccharide acceptor **3** in 85% yield. Subsequent [4 + 3] glycosylation of acceptor **3** with trisaccharide donors **2** or **36** proceeded smoothly, confirming that the *N*-acetyl group was indeed responsible for suppressed reactivity. Notably, different trisaccharide donors were employed in these comparisons: donor **34** contains a carbonothioate on the α­(2,3)-linked sialic acid, whereas donors **2** and **36** bear an acetate at this position.

Optimization of this [4 + 3] glycosylation further established that donor **36** afforded the desired product in 65% yield when two equivalents of acceptor were used (entry 4). The efficiency dropped sharply at lower acceptor loadings (entry 5), and the use of TfOH as promoter negatively affected yields. Donor **2** delivered the heptasaccharide **39** in 49% yield with 1.2 equiv of acceptor (entries 1–3). Notably, heptasaccharide **39** was highly acid-sensitive, rendering TfOH unsuitable as a promoter for this glycosylation (entries 2 and 5).

### Global Deprotection and Completion of SJG-2 Synthesis

Global deprotection of heptasaccharide **39** began with zinc-mediated azide reduction in the presence of AcOH/Ac_2_O during which the liberated amine was immediately acetylated, yielding the corresponding acetamide (NHAc) derivative (Scheme 5). Subsequent treatment with aqueous LiOH removed all ester protecting groups as well as 5-*N*,4-*O*-oxazolidinone motif, and reacetylation with Ac_2_O reintroduced the *N*-acetyl moiety. Final global hydrogenolysis under acidic conditions, followed by sequential purification using activated carbon and a P2 BioGel column chromatography, produced the target heptasaccharide SJG-2 (**1**) in 61% overall yield over four steps.

**Scheme 5:**
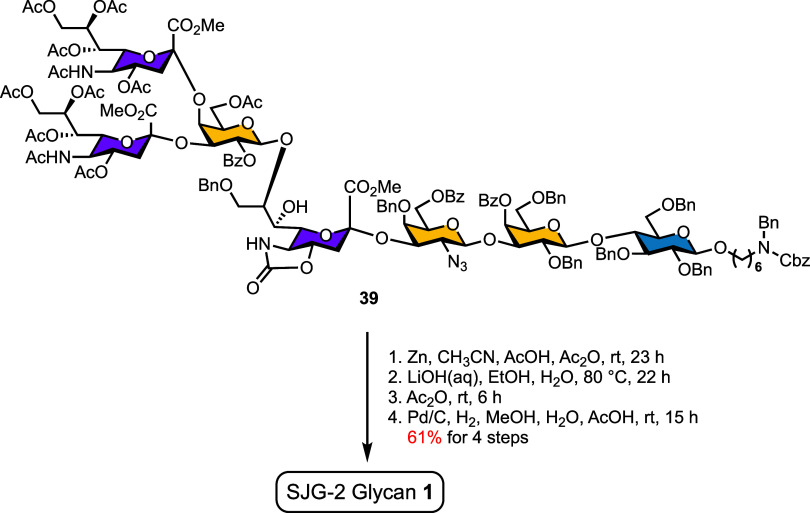
Final Global Deprotection and Completion of SJG-2 Synthesis

### Structural Validation of Synthetic SJG-2 Glycan

To validate the structural fidelity of synthetic compound **1**, we compared the ^13^C NMR chemical shifts of its anomeric carbons (glycan moiety only) with those reported for natural SJG-2 bearing ceramide (Supporting Information, Table S7).^19^ Because the natural product contains a ceramide moiety and the synthetic analog carries a simplified saturated alkyl chain, identical solvent conditions (D_2_O:pyridine-d_5_ = 5:95) could not be applied. Instead, compound **1** was analyzed in D_2_O:pyridine-d_5_ = 95:5. Despite this difference, the relative chemical shift patterns of the anomeric carbons were largely consistent with downfield shifts of 1.6 to 2.2 ppm. Minor Δδ variations were observed for the Glc and GalNAc residues. The Glc-C1 difference likely arises from the absence of the ceramide aglycone in the synthetic sample, while the GalNAc shift may reflect solvent-induced changes in electronic distribution at C2. Overall, the ^13^C NMR data strongly supported the structural equivalence of synthetic SJG-2 (**1**) with the natural sample.

## Conclusion

In summary, we have completed the first total synthesis of the neuroregenerative ganglioside SJG-2 glycan **1** through a convergent [4 + 3] glycosylation strategy that systematically overcomes long-standing barriers in sialylation chemistry. Central to this success were three advances: (i) conformational inversion of the galactose acceptor from ^4^C_1_ to ^1^C_4_ enabled efficient α­(2,4)-sialylation, granting access to key α­(2,3)- and α­(2,4)-linked disialyl trisaccharide motifs (**14**, **34** or **35**) that would otherwise be difficult to obtain (ii) donor tuning combined with strategic protecting-group design of trisaccharide acceptors (**6** or **18**) optimized both the stereoselectivity and efficiency of α­(2,3)-sialylation at the galactosamine C3 position; and (iii) selective modulation of the 5-*N*,4-*O*-oxazolidinone-protected sialoside tetrasaccharide acceptors (**3** or **25**) was crucial for enabling formation of the rare Gal-α­(1,8)-Neu5Ac linkage. Together, these findings establish generalizable principles for addressing reactivity and selectivity challenges in sterically congested sialosides. Beyond the synthesis of SJG-2, this approach offers a broadly applicable framework for accessing structurally complex gangliosides and facilitates opportunities for advancing glycan-based applications in glycobiology, diagnostics, and therapeutic discovery.

## Supplementary Material



## Notes

Detailed experimental procedures, analysis of 1D and 2D NMR spectra, characterization of new compounds, and ^1^H NMR and ^13^C NMR spectra (PDF)

## References

[1] Schnaar, R. L. ; Sandhoff, R. ; Tiemeyer, M. ; Kinoshita, T. Glycosphingolipids. In Essentials of Glycobiology, 4th ed.; Varki, A. ; Cummings, R. D. ; Esko, J. D. ; Stanley, P. ; Hart, G. W. ; Aebi, M. ; Mohnen, D. ; Kinoshita, T. ; Packer, N. H. ; Prestgard, J. H. ; Schnaar, R. L. ; Seeberger, P. H. , Eds.; Cold Spring Harbor: NY, 2022; pp 125–148.

[2] Merrill A. H. (2011). Sphingolipid and Glycosphingolipid
Metabolic Pathways
in the Era of Sphingolipidomics. Chem. Rev..

[3] Schnaar R. L. (2019). The biology
of gangliosides. Adv. Carbohdr. Chem. Biochem..

[4] Cutillo G., Saariaho A.-H., Meri S. (2020). Physiology
of Gangliosides and The
Role of Antiganglioside Antibodies in Human Diseases. Cell. Mol. Immunol..

[5] Inci O. K., Basirli H., Can M., Yanbul S., Seyrantepe V. (2024). Gangliosides
as Therapeutic Targets for Neurodegenerative Diseases. J. Lipids.

[6] Hunter C. D., Guo T., Daskhan G., Richards M. R., Cairo C. W. (2018). Synthetic strategies
for modified glycosphingolipids and their design as probes. Chem. Rev..

[7] Ando H., Komura N. (2024). Recent progress in the synthesis
of glycosphingolipids. Curr. Opin. Chem. Biol..

[8] Bonab M. K. F., Guo Z., Li Q. (2024). Glycosphingolipids:
From metabolism
to chemoenzymatic total synthesis. Org. Biomol.
Chem..

[9] Adak A. K., Tseng H.-K., Chang S.-Y., Chiang Y.-C., Lyu K.-H., Lee Y.-S., Lu W., Kuo W.-H., Angata T., Lin C.-C. (2024). Comprehensive Modular
Synthesis of Ganglioside Glycans
and Evaluation of Their Binding Affinities to Siglec-7 and Siglec-9. Adv. Sci..

[10] Higuchi R., Inagaki M., Yamada K., Miyamoto T. (2007). Biologically active
gangliosides from echinoderms. J. Nat. Med..

[11] Kaneko M., Yamada K., Miyamoto T., Inagaki M., Higuchi R. (2007). Neuritogenic
activity of gangliosides from echinoderms and their structure-activity
relationship. Chem. Pharm. Bull..

[12] Tamai H., Imamura A., Ogawa J., Ando H., Ishida H., Kiso M. (2015). First Total Synthesis
of Ganglioside GAA-7 from Starfish Asterias
amurensis versicolor. Eur. J. Org. Chem..

[13] Tseng H.-K., Su Y.-Y., Chang T.-W., Liu H.-C., Li P.-J., Chiang P.-Y., Lin C.-C. (2021). Acceptor-mediated
regioselective
enzyme catalyzed sialylation: chemoenzymatic synthesis of GAA-7 ganglioside
glycan. Chem. Commun..

[14] Tseng H.-K., Su Y.-Y., Lai P.-J., Lo S.-L., Liu H.-C., Reddy S. R., Chen L. Y., Lin C.-C. (2024). Chemoenzymatic Synthesis
of GAA-7 Glycan Analogues and Evaluation of Their Neuritogenic Activities. ACS Chem. Neurosci..

[15] Cho Y.-T., Adak A. K., Su Y.-Y., Chang T.-W., Lin C.-C. (2022). Chemoenzymatic
Total Synthesis of the Neuritogenic Echinoderm Ganglioside LLG-5 and
Related Analogues. Adv. Synth. Catal..

[16] Tamai H., Ando H., Tanaka H.-N., Hosoda-Yabe R., Yabe T., Ishida H., Kiso M. (2011). The Total
Synthesis
of the Neurogenic Ganglioside LLG-3 Isolated from the Starfish Linckia
Laevigata. Angew. Chem., Int. Ed..

[17] Rich J. R., Withers S. G. (2012). A Chemoenzymatic
Total Synthesis of the Neurogenic
Starfish Ganglioside Llg-3 Using an Engineered and Evolved Synthase. Angew. Chem., Int. Ed..

[18] Wu Y.-F., Tsai Y.-F., Huang Y.-S., Shih J.-F. (2020). Total Synthesis
of the Echinodermatous Ganglioside LLG-3 Possessing the Biological
Function of Promoting the Neurite Outgrowth. Org. Lett..

[19] Kaneko M., Kisa F., Yamada K., Miyamoto T., Higuchi R. (2003). Structure
of a New Neuritogenic-Active Ganglioside from the Sea Cucumber Stichopus
japonicus. Eur. J. Org. Chem..

[20] Zhu W., Zhou Y., Guo L., Feng S. (2024). Biological function
of sialic acid and sialylation in human health and disease. Cell Death Discovery.

[21] Chen X. (2024). Enabling chemoenzymatic
strategies and enzymes for synthesizing sialyl glycans and sialyl
glycoconjugates. Acc. Chem. Res..

[22] Adak A. K., Yu C.-C., Liang C.-F., Lin C.-C. (2013). Synthesis of Sialic
Acid-Containing Saccharides. Curr. Opin. Chem.
Biol..

[23] Vibhute A. M., Komura N., Tanaka H.-N., Imamura A., Ando H. (2021). Advanced Chemical
Methods for Stereoselective Sialylation and Their Applications in
Sialoglycan Syntheses. Chem. Rec..

[24] Singh Y., Geringer S. A., Demchenko A. V. (2022). Synthesis
and Glycosidation of Anomeric
Halides: Evolution from Early Studies to Modern Methods of the 21st
Century. Chem. Rev..

[25] Crich D., Li W. (2007). α-Selective sialylations
at −78 °C in nitrile solvents
with a 1-adamantanyl thiosialoside. J. Org.
Chem..

[26] Hsu C.-H., Chu K.-C., Lin Y.-S., Han J.-L., Peng Y.-S., Ren C.-T., Wu C.-Y., Wong C.-H. (2010). Highly alpha-selective
sialyl phosphate donors for efficient preparation of natural sialosides. Chem. - Eur. J..

[27] Cai S., Yu B. (2003). Efficient
Sialylation with Phenyltrifluoroacetimidates as Leaving
Groups. Org. Lett..

[28] Ando H., Koike Y., Ishida H., Kiso M. (2003). Extending the possibility
of an *N*-Troc-protected sialic acid donor toward variant
sialo-glycoside synthesis. Tetrahedron Lett..

[29] Hanashima S., Castagner B., Esposito D., Nokami T., Seeberger P. H. (2007). Synthesis
of a sialic acid α(2–3) galactose building block and
its use in a linear synthesis of sialyl Lewis X. Org. Lett..

[30] Yu C. S., Niikura K., Lin C. C., Wong C. H. (2001). The thioglycoside
and glycosyl phosphite of 5-azido sialic acid: Excellent donors for
the α-glycosylation of primary hydroxy groups. Angew. Chem., Int. Ed..

[31] Tanaka H., Nishiura Y., Takahashi T. (2006). Stereoselective
synthesis of oligo-α-(2,8)-sialic
acids. J. Am. Chem. Soc..

[32] Crich D., Li W. (2007). *O*-Sialylation
with *N*-acetyl-5-*N*,4-*O*-carbonyl-protected thiosialoside
donors in dichloromethane: facile and selective cleavage of the oxazolidinone
ring. J. Org. Chem..

[33] van
Hengst J. M. A., Hellemons R. J. C., Remmerswaal W. A., van de Vrande K. N. A., Hansen T., van der Vorm S., Overkleeft H. S., van der Marel G. A., Codée J. D. C. (2023). Mapping
the Effect of Configuration and Protecting Group Pattern on Glycosyl
Acceptor Reactivity. Chem. Sci..

[34] Tanaka S.-i., Goi T., Tanaka K., Fukase K. (2007). Highly Efficient α-Sialylation
by Virtue of Fixed Dipole Effects of *N*-Phthalyl Group:
Application to Continuous Flow Synthesis of α(2–3)-and
α(2–6)-Neu5Ac-Gal Motifs by Microreactor. J. Carbohydr. Chem..

[35] Dhakal B., Buda S., Crich D. (2016). Stereoselective synthesis
of 5-epi-α-sialosides
related to the pseudaminic acid glycosides. Reassessment of the stereoselectivity
of the 5-azido-5-deacetamidosialyl thioglycosides and use of triflate
as nucleophile in the Zbiral deamination of sialic acids. J. Org. Chem..

[36] Zhang Y., Yang M., Wang X., Gu G., Cai F. (2020). Improved α-Sialylation
through the Synergy of 5-*N*,4-*O*-Oxazolidinone
Protection and Exocyclic C-1 Neighboring Group Participation. J. Org. Chem..

[37] Tanaka H., Nishiura Y., Takahashi T. (2008). An efficient convergent synthesis
of GP1c ganglioside epitope. J. Am. Chem. Soc..

[38] Tanaka H., Ando H., Ishida H., Kiso M., Ishihara H., Koketsu M. (2009). Synthetic study on
α(2→8)-linked oligosialic
acid employing 1,5-lactamization as a key step. Tetrahedron Lett..

[39] Koinuma R., Tohda K., Aoyagi T., Tanaka H. (2020). Chemical synthesis
of α­(2,8) octasialosides, the minimum structure of polysialic
acids. Chem. Commun..

[40] Lin C.-C., Lin N.-P., Sahabuddin L. S., Reddy V. R., Huang L. D., Hwang K.-C., Lin C.-C. (2010). 5-*N*,4-*O*-carbonyl-7,8,9-tri-*O*-chloroacetyl-protected sialyl
donor for the stereoselective synthesis of α-(2–9)-tetrasialic
acid. J. Org. Chem..

[41] Lin C.-C., Adak A. K., Horng J.-C., Lin C.-C. (2009). Phosphite-based
sialic acid donors in the synthesis of α(2→9) oligosialic
acids. Tetrahedron.

[42] Pedersen C. M., Nordstrøm L. U., Bols M. (2007). “Super Armed” Glycosyl
Donors: Conformational Arming of Thioglycosides by Silylation. J. Am. Chem. Soc..

[43] Hsu Y., Lu X. A., Zulueta M. M. L., Tsai C. M., Lin K. I., Hung S. C., Wong C. H. (2012). Acyl and Silyl Group Effects in Reactivity-Based
One-Pot Glycosylation: Synthesis of Embryonic Stem Cell Surface Carbohydrates
Lc4 and IV2Fuc-Lc4. J. Am. Chem. Soc..

[44] Hori H., Nakajima T., Nishida Y., Ohrui H., Meguro H. (1988). A simple method
to determine the anomeric configuration of sialic acid and its derivatives
by ^13^C-NMR. Tetrahedron Lett..

[45] Xia J., Alderfer J. L., Piskorz C. F., Matta K. L. (2001). The 2-Naphthylmethyl
(NAP) Group in Carbohydrate Synthesis: First Total Synthesis of the
GlyCAM-1 Oligosaccharide Structures. Chem. -
Eur. J..

[46] Kanie O., Kiso M., Hasegawa A. (1988). Glycosylation
Using Methylthioglycosides
of N-Acetylneuraminic Acid and Dimethyl-(Methylthio)­Sulfonium Triflate. J. Carbohydr. Chem..

[47] Schmidt R. R., Behrendt M., Toepfer A. (1990). Nitriles as Solvents
in Glycosylation
Reactions: Highly Selective β-Glycoside Synthesis. Synlett.

[48] Komura N., Kato K., Udagawa T., Asano S., Tanaka H.-N., Imamura A., Ishida H., Kiso M., Ando H. (2019). Constrained
sialic acid donors enable selective synthesis of α-glycosides. Science.

[49] Hasegawa A., Ogawa M., Kojima Y., Kiso M. (1992). Synthetic Studies on
Sialoglycoconjugates 36: α-Selective Glycoside Synthesis of *N*-Acetylneuraminic Acid with the Secondary Hydroxyl Group
in D-Glucofyranose, 2-Acetamido-2-deoxy-D-glucopyranose and D-Galactopyranose
Derivatives. Carbohydr. Chem..

[50] Wilstermann M., Kononov L. O., Nilsson U., Ray A. K., Magnusson G. (1995). Synthesis
of ganglioside lactams corresponding to GM1-, GM2-, GM3-, and GM4-ganglioside
lactones. J. Am. Chem. Soc..

[51] Pazynina G., Tuzikiov A., Chinarev A., Obukhova P., Bovin N. (2002). Simple stereoselective
synthesis of α2–6 sialooligosaccharides. Tetrahedron Lett..

[52] Tamai H., Ando H., Ishida H., Kiso M. (2012). First Synthesis of
a Pentasaccharide Moiety of Ganglioside GAA-7 Containing Unusually
Modified Sialic Acids through the Use of *N*-Troc-sialic
Acid Derivative as a Key Unit. Org. Lett..

[53] Zhou J., Manabe Y., Tanaka K., Fukase K. (2016). Efficient Synthesis
of the Disialylated Tetrasaccharide Motif in N-Glycans through an
Amide-Protection Strategy. Chem. - Asian J..

[54] Takeuchi Y., Tohda K., Tanaka H. (2024). Syntheses of α­(2,8)
Sialosides
Containing NeuAc and NeuGc by Using Double Carbonyl-Protected *N*-Acyl Sialyl Donors. Chem. - Eur.
J..

