# Supplementary material for: Total Synthesis of the SJG‑2 Glycan: General Strategies for Constructing Sterically Congested Sialylated Glycans
Source: J Am Chem Soc. 2026 Mar 17;148(12):13296–308. doi: 10.1021/jacs.6c00368 (PMC13047681; doi:10.1021/jacs.6c00368)
Supplement: Supplementary file 1 [file ja6c00368_si_001.pdf]

Total Synthesis of the SJG-2 Glycan: General Strategies for Constructing Sterically Congested Sialylated Glycans

Yung-Yu Su,[a] Avijit K. Adak,[a] Yan-Ting Kuo, [a] Hong-Jyune Lin, [a] Ya-Syuan Li, [a] Hsin-Kai Tseng, [a] and Chun-Cheng Lin [a],[b],*

[a]Department of Chemistry, National Tsing Hua University, Hsinchu 300044, Taiwan

[b]Department of Medicinal and Applied Chemistry, Kaohsiung Medical University, Kaohsiung 807378, Taiwan

*E-Mail: cclin66@mx.nthu.edu.tw

Table of Content

Detailed Screening of Sialylation Reactions……………………………………………3

Table S7. Comparison of anomeric center chemical shifts between synthetic and reported natural ganglioside……………………………………………………………..11

Confirmation of α-Sialyl Linkage by ^{13}C-Coupled NMR, EXSIDE, and HMBC…….12

Experimental Sections………………………………………………………………..19

Reference……………………………………………………………………………166

NMR Spectrum……………………………………………………………………...169

Detailed Screening of Sialylation Reactions

Table S1 Evaluation of α-(2,4) sialylation between various sialyl donors and $^4C_1$-galactose acceptors

Donor + Acceptor → (condition; DCM/ACN) product

Entry	Donor	Acceptor	Promoter	Temp (°C)	M.S.	Yield%
1	**D11**	**A2**	NIS, TfOH	-78 to -50	4Å	elimination
2	**D6**	**A3**	NIS, TfOH	-78	AW-300	elimination
3	**D3**		NIS, TfOH	-78	AW-300	elimination
4	**D6**	**A6**	NIS, TfOH	-40	AW-300	elimination
5	**D10**		TMSOTf	-78	AW-300	elimination
6	**D2**	**A17**	TMSOTf	-78	4Å	no reaction
7	**D11**		NIS, TfOH	-78	4Å	elimination
8	**D11**	**A18**	NIS, TfOH	-78	4Å	elimination
9	**D1**	**A19**	NIS, TfOH	-78 to -50	4Å	elimination
10	**D3**		NIS, TfOH	-78 to -50	4Å	elimination
11	**D1**	**A20**	NIS, TfOH	-78	4Å	elimination
12	**D3**		NIS, TfOH	-78	4Å	elimination

Donor

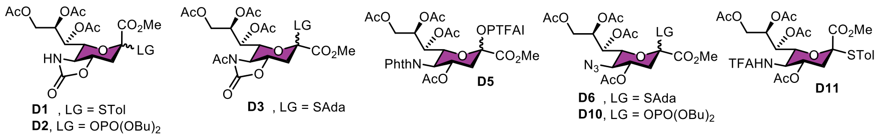

Acceptor

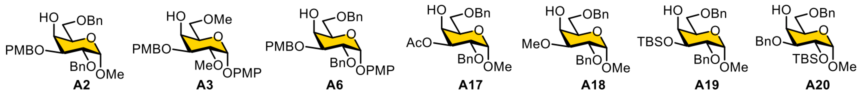

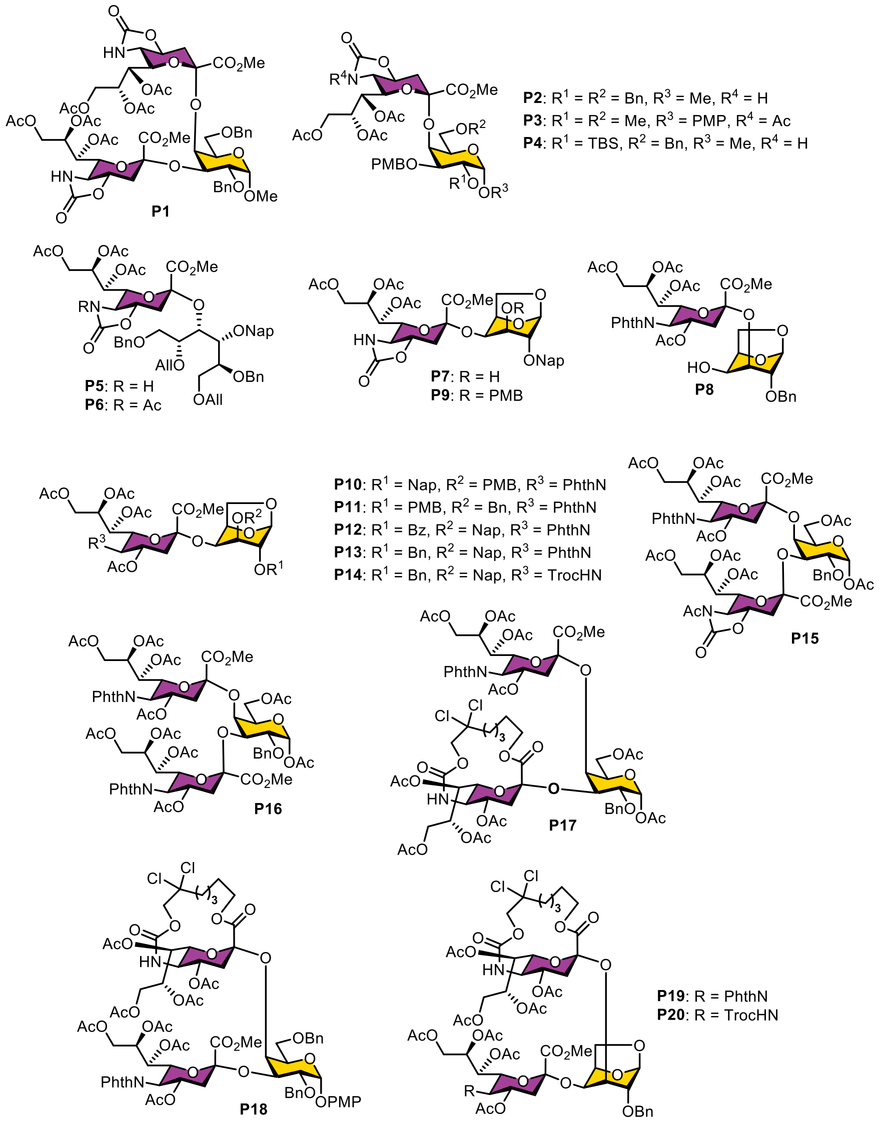

Figure S1. Structures of all products obtained in the sialylation screening study.

Table S2. Evaluation of α-(2,4) sialylation with $^{1}C_4$-galactose acceptors

Donor + Acceptor —(Condition, -78 °C; solvent)→ Product

Entry	Donor	Acceptor	Promoter	Solvent	M.S.	Yield%(α/β)
1[a]	**D1**	**A7**	NIS, TfOH	DCM/ACN	4Å	elimination
2[b]	**D11**	**A7**	NIS, TfOH	DCM/ACN	4Å	elimination
3	**D6**	**A12**	NIS, TfOH	DCM/ACN	AW-300	elimination
4	**D3**	**A12**	NIS, TfOH	DCM/ACN	AW-300	elimination
5	**D5**	**A12**	TMSOTf	DCM/ACN	AW-300	elimination
6	**D5**	**A21**	TMSOTf	EtCN	AW-300	elimination

a: -78 to -40; b: -78 to -50

Donor

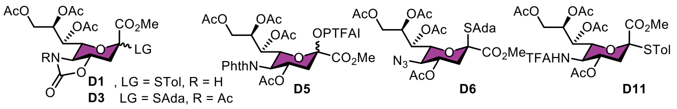

Acceptor

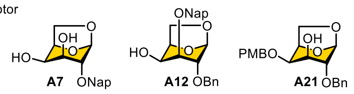

Table S3. Evaluation of α-(2, 3) sialylation with various donors and acceptors

Donor + Acceptor —(Condition, -78 °C; EtCN, MS 3Å)→ Product

Entry	Donor	Acceptor	Promoter	Yield%
1	**D7**		NIS, TMSOTf	elimination
2[c]	**D7**		NIS, AgOTf	elimination
3	**D12**		TMSOTf	elimination
4	**D5**		TMSOTf	elimination
5	**D3**		NIS, TfOH	elimination
6[a]	**D3**	**A13**	NIS, TfOH	elimination
7	**D4**		TMSOTf	elimination
8[a]	**D4**		TMSOTf	elimination
9	**D9**		NIS, TfOH	elimination
10[bd]	**D9**		NIS, TfOH	trace
11[e]	**D5**	**A15**	TMSOTf	elimination

a: added DTBMP (1 eq.), b: Donor (10 eq.), c: reaction temperature was rt, d: DCM, e: AW-300

Donor

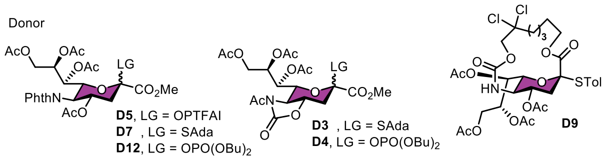

Acceptor

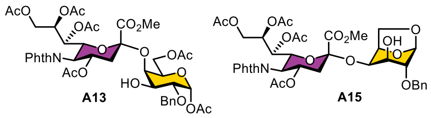

Table S4. Intramolecular glycosylation approaches toward non-reducing end trisaccharide

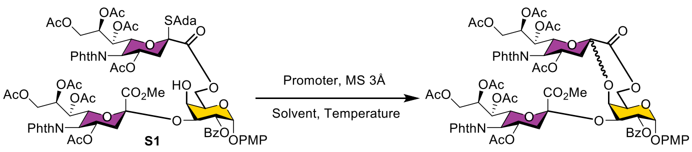

Entry	Promoter	Solvent	Temperature	Yield
1	NIS/TfOH	DCM	-78 °C	Elimination
2	NIS/TfOH	EtCN	-78 °C	Elimination
3	NIS/TMSOTf	EtCN	-78 °C	Elimination
4	NIS/AgOTf	EtCN	rt	Elimination
5	I_2/AgOTf	EtCN	-78 °C	No activation
6	Ph_2SO/DTBMP/Tf_2O	EtCN	-78 °C	PMP decompose
7	TolSCl/AgOTf	EtCN	-78 °C to rt	Elimination

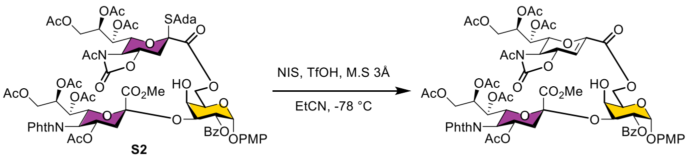

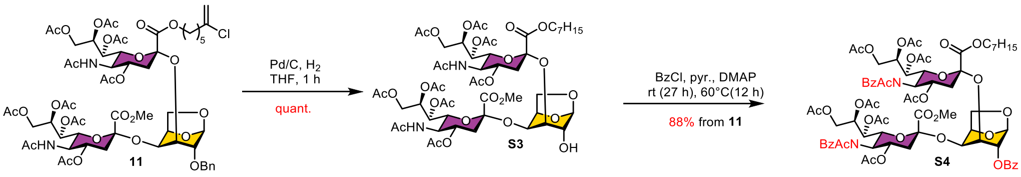

Scheme S1. Overacylation during galactose C2-benzoylation

Table S5. Comparative study of α-(2,3) sialylation with various GalNTroc acceptors

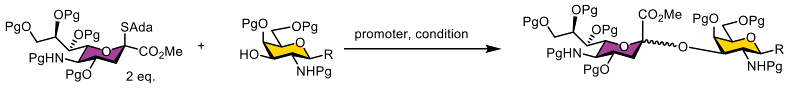

Entry	Donor	Acceptor	Promoter	Condition	Yield
1			NIS, TfOH	DCM/ACN: 2/1 MS 4 Å, -78 to -40 °C	elimination
2	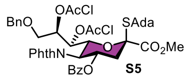 S5	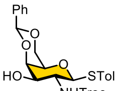 S11	NIS, TfOH	EtCN, MS 4 Å -78 °C	elimination
3			NIS, TfOH	EtCN, AW-300 -78 °C	elimination
4	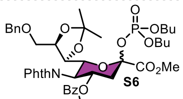 S6		TMSOTf	EtCN, AW-300 -78 °C	elimination
5	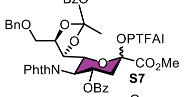 S7		TMSOTf	EtCN, AW-300 -78 °C	elimination
6	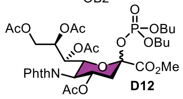 D12	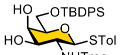 S12	TMSOTf	EtCN, MS 3Å -78 to -20 °C	elimination
7	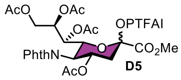 D5		TMSOTf	EtCN, MS 3Å -78 °C	elimination
8	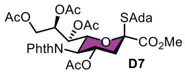 D7		AgOTf, TolSCl	EtCN, MS 3Å -78 °C to -20 °C	elimination
9	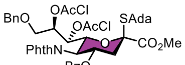 S5	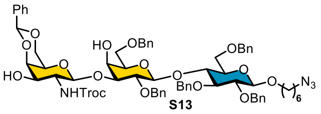 S13	NIS/TMSOTf	EtCN, AW-300 -78 °C	elimination
10			NIS/ TfOH	EtCN, AW-300 -78 °C	elimination
11	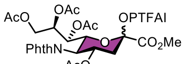 D5	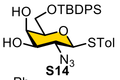 S14	TMSOTf	EtCN, MS 3Å -78 °C	24%
12		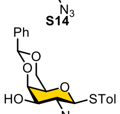 S15	TMSOTf	EtCN, MS 3Å -78 °C	76%
13	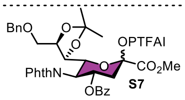 S7	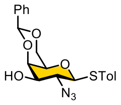 S15	TMSOTf	EtCN, MS 3 Å -78 °C	trace
14	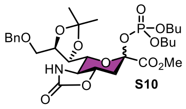 S10		TMSOTf	EtCN, MS 3 Å -78 to -40 °C	elimination

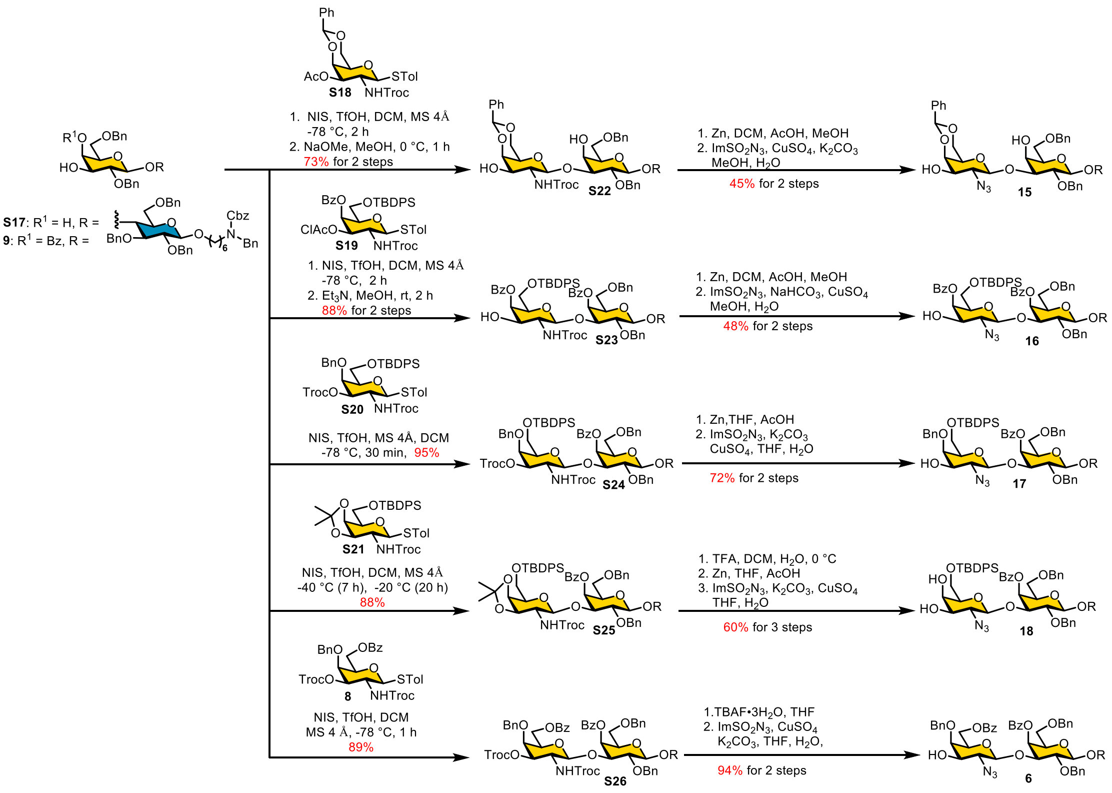

Scheme S2. Synthesis of reducing-end trisaccharides with diverse protecting group

Table S6. Optimization of deprotection conditions for reducing-end tetrasaccharides

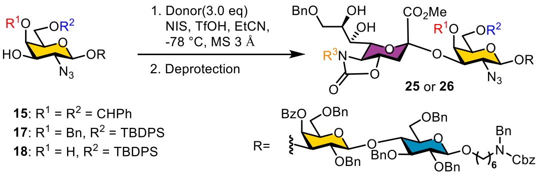

Entry	Donor	R^1	R^2	Deprotection	α Yield
1[a]		CHPh	CHPh	TFA, MeOH, rt	lactonization
2	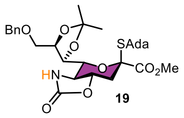	Bn	TBDPS	TFA, MeOH, rt	TBDPS decompose
3		Bn	TBDPS	TFA, MeOH, 4 °C	**25**: 31%
4		Bn	TBDPS	AcOH 80%, 80 °C	**25**: 29%
5	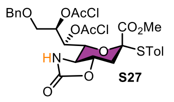	Bn	TBDPS	Et_3N, MeOH, rt	nd
6[b]		Bn	TBDPS	thiourea, 2, 6-lutidine DMF, 80 °C	trace amount of β
7[c]		Bn	TBDPS	thiourea, 2, 6-lutidine DMF, 80 °C	**25**: brsm: 8%
8[b]	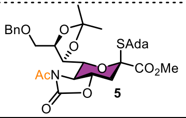	H to Ac	TBDPS	1. Ac_2O, pyr. 2. TFA, H_2O, DCM, 4 °C	**26**: 54%

a: Gal Bz change to OH, b: donor 2 eq., c: donor 4 eq.

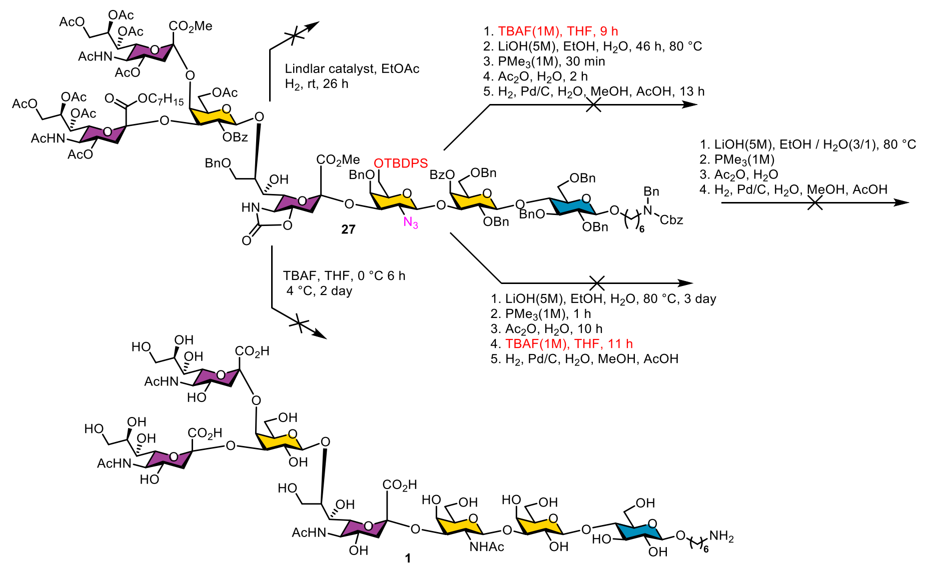

Scheme S3. Evaluation of global deprotection strategies for compound **27**

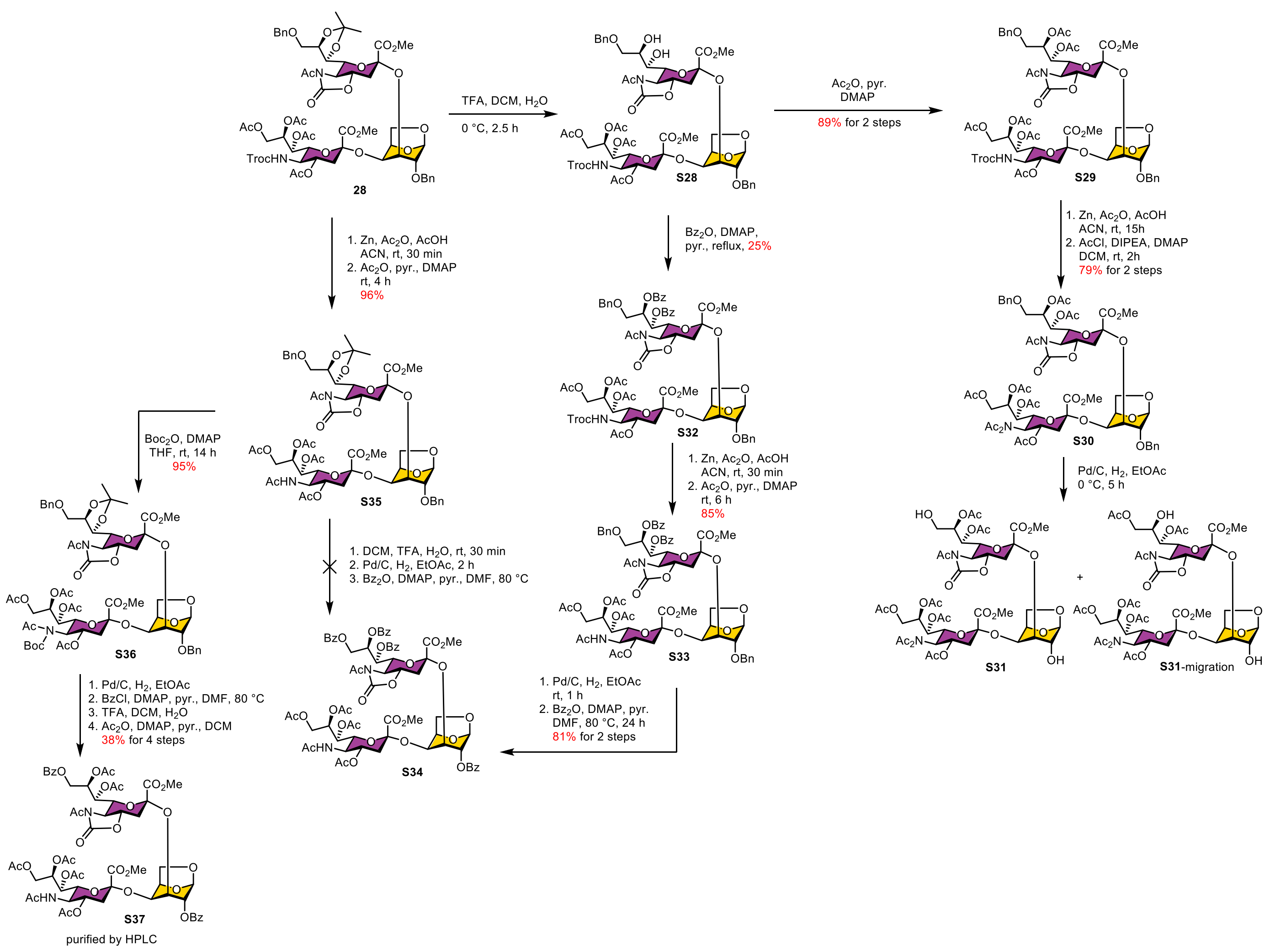

Scheme S4. Strategic and protecting group modifications of compound **28**

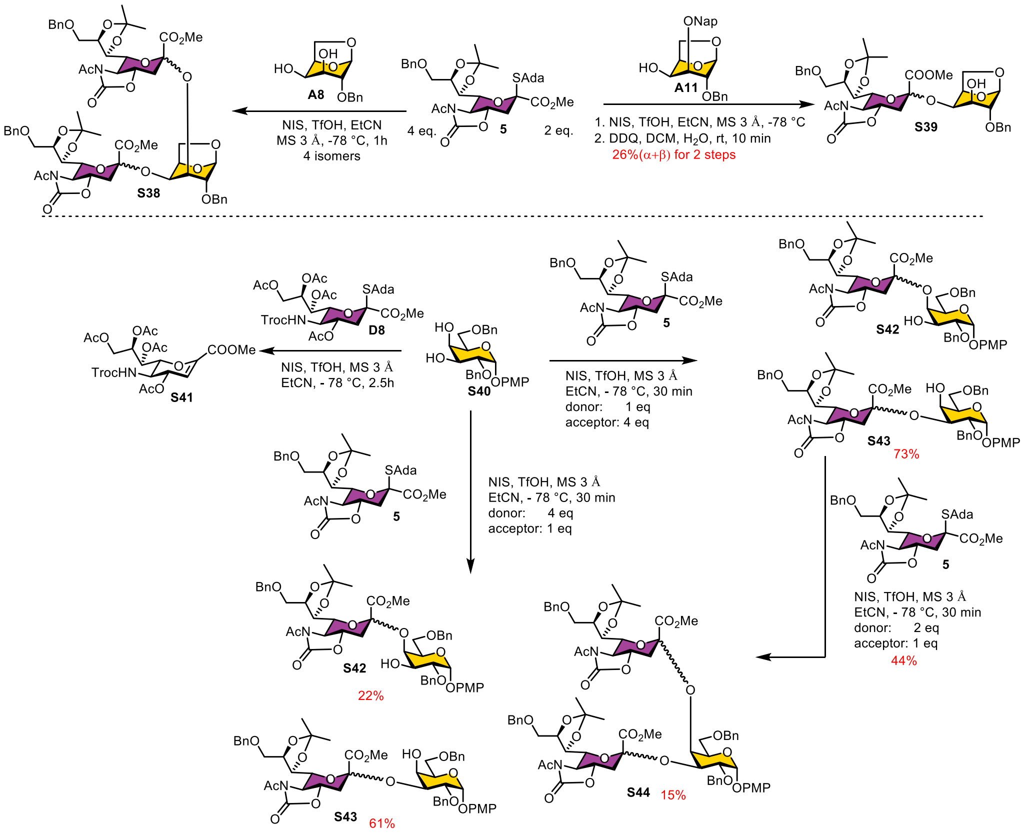

Scheme S5. Glycosylation of diol-acceptors with donor **5**

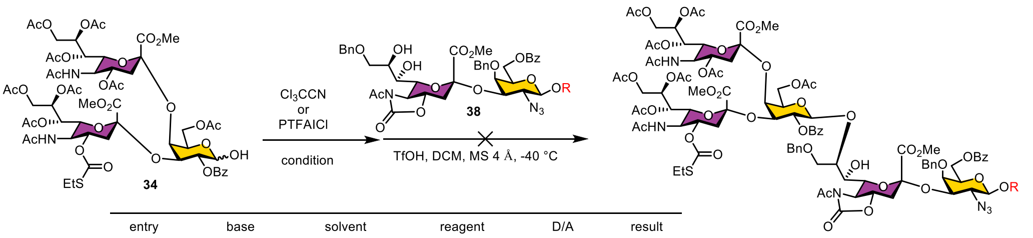

entry	base	solvent	reagent	D/A	result
1	DBU	DCM	Cl_3CCN	1/2	n.r.
2	K_2CO_3	DCM	Cl_3CCN	1/2	n.r.
3	K_2CO_3	DCM	Cl_3CCN	2/1	n.r.
4	K_2CO_3	acetone	PTFAICl	1/2	n.r.

D/A = Donor/Acceptor ratio

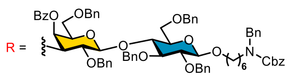

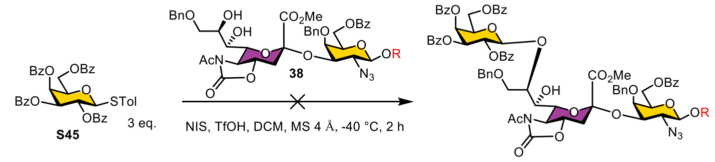

Scheme S6. [4+3] and [4+1] glycosylation with compound **38**

Table S7. Comparison of anomeric center chemical shifts between synthetic and reported natural ganglioside

Glycan residues chemical shifts of anomeric center, δ (ppm)							
Glycan residues	A	B	C	D	E	F	G
reported	103.6	103.7	105.0	100.4	103.8	101.0	101.5
synthetic	104.3	105.3	105.6	102.2	105.6	103.2	103.3
Δδ (ppm)	0.7	1.6	0.6	1.8	1.8	2.2	1.8

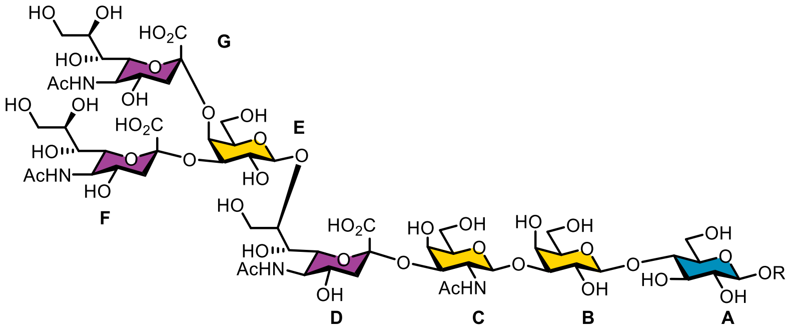

SJG-2 glycan

Confirmation of α-Sialyl Linkage by ^{13}C-Coupled NMR, EXSIDE, and HMBC

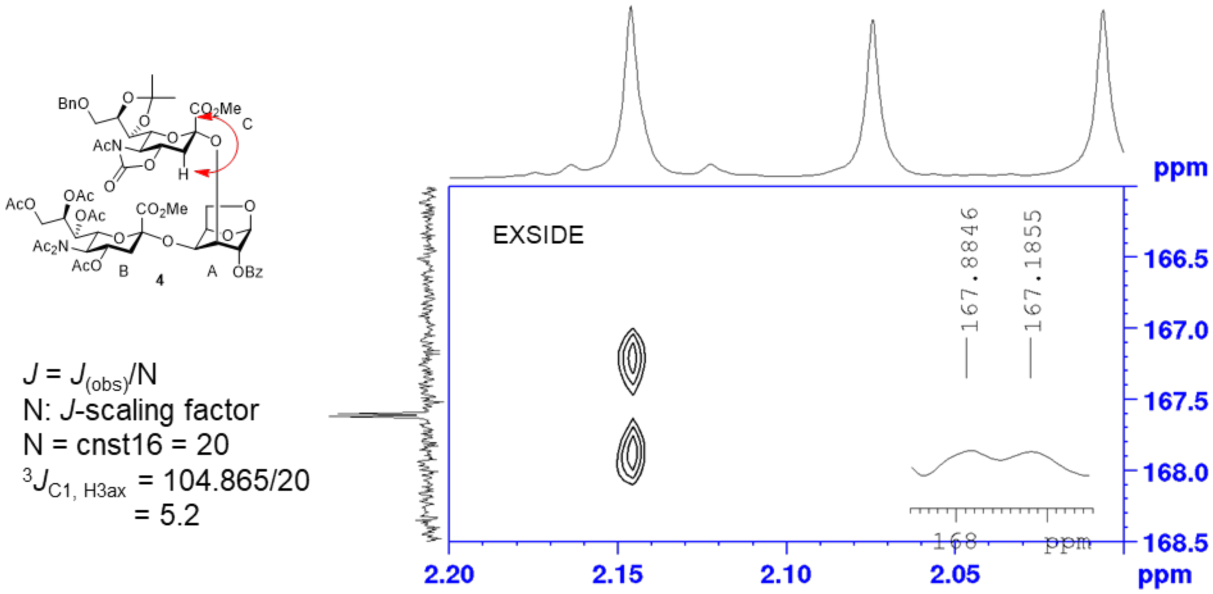

Figure S2. EXSIDE Spectrum of compound **4** (part C)

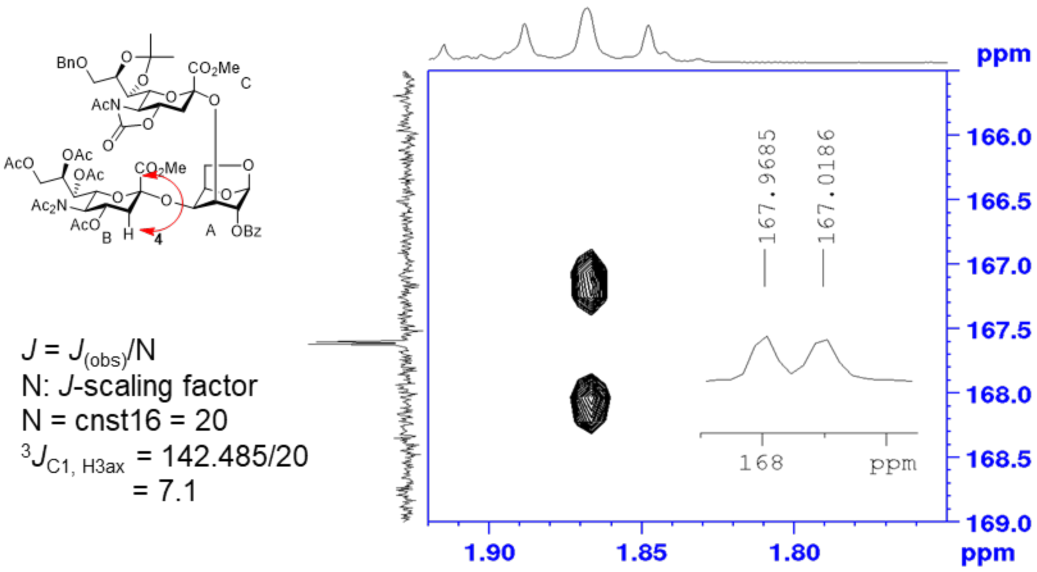

Figure S3. EXSIDE Spectrum of compound **4** (part B)

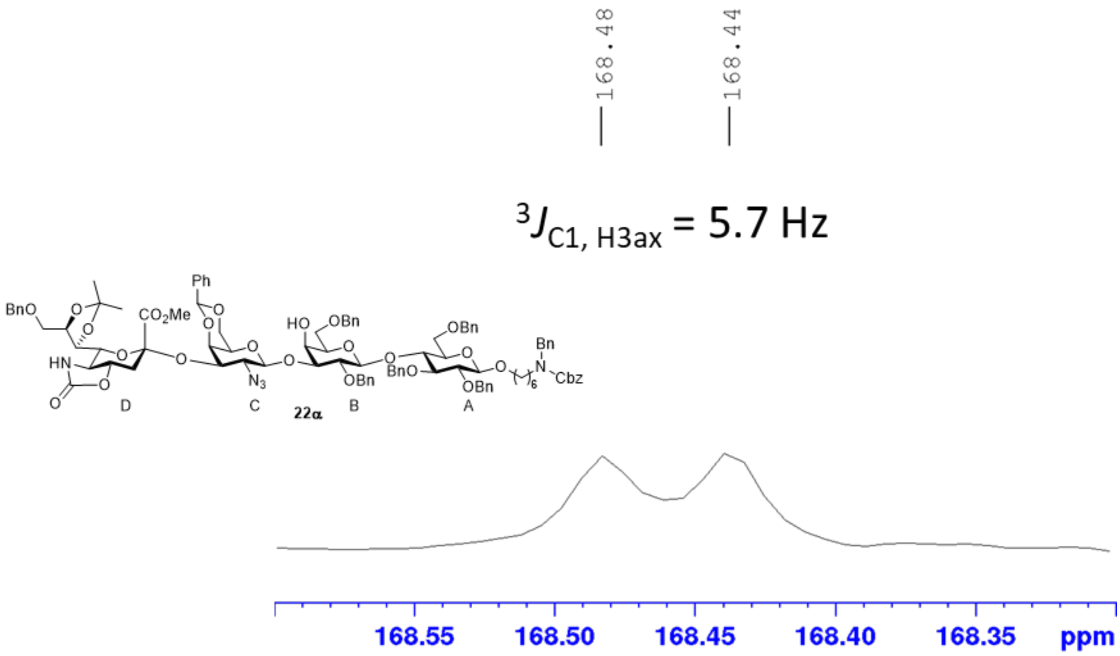

Figure S4. C-H coupled ^{13}C Spectrum (Sialyl OMe Decoupled) of compound **22α**

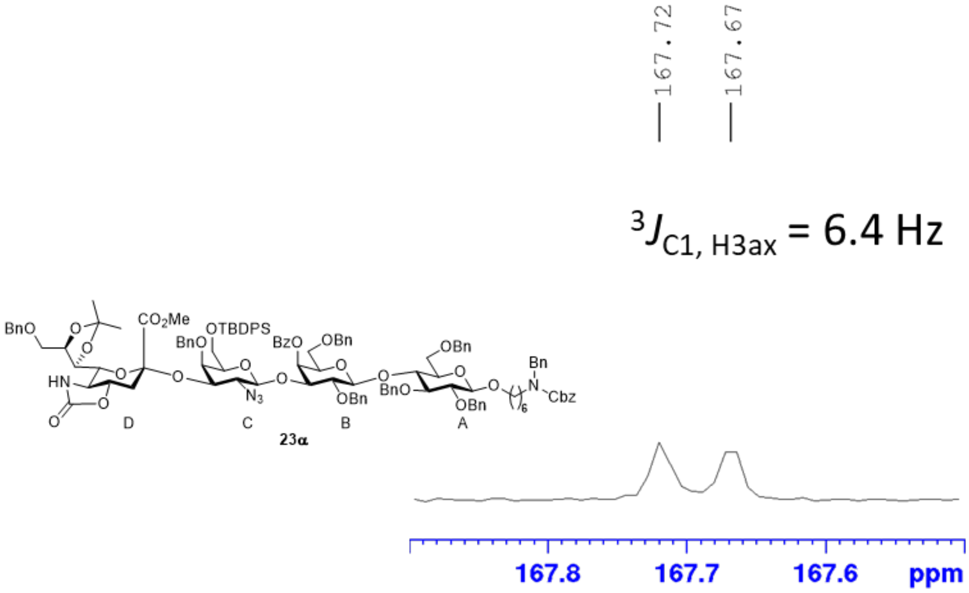

Figure S5. C-H coupled ^{13}C Spectrum (Sialyl OMe Decoupled) of compound **23α**

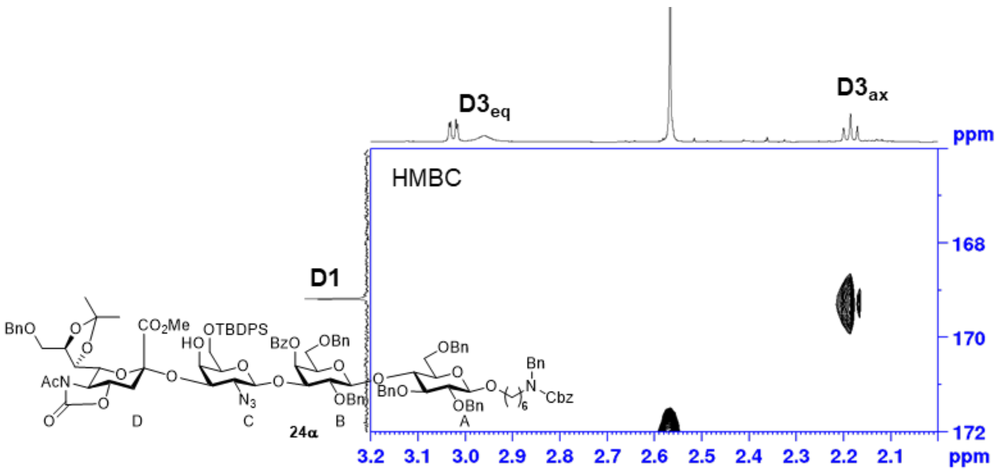

Figure S6. HMBC Spectrum of compound **24α**

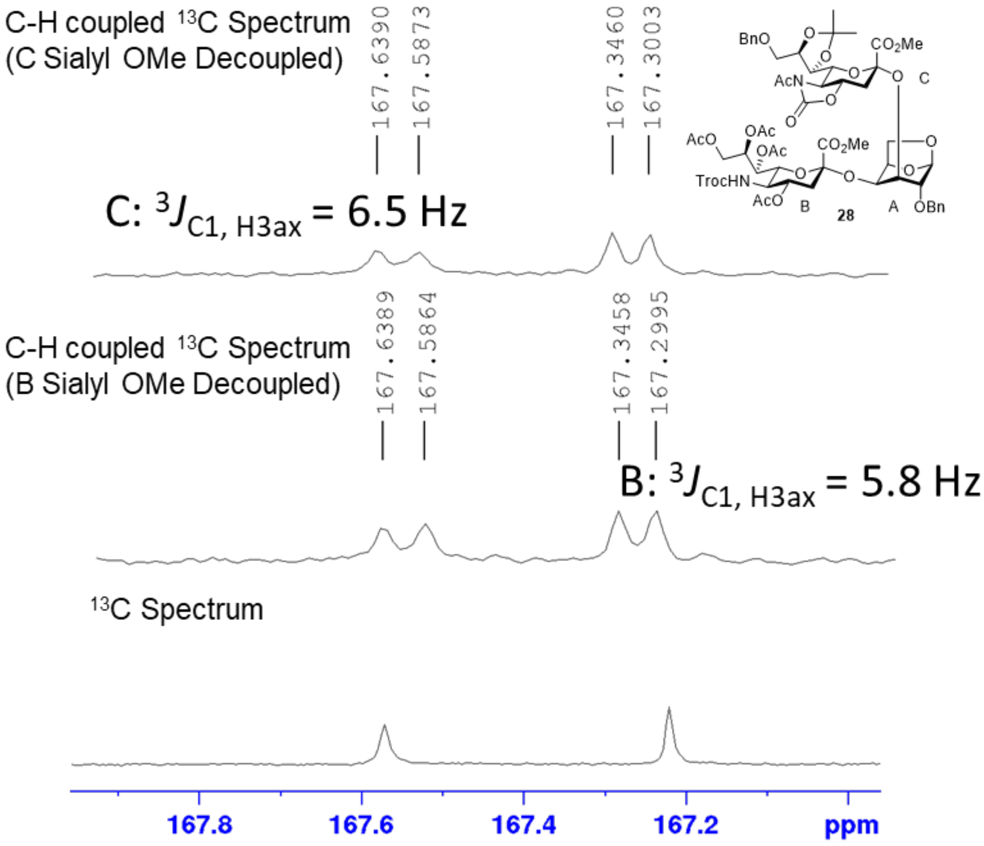

Figure S7. C-H coupled ^{13}C Spectrums (Sialyl OMe Decoupled) of compound **28**

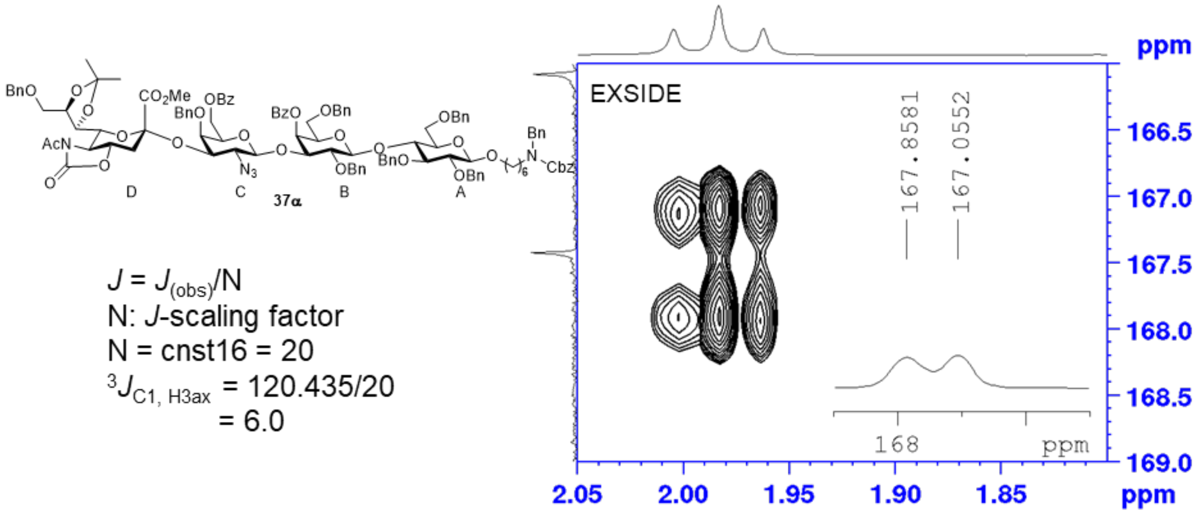

Figure S8. EXSIDE Spectrum of compound **37α**

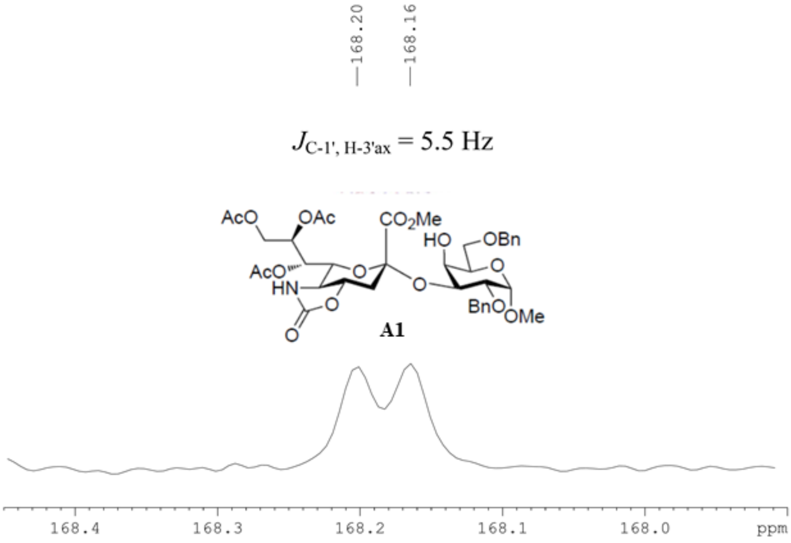

Figure S9. C-H coupled ^{13}C Spectrum (Sialyl OMe Decoupled) of compound **A1**

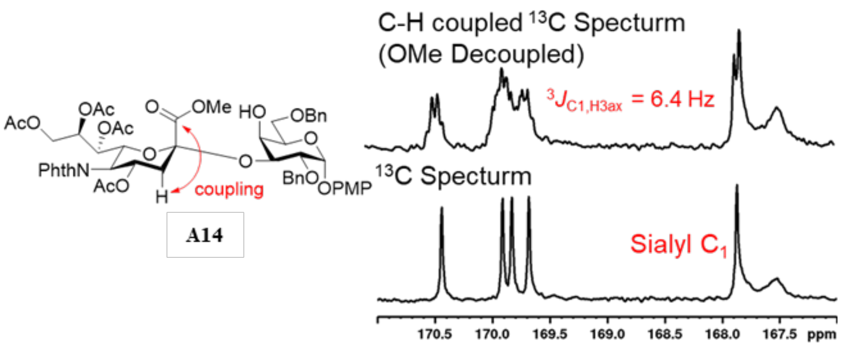

Figure S10. C-H coupled ^{13}C Spectrum (Sialyl OMe Decoupled) of compound **A14**

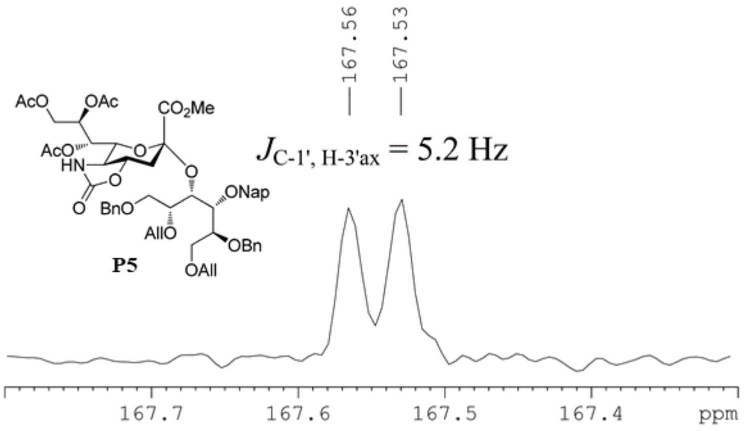

Figure S11. C-H coupled ^{13}C Spectrum (Sialyl OMe Decoupled) of compound **P5**

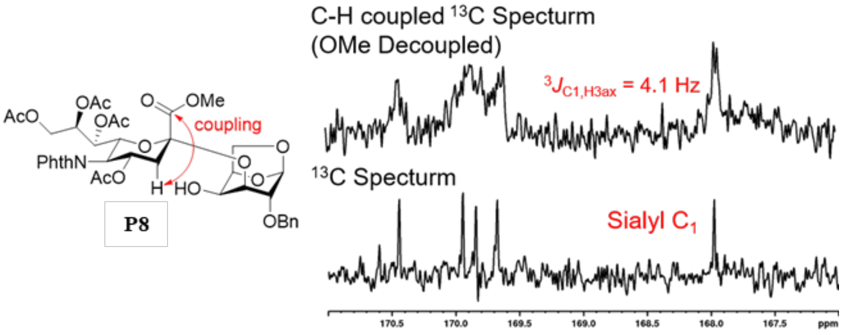

Figure S12. C-H coupled ^{13}C Spectrum (Sialyl OMe Decoupled) of compound **P8**

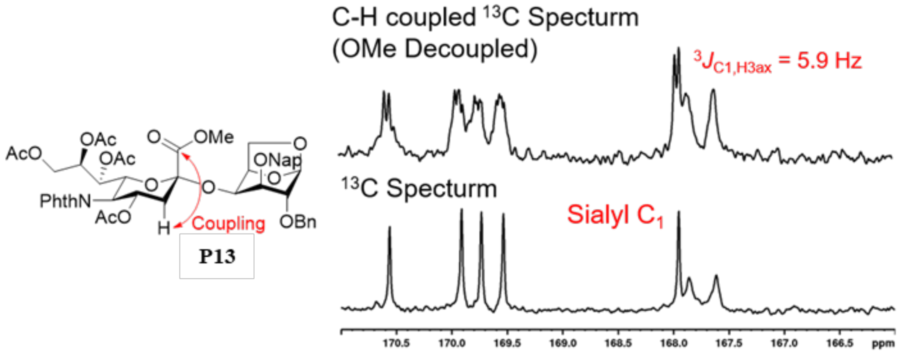

Figure S13. C-H coupled ^{13}C Spectrum (Sialyl OMe Decoupled) of compound **P13**

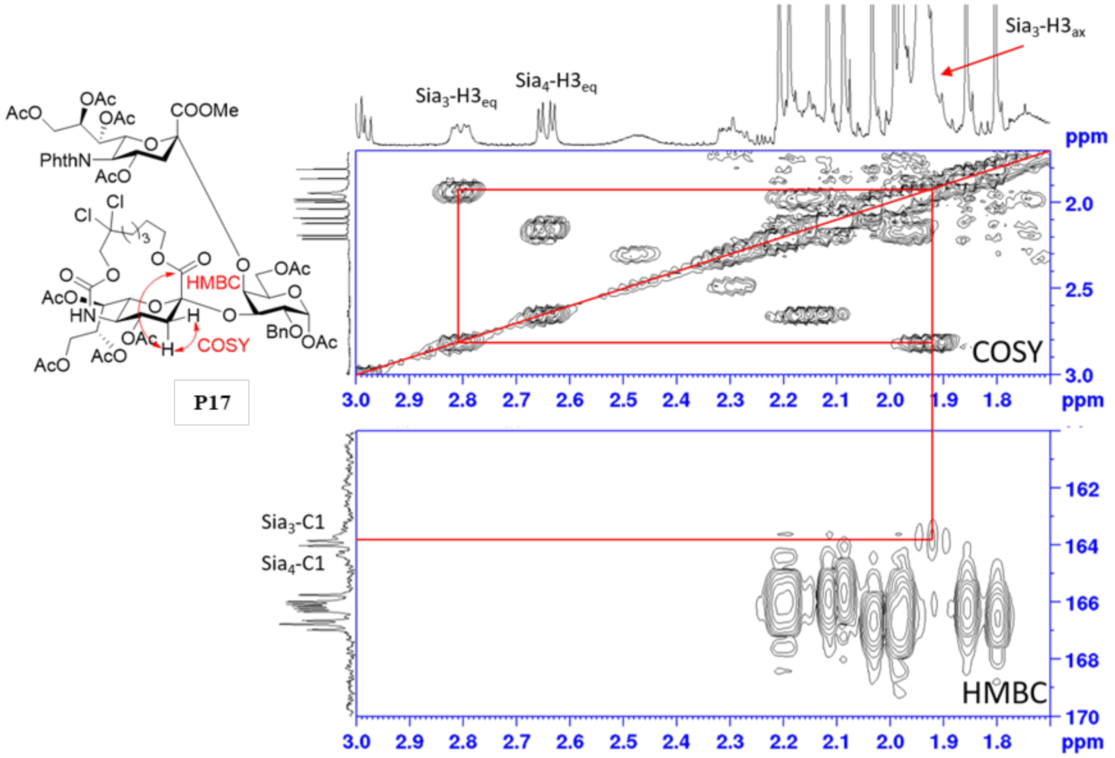

Figure S14. COSY and HMBC Spectrums of compound **P17**

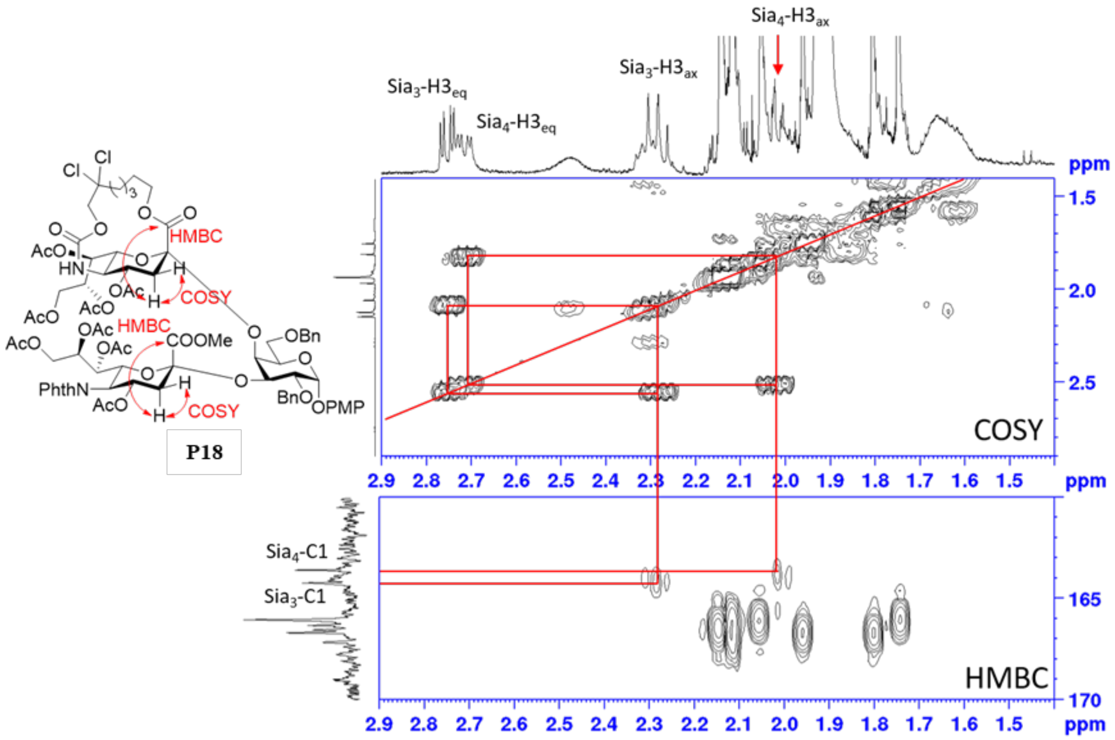

Figure S15. COSY and HMBC Spectrums of compound **P18**

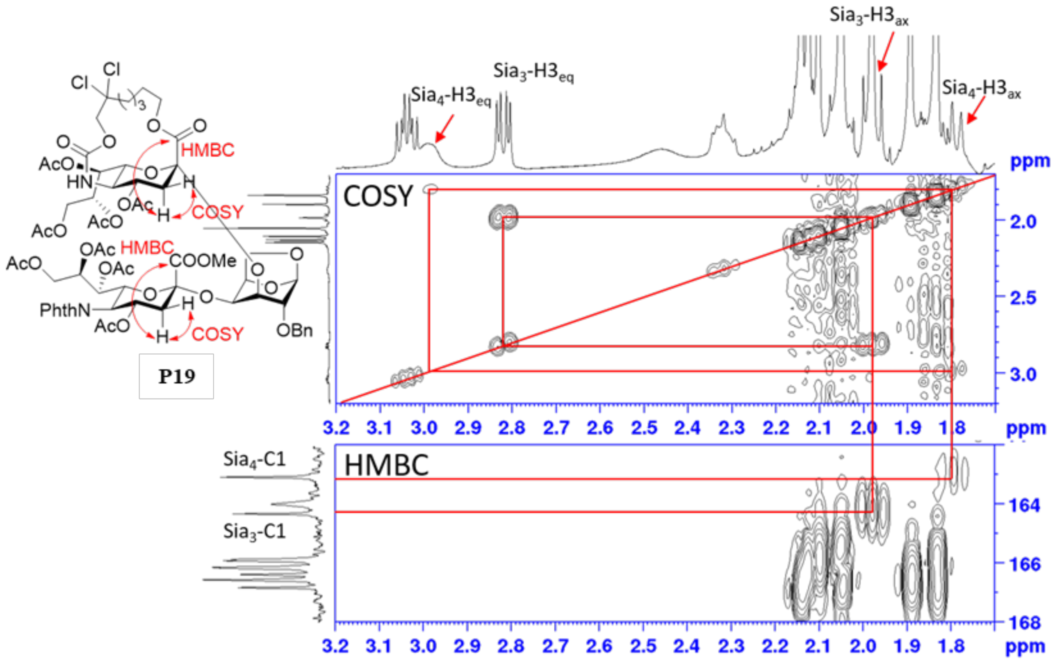

Figure S16. COSY and HMBC Spectrums of compound **P19**

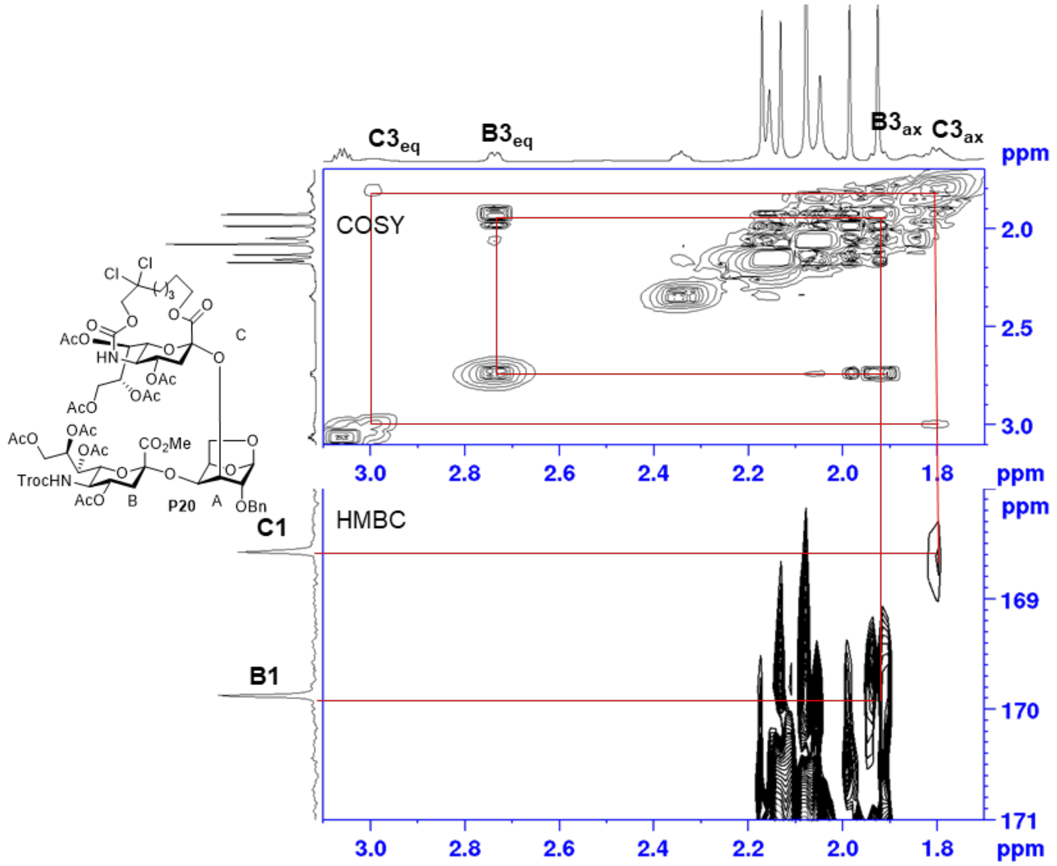

Figure S17. COSY and HMBC Spectrums of compound **P20**

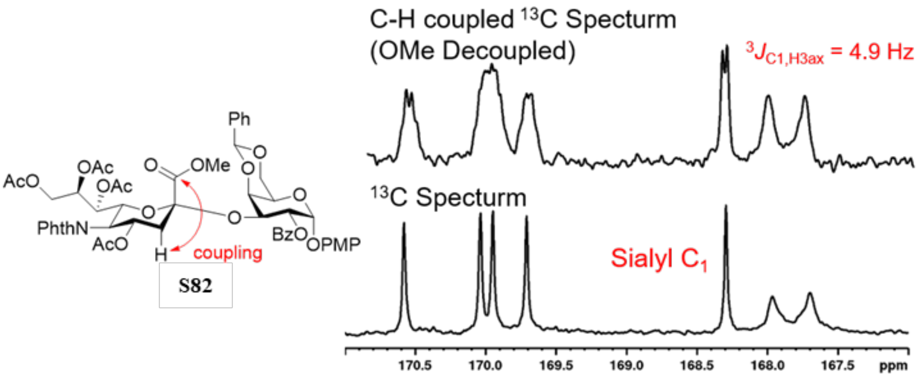

Figure S18. C-H coupled ^{13}C Spectrum (Sialyl OMe Decoupled) of compound **S82**

Experimental Sections.

Reagents and solvents

1. Commercially available reagents were purchased as reagent grade from Merck, TCI, Acros, Fluka, Alfa, Nova, BLD and Aldrich. Reagents were used without further purification.

2. All solvents were dried and distilled by standard techniques. Dichloromethane (DCM), acetonitrile (MeCN), and propionitrile (EtCN) were distilled over calcium hydride. Tetrahydrofuran (THF) was distilled over sodium metal/benzophenone. Methanol (MeOH) was distilled over magnesium and iodine.

3. Flash column chromatography was carried out on Silical Gel 60 (230-400 mesh, E. Merck). TLC was performed on pre-coated glass plates of Silical Gel 60 F254 (0.25 mm, E. Merck) and visualized under UV (254 nm) and by staining with acidic ceric ammonium molybdate, ninhydrin or *p*-anisaldehyde.

4. Reactions at -20 °C to -78 °C were performed in low-temperature reactor PANCHUM Verycool UR-8500.

Spectrum Notes

1. ^{1}H and ^{13}C NMR spectra were recorded by Varian MR-500 or Bruker Avance-400 spectrometer at ambient temperature. ^{1}H, ^{13}C, ^{13}C (C-H coupled), COSY, HSQC, HMBC, EXSIDE and HSQC-TOCSY NMR spectra were obtained by using Bruker Avance-400, Avance-500, Avance 600 and Avance-850. All NMR chemical shifts (δ) are referenced in ppm relative to residual solvent. ^{1}H NMR and ^{13}C NMR chemical shifts were reported in parts per million (δ ppm) units referenced to residual solvent peaks as internal standards ($CDCl_3$ 7.24 ppm, CD_3OD 3.31 ppm, D_2O 4.79 ppm CD_3NO_2 4.34 ppm, $(CD_3)_2CO$ 2.05 ppm, and CD_3CN 1.94 ppm in ^{1}H NMR, C_5D_5N 150.35 ppm, $CDCl_3$ 77.0 ppm, CD_3NO_2 62.9 ppm, CD_3OD 49.0 ppm, and CD_3CN 1.32 ppm in ^{13}C NMR). Carbon NMR spectra are proton decoupled. NMR spectral data are reported as chemical shift (multiplicity, coupling constants (*J*), integration). Multiplicity is reported as follows: singlet (s), doublet (d), doublet of doubles (dd), triplet (t), quartet (q), broad band (br) and multiplet (m) and coupling constant (*J*) in Hertz (Hz).

2. High resolution mass spectra (HRMS) were recorded under ESI-TOF and matrix assisted laser desorption ionization (MALDI) were recorded under Voyager DE-PRO. (Applied Biosystem Houston).

General pretreatment for glycosylation reaction

A glycosyl acceptor and a sialyl donor were combined in a flask, and the mixture was azeotroped three times using dry toluene. The mixture was then dried under vacuum for 2 h. Molecular sieve 3Å, 4 Å or AW-300 were heated at 200 °C under vacuum for overnight and activated by flame under reduced pressure prior to use in the reaction.

General glycosylation procedure A for thioglycoside donors

A solution of glycosyl acceptor (1~4 eq.), thioglycoside donor (1~10 eq.), and activated molecular sieve (100 mg per 1 mL of solvent) in DCM/ACN or EtCN (50 mM) was stirred under an argon atmosphere at rt for 30 min. The solution was then cooled to between 0 °C and -78 °C, followed by the addition of NIS (1.2 eq. per 1 eq. of donor) and slow addition of TfOH (0.15 eq. per 1 eq. of donor). After full consumption of the donor, as confirmed by TLC, the reaction was quenched with Et_3N. The solid was filtered through a pad of Celite®, and the resulting solution was concentrated under reduced pressure. The residue was dissolved in EtOAc and washed with saturated $Na_2S_2O_{3(aq)}$, H_2O, and brine. The organic layer was dried over $MgSO_4$, filtered, and concentrated. The residue was then purified by silica gel chromatography.

General glycosylation procedure B for glycosyl phosphate or imidate donors

A solution of glycosyl acceptor (1 eq.), glycosyl phosphate or imidate donor (1~10 eq.), and activated molecular sieve (100 mg per 1 mL of solvent) in DCM/ACN or EtCN (50 mM) was stirred under an argon atmosphere at rt for 30 min. The solution was then cooled to between -20 °C and - 78 °C, followed by the slow addition of TMSOTf (1.2 eq. per 1 eq. of donor for phosphate donor, 0.15 eq. per 1 eq. of donor for imidate donor). Once the donor was fully consumed, as judged by TLC, the reaction was quenched with Et_3N. The solid was filtered through a pad of Celite®, and the resulting solution was concentrated under reduced pressure. The residue was dissolved in EtOAc and washed with H_2O and brine. The organic layer was dried over $MgSO_4$, filtered, and concentrated. The residue was then purified by silica gel chromatography.

Synthesis procedures and characterization

Compound **D1**[1], **D2**[1], **D3**[2], **D4**[1], **D5**[3], **D6**[4], **D11**[5], **S10**[6], **S11**[7], **S15**[8], **S16**[9], **S18**[10], **S45**[11], **S48**[12], **S50**[2], **S51**[13], **S54**[2], **S56**[14], **S57**[13], **S59**[15], **S60**[16], **S65**[17], **S67**[18], **S72**[19], **S86**[7], and **S89**[12] were previously reported.

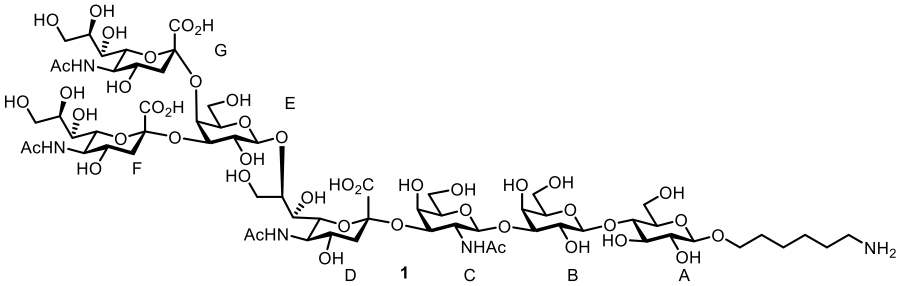

Compound 1. To a solution of compound **39** (40 mg, 12 μmole) in ACN/AOH/Ac_2O (3.0 ml/1.0 ml/1.0 ml) was added zinc dust (240 mg, 3.69 mmole) at rt. The mixture was stirred for 20 hours, then filtered through a pad of celite®. The filtrate was concentrated under reduced pressure. The residue was dissolved in ethanol (3 mL), followed by the addition of 5 M $LiOH_{(aq)}$ (1.5 mL) at rt. The reaction mixture was gradually warmed to 80 °C and stirred for 24 hours. After cooling to 0 °C, Ac_2O (1 mL) was added, and the mixture was allowed to warm to rt. After stirring for 1 hour, the solution was concentrated under reduced pressure. The residue was dissolved in a mixture of H_2O and AcOH (4 mL / 2 mL) and stirred under a H_2 atmosphere in the presence of palladium on carbon (40 mg, 10 wt%). After 16 hours, the mixture was filtered through celite®, neutralized with $NaHCO_{3(aq)}$, and concentrated under reduced pressure. The crude product was purified by activated carbon chromatography (elution: H_2O → H_2O/ACN = 3:2 → H_2O/ACN = 3:2 + 0.1% TFA), followed by size-exclusion chromatography (P-2 Bio-Gel, eluent: H_2O + 0.4% NH_4HCO_3). The desired fractions were combined, concentrated, and lyophilized to give compound **1** (13 mg, 61% over 4 steps); R_f = 0.14 in *n*-PrOH/H_2O/$NH_{3(aq)}$ = 6:2:1. ^{1}H NMR (850 MHz, D_2O/C_5D_5N = 95:5) δ 4.69 (d, J = 8.8 Hz, 1H, C-1), 4.68 (d, J = 8.2 Hz, 1H, E-1), 4.43-4.42 (m, 2H, A-1, B-1), 4.34 (d, J = 2.6 Hz, 1H, E-4), 4.24 (dd, J = 11.1, 2.7 Hz, 1H, C-3), 4.14 (dd, J = 10.5, 2.6 Hz, 1H, E-3), 4.14 (d, J = 2.6 Hz, 1H, B-4), 4.09 (dd, J = 12.2, 2.6 Hz, 1H, D-9), 4.03 (ddd, J = 6.5, 4.7, 2.6 Hz, 1H, D-8), 3.99-3.97 (m, 2H, C-2, C-4), 3.96 (d, J = 12.0 Hz, 1H, A-6), 3.91 (d, J = 4.7 Hz, 1H, D-7), 3.90-3.87 (m, 3H,

D-9, F-8, F-9), 3.86-3.83 (m, 5H, OCH_2CH$_2$, B-6, F-5, G-8, G-9), 3.81-3.79 (m, 3H, A-6, D-5, G-5), 3.76-3.74 (m, 4H, C-6, B-6, E-6, F-6), 3.73-3.71 (m, 3H, B-3, C-6, E-6), 3.71-3.66 (m, 9H, B-5, C-5, D-4, D-6, E-2, E-5, F-4, F-9, G-4), 3.65-3.64 (m, 1H, F-7), 3.62-3.59 (m, 4H, OCH_2CH$_2$, A-3, A-4, B-2, G-9), 3.54-3.52 (m, 3H, A-5, G-6, G-7), 3.29 (dd, J = 8.7, 8.7 Hz, 1H, A-2), 2.95 (t, J = 7.3 Hz, 2H, CH_2NH$_2$), 2.84 (dd, J = 12.5, 4.6 Hz, 1H, F-3$_{eq}$), 2.64-2.62 (m, 2H, D-3$_{eq}$, G-3$_{eq}$), 2.19 (dd, J = 12.2, 12.2 Hz, 1H, G-3$_{ax}$), 2.06 (s, 3H, Ac), 2.01 (s, 9H, Ac), 1.84 (dd, J = 12.5, 12.5 Hz, 1H, F-3$_{ax}$), 1.62-1.58 (m, 5H, OCH$_2$CH_2, CH_2CH$_2$NH$_2$, D-3$_{ax}$), 1.34 (br, 4H); ^{13}C NMR (213 MHz, D$_2$O/C$_5$D$_5$N = 95:5) δ 177.31 (x3), 177.04, 176.58, 175.91, 175.18, 105.58 (x2), 105.28, 104.34, 103.30, 103.23, 102.19, 84.95, 83.93, 80.77, 77.70, 77.40, 77.23, 77.18, 77.08, 76.81, 75.42, 75.39, 75.28, 75.13, 74.67, 74.28, 74.04, 72.66, 72.47, 72.27, 71.87, 71.81, 71.54, 70.77, 70.70, 70.69, 70.56, 70.27, 69.40, 65.14, 64.78, 63.85, 63.38, 63.32, 63.30, 62.40, 54.74, 54.07, 54.05, 53.36, 42.09, 41.93, 41.69, 41.20, 30.73, 28.88, 27.53, 26.80, 24.67, 24.60, 24.34 (x2); HRMS (ESI) *m/z* for $C_{65}H_{110}N_5O_{45}$ [M+H]$^+$: calcd 1680.6473, found 1680.6461.

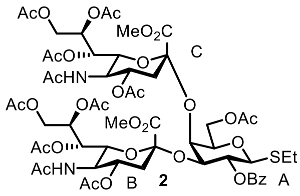

Compound 2. To a solution of compound **35** (90 mg, 0.071 mmol) in DCM (4 mL) was added K_2CO_3 (19.5 mg, 141 mmol) and trichloroacetonitrile (71 μL, 0.71 mmol) at rt. After being stirred for 16 h, the solid was filtered through a pad of celite®, and resulting solution was concentrated under reduced pressure to obtain the glycosyl donor **36**. Next, compound **2** was synthesized from above glycosyl donor **36** and ethanethiol (76 μL, 1.4 mmol) by following the **General glycosylation procedure B**. The residue was purified by silica gel chromatography (DCM/Hexanes/MeOH = 6/4/0.5, 5 times,

R_f = 0.28) to afford compound **2** (93 mg, 94% over 2 steps). ^{1}H NMR (500 MHz, $CDCl_3$) δ 8.06 (d, *J* = 7.5 Hz, 2H), 7.52 (t, *J* = 7.5 Hz, 1H), 7.41 (t, *J* = 7.5 Hz, 2H), 5.52-5.50 (m, 1H, B-8), 5.44, (d, *J* = 9.3 Hz, 1H, C_5-NH), 5.38-5.35 (m, 1H, C-8), 5.35 (dd, *J* = 10.5, 10.5 Hz, 1H, A-2), 5.26 (d, *J* = 9.4 Hz, 1H, C-7), 5.15 (d, *J* = 10.1 Hz, 1H, B_5-NH), 5.12 (dd, *J* = 9.8, 2.1 Hz, 1H, B-7), 4.98 (ddd, *J* = 10.9, 9.2, 4.5 Hz, 1H, C-4), 4.79 (ddd, *J* = 12.5, 4.5, 4.5 Hz, 1H, B-4), 4.74 (d, *J* = 10.5 Hz, 1H, A-1), 4.40 (d, *J* = 10.5 Hz, 1H, A-3), 4.32 (dd, *J* = 11.9, 2.9 Hz, 1H, A-6), 4.21 (dd, *J* = 12.7, 1.7 Hz, 1H, C-9), 4.20-4.16 (m, 1H, A-6), 4.14 (d, *J* = 12.5 Hz, 1H, B-9), 4.05 (dd, *J* = 12.7, 4.5 Hz, 1H, C-9), 3.97 (s, 3H, $CO_2C\mathit{H}_3$), 3.95-3.90 (m, 3H, A-4, C-5, C-6), 3.85 (dd, *J* = 12.5, 5.4 Hz, 1H, B-9), 3.78-3.72 (m, 1H, B-5), 3.74 (s, 3H, $CO_2C\mathit{H}_3$), 3.67 (dd, *J* = 6.5, 2.9 Hz, 1H, A-5), 3.63 (dd, *J* = 10.7, 2.1 Hz, 1H), 2.69 (qd, *J* = 12.7, 7.4 Hz, 1H, $SC\mathit{H}_2CH_3$), 2.57 (qd, *J* = 12.7, 7.4 Hz, 1H, $SC\mathit{H}_2CH_3$), 2.52 (dd, *J* = 12.5, 4.5 Hz, 1H, B-3_{eq}), 2.50 (dd, *J* = 12.8, 4.5 Hz, 1H, C-3_{eq}), 2.14 (s, 3H, Ac), 2.05 (s, 6H, Ac), 2.05-2.02 (m, 1H, C-3_{ax}), 2.02 (s, 3H, Ac), 2.00 (s, 3H, Ac), 1.96 (s, 3H, Ac), 1.93 (s, 3H, Ac), 1.88 (s, 3H, Ac), 1.85 (s, 3H, Ac), 1.77 (dd, *J* = 12.5, 12.5 Hz, 1H, B-3_{ax}), 1.74 (s, 3H, Ac), 1.38 (s, 3H, Ac), 1.18 (t, *J* = 7.4 Hz, 3H, $SCH_2C\mathit{H}_3$); ^{13}C NMR (125 MHz, $CDCl_3$) δ 170.70, 170.61 (x2), 170.59, 170.52, 170.30, 170.24, 170.21, 169.95, 169.36, 169.17, 168.03, 167.67, 165.50, 132.95, 130.36, 130.21, 128.29, 98.59, 97.62, 83.05, 76.10, 73.85, 72.29, 71.66, 70.63, 69.55, 69.25, 68.85, 68.20, 67.28, 66.83, 66.35, 64.83, 62.17, 61.81, 53.21, 52.95, 49.64, 48.52, 37.30, 36.69, 23.40, 23.06, 23.04, 21.34, 20.88 (x2), 20.66 (x2), 20.56 (x2), 20.43, 20.14, 14.89; HRMS (ESI) *m/z* for $C_{57}H_{76}N_2NaO_{31}S$ $[M+Na]^+$: calcd 1339.4050, found 1339.4048.

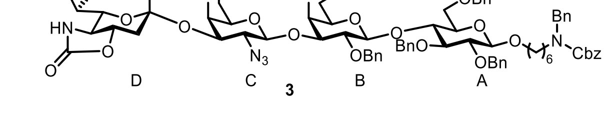

3

Compound 3. To a solution of compound **38** (35 mg, 17 μmol) in DCM/DMF (3 mL/3 mL) was added hydrazine acetate (16 mg, 173 μmol) under a nitrogen atmosphere at rt. After being stirred for 2.5 h, the reaction mixture was extracted with EtOAc, and the resulting organic solution was washed with 1N $HCl_{(aq)}$, saturated $NaHCO_{3(aq)}$, and brine. The organic layer was dried over $MgSO_4$, filtered, and concentrated. The residue was purified by silica gel chromatography (EtOAc/Hexanes = 1/1, R_f = 0.16) to afford compound **3** (29 mg, 85%). ^{1}H NMR (400 MHz, $CDCl_3$) δ 8.03 (d, *J* = 7.2 Hz, 2H), 7.89 (d, *J* = 7.2 Hz, 2H), 7.53-7.47 (m, 2H), 7.37-7.15 (m, 46H), 7.03 (t, *J* = 7.4 Hz, 1H), 7.92 (t, 7.6 Hz, 2H), 5.75 (br, 1H, D_5-NH), 5.66 (d, *J* = 3.2 Hz, 1H, B-4), 5.16 (br, 2H, Cbz), 5.01 (d, *J* = 10.6 Hz, 1H), 4.86-4.83 (m, 3H), 4.78 (d, *J* = 11.2 Hz, 1H), 4.76 (d, *J* = 10.7 Hz, 1H), 4.70 (d, *J* = 11.0 Hz, 1H), 4.58 (d, *J* = 7.1 Hz, 1H), 4.56-4.53 (m, 2H), 4.51-4.47 (m, 6H), 4.37-4.28 (m, 4H), 4.19 (d, *J* = 11.9 Hz, 1H), 4.00 (t, *J* = 9.3 Hz, 1H), 3.96-3.81 (m, 4H), 3.80-3.69 (m, 4H), 3.71 (s, 3H, OCH_3), 3.67-3.63 (m, 2H), 3.62-3.51 (m, 7H), 3.43-3.36 (m, 4H), 3.34-3.28 (m, 2H), 3.24 (br, 1H, CH_2NBnCbz), 3.16 (br, 1H, CH_2NBnCbz), 3.03 (d, *J* = 4.2 Hz, 1H), 2.97-2.93 (m, 2H), 2.12 (dd, *J* = 12.7, 12.7 Hz, 1H, D-3_{ax}), 1.58-1.47 (m, 4H), 1.40-1.28 (m, 4H); ^{13}C NMR (125 MHz, $CDCl_3$) δ 168.34, 165.94, 165.32, 159.59, <u>156.71</u>, <u>156.12</u>, 138.82, 138.58, 138.30, 138.13, 138.08, 137.89, 137.50, 136.76, 133.21, 132.86, 129.92, 129.77, 129.48, 128.44, 128.38, 128.33, 128.25, 128.19, 128.17, 127.87, 127.80, 127.67, 127.48, 127.35, 127.06, 103.54, 102.27, 101.77, 100.04, 82.58, 81.51, 80.20, 77.76, 77.51, 76.33, 75.52, 75.22, 75.12, 74.96, 74.89, 74.66, 74.34, 73.51, 73.40 (x2), 73.14, 73.03, 71.97, 71.91, 70.92, 70.36, 69.77, 69.68, 68.25, 67.97, 67.08, 62.58, 62.35, 57.52, 53.31, <u>50.37</u>, <u>50.06</u>, <u>47.08</u>, <u>46.15</u>, 35.64, 29.58, <u>27.99</u>, <u>27.59</u>, 26.54, 25.83 (conformational isomer peaks are underlined); HRMS (ESI) *m/z* for $C_{113}H_{121}N_5NaO_{27}$ $[M+Na]^+$: calcd 2002.8147, found 2002.8142.

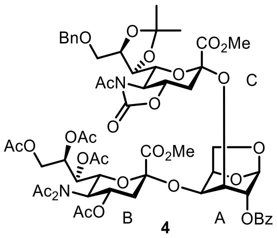

Compound 4. Compound 4 was synthesized from glycosyl acceptor **7** (200 mg, 0.26 mmol) and glycosyl donor **5** (322 mg, 0.51 mmol) by following the **General glycosylation procedure A**. The mixture was purified by silica gel chromatography (EtOAc/Hexanes = 1/1 + 5% MeOH, R_f = 0.43) to afford compound **4** (273 mg, 86%). ^{1}H NMR (600 MHz, $CDCl_3$) δ 8.11 (d, *J* = 7.1 Hz, 2H), 7.51 (t, *J* = 7.4 Hz, 1H), 7.39 (t, *J* = 7.7 Hz, 2H), 7.27-7.22 (m, 3H), 7.17 (d, *J* = 6.8 Hz, 2H), 5.58 (br, 1H, A-1), 5.51 (ddd, *J* = 11.6, 10.2, 5.2 Hz, 1H, B-4), 5.24 (ddd, *J* = 8.8, 4.2, 2.9 Hz, 1H, B-8), 5.17 (br, 1H, A-2), 5.12 (dd, *J* =8.8, 1.7 Hz, 1H, B-7), 4.87-4.86 (m, 1H, C-7), 4.86 (dd, *J* = 10.2, 1.7 Hz, 1H, B-6), 4.47 (dd, *J* = 5.3, 3.6 Hz, 1H, A-4), 4.41 (d, *J* = 11.8 Hz, 1H, CH_2Ph), 4.38 (d, *J* = 7.1 Hz, 1H, A-6), 4.35 (d, *J* = 11.8 Hz, 1H, CH_2Ph), 4.35-4.32 (m, 1H, C-8), 4.28 (dd, *J* = 5.3, 1.2 Hz, 1H, A-3), 4.19-4.18 (m, 1H, A-5), 4.17 (dd, *J* = 10.2, 10.2 Hz, 1H, B-5), 4.12 (dd, *J* = 12.5, 2.9 Hz, 1H, B-9), 4.02 (dd, *J* = 12.5, 4.2 Hz, 1H, B-9), 3.96-3.91 (m, 2H, C-5, C-6), 3.86-3.84 (m, 2H, C-4, C-9), 3.85 (s, 3H, OCH_3), 3.80 (s, 3H, OCH_3), 3.67 (dd, *J* = 10.1, 5.9 Hz, 1H, C-9), 3.60 (dd, *J* = 7.1, 5.2 Hz, 1H, A-6), 3.16 (dd, *J* = 11.6, 3.4 Hz, 1H, C-3_{eq}), 2.78 (dd, *J* = 12.8, 5.2 Hz, 1H, B-3_{eq}), 2.39 (s, 3H, Ac), 2.35 (s, 3H, Ac), 2.28 (s, 3H, Ac), 2.11 (dd, *J* = 13.3, 11.6 Hz, 1H, C-3_{ax}), 2.10 (s, 3H, Ac), 2.04 (s, 3H, Ac), 1.97 (s, 3H, Ac), 1.90 (s, 3H, Ac), 1.83 (dd, *J* = 12.8, 11.6 Hz, 1H, B-3_{ax}), 1.46 (s, 3H, CCH_3), 1.29 (s, 3H, CCH_3); ^{13}C NMR (150 MHz, $CDCl_3$) δ 174.23, 173.62, 172.44, 170.38, 169.78, 169.68, 169.59, 167.53 (x2), 164.73, 153.79, 137.99, 133.19, 129.95, 129.48, 128.33, 128.29, 127.66, 127.62, 108.79, 101.12, 98.80, 98.45, 76.38, 76.04, 75.68, 75.18, 73.54, 73.30, 73.16, 73.13, 69.94, 68.75, 68.00, 66.73 (x2), 66.45, 64.30, 61.29, 60.28, 56.71, 53.13, 52.97, 38.21,

36.56, 27.91, 26.67, 26.04, 25.15, 24.92, 20.93, 20.69, 20.65, 20.50; HRMS (ESI) *m/z* for $C_{58}H_{70}N_2NaO_{28}$ $[M+Na]^+$: calcd 1265.4013, found 1265.4006.

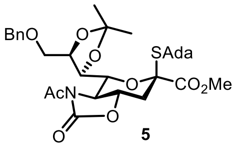

Compound 5. To a solution of compound **19** (455 mg, 0.77 mmol) in DCM (8 mL) was added *N,N*-diisopropylethylamine (2.2 mL, 12.39 mmol) and acetyl chloride (0.44 mL, 6.19 mmol) under a nitrogen atmosphere at 0 °C. After being stirred for 60 min at 0 °C, the reaction mixture was quenched with MeOH and concentrated *in vacuo*. The residue was extracted with EtOAc, and the resulting organic solution was washed with saturated $NaHCO_{3(aq)}$ and brine. The organic layer was dried over $MgSO_4$, filtered, and concentrated. The residue was purified by silica gel chromatography (EtOAc/Hexanes = 1/3, R_f = 0.30) to afford compound **5** (512 mg, quant). ^{1}H NMR (400 MHz, $CDCl_3$) δ 7.36-7.26 (m, 5H), 4.78 (dd, *J* = 7.8, 5.7 Hz, 1H, H7), 4.51 (dd, *J* = 8.6, 7.8 Hz, 1H, H-6), 4.49 (d, *J* = 11.2 Hz, 1H, C*H*2Ph), 4.44 (ddd, *J* = 12.7, 11.3, 3.7 Hz, 1H, H-4), 4.38 (d, *J* = 11.2 Hz, 1H, C*H*2Ph), 4.21 (ddd, *J* = 9.3, 5.7, 4.8 Hz, 1H, H-8), 3.78 (s, 3H, OC*H*3), 3.64 (dd, *J* = 11.3, 8.6 Hz, 1H, H-5), 3.48 (dd, *J* = 9.3, 9.3 Hz, 1H, H-9), 3.43 (dd, *J* = 9.3, 4.8 Hz, 1H, H-9), 2.71 (dd, *J* = 12.7, 3.7 Hz, 1H, H-3_{eq}), 2.27 (s, 3H, Ac), 2.13 (dd, *J* = 12.7, 12.7 Hz, 1H, H-3_{ax}), 2.03-1.97 (m, 6H, SAda), 1.88-1.85 (m, 3H, SAda), 1.63 (m, br, 6H, SAda), 1.33 (s, 3H, CC*H*3), 1.29 (s, 3H, CC*H*3); ^{13}C NMR (100 MHz, $CDCl_3$) δ 173.63, 170.16, 153.37, 137.14, 128.53, 128.37, 128.14, 107.82, 85.76, 76.86, 75.25, 74.56, 73.88, 73.35, 69.14, 62.34, 52.81, 50.92, 43.52 (x2), 39.21, 35.84 (x2), 29.76 (x2), 27.30, 25.20, 24.42; HRMS (ESI) *m/z* for $C_{33}H_{43}NNaO_9S$ $[M+Na]^+$: calcd 652.2556, found 652.2556.

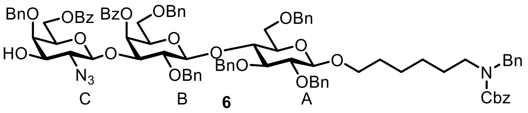

Compound 6. To a solution of compound **S26** (267 mg, 0.14 mmol) in THF (3 mL) was added tetrabutylammonium fluoride trihydrate (262 mg, 0.83 mmol) at rt. After being stirred at rt for 5 h, the reaction was added H_2O (1 mL), K_2CO_3 (364 mg, 1.39 mmol), 1 *H*-imidazole-1-sulfonyl azide HCl salt (48 mg, 0.28 mmol) and $CuSO_4{\cdot}5H_2O$ (10 mg, 69 μmol) at rt. The reaction was stirred at rt for 1 h, and then concentrated *in vacuo*. The residue was extracted with EtOAc, and the resulting organic solution was washed with 1N $HCl_{(aq)}$, saturated $NaHCO_{3(aq)}$, and brine. The organic layer was dried over $MgSO_4$, filtered, and concentrated. The residue was purified by silica gel chromatography (EtOAc/Hexanes = 1/3, R_f = 0.13) to afford compound **6** (209 mg, 94% over 2 steps). ^{1}H NMR (500 MHz, $CDCl_3$) δ 8.06 (d, *J* = 7.3 Hz, 2H), 7.95 (d, *J* = 7.3 Hz, 2H), 7.51 (t, *J* = 7.4 Hz, 2H), 7.39-7.15 (m, 41H), 7.03 (t, *J* = 7.4 Hz, 1H), 6.92 (t, *J* = 7.6 Hz, 2H), 5.67 (d, *J* = 2.8 Hz, 1H, B-4), 5.16 (br, 1H, Cbz), 5.15 (br, 1H, Cbz), 5.03 (d, *J* = 10.7 Hz, 1H), 4.89 (d, *J* = 10.6 Hz, 1H), 4.86 (d, *J* = 11.5 Hz, 1H), 4.78-4.74 (m, 4H), 4.71 (d, *J* = 10.9 Hz, 1H), 4.66 (d, *J* = 7.9 Hz, 1H), 4.60 (dd, *J* = 11.0, 6.1 Hz, 1H, C-6), 4.56 (d, *J* = 12.2 Hz, 1H), 4.50-4.45 (m, 3H), 4.42-4.35 (m, 4H), 4.20 (d, *J* = 11.8 Hz, 1H), 4.02 (dd, *J* = 9.4, 9.4 Hz, 1H, A-4), 3.90-3.80 (m, 2H), 3.86 (dd, *J* = 9.8, 3.2 Hz, 1H), 3.78-3.77 (m, 2H), 3.71-3.67 (m, 2H), 3.60-3.57 (m, 2H), 3.49-3.45 (m, 2H), 3.42-3.36 (m, 3H), 3.33-3.30 (m, 2H), 3.24-3.16 (m, 2H, C*H*$_2$NBnCbz), 2.28 (d, *J* = 7.1 Hz, 1H, C_3-OH), 1.59-1.45 (m, 4H), 1.35-1.21 (m, 4H); ^{13}C NMR (125 MHz, $CDCl_3$) δ 165.91, 165.37, 156.69, 156.11, 138.83, 138.60, 138.22, 138.13, 138.10, 138.08, 137.87, 137.60, 136.82, 136.74, 133.25, 132.89, 129.92, 129.81, 129.54, 129.52, 128.55, 128.45, 128.43, 128.38, 128.31, 128.24, 128.21, 128.18, 128.03, 128.00, 127.89, 127.84, 127.80, 127.75, 127.67, 127.57, 127.48, 127.46, 127.36, 127.31, 127.16, 127.07, 103.55, 102.44, 102.30, 82.59, 81.53, 80.48, 76.92, 76.36, 75.33, 75.25, 75.08, 74.90, 74.89, 73.54, 73.19, 73.13, 72.45, 72.28, 70.71, 69.79, 68.25, 68.03, 67.07, 64.62, 62.46, 50.38, 50.08, 47.08, 46.13, 29.60, 28.00, 27.61, 26.55, 25.84

(conformational isomer peaks are underlined); HRMS (ESI) *m/z* for $C_{95}H_{100}N_4NaO_{19}$ $[M+Na]^+$: calcd 1623.6879, found 1623.6876.

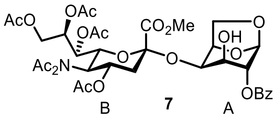

7

Compound 7. To a solution of compound **32** (83 mg, 0.11 mmol) in EtOAc (11 mL) was stirred under a H_2 atmosphere in the presence of palladium on carbon (25 mg, 10 wt%) at rt. After being stirred for 4 h, the solid was filtered through a pad of celite®, and resulting solution was concentrated under reduced pressure. Next, to a solution of above residue in DMF/pyridine (2 mL/2 mL) was added benzoyl chloride (0.13 mL, 1.08 mmol) and 4-(dimethylamino)pyridine (13 mg, 0.11 mmol) under a nitrogen atmosphere at rt. After being stirred for 30 min, the reaction mixture was quenched with MeOH and concentrated *in vacuo*. The residue was extracted with EtOAc, and the resulting organic solution was washed with 1N $HCl_{(aq)}$, saturated $NaHCO_{3(aq)}$, and brine. The organic layer was dried over $MgSO_4$, filtered, and concentrated. Next, to a solution of above residue in DCM/TFA (3 mL/6 mL) was stirred at rt for 2 h. The reaction mixture was extracted with EtOAc, and the resulting organic solution was washed with H_2O, saturated $NaHCO_{3(aq)}$, and brine. The organic layer was dried over $MgSO_4$, filtered, and concentrated. The residue was purified by silica gel chromatography (EtOAc/Hexanes = 3/2, R_f = 0.26) to afford compound **7** (68 mg, 81% over 3 steps). ^1H NMR (500 MHz, $CDCl_3$) δ 8.07 (d, *J* = 7.7 Hz, 2H), 7.55 (t, *J* = 7.7 Hz, 1H), 7.43 (t, *J* = 7.7 Hz, 2H), 5.53 (br, 1H, A-1), 5.47 (ddd, *J* = 6.2, 6.2, 5.2 Hz, 1H, B-4), 5.11 (br, 1H, A-2), 5.09 (br, 1H, B-8), 5.02 (d, *J* = 7.6 Hz, 1H, B-7), 4.91 (d, *J* = 10.4 Hz, 1H, B-6), 4.54 (dd, *J* = 6.3, 6.3 Hz, 1H, A-4), 4.36 (d, *J* = 7.3 Hz, 1H, A-6), 4.32-4.26 (m, 3H, A-3, A-5, B-5), 4.16 (d, *J* = 12.5 Hz, 1H, B-9), 3.84 (s, 3H, OCH_3), 3.78 (dd, *J* = 12.5, 5.3 Hz, 1H, B-9), 3.71 (dd, *J* = 7.3, 7.3 Hz, 1H, A-6), 3.22 (s, 1H, A_3-OH), 2.80 (dd, *J* = 14.7, 5.2 Hz, 1H, B-3_{eq}), 2.40 (s, 3H, Ac), 2.30 (s, 3H, Ac), 2.10 (dd, *J* = 14.7,

6.2 Hz, 1H, B-3_{ax}), 2.04 (m, 6H, Ac(x2)), 2.02 (s, 3H, Ac), 1.87 (s, 3H, Ac); ^{13}C NMR (125 MHz, $CDCl_3$) δ 174.28, 173.04, 170.16, 169.99, 169.66, 169.63, 168.21, 165.21, 133.30, 129.83, 129.37, 128.38, 99.83, 99.54, 72.71, 72.59, 70.09, 69.58, 69.41, 69.12, 68.80, 66.73, 64.31, 61.33, 56.86, 53.19, 36.25, 27.75, 25.97, 20.79 (x2), 20.51, 20.43; HRMS (ESI) *m/z* for $C_{35}H_{43}NNaO_{19}$ $[M+Na]^+$: calcd 804.2327, found 804.2328.

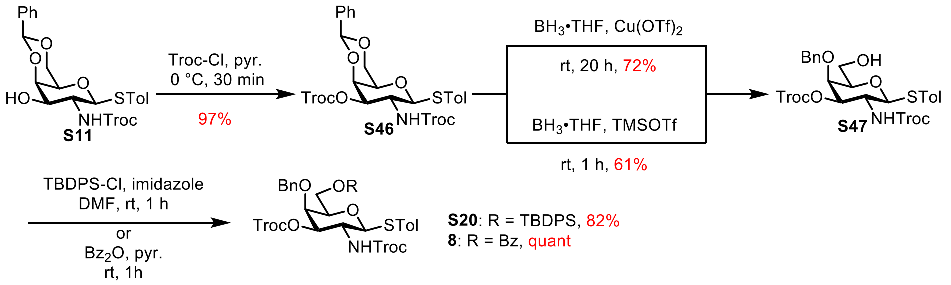

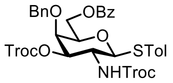

8

Compound 8. To a solution of compound **S11** (334 mg, 0.61 mmol) in pyridine (6 mL) was added 2,2,2-trichloroethoxycarbonyl chloride (0.17 mL, 1.22 mmol) under a nitrogen atmosphere at 0 °C. After being stirred for 30 min at 0 °C, the reaction mixture was quenched with MeOH and concentrated *in vacuo*. The residue was extracted with EtOAc, and the resulting organic solution was washed with 1N $HCl_{(aq)}$, saturated $NaHCO_{3(aq)}$, and brine. The organic layer was dried over $MgSO_4$, filtered, and concentrated. The residue was purified by silica gel chromatography (EtOAc/Hexanes = 1/1, R_f = 0.62) to afford compound **S46** (427 mg, 97%). To a solution of compound **S46** (230 mg, 0.32 mmol) in THF (3 mL) was added borane tetrahydrofuran (1.5 mL, 1.50 mmol) under nitrogen atmosphere at rt. After being stirred for 30 min, and then copper (II) triflate (5.7 mg, 15 μmol) was added to the solution. After being stirred for 20 h, the reaction mixture was quenched with MeOH and concentrated *in vacuo*. The residue was extracted with EtOAc, and the resulting organic solution was washed with

saturated $NaHCO_{3(aq)}$ and brine. The organic layer was dried over $MgSO_4$, filtered, and concentrated. The residue was purified by silica gel chromatography (EtOAc/Hexanes/DCM) = 1/1/1, R_f = 0.50) to afford compound **S47** (165 mg, 72%). To a solution of compound **S47** (679 mg, 0.94 mmol) in pyridine (2 mL) was added benzoic anhydride (423 mg, 1.87 mmol) under a nitrogen atmosphere at rt. After being stirred for 60 min, the reaction mixture was concentrated *in vacuo*. The residue was extracted with EtOAc, and the resulting organic solution was washed with 1N $HCl_{(aq)}$, saturated $NaHCO_{3(aq)}$, and brine. The organic layer was dried over $MgSO_4$, filtered, and concentrated. The residue was purified by silica gel chromatography (EtOAc/Hexanes = 1/2, R_f = 0.58) to afford compound **8** (871 mg, quant). ^1H NMR (500 MHz, $CDCl_3$) δ 7.95 (d, *J* = 7.5 Hz, 2H), 7.58 (t, *J* = 7.5 Hz, 1H), 7.43 (t, *J* = 7.5 Hz, 2H), 7.38 (d, *J* = 7.9 Hz, 2H), 7.31-7.28 (m, 4H), 7.26-7.24 (m, 1H), 6.93 (d, *J* = 7.9 Hz, 2H), 5.31 (d, *J* = 9.6 Hz, 1H, N*H*), 5.23 (d, *J* = 9.6 Hz, 1H, H-3), 5.06 (d, *J* = 9.6 Hz, 1H, H-1), 4.82 (d, *J* = 11.3 Hz, 1H), 4.79 (d, *J* = 11.9 Hz, 1H), 4.72-4.69 (m, 3H), 4.60 (d, *J* = 11.9 Hz, 1H), 4.52 (dd, *J* = 11.0, 6.2 Hz, 1H, H-6), 4.35 (dd, *J* = 11.0, 6.2 Hz, 1H, H-6), 4.07 (br, 1H, H-4), 3.95 (ddd, *J* = 9.6, 9.6, 9.6 Hz, 1H, H-2), 3.91 (dd, *J* = 6.2, 6.2 Hz, 1H, H-5), 2.25 (s, 3H, STol); ^{13}C NMR (125 MHz, $CDCl_3$) δ 166.01, 153.73, 153.54, 137.91, 137.07, 133.19, 132.63, 129.64, 129.59, 129.44, 128.67, 128.36, 128.34, 128.24, 127.95, 95.37, 94.09, 86.20, 78.06, 76.78 ,75.71, 74.85, 74.28, 72.79, 63.20, 51.53, 21.05; HRMS (ESI) *m/z* for $C_{33}H_{31}Cl_6NNaO_9S$ $[M+Na]^+$: calcd 849.9748, found 849.9757.

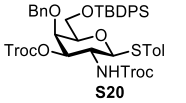

S20

Compound S20. The synthesis of compound **S47** was described in synthesis of compound **8**. To a solution of compound **S47** (292 mg, 0.40 mmol) in DMF (4 mL) was added imidazole (110 mg, 1.62 mmol) at rt under a nitrogen atmosphere. The reaction

mixture was stirred for 30 min, and then tert-butyl(chloro)diphenylsilane (210 μL, 0.81 mmol) was added to the solution. After being stirred for 60 min, the reaction was quenched with MeOH and then concentrated *in vacuo*. The residue was extracted with EtOAc, and the resulting organic solution was washed with saturated $NaHCO_{3(aq)}$, and brine. The organic layer was dried over $MgSO_4$, filtered, and concentrated. The residue was purified by silica gel chromatography (EtOAc/Hexanes = 1/2, R_f = 0.63) to afford compound **S20** (321 mg, 82%). ^{1}H NMR (400 MHz, $CDCl_3$) δ 7.65-7.61 (m, 4H), 7.47-7.22 (m, 14H), 6.98 (d, J = 7.8 Hz, 2H), 5.37 (d, J = 10.4 Hz, 1H, N*H*), 5.20 (d, J = 7.6 Hz, 1H, H-3), 5.08 (d, J = 10.0 Hz, 1H, H-1), 4.81-4.67 (m, 5H), 4.60 (d, J = 11.2 Hz, 1H), 4.21 (br, 1H, H-4), 3.89-3.80 (m, 3H, H-2, H-6, H-6), 3.69 (dd, J = 6.2, 6.2 Hz, 1H, H-5), 2.28 (s, 3H, STol), 1.06 (s, 9H, C(C*H*$_3$)$_3$); ^{13}C NMR (100 MHz, $CDCl_3$) δ 153.58, 153.52, 137.94, 137.82, 135.52, 135.46, 132.95, 132.84, 132.79, 129.85, 129.79, 129.65, 128.31, 128.17, 127.79, 127.76, 127.68, 127.54, 95.39, 94.23, 85.32, 78.30, 78.04, 76.79, 74.90, 74.31, 73.12, 61.85, 51.51, 26.82 (x3), 21.10, 19.12; HRMS (ESI) *m/z* for $C_{42}H_{45}Cl_6NNaO_8SSi$ $[M+Na]^+$: calcd 984.0664, found 984.0654.

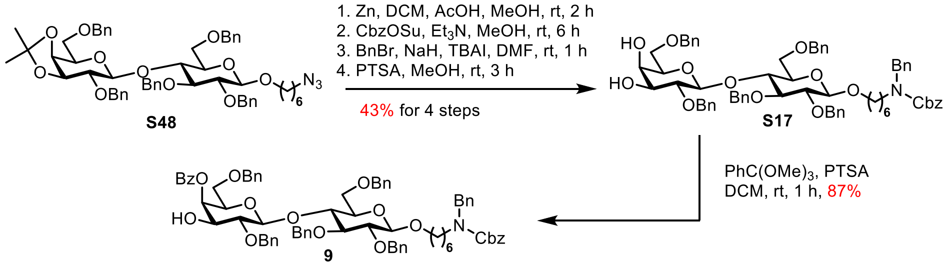

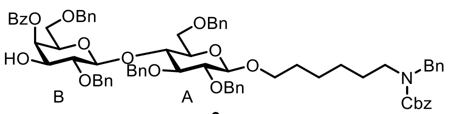

9

Compound 9. To a solution of compound **S17** (1.05 g, 0.94 mmol) in DCM (19 mL) was added *p*-toluenesulfonic acid (49 mg, 0.28 mmol) and trimethyl orthobenzoate (243 μL, 1.42 mmol) under a nitrogen atmosphere at rt. After being stirred at rt for 1h, the

reaction was added H_2O (1 mL) at rt. After being stirred at rt for 5 min, the reaction was quenched with Et_3N and concentrated *in vacuo*. The residue was extracted with EtOAc, and the resulting organic solution was washed with saturated $NaHCO_{3(aq)}$, and brine. The organic layer was dried over $MgSO_4$, filtered, and concentrated. The residue was purified by silica gel chromatography (EtOAc/Hexanes = 1/2, R_f = 0.25) to afford compound **9** (1.00 g, 87%). ^1H NMR (500 MHz, $CDCl_3$) δ 7.96 (d, *J* = 7.4 Hz, 2H), 7.51 (t, *J* = 7.4 Hz, 1H), 7.36-7.21 (m, 27H), 7.15-7.10 (m, 7H), 7.06 (t, *J* = 7.2 Hz, 2H), 5.57 (d, *J* = 3.0 Hz, 1H, B-4), 5.16 (br, 1H, Cbz), 5.14 (br, 1H, Cbz), 5.05 (d, *J* = 10.7 Hz, 1H), 4.86 (d, *J* = 10.7 Hz, 1H), 4.80 (d, *J* = 11.2 Hz, 1H), 4.78 (d, *J* = 10.7 Hz, 1H), 4.70 (d, *J* = 10.7 Hz, 1H), 4.67 (d, *J* = 11.2 Hz, 1H), 4.60 (d, *J* = 12.1 Hz, 1H), 4.52 (d, *J* = 7.8 Hz, 1H, B-1), 4.46 (d, *J* = 10.7 Hz, 1H), 4.42 (d, *J* = 12.1 Hz, 1H), 4.39 (d, *J* = 12.0 Hz, 1H), 4.35 (d, *J* = 7.6 Hz, 1H, A-1), 4.19 (d, *J* = 12.0 Hz, 1H), 4.02 (dd, *J* = 9.3, 9.3 Hz, 1H, A-4), 3.88 (br, 1H, OCH_2CH_2), 3.82 (dd, *J* = 10.8, 3.7 Hz, 1H, A-6), 3.74-3.71 (m, 2H, A-6, B-3), 3.61 (dd, *J* = 7.5, 7.5 Hz, 1H, B-5), 3.58 (dd, *J* = 9.3, 9.3 Hz, 1H, A-3), 3.50 (dd, *J* = 7.8, 7.8 Hz, 1H, B-2), 3.52-3.36 (m, 5H, A-2, A-5, B-6, B-6, OCH_2CH_2), 3.23-3.16 (m, 2H, CH_2NBnCbz), 2.41 (d, *J* = 2.4 Hz, 1H, B_3-OH), 1.60-1.19 (m, 8H); ^{13}C NMR (100 MHz, $CDCl_3$) δ 166.30, 156.62, 156.06, 138.95, 138.56, 138.15, 138.08, 137.82, 137.71, 136.73, 133.01, 129.73, 129.60, 128.40, 128.30, 128.24, 128.14, 127.90, 127.77, 127.72, 127.54, 127.43, 127.39, 127.12, 103.54, 102.38, 82.70, 81.54, 80.05, 76.58, 75.20, 74.98 (x2), 74.83, 73.32, 73.09, 72.58, 72.22, 70.26, 69.74, 68.06, 67.37, 67.02, 50.34, 50.04, 47.05, 46.08, 29.52, 27.94, 27.56, 26.49, 25.78 (conformational isomer peaks are underlined); HRMS (ESI) *m/z* for $C_{75}H_{81}NNaO_{14}$ $[M+Na]^+$: calcd 1242.5555, found 1242.5578.

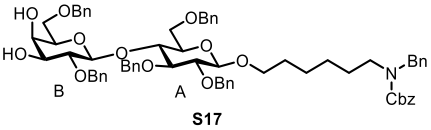

S17

Compound S17. To a solution of compound **S48** (3.66 g, 3.82 mmol) in MeOH/DCM/AcOH (16 mL/16 mL/4 mL) was added zinc dust (5.00 g, 76.48 mmol) at 0 °C. The reaction mixture was gradually warmed to rt and stirred at rt for 2 h. The solid was filtered through a pad of celite®, and resulting solution was concentrated under reduced pressure. Next, a solution of above residue in MeOH/DCM (19 mL/19 mL) was added Et_3N (5.3 mL, 38.20 mmol) and *N*-(benzyloxycarbonyloxy)succinimide (2.86 g, 11.47 mmol) at rt. After being stirred for 6 h, the reaction mixture was concentrated *in vacuo*. The residue was extracted with EtOAc, and the resulting organic solution was washed with saturated $NaHCO_{3(aq)}$ and brine. The organic layer was dried over $MgSO_4$, filtered, and concentrated. The residue was purified by silica gel chromatography. To a solution of above residue in DMF (22 mL) was added NaH (137 mg, 3.42 mmol) at 0 °C under nitrogen atmosphere. The reaction mixture was stirred at 0 °C for 30 min, and then benzyl bromide (0.41 mL, 3.42 mmol) and tetrabutylammonium iodide (168 mg, 0.46 mmol) were added to the solution. The reaction mixture was gradually warmed to rt and stirred for 1 h. The reaction was quenched with MeOH and concentrated *in vacuo*. The residue was extracted with EtOAc, and the resulting organic solution was washed with saturated $NaHCO_{3(aq)}$ and brine. The organic layer was dried over $MgSO_4$, filtered, and concentrated. Next, a solution of above residue in MeOH (20 mL) was added *p*-toluenesulfonic acid (658 mg, 3.82 mmol) at rt. After being stirred for 3 h, the reaction was quenched with Et_3N and concentrated *in vacuo*. The residue was extracted with EtOAc, and the resulting organic solution was washed with saturated $NaHCO_{3(aq)}$ and brine. The organic layer was dried over $MgSO_4$, filtered, and concentrated. The residue was purified by silica gel chromatography (EtOAc/Hexanes = 1/2, R_f = 0.13) to afford compound **S17** (1.84 g, 43% over four steps). ^1H NMR (400 MHz, $CDCl_3$) δ 7.38-7.20 (m, 35H), 5.23 (br, 1H, Cbz), 5.22 (br, 1H, Cbz), 5.06 (d, *J* = 11.0 Hz, 1H), 4.95 (d, *J* = 11.3 Hz, 1H), 4.86 (d,

J = 11.5 Hz, 1H), 4.84 (d, *J* = 11.0 Hz, 1H), 4.77 (d, *J* = 11.3 Hz, 1H), 4.74 (d, *J* = 11.5 Hz, 1H), 4.64 (d, *J* = 12.1 Hz, 1H), 4.54-4.41(m, 7H, A-1, B-1, CH_2Ph (x5)), 4.06 (dd, *J* = 9.3, 9.3 Hz, 1H, A-4), 3.95 (m, 2H, B-4, OCH_2CH_2), 3.87 (dd, *J* = 10.8, 4.0 Hz, 1H, A-6), 3.81 (dd, *J* = 10.8, 1.4 Hz, 1H, A-6), 3.69 (dd, *J* = 10.0, 6.0 Hz, 1H, B-6), 3.65 (dd, *J* = 9.3, 9.3 Hz, 1H, A-3), 3.55 (dd, *J* = 10.0, 6.0 Hz, 1H, B-6), 3.50-3.43 (m, 5H, A-2, A-5, B-2, B-3, OCH_2CH_2), 3.40 (dd, 6.0, 6.0 Hz, 1H, B-5), 3.31 (t, *J* = 6.6 Hz, 1H, CH_2NBnCbz), 3.23 (t, *J* = 6.6 Hz, 1H, CH_2NBnCbz), 2.84 (d, *J* = 3.1 Hz, 1H, B_4-OH), 2.80 (d, *J* = 3.0 Hz, 1H, B_3-OH), 1.68-1.26 (m, 8H); ^{13}C NMR (100 MHz, $CDCl_3$) δ 156.59, 156.02, 138.99, 138.49, 138.28, 138.08, 137.88, 137.74, 136.66, 128.35, 128.27, 128.21, 128.12, 127.88, 127.84, 127.67, 127.64, 127.58, 127.42, 127.07, 103.44, 102.42, 82.62, 81.58, 79.90, 76.43, 75.00, 74.91, 74.69(x2), 73.37, 73.25, 72.98, 72.80, 69.66, 68.64, 68.55, 68.16, 66.99, 50.28, 49.97, 46.99, 46.03, 29.47, 27.87, 27.50, 26.42, 25.72 (conformational isomer peaks are underlined); HRMS (ESI) *m/z* for $C_{68}H_{77}NNaO_{13}$ $[M+Na]^+$: calcd 1138.5293, found 1138.5294.

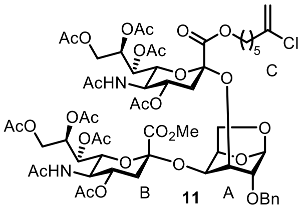

Compound 11. To a solution of compound **P20** (18 mg, 12 μmol) in ACN/AcOH (0.9 mL/0.2 mL) was added zinc dust (317 mg, 4.85 mmol) at rt. After stirring at rt for 24 hours, the solid was filtered through a pad of celite®, and the resulting solution was concentrated under reduced pressure. Next, a solution of above residue in Ac_2O/pyridine (1 mL/2 mL) was stirred at rt for 16 h. The reaction mixture was extracted with EtOAc, and the resulting organic solution was washed with 1N $HCl_{(aq)}$, saturated $NaHCO_{3(aq)}$, and brine. The organic layer was dried over $MgSO_4$, filtered, and concentrated. The residue was purified by silica gel chromatography (EtOAc/Hexanes

= 1/1 + 20% MeOH, R_f = 0.46) to afford compound **11** (15 mg, 96% over 2 steps). ^{1}H NMR (500 MHz, CD_3CN) δ 7.44 (d, *J* = 7.2 Hz, 2H), 7.36 (t, *J* = 7.2 Hz, 2H), 7.29 (t, *J* = 7.2 Hz, 1H), 6.12 (t, *J* =10.1 Hz, 2H), 5.32-5.30 (m, 2H), 5.25-5.20 (m, 5H), 4.96-4.87 (m, 2H), 4.81 (d, *J* = 11.6 Hz, 1H, A_2-Bn), 4.62 (d, *J* = 11.6 Hz, 1H, A_2-Bn), 4.49 (d, *J* = 4.0 Hz, 1H), 4.37-4.31 (m, 2H), 4.29 (t, *J* = 4.5 Hz, 1H), 4.23 (dd, *J* = 12.2, 2.7 Hz, 1H), 4.20 (d, *J* = 7.1 Hz, 1H), 4.12 (dd, *J* = 12.4, 3.1 Hz, 1H), 4.05 (dd, *J* = 12.2, 5.5 Hz, 1H), 4.02-3.95 (m, 2H), 3.93-3.85 (m, 3H), 3.81 (dd, *J* = 10.2, 8.8 Hz, 1H), 3.78 (s, 3H, OCH_3), 3.71 (br, 1H), 3.49 (t, *J* = 6.0 Hz, 1H), 2.74 (dd, *J* = 12.7, 4.8 Hz, 1H), 2.57 (dd, *J* = 13.0, 4.9 Hz, 1H), 2.39 (t, *J* = 7.3 Hz, 2H), 2.05 (s, 3H, Ac), 2.03 (s, 3H, Ac), 2.02 (s, 6H, Ac(x2)), 1.98-1.95 (m, 1H), 1.97 (s, 3H, Ac), 1.95 (s, 3H, Ac), 1.93 (s, 3H, Ac), 1.85 (s, 3H, Ac), 1.76 (s, 6H, Ac(x2)), 1.74-1.71 (m, 3H), 1.63-1.57 (m, 2H), 1.47-1.41 (m, 2H); ^{13}C NMR (125 MHz, CD_3CN) δ 171.36, 171.24, 171.03, 170.96, 170.84, 170.82, 170.69, 170.63, 170.60 (x2), 169.22, 167.64, 143.86, 139.71, 129.28, 128.54, 128.52, 113.08, 100.83, 100.43, 100.32, 81.13, 74.82, 73.29, 73.23, 72.69, 72.62, 70.26, 70.10, 69.62, 69.20, 68.36, 68.23, 67.56, 66.87, 64.79, 62.91, 62.72, 53.80, 49.95, 49.61, 39.59, 39.37, 36.63, 28.93, 27.71, 25.61, 23.19, 23.15, 21.48, 21.26, 21.16, 21.12, 21.11 (x2), 20.89, 20.81; HRMS (ESI) *m/z* for $C_{59}H_{78}ClN_2O_{29}$ $[M-H]^-$: calcd 1313.4379, found 1313.4384.

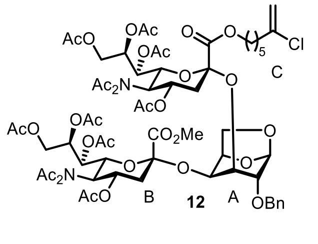

12

Compound 12. To a solution of compound **11** (367 mg, 0.28 mmol) in DCM (30 mL) was added *N*,*N*-diisopropylethylamine (2.43 mL, 13.95 mmol), acetyl chloride (3.98 mL, 55.79 mmol), and 4-(dimethylamino)pyridine (34 mg, 0.28 mmol) under a nitrogen atmosphere at 0 °C. The reaction mixture was gradually warmed rt. After being stirred

for 6 h, the reaction mixture was concentrated *in vacuo*. The residue was extracted with EtOAc, and the resulting organic solution was washed with H_2O, saturated $NaHCO_{3(aq)}$ and brine. The organic layer was dried over $MgSO_4$, filtered, and concentrated. The residue was purified by silica gel chromatography (EtOAc/Hexanes = 1/1 + 10% MeOH, R_f = 0.43) to afford compound **12** (379 mg, 97%). ^{1}H NMR (400 MHz, $CDCl_3$) δ 7.41 (d, *J* = 7.5 Hz, 1H), 7.32 (t, *J* = 7.5 Hz, 2H), 7.26 (t, *J* = 7.5 Hz, 1H), 5.62-5.50 (m, 2H), 5.29-5.25 (m, 2H), 5.15-5.06 (m, 5H), 4.89 (d, *J* = 11.8 Hz, 1H), 4.69-63 (m, 2H), 4.60-4.57 (m, 2H), 4.46-4.39 (m, 2H), 4.30-4.28 (m, 3H), 4.13-4.03 (m, 3H), 3.97-3.89 (m, 3H), 3.79 (s, 3H, OCH_3), 3.78 (br, 1H), 3.59 (t, *J* = 5.9 Hz, 1H), 2.86 (dd, *J* = 12.8, 5.3 Hz, 1H), 2.70 (dd, *J* = 13.0, 5.4 Hz, 1H), 2.34-2.30 (m, 2H), 2.34 (s, 3H, Ac), 2.33 (s, 3H, Ac), 2.30 (s, 3H, Ac), 2.26 (s, 3H, Ac), 2.10 (s, 3H, Ac), 2.07 (s, 3H, Ac), 2.03 (s, 6H, Ac(x2)), 2.01 (s, 3H, Ac), 1.97 (s, 3H, Ac), 1.96 (s, 3H, Ac), 1.87 (s, 3H, Ac), 1.84-1.74 (m, 2H), 1.74-1.69 (m, 2H), 1.63-1.58 (m, 2H), 1.48-1.42 (m, 2H); ^{13}C NMR (100 MHz, $CDCl_3$) δ 174.45 (x2), 173.94, 173.65, 170.42 (x2), 170.21, 169.85, 169.71, 169.68, 169.47 (x2), 167.56, 166.54, 142.90, 138.12, 128.47, 127.83, 127.41, 111.92, 100.04, 100.02, 99.44, 80.50, 74.36, 72.88, 71.21, 69.84, 69.51, 69.19, 68.09, 67.38, 67.12, 66.90, 66.64, 66.51, 66.01, 64.30, 61.58, 61.30, 57.44, 57.19, 52.99, 39.46, 38.77, 35.62, 29.65, 28.30, 27.90, 27.85, 26.84, 25.70, 24.86, 20.98, 20.82 (x2), 20.77 (x2), 20.70, 20.61, 20.49; HRMS (ESI) *m/z* for $C_{63}H_{83}Cl_2N_2O_{31}$ $[M+Cl]^-$: calcd 1433.4357, found 1433.4341.

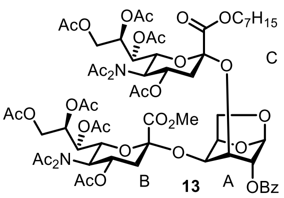

Compound 13. The synthesis of compound **13** is similar as described in the synthesis of compound **S4**, starting from compound **12** (379 mg, 0.27 mmol) to yield compound

13 (279 mg, 74% over 2 steps); EtOAc/Hexanes = 1/1 + 5% MeOH, R_f = 0.43. ^{1}H NMR (400 MHz, $CDCl_3$) δ 8.15 (d, *J* = 7.2 Hz, 2H), 7.53 (t, *J* = 7.2 Hz, 1H), 7.43 (t, *J* = 7.2 Hz, 2H), 5.59-5.48 (m, 3H), 5.30-5.26 (m, 2H), 5.11 (dd, *J* = 8.1, 1.3 Hz, 1H), 5.02 (dd, *J* = 8.9, 0.8 Hz, 1H), 4.96 (ddd, *J* = 8.9, 3.3, 2.4 Hz, 1H), 4.77 (dd, *J* = 10.2, 1.3 Hz, 1H), 4.63 (d, *J* = 5.6 Hz, 1H), 4.59 (dd, *J* = 9.9, 0.8 Hz, 1H), 4.46-4.34 (m, 4H), 4.30 (dd, *J* = 12.6, 2.7 Hz, 1H), 4.22-4.15 (m, 2H), 4.02-3.95 (m, 2H), 3.81 (s, 3H, OCH_3), 3.74 (dd, *J* =12.7, 2.2 Hz, 1H), 3.64 (t, *J* = 6.1 Hz, 1H), 3.59 (dd, *J* = 12.8, 3.8 Hz, 1H), 2.95 (dd, *J* = 12.7, 5.2 Hz, 1H), 2.72 (dd, *J* = 13.1, 5.3 Hz, 1H), 2.36 (s, 3H, Ac), 2.34 (s, 3H, Ac), 2.32 (s, 3H, Ac), 2.24 (s, 3H, Ac), 2.15 (s, 3H, Ac), 2.10 (s, 6H, Ac(x2)), 1.98-1.96 (m, 1H), 1.98 (s, 6H, Ac(x2)), 1.97 (s, 3H, Ac), 1.96 (s, 3H, Ac), 1.82-1.70 (m, 5H), 1.77 (s, 3H, Ac), 1.47-1.36 (m, 2H), 1.36-1.25 (m, 4H), 0.84 (t, *J* = 6.6 Hz, 3H); ^{13}C NMR (100 MHz, $CDCl_3$) δ 174.58, 174.24, 173.81, 173.68, 170.55, 170.23 (x2), 170.14, 169.80, 169.55, 169.35, 169.33, 167.64, 166.14, 165.34, 133.26, 130.08, 129.47, 128.38, 99.21, 99.09, 98.86, 77.20, 74.26, 74.05, 71.73, 69.85, 69.14, 68.97, 68.36, 67.40, 66.88, 66.74, 66.40 (x2), 66.22, 64.37, 61.57, 60.67, 57.21, 57.06, 53.01, 39.34, 36.85, 31.58, 29.66, 28.96, 28.51, 27.95, 27.83, 26.08, 25.80, 25.46, 22.56, 20.99, 20.93, 20.74 (x2), 20.67, 20.45, 14.03; HRMS (ESI) *m/z* for $C_{63}H_{84}N_2NaO_{32}$ $[M+Na]^+$: calcd 1403.4905, found 1403.4904.

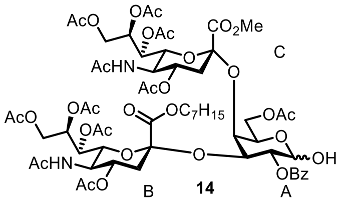

14

Compound 14. To a solution of compound **13** (14 mg, 0.01 mmol) in Ac_2O (1 mL) was added boron trifluoride diethyl etherate (3 μL, 30 μmol) under nitrogen atmosphere at 0 °C. After being stirred at 0 °C for 30 min, the reaction mixture was quenched with Et_3N and concentrated *in vacuo*. The residue was extracted with EtOAc, and the

resulting organic solution was washed with saturated $NaHCO_{3(aq)}$ and brine. The organic layer was dried over $MgSO_4$, filtered, and concentrated. Next, a solution of above residue in DMF (1 mL) was added hydrazine acetate (3.7 mg, 41 μmol) at rt. The reaction mixture was stirred at rt for 30 min, then the reaction was extracted with EtOAc, and the resulting organic solution was washed with 1N $HCl_{(aq)}$, saturated $NaHCO_{3(aq)}$, and brine. The organic layer was dried over $MgSO_4$, filtered, and concentrated. The residue was purified by silica gel chromatography (EtOAc/Hexanes = 1/1 + 10% MeOH, R_f = 0.09) to afford compound **14** as anomeric mixture (13 mg, 91%). ^{1}H NMR (500 MHz, CD_3CN) δ 8.11-8.10 (m, 2H), 7.66-7.61 (m, 1H), 7.57-7.53 (m, 2H), 6.15 (d, *J* = 9.6 Hz, 0.7H), 6.11-6.07 (m, 1H), 6.05 (d, *J* = 9.7 Hz, 0.3H), 5.43-5.39 (m, 1.3H), 5.35 (m, 0.7H), 5.31-5.28 (m, 1.3H), 5.26 (dd, *J* = 10.6, 3.4 Hz, 0.7H), 5.22 (d, *J* = 9.6 Hz, 0.7H), 5.22 (d, *J* = 9.7 Hz, 0.3H), 5.18 (dd, *J* = 8.8, 2.3 Hz, 0.3H), 5.07 (dd, *J* = 10.2, 6.8 Hz, 0.3H), 4.91-4.86 (m, 1.7H), 4.83 (dd, *J* = 10.7, 2.0 Hz, 0.7H), 4.78 (br, 0.6H), 4.76-4.70 (m, 1H), 4.50 (dd, *J* = 10.5, 2.2 Hz, 0.3H), 4.47-4.41 (m, 1.3H), 4.33-4.27 (m, 2.3H), 4.23 (dd, *J* = 8.6, 2.7 Hz, 0.7H), 4.20-4.17 (m, 1H), 4.15-4.03 (m, 5H), 4.00 (dd, *J* = 10.8, 1.3 Hz, 0.7H), 3.96-3.90 (m, 2.3H), 3.83 (dd, *J* = 10.8, 2.4 Hz, 0.3H), 3.81 (s, 1H, OCH_3), 3.78 (s, 2H, OCH_3), 3.73-3.72 (m, 0.6H), 2.43 (dd, *J* = 13.1, 4.6 Hz, 0.3H), 2.35 (dd, *J* = 12.9, 4.6 Hz, 1H), 2.31 (dd, *J* = 12.9, 4.6 Hz, 0.7H), 2.14 (s, 3H, Ac), 2.08 (s, 1H, Ac), 2.07 (s, 2H, Ac), 2.06 (s, 1H, Ac), 2.04 (s, 2H, Ac), 2.03 (s, 1H, Ac), 2.02 (s, 2H, Ac), 2.01 (s, 1H, Ac), 1.98 (s, 2H, Ac), 1.96 (s, 3H, Ac), 1.94 (s, 3H, Ac), 1.94 (s, 3H, Ac), 1.93 (s, 2H, Ac), 1.84 (m, 2H), 1.83-1.79 (m, 2H), 1.77 (s, 3H, Ac), 1.74 (s, 2H, Ac), 1.68 (m, 1.3 H), 1.66-1.63 (m, 1H), 1.62 (s, 1H), 1.35-1.26 (m, 8H), 0.88-0.85 (m, 3H); ^{13}C NMR (125 MHz, CD_3CN) δ 171.55, 171.51, 171.50, 171.46, 171.45, 171.38, 171.34, 171.11, 171.07, 170.95, 170.86, 170.75, 170.74, 170.71, 170.70, 170.64, 170.63, 170.60, 170.50, 170.40, 170.37, 168.98, 168.93, 168.40, 168.32, 166.59, 166.44, 134.40, 134.28, 131.36, 130.98, 130.74, 130.69, 129.71, 129.64,

100.44, 100.21, 99.82, 99.16, 96.09, 91.52, 73.92, 73.45, 73.25, 73.00, 72.90, 72.78, 72.70, 72.39, 70.80, 70.65, 70.55, 70.31, 69.88, 69.80, 69.67, 69.12, 68.98, 68.87, 68.80, 67.98, 67.89, 67.82, 67.63, 67.44, 65.91, 65.83, 63.16, 62.92, 53.81, 53.72, 49.54, 49.45, 49.39, 49.19, 38.68, 38.63, 38.41, 36.99, 36.42, 32.39, 32.37, 29.70, 29.66, 29.27, 29.17, 26.56, 26.48, 23.34, 23.31, 23.25, 23.20, 23.11, 23.08, 21.59, 21.39, 21.38, 21.30, 21.23, 21.19, 21.14, 21.13, 21.07, 21.02, 21.00, 20.97, 20.94, 14.41, 14.39; HRMS (ESI) *m/z* for $C_{61}H_{84}N_2NaO_{32}$ $[M+Na]^+$: calcd 1379.4905, found 1379.4906.

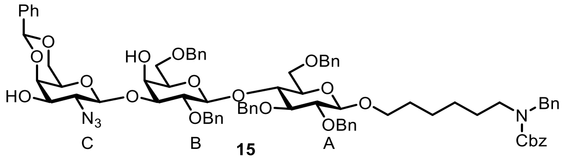

15

Compound 15. The synthesis of compound **15** is similar as described in the synthesis of compound **S14**, starting from compound **S22** (520 mg, 0.34 mmol) to yield compound **15** (209 mg, 45% over 2 steps); EtOAc/Hexanes = 1/1 + 5% MeOH, R_f = 0.63. ^{1}H NMR (400 MHz, $CDCl_3$) δ 7.51-7.49 (m, 2H), 7.42-7.15 (m, 38H), 5.54 (s, 1H, C*H*Ph), 5.15 (br, 2H, Cbz), 5.00 (d, *J* = 10.7 Hz, 1H, A_3-Bn), 4.86 (d, *J* = 11.0 Hz, 1H, A_2-Bn), 4.79 (s, 2H, B_2-Bn), 4.75 (d, *J* = 10.7 Hz, 1H, A_3-Bn), 4.71 (d, *J* = 11.0 Hz, 1H, A_2-Bn), 4.58 (d, *J* = 8.0 Hz, 1H, B-1), 4.54 (d, *J* = 12.1 Hz, 1H, A_6-Bn), 4.49-4.45 (m, 4H, N-Bn (x2), B_6-Bn, C-1), 4.40 (d, *J* = 12.1 Hz, 1H, A_6-Bn), 4.37 (d, *J* = 12.0 Hz, 1H, B_6-Bn), 4.34 (d, *J* = 8.0 Hz, 1H, A-1), 4.25 (dd, *J* = 12.5, 1.0 Hz, 1H, C-6), 4.13 (d, *J* = 3.3 Hz, 1H, C-4), 4.10 (d, *J* = 3.3 Hz, 1H, B-4), 4.03 (dd. *J* = 12.5, 1.6 Hz, 1H, C-6), 3.99 (dd, *J* = 9.0, 9.0 Hz, 1H, A-4), 3.87-3.85 (m, 1H, OC*H*$_2$CH$_2$), 3.77 (dd, *J* = 11.0, 4.2 Hz, 1H, A-6), 3.78-3.64 (m, 4H, A-6, B-2, B-6, C-2), 3.62 (dd, *J* = 9.4, 3.3 Hz, 1H, B-3), 3.56 (dd, *J* = 9.0, 9.0 Hz, 1H, A-3), 3.54-3.34 (m, 6H, OC*H*$_2$CH$_2$, A-2, A-5, B-6, C-3, C-5), 3.43 (dd, *J* = 5.9, 5.9 Hz, 1H, B-5), 3.23-3.16 (m, 2H, C*H*$_2$NBnCbz), 2.62 (d, *J* = 9.8 Hz, 1H, C_3-OH), 2.59 (br, 1H, B_4-OH), 1.59-1.33 (m, 8H); ^{13}C NMR (100 MHz, $CDCl_3$) δ <u>156.67</u>, <u>156.12</u>, 138.98, 138.67, 138.42, 138.35,

138.25, 137.87, 137.15, 136.78, 129.36, 128.44, 128.30, 128.20, 128.00, 127.86, 127.74, 127.60, 127.44, 127.32, 127.21, 126.23, 103.57, 102.40, 102.32, 101.23, 82.69, 81.78, 81.44, 79.30, 76.16, 75.38, 75.21, 75.14, 74.83, 74.37, 73.43, 73.05, 72.87, 71.66, 69.77, 68.86, 68.42(x2), 68.25, 67.07, 66.46, 64.24, 50.37, 50.08, 47.08, 46.14, 29.60, 27.98, 27.60, 26.54, 25.83 (conformational isomer peaks are underlined); HRMS (ESI) *m/z* for $C_{81}H_{90}N_4NaO_{17}$ $[M+Na]^+$: calcd 1413.6199, found 1413.6197.

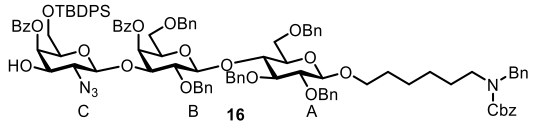

Compound 16. The synthesis of compound **16** is similar as described in the synthesis of compound **S14**, starting from compound **S23** (315 mg, 0.17 mmol) to yield compound **16** (139 mg, 48% over 2 steps); EtOAc/Hexanes = 1/1, $R_f = 0.33$. ^{1}H NMR (400 MHz, $CDCl_3$) δ 8.11 (d, *J* = 7.2 Hz, 2H), 8.02 (d, *J* = 7.1 Hz, 1H), 7.63-7.59 (m ,3H), 7.51-7.42 (m, 6H), 7.37-7.13 (m, 37 H), 7.07 (t, *J* = 7.6 Hz, 2H), 7.03 (t, *J* = 7.3 Hz, 1H), 6.92 (t, *J* = 6.5 Hz, 1), 5.64 (d, *J* = 2.7 Hz, 1H, C-4), 5.62 (d, *J* = 3.6 Hz, 1H, B-4), 5.16 (br, 1H, Cbz), 5.14 (br, 1H, Cbz), 5.02 (d, *J* = 10.6 Hz, 1H), 4.88 (d, *J* = 10.3 Hz, 1H), 4.85 (d, *J* = 10.1 Hz, 1H), 4.77 (d, *J* = 10.6 Hz, 1H), 4.75 (d, *J* = 10.1 Hz, 1H), 4.73 (d, *J* = 7.9 Hz, 1H, C-1), 4.70 (d, *J* = 11.0 Hz, 1H), 4.60 (d, *J* = 12.1 Hz, 1H), 4.48-4.45 (m, 3H), 4.40 (d, *J* = 12.1 Hz, 1H), 4.37 (d, *J* = 10.0 Hz, 1H, A-1), 4.33 (d, *J* = 12.0 Hz, 1H), 4.14 (d, *J* = 12.0 Hz, 1H), 4.01 (dd, *J* = 9.4, 9.4 Hz, 1H, A-4), 3.90-3.69 (m, 8H), 3.65 (ddd, *J* = 10.4, 2.7, 2.7 Hz, 1H, C-3), 3.58 (dd, *J* = 9.4, 9.4 Hz, 1H, A-3), 3.54 (dd, *J* = 6.2, 6.2 Hz, 1H, C-5), 3.45-3.35 (m, 5H), 3.30 (dd, *J* = 10.0, 6.2 Hz, 1H), 3.23 (t, *J* = 6.2 Hz, 1H, CH_2NBnCbz), 3.16 (t, *J* = 6.2 Hz, 1H, CH_2NBnCbz), 2.66 (d, *J* = 2.7 Hz, 1H, C_3-OH), 1.59-1.45 (m, 4H), 1.38-1.24 (m, 4H), 0.94 (s, 9H, $C(CH_3)_3$); ^{13}C NMR (100 MHz, $CDCl_3$) δ 166.79, 165.35, 156.70, 156.12, 138.83, 138.60, 138.05, 137.87, 136.64, 135.46, 135.31, 133.32, 132.88, 132.68, 132.46, 131.88, 130.19, 129.98, 129.74, 129.68, 129.63, 129.31, 128.46, 128.39, 128.22, 128.15, 127.92,

127.83, 127.76, 127.66, 127.60, 127.49, 127.43, 127.34, 127.10, 117.64, 103.57, 102.37, 102.01, 82.60, 81.55, 80.41, 76.58, 76.29, 75.44, 75.30, 75.12, 74.91, 73.43, 73.40, 73.23, 72.81, 71.33, 70.61, 69.81, 69.08, 68.09, 68.02, 67.08, 64.53, 61.04, 50.37, 50.07, 47.09, 46.13, 29.60, 28.01, 27.61, 26.55 (x2), 25.85, 18.91 (conformational isomer peaks are underlined); HRMS (ESI) *m/z* for $C_{104}H_{111}N_4O_{19}Si$ [M-H]$^-$: calcd 1747.7612, found 1747.7631.

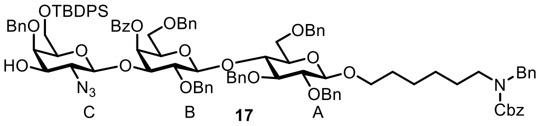

Compound 17. The synthesis of compound **17** is similar as described in the synthesis of compound **S14**, starting from compound **S24** (2.01 g, 0.97 mmol) to yield compound **17** (1.22 g, 72% over 2 steps); EtOAc/Hexanes = 2/3, R_f = 0.55. ^{1}H NMR (500 MHz, $CDCl_3$) δ 8.06 (d, *J* = 7.3 Hz, 2H), 7.70-7.66 (m, 4H), 7.53 (t, *J* = 7.4 Hz, 1H), 7.46-7.20 (m, 45H), 7.07 (t, *J* = 7.4 Hz, 1H), 6.95 (t, *J* = 7.6 Hz, 2H), 5.62 (br, 1H, B-4), 5.23 (br, 1H, Cbz), 5.21 (br, 1H, Cbz), 5.09 (d, *J* = 10.6 Hz, 1H), 4.95 (d, *J* = 10.4 Hz, 1H), 4.94-4.92 (m, 1H), 4.83 (d, *J* = 10.5 Hz, 1H), 4.82 (d, *J* = 11.6 Hz, 1H), 4.80 (d, *J* = 11.6 Hz, 1H), 4.78 (d, *J* = 10.3 Hz, 1H), 4.71 (d, *J* = 11.7 Hz, 1H), 4.66 (d, *J* = 8.1 Hz, 2H), 4.54-4.51 (m, 3H), 4.46-4.44 (m, 1H), 4.45 (d, *J* = 12.1 Hz, 1H), 4.38 (d, *J* = 11.9 Hz, 1H), 4.20 (d, *J* = 11.7 Hz, 1H), 4.08 (dd, *J* = 9.4, 9.4 Hz, 1H, A-4), 3.95 (br, 1H, OCH_2CH_2), 3.94 (d, *J* = 3.1 Hz, 1H, C-4), 3.91 (d, *J* = 7.1 Hz, 2H), 3.86-3.84 (m, 3H), 3.79 (d, *J* = 9.8 Hz, 1H), 3.65 (dd, *J* = 9.4, 9.4 Hz, 1H, A-3), 3.58 (t, *J* = 6.2 Hz, 1H), 3.57-3.50 (br, 1H, OCH_2CH_2), 3.50-3.41 (m, 5H), 3.67-3.29 (m, 3H), 3.22 (t, *J* = 6.4 Hz, 1H, CH_2NBnCbz), 2.32 (d, *J* = 8.5 Hz, 1H, C_3-OH), 1.68-1.51 (m, 4H), 1.47-1.23 (m, 4H), 1.11 (s, 9H, $C(CH_3)_3$); ^{13}C NMR (125 MHz, $CDCl_3$) δ 165.32, 156.63, 156.07, 138.72, 138.52, 138.02, 137.95, 137.82, 137.77, 136.75, 136.64, 135.46, 135.39, 132.81, 132.76, 132.74, 129.83, 129.78, 129.76, 129.73, 128.45, 128.41, 128.32, 128.22, 128.18, 128.17, 128.15, 128.11, 127.85, 127.79, 127.76, 127.74,

127.70, 127.63, 127.59, 127.45, 127.40, 127.28, 127.20, 127.11, 127.02, 103.49, 102.30, 101.93, 82.51, 81.46, 80.58, 76.27, 76.21, 75.29, 75.22, 75.19, 75.03, 74.87, 74.74, 73.42, 73.15, 72.91, 72.17, 70.40, 69.72, 68.10, 67.93, 67.04, 67.00, 64.93, 61.54, 50.31, 49.99, 47.02, 46.05, 29.53, 27.93, 27.55, 26.76 (x3), 26.49, 25.78, 19.04 (conformational isomer peaks are underlined); HRMS (ESI) *m/z* for $C_{104}H_{113}N_4O_{18}Si$ $[M-H]^-$: calcd 1733.7819, found 1733.7819.

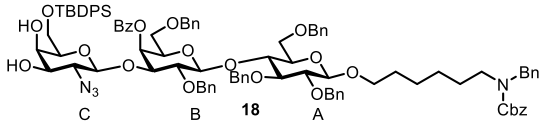

Compound 18. To a solution of compound **S25** (296 mg, 0.18 mmol) in DCM/H_2O/TFA (1.7 mL/0.1 mL/0.2 mL) was stirred at rt. After being stirred for 5 h, the reaction mixture was quenched with Et_3N and concentrated *in vacuo*. The residue was extracted with EtOAc, and the resulting organic solution was washed with saturated $NaHCO_{3(aq)}$ and brine. The organic layer was dried over $MgSO_4$, filtered, and concentrated. Next, the synthesis procedure is similar as described in the synthesis of compound **S14**, starting from above residue to yield compound **18** (170 mg, 60% over 3 steps); EtOAc/Hexanes = 1/1, R_f = 0.63. ^{1}H NMR (400 MHz, $CDCl_3$) δ 8.00 (d, *J* = 7.2 Hz, 2H), 7.68-7.63 (m, 4H), 7.51 (t, *J* = 7.4 Hz, 1H), 7.38-7.14 (m, 40H), 7.03 (t, *J* = 7.4 Hz, 1H), 6.93 (t, *J* = 7.5 Hz, 2H), 5.61 (d, *J* = 3.4 Hz, 1H, B-4), 5.15 (br, 1H, Cbz), 5.14 (br, 1H, Cbz), 5.02 (d, *J* = 10.6 Hz, 1H), 4.85 (d, *J* = 10.4 Hz, 1H), 4.76 (d, *J* = 10.6 Hz, 1H), 4.71 (d, *J* = 10.0 Hz, 1H), 4.68 (d, *J* = 10.9 Hz, 1H), 4.63 (d, *J* = 7.2 Hz, 1H), 4.58 (d, *J* = 12.2 Hz, 1H), 4.48-4.44 (m, 3H), 4.39 (d, *J* = 12.2 Hz, 1H), 4.36 (d, *J* = 8.0 Hz, 1H), 4.33 (d, *J* = 12.0 Hz, 1H), 4.15 (d, *J* = 12.0 Hz, 1H), 4.01 (dd, *J* = 9.4, 9.4 Hz, 1H. A-4), 3.95 (d, *J* = 2.4 Hz, 1H, C-4), 3.89-3.88 (m, 3H), 3.84 (dd, *J* = 9.8, 3.5 Hz, 1H), 3.79 (d, *J* = 11.1, 4.2 Hz, 1H), 3.74 (dd, *J* = 9.6, 7.8 Hz, 1H), 3.71 (d, *J* = 11.1, 1.4 Hz, 1H), 3.57 (dd, *J* = 9.4, 9.4 Hz, 1H), 3.54 (dd, *J* = 6.4, 6.4 Hz, 1H), 3.47-3.15 (m, 10H), 2.55 (d, *J* = 7.7 Hz, 1H), 1.50-1.41 (m, 4H), 1.39-1.17 (m, 4H), 1.02 (s,

9H, C(CH_3)$_3$); ^{13}C NMR (100 MHz, CDCl$_3$) δ 165.73, 156.66, 156.11, 138.81, 138.57, 138.10, 138.01, 137.91, 137.83, 136.79, 136.70, 135.49, 135.45, 132.98, 132.83, 132.71, 129.85, 129.81, 129.73, 128.43, 128.35, 128.24, 128.20, 128.13, 127.87, 127.83, 127.81, 127.73, 127.59, 127.57, 127.57, 127.47, 127.40, 127.34, 127.17, 127.09, 103.55, 102.37, 102.20, 82.60, 81.54, 80.44, 77.20, 76.28, 75.38, 75.30, 75.11, 74.88, 74.48, 73.38, 73.18, 72.61, 72.10, 70.78, 69.77, 68.16, 67.97, 67.76, 67.06, 64.82, 62.59, 50.35, 50.05, 47.06, 46.11, 29.57, 27.97, 27.58, 26.65 (x3), 26.52, 25.82, 19.05 (conformational isomer peaks are underlined); HRMS (ESI) *m/z* for $C_{97}H_{108}ClN_4O_{18}Si$ $[M+Cl]^-$: calcd 1679.7116, found 1679.7097.

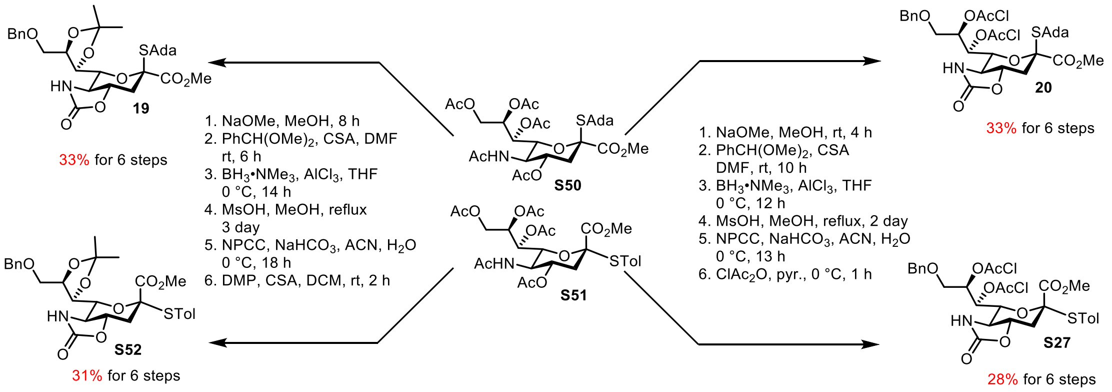

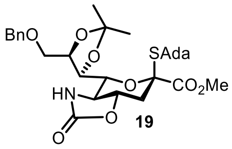

Compound 19. To a solution of compound **S50** (2.00 g, 3.12 mmol) in MeOH (31 mL) was added sodium methoxide (168 mg, 3.12 mmol) under nitrogen atmosphere at 0 °C. The reaction mixture was gradually warmed to rt and stirred for 8 h. The reaction mixture was neutralized with Amberlite® IR-120 (H^+) resin and filtered off the resin. The resulting solution was concentrated under reduced pressure. Next, a solution of above residue in DMF (31 mL) was added camphor sulfonic acid (216 mg, 0.94 mmol) and benzaldehyde dimethyl acetal (0.94 mL, 6.23 mmol) under a nitrogen atmosphere

at rt. After being stirred for 6 h, the reaction was quenched with Et_3N and concentrated *in vacuo*. The residue was extracted with EtOAc, and the resulting organic solution was washed with saturated $NaHCO_{3(aq)}$, and brine. The organic layer was dried over $MgSO_4$, filtered, and concentrated. The residue was purified by silica gel chromatography. Next, to a solution of above residue in THF (31 mL) was added borane trimethylamine complex (1.36 g, 18.70 mmol) under a nitrogen atmosphere at 0 °C. The reaction mixture was stirred at 0 °C for 30 min, and then aluminum chloride (2.49 g, 18.70 mmol) in THF (31 mL) was slowly added at 0 °C. The reaction mixture was stirred for 14 h and then the reaction was quenched with 10% $H_2SO_{4(aq)}$. The residue was extracted with EtOAc, and the resulting organic solution was washed with 1N $HCl_{(aq)}$, saturated $NaHCO_{3(aq)}$, and brine. The organic layer was dried over $MgSO_4$, filtered, and concentrated. The residue was purified by silica gel chromatography. Next, to a solution of above residue in MeOH (31 mL) was added methanesulfonic acid (0.61mL, 9.35 mmol) under nitrogen atmosphere at rt. The reaction mixture was gradually warmed to 80 °C and stirred for 3 days. The reaction mixture was quenched with Et_3N and concentrated *in vacuo*. Next, to a solution of above residue in ACN/H_2O (15 mL/15 mL) was $NaHCO_3$ (1.31 g, 15.59 mmol) at 0 °C. The solution of 4-nitrophenyl chloroformate (1.57 g, 7.79 mmol) in ACN (15 mL) was slowly added. After being stirred at 0 °C for 18 h, the residue was extracted with EtOAc, and the resulting organic solution was washed with saturated $NaHCO_{3(aq)}$ and brine. The organic layer was dried over $MgSO_4$, filtered, and concentrated. The residue was purified by silica gel chromatography. Next, to a solution of above residue in DCM (30 mL) was added camphor sulfonic acid (217 mg, 0.93 mmol) and 2,2-dimethoxypropane (1.15 mL, 9.35 mmol) under nitrogen atmosphere at rt. After being stirred for 2 h, the reaction was quenched with Et_3N and concentrated *in vacuo*. The residue was extracted with EtOAc, and the resulting organic solution was washed with saturated $NaHCO_{3(aq)}$ and brine. The organic layer was dried

over $MgSO_4$, filtered, and concentrated. The residue was purified by silica gel chromatography (EtOAc/Hexanes = 1/1, R_f = 0.08) to afford compound **19** (610 mg, 33% over 6 steps). ^{1}H NMR (400 MHz, $CDCl_3$) δ 7.34-7.25 (m, 5H), 5.56 (s, 1H, N*H*), 4.61-4.54 (m, 1H, H-4), 4.57 (d, *J* = 11.9 Hz, 1H, C*H*$_2$Ph), 4.51 (d, *J* = 11.9 Hz, 1H, C*H*$_2$Ph), 4.51 (dd, *J* = 10.6, 6.2 Hz, 1H, H-6), 4.36 (ddd, *J* = 6.2, 6.2, 6.2 Hz, 1H, H-8), 4.25 (dd, *J* = 6.2, 6.2 Hz, 1H, H-7), 3.77 (s, 3H, OC*H*$_3$), 3.74 (dd, *J* = 10.3, 6.2 Hz, 1H, H-9), 3.67 (dd, *J* = 10.3, 6.2 Hz, 1H, H-9), 3.36 (dd, *J* = 10.6, 10.6 Hz, 1H, H-5), 2.66 (dd, *J* = 12.8, 3.8 Hz, 1H, H-3_{eq}), 2.02 (dd, *J* = 12.8, 12.8 Hz, 1H, H-3_{ax}), 1.97-1.94 (m, 6H, SAda), 1.85-1.83 (m, 3H, SAda, overlap with H_2O), 1.63 (br, 6H, SAda), 1.42 (s, 3H, CC*H*$_3$), 1.34 (s, 3H, CC*H*$_3$); ^{13}C NMR (100 MHz, $CDCl_3$) δ 170.31, 159.41, 137.44, 128.46 (x2), 128.17 (x2), 127.87, 109.12, 86.17, 77.71, 76.48, 75.86, 73.72, 72.24, 69.15, 59.52, 52.85, 50.94, 43.48 (x3), 38.77, 35.92 (x3), 29.83 (x3), 26.92, 25.30; HRMS (ESI) *m/z* for $C_{31}H_{41}NNaO_8S$ $[M+Na]^+$: calcd 610.2451, found 610.2453.

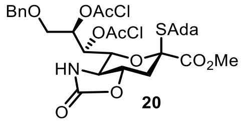

20

Compound 20. The synthesis from the first to the fifth steps were similar to those described in synthesis of compound **19**, starting from compound **16** (789 mg, 1.32 mmol) to yield C7 and C8 free sialic acid. Next, to a solution of above residue in DCM (5 mL) was added chloroacetic anhydride (652 mg, 3.82 mmol) and pyridine (0.6 mL, 7.63 mmol) at 0 °C. After being stirred at 0 °C for 60 min, the reaction mixture was quenched with MeOH and concentrated *in vacuo*. The residue was extracted with EtOAc, and the resulting organic solution was washed with 1N $HCl_{(aq)}$, saturated $NaHCO_{3(aq)}$, and brine. The organic layer was dried over $MgSO_4$, filtered, and concentrated. The residue was purified by silica gel chromatography (EtOAc/Hexanes = 1/1, R_f = 0.58) to afford compound **20** (308 mg, 33% over 6 steps). ^{1}H NMR (400 MHz, $CDCl_3$) δ 7.36-7.26 (m, 5H), 5.69 (s, 1H, N*H*), 5.35-5.32 (m, 2H, H-7, H-8),

4.62-4.55 (m, 1H, H-4), 4.57 (d, J = 12.0 Hz, 1H, CH_2Ph), 4.47 (dd, J = 10.8, 3.0 Hz, 1H, H-6), 4.44 (d, J = 12.0 Hz, 1H, CH_2Ph), 4.14 (d, J = 15.1 Hz, 1H, AcCl), 4.07 (d, J = 15.1 Hz, 1H, AcCl), 4.04 (d, J = 15.0 Hz, 1H AcCl), 3.95 (d, J = 15.0 Hz, 1H, AcCl), 3.93 (dd, J = 11.2, 2.6 Hz, 1H, H-9), 3.79 (s, 3H, OCH_3), 3.70 (dd, J = 11.2, 5.1 Hz, 1H, H-9), 3.26 (dd, J = 10.8, 10.8 Hz, 1H, H-5), 2.72 (dd, J = 12.7, 3.6 Hz, 1H, H-3$_{eq}$), 2.12 (dd, J = 12.7, 12.7 Hz, 1H, H-3$_{ax}$), 1.97-1.92 (m, 6H, SAda), 1.81-1.78 (m, 3H, SAda), 1.62 (br, 6H, SAda, overlap with H_2O); ^{13}C NMR (100 MHz, $CDCl_3$) δ 169.51, 166.79, 166.73, 159.37, 137.30, 128.49, 127.98, 127.87, 86.33, 73.36, 72.94, 71.92, 71.48, 67.11, 59.30, 52.90 (x2), 51.18, 43.41, 40.82, 40.38, 39.23, 35.90, 29.77; HRMS (ESI) *m/z* for $C_{32}H_{39}Cl_2NNaO_{10}S$ $[M+Na]^+$: calcd 722.1569, found 722.1568.

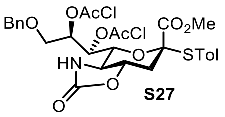

S27

Compound S27. The synthesis of compound **S27** is similar as described in the synthesis of compound **20**, starting from compound **S51** (3.00 g, 5.02 mmol) to yield compound **S27** (936 mg, 28% over 6 steps); EtOAc/Hexanes = 1/1, R_f = 0.50. ^{1}H NMR (400 MHz, $CDCl_3$) δ 7.37-7.22 (m, 7H), 7.11 (d, J = 8.0 Hz, 2H), 5.38-5.29 (m, 3H, NH, H-7, H-8), 4.57 (d, J = 12.0 Hz, 1H, CH_2Ph), 4.34 (d, J = 12.0 Hz, 1H, CH_2Ph), 4.15 (d, J = 15.2 Hz, 1H, AcCl), 4.04 (d, J = 15.2 Hz, 1H, AcCl), 4.03 (d, J = 9.9 Hz, 1H, H-6), 3.95 (d, J = 14.9 Hz, 1H, AcCl), 3.86 (ddd, J = 12.2, 9.9, 3.6 Hz, 1H, H-4), 3.78 (d, J = 14.9 Hz, 1H, AcCl), 3.74 (dd, J = 11.4, 1.5 Hz, 1H, H-9), 3.62 (dd, J = 11.4, 2.5 Hz, 1H, H-9), 3.55 (s, 3H, OCH_3), 3.06 (dd, J = 12.2, 3.6 Hz, 1H, H-3$_{eq}$), 2.96 (dd, J = 9.9, 9.9 Hz, 1H, H-5), 2.34 (s, 3H, STol), 2.09 (dd, J = 12.2, 12.2 Hz, 1H, H-3$_{ax}$); ^{13}C NMR (125 MHz, $CDCl_3$) δ 167.74, 167.73, 166.19, 158.88, 140.52, 137.01, 136.01, 129.77, 128.55, 128.22, 128.13, 124.58, 88.27, 77.38, 74.89, 73.34, 70.52, 70.33, 66.39, 57.58, 52.98, 40.93, 40.24, 37.44, 21.27; HRMS (ESI) *m/z* for $C_{29}H_{30}Cl_2NO_{10}S$ $[M-H]^-$: calcd 654.0968, found 654.0967.

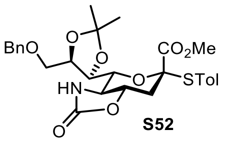

S52

Compound S52. The synthesis of compound **S52** is similar as described in the synthesis of compound **19**, starting from compound **S51** (5.00 g, 8.37 mmol) to yield compound **S52** (1.42 g, 31% over 6 steps); EtOAc/Hexanes = 1/2, R_f = 0.25. ^{1}H NMR (400 MHz, $CDCl_3$) δ 7.52 (d, *J* = 8.2 Hz, 2H, STol), 7.32-7.23 (m, 5H), 7.14 (d, *J* = 8.2 Hz, 2H, STol), 5.23 (br, 1H, N*H*), 4.49-4.45 (m, 1H, H-4), 4.46 (d, *J* = 12.4 Hz, 1H, CH_2Ph), 4.31 (d, *J* = 12.4 Hz, 1H, CH_2Ph), 4.01 (d, *J* = 6.7 Hz, 1H, H-6), 3.92-3.86 (m, 1H, H-8), 3.76-3.75 (m, 2H, H-7, H-9), 3.69-3.62 (m, 2H, H-5, H-9), 3.54 (s, 3H, OCH_3), 3.17 (dd, *J* = 11.8, 3.6 Hz, 1H, H-3_{eq}), 2.32 (s, 3H, STol), 2.17 (dd, *J* = 11.8, 11.8 Hz, 1H, H-3_{ax}), 1.71 (s, 3H, CCH_3), 1.40 (s, 3H, CCH_3); ^{13}C NMR (100 MHz, $CDCl_3$) δ 168.22, 159.64, 140.32, 138.04, 136.80, 129.52, 128.29, 127.80, 127.62, 125.15, 110.19, 88.87, 77.59, 76.89, 76.08, 75.63, 72.98, 67.67, 57.81, 52.57, 37.35, 26.44, 25.60, 21.30; HRMS (ESI) *m/z* for $C_{28}H_{33}NNaO_8S$ $[M+Na]^+$: calcd 566.1825, found 566.1821.

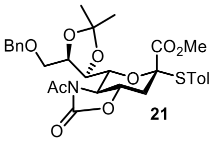

21

Compound 21. The synthesis of compound **21** is similar as described in the synthesis of compound **5**, starting from compound **S52** (842 mg, 1.55 mmol) to yield compound **21** (976 mg, quant); EtOAc/Hexanes = 1/1, R_f = 0.53. ^{1}H NMR (400 MHz, $CDCl_3$) δ 7.59 (d, *J* = 8.0 Hz, 2H, STol), 7.32-7.22 (m, 5H), 7.15 (d, *J* = 8.0 Hz, 2H, STol), 4.94 (dd, *J* = 7.1, 0.6 Hz, 1H, H-7), 4.51-4.46 (m, 1H, H-8), 4.47 (d, *J* = 12.1 Hz, 1H, CH_2Ph), 4.26 (d, *J* = 12.1 Hz, 1H, CH_2Ph), 3.99 (dd, *J* = 10.5, 10.5 Hz, 1H, H-5), 3.93 (dd, *J* = 10.5, 0.6 Hz, 1H, H-6), 3.83 (ddd, *J* = 11.9, 10.5, 3.4 Hz, 1H, H-4), 3.78 (dd, *J* = 10.2, 7.2 Hz, 1H, H-9), 3.70 (dd, *J* = 10.2, 5.0 Hz, 1H, H-9), 3.52 (s, 3H, OCH_3), 3.14 (dd, *J* = 11.9, 3.4 Hz, 1H, H-3_{eq}), 2.44 (s, 3H, Ac), 2.31 (s, 3H, STol), 2.14 (dd, *J* = 11.9, 11.9

Hz, 1H, H-3_{ax}), 1.77 (s, 3H, CCH_3), 1.45 (s, 3H, CCH_3); ^{13}C NMR (100 MHz, $CDCl_3$) δ 172.34, 168.15, 153.72, 140.46, 138.25, 136.94, 129.60, 128.24, 127.70, 127.51, 124.99, 109.80, 88.56, 78.09, 76.59, 76.21, 75.50, 72.81, 67.85, 59.88, 52.56, 36.59, 26.51, 25.76, 25.17, 21.31; HRMS (ESI) *m/z* for $C_{30}H_{35}NNaO_9S$ $[M+Na]^+$: calcd 608.1930, found 608.1925.

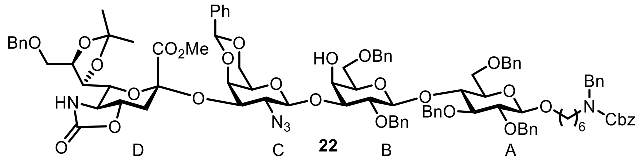

Compound 22α. Compound **22α** was synthesized from glycosyl acceptor **15** (209 mg, 0.15 mmol) and glycosyl donor **19** (177 mg, 0.30 mmol) by following the **General glycosylation procedure A**. The mixture was purified by silica gel chromatography (EtOAc/Hexanes = 1/1, R_f = 0.23) to afford compound **22α** (151 mg, 55%). ^{1}H NMR (850 MHz, $CDCl_3$) δ 7.54 (d, *J* = 7.0 Hz, 2H), 7.43-7.16 (m, 43H), 5.44 (s, 1H, C*H*Ph), 5.43 (br, 1H, D_5-NH), 5.19 (br, 1H, Cbz), 5.17 (br, 1H, Cbz), 5.00 (d, *J* = 10.6 Hz, 1H, A_3-Bn), 4.88 (t, *J* = 11.0 Hz, 1H, A_2-Bn), 4.84 (d, *J* = 11.0 Hz, 1H, B_2-Bn), 4.79 (d, *J* = 11.0 Hz, 1H, B_2-Bn), 4.75 (d, *J* = 10.6 Hz, 1H, A_3-Bn), 4.72 (d, *J* = 11.0 Hz, 1H, A_2-Bn), 4.58-4.57 (m, 2H, N-Bn, B_6-Bn), 4.55-4.52 (m, 2H, A_6-Bn, D-8), 4.49-4.46 (m, 5H, N-Bn, A_6-Bn, B-1, C-1, D_9-Bn), 4.41 (d, *J* = 12.1 Hz, 1H, B_6-Bn), 4.37 (d, *J* = 12.0 Hz, 1H, D_9-Bn), 4.34 (m, 1H, A-1), 4.28 (d, *J* = 3.5 Hz, 1H, C-4), 4.17 (d, *J* = 11.8 Hz, 1H, C-6), 4.14 (dd, *J* = 7.0, 1.4 Hz, 1H, D-7), 4.09 (dd, *J* = 10.3, 5.0 Hz, 1H, D-9), 4.08 (d, *J* = 3.2 Hz, 1H, B-4), 4.05 (dd, *J* = 10.4, 3.5 Hz, 1H, C-3), 3.99 (dd, *J* = 9.3, 9.3 Hz, 1H, A-4), 3.97 (dd, *J* = 10.3, 6.7 Hz, 1H, D-9), 3.92-3.85 (m, 4H, OCH_2CH_2, C-6, D-4, D-6), 3.84 (dd, *J* = 10.4, 8.2 Hz, 1H, C-2), 3.78 (dd, *J* = 11.0, 4.2 Hz, 1H, A-6), 3.72-3.68 (m, 3H, A-6, B-2, B-6), 3.62 (dd, *J* = 10.9, 10.9 Hz, 1H, D-5), 3.60 (dd, *J* = 9.4, 3.2 Hz, 1H, B-3), 3.58 (s, 3H, OCH_3), 3.56 (dd, *J* = 9.3, 9.3 Hz, 1H, A-3), 3.52 (dd, *J* = 9.7, 5.5 Hz, 1H, B-6), 3.49-3.44 (m, 1H, OCH_2CH_2), 3.43 (dd, *J* = 5.5, 3.2 Hz, 1H,

B-5), 3.39 (dd, *J* = 9.3, 7.7 Hz, 1H, A-2), 3.33 (ddd, *J* = 9.3, 4.2, 1.3 Hz, 1H, A-5), 3.25 (m, 1H, C*H*$_2$NBnCbz), 3.18 (br, 1H, C-5), 3.18 (m, 1H, C*H*$_2$NBnCbz), 3.08 (dd, *J* = 11.9, 3.1 Hz, 1H, D-3$_{eq}$), 2.67 (s, 1H, B$_4$-OH), 2.14 (dd, *J* = 11.9, 11.9 Hz, 1H, D-3$_{ax}$), 1.61-1.45 (m, 4H), 1.45 (s, 3H, CC*H*$_3$), 1.38 (s, 3H, CC*H*$_3$), 1.36-1.22 (m, 4H); ^{13}C NMR (213 MHz, $CDCl_3$) δ 168.44, 159.57, 156.72, 156.14, 139.00, 138.69, 138.41, 138.29, 137.91, 137.87, 137.73, 137.41, 136.84, 136.75, 129.14, 128.47, 128.44, 128.37, 128.32, 128.28, 128.25, 128.23, 128.19, 128.00, 127.88, 127.84, 127.77, 127.71, 127.62, 127.57, 127.48, 127.46, 127.42, 127.33, 127.25, 127.21, 127.17, 127.12, 126.12, 109.12, 103.57, 102.32, 102.23, 100.60, 99.82, 82.77, 82.31, 81.78, 79.08, 77.21, 76.26, 76.14, 75.51, 75.43, 75.08, 75.07, 74.99, 74.86, 73.43, 73.32, 73.30, 73.04, 72.91, 72.77, 69.82, 69.77, 68.89, 68.38, 68.37, 68.25, 68.09, 67.12, 67.07, 66.12, 61.15, 57.82, 53.11, 50.40, 50.07, 47.11, 46.15, 36.60, 29.61, 28.01, 27.62, 26.62, 26.58, 25.83, 24.69 (conformational isomer peaks are underlined); HRMS (ESI) *m/z* for $C_{102}H_{115}N_5NaO_{25}$ $[M+Na]^+$: calcd 1832.7779, found 1832.7787.

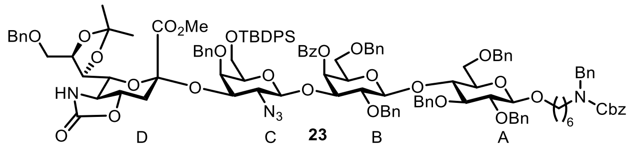

Compound 23α. Compound **23α** and 23**β** was synthesized from glycosyl acceptor **17** (48 mg, 0.03 mmol) and glycosyl donor **19** (49 mg, 0.08 mmol) by following the **General glycosylation procedure A**. The mixture was purified by silica gel chromatography (EtOAc/Hexanes = 1/3 then EtOAc/Hexanes = 1/2, R_f = 0.34) to afford compound **23α** (27 mg, 40%) and (EtOAc/Hexanes = 1/3 then EtOAc/Hexanes = 1/2, R_f = 0.36) to afford compound **23β** (24 mg, 45%). ^{1}H NMR (850 MHz, $CDCl_3$) δ 8.04 (d, *J* = 7.1 Hz, 2H), 7.71-7.70 (m, 2H), 7.65 (d, *J* = 6.7 Hz, 2H), 7.54 (t, *J* = 7.4 Hz, 1H), 7.44-7.19 (m, 50H), 7.08 (t, *J* = 7.4 Hz, 1H), 6.97 (t, *J* = 7.6 Hz, 2H), 5.64 (d, *J* = 3.3 Hz, 1H, B-4), 5.56 (d, *J* = 6.8 Hz, 1H, D$_5$-NH), 5.24 (br, 1H, Cbz), 5.22 (br, 1H,

Cbz), 5.08 (d, *J* = 10.5 Hz, 1H), 5.01 (d, *J* = 11.0 Hz, 1H), 4.92 (t, *J* = 11.0 Hz, 1H), 4.87 (d, *J* = 11.3 Hz, 1H), 4.85 (d, *J* = 10.8 Hz, 1H), 4.83 (d, 10.5 Hz, 1H), 4.77 (d, *J* = 11.0 Hz, 1H), 4.66 (d, *J* = 12.1 Hz, 1H), 4.63 (d, *J* = 7.9 Hz, 1H), 4.60 (d, *J* = 11.9 Hz, 1H), 4.58 (d, *J* = 7.5 Hz, 1H), 4.58 (d, *J* = 11.9 Hz, 1H), 4.55-4.51 (m, 4H), 4.46 (d, *J* = 12.1 Hz, 1H), 4.42 (m, 1H, A-1), 4.38 (d, *J* = 11.8 Hz, 1H), 4.20 (d, *J* =11.9 Hz, 1H), 4.18-4.16 (m, 2H), 4.07 (dd, *J* =9.4, 9.4 Hz, 1H, A-4), 4.02-3.98 (m, 2H), 3.95-3.94 (m, 1H, OCH_2CH_2), 3.92 (dd, *J* = 9.7, 3.4 Hz, 1H), 3.90-3.85 (m, 5H), 3.79-3.77 (m, 2H), 3.72 (s, 3H, OCH_3), 3.67 (dd, *J* = 10.6, 2.9 Hz, 1H), 3.64 (t, *J* = 9.1 Hz, 1H), 3.63 (d, *J* = 7.9 Hz, 1H), 3.61 (t, *J* = 6.4 Hz, 1H), 3.54 (t, *J* = 10.8 Hz, 1H), 3.52-3.48 (m, 1H, OCH_2CH_2), 3.48 (t, *J* = 6.7 Hz, 1H), 3.45-3.42 (m, 3H), 3.33 (dd, *J* = 10.1, 6.2 Hz, 1H), 3.31 (br, 1H, CH_2NBnCbz), 3.23 (br, 1H, CH_2NBnCbz), 2.93 (dd, *J* = 12.2, 3.3 Hz, 1H, D-3_{eq}), 2.11 (dd, *J* = 12.2, 12.2 Hz, 1H, D-3_{ax}), 1.67-1.52 (m, 4H), 1.46 (s, 3H, CCH_3), 1.46-1.27 (m, 4H), 1.37 (s, 3H, CCH_3), 1.08 (s, 9H, C(CH_3)$_3$); ^{13}C NMR (213 MHz, CDCl$_3$) δ 167.70, 165.25, 159.69, 156.70, 156.12, 138.84, 138.61, 138.54, 138.42, 138.16, 138.14, 137.90, 137.84, 137.79, 136.83, 136.73, 135.56, 135.46, 133.08, 132.86, 132.78, 129.85, 129.80, 129.74, 129.70, 128.45, 128.40, 128.37, 128.23, 128.20, 128.16, 128.12, 128.08, 127.91, 127.87, 127.81, 127.78, 127.74, 127.70, 127.68, 127.66, 127.63, 127.46, 127.41, 127.28, 127.23, 127.21, 127.16, 127.11, 127.03, 108.70, 103.55, 102.34, 101.90, 101.19, 82.60, 81.53, 80.80, 77.40, 77.20, 76.89, 76.41, 76.02, 75.37, 75.24, 75.11, 75.07, 75.02, 74.89 (x2), 74.72, 74.49, 73.48, 73.30, 73.27, 73.13, 70.30, 69.80, 69.76, 68.77, 68.29, 67.98, 67.11, 67.05, 62.23, 62.12, 58.08, 53.01, 50.39, 50.06, 47.09, 46.14, 35.56, 29.59, 28.00, 27.61, 26.83, 26.80 (x3), 26.56, 25.82, 24.53, 19.10 (conformational isomer peaks are underlined); HRMS (ESI) *m/z* for $C_{125}H_{139}N_5NaO_{26}Si$ [M+Na]$^+$: calcd 2176.9375, found 2176.9380.

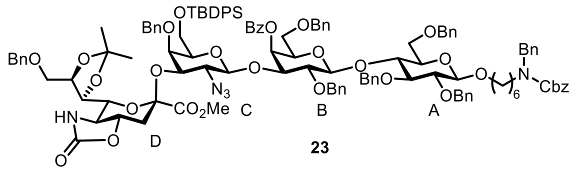

23

Compound 23β. ^{1}H NMR (850 MHz, CDCl$_3$) δ 8.07 (d, *J* = 7.3 Hz, 2H), 7.68 (d, *J* = 6.0 Hz, 2H), 7.61 (d, *J* = 6.8 Hz, 2H), 7.54 (t, *J* = 7.4 Hz, 1H), 7.46-7.21 (m, 47H), 7.16-7.15 (m, 3H), 7.08 (t, *J* = 7.4 Hz, 1H), 6.97 (d, *J* = 7.6 Hz, 2H), 5.66 (d, *J* = 1.6 Hz, 1H, B-4), 5.54 (br, 1H, D$_5$-NH), 5.24 (br, 1H, Cbz), 5.22 (br, 1H, Cbz), 5.10 (d, *J* = 10.5 Hz, 1H), 5.00 (d, *J* = 10.5 Hz, 1H), 4.93 (t, *J* = 11.0 Hz, 1H), 4.86 (d, *J* = 11.7 Hz, 1H), 4.84 (d, *J* = 10.5 Hz, 1H), 4.80 (d, *J* = 11.0 Hz, 1H), 4.78 (d, *J* = 11.7 Hz, 1H), 4.67-4.65 (m, 3H), 4.56-4.52 (m, 3H), 4.47 (d, *J* =12.1 Hz, 1H), 4.45 (d, *J* = 11.9 Hz, 1H), 4.44 (d, *J* = 11.7 Hz, 1H), 4.39 (d, *J* = 11.9 Hz, 1H), 4.38-4.35 (m, 1H), 4.35 (d, *J* =12.3 Hz, 1H), 4.31 (d, *J* = 12.1 Hz, 1H), 4.21 (d, *J* = 11.9 Hz, 1H), 4.19 (dd, *J* = 6.6, 2.9 Hz, 1H, D-7), 4.14 (dd, *J* = 9.9, 3.0 Hz, 1H, C-3), 4.09 (dd, *J* = 9.4, 9.4 Hz, 1H, A-4), 3.95 (m, 1H, OCH_2CH$_2$), 3.89-3.78 (m, 8H), 3.77 (dd, *J* = 10.3, 5.0 Hz, 1H, B-3), 3.72 (s, 3H, OCH_3), 3.66 (dd, *J* = 9.4, 8.8 Hz, 1H, A-3), 3.63 (dd, *J* = 10.6, 7.6 Hz, 1H), 3.61-3.59 (m, 2H), 3.57-3.53 (m, 1H, OCH_2CH$_2$), 3.54 (dd, *J* = 8.8, 8.8 Hz, 1H, A-2), 3.49 (ddd, *J* = 9.4, 3.8, 1.8 Hz, 1H, A-5), 3.45-3.43 (m, 2H), 3.40 (t, *J* = 7.0 Hz, 1H), 3.34 (dd, *J* = 10.2, 6.3 Hz, 1H), 3.32-3.31 (m, 1H, CH_2NBnCbz), 3.23 (br, 1H, CH_2NBnCbz), 2.79 (dd, *J* = 12.7, 3.6 Hz, 1H, D-3$_{eq}$), 2.03 (dd, *J* = 12.7, 12.7 Hz, 1H, D-3$_{ax}$), 1.68-1.52 (m, 4H), 1.48 (s, 3H, CCH_3), 1.48-1.26 (m, 4H), 1.38 (s, 3H, CCH_3), 1.08 (s, 9H, C(CH_3)$_3$); ^{13}C NMR (213 MHz, CDCl$_3$) δ 166.80, 165.22, 159.79, 156.69, 156.12, 138.80, 138.61, 138.12, 138.10, 138.07, 137.89, 137.83, 137.61, 136.82, 136.72, 135.50, 135.39, 132.83, 132.70, 132.67, 129.86, 129.85, 129.80, 129.77, 128.49, 128.45, 128.39, 128.36, 128.30, 128.28, 128.22, 128.20, 128.17, 128.13, 127.90, 127.85, 127.78, 127.74, 127.61, 127.59, 127.47, 127.43, 127.38, 127.29, 127.23, 127.05, 126.73, 109.41, 103.56, 102.43, 102.25, 99.88, 82.53, 81.54, 80.62,

76.75, 76.51, 76.35, 76.00, 75.57, 75.42, 75.25, 75.12, 75.07, 74.88, 74.69, 74.49, 74.37, 74.22, 73.45, 73.21, 73.16, 73.06, 70.52, 69.82, 69.76, 68.64, 68.22, 68.04, 67.10, 67.05, 62.81, 61.77, 57.98, 52.52, 50.38, 50.06, 47.08, 46.12, 35.56, 29.58, 27.99, 27.60, 26.78 (x3), 26.54 (x2), 25.85, 25.82, 25.32, 19.04 (conformational isomer peaks are underlined); HRMS (ESI) *m/z* for $C_{125}H_{139}N_5NaO_{26}Si$ $[M+Na]^+$: calcd 2176.9375, found 2176.9358.

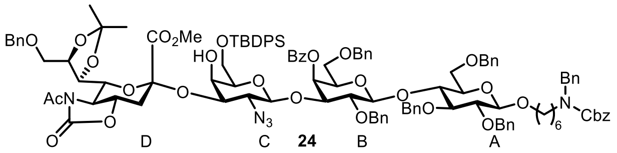

Compound 24α. Compound **24α** was synthesized from glycosyl acceptor **18** (49 mg, 0.03 mmol) and glycosyl donor **5** (38 mg, 0.06 mmol) by following the **General glycosylation procedure A**. The mixture was purified by silica gel chromatography (EtOAc/Hexanes = 2/3, R_f = 0.30) to afford compound **24α** (49 mg, 78%). ^1H NMR (850 MHz, $CDCl_3$) δ 8.10 (d, *J* = 7.3 Hz, 2H), 7.77-7.76 (m, 4H), 7.60 (t, *J* = 7.4 Hz, 1H), 7.49-7.21 (m, 45H), 7.13 (d, *J* = 7.3 Hz, 1H), 7.04 (t, *J* = 7.6 Hz, 2H), 5.73 (d, *J* = 3.4 Hz, 1H, B-4), 5.26 (br, 1H, Cbz), 5.24 (br, 1H, Cbz), 5.12 (d, *J* = 10.5 Hz, 1H), 5.00 (d, *J* = 7.2 Hz, 1H), 4.95 (d, *J* = 11.4 Hz, 1H), 4.93 (d, *J* = 10.9 Hz, 1H), 4.90 (d, *J* = 10.9 Hz, 1H), 4.85 (d, *J* = 10.6 Hz, 1H), 4.79 (d, *J* = 11.0 Hz, 1H), 4.66 (d, *J* = 12.2 Hz, 1H), 4.64 (d, *J* = 8.0 Hz, 1H), 4.60 (d, *J* = 7.7 Hz, 1H), 4.57-4.54 (m, 5H), 4.49 (d, *J* = 12.2 Hz, 1H), 4.44-4.42 (m, 1H), 4.43 (d, *J* = 12.1 Hz, H), 4.36 (dd, *J* = 8.6, 1.1 Hz, 1H), 4.24 (d, *J* = 12.0 Hz, 1H), 4.11-4.08 (m, 3H), 4.07 (dd, *J* = 10.2, 5.0 Hz, 1H), 4.03-3.93 (m, 6H), 3.87 (dd, *J* = 10.1, 6.5 Hz, 1H), 3.85 (s, 3H, OCH_3), 3.83 (t, *J* = 8.1 Hz, 1H), 3.77 (d, *J* = 9.9 Hz, 1H), 3.66-3.64 (m, 2H), 3.61 (dd, *J* = 10.1, 8.0 Hz, 1H), 3.57 (ddd, *J* = 7.0, 7.0, 7.0 Hz, 1H), 3.54 (br, 1H, OCH_2CH_2), 3.49-3.47 (m, 2H), 3.46 (t, *J* = 8.2 Hz, 1H), 3.42 (ddd, *J* = 9.8, 3.6, 1.3 Hz, 1H, A-5), 3.40 (dd, *J* = 9.9, 6.0 Hz, 1H), 3.33 (br, 1H, $CH_2NBnCbz$), 3.26 (br, 1H, $CH_2NBnCbz$), 3.03 (dd, *J* = 12.2, 3.1 Hz, 1H,

D-3_{eq}), 2.96 (br, 1H, C_4-OH), 2.57 (s, 3H, Ac), 2.18 (dd, *J* = 12.2, 12.2 Hz, 1H, D-3_{ax}), 1.70-1.55 (m, 4H), 1.48 (s, 3H, CCH_3), 1.45 (s, 3H, CCH_3), 1.43-1.35 (m, 4H), 1.12 (s, 9H, $C(CH_3)_3$); ^{13}C NMR (213 MHz, $CDCl_3$) δ 172.47, 169.17, 165.39, 156.71, 156.15, 153.88, 138.90, 138.65, 138.37, 138.20, 138.08, 138.03, 137.94, 137.88, 136.87, 136.76, 135.49, 132.88, 132.85, 132.79, 129.96, 129.85, 129.84, 129.81, 128.48, 128.43, 128.36, 128.30, 128.25, 128.23, 128.16, 127.93, 127.90, 127.87, 127.84, 127.78, 127.62, 127.61, 127.57, 127.54, 127.50, 127.43, 127.32, 127.27, 127.18, 127.12, 108.58, 103.59, 102.33, 101.87, 98.13, 82.69, 81.59, 80.15, 76.71, 76.36, 76.16, 75.86, 75.32, 75.18, 75.06, 74.93, 74.72, 74.53, 73.85, 73.43, 73.18(x2), 72.85, 70.78, 69.83, 69.78, 69.17, 68.04, 67.99, 67.11, 67.08, 66.63, 65.82, 63.33, 61.77, 59.90, 53.28, 50.41, 50.09, 47.12, 46.15, 36.26, 29.63, 28.03, 27.64, 26.72 (x3), 26.67, 26.59, 25.89, 25.86, 25.16, 24.76, 19.17 (conformational isomer peaks are underlined); HRMS (ESI) *m/z* for $C_{120}H_{135}N_5NaO_{27}Si$ $[M+Na]^+$: calcd 2128.9011, found 2128.8998.

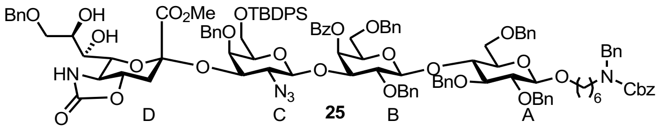

Compound 25. Compound **25** was synthesized from glycosyl acceptor **17** (400 mg, 0.23 mmol) and glycosyl donor **19** (297 mg, 0.51 mmol) by following the **General glycosylation procedure A**. Next, a solution of above residue in TFA/H_2O/DCM (3 mL/3 mL/20 mL) was stirred at 4 °C for 48 h. The reaction mixture was quenched with Et_3N and concentrated *in vacuo*. The residue was extracted with EtOAc, and the resulting organic solution was washed with saturated $NaHCO_{3(aq)}$ and brine. The organic layer was dried over $MgSO_4$, filtered, and concentrated. The residue was purified by silica gel chromatography (EtOAc/Hexanes = 3/2, R_f = 0.39) to afford compound **25** (153 mg, 31% over 2 steps). ^{1}H NMR (400 MHz, $CDCl_3$) δ 7.91 (d, *J* = 7.2 Hz, 2H), 7.65-7.63 (m, 2H), 7.58 (d, *J* = 6.7 Hz, 2H), 7.47 (t, *J* = 7.4 Hz, 1H), 7.41-7.17 (m, 50H), 7.03 (t, *J* = 7.4 Hz, 1H), 6.91 (t, *J* = 7.6 Hz, 2H), 5.94 (br, 1H, D_5-NH),

5.54 (d, *J* = 3.2 Hz, 1H, B-4), 5.55 (br, 1H, Cbz), 5.54 (br, 1H, Cbz), 5.01 (d, *J* = 10.6 Hz, 1H), 4.88-4.82 (m, 4H), 4.76 (d, *J* = 10.6 Hz, 1H), 4.72 (d, *J* = 11.0 Hz, 1H), 4.58 (d, *J* = 12.1 Hz, 1H), 4.57 (d, *J* = 11.4 Hz, 1H), 4.51-4.47 (m, 5H), 4.44 (d, *J* =7.3 Hz, 1H), 4.36 (d, *J* = 10.2 Hz, 1H), 4.36 (d, *J* = 9.0 Hz, 1H), 4.33 (d, *J* = 10.2 Hz, 1H), 4.15 (d, *J* = 12.0 Hz, 1H), 3.99 (dd, *J* = 9.4, 9.4 Hz, 1H, A-4), 3.91 (dd, *J* = 9.8, 4.0 Hz, 1H), 3.85-3.81 (m, 2H), 3.81-3.78 (m, 4H), 3.78-3.75 (m, 3H), 3.75-3.71 (m, 2H), 3.69-3.65 (m, 2H), 3.65-3.60 (m, 3H), 3.57 (t, *J* = 9.0 Hz, 1H), 3.53-3.48 (m, 3H), 3.41-3.33 (m, 4H), 3.28-3.24 (m, 2H), 3.22-3.13 (m, 2H), 3.09 (br, 1H, CH_2NBnCbz), 2.99 (dd, *J* = 12.1, 3.4 Hz, 1H, D-3_{eq}), 2.14 (dd, *J* = 12.1, 12.1 Hz, 1H, D-3_{ax}), 1.61-1.48 (m, 4H), 1.38-1.20 (m, 4H), 1.04 (s, 9H, $C(CH_3)_3$); ^{13}C NMR (100 MHz, $CDCl_3$) δ 168.47, 165.14, 159.65, 156.67, 156.10, 138.75, 138.53, 138.30, 138.04, 137.74, 137.49, 136.68, 135.42, 135.35, 132.81, 132.70, 132.65, 129.79, 129.76, 129.72, 129.68, 128.39, 128.37, 128.27, 128.16, 128.13, 128.07, 128.04, 127.82, 127.77, 127.71, 127.66, 127.63, 127.55, 127.41, 127.23, 127.16, 126.97, 103.48, 102.10, 100.99, 99.52, 82.52, 81.44, 80.14, 77.39, 77.21, 76.23, 75.60, 75.15, 75.10, 74.89 (x2), 74.82, 74.22, 74.15, 73.39, 73.37, 73.08 (x2), 72.85, 71.82, 71.01, 69.71, 69.67, 68.14, 67.90, 67.05, 67.02, 62.80, 61.06, 57.48, 53.32, 50.33, 50.01, 47.02, 46.10, 36.07, 29.51, 27.92, 27.54, 26.73 (x3), 26.47, 25.76, 18.97 (conformational isomer peaks are underlined); HRMS (ESI) *m/z* for $C_{122}H_{134}N_5O_{26}Si$ $[M-H]^-$: calcd 2112.9086, found 2112.9098.

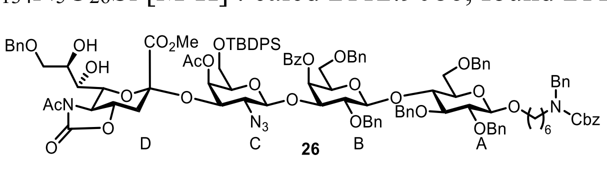

Compound 26. Compound **26** was synthesized from glycosyl acceptor **18** (571 mg, 0.35 mmol) and glycosyl donor **5** (437 mg, 0.69 mmol) by following the **General glycosylation procedure A**. Next, a solution of above residue in Ac_2O/pyridine (2 mL/4 mL) was stirred at rt for 10 h. The reaction mixture was concentrated *in vacuo*. The residue was extracted with EtOAc, and the resulting organic solution was washed

with 1N $HCl_{(aq)}$, saturated $NaHCO_{3(aq)}$, and brine. The organic layer was dried over $MgSO_4$, filtered, and concentrated. Next, a solution of above residue in DCM/H_2O/TFA (4 mL/1 mL/1 mL) was stirred at 4 °C for 24 h. The reaction mixture was quenched with Et_3N and concentrated *in vacuo*. The residue was extracted with EtOAc, and the resulting organic solution was washed with $NaHCO_{3(aq)}$ and brine. The organic layer was dried over $MgSO_4$, filtered, and concentrated. The residue was purified by silica gel chromatography (EtOAc/Hexanes = 1/1, R_f = 0.46) to afford compound **26** (395 mg, 54% over 3 steps). ^1H NMR (850 MHz, $CDCl_3$) δ 7.88 (d, J = 7.3 Hz, 2H), 7.65-7.64 (m, 2H), 7.59 (d, J = 6.7 Hz, 2H), 7.46 (t, J = 7.4 Hz, 1H), 7.42-7.40 (m, 4H), 7.37-7.16 (m, 41H), 7.02 (t, J = 7.4 Hz, 1H), 6.91 (t, J = 7.6 Hz, 2H), 5.55 (d, J = 3.6 Hz, 1H, B-4), 5.28 (d, J = 3.4 Hz, 1H, C-4), 5.18 (br, 1H, Cbz), 5.16 (br, 1H, Cbz), 5.00 (d, J =10.6 Hz, 1H), 4.86 (d, J = 11.0 Hz, 1H), 4.84 (d, J = 11.0 Hz, 1H), 4.79 (d, J = 11.0 Hz, 1H), 4.76 (d, J = 10.6 Hz, 1H), 4.70 (d, J = 11.0 Hz, 1H), 4.61 (d, J = 12.2 Hz, 1H), 4.58 (d, J = 12.1 Hz, 1H), 4.51 (d, J = 12.2 Hz, 1H), 4.50-4.45 (m, 4H), 4.37 (d, J = 12.1 Hz, 1H), 4.34 (br, 1H), 4.33 (d, J = 11.9 Hz, 1H), 4.16 (d, J = 11.9 Hz, 1H), 4.14-4.12 (m, 1H), 4.08 (ddd, J = 13.3, 10.7, 3.6 Hz, 1H, D-4), 3.99-3.97 (m, 2H), 3.93 (dd, J = 9.1, 1.4 Hz, 1H), 3.91-3.86 (m, 1H), 3.90 (dd, J = 10.6, 9.1 Hz, 1H), 3.86 (s, 3H, OCH_3), 3.83 (dd, J = 9.6, 3.5 Hz, 1H), 3.75 (dd, J = 10.9, 4.1 Hz, 1H), 3.73-3.69 (m, 3H), 3.67-3.65 (m, 2H), 3.64 (dd, J =9.9, 4.5 Hz, 1H), 3.58 (dd, J = 8.2, 6.4 Hz, 1H), 3.56 (t, J = 9.1 Hz, 1H), 3.50 (t, J = 9.4 Hz, 1H), 3.49-3.46 (m, 1H), 3.45 (t, J = 6.3 Hz, 1H), 3.42 (t, J = 9.6 Hz, 1H), 3.40 (dd, J = 10.2, 8.2 Hz, 1H), 3.36 (dd, J = 8.9, 8.2 Hz, 1H), 3.34-3.31 (m, 2H), 3.26 (dd, J = 10.1, 6.1 Hz, 1H). 3.25 (br, 1H, CH_2NBnCbz), 3.17 (br, 1H, CH_2NBnCbz), 2.99 (dd, J = 12.0, 3.6 Hz, 1H, D-3_{eq}), 2.55 (s, 3H, Ac), 2.05 (dd, J = 13.3, 12.0 Hz, 1H, D-3_{ax}), 1.91 (s, 3H, Ac), 1.61-1.46 (m, 4H), 1.38-1.21 (m, 4H), 1.01 (s, 9H, $C(CH_3)_3$); ^{13}C NMR (213 MHz, $CDCl_3$) δ 174.82, 169.63, 168.56, 165.28, <u>156.69</u>, <u>156.11</u>, 153.77, 138.84, 138.62, 138.43, 138.36, 138.13, 138.04, <u>137.90</u>,

137.86, 136.84, 136.74, 135.57, 132.85, 132.79, 132.70, 129.79, 129.77, 129.72, 128.46, 128.40, 128.36, 128.31, 128.28, 128.25, 128.20, 128.16, 127.87, 127.79, 127.76, 127.73, 127.72, 127.70, 127.66, 127.52, 127.46, 127.41, 127.36, 127.24, 127.16, 127.11, 127.04, 103.54, 102.18, 100.36, 97.95, 82.61, 81.53, 79.99, 77.93, 77.45, 77.20, 76.42, 75.47, 75.22, 75.09, 74.95, 74.90, 73.49, 73.35, 73.17, 72.96, 72.95, 72.73, 70.98, 69.77, 69.54, 69.34, 68.06, 67.94, 67.09, 67.05, 66.67, 62.76, 60.54, 58.18, 53.72, 50.39, 50.07, 47.10, 46.13, 35.50, 29.59, 28.01, 27.61, 26.67 (x3), 26.56, 25.87, 24.85, 20.47, 18.92 (conformational isomer peaks are underlined); HRMS (ESI) *m/z* for $C_{119}H_{133}N_5NaO_{28}Si$ $[M+Na]^+$: calcd 2130.8804, found 2130.8807.

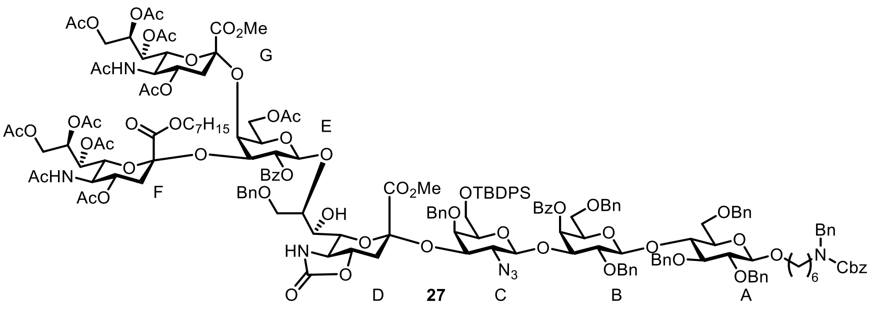

Compound 27. To a solution of compound **14** (9 mg, 6.6 μmol) in DCM (2 mL) was added K_2CO_3 (4.3 mg, 31 μmol) and trichloroacetonitrile (3 μL, 31 μmol) at rt. After being stirred for 16 h, the solid was filtered through a pad of celite®, and resulting solution was concentrated under reduced pressure to obtain the glycosyl donor. Next, compound **27** was synthesized from above glycosyl donor and glycosyl acceptor **25** (28 mg, 13 μmol) by following the **General glycosylation procedure B**. The residue was purified by Sephadex LH-20 (MeOH/DCM = 1/1) (EtOAc/Hexanes = 1/1 + 5% MeOH, twice, R_f = 0.19) to afford compound **27** (11 mg, 48% over 2 steps). ^{1}H NMR (850 MHz, CD_3CN) δ 8.00 (d, J =7.1 Hz, 2H), 7.86 (d, J =7.1 Hz, 2H), 7.64-7.63 (m, 2H), 7.61-7.60 (m, 2H), 7.52 (t, J = 7.4 Hz, 1H), 7.49 (t, J = 7.4 Hz, 1H), 7.45-7.41 (m, 4H), 7.37-7.24 (m, 32H), 7.21-7.16 (m, 12H), 7.14-7.13 (m, 2H), 7.09 (d, J = 7.3 Hz, 2H), 7.02 (t, J = 7.4 Hz, 1H), 6.92 (t, J = 7.6 Hz, 2H), 6.08 (d, J = 9.4 Hz, 1H, G_5-NH), 6.05

(d, J = 9.8 Hz, 1H, F_5-NH), 6.01 (br, 1H, D_5-NH), 5.42-5.40 (m, 2H, F-8, G-8), 5.39 (d, J = 3.4 Hz, 1H, B-4), 5.22-5.21 (m, 2H, F-7, G-7), 5.20 (dd, J = 10.5, 7.5 Hz, 1H, E-2), 5.11 (br, 2H, Cbz), 5.05 (d, J = 7.8 Hz, 1H, E-1), 5.02 (d, J = 10.6 Hz, 1H, A_3-Bn), 4.89 (ddd, J = 11.4, 10.3, 4.6 Hz, 1H, G-4), 4.82 (d, J = 10.7 Hz, 1H, B_2-Bn), 4.80 (d, J = 11.5 Hz, 1H, C_4-Bn), 4.78 (m, 1H, A_2-Bn), 4.74 (d, J = 10.7 Hz, 1H, B_2-Bn), 4.73 (ddd, J = 11.7, 10.1, 4.4 Hz, 1H, F-4), 4.67 (d, J = 10.6 Hz, 1H, A_3-Bn), 4.65 (d, J = 12.7 Hz, 1H, A_2-Bn), 4.55 (dd, J = 10.5, 2.4 Hz, 1H, E-3), 4.48 (d, J = 8.1 Hz, 1H, C-1), 4.47 (d, J = 7.6 Hz, 1H, B-1), 4.47-4.41 (m, 6H, N-Bn, N-Bn, A_6-Bn, C_4-Bn, E-6, $CO_2C\mathit{H}_2CH_2$), 4.38 (br, 1H, A-1), 4.36 (d, J = 12.0 Hz, 1H, B_6-Bn), 4.32 (d, J = 11.9 Hz, 1H, A_6-Bn), 4.25-4.21 (m, 3H, D_9-Bn, E-6, F-9), 4.20-4.18 (m, 2H, E-4, G-9), 4.16 (d, J = 12.0 Hz, 1H, B_6-Bn), 4.14 (d, J = 12.1 Hz, 1H, D_9-Bn), 4.12 (dt, J = 10.6, 6.8 Hz, 1H, $CO_2C\mathit{H}_2CH_2$), 4.09 (dd, J = 12.7, 4.6 Hz, 1H, G-9), 4.05-4.03 (m, 1H, D-8), 3.97-3.88 (m, 6H, A-4, C-3, D-4, F-9, G-5, G-6), 3.87-3.85 (m, 2H, D-6, F-6), 3.81 (br, 2H, D-7, E-5), 3.79-3.70 (m, 14H, A-6, B-2, B-3, C-4, C-6, D-9, F-5, $OC\mathit{H}_2CH_2$, $OC\mathit{H}_3$(x2)), 3.62 (d, J = 10.3 Hz, 1H, A-6), 3.57 (dd, J = 10.2, 6.6 Hz, 1H, C-6), 3.55-3.53 (m, 3H, A-3, B-5, D-9), 3.51 (br, 1H, $OC\mathit{H}_2CH_2$), 3.48-3.45 (m, 3H, A-5, C-5, D-5), 3.36 (dd, J = 10.5, 8.1 Hz, 1H, C-2), 3.35 (dd, J = 10.4, 5.6 Hz, 1H, B-6), 3.27 (d, J = 7.2 Hz, 1H, D_7-OH), 3.23-3.15 (m, 2H, $C\mathit{H}_2$NBnCbz), 3.22 (dd, J = 10.4, 6.6 Hz, 1H, B-6), 3.16 (dd, J = 8.5, 8.5 Hz, 1H, A-2), 2.88 (dd, J = 11.9, 3.6 Hz, 1H, D-3_{eq}), 2.43 (dd, J = 13.1, 4.6 Hz, 1H, G-3_{eq}), 2.29 (dd, J = 12.6, 4.4 Hz, 1H, F-3_{eq}), 2.15 (s, 3H, Ac), 2.07 (s, 3H, Ac), 2.06 (dd, J = 11.9, 11.9 Hz, 1H, D-3_{ax}), 2.04 (s, 3H, Ac), 1.98 (s, 3H, Ac), 1.96 (s, 3H, Ac), 1.95 (s, 3H, Ac), 1.94 (s, 3H, Ac), 1.81 (dd, J = 13.1, 11.4 Hz, 1H, G-3_{ax}), 1.79 (s, 3H, Ac), 1.78 (s, 3H, Ac), 1.73-1.69 (m, 2H, $C\mathit{H}_2$), 1.69 (s, 3H, Ac), 1.67 (s, 3H, Ac), 1.66 (dd, J = 12.6, 11.7 Hz, 1H, F-3_{ax}), 1.56-1.52 (m, 2H, $C\mathit{H}_2$), 1.46 (br, 2H, $C\mathit{H}_2$), 1.39-1.34 (m, 2H, $C\mathit{H}_2$), 1.33-1.26 (m, 10H, $C\mathit{H}_2$), 0.97 (s, 9H, $C(C\mathit{H}_3)_3$), 0.88 (t, J = 7.3 Hz, 3H, $C\mathit{H}_3$); ^{13}C NMR (213 MHz, CD_3CN) δ 171.62, 171.54, 171.46,

171.33, 171.08, 170.86, 170.72, 170.62, 170.53, 170.50, 170.38, 169.40, 168.80, 168.28, 166.36, 166.27, 160.32, 157.40, 156.83, 140.24, 140.05, 139.59, 139.58, 139.56, 139.53, 139.49, 139.39, 138.37, 136.47, 136.37, 134.15, 134.03, 134.01, 131.03, 130.99, 130.96, 130.92, 130.83, 130.51, 129.48, 129.45, 129.36 (x2), 129.26, 129.23, 129.21, 129.15, 129.14, 129.10, 128.96, 128.92, 128.84, 128.82, 128.71, 128.68, 128.65, 128.60, 128.53, 128.51, 128.38, 128.30, 128.28, 128.10, 128.01, 127.96, 104.18, 103.11, 102.75, 101.17, 100.85, 100.36, 99.32, 83.38, 82.46, 80.89, 79.79, 78.69, 77.69, 77.47, 77.06, 75.80 (x4), 75.72, 75.50, 75.40, 75.28, 73.92, 73.79, 73.70, 73.63, 73.05, 72.89, 72.76, 72.54, 71.63, 71.42, 71.26, 70.64, 70.32, 70.28 (x2), 69.27, 69.06, 69.00, 68.65, 67.88, 67.85, 67.60 (x2), 67.54, 65.48, 64.04, 63.22, 63.03, 62.90, 58.49, 54.14, 53.81, 51.19, 50.87, 49.52, 49.24, 48.10, 47.41, 38.37, 36.97, 36.74, 32.38, 30.37, 29.69, 29.24, 28.93, 28.46, 27.23, 27.16, 26.56, 26.45, 23.33, 23.22, 23.10, 21.61, 21.41, 21.32, 21.16, 21.14, 21.11, 21.07, 20.97, 20.96, 19.71, 14.43 (conformational isomer peaks are underlined); HRMS (ESI) *m/z* for $C_{183}H_{217}N_7Na_2O_{57}Si$ $[M+2Na]^{2+}$: calcd 1749.1931, found 1749.1915.

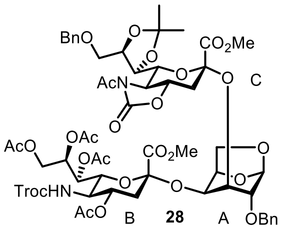

Compound 28. Compound **28** was synthesized from glycosyl acceptor **A15** (200 mg, 0.23 mmol) and glycosyl donor **5** (293 mg, 0.47 mmol) by following the **General glycosylation procedure A**. The mixture was purified by silica gel chromatography (EtOAc/Hexanes = 2/3 twice, R_f = 0.15) to afford compound **28** (278 mg, 91%). ^1H NMR (500 MHz, $CDCl_3$) δ 7.37 (d, *J* = 7.4 Hz, 2H), 7.31-7.21 (m, 8H), 5.34 (d, *J* = 8.0 Hz, 1H, B-7), 5.30 (br, 1H, A-1), 5.28-5.24 (m, 1H, B-8), 5.04 (ddd, *J* = 12.7, 12.7, 4.7 Hz, 1H, B-4), 4.94-4.92 (m, 2H, B_5-NH, C-7), 4.88 (d, *J* = 12.1 Hz, 1H, Troc), 4.75 (d,

J = 11.8 Hz, 1H, C*H*2Ph), 4.64 (d, *J* = 11.8 Hz, 1H, C*H*2Ph), 4.50-4.43 (m, 4H, C-8, Troc, C*H*2Ph(x2)), 4.33-4.30 (m, 3H, A4, A-5, A-6), 4.26 (dd, *J* = 12.3, 2.3 Hz, 1H, B-9), 4.17 (d, *J* = 3.8 Hz, 1H, A-3), 4.12 (br, 1H, A-2), 4.11 (dd, *J* = 12.3, 5.2 Hz, 1H, B-9), 3.99 (d, *J* = 10.9 Hz, 1H, B-6), 3.94 (dd, *J* = 9.9, 6.3 Hz, 1H, C-9), 3.93-3.89 (m, 2H, C-5, C-6), 3.85 (dd, *J* = 9.9, 6.0 Hz, 1H, C-9), 3.82 (s, 3H, OC*H*3), 3.79-3.74 (m, 1H, C-4), 3.77 (s, 3H, OC*H*3), 3.57-3.51 (m, 2H, A-6, B-5), 3.04 (dd, *J* = 11.7, 3.2 Hz, 1H, C-3_{eq}), 2.58 (dd, *J* = 12.7, 4.7 Hz, 1H, B-3_{eq}), 2.42 (s, 3H, Ac), 2.11-2.05 (m, 1H, C-3_{ax}), 2.11 (s, 3H, Ac), 2.08 (s, 3H, Ac), 2.02 (s, 3H, Ac), 1.96 (s, 3H, Ac), 1.87 (dd, *J* = 12.7, 12.7 Hz, 1H, B-3_{ax}), 15.1 (s, 3H, CC*H*3), 1.36 (s, 3H, CC*H*3); ^{13}C NMR (125 MHz, $CDCl_3$) δ 172.47, 170.43, 170.17, 169.73, 169.58, 167.57, 167.22, 154.01, 153.87, 138.52, 137.82, 128.32, 128.15, 127.71, 127.68, 127.53, 127.48, 108.65, 101.71, 99.33, 98.84, 95.28, 78.02, 76.36, 76.15, 75.57, 75.09, 74.36 (x2), 73.59, 73.12, 72.17, 71.58, 68.70, 68.61, 68.35, 67.38, 66.78, 64.27, 61.67, 60.20, 53.01, 52.79, 51.49, 37.11, 35.92, 26.85, 25.17, 24.98, 20.80, 20.74, 20.54 (x2); HRMS (ESI) *m/z* for $C_{57}H_{69}Cl_3N_2NaO_{27}$ $[M+Na]^+$: calcd 1341.3051, found 1341.3052.

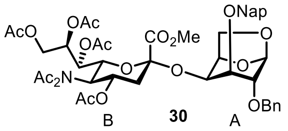

30

Compound 30. To a solution of compound **P14** (183 mg, 0.18 mmol) in ACN/AcOH (4 mL/1 mL) was added Ac_2O (0.69 mL, 7.33 mmol) and zinc dust (239 mg, 3.66 mmol) at rt. After being stirred at rt for 1 h, the solid was filtered through a pad of celite®, and resulting solution was concentrated under reduced pressure. Next, a solution of above residue in DCM (4 mL) was added *N*,*N*-diisopropylethylamine (0.16 mL, 0.92 mmol), acetyl chloride (0.26 mL, 3.66 mmol), and 4-(dimethylamino)pyridine (22 mg, 0.18 mmol) under a nitrogen atmosphere at 0 °C. The reaction mixture was gradually warmed rt. After being stirred for 6 h, the reaction mixture was concentrated *in vacuo*. The residue was extracted with EtOAc, and the resulting organic solution was washed

with H_2O, saturated $NaHCO_{3(aq)}$, and brine. The organic layer was dried over $MgSO_4$, filtered, and concentrated. The residue was purified by silica gel chromatography (EtOAc/Hexanes = 1/1, R_f = 0.50) to afford compound **30** (190 mg, quant.). ^{1}H NMR (500 MHz, $CDCl_3$) δ 7.82 (br, 4H), 7.53 (d, *J* = 8.4 Hz, 1H), 7.47-7.46 (m, 2H), 7.21 (br, 3H), 7.13 (br, 2H), 4.49 (ddd, *J* = 10.7, 10.7, 5.1 Hz, 1H, B-4), 5.44-5.52 (m, 1H, B-8), 5.28 (br, 1H, A-1), 5.15-5.12 (m, 2H, B-6, B-7), 4.91 (d, *J* = 12.0 Hz, 1H), 4.73 (d, *J* = 12.0 Hz, 1H), 4.60 (d, *J* = 12.6 Hz, 1H), 4.45 (d, *J* = 5.9 Hz, 1H, A-6), 4.31-4.21 (m, 5H, A-4, A-5, B-5, B-9, C*H*$_2$Ph), 4.14 (dd. *J* = 12.4, 5.9 Hz, 1H, B-9), 3.96 (d, *J* = 4.2 Hz, 1H, A-3), 3.85 (s, 3H, OC*H*$_3$), 3.60 (dd, *J* = 5.9, 5.9 Hz, 1H, A-6), 3.44 (br, 1H, A-2), 2.76 (dd, *J* = 13.0, 5.1 Hz, 1H, B-3$_{eq}$), 2.37 (s, 3H, Ac), 2.24 (s, 3H, Ac), 2.14 (s, 3H, Ac), 1.94-1.89 (m, 1H, B-3$_{ax}$), 1.94 (s, 3H, Ac), 1.93 (s, 3H, Ac), 1.92 (s, 3H, Ac); ^{13}C NMR (125 MHz, $CDCl_3$) δ 174.19, 173.59, 170.53, 170.04, 169.63, 169.56, 167.99, 137.72, 136.32, 133.24, 132.95, 128.31, 128.07, 127.82, 127.79, 127.72, 127.68, 126.25, 126.14, 125.94, 125.86, 100.53, 99.74, 77.14, 76.50, 74.02, 73.33, 71.58, 70.14, 69.26, 68.07, 67.22, 66.47, 64.19, 62.09, 56.74, 53.05, 38.84, 27.97, 26.14, 20.90, 20.76, 20.68, 20.59; HRMS (ESI) *m/z* for $C_{46}H_{53}NNaO_{18}$ [M+Na]$^+$: calcd 930.3160, found 930.3162.

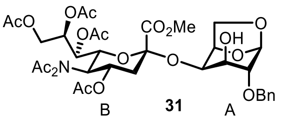

31

Compound 31. To a solution of compound **30** (166 mg, 0.18 mmol) in H_2O/DCM (1 mL/4 mL) was added 2,3-dichloro-5,6-dicyano-1,4-benzoquinone (125 mg, 0.55 mmol) and stirred at rt for 60 min. The reaction mixture was quenched with saturated $Na_2S_2O_{3(aq)}$. The reaction mixture was extracted with EtOAc, and the resulting organic solution was washed with saturated $NaHCO_{3(aq)}$ and brine. The organic layer was dried over $MgSO_4$, filtered, and concentrated. The residue was purified by silica gel chromatography (EtOAc/Hexanes = 3/2, R_f = 0.28) to afford compound **31** (132 mg,

94%). ^{1}H NMR (500 MHz, $CDCl_3$) δ 7.35 (d, *J* = 7.3 Hz, 2H), 7.31 (t, *J* = 7.3 Hz, 2H), 7.25 (t, *J* = 7.3 Hz, 1H), 5.45 (ddd, *J* = 7.8, 7.8, 5.3 Hz, 1H, B-4), 5.35 (br, 1H, A-1), 5.22-5.19 (m, 1H, B-8), 5.08 (d, *J* = 9.4 Hz, 1H, B-7), 4.99 (d, *J* = 9.4 Hz, 1H, B-6), 4.75 (d, *J* = 12.0 Hz, 1H, CH_2Ph), 4.55 (d, *J* = 12.0 Hz, 1H, CH_2Ph), 4.41 (dd, *J* = 4.1, 4.1 Hz, 1H, A-5), 4.29-4.25 (m, 4H, A-3, A-6, B-5, B9), 4.11-4.07 (m, 2H, A-4, B-9), 3.84 (s, 3H, OCH_3), 3.61 (dd, *J* = 6.1, 6.1 Hz, 1H, A-6), 3.55 (br, 1H, A-2), 3.14 (br, 1H, A_3-OH), 2.79 (dd, *J* = 14.1, 5.1 Hz, 1H, B-3_{eq}), 2.40 (s, 3H, Ac), 2.31 (s, 3H, Ac), 2.09 (s, 3H, Ac), 2.08-2.05 (m, 1H, B-3_{ax}), 2.06 (s, 3H, Ac), 2.00 (s, 3H, Ac), 1.96 (s, 3H, Ac); ^{13}C NMR (125 MHz, $CDCl_3$) δ 174.28, 173.28, 170.37, 169.92, 169.67, 169.62, 167.67, 137.68, 128.38, 127.96, 127.82, 100.58, 100.09, 77.79, 72.59, 71.97, 70.42, 70.07, 68.67, 68.32, 67.90, 66.65, 64.07, 61.52, 56.90, 53.27, 37.34, 27.84, 26.03, 20.83, 20.75, 20.70, 20.58; HRMS (ESI) *m/z* for $C_{35}H_{45}NNaO_{18}$ $[M+Na]^+$: calcd 790.2534, found 790.2533.

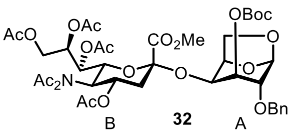

32

Compound 32. To a solution of compound **31** (107 mg, 0.14 mmol) in DCM (3 mL) was added *N*,*N*-diisopropylethylamine (0.12 mL, 0.70 mmol), di-tert-butyldicarbonate (0.32 mL, 1.39 mmol), and 4-(dimethylamino)pyridine (17 mg, 0.14 mmol) under a nitrogen atmosphere at rt. After being stirred for 16 h, the reaction mixture was concentrated *in vacuo*. The residue was extracted with EtOAc, and the resulting organic solution was washed with saturated $NaHCO_{3(aq)}$ and brine. The organic layer was dried over $MgSO_4$, filtered, and concentrated. The residue was purified by silica gel chromatography (EtOAc/Hexanes = 3/2, R_f = 0.40) to afford compound **32** (110 mg, 91%). ^{1}H NMR (500 MHz, $CDCl_3$) δ 7.35-7.29 (m, 4H), 7.26 (d, *J* = 6.9 Hz, 1H), 5.55 (ddd, *J* = 10.5, 10.5, 5.1 Hz, 1H, B-4), 5.27 (br, 1H, A-1), 5.27-5.26 (m, 1H, B-8), 5.18 (d, *J* = 5.0 Hz, 1H, A-3), 5.09 (d, *J* = 8.2 Hz, 1H, B-7), 4.91 (d, *J* = 10.5 Hz, 1H, B-6),

4.79 (d, J = 12.2 Hz, 1H, CH_2Ph), 4.58 (d, J = 12.2 Hz, 1H, CH_2Ph), 4.49 (dd, J = 5.0, 5.0 Hz, 1H, A-4), 4.40 (dd, J = 7.0, 5.0 Hz, 1H, A-5), 4.29 (d, J = 7.0 Hz, 1H, A-6), 4.18 (d, J = 12.3 Hz, 1H, B-9), 4.08 (dd, J = 12.3, 5.0 Hz, 1H, B-9), 4.06 (dd, J = 10.5, 10.5 Hz, 1H, B-5), 3.82 (s, 3H, OCH_3), 3.61 (dd, J = 7.0, 7.0 Hz, 1H, A-6), 3.42 (br, 1H, A-2), 2.71 (dd, J = 13.2, 5.1 Hz, 1H, B-3_{eq}), 2.35 (s, 3H, Ac), 2.27 (s, 3H, Ac), 2.14 (s, 3H, Ac), 2.08 (s, 3H, Ac), 1.96 (s, 3H, Ac), 1.95 (s, 3H, Ac), 1.81 (dd, J = 13.2, 10.5 Hz, 1H, B-3_{ax}), 1.46 (s, 9H, $C(CH_3)_3$); ^{13}C NMR (125 MHz, $CDCl_3$) δ 174.41, 173.63, 170.43, 170.25, 169.70, 169.51, 167.99, 153.14, 137.37, 128.47, 127.98, 100.15, 99.21, 81.93, 76.52, 73.33, 71.98, 71.66, 70.01, 68.83, 67.01, 66.76, 66.70, 64.07, 61.59, 57.26, 53.02, 37.77, 27.91, 27.82, 25.77, 20.88, 20.78, 20.72, 20.63; HRMS (ESI) *m/z* for $C_{40}H_{53}NNaO_{20}$ $[M+Na]^+$: calcd 890.3059, found 890.3061.

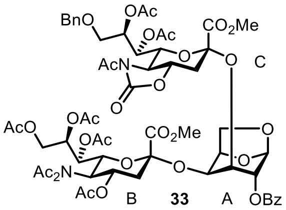

33

Compound 33. The synthesis of compound **33** is similar as described in the synthesis of compound **S29**, starting from compound **4** (273 mg, 0.22 mmol) to yield compound **33** (193 mg, 68%); EtOAc/DCM/Hexanes = 3/3/4 + 3% MeOH, R_f = 0.43. ^1H NMR (500 MHz, $CDCl_3$) δ 8.10 (d, J = 7.6 Hz, 2H), 7.44 (t, J = 7.6 Hz, 1H), 7.32 (t, J = 7.6 Hz, 2H), 7.24-7.20 (m, 3H), 7.04 (d, J = 7.3 Hz, 2H), 5.75 (d, J = 9.4 Hz, 1H, C-7), 5.59 (br, 1H, A-1), 5.54 (ddd, J = 11.3, 10.2, 5.1 Hz, 1H, B-4), 5.31 (ddd, J = 8.1, 4.7, 2.0 Hz, 1H, B-8), 5.17 (br, 1H, A-2), 5.12 (d, J = 8.1 Hz, 1H, B-7), 4.98 (ddd, J = 9.4, 3.3, 3.3 Hz, 1H, C-8), 4.79 (d, J = 10.2 Hz, 1H, B-6), 4.53 (d, J = 4.9 Hz, 1H, A-3), 4.44 (dd, J = 4.9, 4.9 Hz, 1H, A-4), 4.37-4.36 (m, 2H, A-5, A-6), 4.30 (dd, J = 12.4, 2.0 Hz, 1H, B-9), 4.24 (d, J = 10.2 Hz, 1H, B-5), 4.21 (dd, J =12.4, 4.7 Hz, 1H, B-9), 4.20 (d, J = 9.4 Hz, 1H, C-6), 4.06 (d, J = 12.1 Hz, 1H, CH_2Ph), 4.03 (d, J = 12.1 Hz, 1H,

CH_2Ph), 3.87-3.82 (m, 1H, C-4), 3.84 (s, 3H, OCH_3), 3.81 (s, 3H, OCH_3), 3.66 (dd, *J* = 6.2, 6.2 Hz, 1H, A-6), 3.63 (dd, *J* = 11.9, 9.4 Hz, 1H, C-5), 3.12 (dd, *J* = 12.0, 3.4 Hz, 1H, C-3_{eq}), 2.88 (br, 2H, C-9, C-9), 2.72 (dd, *J* = 13.1, 5.1 Hz, 1H, B-3_{eq}), 2.46 (s, 3H, Ac), 2.37 (s, 3H, Ac), 2.35 (s, 3H, Ac), 2.14-2.09 (m, 1H, C-3_{ax}), 2.14 (s, 3H, Ac), 2.10 (s, 3H, Ac), 2.05 (s, 3H, Ac), 2.01 (s, 3H, Ac), 2.00 (s, 3H, Ac), 1.98 (s, 3H, Ac), 1.87 (dd, *J* = 13.1, 11.3 Hz, 1H, B-3_{ax}); ^{13}C NMR (125 MHz, $CDCl_3$) δ 174.19, 173.86, 171.36, 170.56, 170.17, 170.07, 169.75, 169.63, 169.35, 167.53, 166.84, 165.24, 153.63, 137.99, 133.20, 130.06, 129.22, 128.29, 128.05, 127.65, 127.30, 99.40, 99.31, 98.43, 75.56, 74.83, 74.73, 73.98, 72.83, 72.35, 70.70, 69.91, 69.16, 68.77, 67.65, 66.87, 66.68, 66.42, 64.38, 61.55, 59.10, 56.84, 53.13, 53.11, 36.92, 36.84, 27.97, 26.13, 24.59, 21.34, 20.94, 20.78 (x2), 20.69, 20.67; HRMS (ESI) *m/z* for $C_{59}H_{70}N_2NaO_{30}$ $[M+Na]^+$: calcd 1309.3911, found 1309.3922.

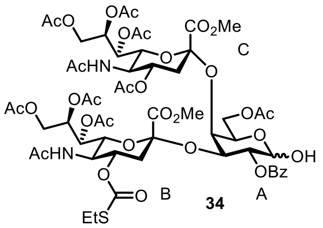

34

Compound 34. To a solution of compound **33** (100 mg, 0.08 mmol) in Ac_2O (4 mL) was added boron trifluoride diethyl etherate (96 μL, 0.78 mmol) under a nitrogen atmosphere at 0 °C. The reaction mixture was gradually warmed to rt and stirred for 6 h. Then the reaction was cooled to 0 °C and added pyridine (4 mL) and 4-(dimethylamino)pyridine (9 mg, 78 μmol). The reaction mixture was gradually warmed to rt and stirred for 10 h. The reaction mixture was concentrated *in vacuo*. The residue was extracted with EtOAc, and the resulting organic solution was washed with 1N $HCl_{(aq)}$, saturated $NaHCO_{3(aq)}$, and brine. The organic layer was dried over $MgSO_4$, filtered, and concentrated. Next, a solution of above residue in ACN (4 mL) was added ethanethiol (288 μL, 3.88 mmol) and potassium carbonate (32 mg, 0.23 mmol) at 0 °C.

The reaction mixture was gradually warmed to rt and stirred for 3 h. The reaction mixture was concentrated *in vacuo*. The residue was extracted with EtOAc, and the resulting organic solution was washed with saturated $NaHCO_{3(aq)}$ and brine. The organic layer was dried over $MgSO_4$, filtered, and concentrated. Next, a solution of above residue in DMF (4 mL) was added hydrazine acetate (43 mg, 0.47 mmol) at rt. The reaction mixture was stirred at rt for 6 h, then the reaction was extracted with EtOAc, and the resulting organic solution was washed with 1N $HCl_{(aq)}$, saturated $NaHCO_{3(aq)}$, and brine. The organic layer was dried over $MgSO_4$, filtered, and concentrated. The residue was purified by silica gel chromatography (EtOAc/Hexanes = 1/1 + 10% MeOH, twice, R_f = 0.34) to afford compound **34** as anomeric mixture (91 mg, 88% over 4 steps). ^{1}H NMR (500 MHz, $CDCl_3$) δ 8.11 (d, *J* = 7.3 Hz, 2H), 7.54 (t, *J* = 7.3 Hz, 1H), 7.48-7.45 (m, 2H), 5.52-5.50 (m, 0.3H), 5.42-5.38 (m, 3 H), 5.36-5.33 (m, 1H), 5.31-5.16 (m, 3.6H), 5.07 (ddd, *J* = 11.7, 11.7, 4.5 Hz, 0.3H), 5.02-4.95 (m, 2H), 4.66 (d, *J* = 10.3 Hz, 0.6H), 4.50-4.40 (m, 1.3H), 4.28-4.08 (m, 6.6H), 4.02-4.87 (m, 9H), 3.78-3.70 (m, 4H), 3.61 (m, 0.3H), 2.76-2.71 (m, 2H, SCH_2CH_3), 2.60 (dd, *J* = 12.6, 4.5 Hz, 0.3H), 2.56 (dd, *J* = 12.6, 3.9 Hz, 0.7H), 2.50 (dd, *J* = 13.2, 4.4 Hz, 0.3H), 2.44 (dd, *J* =13.1, 4.4 Hz, 0.7H), 2.18 (s, 2H), 2.15 (s, 1H), 2.08-2.07 (m, 6H), 2.05 (s, 3H), 2.02 (s, 1H), 2.01 (s, 2H), 1.99-1.97 (m, 10H), 1.87 (s, 3H), 1.83 (s, 2H), 1.81 (s, 1H), 1.76 (s, 2H), 1.22-1.19 (m, 3H, SCH_2CH_3); ^{13}C NMR (125 MHz, $CDCl_3$) δ 171.36, 171.22, 170.92, 170.81, 170.76, 170.70, 170.66, 170.55, 170.44, 170.31, 170.15, 169.70, 169.49, 169.36, 168.00, 167.74, 167.61, 167.55, 166.53, 165.73, 133.21, 130.16, 130.04, 129.85, 128.54, 128.47, 98.93, 98.78, 98.46, 97.77, 95.66, 91.32, 72.72, 72.66, 72.33, 72.15, 71.54, 71.46, 71.38, 70.68, 69.61, 69.51, 69.40, 68.35, 68.27, 68.17, 67.46, 67.03, 66.84, 66.74, 64.78, 64.65, 62.29, 62.16, 61.93, 53.30, 53.17, 53.09, 49.63, 49.57, 49.36, 49.26, 37.64, 37.57, 36.61, 36.29, 31.86, 29.63, 29.29, 25.37, 23.18, 23.13, 22.63, 21.31, 21.19, 20.96, 20.71, 20.68, 20.61, 20.58, 20.52, 20.41, 14.85,

14.06; HRMS (ESI) *m/z* for $C_{56}H_{74}N_2NaO_{32}S$ $[M+Na]^+$: calcd 1341.3843, found 1341.3849.

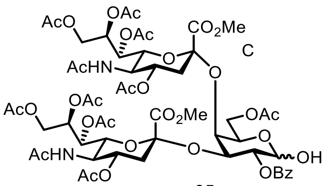

35

Compound 35. To a solution of compound **33** (100 mg, 77 μmol) in EtOAc (7 mL) was stirred under a H_2 atmosphere in the presence of palladium on carbon (80 mg, 10 wt%) at rt. After being stirred for 60 min, the solid was filtered through a pad of celite®, and resulting solution was concentrated *in vacuo*. Next, to a solution of above residue in THF (7 mL) was added tetrabutylammonium fluoride (233 μL, 233 μmol) under nitrogen atmosphere at rt. The reaction mixture was stirred for 30 min. The reaction mixture was concentrated *in vacuo*. Next, to a solution of above residue in pyridine (7 mL) was added Ac_2O (73 μL, 777 μmol) and 4-(dimethylamino)pyridine (10 mg, 77 μmol). The reaction mixture was stirred for 14 h. The reaction mixture was concentrated *in vacuo*. The residue was extracted with EtOAc, and the resulting organic solution was washed with 1N $HCl_{(aq)}$, saturated $NaHCO_{3(aq)}$, and brine. The organic layer was dried over $MgSO_4$, filtered, and concentrated. Next, to a solution of above residue in Ac_2O (4 mL) was added boron trifluoride diethyl etherate (96 μL, 0.78 mmol) under a nitrogen atmosphere at 0 °C. The reaction mixture was gradually warmed to rt and stirred for 1 h. The reaction mixture was quenched with Et_3N and concentrated *in vacuo*. The residue was extracted with EtOAc, and the resulting organic solution was washed with saturated $NaHCO_{3(aq)}$ and brine. The organic layer was dried over $MgSO_4$, filtered, and concentrated. Next, a solution of above residue in DMF (4 mL) was added hydrazine acetate (72 mg, 0.78 mmol) at rt. The reaction mixture was stirred at rt for 1.5 h, then the reaction was extracted with EtOAc, and the resulting organic solution

was washed with 1N $HCl_{(aq)}$, saturated $NaHCO_{3(aq)}$, and brine. The organic layer was dried over $MgSO_4$, filtered, and concentrated. The residue was purified by silica gel chromatography (DCM/Hexanes/MeOH = 6/4/1, twice, $R_f = 0.40$) to afford compound **35** as anomeric mixture (87 mg, 88% over 5 steps). ^{1}H NMR (500 MHz, CD_3CN) δ 8.11 (d, J = 7.7 Hz, 2H), 7.67-7.62 (m, 1H), 7.57-7.51 (m, 2H), 6.12-6.06 (m, 1.5H), 5.98 (d, J = 9.5 Hz, 0.5H), 5.48-5.45 (m, 0.5H), 5.43-5.40 (m, 1H), 5.34-5.33 (m, 1H), 5.27-5.22 (m, 2H), 5.15 (d, J = 9.1 Hz, 0.5H), 5.06 (dd, J = 10.1, 7.8 Hz, 0.5H), 4.89-4.88 (m, 1.5H), 4.83-4.71 (m, 2.5H), 4.49-4.40 (m, 1.5H), 4.32 (br, 0.5H), 4.29-4.17 (m, 2.5H), 4.14-4.02 (m, 3.5H), 3.98-3.91 (m, 4.5H), 3.82-3.81 (m, 3H),3.78 (s, 2H), 3.76-3.72 (m, 1H), 2.45 (dd, J = 13.0, 4.4 Hz, 0.5H), 2.40-2.35 (m, 1H), 2.32 (dd, J = 12.6, 4.1 Hz, 0.5H), 2.08-2.07 (m, 4.5H), 2.05 (d, 1.5H), 2.02-2.01 (m, 3H), 2.01 (s, 1.5H),1.98 (s, 1.5H), 1.96 (m, 3H), 1.94 (s, 3H), 1.91 (s, 1.5H), 1.91-1.85 (m, 2H), 1.85-1.80 (m, 2.5H), 1.78 (m, 3H), 1.74 (s, 1.5H), 1.68 (s, 1.5H), 1.63-1.57 (m, 0.5H), 1.57 (s, 1.5H); ^{13}C NMR (100 MHz, $CDCl_3$) δ 170.75, 170.75, 170.71, 170.69, 170.68, 170.62, 170.62, 170.60, 170.56, 170.41, 170.38, 170.35, 170.17, 169.91, 169.66, 169.41, 169.32, 169.30, 169.29, 168.01, 167.77, 167.55, 167.50, 167.49, 166.29, 165.71, 133.11, 130.04, 129.95, 129.85, 128.37, 98.90, 98.74, 98.44, 97.72, 95.50, 90.99, 72.58, 72.26, 72.22, 71.86, 70.65, 69.58, 69.49, 69.27, 69.18, 69.05, 68.38, 68.33, 68.26, 68.25, 67.44, 67.02, 66.83, 66.80, 66.51, 64.68, 64.60, 64.55, 62.23, 62.10, 61.88, 53.15, 53.04, 53.00, 52.95, 49.45, 49.35, 48.78, 48.53, 37.41, 37.32, 36.39, 36.02, 29.51, 23.04, 22.96, 21.19, 21.00, 20.83, 20.60, 20.58, 20.52, 20.48, 20.40, 20.27; HRMS (ESI) *m/z* for $C_{55}H_{72}N_2NaO_{32}$ $[M+Na]^+$: calcd 1295.3966, found 1295.3960.

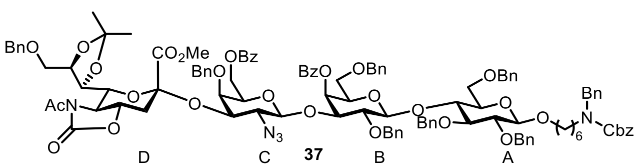

37

Compound 37α. Compound **37α** and **37β** was synthesized from glycosyl acceptor **6** (250 mg, 0.16 mmol) and glycosyl donor **5** (197 mg, 0.31 mmol) by following the **General glycosylation procedure A**. The mixture was purified by silica gel chromatography (EtOAc/Hexanes = 1/2, R_f = 0.20) to afford compound **37α** (234 mg, 73%) and (EtOAc/Hexanes = 1/2, R_f = 0.25) to afford compound **37β** (53 mg, 16%). ^{1}H NMR (600 MHz, $CDCl_3$) δ 8.06 (d, *J* = 7.4 Hz, 2H), 8.04 (d, *J* = 7.3 Hz, 2H), 7.51 (t, *J* = 7.4 Hz, 1H), 7.46 (t, *J* = 7.4 Hz, 1H), 7.36-7.18 (m, 46 H), 7.03 (t, *J* = 7.4 Hz, 1H), 6.91 (t, *J* = 7.6 Hz, 2H), 5.66 (d, *J* = 2.6 Hz, 1H, B-4), 5.17 (br, 1H, Cbz), 5.15 (br, 1H, Cbz), 5.02 (d, *J* = 10.6 Hz, 1H), 4.98 (d, *J* = 11.3 Hz, 1H), 4.93 (d, *J* = 7.7 Hz, 1H), 4.92 (d, *J* = 8.6 Hz, 1H), 4.86 (d, *J* = 10.8 Hz, 1H), 4.77 (d, *J* = 10.6 Hz, 1H), 4.75 (d, *J* = 11.4 Hz, 1H), 4.71 (d, *J* = 11.1 Hz, 1H), 4.65 (d, *J* = 7.3 Hz, 1H), 4.61-4.57 (m, 3H), 4.52 (d, *J* = 7.0 Hz, 1H), 4.48-4.42 (m, 5H), 4.39 (d, *J* = 10.7 Hz, 1H), 4.38 (d, *J* = 12.0 Hz, 1H), 4.36-4.34 (m, 2H), 4.19 (d, *J* = 11.8 Hz, 1H), 4.10-4.06 (m, 2H), 4.04 (d, *J* = 1.8 Hz, 1H), 4.02 (t, *J* = 9.4 Hz, 1H), 3.95-3.91 (m, 2H), 3.87-3.82 (m, 3H), 3.79-3.76 (m, 2H), 3.70-3.66 (m, 5H), 3.63-3.56 (m, 4H), 3.48-3.43 (m, 1H, OCH_2CH_2), 3.41-3.36 (m, 3H), 3.30 (dd, *J* = 10.1, 6.1 Hz, 1H), 3.24 (br, 1H, CH_2NBnCbz), 3.16 (br, 1H, CH_2NBnCbz), 2.77 (dd, *J* = 12.4, 3.4 Hz, 1H, D-3_{eq}), 2.49 (s, 3H, Ac), 1.98 (dd, *J* = 12.4, 12.4 Hz, 1H, D-3_{ax}), 1.60-1.46 (m, 4H), 1.37-1.21 (m, 4H), 1.35 (s, 3H, CCH_3), 1.23 (s, 3H, CCH_3); ^{13}C NMR (150 MHz, $CDCl_3$) δ 172.30, 167.42, 166.08, 165.28, <u>156.71</u>, <u>156.15</u>, 153.92, 138.86, 138.63, 138.58, 138.27, 138.17, 138.11, 137.91, 136.85, 133.12, 132.89, 129.91, 129.86, 129.48, 128.48, 128.39, 128.32, 128.28, 128.26, 128.23, 128.22, 127.89, 127.83, 127.81, 127.78, 127.73, 127.65, 127.52, 127.42, 127.40, 127.06, 107.78, 103.58, 102.44 (x2), 100.50, 82.62, 81.55, 80.93, 76.92, 76.47, 76.16, 76.13, 75.81, 75.74, 75.62, 75.43, 75.29, 75.06, 74.93 (x2), 73.71, 73.58, 73.35, 73.19, 73.17, 71.87, 70.54, 69.81, 69.02, 68.24, 68.03, 67.10, 62.15, 61.94, 59.90, 53.04, <u>50.41</u>, <u>50.11</u>, <u>47.12</u>, <u>46.15</u>, 34.22, 29.61, <u>28.02</u>, <u>27.63</u>, 27.17, 26.59, 25.87, 25.20,

24.11 (conformational isomer peaks are underlined); HRMS (ESI) *m/z* for $C_{118}H_{127}N_5NaO_{28}$ $[M+Na]^+$: calcd 2084.8565, found 2084.8536.

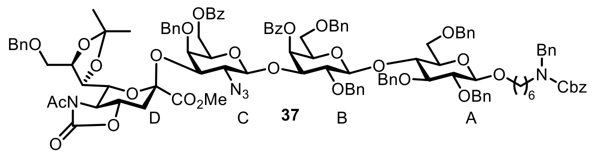

Compound 37β. ^{1}H NMR (600 MHz, $CDCl_3$) δ 8.05 (d, *J* = 7.1 Hz, 2H), 7.95 (d, *J* = 7.1 Hz, 2H), 7.52-7.49 (m, 2H), 7.40-7.16 (m, 44H), 7.10 (d, 6.7 Hz, 2H), 7.03 (t, *J* = 7.4 Hz, 1H), 6.92 (t, *J* = 7.6 Hz, 2H), 5.71 (d, *J* = 2.9 Hz, 1H, B-4), 5.17 (br, 1H, Cbz), 5.16 (br, 1H, Cbz), 5.04 (d, *J* = 10.6 Hz, 1H), 4.95 (d, *J* = 10.6 Hz, 1H), 4.89-4.87 (m, 2H), 4.78 (d, *J* = 10.6 Hz, 1H), 4.76 (d, *J* = 12.1 Hz, 1H), 4.74 (d, *J* = 10.7 Hz, 1H), 4.72 (d, *J* = 10.8 Hz, 1H), 4.71 (d, *J* = 11.8 Hz, 1H), 4.68 (d, *J* = 6.8 Hz, 1H), 4.57 (d, *J* = 12.1 Hz, 1H), 4.52 (d, *J* = 7.2 Hz, 1H), 4.50-4.46 (m, 3H), 4.41-4.34 (m, 5H), 4.29-4.24 (m, 4H), 4.20 (d, *J* = 11.9 Hz, 1H), 4.03 (dd, *J* = 9.4, 9.4 Hz, 1H, A-4), 3.89-3.83 (m, 3H), 3.82-3.77 (m, 3H), 3.74 (d, *J* = 9.6 Hz, 1H), 3.68-3.59 (m, 9H), 3.51 (t, *J* = 6.2 Hz, 1H), 3.50-3.44 (m, 1H, OCH_2CH_2), 3.44-3.37 (m, 3H), 3.32 (dd, *J* = 10.1, 6.2 Hz, 1H), 3.25 (br, 1H, $CH_2NBnCbz$), 3.17 (br, 1H, $CH_2NBnCbz$), 2.74 (dd, *J* = 12.7, 3.7 Hz, 1H, D-3_{eq}), 2.37 (s, 3H, Ac), 2.04 (dd, *J* = 12.7, 12.7 Hz, 1H, D-3_{ax}), 1.62-1.46 (m, 4H), 1.38 (s, 3H, CCH_3), 1.36-1.22 (m, 4H), 1.34 (s, 3H, CCH_3); ^{13}C NMR (150 MHz, $CDCl_3$) δ 172.67, 166.62, 165.88, 165.24, 156.70, 156.13, 153.61, 138.84, 138.62, 138.13, 138.11, 138.06, 137.90, 137.73, 137.61, 136.86, 136.76, 133.29, 132.90, 129.91, 129.81, 129.55, 128.50, 128.47, 128.41, 128.26, 128.23, 128.18, 128.04, 127.91, 127.82, 127.78, 127.77, 127.67, 127.63, 127.54, 127.49, 127.37, 127.19, 127.09, 108.66, 103.59, 102.55, 102.36, 99.24, 82.58, 81.56, 80.51, 76.67, 76.47, 75.47, 75.41, 75.30 (x2), 75.28, 75.13, 74.91, 74.64, 74.42, 73.98, 73.54, 73.22, 73.20, 73.17, 72.33, 70.76, 69.80, 68.94, 68.23, 68.05, 67.08 (x2), 62.88, 62.55, 60.28, 52.51, 50.40, 50.08, 47.11, 46.13, 35.39, 29.61, 28.02, 27.63, 26.85, 26.57, 25.86, 25.45, 24.74

(conformational isomer peaks are underlined); HRMS (ESI) *m/z* for $C_{118}H_{127}N_5NaO_{28}$ $[M+Na]^+$: calcd 2084.8565, found 2084.8564.

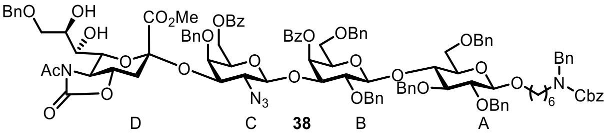

Compound 38. The synthesis of compound **38** is similar as described in the synthesis of compound **25**, starting from glycosyl acceptor **6** (620 mg, 0.39 mmol) and glycosyl donor **5** (486 mg, 0.77 mmol) to yield compound **38** (468 mg, 64% over 2 steps); EtOAc/Hexanes = 2/3, R_f = 0.28. ^{1}H NMR (500 MHz, $CDCl_3$) δ 8.06 (d, *J* = 7.9 Hz, 2H), 7.92 (d, *J* = 8.0 Hz, 2H), 7.53 (t, *J* = 7.1 Hz, 1H), 7.46 (t, *J* = 7.4 Hz, 1H), 7.37-7.19 (m, 46H), 7.05 (t, *J* = 7.3 Hz, 1H), 6.94 (t, *J* = 7.3 Hz, 2H), 5.68 (d, *J* = 2.8 Hz, 1H, B-4), 5.19 (br, 1H, Cbz), 5.18 (br, 1H, Cbz), 5.04 (d, *J* = 10.7 Hz, 1H), 4.94 (d, *J* = 11.3 Hz, 1H), 4.90-4.87 (m, 2H), 4.83 (d, *J* = 11.3 Hz, 1H), 4.79 (d, *J* = 10.6 Hz, 1H), 4.73 (d, *J* = 11.1 Hz, 1H), 4.67 (t, *J* = 3.5 Hz, 1H), 4.62-4.57 (m, 2H), 4.56-4.48 (m, 5H), 4.44 (d, *J* = 12.4 Hz, 1H), 4.41-4.33 (m, 4H), 4.21 (d, *J* = 11.8 Hz, 1H), 4.10-3.98 (m, 3H), 3.93-3.83 (m, 5H), 3.81-3.65 (m, 11H), 3.62-3.56 (m, 3H), 3.51-3.43 (m, 2H), 3.43-3.39 (m, 2H), 3.37-3.32 (m, 3H), 3.26 (br, 1H, CH_2NBnCbz), 3.19 (br, 1H, CH_2NBnCbz), 2.96 (d, *J* = 12.5, 3.2 Hz, 1H, D-3_{eq}), 2.59 (s, 3H, Ac), 2.16 (dd, *J* = 12.5, 12.5 Hz, 1H, D-3_{ax}), 1.61-1.48 (m, 4H), 1.38-1.24 (m, 4H); ^{13}C NMR (125 MHz, $CDCl_3$) δ 174.89, 168.30, 165.72, 165.17, <u>156.54</u>, <u>155.98</u>, 153.51, 138.75, 138.52, 138.19, 138.02, 137.96, 137.77, <u>136.71</u>, <u>136.64</u>, 132.95, 132.74, 129.82, 129.68, 129.48, 129.42, 128.34, 128.24, 128.19, 128.17, 128.13, 128.09, 128.06, 127.77, 127.75, 127.69, 127.64, 127.55, 127.52, 127.50, 127.44, 127.38, 127.23, 127.16, 127.12, 127.07, 126.95, 103.44, 102.21, 101.89, 99.80, 82.48, 81.41, 80.40, 77.46, 77.38, 76.30, 75.11, 75.06, 75.04, 74.94, 74.86, 74.77, 74.73, 74.62, 73.40, 73.10, 73.03, 73.01, 71.69, 70.22, 69.69, 69.64, 69.24, 68.14, 67.87, 66.94 (x2), 62.05, 61.83, 58.03, 53.52, <u>50.27</u>, <u>49.96</u>, <u>46.98</u>, <u>46.01</u>, 34.01, 29.46, <u>27.88</u>, <u>27.49</u>, 26.41, 25.71, 24.70 (conformational

isomer peaks are underlined); HRMS (ESI) *m/z* for $C_{115}H_{123}N_5NaO_{28}$ [M+Na]$^+$: calcd 2044.8252, found 2044.8227.

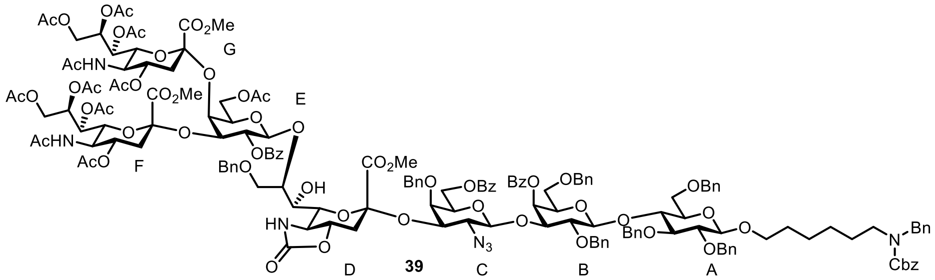

Compound 39. Compound **39** was synthesized from glycosyl acceptor **3** (27 mg, 13 μmole) and glycosyl donor **2** (15 mg, 11 μmole) by following the **General glycosylation procedure A**. The mixture was purified by silica gel chromatography (DCM/Hexanes/MeOH = 6/4/1, R_f = 0.43) to afford compound **39** (18 mg, 49%). ^{1}H NMR (850 MHz, CD_3CN) δ 8.02 (d, *J* = 7.3 Hz, 2H), 7.96 (t, *J* = 7.0 Hz, 4H), 7.56 (t, *J* = 7.5 Hz, 1H), 7.54 (t, *J* = 7.5 Hz, 1H), 7.48 (t, *J* = 7.4 Hz, 1H), 7.42 (t, *J* = 7.5 Hz, 2H), 7.39 (d, *J* = 7.5 Hz, 2H), 7.36-7.19 (m, 41H), 7.13 (t, *J* = 7.4 Hz, 1H), 7.10 (d, *J* = 7.4 Hz, 2H), 7.02 (t, *J* = 7.4 Hz, 1H), 6.92 (t, *J* = 7.5 Hz, 2H), 6.14 (d, *J* = 9.5 Hz, 1H, G_5-NH), 6.05 (s, 1H, D_5-NH), 6.03 (d, *J* = 9.7 Hz, 1H, F_5-NH), 5.48 (d, *J* = 3.0 Hz, 1H, B-4), 5.48-5.46 (m, 1H, F-8), 5.43-5.41 (m, 1H, G-8), 5.25 (dd, *J* = 9.8, 0.6 Hz, 1H, G-7), 5.22 (dd, *J* = 10.3, 7.8 Hz, 1H, E-2), 5.20 (dd, *J* = 9.4, 2.4 Hz, 1H, F-7), 5.11-5.09 (br, 2H, Cbz), 5.09 (d, *J* = 7.8 Hz, 1H, E-1), 5.03 (d, *J* = 10.6 Hz, 1H, A_3-Bn), 4.92 (ddd, *J* = 11.7, 9.7, 4.6 Hz, 1H, G-4), 4.86 (d, *J* = 11.2 Hz, 1H, C_4-Bn), 4.85 (d, *J* = 10.7 Hz, 1H, B_2-Bn), 4.82-4.79 (br, 1H, A_2-Bn), 4.78 (d, *J* = 10.7 Hz, 1H, B_2-Bn), 4.78-4.76 (m, 1H, F-4), 4.68 (d, *J* = 10.6 Hz, 1H, A_3-Bn), 4.67-4.66 (br, 1H, A_2-Bn), 4.56 (dd, *J* = 10.3, 2.0 Hz, 1H, E-3), 4.53 (d, *J* = 11.2 Hz, 1H, C_4-Bn), 4.51 (d, *J* = 7.4 Hz, 1H, B-1), 4.48 (d, *J* = 8.2 Hz, 1H, C-1), 4.46 (d, *J* = 12.1 Hz, 1H, A_6-Bn), 4.46 (dd, *J* = 11.7, 4.3 Hz, 1H, E-6), 4.44 (br, 2H, NBn), 4.41 (dd, *J* = 11.0, 6.9 Hz, 1H, C-6), 4.40-4.37 (br, 1H, A-1), 4.36 (d, *J* = 12.1 Hz, 1H, B_6-Bn), 4.34 (d, *J* = 12.1 Hz, 1H, A_6-Bn), 4.26 (d, *J* = 12.0 Hz, 1H, D_9-Bn), 4.24 (dd, *J* = 11.7, 7.7 Hz, 1H, E-6), 4.22-4.19 (m, 2H, F-

9, G-9), 4.16-4.13 (m, 4H, B_6-Bn, C-6, D_9-Bn, E-4), 4.09 (dd, J = 12.6, 4.5 Hz, 1H, G-9), 4.08-4.06 (m, 1H, D-8), 3.98-3.93 (m, 3H, C-3, D-4, G-5), 3.92-3.90 (m, 3H, A-4, F-9, G-6), 3.92 (s, 3H, F_1-OCH_3), 3.88 (d, J = 11.0 Hz, 1H, D-6), 3.86-3.85 (m, 1H, D-7), 3.84-3.80 (m, 3H, D-9, E-5, F-6), 3.79 (s, 3H, D_1-OCH_3), 3.78-3.73 (m, 6H, OCH_2CH$_2$, A-6, B-2, B-3, C-4, F-5), 3.76 (s, 3H, G_1-OCH_3), 3.64-3.59 (m, 3H, A-6, B-5, C-5), 3.56 (dd, J = 9.1, 9.1 Hz, 1H, A-3), 3.55-3.53 (m, 1H, D-9), 3.51 (dd, J = 11.0, 11.0 Hz, 1H, D-5), 3.50-3.48 (m, 2H, OCH_2CH$_2$, A-5), 3.44 (dd, J = 10.4, 8.2 Hz, 1H, C-2), 3.35 (dd, J = 10.4, 5.1 Hz, 1H, B-6), 3.28 (d, J = 7.1 Hz, 1H, D_7-OH), 3.22 (dd, J = 10.4, 6.9 Hz, 1H, B-6), 3.22-3.17 (br, 2H, CH_2NBnCbz), 3.17 (dd, J = 9.1, 9.1 Hz, 1H, A-2), 2.88 (dd, J = 12.1, 3.7 Hz, 1H, D-3_{eq}), 2.48 (dd, J = 13.1, 4.6 Hz, 1H, G-3_{eq}), 2.35 (dd, J = 12.6, 4.5 Hz, 1H, F-3_{eq}), 2.16 (s, 3H, Ac), 2.13 (dd, J = 13.1, 12.1 Hz, 1H, D-3_{ax}), 2.08 (s, 3H, Ac), 2.04 (d, 3H, Ac), 1.98 (s, 3H, Ac), 1.96 (s, 3H, Ac), 1.95 (s, 6H, Ac), 1.86 (dd, J = 13.1, 11.7 Hz, 1H, G-3_{ax}), 1.81 (s, 3H, Ac), 1.79 (s, 3H, Ac), 1.69 (s, 3H, Ac), 1.64 (dd, J = 12.6, 12.6 Hz, 1H, F-3_{ax}), 1.63 (s, 3H, Ac), 1.56-1.52 (br, 2H,OCH$_2$CH_2), 1.46 (br, 2H, CH$_2$CH_2NBnCbz), 1.35-1.29 (br, 2H), 1.26-1.20 (br, 2H); ^{13}C NMR (213 MHz, CD_3CN) δ 171.59 , 171.53 , 171.44 , 171.30 , 171.11 , 170.86 , 170.82 , 170.67 , 170.59 , 170.45 , 170.38 , 169.31 , 168.95 , 168.82 , 166.71 , 166.39 , 166.29 , 160.32 , 157.38 , 156.82 , 140.25 , 140.05 , 139.60 , 139.55 , 139.48 , 139.41 , 138.36 , 134.32 , 134.15 , 134.07 , 131.02 , 130.99 , 130.90 , 130.84 , 130.53 , 130.35 , 129.64 , 129.48 , 129.44 , 129.36 , 129.32 , 129.29 , 129.22 , 129.14 , 129.10 , 129.06 , 128.80 , 128.71 , 128.69 , 128.64 , 128.52 , 128.47 , 128.37 , 128.26 , 128.21 , 128.10 , 127.98 , 127.93 , 104.17 , 103.16 , 103.10 , 101.42 , 100.89 , 100.30 , 99.19 , 83.36 , 82.45 , 80.55 , 79.79 , 79.59 , 77.70 , 77.51 , 77.06 , 75.98 , 75.78 , 75.75 , 75.71 , 75.61 , 75.39 , 75.28 , 74.01 , 73.93 , 73.72 , 73.66 , 73.60 , 73.15 , 72.91 , 72.82 , 72.77 , 72.29 , 72.10 , 71.49 , 71.23 , 70.62 , 70.35 , 70.27 , 70.23 , 69.58 , 69.02 , 69.00 , 68.42 , 67.86 , 67.69 , 67.61 , 65.32 , 63.79 , 63.71 , 63.01 , 62.81 , 58.29 , 54.10 , 54.04 , 53.83 , 51.19 ,

50.87 , 49.57 , 48.99 , 48.09 , 47.40 , 38.30 , 37.04 , 36.29 , 30.36 , 28.93 , 28.45 , 27.16 , 26.56 , 23.20 , 23.12 , 21.66 , 21.40 , 21.29 , 21.12 , 21.08 , 21.05 , 21.00 , 20.97 (x2) (underline is conformational isomer); HRMS (ESI) *m/z* for $C_{168}H_{191}N_7Na_2O_{58}$ $[M+2Na]^{2+}$: calcd 1640.1003, found 1640.1013.

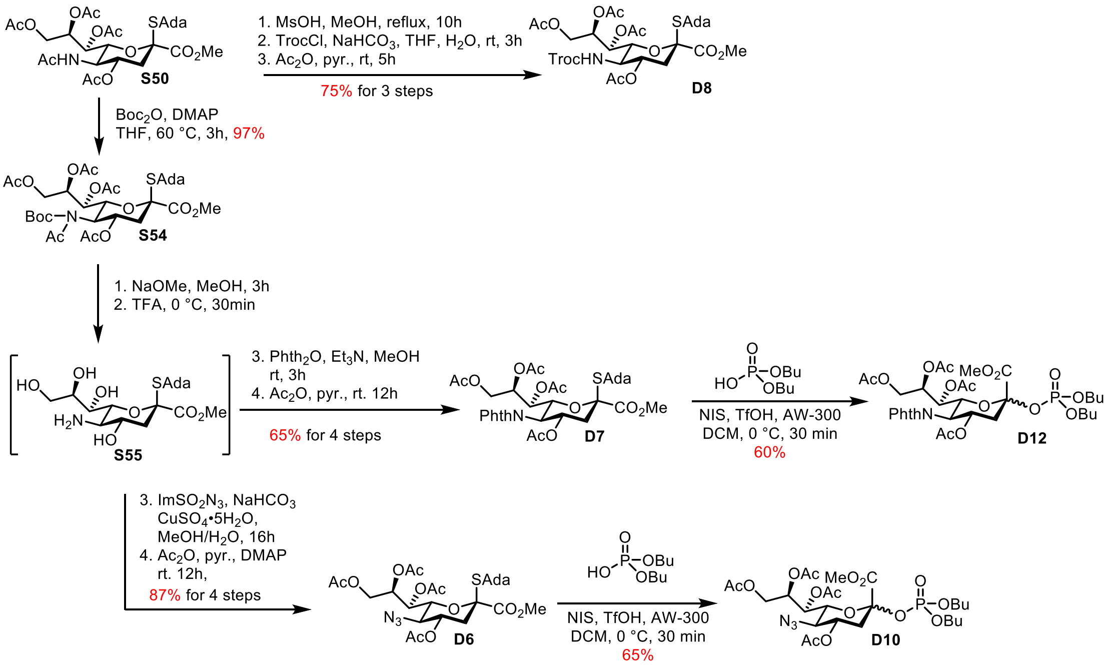

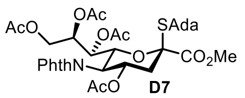

Compound D7. To a solution of compound **S54** (257 mg, 0.40 mmol) in MeOH (8 mL) was added sodium methoxide (6 mg, 0.12 mmol) under a nitrogen atmosphere at 0 °C. The reaction mixture was gradually warmed to rt and stirred for 3 h. The reaction mixture was neutralized with Amberlite® IR-120 (H^+) resin and filtered off the resin. The resulting solution was concentrated under reduced pressure. Next, a solution of above residue in TFA (2 mL) was stirred at 0 °C for 30 min and then concentrated *in vacuo*. To a solution of above residue in MeOH (4 mL) was added Et_3N (110 µL, 0.79 mmol) and phthalic anhydride (116 mg, 0.79 mmol) at 0 °C. The reaction mixture was gradually warmed to rt and stirred for 3 h. The mixture was concentrated *in vacuo*. To

a solution of the above residue in pyridine (4 mL) was added acetic anhydride (237 μL, 2.37 mmol) under a nitrogen atmosphere at 0 °C. The reaction mixture was gradually warmed to rt and stirred for 12 h. The mixture was concentrated *in vacuo*. The residue was dissolved in EtOAc and the resulting organic solution was washed with 1N HCl, saturated $NaHCO_3$, and brine. The organic layer was dried over $MgSO_4$, filtered, and concentrated. The residue was purified by silica gel chromatography (EtOAc/Hexanes = 1/2, R_f = 0.29) to afford compound **D7** (187 mg, 65% over four steps). ^{1}H NMR (500 MHz, $CDCl_3$) δ 7.86-7.71 (m, 2H, NPhth), 7.71-7.62 (m, 2H, NPhth), 5.83 (td, *J* = 10.6, 4.8 Hz, 1H, H-4), 5.69 (dd, *J* = 10.6, 2.4 Hz, 1H, H-6), 5.27-5.24 (m, 2H, H-7, H-8), 4.93 (d, *J* = 12.1 Hz, 1H, H-9), 4.21 (dd, *J* = 12.1 8.2 Hz, 1H, H-9), 4.11 (t, *J* = 10.6 Hz, 1H, H-5), 3.80 (s, 3H, OMe), 2.67 (dd, *J* = 13.5, 4.8 Hz, 1H, H-3_{eq}), 2.06 (s, 3H, Ac), 2.04-1.96 (m, 6H, SAda), 1.93-1.88 (m, 10H, H-3_{ax}, Ac, SAda), 1.75 (s, 3H, Ac), 1.63 (br, 6H, SAda); ^{13}C NMR (125 MHz, $CDCl_3$) δ 170.37, 170.19, 170.02, 169.98, 169.58, 167.65 (x2), 134.29 (x2), 131.26 (x2), 123.46 (x2), 85.88, 72.62, 69.49, 68.68, 66.36, 63.32, 52.72, 50.88, 50.46, 43.31 (x3), 40.31, 35.83 (x3), 29.72 (x3), 20.82, 20.58, 20.54, 20.51; HRMS (ESI) *m/z* for $C_{36}H_{43}N_1O_{13}S_1Na$ $[M+Na]^+$: calcd 752.2353, found 752.2362.

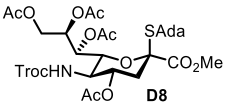

D8

Compound D8. To a solution of compound **S50** (2.00 g, 3.12 mmol) in MeOH (31 mL) was added methanesulfonic acid (0.61 mL, 9.35 mmol) under a nitrogen atmosphere at rt. The reaction was gradually warmed to 80 °C. After being stirred for 10 h, the reaction mixture was quenched with Et_3N and concentrated *in vacuo*. Next, to a solution of above residue in THF/H_2O (15 mL/15 mL) was added $NaHCO_3$ (1.31 g, 15.58 mmol) and 2,2,2-trichloroethoxycarbonyl chloride (0.85 mL, 6.23 mmol) at rt. After being stirred for 3 h, the reaction mixture was concentrated *in vacuo*. The residue was

extracted with EtOAc, and the resulting organic solution was washed with H_2O and brine. The organic layer was dried over $MgSO_4$, filtered, and concentrated. Next, to a solution of above residue in Ac_2O/pyridine (10 mL/10 mL) was stirred at rt for 5 h. The reaction mixture was concentrated *in vacuo*. The residue was extracted with EtOAc, and the resulting organic solution was washed with 1N $HCl_{(aq)}$, saturated $NaHCO_{3(aq)}$, and brine. The organic layer was dried over $MgSO_4$, filtered, and concentrated. The residue was purified by silica gel chromatography (EtOAc/Hexanes = 1/2, R_f = 0.28) to afford compound **D8** (1.82 g, 75% over 3 steps). ^{1}H NMR (500 MHz, $CDCl_3$) δ 5.49 (dd, J = 2.3, 2.3 Hz, 1H, H-7), 5.31 (ddd, J = 12.0, 10.4, 4.7 Hz, 1H, H-4), 5.17 (ddd, J = 8.6, 2.3, 1.4 Hz, 1H, H-8), 4.95 (dd, J = 12.4, 1.4 Hz, 1H, H-9), 4.91 (d, J = 10.4 Hz, 1H, NH), 4.85 (d, J = 12.0 Hz, 1H, C$H_2$$CCl_3$), 4.56 (dd, J = 10.4, 2.3 Hz, 1H, H-6), 4.45 (d, J = 12.0 Hz, 1H, C$H_2$$CCl_3$), 4.21 (dd, J = 12.4, 8.6 Hz, 1H, H-9), 3.81 (s, 3H, OCH_3), 3.71 (ddd, J = 10.4, 10.4, 10.4 Hz, 1H, H-5), 2.59 (dd, J = 13.6, 4.7 Hz, 1H, H-3_{eq}), 2.12 (s, 3H, Ac), 2.05 (s, 3H, Ac), 1.99 (s, 3H, Ac), 1.99-1.96 (m, 6H, SAda), 1.96 (s, 3H, Ac), 1.90 (dd, J = 13.6, 12.0 Hz, 1H, H-3_{ax}), 1.85-1.83 (m, 3H, SAda), 1.67-1.61 (m, 6H, SAda); ^{13}C NMR (125 MHz, $CDCl_3$) δ 170.60, 170.03, 169.80, 169.56, 169.47, 154.06, 95.16, 85.60, 73.91, 73.23, 71.98, 69.13, 68.37, 62.88, 52.38, 51.29, 49.95, 43.04 (x3), 39.55, 35.49 (x2), 29.33 (x3), 20.56, 20.44, 20.28, 20.22; HRMS (ESI) *m/z* for $C_{31}H_{42}Cl_3NNaO_{13}S$ $[M+Na]^+$: calcd 796.1340, found 796.1334.

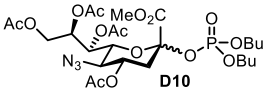

D10

Compound D10. Compound **D10** was synthesized from dibutyl phosphate (68 μL, 343.6 μmol) and glycosyl donor **D6** (50 mg, 85.9 mmol) by following the **General glycosylation procedure A**. The mixture was purified by silica gel chromatography (EtOAc/Hexanes = 1/1.5, R_f = 0.19) to give compound **D10** as anomers (65% over two steps, α : β = 2:1). For anomers: ^{1}H NMR (500 MHz, $CDCl_3$) δ 5.55 (d, J = 6.3 Hz, 1H,

β-H7), 5.49 (d, *J* = 9.2 Hz, 1H, α-H7), 5.30-5.20 (m, 3H, β-H4, H6, H8), 4.88 (ddd, *J* = 12.0, 9.6, 4.8 Hz, 1H, α-H4), 4.45 (dd, *J* = 12.6, 2.0 Hz, 1H, β-H9), 4.32 (dd, *J* = 12.6, 1.7 Hz, 1H, α-H9), 4.26 (dd, *J* = 12.6, 5.7 Hz, 1H, β-H9), 4.20 (dd, *J* = 12.6, 4.0 Hz, 1H, α-H9), 4.11-3.95 (m, 4H, O C*H*$_2$), 3.83 (s, 3H, β-OMe), 3.80 (s, 3H, OMe), 3.32-3.26 (m, 1H, H5), 2.79-2.72 (m, 1H, H3$_{eq}$), 2.32 (t, *J* = 12.4 Hz, 1H, α-H3$_{ax}$), 2.16 (s, 3H, Ac), 2.10-2,02 (m, 10H, β-H3$_{ax}$, Ac), 1.68-1.60 (m, 4H, C*H*$_2$), 1.41-1.35 (m, 4H, C*H*$_2$), 0.93-0.89 (m, 6H, C*H*$_3$); ^{13}C NMR (125 MHz, CDCl$_3$) δ 170.64, 169.85, 169.60, 169.58, 166.63, 97.98 (d, *J* = 7.0 Hz), 72.60, 70.62, 68.34, 67.97 (d, *J* = 6.2 Hz), 67.80 (d, *J* = 6.2 Hz), 67.40, 61.49, 59.42, 53.22, 36.72 (d, *J* = 4.4 Hz), 32.04 (d, *J* = 4.5 Hz), 31.98 (d, *J* = 4.5 Hz), 20.78, 20.77, 20.70, 20.62, 18.56, 18.52, 13.49 (x2). HRMS (ESI) *m/z* for $C_{26}H_{42}N_3O_{15}P_1Na$ [M+Na]$^+$: calcd 690.2251, found 690.2263.

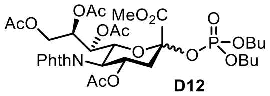

D12

Compound D12. Compound **D12** was synthesized from dibutyl phosphate (82 μL, 0.41 mmol) and glycosyl donor **D7** (100 mg, 0.14 mmol) by following the **General glycosylation procedure A**. The mixture was purified by silica gel chromatography (EtOAc/Hexanes = 1/1, R$_f$ = 0.47) to give compound **D12** (63 mg, 60% for β-anomer). For β-anomer: ^{1}H NMR (500 MHz, CDCl$_3$) δ 7.82 (br, 2H), 7.71-7.70 (m, 2H), 5.85 (td, *J* = 10.8, 5.1 Hz, 1H, H-4), 5.39 (dd, *J* = 10.7, 2.0 Hz, 1H, H-6), 5.27-5.17 (m, 3H, H-5, H-7, H-8), 4.44 (dd, *J* = 12.3, 1.9 Hz, 1H, H-9), 4.27-4.12 (m, 5H, H-9, OC*H*$_2$), 3.84 (s, 3H, OMe), 2.86 (dd, *J* = 13.8, 5.1 Hz, 1H, H-3$_{eq}$), 2.15-2.08 (m, 4H, H-3$_{ax}$, Ac), 2.00 (s, 3H, Ac), 1.95 (s, 3H, Ac), 1.81 (s, 3H, Ac), 1.72-1.67 (m, 4H, C*H*$_2$), 1.45-1.38 (m, 4H, C*H*$_2$), 0.96-0.91 (m, 6H, C*H*$_3$); ^{13}C NMR (125 MHz, CDCl$_3$) δ 170.62, 170.07, 169.75, 169.72, 167.72, 167.63, 165.95, 134.48 (x2), 131.31, 131.16, 123.62 (x2), 99.34 (d, *J* = 4.9 Hz), 70.22, 69.14, 68.53 (d, *J* = 6.4 Hz), 68.34 (d, *J* = 6.4 Hz), 67.60, 65.98, 62.21, 53.33, 50.08, 37.20 (d, *J* = 4.2 Hz), 32.15 (d, *J* = 4.6 Hz), 32.10 (d, *J* =

4.6 Hz), 20.73, 20.72, 20.65, 20.55, 18.59 (x2), 13.59 (x2); HRMS (ESI) *m/z* for $C_{34}H_{46}NO_{17}P_1Na$ $[M+Na]^+$: calcd 794.2401, found 794.2407.

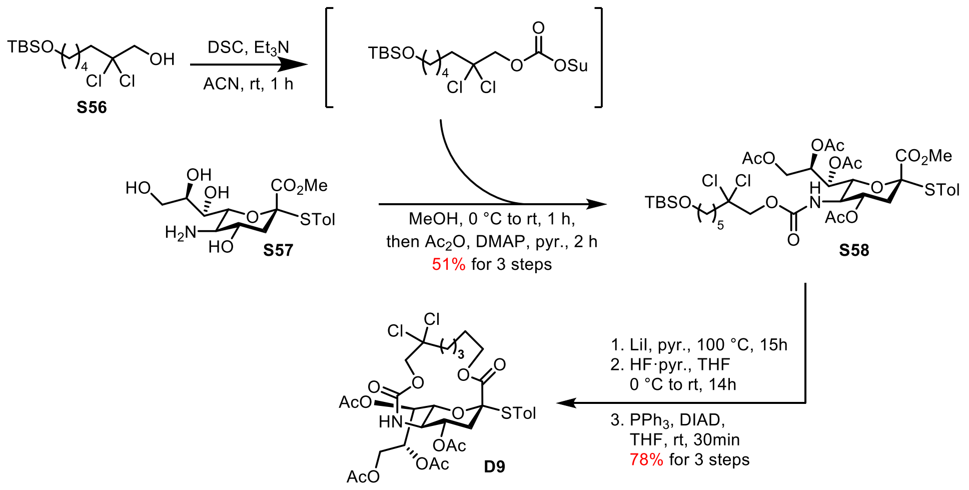

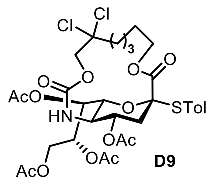

Compound D9. To a solution of compound **S58** (35 mg, 39.3 μmol) in pyridine (2 mL) was added LiI (26 mg, 196.4 μmol) at rt. The reaction mixture was stirred at 100 °C for 15 h and then concentrated *in vacuo*. The residue was extracted with EtOAc, and the resulting solution was washed with 1N $HCl_{(aq)}$ and brine. The organic layer was dried over $MgSO_4$, filtered, and concentrated. The residue was purified by silica gel chromatography (MeOH/DCM = 1/9, R_f = 0.22) to afford demethylated product. Next, to a solution of demethylated product in THF (5 mL) was slowly added HF·pyridine (36 μL, 1.97 μmol) at 0 °C. The reaction mixture was gradually warmed to rt and stirred for 14 h. The reaction was quenched with saturated $NaHCO_{3(aq)}$ and concentrated *in vacuo*. The residue was extracted with EtOAc, and the resulting solution was washed with H_2O and brine. The organic layer was dried over $MgSO_4$, filtered, and concentrated. A solution of triphenylphosphine (103 mg, 0.39 mmol) in THF (3 mL)

was slowly added a solution of above residue and diisopropyl azodicarboxylate (77 μL, 0.39 mmol) in THF (3 mL) at rt for 2 h. After being stirred at rt for 30 min, the reaction mixture was quenched with AcOH and MeOH, and then concentrated *in vacuo*. The residue was purified by silica gel chromatography (EtOAc/Hexanes = 1/3, R_f = 0.08) to afford compound **D9** (23 mg, 78% over three steps). ^{1}H NMR (600 MHz, 80℃, CD_3NO_2) δ 7.44 (d, *J* = 8.0 Hz, 2H), 7.20 (d, *J* = 8.0 Hz, 2H), 5.48-5.43 (m, 2H, H-4. NH), 5.39 (dd, *J* = 6.5, 1.4 Hz, 1H, H-7), 5.27 (td, *J* = 6.5, 2.9 Hz, 1H, H-8), 4.66 (d, *J* = 12.1 Hz, 1H, NH(CO)OC*H*$_2$), 4.48-4.40 (m, 4H, H-6, H-9, CO_2C*H*$_2$, NH(CO)OC*H*$_2$), 4.33-4.27 (m, 1H, H-9), 3.99-3.95 (m, CO_2C*H*$_2$), 2.97 (dd, *J* = 13.0, 5.1 Hz, 1H, H-3_{eq}), 2.90 (td, *J* = 10.5, 6.9 Hz, 1H, H-5), 2.47-2.42 (m, 1H, Cl_2CC*H*$_2$), 2.36 (s, 3H, PhMe), 2.27-2.23 (m, 1H, Cl_2CC*H*$_2$,), 2.01 (s, 3H, Ac), 2.00 (s, 3H, Ac), 2.00 (s, 3H, Ac), 1.87-1.77 (m, 3H, H-3_{ax}, C*H*$_2$), 1.66-1.53 (m, 4H, C*H*$_2$); ^{13}C NMR (150 MHz, 80℃, CD_3NO_2) δ 167.01, 166.70, 166.28, 166.06, 163.91, 150.23, 136.76, 132.52 (x2), 125.62 (x2), 121.33, 85.92, 83.11, 66.86, 66.16 (x2), 65.81, 64.88, 63.85 ,60.53, 48.83, 41.04, 33.98, 22.58, 20.07, 18.43, 15.97, 15.79, 15.49. 15.37; HRMS (ESI) *m/z* for $C_{32}H_{41}Cl_2NO_{13}SNa$ [M+Na]$^+$: calcd 772.1563, found 772.1565.

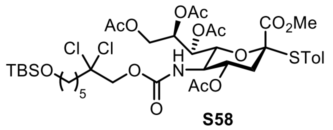

S58

Compound S58. To a solution of compound **S56** (3.90 g, 12.37 mmol) in ACN (30 mL) were added Et_3N (3.4 mL, 24.73 mmol) and *N,N'*-disuccinimidyl carbonate (3.17 g, 12.37 mmol) at rt under a nitrogen atmosphere. After being stirred at rt for 1 h, the reaction mixture was used for the condensation reaction without any purification. Next, to a solution of **S57** (1.28 g, 2.63 mmol) in MeOH (30 mL) was added to the above solution at 0 °C. The reaction mixture was gradually warmed to rt and stirred for 1 h. The mixture was concentrated *in vacuo*. The residue was extracted with EtOAc, and the resulting solution was washed with H_2O and brine. The organic layer was dried over

$MgSO_4$, filtered, and concentrated. To a solution of the above residue in DCM (50 mL) was added pyridine (1.7 mL, 21.00 mmol), acetic anhydride (2.0 mL, 21.00 mmol) and 4-dimethylaminopyridine (32 mg, 0.26 mmol) at 0 °C under a nitrogen atmosphere. The reaction mixture was gradually warmed to rt and stirred for 2 h. The mixture was concentrated *in vacuo*. The residue was extracted with EtOAc, and the resulting solution was washed with 1N HCl, saturated $NaHCO_{3(aq)}$, and brine. The organic layer was dried over $MgSO_4$, filtered, and concentrated. The residue was purified by silica gel chromatography (EtOAc/Hexanes = 1/3, R_f = 0.24) to afford compound **S58** (1.20 g, 51% over 3 steps). ^{1}H NMR (500 MHz, $CDCl_3$) δ 7.36 (d, *J* = 7.9 Hz, 2H), 7.11 (d, *J* = 7.9 Hz, 2H), 5.33 (d, *J* = 7.4 Hz, 1H, H-7), 5.27-5.24 (m, 1H, H-8), 4.87 (dd, *J* = 12.3, 4.6 Hz, 1H, H-4), 4.73 (d, *J* = 10.0 Hz, NH), 4.59 (d, *J* = 11.9 Hz, 1H, NH(CO)OCH_2), 4.36 (dd, *J* = 12.4, 2.2 Hz, 1H, H-9), 4.21-4.17 (m, 2H, H-9, NH(CO)OCH_2), 3.91 (d, *J* = 10.7 Hz, 1H, H-6), 3.60-3.52 (m, 6H, H-5, CH_2OTBDMS, OMe), 2.80 (dd, *J* = 12.3, 4.6 Hz, 1H, H-3_{eq}), 2.33 (s, 3H, PhMe), 2.15-2.07 (m, 5H, Cl_2CCH_2, Ac), 2.03 (s, 3H, Ac), 2.02 (s, 3H, Ac), 1.98 (s, 3H, Ac), 1.91 (t, *J* = 12.3 Hz, 1H, H-3_{ax}), 1.66-1.59 (m, 2H, CH_2), 1.55-1.49 (m, 2H, CH_2), 1.39-1.35 (m, 2H, CH_2), 0.86 (s, 9H, *t*Bu), 0.02 (s, 6H, CH_3); ^{13}C NMR (125 MHz, $CDCl_3$) δ 170.62, 170.38, 169.86 (x2), 167.83, 164.60, 140.15, 136.38 (x2), 129.56 (x2), 124.93, 90.19, 87.22, 74.25, 71.47, 69.60, 69.28, 67.65, 62.79, 61.82, 52.68, 51.28, 44.05, 38.09, 32.44, 25.89 (x3), 25.21, 24.42, 21.26, 20.88, 20.80, 20.75, 20.69, 18.27, -5.37 (x2); HRMS (ESI) *m/z* for $C_{39}H_{59}Cl_2NO_{14}SNa$ $[M+Na]^+$: calcd 918.2700, found 918.2694.

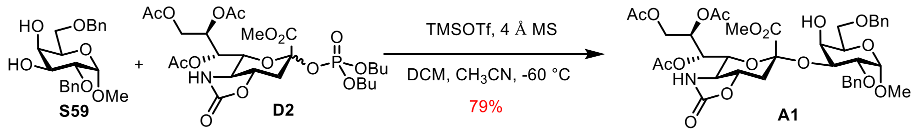

Compound A1. Compound **A1** was synthesized from glycosyl acceptor **S59** (34 mg, 0.11 mmole) and glycosyl donor **D2** (57 mg, 0.09 mmole) by following the **General**

glycosylation procedure B. The mixture was purified by silica gel chromatography (EtOAc/Hexanes = 2/1, R_f = 0.33) to afford compound **A1** (57 mg, 79%). ^{1}H NMR (400 MHz, $CDCl_3$) δ 7.24-7.30 (m, 10H, ArH), 5.30-5.33 (m, 2H, H-8', NH), 5.06 (dd, J = 9.2, 1.4 Hz, 1H, H-7'), 4.70 (d, J = 12.1 Hz, 1H, $PhCH_2$), 4.65 (d, J = 3.7 Hz, 1H, H-1), 4.50-4.58 (m, 3H, $PhCH_2$), 4.21-4.26 (m, 2H, H-3, H-9'), 4.15 (dd, J = 10.1, 1.4 Hz, 1H, H-6'), 3.97-4.12 (m, 3H, H-4, H-4', H-9'), 3.86 (dd, J = 5.9, 5.7 Hz, 1H, H-5), 3.72-3.79 (m, 5H, H-2, H-6, CO_2Me), 3.65 (dd, J = 9.8, 5.9 Hz, 1H, H-6), 3.37 (s, 3H, OMe), 3.01 (dd, J = 10.7, 10.1 Hz, 1H, H-5'), 2.78-2.83 (m, 2H, H-3'$_{eq}$, OH), 2.37 (dd, J = 12.8, 12.5 Hz, 1H, H-3'$_{ax}$), 2.11 (s, 3H, $(CO)CH_3$), 2.01 (s, 6H, $(CO)CH_3$); ^{13}C NMR (100 MHz, $CDCl_3$) δ 171.17, 170.38, 169.60, 168.19 ($^3J_{C1',H3'ax}$ = 5.5 Hz), 159.16, 138.16, 138.04, 128.34 (2C), 128.32 (2C), 127.79 (3C), 127.62, 127.56 (2C), 100.40, 98.70, 76.59, 73.85, 73.83, 73.60, 73.53, 73.38, 69.82, 69.41, 68.86, 68.22, 67.44, 61.62, 57.71, 55.28, 53.18, 35.26, 20.90, 20.56, 20.42; HRMS (ESI) m/z for $C_{38}H_{47}NO_{17}Na$ $[M+Na]^+$: calcd 812.2742, found 812.2750.

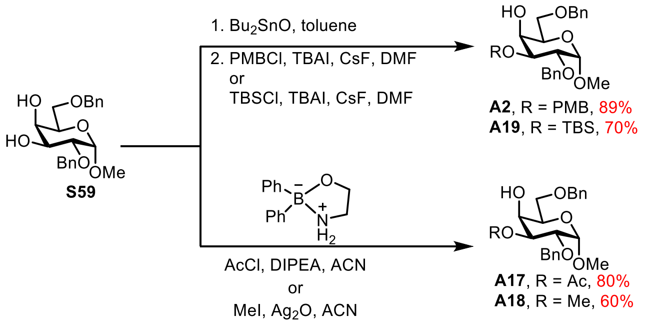

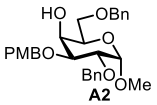

Compound A2. Compound **S59** (370 mg, 0.99 mmol) was dissolved in toluene (10.0 mL). To this solution, dibutyltin oxide (295 mg, 1.19 mmol) was added. The mixture was refluxed at 110 °C for 3 hours using a Dean-Stark apparatus. After cooling to room temperature, the Dean-Stark apparatus was removed. *p*-Methoxybenzyl chloride (0.27

mL, 2.0 mmol), tetrabutylammonium iodide (730 mg, 1.98 mmol), and cesium fluoride (300 mg, 1.98 mmol) were then added. The reaction mixture was heated to 80 °C and refluxed for 16 hours. After the reaction was complete, the solvent was removed. The residue was extracted with ethyl acetate and brine. The organic layer was dried over $MgSO_4$, filtered, and concentrated in vacuo. The residue was purified by column chromatography to afford compound **A2** (435 mg, 89% for two steps, EtOAc/Hexanes = 1/2, R_f = 0.31). ^{1}H NMR (400 MHz, $CDCl_3$) δ 7.27-7.39 (m, 12H, ArH), 6.88 (d, *J* = 8.2 Hz, 2H, ArH), 4.82 (d, *J* = 12.0 Hz, 1H, $PhCH_2$), 4.58-4.73 (m, 5H, H-1, $PhCH_2$), 4.55 (d, *J* = 12.0 Hz, 1H, $PhCH_2$), 4.01 (s, 1H, H-4), 3.90 (dd, *J* = 6.5, 5.6 Hz, 1H, H-5), 3.86 (br, 2H, H-2, H-3), 3.78 (s, 3H, OMe), 3.74 (dd, *J* = 9.8, 5.6 Hz, 1H, H-6), 3.68 (dd, *J* = 9.8, 6.5 Hz, 1H, H-6), 3.39 (s, 3H, OMe), 2.73 (s, 1H, OH); ^{13}C NMR (100 MHz, $CDCl_3$) δ 159.16, 138.28, 137.89, 130.09, 129.31 (2C), 128.20 (4C), 127.84 (2C), 127.57 (2C), 127.46 (3C), 113.69, 98.42, 77.14, 75.53, 73.39, 73.32, 72.23, 69.46, 68.25, 67.89, 55.11, 55.05; HRMS (ESI) *m/z* for $C_{29}H_{34}O_7Na$ $[M+Na]^+$: calcd 517.2202, found 517.2197.

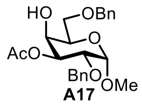

A17

Compound A17. Compound **S59** (90 mg, 0.24 mmol) and borane catalyst (5.4 mg, 0.02 mmol) were dissolved in acetonitrile (2.4 mL). To this solution, diisopropylethylamine (63 μL, 0.36 mmol) and acetyl chloride (21 μL, 0.29 mmol) were added. The mixture was heated to 70 °C and reacted for 4 hours. After the reaction was complete, the mixture was filtered through Celite. The filtrate was concentrated in vacuo, and the residue was purified by column chromatography to afford compound **A17** (80 mg, 80%) (EtOAc/Hexanes = 1/1, R_f = 0.53). ^{1}H NMR (400 MHz, $CDCl_3$) δ 7.25-7.34 (m, 10H, ArH), 5.20 (dd, *J* = 10.3, 2.7 Hz, 1H, H-3), 4.72 (d, *J* = 3.4 Hz, 1H, H-1), 4.68 (d, *J* = 12.3 Hz, 1H, $PhCH_2$), 4.59 (d, *J* = 12.3 Hz, 1H, $PhCH_2$), 4.58 (d, *J* =

11.9 Hz, 1H, $PhCH_2$), 4.52 (d, J = 11.9 Hz, 1H, $PhCH_2$), 4.22 (br, 1H, H-4), 3.98 (dd, J = 10.3, 3.4 Hz, 1H, H-2), 3.93 (dd, J = 4.5, 4.4 Hz, 1H, H-5), 3.74 (dd, J = 10.4, 4.5 Hz, 1H, H-6), 3.71 (dd, J = 10.4, 4.4 Hz, 1H, H-6), 3.36 (s, 3H, OMe), 3.06 (s, 1H, OH), 2.08 (s, 3H, (CO)CH_3); ^{13}C NMR (100 MHz, $CDCl_3$) δ170.18, 138.21, 137.25, 128.47 (2C), 128.37 (2C), 127.90, 127.76 (3C), 127.70 (2C), 98.70, 73.84, 73.57, 73.28, 72.24, 70.46, 69.52, 67.68, 55.41, 21.07; HRMS (ESI) *m/z* for $C_{23}H_{28}O_7Na$ $[M+Na]^+$: calcd 439.1733, found: 439.1733.

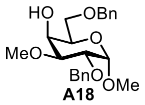

Compound A2. Compound **S59** (275 mg, 0.73 mmol), silver oxide (188 mg, 0.810 mmol), and borane catalyst (33 mg, 0.15 mmol) were dissolved in acetonitrile (7.3 mL). To this solution, methyl iodide (MeI, 69 µL, 1.10 mmol) was added. The mixture was heated to 70 °C and reacted for 4 hours. After the reaction was complete, the mixture was filtered through Celite. The filtrate was concentrated in vacuo, and the residue was purified by column chromatography (Hexanes/EtOAc, 3/1 v/v) to afford compound **A18** (171 mg, 60%) (EtOAc/Hexanes = 1/1, R_f = 0.38). ^{1}H NMR (400 MHz, $CDCl_3$) δ 7.24-7.36 (m, 10H, ArH), 4.79 (d, J = 12.2 Hz, 1H, $PhCH_2$), 4.53-4.64 (m, 4H, $PhCH_2$), 4.11 (d, J = 2.4 Hz, 1H, H-4), 3.89 (dd, J = 6.1, 5.7 Hz, 1H, H-5), 3.72-3.79 (m, 2H, H-3, H-6), 3.67 (dd, J = 10.0, 6.1 Hz, 1H, H-6), 3.58 (dd, J = 9.8, 3.2 Hz, 1H, H-2), 3.51 (s, 3H, OMe), 3.36 (s, 3H, OMe), 2.67 (bs, 1H, OH); ^{13}C NMR (100 MHz, $CDCl_3$) δ 138.38, 137.84, 128.28 (2C), 128.24 (2C), 127.85 (2C), 127.55 (4C), 98.49, 79.23, 75.39, 73.51, 73.36, 69.57, 68.23, 67.10, 57.94, 55.19; HRMS (ESI) *m/z* for $C_{22}H_{28}O_6Na$ $[M+Na]^+$: calcd 411.1784, found 411.1784.

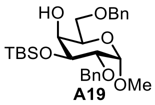

Compound A19. Compound **S59** (190 mg, 0.51 mmol) was dissolved in toluene (10.0 mL). To this solution, dibutyltin oxide (152 mg, 0.61 mmol) was added. The mixture was refluxed at 110 °C for 3 hours using a Dean-Stark apparatus. After cooling to room temperature, the Dean-Stark apparatus was removed. *tert*-Butyldimethylsilyl chloride (TBSCl, 153 mg, 1.02 mmol), tetrabutylammonium iodide (375 mg, 1.02 mmol), and cesium fluoride (154 mg, 1.02 mmol) were then added. The reaction mixture was heated to 80 °C and refluxed for 1 hour. After the reaction was complete, the solvent was removed. The residue was extracted with ethyl acetate and saturated brine. The organic layer was dried over magnesium sulfate, filtered, and concentrated in vacuo. The residue was purified by column chromatography to afford compound **A19** (174 mg, 70% for two steps) (EtOAc/Hexanes = 1/2, R_f = 0.60). ^{1}H NMR (400 MHz, $CDCl_3$) δ; ^{13}C NMR (100 MHz, $CDCl_3$) δ; HRMS (ESI) *m/z* for $C_{29}H_{34}O_7Na$ $[M+Na]^+$: calcd 517.2202, found 517.2197.

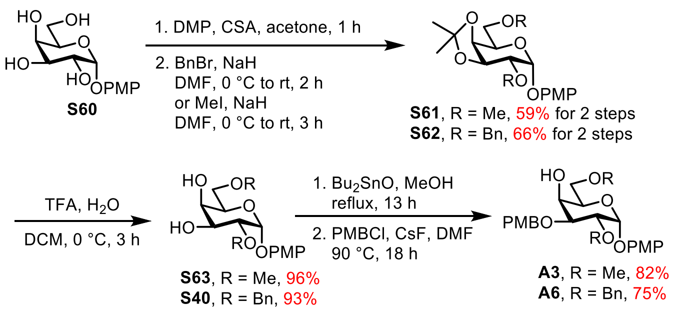

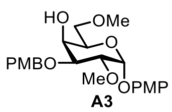

A3

Compound A3. The synthesis of compound **A3** is similar as described in the synthesis of compound **A6**, starting from compound **S63** (114 mg, 0.36 mmol) to yield compound **A3** (130 mg, 82% over two steps); EtOAc/Hexanes = 1/1, R_f = 0.32. ^{1}H NMR (500 MHz, $CDCl_3$) δ 7.31 (d, J = 8.2 Hz, 2H), 7.04 (d, J = 8.7 Hz, 2H), 6.87 (d, J = 8.2 Hz,

2H), 6.80 (d, J = 8.7 Hz, 2H), 5.52 (d, J = 3.3 Hz, 1H, H-1), 4.72 (d, J = 11.1 Hz, 1H, CH_2Ph), 4.64 (d, J = 11.1 Hz, 1H, CH_2Ph), 4.08 (s, 1H, H-4), 4.03 (t, J = 5.6 Hz, 1H, H-5), 3.94 (dd, J = 9.8, 2.7 Hz, 1H, H-3), 3.78-3.76 (m, 4H, H-2, OMe), 3.74 (s, 3H, OMe), 3.63 (dd, J = 9.9, 5.6 Hz, 1H, H-6), 3.56-3.54 (m, 4H, H-6, OMe), 3.32 (s, 3H, OMe), 2.83 (br, 1H, OH); ^{13}C NMR (125 MHz, $CDCl_3$) δ 159.24, 154.98, 150.83, 130.12, 129.36 (x2), 118.34 (x2), 114.41 (x2), 113.79 (x2), 96.63, 77.30, 77.04, 72.11, 71.67, 68.67, 67.79, 59.16, 59.10, 55.48, 55.15; HRMS (ESI) *m/z* for $C_{23}H_{30}O_8Na$ $[M+Na]^+$: calcd 457.1838, found 457.1896.

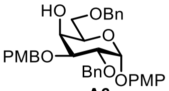

A6

Compound A6. A solution of compound **S40** (610 mg, 1.31 mmol) and dibutyltin oxide (373 mg, 1.96 mmol) in MeOH (15 mL) was refluxed for 13 h under a nitrogen atmosphere and then concentrated *in vacuo*. To a solution of the above residue and CsF (218 mg, 1.44 mmol) in DMF (15 mL) was added 4-methoxybenzyl chloride (264 μL, 1.96 mmol) under a nitrogen atmosphere. After being stirred at 90 °C for 18 h, the solution was concentrated under reduced pressure. The residue was dissolved in EtOAc, and the resulting organic solution was washed with H_2O and brine. The organic layer was dried over $MgSO_4$, filtered, and concentrated. The residue was purified by silica gel chromatography (EtOAc/Hexanes = 1/2, R_f = 0.62) to get compound **A6** (576 mg, 75% over two steps). ^{1}H NMR (400 MHz, $CDCl_3$) δ 7.36-7.23 (m, 12H), 7.05-7.01 (m, 2H), 6.91-6.86 (m, 2H), 6.80-6.75 (m, 2H), 5.34 (d, J = 3.6 Hz, 1H, H-1), 4.81 (d, J = 12.0 Hz, 1H, CH_2Ph), 4.78 (d, J = 10.9 Hz, 1H, CH_2Ph), 4.68 (d, J = 12.0 Hz, 1H, CH_2Ph), 4.68 (d, J = 10.9 Hz, 1H, CH_2Ph), 4.49 (s, 2H, CH_2Ph), 4.14-4.11 (m, 2H, H-4, H-5), 4.04 (dd, J = 9.7, 3.1 Hz, 1H, H-3), 3.96 (dd, J = 9.7, 3.6 Hz, 1H, H-2), 3.80 (s, 3H, OMe), 3.76-3.72 (m, 4H, H-6, OMe), 3.66 (dd, J = 10.1, 6.2 Hz, 1H, H-6), 2.67 (br, 1H, OH); ^{13}C NMR (100 MHz, $CDCl_3$) δ 159.36, 155.10, 151.04, 138.29, 137.98,

130.25, 129.47 (x2), 128.39 (x2), 128.30 (x2), 127.86 (x2), 127.74, 127.57 (x3), 118.74 (x2), 114.46 (x2), 113.88 (x2), 97.42, 77.26, 75.54, 73.43, 73.33, 72.48, 69.49, 69.06, 67.92, 55.57, 55.27. HRMS (ESI) *m/z* for $C_{35}H_{38}O_8Na$ [M+Na]$^+$: calcd 609.2464, found 609.2498.

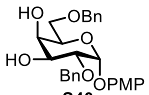

S40

Compound S40. To a solution of compound **S62** (1.00 g, 1.97 mmol) in DCM (20 mL) was added TFA (2.5 mL) and H_2O (1.25 mL) at 0 °C. The reaction mixture was stirred at 0 °C for 3 h and then neutralized with saturated $NaHCO_{3(aq)}$. The reaction mixture was extracted with EtOAc, and the resulting organic solution was washed with H_2O, and brine. The organic layer was dried over $MgSO_4$, filtered, and concentrated. The residue was purified by silica gel chromatography (EtOAc/Hexanes = 1/1, R_f = 0.35) to afford compound **S40** (852 mg, 93%). ^{1}H NMR (400 MHz, $CDCl_3$) δ 7.33-7.24 (m, 10H), 7.02-6.98 (m, 2H), 6.80-6.75 (m, 2H), 5.40 (d, *J* = 3.5 Hz, 1H, H-1), 4.67 (s, 2H, C*H2*Ph), 4.54 (d, *J* = 12.0 Hz, 1H, C*H2*Ph), 4.50 (d, *J* = 12.0 Hz, 1H, C*H2*Ph), 4.18-4.15 (m, 2H, H-3, H-4), 4.11 (t, *J* = 5.3 Hz, 1H, H-5), 3.89-3.86 (m, 1H, H-2), 3.78-3.69 (m, 2H, H-6, OMe), 2.92 (br, 1H, O*H*), 2.58 (br, 1H, O*H*); ^{13}C NMR (125 MHz, $CDCl_3$) δ 155.05, 150.98, 137.73, 137.69, 128.50 (x2), 128.32 (x2), 127.99 (x3), 127.66, 127.56 (x2), 118.41 (x2), 114.46 (x2), 96.58, 76.33, 73.45, 72.68, 69.80, 69.66, 69.23, 69.05, 55.54; HRMS (ESI) *m/z* for $C_{27}H_{30}O_7Na$ [M+Na]$^+$: calcd 489.1889, found 489.1900.

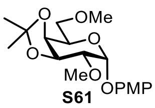

S61

Compound S61. To a solution of compound **S60** (462 mg, 1.61 mmol) in acetone (16 mL) was added camphor sulfonic acid (112 mg, 0.48 mmol) and 2,2-dimethoxypropane (297 μL, 2.42 mmol) at rt. After being stirred at rt for 1 h, the reaction mixture was quenched with Et_3N and concentrated *in vacuo*. To a solution of above residue in DMF

(16 mL) was added NaH (194 mg, 4.84 mmol) at 0 °C under a nitrogen atmosphere. The reaction mixture was stirred at 0 °C for 30 min, and then iodomethane (301 μL, 4.84 mmol) was added to the solution. The reaction mixture was gradually warmed to rt and stirred for 3 h. The reaction was quenched with MeOH and concentrated *in vacuo*. The residue was extracted with EtOAc, and the resulting organic solution was washed with saturated H_2O and brine. The organic layer was dried over $MgSO_4$, filtered, and concentrated. The residue was purified by silica gel chromatography (EtOAc/Hexanes = 1/1, R_f = 0.47) to afford compound **S61** (337 mg, 59% over two steps). ^{1}H NMR (500 MHz, $CDCl_3$) δ 7.04 (d, *J* = 8.9 Hz, 2H), 6.80 (d, *J* = 8.9 Hz, 2H), 5.46 (d, *J* = 3.3 Hz, 1H, H-1), 4.41 (dd, *J* = 7.4, 5.7 Hz, 1H, H-3), 4.28 (td, *J* = 6.2, 2.2 Hz, 1H, H-5), 4.22 (dd, *J* = 5.7, 2.2 Hz, 1H, H-4), 3.74 (s, 3H, OMe), 3.63 (d, *J* = 6.2 Hz, 2H, H-6), 3.53 (s, 3H, OMe), 3.46 (dd, *J* = 7.4, 3.3 Hz, 1H, H-2), 3.32 (s, 3H, OMe), 1.54 (s, 3H, $C(CH_3)_2$), 1.35 (s, 3H, $C(CH_3)_2$); ^{13}C NMR (125 MHz, $CDCl_3$) δ 155.04, 150.75, 118.03 (x2), 114.45 (x2), 109.23, 95.99, 79.12, 75.54, 73.50, 71.57, 66.80, 59.03, 58.58, 55.49, 28.23, 26.26, HRMS (ESI) *m/z* for $C_{18}H_{26}O_7Na$ $[M+Na]^+$: calcd 377.1576, found 377.1578.

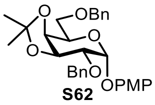

S62

Compound S62. To a solution of compound **S60** (462 mg, 1.61 mmol) in acetone (16 mL) was added camphor sulfonic acid (112 mg, 0.48 mmol) and 2,2-dimethoxypropane (297 μL, 2.42 mmol) at rt. After being stirred at rt for 1 h, the reaction mixture was quenched with Et_3N and then concentrated *in vacuo*. To a solution of above residue in DMF (16 mL) was added NaH (194 mg, 4.84 mmol) at 0 °C under a nitrogen atmosphere. The reaction mixture was stirred at 0 °C for 30 min, and then benzyl bromide (575 μL, 4.84 mmol) was added to the solution. The reaction mixture was gradually warmed to rt and stirred for 2 h. The reaction was quenched with MeOH, and

then concentrated *in vacuo*. The residue was extracted with EtOAc and the resulting organic solution was washed with H_2O and brine. The organic layer was dried over $MgSO_4$, filtered, and concentrated. The residue was purified by silica gel chromatography (EtOAc/Hexanes = 1/5, R_f = 0.41) to afford compound **S62** (536 mg, 66% over two steps). ^{1}H NMR (500 MHz, $CDCl_3$) δ 7.45 (d, *J* = 7.2 Hz, 2H), 7.31-7.26 (m, 8H), 7.07 (d, *J* = 8.8 Hz, 2H), 6.80 (d, *J* = 8.8 Hz, 2H), 5.36 (d, *J* = 3.2, 1H, H-1), 4.82 (d, *J* = 12.4 Hz, 1H, CH_2Ph), 4.76 (d, *J* = 12.4 Hz, 1H, CH_2Ph), 4.56-4.47 (m, 3H, H-3, CH_2Ph), 4.38 (t, *J* = 5.1 Hz, H-5), 4.28 (dd, *J* = 5.1, 1.7 Hz, 1H, H-4), 3.78-3.71 (m, 5H, H-6, OMe), 3,66 (dd, *J* = 7.7, 3.2 Hz, 1H, H-2), 1.42 (s, 3H, $C(CH_3)_2$), 1.37 (s, 3H, $C(CH_3)_2$); ^{13}C NMR (125 MHz, $CDCl_3$) δ 155.03, 150.83, 138.16, 137.98, 128.29 (x2), 128.18 (x2), 127.80 (x2), 127.67, 127.42, 127.36 (x2), 118.21 (x2), 114.43 (x2), 109.23, 96.53, 76.75, 76.16, 75.78, 73.57, 73.07, 72.22, 69.31, 67.06, 55.50, 28.04, 26.31. HRMS (ESI) *m/z* for $C_{30}H_{34}O_7Na$ $[M+Na]^+$: calcd 529.2202, found 529.2201.

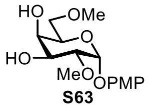

S63

Compound S63. The synthesis of compound **S63** is similar as described in the synthesis of compound **S40**, starting from compound **S61** (243 mg, 0.69 mmol) to afford compound **S63** (208 mg, 96%); EtOAc/Hexanes = 1/1 + 5% MeOH, R_f = 0.14. ^{1}H NMR (400 MHz, $CDCl_3$) δ 7.06-7.02 (m, 2H), 6.83-6.79 (m, 2H), 5.59 (d, *J* = 3.5 Hz, 1H, H-1), 4.15 (d, *J* = 3.5 Hz, 1H, H-4), 4.10 (td, *J* = 9.6, 3.5 Hz, 1H, H-3), 4.05 (t, *J* = 4.6 Hz, 1H, H-5), 3.75 (s, 3H, OMe), 3.70 (dd, *J* = 9.6, 3.5 Hz, 1H, H-2), 3.71-3.63 (m, 2H, H-6), 3.50 (s, 3H, OMe), 3.37 (s, 3H, OMe), 3.15 (br, 1H, O*H*), 2.65 (br, 1H, O*H*); ^{13}C NMR (125 MHz, $CDCl_3$) δ 155.04, 150.09, 118.23 (x2), 114.49 (x2), 95.94, 77.81, 72.32, 69.81, 69.19, 68.74, 59.29, 57.95, 55.53; HRMS (ESI) *m/z* for $C_{15}H_{22}O_7Na$ $[M+Na]^+$: calcd 337.1263, found 337.1257.

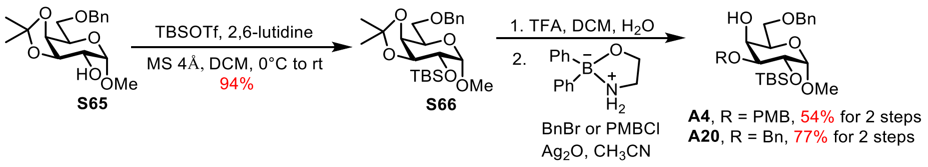

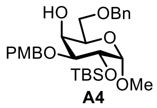

A4

Compound A4. Compound **S66** (280 mg, 0.64 mmol) was dissolved in dichloromethane (DCM, 3.0 mL). To this solution, water (0.5 mL) and trifluoroacetic acid (TFA, 1.0 mL, 13 mmol) were added in an ice bath. After the reaction was complete, the mixture was diluted with ethyl acetate and extracted with saturated aqueous sodium bicarbonate and brine. The organic layer was dried over magnesium sulfate, filtered, and concentrated in vacuo. Silver oxide (147 mg, 0.63 mmol) and borane catalyst (26 mg, 0.12 mmol) were then added and dissolved in acetonitrile (5.8 mL). Next, *p*-methoxybenzyl chloride (PMBCl, 0.12 mL, 0.86 mmol) was added. The mixture was heated to 70 °C and reacted for 4 hours. After the reaction was complete, the mixture was filtered through Celite. The filtrate was concentrated in vacuo, and the residue was purified by column chromatography to afford compound **A4** (179 mg, 54% for two steps) (EtOAc/Hexanes = 1/3, R_f = 0.60). ^{1}H NMR (400 MHz, $CDCl_3$) δ 7.24-7.32 (m, 7H, ArH), 6.85 (d, *J* = 8.6 Hz, 2H, ArH), 4.51-4.68 (m, 5H, H-1, $PhCH_2$), 4.00 (dd, *J* = 9.6, 3.8 Hz, 1H, H-2), 3.93 (d, *J* = 2.9 Hz, 1H, H-4), 3.89 (dd, *J* = 6.0, 5.3 Hz, 1H, H-5), 3.78 (s, 3H, Ph-OMe), 3.72 (dd, *J* = 10.0, 5.3 Hz, 1H, H-6), 3.64-3.68 (m, 2H, H-3, H-6), 3.40 (s, 3H, OMe), 2.52 (br, 1H, OH), 0.90 (s, 9H, Si-*t*Bu), 0.08 (s, 3H, $SiCH_3$), 0.07 (s, 3H, $SiCH_3$).; ^{13}C NMR (100 MHz, $CDCl_3$) δ 159.28, 138.09, 130.30, 129.43 (2C), 128.36 (2C), 127.65 (3C), 113.81 (2C), 100.55, 77.89, 73.61, 72.35, 69.62 (2C), 68.46, 67.89, 55.44, 55.23, 25.82 (3C), 18.20, -4.38, -4.86; HRMS (ESI) *m/z* for $C_{28}H_{42}O_7Na$ $[M+Na]^+$: calcd 541.2598, found 541.2598.

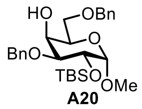

A20

Compound A20. Compound **S66** (280 mg, 0.64 mmol) was dissolved in dichloromethane (DCM, 3.0 mL). To this solution, water (0.5 mL) and trifluoroacetic acid (TFA, 1.0 mL, 13 mmol) were added in an ice bath. After the reaction was complete, the mixture was diluted with ethyl acetate and extracted with saturated aqueous sodium bicarbonate and brine. The organic layer was dried over magnesium sulfate, filtered, and concentrated in vacuo. Silver oxide (147 mg, 0.63 mmol) and borane catalyst (26 mg, 0.12 mmol) were then added and dissolved in acetonitrile (5.8 mL). Next, benzyl bromide (BnBr, 0.1 mL, 0.9 mmol) was added. The mixture was heated to 70 °C and reacted for 4 hours. After the reaction was complete, the mixture was filtered through Celite. The filtrate was concentrated in vacuo, and the residue was purified by column chromatography to afford compound **A20** (239 mg, 77% for two steps) (EtOAc/Hexanes = 1/3, R_f = 0.65). ^{1}H NMR (400 MHz, $CDCl_3$) δ 7.24-7.33 (m, 5H, ArH), 4.72 (d, *J* = 11.6 Hz, 1H, $PhCH_2$), 4.68 (d, *J* = 3.7 Hz, 1H, H-1), 4.58-4.63 (m, 2H, $PhCH_2$), 4.54 (d, *J* = 12.0 Hz, 1H, $PhCH_2$), 4.04 (dd, *J* = 9.6, 3.7 Hz, 1H, H-2), 3.98 (d, *J* = 2.6 Hz, 1H, H-4), 3.90 (dd, *J* = 6.0, 5.4 Hz, 1H, H-5), 3.73 (dd, *J* = 10.0, 5.4 Hz, 1H, H-6), 3.65-3.70 (m, 2H, H-3, H-6), 3.40 (s, 3H, OMe), 2.53 (br, 1H, OH), 0.90 (s, 9H, Si-*t*Bu), 0.08 (s, 6H, $SiCH_3$); ^{13}C NMR (100 MHz, $CDCl_3$) δ 138.14, 138.04, 128.94, 128.38 (2C), 128.34 (2C), 127.74 (2C), 127.63 (2C), 127.19, 100.54, 78.18, 73.58, 72.65, 69.62 (2C), 68.45, 67.84, 55.42, 25.79 (3C), 18.16, -4.43, -4.87.; HRMS (ESI) *m/z* for $C_{27}H_{41}O_6Si$ $[M+H]^+$: calcd 489.2672, found 489.2671.

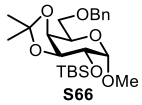

S66

Compound S66. Compound **S65** (222 mg, 0.68 mmol) and 4 Å molecular sieves were dissolved in dichloromethane (DCM, 6.8 mL). To this solution, 2,6-lutidine (0.12 mL,

1.0 mmol) and *tert*-butyldimethylsilyl trifluoromethanesulfonate (TBSOTf, 0.19 mL, 0.82 mmol) were added in an ice bath and reacted for 1 hour. After the reaction was complete, the mixture was filtered through Celite. The filtrate was concentrated in vacuo and purified by column chromatography to afford compound **S66** (282 mg, 94%) (EtOAc/Hexanes = 1/2, R_f = 0.63). ^{1}H NMR (400 MHz, $CDCl_3$) δ 7.24-7.35 (m, 5H, ArH), 4.65 (d, J = 12.1 Hz, 1H, $PhCH_2$), 4.59 (d, J = 3.6 Hz, 1H, H-1), 4.54 (d, J = 12.1 Hz, 1H, $PhCH_2$), 4.09-4.20 (m, 3H, H-3, H-4, H-5), 3.69-3.77 (m, 3H, H-2, H-6), 3.41 (s, 3H, OMe), 1.48 (s, 3H, CH_3), 1.32 (s, 3H, CH_3), 0.89 (s, 9H, Si-*t*Bu), 0.10 (s, 3H, $SiCH_3$), 0.08 (s, 3H, $SiCH_3$); ^{13}C NMR (100 MHz, $CDCl_3$) δ 138.26, 128.28 (2C), 127.51 (3C), 108.94, 100.05, 76.91, 73.72, 73.36, 71.76, 69.61, 66.67, 55.63, 28.20, 26.27, 25.76 (3C), 18.08, -4.53, -4.80; HRMS (ESI) *m/z* for $C_{23}H_{38}O_6NaSi$ $[M+Na]^+$: calcd 461.2335, found 461.2330.

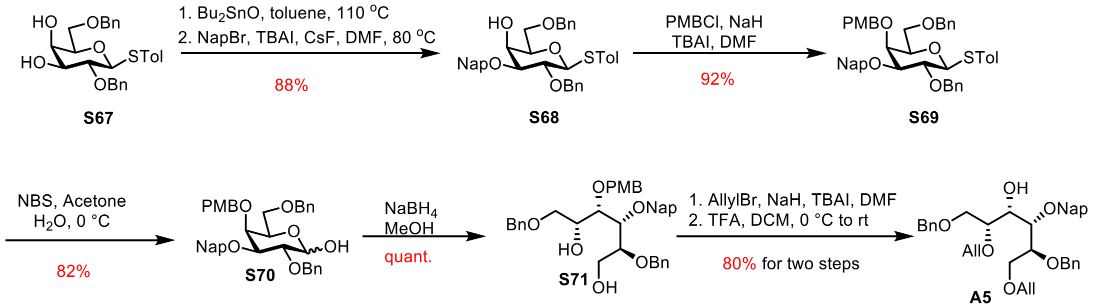

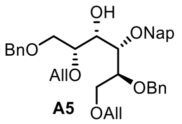

Compound A5. Compound **S71** (607 mg, 0.98 mmol) was dissolved in dimethylformamide (DMF, 9.7 mL). To this solution, sodium hydride (NaH, 156 mg, 3.90 mmol) and allyl bromide (340 μL, 3.90 mmol) were added in an ice bath, and the mixture was allowed to warm to room temperature and stirred for 3 hours. After the reaction was complete, methanol was added to quench the reaction. The mixture was extracted with ethyl acetate and brine. The organic layer was dried over magnesium sulfate, filtered, and concentrated in vacuo. The residue was then dissolved in

dichloromethane (DCM, 8.4 mL). To this solution, water (0.5 mL) and trifluoroacetic acid (TFA, 0.5 mL, 7 mmol) were added in an ice bath. After the reaction was complete, the mixture was diluted with ethyl acetate and extracted with saturated aqueous sodium bicarbonate and brine. The organic layer was dried over magnesium sulfate and filtered. The residue was purified by column chromatography to afford compound **A5** (454 mg, 80% for two steps) (EtOAc/Hexanes = 1/4, R_f = 0.36). ^{1}H NMR (400 MHz, $CDCl_3$) δ 7.81-7.87 (m, 4H, ArH), 7.31-7.52 (m, 13H, ArH), 5.88-6.00 (m, 2H), 5.16-5.32 (m, 4H), 4.75-4.86 (m, 4H, $PhCH_2$), 4.58 (s, 2H, $PhCH_2$), 4.28 (dd, J = 12.7, 5.3 Hz, 1H), 4.10-4.14 (m, 1H), 3.89-4.04 (m, 6H), 3.76-3.80 (m, 4H), 3.11 (br, 1H, OH); ^{13}C NMR (100 MHz, $CDCl_3$) δ 138.29, 137.98, 135.62, 134.97, 134.45, 133.08, 132.78, 128.19 (2C), 128.14 (2C), 127.96 (2C), 127.91, 127.73, 127.49 (2C), 127.42 (3C), 126.32, 125.92, 125.75, 125.71, 116.68, 116.30, 77.60, 77.43, 76.33, 73.43, 73.28, 73.22, 71.95, 71.30, 70.76, 69.77; HRMS (ESI) *m/z* for $C_{37}H_{42}O_6Na$ $[M+Na]^+$: calcd 605.2879, found 605.2884.

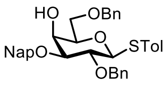

S68

Compound S68. Compound **S67** (720 mg, 1.54 mmol) was dissolved in toluene (15.0 mL). To this solution, dibutyltin oxide (461 mg, 1.85 mmol) was added. The mixture was refluxed at 110 °C for 3 hours using a Dean-Stark apparatus. After cooling to room temperature, the Dean-Stark apparatus was removed. 2-Bromomethylnaphthalene (683 mg, 2.00 mmol), tetrabutylammonium iodide (TBAI, 1.14 g, 3.08 mmol), and cesium fluoride (CsF, 469 mg, 3.08 mmol) were then added. The reaction mixture was heated to 80 °C and reacted for 12 hours. After the reaction was complete, the solvent was removed. The residue was extracted with ethyl acetate and saturated brine. The organic layer was dried over magnesium sulfate, filtered, and concentrated in vacuo. The residue was purified by column chromatography to afford compound **S68** (824 mg,

88% for two steps) (EtOAc/Hexanes = 1/2, R_f = 0.53). ^{1}H NMR (400 MHz, $CDCl_3$) δ 7.73-7.84 (m, 4H, ArH), 7.43-7.51 (m, 7H, ArH), 7.30-7.37 (m, 8H, ArH), 7.06 (d, *J* = 7.9 Hz, 2H, ArH), 4.78-4.90 (m, 4H, $PhCH_2$), 4.60 (d, *J* = 9.8 Hz, 1H, H-1), 4.56 (s, 2H, $PhCH_2$), 4.14 (dd, *J* = 2.8, 2.0 Hz, 1H, H-4), 3.74-3.84 (m, 3H, H-2, H-6), 3.62 (dd, *J* = 9.0, 2.8 Hz, 1H, H-3), 3.58 (dd, *J* = 5.7, 5.7 Hz, 1H, H-5), 2.61 (d, *J* = 2.0 Hz, 1H, OH), 2.31 (s, 3H, CH_3); ^{13}C NMR (100 MHz, $CDCl_3$) δ 138.25, 137.92, 137.54, 135.08, 133.17, 133.02, 132.51 (2C), 129.86, 129.59 (2C), 128.36 (2C), 128.33 (3C), 128.21 (2C), 127.86, 127.74 (2C), 127.69 (2C), 127.66 (2C), 126.68, 126.16, 126.01, 125.73, 87.97, 82.46, 77.00, 75.68, 73.67, 72.10, 69.47, 66.98, 21.05; HRMS (ESI) *m/z* for $C_{38}H_{38}O_5NaS$ $[M+Na]^+$: calcd 629.2338, found 629.2336.

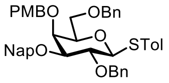

S69

Compound S69. Compound **S68** (800 mg, 1.32 mmol) was dissolved in dimethylformamide (DMF, 13.0 mL). To this solution, sodium hydride (NaH, 106 mg, 2.64 mmol) and *p*-methoxybenzyl chloride (PMBCl, 0.23 mL, 1.7 mmol) were added in an ice bath, and the mixture was allowed to warm to room temperature and stirred for 3 hours. After the reaction was complete, methanol was added to quench the reaction. The mixture was extracted with ethyl acetate and saturated brine. The organic layer was dried over magnesium sulfate, filtered, and concentrated in vacuo. The residue was purified by column chromatography to afford compound **S69** (882 mg, 92%) (EtOAc/Hexanes = 1/4, R_f = 0.21). ^{1}H NMR (400 MHz, $CDCl_3$) δ 7.73-7.86 (m, 4H, ArH), 7.24-7.51 (m, 17H, ArH), 7.02 (d, *J* = 6.9 Hz, 2H, ArH), 6.86 (d, *J* = 6.9 Hz, 2H, ArH), 4.80-4.95 (m, 5H, $PhCH_2$), 4.61-4.65 (m, 2H, H-1, $PhCH_2$), 4.50 (d, *J* = 11.7 Hz, 1H, $PhCH_2$), 4.44 (d, *J* = 11.7 Hz, 1H $PhCH_2$), 4.01 (bs, 1H, H-4), 3.97 (dd, *J* = 9.5, 8.4 Hz, 1H, H-2), 3.58-3.70 (m, 4H, H-3, H-5, H-6), 3.80 (s, 3H, Ph-OMe), 2.31 (s, 3H, CH_3); ^{13}C NMR (100 MHz, $CDCl_3$) δ 159.04, 138.36, 137.87, 137.07, 135.72, 133.20,

132.04 (2C), 130.84, 130.33, 129.49 (4C), 128.34 (2C), 128.27 (4C), 128.09, 127.84 (4C), 127.69, 127.63 (2C), 126.16, 126.06, 125.83, 125.57, 113.51 (2C), 88.14, 84.08, 77.44, 75.58, 73.94, 73.49, 73.02, 72.67, 68.81, 55.17, 21.03; HRMS (ESI) *m/z* for $C_{46}H_{46}O_6NaS$ $[M+Na]^+$: calcd 749.2913, found 749.2912.

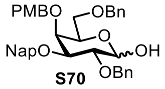

Compound S70. Compound **S69** (874 mg, 1.20 mmol) was dissolved in acetone (11.0 mL). Water (0.6 mL) was added, followed by portionwise addition of N-bromosuccinimide (NBS, 258 mg, 1.44 mmol). The reaction was monitored by TLC, and upon completion, it was diluted with ethyl acetate (EtOAc) and extracted with saturated aqueous sodium $Na_2S_2O_3$, sodium bicarbonate, and brine. The organic layer was dried over magnesium sulfate, filtered, and concentrated in vacuo. The residue was purified by column chromatography to afford compound **S70** (612 mg, 82%) (EtOAc/Hexanes = 1/4, R_f = 0.05). ^{1}H NMR (400 MHz, $CDCl_3$) δ 7.75-7.85 (m, 4H, ArH), 7.19-7.50 (m, 15H, ArH), 6.79 (d, J = 8.5 Hz, 2H, ArH), 4.37-5.30 (m, 9H, H-1, $PhCH_2$), 3.88-4.17 (m, 3H), 3.75 (s, 3H, Ph-OMe), 3.43-3.61 (m, 3H), 3.27 (br, 1H, OH); ^{13}C NMR (100 MHz, $CDCl_3$) δ 159.10, 138.61, 138.20, 137.73, 137.65, 136.08, 135.82, 133.21, 132.83, 130.57, 129.84 (4C), 128.28, 128.21, 128.07, 128.00, 127.90, 127.85, 127.63, 127.47, 126.10, 126.01, 125.75, 125.58, 113.55 (2C), 97.73, 91.68, 81.99, 80.69, 78.61, 76.56, 74.98, 74.20, 74.10, 73.98, 73.39, 73.33, 73.23, 72.92, 72.85, 69.33, 69.09, 68.87, 55.12; HRMS (ESI) *m/z* for $C_{39}H_{40}O_7Na$ $[M+Na]^+$: calcd 643.2672, found 643.2664.

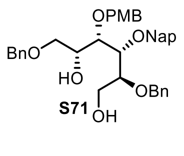

Compound S71. Compound **S70** (604 mg, 0.97 mmol) was dissolved in methanol (MeOH, 9.7 mL). Sodium borohydride ($NaBH_4$, 296 mg, 7.79 mmol) was added

portionwise. The reaction was monitored by TLC, and upon completion, acetone was added in an ice bath to quench the reaction. The mixture was concentrated in vacuo and purified by column chromatography to afford compound **S71** (606 mg, quant.) (EtOAc/Hexanes = 1/2, R_f = 0.20). ^{1}H NMR (400 MHz, $CDCl_3$) δ 7.75-7.83 (m, 4H, ArH), 7.45-7.49 (m, 3H, ArH), 7.24-7.34 (m, 10H, ArH), 7.16 (d, *J* = 8.1 Hz, 2H, ArH), 6.18 (d, *J* = 8.1 Hz, 2H, ArH), 4.90 (d, *J* = 11.5 Hz, 1H, $PhCH_2$), 4.84 (d, *J* = 11.5 Hz, 1H, $PhCH_2$), 4.68 (d, *J* = 11.2 Hz, 1H, $PhCH_2$), 4.58-4.63 (m, 2H, $PhCH_2$), 4.39-4.47 (m, 3H, $PhCH_2$), 4.04-4.07 (m, 1H), 3.94 (dd, *J* = 4.7, 4.6 Hz, 1H), 3.89-3.90 (m, 1H), 3.71-3.82 (m, 6H), 3.47-3.57 (m, 2H); ^{13}C NMR (100 MHz, $CDCl_3$) δ 159.33, 137.98 (2C), 135.38, 133.18, 132.99, 129.88, 129.79 (2C), 128.43 (2C), 128.33 (2C), 128.19, 127.90, 127.84 (2C), 127.78 (3C), 127.65 (2C), 126.81, 126.09, 126.00, 125.95, 113.76 (2C), 80.31, 79.21, 77.00, 74.40, 73.37, 73.25, 72.36, 70.88, 69.88, 60.97, 55.17; HRMS (ESI) *m/z* for $C_{39}H_{42}O_7Na$ [M+Na]$^+$: calcd 645.2828, found 645.2817.

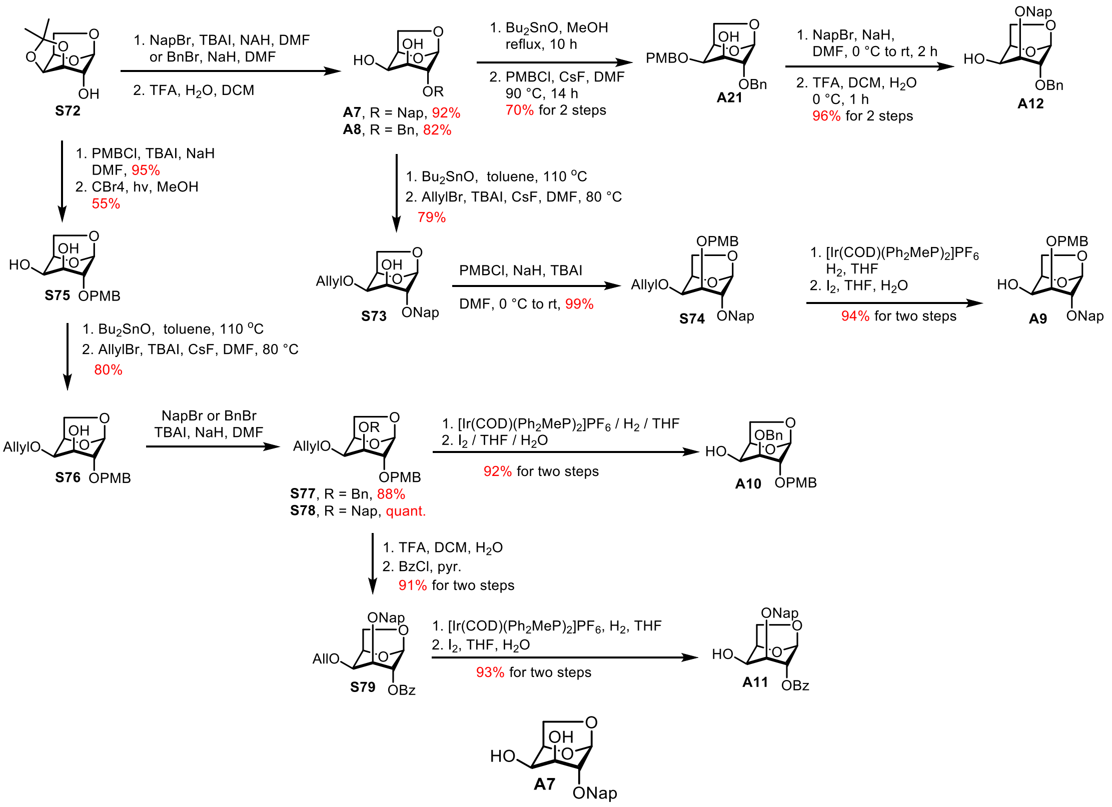

Compound A7. Compound **S72** (630 mg, 3.10 mmol) was dissolved in dimethylformamide (DMF, 16.0 mL). To this solution, sodium hydride (NaH, 250 mg, 6.20 mmol) and 2-bromomethylnaphthalene (827 mg, 4.70 mmol) were added in an ice bath, and the mixture was allowed to warm to room temperature and stirred for 3 hours. After the reaction was complete, methanol was added to quench the reaction. The mixture was extracted with ethyl acetate and saturated brine. The organic layer was dried over magnesium sulfate, filtered, and concentrated in vacuo. The residue was purified by column chromatography (1.0 g, 97%) (EtOAc/Hexanes = 1/2, R_f = 0.60). Next, trifluoroacetic acid (TFA, 0.5 mL, 6 mmol) and water (0.1 mL) were added to a solution of the above residue (33 mg, 0.10 mmol) in DCM (2.0 mL) at ice bath. After the reaction was complete, the mixture was diluted with ethyl acetate and extracted with saturated aqueous sodium bicarbonate and brine. The organic layer was dried over magnesium sulfate, filtered, and concentrated in vacuo to afford product **A7** (28 mg, 95%) (EtOAc/Hexanes = 1/2, R_f = 0.05). ^{1}H NMR (400 MHz, $CDCl_3$) δ 7.75-7.84 (m, 4H, ArH), 7.44-7.48 (m, 3H, ArH), 5.41 (s, 1H, H-1), 4.82 (d, J = 12.2 Hz, 1H, $PhCH_2$), 4.72 (d, J = 12.2 Hz, 1H, $PhCH_2$), 4.43 (dd, J = 4.5, 4.4 Hz, 1H, H-5), 4.14 (d, J = 7.8 Hz, 1H, H-6), 3.99-4.05 (m, 2H, H-3, H-4), 3.57-3.61 (m, 2H, H-2, H-6), 2.96 (d, J = 8.2 Hz, 1H, OH), 2.56 (d, J = 7.4 Hz, 1H, OH); ^{13}C NMR (100 MHz, $CDCl_3$) δ 134.64, 132.88, 132.79, 127.96, 127.47, 127.26, 126.45, 125.83, 125.69, 125.31, 99.77, 78.09, 74.09, 72.07, 67.71, 64.20, 63.06; HRMS (ESI) m/z for $C_{17}H_{18}O_5Na$ $[M+Na]^+$: calcd 325.1052, found 325.1045.

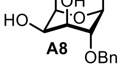

A8

Compound A8. To a solution of compound **S72** (621 mg, 3.07 mmol) in dry DMF (30 mL) was added NaH (246 mg, 6.14 mmol) at 0°C under a nitrogen atmosphere. The reaction mixture stirred at 0°C for 30 min and then benzyl bromide (729 µL, 6.14 mmol)

was added to the solution. The reaction mixture was gradually warmed to rt and stirred for 2 h. The reaction was quenched by MeOH and then concentrated *in vacuo*. The residue was extracted with EtOAc, and the resulting organic solution was washed with saturated H_2O and brine. The organic layer was dried over $MgSO_4$, filtered, and concentrated. Next, to a solution of above residue in DCM (30 mL) was added TFA (1.9 mL) and H_2O (1 mL) at 0°C. The reaction mixture was stirred at 0°C for 3 h and then neutralized with saturated $NaHCO_{3(aq)}$. The residue was extracted with EtOAc, and the resulting organic solution was washed with H_2O and brine. The organic layer was dried over $MgSO_4$, filtered, and concentrated. The residue was purified by silica gel chromatography (EtOAc/Hexanes = 1/1, R_f = 0.11) to afford compound **A8** (633 mg, 82%).^{1}H NMR (400 MHz, $CDCl_3$) δ 7.36-7.24 (m, 5H), 5.40 (t, *J* = 1.5 Hz, 1H, H-1), 4.68 (d, *J* = 12.2 Hz, 1H, C*H*$_2$Ph), 4.58 (d, *J* = 12.2 Hz, 1H, C*H*$_2$Ph), 4.45-4.43 (m, 1H, H-5), 4.15 (d, *J* = 7.9 Hz, 1H, H-6), 4.05-3.95 (m , 2H, H-3 and H-4), 3.60 (ddd, *J* = 7.9, 4.4, 1.5 Hz, 1H, H-6), 3.57 (t, *J* = 1.5 Hz, 1H, H-2), 2.90 (d, *J* = 8.3 Hz, 1H, OH), 2.48 (d, *J* = 7.9 Hz, 1H, OH); ^{13}C NMR (100 MHz, $CDCl_3$) δ 137.22, 128.53 (x2), 128.10 127.83 (x2), 100.15, 77.58, 74.54, 72.25, 68.40, 64.48, 63.44; HRMS (ESI) *m/z* for $C_{13}H_{16}O_5Na$ $[M+Na]^+$: calcd 275.0895, found 275.0895.

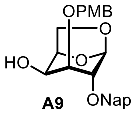

A9

Compound A9. The synthesis of compound **A9** is similar as described in the synthesis of compound **S90**, starting from compound **S74** to yield compound **A9** (77 mg, 94% for two steps) (EtOAc/Hexanes = 1/3, R_f = 0.18). ^{1}H NMR (400 MHz, $CDCl_3$) δ 7.80-7.85 (m, 3H, ArH), 7.72 (br, 1H, ArH), 7.44-7.51 (m, 3H, ArH), 7.10 (d, *J* = 8.6 Hz, 2H, ArH), 6.83 (d, *J* = 8.6 Hz, 2H, ArH), 5.40 (s, 1H, H-1), 4.71 (d, *J* = 12.4 Hz, 1H, $PhCH_2$), 4.67 (d, *J* = 12.4 Hz, 1H, $PhCH_2$), 4.46 (d, *J* = 11.4 Hz, 1H, $PhCH_2$), 4.38 (dd, *J* = 4.7, 4.7 Hz, 1H, H-5), 4.21 (d, *J* = 11.4 Hz, 1H, $PhCH_2$), 4.13 (d, *J* = 7.4 Hz, 1H,

H-6), 4.08 (ddd, J = 9.8, 5.8, 4.7 Hz, 1H, H-4), 3.77 (s, 3H, OMe), 3.74 (dd, J = 5.8, 1.0 Hz, 1H, H-3), 3.57-3.60 (m, 2H, H-2, H-6), 2.99 (d, J = 9.8 Hz, 1H, OH).; ^{13}C NMR (100 MHz, $CDCl_3$) δ 159.46, 134.81, 133.09, 129.29 (3C), 129.04, 128.48, 127.79, 127.68, 126.81, 126.31, 126.15, 125.63, 113.92 (2C), 99.86, 75.51, 74.58, 74.38, 72.38, 72.28, 64.57, 63.27, 55.19; HRMS (ESI) *m/z* for $C_{25}H_{26}O_6Na$ [M+Na]$^+$: calcd 445.1627, found 445.1634.

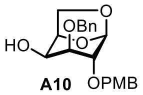

A10

Compound A10. The synthesis of compound **A10** is similar as described in the synthesis of compound **S90**, starting from compound **S77** to yield compound **A10** (153 mg, 92% for two steps) (EtOAc/Hexanes = 1/3, R_f = 0.11). ^{1}H NMR (400 MHz, $CDCl_3$) δ 7.21-7.33 (m, 7H, ArH), 6.86 (d, J = 8.7 Hz, 2H, ArH), 5.34 (s, 1H, H-1), 4.54 (d, J = 11.6 Hz, 1H, $PhCH_2$), 4.49 (d, J = 12.0 Hz, 1H, $PhCH_2$), 4.45 (d, J = 12.0 Hz, 1H, $PhCH_2$), 4.37 (dd, J = 4.7, 4.7 Hz, 1H, H-5), 4.29 (d, J = 11.6 Hz, 1H, $PhCH_2$), 4.13 (d, J = 7.4 Hz, 1H, H-6), 4.05 (ddd, J = 9.9, 5.7, 4.7 Hz, 1H, H-4), 3.79 (s, 3H, Ph-OMe), 3.70 (dd, J = 5.7, 1.2 Hz, 1H, H-3), 3.59 (dd, J = 7.4, 4.7 Hz, 1H, H-6), 3.54 (s, 1H, H-2), 2.99 (d, J = 9.9 Hz, 1H, OH); ^{13}C NMR (100 MHz, $CDCl_3$) δ 159.56, 137.13, 129.60 (2C), 129.39, 128.59 (2C), 128.12, 127.69 (2C), 113.96 (2C), 99.91, 75.98, 74.36, 73.99, 72.58, 71.91, 64.66, 63.29, 55.26; HRMS (ESI) *m/z* for $C_{21}H_{24}O6Na$ [M+Na]$^+$: calcd 395.1471, found 395.1473.

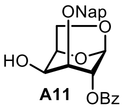

A11

Compound A11. The synthesis of compound **A11** is similar as described in the synthesis of compound **S90**, starting from compound **S79** to yield compound **A11** (500 mg, 93% for two steps) (EtOAc/Hexanes = 1/2, R_f = 0.40). ^{1}H NMR (400 MHz, $CDCl_3$) δ 8.06-8.08 (m, 2H, ArH), 7.81-7.86 (m, 4H, ArH), 7.44-7.61 (m, 6H, ArH), 5.55 (dd,

J = 1.4, 1.4 Hz, 1H, H-2), 5.27 (s, 1H, H-1), 5.17 (d, *J* = 11.7 Hz, 1H, $PhCH_2$), 4.75 (d, *J* = 11.7 Hz, 1H, $PhCH_2$), 4.47 (dd, *J* = 4.7, 4.7 Hz, 1H, H-5), 4.29 (d, *J* = 7.8 Hz, 1H, H-6), 4.07 (m, 1H, H-4), 3.89 (dd, *J* = 5.9, 1.6 Hz, 1H, H-3), 3.71 (dd, *J* = 6.2, 5.7 Hz, 1H, H-6), 3.10 (d, *J* = 9.5 Hz, 1H, OH); ^{13}C NMR (100 MHz, $CDCl_3$) δ 134.37, 133.54, 133.11, 133.06, 129.81 (2C), 129.18, 128.48 (3C), 127.92, 127.67, 127.08, 126.23, 126.14, 125.64, 99.24, 75.16, 74.42, 72.66, 69.41, 64.68, 63.57; HRMS (ESI) *m/z* for $C_{24}H_{22}O_6Na$ $[M+Na]^+$: calcd 429.1314, found 429.1306.

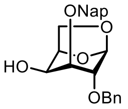

A12

Compound A12. To a solution of compound **A21** (2.20 g, 5.91 mmol) in DMF (60 mL) was added NaH (473 mg, 11.81 mmol) at 0 °C under a nitrogen atmosphere. The reaction mixture was stirred at 0 °C for 30 min and then 2-(bromomethyl)naphthalene (2.61 g, 11.81 mmol) was added to the solution. The reaction mixture was gradually warmed to rt and stirred for 2 h. The reaction was quenched by MeOH and then concentrated *in vacuo*. The residue was extracted with EtOAc, and the resulting organic solution was washed with saturated H_2O and brine. The organic layer was dried over $MgSO_4$, filtered, and concentrated. To a solution of above residue in DCM (30 mL) was added TFA (3 mL) and H_2O (1.5 mL) at 0 °C. The reaction mixture was stirred at 0 °C for 1 h and then neutralized with saturated $NaHCO_{3(aq)}$. The mixture was extracted with EtOAc, and the resulting organic solution was washed with H_2O and brine. The organic layer was dried over $MgSO_4$, filtered, and concentrated. The residue was purified by silica gel chromatography (EtOAc/Hexanes = 1/2, R_f = 0.28) to afford compound **A12** (2.23 g, 96%). ^{1}H NMR (400 MHz, $CDCl_3$) δ 7.86-7.79 (m, 3H), 7.68 (br, 1H), 7.51-7.46 (m, 2H), 7.39 (dd, *J* = 8.4, 1.7 Hz, 1H), 7.34-7.26 (m, 4H), 7.25-7.22 (m, 2H), 5.38 (t, *J* = 1.4 Hz, 1H, H-1), 4.67 (d, *J* = 11.6 Hz, 1H, C*H₂*Ph), 4.53 (d, *J* = 12.3 Hz, 1H, C*H₂*Ph), 4.48 (d, *J* = 12.3 Hz, 1H, C*H₂*Ph), 4.46 (d, *J* = 11.6 Hz, 1H,

CH_2Ph), 4.39 (t, J = 4.9 Hz, 1H, H-5), 4.17 (d, J = 7.4 Hz, 1H, H-6), 4.09-4.05 (m, 1H, H-4), 3.78 (dq, J = 5.7, 1.4 Hz, 1H, H-3), 3.61 (ddd, J = 7.4, 4.9, 1.4 Hz, 1H, H-6), 3.57 (t, J = 1.4 Hz, 1H, H-2), 3.02 (d, J = 9.5 Hz, OH); ^{13}C NMR (125 MHz, $CDCl_3$) δ 137.27, 134.40, 133.03, 128.50 (x3), 128.06, 127.86 (x2), 127.80, 127.68, 126.73, 126.34, 126.21, 125.52, 99.82, 75.88, 74.43, 74.34, 72.84, 72.61, 64.63, 63.28; HRMS (ESI) *m/z* for $C_{24}H_{24}O_5Na$ [M+Na]$^+$: calcd 415.1521, found 415.1515.

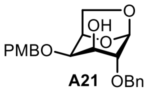

A21

Compound A21. A solution of compound **A8** (100 mg, 0.40 mmol) and dibutyltin oxide (148 mg, 0.60 mmol) in MeOH (10 mL) was refluxed for 10 h under a nitrogen atmosphere and then concentrated *in vacuo*. Next, to a solution of the above residue and CsF (66 mg, 0.44 mmol) in DMF (4 mL) was added 4-methoxybenzyl chloride (80 μL, 0.60 mmol). After being stirred at 90 °C under a nitrogen atmosphere for 14 h, the solution was concentrated under reduced pressure. The residue was extracted with EtOAc, and the resulting organic solution was washed with H_2O and brine. The organic layer was dried over $MgSO_4$, filtered, and concentrated. The residue was purified by silica gel chromatography (EtOAc/Hexanes = 1/1, R_f = 0.36) to get compound **A21** (104 mg, 70% over two steps). ^{1}H NMR (400 MHz, $CDCl_3$) δ 7.34-7.24 (m, 8H), 6.90-6.86 (m, 2H), 5.35 (t, J = 1.4 Hz, 1H, H-1), 4.64 (d, J = 12.1 Hz, 1H, CH_2Ph), 4.60 (d, J = 11.5 Hz, 1H, CH_2Ph), 4.55 (d, J = 11.5 Hz, 1H, CH_2Ph), 4.54 (d, J = 12.1 Hz, 1H, CH_2Ph), 4.36 (t, J = 4.6 Hz, 1H, H-5), 4.22 (d, J = 7.2 Hz, 1H, H-6), 4.04 (dt, J = 4.6, 1.4 Hz, 1H, H-3), 3.84 (t, J = 4.6 Hz, 1H, H-4), 3.79 (s, 3H, OMe), 3.57 (dd, J = 7.2, 4.6 Hz, 1H, H-6), 3.54 (t, J = 1.4 Hz, 1H, H-2), 2.71 (d, J = 1.4 Hz, 1H, OH); ^{13}C NMR (100 MHz, $CDCl_3$) δ 159.62, 137.52, 129.53 (x2), 129.30, 128.44 (x2), 127.91, 127.79 (x2), 114.02 (x2), 100.34, 77.77, 72.28, 72.16, 71.76, 71.53, 67.24, 63.91, 55.23; HRMS (ESI) *m/z* for $C_{21}H_{24}O_6Na$ [M+Na]$^+$: calcd 395.1471, found 395.1480..

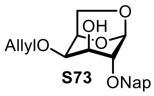

S73

Compound S73. Compound **A7** (168 mg, 0.56 mmol) was dissolved in toluene (11.0 mL). To this solution, dibutyltin oxide (166 mg, 0.67 mmol) was added. The mixture was refluxed at 110 °C for 3 hours using a Dean-Stark apparatus. After cooling to room temperature, the Dean-Stark apparatus was removed. Allyl bromide (0.1 mL, 1 mmol), tetrabutylammonium iodide (TBAI, 411 mg, 1.11 mmol), and cesium fluoride (CsF, 169 mg, 1.11 mmol) were then added. The reaction mixture was heated to 80 °C and reacted for 12 hours. After the reaction was complete, the solvent was removed. The residue was extracted with ethyl acetate and saturated brine. The organic layer was dried over magnesium sulfate, filtered, and concentrated in vacuo. The residue was purified by column chromatography to afford compounds **S73** (150 mg, 79%) (EtOAc/Hexanes = 1/2, R_f = 0.33). ^{1}H NMR (400 MHz, $CDCl_3$) δ 7.76-7.82 (m, 4H, ArH), 7.44-7.47 (m, 3H, ArH), 5.89 (ddd, *J* = 17.2, 10.3, 5.9 Hz, 1H, CH=C), 5.41 (s, 1H, H-1), 5.29 (dd, *J* = 17.2, 1.4 Hz, 1H, C=CH_2), 5.22 (dd, *J* = 10.3, 1.4 Hz, 1H, C=CH_2), 4.81 (d, *J* = 12.2 Hz, 1H, PhCH_2), 4.71 (d, *J* = 12.2 Hz, 1H, PhCH_2), 4.45 (dd, *J* = 4.7, 4.6 Hz, 1H, H-5), 4.24 (d, *J* = 7.4 Hz, 1H, H-6), 4.05-4.13 (m, 3H, H-3, OCH_2), 3.82 (dd, *J* = 4.6, 4.5 Hz, 1H, H-4), 3.59-3.63 (m, 2H, H-2, H-6), 2.78 (d, *J* = 1.5 Hz, 1H, OH); ^{13}C NMR (100 MHz, $CDCl_3$) δ 134.89, 133.76, 133.04, 132.93, 128.21, 127.74, 127.52, 126.61, 126.05, 125.89, 125.51, 118.09, 100.25, 77.83, 72.35, 72.01, 71.83, 70.54, 67.27, 63.81; HRMS (ESI) *m/z* for $C_{20}H_{22}O_5Na$ $[M+Na]^+$: calcd 365.1365, found 365.1357.

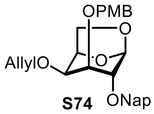

S74

Compound S74. Compound **S73** (77 mg, 0.23 mmol) was dissolved in dimethylformamide (DMF, 2.3 mL). To this solution, sodium hydride (NaH, 18 mg,

0.46 mmol) and *p*-methoxybenzyl chloride (PMBCl, 50 μL, 0.35 mmol) were added in an ice bath. The reaction mixture was allowed to warm naturally to room temperature and stirred for 3 hours. After the reaction was complete, methanol was added to quench the reaction, and the mixture was extracted with ethyl acetate and saturated brine. The organic layer was dried over magnesium sulfate, filtered, and concentrated in vacuo. The crude product was purified by column chromatography to afford compound **S74** (103 mg, 99%) (EtOAc/Hexanes = 1/3, R_f = 0.38). ^{1}H NMR (400 MHz, $CDCl_3$) δ 7.80-7.84 (m, 3H, ArH), 7.66 (s, 1H, ArH), 7.45-7.51 (m, 2H, ArH), 7.40 (dd, *J* = 8.4, 1.5 Hz, 1H, ArH), 7.19 (d, *J* = 8.6 Hz, 2H, ArH), 6.83 (d, *J* = 8.6 Hz, 2H, ArH), 5.89 (ddd, *J* = 17.2, 10.4, 5.6 Hz, 1H, CH=C), 5.37 (s, 1H, H-1), 5.27 (dd, *J* = 17.2, 1.4 Hz, 1H, C=CH_2), 5.19 (dd, *J* = 10.4, 1.4 Hz, 1H, C=CH_2), 4.63 (d, *J* = 12.4 Hz, 1H, PhCH_2), 4.55 (d, *J* = 12.4 Hz, 1H, PhCH_2), 4.43-4.51 (m, 4H, H-5, H-6, PhCH_2), 3.96-4.06 (m, 2H, OCH_2), 3.78-3.84 (m, 5H, OMe, H-3, H-4), 3.62 (dd, *J* = 6.0, 6.0 Hz, 1H, H-6), 3.54 (s, 1H, H-2); ^{13}C NMR (100 MHz, $CDCl_3$) δ 159.08, 135.00, 134.37, 133.06, 132.99, 130.25, 129.20 (2C), 128.29, 127.75, 127.61, 126.66, 126.18, 126.00, 125.55, 117.26, 113.60 (2C), 100.14, 76.59, 73.76, 73.03, 72.65, 72.58, 72.19, 70.08, 64.19, 55.14; HRMS (ESI) *m/z* for $C_{28}H_{30}O_6Na$ [M+Na]$^+$: calcd 485.1940, found 485.1944.

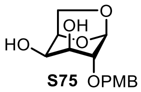

S75

Compound S75. The synthesis of PMB protection is similar as described in the synthesis of compound **S74**, starting from compound **S72** (500 mg, 93%) (EtOAc/Hexanes = 1/2, R_f = 0.60). Next, the above residue (345 mg, 1.07 mmol) was dissolved in methanol (MeOH, 11.0 mL). Carbon tetrabromide (CBr_4, 71 mg, 0.21 mmol) was added, and the mixture was irradiated with a 365 nm UV lamp for 30 minutes. The reaction was then stirred at room temperature for 16 hours. Triethylamine (Et_3N) was added to quench the reaction. The mixture was concentrated in vacuo, and

the residue was purified by column chromatography to afford compound **S75** (165 mg, 55%, quant) (EtOAc/Hexanes = 1/1, R_f = 0.05). ^{1}H NMR (400 MHz, $CDCl_3$) δ 7.25 (d, *J* = 8.6 Hz, 2H, ArH), 6.86 (d, *J* = 8.6 Hz, 2H, ArH), 5.36 (s, 1H, H-1), 4.61 (d, *J* = 11.6 Hz, 1H, $PhCH_2$), 4.50 (d, *J* = 11.6 Hz, 1H, $PhCH_2$), 4.42 (dd, *J* = 4.5, 4.4 Hz, 1H, H-5), 4.14 (d, *J* = 8.0 Hz, 1H, H-6), 3.97-4.02 (m, 1H, H-3), 3.93 (dd, *J* = 5.8, 5.4 Hz, 1H, H-4), 3.79 (s, 3H, Ph-OMe), 3.59 (dd, *J* = 8.0, 4.5 Hz, 1H, H-6), 3.55 (s, 1H, H-2), 2.92 (d, *J* = 7.9 Hz, 1H, OH), 2.50 (d, *J* = 7.4 Hz, 1H, OH); ^{13}C NMR (100 MHz, $CDCl_3$) δ 159.48, 129.53 (2C), 129.30, 113.92 (2C), 100.24, 77.19, 74.55, 71.92, 68.47, 64.48, 63.42, 55.23; HRMS (ESI) *m/z* for $C_{14}H_{18}O_6Na$ [M+Na]$^+$: calcd 305.1001, found 305.0995.

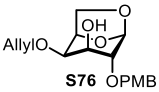

S76

Compound S76. The synthesis of compound **S76** is similar as described in the synthesis of compound **S73**, starting from compound **S75** to yield compound **S76** (208 mg, 80%) (EtOAc/Hexanes = 1/1, R_f = 0.38). ^{1}H NMR (400 MHz, $CDCl_3$) δ 7.24 (d, *J* = 8.6 Hz, 2H, ArH), 6.84 (d, *J* = 8.6 Hz, 2H, ArH), 5.88 (ddd, *J* = 17.2, 10.3, 5.7 Hz, 1H, CH=C), 5.26-5.32 (m, 2H, H-1, $C=CH_2$), 5.22 (dd, *J* = 10.3, 1.0 Hz, 1H, $C=CH_2$), 4.59 (d, *J* = 11.8 Hz, 1H, $PhCH_2$), 4.48 (d, *J* = 11.8 Hz, 1H, $PhCH_2$), 4.42 (dd, *J* = 4.6, 4.5 Hz, 1H, H-5), 4.20 (d, *J* = 7.4 Hz, 1H, H-6), 4.08-4.15 (m, 2H, OCH_2), 4.05 (dd, *J* = 5.0, 1.3 Hz, 1H, H-3), 3.73-3.79 (m, 4H, H-3, Ph-OMe), 3.59 (dd, *J* = 7.0, 5.4 Hz, 1H, H-6), 3.53 (s, 1H, H-2), 2.69 (br, 1H, OH); ^{13}C NMR (100 MHz, $CDCl_3$) δ 159.39, 133.83, 129.57, 129.48 (2C), 118.24, 113.85 (2C), 100.39, 77.32, 72.08, 71.96, 71.89, 70.66, 67.37, 63.89, 55.21; HRMS (ESI) *m/z* for $C_{17}H_{22}O_6Na$ [M+Na]$^+$: calcd 345.1314, found 345.1316.

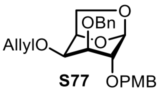

S77

Compound S77. Compound **S76** (170 mg, 0.53 mmol) was dissolved in dimethylformamide (DMF, 5.0 mL). To this solution, sodium hydride (NaH, 32 mg, 0.79 mmol) and benzyl bromide (BnBr, 0.13 mL, 1.1 mmol) were added in an ice bath. The reaction mixture was allowed to warm naturally to room temperature and stirred for 3 hours. After the reaction was complete, methanol was added to quench the reaction, and the mixture was extracted with ethyl acetate and saturated brine. The organic layer was dried over magnesium sulfate, filtered, and concentrated in vacuo. The crude product was purified by column chromatography to afford compound **S77** (191 mg, 88%) (EtOAc/Hexanes = 1/3, R_f = 0.32). ^{1}H NMR (400 MHz, $CDCl_3$) δ 7.24-7.32 (m, 5H, ArH), 7.16 (d, *J* = 8.6 Hz, 2H, ArH), 6.84 (d, *J* = 8.6 Hz, 2H, ArH), 5.88 (ddd, *J* = 17.2, 10.5, 5.6 Hz, 1H, CH=C), 5.24-5.30 (m, 2H, H-1, C=CH_2), 5.18 (dd, *J* = 10.5, 1.3 Hz, 1H, C=CH_2), 4.57 (d, *J* = 12.2 Hz, 1H, $PhCH_2$), 4.52 (d, *J* = 12.2 Hz, 1H, $PhCH_2$), 4.40-4.46 (m, 3H, H-5, H-6, $PhCH_2$), 4.32 (d, *J* = 12.0 Hz, 1H, $PhCH_2$), 3.96-4.09 (m, 2H, OCH_2), 3.74-3.78 (m, 5H, H-3, H-4, Ph-OMe), 3.61 (dd, *J* = 6.0, 5.8 Hz, 1H, H-6), 3.49 (s, 1H, H-2); ^{13}C NMR (100 MHz, $CDCl_3$) δ 159.49, 138.38, 134.43, 129.66, 129.52 (2C), 128.30 (2C), 127.69 (2C), 127.57, 117.41, 113.91 (2C), 100.26, 76.12, 74.37, 73.16, 73.05, 72.71, 71.81, 70.21, 64.30, 55.29; HRMS (ESI) *m/z* for $C_{24}H_{28}O_6Na$ $[M+Na]^+$: calcd 435.1784, found 435.1772.

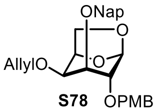

S78

Compound S78. The synthesis of compound **S78** is similar as described in the synthesis of compound **A7**, starting from compound **S76** to yield compound **S78** (160 mg, quant.) (EtOAc/Hexanes = 1/3, R_f = 0.28). (EtOAc/Hexanes = 1/2, R_f = 0.31). ^{1}H NMR (400 MHz, $CDCl_3$) δ 7.77-7.85 (m, 4H, ArH), 7.45-7.51 (m, 3H, ArH), 7.09 (d, *J* = 8.6 Hz, 2H, ArH), 6.80 (d, *J* = 8.6 Hz, 2H, ArH), 5.92 (ddd, *J* = 17.2, 10.3, 5.5 Hz, 1H, CH=C), 5.27-5.33 (m, 2H, H-1, C=CH_2), 5.21 (d, *J* = 10.3 Hz, 1H, C=CH_2), 4.74

(d, J = 12.5 Hz, 1H, $PhCH_2$), 4.70 (d, J = 12.5 Hz, 1H, $PhCH_2$), 4.51 (d, J = 6.7 Hz, 1H, H-6), 4.47-4.48 (m, 1H, H-5), 4.40 (d, J = 12.0 Hz, 1H, $PhCH_2$), 4.30 (d, J = 12.0 Hz, 1H, $PhCH_2$), 4.01-4.10 (m, 2H, OCH_2), 3.82 (m, 2H, H-3, H-4), 3.77 (s, 3H, Ph-OMe), 3.65 (dd, J = 6.7, 5.7 Hz, 1H, H-6), 3.54 (s, 1H, H-2); ^{13}C NMR (100 MHz, $CDCl_3$) δ 159.33, 135.78, 134.36, 133.11, 132.87, 129.51, 129.33 (2C), 127.98, 127.75, 127.59, 126.30, 126.02, 125.77 (2C), 117.31, 113.76 (2C), 100.18, 76.04, 74.41, 73.10 (2C), 72.59, 71.68, 70.16, 64.23, 55.14; HRMS (ESI) *m/z* for $C_{28}H_{30}O_6Na$ $[M+Na]^+$: calcd 485.1940, found 485.1938.

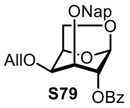

S79

Compound S79. Compound **S78** (89 mg, 0.19 mmol) was dissolved in dichloromethane (DCM, 3.0 mL). To this solution, water (0.5 mL) and trifluoroacetic acid (TFA, 1.0 mL, 13 mmol) were added in an ice bath. After the reaction was complete, the mixture was diluted with ethyl acetate (EtOAc) and extracted with saturated aqueous sodium bicarbonate and brine. The organic layer was dried over magnesium sulfate, filtered, and concentrated in vacuo. The residue was then dissolved in pyridine. To this solution, benzoyl chloride (88 μL, 0.76 mmol) was added in an ice bath. The reaction mixture was allowed to warm naturally to room temperature and stirred for 3 hours. Methanol was added to quench the reaction. The solvent was removed in vacuo, and the residue was extracted with ethyl acetate, water, and saturated brine. The organic layer was dried over magnesium sulfate, filtered, and concentrated in vacuo. The crude product was purified by column chromatography to afford compound **S79** (77 mg, 91% for two steps) (EtOAc/Hexanes = 1/3, R_f = 0.53). ^1H NMR (400 MHz, $CDCl_3$) δ 8.08 (d, J = 8.2 Hz, 2H, ArH), 7.81-7.89 (m, 4H, ArH), 7.56-7.60 (m, 2H, ArH), 7.44-7.47 (m, 4H, ArH), 5.88 (ddd, J = 17.2, 10.4, 5.5 Hz, 1H, CH=C),

5.57 (s, 1H, H-2), 5.23-5.28 (m, 2H, H-1, C=CH$_2$), 5.17 (d, J = 10.4 Hz, 1H, C=CH$_2$), 5.08 (d, J = 12.2 Hz, 1H, PhCH$_2$), 4.88 (d, J = 12.2 Hz, 1H, PhCH$_2$), 4.63 (d, J = 7.0 Hz, 1H, H-6), 4.57 (dd, J = 5.9, 4.2 Hz, 1H, H-5), 3.93-4.05 (m, 3H, H-3, OCH$_2$), 3.87 (dd, J = 4.6, 4.2 Hz, 1H, H-4), 3.75 (dd, J = 7.0, 5.9 Hz, 1H, H-6); ^{13}C NMR (100 MHz, CDCl$_3$) δ 165.32, 135.51, 134.18, 133.41, 133.15, 132.88, 129.77 (2C), 129.35, 128.42 (2C), 127.91 (2C), 127.57, 126.40, 125.90, 125.79, 125.70, 117.38, 99.50, 74.30, 72.92, 72.78, 72.60, 70.56, 69.86, 64.64; HRMS (ESI) *m/z* for $C_{27}H_{26}O_6Na$ $[M+Na]^+$: calcd 469.1627, found 469.1623.

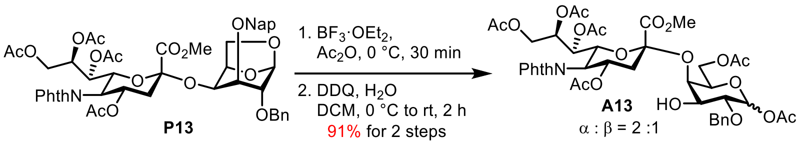

Compound A13α and A13β. To a solution of compound **P13** (30 mg, 31.4 μmol**)** in acetic anhydride (2 mL) was added boron trifluoride etherate (1.2 μL, 9.4 μmol) at 0 °C. The reaction mixture was stirred at 0 °C for 30 min and then neutralized with saturated $NaHCO_{3(aq)}$. The reaction mixture was extracted with EtOAc, and the resulting organic solution was washed with H_2O and brine. The organic layer was dried over $MgSO_4$, filtered, and concentrated. Next, to a solution of above residue in DCM (2 mL) was added 2,3-dichloro-5,6-dicyano-1,4-benzoquinone (1.4 mg, 62.8 μmol) and H_2O (200 μL) at 0 °C. The reaction mixture was gradually warmed to rt and stirred for 2 h. The reaction was quenched with saturated $Na_2S_2O_{3(aq)}$. The mixture was extracted with EtOAc, and the resulting organic solution was washed with saturated $NaHCO_{3(aq)}$ and brine. The organic layer was dried over $MgSO_4$, filtered, and concentrated. The residue was purified by silica gel chromatography (EtOAc/Hexanes = 1.5/1, R_f = 0.19 and 0.28 for compound **A13α** and **A13β**, respectively) to afford compound **A13α** (20 mg, 60% over two steps) and compound **A13β** (10 mg, 31% over two steps). For

compound **A13α**: ^{1}H NMR (400 MHz, $CDCl_3$) δ 7.85-7.81 (m, 2H), 7.76-7.71 (m, 2H), 7.38-7.26 (m, 5H), 6.29 (d, *J* = 3.6 Hz, 1H, Gal-1), 5.50-5.42 (m, 2H, Sia-4, Sia-8), 5.16 (dd, *J* = 9.4, 1.7 Hz, 1H, Sia-7), 4.92 (dd, *J* = 10.8, 1.7 Hz, 1H, Sia-6), 4.72 (s, 2H, CH_2Ph), 4.59 (d, *J* = 2.6 Hz, 1H, Gal-4), 4.23-4.18 (m ,2H, Sia-5, Sia-9), 4.13-4.00 (m, 4H, Gal-3, Gal-5, Gal-6, Sia-9), 3.94 (s, 3H, OMe), 3.77 (dd, *J* = 10.9, 4.2 Hz, 1H, Gal-6), 3.73 (dd, *J* = 10.3, 3.6 Hz, 1H, Gal-2), 3.44 (d, *J* = 6.3 Hz, 1H, OH), 2.95 (dd, *J* = 12.9, 4.9 Hz, 1H, Sia-3_{eq}), 2.18 (s, 3H, Ac), 2.15 (s, 3H, Ac), 2.09 (s, 3H, Ac), 2.05 (s, 3H, Ac), 1.95-1.87 (m, 4H, Sia-3_{ax}, Ac), 1.84 (s, 3H, Ac); ^{13}C NMR (125 MHz, $CDCl_3$) δ 170.84, 170.52, 170.06, 169.72, 169.51, 169.41, 167.69 (x2), 166.95, 138.07, 134.52 (x2), 131.24 (x2), 128.35 (x2), 127.98 (x2), 127.78, 123.72 (x2), 97.89, 90.29, 75.44, 73.38, 73.18, 69.90, 69.48, 68.28, 67.58, 66.37, 66.27, 62.72, 61.78, 53.60, 50.15, 38.62, 21.18, 21.02, 20.76, 20.64, 20.59, 20.54. HRMS (ESI) *m/z* for $C_{43}H_{49}N_1O_{21}Na$ $[M+Na]^+$: calcd 938.2695, found 938.2724. For compound **A13β**: ^{1}H NMR (400 MHz, $CDCl_3$) δ 7.84-7.82 (m, 2H), 7.75-7.73 (m, 2H), 7.37-7.27 (m, 5H), 5.56 (d, *J* = 8.1 Hz, 1H, Gal-1), 5.50-5.43 (m, 2H, Sia-4, Sia-8), 5.17 (dd, *J* = 9.4, 1.7 Hz, 1H, Sia-7), 4.93 (dd, *J* = 10.7, 1.7 Hz, 1H, Sia-6), 4.92 (d, *J* = 11.7 Hz, 1H, CH_2Ph), 4.74 (d, *J* = 11.7 Hz, 1H, CH_2Ph), 4.53 (d, *J* = 3.0 Hz, 1H, Gal-4), 4.22 (t, *J* = 10.7 Hz, 1H, Sia-5), 4.19 (dd, *J* = 12.6, 2.8 Hz, 1H, Sia-9), 4.11-4.05 (m, 2H, Gal-6, Sia-9), 3.94 (s, 3H, OMe), 3.82-3.76 (m, 3H, Gal-3, Gal-5, Gal-6), 3.59 (dd, *J* = 9.5, 8.1 Hz, 1H, Gal-2), 3.00 (dd, *J* = 13.0, 5.0 Hz, 1H, Sia-3_{eq}), 2.17 (s, 3H, Ac), 2.15 (s, 3H, Ac), 2.07-2.03 (m ,4H, Sia-3_{ax}, Ac), 2.01 (s, 3H, Ac), 1.89 (s, 3H, Ac), 1.84 (s, 3H, Ac); ^{13}C NMR (125 MHz, $CDCl_3$) δ 170.75, 170.43, 169.94, 169.65, 169,37, 169.01, 167.56 (x2), 166.83, 138.45, 134.47 (x2), 131.14 (x2), 128.22 (x2), 127.71 (x2), 127.56, 123.63 (x2), 97.91, 93.69, 78.84, 74.94, 72.63, 72.56 (x2), 69.43, 67.46, 66.27, 66.14, 62.37, 61.67, 53.55, 50.10, 38.60, 21.08, 20.87, 20.66, 20.50 (x3); HRMS (ESI) *m/z* for $C_{43}H_{49}N_1O_{21}Na$ $[M+Na]^+$: calcd 938.2695, found 938.2692.

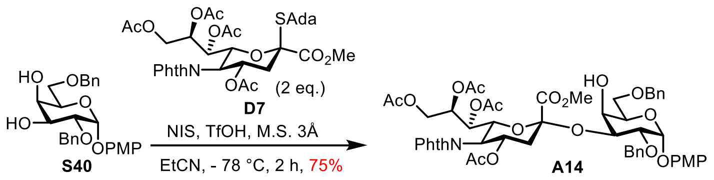

Compound A14. Compound **A14** was synthesized from glycosyl acceptor **S40** (100 mg 0.21 mmol) and glycosyl donor **D7** (384 mg, 0.32 mmol) by following the **General glycosylation procedure A**. The mixture was purified by silica gel chromatography (Acetone/Hexanes = 1/2, R_f = 0.19) to give compound **A14** (164 mg, 75%). ^{1}H NMR (600 MHz, $CDCl_3$) δ 7.80-7.79 (m, 2H), 7.70-7.68 (m, 2H), 7.34 (d, *J* = 7.3 Hz, 2H), 7.29-7.20 (m, 8H), 7.10-7.08 (m, 2H), 6.79-6.76 (m, 2H), 5.56 (td, *J* = 10.9, 5.1 Hz, 1H, Sia-4), 5.40 (ddd, *J* = 8.4, 5.7, 2.8 Hz, 1H, Sia- 8), 5.36 (d, *J* = 3.7 Hz, 1H, Gal-1), 5.14 (dd, *J* = 8.4, 2.2 Hz, 1H, Sia-7), 5.05 (dd, *J* = 10.8, 2.2 Hz, 1H, Sia-6), 4.79 (d, *J* = 12.2 Hz, 1H, C*H*$_2$Ph), 4.64 (dd, *J* = 9.9, 3.3 Hz, 1H, Gal-3), 4.63 (d, *J* = 12.2 Hz, 1H, C*H*$_2$Ph), 4.49 (s, 2H, C*H*$_2$Ph), 4.26 (dd, *J* = 12.5, 2.8 Hz, 1H, Sia-9), 4.23-4.19 (m, 2H, Gal-5, Sia-5), 4.11 (br, 1H, Gal-4), 3.97 (dd, *J* = 12.5, 5.7 Hz, 1H, Sia-9), 3.93 (dd, *J* = 9.9, 3.7 Hz, 1H, Gal-2), 3.90 (s, 3H, OMe), 3.79 (dd, *J* = 10.3, 5.1 Hz, 1H, Gal-6), 3.73 (s, 3H, OMe), 3.70 (dd, *J* = 10.3, 6.4 Hz, 1H, Gal-6), 3.10 (br, 1H, OH), 2.77 (dd, *J* = 13.1, 5.1 Hz, 1H, Sia-3$_{eq}$), 2.22 (dd, *J* = 13.1, 10.9 Hz, 1H, Sia-3$_{ax}$), 2.10 (s, 3H, Ac), 2.01 (s, 3H, Ac), 1.86 (s, 3H, Ac), 1.80 (s, 3H, Ac); ^{13}C NMR (150 MHz, $CDCl_3$) δ 170.44, 169.91, 169.83, 169.69 167.87 (x2), 167.52, 155.01. 150.96, 138.42, 138.07, 134.40 (x2), 131.23, 131.06, 128.23 (x2), 128.15 (x2), 127.60 (x2), 127.53, 127.39 (x3), 123.50 (x2), 118.86 (x2), 114.34 (x2), 99.01, 97.63, 73.95, 73.23 (x3), 72.97, 69.73, 69.56, 69.10, 69.02, 68.07, 66.92, 66.74, 62.05, 55.35, 53.13, 36.33, 21.00, 20.51 (x2), 20.48; HRMS (ESI) *m/z* for $C_{53}H_{57}NO_{20}Na$ [M+Na]$^+$: calcd 1050.3372, found 1050.3344.

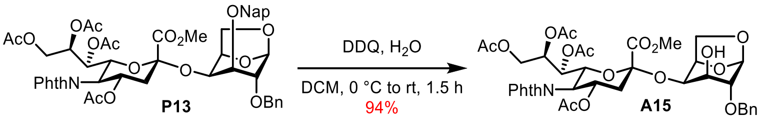

Compound A15. To a solution of compound **P13** (200 mg, 0.21 mmol) in DCM (5 mL) was added 2,3-dichloro-5,6-dicyano-1,4-benzoquinone (95 mg, 0.42 mmol) and H_2O (0.5 mL) at 0 °C. The reaction mixture was gradually warmed to rt and stirred for 1.5 h. The reaction was quenched with saturated $Na_2S_2O_{3(aq)}$. The mixture was extracted with EtOAc, and the resulting organic solution was washed with saturated $NaHCO_{3(aq)}$ and brine. The organic layer was dried over $MgSO_4$, filtered, and concentrated. The residue was purified by silica gel chromatography to afford compound **A15** (160 mg, 94%). ^{1}H NMR (400 MHz, $CDCl_3$) δ 7.85-7.81 (m, 2H), 7.75-7.71 (m , 2H), 7.38 (d, *J* = 7.0 Hz, 2H), 7.31 (dd, *J* = 7.0, 7.0 Hz, 2H), 7.04 (dd, *J* = 7.0, 7.0 Hz, 1H), 5.45 (td, *J* = 10.5, 4.9 Hz, 1H, Sia-4), 5.34 (br, 1H, Gal-1), 5.34-5.30 (m, 1H, Sia-8), 5.20 (dd, *J* = 10.9, 1.5 Hz,1H, Sia-6), 5.12 (dd, *J* = 9.5, 1.5 Hz, 1H, Sia-7), 4.83 (d, *J* = 12.1 Hz, 1H, C*H₂*Ph), 4.56 (d, *J* = 12.1 Hz, 1H, C*H₂*Ph), 4.36 (t, *J* = 4.5 Hz, 1H, Gal-5), 4.31-4.29 (m , 2H, Gal-3 and Gal-6), 4.22-4.17 (m, 2H, Sia-5, Sia-9), 4.06-4.00 (m, 2H, Gal-4, Sia-9), 3.89 (s, 3H, OMe), 3.61 (dd, *J* = 6.7, 4.5 Hz, 1H, Gal-6), 3.55 (br, 1H, Gal-2), 2.99 (br, 1H, OH), 2.84 (dd, *J* = 13.4, 4.9 Hz, 1H, Sia-3_{eq}), 2.14 (s, 3H, Ac), 2.14 (s, 3H, Ac), 2.08 (dd, *J* = 13.4, 10.5 Hz, 1H, Sia-3_{ax}), 1.85 (s, 3H, Ac), 1.84 (s, 3H, Ac); ^{13}C NMR (100 MHz, $CDCl_3$) δ 170.44, 169.73 (x2), 169.59, 167.63 (x2), 167.17, 137.88, 134.44 (x2), 131.25 (x2), 128.34 (x2), 128.11 (x2), 127.76, 123.68 (x2), 100.87, 100.08, 77.73, 72.58, 71.93, 70.87, 69.46, 67.81, 67.57, 66.60 (x2), 64.07, 61.92, 53.38, 50.32, 38.31, 20.89, 20.62, 20.56 (x2); HRMS (ESI) *m/z* for $C_{39}H_{43}NO_{18}Na$ $[M+Na]^+$: calcd 836.2379, found 836.2406.

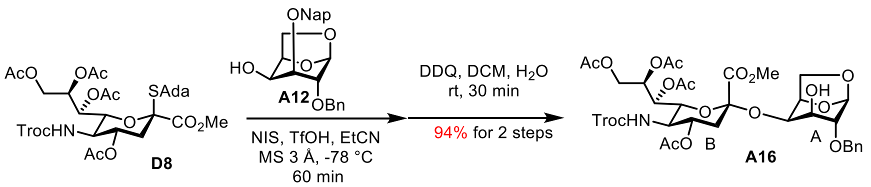

Compound A16. Compound **A16** was synthesized from glycosyl acceptor **A12** (3.72 g, 9.49 mmol) and glycosyl donor **D8** (1.82 g, 2.35 mmol) by following the **General**

glycosylation procedure A. Next, a solution of above residue in H_2O/DCM (4 mL/40 mL) was added 2,3-dichloro-5,6-dicyano-1,4-benzoquinone (1.06 g, 4.71 mmol) and stirred at rt for 30 min. The reaction mixture was quenched with saturated $Na_2S_2O_{3(aq)}$. The reaction mixture was extracted with EtOAc, and the resulting organic solution was washed with saturated $NaHCO_{3(aq)}$ and brine. The organic layer was dried over $MgSO_4$, filtered, and concentrated. The residue was purified by silica gel chromatography (EtOAc/Hexanes = 1/1, R_f = 0.20) to afford compound **A16** (1.89 g, 94% over 2 steps). ^{1}H NMR (500 MHz, $CDCl_3$) δ 7.37 (d, *J* = 7.4 Hz, 2H), 7.30 (t, *J* = 7.4 Hz, 2H), 7.24 (t, *J* = 7.4 Hz, 1H), 5.34 (d, *J* = 9.8 Hz, 1H, B-7), 5.32 (br, 1H, A-1), 5.27-5.25 (m, 1H, B-8), 5.11-5.06 (m, 2H, B-4, B_5-NH), 4.91 (d, *J* = 12.1 Hz, 1H), 4.82 (d, *J* = 12.1 Hz, 1H), 4.54 (d, *J* = 12.1 Hz, 1H), 4.48 (d, *J* = 12.1 Hz, 1H), 4.38 (d, *J* = 9.9 Hz, 1H, B-6), 4.32 (dd, *J* = 4.4, 4.4 Hz, 1H, A-5), 4.27-4.23 (m, 3H, A-3, A-6, B-9), 4.13 (dd, *J* = 12.6, 4.4 Hz, 1H, B-9), 3.91 (dd, *J* = 4.4, 4.4 Hz, 1H, A-4), 3.83 (s, 3H, OCH_3), 3.59 (dd, *J* = 7.8, 4.4 Hz, 1H, A-6), 3.52 (br, 1H, A-2), 3.48 (ddd, *J* = 9.9, 9.9, 9.9 Hz, 1H, B-5), 2.93 (br, 1H, A_3-OH), 2.69 (dd, *J* = 13.2, 4.5 Hz, 1H, B-3_{eq}), 2.14 (s, 3H, Ac), 2.11 (s, 3H, Ac), 2.01 (s, 3H, Ac), 2.01-1.97 (m, 1H, B-3_{ax}), 1.97 (s, 3H, Ac); ^{13}C NMR (125 MHz, $CDCl_3$) δ 170.58, 170.20, 170.15, 169.40, 167.50, 153.96, 137.85, 128.34, 128.13, 127.77, 100.87, 100.04, 95.36, 77.61, 74.40, 72.49, 71.94 (x2), 71.24, 67.73, 67.65 (x2), 66.87, 64.04, 61.88, 53.39, 51.79, 38.26, 20.85, 20.81, 20.76, 20.66; HRMS (ESI) *m/z* for $C_{34}H_{42}Cl_3NNaO_{18}$ $[M+Na]^+$: calcd 880.1365, found 880.1385.

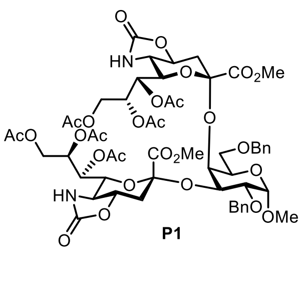

P1

Compound P1. Compound **P1** was synthesized from glycosyl acceptor **A1** (60 mg 0.08 mmol) and glycosyl donor **D2** (83 mg, 0.13 mmol) by following the **General**

glycosylation procedure B. The mixture was purified by silica gel chromatography (EtOAc/Hexanes = 2/1, R_f = 0.18) to afford compound **P1** (38 mg, 42%). ^{1}H NMR (400 MHz, $CDCl_3$) δ 7.37-7.22 (m, 10H, ArH), 5.59 (dd, *J* = 10.2, 4.0 Hz, 1H, H-8'), 5.36-5.32 (m, 2H, H-8", NH), 5.28 (br, 1H, NH), 5.09 (dd, *J* = 10.2, 1.0 Hz, 1H, H-7'), 5.04 (dd, *J* = 8.9, 1.8 Hz, 1H, H-7"), 4.74 (d, *J* = 12.5 Hz, 1H, $PhCH_2$), 4.65 (d, *J* = 12.5 Hz, 1H, $PhCH_2$), 4.58-4.54 (m, 2H, H-1, $PhCH_2$), 4.43-4.28 (m, 7H, H-4, H-4", H-6', H-9', H-9", $PhCH_2$), 4.24 (dd, *J* = 12.8, 4.0 Hz, 1H, H-9"), 4.15 (dd, *J* = 10.6, 2.4 Hz, 1H, H-3), 4.00-3.89 (m, 5H, H-4', H-6", CO_2Me), 3.74 (dd, *J* = 6.4, 5.8 Hz, 1H, H-5), 3.59 (dd, *J* = 10.6, 3.5 Hz, 1H, H-2), 3.52 (s, 3H, CO_2Me), 3.39 (s, 3H, OMe), 3.28-3.26 (m, 2H, H-6), 3.02-2.93 (m, 3H, H-3"$_{eq}$, H-5', H-5"), 2.81 (dd, *J* = 12.9, 3.7 Hz, 1H, H-3'$_{eq}$), 2.50 (dd, *J* = 13.0, Hz, 1H, H-3'$_{ax}$), 2.13 (s, 6H, (CO)CH_3), 2.04 (s, 3H, (CO)CH_3), 2.03 (s, 3H, (CO)CH_3), 2.00 (s, 3H, (CO)CH_3), 1.89-1.83 (m, 4H, (CO)CH_3, H-3"$_{ax}$); ^{13}C NMR (100 MHz, $CDCl_3$) δ 171.32, 171.05, 170.57, 170.43, 169.55, 169.20, 167.08, 166.98, 159.73, 159.22, 138.25, 137.43, 128.59 (2C), 128.35 (2C), 128.24, 128.22 (2C), 127.59, 127.23 (2C), 101.69, 99.27, 98.26, 77.32, 76.61, 74.61, 73.49, 73.31, 72.78 (2C), 72.29, 71.31, 70.09, 68.99, 68.73, 68.37, 67.77, 67.48, 61.65, 61.35, 57.88, 57.26, 55.39, 53.99, 52.61, 35.71, 34.43, 20.86, 20.69, 20.64, 20.63, 20.45, 20.37; HRMS (ESI) *m/z* for $C_{55}H_{68}N_2O_{28}Na$ $[M+Na]^+$: calcd 1227.3856, found 1227.3844.

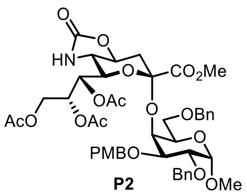

P2

Compound P2. Compound **P2** was synthesized from glycosyl acceptor **A2** (89 mg 0.18 mmol) and glycosyl donor **D1** (75 mg, 0.14 mmol) by following the **General glycosylation procedure A**. The mixture was purified by silica gel chromatography (EtOAc/Hexanes = 1/2 twice, R_f = 0.15) to afford compound **P2** (104 mg, 82%). ^{1}H

NMR (400 MHz, $CDCl_3$) δ 7.36-7.24 (m, 14H, ArH), 6.79 (d, *J* = 8.6 Hz, 2H, ArH), 5.50-5.47 (m, 1H, H-8'), 5.32 (br, 1H, NH), 5.04 (dd, *J* = 8.1, 1.4 Hz, 1H, H-7'), 4.69 (d, *J* = 12.0 Hz, 1H, $PhCH_2$), 4.65 (d, *J* = 11.1 Hz, 1H, $PhCH_2$), 4.59-4.37 (m, 8H, H-4', H-6', H-9', H-1, $PhCH_2$), 4.28 (dd, *J* = 12.8, 4.4 Hz, H-9'), 4.11-4.07 (m, 2H, H-2, H-4), 3.87 (dd, *J* = 8.9, 5.4 Hz, 1H, H-5), 3.78-3.75 (m, 4H, H-3', Ph-OMe), 3.62 (dd, *J* = 8.9, 8.9 Hz, 1H, H-6'), 3.45 (dd, *J* = 8.9, 5.4 Hz, 1H, H-6'), 3.32 (s, 3H, CO_2Me), 3.30 (s, 3H, OMe), 3.06 (dd, *J* = 10.8, 10.2 Hz, 1H, H-5'), 2.78 (dd, *J* = 12.2, 3.9 Hz, 1H, H-3'$_{eq}$), 2.18 (s, 3H, $(CO)CH_3$), 2.00 (s, 3H, $(CO)CH_3$), 1.96 (s, 3H, $(CO)CH_3$), 1.86 (dd, *J* = 12.2, 12.1 Hz, 1H, H-3'$_{ax}$); ^{13}C NMR (100 MHz, $CDCl_3$) δ 171.23, 170.39, 169.92, 167.80, 159.37, 159.07, 138.59, 136.78, 130.40, 130.14 (2C), 128.50 (2C), 128.46 (2C), 128.30 (2C), 128.16, 127.97 (2C), 127.66, 113.35 (2C), 99.10 (2C), 78.53, 76.50, 75.32, 73.86, 73.84, 73.53, 72.85, 69.40, 68.68, 68.47, 68.35, 67.66, 61.63, 57.73, 55.62, 55.17, 52.15, 39.21, 20.87, 20.75, 20.59; HRMS (ESI) *m/z* for $C_{46}H_{55}NO_{18}Na$ $[M+Na]^+$: calcd 932.3311, found 932.3309.

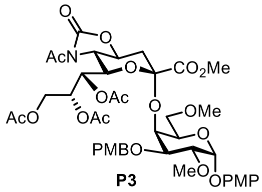

P3

Compound P3. Compound **P3** was synthesized from glycosyl acceptor **A3** (30 mg, 69.0 μmol) and glycosyl donor **D3** (78 mg, 124.0 μmol) by following **General glycosylation procedure A**. The mixture was purified by silica gel chromatography (Acetone/Hexanes = 1/3, R*f* = 0.16) to give compound **P3** (20 mg, 32%). ^1H NMR (600 MHz, $CDCl_3$) δ 7.36-7.34 (m, 2H), 7.00-6.97 (m, 2H), 6.86-6.84 (m, 2H), 6.81-6.78 (m, 2H), 5.84 (dd, *J* = 7.0, 1.0 Hz, 1H, Sia-7), 5.63 (td, *J* = 7.0, 2.3 Hz, 1H. Sia-8), 5.51 (d, *J* = 3.3 Hz, 1H. Gal-1), 4.76 (dd, *J* = 9.9, 1.0 Hz, 1H, Sia-6), 4.72 (d, *J* = 11.2 Hz, 1H, C*H*$_2$Ph), 4.53 (d, *J* = 11.2 Hz, C*H*$_2$Ph), 4.48 (dd, *J* = 12.3, 2.3 Hz, 1H, Sia-9), 4.44 (td,

J = 12.0, 3.8 Hz, 1H, Sia-4), 4.30-4.27 (m, 2H, Sia-9, Gal-4), 4.06 (dd, *J* = 8.8, 5.7 Hz, 1H, Gal-6), 3.98 (dd, *J* = 10.4, 3.3 Hz, 1H, Gal-2), 3.94 (dd, *J* = 10.4, 2.2 Hz, 1H, Gal-3), 3.79 (s, 3H, OMe), 3.75 (s, 3H, OMe), 3.63 (dd, *J* = 12.0 9.9 Hz, 1H, Sia-5), 3.51 (t, *J* = 8.8 Hz, 1H, Gal-5), 3.49 (s, 3H, OMe), 3.50 (s, 3H, OMe), 3.28 (dd, *J* = 8.8, 5.7 Hz, 1H, Gal-6), 3.21 (s, 3H, OMe), 2.81 (dd, *J* = 12.0, 3.8 Hz, 1H, Sia-3_{eq}), 2.49 (s, 3H, Ac), 2.15 (s, 3H, Ac), 2.07 (s, 3H, Ac), 1.96 (s, 3H, Ac), 1.87 (t, *J* = 12.0 Hz, 1H, Sia-3_{ax}); ^{13}C NMR (150 MHz, $CDCl_3$) δ 171.51, 170.76, 170.62, 170.28, 167.54, 159.10, 154.94, 153.75, 150.81, 149.47, 130.10 (x2), 118.02 (x2), 114.47 (x2), 113.40 (x2), 98.97, 96.71, 78.28, 77.09, 74.86, 74.11, 73.53, 71.71, 70,01, 69.64, 68.64, 68.53, 62.82, 59.71, 59.52, 58.36, 55.59, 55.19, 52.22, 24.69, 21.01, 20.84, 20.67; HRMS (ESI) *m/z* for $C_{42}H_{53}NO_{20}Na$ $[M+Na]^+$: calcd 914.3059, found 914.3085.

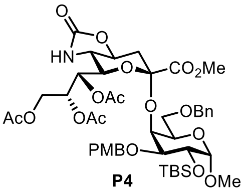

P4

Compound P4. Compound **P4** was synthesized from glycosyl acceptor **A4** (42 mg, 0.08 mmol) and glycosyl donor **D1** (53 mg, 0.10 mmol) by following **General glycosylation procedure A**. The mixture was purified by silica gel chromatography (EtOAc /Hexanes = 1/1, R_f = 0.33) to give compound **P4** (30 mg, 40%). ^{1}H NMR (400 MHz, $CDCl_3$) δ 7.35-7.24 (m, 7H, ArH), 6.80 (d, *J* = 8.6 Hz, 2H, ArH), 5.50-5.47 (m, 1H, H-8'), 5.32 (br, 1H, NH), 5.07 (dd, *J* = 8.6, 1.5 Hz, 1H, H-7'), 4.62-4.56 (m, 3H, H-1, $PhCH_2$), 4.51-4.34 (m, 5H, H-4', H-6', H-9', $PhCH_2$), 4.29 (dd, *J* = 10.0, 3.8 Hz, 1H, H-2), 4.24 (dd, *J* = 12.8, 4.0 Hz, 1H, H-9'), 4.05 (br, 1H, H-4), 3.88 (dd, *J* = 8.3, 5.5 Hz, 1H, H-5), 3.78 (s, 3H, Ph-OMe), 3.66-3.61 (m, 2H, H-3, H-6), 3.46 (dd, *J* = 8.9, 5.5 Hz, 1H, H-6), 3.34 (s, 3H, CO_2Me), 3.28 (s, 3H, OMe), 3.06 (dd, *J* = 10.9, 10.9 Hz, 1H, H-5'), 2.81 (dd, *J* = 12.2, 3.8 Hz, 1H, H-$3'_{eq}$), 2.18 (s, 3H, $(CO)CH_3$), 2.00 (s, 3H,

(CO)CH_3), 1.95 (s, 3H, (CO)CH_3), 1.85 (dd, *J* = 12.2, 12.1 Hz, 1H, H-3'$_{ax}$), 0.88 (s, 9H, Si-*t*Bu), 0.08 (s, 3H, $SiCH_3$), 0.04 (s, 3H, $SiCH_3$); ^{13}C NMR (100 MHz, $CDCl_3$) δ 171.16, 170.39, 169.88, 167.75, 159.43, 159.01, 136.93, 130.48, 129.98 (2C), 128.52 (2C), 128.46 (2C), 128.16, 113.24 (2C), 100.77, 99.22, 78.74, 74.06, 73.86, 72.79, 69.61, 69.24, 68.97, 68.62, 67.91, 61.52, 57.86, 55.58, 55.23, 52.37, 39.16, 29.68, 29.27, 25.77 (3C), 20.86, 20.76, 20.61, 18.05, -4.41, -4.70; HRMS (ESI) *m/z* for C45H63NO18SiNa $[M+Na]^+$: calcd 956.3712, found 956.3708.

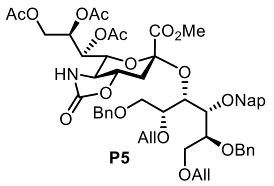

P5

Compound P5. Compound **P5** was synthesized from glycosyl acceptor **A5** (59 mg, 0.10 mmol) and glycosyl donor **D2** (76 mg, 0.12 mmol) by following **General glycosylation procedure B**. The mixture was purified by silica gel chromatography (EtOAc /Hexanes = 1/1, R_f = 0.45) to give compound **P5** (94 mg, 93%). ^{1}H NMR (400 MHz, $CDCl_3$) δ 7.82-7.70 (m, 4H, ArH), 7.48-7.42 (m, 3H, ArH), 7.31-7.22 (m, 10H, ArH), 5.93-5.80 (m, 2H, H-C=C), 5.47 (dd, *J* = 10.2, 2.0 Hz, 1H, H-8'), 5.33 (br, 1H, NH), 5.27-5.06 (m, 5H, H-7', C=CH_2), 4.89 (d, *J* = 11.6 Hz, 1H, PhCH_2), 4.85 (d, *J* = 11.6 Hz, 1H, PhCH_2), 4.71 (d, *J* = 11.4 Hz, 1H, PhCH_2), 4.67 (d, *J* = 11.4 Hz, 1H, PhCH_2), 4.41 (m, 2H, PhCH_2), 4.24 (d, *J* = 2.0 Hz, 2H, H-9'), 4.20 (d, *J* = 2.7 Hz, 1H), 4.10 (dd, *J* = 5.0, 4.6 Hz, 2H), 4.02-3.95 (m, 5H), 3.89 (ddd, *J* = 13.9, 11.0, 3.4 Hz, 1H, H-4'), 3.75-3.62 (m, 8H), 3.00-2.91 (m, 2H, H-3'$_{eq}$, H-5), 2.17 (s, 3H, (CO)CH_3), 2.05-2.02 (m, 4H, H-3'$_{ax}$, (CO)CH_3), 1.98 (s, 3H, (CO)CH_3); ^{13}C NMR (100 MHz, $CDCl_3$) δ 171.24, 170.37, 169.35, 167.55, 159.23, 138.92, 138.69, 136.58, 135.31, 134.97, 133.15, 132.83, 128.14 (4C), 127.86 (2C), 127.77, 127.71, 127.64, 127.32, 127.25 (2C), 127.21, 126.36, 126.23, 126.00, 125.75, 116.76, 116.60, 100.55, 83.06, 80.17, 79.15, 76.91, 75.33, 74.84, 73.92, 73.26, 72.75, 72.36, 72.30, 71.30, 70.83, 68.44, 67.15, 61.33,

57.91, 52.92, 37.30, 20.98, 20.56, 20.49; HRMS (ESI) *m/z* for $C_{54}H_{63}NO_{17}Na$ [M+Na]$^+$: calcd 1020.3994, found 1020.3990.

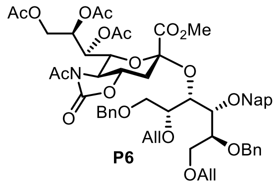

Compound P6. Compound **P6** was synthesized from glycosyl acceptor **A5** (43 mg, 0.07 mmol) and glycosyl donor **D4** (50 mg, 0.07 mmol) by following **General glycosylation procedure B**. The mixture was purified by silica gel chromatography (EtOAc /Hexanes = 1/1, R_f = 0.50) to give compound **P6** (31 mg, 40%). ^{1}H NMR (400 MHz, $CDCl_3$) δ 7.45-7.39 (m, 3H, ArH), 7.30-7.21 (m, 10H, ArH), 5.92-5.83 (m, 2H, CH=C), 5.64 (dd, *J* = 8.8, 0.8 Hz, 1H, H-7'), 5.38 (m, 1H, H-8'), 5.21 (dd, *J* = 10.8, 1.6 Hz, 1H, C=CH_2), 5.17 (dd, *J* = 10.8, 1.6 Hz, 1H, C=CH_2), 5.08 (m, 2H, C=CH_2), 4.89 (d, *J* = 11.5 Hz, 1H, PhCH_2), 4.85 (d, *J* = 11.5 Hz, 1H, PhCH_2), 4.71 (d, *J* = 11.4 Hz, 1H, PhCH_2), 4.66 (d, *J* = 11.4 Hz, 1H, PhCH_2), 4.43-4.40 (m, 3H, H-6, PhCH_2), 4.30 (dd, *J* = 12.4, 3.1 Hz, 1H, H-9'), 4.26 (d, *J* = 4.0 Hz, 1H), 4.15-4.11 (m, 3H), 4.02-3.93 (m, 5H), 3.83-3.77 (m, 2H), 3.72-3.65 (m, 6H), 3.50 (dd, *J* = 11.0, 9.6 Hz, 1H, H-5'), 2.82 (dd, *J* = 12.1, 3.6 Hz, 1H, H-3'$_{eq}$), 2.47 (s, 3H, (CO)CH_3), 2.12-2.05 (m, 4H, H-3'$_{ax}$, (CO)CH_3), 2.01 (s, 3H, (CO)CH_3), 1.98 (s, 3H, (CO)CH_3); ^{13}C NMR (100 MHz, $CDCl_3$) δ 171.86, 170.57, 169.72, 169.57, 167.97, 153.61, 139.00, 138.80, 136.61, 135.34, 134.98, 133.17, 132.85, 128.16 (2C), 128.15 (2C), 127.86 (2C), 127.84, 127.75, 127.67, 127.34 (2C), 127.32, 127.21, 126.33, 126.25, 125.99, 125.77, 116.74, 116.39, 99.44, 83.39, 80.13, 79.71, 75.64, 75.19, 74.95, 74.32, 73.85, 72.75, 72.44, 72.35, 71.54, 71.30, 71.14, 68.88, 62.49, 59.09, 52.97, 35.89, 24.64, 21.10, 20.80, 20.64; HRMS (ESI) *m/z* for $C_{56}H_{65}NO_{18}Na$ [M+Na]$^+$: calcd 1062.4099, found 1062.4098.

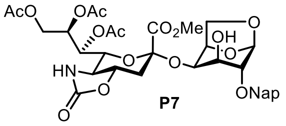

Compound P7. Compound **P7** was synthesized from glycosyl acceptor **A7** (29 mg, 0.10 mmol) and glycosyl donor **D2** (60 mg, 0.10 mmol) by following **General glycosylation procedure B**. The mixture was purified by silica gel chromatography (EtOAc /Hexanes = 2/1, R_f = 0.31) to give compound **P7** (20 mg, 29%, α:β = 2:1). ^{1}H NMR (400 MHz, $CDCl_3$) δ 7.51-7.44 (m, 3H, ArH), 7.83-7.79 (m, 4H, ArH), 5.37-5.35 (m, 3H, H-1, H-8', NH), 5.13 (dd, *J* = 10.5, 1.1 Hz, 1H, H-7'), 4.99 (d, *J* = 12.1 Hz, 1H, $PhCH_2$), 4.71 (d, *J* = 12.1 Hz, 1H, $PhCH_2$), 4.38-4.28 (m, 5H, H-9', H-6', H-5, H-6), 4.23 (d, *J* = 4.1 Hz, 1H, H-3), 3.97 (ddd, *J* = 12.4, 10.3, 3.5 Hz, 1H, H-4'), 3.88-3.85 (m, 4H, CO_2Me, H-4), 3.62-3.59 (m, 2H, H-2, H-6), 3.07 (dd, *J* = 10.3, 10.2 Hz, 1H, H-5'), 2.95 (dd, *J* = 12.4, 3.5 Hz, 1H, H-3'$_{eq}$), 2.84 (br, 1H, OH), 2.18 (s, 3H, $(CO)CH_3$), 2.17-2.14 (m, 4H, H-3'$_{ax}$, $(CO)CH_3$), 2.02 (s, 3H, $(CO)CH_3$); ^{13}C NMR (100 MHz, $CDCl_3$) δ 171.34, 170.42, 169.37, 167.44, 158.93, 135.28, 133.26, 133.08, 128.17, 127.89, 127.66, 127.09, 126.06 (2C), 125.91, 101.82, 100.90, 77.74, 75.98, 74.61, 72.50, 72.37, 72.18, 68.24, 67.85, 66.97, 64.13, 61.47, 57.92, 53.64, 38.26, 20.86, 20.75, 20.60; HRMS (ESI) *m/z* for $C_{34}H_{39}NO_{16}Na$ $[M+Na]^+$: calcd 740.2167, found 740.2166.

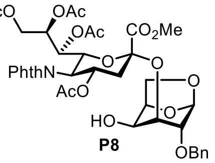

P8

Compound P8. A solution of glycosyl acceptor **A8** (11 mg, 43.6 μmol) and activated molecular sieve AW-300 (200 mg) in EtCN (2 mL) was stirred under an argon atmosphere at rt for 30 min. The solution was cooled to -78 °C, and then TMSOTf (2.3 μL, 13.1 μmol) was slowly added. After being stirred for 10 min, a solution of glycosyl donor **D5** (100 mg, 131.0 μmol) in EtCN (1 mL) was slowly added to the reaction mixture and the resulting mixture was stirred for 1 h. After completion of the reaction, the reaction was quenched with Et_3N. The solid was filtered through a pad of Celite®, and the resulting solution was concentrated under reduced pressure. The residue was

dissolved in EtOAc and then washed with H_2O and brine. The organic layer was dried over $MgSO_4$, filtered, and concentrated. The residue was then purified by silica gel chromatographyto give compound **A15** (15 mg, 42%, EtOAc/Hexanes = 1/1, R_f = 0.27) and compound **P8** (5 mg, 14%, EtOAc/Hexanes = 1/1, R_f = 0.17). ^{1}H NMR (600 MHz, $CDCl_3$) δ 7.83-7.82 (m, 2H), 7.73-7.72 (m, 2H), 7.38 (d, *J* = 7.5 Hz, 2H), 7.33 (dd, *J* = 7.5, 7.5 Hz, 2H), 7.26 (dd, *J* = 7.5, 7.5 Hz, 1H), 5.52 (td, *J* = 10.2, 5.4 Hz, 1H, Sia-4), 5.42 (ddd, *J* = 9.5, 4.9, 2.9 Hz, 1H, Sia-8), 5.39 (t, *J* = 1.4 Hz, 1H, Gal-1), 5.13 (dd, *J* = 9.5, 1.8 Hz, 1H, Sia-7), 5.04 (dd, *J* = 10.7, 1.8 Hz, 1H, Sia-6), 4.79 (d, *J* = 11.9 Hz, 1H, CH_2Ph), 4.65 (d, *J* = 11.9 Hz, 1H, CH_2Ph), 4.38 (t, *J* = 5.0 Hz, 1H, Gal-5), 4.34 (dq, *J* = 5.4, 1.4 Hz, 1H, Gal-3), 4.26 (dd, *J* = 10.7, 10.2 Hz, 1H, Sia-5), 4.21 (d, *J* = 7.0 Hz, 1H, Gal-6), 4.08-4.06 (m, 1H, Gal-4), 4.05 (dd, *J* = 12.6, 2.9 Hz, 1H, Sia-9), 3.91 (dd, *J* = 12.6, 4.9 Hz, 1H, Sia-9), 3.88 (s, 3H, OMe), 3.80 (t, *J* = 1.4 Hz, 1H, Gal-2), 3.60 (dd, *J* = 7.0, 5.0 Hz, 1H, Gal-6), 2.96 (d. *J* = 8.2 Hz, 1H, OH), 2.76 (dd, *J* = 13.3, 5.4 Hz, 1H, Sia-3_{eq}), 2.16 (s, 3H, Ac), 2.11 (s, 3H, Ac), 2.00 (dd, *J* = 13.3, 10.2 Hz, 1H, Sia-3_{ax}), 1.86 (s, 3H, Ac), 1.78 (s, 3H, Ac); ^{13}C NMR (150 MHz, $CDCl_3$) δ 170.42, 169.92, 169.82, 169.66, 167.98, 167.64 (x2), 137.86, 134.41 (x2), 131.36 (x2), 128.49 (x2), 127.89, 127.50 (x2), 123.63 (x2), 99.78 (x2), 78.80, 74.47, 72.56, 72.42, 69.33, 67.66, 66.74, 64.47, 63.48, 61.84, 53.39, 50.34, 37.81, 21.03, 20.67, 20.59, 20.46. HRMS (ESI) *m/z* for $C_{39}H_{43}NO_{18}Na$ $[M+Na]^+$: calcd 836.2379, found 836.2374.

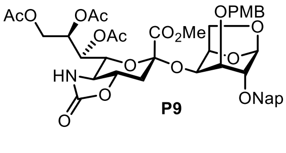

P9

Compound P9. Compound **P9** was synthesized from glycosyl acceptor **A9** (31 mg, 0.07 mmol) and glycosyl donor **D1** (48 mg, 0.09 mmol) by following **General glycosylation procedure A**. The mixture was purified by silica gel chromatography (EtOAc /Hexanes = 2/1, R_f = 0.44) to give compound **P9** (31 mg, 50%, α:β = 1:1.2). ^{1}H NMR (400 MHz, $CDCl_3$) δ 7.49-7.41 (m, 3H, ArH), 7.22 (d, *J* = 8.5 Hz, 2H, ArH),

6.83 (d, J = 8.5 Hz, 2H, ArH), 5.57 (m, 1H, H-8'), 5.36 (s, 1H, NH), 5.27 (s, 1H, H-1), 5.13 (dd, J = 10.1, 1.6 Hz, 1H, H-7'), 4.80 (d, J = 11.3 Hz, 1H, $PhCH_2$), 4.65 (d, J = 11.3 Hz, 1H, $PhCH_2$), 4.47 (dd, J = 10.0, 1.7 Hz, 1H, H-9'), 4.43-4.28 (m, 6H, H-5, H-6, H-6', H-9', $PhCH_2$), 4.06-3.99 (m, 2H, H-4', H-4), 3.91 (d, J = 5.0 Hz, 1H, H-3), 3.82 (s, 3H, CO_2Me), 3.78 (s, 3H, Ph-OMe), 3.58 (dd, J = 6.6, 5.9 Hz, 1H, H-6), 3.43 (s, 1H, H-2), 3.00 (dd, J = 10.8, 10.1 Hz, 1H, H-5'), 2.86 (dd, J = 12.6, 3.8 Hz, 1H, H-3'$_{eq}$), 2.19 (s, 3H, (CO)CH_3), 2.10 (dd, J = 13.1, 12.6 Hz, 1H, H-3'$_{ax}$), 1.98 (s, 3H, (CO)CH_3), 1.93 (s, 3H, (CO)CH_3); ^{13}C NMR (100 MHz, $CDCl_3$) δ 171.23, 170.53, 169.27, 168.77, 159.31, 159.06, 135.21, 133.17, 133.06, 130.74, 129.05 (2C), 128.26, 127.84, 127.69, 126.92, 126.18, 126.01, 125.83, 113.83 (2C), 101.45, 100.60, 76.17, 74.23, 73.74, 73.18, 71.90, 70.89, 68.98, 66.59, 64.20, 61.95, 58.09, 55.31, 53.23, 37.81, 20.84, 20.57, 20.49; HRMS (ESI) *m/z* for $C_{42}H_{47}NO_{17}Na$ $[M+Na]^+$: calcd 860.2742, found 860.2733.

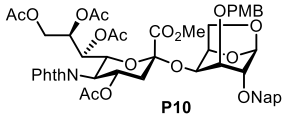

P10

Compound P10. Compound **P10** was synthesized from glycosyl acceptor **A9** (25 mg, 0.05 mmol) and glycosyl donor **D5** (45 mg, 0.06 mmol) by following **General glycosylation procedure B**. The mixture was purified by silica gel chromatography (EtOAc /Hexanes = 1/1, R_f = 0.26) to give compound **P10** (42 mg, 72%, α:β = 17:1). ^{1}H NMR (400 MHz, $CDCl_3$) δ 7.83-7.72 (m, 7H, ArH), 7.70 (s, 1H, ArH), 7.47-7.34 (m, 5H, ArH), 6.90 (d, J = 8.6 Hz, 2H, ArH), 5.58 (ddd, J = 9.6, 6.2, 2.7 Hz, 1H, H-8'), 5.50 (ddd, J = 12.4, 10.5, 5.0 Hz, 1H, H-4'), 5.32 (dd, J = 10.8, 2.0 Hz, 1H, H-6'), 5.27 (s, 1H, H-1), 5.16 (dd, J = 9.6, 2.0 Hz, 1H, H-7'), 4.81-4.77 (m, 2H, $PhCH_2$), 4.54 (d, J = 11.6 Hz, 1H, $PhCH_2$), 4.43 (d, J = 7.0 Hz, 1H, H-6), 4.30-4.18 (m, 5H, H-5', H-9', H-4, H-5, $PhCH_2$), 4.06-4.00 (m, 2H, H-3, H-9'), 3.90 (s, 3H, CO_2Me), 3.81 (s, 3H, Ph-OMe), 3.59 (dd, J = 6.2, 5.3 Hz, 1H, H-6), 3.39 (s, 1H, H-2), 2.80 (dd, J = 13.0, 5.0 Hz, 1H, H-3'$_{eq}$), 2.19 (s, 3H, (CO)CH_3), 2.05 (s, 3H, (CO)CH_3), 1.99 (dd, J = 13.0, 12.4 Hz,

1H, H-3'$_{ax}$), 1.82 (s, 3H, (CO)CH_3), 1.80 (s, 3H, (CO)CH_3); ^{13}C NMR (100 MHz, $CDCl_3$) δ 170.51, 169.90, 169.73, 169.53, 167.92 (C-1', 3JC−1′,H−3′ax = 6.2 Hz), 167.64 (2C), 159.26, 135.40, 134.40 (2C), 133.14, 132.98, 131.40, 131.18, 130.94, 129.60 (2C), 128.13, 127.83, 127.61, 126.81, 126.03, 125.84 (2C), 123.62 (2C), 113.79 (2C), 100.71, 99.74, 77.46, 75.31, 73.67, 73.19, 71.51, 69.47, 69.21, 67.21, 67.10, 66.39, 64.13, 62.48, 55.27, 53.07, 50.44, 38.73, 20.94, 20.64, 20.55, 20.52; HRMS (ESI) *m/z* for $C_{51}H_{53}NO_{19}Na$ $[M+Na]^+$: calcd 1006.3109, found 1006.3117.

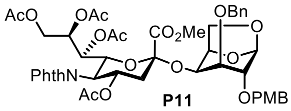

P11

Compound P11. Compound **P11** was synthesized from glycosyl acceptor **A10** (26 mg, 0.07 mmol) and glycosyl donor **D5** (53 mg, 0.07 mmol) by following **General glycosylation procedure B**. The mixture was purified by silica gel chromatography (EtOAc /Hexanes = 1/1, R_f = 0.23) to give compound **P11** (55 mg, 77%, α:β = 19:1). ^{1}H NMR (400 MHz, $CDCl_3$) δ 7.82-7.71 (m, 4H, ArH), 7.45-7.30 (m, 5H, ArH), 7.15 (d, *J* = 8.6 Hz, 2H, ArH), 6.80 (d, *J* = 8.6 Hz, 2H, ArH), 5.56-5.45 (m, 2H, H-4', H-8'), 5.31 (dd, *J* = 10.8, 2.0 Hz, 1H, H-6'), 5.21 (s, 1H, H-1), 5.14 (dd, *J* = 9.6, 2.0 Hz, 1H, H-7'), 4.84 (d, *J* = 12.0 Hz, 1H, $PhCH_2$), 4.62-4.57 (m, 2H, $PhCH_2$), 4.43 (d, *J* = 7.0 Hz, 1H, H-6), 4.26 (dd, *J* = 6.0, 4.2 Hz, 1H, H-5), 4.22-4.15 (m, 3H, H-9', H-4, H-5'), 4.12 (d, *J* = 12.3 Hz, 1H, $PhCH_2$), 4.01 (dd, *J* = 12.4, 6.1 Hz, 1H, H-9'), 3.96 (dd, *J* = 5.1, 1.0 Hz, 1H, H-3), 3.88 (s, 3H, CO_2Me), 3.76 (s, 3H, Ph-OMe), 3.59 (dd, *J* = 7.0, 6.0 Hz, 1H, H-6), 3.38 (s, 1H, H-2), 2.79 (dd, *J* = 13.0, 4.9 Hz, 1H, H-3'$_{eq}$), 2.18 (s, 3H, (CO)CH_3), 2.01 (s, 3H, (CO)CH_3), 1.97 (dd, *J* = 13.0, 12.4 Hz, 1H, H-3'$_{ax}$), 1.82 (s, 3H, (CO)CH_3), 1.81 (s, 3H, (CO)CH_3); ^{13}C NMR (100 MHz, $CDCl_3$) δ 170.50, 169.87, 169.71, 169.49, 167.88, 167.82, 167.58, 159.23, 138.88, 134.34 (2C), 131.34, 131.12, 129.92, 129.60 (2C), 128.31 (2C), 127.83 (2C), 127.57, 123.58 (2C), 113.69 (2C), 100.72, 99.73, 76.82, 75.80, 73.94, 73.13, 71.03, 69.53, 69.17, 67.10, 67.07, 66.34,

64.10, 62.40, 55.18, 53.04, 50.38, 38.63, 20.89, 20.52 (3C); HRMS (ESI) *m/z* for $C_{47}H_{51}NO_{19}Na$ $[M+Na]^+$: calcd 956.2953, found 956.2962.

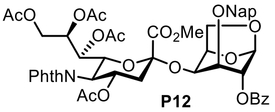

P12

Compound P12. Compound **P12** was synthesized from glycosyl acceptor **A11** (33 mg, 0.08 mmol) and glycosyl donor **D5** (60 mg, 0.08 mmol) by following **General glycosylation procedure B**. The mixture was purified by silica gel chromatography (EtOAc /Hexanes = 1/1, R_f = 0.39) to give compound **P12** (39 mg, 50%, α:β = 10:1). ^{1}H NMR (400 MHz, $CDCl_3$) δ 8.18-8.09 (m, 2H, ArH), 7.92-7.39 (m, 14H, ArH), 5.54-5.46 (m, 2H, H-4', H-2), 5.34-5.30 (m, 1H, H-8'), 5.23 (dd, *J* = 10.1, 1.8 Hz, 1H, H-6'), 5.17 (t, *J* = 1.5 Hz, 1H, H-1), 5.11-5.00 (m, 3H, H-7', $PhCH_2$), 4.57 (d, *J* = 7.1 Hz, 1H, H-6), 4.38 (m, 2H, H-5, H-9'), 4.21-4.16 (m, 2H, H-4, H-5'), 3.91 (s, 3H, CO_2Me), 3.83-3.79 (m, 2H, H-3, H-9'), 3.72-3.68 (m, 1H, H-6), 2.73 (dd, *J* = 13.1, 5.1 Hz, 1H, H-3'$_{eq}$), 2.09 (s, 3H, $(CO)CH_3$), 1.95-1.90 (m, 4H, H-3'$_{ax}$, $(CO)CH_3$), 1.75 (s, 3H, $(CO)CH_3$), 1.69 (s, 3H, $(CO)CH_3$); ^{13}C NMR (100 MHz, $CDCl_3$) δ 171.12, 170.38, 170.27, 169.78, 169.68, 169.65, 169.15, 168.24, 167.90, 167.71, 167.50, 165.80, 136.45, 134.36, 133.37, 133.16, 132.99, 131.42, 131.25, 130.18, 129.67, 128.24, 128.09, 127.98, 127.70, 126.01, 125.98, 125.76, 125.73, 123.68, 123.63, 123.60, 123.55, 99.69, 99.41, 78.11, 74.31, 73.36, 72.19, 69.24, 69.16, 67.06, 66.77, 66.46, 64.55, 61.82, 60.37, 53.09, 50.45, 38.70, 21.01, 20.88, 20.53, 20.49, 20.38; HRMS (ESI) *m/z* for $C_{50}H_{49}NO_{19}Na$ $[M+Na]^+$: calcd 990.2796, found 990.2795.

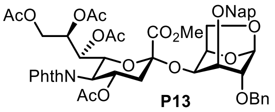

P13

Compound P13. Compound **P13** was synthesized from glycosyl acceptor **A12** (115 mg, 0.29 mmol) and glycosyl donor **D7** (384 mg, 0.59 mmol) by following the **General glycosylation procedure A**. The mixture was purified by silica gel chromatography

(EtOAc/Hexanes = 1/1, R_f = 0.30) to give compound **P13** (236 mg, 85%). ^{1}H NMR (600 MHz, $CDCl_3$) δ 7.86-7.82 (m, 6H), 7.73-7.72 (m, 2H), 7.58 (dd, *J* = 8.4, 1.5 Hz, 1H) 7.49-7.46 (m, 2H), 7.19-7.18 (m, 3H), 7.09-7.08 (m, 2H), 5.59 (ddd, *J* = 9.6, 6.3, 2.9 Hz, 1H, Sia-8), 5.50 (ddd, *J* = 11.9, 10.4, 4.9 Hz, 1H, Sia-4), 5.34 (dd, *J* = 10.8, 2.0 Hz, 1H, Sia-6), 5.28 (t, *J* = 1.4 Hz, 1H, Gal-1), 5.17 (dd, *J* = 9.6, 2.0 Hz, 1H, Sia-7), 4.99 (d, *J* = 12.0 Hz, 1H, C*H*$_2$Ph), 4.77 (d, *J* = 12.0 Hz, 1H, C*H*$_2$Ph), 4.63 (d, *J* = 12.7 Hz, 1H, C*H*$_2$Ph), 4.47 (d, *J* = 6.9 Hz, 1H, Gal-6), 4.31 (t, *J* = 5.3 Hz, 1H, Gal-5), 4.25-4.21 (m ,3H, Gal-4, Sia-5, Sia-9), 4.19 (d, *J* = 12.7 Hz, 1H, C*H*$_2$Ph), 4.07 (dd, *J* = 12.4, 6.3 Hz, 1H, Sia-9), 4.05 (dd, *J* = 5.3, 1.4 Hz, 1H, Gal-3), 3.90 (s, 3H, OMe), 3.62 (t, *J* = 5.3 Hz, 1H, Gal-6), 3.42 (t, *J* = 1.4 Hz, 1H, Gal-2), 2.79 (dd, *J* = 12.9, 4.9 Hz, 1H, Sia-3$_{eq}$), 2.19 (s, 3H, Ac), 2.02 (s, 3H, Ac), 1.99 (dd, *J* = 12.9, 11.9 Hz, 1H, Sia-3$_{ax}$), 1.82 (s, 3H, Ac), 1.80 (s, 3H, Ac); ^{13}C NMR (150 MHz, $CDCl_3$) δ 170.56, 169.91, 169.73, 169.54, 167.95, 167.86, 167.61, 137.82, 136.37, 134.44, 134.36, 133.29, 133.01, 131.41, 131.17, 128.25 (x2), 128.14, 127.84 (x3), 127.71, 127.64, 126.44, 126.17 (x2), 125.88, 123.65 (x2), 100.71, 99.78, 77.21, 76.18, 74.24, 73.24, 71.44, 69.48, 69.26, 67.18, 67.15, 66.38, 64.19, 62.50, 53.09, 50.44, 38.62, 20.93, 20.63, 20.55 (x2). HRMS (ESI) *m/z* for $C_{50}H_{51}NO_{18}Na$ [M+Na]$^+$: calcd 976.3004, found 976.2994.

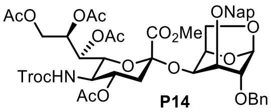

P14

Compound P14. Compound **P14** was synthesized from glycosyl acceptor **A12** (2.67 g, 6.80 mmol) and glycosyl donor **D8** (1.76 g, 2.40 mmol) by following the **General glycosylation procedure A**. The mixture was purified by silica gel chromatography (EtOAc/Hexanes = 2/3, R_f = 0.30) to afford compound **P14** (2.36 g, 98%). ^{1}H NMR (500 MHz, $CDCl_3$) δ 7.84-7.81 (m, 4H), 7.53 (d, *J* = 8.5 H, 1H), 7.48-7.46 (m, 2H), 7.20-7.19 (m, 3H), 7.11-7.10 (m, 2H), 5.56-5.53 (m, 1H, B-8), 5.39 (d, *J* = 9.7 Hz, 1H, B-7), 5.25 (br, 1H, A-1), 5.01-4.95 (m, 2H, B-4, B$_5$-NH), 4.89 (d, *J* = 10.4 Hz, 2H),

4.72 (d, J = 11.9 Hz, 1H), 4.65 (d, J = 12.7 Hz, 1H), 4.47 (d, J = 12.7 Hz, 1H), 4.46-4.42 (m, 2H, A-6, B-6), 4.30-4.28 (m, 2H, A-5, B-9), 4.20 (d, J = 12.7 Hz, 1H), 4.15 (dd, J = 12.4, 5.9 Hz, 1H, B-9), 4.11 (dd, J = 4.6, 4.6 Hz, 1H, A-4), 4.02 (d, J = 4.6 Hz, 1H, A-3), 3.82 (s, 3H, OCH_3), 3.68 (ddd, J = 10.4, 10.4, 10.4 Hz, 1H, B-5), 3.60 (dd, J = 7.3, 4.6 Hz, 1H, A-6), 3.41 (br, 1H, A-2), 2.63 (dd, J = 13.0, 4.4 Hz, 1H, B-3_{eq}), 2.15 (s, 3H, Ac), 1.99 (s, 3H, Ac), 1.97 (s, 3H, Ac), 1.94-1.91 (m, 1H, B-3_{ax}), 1.91 (s, 3H, Ac); ^{13}C NMR (125 MHz, $CDCl_3$) δ 170.63, 170.36, 170.01, 169.30, 168.60, 154.12, 137.81, 136.36, 133.27, 133.00, 128.29, 128.15, 127.90, 127.82, 127.71, 127.70, 126.28, 126.19, 126.03, 125.89, 100.73, 99.81, 95.32, 77.18, 76.40, 74.56, 74.32, 73.20, 72.54, 71.50, 70.00, 68.15, 67.48, 67.40, 64.19, 62.62, 53.05, 51.70, 38.42, 20.87, 20.80, 20.67, 20.62; HRMS (ESI) *m/z* for $C_{45}H_{49}Cl_3NO_{18}$ $[M-H]^-$: calcd 996.2015, found 996.2018.

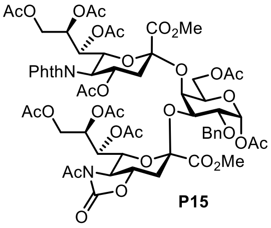

P15

Compound P15. Compound **P15** was synthesized from glycosyl acceptor **A13** (50 mg, 54.6 μmol) and glycosyl donor **D4** (364 mg, 545.9 μmol) by following the **General glycosylation procedure B**. The mixture was purified by silica gel chromatography (EtOAc/Hexanes = 1/1 + 5% MeOH, R_f = 0.37) to give compound **P15** (40 mg, 53%). ^1H NMR (600 MHz, d_6-Acetone) δ 7.90-7.86 (m, 4H), 7.32-7.30 (m, 5H), 6.29 (d, J = 3.3 Hz, 1H, Gla-1), 5.84 (dd, J = 7.0, 1.8 Hz, 1H, Sia_3-7), 5.70 (ddd, J = 8.1, 7.0, 2.6 Hz, 1H, Sia_3-8), 5.59 (td, J = 10.9, 4.9 Hz, 1H, Sia_4-4), 5.42 (ddd, J = 8.9, 4.3, 3.0 Hz, 1H, Sia_4-8), 5.13, (dd, J = 8.9, 1.8 Hz, 1H, Sia_4-7), 5.03 (dd, J = 10.5, 2.5 Hz, 1H, Gal-3), 4.87 (dd, J = 10.9, 1.8, Sia_4-6), 4.72-4.68 (m, 2H, Sia_3-6, Sia3-4), 4.53 (br, 3H, CH_2Ph, Gal-4), 4.49 (dd, J = 12.1, 2.6 Hz, 1H, Sia_3-9), 4.46 (dd, 11.7, 4.7 Hz, 1H, Gal-

6), 4.33 (dd, J = 7.6, 4.7 Hz, 1H, Gal-5), 4.22 (dd, J = 12.6, 3.0 Hz, 1H, Sia_4-9), 4.19 (dd, J = 11.7, 7.6 Hz, 1H, Gal-6), 4.10 (dd, J = 12.6, 4.3 Hz, 1H, Sia_4-9), 4.08 (t, J = 10.9 Hz, 1H, Sia_4-5), 4.05 (dd, J = 12.1, 8.1 Hz, 1H, Sia_3-9), 3.98 (dd, J = 11.5, 9.3 Hz, 1H, Sia_3-5), 3.88 (dd, J = 10.5, 3.3 Hz, 1H, Gal-2), 3.84 (s, 3H, OMe), 3.69 (s, 3H, OMe), 2.89 (dd, J = 12.8, 4.9 Hz, 1H, Sia_4-3_{eq}), 2.88 (t, J = 12.8 Hz, 1H, Sia_4-3_{ax}), 2.70 (dd, J = 12.7, 4.7 Hz, 1H, Sia_3-3_{eq}), 2.46 (s, 3H, Ac), 2.26 (dd, J = 12.7, 11.6 Hz, 1H, Sia_3-3_{ax}), 2.19 (s, 3H, Ac), 2.18 (s, 3H, Ac), 2.08 (s, 3H, Ac), 2.08 (s, 3H, Ac), 2.06 (s, 3H, Ac), 2.04 (s, 3H, Ac), 2.01 (s, 3H, Ac), 1.84 (s, 3H, Ac), 1.80 (s, 3H, Ac); ^{13}C NMR (150 MHz, $CDCl_3$) δ 172.33, 170.51 (x2), 170.44, 170.29, 169.94, 169.61, 169.55, 169.48, 169.13, 167.73, 167.58, 167.22, 166.61, 153.42, 136.99, 134.45, 134.35, 131.31, 131.20, 128.93 (x2), 128.25 (x2), 128.07, 123.60 (x2), 100.25, 99.07, 90.38, 76.41, 73.62, 73.33, 73.27, 73.08, 71.52, 70.82, 70.35, 69.16, 69.16, 68.31, 67.49, 66.71, 63.46, 63.03, 61.52, 58.47, 53.16, 52.36, 50.51, 36.59, 36.06, 29.63, 24.48, 20.91, 20.83, 20.78 (x2), 20.68, 20.60, 20.42, 20.40; HRMS (ESI) *m/z* for $C_{62}H_{72}N_2O_{33}Na$ $[M+Na]^+$: calcd 1395.3915, found 1395.3924.

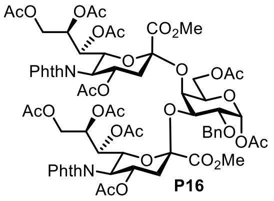

P16

Compound P16. Compound **P16** was synthesized from glycosyl acceptor **A13** (78 mg, 85.2 μmol) and glycosyl donor **D7** (124 mg, 170.3 μmol) by following the **General glycosylation procedure A**. The mixture was purified by silica gel chromatography (EtOAc/Hexanes = 1/1 + 5% MeOH, R_f = 0.12) to give compound **P16** (11 mg, 9%). ^1H NMR (600 MHz, d_6-Acetone) δ 7.93 (br, 2H), 7.89 (br, 2H), 7.40-7.34 (m, 4H), 7.31-7.29 (m, 1H), 6.22 (d, J = 3.3 Hz, 1H, Gal-1), 5.76 (td, J = 10.8, 4.9 Hz, 1H, Sia_4-4), 5.69 (td, J = 10.5, 5.1 Hz, 1H, Sia_3-4), 5.53 (ddd, J = 9.4, 4.2, 2.9 Hz, 1H, Sia_4-8),

5.44 (dd, J = 10.5, 2.4 Hz, 1H, Sia$_3$-6), 5.35 (t, J = 2.4 Hz, 1H, Sia$_3$-7), 5.29 (dt, J = 8.8, 2.4 Hz, 1H, Sia$_3$-8), 5.18 (dd, J = 9.4, 1.3 Hz, 1H, Sia$_4$-7), 5.09 (dd, J = 11.0, 1.3 Hz, 1H, Sia$_4$-6), 4.87 (dd, J = 12.2, 2.4 Hz, 1H, Sia$_3$-9), 4.77-4.75 (m ,2H, Gal-3, Gal-4), 4.61 (d, J = 12.6 Hz, 1H, CH_2Ph), 4.58-4.53 (m, 2H, Sia$_3$-9, Sia$_4$-5), 4.52 (d, J = 12.6 Hz, 1H, CH_2Ph), 4.36 (t, J = 10.5 Hz, 1H, Sia$_3$-5), 4.32-4.27 (m, 3H, Gal-5, Gal-6), 4.23 (dd, J = 12.7, 2.9 Hz, 1H, Sia$_4$-9), 4.18 (dd, J = 12.7, 4.2 Hz, 1H, Sia$_4$-9), 4.12 (dd, 10.5, 3.3 Hz, 1H, Gal-2), 3.00 (dd, J = 12.1, 4.9 Hz, 1H, Sia$_4$-3$_{eq}$), 2.96 (dd, J = 14.0, 5.1 Hz, 1H, Sia$_3$-3$_{eq}$), 2.62 (t, J = 12.1 Hz, 1H, Sia4-3$_{ax}$), 2.16 (s, 3H, Ac), 2.13 (s, 3H, Ac), 2.10 (s, 3H, Ac), 2.08 (s, 3H, Ac), 2.06 (s, 3H, Ac), 2.06-2.04 (m, 4H, Sia$_3$-3$_{ax}$, Ac), 2.01 (s, 3H, Ac), 1.96 (s, 3H, Ac), 1.82 (s, 3H, Ac), 1.81 (s, 3H, Ac); ^{13}C NMR (150 MHz, d_6-Acetone) δ 171.07, 170.95, 170.85, 170.77, 170.63, 170.56, 170.32, 170.25, 169.94, 169.76, 168.81 (x2), 168.37, 168.19 (x2), 167.75, 138.77, 135.72 (x2), 135.59, 135.45, 132.78, 132.40 (x3), 129.38 (x2), 129.12 (x2), 128.66, 124.15 (x4), 100.68 (x2), 91.17, 74.49. 73.89, 73.46, 73.05, 73.00, 71.23, 70.31, 70.22, 69.61, 69.21, 68.63, 68.02, 67.43, 64.57, 63.13, 62.58, 53.57, 52.93, 51.49, 51.28, 38.82, 36.40, 21.14, 21.02, 20.93, 20.82 (x2), 20.80, 20.64 (x2), 20.51; HRMS (ESI) m/z for $C_{69}H_{76}N_2O_{34}Na$ $[M+Na]^+$: calcd 1499.4177, found 1499.4157.

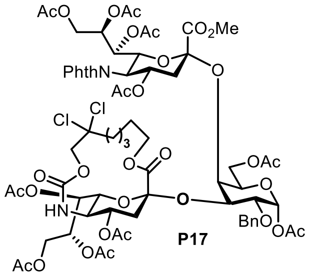

P17

Compound P17. Compound **P17** was synthesized from glycosyl acceptor **A13** (80 mg, 87.4 μmol) and glycosyl donor **D9** (384 mg, 874.0 μmol) by following the **General glycosylation procedure A**. The mixture was purified by silica gel chromatography (EtOAc/Hexanes = 1/1 + 5% MeOH, R$_f$ = 0.39) to give compound **P17** (45 mg, 33%).^{1}H NMR (600 MHz, 80℃, CD_3NO_2) δ 7.85-7.82 (m, 4H), 7.41-7.38 (m, 2H), 7.36-7.34

(m, 2H), 7.30-7.27 (m, 1H), 6.29 (d, *J* = 3.7 Hz, 1H, Gal-1), 5.59-5.53 (m, 4H, Sia_3-4, Sia_3-8, Sia_4-4, NH), 5.45-5.42 (m, 2H, Sia_3-7, Sia_4-8), 5.13 (dd, *J* =9.3, 1.4 Hz, 1H, Sia_4-4-7), 4.97 (dd, *J* = 11.0, 1.4 Hz, 1H, Sia_4-4-6), 4.88 (br, 1H, CO_2CH_2), 4.81 (d, *J* = 12.6 Hz, 1H, C*H*$_2$Ph), 4.67 (d, *J* = 12.6 Hz, 1H, C*H*$_2$Ph), 4.61 (br, 2H, Gal-3, NH(CO)OC*H*$_2$), 4.49 (dd, *J* = 11.7, 3.3 Hz, 1H, Gal-6), 4.38 (dd, *J* = 12.7, 2.8 Hz, 1H, Sia_3-9), 4.37 (d, *J* = 1.9 Hz, 1H, Gal-4), 4.33-4.31 (m, 1H, Sia_4-5), 4.25-4.15 (m, 6H, Gal-5, Gal-6, Sia_3-9, Sia_4-9, CO_2CH_2, NH(CO)OC*H*$_2$), 4.03 (dd, *J* = 12.6, 4.5 Hz, 1H, Sia_4-9), 3.93 (dd, *J* = 10.2, 3.7 Hz, 1H, Gal-2), 3.88 (m, 3H, OMe), 3.00 (td, *J* = 10.5, 6.7 Hz, 1H, Sia_3-5), 2.80 (dd, *J* = 12.9, 4.7 Hz, 1H, Sia_3-3_{eq}), 2.64 (dd, *J* = 13.1, 4.7 Hz, 1H, Sia_3-3_{eq}), 2.48 (br, 1H, Cl_2CCH_2), 2.32-2.27 (m, 1H, Cl_2CCH_2), 2.21-2.15 (m, 7H, Sia_4-3_{ax}, Ac), 2.12 (s, 3H, Ac), 2.09 (s, 3H, Ac), 2.03 (s, 3H, Ac), 1.99 (s, 3H, Ac), 1.98 (s, 3H, Ac), 1.98 (s, 3H, Ac), 1.92-1.90 (m, 1H, Sia3-3_{ax}), 1.86 (s, 3H, Ac), 1.80 (s, 3H, Ac), 1.74 (br, 3H, C*H*$_2$), 1.64 (br, 3H, C*H*$_2$); ^{13}C NMR (150 MHz, 80℃, CD_3NO_2) δ 166.95, 166.74 (x2), 166.62, 166.29, 166.21, 166.12, 166.05, 165.96, 165.72, 164.30, 163.99, 163.82, 163.66, 150.36, 135.09, 130.62 (x2), 127.53 (x2), 124.23 (x2), 123.69 (x2), 123.46, 119.20 (x2), 95.72, 94.32, 86.91, 86.06, 69.23, 69.06, 68.41, 67.70, 67.60, 66.42, 65.85, 64.99, 64.57, 64.34, 64.29, 63.48, 62.95, 60.93, 60.65, 57.99, 57.86, 48.59, 46.59, 40.76, 33.71, 32.35, 25.30, 22.81, 21.82, 20.00, 18.48, 16.11,15.87, 15.76, 15.59 (x5), 15.42, 15.18; HRMS (ESI) *m/z* for $C_{68}H_{82}Cl_2N_2O_{34}Na$ [M+Na]$^+$: calcd 1563.4024, found 1563.4026.

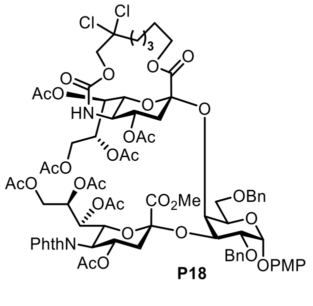

P18

Compound P18. Compound **P18** was synthesized from glycosyl acceptor **A14** (53 mg, 51.6 μmol) glycosyl donor **D9** (77 mg, 103.2 μmol) by following the **General glycosylation procedure A**. The mixture was purified by silica gel chromatography (EtOAc/Hexanes = 1/1, R_f = 0.23) to give compound **P18** (8 mg. 9%). ^{1}H NMR (600 MHz, 80℃, CD_3NO_2) δ 7.83-7.81 (m, 4H), 7.47-7.45 (m, 2H), 7.38-7.25 (m, 8H), 7.20-7.17 (m, 2H), 6.83-6.80 (m, 2H), 5.54 (d, *J* = 3.5 Hz, 1H, Gal-1), 5.51-5.40 (m, 6H, Sia_3-4, Sia_3-8, Sia_4-4, Sia_4-7, Sia_4-8, NH), 5.22 (dd, *J* = 8.0, 1.9 Hz, 1H, Sia_3-7), 5.17 (dd, *J* = 10.7, 1.9 Hz, 1H, Sia_3-6), 4.92 (dd, *J* = 10.4, 2.5 Hz, 1H, Gal-3), 4.80 (d, *J* = 12.1 Hz, 1H, C*H$_2$*Ph), 4.77 (d, *J* = 12.1 Hz, 1H, C*H$_2$*Ph), 4.74 (br, 1H, CO_2C*H$_2$*), 4.64 (d, *J* = 11.7 Hz, 1H, C*H$_2$*Ph), 4.55 (d, *J* = 11,7 Hz, 1H, C*H$_2$*Ph), 4.42-4.37 (m, 4H, Gal-4, Sia_3-9, Sia_4-6, Sia_4-9), 4.35-4.32 (m, 2H, Gal-5, NH(CO)OC*H$_2$*), 4.27 (t, *J* = 10.7 Hz, 1H, Sia_3-5), 4.20-4.17 (m, 2H, Sia_4-9, NH(CO)OC*H$_2$*), 4.09 (dd, *J* = 12.5, 5.1 Hz, 1H, Sia_3-9), 4.05-4.02 (m ,1H, CO_2C*H$_2$*), 4.00-3.96 (m ,4H, Gal-2, OMe), 3.91 (dd, *J* = 11.2, 2.8 Hz, 1H, Gal-6), 3.87 (dd, *J* = 11.2, 8.6 Hz, 1H, Gal-6), 3.75 (s, 3H, OMe), 3.28 (td, *J* = 10.5, 6.8 Hz, 1H, Sia_4-5), 2.75 (dd, *J* = 13.3, 4.7 Hz, 1H, Sia_3-3_{eq}), 2.71 (dd, *J* = 13.1, 5.0 Hz, 1H, Sia_4-3_{eq}), 2.48 (br, 1H, Cl_2CC*H$_2$*), 2.35-2.26 (m, 2H, Sia_3-3_{ax}, Cl_2CC*H$_2$*), 2.15 (s, 3H, Ac), 2.14 (s, 3H, Ac), 2.12 (s, 3H, Ac), 2.12 (s, 3H, Ac), 2.05-2.02 (m, 4H, Sia4-3_{ax}), 1.96 (s, 3H, Ac), 1.81-1.75 (m, 8H, Ac, C*H$_2$*), 1.66 (br, 4H, C*H$_2$*); ^{13}C NMR (150 MHz, 80℃, CD_3NO_2) δ 167.14, 166.67 (x2), 166.54, 166.31, 166.03 (x3), 164.21, 164.00 (x2), 163.57, 151.58, 150.38, 147.35, 135.38, 134.83, 130.65 (x2), 127.49 (x2), 124.29 (x2), 124.19 (x2), 123.99 (x2), 123.57, 123.24 (x3), 119.23 (x2), 115.48 (x2), 110.51 (x2), 95.50 (x2), 93.71, 86.17, 70.17, 69.71, 68.85, 68.60, 68.03, 67.47, 67.28, 66.39, 65.66, 65.53, 64.72, 64.43, 64.10, 63.76, 62.44, 60.78, 58.31, 57.87, 51.03, 48.62, 48.21, 46.50, 40.93, 33.76, 31.78, 25.28, 22.77, 20.02, 18.48, 16.05 (x2), 15.96, 15.88, 15.66, 15.32 (x3); HRMS (ESI) *m/z* for $C_{78}H_{90}Cl_2N_2O_{33}Na$ $[M+Na]^+$: calcd 1675.4701, found 1675.4683.

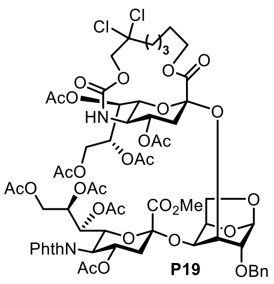

P19

Compound P19. Compound **P19** was synthesized from glycosyl acceptor **A15** (53 mg, 65.1 μmol) and glycosyl donor **D9** (384 mg, 130.2 μmol) by following the **General glycosylation procedure A**. The mixture was purified by silica gel chromatography (EtOAc/Hexanes = 1/1 + 2.5 % MeOH, R_f = 0.32) to give compound **P19** (70 mg, 75%). ^{1}H NMR (600 MHz, 80℃, CD_3NO_2) δ 7.84-7.81 (m, 4H), 7.49 (t, *J* = 7.6 Hz, 2H), 7.39 (dd, *J* = 7.6, 7.6 Hz, 2H), 7.31 (dd, *J* = 7.6, 7.6 Hz, 1H), 5.62 (td, *J* = 10.9, 5.2 Hz, 1H, Sia_4-4), 5.54-5,50 (m, 2H, Sia_3-4, NH), 5.43-5.39 (m ,2H, Sia_3-7, Sia_3-8), 5.34 (br, 1H, Gal-1), 5.30 (ddd, *J* = 7.8, 5.5, 3.3 Hz, 1H, Sia_4-8), 5.13 (dd, *J* = 7.8, 1.8 Hz, 1H, Sia_4-7), 4.93 (d, *J* = 11.6 Hz, 1H, C*H*$_2$Ph), 4.90 (dd, *J* = 10.7, 1.8 Hz, 1H, Sia_4-6), 4.72 (d, *J* = 11.6 Hz, 1H, C*H*$_2$Ph), 4.69 (br, 3H, Gal-3, NH(CO)OC*H*$_2$, CO_2C*H*$_2$), 4.51 (t, *J* = 4.4 Hz, 1H, Gal-4), 4.47 (t, *J* = 4.4 Hz, 1H, Gal-5), 4.33-4.23 (m, 6H, Gal-6, Sia_3-6, Sia_3-9, Sia_4-5, Sia_4-9, NH(CO)OC*H*$_2$), 4.18-4.17 (m, 1H, CO_2C*H*$_2$), 4.09-4.05 (m, 2H, Sia_3-9, Sia_4-9), 3.94 (s, 3H, OMe), 3.84 (br , 1H, Gal-2), 3.55 (dd, *J* = 6.3, 4.5 Hz, 1H, Gal-6), 3.04 (td, *J* = 10.4, 6.7 Hz, 1H, Sia_3-5), 2.99 (br, 1H, Sia_3-3_{eq}), 2.82 (dd, *J* = 13.2, 5.2 Hz, 1H, Sia_4-3_{eq}), 2.46 (br, 1H, Cl_2CC*H*$_2$), 2.34-2.29 (m, 1H, Cl_2CC*H*$_2$), 2.14 (s, 3H, Ac), 2.12 (s, 3H, Ac), 2.10 (s, 3H, Ac), 2.05 (s, 6H, Ac), 2.00-1.96 (m, 3H, Sia_4-3_{ax}, C*H*$_2$), 1.89-1.78 (m, 12H, Sia_3-3_{ax}, Ac, C*H*$_2$), 1.64 (br, 2H, C*H*$_2$); ^{13}C NMR (150 MHz, 80℃, CD_3NO_2) δ 166.82, 166.55 (x2), 166.37, 166.16, 166.12, 165.90, 165.85, 164.31, 163.98 (x2), 163.05, 150.45, 134.91, 130.64 (x2), 127.50 (x2), 124.32 (x2), 123.54 (x3), 119.22 (x2), 96.06, 95.65, 95.49, 88.11, 76.73, 70.04, 68.64 (x2), 67.86, 66.02, 65.48, 64.83, 64.75, 64.56, 64.06, 63.41, 62.86, 62.80, 60.69, 59.86, 57.76, 57.62, 57.53, 49.02, 48.65, 46.48, 40.76, 34.56, 32.16, 22.64, 19.75, 18.51, 16.11, 15.78, 15.60 (x3), 15.37,

15.28, 15.22; HRMS (ESI) *m/z* for $C_{64}H_{76}Cl_2N_2O_{31}Na$ $[M+Na]^+$: calcd 1461.3707, found 1461.3696.

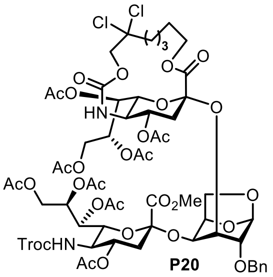

P20

Compound P20. Compound **P20** was synthesized from glycosyl acceptor **A16** (387 mg, 0.45 mmol) and glycosyl donor **D9** (677 mg, 0.90 mmol) by following the **General glycosylation procedure A**. The mixture was purified by silica gel chromatography (EtOAc/Hexanes = 1/1, twice, R_f = 0.18) to afford compound **P20** (379 mg, 57%, brsm: 98%). ^1H NMR (850 MHz, CD_3NO_2, 50 °C) δ 7.51 (d, *J* = 7.5 Hz, 2H), 7.41 (t, *J* = 7.5 Hz, 2H), 7.34 (t, *J* = 7.5 Hz, 1H), 5.62 (d, *J* = 10.1 Hz, 1H, B_5-NH), 5.53 (br, 1H, C-4), 5.45-5.44 (m, 2H, C_5-NH, C-8), 5.39 (d, *J* = 7.3 Hz, 1H, B-7), 5.36 (br, 1H, A-1), 5.31 (br, 1H, B-8), 5.17 (br, 1H, B-4), 4.94 (d, *J* = 11.5 Hz, 1H, A_2-Bn), 4.88 (d, *J* = 12.2 Hz, 1H, Troc), 4.74 (d, *J* = 11.5 Hz, 1H, A_2-Bn), 4.73-4.65 (m, 2H, $NHCO_2CH_2$, $CO_2CH_2CH_2$), 4.66 (d, *J* = 12.2 Hz, 1H, Troc), 4.49 (br, 1H, A-5), 4.45 (br, 1H, A-3), 4.36-4.32 (m, 6H, A-4, A-6, B-9, C-6, C-7, $CO_2CH_2CH_2$), 4.29 (d, *J* = 12.3 Hz, 1H, C-9), 4.16-4.14 (m, 2H, B-9, $NHCO_2CH_2$), 4.10 (d, *J* = 12.3 Hz, 1H, C-9), 4.06 (d, *J* = 10.1 Hz, 1H, B-6), 3.88 (s, 3H, OCH_3), 3.85 (br, 1H, B-2), 3.69 (ddd, *J* = 10.1, 10.1, 10.1 Hz, 1H, B-5), 3.57 (dd, *J* = 6.0, 6.0 Hz, 1H, A-6), 3.06 (ddd, *J* = 10.2, 10.2, 6.9 Hz, 1H, C-5), 2.99 (br, 1H, C-3_{eq}), 2.74 (dd, *J* = 12.9, 3.7 Hz, 1H, B-3_{eq}), 2.50 (br, 1H, $OCH_2CCl_2CH_2$), 2.34 (br, 1H, $OCH_2CCl_2CH_2$), 2.17 (s, 3H, Ac), 2.15 (s, 3H, Ac), 2.13 (s, 3H, Ac), 2.08-2.05 (m, 2H, CH_2), 2.08 (s, 6H, Ac(x2)), 2.05 (s, 3H, Ac), 1.98 (s, 3H, Ac), 1.94-1.91 (m, 1H, B-3_{ax}), 1.92 (s, 3H, Ac), 1.85-1.79 (m, 3H, C-3_{ax}, CH_2), 1.64 (br, 2H, CH_2); ^{13}C NMR (213 MHz, CD_3NO_2, 50 °C) δ 172.38, 172.26, 172.15, 171.95, 171.92 (x2), 171.75, 171.53, 171.44, 169.88, 168.57, 155.88, 140.42, 129.89, 129.11,

129.09, 101.54, 101.06, 100.91, 97.23, 91.59, 82.26, 75.77, 75.68, 74.17, 73.65, 73.28, 71.50, 70.59 (x2), 70.35, 69.99 (x2), 69.47, 69.30, 68.29, 66.11, 65.39, 63.43, 63.34, 54.47, 54.15, 53.01, 46.29, 40.06, 37.06, 28.13, 25.23, 24.02, 21.69, 21.34, 21.28, 21.19 (x3), 20.97, 20.87; HRMS (ESI) *m/z* for $C_{59}H_{74}Cl_5N_2O_{31}$ $[M-H]^-$: calcd 1481.2718, found 1481.2713.

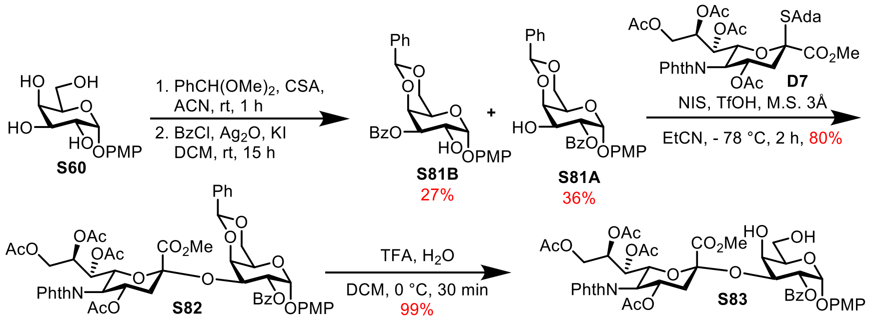

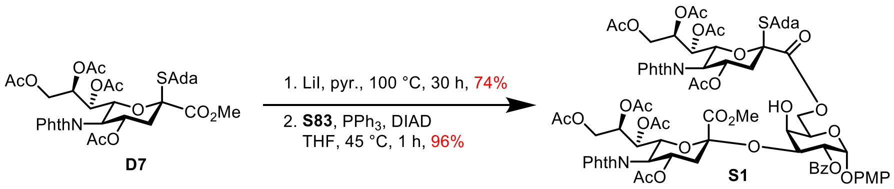

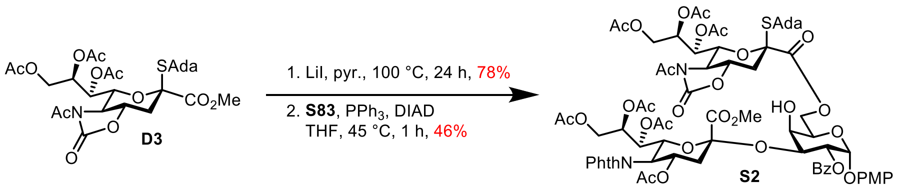

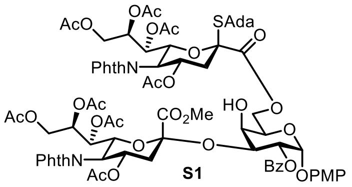

Compound S1. To a solution of compound **D7** (84 mg, 0.12 mmol) in pyridine (2 mL) was added LiI (77 mg, 0.58 mmol) at rt. The reaction mixture was stirred at 100 °C for 30 h and then concentrated *in vacuo*. The residue was extracted with EtOAc, and the resulting solution was washed with 1N $HCl_{(aq)}$ and brine. The organic layer was dried over $MgSO_4$, filtered, and concentrated. The residue was purified by silica gel chromatography (MeOH/DCM = 1/9, R_f = 0.13) to afford demethylated product (61 mg, 74%). Next, to a solution of demethylated product (61 mg, 85.1 μmol), compound **S83**

(27 mg, 28.4 μmol) and triphenylphosphine (22 mg, 85.1 μmol) in THF (2 mL) was slowly added a solution of diisopropyl azodicarboxylate (17 μL, 85.1 μmol) in THF (2 mL) at 45 °C for 30 min. After being stirred at 45 °C for 1 h, the reaction mixture was quenched with AcOH and MeOH, and then concentrated *in vacuo*. The residue was purified by silica gel chromatography (EtOAc/Hexanes = 1/1 + 5% MeOH, R_f = 0.24) to afford compound **S1** (45 mg, 96%). ^{1}H NMR (600 MHz, $CDCl_3$) δ 8.14 (d, *J* = 7.4 Hz, 2H), 7.84-7.80 (m, 4H), 7.71-7.70 (m ,4H), 7.56 (dd, *J* = 7.4, 7.4 Hz, 1H), 7.46 (dd, *J* = 7.4, 7.4 Hz, 2H), 7.08 (d, *J* = 9.0 Hz, 2H), 6.80 (d, *J* = 9.0 Hz, 2H), 5.87 (td, *J* = 10.8, 4.8 Hz, 1H, Sia_6-4), 5.70 (dd, *J* = 10.8, 2.3 Hz, 1H, Sia_6-6), 5.61 (d, *J* = 3.6 Hz, 1H, Gal-1), 5.55 (ddd, *J* = 9.0, 6.2, 2.5 Hz, 1H, Sia_3-8), 5.51-5.44 (m ,2H, Gal-2, Sia_3-4), 5.32 (dt, *J* = 8.0, 2.3 Hz, 1H, Sia_6-8), 5.27 (t, *J* = 2.3 Hz, 1H, Sia_6-7), 5.13 (dd, *J* = 9.0, 2.4 Hz, 1H, Sia_3-7), 5.04 (dd, *J* = 10.7, 2.4 Hz, 1H, Sia_3-6), 4.98 (dd, *J* = 10.2, 3.3 Hz, 1H, Gal-3), 4.88 (dd, *J* = 12.3, 2.3 Hz, 1H, Sia_6-9), 4.62 (dd, *J* = 11.5, 7.9 Hz, 1H, Gal-6), 4.45 (dd, *J* = 11.5, 4.0 Hz, 1H, Gal-6), 4.41 (dd, *J* = 7.9, 4.0 Hz, 1H, Gal-5), 4.34 (dd, *J* = 12.3, 8.0 Hz, 1H, Sia_6-9), 4.28 (dd, *J* = 12.4, 2.5 Hz, 1H, Sia_3-9), 4.19 (dd, *J* = 10.8, 10.8 Hz, 1H, Sia_6-5), 4.16 (dd, *J* = 10.7, 10.7 Hz, 1H, Sia_3-5), 4.03 (br, 1H, Gal-4), 3.98 (dd, *J* = 12.4, 6.2 Hz, 1H, Sia_3-9), 3.88 (s, 3H, OMe), 3.72 (s, 3H, OMe), 2.85 (br, 1H, OH), 2.69 (dd, *J* = 12.8, 5.0 Hz, 1H, Sia_3-3_{eq}), 2.56 (dd, *J* = 13.5, 4.8 Hz, 1H, Sia_6-3_{eq}), 2.12 (s, 3H, Ac), 2.07 (s, 3H, Ac), 2.03-1.99 (m, 7H, Sia_3-3_{ax}, SAda), 1.95-1.90 (m, 16H, Sia_6-3_{ax}, Ac, SAda), 1.79 (s, 3H, Ac), 1.79 (s, 3H, Ac), 1.67-1.61 (m, 6H, SAda); ^{13}C NMR (150 MHz, $CDCl_3$) δ 170.59, 170.37, 170.35, 169.99, 169.96, 169.88, 169.75, 169.66, 168.83, 167.89, 167.70 (x4), 165.75, 155.37, 150.93, 134.38 (x3), 134.23, 133.22, 131.31 (x2), 131.09 (x2), 129.92 (x2), 129.88, 128.43 (x2), 123.58 (x4), 118.60 (x2), 114.66 (x2), 97.99, 96.88, 85.76, 72.53, 70.48, 69.66, 69.05, 68.82, 68.64, 68.58, 68.01, 67.50, 66.92, 66.62, 66.35, 64.98, 63.23, 62.39, 55.59, 53.47, 51.04, 50.66, 50.11, 43.33 (x3), 39.99, 37.45, 35.88 (x3), 29.81 (x3), 21.15, 20.90, 20.63 (x2),

20.60, 20.54, 20.51 (x2); HRMS (ESI) *m/z* for $C_{81}H_{88}N_2O_{33}SNa$ $[M+Na]^+$: calcd 1671.4888, found 1671.4870.

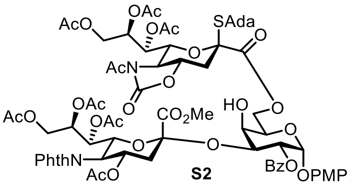

S2

Compound S2. To a solution of compound **D3** (197 mg, 0.31 mmol) in pyridine (3 mL) was added LiI (210 mg, 1.57 mmol) at rt. The reaction mixture was stirred at 100 °C for 24 h and then concentrated *in vacuo*. The residue was extracted with EtOAc, and the resulting solution was washed with 1N $HCl_{(aq)}$ and brine. The organic layer was dried over $MgSO_4$, filtered, and concentrated. The residue was purified by silica gel chromatography (MeOH/DCM = 1/9, R_f = 0.17) to afford demethylated product (150 mg, 78%). Next, to a solution of demethylated product (150 mg, 0.25 mmol), compound **S83** (78 mg, 81.7 μmol) and triphenylphosphine (22 mg, 0.25 mmol) in THF (5 mL) was slowly added a solution of diisopropyl azodicarboxylate (48 μL, 0.25 μmol) in THF (5 mL) at 45 °C for 30 min. After being stirred at 45 °C for 1 h, the reaction mixture was quenched with AcOH and MeOH, and then concentrated *in vacuo*. The residue was purified by silica gel chromatography (Acetone/Hexanes, R_f = 0.20) to afford compound **S2** (58 mg, 46%). ^{1}H NMR (500 MHz, $CDCl_3$) δ 8.12 (d, *J* = 7.7 Hz, 2H), 7.81 (br, 2H), 7.72 (br , 2H), 7.55 (dd, *J* = 7.7, 7.7 Hz, 1H), 7.45 (dd, *J* = 7.7, 7.7 Hz, 2H), 7.05 (d, *J* = 8.9 Hz, 2H), 6.80 (d, *J* = 8.9 Hz, 2H), 5.70-5.69 (m, 2H, Gal-1, Sia_6-7), 5.53-5.47 (m, 3H, Gal-2, Sia_3-4, Sia_3-8), 5.33-5.32 (m, 1H, Sia_6-8), 5.14 (dd, *J* = 9.1, 1.8 Hz, 1H, Sia_3-7), 5.03 (dd, *J* = 10.7, 1.8 Hz, 1H, Sia_3-6), 4.95 (dd, *J* = 10.2, 3.0 Hz, 1H, Gal-3), 4.80 (dd, *J* = 11.6, 7.8 Hz, 1H, Gal-6), 4.70-4.66 (m ,2H, Sia_6-5, Sia_6-5), 4.59 (td, *J* = 11.0, 3.4 Hz, 1H, Sia_6-4), 4.36 (d, *J* = 7.8 Hz, 1H, Gal-5), 4.28-4.24 (m, 3H, Gal-6, Sia_3-9, Sia_6-9), 4.18 (t, *J* = 10.7 Hz, 1H, Sia_3-5), 4.04 (br, 1H, Gal-4), 3.95 (dd, *J* = 12.4, 6.0 Hz, 1H, Sia_3-9), 3.87 (s, 3H, OMe), 3.72 (s, 3H, OMe), 3.67 (dd, *J* =

10.2. 10.2 Hz, 1H, Sia_6-9), 2.65 (dd, J = 12.8, 4.9 Hz, 1H, Sia_3-3_{eq}), 2.53 (dd, J = 12.8, 3.4 Hz, 1H, Sia_6-3_{eq}), 2.47 (s, 3H, Ac), 2.16-2.08 (m, 10H, Sia_6-3ax, Ac), 2.02-1.91 (m, 13H, Sia_3-3ax, Ac, SAda), 1.87-1.85 (m, 3H, SAda), 1.79 (s, 3H, Ac), 1.61 (br, 9H, SAda); ^{13}C NMR (125 MHz, $CDCl_3$) δ 172.26, 171.00, 170.56, 170.44, 169.98, 169.89, 169.63, 167.92, 167.81, 167.79, 167.68, 165.70, 155.20, 153.67, 150.43, 134.47, 134.42, 131.26, 131.05, 129.87 (x2), 129.74, 128.40 (x2), 123.59 (x2), 117.95 (x2), 114.69 (x2), 98.19, 95.82, 85.21, 74.92, 74.17, 72.98, 72.11, 70.40, 69.03, 68.95, 68.65, 68.32, 67.39, 66.82, 66.61, 65.40, 63.07, 62.37, 60.08, 55.60, 53.32, 51.34, 50.04, 43.38 (x3), 38.27, 37.18, 35.79 (x3), 29.76 (x3), 24.65, 21.17, 21.12, 20.72, 20.65, 20.58, 20.56, 20.49; HRMS (ESI) *m/z* for $C_{74}H_{84}N_2O_{32}SNa$ $[M+Na]^+$: calcd 1567.4626, found 1567.4645.

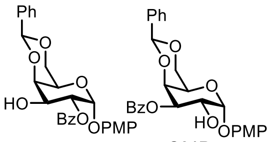

S81A **S81B**

Compound 81A and 81B. To a solution of compound **S60** (500 mg, 1.75 mmol) in ACN (20 mL) was added camphor sulfonic acid (203 mg, 0.87 mmol) and benzaldehyde dimethyl acetal (524 μL, 3.49 mmol) at rt under a nitrogen atmosphere. After being stirred at rt for 1 h, the reaction mixture was quenched with Et_3N and concentrated *in vacuo*. Next, to a solution of above residue, KI (58 mg, 0.35 mmol), and Ag_2O (608 mg, 2.63 mmol) in dry DCM (20 mL) was added benzoyl chloride (224 μL, 1.93 mmol) at 0 °C under a nitrogen atmosphere. The reaction mixture was gradually warmed to rt and stirred for 15 h. The solid was filtered through a pad of Celite®, and the resulting solution was concentrated under reduced pressure. The residue was dissolved in EtOAc and the resulting organic solution was washed with saturated $NaHCO_{3(aq)}$, H_2O, and brine. The organic layer was dried over $MgSO_4$, filtered, and concentrated. The residue was purified by silica gel chromatography

(EtOAc/Hexanes = 1/1 R_f = 0.39 and 0.50 for compound **81A** and **81B**, respectively) to afford compound **81A** (300 mg, 36% over two steps) and compound **81B** (226 mg, 27% over two steps). For compound **81A**: ^{1}H NMR (500 MHz, $CDCl_3$) δ 8.07 (d, *J* = 7. 5 Hz, 1H), 7.54 (d, *J* = 6.9 Hz, 1H), 7.44-7.38 (m, 5H), 7.02 (d, *J* = 9.0 Hz, 2H), 6.80 (d, *J* = 9.0 Hz, 2H), 5.78 (d, *J* = 3.4 Hz, 1H, H-1), 5.61 (s, 1H, PhC*H*), 5.51 (dd, *J* = 10.4, 3.4 Hz, 1H, H-2), 4.49 (td, *J* = 10.4, 3.3 Hz, 1H, H-3), 4.41 (d, *J* = 3.3 Hz, 1H, H-4), 4.29 (d, *J* = 12.6 Hz, 1H, H-6), 4.09 (d, *J* = 12.6 Hz, 1H, H-6), 3.96 (s, 1H, H-5), 3.74 (s, 3H, OMe), 2.55 (d, *J* = 10.4 Hz, 1H, OH); ^{13}C NMR (125 MHz, $CDCl_3$) δ 166.43, 155.05, 150.73, 137.24, 133.20, 129.79 (x2), 129.50, 129.23, 128.31 (x2), 128.24 (x2), 126.22 (x2), 117.59 (x2), 114.59 (x2), 101.24, 96.70, 75.93, 71.33, 69.03, 67.26, 63.20, 55.54; HRMS (ESI) *m/z* for $C_{27}H_{26}O_8Na$ $[M+Na]^+$: calcd 501.1525, found 501.1524. For compound **81B**: ^{1}H NMR (500 MHz, $CDCl_3$) δ 8.11 (d, *J* = 7.6 Hz, 1H), 7.57 (t, *J* = 7.6 Hz, 1H), 7.51 (d, *J* = 6.8 Hz, 1H), 7.44 (t, *J* = 7.6 Hz, 1H), 7.38-7.34 (m, 3H), 7.07 (d, *J* = 8.9 Hz, 2H), 6.84 (d, *J* = 8.9 Hz, 2H), 5.71 (d, *J* = 3.5 Hz, 1H, H-1), 5.61 (dd, *J* = 10.4, 3.3 Hz, 1H, H-3), 5.55 (s, 1H, PhC*H*), 4.55 (d, *J* = 3.3 Hz, 1H, H-4), 4.51-4.48 (m, 1H, H-2), 4.27 (d, *J* = 12.6 Hz, 1H, H-6), 4.06 (d, *J* = 12.6 Hz, 1H, H-6), 3.94 (s, 1H, H-5), 3.77 (s, 3H, OMe), 2.15 (d, *J* = 10.0 Hz, 1H, OH); ^{13}C NMR (100 MHz, $CDCl_3$) δ 166.88, 155.20, 150.42, 137.54, 133.36, 129.96 (x2), 129.67, 128.92 128.42 (x2), 128.14 (x2), 117.42 (x2), 114.73 (x2), 100.56, 98.53, 74.32, 71.95, 69.11, 66.94, 63.32, 56.65; HRMS (ESI) *m/z* for $C_{27}H_{26}O_8Na$ $[M+Na]^+$: calcd 501.1525, found 501.1541.

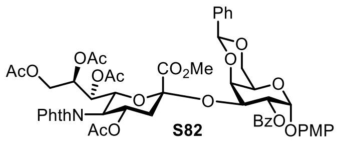

S82

Compound S82. Compound **S82** was synthesized from glycosyl acceptor **S81A** (244 mg, 0.51 mmol) and glycosyl donor **D7** (744 mg, 1.02 mmol) by following the **General glycosylation procedure A**. The mixture was purified by silica gel chromatography

(EtOAc/Hexanes = 1/1, R_f = 0.23) to give compound **S82** (426 mg, 80%). ^{1}H NMR (600 MHz, $CDCl_3$) δ 8.17-8.15 (m, 2H), 7.82-7.81 (m, 2H), 7.72-7.71 (m, 2H), 7.60-7.57 (m, 1H), 7.56-7.54 (m, 2H), 7.52-7.49 (m, 2H), 7.37-7.32 (m, 3H), 7.08-7.05 (m, 2H), 6.79-6.77 (m, 2H), 5.89 (d, *J* = 3.6 Hz, 1H, Gal-1), 5.62-5.58 (m, 2H, Gal-2, Sia-8), 5.51 (s, 1H. PhC*H*), 5.45 (ddd, *J* = 11.8, 10.4, 4.9 Hz, 1H, Sia-4), 5.24-5.20 (m, 2H, Gal-3, Sia-7), 5.04 (dd, *J* = 10.8, 2.3 Hz, 1H, Sia-6), 4.37 (d, *J* = 2.8 Hz, 1H, Gal-4), 4.32 (dd, *J* = 12.3, 2.6 Hz, 1H, Sia-9), 4.24-4.20 (m, 2H, Gal-6, Sia-5), 4.16 (dd, *J* = 12.5, 1.5 Hz, 1H, Gal-6), 4.08 (dd, *J* = 12.3, 5.6 Hz, 1H, Sia-9), 4.02 (br, 1H, Gal-5), 3.72 (s, 3H, OMe), 3.68 (s, 3H, OMe), 2.79 (dd, *J* = 12.9, 4.9 Hz, 1H, Sia-3_{eq}), 2.20 (s, 3H, Ac), 2.06 (s, 3H, Ac), 1.90 (s, 3H, Ac), 1.87 (dd, *J* = 12.9, 11.8 Hz, 1H, Sia-3_{ax}), 1.76 (s, 3H, Ac); ^{13}C NMR (150 MHz, $CDCl_3$) δ 170.61, 170.07, 169.99, 169.75, 168.34, 167.99, 167.74, 165.97, 155.06, 150.85, 137.84, 134.37 (x2), 133.13, 131.40, 131.21, 130.11, 129.91 (x2), 128.87, 128.44 (x2), 128.12 (x2), 126.29 (x2), 123.57 (x2), 117.95, 114.60, 100.72, 97.33, 96.56, 74.34, 69.22, 69.14, 69.06, 67.58, 67.07, 66.63, 63.13, 62.24, 55.63, 52.88, 50.39, 38.76, 21.30, 20.75, 20.66, 20.49; HRMS (ESI) *m/z* for $C_{53}H_{53}NO_{21}Na$ $[M+Na]^+$: calcd 1062.3008, found 1062.2968.

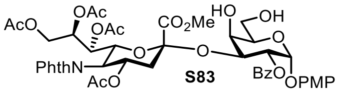

S83

Compound S83. To a solution of compound **S82** (390 mg, 0.38) in DCM (8 mL) was added TFA (1 mL) and H_2O (0.5 mL) at 0 °C. The reaction mixture was stirred at 0 °C for 30 min and then neutralized with saturated $NaHCO_{3(aq)}$. The reaction mixture was extracted with EtOAc, and the resulting solution was washed uwth H_2O and brine. The organic layer was dried over $MgSO_4$, filtered, and concentrated. The residue was purified by silica gel chromatography (EtOAc/Hexanes = 1/1 + 5% MeOH, R_f = 0.38) to afford compound **S83** (355 mg, 99%). ^{1}H NMR (500 MHz, $CDCl_3$) δ 8.11 (d, *J* = 7.5 Hz, 2H), 7.82-7.80 (m, 2H), 7.73-7.71 (m ,2H), 7.56 (dd, *J* = 7.5, 7.5 Hz, 1H), 7.44 (dd,

J = 7.5, 7.5 Hz, 2H), 7.04 (d, J = 9.0 Hz, 2H), 6.74 (d, J = 9.0 Hz, 2H), 5.71 (d, J = 3.7 Hz, 1H, Gal-1), 5.56-5.46 (m, 3H, Gal-2, Sia-4, Sia-8), 5.11 (dd, J = 8.9, 2.0 Hz, 1H, Sia-7), 5.01 (dd, J = 10.8, 2.0 Hz, 1H, Sia-6), 4.88 (dd, J = 10.3, 2.8 Hz, 1H, Gal-3), 4.29 (dd, J = 12.3, 2.0 Hz, 1H, Sia-9), 4.25 (d, J = 2.8 Hz, 1H, Gal-4), 4.22-4.18 (m, 2H, Gal-6, Sia-5), 3.95-3.89 (m, 3H, Gal-5, Gal-6, Sia-9), 3.85 (s, 3H, OMe), 3.73 (s, 3H, OMe), 2.63 (d, J = 12.9, 5.0 Hz, 1H, Sia-3_{eq}), 2.15-2.10 (m, 2H, Sia-3_{ax}, Ac), 2.03 (s, 3H, Ac), 1.93 (s, 3H, Ac), 1.81 (s, 3H, Ac); ^{13}C NMR (125 MHz, $CDCl_3$) δ 170.86, 170.15, 169.98, 169.61, 167.83, 167.65 (x2), 165.74, 155.19, 150.78, 134.42 (x2), 133.17, 131.23 (x2), 129.79 (x3), 128.36, 123.57 (x2), 118.50 (x2), 114.54 (x2), 98.58, 96.75, 77.20, 70.75, 69.86, 69.68, 68.95 (x2), 67.65, 66.91, 66.64, 62.54, 62.19, 55.53, 53.24, 50.05, 36.72, 21.07, 20.58 (x2), 20.48; HRMS (ESI) *m/z* for $C_{46}H_{49}N_1O_{21}Na$ $[M+Na]^+$: calcd 974.2695, found 974.2660.

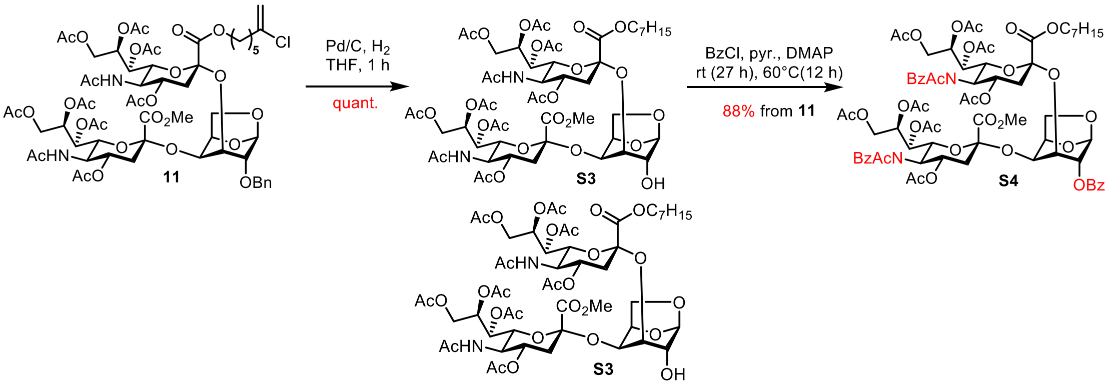

Compound S3. To a solution of compound **11** (19 mg, 14 μmol) in THF (2 mL) was stirred under H_2 atmosphere in the presence of palladium on carbon (16 mg, 10 wt%) at rt. After being stirred for 1 h, the solid was filtered through a pad of celite®, and resulting solution was concentrated under reduced pressure. The residue was purified by silica gel chromatography (EtOAc/Hexanes = 1/1 + 10% MeOH, 3 times, R_f = 0.30) to afford compound **S3** (18 mg, quant). ^{1}H NMR (500 MHz, CD_3CN) δ 6.08 (d, J = 9.9 Hz, 1H), 6.06 (d, J = 10.1 Hz, 1H), 5.29-5.23 (m, 4H), 5.20 (br, 1H, A-1), 4.86 (td, J =

11.7, 4.9 Hz, 1H), 4.84 (td, *J* = 11.7, 4.6 Hz, 1H), 4.41 (dt, *J* =10.8, 7.2 Hz, 1H), 4.37 (t, *J* = 4.4 Hz, 1H), 4.24 (dd, *J* = 7.7, 2.4 Hz, 1H), 4.22 (dd, *J* = 7.7, 2.6 Hz, 1H), 4.18-4.14 (m, 3H), 4.11 (t, *J* = 4.2 Hz, 1H), 4.07 (dd, *J* = 12.3, 5.5 Hz, 1H), 4.02 (d, *J* = 4.6 Hz, 1H), 3.94 (q, *J* = 10.2 Hz, 1H), 3.88-3.84 (m, 3H), 3.82-3.80 (m, 4H), 3.54 (d, *J* = 5.5 Hz, 1H), 3.49 (t, *J* = 6.1 Hz, 1H), 2.72 (dd, *J* = 12.4, 4.6 Hz, 1H), 2.60 (dd, *J* = 12.7, 4.9 Hz, 1H), 2.12 (s, 3H, Ac), 2.07 (s, 3H, Ac), 2.06 (s, 3H, Ac), 2.05 (s, 3H, Ac), 1.97 (s, 6H, Ac(x2)), 1.97-1.94 (m, 1H), 1.94 (s, 6H, Ac(x2)), 1.77-1.68 (m, 5H), 1.74 (s, 6H, Ac(x2)), 1.49-1.42 (m, 2H), 1.36-1.27 (m, 4H), 0.89 (t, *J* = 6.6 Hz, 3H); ^{13}C NMR (125 MHz, CD_3CN) δ 171.77, 171.48, 171.47, 171.07, 170.93, 170.87, 170.79, 170.78, 170.76, 170.64, 169.21, 167.27, 102.33, 101.10, 99.75, 76.27, 74.21, 73.50, 73.25, 72.87, 70.30, 70.17, 70.00, 69.43, 68.62, 68.25, 67.51, 67.10, 64.62, 63.19, 62.82, 53.90, 49.59, 49.41, 39.39, 37.60, 32.40, 29.89, 29.26, 26.59, 23.34, 23.15, 23.13, 21.70, 21.39, 21.22, 21.16, 21.06 (x2), 20.98, 20.94, 14.40; HRMS (ESI) *m/z* for $C_{52}H_{75}N_2O_{29}$ [M-H]$^-$: calcd 1191.4456, found 1191.4487.

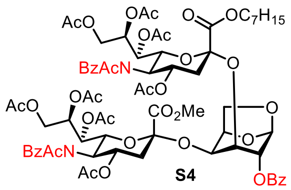

S4

Compound S4. To a solution of compound **96** (87 mg, 66 μmol) in THF (6 mL) was stirred under H_2 atmosphere in the presence of palladium on carbon (70 mg, 10 wt%) at rt. After being stirred for 1 h, the solid was filtered through a pad of celite®, and resulting solution was concentrated under reduced pressure. Next, to a solution of above residue in pyridine (6 mL) was added benzoyl chloride (30 μL, 265 μmol) and 4-(dimethylamino)pyridine (0.8 mg, 7 μmol) at rt. The reaction mixture was stirred at rt for 27 h, and then gradually warmed to 60 °C and stirred for 12 h. The reaction was quenched with MeOH and concentrated *in vacuo*. The residue was extracted with EtOAc, and the resulting organic solution was washed with 1N $HCl_{(aq)}$, saturated

$NaHCO_{3(aq)}$, and brine. The organic layer was dried over $MgSO_4$, filtered, and concentrated. The residue was purified by silica gel chromatography (EtOAc/Hexanes = 3/1, R_f = 0.45) to afford compound **S4** (88 mg, 88% over 2 steps). ^1H NMR (400 MHz, CD_3CN) δ 8.22 (d, J = 7.3 Hz, 2H), 7.76 (d, J = 7.5 Hz, 1H), 7.75-7.67 (m, 2H), 7.66-7.60 (m, 4H), 7.55-7.47 (m, 6H), 5.64-5.55 (m, 2H), 5.45 (br, 1H, A-1), 5.31-5.28 (m, 3H), 5.17 (d, J = 9.0 Hz, 1H), 4.99 (br, 1H), 4.77 (t, J =10.5 Hz, 1H), 4.67-4.53 (m, 6H), 4.32-4.24 (m, 3H), 4.17-4.14 (m, 2H), 3.85 (s, 3H, OCH_3), 3.63-3.59 (m, 3H), 2.96 (dd, J = 12.4, 4.8 Hz, 1H), 2.81 (dd, J = 12.7, 5.0 Hz, 1H), 2.17-1.67 (m, 34H), 1.48-1.43 (m, 2H), 1.33-1.27 (m, 6H), 0.86 (br, 3H); ^{13}C NMR (100 MHz, CD_3CN) δ 175.54, 174.57, 171.37 (x2), 171.28, 170.98 (x3), 170.61, 170.53, 170.06, 169.08 (x2), 167.17, 166.19, 137.43, 134.35, 134.11, 131.07, 130.64, 130.13, 130.05, 129.81, 129.69, 129.46, 100.29, 99.85, 99.66, 75.18, 74.58, 73.00, 70.76, 70.62, 70.31, 69.22, 68.55, 68.38, 67.68, 67.59, 67.37, 67.14, 65.03, 62.23, 61.35, 56.47, 56.37, 53.96, 40.33, 38.27, 32.40, 29.84, 29.46, 28.05, 27.55, 26.59, 23.34, 21.52, 21.40, 21.33, 21.19, 21.12, 20.92, 20.75, 14.41; HRMS (ESI) *m/z* for $C_{73}H_{88}N_2NaO_{32}$ $[M+Na]^+$: calcd 1527.5218, found 1527.5217.

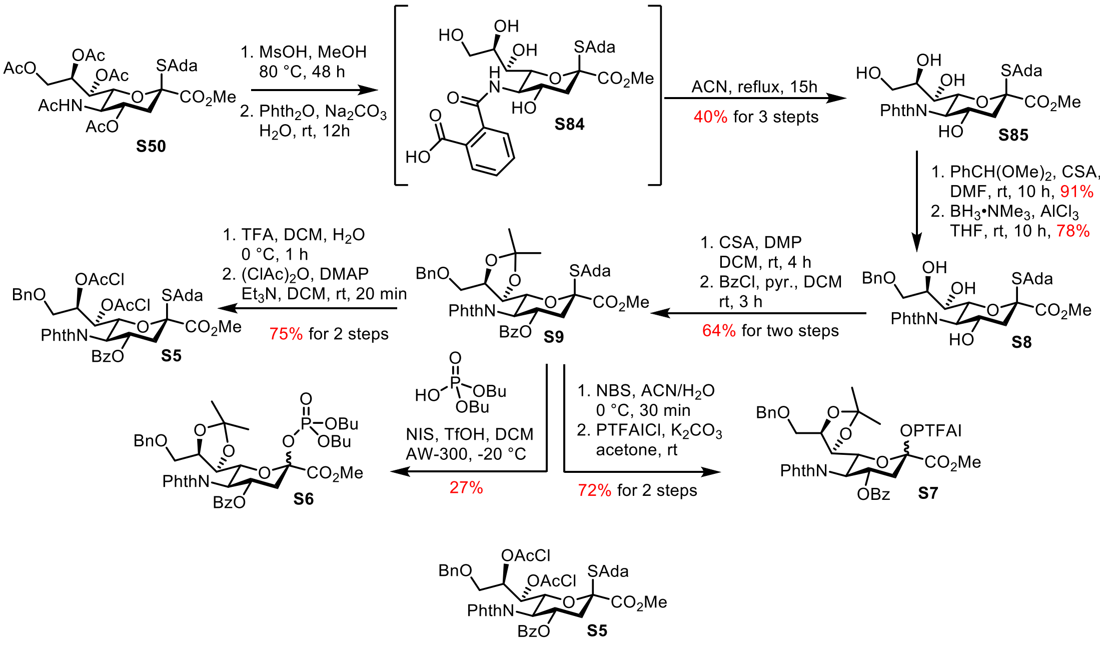

Compound S5. To compound **S9** (87 mg, 0.11 mmol), a solution of TFA/DCM/H_2O (1.5 mL/1 mL/ 0.5 mL) was added at 0 °C. After being stirred at 0 °C for 60 min, the reaction mixture was quenched with Et_3N and concentrated *in vacuo*. The residue was extracted with EtOAc, and the resulting organic solution was washed with saturated $NaHCO_{3(aq)}$ and brine. The organic layer was dried over $MgSO_4$, filtered, and concentrated. To a solution of the above residue in DCM/Et_3N (4 mL/1 mL) was added chloroacetic anhydride (74 mg, 0.44 mmol) and 4-(dimethylamino)pyridine (3 mg, 22 μmol) at rt. After being stirred for 20 min, the reaction mixture was quenched with MeOH and concentrated *in vacuo*. The residue was extracted with EtOAc, and the resulting organic solution was washed with 1N $HCl_{(aq)}$, saturated $NaHCO_{3(aq)}$, and brine. The organic layer was dried over $MgSO_4$, filtered, and concentrated. The residue was purified by silica gel chromatography (EtOAc/Hexanes = 1/1, R_f = 0.55) to afford compound **S5** (74 mg, 75% over two steps). ^1H NMR (500 MHz, $CDCl_3$) δ 7.77-7.76 (m, 4H), 7.66 (m, 2H), 7.45 (t, *J* = 7.3 Hz, 1H), 7.32-7.22 (m, 7H), 6.09 (ddd, *J* = 10.5, 10.5, 4.7 Hz, 1H, H-4), 5.94 (d, *J* = 10.5 Hz, 1H, H-6), 5.49-5.48 (m, 1H, H-8), 5.39 (br, 1H, H-7), 4.57 (d, *J* = 11.8 Hz, 1H), 4.46 (d, *J* = 11.8 Hz, 1H), 4.44 (dd, *J* = 10.5, 10.5 Hz, 1H, H-5), 4.30 (d, *J* = 10.9 Hz, 1H, H-9), 4.09 (d, *J* = 15.5 Hz, 2H), 4.01 (d, *J* = 15.5 Hz, 1H), 4.00 (d, *J* = 15.5 Hz, 1H), 3.81 (s, 3H, OCH_3), 3.66 (dd, *J* = 10.9, 7.6 Hz, 1H, H-9), 2.94 (dd, *J* = 13.5, 4.7 Hz, 1H, H-3_{eq}), 2.11-1.95 (m, 10H, H-3_{ax}, SAda), 1.69-1.59 (m, 6H, SAda); ^{13}C NMR (125 MHz, $CDCl_3$) δ 169.98, 167.81, 166.80, 166.57, 165.01, 137.86, 134.39, 133.21, 131.15, 129.53, 129.01, 128.32, 128.26, 127.54, 127.45, 123.59, 85.85, 74.59, 73.21, 71.49, 68.39, 68.34, 67.17, 52.73, 50.81, 50.72, 43.40, 40.99, 40.67, 40.41, 35.91, 29.76; HRMS (ESI) *m/z* for $C_{46}H_{47}Cl_2NNaO_{12}S$ $[M+Na]^+$: calcd 930.2094, found 930.2095.

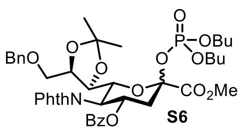

S6

Compound S6. Compound **S6** was synthesized from dibutyl phosphate (72 μL, 0.38 mmol) and glycosyl donor **S9** (100 mg, 0.13 mmol) by following the **General glycosylation procedure A**. The mixture was purified by silica gel chromatography (EtOAc/Hexanes = 1/2, R_f = 0.20) to afford compound **S6** as anomeric mixture (28 mg, 27%). ^{1}H NMR (400 MHz, $CDCl_3$) δ 7.85-7.81 (m, 2H), 7.76-7.74 (m, 2H, Phth), 7.65-7.63 (m, 2H, Phth), 7.49-7.44 (m, 1H), 7.39-7.29 (m, 6H), 7.26-7.22 (m, 1H), 6.14 (ddd, *J* =10.6, 10.6, 5.0 Hz, 0.5H, β-H4), 5.84 (ddd, *J* = 12.3, 10.6, 5.0 Hz, 0.5H, α-H4), 5.14-5.08 (m, 1H, H-6), 4.80 (dd, *J* = 10.6, 5.0 Hz, 0.5H, β-H5), 4.77 (dd, *J* = 10.6, 5.0 Hz, 0.5H, α-H5), 4.66-4.58 (m, 2H, CH_2Ph), 4.44-4.40 (m, 1H, H-8), 4.29-4.01 (m, 6H, PO_2CH_2 (x2), H-7 (x0.5), H-9 (x1.5)), 3.98 (dd, *J* = 7.2, 1.0 Hz, 0.5H, H-7), 3.93 (s, 1.5H, OCH_3), 3.89 (dd, *J* = 10.0, 5.3 Hz, 0.5H, H-9), 3.82 (s, 1.5H, OCH_3), 3.09 (dd, *J* = 12.3, 5.0 Hz, 0.5H, α-$H3_{eq}$), 3.06 (dd, *J* = 13.2, 5.0 Hz, 0.5H, β-$H3_{eq}$), 2.37 (dd, *J* = 13.2, 10.6 Hz, 0.5H, β-$H3_{ax}$), 2.30 (dd, *J* = 12.3, 12.3 Hz, 0.5H, α-$H3_{ax}$), 1.73-1.61 (m, 4H, OCH_2CH_2), 1.58 (s, 1.5H, CCH_3), 1.48 (s, 1.5H, CCH_3), 1.45-1.36 (m, 4H, CH_2CH_3), 1.29 (s, 1.5H, CCH_3), 1.28 (s, 1.5H, CCH_3), 0.94-0.89 (m, 6H, CH_2CH_3); ^{13}C NMR (100 MHz, $CDCl_3$) δ 167.21, 167.17, 166.23, 165.29, 165.24, 138.31, 138.21, 134.16, 134.13, 133.20, 133.11, 131.39, 129.65, 129.61, 129.20, 129.09, 128.32, 128.29, 128.22, 128.03, 127.78, 127.44, 123.46, 109.78, 109.53, 100.11, 100.06, 98.78, 98.72, 75.98, 75.89, 74.02, 73.84, 73.41, 73.36, 70.56, 69.08, 69.06, 68.81, 68.26 (d, *J* = 6.6 Hz), 68.21 (d, *J* = 6.4 Hz), 68.11 (d, *J* = 6.1 Hz), 67.81 (d, *J* = 6.4 Hz), 66.91, 66.66, 53.16, 53.15, 50.89, 50.57, 38.52 (d, *J* = 6.4 Hz), 37.07 (d, *J* = 2.2 Hz), 32.21 (d, *J* = 5.6 Hz), 32.14 (d, *J* =5.5 Hz), 32.08 (d, *J* = 6.9 Hz), 32.01 (d, *J* = 7.4 Hz), 26.37, 26.22, 25.60, 25.30, 18.61 (d, *J* = 2.7 Hz), 18.57 (d, *J* = 1.3 Hz), 13.58 (d, *J* = 2.3 Hz),

13.52 (d, *J* = 2.8 Hz); HRMS (ESI) *m/z* for $C_{43}H_{52}NNaO_{14}P$ [M+Na]$^+$: calcd 860.3023, found 860.3019.

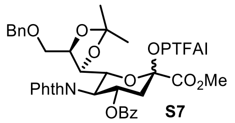

S7

Compound S7. To a solution of compound **S9** (300 mg, 0.38 mmol) in ACN/H_2O (6 mL/1.5 mL) was added *N*-bromosuccinimide (134 mg, 0.75 mmol) at 0 °C. The reaction mixture was stirred at 0 °C for 30 min and then quenched with saturated $Na_2S_2O_{3(aq)}$. The mixture was extracted with EtOAc, and the resulting organic solution was washed with H_2O and brine. The organic layer was dried over $MgSO_4$, filtered, and concentrated. The residue was purified by silica gel chromatography (EtOAc/Hexanes = 1/2, R_f = 0.23) to afford hydrolyzed product. Next, to a solution of hydrolyzed product in acetone (4 mL) was added 2,2,2-trifluoro-*N*-phenylacetimidoyl chloride (245 μL, 1.51 mmol) and K_2CO_3 (208 mg, 1.51 mmol) at rt. The reaction mixture was stirred at rt for 12 h and then concentrated *in vacuo*. The residue was purified by silica gel chromatography (EtOAc/Hexanes = 1/1, R_f = 0.55) to afford compound **S7** as anomeric mixture (223 mg, 72% over two steps). ^{1}H NMR (500 MHz, $CDCl_3$) δ 7.88-7.84 (m, 2.6H), 7.80-7.76 (m, 2.6H, Phth), 7.67-7.65 (m, 2.6H, Phth), 7.49 (t, *J* = 7.4 Hz, 1.3H), 7.36-7.33 (m, 3.5H), 7.31-7.26 (m, 3.5H), 7.23-7.20 (m, 2.9H), 7-14-7.12 (m, 1.8H), 7.09 (t, *J* = 7.4 Hz, 1.3H), 6.81 (d, *J* = 7.7 Hz, 2.6H), 6.05 (ddd, *J* = 11.0, 11.0, 5.6 Hz, 0.3H, α-H4), 5.87 (ddd, *J* = 10.9, 10.9, 4.8 Hz, 1H, β-H4), 5.50 (d, *J* = 10.9 Hz, 1H, β-H6), 5.20 (d, *J* = 11.0 Hz, 0.3H, α-H6), 4.86 (dd, *J* = 11.0, 11.0 Hz, 0.3H, α-H5), 4.83 (dd, *J* = 10.9, 10.9 Hz, 1H, β-H5), 4.55 (d, *J* = 11.6 Hz, 0.3H, α-C*H*2Ph), 4.50 (d, *J* = 11.6 Hz, 0.3H, α-C*H*2Ph), 4.45-4.41 (m, 0.3H, α-H8), 4.41 (ddd, *J* = 6.3, 6.3, 6.3 Hz, 1H, β-H8), 4.20 (d, *J* = 11.6 Hz, 1H, β-C*H*2Ph), 4.16 (d, *J* = 11.6 Hz, 1H, β-C*H*2Ph), 4.07 (d, *J* = 6.9 Hz, 0.3H, α-H7), 3.99-3.98 (m, 0.3H, α-H9), 3.98 (d, *J* = 6.3 Hz, 1H, β-H7), 3.90 (s, 3H, β-OC*H*3), 3.88-3.87 (m, 2.3H, α-H9, β-H9, β-H9), 3.79 (s, 1H, α-

OCH_3), 3.36 (dd, J = 13.9, 5.2 Hz, 0.3H, α-H3$_{eq}$), 3.01 (dd, J = 12.8, 4.8 Hz, 1H, β-H3$_{eq}$), 2.39 (dd, J = 13.9, 11.0 Hz, 0.3H, α-H3$_{ax}$), 2.35 (dd, J = 12.8, 10.9 Hz, 1H, β-H3$_{ax}$), 1.56 (s, 3H, β-CCH_3, overlap with H_2O), 1.51 (s, 1H, α-CCH_3), 1.33 (s, 3H, β-CCH_3), 1.30 (s, 1H, α-CCH_3); ^{13}C NMR (125 MHz, $CDCl_3$) δ 168.03, 167.52, 165.30, 165.28, 165.23, 142.96, 142.81, 141.87 (d, J = 38.6 Hz), 140.86 (d, J = 36.2 Hz), 137.89, 137.80, 134.19, 133.30, 133.22, 131.31, 129.59, 129.09, 128.93, 128.80, 128.76, 128.35, 128.23, 128.10, 128.06, 128.00, 127.57, 127.38, 124.53, 124.29, 123.48, 119.27, 119.04, 117.84(d, J =260.5 Hz), 115.46 (d, J = 285.4 Hz), 110.02, 109.98, 100.72, 97.17, 75.95, 75.50, 74.17, 73.82, 73.52, 73.09, 71.09, 69.76, 68.67, 68.32, 66.62, 66.24, 52.98, 52.74, 50.58, 50.54, 37.63, 34.35, 26.09, 26.06, 25.73, 25.63; HRMS (ESI) *m/z* for $C_{43}H_{39}F_3N_2NaO_{11}$ [M+Na]$^+$: calcd 839.2404, found 839.2400.

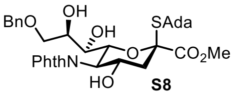

S8

Compound S8. To a solution of compound **S85** (3.16 g, 5.63 mmol) in DMF (56 mL) was added camphor sulfonic acid (392 mg, 1.69 mmol) and benzaldehyde dimethyl acetal (1.70 mL, 11.25 mmol) under a nitrogen atmosphere at rt. After being stirred for 10 h, the reaction was quenched with Et_3N and concentrated *in vacuo*. The residue was extracted with EtOAc, and the resulting organic solution was washed with saturated $NaHCO_{3(aq)}$ and brine. The organic layer was dried over $MgSO_4$, filtered, and concentrated. The residue was purified by silica gel chromatography (EtOAc/Hexanes = 1/1 + 5% MeOH, R_f = 0.38) to afford benzylidene protected mixture compound (3315 mg, 91%). Next, to a solution of above compound (2.52 g, 3.88 mmol) in THF (40 mL) was added borane trimethylamine complex (1.69 g, 23.19 mmol) under a nitrogen atmosphere at 0 °C. The reaction mixture was stirred at 0 °C for 30 min, and then aluminum chloride (3.10 g, 23.19 mmol) was added in 3 portions at 0 °C. The reaction mixture was gradually warmed to rt and stirred for 10 h. The reaction was quenched

with 10% $H_2SO_{4(aq)}$. The reaction mixture was extracted with EtOAc, and the resulting organic solution was washed with 1N $HCl_{(aq)}$, saturated $NaHCO_{3(aq)}$, and brine. The organic layer was dried over $MgSO_4$, filtered, and concentrated. The residue was purified by silica gel chromatography (EtOAc/Hexanes = 2/1, R_f = 0.30) to afford compound **S8** (1.97 g, 78%). ^{1}H NMR (400 MHz, $CDCl_3$) δ 7.81-7.79 (m, 2H, Phth), 7.70-7.68 (m, 2H, Phth), 7.28-7.22 (m, 5H), 5.31 (d, *J* = 10.5 Hz, 1H, H-6), 4.92 (ddd, *J* = 10.5, 10.5, 4.6 Hz, 1H, H-4), 4.51 (m, 2H, CH_2Ph), 4.35 (dd, *J* = 10.5, 10.5 Hz, 1H, H5), 3.92 (br, 1H, H-8), 3.81 (dd, *J* = 9.8, 3.2 Hz, 1H, H-9), 3.76 (s, 3H, OCH_3), 3.59 (dd, *J* = 9.8, 6.5 Hz, 1H, H-9), 3.50 (d, *J* = 9.1 Hz, 1H, 7-OH), 3.39 (dd, *J* = 9.1, 9.1 Hz, 1H, H-7), 2.65 (dd, *J* = 13.5, 4.6 Hz, 1H, H-3_{eq}), 2.59 (d, *J* = 3.0 Hz, 1H, 8-OH), 2.04-1.92 (m, 10H, SAda, H-3_{ax}), 1.65 (br, 6H); ^{13}C NMR (125 MHz, $CDCl_3$) δ 171.82 (x2), 168.32, 137.90, 133.95, 131.61, 128.20, 127.51, 127.48, 123.32, 85.95, 73.07, 71.90, 70.64, 68.81, 68.41, 63.72, 54.47, 52.72, 49.91, 43.43, 43.11 (x3), 35.95 (x3), 29.76 (x3); HRMS (ESI) *m/z* for $C_{35}H_{41}NNaO_9S$ $[M+Na]^+$: calcd 674.2400, found 674.2389.

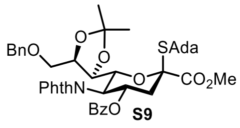

Compound S9. To a solution of compound **S8** (1.84 g, 2.82 mmol) in DCM (28 mL) was added camphor sulfonic acid (196 mg, 0.85 mmol) and 2,2-dimethoxypropane (1.73 mL, 14.08 mmol) under a nitrogen atmosphere at rt. After being stirred for 4 h, the reaction was quenched with Et_3N and concentrated *in vacuo*. The residue was extracted with EtOAc, and the resulting organic solution was washed with saturated $NaHCO_{3(aq)}$ and brine. The organic layer was dried over $MgSO_4$, filtered, and concentrated. To a solution of above residue in DCM (20 mL) was added benzoyl chloride (460 μL, 3.93 mmol) and pyridine (1.6 mL, 19.66 mmol) under nitrogen atmosphere at rt. After being stirred for 3 h, the reaction was quenched with MeOH and concentrated *in vacuo*. The residue was extracted with EtOAc, and the resulting organic

solution was washed with 1N $HCl_{(aq)}$, saturated $NaHCO_{3(aq)}$, and brine. The organic layer was dried over $MgSO_4$, filtered, and concentrated. The residue was purified by silica gel chromatography (EtOAc/Hexanes = 1/3, R_f = 0.20) to afford compound **S9** (1.44 g, 64% over two steps). ^{1}H NMR (500 MHz, $CDCl_3$) δ 7.86 (d, *J* = 7.8 Hz, 2H), 7.78-7.77 (m, 2H, Phth), 7.67-7.65 (m, 2H, Phth), 7.18 (t, *J* = 7.4 Hz, 1H), 7.39-7.30 (m, 6H), 7.26 (d, *J* = 7.2 Hz, 1H), 6.07 (ddd, *J* = 9.2, 9.2 4.3 Hz, 1H, H-4), 5.25 (d, *J* = 9.2 Hz, 1H, H-6), 4.65 (dd, *J* = 9.2, 9.2 Hz, 1H, H-5), 4.64 (d, *J* = 12.8 Hz, 1H, CH_2Ph), 4.59 (d, *J* = 12.8 Hz, 1H, CH_2Ph), 4.37 (ddd, *J* = 6.5, 6.5, 6.5 Hz, 1H, H-8), 4.08 (d, *J* = 6.5 Hz, 1H, H-7), 3.98 (dd, *J* = 9.3, 6.5 Hz, 1H, H-9), 3.93 (dd, 9.3, 6.5 Hz, 1H, H-9), 3.72 (s, 3H, OCH_3), 3.02 (dd, *J* = 14.1, 4.3 Hz, 1H, H-3_{eq}), 2.32 (dd, *J* = 14.1, 9.2 Hz, 1H, H-3_{ax}), 2.07-1.98 (m, 9H, SAda), 1.66 (br, 6H, SAda), 1.41 (s, 3H, CCH_3), 1.25 (s, 3H, CCH_3); ^{13}C NMR (125 MHz, $CDCl_3$) δ 170.82 (x2), 165.26, 138.08, 134.12, 133.12, 131.52, 129.68, 129.37, 128.30, 128.27, 128.10, 127.54, 123.43, 109.36, 84.97, 76.14, 74.72, 73.52, 69.11, 68.82, 67.44, 52.53, 51.81, 50.43, 43.67 (x3), 39.02, 36.03 (x3), 29.91 (x3), 26.33, 25.55; HRMS (ESI) *m/z* for $C_{45}H_{49}NNaO_{10}S$ $[M+Na]^+$: calcd 818.2975, found 818.2979.

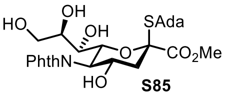

S85

Compound S85. To a solution of compound **S50** (500 mg, 0.78 mmol) in MeOH (8 mL) was added methanesulfonic acid (150 µL, 2.34 mmol) under a nitrogen atmosphere at rt. The reaction mixture was gradually warmed to 80 °C and stirred for 2 days. The reaction mixture was quenched with Et_3N and concentrated under reduced pressure to afford crude amine compound. Next, to a solution of crude amine compound in H_2O (15 mL) was added Na_2CO_3 (233 mg, 2.21 mmol) and phthalic anhydride (326 mg, 2.20 mmol) at rt. After being stirred for 12 h, the reaction mixture was concentrated *in vacuo*. The residue was purified by silica gel chromatography to afford crude compound **S84**.

Next, to a solution of crude compound **S84** in ACN (30 mL), the mixture was gradually warmed to 90 °C. After being stirred at 90 °C for 15 h, the reaction mixture was concentrated *in vacuo*. The residue was purified by silica gel chromatography (EtOAc/Hexanes = 1/1 + 5% MeOH, R_f = 0.13) to afford compound **S85** (176 mg, 40% over three steps). ^{1}H NMR (400 MHz, CD_3OD) δ 7.89-7.85 (m, 2H, Phth), 7.84-7.81 (m, 2H, Phth), 5.29 (dd, *J* = 10.5, 0.8 Hz, 1H, H-6), 4.92 (ddd, *J* = 11.8, 10.5, 4.7 Hz, 1H, H-4), 4.27 (dd, *J* = 10.5, 10.5 Hz, 1H, H-5), 3.87 (s, 3H, OCH_3), 3.76 (dd, *J* = 11.2, 2.8 Hz, 1H, H-9), 3.74-3.70 (m, 1H, H-8), 3.60 (dd, *J* = 11.2, 4.9 Hz, 1H, H-9), 3.32 (dd, *J* = 8.5, 0.8 Hz, 1H, H-9), 2.58 (dd, *J* = 13.5, 4.7 Hz, 1H, H-3_{eq}), 2.09-2.00 (m, 9H), 1.84 (dd, *J* = 13.5, 11.8 Hz, 1H, H-3_{ax}), 1.72 (br, 6H); ^{13}C NMR (100 MHz, CD_3OD) δ 174.04 (x2), 169.75, 135.36 (x2), 133.08 (x2), 124.13 (x2), 87.34, 71.34, 71.11, 69.92, 64.93, 63.98, 56.04, 53.49, 50.84, 44.79, 44.47 (x3), 37.17 (x3), 31.29 (x3); HRMS (ESI) *m/z* for $C_{28}H_{35}NNaO_9S$ $[M+Na]^+$: calcd 584.1930, found 584.1922.

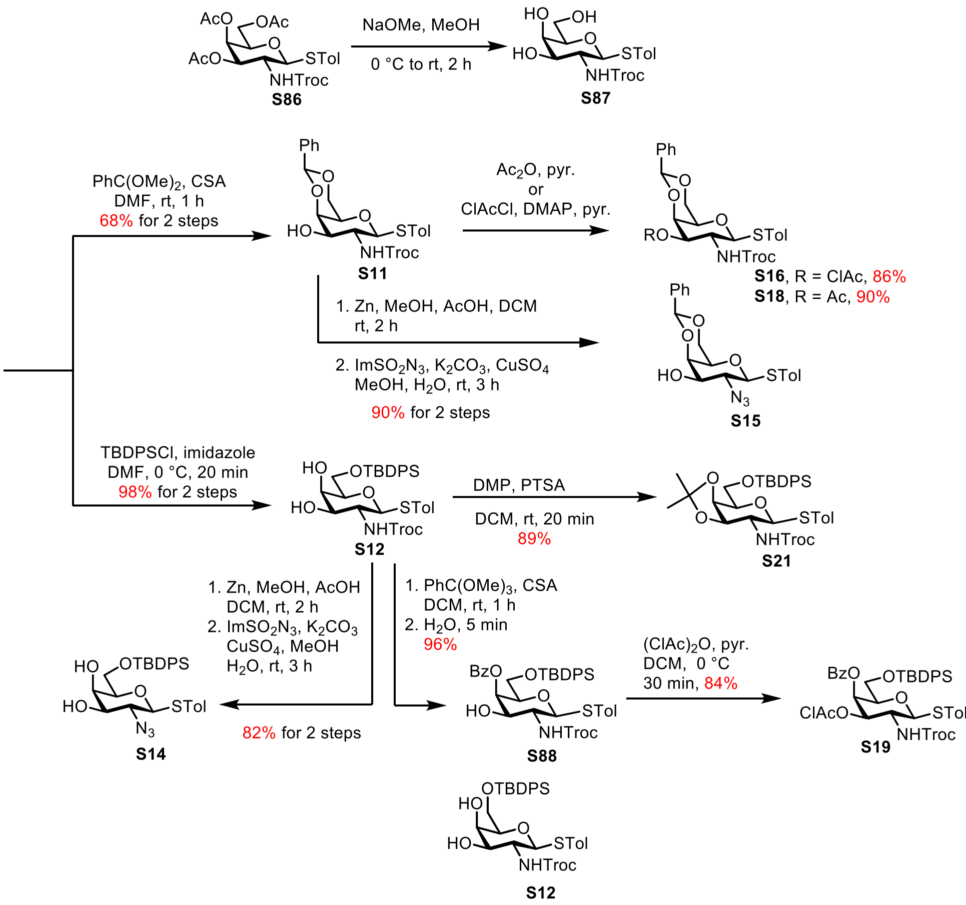

Compound S12. To a solution of compound **S86** (634 mg, 1.08 mmole) in MeOH (11 mL) was added sodium methoxide (12 mg, 216 μmol) under a nitrogen atmosphere at 0 °C. The reaction mixture was gradually warmed to rt and stirred for 2 h. The reaction mixture was neutralized with Amberlite® IR-120 (H^+) resin and the resin was filtered off. The resulting solution was concentrated under reduced pressure to afford compound **S87**. Next, a solution of above compound **S87** in DMF (11 mL) was added imidazole (221 mg, 3.26 mmol) at 0 °C under a nitrogen atmosphere. The reaction mixture was stirred at 0 °C for 30 min, and then tert-butyl(chloro)diphenylsilane (306 μL, 1.19 mmol) was added to the solution. The reaction mixture was stirred at 0 °C for 20 min. The reaction was quenched with MeOH and then concentrated *in vacuo*. The residue was extracted with EtOAc, and the resulting organic solution was washed with saturated $NaHCO_{3(aq)}$ and brine. The organic layer was dried over $MgSO_4$, filtered, and concentrated. The residue was purified by silica gel chromatography (EtOAc/Hexanes = 1/1, R_f = 0.50) to afford compound **S12** (723 mg, 98%). ^{1}H NMR (400 MHz, $CDCl_3$) δ 7.71-7.65 (m, 4H), 7.44-7.34 (m, 8H), 7.03 (d, *J* = 8.0 Hz, 2H), 5.26 (d, *J* = 6.9 Hz, 1H, N*H*), 4.76 (d, *J* = 12.0 Hz, 1H, CH_2CCl$_3$), 4.70 (d, *J* = 12.0 Hz, 1H, CH_2CCl$_3$), 4.69 (d, *J* = 9.6 Hz, 1H, H-1), 4.08 (dd, *J* = 3.6, 3.6 Hz, 1H, H-4), 3.95 (dd, *J* = 10.8, 5.4 Hz, 1H, H-6), 3.92 (dd, *J* = 10.8, 4.8 Hz, 1H, H-6), 3.76 (dd, *J* = 8.4, 8.4 Hz, 1H, H-3), 3.60 (dd, *J* = 9.6, 8.4 Hz, 1H, H-3), 3.53 (dd, *J* = 5.4, 4.8 Hz, 1H, H-5), 3.27 (d, *J* = 8.4 Hz, 1H, 3-OH), 3.04 (d, *J* = 3.6 Hz, 1H, 4-OH), 2.29 (s, 3H, STol), 1.05 (s, 9H, C(CH_3)$_3$); ^{13}C NMR (100 MHz, $CDCl_3$) δ 155.01, 138.07, 135.64, 135.55, 132.79, 132.63, 129.89, 129.72, 127.82, 95.34, 86.72, 78.06, 74.69, 73.12, 69.10, 63.91, 53.97, 26.76, 21.10, 19.12; HRMS (ESI) *m/z* for $C_{32}H_{39}Cl_3NO_6SSi$ $[M+H]^+$: calcd 698.1333, found 698.1333.

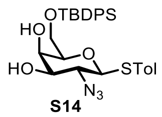

S14

Compound S14. To a solution of compound **S12** (389 mg, 0.57 mmol) in MeOH/DCM/AcOH (2.8 mL/1.4 mL/1.4 mL) was added zinc dust (744 mg, 11.38 mmol) at rt. After being stirred at rt for 2 h, the solid was filtered through a pad of celite®, and resulting solution was concentrated under reduced pressure. Next, a solution of above residue in MeOH/H_2O (3.8 mL/1.8 mL) was added K_2CO_3 (236 mg, 1.71 mmol), 1 *H*-imidazole-1-sulfonyl azide HCl salt (237 mg, 1.14 mmol) and $CuSO_4{\cdot}5H_2O$ (1.4 mg, 6 μmol) at rt. The reaction was stirred at rt for 3 h, and then concentrated *in vacuo*. The residue was extracted with EtOAc, and the resulting organic solution was washed with 1N $HCl_{(aq)}$, saturated $NaHCO_{3(aq)}$, and brine. The organic layer was dried over $MgSO_4$, filtered, and concentrated. The residue was purified by silica gel chromatography (EtOAc/Hexanes = 2/3, R_f = 0.38) to afford compound **S14** (258 mg, 82% over 2 steps). ^{1}H NMR (400 MHz, $CDCl_3$) δ 7.73-7.71 (m, 2H), 7.67-7.65 (m, 2H), 7.48-7.46 (m, 2H), 7.44-7.36 (m, 6H), 7.06 (d, *J* = 8.0 Hz, 2H), 4.32 (d, *J* = 9.8 Hz, 1H, H-1), 4.09 (dd, *J* = 3.4, 3.4 Hz, 1H, H-4), 3.97 (dd, *J* = 10.8, 5.1 Hz, 1H, H-6), 3.91 (dd, *J* = 10.8, 4.2 Hz, 1H, H-6), 3.49-3.47 (m, 2H, H-2, H-3), 3.43 (dd, *J* = 5.1, 4.2 Hz, 1H, H-5), 3.16 (d, *J* = 3.4 Hz, 1H, 4-OH), 2.63 (d, *J* = 7.9 Hz, 1H, 3-OH), 2.31 (s, 3H, STol), 1.06 (s, 9H, $C(CH_3)_3$); ^{13}C NMR (100 MHz, $CDCl_3$) δ 138.04, 135.44, 135.38, 133.06, 132.50, 132.38, 129.79, 129.58, 128.06, 127.70, 86.78, 77.79, 74.14, 68.89, 64.01, 62.58, 26.60, 20.97, 18.92; HRMS (ESI) *m/z* for $C_{29}H_{35}N_3NaO_4SSi$ $[M+Na]^+$: calcd 572.2015, found 572.2010.

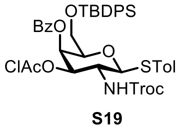

S19

Compound S19. To a solution of compound **S12** (737 mg, 1.08 mmol) in DCM (10 mL) was added camphor sulfonic acid (75 mg, 0.32 mmol) and trimethyl orthobenzoate (223 μL, 1.30 mmol) under nitrogen atmosphere at rt. After being stirred at rt for 1 h, the reaction was added H_2O (1 ml) at rt. After being stirred at rt for 5 min, the reaction

was quenched with Et_3N and concentrated under reduced pressure. The residue was extracted with EtOAc, and the resulting organic solution was washed with saturated $NaHCO_{3(aq)}$ and brine. The organic layer was dried over $MgSO_4$, filtered, and concentrated. The residue was purified by silica gel chromatography (EtOAc/Hexanes = 1/3, R_f = 0.25) to afford compound **S88** (836 mg, 96%). To a solution of compound **S88** (736 mg, 0.92 mmol) in DCM (9.2 mL) was added chloroacetic anhydride (313 mg, 1.83 mmol) and pyridine (300 μL, 3.67 mmol) under nitrogen atmosphere at 0 °C. After being stirred at 0 °C for 30 min, the reaction mixture was concentrated *in vacuo*. The residue was extracted with EtOAc, and the resulting organic solution was washed with 1N $HCl_{(aq)}$, saturated $NaHCO_{3(aq)}$, and brine. The organic layer was dried over $MgSO_4$, filtered, and concentrated. The residue was purified by silica gel chromatography (EtOAc/Hexanes = 1/3, R_f = 0.38) to afford compound **S19** (678 mg, 84%). ^{1}H NMR (400 MHz, $CDCl_3$) δ 7.88 (d, *J* = 7.6 Hz, 2H), 7.63-7.59 (m, 3H), 7.44-7.33 (m, 9H), 7.27-7.23 (m, 1H), 7.12-7.07 (m, 4H), 5.82 (d, *J* = 3.0 Hz, 1H, H-4), 5.47 (d, *J* = 9.4 Hz, 1H, N*H*), 5.07 (d, *J* = 9.4 Hz, 1H, H-3), 4.92 (d, *J* = 9.4 Hz, 1H, H-1), 4.72 (m, 2H), 3.98-3.91 (m, 1H, H-6), 3.96 (d, *J* = 15.3 Hz, 1H), 3.89 (d, *J* = 15.3 Hz, 1H), 3.81-3.75 (m, 2H, H-5, H-6), 3.69 (ddd, *J* = 9.4, 9.4, 9.4 Hz, 1H, H-2), 2.35 (s, 3H, STol), 0.96 (s, 9H, $C(CH_3)_3$); ^{13}C NMR (100 MHz, $CDCl_3$) δ 166.79, 165.51, 153.70, 138.65, 135.55, 135.40, 134.20, 133.43, 132.71, 132.50, 129.92, 129.83, 129.73, 129.65, 129.25, 128.45, 127.79, 127.58, 95.34, 85.77, 77.20, 74.48, 73.17, 67.14, 61.15, 51.32, 40.56, 26.61, 21.28, 18.95; HRMS (ESI) *m/z* for $C_{41}H_{43}Cl_4NNaO_8SSi$ $[M+Na]^+$: calcd 900.1130, found 900.1127.

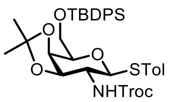

S21

Compound S21. To a solution of compound **S12** (130 mg, 0.19 mmol) in DCM (2 mL) was added *p*-toluenesulfonic acid (10 mg, 57 μmol) and 2,2-dimethoxypropane (230

μL, 1.90 mmol) under a nitrogen atmosphere at rt. After stirring for 20 min, the reaction was quenched with Et_3N and concentrated under reduced pressure. The residue was extracted with EtOAc, and the resulting organic solution was washed with saturated $NaHCO_{3(aq)}$ and brine. The organic layer was dried over $MgSO_4$, filtered, and concentrated. The residue was purified by silica gel chromatography (EtOAc/Hexanes = 1/3, R_f = 0.38) to afford compound **S21** (125 mg, 89%). ^{1}H NMR (500 MHz, $CDCl_3$) δ 7.68-7.66 (m, 4H), 7.43-7.33 (m, 8H), 7.02 (d, *J* = 7.9 Hz, 2H), 5.26 (d, *J* = 8.2 Hz, 1H, N*H*), 4.93 (d, *J* = 8.2 Hz, 1H, H-1), 4.72 (d, *J* = 12.0 Hz, 1H, CH_2CCl$_3$), 4.69 (d, *J* = 12.0 Hz, 1H, CH_2CCl$_3$), 4.40 (br, 1H, H-3), 4.28 (d, *J* = 4.9 Hz, 1H, H-4), 3.94-3.91 (m, 3H, H-5, H-6, H-6), 3.30 (ddd, *J* = 8.2, 8.2, 8.2 Hz, 1H, H-2), 2.28 (s, 3H, STol), 1.42 (s, 3H, CCH_3), 1.30 (s, 3H, CCH_3), 1.03 (s, 9H, C(CH_3)$_3$); ^{13}C NMR (100 MHz, $CDCl_3$) δ 153.94, 137.84, 135.60, 135.59, 133.29, 132.63, 129.68, 127.68, 127.61, 109.95, 95.37, 85.77, 77.04, 74.53, 72.97, 62.89, 55.68, 28.15, 26.74, 26.25, 21.08, 19.17; HRMS (ESI) *m/z* for $C_{35}H_{42}Cl_3NNaO_6SSi$ $[M+Na]^+$: calcd 760.1465, found 760.1466.

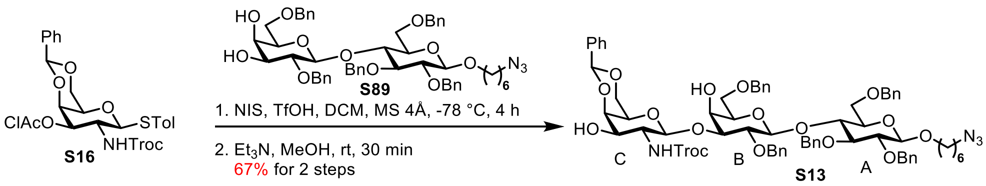

Compound S13. Compound **S13** was synthesized from glycosyl acceptor **S89** (216 mg, 0.24 mmol) and glycosyl donor **S16** (113 mg, 0.18 mmol) by following the **General glycosylation procedure A**. Next, a solution of above residue in MeOH/Et_3N (4 mL/1 mL) was stirred at rt for 30 min. The reaction mixture was concentrated *in vacuo*. The residue was extracted with EtOAc, and the resulting organic solution was washed with saturated $NaHCO_{3(aq)}$ and brine. The organic layer was dried over $MgSO_4$, filtered, and concentrated. The residue was purified by silica gel chromatography (EtOAc/Hexanes

= 1/1, R$_f$ = 0.20) to afford compound **S13** (162 mg, 67% over 2 steps). ^{1}H NMR (700 MHz, $CDCl_3$) δ 7.49-7.48 (m, 2H), 7.40-7.20 (m, 28H), 5.54 (s, 1H, C*H*Ph), 4.98 (d, *J* = 11.0 Hz, 1H, A_3-Bn), 4.88 (d, *J* = 12.0 Hz, 1H, B_2-Bn), 4.86 (d, *J* = 11.1 Hz, 1H, A_2-Bn), 4.73 (d, *J* = 11.0 Hz, 1H, A_3-Bn), 4.72 (d, *J* = 11.1 Hz, 1H, A_2-Bn), 4.66-4.63 (m, 2H, C-1, Troc), 4.65 (d, *J* = 12.0 Hz, 1H, B_2-Bn), 4.60 (d, *J* = 12.1 Hz, 1H, A_6-Bn), 4.50 (d, *J* = 11.9 Hz, 1H, Troc), 4.47 (d, *J* = 12.0 Hz, 1H, B_6-Bn), 4.46 (d, *J* = 8.2 Hz, 1H, B-1), 4.43 (d, *J* = 12.1 Hz, 1H, A_6-Bn), 4.36 (d, *J* = 12.0 Hz, 1H, B_6-Bn), 4.32 (d, *J* = 7.8 Hz, 1H, A-1), 4.27 (d, *J* = 12.4 Hz, 1H, C-6), 4.12 (d, *J* = 3.6 Hz, 1H, C-4), 4.07 (d, *J* = 2.0 Hz, 1H, B-4), 4.05 (d, *J* = 12.4 Hz, 1H, C-6), 3.99 (dd, *J* = 9.1, 9.1 Hz, 1H, A-4), 3.88 (td, *J* = 9.6, 6.3 Hz, 1H, OC*H*$_2$CH$_3$), 3.77 (dd, *J* = 11.1, 4.0 Hz, 1H, A-6), 3.72-3.69 (m, 3H, A-6, B-6, C-2), 3.66 (dd, *J* = 9.2, 8.2 Hz, 1H, B-2), 3.58 (dd, *J* = 9.2, 2.0 Hz, 1H, B-3), 3.54 (dd, *J* = 9.1, 9.1 Hz, 1H, A-3), 3.51 (dd, *J* = 9.6, 5.5 Hz, 1H, B-6), 3.47 (td, *J* = 9.6, 6.3 Hz, 1H, OC*H*$_2$CH$_3$), 3.45-3.41 (m, 2H, B-5, C-3), 3.39 (br, 1H, C-5), 3.37 (dd, *J* = 9.1, 7.8 Hz, 1H, A-2), 3.32 (dd, *J* = 9.8, 4.0 Hz, 1H, A-5), 3.19 (t, *J* = 7.0 Hz, 2H, C*H*$_2$N$_3$), 2.65 (m, 2H, B_4-OH, C_3-OH), 1.64-1.58 (m, 2H), 1.53 (quin, *J* = 7.0 Hz, 2H), 1.37-1.35 (m, 4H); ^{13}C NMR (175 MHz, $CDCl_3$) δ 154.98, 138.95, 138.80, 138.71, 138.38, 138.36, 137.22, 129.34, 128.57, 128.37, 128.31, 128.29, 128.27, 128.05, 127.85, 127.74, 127.64, 127.60, 127.52, 127.50, 127.46, 127.28, 127.21, 126.32, 103.60, 102.32, 101.32 (x2), 95.37, 82.72, 81.76, 80.40, 80.11, 76.28, 75.48, 75.12, 74.89, 74.78, 74.71, 74.51, 73.48, 73.28, 73.06, 70.79, 69.70, 69.01, 68.40, 68.33, 68.15, 66.60, 55.44, 51.34, 29.57, 28.75, 26.50, 25.71; HRMS (ESI) *m/z* for $C_{69}H_{79}Cl_3N_4NaO_{17}$ [M+Na]$^+$: calcd 1363.4404, found 1363.4394.

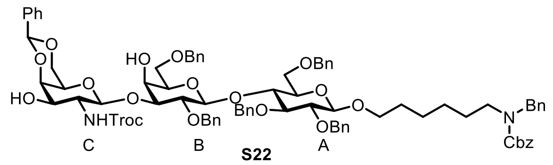

S22

Compound S22. Compound **S22** was synthesized from glycosyl acceptor **S17** (521 mg, 0.47 mmol) and glycosyl donor **S18** (331 mg, 0.56 mmol) by following the **General glycosylation procedure A**. Next, a solution of above residue in MeOH (20 mL) was added sodium methoxide (40 mg, 0.74 mmol) under a nitrogen atmosphere at 0 °C. After being stirred for 1 h, the reaction mixture was neutralized with Amberlite® IR-120 (H^+) resin and filtered off the resin. The resulting solution was concentrated *in vacuo*. The residue was purified by silica gel chromatography (EtOAc/Hexanes = 1/1, R_f = 0.15) to afford compound **S22** (525 mg, 73% over 2 steps). ^{1}H NMR (400 MHz, $CDCl_3$) δ 7.50-7.47 (m, 2H), 7.41-7.14 (m, 38H), 5.54 (s, 1H, C*H*Ph), 5.14 (br, 2H, Cbz), 4.98 (d, *J* = 10.6 Hz, 1H, A_3-Bn), 4.88 (d, *J* = 12.2 Hz, 1H, B_2-Bn), 4.85 (d, *J* = 11.0 Hz, 1H, A_2-Bn), 4.73 (d, *J* = 10.6 Hz, 1H, A_3-Bn), 4.69 (d, *J* = 11.0 Hz, 1H, A_2-Bn), 4.66-4.62 (m, 4H, N-Bn, B_2-Bn, C-1, Troc), 4.60 (d, *J* = 12.1 Hz, 1H, A_6-Bn), 4.50 (d, *J* = 12.2 Hz, 1H, Troc), 4.47-4.43 (m, 4H, N-Bn, B-1, B_6-Bn, N*H*), 4.42 (d, *J* = 12.1 Hz, 1H, A_6-Bn), 4.35 (d, *J* = 12.0 Hz, 1H, B_6-Bn), 4.29 (d, *J* = 8.0 Hz, 1H, A-1), 4.27 (d, *J* = 12.4 Hz, 1H, C-6), 4.13 (d, *J* = 3.6 Hz, 1H, C-4), 4.06 (d, *J* = 2.2 Hz, 1H, B-4), 4.05 (dd, *J* = 12.4, 2.2 Hz, 1H, C-6), 3.99 (dd, *J* = 9.1, 9.1 Hz, 1H, A-4), 3.84-3.81 (m, 1H, OCH_2CH_2), 3.77 (dd, *J* = 11.2, 4.1 Hz, 1H, A-6), 3.72-3.64 (m, 4H, A-6, B-2, B-6, C-2), 3.57 (dd, *J* = 9.3, 2.2 Hz, 1H, B-3), 3.53 (dd, *J* = 9.1, 9.1 Hz, 1H, A-3), 3.51 (dd, *J* = 9.4, 5.6 Hz, 1H, B-6), 3.46-3.40 (m, 4H, OCH_2CH_2, B-5, C-3, C-5), 3.36 (dd, *J* = 9.1, 8.0 Hz, 1H, A-2), 3.31 (ddd, *J* = 9.1, 4.1, 1.7 Hz, 1H, A-5), 3.21-3.14 (m, 2H, CH_2NBnCbz), 1.71-1.20 (m, 8H); ^{13}C NMR (100 MHz, $CDCl_3$) δ 156.58, 156.00, 154.85, 138.84, 138.71, 138.56, 138.28, 138.24, 137.74, 137.22, 136.65, 129.13, 128.35, 128.33, 128.25, 128.15, 128.14, 128.10, 127.89, 127.75, 127.62, 127.49, 127.40, 127.35, 127.33, 127.30, 127.12, 126.21, 103.43, 102.08, 101.30, 100.97, 95.32, 82.55, 81.63, 80.79, 79.70, 76.08, 75.25, 74.94, 74.71, 74.61(x2), 74.26, 73.30, 73.05, 73.01, 70.25, 69.62, 68.82, 68.50, 68.20, 67.97, 66.98, 66.36, 55.15, 50.27, 49.98, 46.99,

46.08, 29.46, 27.87, 27.49, 26.41, 25.70 (conformational isomer peaks are underlined); HRMS (ESI) *m/z* for $C_{84}H_{93}Cl_3N_2NaO_{19}$ [M+Na]$^+$: calcd 1561.5336, found 1561.5331.

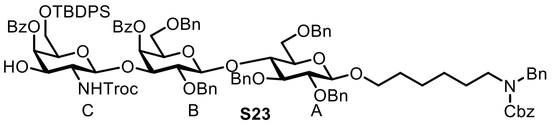

Compound S23. The synthesis of compound **S23** is similar as described in the synthesis of compound **S13**, starting from glycosyl acceptor **9** (231 mg, 0.19 mmol) and glycosyl donor **S19** (200 mg, 0.23 mmol) to yield compound **S23** (315 mg, 88% over 2 steps); EtOAc/Hexanes = 1/2, R_f = 0.20. ^{1}H NMR (400 MHz, $CDCl_3$) δ 8.09 (d, *J* = 7.2 Hz, 2H), 7.99 (d, *J* = 7.4 Hz, 2H), 7.68-7.66 (m, 2H), 7.60 (t, *J* = 7.4 Hz, 1H), 7.54-7.43 (m, 5H), 7.41-7.06 (m, 41H), 6.98 (t, *J* = 7.5 Hz, 2H), 5.71 (d, *J* = 3.1 Hz, 1H, C-4), 5.67 (d, *J* = 3.0 Hz, 1H, B-4), 5.19 (br, 1H, Cbz), 5.17 (br, 1H, Cbz), 5.04 (d, *J* = 10.6 Hz, 1H, A_3-Bn), 4.95 (d, *J* = 11.9 Hz, 1H, B_2-Bn), 4.88 (d, *J* = 10.0 Hz, 1H, A_2-Bn), 4.80 (d, *J* = 10.6 Hz, 1H, A_3-Bn), 4.79-4.65 (m, 5H, N-Bn, A_2-Bn, A_6-Bn, B_2-Bn, Troc), 4.53 (d, *J* = 7.6 Hz, 1H, C-1), 4.50-4.35 (m, 6H, N-Bn, A_6-Bn, B-1, B_6-Bn, N*H*, Troc), 4.22 (br, 1H, A-1), 4.18 (d, *J* = 11.9 Hz, 1H, B_6-Bn), 4.05 (dd, *J* = 9.4, 9.4 Hz, 1H, A-4), 3.89-3.74 (m, 7H, OCH_2CH$_2$, A-6, A-6, B-6, C-2, C-6, C-6), 3.70 (d, *J* = 8.5 Hz, 1H, B-3), 3.69 (dd, *J* = 8.5, 8.5 Hz, 1H, B-2), 3.59 (t, *J* = 9.0 Hz, 1H), 3.58 (t, *J* = 6.0 Hz, 1H), 3.49-3.46 (m, 1H, OCH_2CH$_2$), 3.43-3.32 (m, 5H, A-2, A-5, B-5, B-6, C-3), 3.26 (t, *J* = 6.6 Hz, 1H, CH_2NBnCbz), 3.18 (t, *J* = 6.6 Hz, 1H, CH_2NBnCbz), 3.01 (br, 1H, C_3-OH), 161-1.47 (m, 4H), 1.37-1.22 (m, 4H), 1.01 (s, 9H, C(CH_3)$_3$); ^{13}C NMR (100 MHz, $CDCl_3$) δ 166.58, 165.01, 156.68, 156.11, 154.51, 138.83, 138.58, 138.38, 138.12, 138.01, 137.85, 136.71, 135.35, 133.17, 132.81, 132.76, 132.53, 130.16, 129.72, 129.68, 129.61, 129.37, 128.52, 128.45, 128.38, 128.28, 128.20, 128.14, 127.87, 127.82, 127.76, 127.74, 127.60, 127.58, 127.53, 127.48, 127.33, 127.12, 126.98, 103.55, 102.23, 100.85, 95.22, 82.58, 81.47, 80.48, 77.20, 76.28, 75.21, 74.92, 74.87, 74.76, 74.23, 73.50, 73.43, 73.33, 72.90, 70.84, 70.27, 69.78, 69.28, 68.06,

67.94, 67.07, 61.17, 56.06, 50.36, 50.05, 47.07, 46.11, 29.56, 27.98, 27.59, 26.59, 26.54, 25.82, 18.94 (conformational isomer peaks are underlined); HRMS (ESI) *m/z* for $C_{107}H_{114}Cl_3N_2O_{21}Si$ [M-H]$^-$: calcd 1895.6749, found 1895.6703.

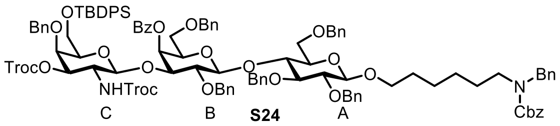

Compound S24. Compound **S24** was synthesized from glycosyl acceptor **9** (1.30 g, 1.07 mmol) and glycosyl donor **S20** (1.54 g, 1.60 mmol) by following the **General glycosylation procedure A**. The mixture was purified by silica gel chromatography (EtOAc/Hexanes = 1/3, R_f = 0.23) to afford compound **S24** (2.08 g, 95%). ^{1}H NMR (400 MHz, $CDCl_3$) δ 7.93 (d, *J* = 6.8 Hz, 2H), 7.72-7.70 (m, 2H), 7.65 (d, *J* = 7.0 Hz, 2H), 7.53 (t, *J* = 7.7 Hz, 1H), 7.48-7.24 (m, 45H), 7.09 (t, *J* = 7.4 Hz, 1H), 6.98 (t, *J* = 7.5 Hz, 2H), 5.62 (d, *J* = 3.1 Hz, 1H, B-4), 5.22 (br, 2H, Cbz), 5.04 (d, *J* = 10.6 Hz, 2H), 4.90 (d, *J* = 11.1 Hz, 2H), 4.81 (d, *J* = 10.6 Hz, 1H), 4.75-4.60 (m, 7H), 4.58 (d, *J* = 11.3 Hz, 1H), 4.53-4.51 (m, 3H), 4.44-4.37 (m, 4H), 4.22-4.16 (m, 3H), 4.04 (dd, *J* = 9.0, 9.0 Hz, 1H, A-4), 3.90-3.86 (m, 3H), 3.82-3.79 (m, 3H), 3.73-3.67 (m, 3H), 3.62-3.56 (m, 2H), 3.60 (t, *J* = 9.1 Hz, 1H), 3.51-3.45 (m, 1H), 3.43-3.39 (m, 2H), 3.39-3.30 (m, 2H), 3.30-3.21 (m, 2H), 1.65-1.50 (m, 4H), 1.43-1.24 (m, 4H), 1.11 (s, 9H, $C(CH_3)_3$); ^{13}C NMR (100 MHz, $CDCl_3$) δ 164.98, 156.56, 156.01, 153.34(x2), 138.75, 138.52, 138.01, 137.78, 136.68, 135.47, 135.39, 132.87, 132.58, 129.93, 129.78, 129.67, 128.36, 128.28, 128.20, 128.10, 128.06, 127.76, 127.67, 127.61, 127.55, 127.46, 127.37, 127.27, 127.24, 126.99, 126.91, 103.46, 102.12, 99.52, 95.36, 94.21, 82.52, 81.40, 80.39, 77.20, 76.55, 76.29, 75.10, 74.86 (x2), 74.76, 74.69, 74.19, 73.88, 73.40, 73.39, 73.17, 72.92, 72.79, 69.63, 69.54, 68.15, 67.90, 66.98, 61.25, 53.38, 50.29, 49.99, 46.99, 46.03, 29.46, 27.89, 27.51, 26.78 (x3), 26.42, 25.72, 19.01 (conformational isomer peaks are underlined); HRMS (ESI) *m/z* for $C_{110}H_{118}Cl_6N_2NaO_{22}Si$ [M+Na]$^+$: calcd 2079.5974, found 2079.5971.

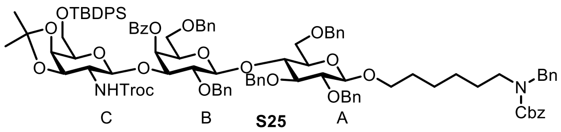

S25

Compound S25. Compound **S25** was synthesized from glycosyl acceptor **9** (244 mg, 0.20 mmol) and glycosyl donor **S21** (296 mg, 0.40 mmol) by following the **General glycosylation procedure A**. The mixture was purified by silica gel chromatography (EtOAc/Hexanes = 1/3, R_f = 0.33) to afford compound **S25** (296 mg, 88%). ^{1}H NMR (500 MHz, $CDCl_3$) δ 8.01 (d, J = 5.0 Hz, 2H), 7.78-7.77 (m, 4H), 7.54 (t, J = 7.0 Hz, 1H), 7.49-7.21 (m, 40H), 7.12 (t, J = 7.3 Hz, 1H), 7.03 (t, J = 7.5 Hz, 2H), 5.66 (br, 1H, B-4), 5.23 (br, 1H, Cbz), 5.22 (br, 1H, Cbz), 5.08 (d, J = 10.7 Hz, 1H), 5.02 (d, J = 10.7 Hz, 1H), 4.92 (d, J = 10.6 Hz, 1H), 4.84 (d, J = 10.7 Hz, 1H), 4.77 (d, J = 11.1 Hz, 1H), 4.75-4.68 (m, 3H), 4.59 (d, J = 7.4 Hz, 1H), 4.54-4.50 (m, 3H), 4.40 (d, J = 11.6 Hz, 2H), 4.34 (br, 1H), 4.26-4.22 (m, 3H), 4.10 (dd, J = 9.2, 9.2 Hz, 1H, A-4), 4.03-4.02 (m, 2H), 3.95-3.86 (m, 3H), 3.83-3.78 (m, 3H), 3.71 (dd, J = 9.2, 9.2 Hz, 1H, A-3), 3.65 (t, J = 9.0 Hz, 2H), 3.50 (br, 1H, OCH_2CH_2), 3.46-3.36 (m, 5H), 3.30 (t, J = 6.0 Hz, 1H, CH_2NBnCbz), 3.22 (t, J = 6.0 Hz, 1H, CH_2NBnCbz), 1.68-1.51 (m, 4H), 1.51 (s, 3H, CCH_3), 1.44-1.26 (m, 4H), 1.34 (s, 3H, CCH_3), 1.12 (s, 9H, $C(CH_3)_3$); ^{13}C NMR (125 MHz, $CDCl_3$) δ 165.01, 156.67, 156.10, 153.77, 138.82, 138.68, 138.60, 138.13, 137.97, 137.87, 136.81, 135.53, 135.51, 133.25, 133.19, 132.77, 129.71, 129.66, 128.59, 128.44, 128.36, 128.30, 128.18, 128.15, 127.85, 127.83, 127.74, 127.65, 127.61, 127.57, 127.45, 127.33, 127.11, 109.91, 103.55, 102.29, 100.18, 95.38, 82.58, 81.47(x2), 77.20, 76.40, 76.19, 75.18, 75.02, 74.85(x2), 74.80, 74.23, 73.46, 73.42, 73.32, 72.98, 72.57, 70.06, 69.76, 68.05, 67.06, 62.37, 56.35, 50.38, 50.06, 47.07, 46.12, 29.55, 28.02 (x2), 27.59, 26.69 (x3), 26.52, 26.19, 25.81, 19.16 (conformational isomer peaks are underlined); HRMS (ESI) *m/z* for $C_{103}H_{114}Cl_3N_2O_{20}Si$ $[M-H]^-$: calcd 1831.6800, found 1831.6822.

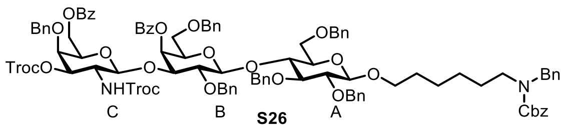

Compound S26. Compound **S26** was synthesized from glycosyl acceptor **9** (178 mg, 0.15 mmol) and glycosyl donor **8** (157 mg, 0.19 mmol) by following the **General glycosylation procedure A**. The mixture was purified by silica gel chromatography (EtOAc/Hexanes = 1/3, R_f = 0.28) to afford compound **S26** (251 mg, 89%). ^{1}H NMR (500 MHz, $CDCl_3$) δ 8.03 (d, *J* = 7.4 Hz, 2H), 7.96 (d, *J* = 7.5 Hz, 2H), 7.56-7.51 (m, 2H), 7.41 (t, *J* = 7.8 Hz, 2H), 7.38-7.16 (m, 39H), 7.07 (t, *J* = 7.2 Hz, 1H), 6.97 (t, *J* = 7.4 Hz, 2H), 5.70 (d, 2.9 Hz, 1H, B-4), 5.17 (br, 1H, Cbz), 5.16 (br, 1H, Cbz), 5.03 (d, *J* = 10.7 Hz, 1H), 4.95-4.93 (m, 1H), 4.88-4.86 (m, 2H), 4.78 (d, *J* = 10.5 Hz, 1H), 4.77 (d, *J* = 11.6 Hz, 1H), 4.72-4.65 (m, 5H), 4.61-4.56 (m, 3H), 4.53 (d, *J* = 7.6 Hz, 1H), 4.47 (d, *J* = 11.1 Hz, 2H), 4.42-4.33 (m, 5H), 4.23 (d, *J* = 11.9 Hz, 1H), 4.14-4.11 (m, 1H), 4.04 (dd, *J* = 9.0, 9.0 Hz, 1H, A-4), 3.98 (br, 1H), 3.88-3.74 (m, 4H), 3.83 (dd, *J* = 9.6, 3.3 Hz, 1H), 3.70 (t, *J* = 9.0 Hz, 2H), 3.58 (t, *J* = 9.1 Hz, 2H), 3.46-3.33 (m, 6H), 3.25-3.17 (m, 2H, CH_2NBnCbz), 1.60-1.46 (m, 4H), 1.36-1.23 (m, 4H); ^{13}C NMR (125 MHz, $CDCl_3$) δ 165.78, 165.08, 156.56, 155.98, 153.32, 138.74, 138.50, 138.38, 138.03, 137.96, 137.75, 137.09, 136.67, 136.64, 133.11, 132.70, 130.01, 129.68, 129.50, 129.43, 128.35, 128.29, 128.23, 128.21, 128.10, 128.08, 127.74, 127.69, 127.67, 127.64, 127.57, 127.47, 127.36, 127.26, 127.10, 126.99, 103.43, 102.11, 100.01, 95.34, 94.08, 82.51, 81.37, 80.23, 77.82, 76.62, 76.21, 75.07, 74.82, 74.74, 74.71, 74.68, 73.85, 73.42, 73.16, 72.96, 72.19, 71.64, 70.00, 69.61, 68.16, 67.90, 66.97, 66.94, 62.12, 53.11, 50.29, 49.96, 46.99, 46.03, 29.44, 27.88, 27.48, 26.40, 25.69 (conformational isomer peaks are underlined); HRMS (ESI) *m/z* for $C_{101}H_{104}Cl_6N_2NaO_{23}$ $[M+Na]^+$: calcd 1945.5059, found 1945.5087.

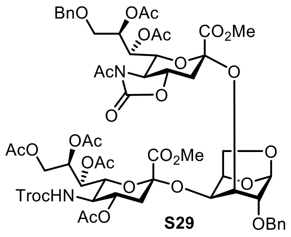

Compound S29. To a solution of compound **28** (278 mg, 0.21 mmol) in TFA/DCM/H_2O (0.5 mL/4 mL/1 mL) was stirred at 0 °C. After being stirred for 2.5 h, the reaction mixture was quenched with Et_3N and concentrated *in vacuo*. The residue was extracted with EtOAc, and the resulting organic solution was washed with saturated $NaHCO_{3(aq)}$ and brine. The organic layer was dried over $MgSO_4$, filtered, and concentrated. To a solution of above residue in Ac_2O/pyridine (0.5 mL/1 mL) was added 4-(dimethylamino)pyridine (5 mg, 0.04 mmol) at rt. The reaction mixture was stirred at rt for 2 h. The residue was extracted with EtOAc, and the resulting organic solution was washed with 1N $HCl_{(aq)}$, saturated $NaHCO_{3(aq)}$, and brine. The organic layer was dried over $MgSO_4$, filtered, and concentrated. The residue was purified by silica gel chromatography (EtOAc/Hexanes = 1/1, R_f = 0.23) to afford compound **S29** (255 mg, 89% over 2 steps). ^{1}H NMR (500 MHz, $CDCl_3$) δ 7.32 (d, *J* = 7.1 Hz, 2H), 7.27-7.21 (m, 6H), 7.13-7.12 (m, 2H), 5.80 (d, *J* = 8.9 Hz, 1H, C-7), 5.35 (ddd, *J* = 8.9, 4.7, 4.3 Hz, 1H, C-8), 5.31-5.30 (m, 1H, B-7), 5.31 (br, 1H, A-1), 5.24-5.21 (m, 1H, B-8), 5.15 (ddd, *J* = 11.6, 11.6, 4.9 Hz, 1H, B-4), 5.00 (d, *J* = 11.6 Hz, 1H, B_5-NH), 4.89 (d, *J* = 12.1 Hz, 1H, Troc), 4.77 (d, *J* = 11.7 Hz, 1H, CH_2Ph), 4.55 (d, *J* = 11.7 Hz, 1H, CH_2Ph), 4.47 (d, *J* = 12.1 Hz, 1H, Troc), 4.45 (d, *J* = 4.4 Hz, 1H, A-3), 4.42 (dd, *J* = 4.4, 4.4 Hz, 1H, A-4), 4.35 (d, *J* = 11.7 Hz, 1H, CH_2Ph), 4.31 (d, *J* = 7.2 Hz, 1H, A-6), 4.29 (dd, *J* = 4.4, 4.4 Hz, 1H, A-5), 4.25 (dd, *J* = 12.5, 2.5 Hz, 1H, B-9), 4.24 (d, *J* = 11.7 Hz, 1H, CH_2Ph), 4.20 (d, *J* = 9.8 Hz, 1H, C-6), 4.11 (dd, *J* = 12.5, 4.9 Hz, 1H, B-9), 4.00 (d, *J* = 11.6 Hz, 1H, B-6), 3.87 (ddd, *J* = 13.7, 11.0, 3.5 Hz, 1H, C-4), 3.79 (s, 3H, OCH_3), 3.74 (br, 1H, A-2), 3.73 (s, 3H, OCH_3), 3.61 (dd, *J* = 11.0, 9.8 Hz, 1H, C-

5), 3.58 (dd, J = 7.2, 4.4 Hz, 1H, A-6), 3.54 (dd, J = 10.4, 4.3 Hz, 1H, C-9), 3.48 (ddd, J = 11.6, 11.6, 11.6 Hz, 1H, B-5), 3.36 (dd, J = 10.4, 4.7 Hz, 1H, C-9), 3.07 (dd, J = 11.9, 3.5 Hz, 1H, C-3_{eq}), 2.53 (dd, J = 13.1, 4.9 Hz, 1H, B-3_{eq}), 2.47 (s, 3H, Ac), 2.10-2.08 (m, 1H, C-3_{ax}), 2.08 (s, 3H, Ac), 2.07 (s, 3H, Ac), 2.06 (s, 3H, Ac), 2.02 (s, 3H, Ac), 1.99 (s, 3H, Ac), 1.96 (s, 3H, Ac), 1.85 (dd, J = 13.1, 11.6 Hz, 1H, B-3_{ax}); ^{13}C NMR (125 MHz, $CDCl_3$) δ 171.63, 170.52, 170.23, 170.22, 169.64 (x2), 169.54, 167.61, 166.98, 153.83, 153.71, 138.10, 137.70, 128.31, 128.19, 127.88, 127.62, 127.52, 127.28, 99.85, 99.68, 99.38, 95.41, 80.38, 75.68, 74.84, 74.36, 74.24, 73.29, 72.62, 72.52, 71.90, 71.78, 69.43, 68.88, 68.55, 68.13, 67.30, 67.02, 64.39, 61.77, 59.16, 53.00, 52.90, 51.56, 36.91, 35.29, 24.69, 21.21, 20.94, 20.86 (x2), 20.65, 20.61; HRMS (ESI) *m/z* for $C_{58}H_{69}Cl_3N_2NaO_{29}$ $[M+Na]^+$: calcd 1385.2949, found 1385.2942.

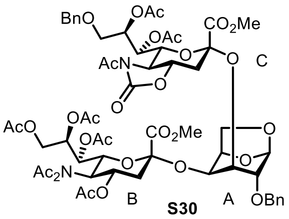

S30

Compound S30. To a solution of compound **S29** (250 mg, 0.18 mmol) in ACN/AcOH (15 mL/3.6 mL) was added Ac_2O (3.4 ml, 36.64 mmol) and zinc dust (1.20 g, 18.30 mmol) at rt. After being stirred at rt for 15 h, the solid was filtered through a pad of celite®, and resulting solution was concentrated under reduced pressure. Next, a solution of above residue in DCM (18 mL) was added *N*,*N*-diisopropylethylamine (1.60 mL, 9.16 mmol), acetyl chloride (2.62 mL, 36.64 mmol), and 4-(dimethylamino)pyridine (22 mg, 0.18 mmol) under a nitrogen atmosphere at 0 °C. The reaction mixture was gradually warmed to rt. After being stirred for 2 h, the reaction mixture was concentrated *in vacuo*. The residue was extracted with EtOAc, and the resulting organic solution was washed with H_2O, saturated $NaHCO_{3(aq)}$, and brine. The organic layer was dried over $MgSO_4$, filtered, and concentrated. The residue was

purified by silica gel chromatography (EtOAc/Hexanes = 1/1 + 10% MeOH, R_f = 0.31) to afford compound **S30** (185 mg, 79% over 2 steps). ^{1}H NMR (500 MHz, $CDCl_3$) δ 7.34 (d, *J* = 6.6 Hz, 2H), 7.27-7.26 (m, 2H), 7.24-7.19 (m, 4H), 7.11-7.09 (m, 2H), 5.80 (d, *J* = 9.2 Hz, 1H, C-7), 5.57 (ddd, *J* = 10.2, 10.2, 5.3 Hz, 1H, B-4), 5.36 (ddd, *J* = 9.2, 4.7, 4.7 Hz, 1H, C-8), 5.30 (br, 1H, A-1), 5.16 (ddd, *J* = 7.4, 5.6, 2.8 Hz, 1H, B-8), 5.08 (dd, *J* = 7.4, 1.0 Hz, 1H, B-7), 4.81 (d, *J* = 11.7 Hz, 1H, CH_2Ph), 4.73 (dd, *J* = 10.2, 1.0 Hz, 1H, B-6), 4.55 (d, *J* = 11.7 Hz, 1H, CH_2Ph), 4.49-4.46 (m, 2H, A-3, A-4), 4.35-4.32 (m, 3H, A-5, A-6, CH_2Ph), 4.28 (dd, *J* = 12.4, 2.8 Hz, 1H, B-9), 4.23 (d, *J* = 11.7 Hz, 1H, CH_2Ph), 4.19 (d, *J* = 9.7 Hz, 1H, C-6), 4.09 (dd, *J* = 12.4, 5.6 Hz, 1H, B-9), 3.96 (dd, *J* = 10.2, 10.2 Hz, 1H, B-5), 3.86 (ddd, *J* = 13.2, 11.1, 3.5 Hz, 1H, C-4), 3.82 (s, 3H, OCH_3), 3.75 (br, 1H, A-2), 3.72 (s, 3H, OCH_3), 3.63 (dd, *J* = 11.1, 9.7 Hz, 1H, C-5), 3.60 (dd, *J* =6.6, 5.1 Hz, 1H, A-6), 3.55 (dd, *J* = 10.3, 4.7 Hz, 1H, C-9), 3.38 (dd, *J* = 10.3, 4.7 Hz, 1H, C-9), 3.08 (dd, *J* = 11.9, 3.5 Hz, 1H, C-3_{eq}), 2.63 (dd, *J* = 13.1, 5.3 Hz, 1H, B-3_{eq}), 2.48 (s, 3H, Ac), 2.34 (s, 3H, Ac), 2.29 (s, 3H, Ac), 2.09-2.05 (m, 1H, C-3_{ax}), 2.07 (s, 6H, Ac(x2)), 2.05 (s, 3H, Ac), 1.99 (s, 3H, Ac), 1.97 (s, 3H, Ac), 1.95 (s, 3H, Ac), 1.84 (dd, *J* = 13.1, 10.2 Hz, 1H, B-3_{ax}); ^{13}C NMR (125 MHz, $CDCl_3$) δ 174.39, 173.90, 171.56, 170.32, 170.02, 169.69, 169.58, 169.51, 169.38, 167.41, 166.78, 153.59, 138.00, 137.59, 128.23, 128.06, 127.79, 127.59, 127.41, 127.27, 99.75, 99.66, 99.44, 80.39, 75.60, 74.65, 74.29, 73.21, 72.67, 72.47, 71.80, 69.68, 69.19, 69.14, 68.95, 66.94, 66.76 (x2), 64.33, 61.46, 59.04, 57.20, 52.96, 52.86, 36.87, 35.75, 27.74, 25.51, 24.59, 21.15, 20.87, 20.72, 20.67, 20.55, 20.51; HRMS (ESI) *m/z* for $C_{59}H_{72}N_2NaO_{29}$ $[M+Na]^+$: calcd 1295.4118, found 1295.4106.

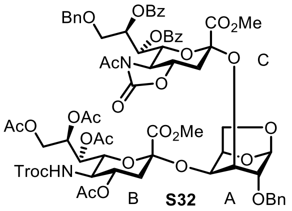

Compound S32. To a solution of compound **28** (1.64 g, 1.24 mmol) in TFA/DCM/H_2O (2.5 mL/25 mL/1.3 mL) was stirred at 0 °C. After being stirred for 3 h at 0 °C, the reaction mixture was quenched with Et_3N and concentrated *in vacuo*. The residue was extracted with EtOAc, and the resulting organic solution was washed with saturated $NaHCO_{3(aq)}$ and brine. The organic layer was dried over $MgSO_4$, filtered, and concentrated. To a solution of above residue in pyridine (20 mL) was added benzoic anhydride (2.601 g, 11.50 mmol) and 4-(dimethylamino)pyridine (70 mg, 0.58 mmol) under a nitrogen atmosphere at rt. The reaction was gradually warmed to 115 °C and stirred for 15 h. The reaction mixture was quenched with MeOH and concentrated *in vacuo*. The residue was extracted with EtOAc, and the resulting organic solution was washed with 1N $HCl_{(aq)}$, saturated $NaHCO_{3(aq)}$, and brine. The organic layer was dried over $MgSO_4$, filtered, and concentrated. The residue was purified by silica gel chromatography (EtOAc/Hexanes = 1/1, R_f = 0.48) to afford compound **S32** (465 mg, 25% over 2 steps). ^1H NMR (500 MHz, $CDCl_3$) δ 8.08 (d, *J* = 8.1 Hz, 4H), 7.61-7.56 (m, 2H), 7.47-7.43 (m, 4H), 7.27-7.25 (m, 2H), 7.18-7.17 (m, 3H), 7.06 (t, *J* = 7.1 Hz, 1H), 7.01 (t, *J* = 7.3 Hz, 2H), 6.95 (d, *J* = 7.2 Hz, 2H), 6.31 (d, *J* = 9.2 Hz, 1H, C-7), 5.81 (ddd, *J* = 9.2, 3.8, 3.8 Hz, 1H, C-8), 5.33 (br, 1H, A-1), 5.31 (d, *J* = 8.4 Hz, 1H, B-7), 5.23 (ddd, *J* = 8.4, 5.0, 2.4 Hz, 1H, B-8), 5.05 (ddd, *J* = 10.5, 10.5, 4.8 Hz, 1H, B-4), 4.92 (d, *J* = 10.5 Hz, 1H, B_5-NH), 4.88 (d, *J* = 12.1 Hz, 1H, CH_2Ph), 4.81 (d, *J* = 11.8 Hz, 1H, Troc), 4.60 (d, *J* = 11.8 Hz, 1H, Troc), 4.47 (m, 1H, A-3), 4.46 (d, *J* = 12.1 Hz, 1H, CH_2Ph), 4.42 (dd, *J* = 4.1, 4.1 Hz, 1H, A-4), 4.39 (d, *J* = 9.7 Hz, 1H, A-6), 4.37-4.35 (m, 2H, A-5, C-6), 4.34 (d, *J* = 12.0 Hz, 1H, CH_2Ph), 4.23 (dd, *J* = 12.5, 2.4 Hz, 1H, B-9), 4.19 (d, *J* = 12.0 Hz, 1H, CH_2Ph), 4.10 (d, *J* = 12.5, 5.0 Hz, 1H, B-9), 4.00 (d, *J* = 10.5 Hz, 1H, B-6), 3.97 (br, 1H, A-2), 3.94 (ddd, *J* = 12.3, 11.9, 3.5 Hz, 1H, C-4), 3.78 (s, 3H, OCH_3), 3.74 (dd, *J* = 10.7, 3.8 Hz, 1H, C-9), 3.71 (dd, *J* = 12.3, 8.8 Hz, 1H, C-5), 3.61-3.58 (m, 2H, A-6, C-9), 3.53 (ddd, *J* = 10.5, 10.5, 10.5 Hz, 1H,

B-5), 3.47 (s, 3H, OCH_3), 3.06 (dd, J = 11.9, 3.5 Hz, 1H, C-3$_{eq}$), 2.52 (dd, J = 14.3, 4.8 Hz, 1H, B-3$_{eq}$), 2.51 (s, 3H, Ac), 2.10 (dd, J = 11.9, 11.9 Hz, 1H, C-3$_{ax}$), 2.08 (s, 3H, Ac), 2.03 (s, 3H, Ac), 1.98 (s, 3H, Ac), 1.97 (s, 3H, Ac), 1.89 (dd, J = 14.3, 10.5 Hz, 1H, B-3$_{ax}$); ^{13}C NMR (125 MHz, $CDCl_3$) δ 171.53, 170.55, 170.30, 170.05, 169.57, 167.58, 167.19, 165.41, 165.12, 153.94, 153.66, 138.14, 137.56, 133.48, 133.19, 130.14, 129.99, 129.84, 129.58, 128.68, 128.51, 128.24, 128.00, 127.54, 127.49, 127.28, 127.22, 100.66, 99.93, 99.10, 95.38, 80.10, 75.61, 75.00, 74.43, 73.91, 73.26, 72.92, 72.50, 72.22, 72.01, 70.47, 69.01, 68.47, 68.25, 67.29, 67.17, 64.41, 61.78, 59.48, 53.04, 52.83, 51.61, 36.93, 35.68, 24.77, 20.91, 20.82, 20.67, 20.60; HRMS (ESI) *m/z* for $C_{68}H_{73}Cl_3N_2NaO_{29}$ $[M+Na]^+$: calcd 1509.3262, found 1509.3263

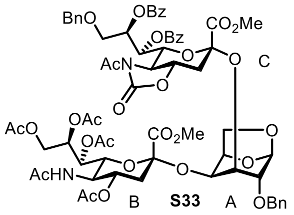

S33

Compound S33. To a solution of compound **S32** (465 mg, 0.31 mmol) in ACN/AcOH (5 mL/1.2 mL) was added Ac_2O (5.9 mL, 62.47 mmol) and zinc dust (408 mg, 6.25 mmol) at rt. After being stirred at rt for 30 min, the solid was filtered through a pad of celite®, and resulting solution was concentrated under reduced pressure. Next, a solution of above residue in DCM (6 mL) was added Ac_2O (240 μL, 2.50 mmol), pyridine (0.4 mL, 5.00 mmol), and 4-(dimethylamino)pyridine (38 mg, 0.31 mmol) under a nitrogen atmosphere at rt. After being stirred for 6 h, the reaction mixture was concentrated *in vacuo*. The residue was extracted with EtOAc, and the resulting organic solution was washed with 1N $HCl_{(aq)}$, saturated $NaHCO_{3(aq)}$, and brine. The organic layer was dried over $MgSO_4$, filtered, and concentrated. The residue was purified by silica gel chromatography (EtOAc/Hexanes = 1/1 + 10% MeOH, R_f = 0.44) to afford compound **S33** (361 mg, 85% over 2 steps). ^{1}H NMR (500 MHz, $CDCl_3$) δ 8.08-8.05

(m, 4H), 7.60-7.54 (m, 2H), 7.46-7.42 (m, 4H), 7.28-7.27 (m, 2H), 7.19-7.15 (m, 3H), 7.04 (t, *J* = 7.1 Hz, 1H), 6.99 (t, *J* = 7.3 Hz, 2H), 6.94 (d, *J* = 7.1 Hz, 2H), 6.32 (d, *J* = 9.2 Hz, 1H, C-7), 5.81 (ddd, *J* = 9.2, 4.2, 4.2 Hz, 1H, C-8), 5.36 (d, *J* = 9.7 Hz, 1H, B-7), 5.32 (br, 1H, A-1), 5.27-5.22 (m, 2H, B_5-NH, B-8), 4.89 (ddd, *J* = 11.6, 11.6, 4.6 Hz, 1H, B-4), 4.83 (d, *J* = 11.9 Hz, 1H, CH_2Ph), 4.60 (d, *J* = 11.9 Hz, 1H, CH_2Ph), 4.45 (br, 1H, A-3), 4.39-4.37 (m, 3H, A-4, A-5, C-6), 4.34 (d, *J* = 5.9 Hz, 1H, A-6), 4.32 (d, *J* = 12.0 Hz, 1H, CH_2Ph), 4.25 (dd, *J* = 12.2, 1.7 Hz, 1H, B-9), 4.17 (d, *J* = 12.0 Hz, 1H, CH_2Ph), 4.08 (dd, *J* = 12.2, 5.3 Hz, 1H, B-9), 3.99 (br, 1H, A-2), 3.97-3.91 (m, 2H, B-5, C-4), 3.87 (d, *J* = 10.8 Hz, 1H, B-6), 3.77 (s, 3H, OCH_3), 3.74-3.69 (m, 2H, C-5, C-9), 3.60-3.56 (m, 2H, A-6, C-9), 3.48 (s, 3H, OCH_3), 3.08 (dd, *J* = 11.9, 3.3 Hz, 1H, C-3_{eq}), 2.50 (s, 3H, Ac), 2.48 (dd, *J* = 12.8, 4.5 Hz, 1H, B-3_{eq}), 2.09-2.04 (m, 1H, C-3_{ax}), 2.07 (s, 3H, Ac), 2.01 (s, 3H, Ac), 1.98 (s, 3H, Ac), 1.97-1.92 (m, 1H, B-3_{ax}), 1.95 (s, 3H, Ac), 1.84 (s, 3H, Ac); ^{13}C NMR (125 MHz, $CDCl_3$) δ 171.40, 170.78, 170.47, 170.18, 169.85, 169.75, 167.76, 167.07, 165.36, 165.01, 153.65, 138.12, 137.49, 133.39, 133.14, 130.03, 129.90, 129.76, 129.54, 128.60, 128.46, 128.16, 127.91, 127.47, 127.37, 127.18, 127.09, 100.76, 99.73, 99.06, 80.02, 75.61, 74.83, 73.56, 73.17, 73.07, 72.73, 72.40, 72.07, 70.43, 68.96, 68.90, 68.83, 67.24, 66.89, 64.30, 61.89, 59.48, 52.95, 52.85, 49.32, 36.89, 35.77, 24.70, 23.05, 20.91, 20.75, 20.58, 20.55; HRMS (ESI) *m/z* for $C_{67}H_{74}N_2NaO_{28}$ $[M+Na]^+$: calcd 1377.4326, found 1377.4359.

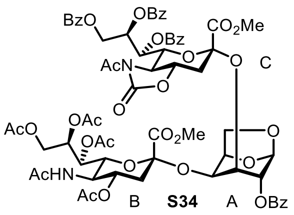

S34

Compound S34. To a solution of compound **S33** (196 mg, 0.15 mmol) in EtOAc (20 mL) was stirred under H_2 atmosphere in the presence of palladium on carbon (40 mg, 10 wt%) at rt. After being stirred for 1 h, the solid was filtered through a pad of celite®,

and resulting solution was concentrated under reduced pressure. Next, to a solution of above residue in pyridine/DMF (7 mL/7 mL) was added benzoic anhydride (3.27 g, 14.46 mmol) and 4-(dimethylamino)pyridine (18 mg, 0.15 mmol) at rt. The reaction mixture was gradually warmed to 80 °C and stirred for 24 h. The reaction was quenched with MeOH and concentrated *in vacuo*. The residue was extracted with EtOAc, and the resulting organic solution was washed with 1N $HCl_{(aq)}$, saturated $NaHCO_{3(aq)}$, and brine. The organic layer was dried over $MgSO_4$, filtered, and concentrated. The residue was purified by silica gel chromatography (Acetone/Hexanes = 2/3, three times, R_f = 0.35) to afford compound **S34** (162 mg, 81% over 2 steps). ^{1}H NMR (500 MHz, $CDCl_3$) δ 8.28 (d, *J* = 7.4 Hz, 2H), 8.13 (d, *J* = 7.4 Hz, 2H), 7.94 (d, *J* = 7.4 Hz, 2H), 7.74 (d, *J* = 7.4 Hz, 2H), 7.61 (t, *J* = 7.4 Hz, 1H), 7.52 (t, *J* = 7.4 Hz, 2H), 7.47 (t, *J* = 7.4 Hz, 1H), 7.42 (t, *J* = 7.4 Hz, 1H), 7.35 (t, *J* = 7.4 Hz, 2H), 7.30 (t, *J* = 7.4 Hz, 1H), 7.26 (t, *J* = 7.4 Hz, 2H), 7.22 (t, *J* = 7.4 Hz, 2H), 6.47 (d, *J* = 9.2 Hz, 1H, C-7), 5.78 (ddd, *J* = 9.2, 5.6, 2.3 Hz, 1H, C-8), 5.57 (br, 2H, A-1, A-2), 5.42 (ddd, *J* = 8.7, 5.6, 2.7 Hz, 1H, B-8), 5.36 (dd, *J* = 8.7, 1.7 Hz, 1H, B-7), 5.19 (d, *J* = 10.5 Hz, 1H, B_5-NH), 4.90 (ddd, *J* = 11.9, 10.5, 4.6 Hz, 1H, B-4), 4.60 (d, *J* = 4.8 Hz, 1H, A-3), 4.57 (t, *J* = 4.8 Hz, 1H, A-4), 4.53 (d, *J* = 9.8 Hz, 1H, C-6), 4.46 (dd, *J* = 12.4, 2.3 Hz, 1H, C-9), 4.42 (d, *J* = 7.2 Hz, 1H, A-6), 4.34 (dd, *J* = 12.5, 2.7 Hz, 1H, B-9), 4.29-4.26 (m, 1H, A-5), 4.26 (dd, *J* = 12.4, 5.6 Hz, 1H, C-9), 4.23 (dd, *J* = 12.5, 5.6 Hz, 1H, B-9), 4.04 (ddd, *J* = 10.5, 10.5, 10.5 Hz, 1H, B-5), 3.95-3.89 (m, 2H, B-6, C-4), 3.80 (s, 3H, OCH_3), 3.68-3.63 (m, 2H, A-6, C-5), 3.51 (s, 3H, OCH_3), 3.13 (dd, *J* = 11.7, 3.4 Hz, 1H, C-3_{eq}), 2.58 (dd, *J* = 12.8, 4.6 Hz, 1H, B-3_{eq}), 2.51 (s, 3H, Ac), 2.16 (s, 3H, Ac), 2.10 (dd, *J* = 11.7, 11.7 Hz, 1H, C-3_{ax}), 2.04 (dd, *J* = 12.8, 11.9 Hz, 1H, B-3_{ax}), 2.02 (s, 6H, Ac(x2)), 1.90 (s, 3H, Ac), 1.88 (s, 3H, Ac); ^{13}C NMR (125 MHz, $CDCl_3$) δ 170.90, 170.79, 170.67, 170.35, 169.65, 169.63, 167.95, 166.89, 165.83, 165.71, 165.56, 164.64, 153.52, 133.62, 133.09, 133.00, 132.59, 130.43, 130.15, 130.12, 129.69, 129.64, 129.06,

129.04, 128.67, 128.43, 128.17, 128.06, 100.71, 98.69, 98.68, 75.81, 74.27, 73.70, 73.32, 73.27, 72.66, 71.57, 69.05, 68.72, 68.32, 66.88, 66.45, 64.46, 62.96, 61.77, 59.54, 53.06 (x2), 49.35, 36.69, 36.55, 24.73, 23.18, 21.10, 20.84, 20.74, 20.51; HRMS (ESI) *m/z* for $C_{67}H_{70}N_2NaO_{30}$ $[M+Na]^+$: calcd 1405.3911, found 1405.3903.

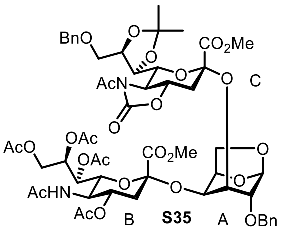

S35

Compound S35. The synthesis of compound **S35** is similar as described in the synthesis of compound **S33**, starting from compound **28** (75 mg, 0.06 mmol) to yield compound **S35** (65 mg, 96%); EtOAc/Hexanes = 1/1 + 10% MeOH, R_f = 0.50. ^{1}H NMR (400 MHz, $CDCl_3$) δ7.38-7.36 (m, 2H), 7.30-7.22 (m, 8H), 5.30-5.26 (m, 4H, A-1, B_5-NH, B-7, B-8), 4.94-4.87 (m, 2H, B-4, C-7), 4.76 (d, *J* =11.9 Hz, 1H, C*H*2Ph), 4.64 (d, *J* =11.9 Hz, 1H, C*H*2Ph), 4.49 (d, *J* =11.9 Hz, 1H, C*H*2Ph), 4.47-4.43 (m, 1H, C-8), 4.45 (d, *J* =11.9 Hz, 1H, C*H*2Ph), 4.33 (dd, *J* = 4.2, 4.2 Hz, 1H, A-4), 4.31-4.26 (m, 3H, A-5, A-6, B-9), 4.16-4.14 (m, 2H, A-2, A-3), 4.08 (dd, *J* = 12.4, 5.6 Hz, 1H, B-9), 3.99 (ddd, *J* = 10.3, 10.3, 10.3 Hz, 1H, B-5), 3.96-3.84 (m, 5H, B-6, C-5, C-6, C-9, C-9), 3.81-3.80 (m, 1H, C-4), 3.81 (s, 3H, OC*H*3), 3.80 (s, 3H, OC*H*3), 3.55 (dd, *J* = 6.0, 6.0 Hz, 1H, A-6), 3.05 (dd, *J* = 11.8, 3.4 Hz, 1H, C-3_{eq}), 2.54 (dd, *J* = 12.7, 4.7 Hz, 1H, B-3_{eq}), 2.42 (s, 3H, Ac), 2.13-2.02 (m, 1H, C-3_{ax}), 2.09 (s, 3H, Ac), 2.07 (s, 3H, Ac), 2.02 (s, 3H, Ac), 1.97-1.90 (m, 1H, B-3_{ax}), 1.93 (s, 3H, Ac), 1.86 (s, 3H, Ac), 1.51 (s, 3H, CC*H*3), 1.36 (s, 3H, CC*H*3); ^{13}C NMR (100 MHz, $CDCl_3$) δ 172.42, 170.66, 170.34, 170.24, 169.79, 169.65, 167.76, 167.16, 153.87, 138.52, 137.80, 128.27, 128.09, 127.67, 127.62, 127.45, 127.40, 108.63, 101.77, 99.26, 98.84, 77.96, 76.32, 76.10, 75.57, 75.06, 74.54, 73.34, 73.07, 72.80, 71.57, 69.17, 68.89, 68.53, 67.36, 66.60, 64.20, 61.77, 60.16, 52.94, 52.84, 49.13, 37.11, 36.09, 26.79, 25.13, 24.93, 22.93, 20.81, 20.70,

20.51, 20.47; HRMS (ESI) *m/z* for $C_{56}H_{70}N_2NaO_{26}$ $[M+Na]^+$: calcd 1209.4115, found 1209.4112.

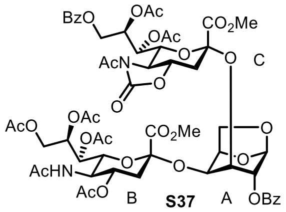

S37

Compound S37. To a solution of compound **S35** (21 mg, 18 μmol) in THF (2 mL) was added *N*,*N*-diisopropylethylamine (18 μL, 0.11 mmol), di-tert-butyldicarbonate (12 μL, 53 μmol), and 4-(dimethylamino)pyridine (2 mg, 18 μmol) under a nitrogen atmosphere at rt. After being stirred for 14 h, the reaction mixture was concentrated *in vacuo*. The residue was extracted with EtOAc, and the resulting organic solution was washed with saturated $NaHCO_{3(aq)}$ and brine. The organic layer was dried over $MgSO_4$, filtered, and concentrated. The residue was purified by silica gel chromatography (EtOAc/Hexanes = 1/1, R_f = 0.20) to afford compound **S36** (23 mg, 95%). Next, to a solution of compound **S36** (30 mg, 23 μmol) in EtOAc (2 mL) was stirred under H_2 atmosphere in the presence of palladium on carbon (30 mg, 10 wt%) at rt. After being stirred for 30 min, the solid was filtered through a pad of celite®, and resulting solution was concentrated under reduced pressure. Next, to a solution of above residue in pyridine/DMF (2 mL/2 mL) was added benzoyl chloride (135 μL, 1.17mmol) and 4-(dimethylamino)pyridine (3 mg, 23 μmol) at rt. The reaction mixture was gradually warmed to 80 °C and stirred for 15 h. The reaction was quenched with MeOH and concentrated *in vacuo*. The residue was extracted with EtOAc, and the resulting organic solution was washed with 1N $HCl_{(aq)}$, saturated $NaHCO_{3(aq)}$, and brine. The organic layer was dried over $MgSO_4$, filtered, and concentrated. Next, to a solution of above residue in DCM/TFA/H_2O (10 mL/4 mL/ 0.5 mL) was stirred at rt for 21 h. The reaction mixture was extracted with EtOAc, and the resulting organic solution was washed with

H_2O, saturated $NaHCO_{3(aq)}$, and brine. The organic layer was dried over $MgSO_4$, filtered, and concentrated. Next, to a solution of above residue in DCM/pyridine (2 mL/ 0.5mL) was added Ac_2O (13 μL, 0.14 mmol) and 4-(dimethylamino)pyridine (3 mg, 23 μmol) at rt. After being stirred for 2 h, the reaction mixture was concentrated *in vacuo*. The residue was extracted with EtOAc, and the resulting organic solution was washed with 1N $HCl_{(aq)}$, saturated $NaHCO_{3(aq)}$, and brine. The organic layer was dried over $MgSO_4$, filtered, and concentrated. The residue was purified by silica gel chromatography (EtOAc/Hexanes = 1/1 + 5% MeOH, R_f = 0.30) to afford compound **S37** (11 mg, 38% over 4 steps). ^{1}H NMR (500 MHz, $CDCl_3$) δ 8.14 (d, J = 7.3 Hz, 2H), 7.82 (d, J = 7.5 Hz, 2H), 7.47 (t, J = 7.3 Hz, 1H), 7.35-7.31 (m, 3H), 7.27 (t, J = 7.5 Hz, 2H), 5.83 (d, J = 9.2 Hz, 1H, C-7), 5.58 (br, 1H, A-1), 5.42 (ddd, J = 8.5, 3.9, 2.9 Hz, 1H, B-8), 5.35 (d, J = 8.5 Hz, 1H, B-7), 5.27 (br, 1H, A-2), 5.24 (d, J = 10.3 Hz, 1H, B_5-NH), 5.17 (ddd, J = 9.2, 5.3, 2.1 Hz, 1H, C-8), 4.95 (ddd, J = 11.9, 10.3, 4.6 Hz, 1H, B-4), 4.59 (d, J = 4.3 Hz, 1H, A-3), 4.47 (dd, J = 4.3, 4.3 Hz, 1H, A-4), 4.38-4.32 (m, 3H, A-5, B-9, C-6), 4.26-4.22 (m, 2H, A-6, B-9), 4.06 (ddd, J = 10.3, 10.3, 10.3 Hz, 1H, B-5), 4.00 (dd, J = 12.5, 2.1 Hz, 1H, C-9), 3.90 (d, J = 10.3 Hz, 1H, B-6), 3.89-3.83 (m, 1H, C-4), 3.83 (s, 3H, OCH_3), 3.81 (s, 3H, OCH_3), 3.70-3.66 (m, 2H, A-6, C-5), 3.61 (dd, J = 12.5, 5.3 Hz, 1H, C-9), 3.14 (dd, J = 11.7, 3.1 Hz, 1H, C-3_{eq}), 2.58 (dd, J = 13.0, 4.6 Hz, 1H, B-3_{eq}), 2.46 (s, 3H, Ac), 2.25 (s, 3H, Ac), 2.15 (dd, J = 11.7, 11.7 Hz, 1H, C-3_{ax}), 2.13 (s, 3H, Ac), 2.10 (s, 3H, Ac), 2.03-1.97 (m, 1H, B-3_{ax}), 2.03 (s, 3H, Ac), 2.01 (s, 3H, Ac), 1.97 (s, 3H, Ac), 1.90 (s, 3H, Ac); ^{13}C NMR (125 MHz, $CDCl_3$) δ 171.46, 170.89, 170.64, 170.35, 170.19, 169.74, 169.63, 169.14, 167.86, 166.87, 165.84, 165.46, 153.60, 133.32, 132.73, 130.05, 129.84, 129.71, 128.87, 128.41, 128.17, 99.68, 99.16, 98.59, 75.50, 74.84, 74.50, 73.70, 72.70, 72.56, 70.68, 68.99, 68.58, 68.04, 67.03, 66.51, 64.36, 62.12, 61.84, 59.07, 53.23, 53.07, 49.47, 36.91,

36.18, 24.68, 23.22, 21.14, 21.07, 20.86, 20.76, 20.75, 20.69; HRMS (ESI) *m/z* for $C_{57}H_{66}N_2NaO_{30}$ [M+Na]$^+$: calcd 1281.3598, found 1281.3600.

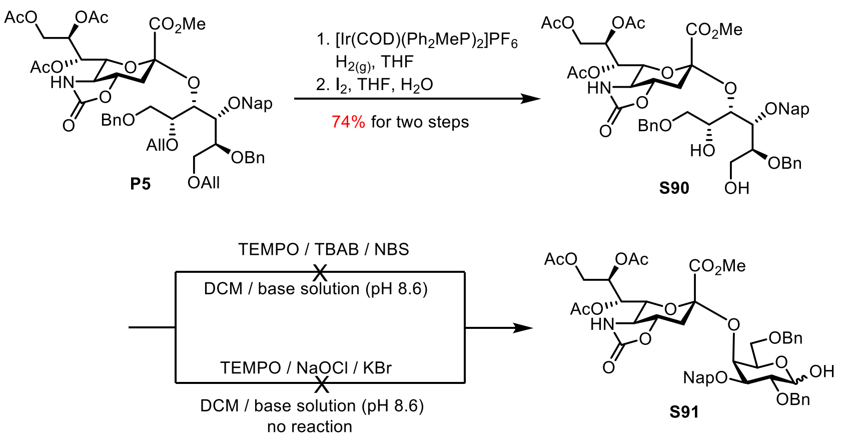

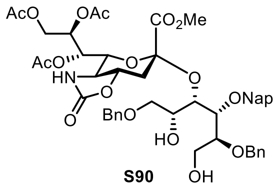

Compound S90. $[Ir(COD)(Ph_2MeP)_2]PF_6$ (2.4 mg, 3 μmol) was dissolved in tetrahydrofuran (THF, 0.9 mL) and activated by bubbling hydrogen gas for 15 minutes. This solution was then added to a solution of compound **P5** (93 mg, 0.09 mmol) in tetrahydrofuran (THF, 0.9 mL). The mixture was reacted under a nitrogen atmosphere for 18 hours. After removing the solvent, the residue was dissolved in a mixture of tetrahydrofuran (THF, 1.8 mL) and water (0.5 mL). Iodine (48 mg, 0.18 mmol) was then added, and the reaction was stirred at room temperature for 1 hour. Upon completion, the reaction mixture was diluted with water and extracted with dichloromethane and saturated aqueous sodium thiosulfate. The organic layer was dried over magnesium sulfate, filtered, and purified by column chromatography to afford compound **S90** (63 mg, 74% for two steps) (EtOAc /Hexanes = 1/1, R_f = 0.13). ^{1}H NMR (400 MHz, $CDCl_3$) δ 7.82-7.71 (m, 4H, ArH), 7.49-7.45 (m, 3H, ArH), 7.31-7.24 (m, 10H, ArH), 5.58 (d, *J* = 10.3 Hz, 1H, H-8'), 5.32 (br, 1H, NH), 5.08 (d, *J* = 10.3 Hz, 1H, H-7'), 5.02 (d, *J* = 11.1 Hz, 1H, $PhCH_2$), 4.91 (d, *J* = 11.1 Hz, 1H, $PhCH_2$), 4.69-4.63

(m, 2H, $PhCH_2$), 4.48-4.45 (m, 3H, H-6, $PhCH_2$), 4.34 (dd, J = 12.3, 1.6 Hz, 1H, H-9'), 4.24 (dd, J = 12.3, 3.6 Hz, 1H, H-9'), 4.16 (dd, J = 5.8, 5.4 Hz, 1H), 4.07-4.03 (m, 2H), 3.90-3.80 (m, 3H), 3.71-3.63 (m, 5H), 3.55 (dd, J = 9.8, 6.4 Hz, 1H), 3.44 (dd, J = 9.8, 5.8 Hz, 1H), 3.08 (dd, J = 12.0, 3.5 Hz, 1H, H-3'$_{eq}$), 2.99 (dd, J = 12.8, 10.8 Hz, 1H, H-5'), 2.72 (br, 1H, OH), 2.18 (s, 3H, (CO)CH_3), 2.09 (dd, J = 12.8, 12.0 Hz, 1H, H-3'$_{ax}$), 2.03 (s, 3H, (CO)CH_3), 1.91 (s, 3H, (CO)CH_3); ^{13}C NMR (100 MHz, $CDCl_3$) δ 171.17, 170.59, 170.51, 167.30, 159.16, 138.23 (2C), 135.67, 133.18, 133.04, 128.38 (2C), 128.35, 128.32 (2C), 127.79, 127.71, 127.66 (3C), 127.51, 127.38 (2C), 126.69, 126.23, 126.08, 126.00, 101.92, 85.09, 80.66, 76.42, 75.31, 73.63, 73.13, 72.95, 70.65, 70.45, 68.44, 67.38, 61.63, 60.88, 57.99, 53.12, 38.34, 29.66, 21.08, 20.61, 20.42; HRMS (ESI) *m/z* for $C_{48}H_{55}NO_{17}Na$ [M+Na]$^+$: calcd 940.3368, found 940.3371.

Reference

1. Noel, A.; Delpech, B.; Crich, D. Stereoselective, Electrophilic α-*C*-Sialidation. *Org. Lett*. **2012**, *14*, 1342-1345.
2. Crich, D.; Li, W. α-Selective Sialylations at -78 °C in Nitrile Solvents with a 1-Adamantanyl Thiosialoside. *J. Org. Chem*. **2007**, *72*, 7794-7797.
3. Tanaka, S.-I.; Goi, T.; Tanaka, K.; Fukase, K. Highly efficient α-sialylation by virtue of fixed dipole effects of *N*-phthalyl group: Application to continuous flow synthesis of α(2-3)- and α(2-6)-Neu5Ac-Gal motifs by microreactor. *J. Carbohydr. Chem*. **2007**, *26*, 369-394.
4. Dhakal, B.; Buda, S.; Crich, D. Stereoselective Synthesis of 5-epi-α-Sialosides Related to the Pseudaminic Acid Glycosides. Reassessment of the Stereoselectivity of the 5-Azido-5-deacetamidosialyl Thioglycosides and Use of Triflate as Nucleophile in the Zbiral Deamination of Sialic Acids. *J. Org. Chem.* **2016**, *81*, 10617-10630.
5. Lin, C.-C.; Huang, K.-T.; Lin, C.-C. *N*-Trifluoroacetyl Sialyl Phosphite Donors for the Synthesis of α(2→9) Oligosialic Acids. *Org. Lett*. **2005**, *7*, 4169-4172.
6. Wu, Y.-F.; Tsai, Y.-F.; Huang, Y.-S.; Shih, J.-F. Total Synthesis of the Echinodermatous Ganglioside LLG-3 Possessing the Biological Function of Promoting the Neurite Outgrowth. *Org. Lett*. **2020**, *22*, 7491-7495.
7. Sun, B.; Jiang, H. An efficient approach for total synthesis of aminopropyl functionalized ganglioside GM1b. *Tetrahedron Lett*. **2012**, *53*, 5711-5715.
8. Wang, Z.; Zhou, L.; El-Boubbou, K.; Ye, X.-s.; Huang, X. Multi-Component One-Pot Synthesis of the Tumor-Associated Carbohydrate Antigen Globo-H Based on Preactivation of Thioglycosyl Donors. *J. Org. Chem*. **2007**, *72*, 6409-6420.

9. Halder, T.; Yadav, S. Total synthesis of the *O*-antigen repeating unit of Providencia stuartii O49 serotype through linear and one-pot assemblies. *Beilstein J. Org. Chem.* **2021**, *17*, 2915-2921.

10. Halder, T.; Yadav, S.K.; Yadav, S. Chemical synthesis of the *O*-antigen repeating unit of Actinobacillus actinomycetemcomitans serotype f. *Carbohydr. Res.* **2023**, *534*, 108977.

11. Liu, G.-j.; Zhang, X.-t.; Xing, G.-w. A general method for *N*-glycosylation of nucleobases promoted by (p-Tol)$_2$SO/Tf$_2$O with thioglycoside as donor. *Chem. Commun*. **2015**, *51*, 12803-12806.

12. Michalik, D.; Vliegenthart J. F. G.; Kamerling, J. P. J. Chemoenzymic synthesis of oligosaccharide fragments of the capsular polysaccharide of Streptococcus pneumoniae type 14.*Chem. Soc., Perkin Trans*. **2002**, *1*, 1973-1981.

13. Asressu, K.H.; Chang, C.W.; Lam, S.; Wang, C.C. Donor-Reactivity-Controlled Sialylation Reactions. *Eur. J. Org. Chem*. **2021**, *2021*, 4525-4530.

14. Komura, N.; Kato, K.; Udagawa, T.; Asano, S.; Tanaka, H.-N.; Imamura, A.; Ishida, H.; Kiso, M.; Ando, H. Constrained sialic acid donors enable selective synthesis of α-glycosides. *Science* **2019**, *364*, 677-680.

15. Attouche, A.; Urban, D.; Beau, J.-M. A Tin-Free Regioselective Radical De-*O*-benzylation by an Intramolecular Hydrogen Atom Transfer on Carbohydrate Templates. *Angew. Chem., Int. Ed*. **2013**, *52*, 9572-9575.

16. Comfort, D. A.; Bobrov, K. S.; Ivanen, D. R.; Shabalin, K. A.; Harris, J. M.; Kulminskaya, A. A.; Brumer, H.; Kelly, R. M. Biochemical analysis of Thermotoga maritima GH36 α-galactosidase (TmGalA) confirms the mechanistic commonality of clan GH-D glycoside hydrolases. *Biochemistry* **2007**, *46*, 3319-3330.

17. Wang, C.-C.; Lee, J.-C.; Luo, S.-Y.; Fan, H.-F.; Pai, C.-L.; Yang, W.-C.; Lu, L.-D.; Hung, S.-C. Synthesis of Biologically Potent α1→2-Linked Disaccharide Derivatives via Regioselective One-Pot Protection-Glycosylation. *Angew. Chem. Int. Ed.* **2002**, *41*, 2360-2362.

18. Kim, T.; Bell, M. R.; Narasimharao Thota, V.; Lowary, T. L. One-Pot Regioselective Diacylation of Pyranoside 1,2-cis Diols. *J. Org. Chem*. **2022**, *87*, 4894-4907.

19. He, D. Y.; Li, Z. J.; Li, Z. J.; Liu, Y. Q.; Qiu, D. X.; Cai, M. S. Studies on carbohydrates X. A new method for the preparation of isopropylidene saccharides. *Synth. Commun*. **1992**, *22*, 2653-2658.

NMR Spectrum

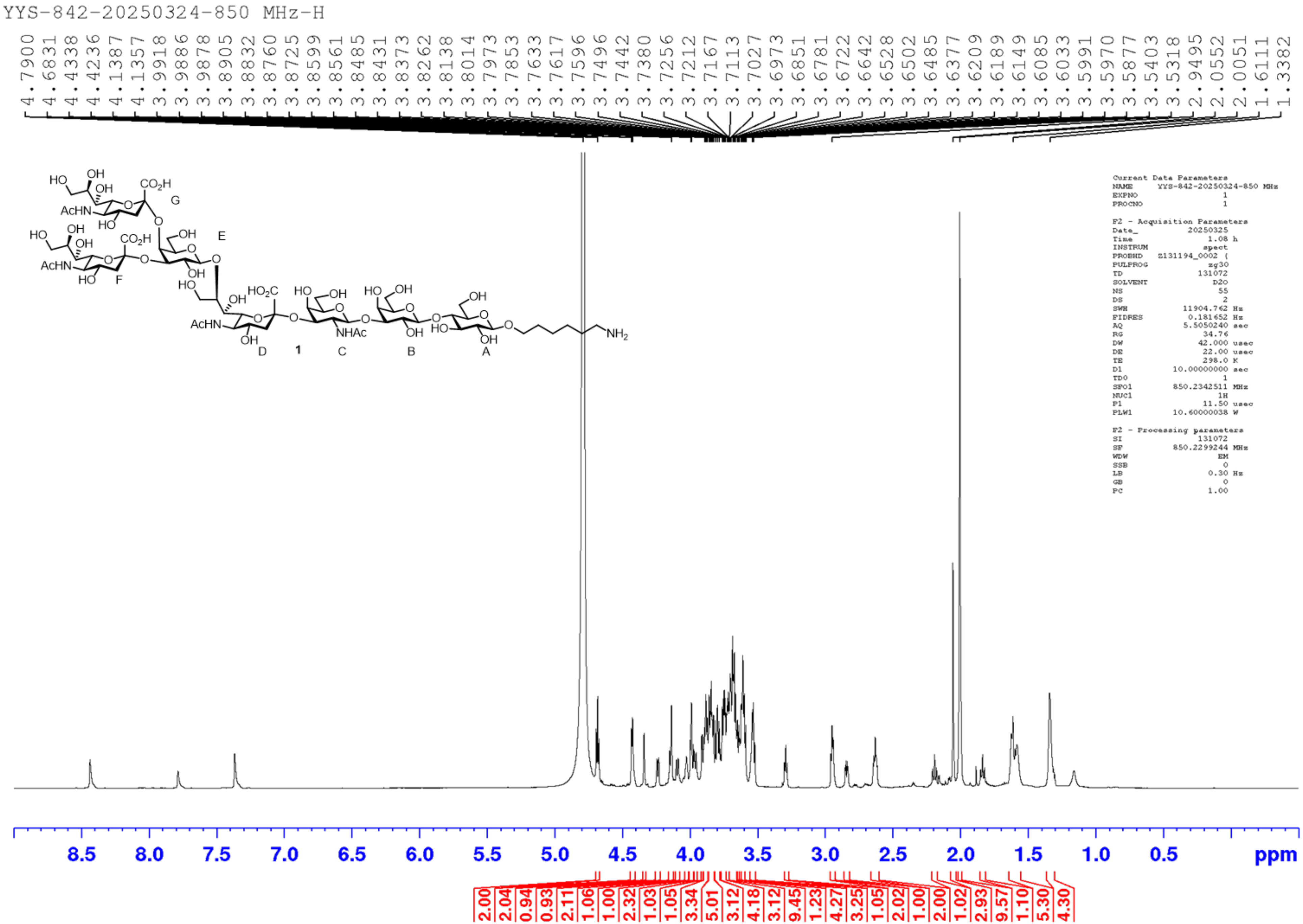
YYS-842-20250324-850 MHz-H
Current Data Parameters
NAME YYS-842-20250324-850 MHz
EXPNO 1
PROCNO 1
F2 - Acquisition Parameters
Date_ 20250325
Time 1.08 h
INSTRUM spect
PULPROG zg30
TD 131072
SOLVENT D2O
NS 55
DS 2
SWH 11904.762 Hz
FIDRES 0.181652 Hz
AQ 5.5050240 sec
RG 34.76
DW 42.000 usec
DE 22.00 usec
TE 298.0 K
D1 10.00000000 sec
TD0 1
SFO1 850.2342511 MHz
NUC1 1H
P1 11.50 usec
PLW1 10.60000038 W
F2 - Processing parameters
SI 131072
SF 850.2299244 MHz
WDW EM
SSB 0
LB 0.30 Hz
GB 0
PC 1.00
ppm

YYS-842-20250324-850 MHz-C

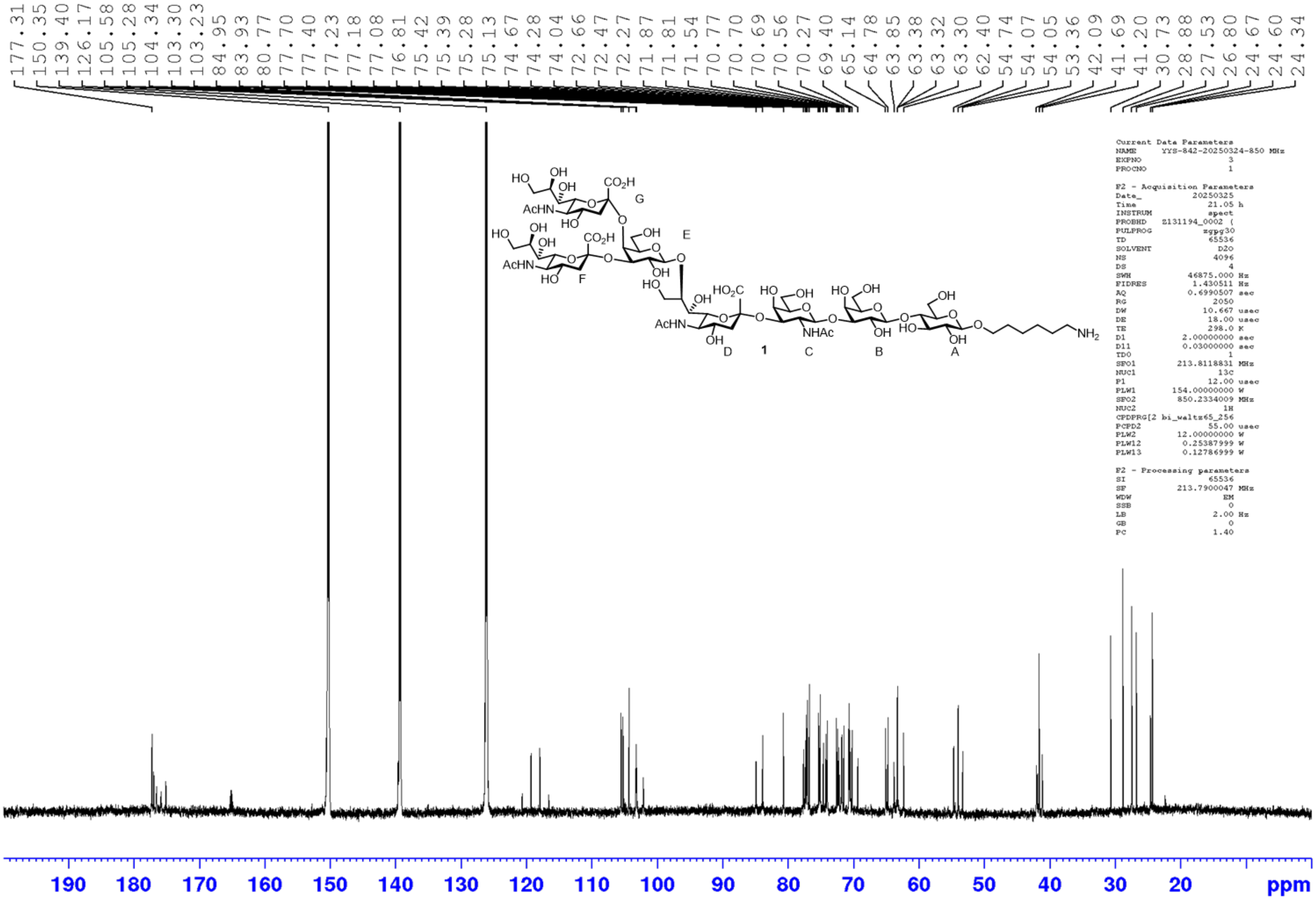

cosy

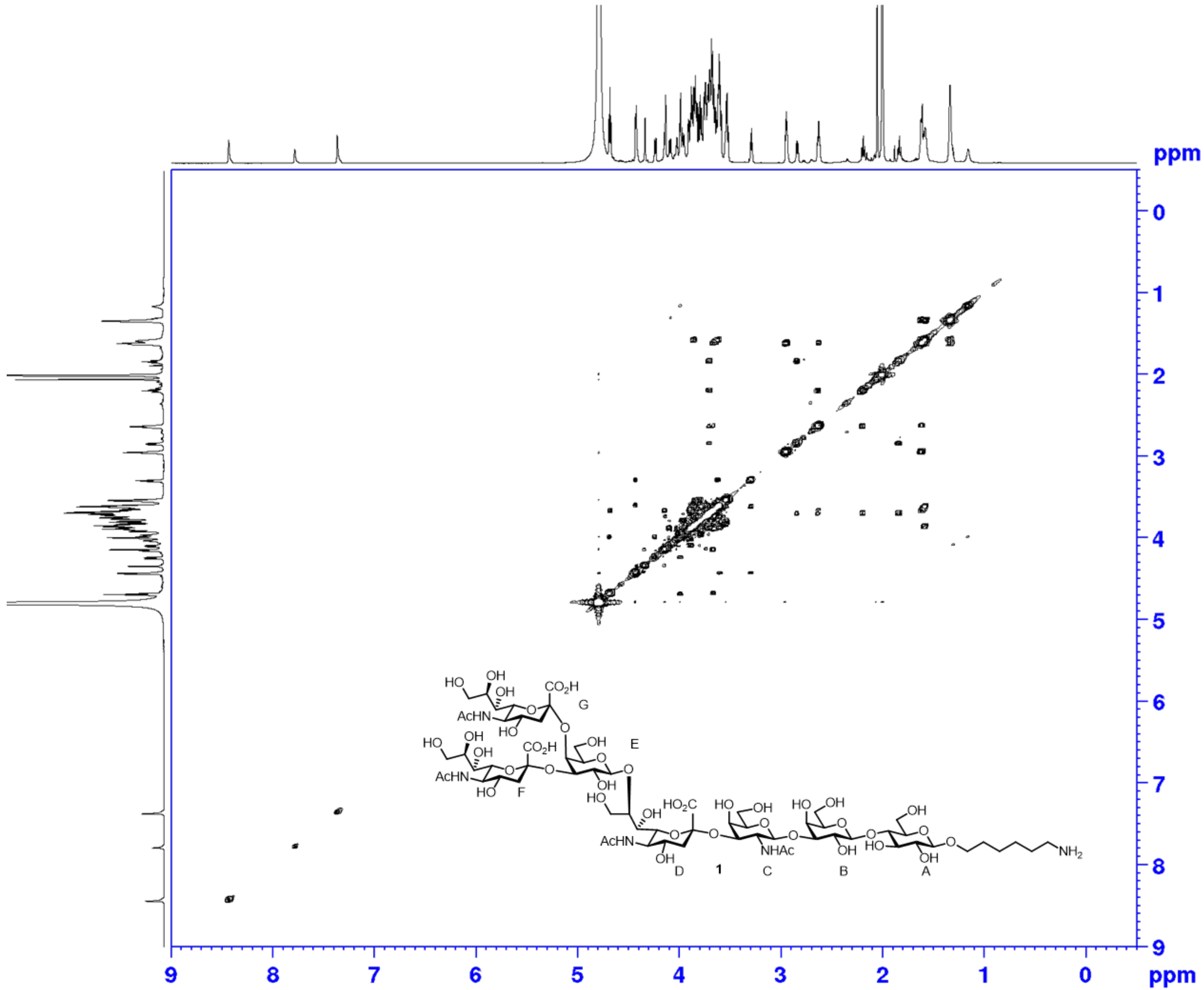

Current Data Parameters
NAME YYS-842-20250324-850 MHz
EXPNO 5
PROCNO 1

F2 - Acquisition Parameters
Date_ 20250325
Time 1.56 h
INSTRUM spect
PROBHD Z131194_0002 (
PULPROG cosygf90
TD 2048
SOLVENT D2O
NS 8
DS 0
SWH 8503.401 Hz
FIDRES 8.304103 Hz
AQ 0.1204224 sec
RG 34.76
DW 58.800 usec
DE 22.00 usec
TE 298.0 K
D0 0.00000300 sec
D1 2.00000000 sec
IN0 0.00011760 sec
TDav 1
SFO1 850.2339961 MHz
NUC1 1H
P1 11.50 usec
PLW1 10.59300041 W

F1 - Acquisition parameters
TD 256
SFO1 850.234 MHz
FIDRES 66.432823 Hz
SW 10.001 ppm
FnMODE QF

F2 - Processing parameters
SI 1024
SF 850.2299219 MHz
WDW SINE
SSB 0
LB 0 Hz
GB 0
PC 1.40

F1 - Processing parameters
SI 1024
MC2 QF
SF 850.2299195 MHz
WDW SINE
SSB 0
LB 0 Hz
GB 0

hsqc

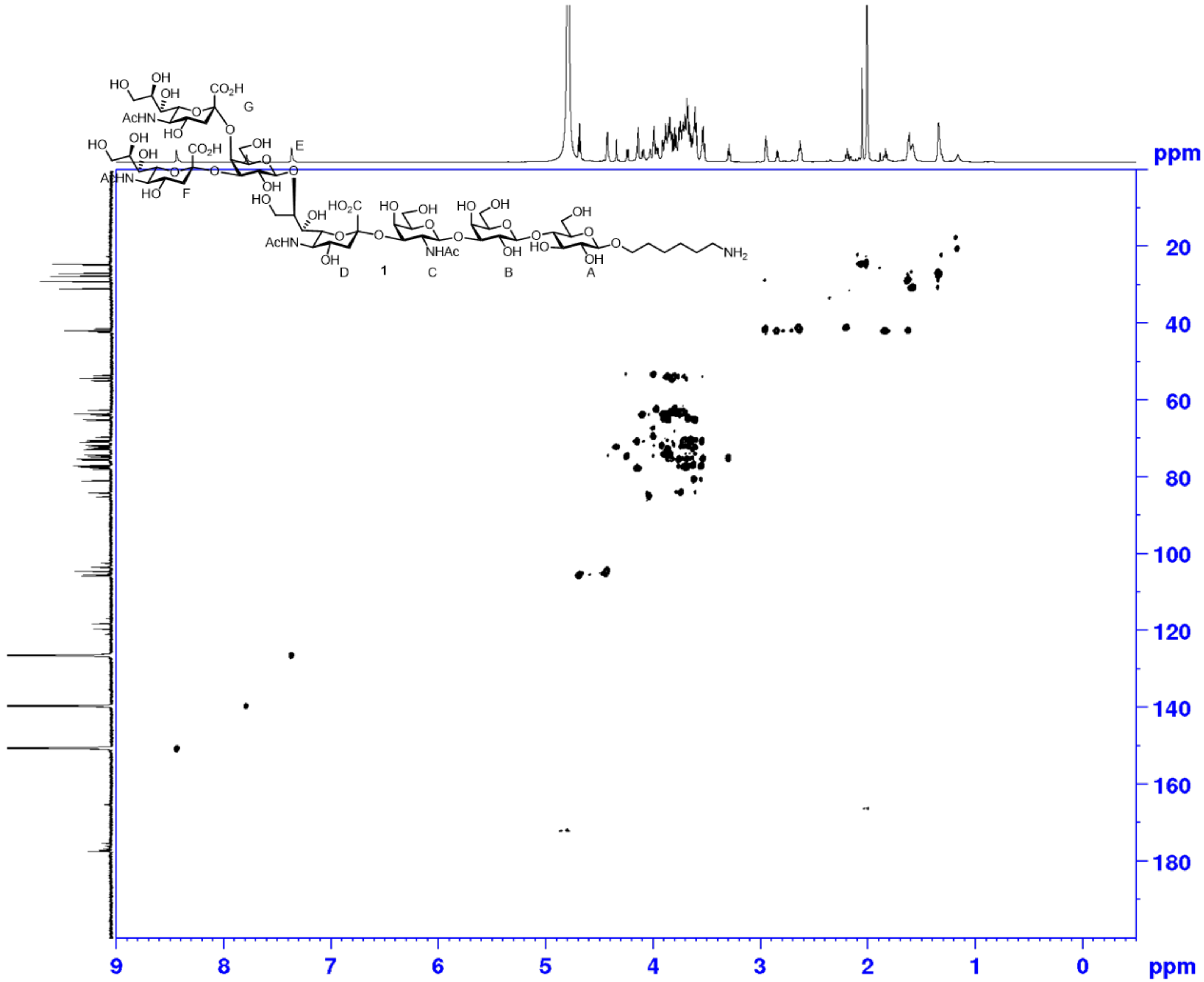


```
Current Data Parameters
NAME     YYS-842-20250324-850 MHz
EXPNO                      6
PROCNO                     1

F2 - Acquisition Parameters
Date_               20250325
Time                  3.10 h
INSTRUM                spect
PROBHD    Z131194_0002 (
PULPROG   hsqcedetgpsisp2.3
TD                      2048
SOLVENT                  D2O
NS                         8
DS                        16
SWH                 8503.401 Hz
FIDRES              8.304103 Hz
AQ                 0.1204224 sec
RG                    184.37
DW                    58.800 usec
DE                     22.00 usec
TE                     298.0 K
CNST2            145.0000000
CNST17            -0.5000000
D0                0.00000300 sec
D1                1.50000000 sec
D4                0.00172414 sec
D11               0.03000000 sec
D16               0.00020000 sec
D21               0.00344828 sec
D24               0.00086207 sec
IN0               0.00001300 sec
TDav                       1
SFO1             850.2339961 MHz
NUC1                      1H
P1                     11.50 usec
P2                     23.00 usec
P28                        0 usec
PLW1             10.59300041 W
SFO2             213.8076072 MHz
NUC2                     13C
CPDPRG[2  bi_p5m4sp_4sp.2
P3                     12.00 usec
P14                   500.00 usec
P24                  2000.00 usec
P31                  1453.00 usec
P63                  1500.00 usec
PLW0                       0 W
PLW2            168.00000000 W
PLW12             9.67679977 W
SPNAM[3]  Crp80,0.5,20.1
SPOAL3                 0.500
SPOFFS3                    0 Hz
SPW3             49.28400040 W
SPNAM[7]      Crp80comp.4
SPOAL7                 0.500
SPOFFS7                    0 Hz
SPW7             49.28400040 W
SPNAM[14  Crp48,1.5,20.2
SPOAL14                0.500
SPOFFS14                   0 Hz
SPW14            23.65600014 W
SPNAM[18   Crp60_xfilt.2
SPOAL18                0.500
SPOFFS18                   0 Hz
SPW18            15.14400005 W
SPNAM[31  Crp48,1.5,20.2
SPOAL31                0.500
SPOFFS31                   0 Hz
SPW31             5.91400003 W
GPNAM[1]         SMSQ10.100
GPZ1                   80.00 %
GPNAM[2]         SMSQ10.100
GPZ2                   20.10 %
GPNAM[3]         SMSQ10.100
GPZ3                   11.00 %
GPNAM[4]         SMSQ10.100
GPZ4                   -5.00 %
P16                  1000.00 usec
P19                   600.00 usec

F1 - Acquisition parameters
TD                       360
SFO1                213.8076 MHz
FIDRES            213.675217 Hz
SW                   179.889 ppm
FnMODE        Echo-Antiecho

F2 - Processing parameters
SI                      1024
SF               850.2299242 MHz
WDW                    QSINE
SSB                        2
LB                         0 Hz
GB                         0
PC                      1.40

F1 - Processing parameters
SI                      1024
MC2            echo-antiecho
```

hmbc

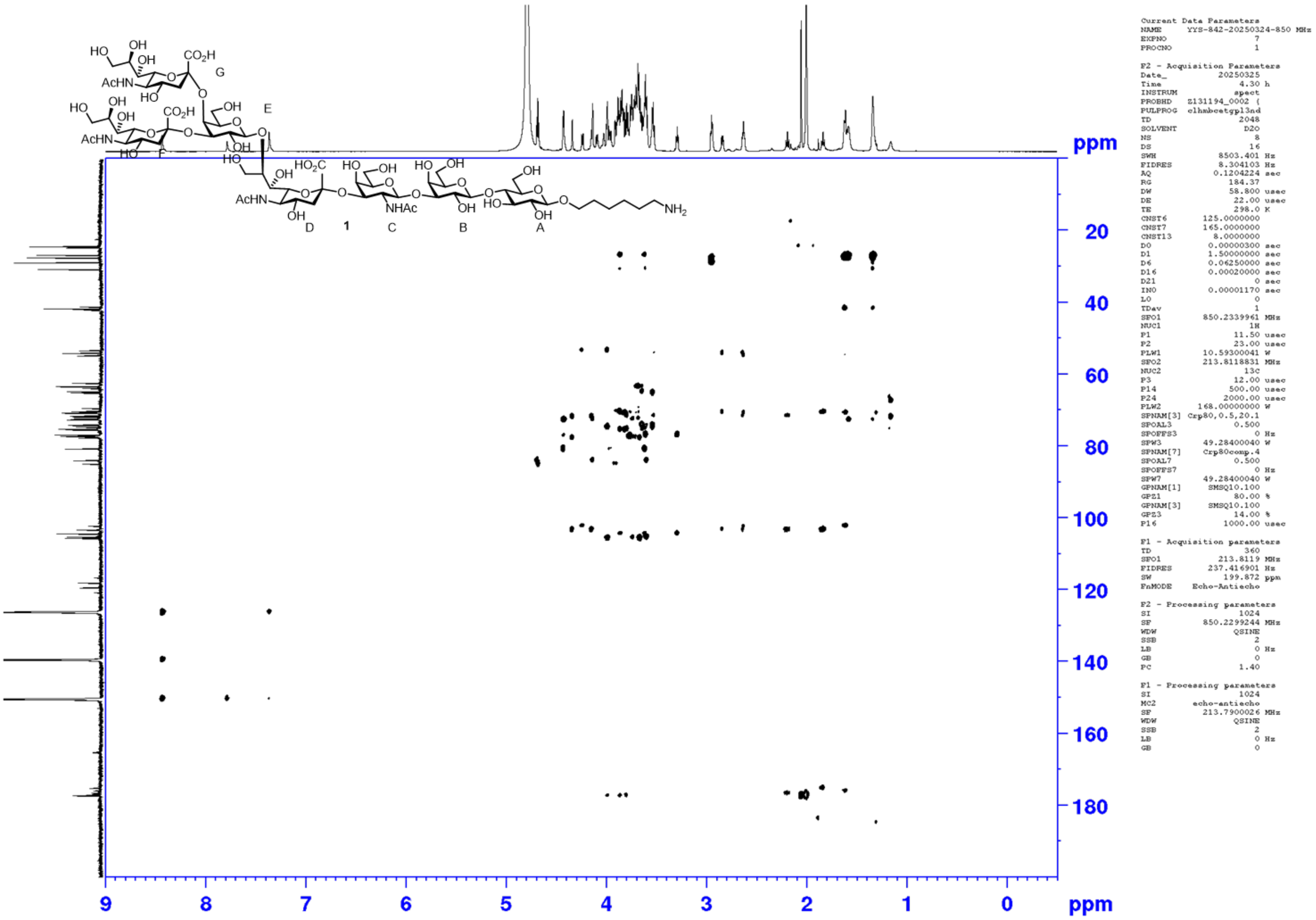
ppm
20
40
60
80
100
120
140
160
180
9
8
7
6
5
4
3
2
1
0
ppm
A
B
C
D
E
F
G
1
NHAc
NH2
Current Data Parameters
NAME YYS-842-20250324-850 MHz
EXPNO 7
PROCNO 1
F2 - Acquisition Parameters
Date_ 20250325
Time 4.30 h
INSTRUM spect
PROBHD Z131194_0002 (
PULPROG clhmbcetgpl3nd
TD 2048
SOLVENT D2O
NS 8
DS 16
SWH 8503.401 Hz
FIDRES 8.304103 Hz
AQ 0.1204224 sec
RG 184.37
DW 58.800 usec
DE 22.00 usec
TE 298.0 K
CNST6 125.0000000
CNST7 165.0000000
CNST13 8.0000000
D0 0.00000300 sec
D1 1.50000000 sec
D6 0.06250000 sec
D16 0.00020000 sec
D21 0 sec
IN0 0.00001170 sec
L0 0
TDav 1
SFO1 850.2339961 MHz
NUC1 1H
P1 11.50 usec
P2 23.00 usec
PLW1 10.59300041 W
SFO2 213.8118831 MHz
NUC2 13C
P3 12.00 usec
P14 500.00 usec
P24 2000.00 usec
PLW2 168.00000000 W
SPNAM[3] Crp80,0.5,20.1
SPOAL3 0.500
SPOFFS3 0 Hz
SPW3 49.28400040 W
SPNAM[7] Crp80comp.4
SPOAL7 0.500
SPOFFS7 0 Hz
SPW7 49.28400040 W
GPNAM[1] SMSQ10.100
GPZ1 80.00 %
GPNAM[3] SMSQ10.100
GPZ3 14.00 %
P16 1000.00 usec
F1 - Acquisition parameters
TD 360
SFO1 213.8119 MHz
FIDRES 237.416901 Hz
SW 199.872 ppm
FnMODE Echo-Antiecho
F2 - Processing parameters
SI 1024
SF 850.2299244 MHz
WDW QSINE
SSB 2
LB 0 Hz
GB 0
PC 1.40
F1 - Processing parameters
SI 1024
MC2 echo-antiecho
SF 213.7900026 MHz
WDW QSINE
SSB 2
LB 0 Hz
GB 0

hsqctocsy

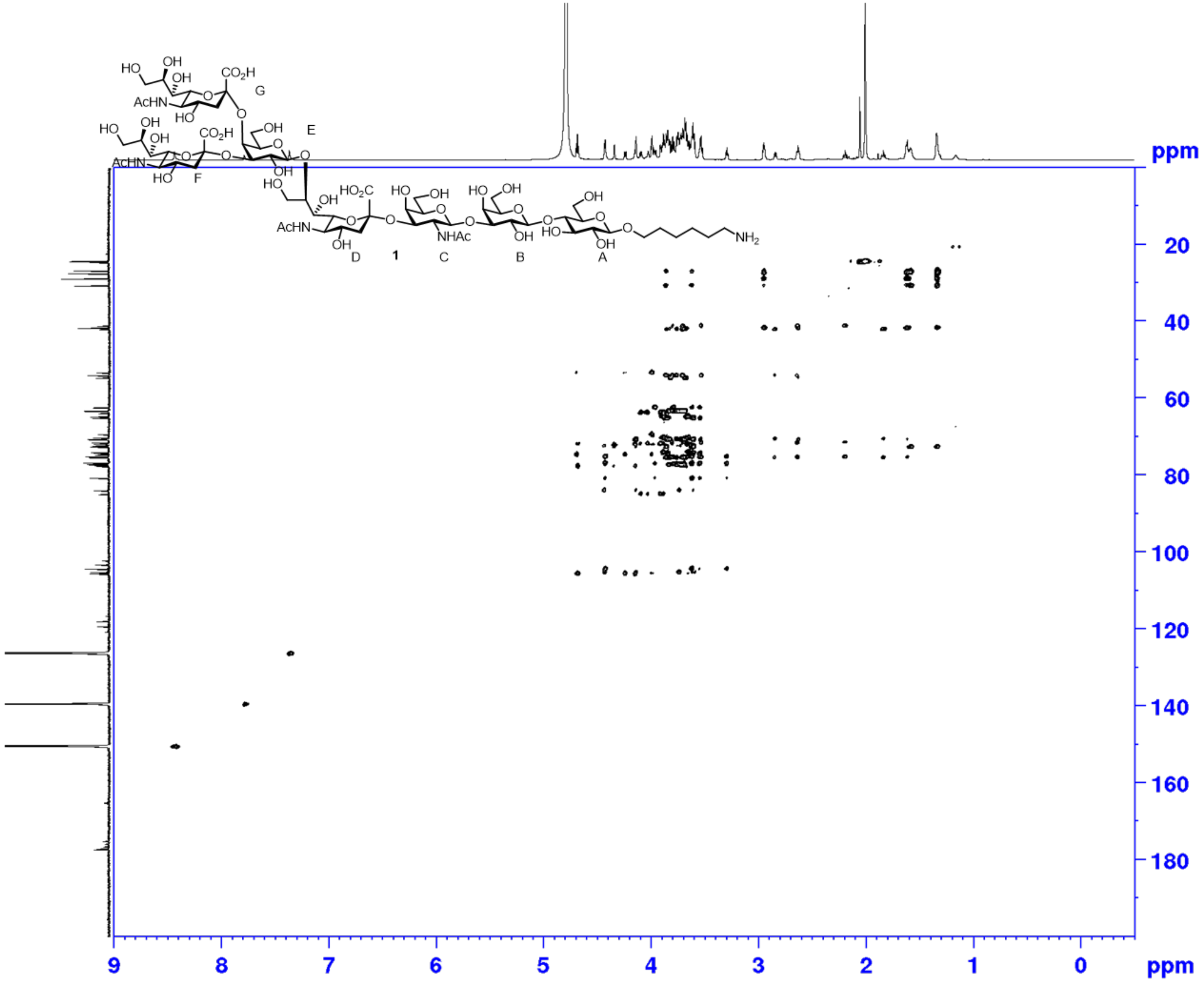


```
Current Data Parameters
NAME      YYS-842-20250324-850 MHz
EXPNO                   8
PROCNO                  1

F2 - Acquisition Parameters
Date_            20250325
Time                 5.53 h
INSTRUM             spect
PROBHD   Z131194_0002 (
PULPROG  hsqcdietgpsisp.2
TD                   2048
SOLVENT               D2O
NS                     16
DS                     32
SWH              8503.401 Hz
FIDRES           8.304103 Hz
AQ              0.1204224 sec
RG                 184.37
DW                 58.800 usec
DE                  22.00 usec
TE                  298.0 K
CNST2         145.0000000
CNST17         -0.5000000
D0             0.00000300 sec
D1             2.00000000 sec
D4             0.00172414 sec
D9             0.06000000 sec
D11            0.03000000 sec
D16            0.00020000 sec
D24            0.00089000 sec
IN0            0.00001020 sec
L1                     26
TDav                    1
SFO1          850.2339961 MHz
NUC1                   1H
P1                  11.50 usec
P2                  23.00 usec
P6                  20.00 usec
P28                     0 usec
PLW1          10.59300041 W
PLW10          3.50219989 W
SFO2          213.8086762 MHz
NUC2                  13C
CPDPRG[2             garp
P3                  12.00 usec
P14                500.00 usec
P24               2000.00 usec
PCPD2               50.00 usec
PLW0                    0 W
PLW2         168.00000000 W
PLW12          9.67679977 W
SPNAM[3] Crp80,0.5,20.1
SPOAL3              0.500
SPOFFS3                 0 Hz
SPW3          49.28400040 W
SPNAM[7]    Crp80comp.4
SPOAL7              0.500
SPOFFS7                 0 Hz
SPW7          49.28400040 W
GPNAM[1]      SMSQ10.100
GPZ1                80.00 %
GPNAM[2]      SMSQ10.100
GPZ2                20.10 %
P16               1000.00 usec

F1 - Acquisition parameters
TD                    360
SFO1             213.8087 MHz
FIDRES         272.331146 Hz
SW                229.269 ppm
FnMODE     Echo-Antiecho

F2 - Processing parameters
SI                   1024
SF            850.2299264 MHz
WDW                 QSINE
SSB                     2
LB                      0 Hz
GB                      0
PC                   1.40

F1 - Processing parameters
SI                   1024
MC2         echo-antiecho
SF            213.7900056 MHz
WDW                 QSINE
SSB                     2
LB                      0 Hz
GB                      0
```

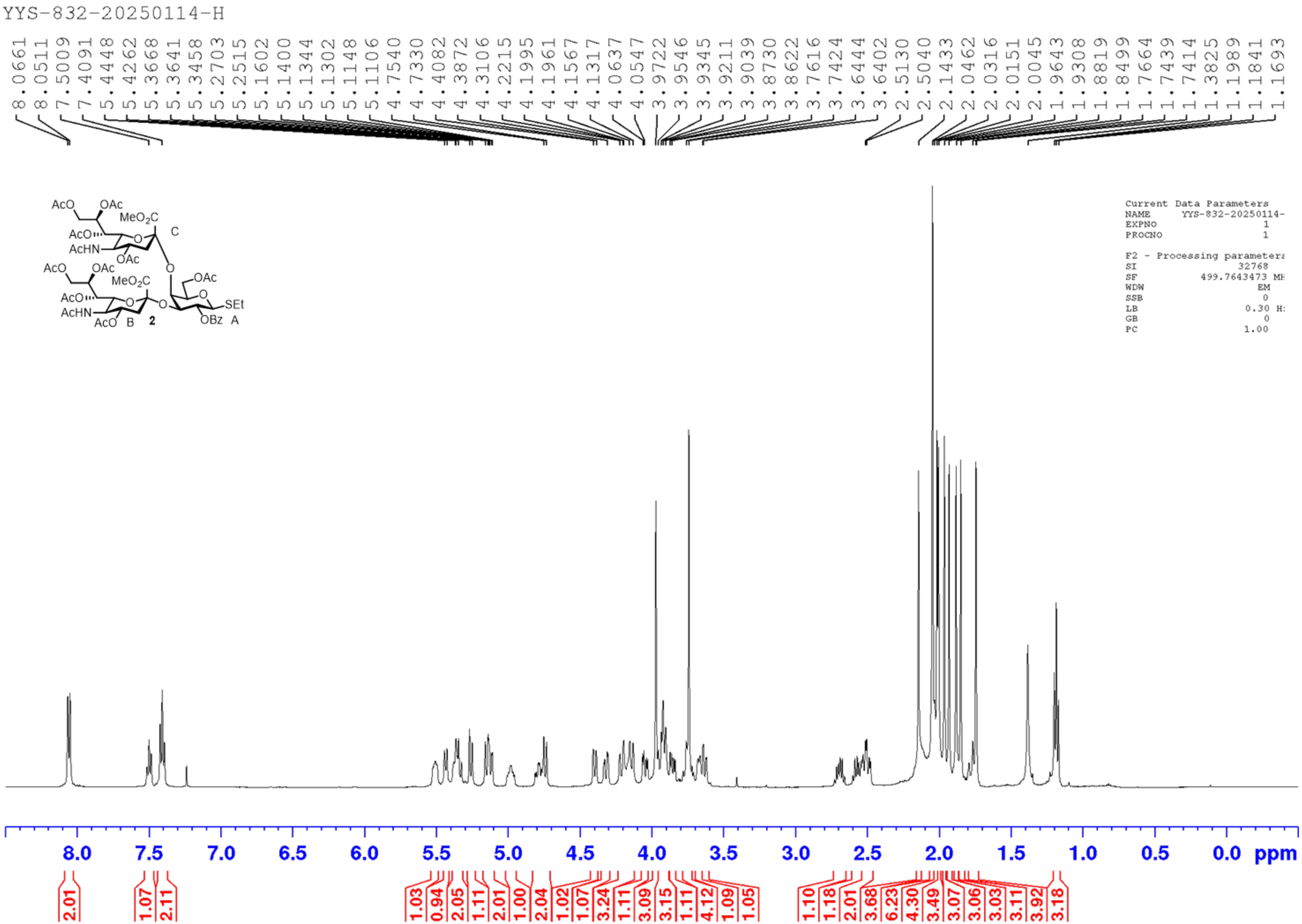
YYS-832-20250114-H
8.0661
8.0511
7.5009
7.4091
5.4448
5.4262
5.3668
5.3641
5.3458
5.2703
5.2515
5.1602
5.1400
5.1344
5.1302
5.1148
5.1106
4.7540
4.7330
4.4082
4.3872
4.3106
4.2215
4.1995
4.1961
4.1567
4.1317
4.0637
4.0547
3.9722
3.9546
3.9345
3.9211
3.9039
3.8730
3.8622
3.7616
3.7424
3.6444
3.6402
2.5130
2.5040
2.1433
2.0462
2.0316
2.0151
2.0045
1.9643
1.9308
1.8819
1.8499
1.7664
1.7439
1.7414
1.3825
1.1989
1.1841
1.1693
Current Data Parameters
NAME YYS-832-20250114-
EXPNO 1
PROCNO 1
F2 - Processing parameters
SI 32768
SF 499.7643473 MH
WDW EM
SSB 0
LB 0.30 H:
GB 0
PC 1.00
AcO
OAc
MeO2C
C
AcO
AcHN
O
OAc
O
AcO
OAc
MeO2C
OAc
AcO
AcHN
AcO
B
2
O
O
SEt
OBz
A
8.0
7.5
7.0
6.5
6.0
5.5
5.0
4.5
4.0
3.5
3.0
2.5
2.0
1.5
1.0
0.5
0.0
ppm
2.01
1.07
2.11
1.03
0.94
2.05
1.11
2.01
1.00
2.04
1.02
1.07
3.24
1.11
3.09
3.15
1.11
4.12
1.09
1.05
1.10
1.18
2.01
3.68
6.23
4.30
3.49
3.07
3.06
3.03
3.11
3.92
3.18

YYS-832-20250114-C

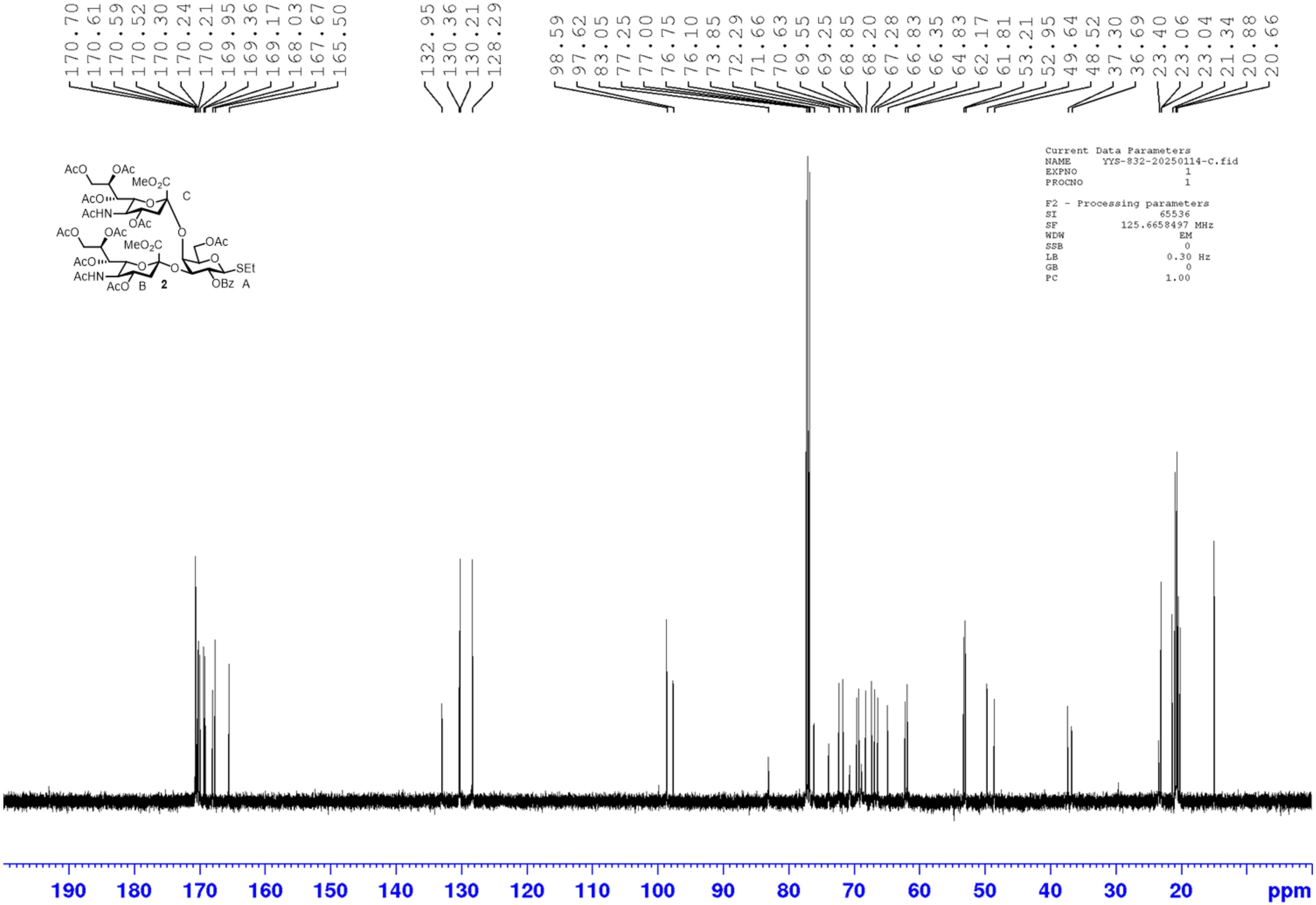

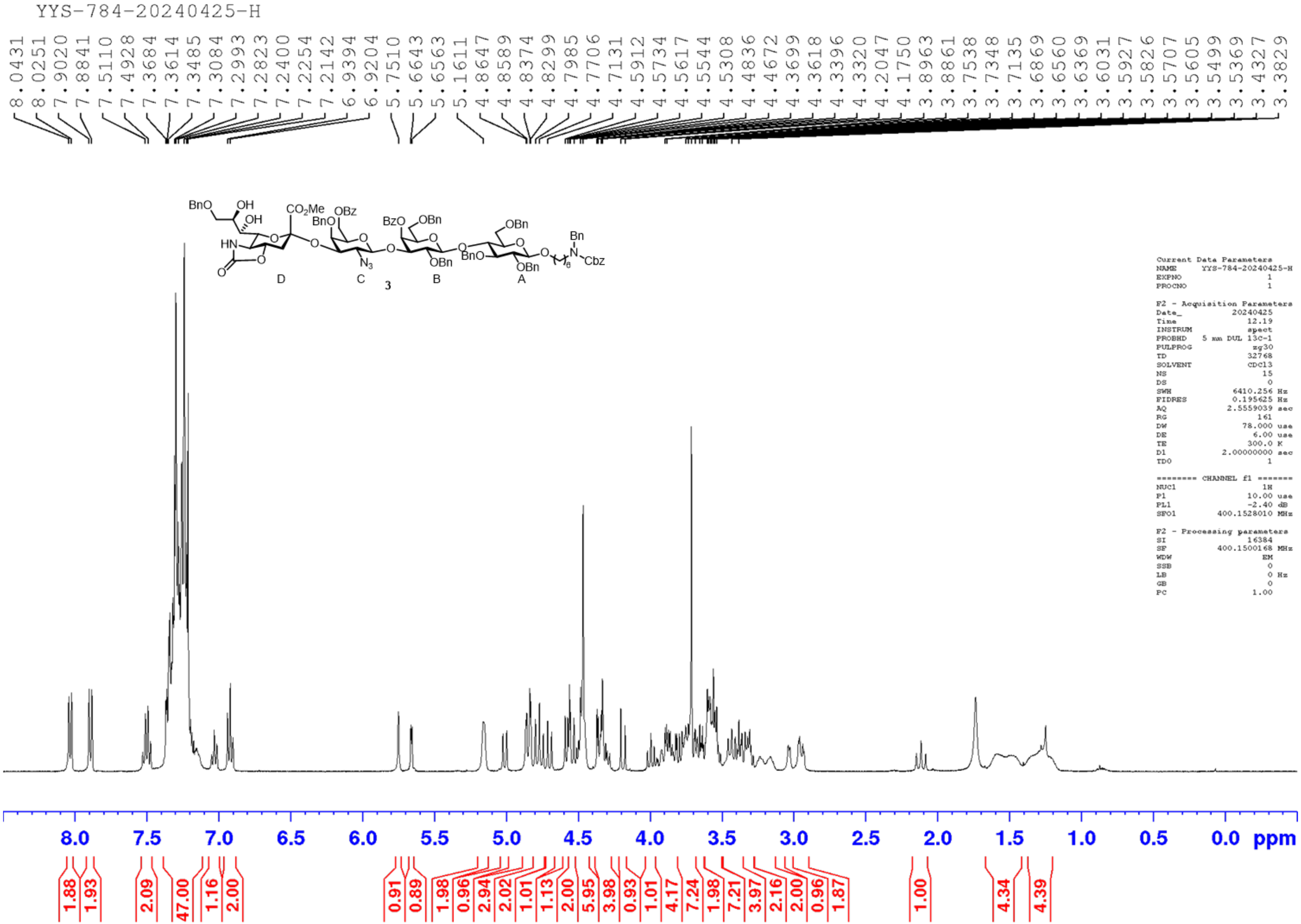

YYS-784-20240425-H
8.0431 8.0251 7.9020 7.8841 7.5110 7.4928 7.3684 7.3614 7.3485 7.3084 7.2993 7.2823 7.2400 7.2254 7.2142 6.9394 6.9204 5.7510 5.6643 5.6563 5.1611 4.8647 4.8589 4.8374 4.8299 4.7985 4.7706 4.7131 4.5912 4.5734 4.5617 4.5544 4.5308 4.4836 4.4672 4.3699 4.3618 4.3396 4.3320 4.2047 4.1750 3.8963 3.8861 3.7538 3.7348 3.7135 3.6869 3.6560 3.6369 3.6031 3.5927 3.5826 3.5707 3.5605 3.5499 3.5369 3.4327 3.3829
BnO
OH
OH
CO2Me
OBz
BnO
BzO
OBn
OBn
Bn
HN
O
N3
OBn
BnO
OBn
N
Cbz
8
D
C
3
B
A
Current Data Parameters
NAME YYS-784-20240425-H
EXPNO 1
PROCNO 1
F2 - Acquisition Parameters
Date_ 20240425
Time 12.19
INSTRUM spect
PROBHD 5 mm DUL 13C-1
PULPROG zg30
TD 32768
SOLVENT CDCl3
NS 15
DS 0
SWH 6410.256 Hz
FIDRES 0.195625 Hz
AQ 2.5559039 sec
RG 161
DW 78.000 use
DE 6.00 use
TE 300.0 K
D1 2.00000000 sec
TD0 1
======== CHANNEL f1 =======
NUC1 1H
P1 10.00 use
PL1 -2.40 dB
SFO1 400.1528010 MHz
F2 - Processing parameters
SI 16384
SF 400.1500168 MHz
WDW EM
SSB 0
LB 0 Hz
GB 0
PC 1.00
8.0 7.5 7.0 6.5 6.0 5.5 5.0 4.5 4.0 3.5 3.0 2.5 2.0 1.5 1.0 0.5 0.0 ppm
1.88 1.93 2.09 47.00 1.16 2.00 0.91 0.89 1.98 0.96 2.94 2.02 1.01 1.13 2.00 5.95 3.98 0.93 1.01 4.17 7.24 1.98 7.21 3.97 2.16 2.00 0.96 1.87 1.00 4.34 4.39

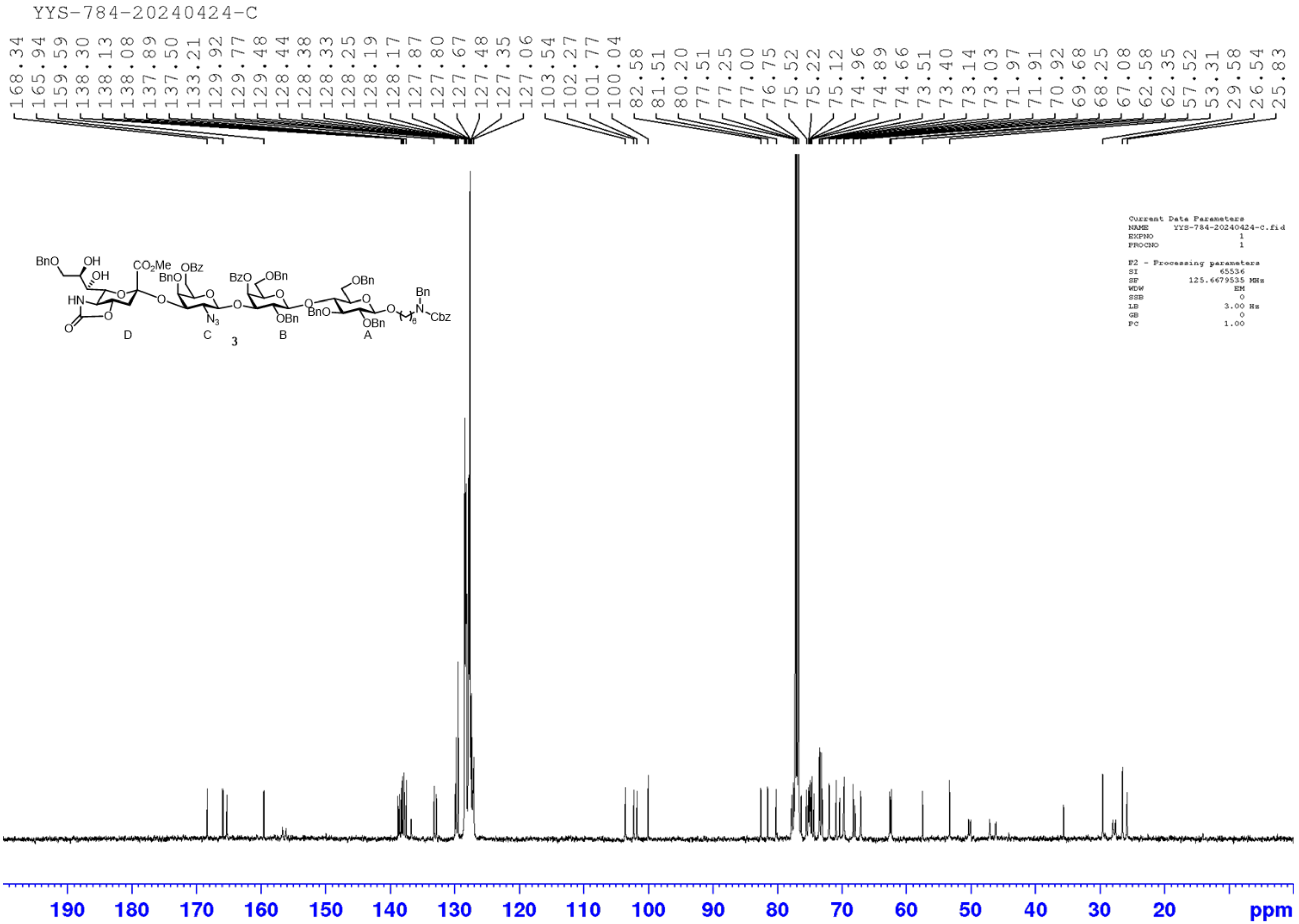
YYS-784-20240424-C
168.34
165.94
159.59
138.30
138.13
138.08
137.89
137.50
133.21
129.92
129.77
129.48
128.44
128.38
128.33
128.25
128.19
128.17
127.87
127.80
127.67
127.48
127.35
127.06
103.54
102.27
101.77
100.04
82.58
81.51
80.20
77.51
77.25
77.00
76.75
75.52
75.22
75.12
74.96
74.89
74.66
73.51
73.40
73.14
73.03
71.97
71.91
70.92
69.68
68.25
67.08
62.58
62.35
57.52
53.31
29.58
26.54
25.83
Current Data Parameters
NAME YYS-784-20240424-C.fid
EXPNO 1
PROCNO 1
F2 - Processing parameters
SI 65536
SF 125.6679535 MHz
WDW EM
SSB 0
LB 3.00 Hz
GB 0
PC 1.00
D
C
3
B
A
190 180 170 160 150 140 130 120 110 100 90 80 70 60 50 40 30 20 ppm

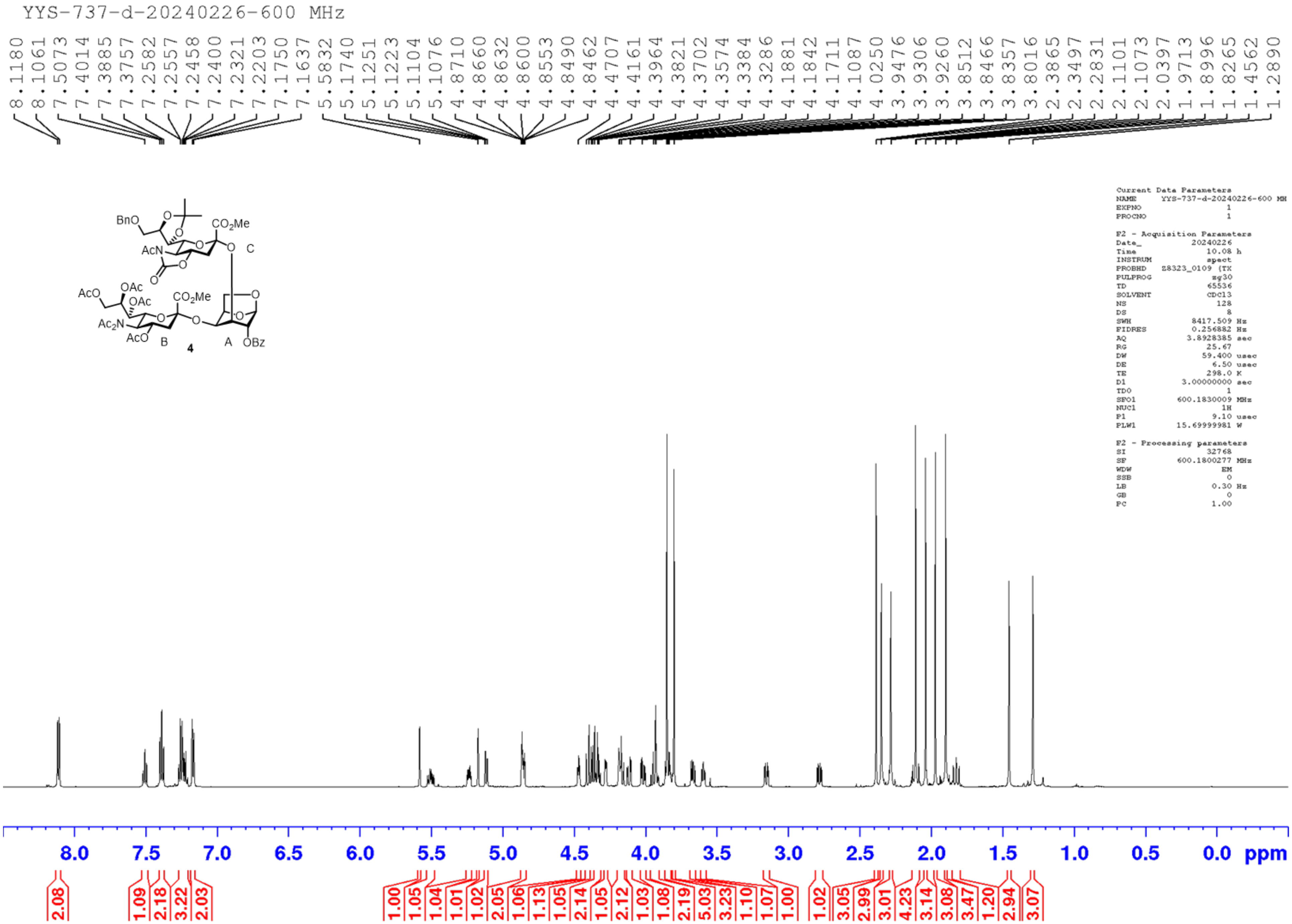
YYS-737-d-20240226-600 MHz
8.1180
8.1061
7.5073
7.4014
7.3885
7.3757
7.2582
7.2557
7.2458
7.2400
7.2321
7.2203
7.1750
7.1637
5.5832
5.1740
5.1251
5.1223
5.1104
5.1076
4.8710
4.8660
4.8632
4.8600
4.8553
4.8490
4.8462
4.4707
4.4161
4.3964
4.3821
4.3702
4.3574
4.3384
4.3286
4.1881
4.1842
4.1711
4.1087
4.0250
3.9476
3.9306
3.9260
3.8512
3.8466
3.8357
3.8016
2.3865
2.3497
2.2831
2.1101
2.1073
2.0397
1.9713
1.8996
1.8265
1.4562
1.2890
BnO
CO2Me
AcN
O
C
AcO
OAc
OAc
CO2Me
Ac2N
AcO
B
4
A
OBz
Current Data Parameters
NAME YYS-737-d-20240226-600 MH
EXPNO 1
PROCNO 1
F2 - Acquisition Parameters
Date_ 20240226
Time 10.08 h
INSTRUM spect
PROBHD Z8323_0109 (TX
PULPROG zg30
TD 65536
SOLVENT CDCl3
NS 128
DS 8
SWH 8417.509 Hz
FIDRES 0.256882 Hz
AQ 3.8928385 sec
RG 25.67
DW 59.400 usec
DE 6.50 usec
TE 298.0 K
D1 3.00000000 sec
TD0 1
SFO1 600.1830009 MHz
NUC1 1H
P1 9.10 usec
PLW1 15.69999981 W
F2 - Processing parameters
SI 32768
SF 600.1800277 MHz
WDW EM
SSB 0
LB 0.30 Hz
GB 0
PC 1.00
8.0
7.5
7.0
6.5
6.0
5.5
5.0
4.5
4.0
3.5
3.0
2.5
2.0
1.5
1.0
0.5
0.0
ppm
2.08
1.09
2.18
3.22
2.03
1.00
1.05
1.04
1.01
1.02
2.05
1.06
1.13
1.05
2.14
1.05
2.12
1.03
1.08
2.19
5.03
3.23
1.10
1.07
1.00
1.02
3.05
2.99
3.01
4.23
3.14
3.08
3.47
1.20
2.94
3.07

YYS-737-d-20240226-600 MHz-4

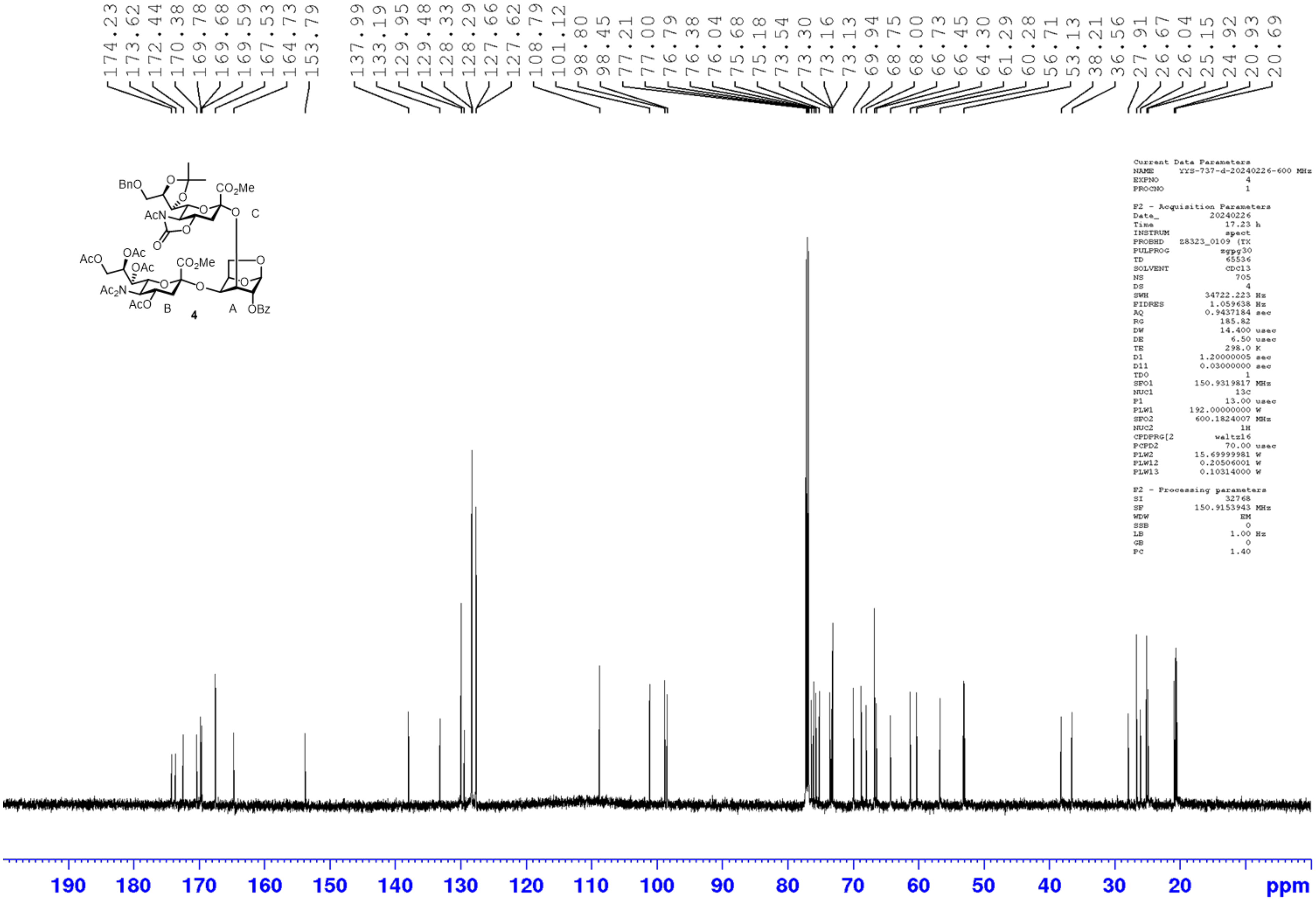

EXSIDE 2.144ppm

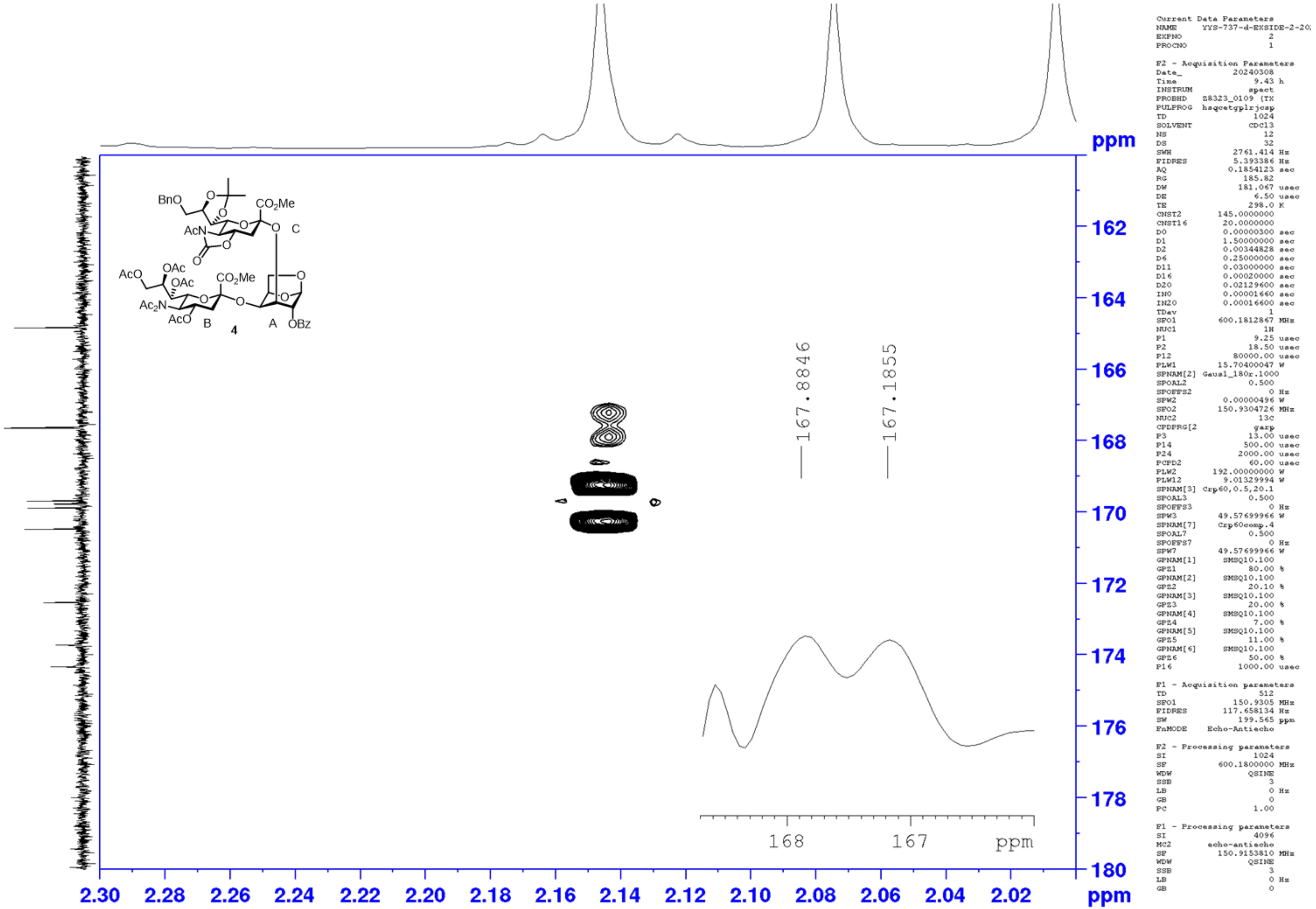

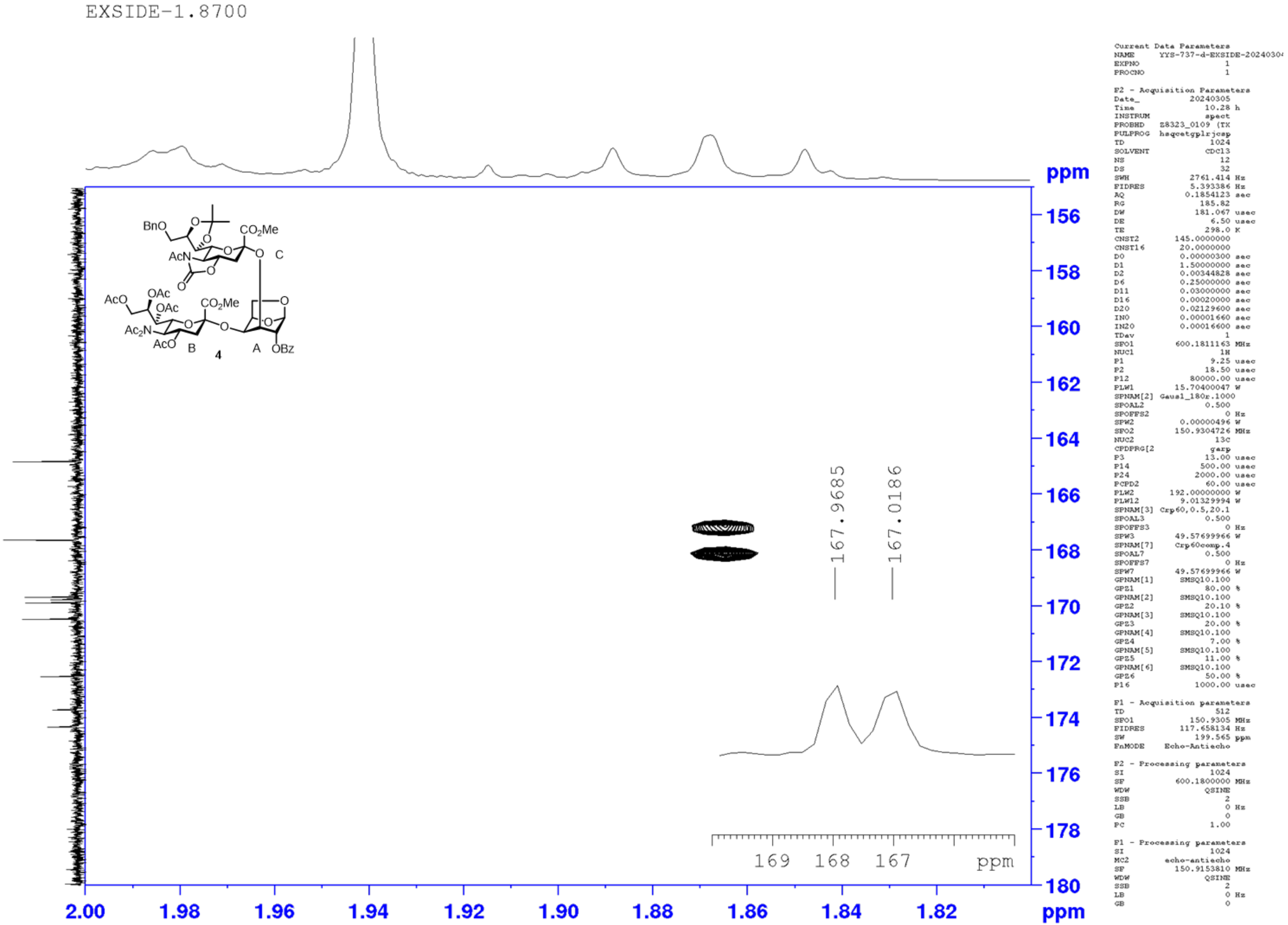
EXSIDE-1.8700
BnO
CO2Me
AcN
O
C
AcO
OAc
OAc
CO2Me
Ac2N
AcO
B
4
A
OBz
167.9685
167.0186
169
168
167
ppm
156
158
160
162
164
166
168
170
172
174
176
178
180
ppm
2.00
1.98
1.96
1.94
1.92
1.90
1.88
1.86
1.84
1.82
ppm
Current Data Parameters
NAME YYS-737-d-EXSIDE-2024030
EXPNO 1
PROCNO 1
F2 - Acquisition Parameters
Date_ 20240305
Time 10.28 h
INSTRUM spect
PULPROG hsqcetgplrjcsp
TD 1024
SOLVENT CDCl3
NS 12
DS 32
SWH 2761.414 Hz
FIDRES 5.393386 Hz
AQ 0.1854123 sec
RG 185.82
DW 181.067 usec
DE 6.50 usec
TE 298.0 K
CNST2 145.0000000
CNST16 20.0000000
SFO1 600.1811163 MHz
NUC1 1H
NUC2 13C
F1 - Acquisition parameters
TD 512
SFO1 150.9305 MHz
FIDRES 117.658134 Hz
SW 199.565 ppm
FnMODE Echo-Antiecho
F2 - Processing parameters
SI 1024
SF 600.1800000 MHz
WDW QSINE
SSB 2
LB 0 Hz
GB 0
PC 1.00
F1 - Processing parameters
SI 1024
MC2 echo-antiecho
SF 150.9153810 MHz
WDW QSINE
SSB 2
LB 0 Hz
GB 0

YYS-737-d-20240226-cosy

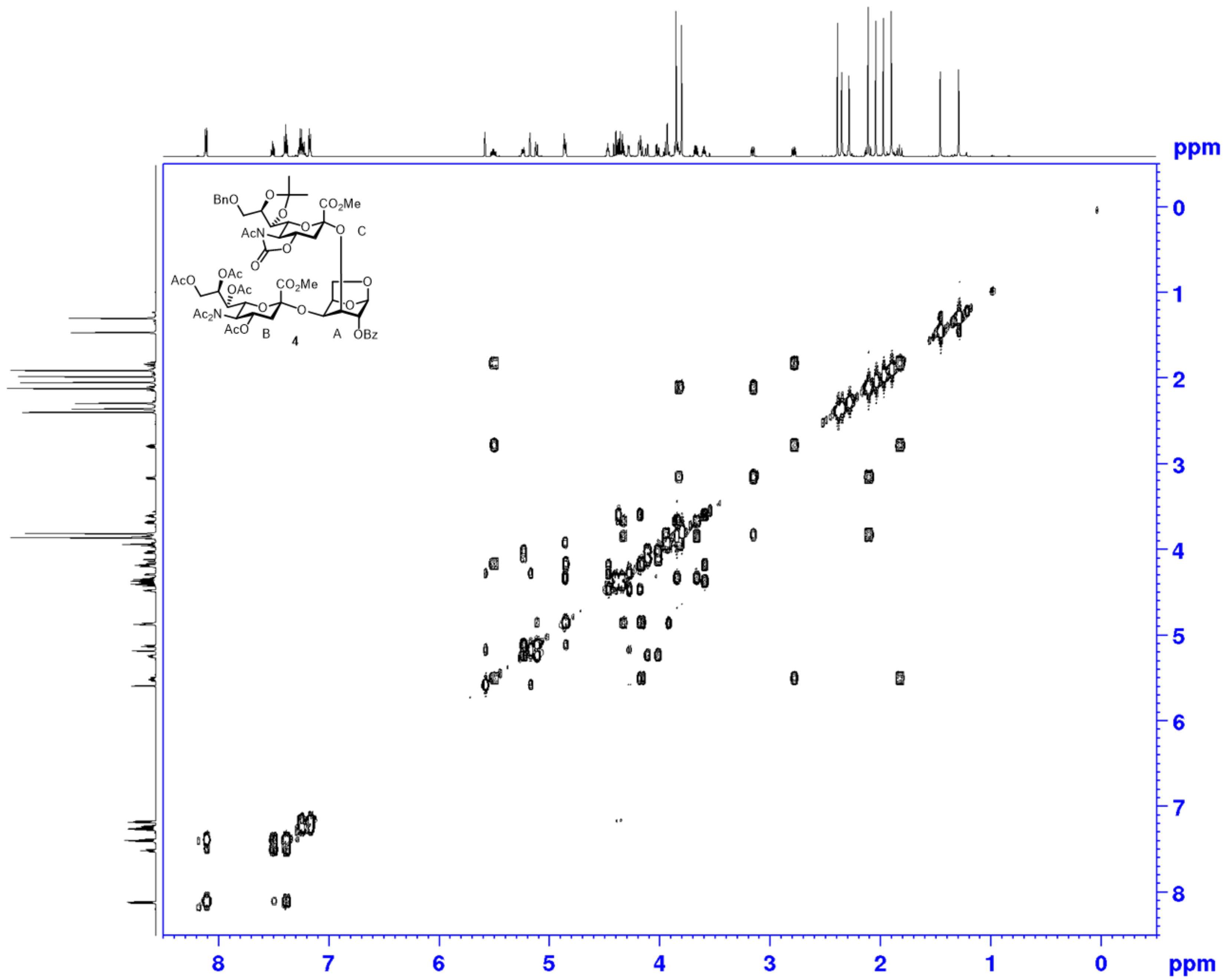


```
Current Data Parameters
NAME       YYS-737-d-20240226-600 MH
EXPNO                     2
PROCNO                    1

F2 - Acquisition Parameters
Date_              20240226
Time                  10.09 h
INSTRUM               spect
PROBHD     Z8323_0109 (TX
PULPROG          cosygpppqf
TD                     2048
SOLVENT               CDCl3
NS                        8
DS                       16
SWH                6009.615 Hz
FIDRES             5.868765 Hz
AQ                0.1703936 sec
RG                    31.56
DW                   83.200 usec
DE                     6.50 usec
TE                    298.0 K
D0               0.00000300 sec
D1               2.00000000 sec
D11              0.03000000 sec
D12              0.00002000 sec
D13              0.00000400 sec
D16              0.00020000 sec
IN0              0.00016660 sec
TDav                      1
SFO1            600.1828208 MHz
NUC1                     1H
P0                     9.10 usec
P1                     9.10 usec
P17                 2500.00 usec
PLW1            15.70400047 W
PLW10            2.08069992 W
GPNAM[1]         SMSQ10.100
GPZ1                  10.00 %
P16                 1000.00 usec

F1 - Acquisition parameters
TD                      256
SFO1               600.1828 MHz
FIDRES            46.893757 Hz
SW                   10.001 ppm
FnMODE                   QF

F2 - Processing parameters
SI                     1024
SF              600.1800294 MHz
WDW                   QSINE
SSB                       0
LB                        0 Hz
GB                        0
PC                     1.40

F1 - Processing parameters
SI                     1024
MC2                      QF
SF              600.1800289 MHz
WDW                   QSINE
SSB                       0
LB                        0 Hz
GB                        0
```

YYS-737-d-20240226-600 MHz-HMBC

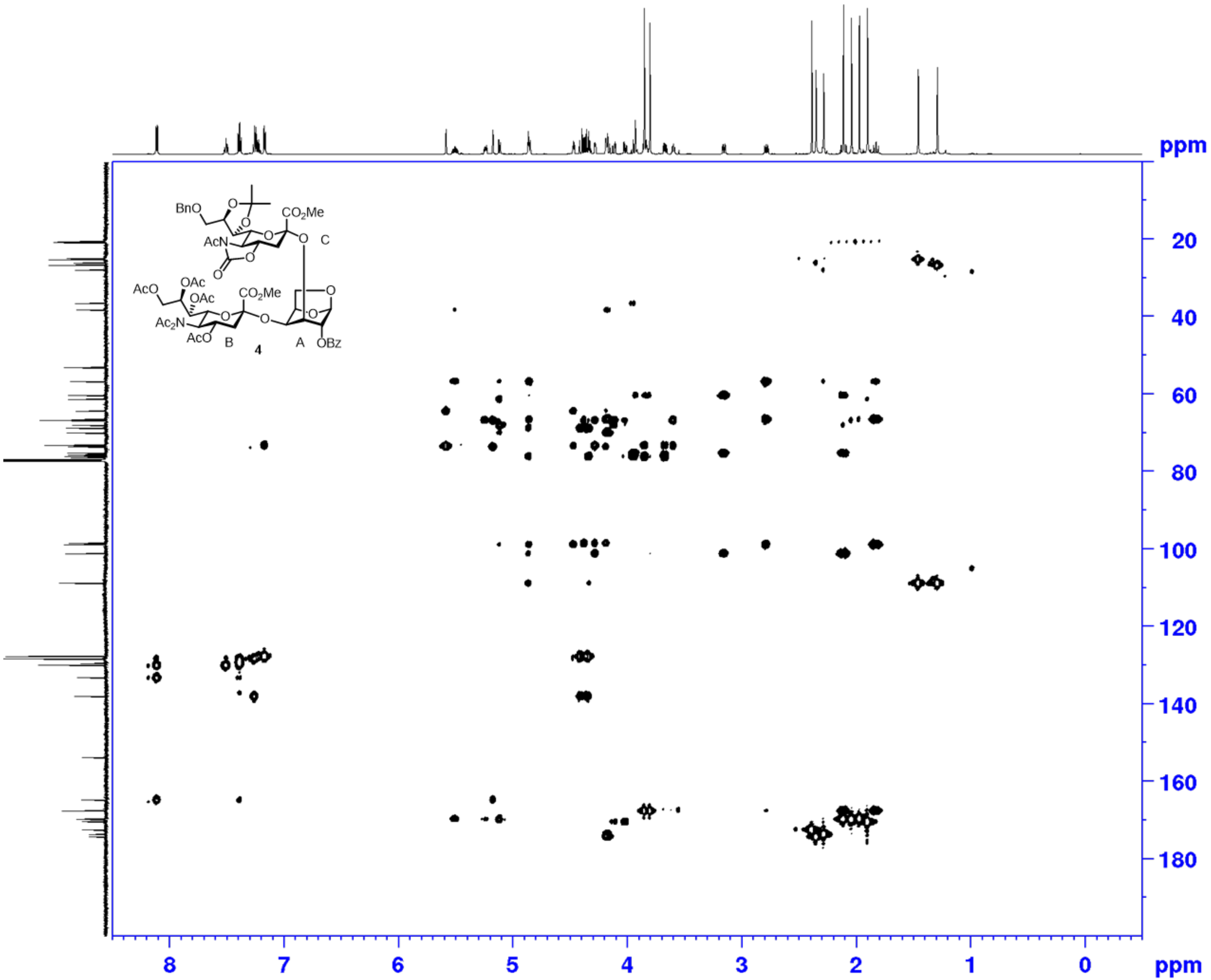

Current Data Parameters
NAME YYS-737-d-20240226-600 MHz
EXPNO 3
PROCNO 1

F2 - Acquisition Parameters
Date_ 20240226
Time 11.26 h
INSTRUM spect
PROBHD Z8323_0109 (TX
PULPROG clhmbcetgpl3nd
TD 2048
SOLVENT CDCl3
NS 16
DS 16
SWH 6009.615 Hz
FIDRES 5.868765 Hz
AQ 0.1703936 sec
RG 185.82
DW 83.200 usec
DE 6.50 usec
TE 298.0 K
CNST6 125.0000000
CNST7 165.0000000
CNST13 8.0000000
D0 0.00000300 sec
D1 1.50000000 sec
D6 0.06250000 sec
D16 0.00020000 sec
D21 0 sec
IN0 0.00001660 sec
L0 0
TDav 1
SFO1 600.1828304 MHz
NUC1 1H
P1 9.10 usec
P2 18.20 usec
PLW1 15.70400047 W
SFO2 150.9304726 MHz
NUC2 13C
P3 13.00 usec
P14 500.00 usec
P24 2000.00 usec
PLW2 192.00000000 W
SPNAM[3] Crp60,0.5,20.1
SPOAL3 0.500
SPOFFS3 0 Hz
SPW3 49.57699966 W
SPNAM[7] Crp60comp.4
SPOAL7 0.500
SPOFFS7 0 Hz
SPW7 49.57699966 W
GPNAM[1] SMSQ10.100
GPZ1 80.00 %
GPNAM[3] SMSQ10.100
GPZ3 14.00 %
P16 1000.00 usec

F1 - Acquisition parameters
TD 360
SFO1 150.9305 MHz
FIDRES 167.336014 Hz
SW 199.565 ppm
FnMODE Echo-Antiecho

F2 - Processing parameters
SI 1024
SF 600.1800285 MHz
WDW QSINE
SSB 2
LB 0 Hz
GB 0
PC 1.40

F1 - Processing parameters
SI 1024
MC2 echo-antiecho
SF 150.9153810 MHz
WDW QSINE
SSB 2
LB 0 Hz
GB 0

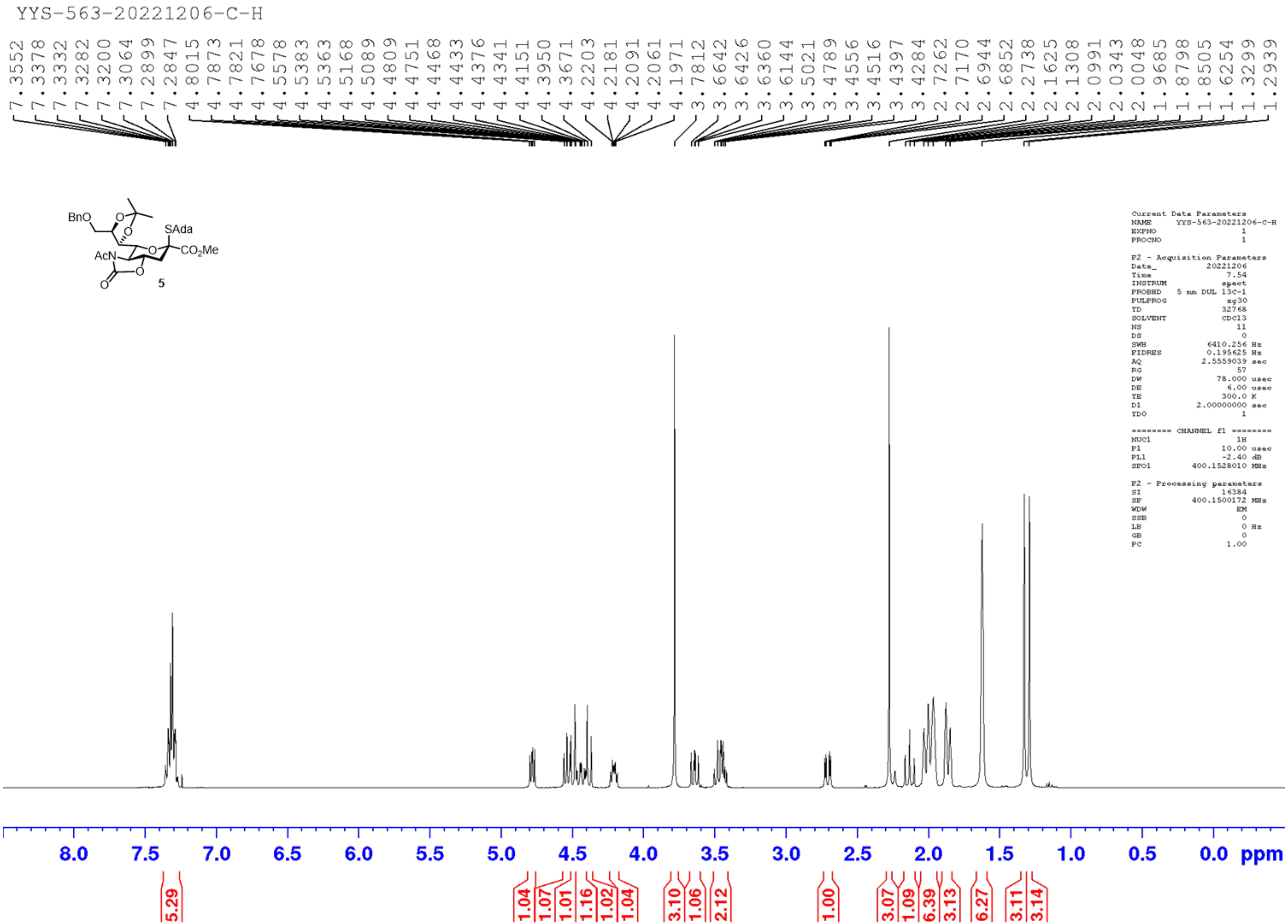
YYS-563-20221206-C-H
7.3552
7.3378
7.3332
7.3282
7.3200
7.3064
7.2899
7.2847
4.8015
4.7873
4.7821
4.7678
4.5578
4.5383
4.5363
4.5168
4.5089
4.4809
4.4751
4.4468
4.4433
4.4376
4.4341
4.4151
4.3950
4.3671
4.2203
4.2181
4.2091
4.2061
4.1971
3.7812
3.6642
3.6426
3.6360
3.6144
3.5021
3.4789
3.4556
3.4516
3.4397
3.4284
2.7262
2.7170
2.6944
2.6852
2.2738
2.1625
2.1308
2.0991
2.0343
2.0048
1.9685
1.8798
1.8505
1.6254
1.3299
1.2939
BnO
SAda
CO2Me
AcN
O
5
Current Data Parameters
NAME YYS-563-20221206-C-H
EXPNO 1
PROCNO 1
F2 - Acquisition Parameters
Date_ 20221206
Time 7.54
INSTRUM spect
PROBHD 5 mm DUL 13C-1
PULPROG zg30
TD 32768
SOLVENT CDCl3
NS 11
DS 0
SWH 6410.256 Hz
FIDRES 0.195625 Hz
AQ 2.5559039 sec
RG 57
DW 78.000 usec
DE 6.00 usec
TE 300.0 K
D1 2.00000000 sec
TD0 1
======== CHANNEL f1 ========
NUC1 1H
P1 10.00 usec
PL1 -2.40 dB
SFO1 400.1528010 MHz
F2 - Processing parameters
SI 16384
SF 400.1500172 MHz
WDW EM
SSB 0
LB 0 Hz
GB 0
PC 1.00
8.0
7.5
7.0
6.5
6.0
5.5
5.0
4.5
4.0
3.5
3.0
2.5
2.0
1.5
1.0
0.5
0.0
ppm
5.29
1.04
1.07
1.01
1.16
1.02
1.04
3.10
1.06
2.12
1.00
3.07
1.09
6.39
3.13
6.27
3.11
3.14

YYS-563-20221206-C

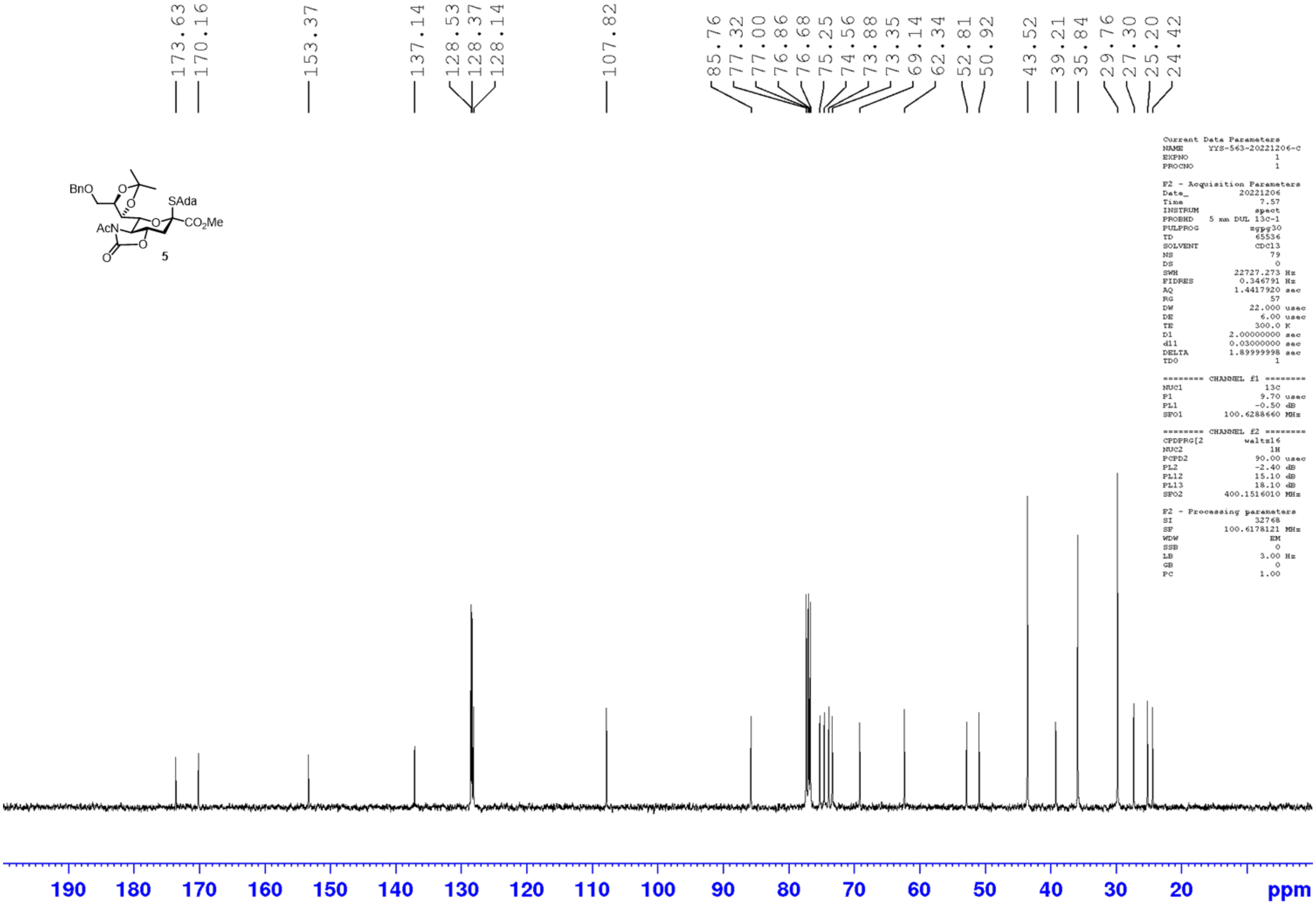

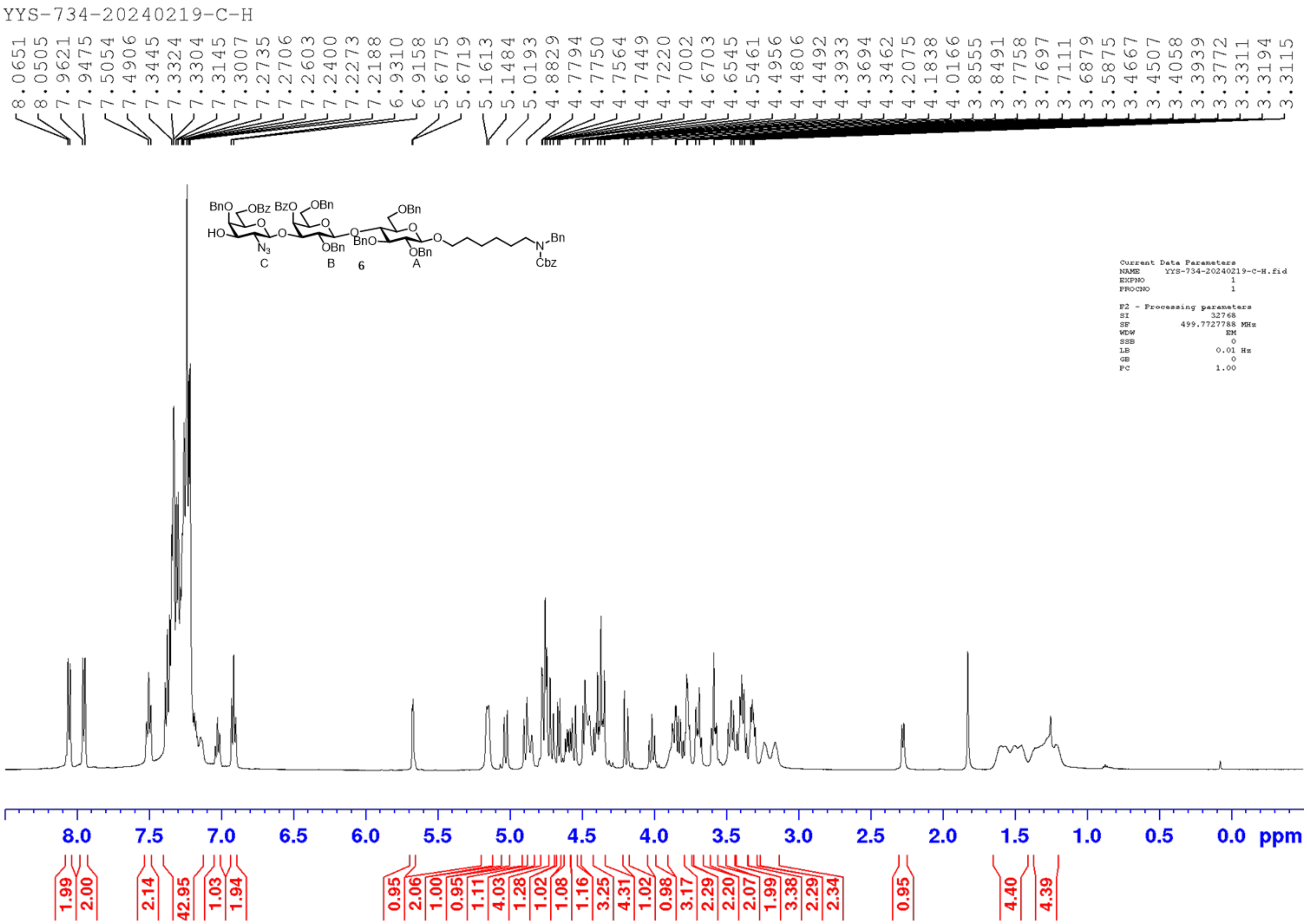

YYS-734-20240219-C-H
8.0651 8.0505 7.9621 7.9475 7.5054 7.4906 7.3445 7.3324 7.3304 7.3145 7.3007 7.2735 7.2706 7.2603 7.2400 7.2273 7.2188 6.9310 6.9158 5.6775 5.6719 5.1613 5.1484 5.0193 4.8829 4.7794 4.7750 4.7564 4.7449 4.7220 4.7002 4.6703 4.6545 4.5461 4.4956 4.4806 4.4492 4.3933 4.3694 4.3462 4.2075 4.1838 4.0166 3.8555 3.8491 3.7758 3.7697 3.7111 3.6879 3.5875 3.4667 3.4507 3.4058 3.3939 3.3772 3.3311 3.3194 3.3115
BnO OBz BzO OBn OBn
HO N3 OBn BnO OBn N Bn Cbz
C B 6 A
Current Data Parameters
NAME YYS-734-20240219-C-H.fid
EXPNO 1
PROCNO 1
F2 - Processing parameters
SI 32768
SF 499.7727788 MHz
WDW EM
SSB 0
LB 0.01 Hz
GB 0
PC 1.00
8.0 7.5 7.0 6.5 6.0 5.5 5.0 4.5 4.0 3.5 3.0 2.5 2.0 1.5 1.0 0.5 0.0 ppm
1.99 2.00 2.14 42.95 1.03 1.94 0.95 2.06 1.00 0.95 1.11 4.03 1.28 1.02 1.08 1.16 3.25 4.31 1.02 0.98 3.17 2.29 2.20 2.07 1.99 3.38 2.29 2.34 0.95 4.40 4.39

YYS-734-C-20240219-C

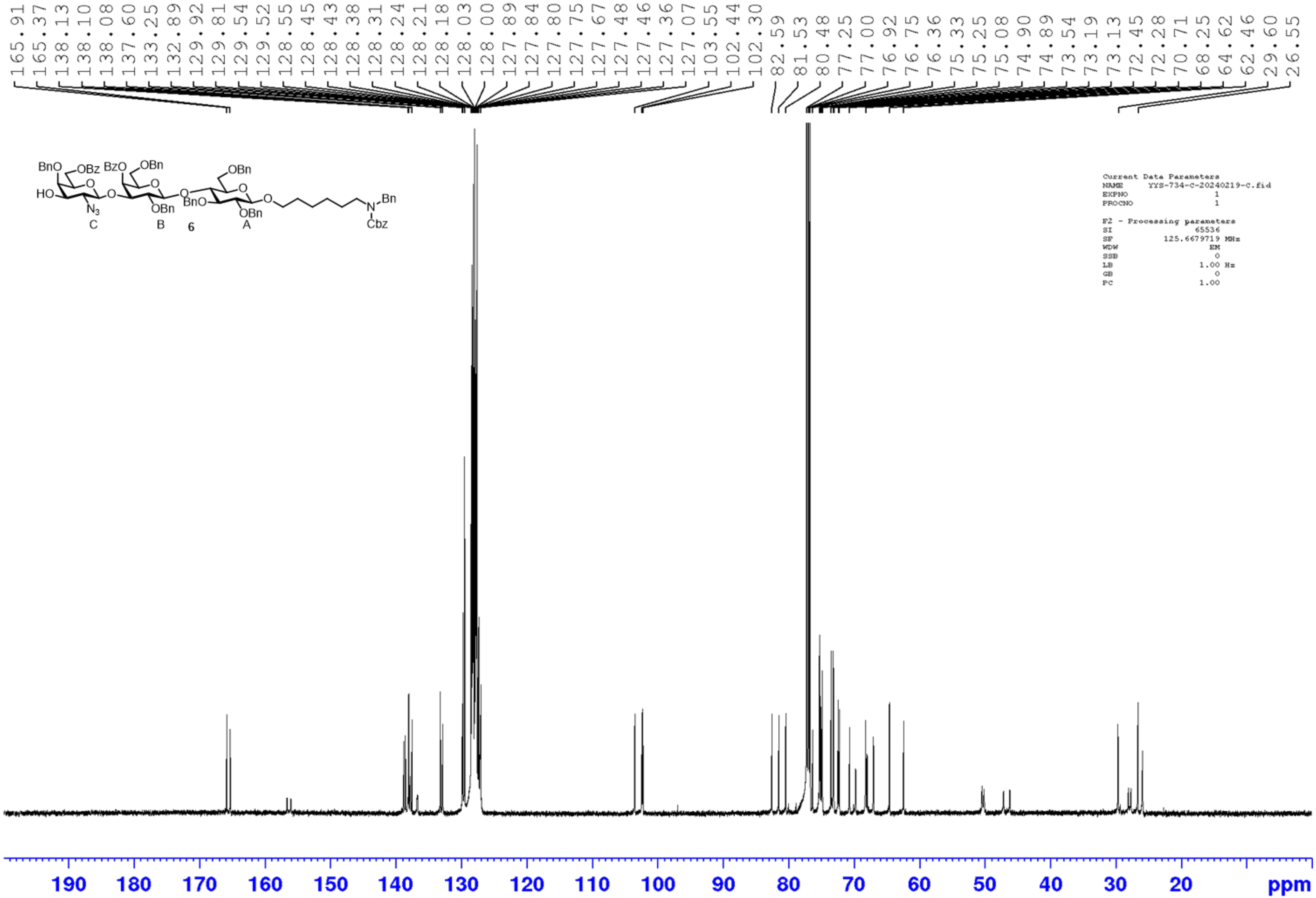

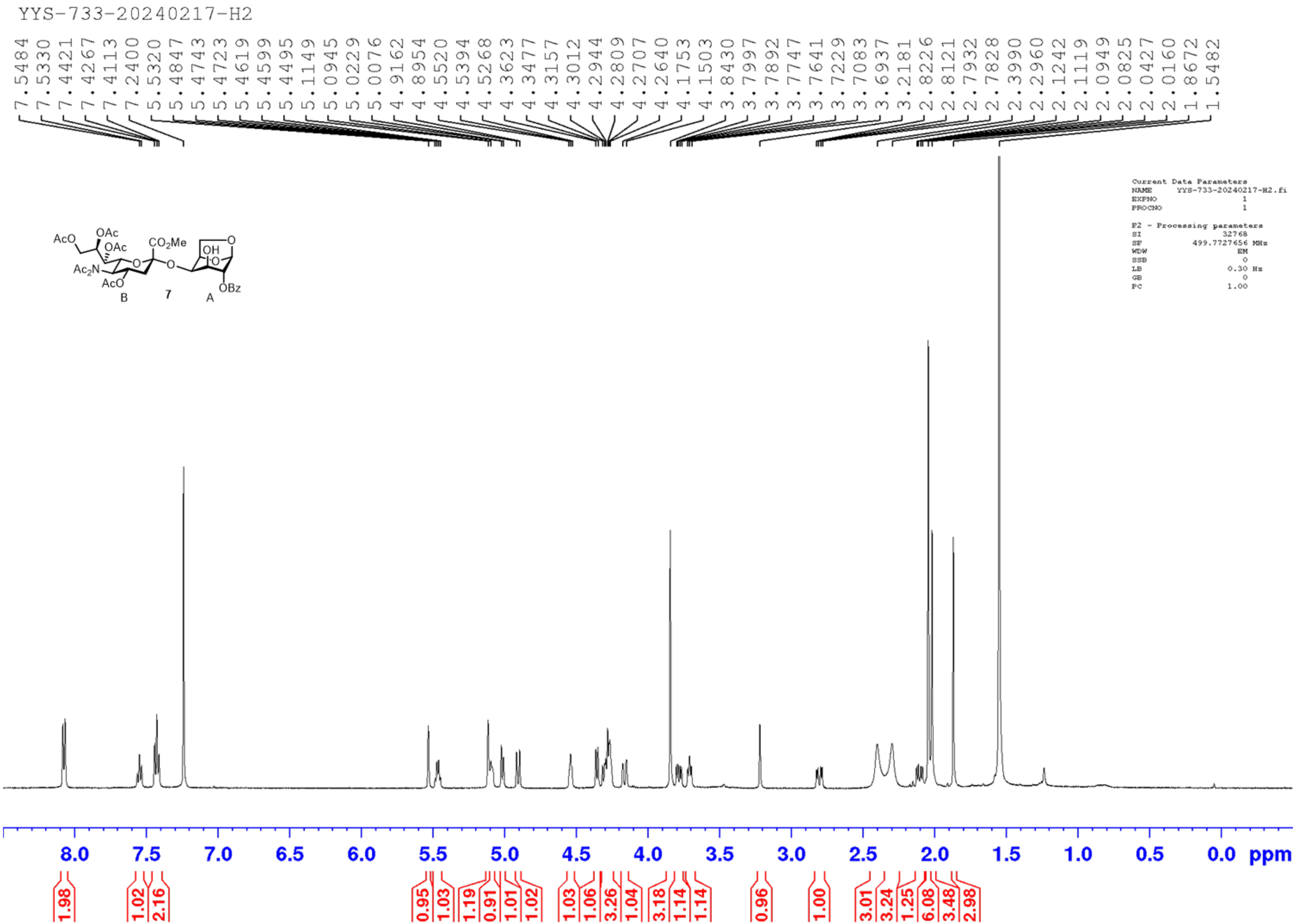
YYS-733-20240217-H2
7.5484
7.5330
7.4421
7.4267
7.4113
7.2400
5.5320
5.4847
5.4743
5.4723
5.4619
5.4599
5.4495
5.1149
5.0945
5.0229
5.0076
4.9162
4.8954
4.5520
4.5394
4.5268
4.3623
4.3477
4.3157
4.3012
4.2944
4.2809
4.2707
4.2640
4.1753
4.1503
3.8430
3.7997
3.7892
3.7747
3.7641
3.7229
3.7083
3.6937
3.2181
2.8226
2.8121
2.7932
2.7828
2.3990
2.2960
2.1242
2.1119
2.0949
2.0825
2.0427
2.0160
1.8672
1.5482
Current Data Parameters
NAME YYS-733-20240217-H2.fi
EXPNO 1
PROCNO 1
F2 - Processing parameters
SI 32768
SF 499.7727656 MHz
WDW EM
SSB 0
LB 0.30 Hz
GB 0
PC 1.00
AcO
OAc
OAc
CO_2Me
OH
Ac_2N
AcO
OBz
B
7
A
8.0
7.5
7.0
6.5
6.0
5.5
5.0
4.5
4.0
3.5
3.0
2.5
2.0
1.5
1.0
0.5
0.0
ppm
1.98
1.02
2.16
0.95
1.03
1.19
0.91
1.01
1.02
1.03
1.06
3.26
1.04
3.18
1.14
1.14
0.96
1.00
3.01
3.24
1.25
6.08
3.48
2.98

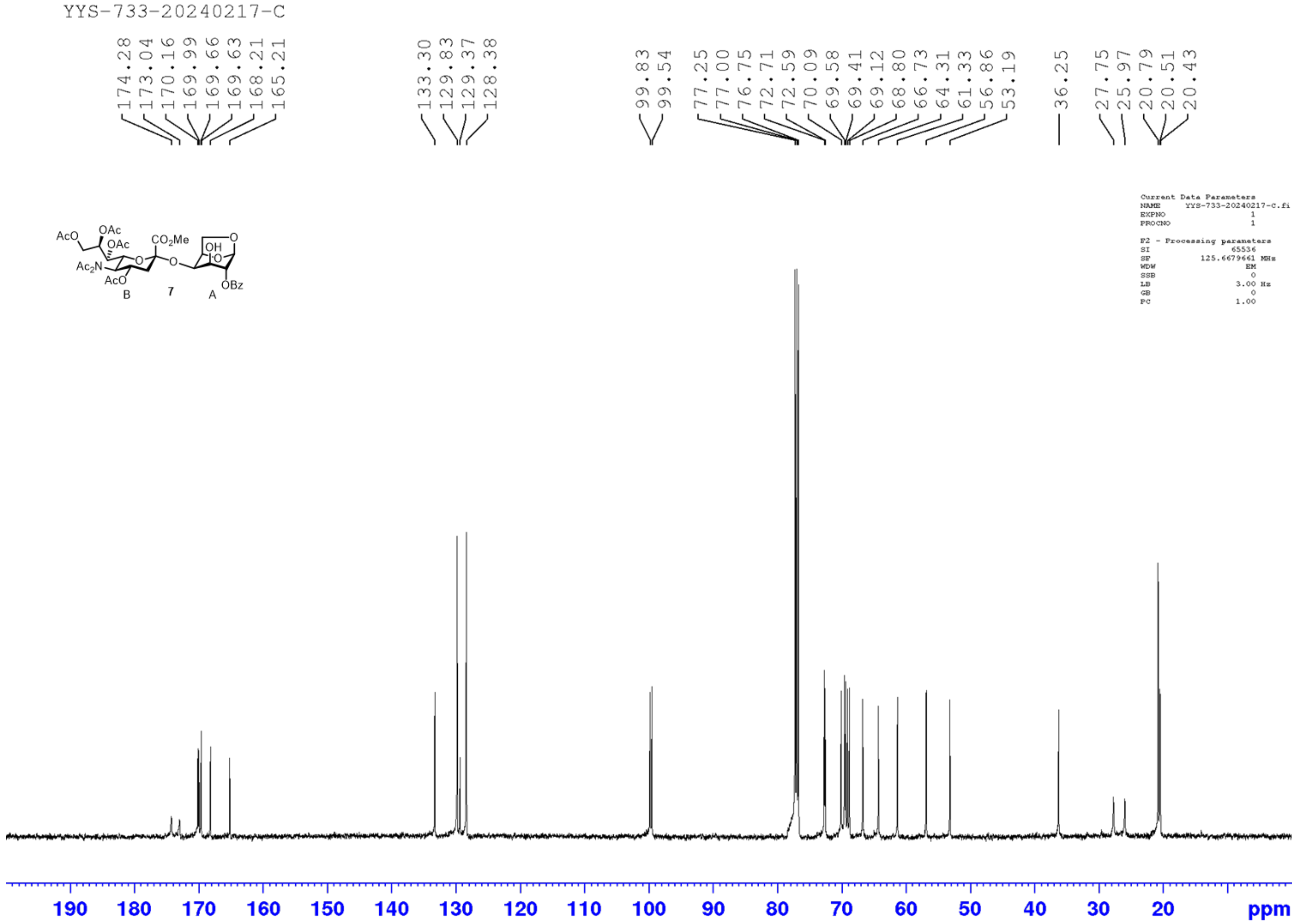
YYS-733-20240217-C
174.28
173.04
170.16
169.99
169.66
169.63
168.21
165.21
133.30
129.83
129.37
128.38
99.83
99.54
77.25
77.00
76.75
72.71
72.59
70.09
69.58
69.41
69.12
68.80
66.73
64.31
61.33
56.86
53.19
36.25
27.75
25.97
20.79
20.51
20.43
AcO
OAc
OAc
CO_2Me
Ac_2N
AcO
O
OH
OBz
B
7
A
Current Data Parameters
NAME YYS-733-20240217-C.fi
EXPNO 1
PROCNO 1
F2 - Processing parameters
SI 65536
SF 125.6679661 MHz
WDW EM
SSB 0
LB 3.00 Hz
GB 0
PC 1.00
190
180
170
160
150
140
130
120
110
100
90
80
70
60
50
40
30
20
ppm

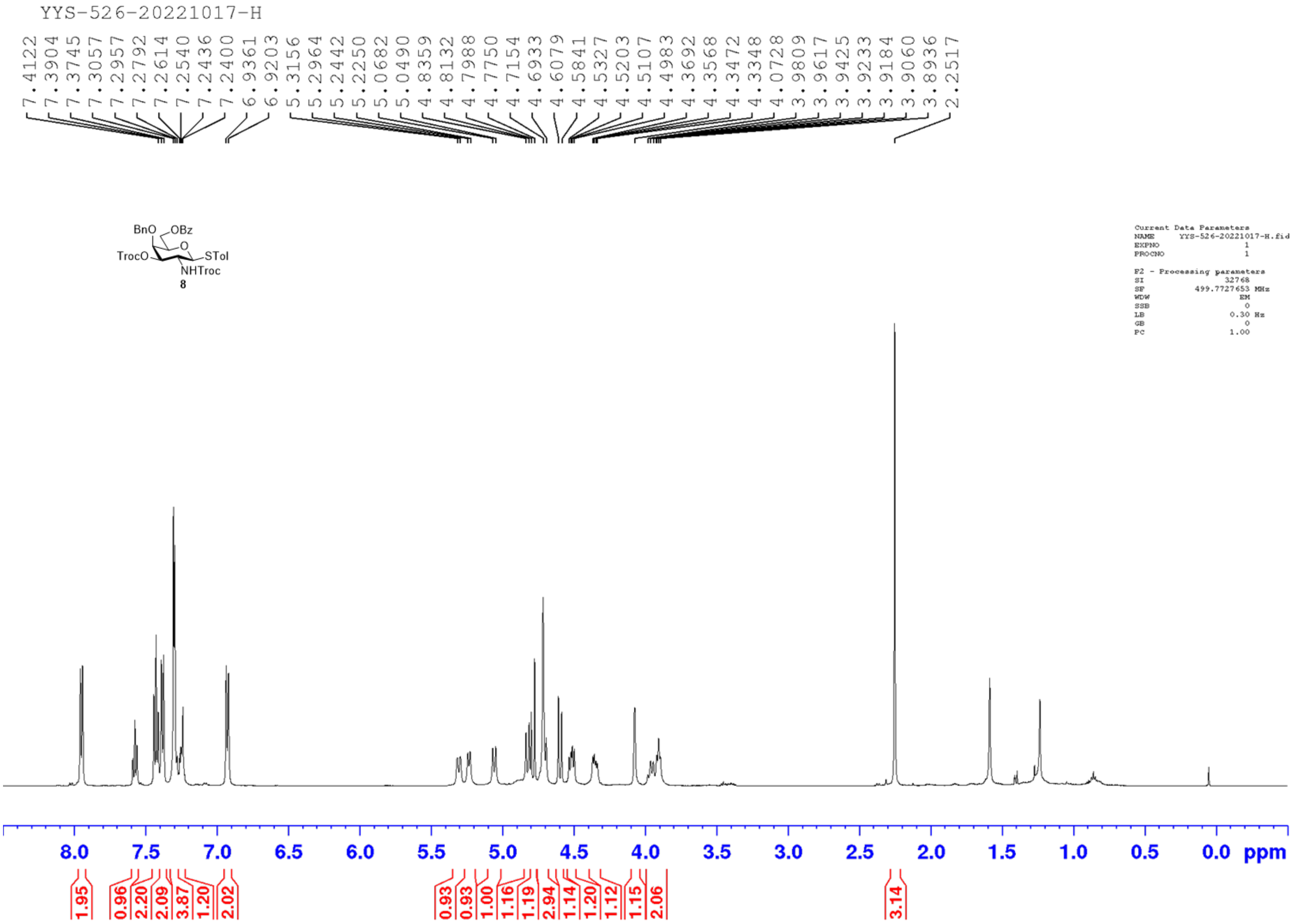
YYS-526-20221017-H
7.4122
7.3904
7.3745
7.3057
7.2957
7.2792
7.2614
7.2540
7.2436
7.2400
6.9361
6.9203
5.3156
5.2964
5.2442
5.2250
5.0682
5.0490
4.8359
4.8132
4.7988
4.7750
4.7154
4.6933
4.6079
4.5841
4.5327
4.5203
4.5107
4.4983
4.3692
4.3568
4.3472
4.3348
4.0728
3.9809
3.9617
3.9425
3.9233
3.9184
3.9060
3.8936
2.2517
BnO
OBz
O
TrocO
STol
NHTroc
8
Current Data Parameters
NAME YYS-526-20221017-H.fid
EXPNO 1
PROCNO 1
F2 - Processing parameters
SI 32768
SF 499.7727653 MHz
WDW EM
SSB 0
LB 0.30 Hz
GB 0
PC 1.00
8.0
7.5
7.0
6.5
6.0
5.5
5.0
4.5
4.0
3.5
3.0
2.5
2.0
1.5
1.0
0.5
0.0
ppm
1.95
0.96
2.20
2.09
3.87
1.20
2.02
0.93
0.93
1.00
1.16
1.19
2.94
1.14
1.20
1.12
1.15
2.06
3.14

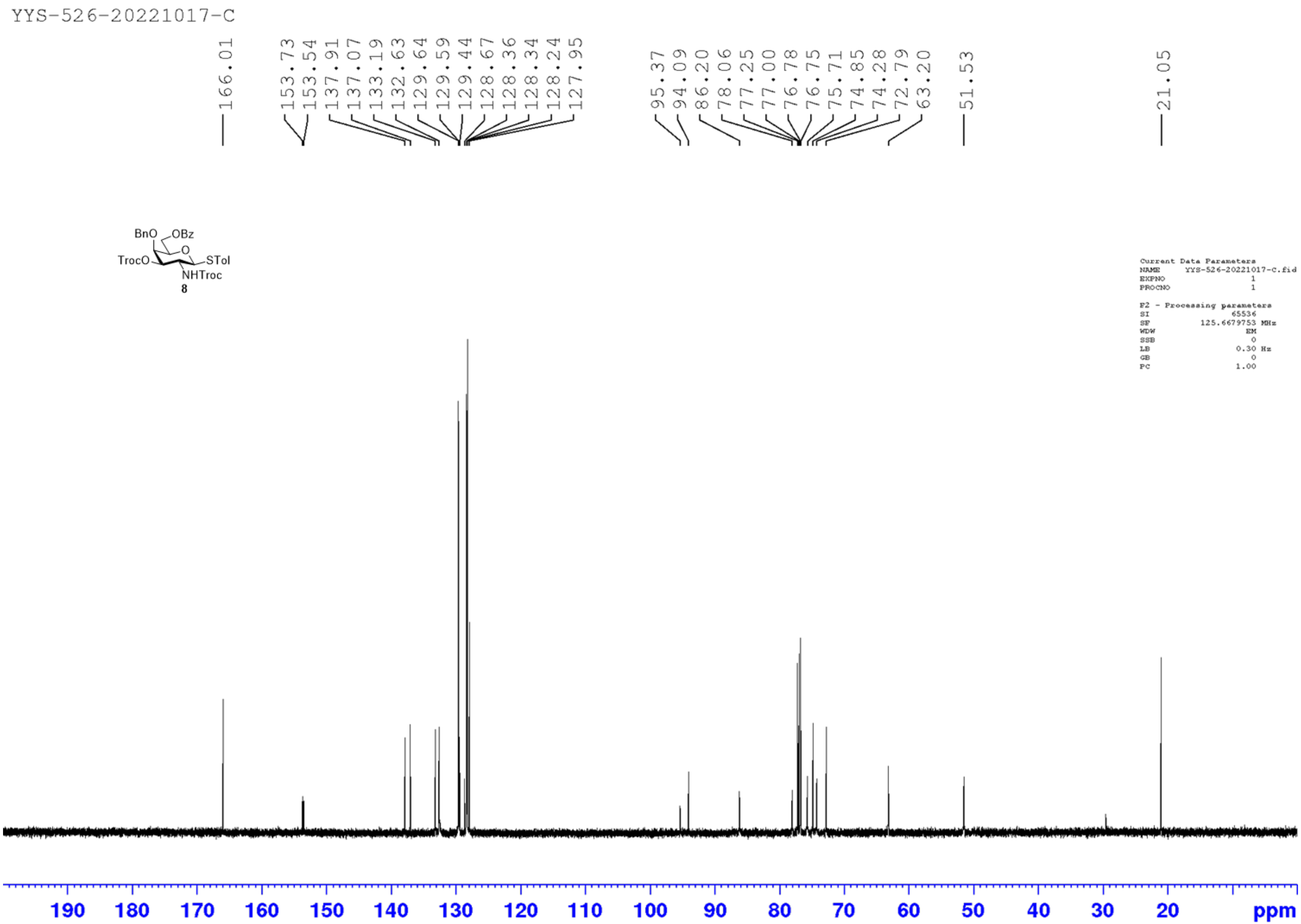
YYS-526-20221017-C
166.01
153.73
153.54
137.91
137.07
133.19
132.63
129.64
129.59
129.44
128.67
128.36
128.34
128.24
127.95
95.37
94.09
86.20
78.06
77.25
77.00
76.78
76.75
75.71
74.85
74.28
72.79
63.20
51.53
21.05
BnO
OBz
O
TrocO
STol
NHTroc
8
Current Data Parameters
NAME YYS-526-20221017-C.fid
EXPNO 1
PROCNO 1
F2 - Processing parameters
SI 65536
SF 125.6679753 MHz
WDW EM
SSB 0
LB 0.30 Hz
GB 0
PC 1.00
190 180 170 160 150 140 130 120 110 100 90 80 70 60 50 40 30 20 ppm

YYS-717-20231226-H

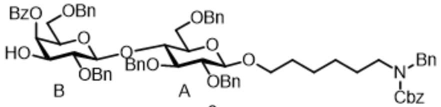

9

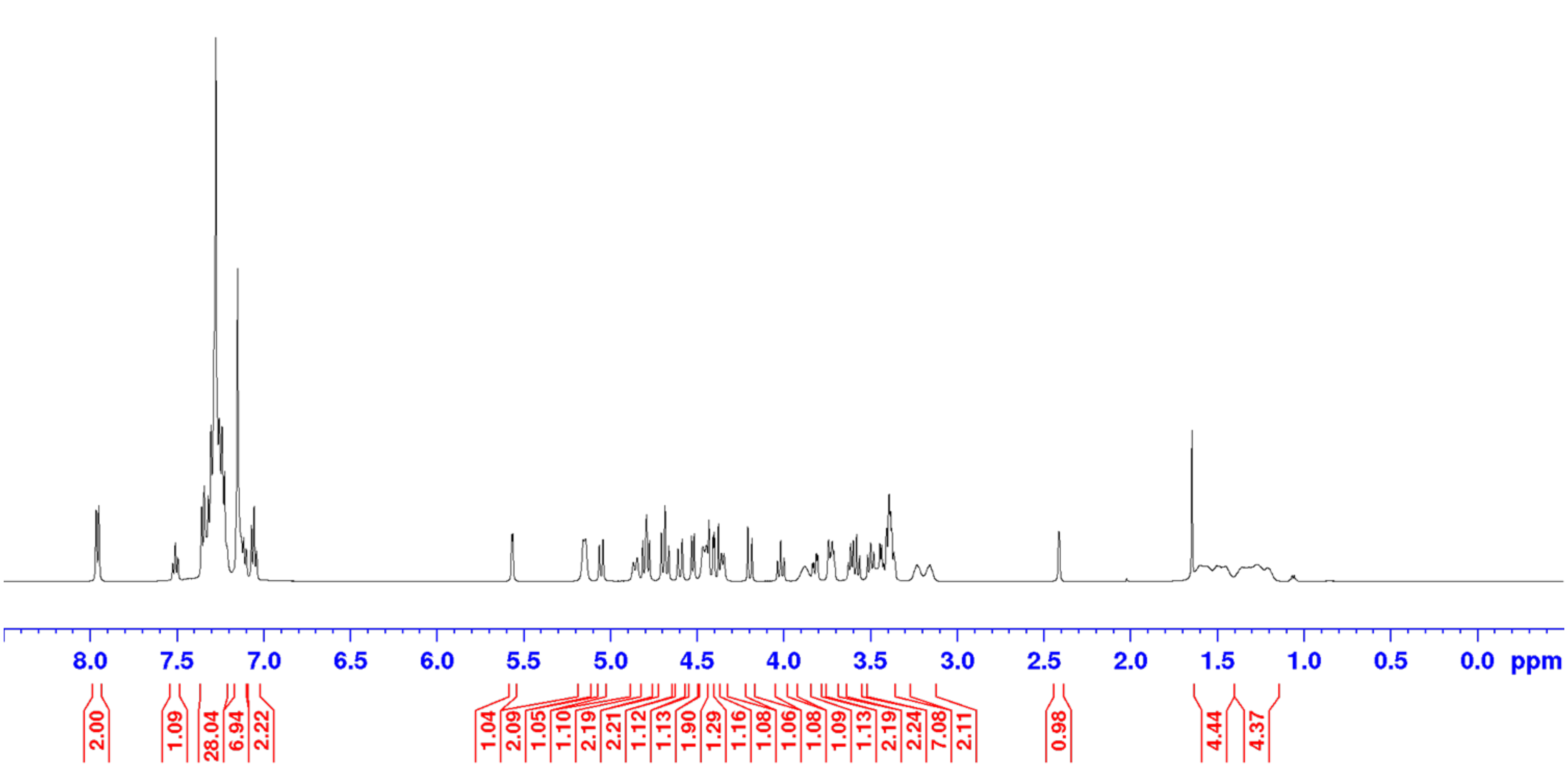

YYS-429-20220104-C

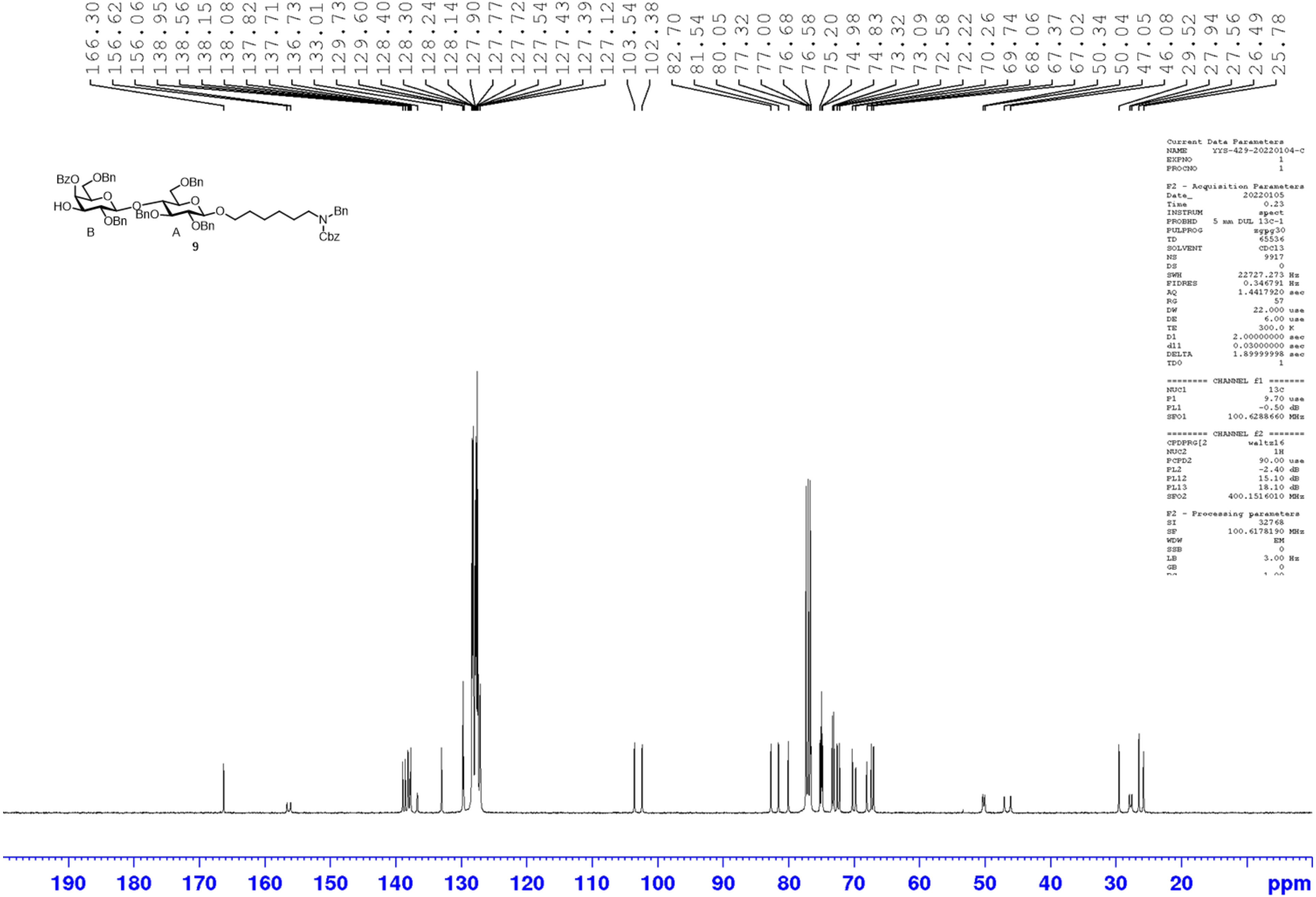

YYS-550-20221117-C-H

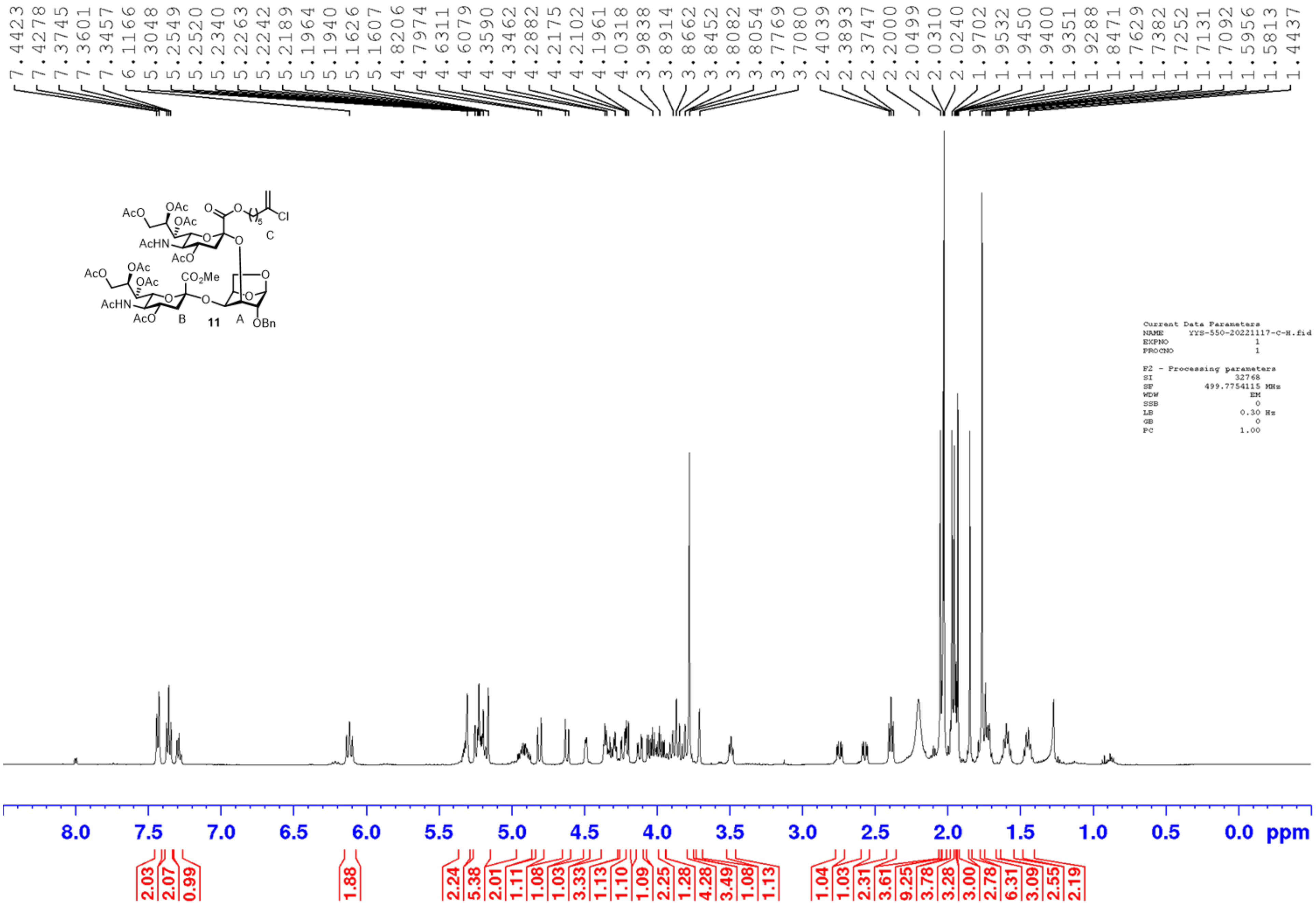

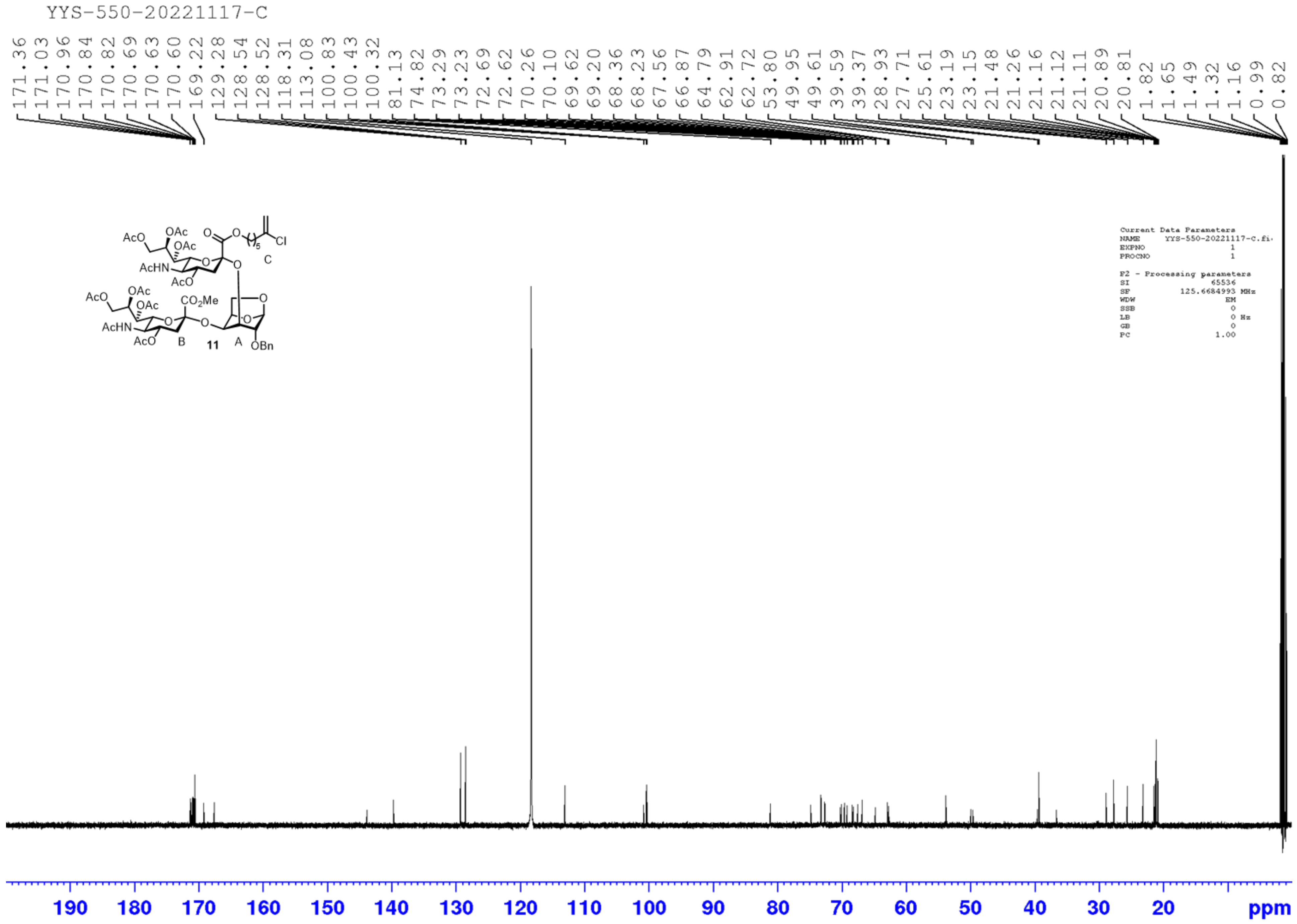

YYS-550-20221117-C
171.36
171.03
170.96
170.84
170.82
170.69
170.63
170.60
169.22
129.28
128.54
128.52
118.31
113.08
100.83
100.43
100.32
81.13
74.82
73.29
73.23
72.69
72.62
70.26
70.10
69.62
69.20
68.36
68.23
67.56
66.87
64.79
62.91
62.72
53.80
49.95
49.61
39.59
39.37
28.93
27.71
25.61
23.19
23.15
21.48
21.26
21.16
21.12
21.11
20.89
20.81
1.82
1.65
1.49
1.32
1.16
0.99
0.82
AcO
OAc
OAc
AcHN
AcO
O
C
Cl
5
CO2Me
B
11
A
OBn
Current Data Parameters
NAME YYS-550-20221117-C.fi
EXPNO 1
PROCNO 1
F2 - Processing parameters
SI 65536
SF 125.6684993 MHz
WDW EM
SSB 0
LB 0 Hz
GB 0
PC 1.00
190
180
170
160
150
140
130
120
110
100
90
80
70
60
50
40
30
20
ppm

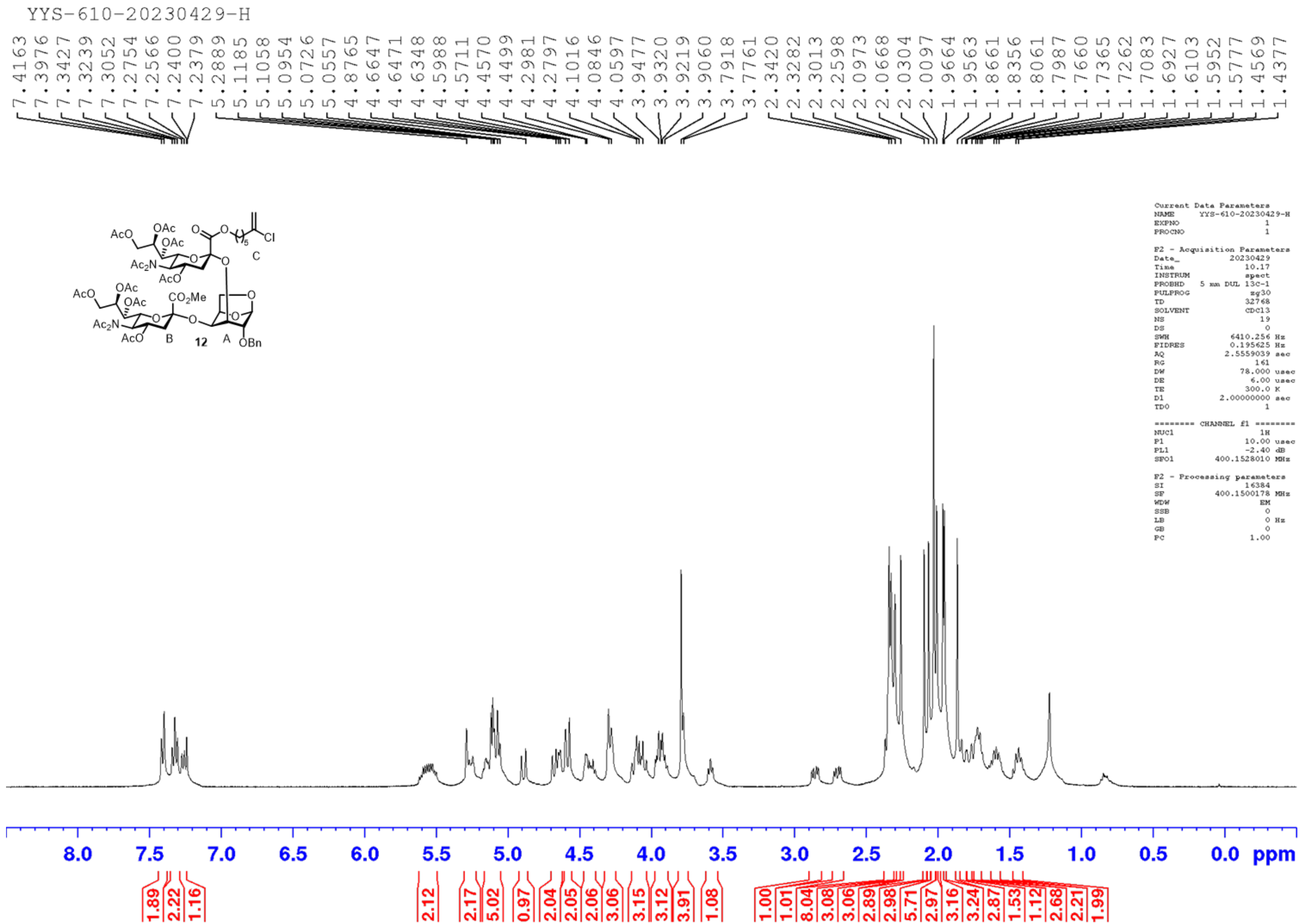

YYS-610-20230429-H
7.4163
7.3976
7.3427
7.3239
7.3052
7.2754
7.2566
7.2400
7.2379
5.2889
5.1185
5.1058
5.0954
5.0726
5.0557
4.8765
4.6647
4.6471
4.6348
4.5988
4.5711
4.4570
4.4499
4.2981
4.2797
4.1016
4.0846
4.0597
3.9477
3.9320
3.9219
3.9060
3.7918
3.7761
2.3420
2.3282
2.3013
2.2598
2.0973
2.0668
2.0304
2.0097
1.9664
1.9563
1.8661
1.8356
1.8061
1.7987
1.7660
1.7365
1.7262
1.7083
1.6927
1.6103
1.5952
1.5777
1.4569
1.4377
AcO
OAc
OAc
Ac$_2$N
AcO
O
O
O
O
Cl
C
AcO
OAc
OAc
CO$_2$Me
Ac$_2$N
AcO
B
12
A
OBn
Current Data Parameters
NAME YYS-610-20230429-H
EXPNO 1
PROCNO 1
F2 - Acquisition Parameters
Date_ 20230429
Time 10.17
INSTRUM spect
PROBHD 5 mm DUL 13C-1
PULPROG zg30
TD 32768
SOLVENT CDCl3
NS 19
DS 0
SWH 6410.256 Hz
FIDRES 0.195625 Hz
AQ 2.5559039 sec
RG 161
DW 78.000 usec
DE 6.00 usec
TE 300.0 K
D1 2.00000000 sec
TD0 1
======== CHANNEL f1 ========
NUC1 1H
P1 10.00 usec
PL1 -2.40 dB
SFO1 400.1528010 MHz
F2 - Processing parameters
SI 16384
SF 400.1500178 MHz
WDW EM
SSB 0
LB 0 Hz
GB 0
PC 1.00
8.0
7.5
7.0
6.5
6.0
5.5
5.0
4.5
4.0
3.5
3.0
2.5
2.0
1.5
1.0
0.5
0.0
ppm
1.89
2.22
1.16
2.12
2.17
5.02
0.97
2.04
2.05
2.06
3.06
3.15
3.12
3.91
1.08
1.00
1.01
8.04
3.08
3.06
2.89
2.98
5.71
2.97
3.16
3.24
2.87
1.53
1.12
2.68
2.21
1.99

YYS-610-20230429-C

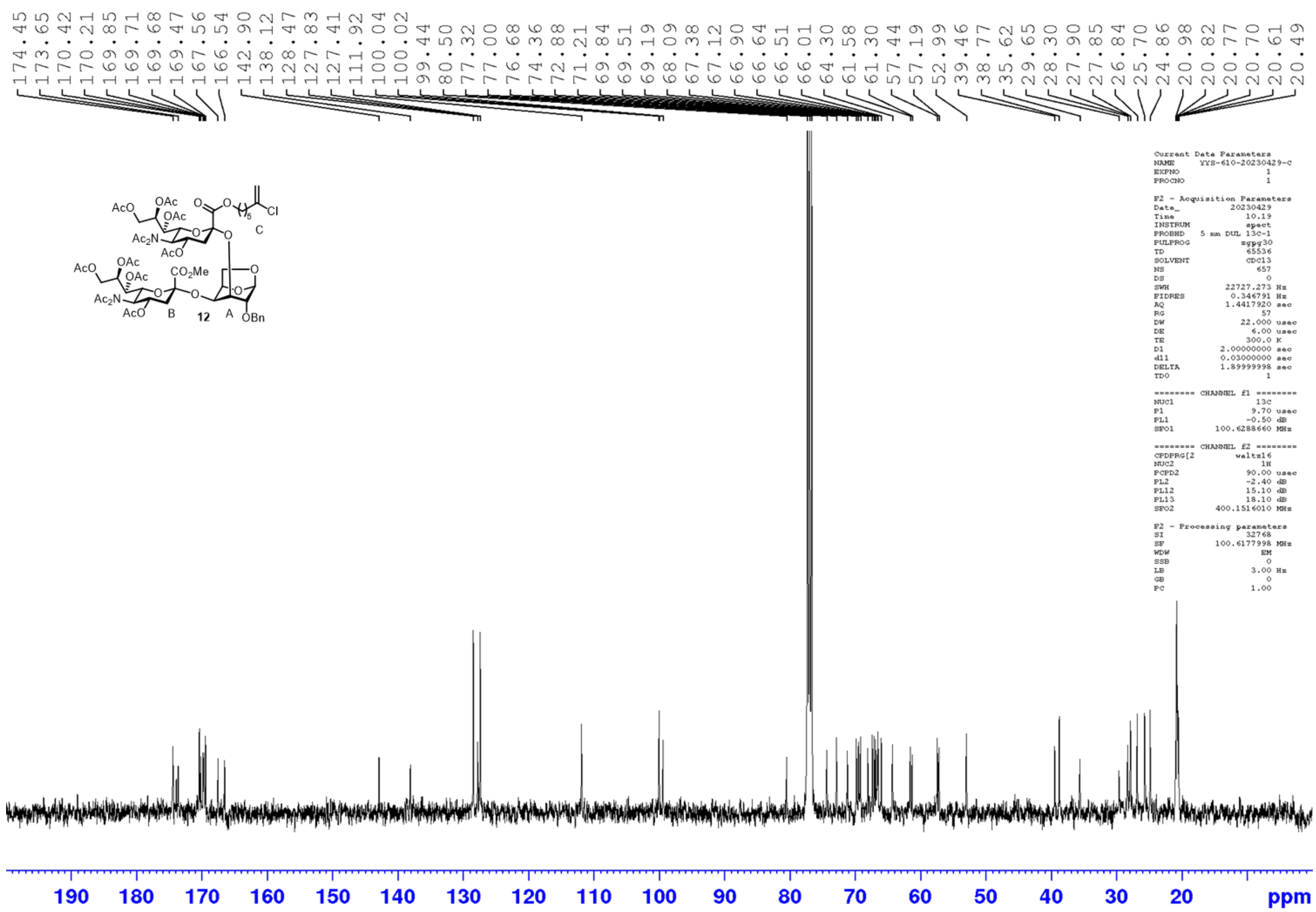

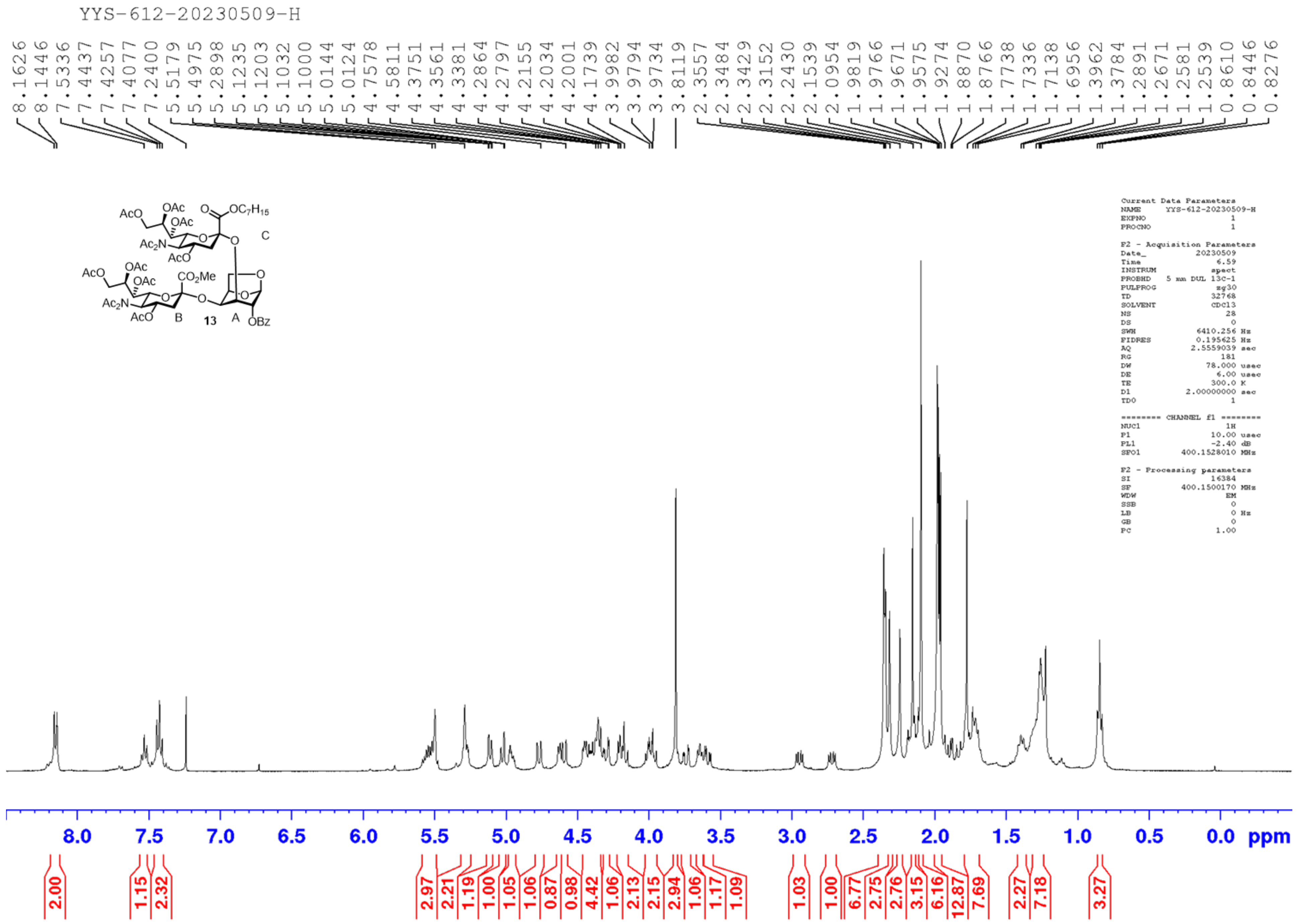
YYS-612-20230509-H
13
A
B
C
ppm
Current Data Parameters
NAME YYS-612-20230509-H
EXPNO 1
PROCNO 1
F2 - Acquisition Parameters
Date_ 20230509
Time 6.59
INSTRUM spect
PROBHD 5 mm DUL 13C-1
PULPROG zg30
TD 32768
SOLVENT CDCl3
NS 28
DS 0
SWH 6410.256 Hz
FIDRES 0.195625 Hz
AQ 2.5559039 sec
RG 181
DW 78.000 usec
DE 6.00 usec
TE 300.0 K
D1 2.00000000 sec
TD0 1
======== CHANNEL f1 ========
NUC1 1H
P1 10.00 usec
PL1 -2.40 dB
SFO1 400.1528010 MHz
F2 - Processing parameters
SI 16384
SF 400.1500170 MHz
WDW EM
SSB 0
LB 0 Hz
GB 0
PC 1.00

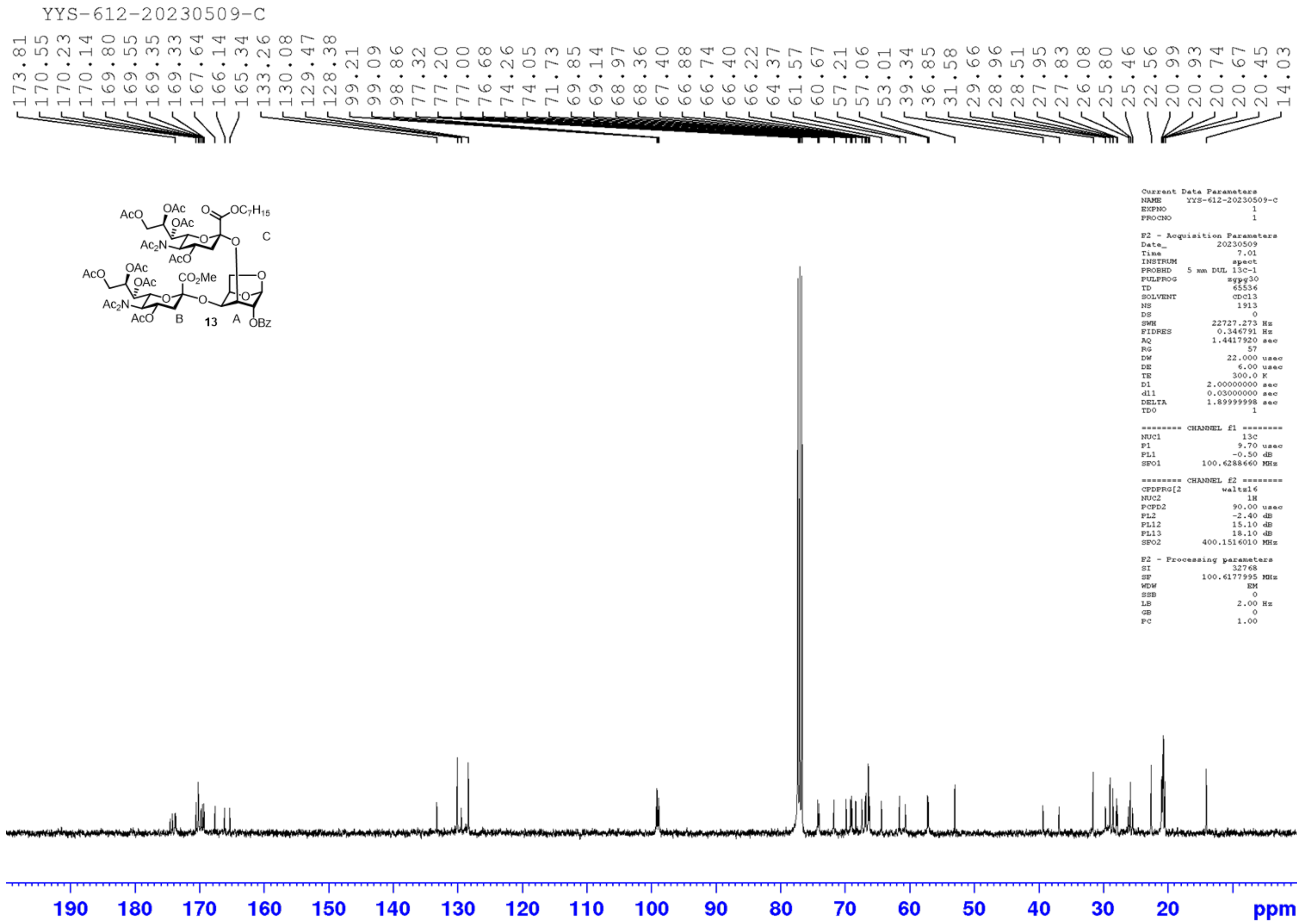

YYS-612-20230509-C
173.81 170.55 170.23 170.14 169.80 169.55 169.35 169.33 167.64 166.14 165.34 133.26 130.08 129.47 128.38 99.21 99.09 98.86 77.32 77.20 77.00 76.68 74.26 74.05 71.73 69.85 69.14 68.97 68.36 67.40 66.88 66.74 66.40 66.22 64.37 61.57 60.67 57.21 57.06 53.01 39.34 36.85 31.58 29.66 28.96 28.51 27.95 27.83 26.08 25.80 25.46 22.56 20.99 20.93 20.74 20.67 20.45 14.03
AcO
OAc
OAc
OC_7H_{15}
O
C
Ac_2N
AcO
OAc
OAc
CO_2Me
Ac_2N
AcO
B
13
A
OBz
Current Data Parameters
NAME YYS-612-20230509-C
EXPNO 1
PROCNO 1
F2 - Acquisition Parameters
Date_ 20230509
Time 7.01
INSTRUM spect
PROBHD 5 mm DUL 13C-1
PULPROG zgpg30
TD 65536
SOLVENT CDCl3
NS 1913
DS 0
SWH 22727.273 Hz
FIDRES 0.346791 Hz
AQ 1.4417920 sec
RG 57
DW 22.000 usec
DE 6.00 usec
TE 300.0 K
D1 2.00000000 sec
d11 0.03000000 sec
DELTA 1.89999998 sec
TD0 1
======== CHANNEL f1 ========
NUC1 13C
P1 9.70 usec
PL1 -0.50 dB
SFO1 100.6288660 MHz
======== CHANNEL f2 ========
CPDPRG[2 waltz16
NUC2 1H
PCPD2 90.00 usec
PL2 -2.40 dB
PL12 15.10 dB
PL13 18.10 dB
SFO2 400.1516010 MHz
F2 - Processing parameters
SI 32768
SF 100.6177995 MHz
WDW EM
SSB 0
LB 2.00 Hz
GB 0
PC 1.00
190 180 170 160 150 140 130 120 110 100 90 80 70 60 50 40 30 20 ppm

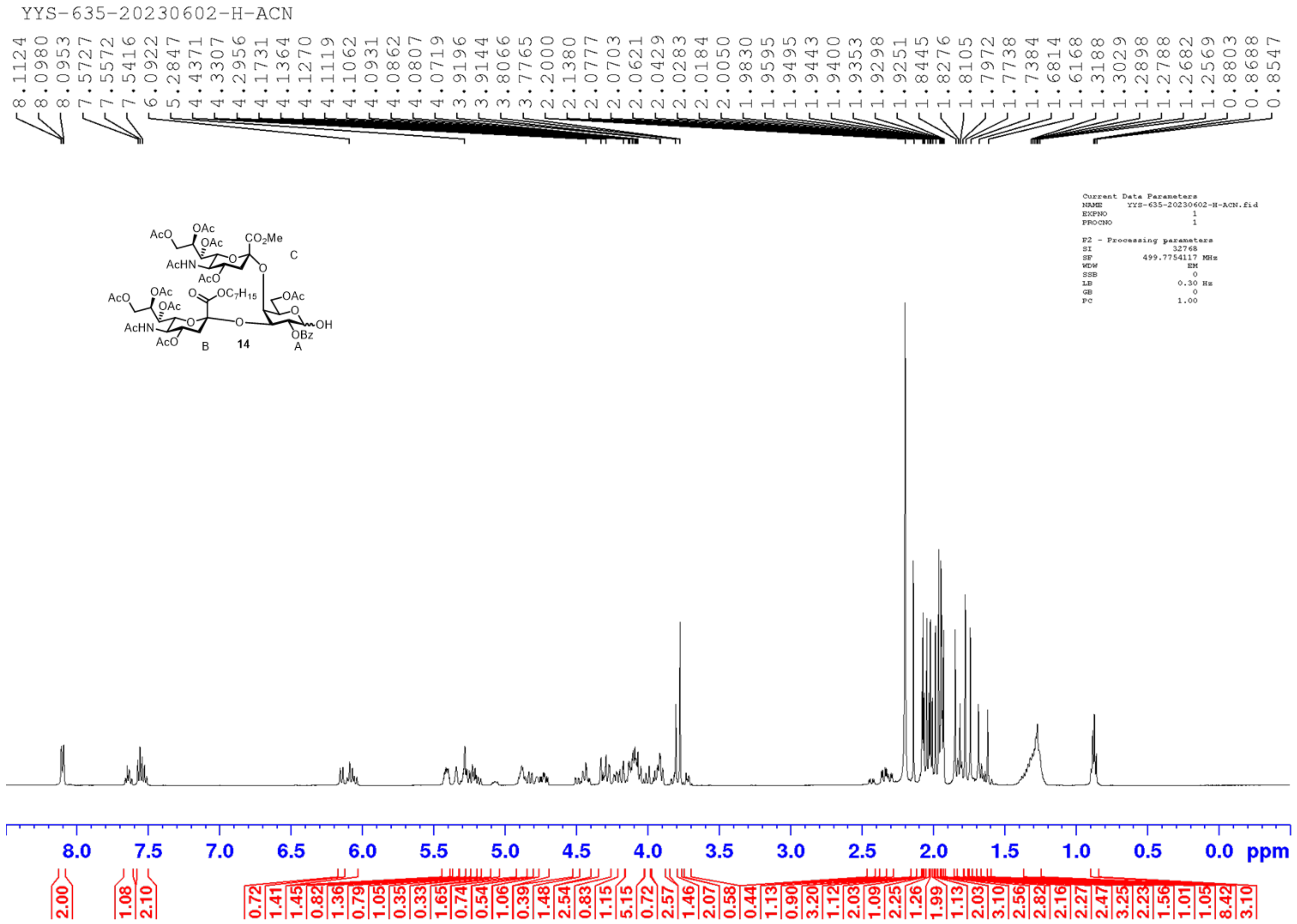

YYS-635-20230602-H-ACN
8.1124 8.0980 8.0953 7.5727 7.5572 7.5416 6.0922 5.2847 4.4371 4.3307 4.2956 4.1731 4.1364 4.1270 4.1119 4.1062 4.0931 4.0862 4.0807 4.0719 3.9196 3.9144 3.8066 3.7765 2.2000 2.1380 2.0777 2.0703 2.0621 2.0429 2.0283 2.0184 2.0050 1.9830 1.9595 1.9495 1.9443 1.9400 1.9353 1.9298 1.9251 1.8445 1.8276 1.8105 1.7972 1.7738 1.7384 1.6814 1.6168 1.3188 1.3029 1.2898 1.2788 1.2682 1.2569 0.8803 0.8688 0.8547
AcO
OAc
OAc
CO2Me
AcHN
O
AcO
C
O
OAc
O
OC7H15
OAc
AcHN
O
AcO
B
14
OBz
A
OH
Current Data Parameters
NAME YYS-635-20230602-H-ACN.fid
EXPNO 1
PROCNO 1
F2 - Processing parameters
SI 32768
SF 499.7754117 MHz
WDW EM
SSB 0
LB 0.30 Hz
GB 0
PC 1.00
8.0 7.5 7.0 6.5 6.0 5.5 5.0 4.5 4.0 3.5 3.0 2.5 2.0 1.5 1.0 0.5 0.0 ppm
2.00 1.08 2.10 0.72 1.41 1.45 0.82 1.36 0.79 1.05 0.35 0.33 1.65 0.74 0.54 1.06 0.39 1.48 2.54 0.83 1.15 5.15 0.72 2.57 1.46 2.07 0.58 0.44 1.13 0.90 3.20 1.12 2.03 1.09 2.25 1.26 1.99 1.13 2.03 3.10 2.56 2.82 2.16 2.27 2.47 3.25 2.23 1.56 1.01 1.05 8.42 3.10

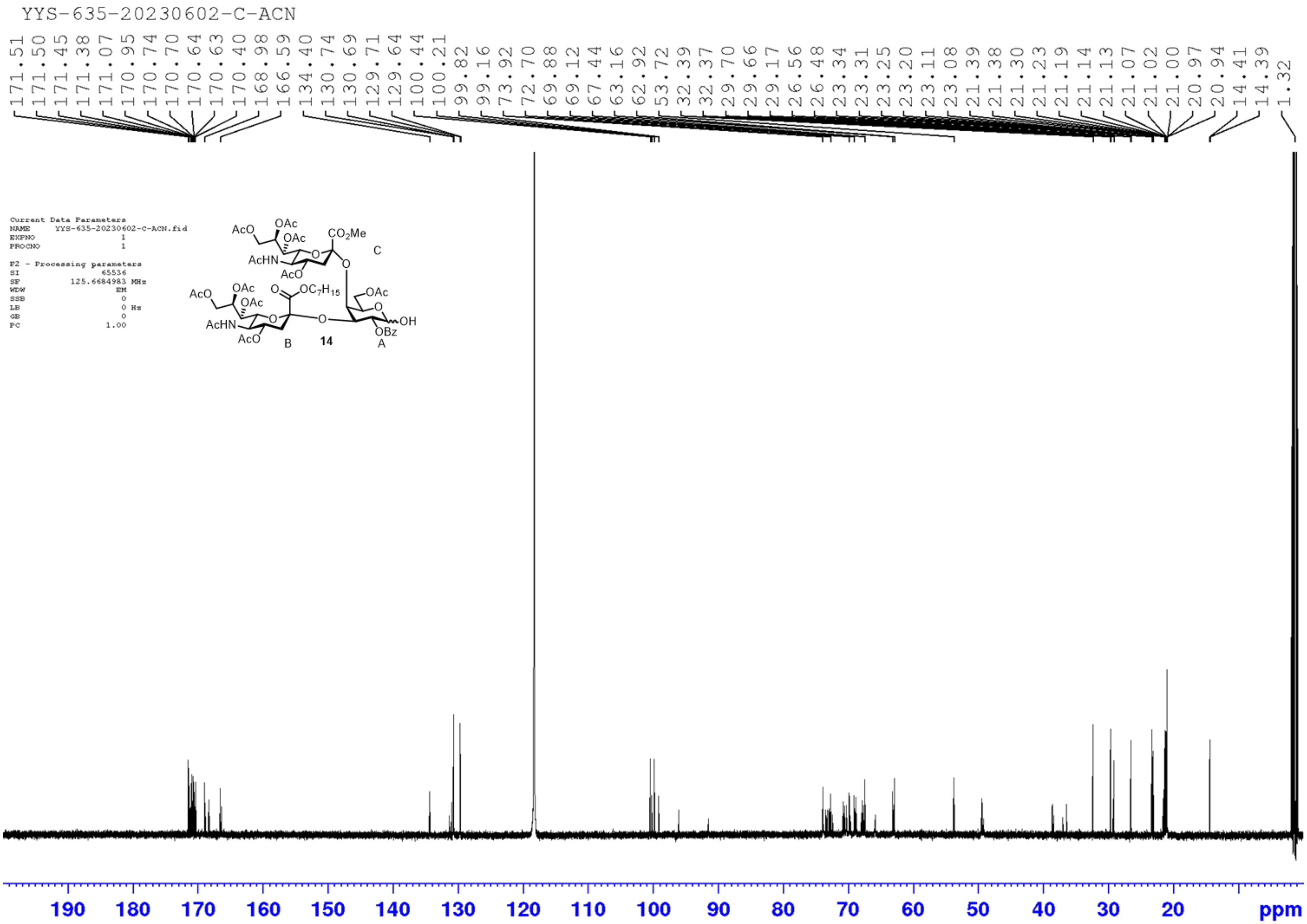

YYS-635-20230602-C-ACN
171.51 171.50 171.45 171.38 171.07 170.95 170.74 170.70 170.64 170.63 170.40 168.98 166.59 134.40 130.74 130.69 129.71 129.64 100.44 100.21 99.82 99.16 73.92 72.70 69.88 69.12 67.44 63.16 62.92 53.72 32.39 32.37 29.70 29.66 29.17 26.56 26.48 23.34 23.31 23.25 23.20 23.11 23.08 21.39 21.38 21.30 21.23 21.19 21.14 21.13 21.07 21.02 21.00 20.97 20.94 14.41 14.39 1.32
Current Data Parameters
NAME YYS-635-20230602-C-ACN.fid
EXPNO 1
PROCNO 1
F2 - Processing parameters
SI 65536
SF 125.6684983 MHz
WDW EM
SSB 0
LB 0 Hz
GB 0
PC 1.00
AcO
OAc
OAc
CO2Me
C
AcHN
AcO
O
OC7H15
OAc
B
14
A
OBz
OH
190 180 170 160 150 140 130 120 110 100 90 80 70 60 50 40 30 20 ppm

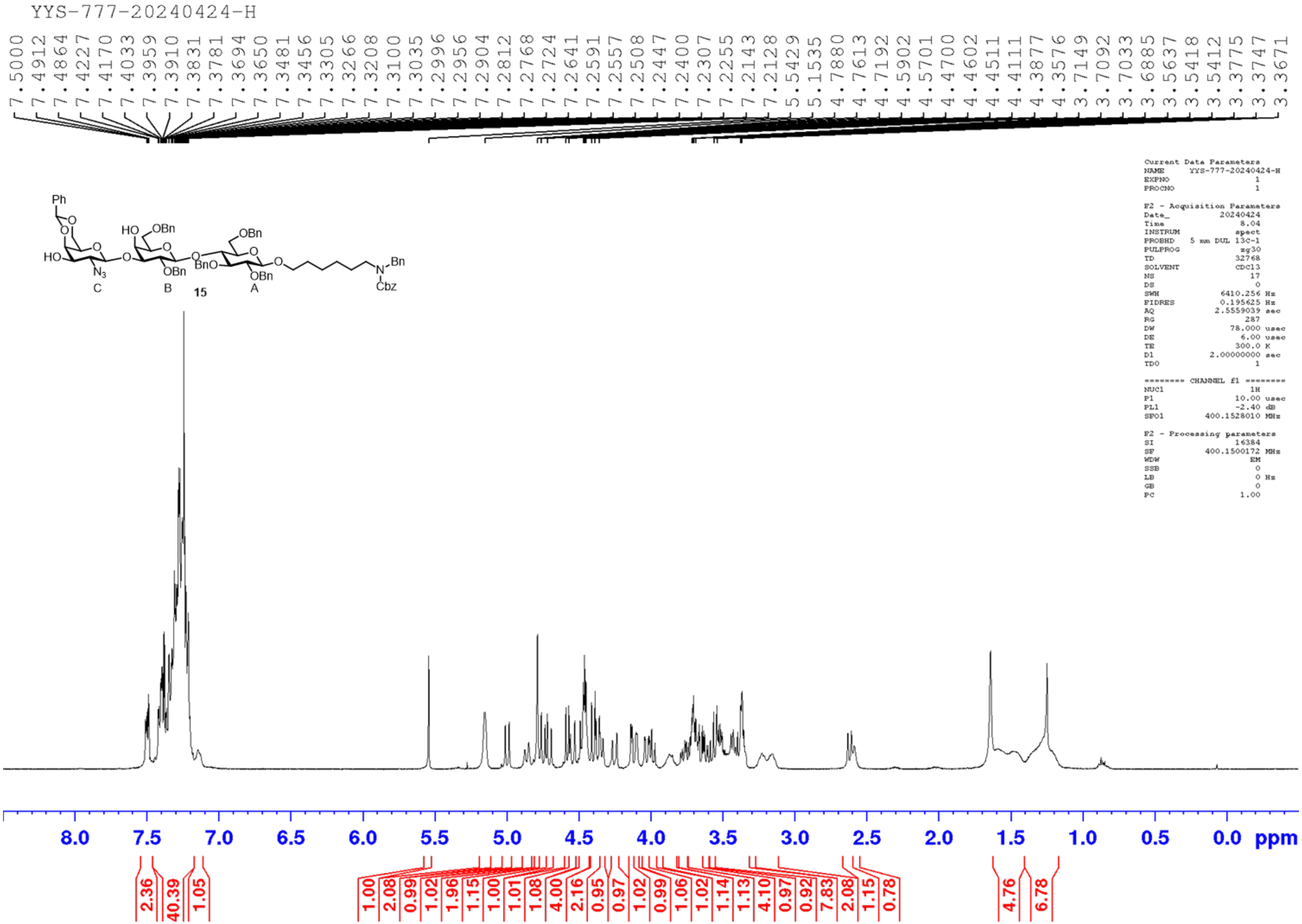
YYS-777-20240424-H
7.5000 7.4912 7.4864 7.4227 7.4170 7.4033 7.3959 7.3910 7.3831 7.3781 7.3694 7.3650 7.3481 7.3456 7.3305 7.3266 7.3208 7.3100 7.3035 7.2996 7.2956 7.2904 7.2812 7.2768 7.2724 7.2641 7.2591 7.2557 7.2508 7.2447 7.2400 7.2307 7.2255 7.2143 7.2128 5.5429 5.1535 4.7880 4.7613 4.7192 4.5902 4.5701 4.4700 4.4602 4.4511 4.4111 4.3877 4.3576 3.7149 3.7092 3.7033 3.6885 3.5637 3.5418 3.5412 3.3775 3.3747 3.3671
Ph
HO
OBn
HO
O
N3
C
B
BnO
OBn
A
15
N
Bn
Cbz
Current Data Parameters
NAME YYS-777-20240424-H
EXPNO 1
PROCNO 1
F2 - Acquisition Parameters
Date_ 20240424
Time 8.04
INSTRUM spect
PROBHD 5 mm DUL 13C-1
PULPROG zg30
TD 32768
SOLVENT CDCl3
NS 17
DS 0
SWH 6410.256 Hz
FIDRES 0.195625 Hz
AQ 2.5559039 sec
RG 287
DW 78.000 usec
DE 6.00 usec
TE 300.0 K
D1 2.00000000 sec
TD0 1
======== CHANNEL f1 ========
NUC1 1H
P1 10.00 usec
PL1 -2.40 dB
SFO1 400.1528010 MHz
F2 - Processing parameters
SI 16384
SF 400.1500172 MHz
WDW EM
SSB 0
LB 0 Hz
GB 0
PC 1.00
8.0 7.5 7.0 6.5 6.0 5.5 5.0 4.5 4.0 3.5 3.0 2.5 2.0 1.5 1.0 0.5 0.0 ppm
2.36 40.39 1.05 1.00 2.08 0.99 1.02 1.96 1.15 1.00 1.01 1.08 4.00 2.16 0.95 0.97 1.02 0.99 1.06 1.02 1.14 1.13 4.10 0.97 0.92 7.83 2.08 1.15 0.78 4.76 6.78

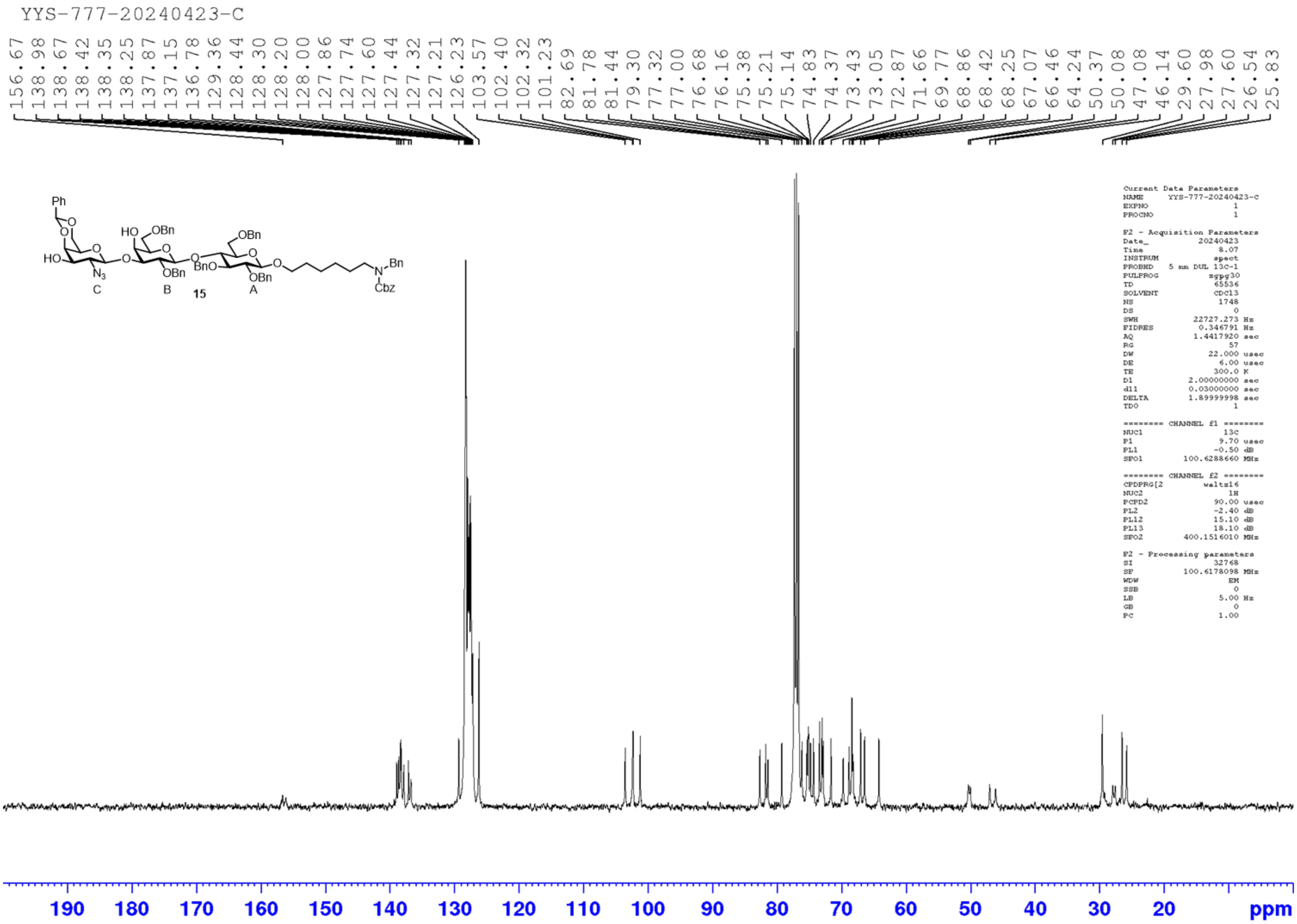
YYS-777-20240423-C
156.67
138.98
138.67
138.42
138.35
138.25
137.87
137.15
136.78
129.36
128.44
128.30
128.20
128.00
127.86
127.74
127.60
127.44
127.32
127.21
126.23
103.57
102.40
102.32
101.23
82.69
81.78
81.44
79.30
77.32
77.00
76.68
76.16
75.38
75.21
75.14
74.83
74.37
73.43
73.05
72.87
71.66
69.77
68.86
68.42
68.25
67.07
66.46
64.24
50.37
50.08
47.08
46.14
29.60
27.98
27.60
26.54
25.83
Ph
HO
OBn
N_3
BnO
Bn
Cbz
C
B
15
A
Current Data Parameters
NAME YYS-777-20240423-C
EXPNO 1
PROCNO 1
F2 - Acquisition Parameters
Date_ 20240423
Time 8.07
INSTRUM spect
PROBHD 5 mm DUL 13C-1
PULPROG zgpg30
TD 65536
SOLVENT CDCl3
NS 1748
DS 0
SWH 22727.273 Hz
FIDRES 0.346791 Hz
AQ 1.4417920 sec
RG 57
DW 22.000 usec
DE 6.00 usec
TE 300.0 K
D1 2.00000000 sec
d11 0.03000000 sec
DELTA 1.89999998 sec
TD0 1
======== CHANNEL f1 ========
NUC1 13C
P1 9.70 usec
PL1 -0.50 dB
SFO1 100.6288660 MHz
======== CHANNEL f2 ========
CPDPRG[2 waltz16
NUC2 1H
PCPD2 90.00 usec
PL2 -2.40 dB
PL12 15.10 dB
PL13 18.10 dB
SFO2 400.1516010 MHz
F2 - Processing parameters
SI 32768
SF 100.6178098 MHz
WDW EM
SSB 0
LB 5.00 Hz
GB 0
PC 1.00
190 180 170 160 150 140 130 120 110 100 90 80 70 60 50 40 30 20 ppm

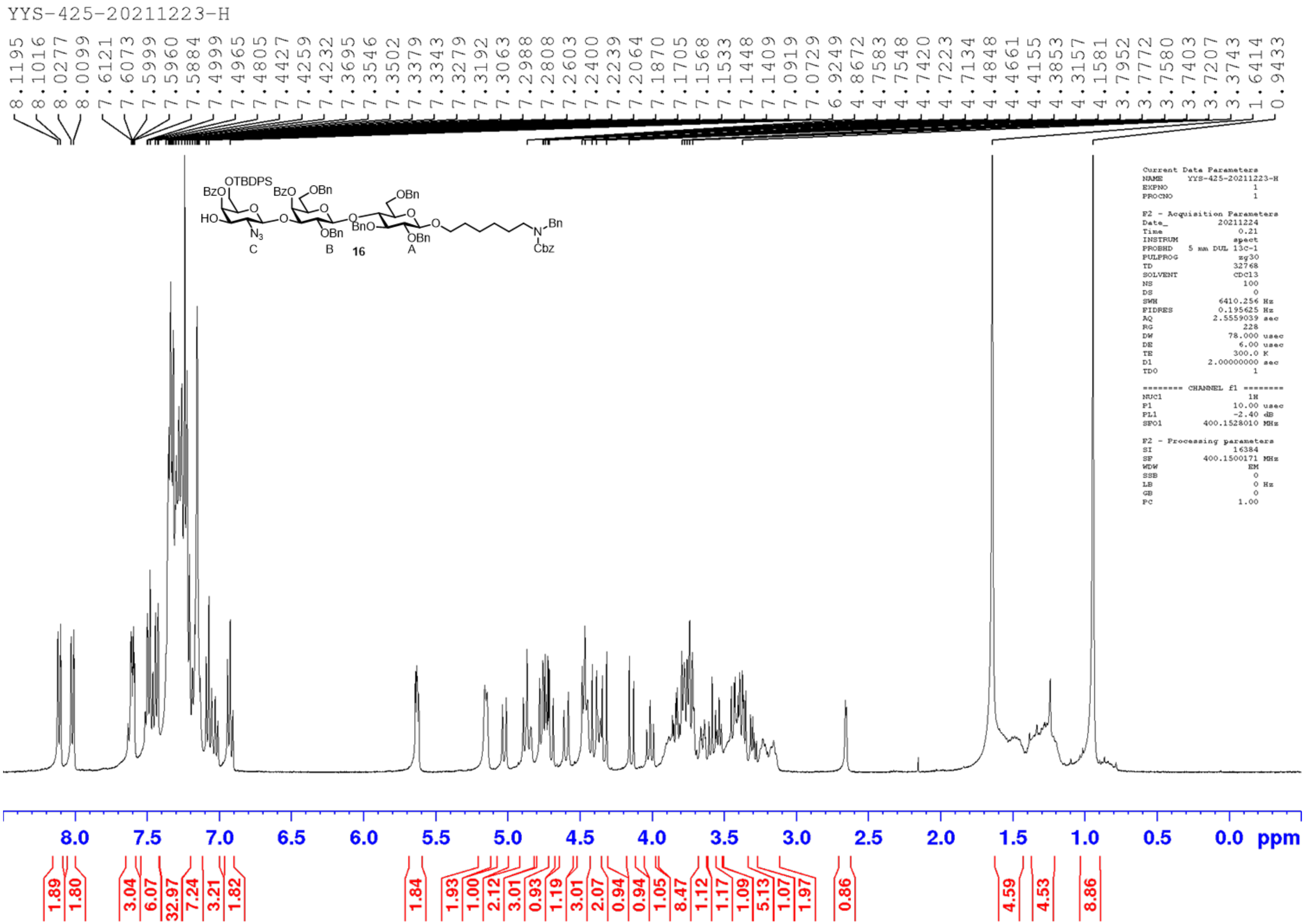
YYS-425-20211223-H
8.1195 8.1016 8.0277 8.0099 7.6121 7.6073 7.5999 7.5960 7.5884 7.4999 7.4965 7.4805 7.4427 7.4259 7.4232 7.3695 7.3546 7.3502 7.3379 7.3343 7.3279 7.3192 7.3063 7.2988 7.2808 7.2603 7.2400 7.2239 7.2064 7.1870 7.1705 7.1568 7.1533 7.1448 7.1409 7.0919 7.0729 6.9249 4.8672 4.7583 4.7548 4.7420 4.7223 4.7134 4.4848 4.4661 4.4155 4.3853 4.3157 4.1581 3.7952 3.7772 3.7580 3.7403 3.7207 3.3743 1.6414 0.9433
OTBDPS
BzO
HO
N_3
C
BzO
OBn
OBn
B
16
BnO
OBn
OBn
A
N
Bn
Cbz
Current Data Parameters
NAME YYS-425-20211223-H
EXPNO 1
PROCNO 1
F2 - Acquisition Parameters
Date_ 20211224
Time 0.21
INSTRUM spect
PROBHD 5 mm DUL 13C-1
PULPROG zg30
TD 32768
SOLVENT CDCl3
NS 100
DS 0
SWH 6410.256 Hz
FIDRES 0.195625 Hz
AQ 2.5559039 sec
RG 228
DW 78.000 usec
DE 6.00 usec
TE 300.0 K
D1 2.00000000 sec
TD0 1
======== CHANNEL f1 ========
NUC1 1H
P1 10.00 usec
PL1 -2.40 dB
SFO1 400.1528010 MHz
F2 - Processing parameters
SI 16384
SF 400.1500171 MHz
WDW EM
SSB 0
LB 0 Hz
GB 0
PC 1.00
8.0 7.5 7.0 6.5 6.0 5.5 5.0 4.5 4.0 3.5 3.0 2.5 2.0 1.5 1.0 0.5 0.0 ppm
1.89 1.80 3.04 6.07 32.97 7.24 3.21 1.82 1.84 1.93 1.00 2.12 3.01 0.93 1.19 3.01 2.07 0.94 0.94 1.05 8.47 1.12 1.17 1.09 5.13 1.07 1.97 0.86 4.59 4.53 8.86

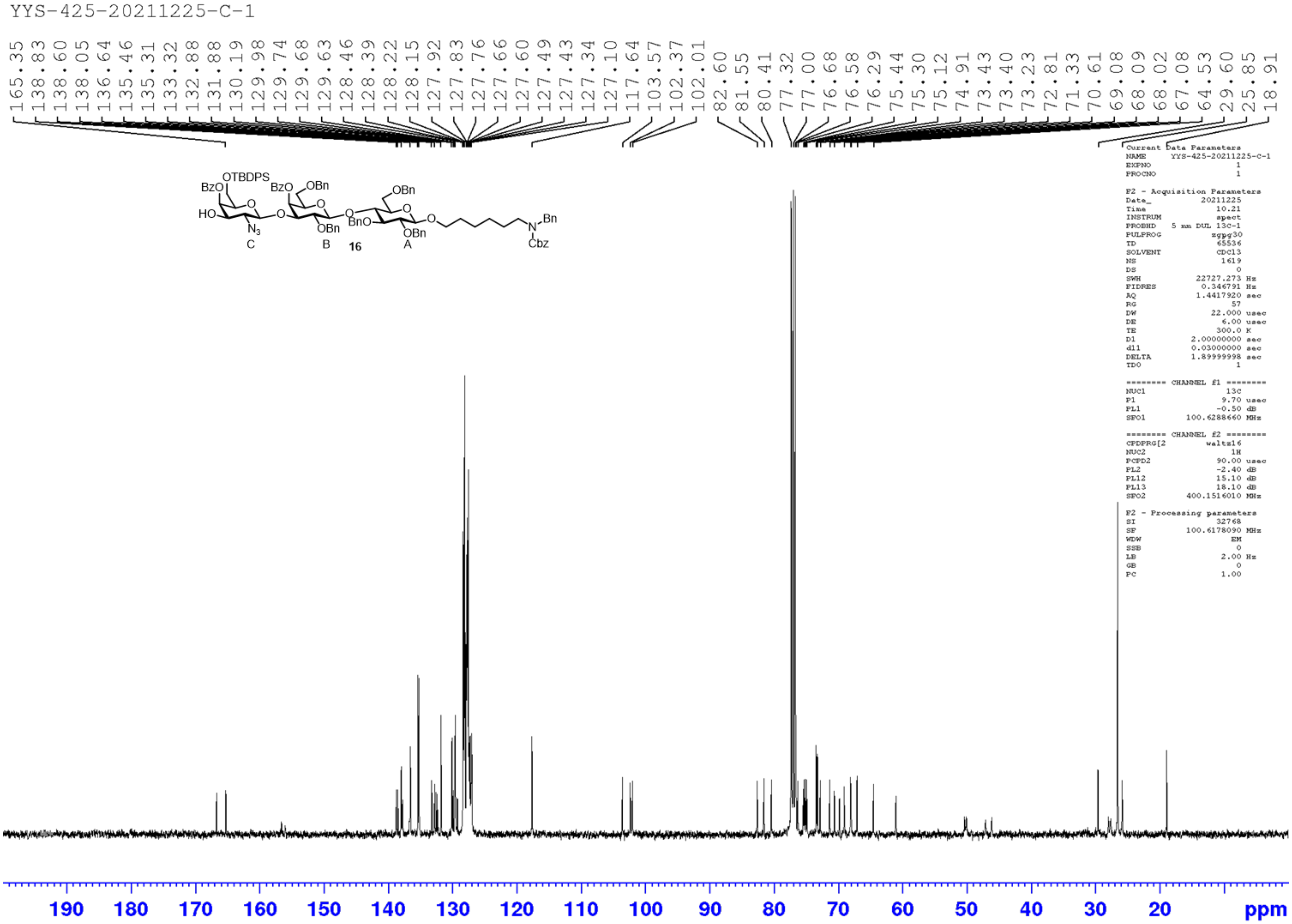
YYS-425-20211225-C-1
165.35 138.83 138.60 138.05 136.64 135.46 135.31 133.32 132.88 131.88 130.19 129.98 129.74 129.68 129.63 128.46 128.39 128.22 128.15 127.92 127.83 127.76 127.66 127.60 127.49 127.43 127.34 127.10 117.64 103.57 102.37 102.01 82.60 81.55 80.41 77.32 77.00 76.68 76.58 76.29 75.44 75.30 75.12 74.91 73.43 73.40 73.23 72.81 71.33 70.61 69.08 68.09 68.02 67.08 64.53 29.60 25.85 18.91
OTBDPS
BzO
HO
N3
C
BzO
OBn
OBn
B
16
BnO
OBn
OBn
A
O
N
Bn
Cbz
Current Data Parameters
NAME YYS-425-20211225-C-1
EXPNO 1
PROCNO 1
F2 - Acquisition Parameters
Date_ 20211225
Time 10.21
INSTRUM spect
PROBHD 5 mm DUL 13C-1
PULPROG zgpg30
TD 65536
SOLVENT CDCl3
NS 1619
DS 0
SWH 22727.273 Hz
FIDRES 0.346791 Hz
AQ 1.4417920 sec
RG 57
DW 22.000 usec
DE 6.00 usec
TE 300.0 K
D1 2.00000000 sec
d11 0.03000000 sec
DELTA 1.89999998 sec
TD0 1
======== CHANNEL f1 ========
NUC1 13C
P1 9.70 usec
PL1 -0.50 dB
SFO1 100.6288660 MHz
======== CHANNEL f2 ========
CPDPRG[2 waltz16
NUC2 1H
PCPD2 90.00 usec
PL2 -2.40 dB
PL12 15.10 dB
PL13 18.10 dB
SFO2 400.1516010 MHz
F2 - Processing parameters
SI 32768
SF 100.6178090 MHz
WDW EM
SSB 0
LB 2.00 Hz
GB 0
PC 1.00
190 180 170 160 150 140 130 120 110 100 90 80 70 60 50 40 30 20 ppm

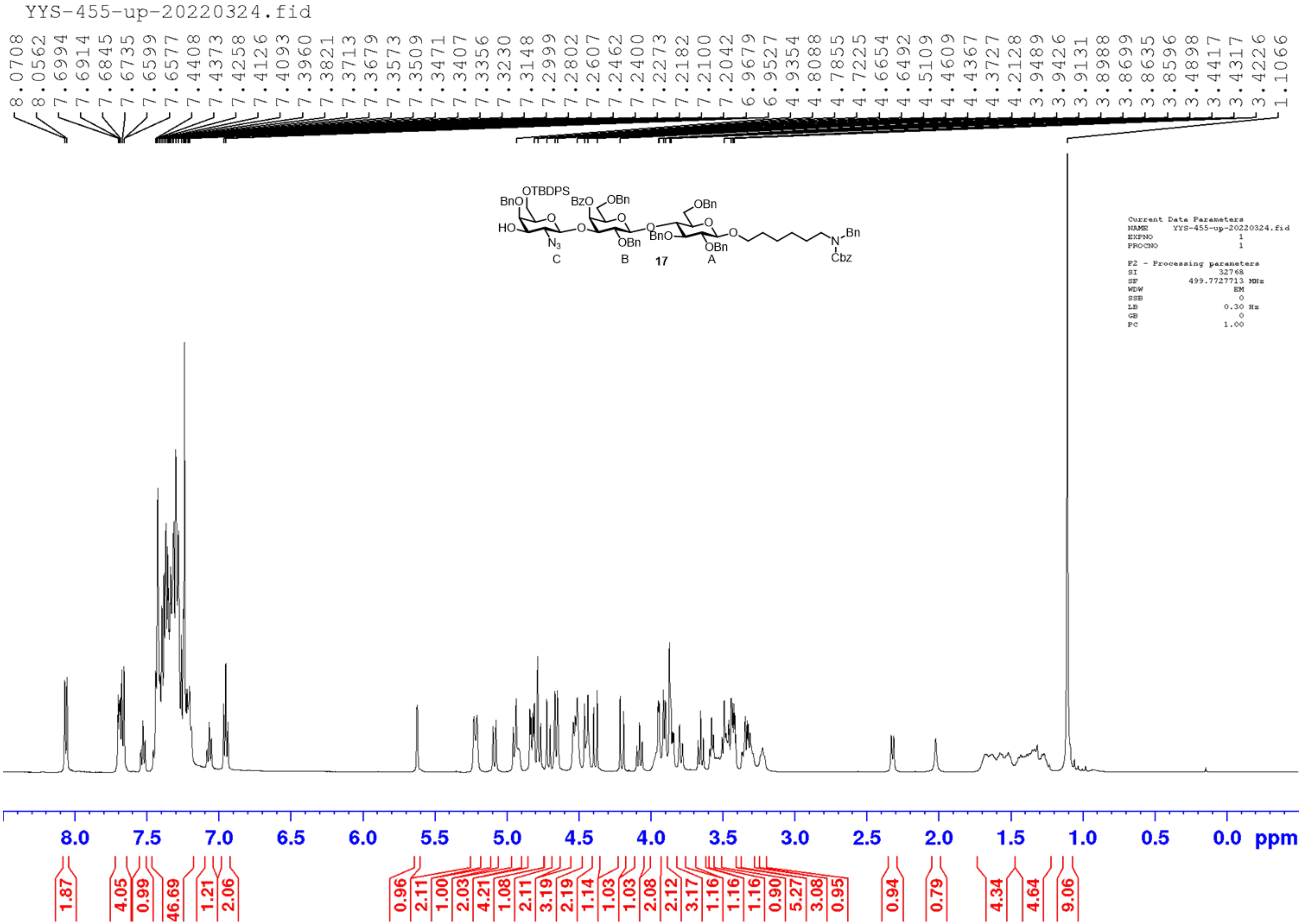
YYS-455-up-20220324.fid
8.0708
8.0562
7.6994
7.6914
7.6845
7.6735
7.6599
7.6577
7.4408
7.4373
7.4258
7.4126
7.4093
7.3960
7.3821
7.3713
7.3679
7.3573
7.3509
7.3471
7.3407
7.3356
7.3230
7.3148
7.2999
7.2802
7.2607
7.2462
7.2400
7.2273
7.2182
7.2100
7.2042
6.9679
6.9527
4.9354
4.8088
4.7855
4.7225
4.6654
4.6492
4.5109
4.4609
4.4367
4.3727
4.2128
3.9489
3.9426
3.9131
3.8988
3.8699
3.8635
3.8596
3.4898
3.4417
3.4317
3.4226
1.1066
OTBDPS
BnO
HO
N_3
C
BzO
OBn
B
17
BnO
OBn
A
N
Bn
Cbz
Current Data Parameters
NAME YYS-455-up-20220324.fid
EXPNO 1
PROCNO 1
F2 - Processing parameters
SI 32768
SF 499.7727713 MHz
WDW EM
SSB 0
LB 0.30 Hz
GB 0
PC 1.00
8.0
7.5
7.0
6.5
6.0
5.5
5.0
4.5
4.0
3.5
3.0
2.5
2.0
1.5
1.0
0.5
0.0
ppm
1.87
4.05
0.99
46.69
1.21
2.06
0.96
2.11
1.00
2.03
4.21
1.08
2.11
3.19
2.19
1.14
1.03
1.03
2.08
2.12
3.17
1.16
1.16
1.16
0.90
5.27
3.08
0.95
0.94
0.79
4.34
4.64
9.06

YYS-455-up-13C

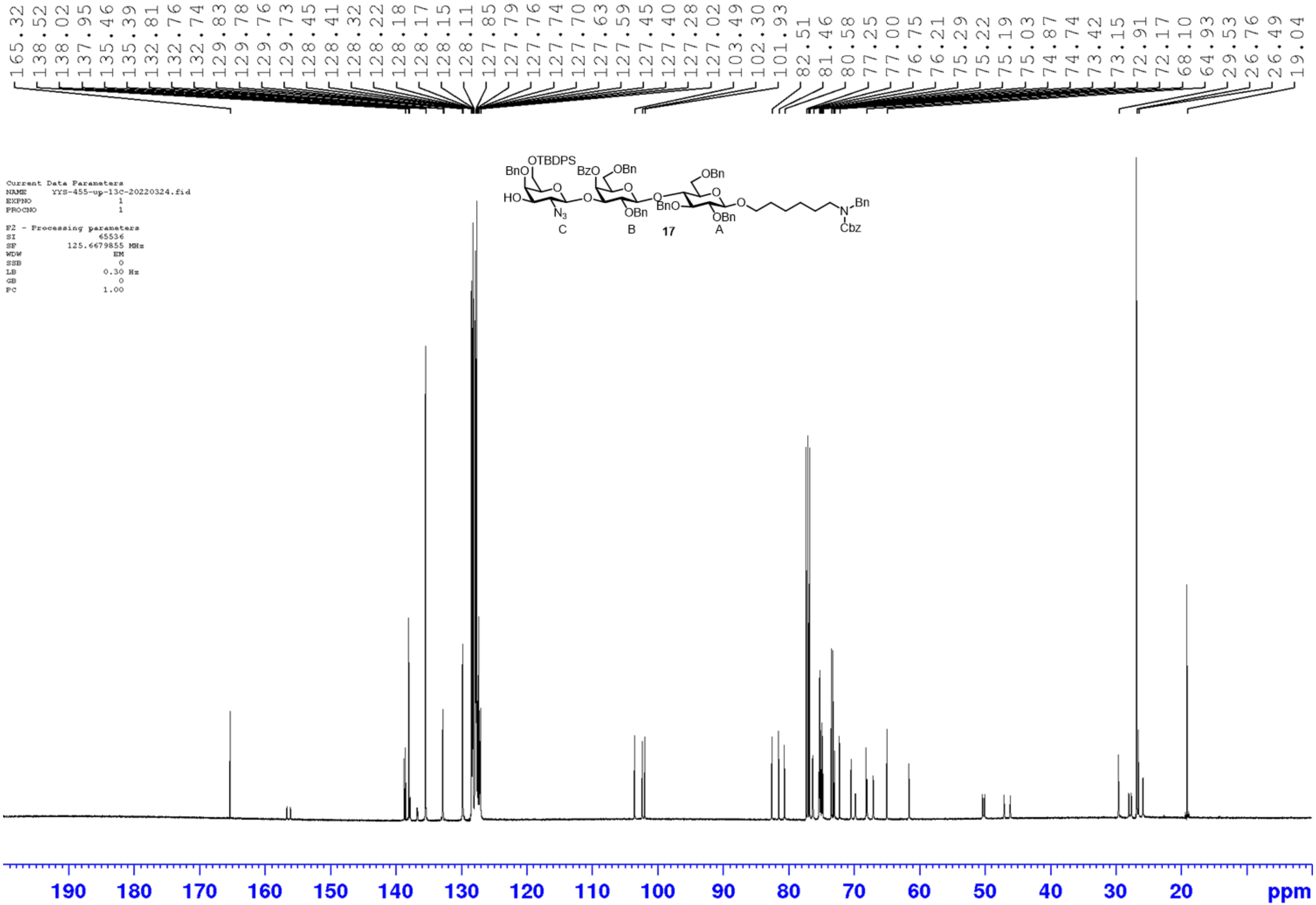

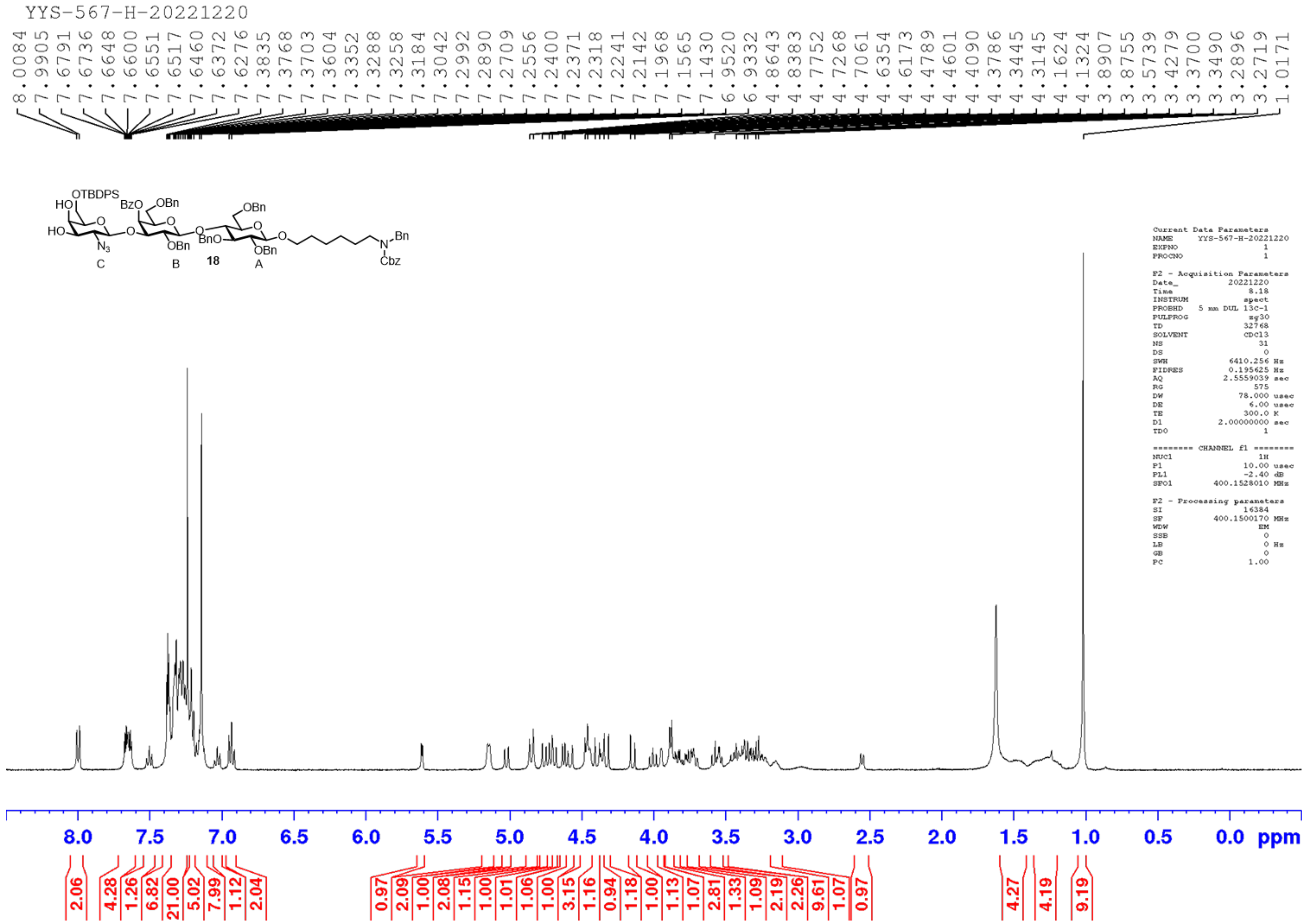
YYS-567-H-20221220
8.0084
7.9905
7.6791
7.6736
7.6648
7.6600
7.6551
7.6517
7.6460
7.6372
7.6276
7.3835
7.3768
7.3703
7.3604
7.3352
7.3288
7.3258
7.3184
7.3042
7.2992
7.2890
7.2709
7.2556
7.2400
7.2371
7.2318
7.2241
7.2142
7.1968
7.1565
7.1430
6.9520
6.9332
4.8643
4.8383
4.7752
4.7268
4.7061
4.6354
4.6173
4.4789
4.4601
4.4090
4.3786
4.3445
4.3145
4.1624
4.1324
3.8907
3.8755
3.5739
3.4279
3.3700
3.3490
3.2896
3.2719
1.0171
OTBDPS
HO
HO
N3
BzO
OBn
OBn
BnO
OBn
OBn
N
Bn
Cbz
C
B
18
A
Current Data Parameters
NAME YYS-567-H-20221220
EXPNO 1
PROCNO 1
F2 - Acquisition Parameters
Date_ 20221220
Time 8.18
INSTRUM spect
PROBHD 5 mm DUL 13C-1
PULPROG zg30
TD 32768
SOLVENT CDCl3
NS 31
DS 0
SWH 6410.256 Hz
FIDRES 0.195625 Hz
AQ 2.5559039 sec
RG 575
DW 78.000 usec
DE 6.00 usec
TE 300.0 K
D1 2.00000000 sec
TD0 1
======== CHANNEL f1 ========
NUC1 1H
P1 10.00 usec
PL1 -2.40 dB
SFO1 400.1528010 MHz
F2 - Processing parameters
SI 16384
SF 400.1500170 MHz
WDW EM
SSB 0
LB 0 Hz
GB 0
PC 1.00
8.0
7.5
7.0
6.5
6.0
5.5
5.0
4.5
4.0
3.5
3.0
2.5
2.0
1.5
1.0
0.5
0.0
ppm
2.06
4.28
1.26
6.82
21.00
5.02
7.99
1.12
2.04
0.97
2.09
1.00
2.08
1.15
1.00
1.01
1.06
1.00
3.15
1.16
0.94
1.18
1.00
1.13
1.07
2.81
1.33
1.09
2.19
2.26
9.61
1.07
0.97
4.27
4.19
9.19

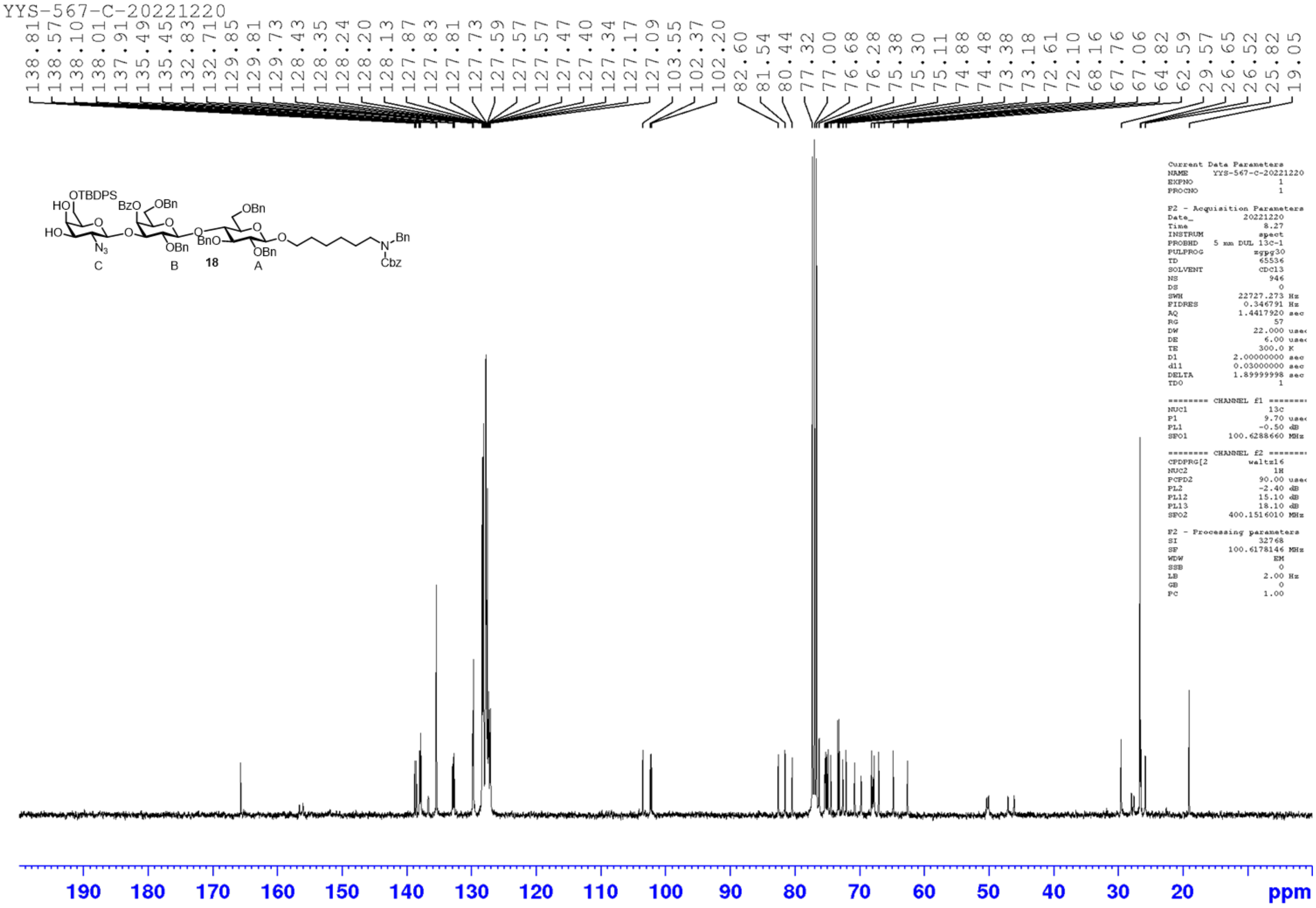
YYS-567-C-20221220
138.81 138.57 138.10 138.01 137.91 135.49 135.45 132.83 132.71 129.85 129.81 129.73 128.43 128.35 128.24 128.20 128.13 127.87 127.83 127.81 127.73 127.59 127.57 127.57 127.47 127.40 127.34 127.17 127.09 103.55 102.37 102.20 82.60 81.54 80.44 77.32 77.00 76.68 76.28 75.38 75.30 75.11 74.88 74.48 73.38 73.18 72.61 72.10 68.16 67.76 67.06 64.82 62.59 29.57 26.65 26.52 25.82 19.05
OTBDPS
HO
HO
N3
C
BzO
OBn
OBn
B
18
BnO
OBn
OBn
A
N
Bn
Cbz
Current Data Parameters
NAME YYS-567-C-20221220
EXPNO 1
PROCNO 1
F2 - Acquisition Parameters
Date_ 20221220
Time 8.27
INSTRUM spect
PROBHD 5 mm DUL 13C-1
PULPROG zgpg30
TD 65536
SOLVENT CDCl3
NS 946
DS 0
SWH 22727.273 Hz
FIDRES 0.346791 Hz
AQ 1.4417920 sec
RG 57
DW 22.000 usec
DE 6.00 usec
TE 300.0 K
D1 2.00000000 sec
d11 0.03000000 sec
DELTA 1.89999998 sec
TD0 1
======== CHANNEL f1 ========
NUC1 13C
P1 9.70 usec
PL1 -0.50 dB
SFO1 100.6288660 MHz
======== CHANNEL f2 ========
CPDPRG[2 waltz16
NUC2 1H
PCPD2 90.00 usec
PL2 -2.40 dB
PL12 15.10 dB
PL13 18.10 dB
SFO2 400.1516010 MHz
F2 - Processing parameters
SI 32768
SF 100.6178146 MHz
WDW EM
SSB 0
LB 2.00 Hz
GB 0
PC 1.00
190 180 170 160 150 140 130 120 110 100 90 80 70 60 50 40 30 20 ppm

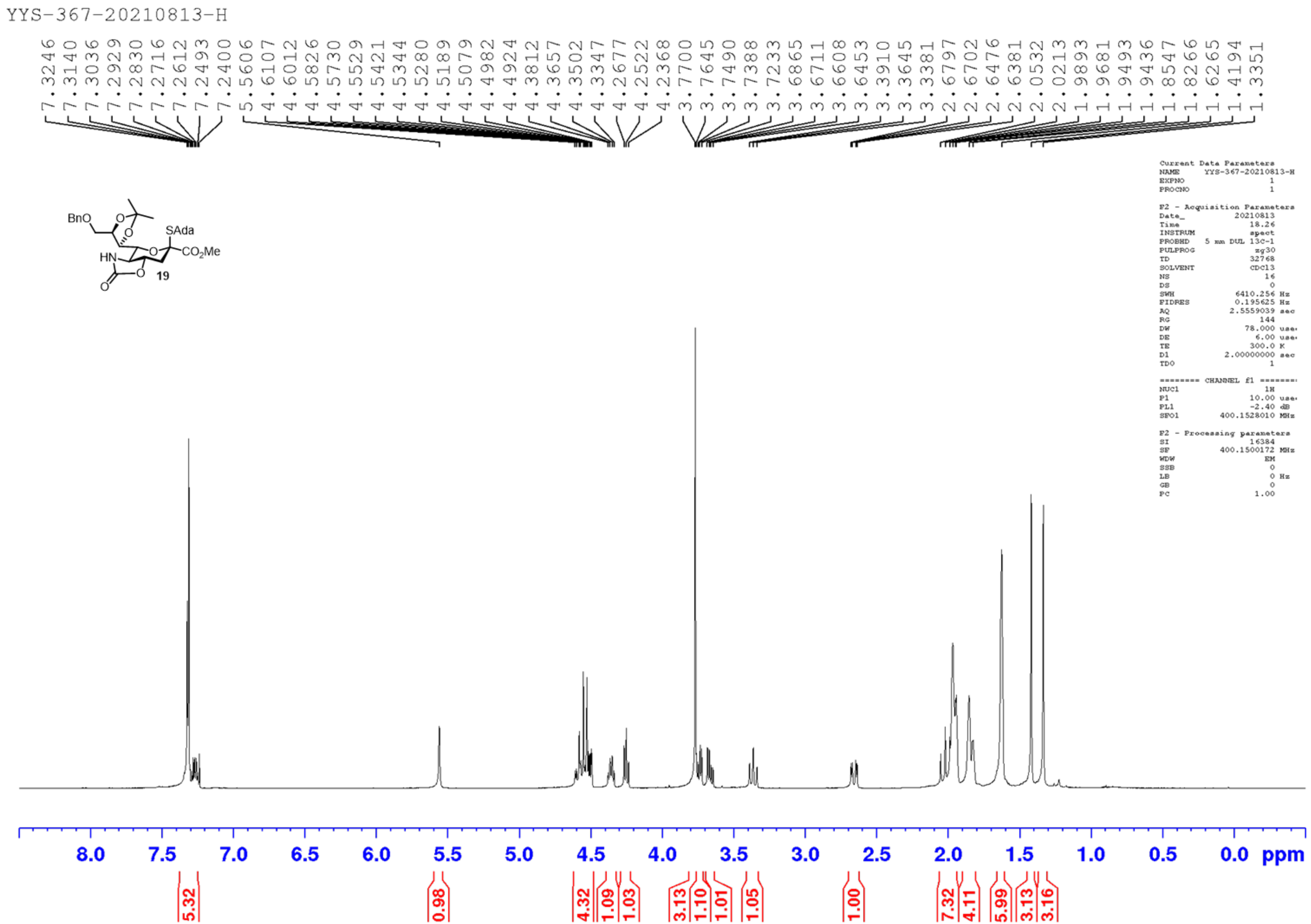

YYS-367-20210813-H
7.3246
7.3140
7.3036
7.2929
7.2830
7.2716
7.2612
7.2493
7.2400
5.5606
4.6107
4.6012
4.5826
4.5730
4.5529
4.5421
4.5344
4.5280
4.5189
4.5079
4.4982
4.4924
4.3812
4.3657
4.3502
4.3347
4.2677
4.2522
4.2368
3.7700
3.7645
3.7490
3.7388
3.7233
3.6865
3.6711
3.6608
3.6453
3.3910
3.3645
3.3381
2.6797
2.6702
2.6476
2.6381
2.0532
2.0213
1.9893
1.9681
1.9493
1.9436
1.8547
1.8266
1.6265
1.4194
1.3351
BnO
SAda
HN
CO2Me
19
Current Data Parameters
NAME YYS-367-20210813-H
EXPNO 1
PROCNO 1
F2 - Acquisition Parameters
Date_ 20210813
Time 18.26
INSTRUM spect
PROBHD 5 mm DUL 13C-1
PULPROG zg30
TD 32768
SOLVENT CDCl3
NS 16
DS 0
SWH 6410.256 Hz
FIDRES 0.195625 Hz
AQ 2.5559039 sec
RG 144
DW 78.000 usec
DE 6.00 usec
TE 300.0 K
D1 2.00000000 sec
TD0 1
======== CHANNEL f1 ========
NUC1 1H
P1 10.00 usec
PL1 -2.40 dB
SFO1 400.1528010 MHz
F2 - Processing parameters
SI 16384
SF 400.1500172 MHz
WDW EM
SSB 0
LB 0 Hz
GB 0
PC 1.00
8.0
7.5
7.0
6.5
6.0
5.5
5.0
4.5
4.0
3.5
3.0
2.5
2.0
1.5
1.0
0.5
0.0 ppm
5.32
0.98
4.32
1.09
1.03
3.13
1.10
1.01
1.05
1.00
7.32
4.11
5.99
3.13
3.16

YYS-367-20210813-C

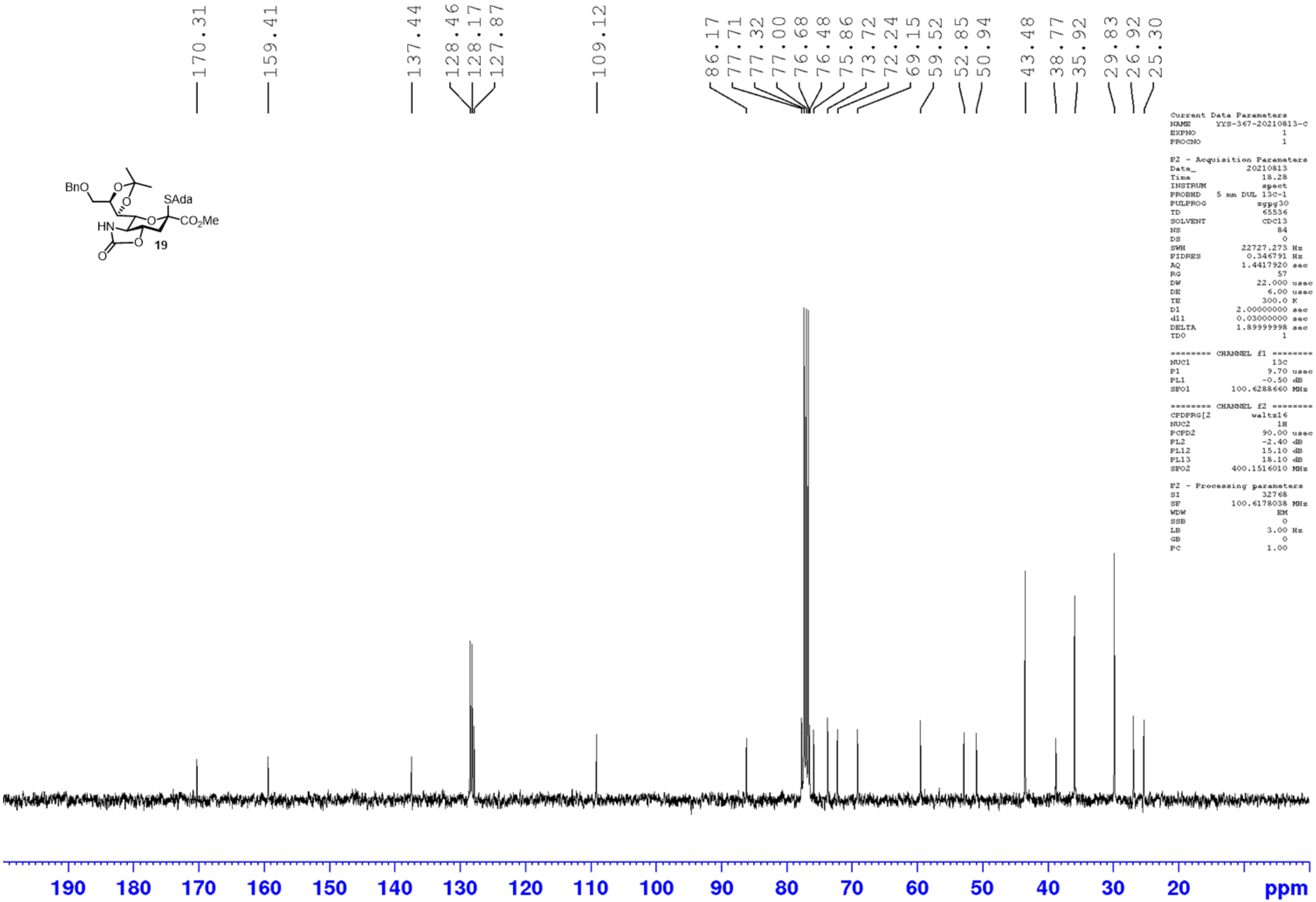

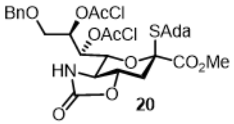
BnO
OAcCl
OAcCl
SAda
O
CO2Me
HN
O
O
20

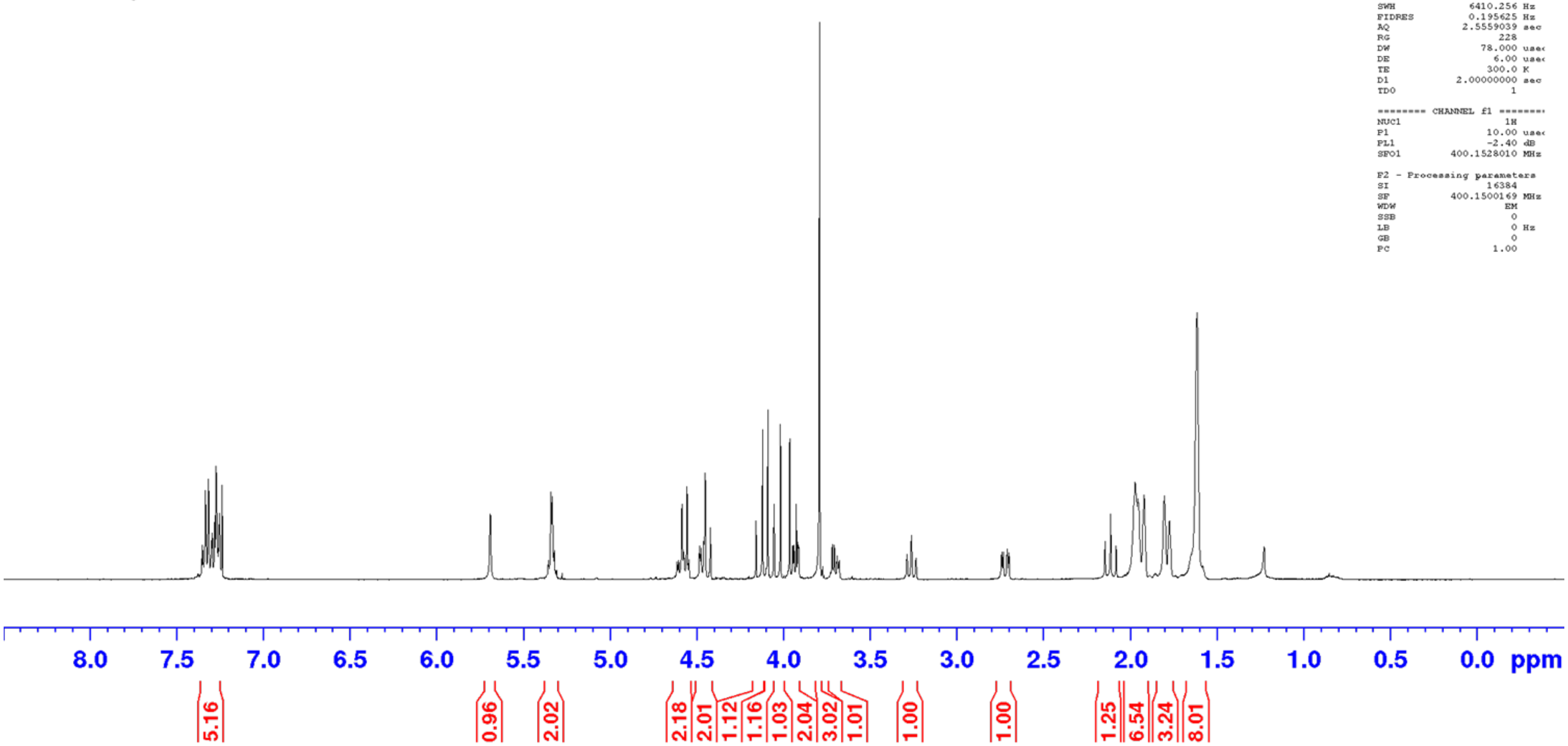
YYS-418-20211214-H
7.3577 7.3533 7.3413 7.3371 7.3329 7.3217 7.3187 7.3040 7.2998 7.2959 7.2826 7.2752 7.2714 7.2644 7.2590 7.2553 7.2400 5.6891 5.3400 5.3322 5.3205 4.5892 4.5795 4.5591 4.4617 4.4543 4.4242 4.1609 4.1232 4.0925 4.0574 4.0548 4.0199 3.9667 3.9487 3.9422 3.9292 3.9207 3.9142 3.7949 3.7220 3.7092 3.6940 3.2916 3.2645 2.7441 2.7350 2.7123 2.7032 2.1477 2.1159 2.0841 1.9747 1.9594 1.9229 1.8055 1.7763 1.6174
Current Data Parameters
NAME YYS-418-20211214-H
EXPNO 1
PROCNO 1
F2 - Acquisition Parameters
Date_ 20211214
Time 17.51
INSTRUM spect
PROBHD 5 mm DUL 13C-1
PULPROG zg30
TD 32768
SOLVENT CDCl3
NS 12
DS 0
SWH 6410.256 Hz
FIDRES 0.195625 Hz
AQ 2.5559039 sec
RG 228
DW 78.000 usec
DE 6.00 usec
TE 300.0 K
D1 2.00000000 sec
TD0 1
======== CHANNEL f1 ========
NUC1 1H
P1 10.00 usec
PL1 -2.40 dB
SFO1 400.1528010 MHz
F2 - Processing parameters
SI 16384
SF 400.1500169 MHz
WDW EM
SSB 0
LB 0 Hz
GB 0
PC 1.00
8.0 7.5 7.0 6.5 6.0 5.5 5.0 4.5 4.0 3.5 3.0 2.5 2.0 1.5 1.0 0.5 0.0 ppm
5.16 0.96 2.02 2.18 2.01 1.12 1.16 1.03 2.04 3.02 1.01 1.00 1.00 1.25 6.54 3.24 8.01

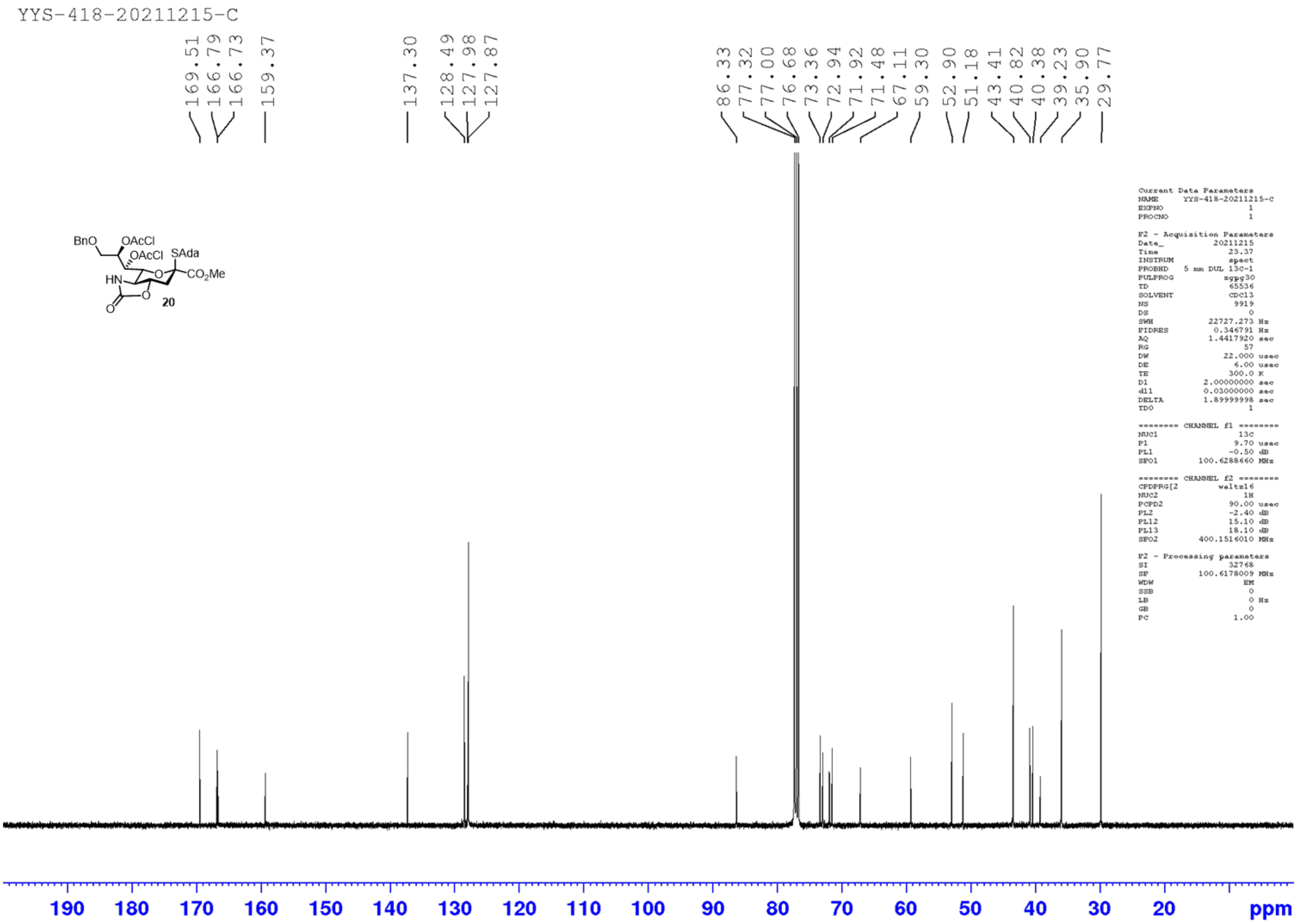
YYS-418-20211215-C
169.51
166.79
166.73
159.37
137.30
128.49
127.98
127.87
86.33
77.32
77.00
76.68
73.36
72.94
71.92
71.48
67.11
59.30
52.90
51.18
43.41
40.82
40.38
39.23
35.90
29.77
BnO
OAcCl
OAcCl
SAda
O
CO_2Me
HN
O
O
20
Current Data Parameters
NAME YYS-418-20211215-C
EXPNO 1
PROCNO 1
F2 - Acquisition Parameters
Date_ 20211215
Time 23.37
INSTRUM spect
PROBHD 5 mm DUL 13C-1
PULPROG zgpg30
TD 65536
SOLVENT CDCl3
NS 9919
DS 0
SWH 22727.273 Hz
FIDRES 0.346791 Hz
AQ 1.4417920 sec
RG 57
DW 22.000 usec
DE 6.00 usec
TE 300.0 K
D1 2.00000000 sec
d11 0.03000000 sec
DELTA 1.89999998 sec
TD0 1
======== CHANNEL f1 ========
NUC1 13C
P1 9.70 usec
PL1 -0.50 dB
SFO1 100.6288660 MHz
======== CHANNEL f2 ========
CPDPRG[2 waltz16
NUC2 1H
PCPD2 90.00 usec
PL2 -2.40 dB
PL12 15.10 dB
PL13 18.10 dB
SFO2 400.1516010 MHz
F2 - Processing parameters
SI 32768
SF 100.6178009 MHz
WDW EM
SSB 0
LB 0 Hz
GB 0
PC 1.00
190
180
170
160
150
140
130
120
110
100
90
80
70
60
50
40
30
20
ppm

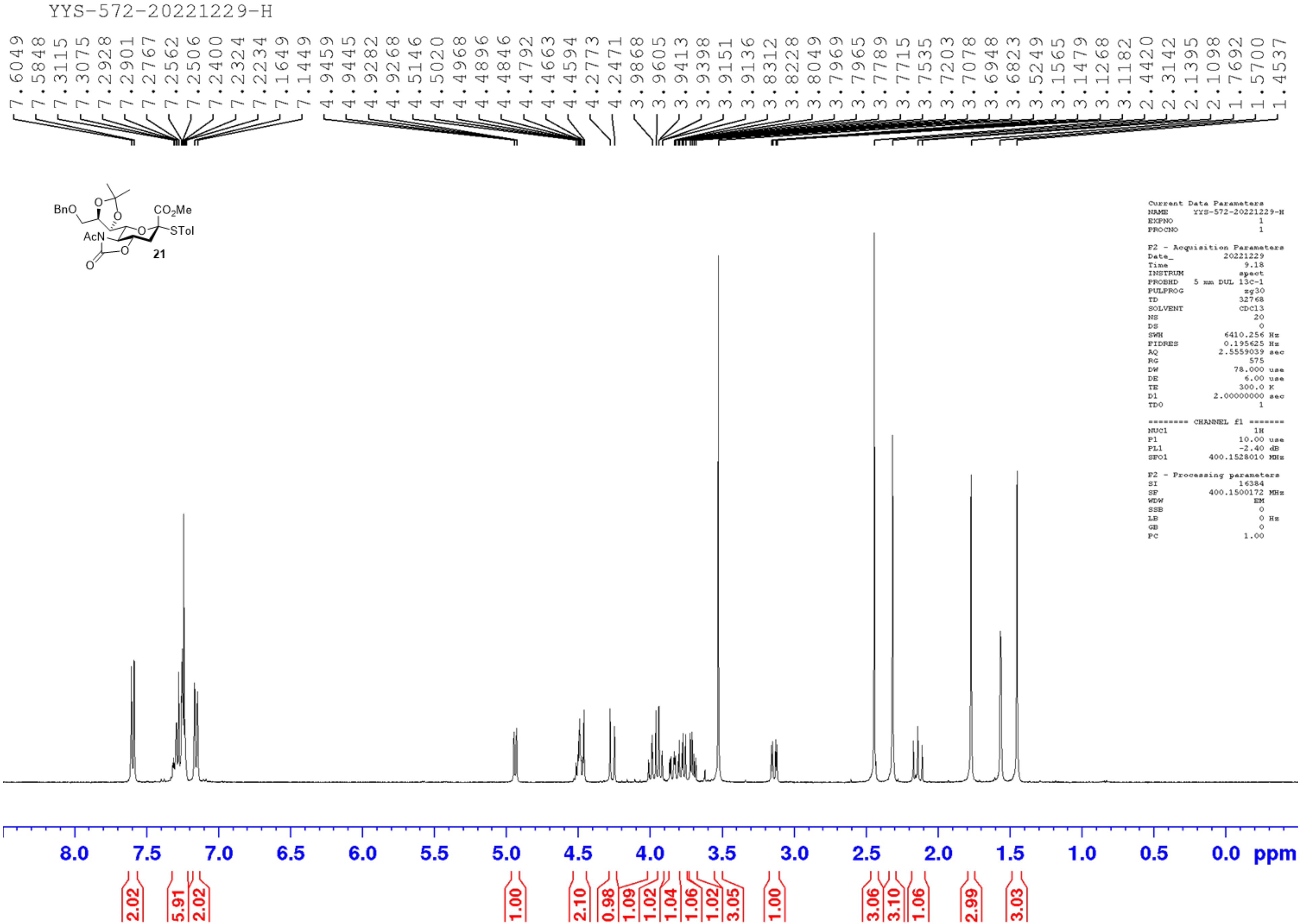

YYS-572-20221229-H
7.6049 7.5848 7.3115 7.3075 7.2928 7.2901 7.2767 7.2562 7.2506 7.2400 7.2324 7.2234 7.1649 7.1449 4.9459 4.9445 4.9282 4.9268 4.5146 4.5020 4.4968 4.4896 4.4846 4.4792 4.4663 4.4594 4.2773 4.2471 3.9868 3.9605 3.9413 3.9398 3.9151 3.9136 3.8312 3.8228 3.8049 3.7969 3.7965 3.7789 3.7715 3.7535 3.7203 3.7078 3.6948 3.6823 3.5249 3.1565 3.1479 3.1268 3.1182 2.4420 2.3142 2.1395 2.1098 1.7692 1.5700 1.4537
BnO
CO2Me
AcN
STol
O
21
Current Data Parameters
NAME YYS-572-20221229-H
EXPNO 1
PROCNO 1
F2 - Acquisition Parameters
Date_ 20221229
Time 9.18
INSTRUM spect
PROBHD 5 mm DUL 13C-1
PULPROG zg30
TD 32768
SOLVENT CDCl3
NS 20
DS 0
SWH 6410.256 Hz
FIDRES 0.195625 Hz
AQ 2.5559039 sec
RG 575
DW 78.000 use
DE 6.00 use
TE 300.0 K
D1 2.00000000 sec
TD0 1
======== CHANNEL f1 ========
NUC1 1H
P1 10.00 use
PL1 -2.40 dB
SFO1 400.1528010 MHz
F2 - Processing parameters
SI 16384
SF 400.1500172 MHz
WDW EM
SSB 0
LB 0 Hz
GB 0
PC 1.00
8.0 7.5 7.0 6.5 6.0 5.5 5.0 4.5 4.0 3.5 3.0 2.5 2.0 1.5 1.0 0.5 0.0 ppm
2.02 5.91 2.02 1.00 2.10 0.98 1.09 1.02 1.04 1.06 1.02 3.05 1.00 3.06 3.10 1.06 2.99 3.03

YYS-572-20221229-C

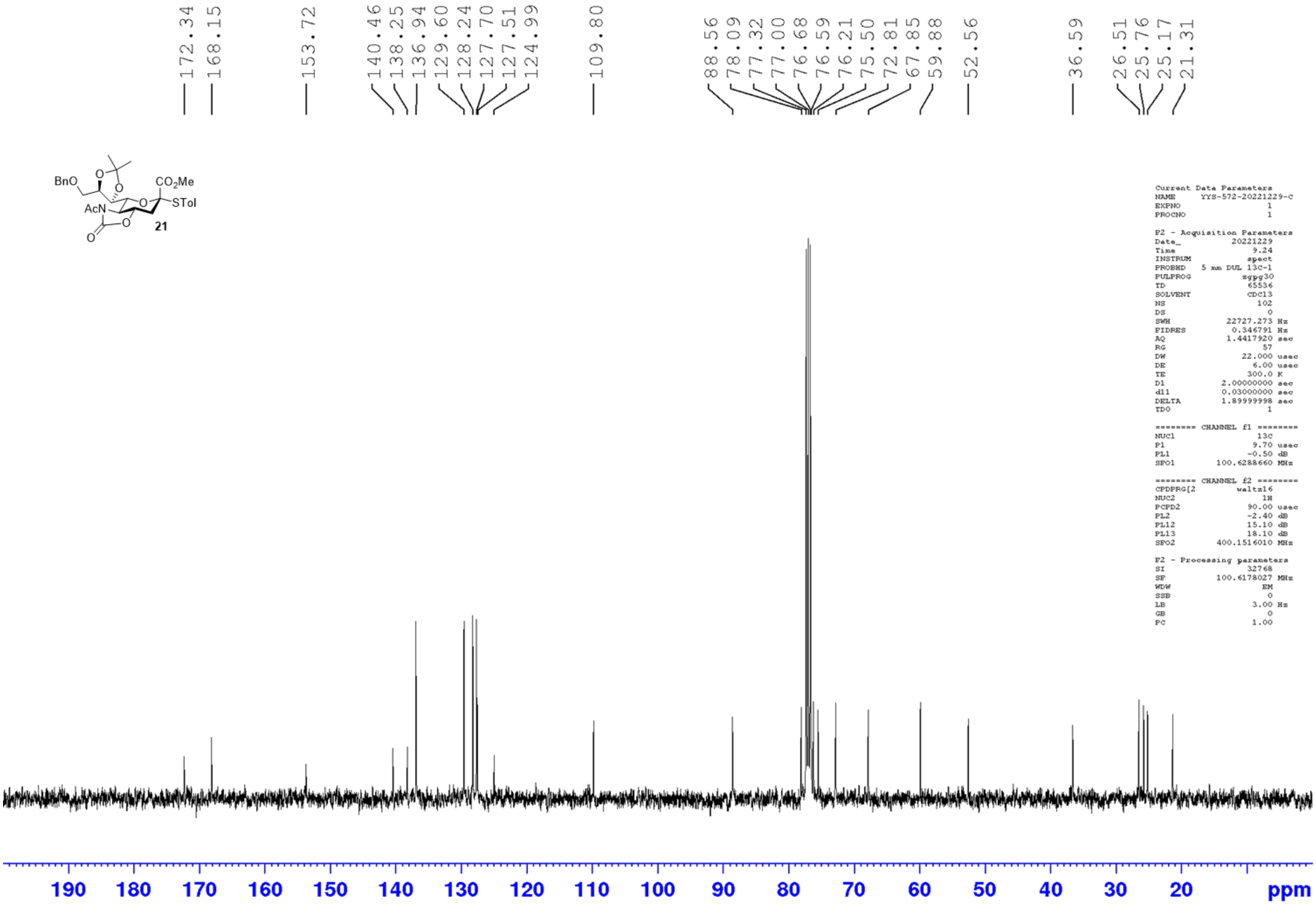

YYS_381-1_20211026

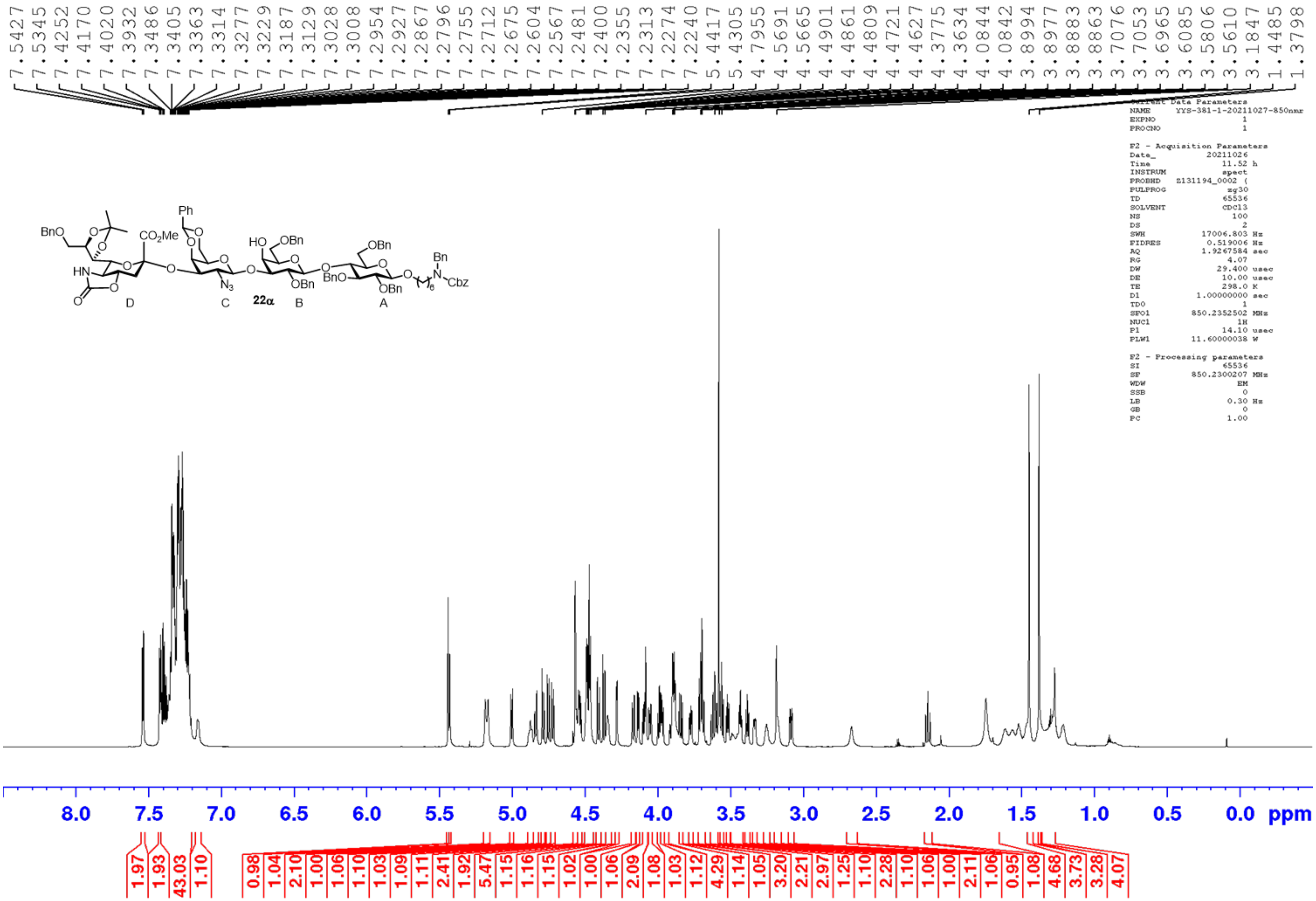

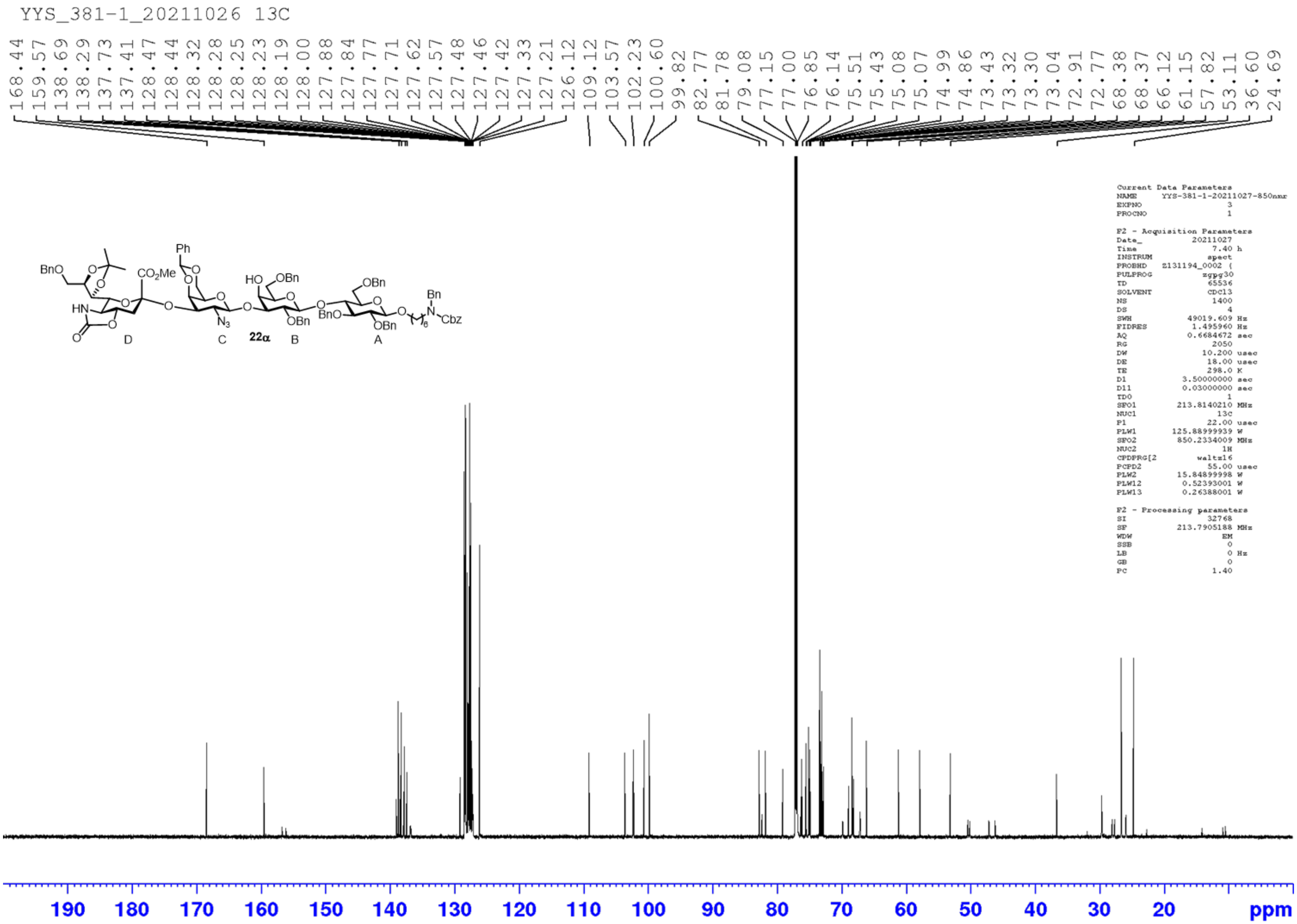

YYS_381-1_20211026 13C
168.44 159.57 138.69 138.29 137.73 137.41 128.47 128.44 128.32 128.28 128.25 128.23 128.19 128.00 127.88 127.84 127.77 127.71 127.62 127.57 127.48 127.46 127.42 127.33 127.21 126.12 109.12 103.57 102.23 100.60 99.82 82.77 81.78 79.08 77.15 77.00 76.85 76.14 75.51 75.43 75.08 75.07 74.99 74.86 73.43 73.32 73.30 73.04 72.91 72.77 68.38 68.37 66.12 61.15 57.82 53.11 36.60 24.69
BnO
CO2Me
Ph
HO
OBn
HN
N3
OBn
BnO
Bn
N
Cbz
D
C
22α
B
A
Current Data Parameters
NAME YYS-381-1-20211027-850nmr
EXPNO 3
PROCNO 1
F2 - Acquisition Parameters
Date_ 20211027
Time 7.40 h
INSTRUM spect
PROBHD Z131194_0002 (
PULPROG zgpg30
TD 65536
SOLVENT CDCl3
NS 1400
DS 4
SWH 49019.609 Hz
FIDRES 1.495960 Hz
AQ 0.6684672 sec
RG 2050
DW 10.200 usec
DE 18.00 usec
TE 298.0 K
D1 3.50000000 sec
D11 0.03000000 sec
TD0 1
SFO1 213.8140210 MHz
NUC1 13C
P1 22.00 usec
PLW1 125.88999939 W
SFO2 850.2334009 MHz
NUC2 1H
CPDPRG[2 waltz16
PCPD2 55.00 usec
PLW2 15.84899998 W
PLW12 0.52393001 W
PLW13 0.26388001 W
F2 - Processing parameters
SI 32768
SF 213.7905188 MHz
WDW EM
SSB 0
LB 0 Hz
GB 0
PC 1.40
190 180 170 160 150 140 130 120 110 100 90 80 70 60 50 40 30 20 ppm

YYS-381-1-20211006-decouple

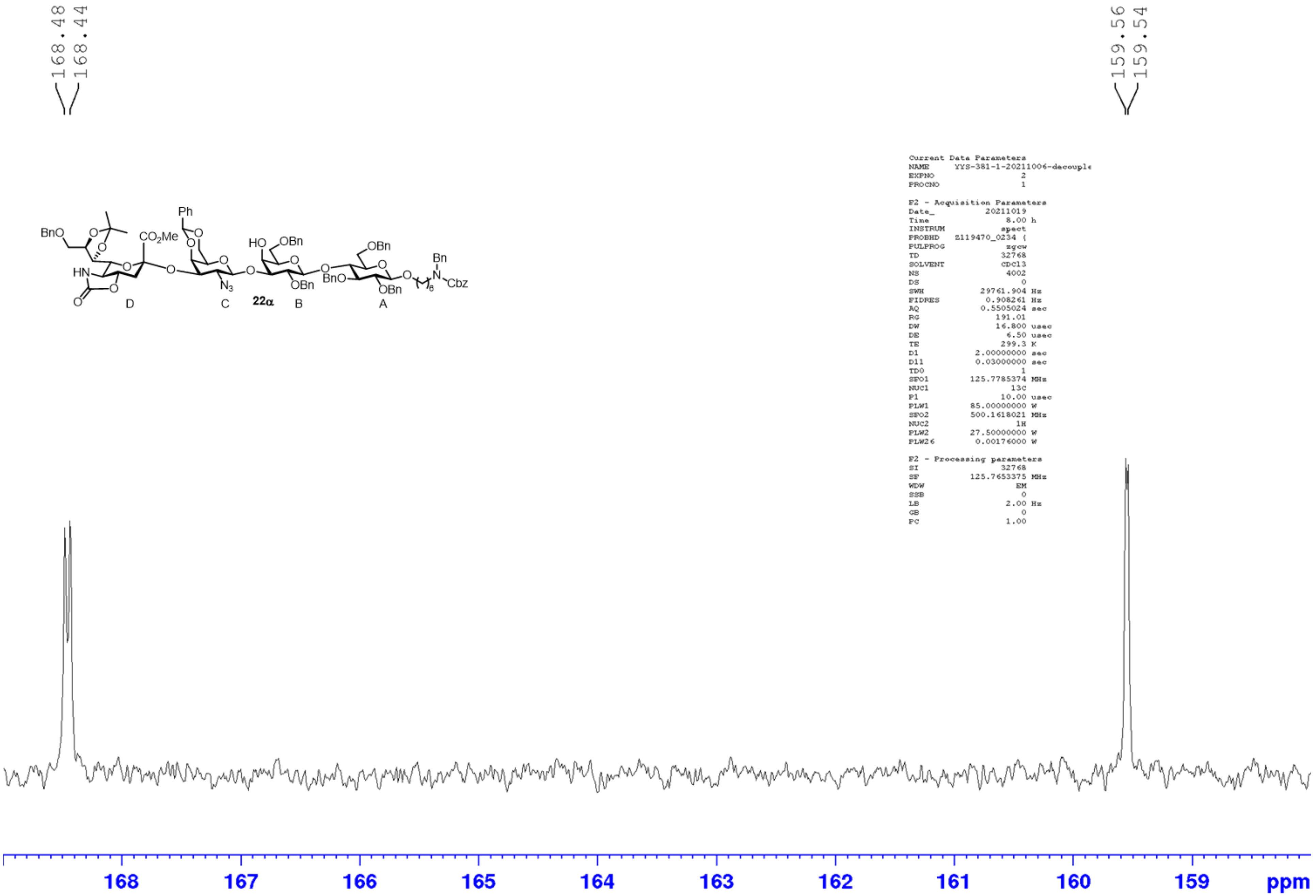

YYS_381-1_20211026 H-H COSY

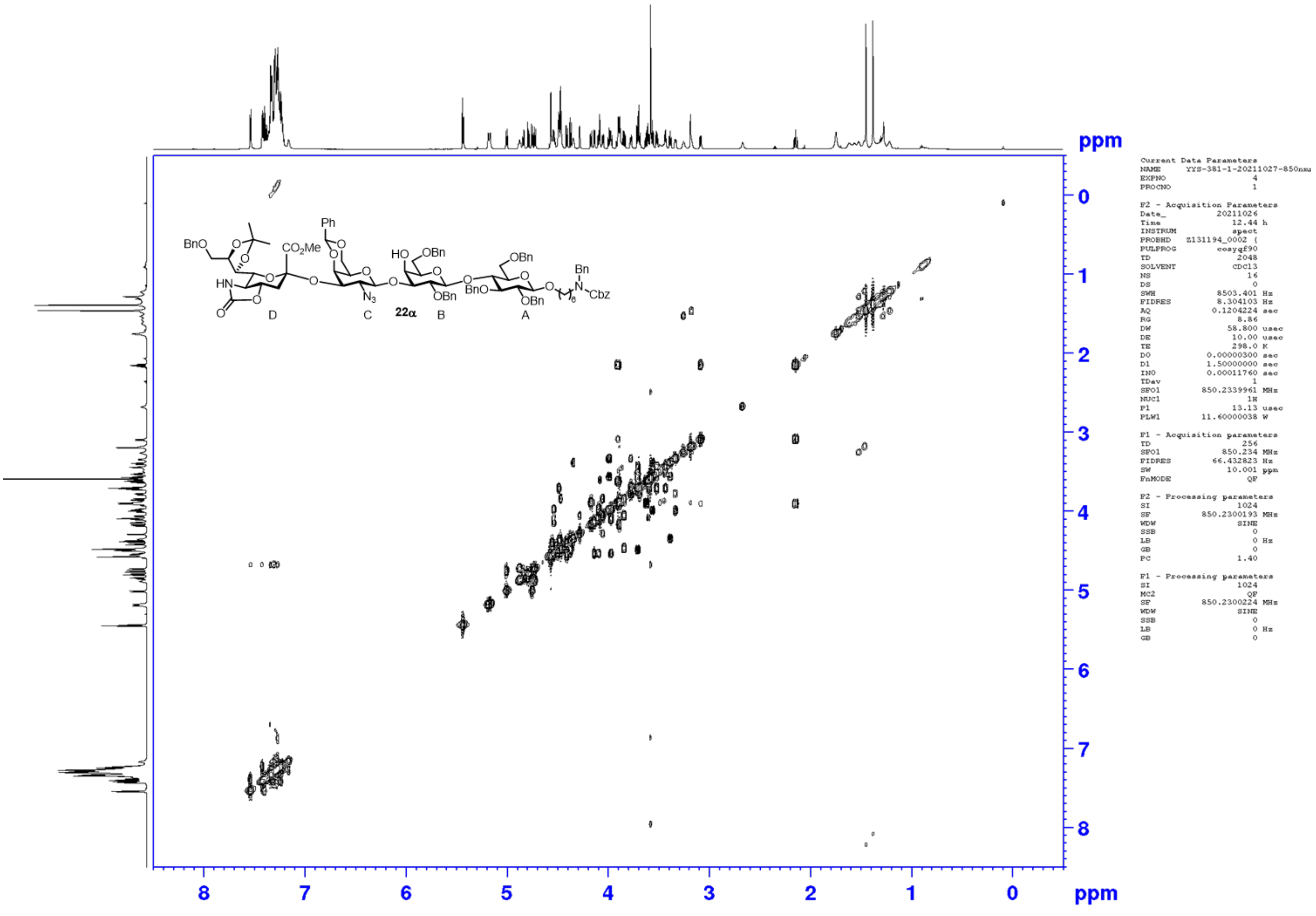

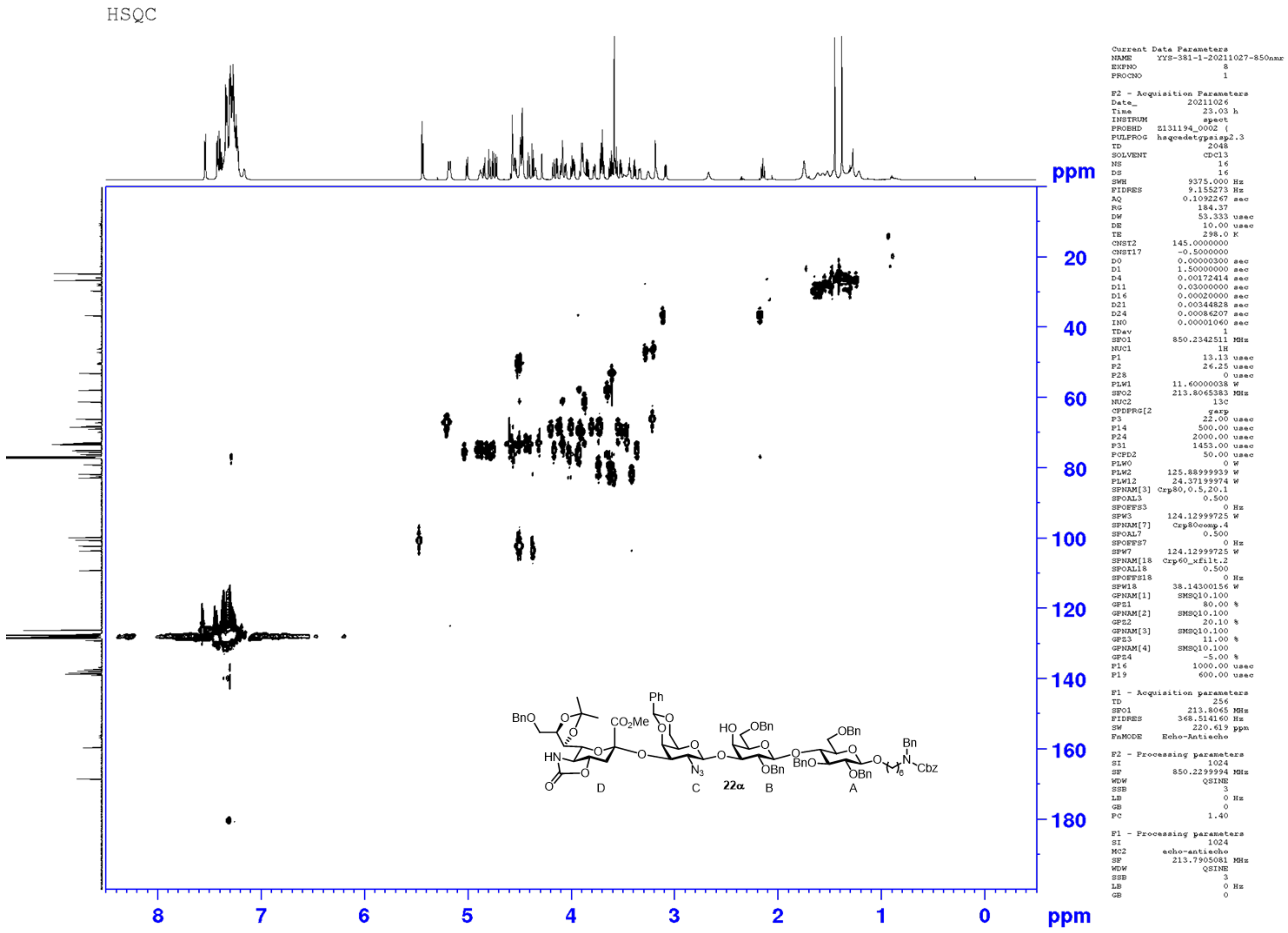
HSQC
Current Data Parameters
NAME YYS-381-1-20211027-850nmr
EXPNO 8
PROCNO 1
F2 - Acquisition Parameters
Date_ 20211026
Time 23.03 h
INSTRUM spect
PULPROG hsqcedetgpsisp2.3
TD 2048
SOLVENT CDCl3
NS 16
DS 16
SWH 9375.000 Hz
FIDRES 9.155273 Hz
AQ 0.1092267 sec
RG 184.37
DW 53.333 usec
DE 10.00 usec
TE 298.0 K
SFO1 850.2342511 MHz
NUC1 1H
SFO2 213.8065383 MHz
NUC2 13C
F1 - Acquisition parameters
TD 256
SFO1 213.8065 MHz
FIDRES 368.514160 Hz
SW 220.619 ppm
FnMODE Echo-Antiecho
F2 - Processing parameters
SI 1024
SF 850.2299994 MHz
WDW QSINE
SSB 3
LB 0 Hz
GB 0
PC 1.40
F1 - Processing parameters
SI 1024
MC2 echo-antiecho
SF 213.7905081 MHz
WDW QSINE
SSB 3
LB 0 Hz
GB 0
ppm
20
40
60
80
100
120
140
160
180
8
7
6
5
4
3
2
1
0
ppm
BnO
CO2Me
Ph
HO
OBn
OBn
Bn
HN
N3
OBn
BnO
OBn
Cbz
D
C
22α
B
A

HMBC

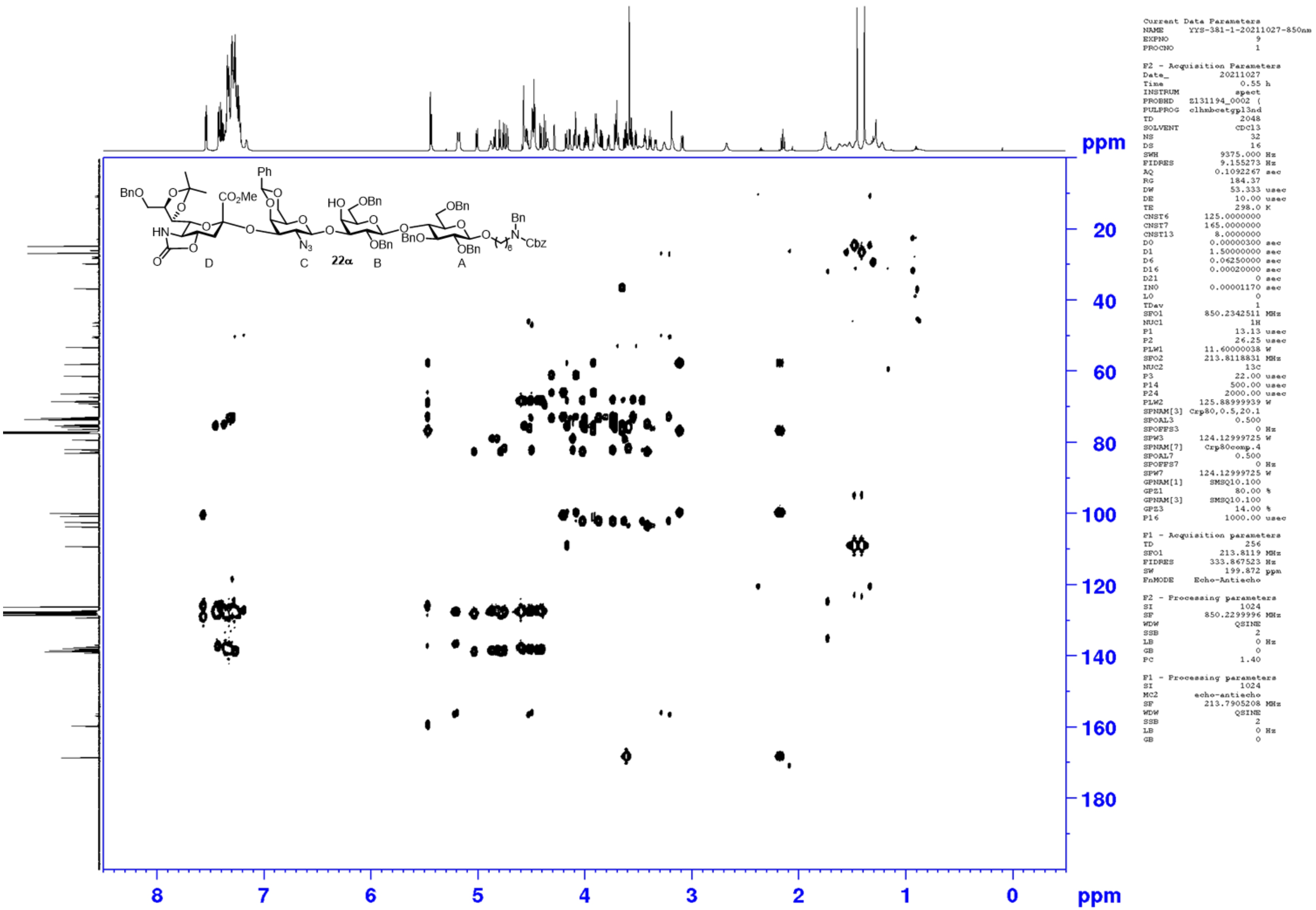
BnO
HN
D
CO2Me
Ph
N3
C
22α
HO
OBn
B
BnO
A
Bn
N
Cbz
ppm
20
40
60
80
100
120
140
160
180
8
7
6
5
4
3
2
1
0
ppm
Current Data Parameters
NAME YYS-381-1-20211027-850nm
EXPNO 9
PROCNO 1
F2 - Acquisition Parameters
Date_ 20211027
Time 0.55 h
INSTRUM spect
PROBHD Z131194_0002 (
PULPROG clhmbcetgpl3nd
TD 2048
SOLVENT CDCl3
NS 32
DS 16
SWH 9375.000 Hz
FIDRES 9.155273 Hz
AQ 0.1092267 sec
RG 184.37
DW 53.333 usec
DE 10.00 usec
TE 298.0 K
CNST6 125.0000000
CNST7 165.0000000
CNST13 8.0000000
D0 0.00000300 sec
D1 1.50000000 sec
D6 0.06250000 sec
D16 0.00020000 sec
D21 0 sec
IN0 0.00001170 sec
L0 0
TDav 1
SFO1 850.2342511 MHz
NUC1 1H
P1 13.13 usec
P2 26.25 usec
PLW1 11.60000038 W
SFO2 213.8118831 MHz
NUC2 13C
P3 22.00 usec
P14 500.00 usec
P24 2000.00 usec
PLW2 125.88999939 W
SPNAM[3] Crp80,0.5,20.1
SPOAL3 0.500
SPOFFS3 0 Hz
SPW3 124.12999725 W
SPNAM[7] Crp80comp.4
SPOAL7 0.500
SPOFFS7 0 Hz
SPW7 124.12999725 W
GPNAM[1] SMSQ10.100
GPZ1 80.00 %
GPNAM[3] SMSQ10.100
GPZ3 14.00 %
P16 1000.00 usec
F1 - Acquisition parameters
TD 256
SFO1 213.8119 MHz
FIDRES 333.867523 Hz
SW 199.872 ppm
FnMODE Echo-Antiecho
F2 - Processing parameters
SI 1024
SF 850.2299996 MHz
WDW QSINE
SSB 2
LB 0 Hz
GB 0
PC 1.40
F1 - Processing parameters
SI 1024
MC2 echo-antiecho
SF 213.7905208 MHz
WDW QSINE
SSB 2
LB 0 Hz
GB 0

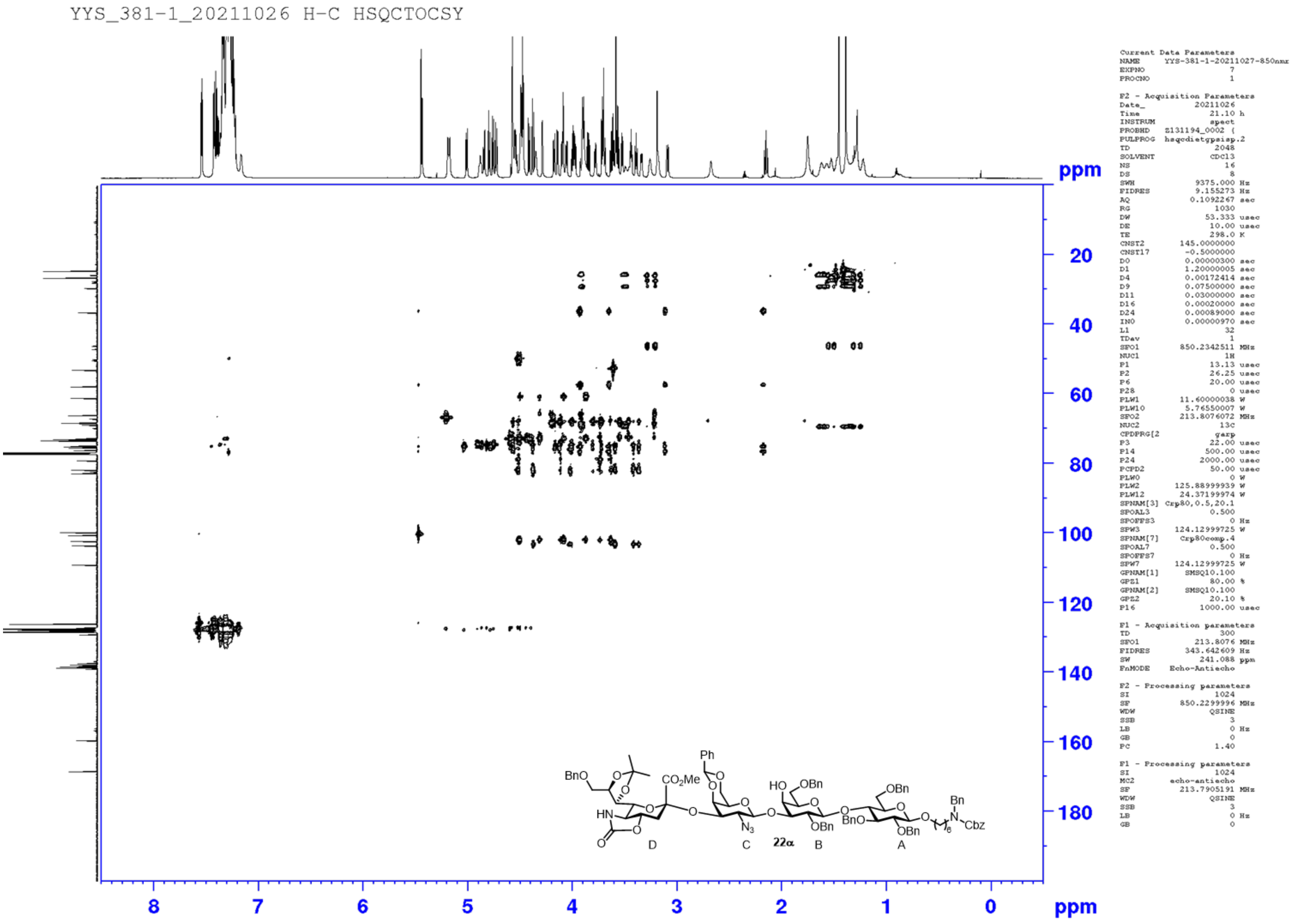
YYS_381-1_20211026 H-C HSQCTOCSY
ppm
20
40
60
80
100
120
140
160
180
8
7
6
5
4
3
2
1
0
ppm
BnO
CO2Me
Ph
HO
OBn
OBn
Bn
HN
O
D
N3
C
22α
OBn
B
BnO
OBn
A
N
Cbz
Current Data Parameters
NAME YYS-381-1-20211027-850nmr
EXPNO 7
PROCNO 1
F2 - Acquisition Parameters
Date_ 20211026
Time 21.10 h
INSTRUM spect
PROBHD Z131194_0002 (
PULPROG hsqcdietgpsisp.2
TD 2048
SOLVENT CDCl3
NS 16
DS 8
SWH 9375.000 Hz
FIDRES 9.155273 Hz
AQ 0.1092267 sec
RG 1030
DW 53.333 usec
DE 10.00 usec
TE 298.0 K
CNST2 145.0000000
CNST17 -0.5000000
D0 0.00000300 sec
D1 1.20000005 sec
D4 0.00172414 sec
D9 0.07500000 sec
D11 0.03000000 sec
D16 0.00020000 sec
D24 0.00089000 sec
IN0 0.00000970 sec
L1 32
TDav 1
SFO1 850.2342511 MHz
NUC1 1H
P1 13.13 usec
P2 26.25 usec
P6 20.00 usec
P28 0 usec
PLW1 11.60000038 W
PLW10 5.76550007 W
SFO2 213.8076072 MHz
NUC2 13C
CPDPRG[2 garp
P3 22.00 usec
P14 500.00 usec
P24 2000.00 usec
PCPD2 50.00 usec
PLW0 0 W
PLW2 125.88999939 W
PLW12 24.37199974 W
SPNAM[3] Crp80,0.5,20.1
SPOAL3 0.500
SPOFFS3 0 Hz
SPW3 124.12999725 W
SPNAM[7] Crp80comp.4
SPOAL7 0.500
SPOFFS7 0 Hz
SPW7 124.12999725 W
GPNAM[1] SMSQ10.100
GPZ1 80.00 %
GPNAM[2] SMSQ10.100
GPZ2 20.10 %
P16 1000.00 usec
F1 - Acquisition parameters
TD 300
SFO1 213.8076 MHz
FIDRES 343.642609 Hz
SW 241.088 ppm
FnMODE Echo-Antiecho
F2 - Processing parameters
SI 1024
SF 850.2299996 MHz
WDW QSINE
SSB 3
LB 0 Hz
GB 0
PC 1.40
F1 - Processing parameters
SI 1024
MC2 echo-antiecho
SF 213.7905191 MHz
WDW QSINE
SSB 3
LB 0 Hz
GB 0

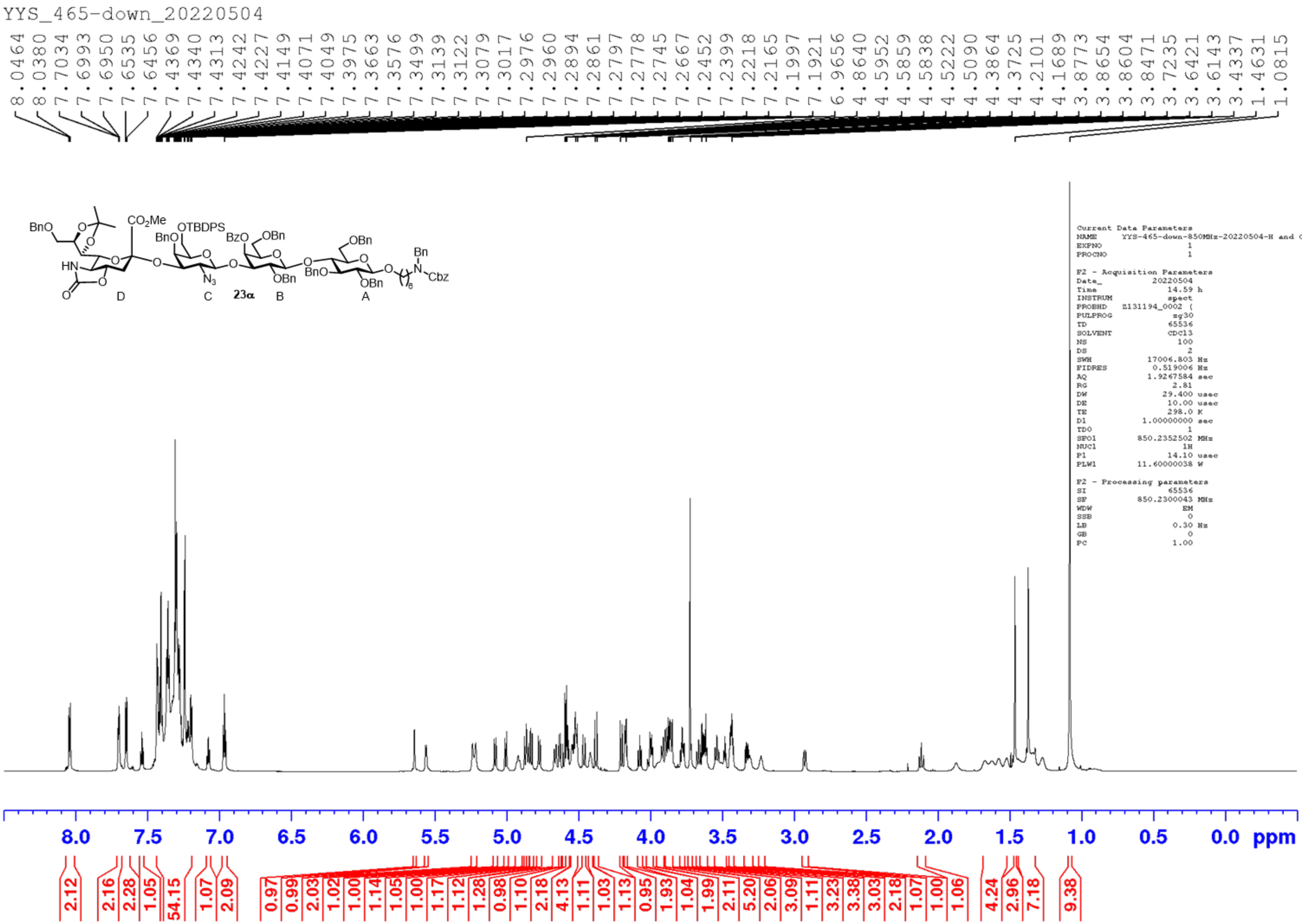

YYS_465-down_20220504
8.0464 8.0380 7.7034 7.6993 7.6950 7.6535 7.6456 7.4369 7.4340 7.4313 7.4242 7.4227 7.4149 7.4071 7.4049 7.3975 7.3663 7.3576 7.3499 7.3139 7.3122 7.3079 7.3017 7.2976 7.2960 7.2894 7.2861 7.2797 7.2778 7.2745 7.2667 7.2452 7.2399 7.2218 7.2165 7.1997 7.1921 6.9656 4.8640 4.5952 4.5859 4.5838 4.5222 4.5090 4.3864 4.3725 4.2101 4.1689 3.8773 3.8654 3.8604 3.8471 3.7235 3.6421 3.6143 3.4337 1.4631 1.0815
BnO
CO2Me
OTBDPS
BnO
BzO
OBn
OBn
Bn
HN
O
N3
OBn
BnO
OBn
Cbz
D
C
23α
B
A
Current Data Parameters
NAME YYS-465-down-850MHz-20220504-H and C
EXPNO 1
PROCNO 1
F2 - Acquisition Parameters
Date_ 20220504
Time 14.59 h
INSTRUM spect
PROBHD Z131194_0002 (
PULPROG zg30
TD 65536
SOLVENT CDCl3
NS 100
DS 2
SWH 17006.803 Hz
FIDRES 0.519006 Hz
AQ 1.9267584 sec
RG 2.81
DW 29.400 usec
DE 10.00 usec
TE 298.0 K
D1 1.00000000 sec
TD0 1
SFO1 850.2352502 MHz
NUC1 1H
P1 14.10 usec
PLW1 11.60000038 W
F2 - Processing parameters
SI 65536
SF 850.2300043 MHz
WDW EM
SSB 0
LB 0.30 Hz
GB 0
PC 1.00
8.0 7.5 7.0 6.5 6.0 5.5 5.0 4.5 4.0 3.5 3.0 2.5 2.0 1.5 1.0 0.5 0.0 ppm
2.12 2.16 2.28 1.05 54.15 1.07 2.09 0.97 0.99 2.03 1.02 1.00 1.14 1.05 1.00 1.17 1.12 1.28 0.98 1.10 2.18 4.13 1.11 1.03 1.13 0.95 1.93 1.04 1.99 2.11 5.20 2.06 3.09 1.11 3.23 3.38 3.03 2.18 1.07 1.00 1.06 4.24 2.96 7.18 9.38

YYS_465-down_20220504 13C

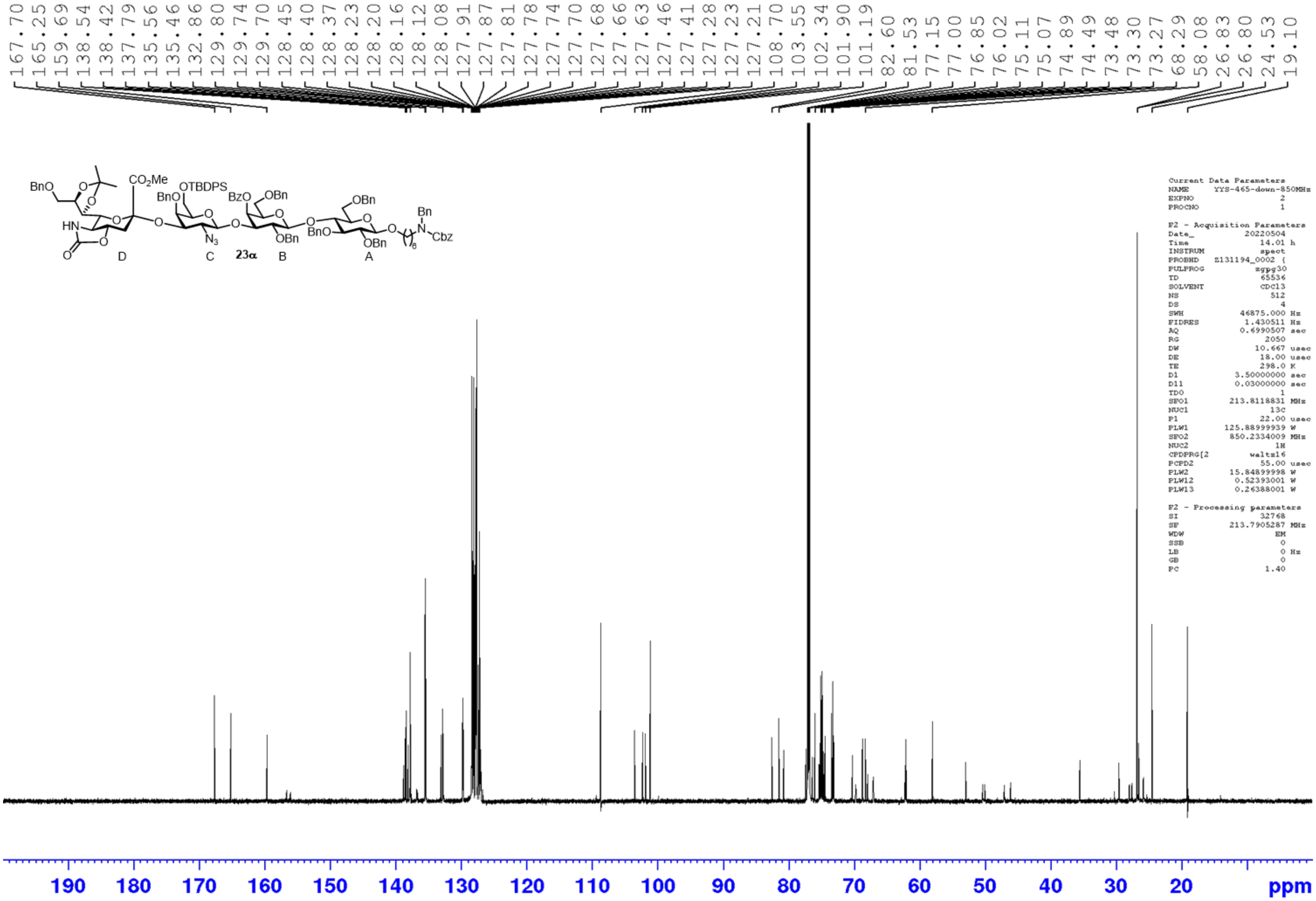

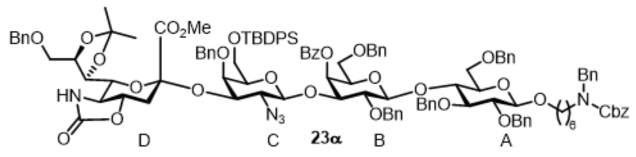
BnO
CO2Me
OTBDPS
BnO
BzO
OBn
OBn
Bn
HN
N3
BnO
OBn
OBn
N
Cbz
O
D
C
23α
B
A

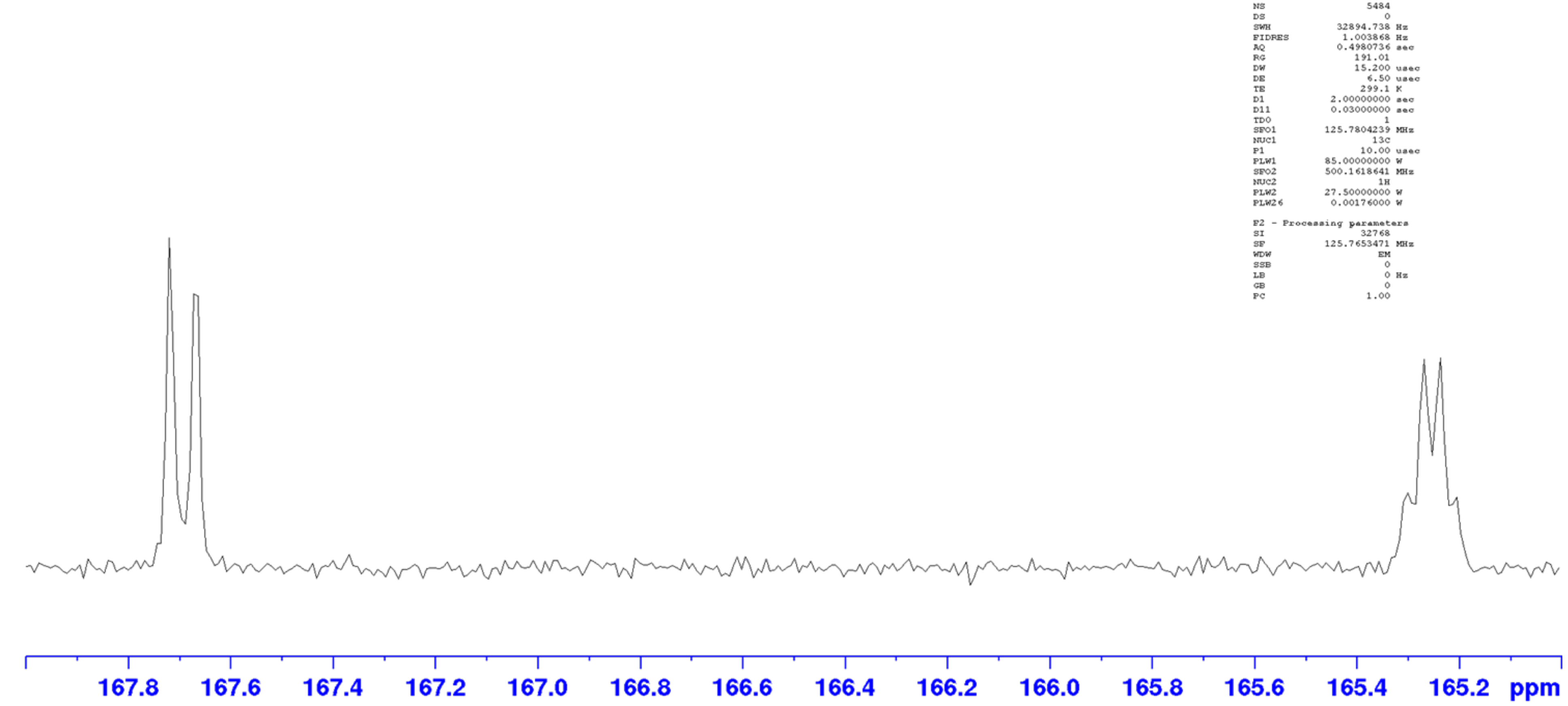
YYS-465-down
167.72
167.67
165.31
165.27
165.24
165.21
Current Data Parameters
NAME YYS-465-down-500MHz-c
EXPNO 2
PROCNO 2
F2 - Acquisition Parameters
Date_ 20220426
Time 11.09 h
INSTRUM spect
PROBHD Z119470_0234 (
PULPROG zgcw
TD 32768
SOLVENT CDCl3
NS 5484
DS 0
SWH 32894.738 Hz
FIDRES 1.003868 Hz
AQ 0.4980736 sec
RG 191.01
DW 15.200 usec
DE 6.50 usec
TE 299.1 K
D1 2.00000000 sec
D11 0.03000000 sec
TD0 1
SFO1 125.7804239 MHz
NUC1 13C
P1 10.00 usec
PLW1 85.00000000 W
SFO2 500.1618641 MHz
NUC2 1H
PLW2 27.50000000 W
PLW26 0.00176000 W
F2 - Processing parameters
SI 32768
SF 125.7653471 MHz
WDW EM
SSB 0
LB 0 Hz
GB 0
PC 1.00
167.8
167.6
167.4
167.2
167.0
166.8
166.6
166.4
166.2
166.0
165.8
165.6
165.4
165.2
ppm

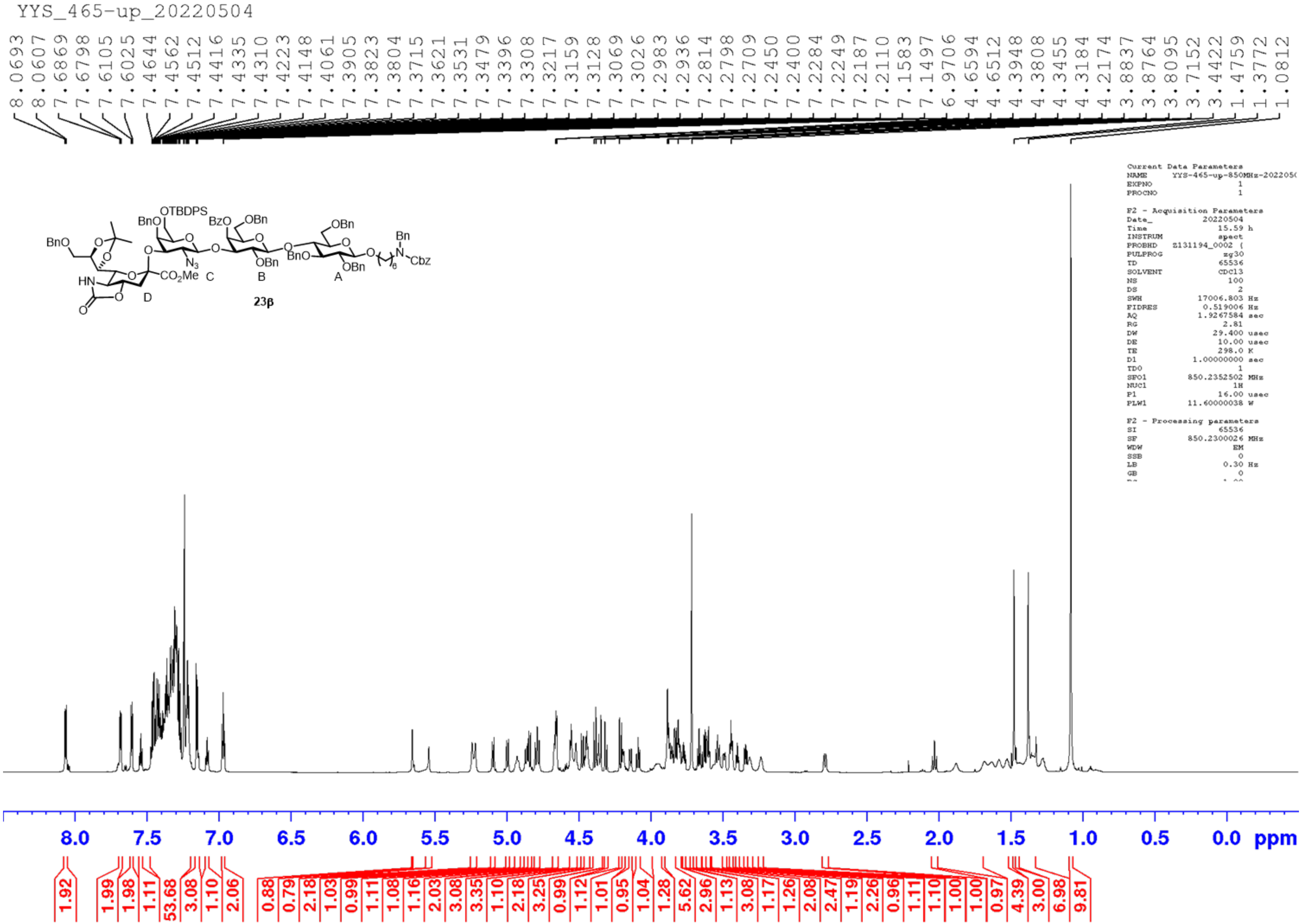
YYS_465-up_20220504
23β
Current Data Parameters
F2 - Acquisition Parameters
F2 - Processing parameters
ppm

YYS_465-up_20220504 13C

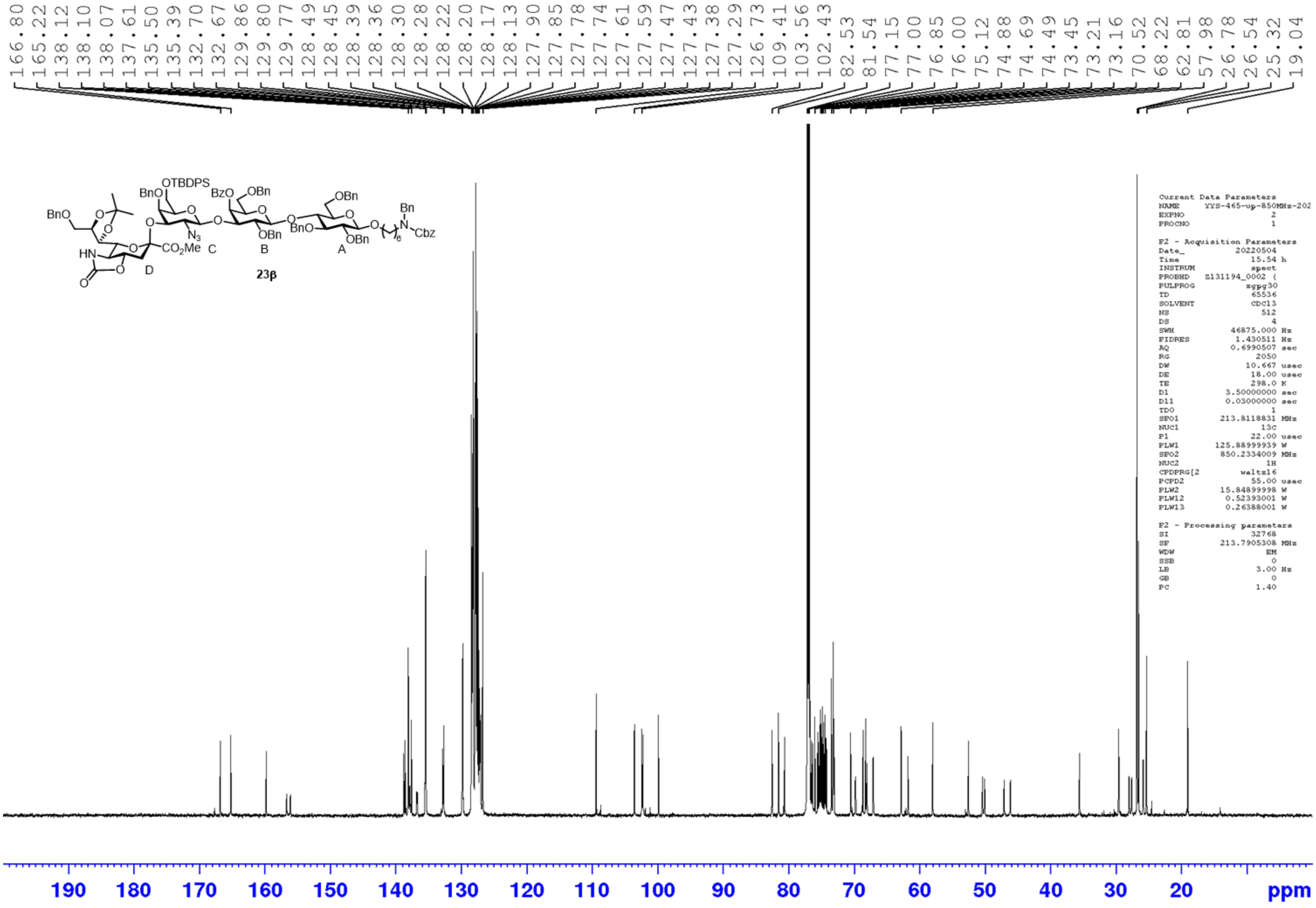

YYS-465-up

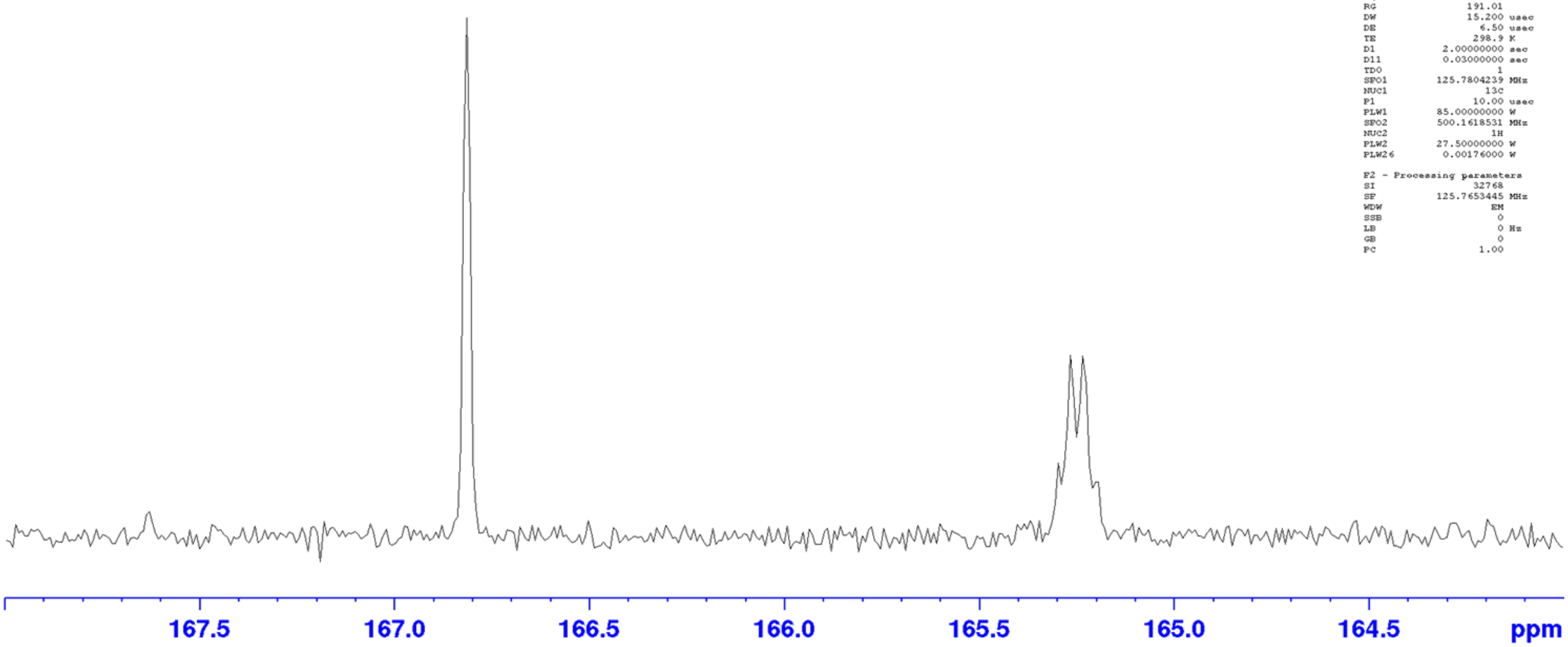

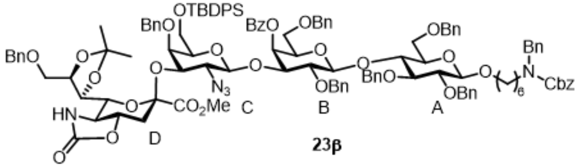

23β

Current Data Parameters
NAME YYS-465-up-500MHz-deco
EXPNO 2
PROCNO 2

F2 - Acquisition Parameters
Date_ 20220427
Time 8.48 h
INSTRUM spect
PROBHD Z119470_0234 (
PULPROG zgcw
TD 32768
SOLVENT CDCl3
NS 2911
DS 0
SWH 32894.738 Hz
FIDRES 1.003868 Hz
AQ 0.4980736 sec
RG 191.01
DW 15.200 usec
DE 6.50 usec
TE 298.9 K
D1 2.00000000 sec
D11 0.03000000 sec
TD0 1
SFO1 125.7804239 MHz
NUC1 13C
P1 10.00 usec
PLW1 85.00000000 W
SFO2 500.1618531 MHz
NUC2 1H
PLW2 27.50000000 W
PLW26 0.00176000 W

F2 - Processing parameters
SI 32768
SF 125.7653445 MHz
WDW EM
SSB 0
LB 0 Hz
GB 0
PC 1.00

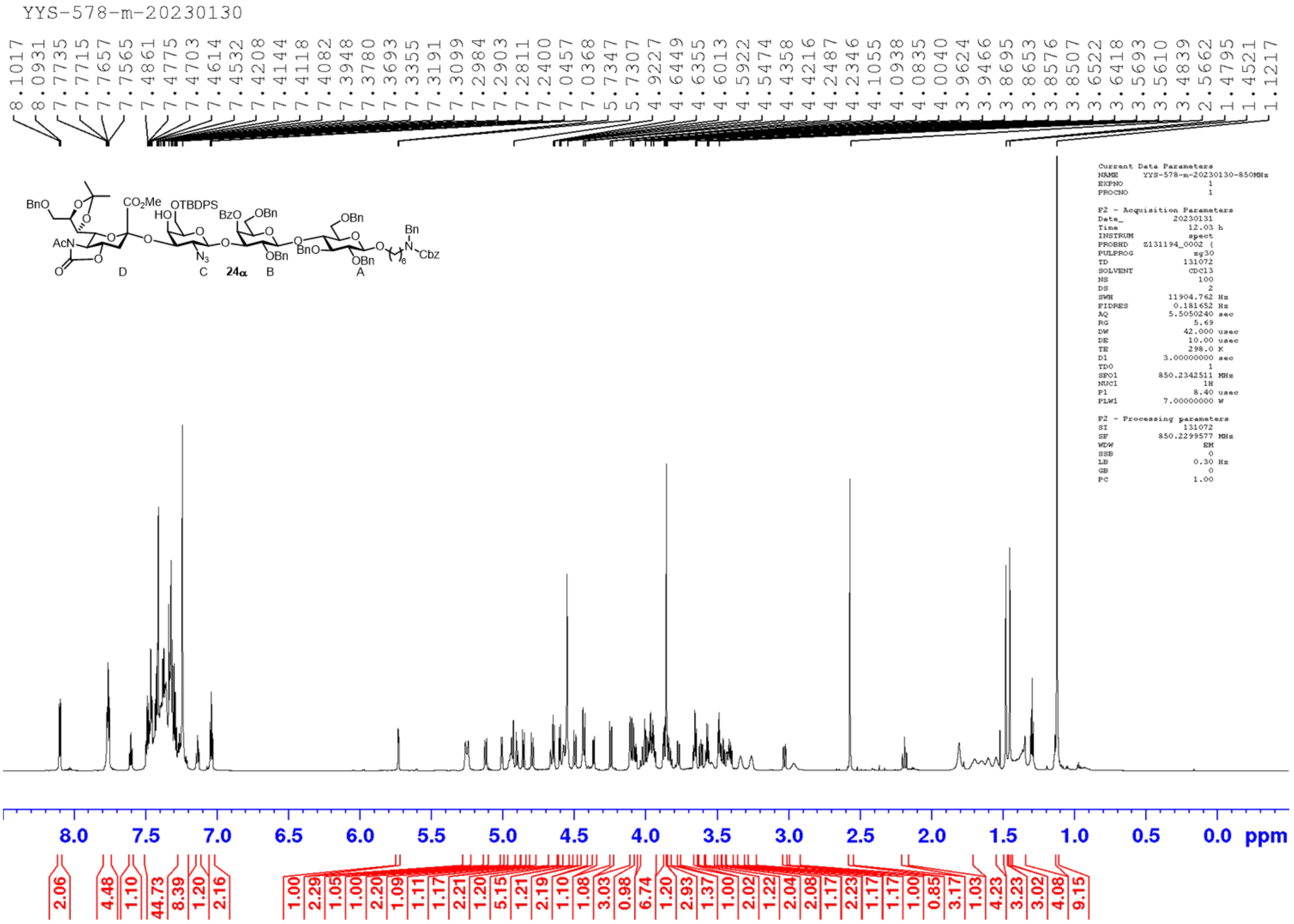

YYS-578-m-20230130
24α
Current Data Parameters
NAME YYS-578-m-20230130-850MHz
EXPNO 1
PROCNO 1
F2 - Acquisition Parameters
Date_ 20230131
Time 12.03 h
INSTRUM spect
PROBHD Z131194_0002 (
PULPROG zg30
TD 131072
SOLVENT CDCl3
NS 100
DS 2
SWH 11904.762 Hz
FIDRES 0.181652 Hz
AQ 5.5050240 sec
RG 5.69
DW 42.000 usec
DE 10.00 usec
TE 298.0 K
D1 3.00000000 sec
TD0 1
SFO1 850.2342511 MHz
NUC1 1H
P1 8.40 usec
PLW1 7.00000000 W
F2 - Processing parameters
SI 131072
SF 850.2299577 MHz
WDW EM
SSB 0
LB 0.30 Hz
GB 0
PC 1.00
ppm

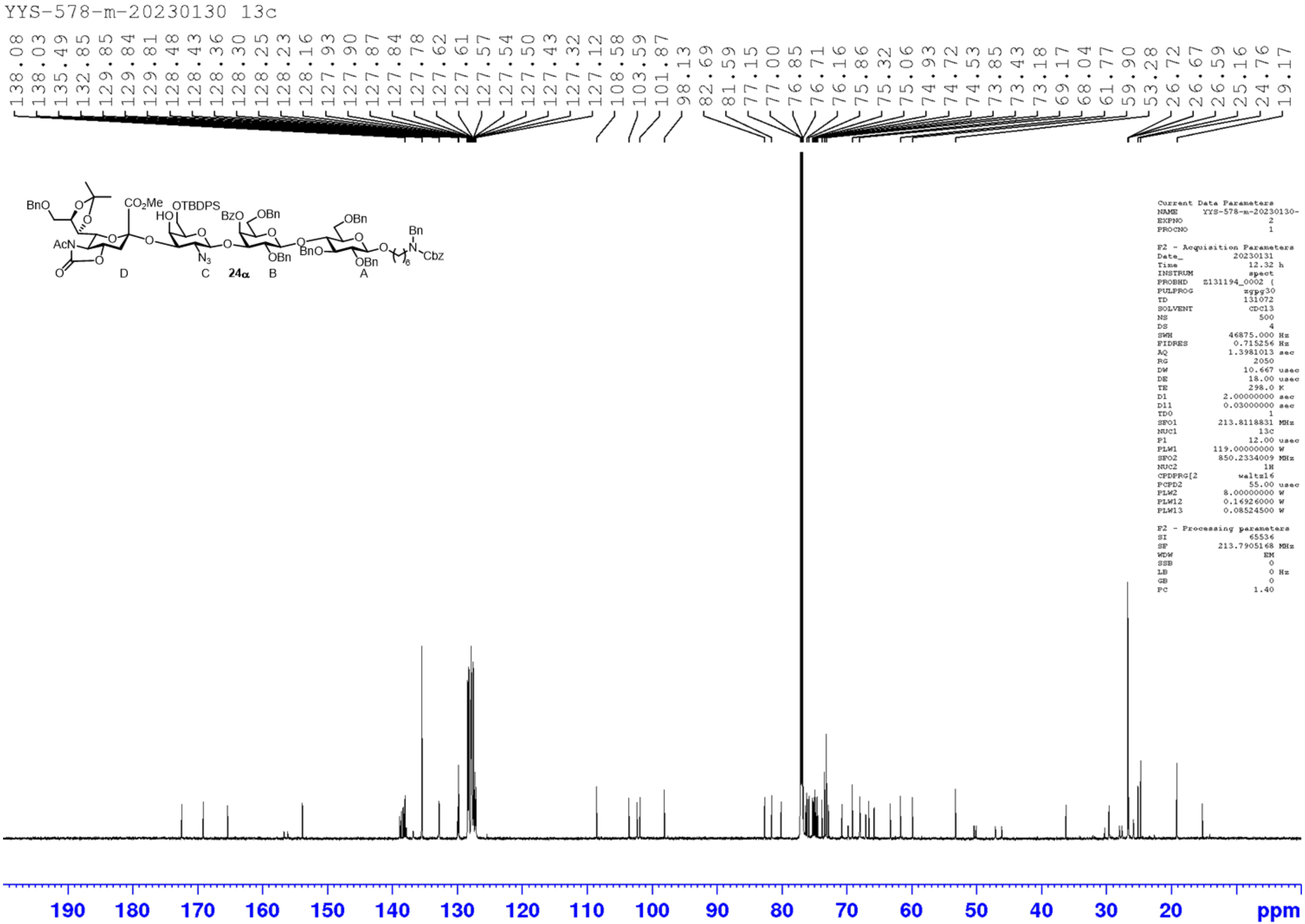
YYS-578-m-20230130 13c
138.08
138.03
135.49
132.85
129.85
129.84
129.81
128.48
128.43
128.36
128.30
128.25
128.23
128.16
127.93
127.90
127.87
127.84
127.78
127.62
127.61
127.57
127.54
127.50
127.43
127.32
127.12
108.58
103.59
101.87
98.13
82.69
81.59
77.15
77.00
76.85
76.71
76.16
75.86
75.32
75.06
74.93
74.72
74.53
73.85
73.43
73.18
69.17
68.04
61.77
59.90
53.28
26.72
26.67
26.59
25.16
24.76
19.17
BnO
CO2Me
OTBDPS
HO
BzO
OBn
OBn
AcN
N3
OBn
BnO
OBn
Bn
N
Cbz
D
C
24α
B
A
Current Data Parameters
NAME YYS-578-m-20230130-
EXPNO 2
PROCNO 1
F2 - Acquisition Parameters
Date_ 20230131
Time 12.32 h
INSTRUM spect
PROBHD Z131194_0002 (
PULPROG zgpg30
TD 131072
SOLVENT CDCl3
NS 500
DS 4
SWH 46875.000 Hz
FIDRES 0.715256 Hz
AQ 1.3981013 sec
RG 2050
DW 10.667 usec
DE 18.00 usec
TE 298.0 K
D1 2.00000000 sec
D11 0.03000000 sec
TD0 1
SFO1 213.8118831 MHz
NUC1 13C
P1 12.00 usec
PLW1 119.00000000 W
SFO2 850.2334009 MHz
NUC2 1H
CPDPRG[2 waltz16
PCPD2 55.00 usec
PLW2 8.00000000 W
PLW12 0.16926000 W
PLW13 0.08524500 W
F2 - Processing parameters
SI 65536
SF 213.7905168 MHz
WDW EM
SSB 0
LB 0 Hz
GB 0
PC 1.40
190 180 170 160 150 140 130 120 110 100 90 80 70 60 50 40 30 20 ppm

YYS-578-m-20230130 HMBC

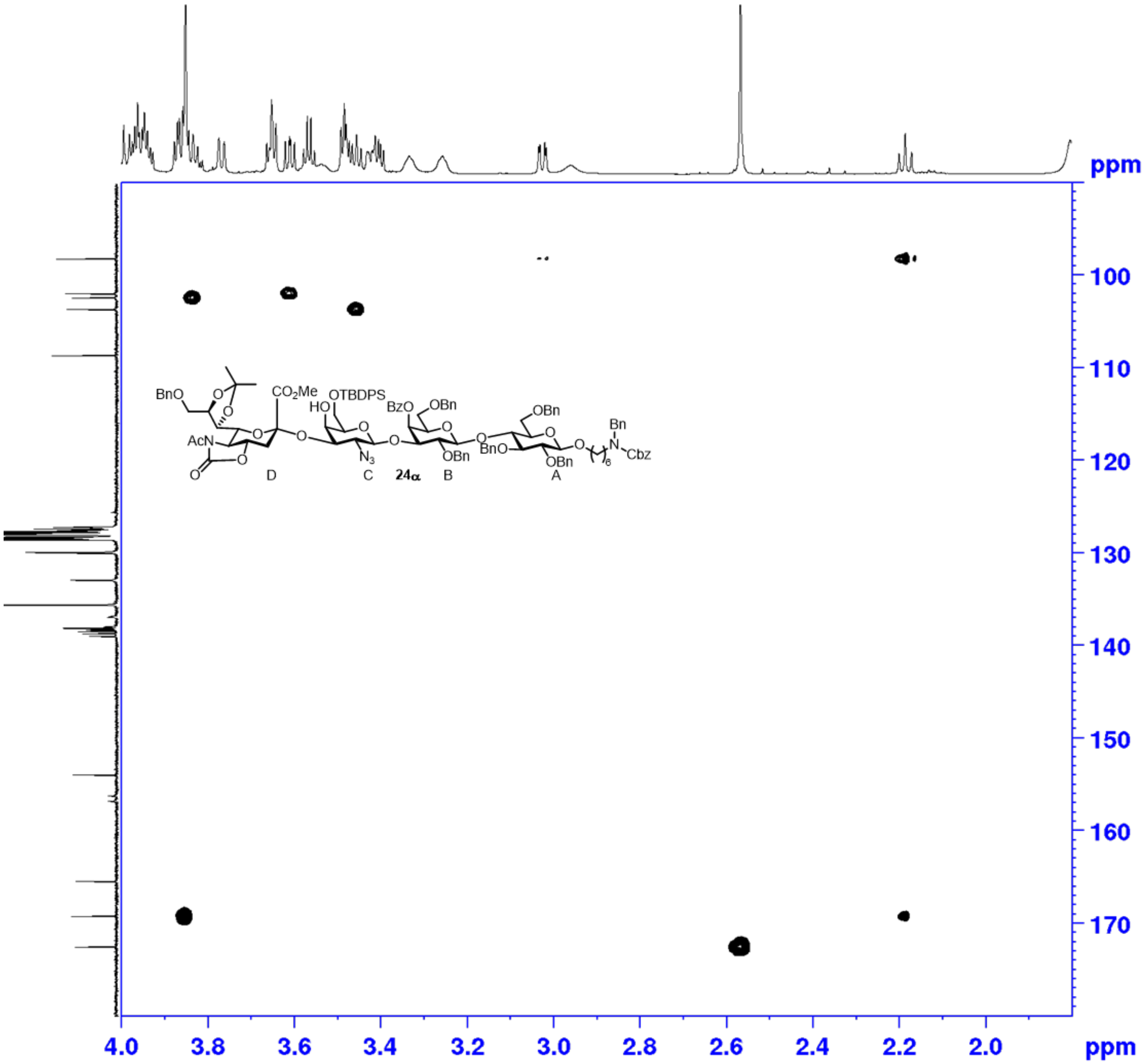


```
Current Data Parameters
NAME      YYS-578-m-20230130-850MHz
EXPNO                     3
PROCNO                    1

F2 - Acquisition Parameters
Date_              20230131
Time                  12.34 h
INSTRUM               spect
PROBHD      Z131194_0002 (
PULPROG     clhmbcetgpl3nd
TD                     2048
SOLVENT               CDCl3
NS                       24
DS                       16
SWH                8503.401 Hz
FIDRES             8.304103 Hz
AQ                0.1204224 sec
RG                   184.37
DW                   58.800 usec
DE                    10.00 usec
TE                    298.0 K
CNST6           125.0000000
CNST7           165.0000000
CNST13            8.0000000
D0               0.00000300 sec
D1               1.50000000 sec
D6               0.06250000 sec
D16              0.00020000 sec
D21                       0 sec
IN0              0.00001170 sec
L0                        0
TDav                      1
SFO1            850.2339961 MHz
NUC1                     1H
P1                     8.40 usec
P2                    16.80 usec
PLW1             6.99840021 W
SFO2            213.8118831 MHz
NUC2                    13C
P3                    12.00 usec
P14                  500.00 usec
P24                 2000.00 usec
PLW2           130.00000000 W
SPNAM[3]   Crp80,0.5,20.1
SPOAL3                0.500
SPOFFS3                   0 Hz
SPW3            38.13600159 W
SPNAM[7]      Crp80comp.4
SPOAL7                0.500
SPOFFS7                   0 Hz
SPW7            38.13600159 W
GPNAM[1]        SMSQ10.100
GPZ1                  80.00 %
GPNAM[3]        SMSQ10.100
GPZ3                  14.00 %
P16                 1000.00 usec

F1 - Acquisition parameters
TD                      360
SFO1               213.8119 MHz
FIDRES           237.416901 Hz
SW                  199.872 ppm
FnMODE       Echo-Antiecho

F2 - Processing parameters
SI                     1024
SF              850.2299537 MHz
WDW                   QSINE
SSB                       2
LB                        0 Hz
GB                        0
PC                     1.40

F1 - Processing parameters
SI                     1024
MC2          echo-antiecho
SF              213.7905200 MHz
WDW                   QSINE
SSB                       2
LB                        0 Hz
GB                        0
```

YYS-578-m-20230130 HMBC

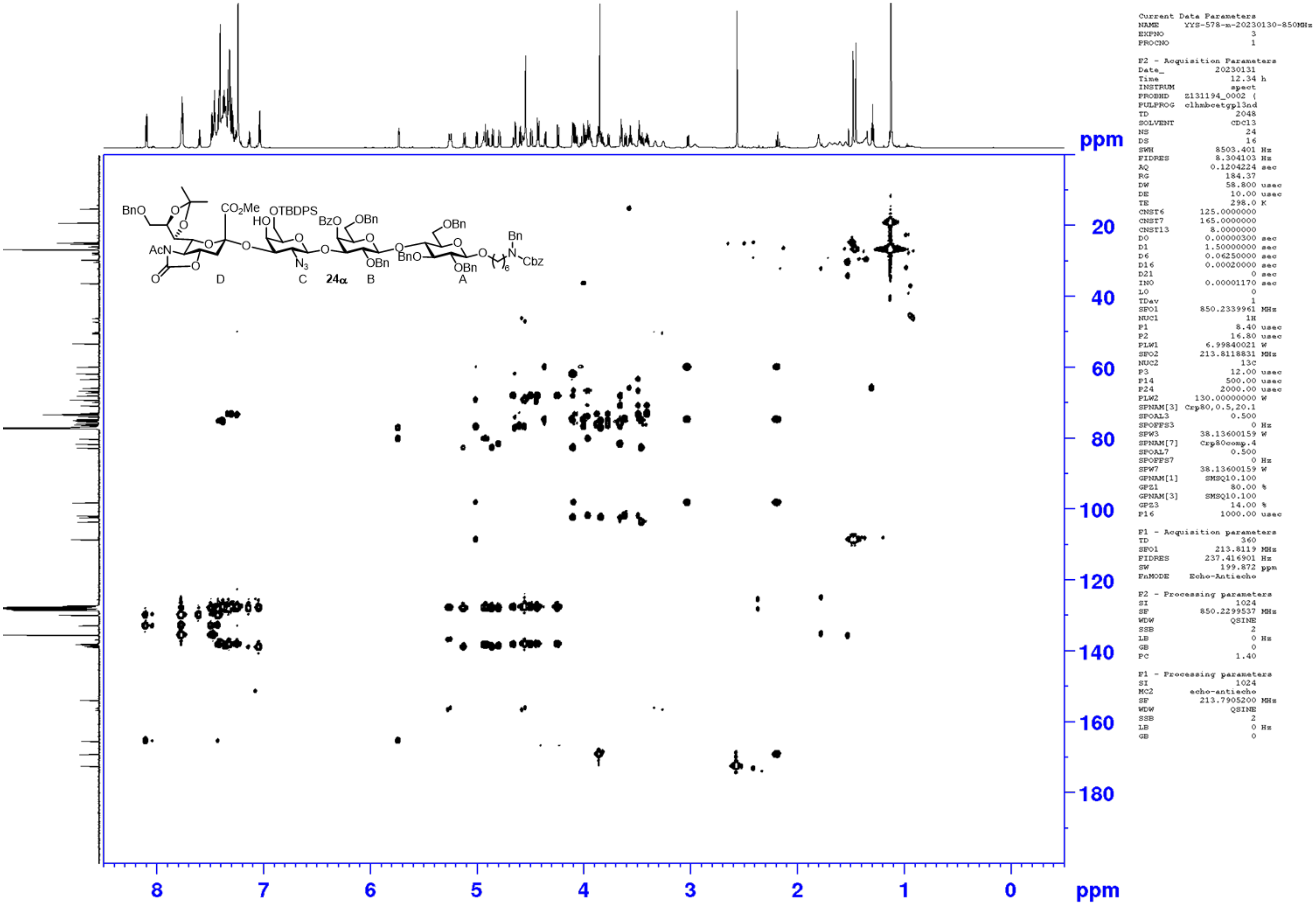

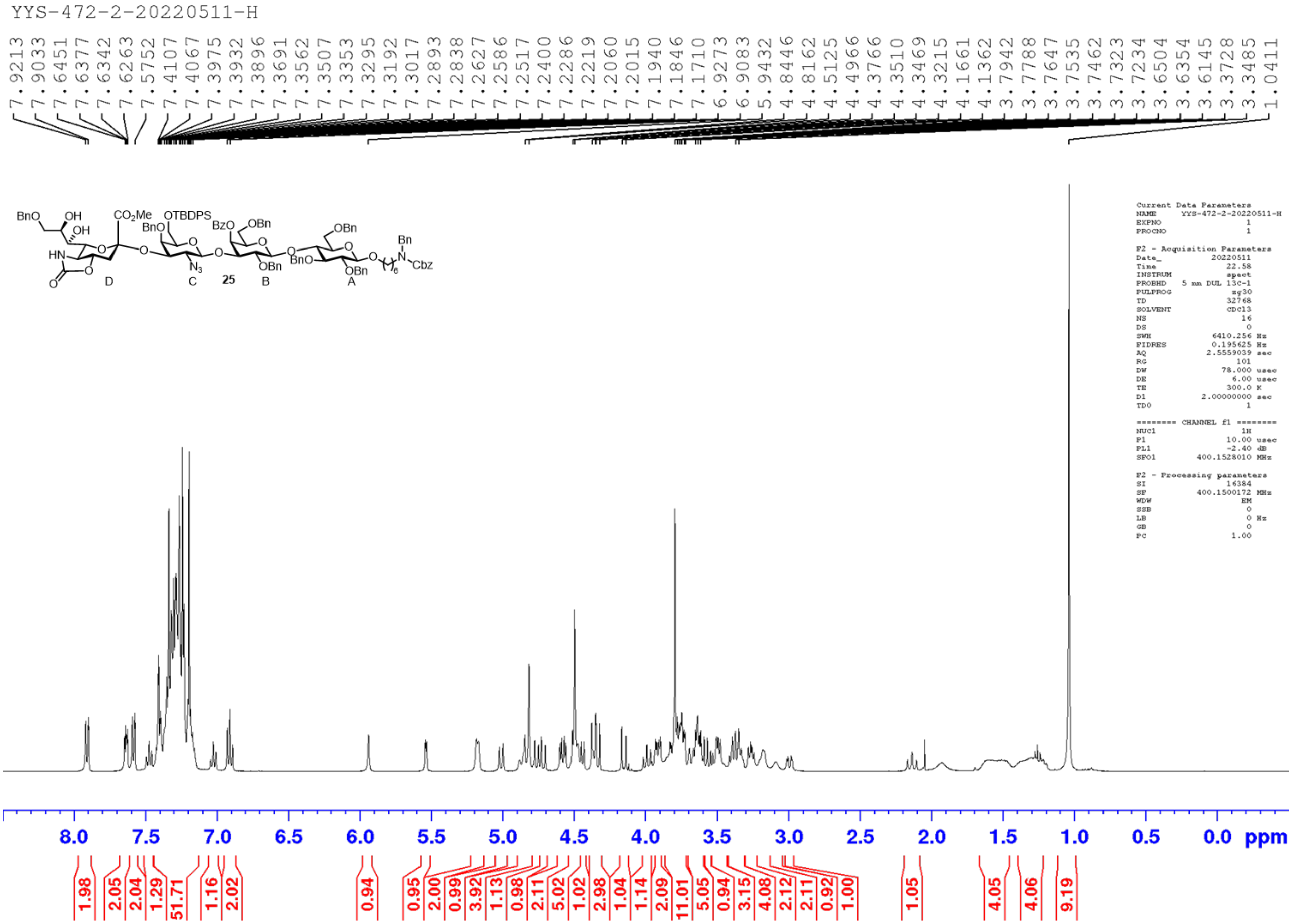

YYS-472-2-20220511-H
7.9213 7.9033 7.6451 7.6377 7.6342 7.6263 7.5752 7.4107 7.4067 7.3975 7.3932 7.3896 7.3691 7.3562 7.3507 7.3353 7.3295 7.3192 7.3017 7.2893 7.2838 7.2627 7.2586 7.2517 7.2400 7.2286 7.2219 7.2060 7.2015 7.1940 7.1846 7.1710 6.9273 6.9083 5.9432 4.8446 4.8162 4.5125 4.4966 4.3766 4.3510 4.3469 4.3215 4.1661 4.1362 3.7942 3.7788 3.7647 3.7535 3.7462 3.7323 3.7234 3.6504 3.6354 3.6145 3.3728 3.3485 1.0411
BnO OH OH CO2Me OTBDPS BnO BzO OBn OBn Bn HN O O N3 OBn BnO OBn N Cbz
D C 25 B A
Current Data Parameters
NAME YYS-472-2-20220511-H
EXPNO 1
PROCNO 1
F2 - Acquisition Parameters
Date_ 20220511
Time 22.58
INSTRUM spect
PROBHD 5 mm DUL 13C-1
PULPROG zg30
TD 32768
SOLVENT CDCl3
NS 16
DS 0
SWH 6410.256 Hz
FIDRES 0.195625 Hz
AQ 2.5559039 sec
RG 101
DW 78.000 usec
DE 6.00 usec
TE 300.0 K
D1 2.00000000 sec
TD0 1
======== CHANNEL f1 ========
NUC1 1H
P1 10.00 usec
PL1 -2.40 dB
SFO1 400.1528010 MHz
F2 - Processing parameters
SI 16384
SF 400.1500172 MHz
WDW EM
SSB 0
LB 0 Hz
GB 0
PC 1.00
8.0 7.5 7.0 6.5 6.0 5.5 5.0 4.5 4.0 3.5 3.0 2.5 2.0 1.5 1.0 0.5 0.0 ppm
1.98 2.05 2.04 1.29 51.71 1.16 2.02 0.94 0.95 2.00 0.99 3.92 1.13 0.98 2.11 5.02 1.02 2.98 1.04 1.14 2.09 11.01 5.05 0.94 3.15 4.08 2.12 2.11 0.92 1.00 1.05 4.05 4.06 9.19

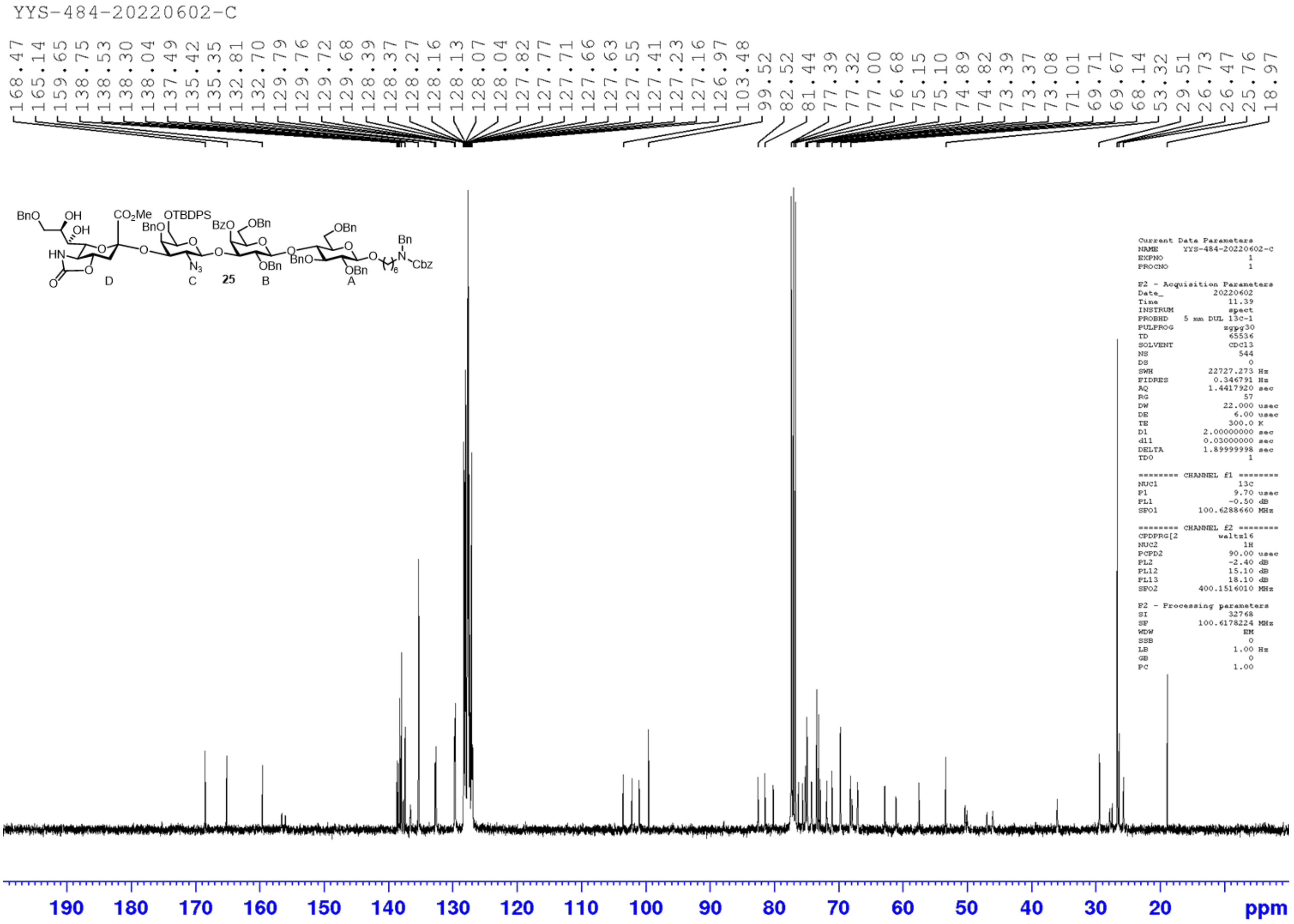
YYS-484-20220602-C
168.47 165.14 159.65 138.75 138.53 138.30 138.04 137.49 135.42 135.35 132.81 132.70 129.79 129.76 129.72 129.68 128.39 128.37 128.27 128.16 128.13 128.07 128.04 127.82 127.77 127.71 127.66 127.63 127.55 127.41 127.23 127.16 126.97 103.48 99.52 82.52 81.44 77.39 77.32 77.00 76.68 75.15 75.10 74.89 74.82 73.39 73.37 73.08 71.01 69.71 69.67 68.14 53.32 29.51 26.73 26.47 25.76 18.97
BnO
OH
OH
CO_2Me
OTBDPS
BnO
BzO
OBn
OBn
Bn
HN
O
O
O
O
O
N_3
OBn
BnO
O
OBn
O
N
Cbz
8
D
C
25
B
A
Current Data Parameters
NAME YYS-484-20220602-c
EXPNO 1
PROCNO 1
F2 - Acquisition Parameters
Date_ 20220602
Time 11.39
INSTRUM spect
PROBHD 5 mm DUL 13C-1
PULPROG zgpg30
TD 65536
SOLVENT CDCl3
NS 544
DS 0
SWH 22727.273 Hz
FIDRES 0.346791 Hz
AQ 1.4417920 sec
RG 57
DW 22.000 usec
DE 6.00 usec
TE 300.0 K
D1 2.00000000 sec
d11 0.03000000 sec
DELTA 1.89999998 sec
TD0 1
======== CHANNEL f1 ========
NUC1 13C
P1 9.70 usec
PL1 -0.50 dB
SFO1 100.6288660 MHz
======== CHANNEL f2 ========
CPDPRG[2 waltz16
NUC2 1H
PCPD2 90.00 usec
PL2 -2.40 dB
PL12 15.10 dB
PL13 18.10 dB
SFO2 400.1516010 MHz
F2 - Processing parameters
SI 32768
SF 100.6178224 MHz
WDW EM
SSB 0
LB 1.00 Hz
GB 0
PC 1.00
190 180 170 160 150 140 130 120 110 100 90 80 70 60 50 40 30 20 ppm

YYS-669-u-20231018

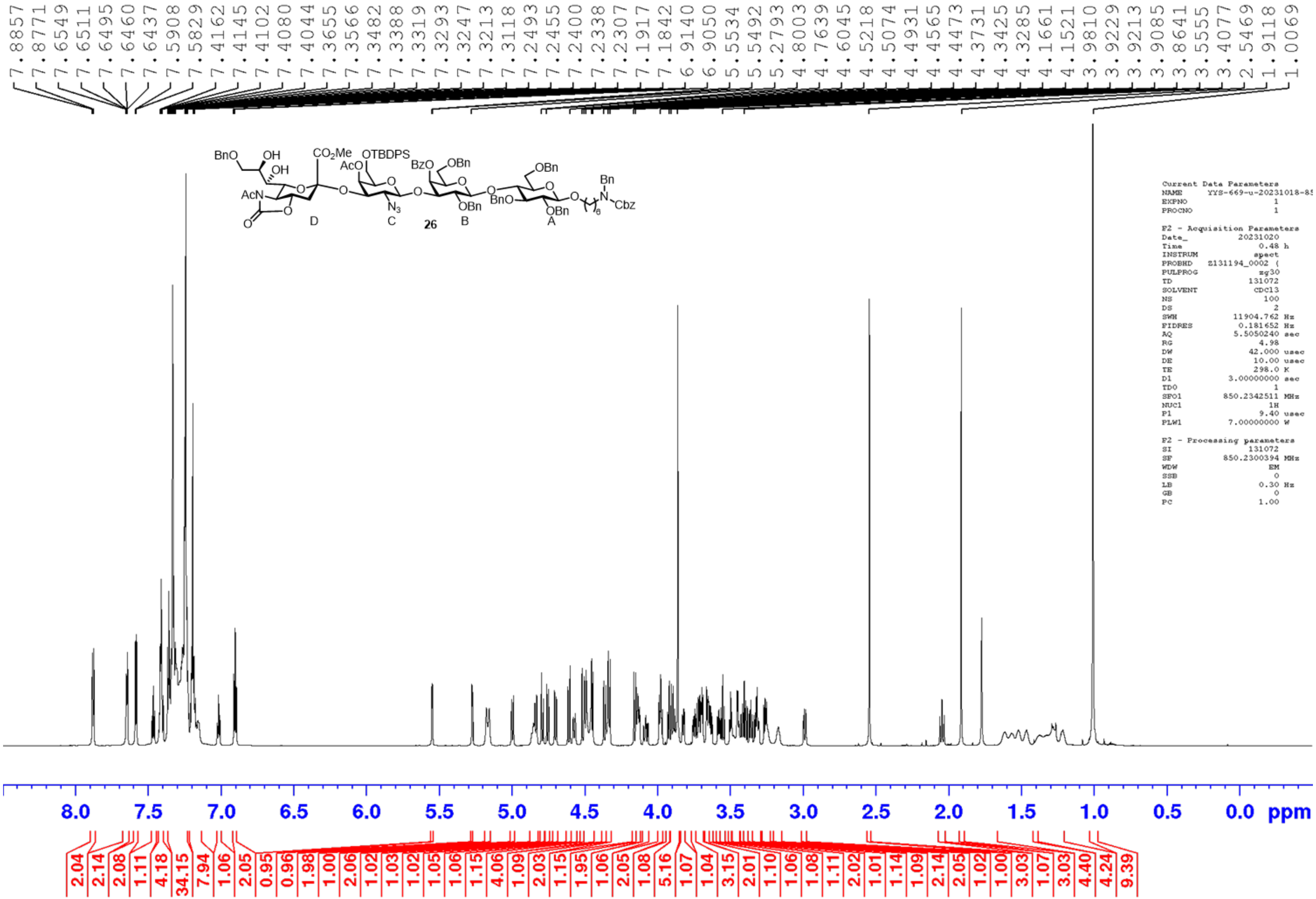

YYS-669-u-20231018 13c

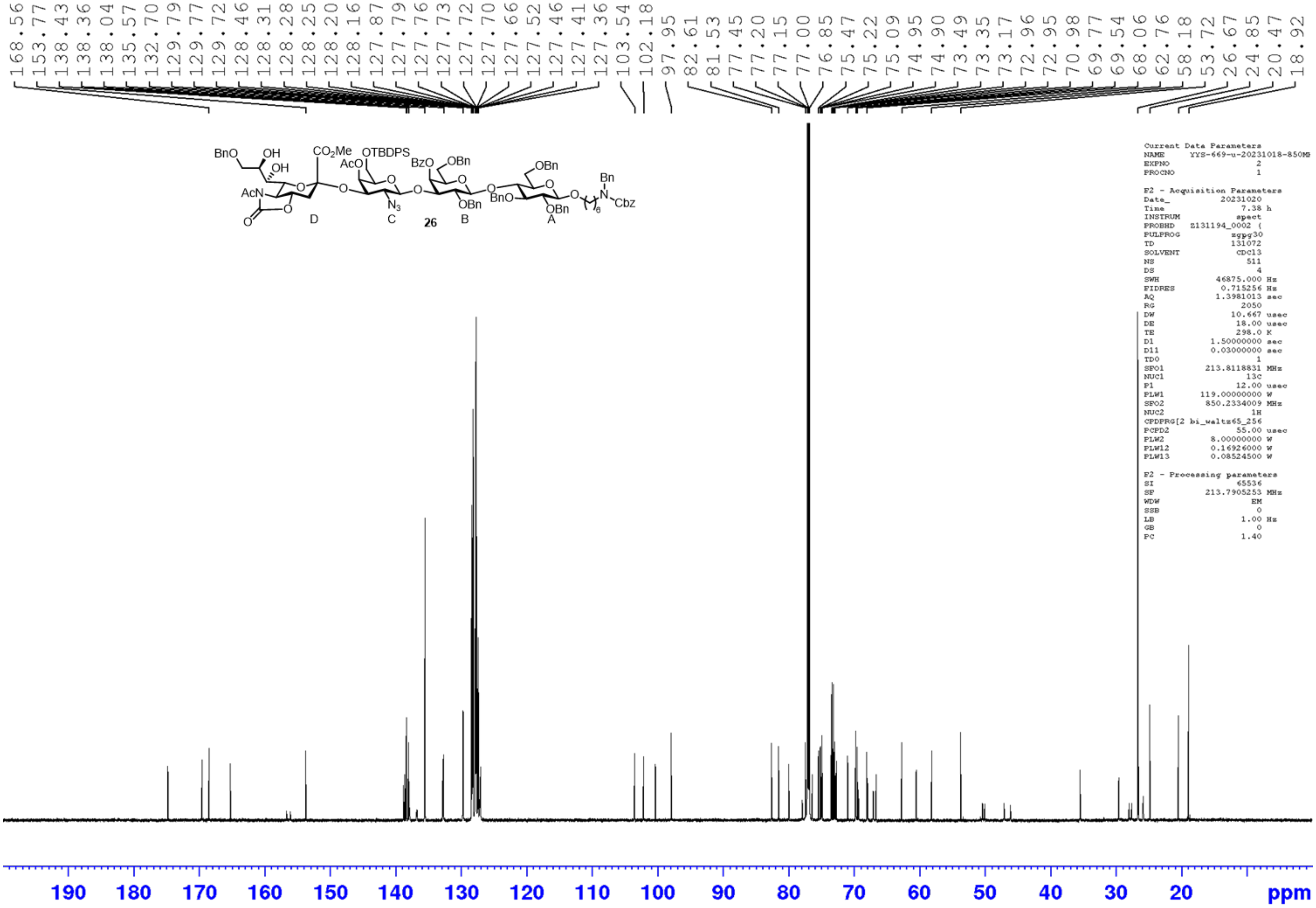

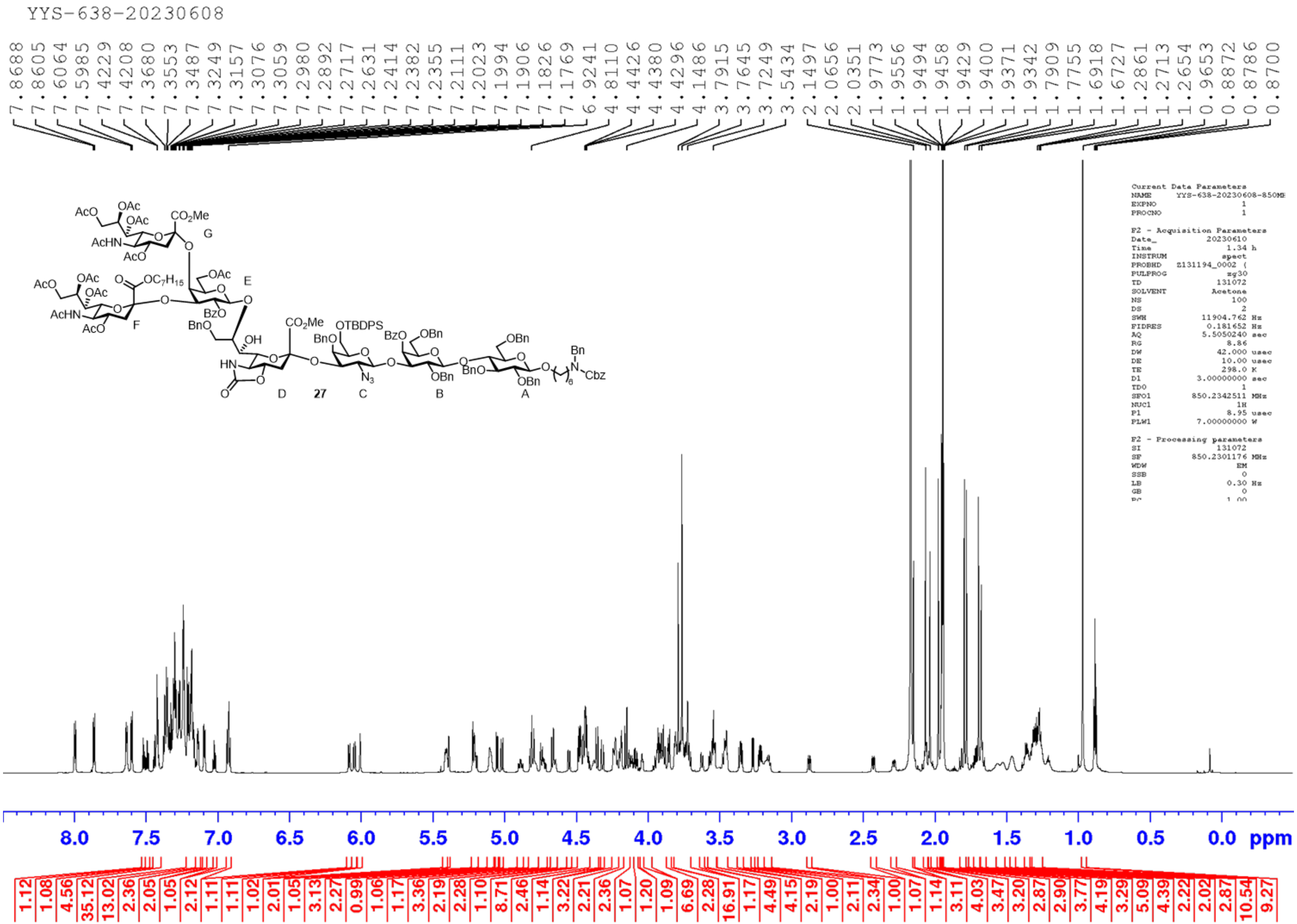
YYS-638-20230608
7.8688 7.8605 7.6064 7.5985 7.4229 7.4208 7.3680 7.3553 7.3487 7.3249 7.3157 7.3076 7.3059 7.2980 7.2892 7.2717 7.2631 7.2414 7.2382 7.2355 7.2111 7.2023 7.1994 7.1906 7.1826 7.1769 6.9241 4.8110 4.4426 4.4380 4.4296 4.1486 3.7915 3.7645 3.7249 3.5434 2.1497 2.0656 2.0351 1.9773 1.9556 1.9494 1.9458 1.9429 1.9400 1.9371 1.9342 1.7909 1.7755 1.6918 1.6727 1.2861 1.2713 1.2654 0.9653 0.8872 0.8786 0.8700
AcO OAc OAc CO2Me AcHN AcO G
AcO OAc OAc O OC7H15 OAc E AcHN AcO F BzO BnO OH
CO2Me OTBDPS BnO BzO OBn OBn HN N3 OBn BnO OBn Bn N Cbz 6
O D 27 C B A
Current Data Parameters
NAME YYS-638-20230608-850MH
EXPNO 1
PROCNO 1
F2 - Acquisition Parameters
Date_ 20230610
Time 1.34 h
INSTRUM spect
PROBHD Z131194_0002 (
PULPROG zg30
TD 131072
SOLVENT Acetone
NS 100
DS 2
SWH 11904.762 Hz
FIDRES 0.181652 Hz
AQ 5.5050240 sec
RG 8.86
DW 42.000 usec
DE 10.00 usec
TE 298.0 K
D1 3.00000000 sec
TD0 1
SFO1 850.2342511 MHz
NUC1 1H
P1 8.95 usec
PLW1 7.00000000 W
F2 - Processing parameters
SI 131072
SF 850.2301176 MHz
WDW EM
SSB 0
LB 0.30 Hz
GB 0
8.0 7.5 7.0 6.5 6.0 5.5 5.0 4.5 4.0 3.5 3.0 2.5 2.0 1.5 1.0 0.5 0.0 ppm
1.12 1.08 4.56 35.12 13.02 2.36 2.05 1.05 2.12 1.11 1.11 1.02 2.01 1.05 3.13 2.27 0.99 1.06 1.17 3.36 2.19 2.28 1.10 8.71 2.46 1.14 3.22 2.21 2.36 1.07 1.20 1.09 6.69 2.28 16.91 1.17 4.49 4.15 2.19 1.00 2.11 2.34 1.00 1.07 1.14 3.11 4.03 3.47 3.20 2.87 2.90 3.77 4.19 3.29 5.09 4.39 2.22 2.02 2.87 10.54 9.27

YYS-639-20230615 13c

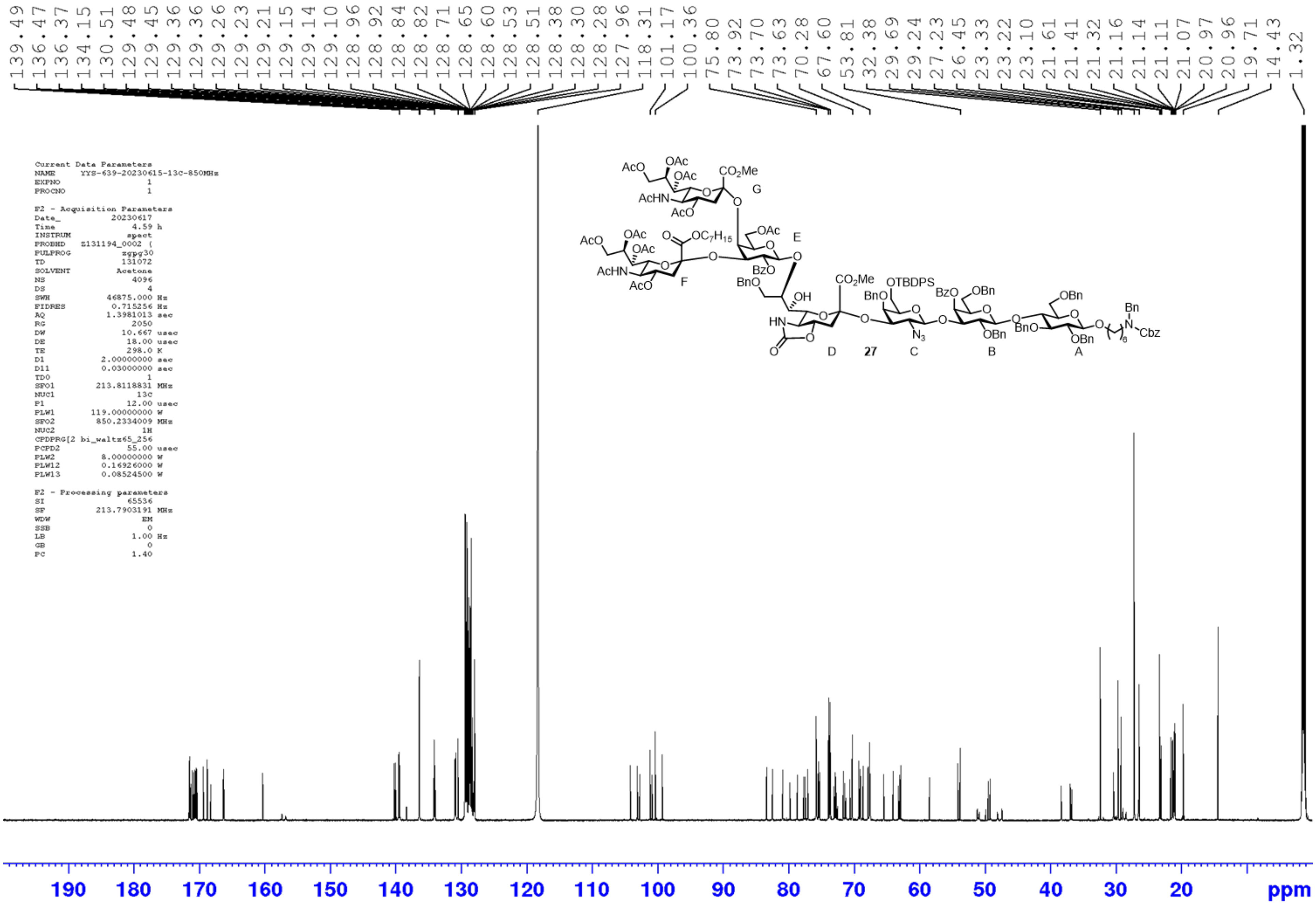

YYS-639-20230615 COSY

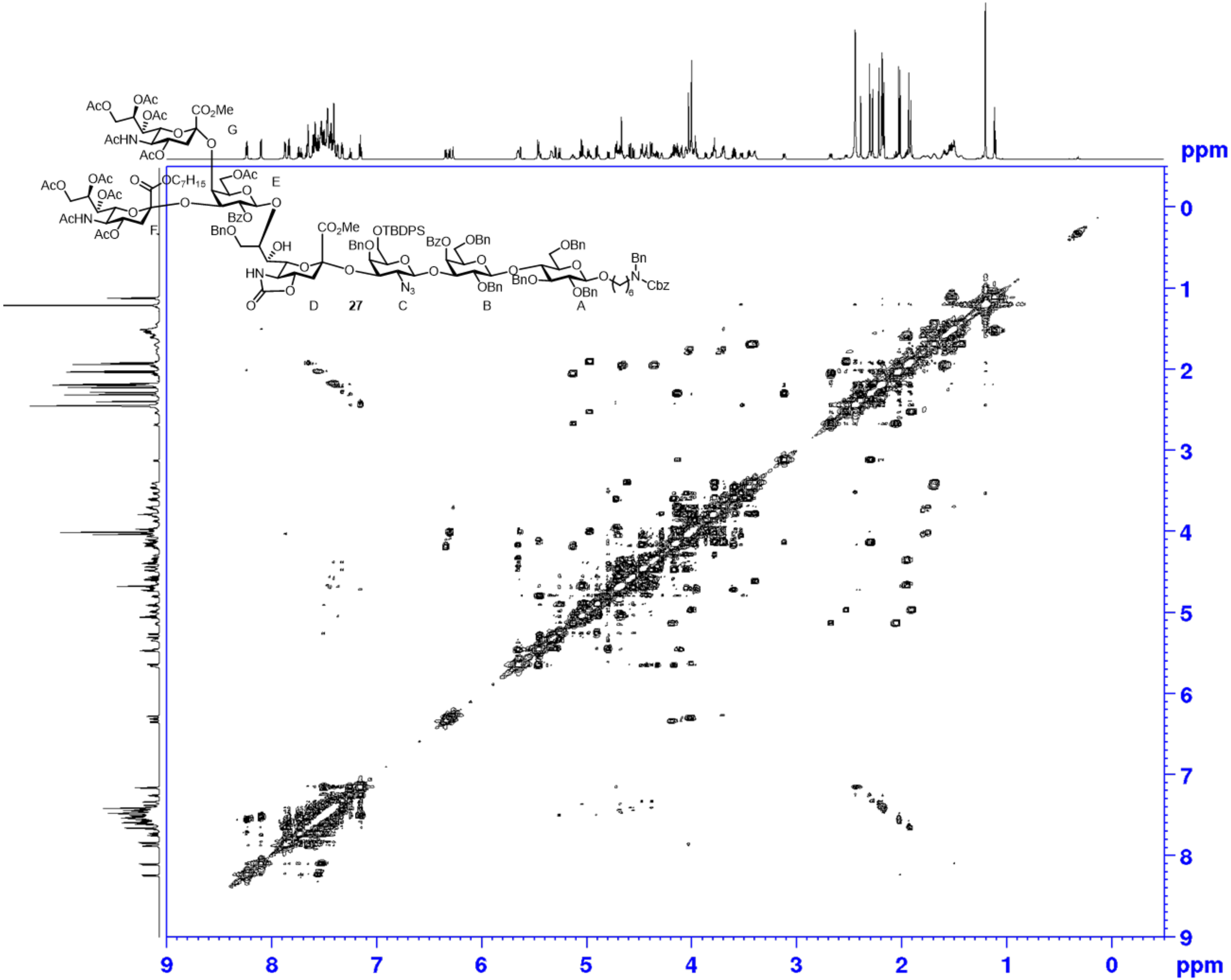

**Current Data Parameters**		
NAME	YYS-639-20230615-850MHz	
EXPNO	3	
PROCNO	1	
**F2 - Acquisition Parameters**		
Date_	20230616	
Time	9.56	h
INSTRUM	spect	
PROBHD	Z131194_0002 (	
PULPROG	cosygf90	
TD	2048	
SOLVENT	Acetone	
NS	8	
DS	0	
SWH	8503.401	Hz
FIDRES	8.304103	Hz
AQ	0.1204224	sec
RG	4.98	
DW	58.800	usec
DE	10.00	usec
TE	298.0	K
D0	0.00000300	sec
D1	2.00000000	sec
IN0	0.00011760	sec
TDav	1	
SFO1	850.2339961	MHz
NUC1	1H	
P1	9.10	usec
PLW1	6.99840021	W
**F1 - Acquisition parameters**		
TD	256	
SFO1	850.234	MHz
FIDRES	66.432823	Hz
SW	10.001	ppm
FnMODE	QF	
**F2 - Processing parameters**		
SI	1024	
SF	850.2299236	MHz
WDW	SINE	
SSB	0	
LB	0	Hz
GB	0	
PC	1.40	
**F1 - Processing parameters**		
SI	1024	
MC2	QF	
SF	850.2299232	MHz
WDW	SINE	
SSB	0	
LB	0	Hz
GB	0	

YYS-639-20230615 hsqc

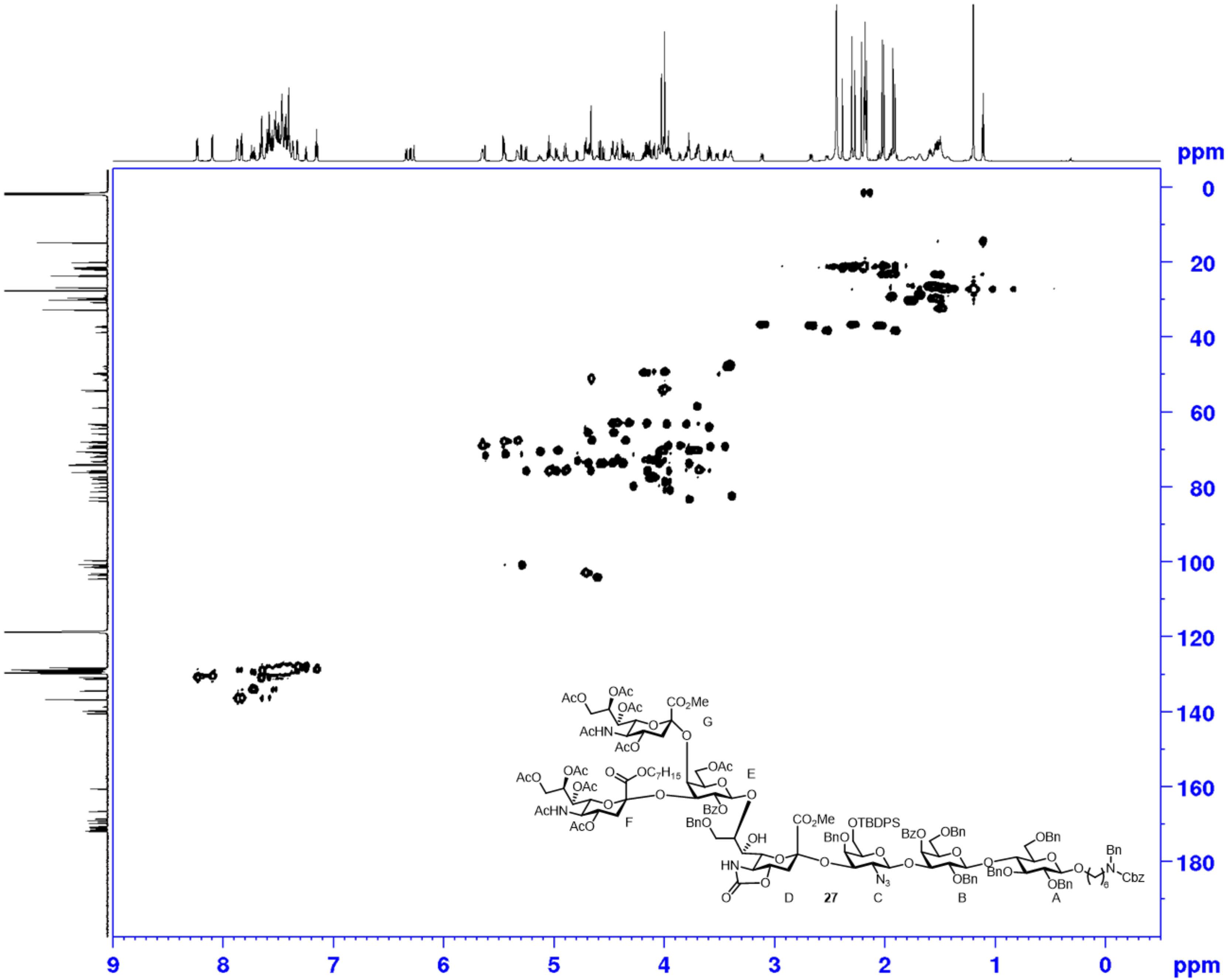

Current Data Parameters
NAME YYS-639-20230615-850MHz
EXPNO 5
PROCNO 1

F2 - Acquisition Parameters
Date_ 20230616
Time 15.40 h
INSTRUM spect
PROBHD Z131194_0002 (
PULPROG hsqcetgpsisp2.2
TD 2048
SOLVENT Acetone
NS 24
DS 16
SWH 11904.762 Hz
FIDRES 11.625744 Hz
AQ 0.0860160 sec
RG 184.37
DW 42.000 usec
DE 10.00 usec
TE 298.0 K
CNST2 145.0000000
CNST17 -0.5000000
D0 0.00000300 sec
D1 1.50000000 sec
D4 0.00172414 sec
D11 0.03000000 sec
D16 0.00020000 sec
D24 0.00086207 sec
IN0 0.00001060 sec
TDav 1
SFO1 850.2351014 MHz
NUC1 1H
P1 9.10 usec
P2 18.20 usec
P28 0 usec
PLW1 6.99840021 W
SFO2 213.8118826 MHz
NUC2 13C
CPDPRG[2 garp
P3 12.00 usec
P14 500.00 usec
P24 2000.00 usec
PCPD2 50.00 usec
PLW0 0 W
PLW2 130.00000000 W
PLW12 7.48799992 W
SPNAM[3] Crp80,0.5,20.1
SPOAL3 0.500
SPOFFS3 0 Hz
SPW3 38.13600159 W
SPNAM[7] Crp80comp.4
SPOAL7 0.500
SPOFFS7 0 Hz
SPW7 38.13600159 W
GPNAM[1] SMSQ10.100
GPZ1 80.00 %
GPNAM[2] SMSQ10.100
GPZ2 20.10 %
GPNAM[3] SMSQ10.100
GPZ3 11.00 %
GPNAM[4] SMSQ10.100
GPZ4 -5.00 %
P16 1000.00 usec
P19 600.00 usec

F1 - Acquisition parameters
TD 360
SFO1 213.8119 MHz
FIDRES 262.054504 Hz
SW 220.614 ppm
FnMODE Echo-Antiecho

F2 - Processing parameters
SI 1024
SF 850.2299217 MHz
WDW QSINE
SSB 2
LB 0 Hz
GB 0
PC 1.40

F1 - Processing parameters
SI 1024
MC2 echo-antiecho
SF 213.7902965 MHz
WDW QSINE
SSB 2
LB 0 Hz

YYS-639-20230615 HMBC

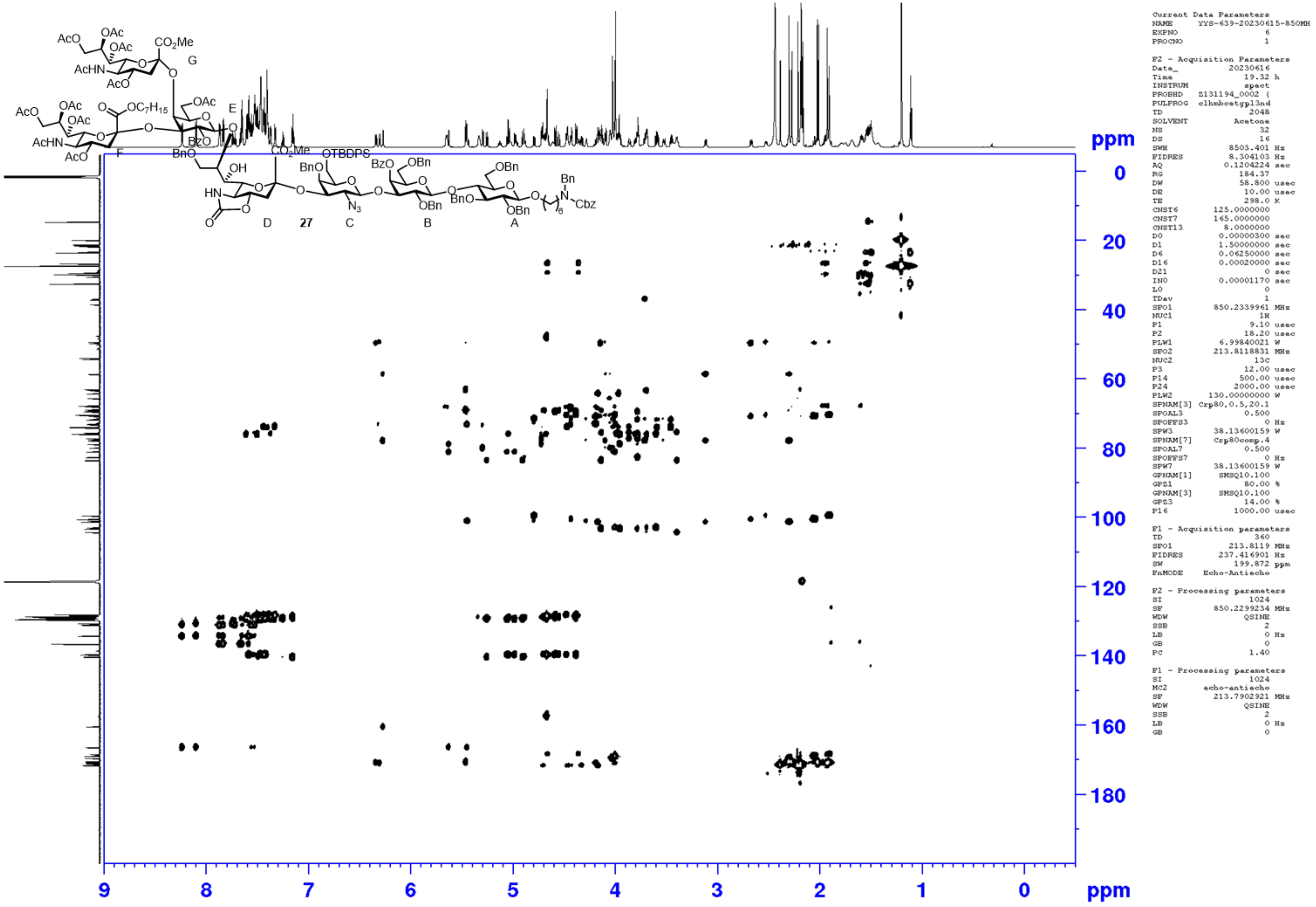

YYS-639-20230615 HSQC-TOCSY

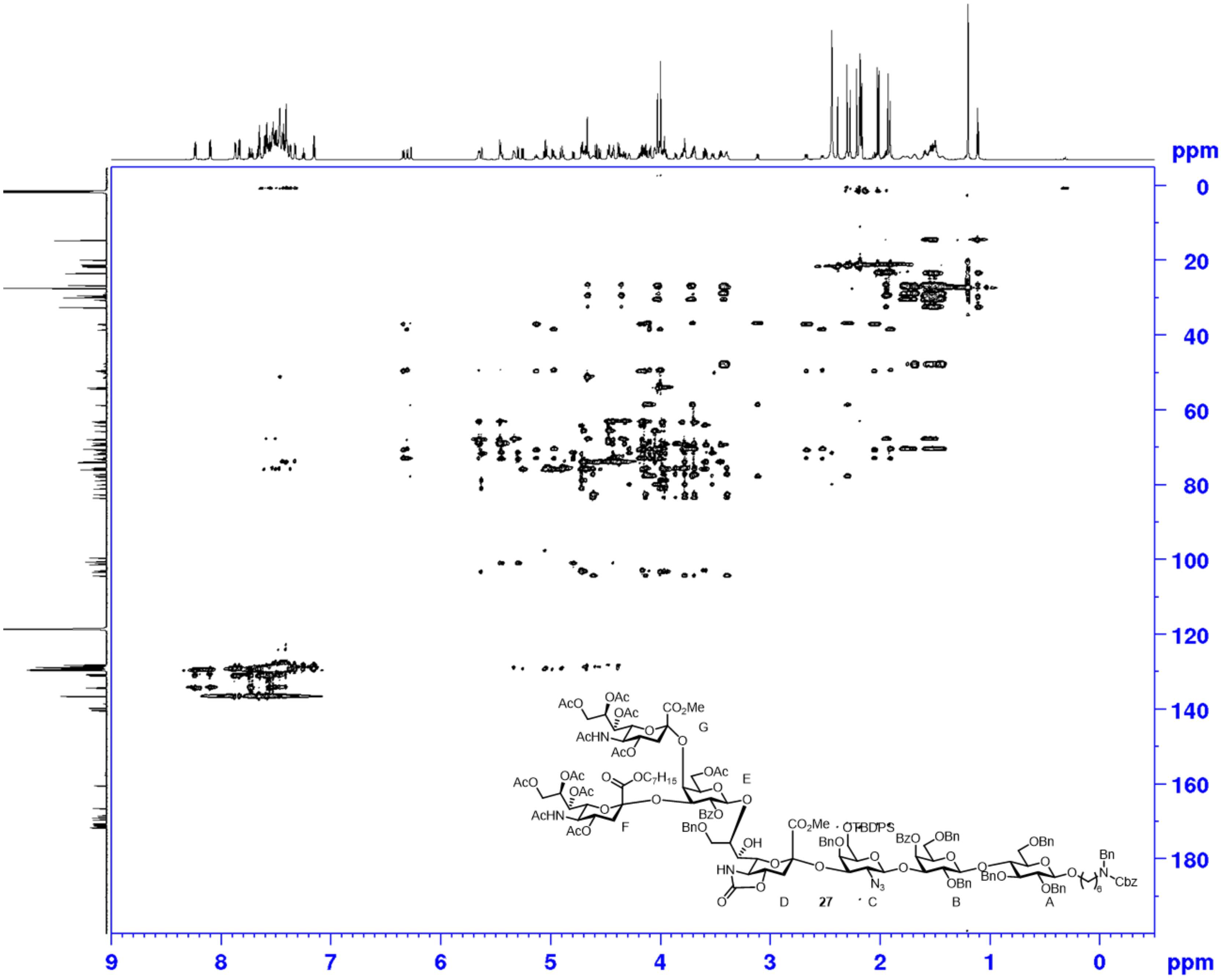

Current Data Parameters
NAME YYS-639-20230615-850MH
EXPNO 4
PROCNO 1

F2 - Acquisition Parameters
Date_ 20230616
Time 11.10 h
INSTRUM spect
PROBHD Z131194_0002 (
PULPROG hsqcdietgpsisp.2
TD 2048
SOLVENT Acetone
NS 32
DS 8
SWH 8503.401 Hz
FIDRES 8.304103 Hz
AQ 0.1204224 sec
RG 184.37
DW 58.800 usec
DE 10.00 usec
TE 298.0 K
CNST2 145.0000000
CNST17 -0.5000000
D0 0.00000300 sec
D1 1.20000005 sec
D4 0.00172414 sec
D9 0.06000000 sec
D11 0.03000000 sec
D16 0.00020000 sec
D24 0.00089000 sec
IN0 0.00001110 sec
L1 26
TDav 1
SFO1 850.2340046 MHz
NUC1 1H
P1 9.10 usec
P2 18.20 usec
P6 20.00 usec
P28 0 usec
PLW1 6.99840021 W
PLW10 1.44879997 W
SFO2 213.8118831 MHz
NUC2 13C
CPDPRG[2 garp
P3 12.00 usec
P14 500.00 usec
P24 2000.00 usec
PCPD2 50.00 usec
PLW0 0 W
PLW2 130.00000000 W
PLW12 7.48799992 W
SPNAM[3] Crp80,0.5,20.1
SPOAL3 0.500
SPOFFS3 0 Hz
SPW3 38.13600159 W
SPNAM[7] Crp80comp.4
SPOAL7 0.500
SPOFFS7 0 Hz
SPW7 38.13600159 W
GPNAM[1] SMSQ10.100
GPZ1 80.00 %
GPNAM[2] SMSQ10.100
GPZ2 20.10 %
P16 1000.00 usec

F1 - Acquisition parameters
TD 360
SFO1 213.8119 MHz
FIDRES 250.250244 Hz
SW 210.676 ppm
FnMODE Echo-Antiecho

F2 - Processing parameters
SI 1024
SF 850.2299260 MHz
WDW QSINE
SSB 2
LB 0 Hz
GB 0
PC 1.40

F1 - Processing parameters
SI 1024
MC2 echo-antiecho
SF 213.7902935 MHz
WDW QSINE
SSB 2
LB 0 Hz
GB 0

YYS-653-3-20230816

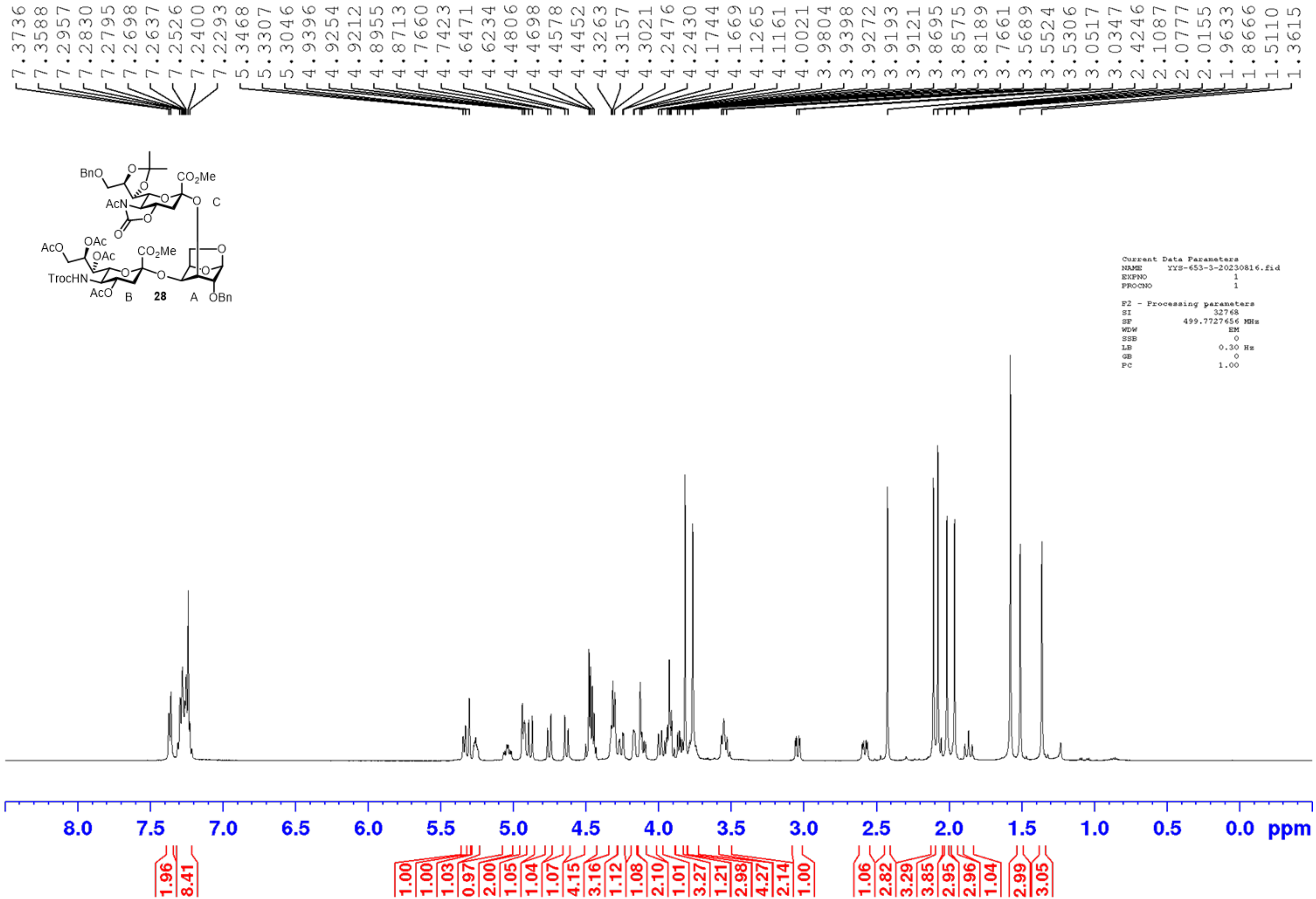

YYS-653-1-20230816-C

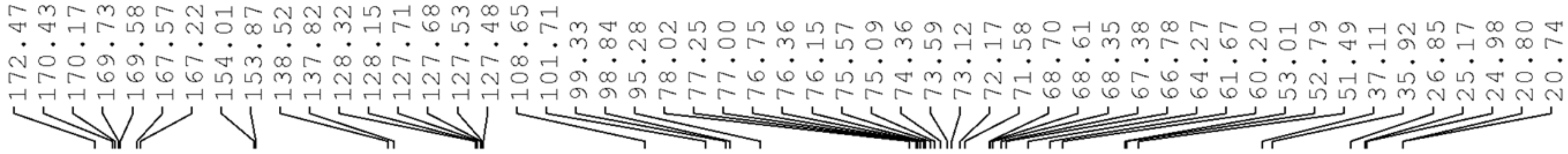

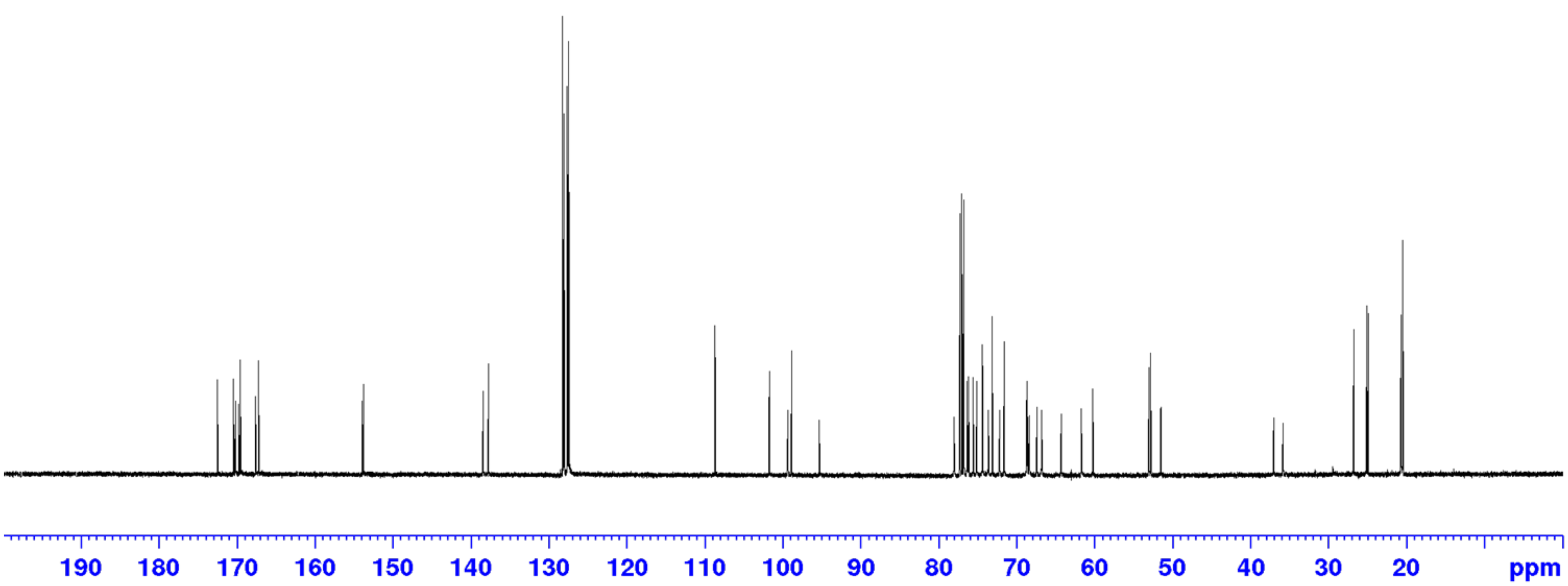

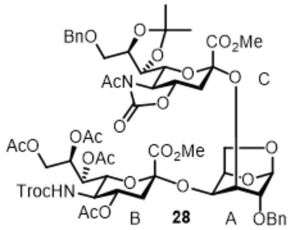

Current Data Parameters
NAME YYS-653-1-20230816-C.fid
EXPNO 1
PROCNO 1

F2 - Processing parameters
SI 65536
SF 125.6679723 MHz
WDW EM
SSB 0
LB 0.30 Hz
GB 0
PC 1.00

YYS-643-20230627 HMBC

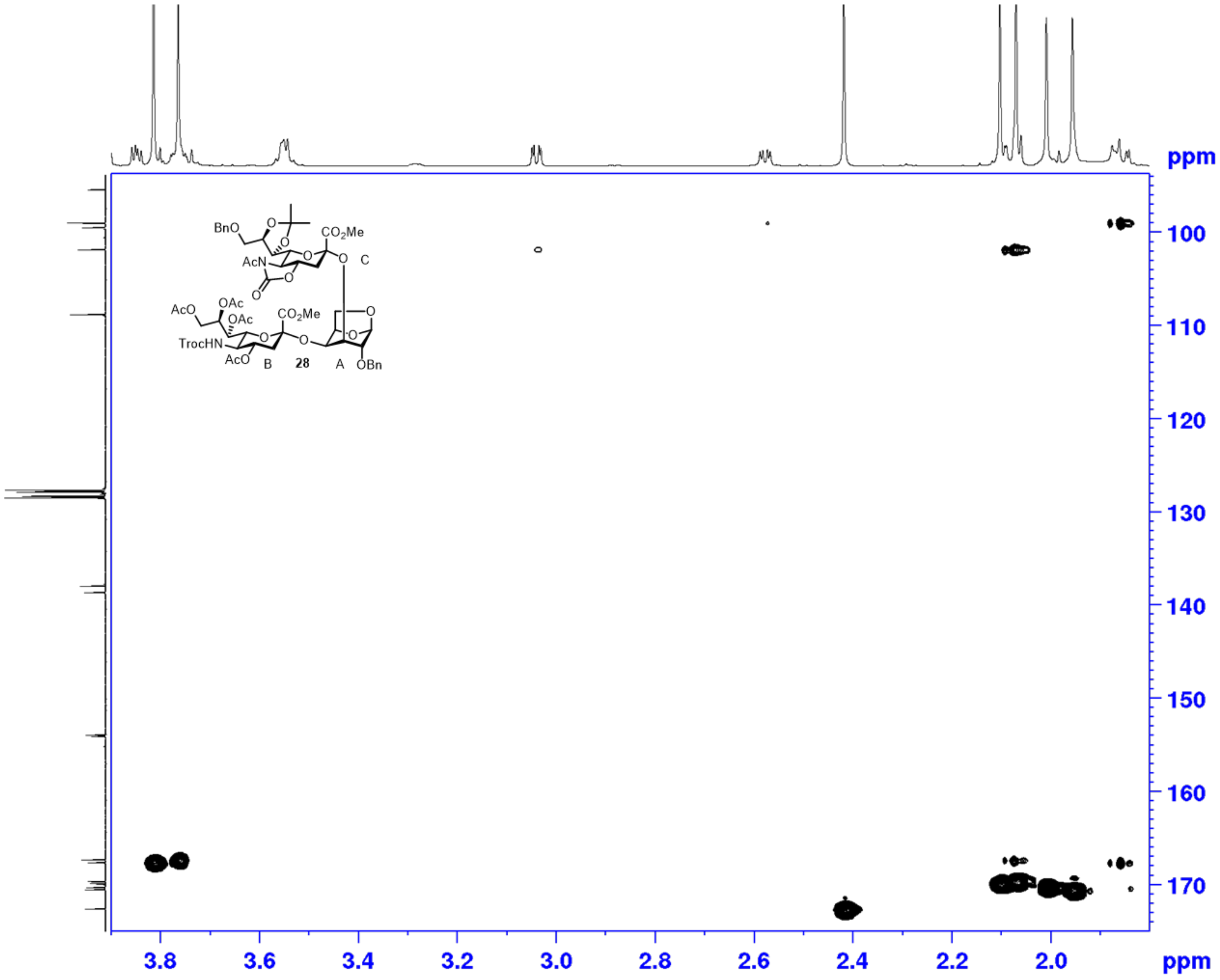


```
Current Data Parameters
NAME      YYS-643-20230627-850MHz-dec
EXPNO                     4
PROCNO                    1

F2 - Acquisition Parameters
Date_          20230629
Time               9.25 h
INSTRUM           spect
PROBHD    Z131194_0002 (
PULPROG   clhmbcetgpl3nd
TD                 2048
SOLVENT           CDCl3
NS                   32
DS                   16
SWH            8503.401 Hz
FIDRES         8.304103 Hz
AQ            0.1204224 sec
RG               184.37
DW               58.800 usec
DE                10.00 usec
TE                298.0 K
CNST6       125.0000000
CNST7       165.0000000
CNST13        8.0000000
D0           0.00000300 sec
D1           1.50000000 sec
D6           0.06250000 sec
D16          0.00020000 sec
D21                   0 sec
IN0          0.00001170 sec
L0                    0
TDav                  1
SFO1        850.2339961 MHz
NUC1                 1H
P1                 8.95 usec
P2                17.90 usec
PLW1         6.99840021 W
SFO2        213.8118831 MHz
NUC2                13C
P3                12.00 usec
P14              500.00 usec
P24             2000.00 usec
PLW2       130.00000000 W
SPNAM[3]  Crp80,0.5,20.1
SPOAL3            0.500
SPOFFS3               0 Hz
SPW3        38.13600159 W
SPNAM[7]     Crp80comp.4
SPOAL7            0.500
SPOFFS7               0 Hz
SPW7        38.13600159 W
GPNAM[1]     SMSQ10.100
GPZ1              80.00 %
GPNAM[3]     SMSQ10.100
GPZ3              14.00 %
P16             1000.00 usec

F1 - Acquisition parameters
TD                  360
SFO1           213.8119 MHz
FIDRES       237.416901 Hz
SW              199.872 ppm
FnMODE    Echo-Antiecho

F2 - Processing parameters
SI                 1024
SF          850.2300381 MHz
WDW               QSINE
SSB                   2
LB                    0 Hz
GB                    0
PC                 1.40

F1 - Processing parameters
SI                 1024
MC2      echo-antiecho
SF          213.7905140 MHz
WDW               QSINE
SSB                   2
LB                    0 Hz
GB                    0
```

YYS-643-20230627 COSY

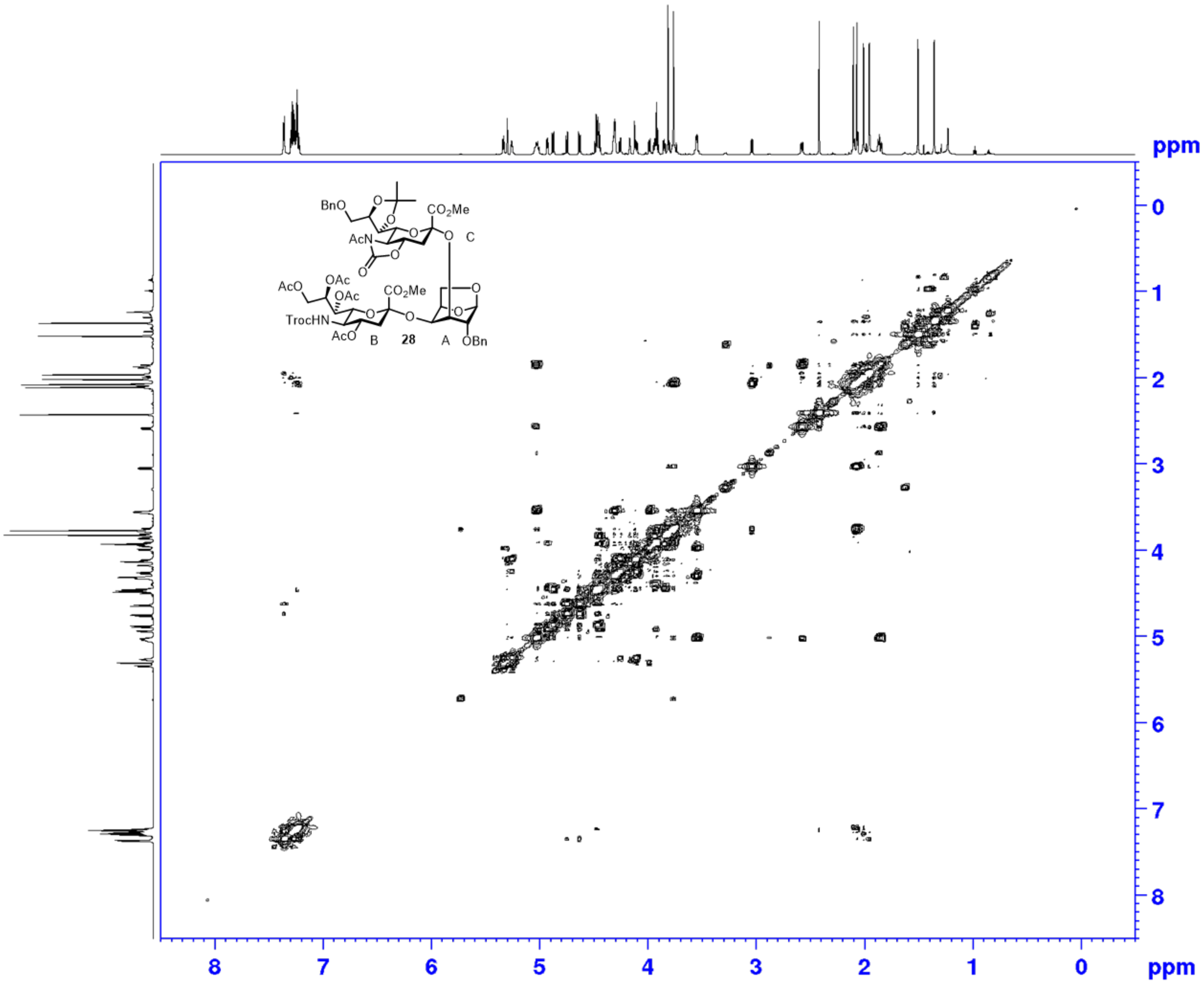

Current Data Parameters	
NAME	YYS-643-20230627-850MHz-deco
EXPNO	3
PROCNO	1
F2 - Acquisition Parameters	
Date_	20230629
Time	22.41 h
INSTRUM	spect
PROBHD	Z131194_0002 (
PULPROG	cosyqf90
TD	2048
SOLVENT	CDCl3
NS	8
DS	0
SWH	8503.401 Hz
FIDRES	8.304103 Hz
AQ	0.1204224 sec
RG	4.98
DW	58.800 usec
DE	10.00 usec
TE	298.0 K
D0	0.00000300 sec
D1	2.00000000 sec
IN0	0.00011760 sec
TDav	1
SFO1	850.2339961 MHz
NUC1	1H
P1	8.95 usec
PLW1	6.99840021 W
F1 - Acquisition parameters	
TD	256
SFO1	850.234 MHz
FIDRES	66.432823 Hz
SW	10.001 ppm
FnMODE	QF
F2 - Processing parameters	
SI	1024
SF	850.2300393 MHz
WDW	SINE
SSB	0
LB	0 Hz
GB	0
PC	1.40
F1 - Processing parameters	
SI	1024
MC2	QF
SF	850.2300369 MHz
WDW	SINE
SSB	0
LB	0 Hz
GB	0

YYS-643-20230627 HMBC

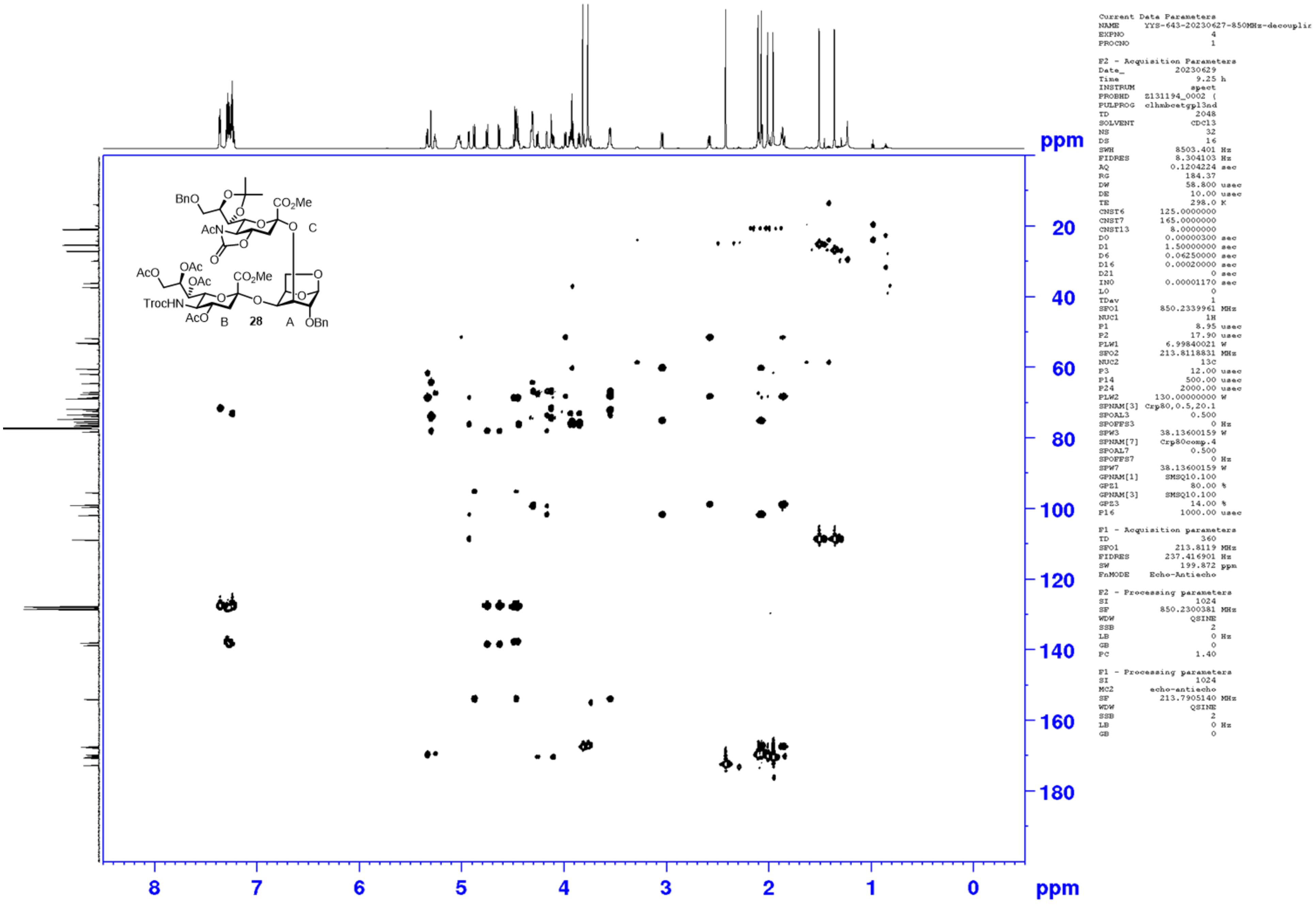

YYS-722-20240122-H1

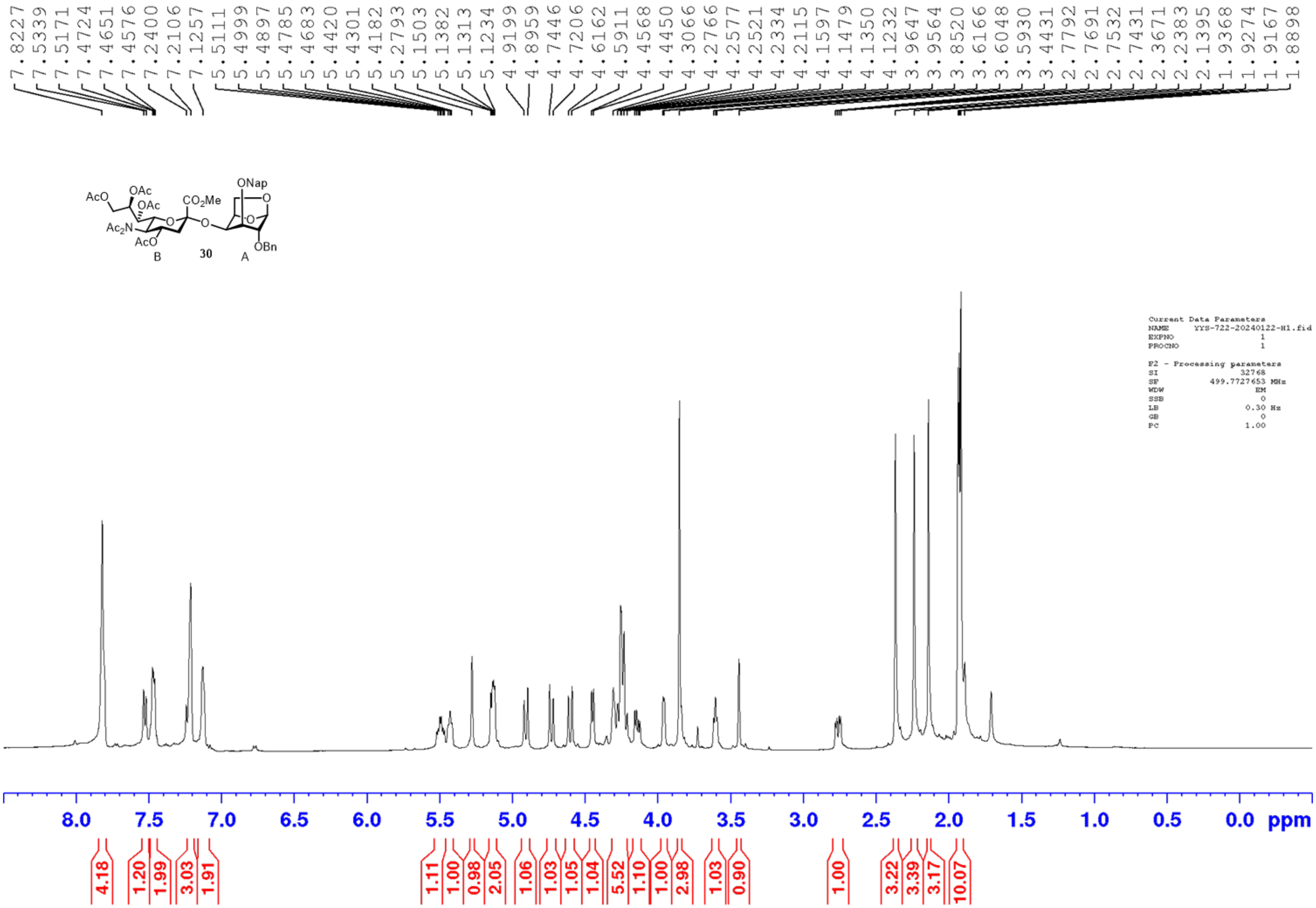

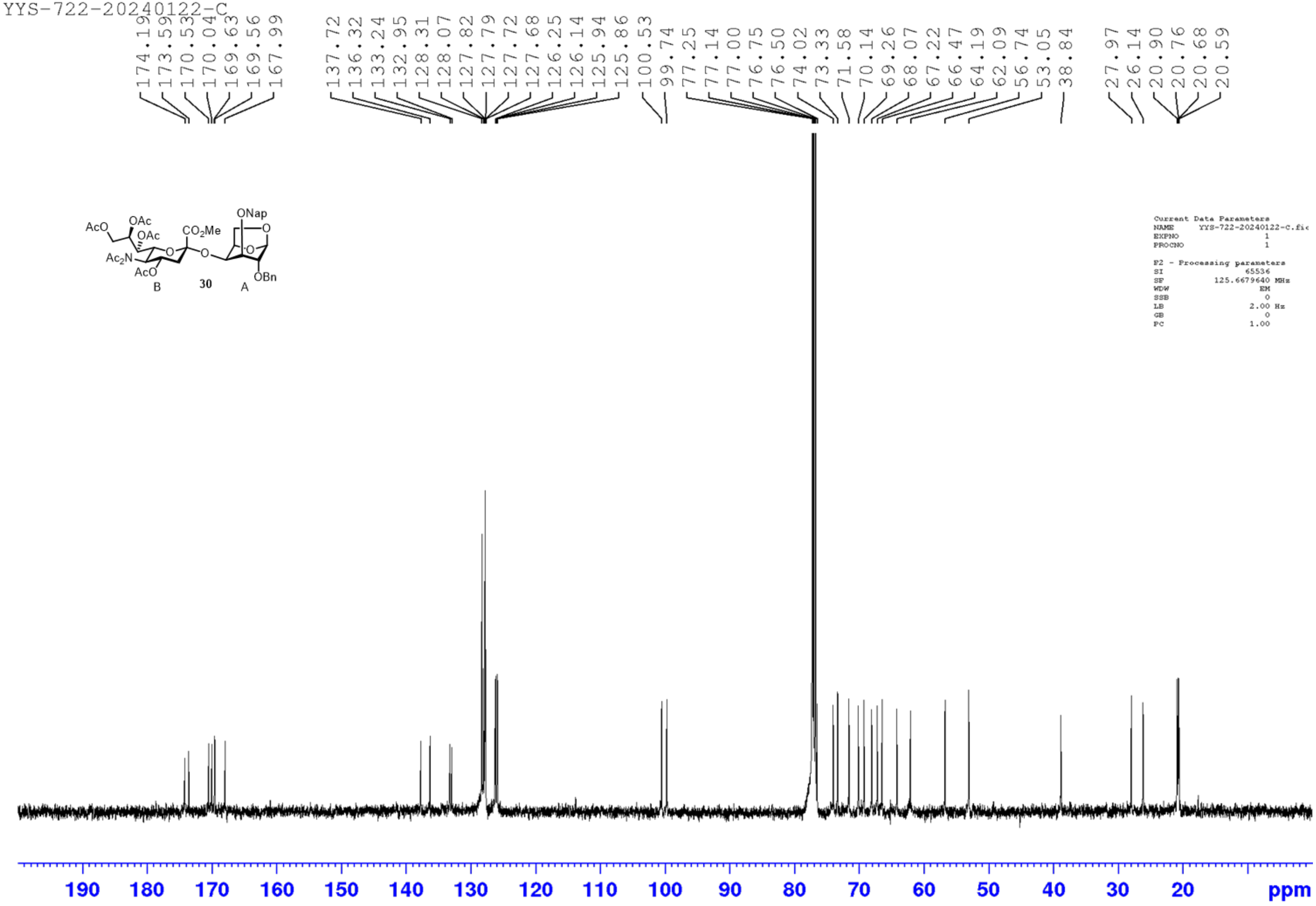
YYS-722-20240122-C
174.19
173.59
170.53
170.04
169.63
169.56
167.99
137.72
136.32
133.24
132.95
128.31
128.07
127.82
127.79
127.72
127.68
126.25
126.14
125.94
125.86
100.53
99.74
77.25
77.14
77.00
76.75
76.50
74.02
73.33
71.58
70.14
69.26
68.07
67.22
66.47
64.19
62.09
56.74
53.05
38.84
27.97
26.14
20.90
20.76
20.68
20.59
AcO
OAc
OAc
CO2Me
ONap
Ac2N
O
O
O
O
AcO
OBn
B
30
A
Current Data Parameters
NAME YYS-722-20240122-C.fid
EXPNO 1
PROCNO 1
F2 - Processing parameters
SI 65536
SF 125.6679640 MHz
WDW EM
SSB 0
LB 2.00 Hz
GB 0
PC 1.00
190
180
170
160
150
140
130
120
110
100
90
80
70
60
50
40
30
20
ppm

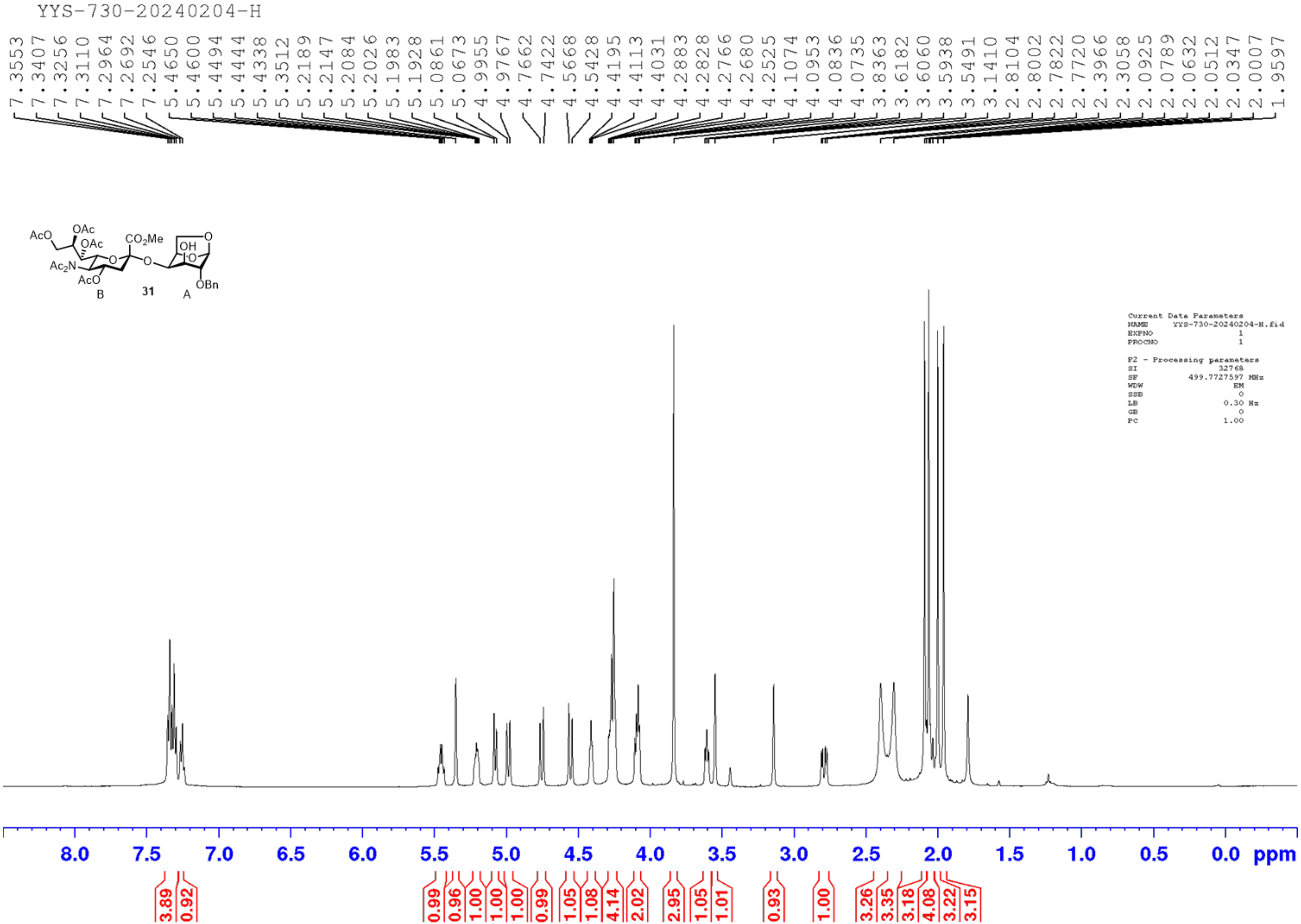
YYS-730-20240204-H
7.3553 7.3407 7.3256 7.3110 7.2964 7.2692 7.2546 5.4650 5.4600 5.4494 5.4444 5.4338 5.3512 5.2189 5.2147 5.2084 5.2026 5.1983 5.1928 5.0861 5.0673 4.9955 4.9767 4.7662 4.7422 4.5668 4.5428 4.4195 4.4113 4.4031 4.2883 4.2828 4.2766 4.2680 4.2525 4.1074 4.0953 4.0836 4.0735 3.8363 3.6182 3.6060 3.5938 3.5491 3.1410 2.8104 2.8002 2.7822 2.7720 2.3966 2.3058 2.0925 2.0789 2.0632 2.0512 2.0347 2.0007 1.9597
AcO
OAc
OAc
CO2Me
O
OH
Ac2N
O
O
O
AcO
OBn
B
31
A
Current Data Parameters
NAME YYS-730-20240204-H.fid
EXPNO 1
PROCNO 1
F2 - Processing parameters
SI 32768
SF 499.7727597 MHz
WDW EM
SSB 0
LB 0.30 Hz
GB 0
PC 1.00
8.0 7.5 7.0 6.5 6.0 5.5 5.0 4.5 4.0 3.5 3.0 2.5 2.0 1.5 1.0 0.5 0.0 ppm
3.89 0.92 0.99 0.96 1.00 1.00 1.00 0.99 1.05 1.08 4.14 2.02 2.95 1.05 1.01 0.93 1.00 3.26 3.35 3.18 4.08 3.22 3.15

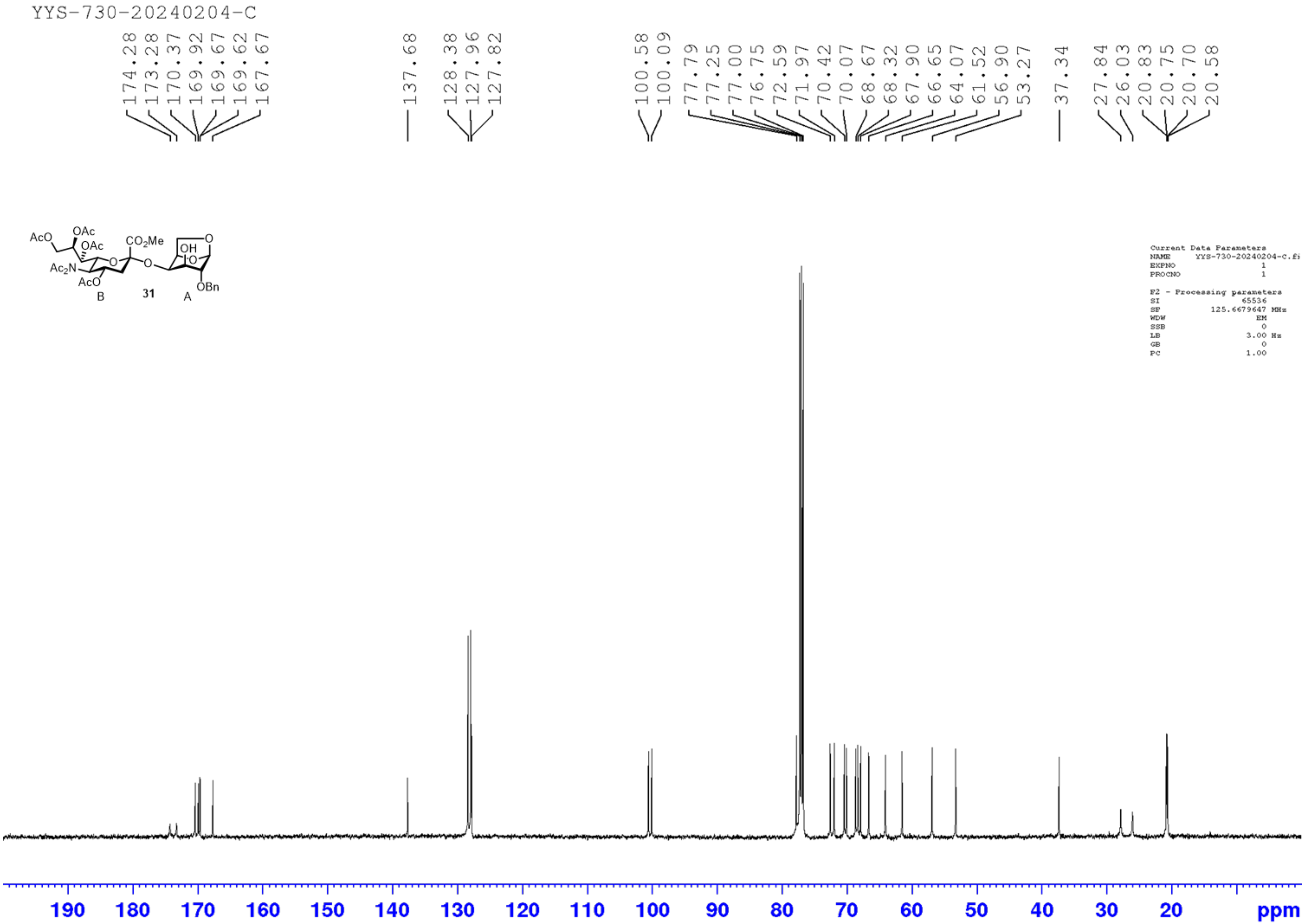
AcO
OAc
OAc
CO_2Me
O
OH
O
Ac_2N
O
O
AcO
OBn
B
31
A
YYS-730-20240204-C
174.28
173.28
170.37
169.92
169.67
169.62
167.67
137.68
128.38
127.96
127.82
100.58
100.09
77.79
77.25
77.00
76.75
72.59
71.97
70.42
70.07
68.67
68.32
67.90
66.65
64.07
61.52
56.90
53.27
37.34
27.84
26.03
20.83
20.75
20.70
20.58
Current Data Parameters
NAME YYS-730-20240204-C.fi
EXPNO 1
PROCNO 1
F2 - Processing parameters
SI 65536
SF 125.6679647 MHz
WDW EM
SSB 0
LB 3.00 Hz
GB 0
PC 1.00
190 180 170 160 150 140 130 120 110 100 90 80 70 60 50 40 30 20 ppm

YYS-725-20230123-H

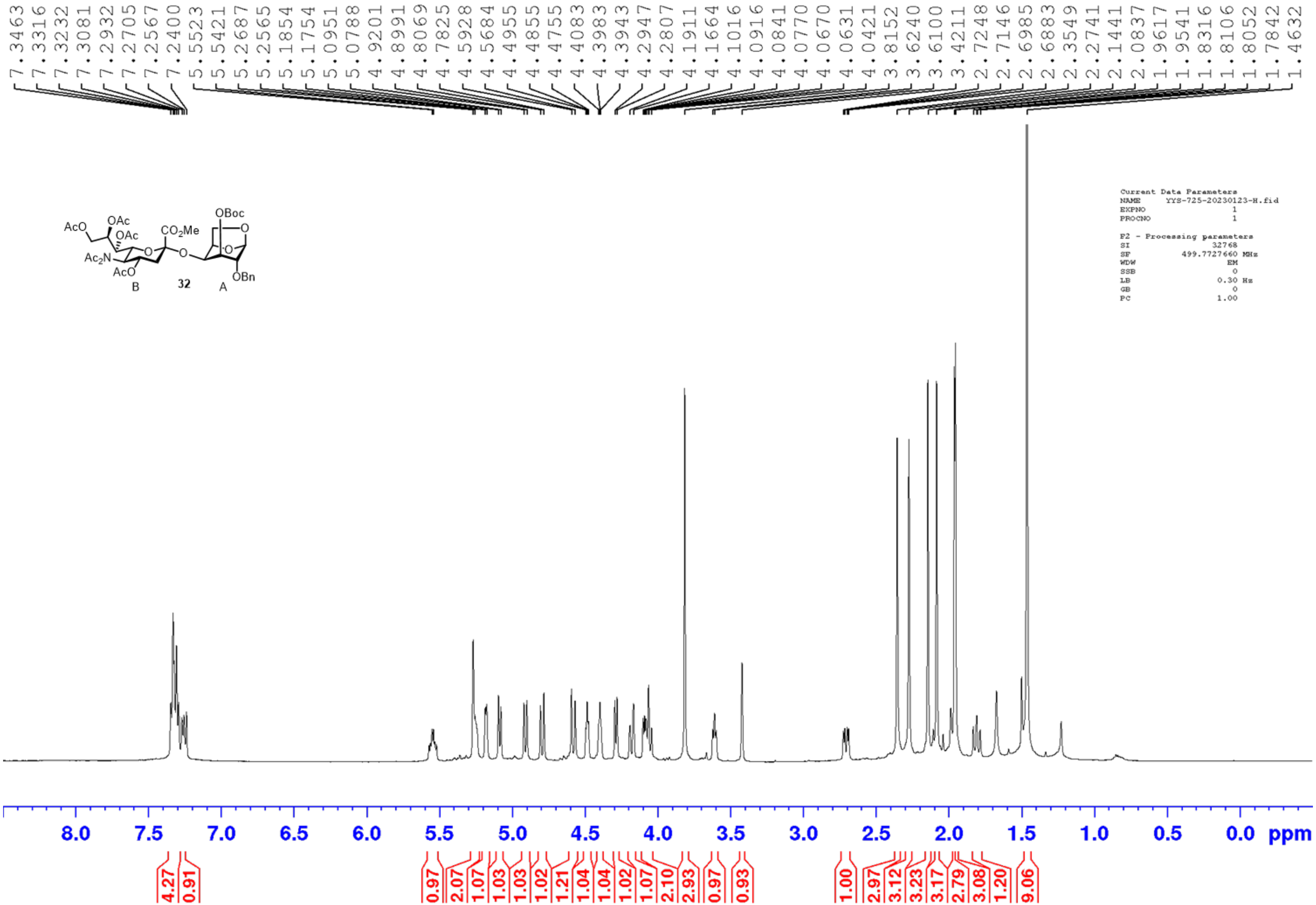

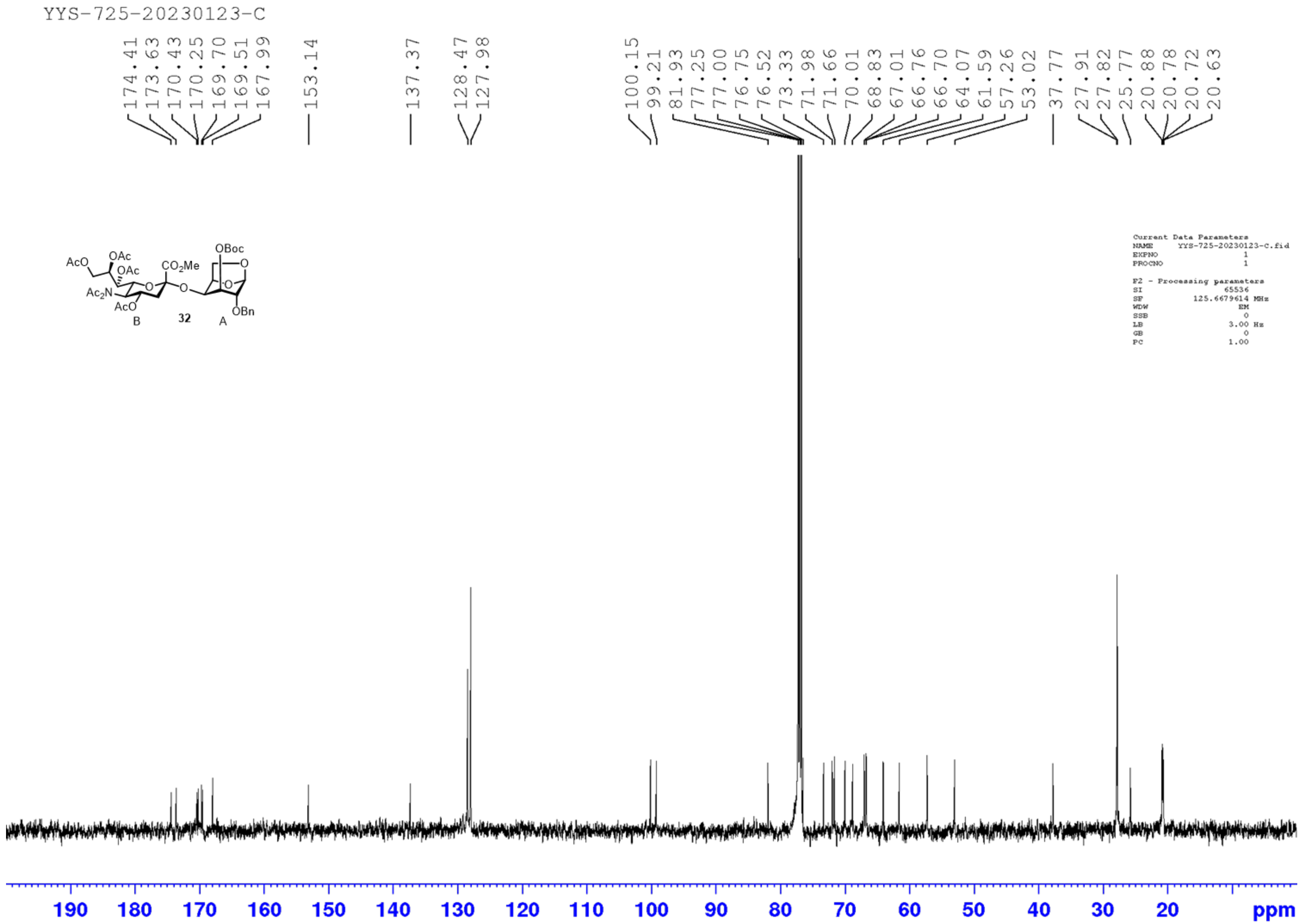
YYS-725-20230123-C
174.41
173.63
170.43
170.25
169.70
169.51
167.99
153.14
137.37
128.47
127.98
100.15
99.21
81.93
77.25
77.00
76.75
76.52
73.33
71.98
71.66
70.01
68.83
67.01
66.76
66.70
64.07
61.59
57.26
53.02
37.77
27.91
27.82
25.77
20.88
20.78
20.72
20.63
AcO
OAc
OAc
CO_2Me
OBoc
Ac_2N
AcO
OBn
B
32
A
Current Data Parameters
NAME YYS-725-20230123-c.fid
EXPNO 1
PROCNO 1
F2 - Processing parameters
SI 65536
SF 125.6679614 MHz
WDW EM
SSB 0
LB 3.00 Hz
GB 0
PC 1.00
190
180
170
160
150
140
130
120
110
100
90
80
70
60
50
40
30
20
ppm

YYS-741-20240317-H

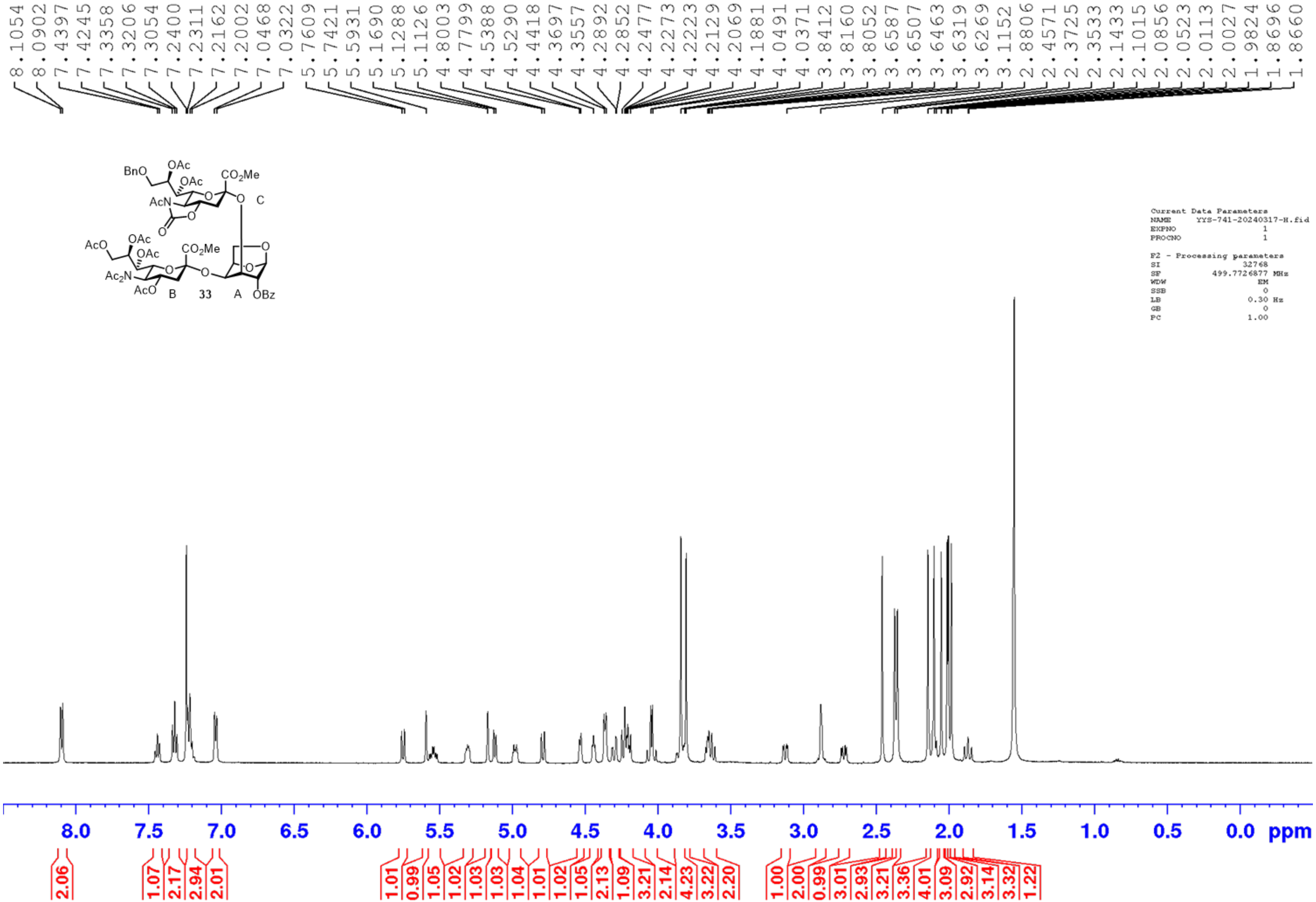

YYS-741-20240317-C

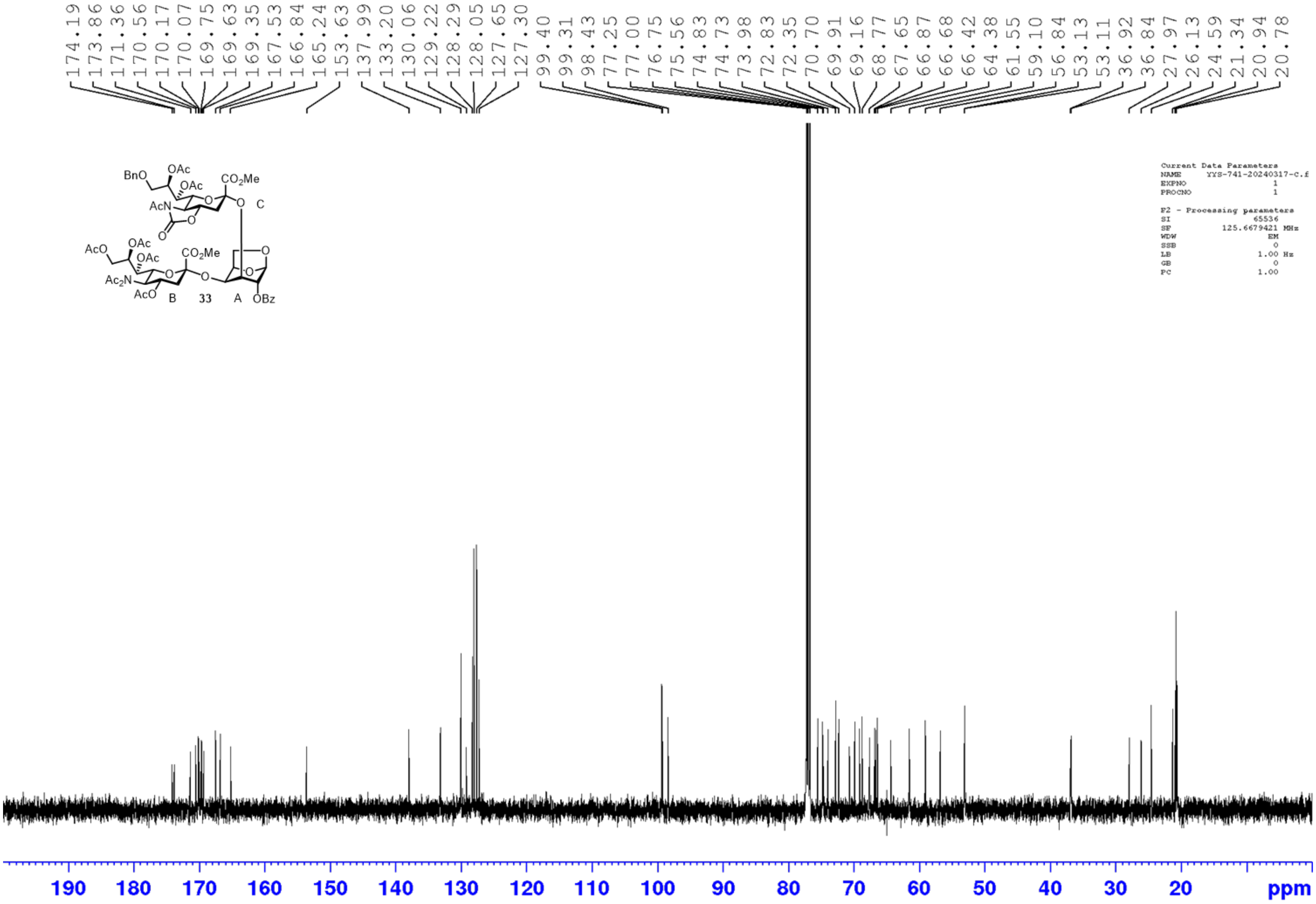

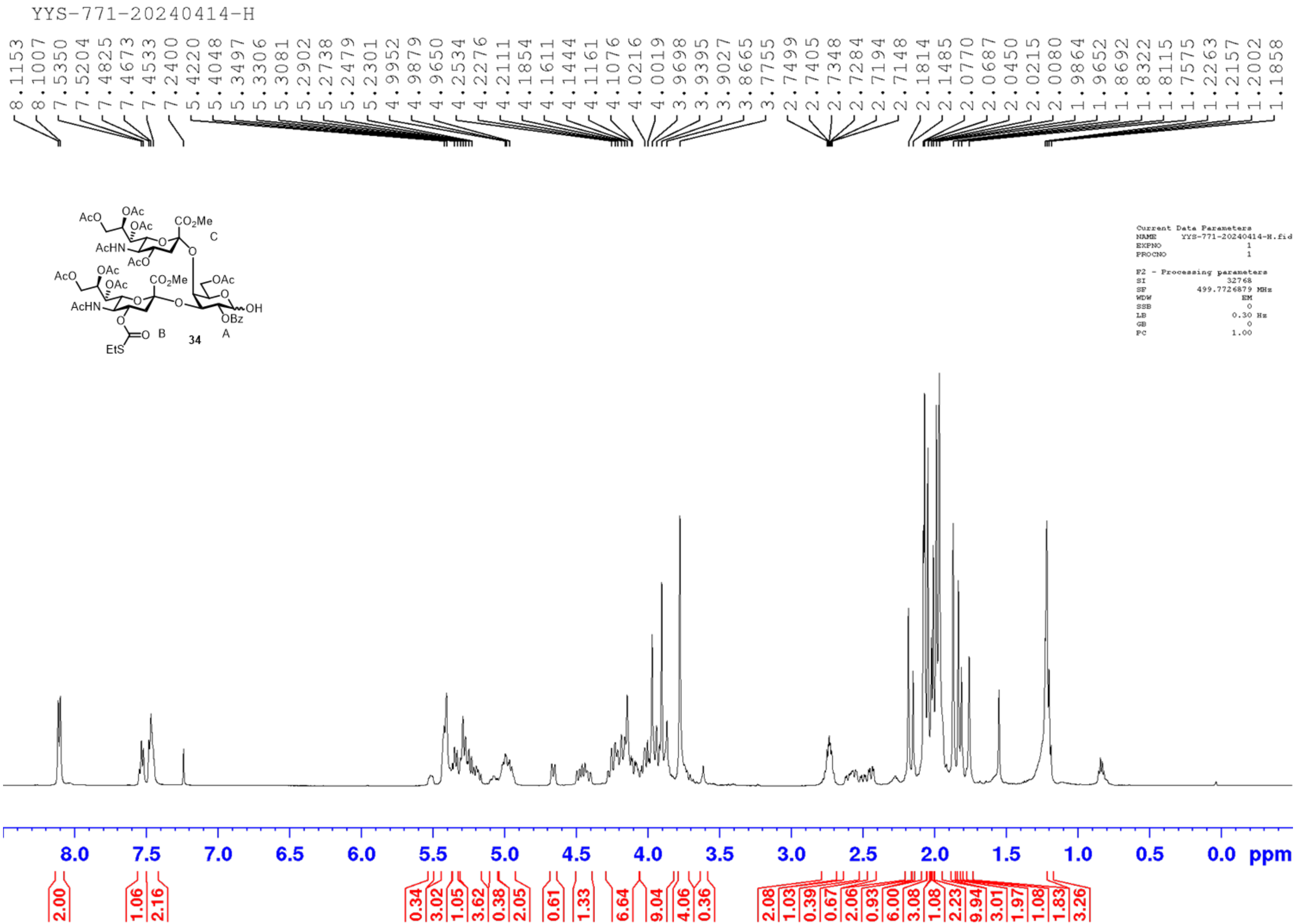
YYS-771-20240414-H
8.1153 8.1007 7.5350 7.5204 7.4825 7.4673 7.4533 7.2400 5.4220 5.4048 5.3497 5.3306 5.3081 5.2902 5.2738 5.2479 5.2301 4.9952 4.9879 4.9650 4.2534 4.2276 4.2111 4.1854 4.1611 4.1444 4.1161 4.1076 4.0216 4.0019 3.9698 3.9395 3.9027 3.8665 3.7755 2.7499 2.7405 2.7348 2.7284 2.7194 2.7148 2.1814 2.1485 2.0770 2.0687 2.0450 2.0215 2.0080 1.9864 1.9652 1.8692 1.8322 1.8115 1.7575 1.2263 1.2157 1.2002 1.1858
AcO
OAc
OAc
CO2Me
C
AcHN
AcO
OAc
CO2Me
OAc
OH
OBz
EtS
B
34
A
Current Data Parameters
NAME YYS-771-20240414-H.fid
EXPNO 1
PROCNO 1
F2 - Processing parameters
SI 32768
SF 499.7726879 MHz
WDW EM
SSB 0
LB 0.30 Hz
GB 0
PC 1.00
8.0 7.5 7.0 6.5 6.0 5.5 5.0 4.5 4.0 3.5 3.0 2.5 2.0 1.5 1.0 0.5 0.0 ppm
2.00 1.06 2.16 0.34 3.02 1.05 3.62 0.38 2.05 0.61 1.33 6.64 9.04 4.06 0.36 2.08 1.03 0.39 0.67 2.06 0.93 6.00 3.08 1.08 2.23 9.94 3.01 1.97 1.08 1.83 3.26

YYS-771-20240414-C

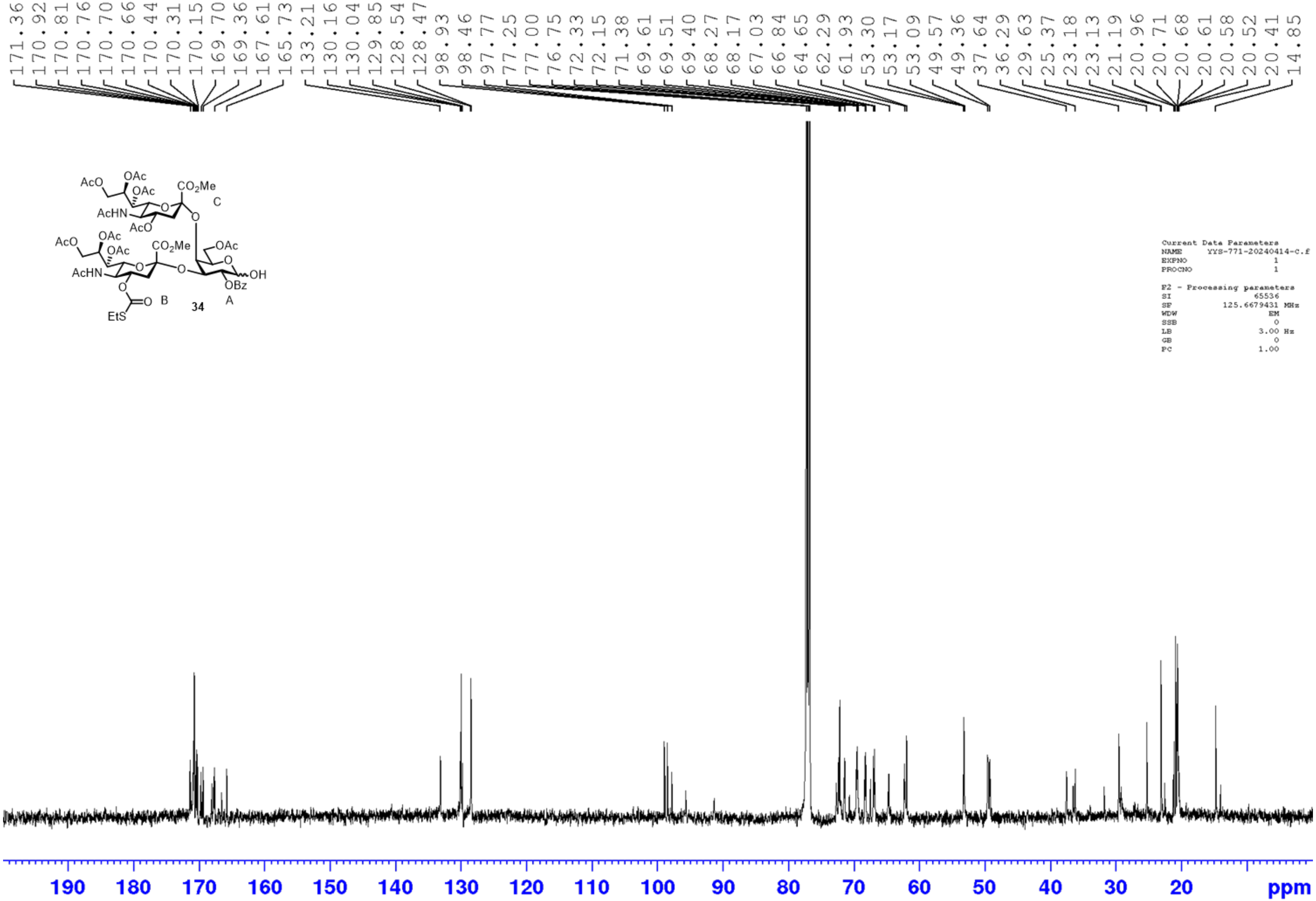

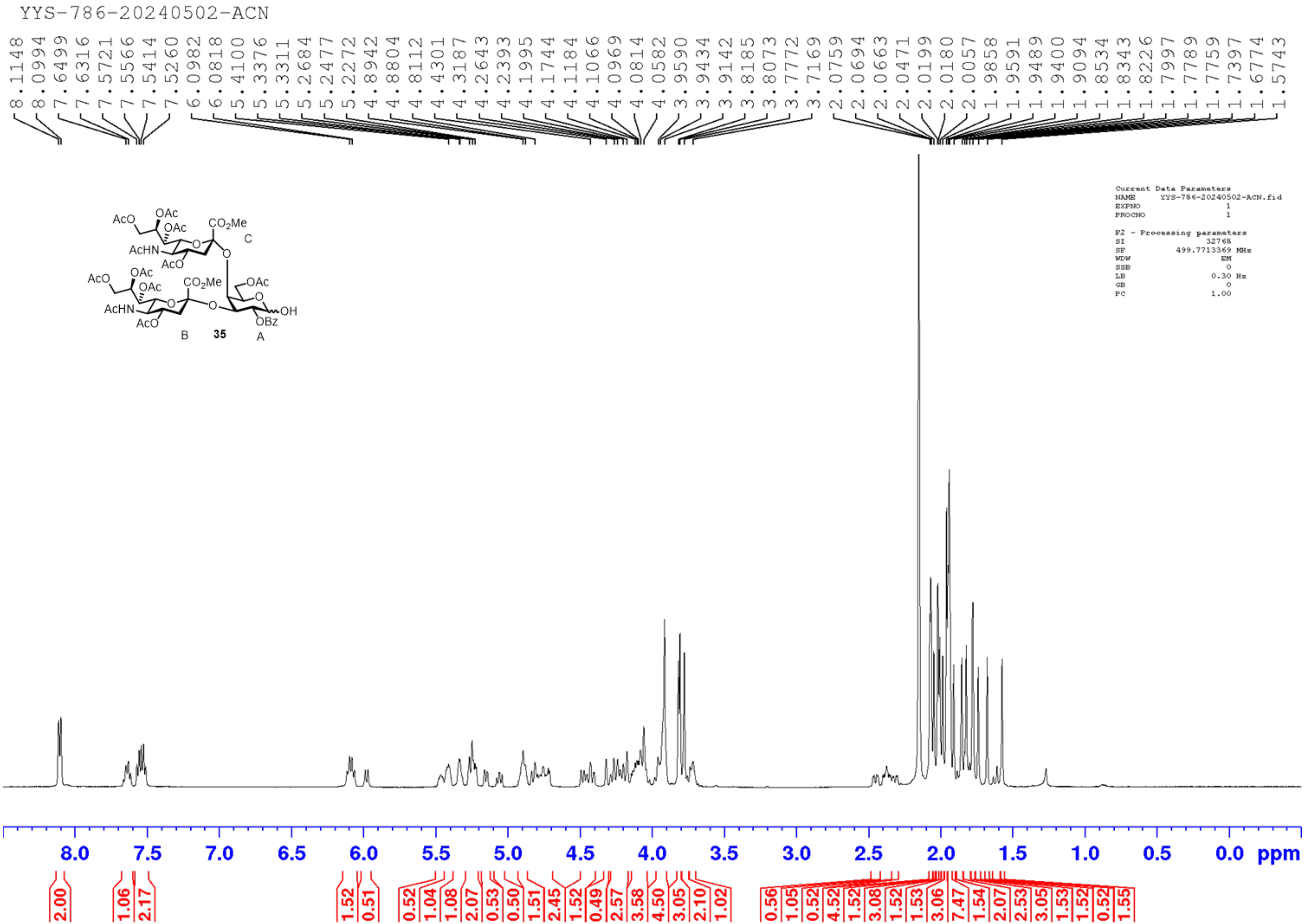
YYS-786-20240502-ACN
8.1148 8.0994 7.6499 7.6316 7.5721 7.5566 7.5414 7.5260 6.0982 6.0818 5.4100 5.3376 5.3311 5.2684 5.2477 5.2272 4.8942 4.8804 4.8112 4.4301 4.3187 4.2643 4.2393 4.1995 4.1744 4.1184 4.1066 4.0969 4.0814 4.0582 3.9590 3.9434 3.9142 3.8185 3.8073 3.7772 3.7169 2.0759 2.0694 2.0663 2.0471 2.0199 2.0180 2.0057 1.9858 1.9591 1.9489 1.9400 1.9094 1.8534 1.8343 1.8226 1.7997 1.7789 1.7759 1.7397 1.6774 1.5743
Current Data Parameters
NAME YYS-786-20240502-ACN.fid
EXPNO 1
PROCNO 1
F2 - Processing parameters
SI 32768
SF 499.7713369 MHz
WDW EM
SSB 0
LB 0.30 Hz
GB 0
PC 1.00
AcO
OAc
OAc
CO2Me
C
AcHN
AcO
O
O
OAc
CO2Me
OAc
AcHN
AcO
OBz
OH
B
35
A
8.0 7.5 7.0 6.5 6.0 5.5 5.0 4.5 4.0 3.5 3.0 2.5 2.0 1.5 1.0 0.5 0.0 ppm
2.00 1.06 2.17 1.52 0.51 0.52 1.04 1.08 2.07 0.53 0.50 1.51 2.45 1.52 0.49 2.57 3.58 4.50 3.05 2.10 1.02 0.56 1.05 0.52 4.52 1.52 3.08 1.52 1.53 3.06 7.47 1.54 2.07 2.53 3.05 1.53 1.52 0.52 1.55

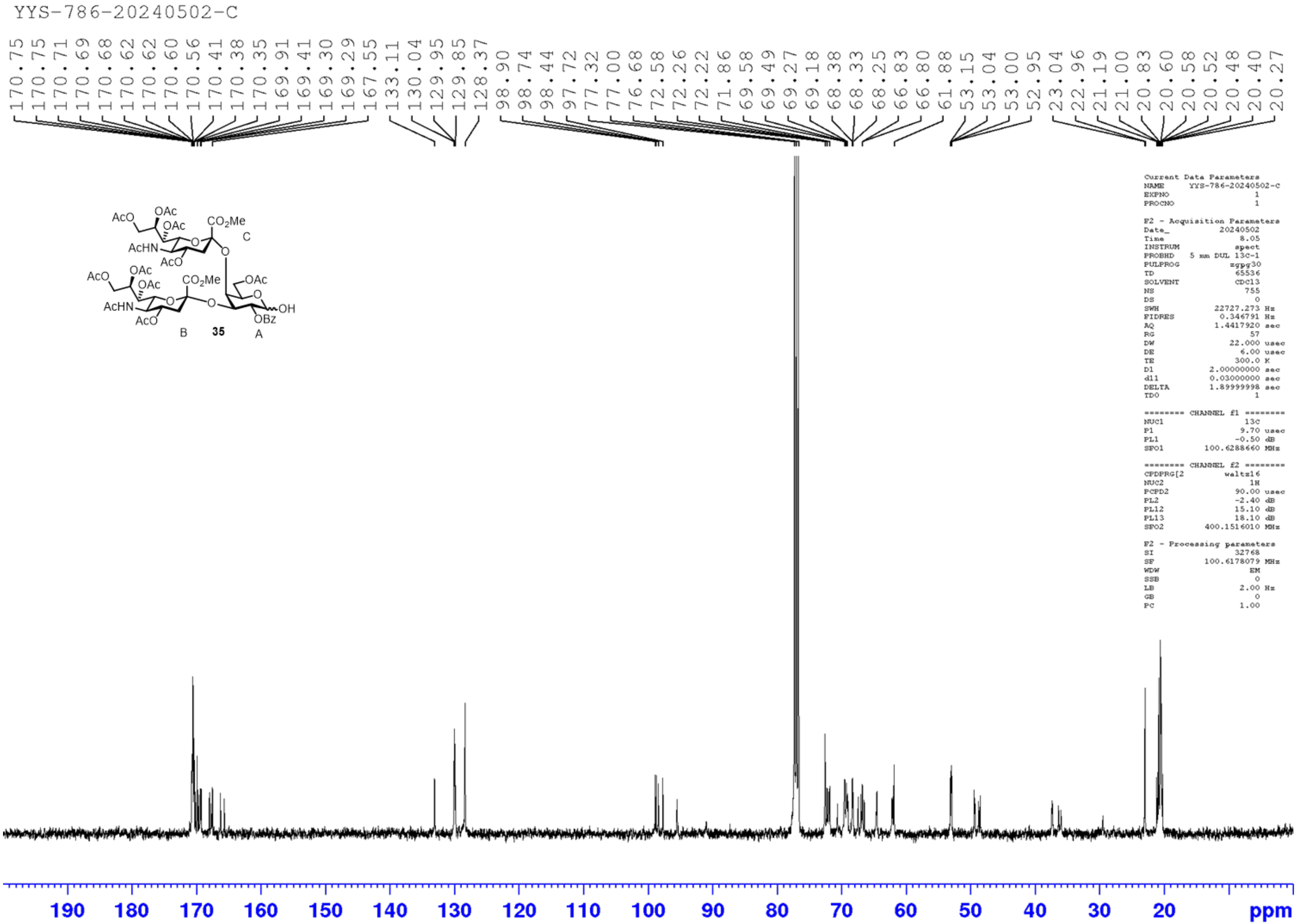
YYS-786-20240502-C
170.75
170.75
170.71
170.69
170.68
170.62
170.62
170.60
170.56
170.41
170.38
170.35
169.91
169.41
169.30
169.29
167.55
133.11
130.04
129.95
129.85
128.37
98.90
98.74
98.44
97.72
77.32
77.00
76.68
72.58
72.26
72.22
71.86
69.58
69.49
69.27
69.18
68.38
68.33
68.25
66.83
66.80
61.88
53.15
53.04
53.00
52.95
23.04
22.96
21.19
21.00
20.83
20.60
20.58
20.52
20.48
20.40
20.27
AcO
OAc
OAc
CO2Me
C
AcHN
AcO
O
OAc
AcO
OAc
CO2Me
OAc
AcHN
AcO
OH
OBz
B
35
A
Current Data Parameters
NAME YYS-786-20240502-C
EXPNO 1
PROCNO 1
F2 - Acquisition Parameters
Date_ 20240502
Time 8.05
INSTRUM spect
PROBHD 5 mm DUL 13C-1
PULPROG zgpg30
TD 65536
SOLVENT CDCl3
NS 755
DS 0
SWH 22727.273 Hz
FIDRES 0.346791 Hz
AQ 1.4417920 sec
RG 57
DW 22.000 usec
DE 6.00 usec
TE 300.0 K
D1 2.00000000 sec
d11 0.03000000 sec
DELTA 1.89999998 sec
TD0 1
======== CHANNEL f1 ========
NUC1 13C
P1 9.70 usec
PL1 -0.50 dB
SFO1 100.6288660 MHz
======== CHANNEL f2 ========
CPDPRG[2 waltz16
NUC2 1H
PCPD2 90.00 usec
PL2 -2.40 dB
PL12 15.10 dB
PL13 18.10 dB
SFO2 400.1516010 MHz
F2 - Processing parameters
SI 32768
SF 100.6178079 MHz
WDW EM
SSB 0
LB 2.00 Hz
GB 0
PC 1.00
190
180
170
160
150
140
130
120
110
100
90
80
70
60
50
40
30
20
ppm

YYS-739-d-20240304-600 MHz-H

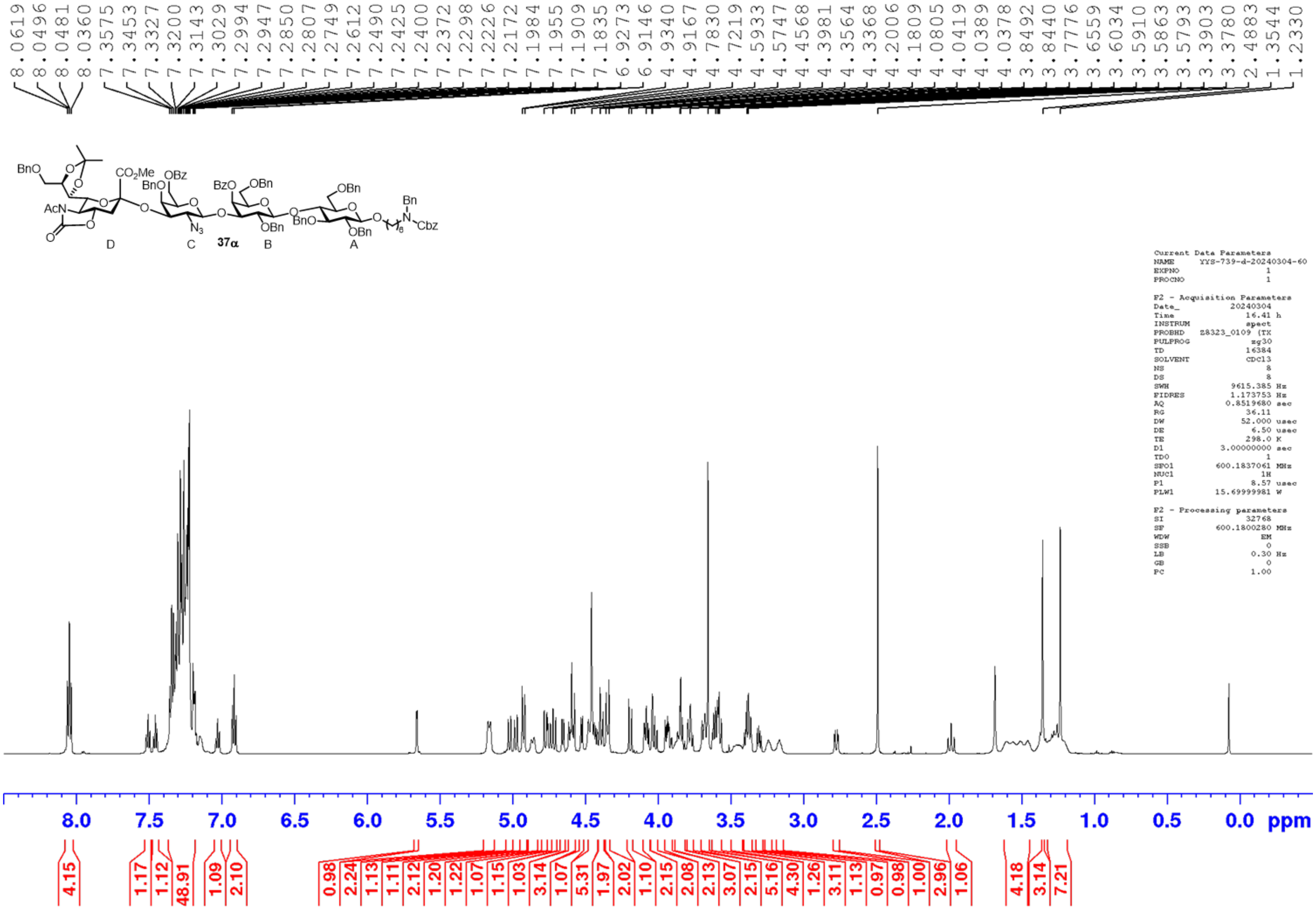

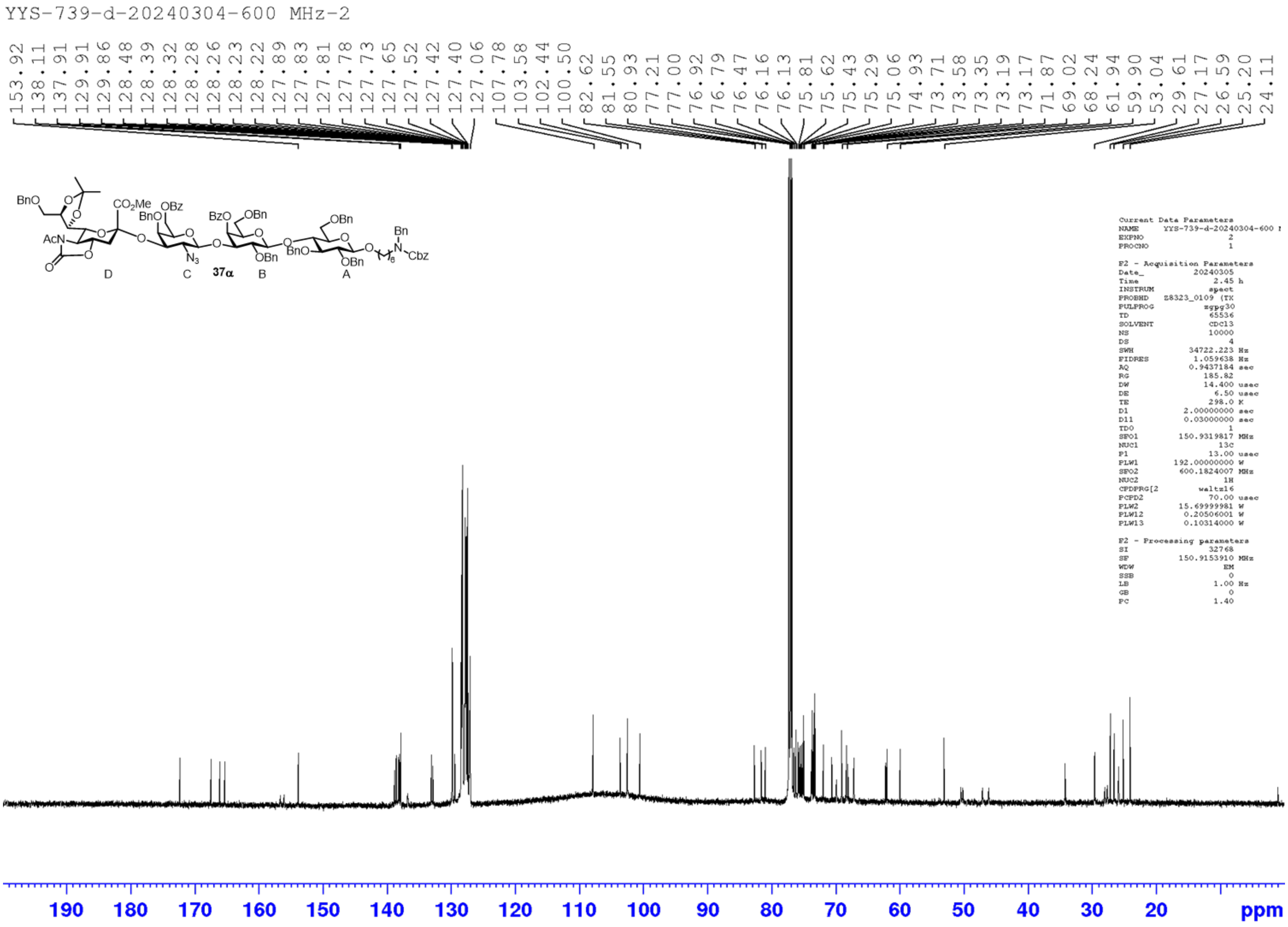

YYS-739-d-20240304-600 MHz-2
153.92
138.11
137.91
129.91
129.86
128.48
128.39
128.32
128.28
128.26
128.23
128.22
127.89
127.83
127.81
127.78
127.73
127.65
127.52
127.42
127.40
127.06
107.78
103.58
102.44
100.50
82.62
81.55
80.93
77.21
77.00
76.92
76.79
76.47
76.16
76.13
75.81
75.62
75.43
75.29
75.06
74.93
73.71
73.58
73.35
73.19
73.17
71.87
69.02
68.24
61.94
59.90
53.04
29.61
27.17
26.59
25.20
24.11
BnO
CO2Me
OBz
BnO
BzO
OBn
OBn
AcN
O
O
O
O
O
O
O
O
Bn
N
Cbz
N3
OBn
BnO
OBn
6
D
C
37α
B
A
Current Data Parameters
NAME YYS-739-d-20240304-600 M
EXPNO 2
PROCNO 1
F2 - Acquisition Parameters
Date_ 20240305
Time 2.45 h
INSTRUM spect
PROBHD Z8323_0109 (TX
PULPROG zgpg30
TD 65536
SOLVENT CDCl3
NS 10000
DS 4
SWH 34722.223 Hz
FIDRES 1.059638 Hz
AQ 0.9437184 sec
RG 185.82
DW 14.400 usec
DE 6.50 usec
TE 298.0 K
D1 2.00000000 sec
D11 0.03000000 sec
TD0 1
SFO1 150.9319817 MHz
NUC1 13C
P1 13.00 usec
PLW1 192.00000000 W
SFO2 600.1824007 MHz
NUC2 1H
CPDPRG[2 waltz16
PCPD2 70.00 usec
PLW2 15.69999981 W
PLW12 0.20506001 W
PLW13 0.10314000 W
F2 - Processing parameters
SI 32768
SF 150.9153910 MHz
WDW EM
SSB 0
LB 1.00 Hz
GB 0
PC 1.40
190
180
170
160
150
140
130
120
110
100
90
80
70
60
50
40
30
20
ppm

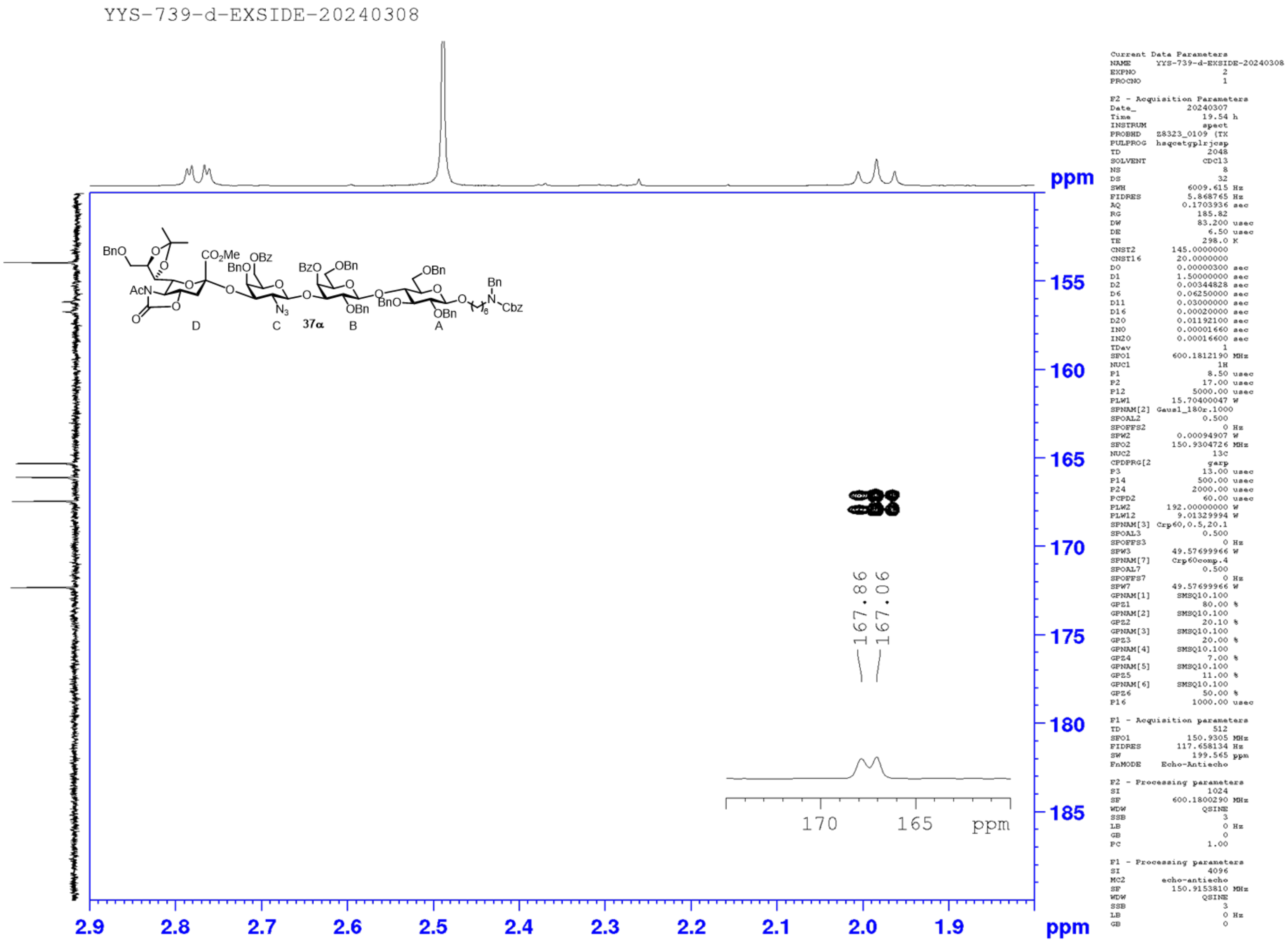
YYS-739-d-EXSIDE-20240308
BnO
CO2Me
OBz
BnO
BzO
OBn
OBn
Bn
AcN
N3
OBn
BnO
OBn
N
Cbz
D
C
37α
B
A
167.86
167.06
170
165
ppm
155
160
165
170
175
180
185
ppm
2.9
2.8
2.7
2.6
2.5
2.4
2.3
2.2
2.1
2.0
1.9
ppm
Current Data Parameters
NAME YYS-739-d-EXSIDE-20240308
EXPNO 2
PROCNO 1
F2 - Acquisition Parameters
Date_ 20240307
Time 19.54 h
INSTRUM spect
PROBHD Z8323_0109 (TX
PULPROG hsqcetgplrjcsp
TD 2048
SOLVENT CDCl3
NS 8
DS 32
SWH 6009.615 Hz
FIDRES 5.868765 Hz
AQ 0.1703936 sec
RG 185.82
DW 83.200 usec
DE 6.50 usec
TE 298.0 K
CNST2 145.0000000
CNST16 20.0000000
D0 0.00000300 sec
D1 1.50000000 sec
D2 0.00344828 sec
D6 0.06250000 sec
D11 0.03000000 sec
D16 0.00020000 sec
D20 0.01192100 sec
IN0 0.00001660 sec
IN20 0.00016600 sec
TDav 1
SFO1 600.1812190 MHz
NUC1 1H
P1 8.50 usec
P2 17.00 usec
P12 5000.00 usec
PLW1 15.70400047 W
SPNAM[2] Gaus1_180r.1000
SPOAL2 0.500
SPOFFS2 0 Hz
SPW2 0.00094907 W
SFO2 150.9304726 MHz
NUC2 13C
CPDPRG[2 garp
P3 13.00 usec
P14 500.00 usec
P24 2000.00 usec
PCPD2 60.00 usec
PLW2 192.00000000 W
PLW12 9.01329994 W
SPNAM[3] Crp60,0.5,20.1
SPOAL3 0.500
SPOFFS3 0 Hz
SPW3 49.57699966 W
SPNAM[7] Crp60comp.4
SPOAL7 0.500
SPOFFS7 0 Hz
SPW7 49.57699966 W
GPNAM[1] SMSQ10.100
GPZ1 80.00 %
GPNAM[2] SMSQ10.100
GPZ2 20.10 %
GPNAM[3] SMSQ10.100
GPZ3 20.00 %
GPNAM[4] SMSQ10.100
GPZ4 7.00 %
GPNAM[5] SMSQ10.100
GPZ5 11.00 %
GPNAM[6] SMSQ10.100
GPZ6 50.00 %
P16 1000.00 usec
F1 - Acquisition parameters
TD 512
SFO1 150.9305 MHz
FIDRES 117.658134 Hz
SW 199.565 ppm
FnMODE Echo-Antiecho
F2 - Processing parameters
SI 1024
SF 600.1800290 MHz
WDW QSINE
SSB 3
LB 0 Hz
GB 0
PC 1.00
F1 - Processing parameters
SI 4096
MC2 echo-antiecho
SF 150.9153810 MHz
WDW QSINE
SSB 3
LB 0 Hz
GB 0

YYS-739-d-20240304-600 MHz-HMBC

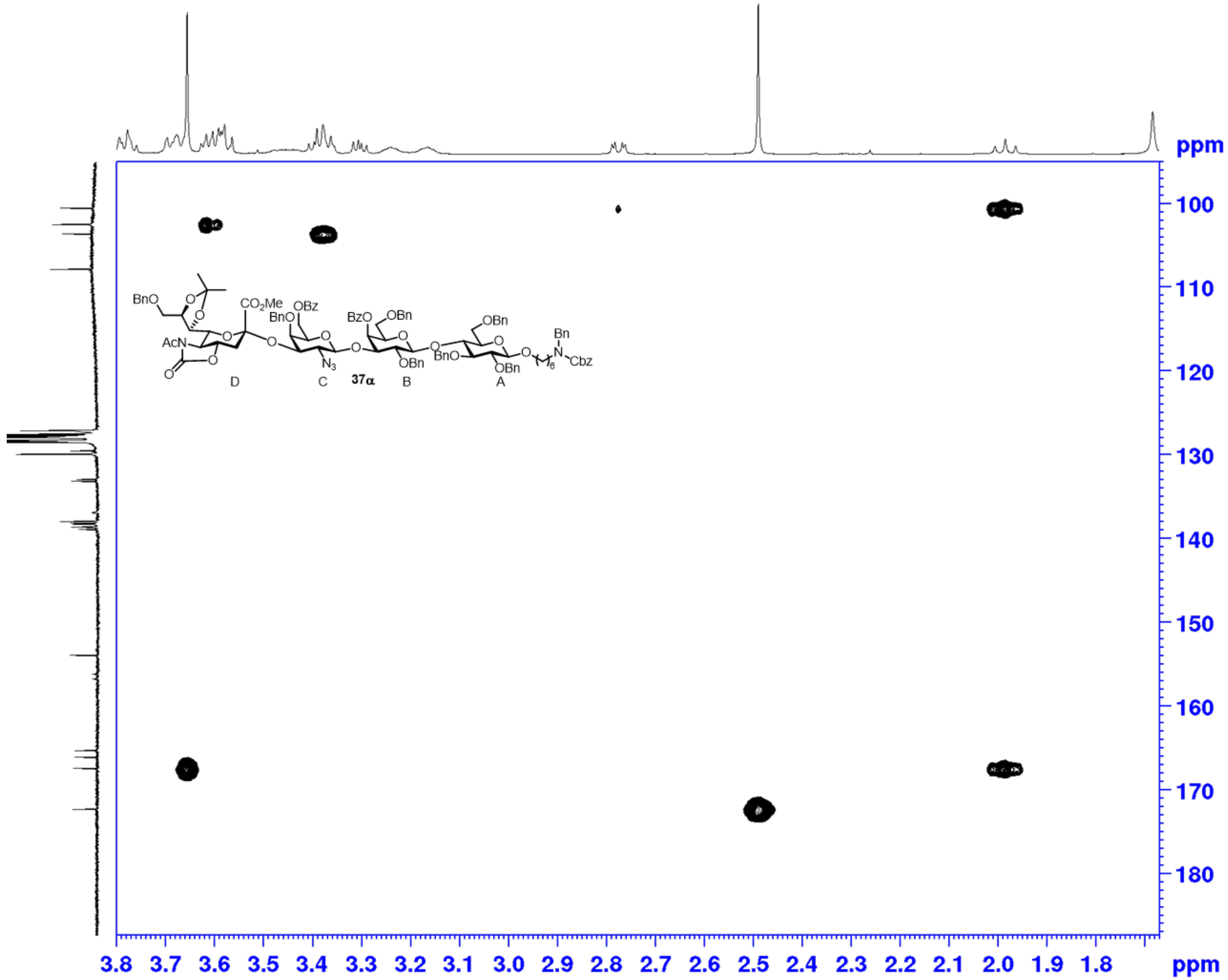

Current Data Parameters
NAME YYS-739-d-20240304-600 MH
EXPNO 3
PROCNO 1

F2 - Acquisition Parameters
Date_ 20240304
Time 17.26 h
INSTRUM spect
PROBHD Z8323_0109 (TX
PULPROG clhmbcetgpl3nd
TD 2048
SOLVENT CDCl3
NS 8
DS 16
SWH 6009.615 Hz
FIDRES 5.868765 Hz
AQ 0.1703936 sec
RG 185.82
DW 83.200 usec
DE 6.50 usec
TE 298.0 K
CNST6 125.0000000
CNST7 165.0000000
CNST13 8.0000000
D0 0.00000300 sec
D1 1.50000000 sec
D6 0.06250000 sec
D16 0.00020000 sec
D21 0 sec
IN0 0.00001660 sec
L0 0
TDav 1
SFO1 600.1828208 MHz
NUC1 1H
P1 8.58 usec
P2 17.16 usec
PLW1 15.70400047 W
SFO2 150.9304726 MHz
NUC2 13C
P3 13.00 usec
P14 500.00 usec
P24 2000.00 usec
PLW2 192.00000000 W
SPNAM[3] Crp60,0.5,20.1
SPOAL3 0.500
SPOFFS3 0 Hz
SPW3 49.57699966 W
SPNAM[7] Crp60comp.4
SPOAL7 0.500
SPOFFS7 0 Hz
SPW7 49.57699966 W
GPNAM[1] SMSQ10.100
GPZ1 80.00 %
GPNAM[3] SMSQ10.100
GPZ3 14.00 %
P16 1000.00 usec

F1 - Acquisition parameters
TD 256
SFO1 150.9305 MHz
FIDRES 235.316269 Hz
SW 199.565 ppm
FnMODE Echo-Antiecho

F2 - Processing parameters
SI 1024
SF 600.1800286 MHz
WDW QSINE
SSB 2
LB 0 Hz
GB 0
PC 1.40

F1 - Processing parameters
SI 1024
MC2 echo-antiecho
SF 150.9153810 MHz
WDW QSINE
SSB 2
LB 0 Hz
GB 0

YYS-739-d-20240304-600 MHz-HMBC

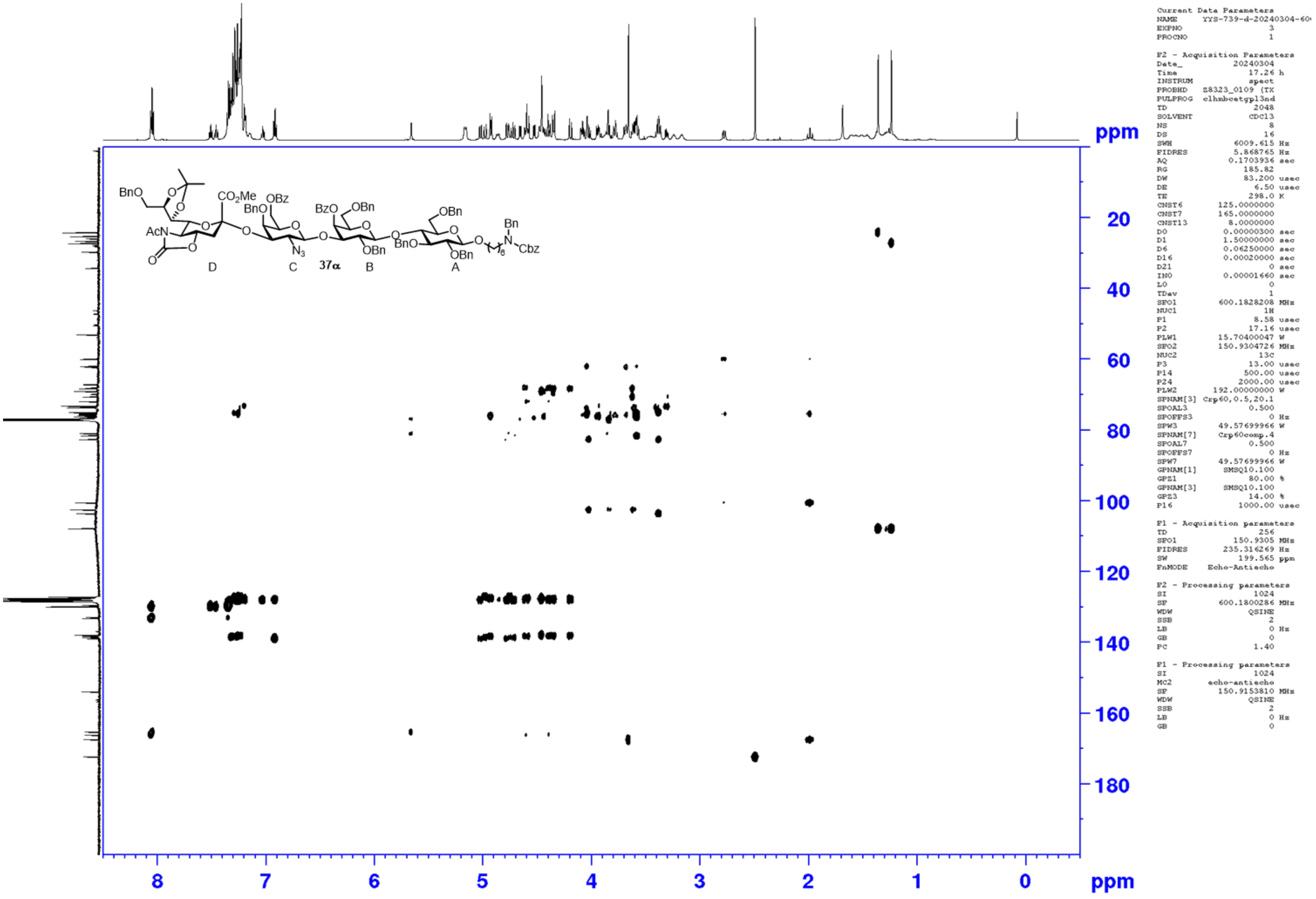

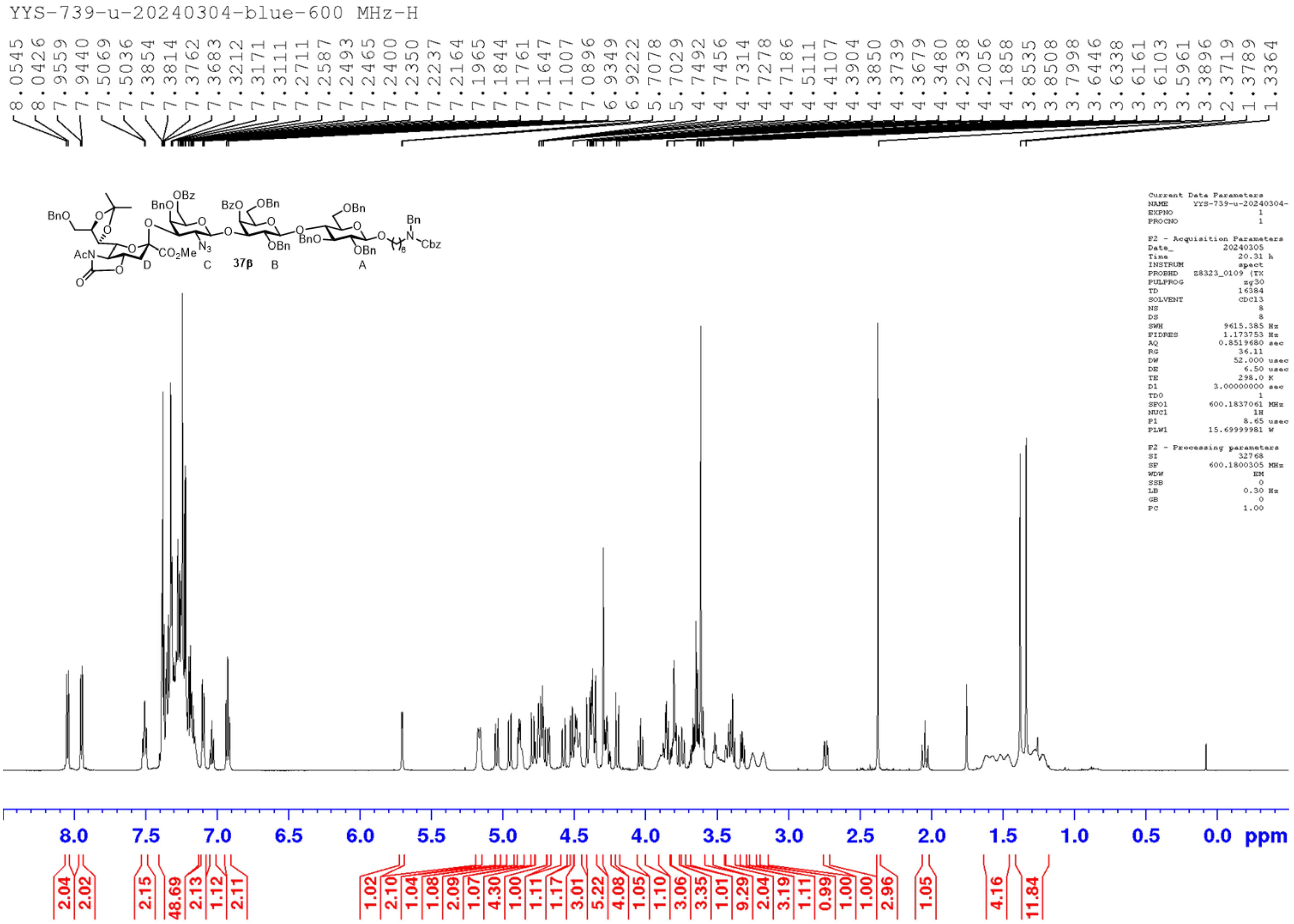
YYS-739-u-20240304-blue-600 MHz-H
37β
Current Data Parameters
NAME YYS-739-u-20240304-
EXPNO 1
PROCNO 1
F2 - Acquisition Parameters
Date_ 20240305
Time 20.31 h
INSTRUM spect
PULPROG zg30
TD 16384
SOLVENT CDCl3
NS 8
DS 8
SWH 9615.385 Hz
FIDRES 1.173753 Hz
AQ 0.8519680 sec
RG 36.11
DW 52.000 usec
DE 6.50 usec
TE 298.0 K
D1 3.00000000 sec
TD0 1
SFO1 600.1837061 MHz
NUC1 1H
P1 8.65 usec
PLW1 15.69999981 W
F2 - Processing parameters
SI 32768
SF 600.1800305 MHz
WDW EM
SSB 0
LB 0.30 Hz
GB 0
PC 1.00
ppm

YYS-739-u-20240304-blue-600 MHz-2

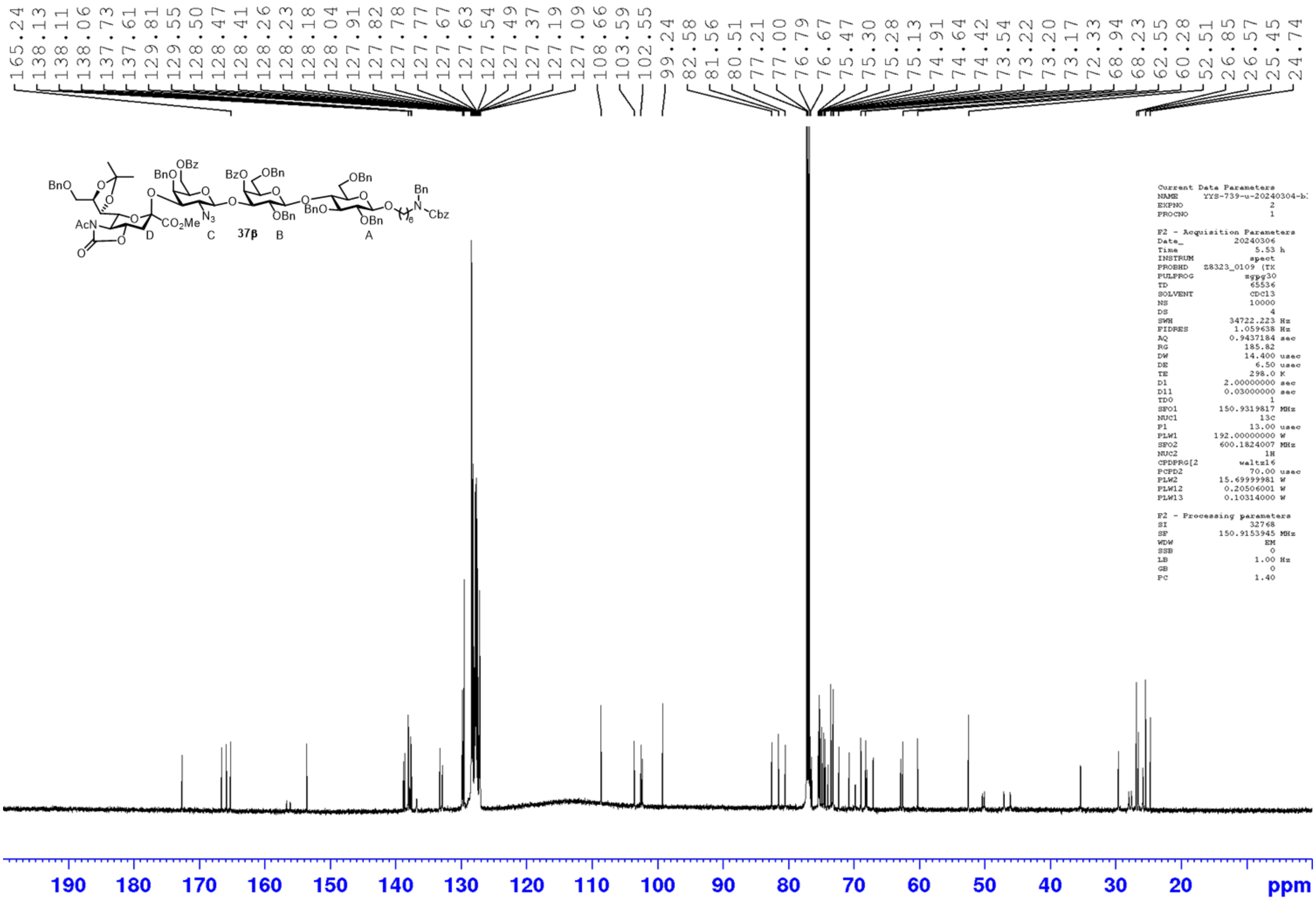

YYS-739-u-blue-EXSIDE-20240312

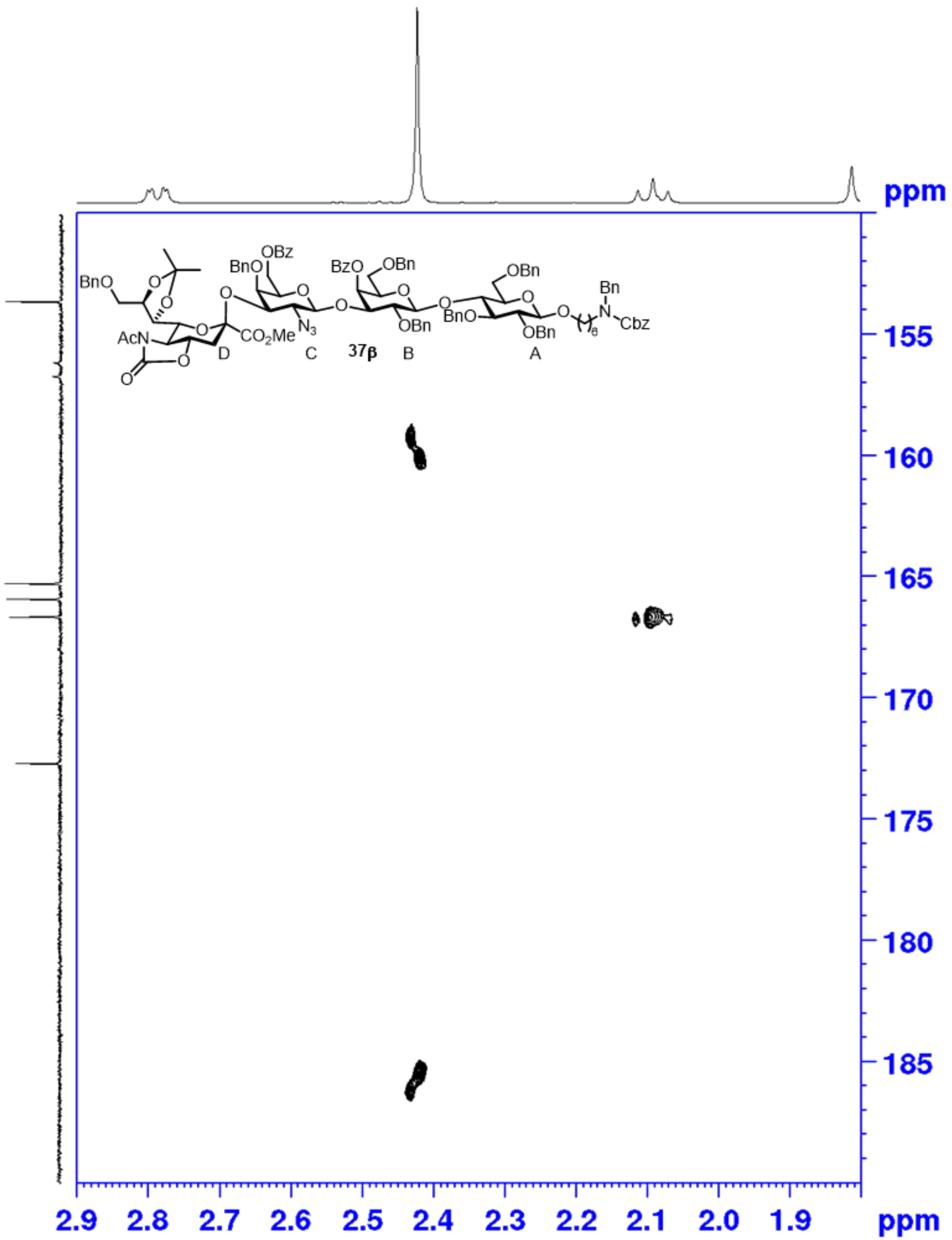


```
Current Data Parameters
NAME      YYS-739-u-blue-EXSIDE-2024031
EXPNO                    2
PROCNO                   1

F2 - Acquisition Parameters
Date_            20240308
Time                17.11 h
INSTRUM             spect
PROBHD      Z8323_0109 (TX
PULPROG    hsqcetgplrjcsp
TD                   2048
SOLVENT             CDCl3
NS                      4
DS                     32
SWH              6009.615 Hz
FIDRES           5.868765 Hz
AQ              0.1703936 sec
RG                 185.82
DW                 83.200 usec
DE                   6.50 usec
TE                  298.0 K
CNST2         145.0000000
CNST16         20.0000000
D0             0.00000300 sec
D1             1.50000000 sec
D2             0.00344828 sec
D6             0.06250000 sec
D11            0.03000000 sec
D16            0.00020000 sec
D20            0.01192100 sec
IN0            0.00001660 sec
IN20           0.00016600 sec
TDav                    1
SFO1          600.1812574 MHz
NUC1                   1H
P1                   8.65 usec
P2                  17.30 usec
P12               5000.00 usec
PLW1          15.70400047 W
SPNAM[2]  Gaus1_180r.1000
SPOAL2              0.500
SPOFFS2                 0 Hz
SPW2           0.00094907 W
SFO2          150.9304726 MHz
NUC2                  13C
CPDPRG[2             garp
P3                  13.00 usec
P14                500.00 usec
P24               2000.00 usec
PCPD2               60.00 usec
PLW2         192.00000000 W
PLW12          9.01329994 W
SPNAM[3]   Crp60,0.5,20.1
SPOAL3              0.500
SPOFFS3                 0 Hz
SPW3          49.57699966 W
SPNAM[7]    Crp60comp.4
SPOAL7              0.500
SPOFFS7                 0 Hz
SPW7          49.57699966 W
GPNAM[1]       SMSQ10.100
GPZ1                80.00 %
GPNAM[2]       SMSQ10.100
GPZ2                20.10 %
GPNAM[3]       SMSQ10.100
GPZ3                20.00 %
GPNAM[4]       SMSQ10.100
GPZ4                 7.00 %
GPNAM[5]       SMSQ10.100
GPZ5                11.00 %
GPNAM[6]       SMSQ10.100
GPZ6                50.00 %
P16               1000.00 usec

F1 - Acquisition parameters
TD                    254
SFO1             150.9305 MHz
FIDRES         237.169144 Hz
SW                199.565 ppm
FnMODE      Echo-Antiecho

F2 - Processing parameters
SI                   1024
SF            600.1800000 MHz
WDW                 QSINE
SSB                     2
LB                      0 Hz
GB                      0
PC                   1.00

F1 - Processing parameters
SI                   4096
MC2         echo-antiecho
SF            150.9153810 MHz
WDW                 QSINE
SSB                     2
LB                      0 Hz
GB                      0
```

YYS-739-u-20240304-blue-600 MHz-HMBC

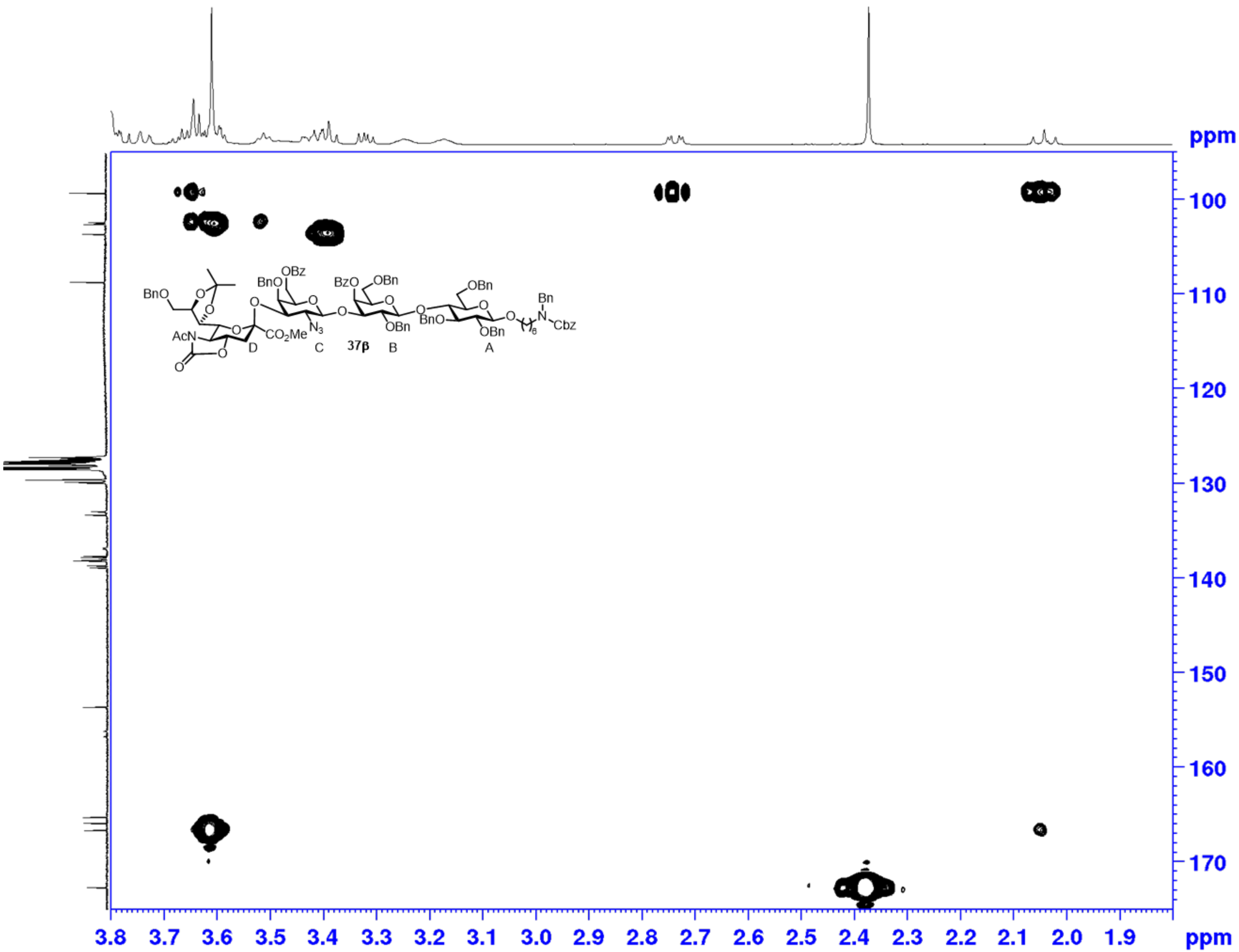

Current Data Parameters
NAME YYS-739-u-20240304-
EXPNO 3
PROCNO 1

F2 - Acquisition Parameters
Date_ 20240305
Time 20.32 h
INSTRUM spect
PROBHD Z8323_0109 (TX
PULPROG clhmbcetgpl3nd
TD 2048
SOLVENT CDCl3
NS 8
DS 16
SWH 6009.615 Hz
FIDRES 5.868765 Hz
AQ 0.1703936 sec
RG 185.82
DW 83.200 usec
DE 6.50 usec
TE 298.0 K
CNST6 125.0000000
CNST7 165.0000000
CNST13 8.0000000
D0 0.00000300 sec
D1 1.50000000 sec
D6 0.06250000 sec
D16 0.00020000 sec
D21 0 sec
IN0 0.00001660 sec
L0 0
TDav 1
SFO1 600.1828208 MHz
NUC1 1H
P1 8.65 usec
P2 17.30 usec
PLW1 15.70400047 W
SFO2 150.9304726 MHz
NUC2 13C
P3 13.00 usec
P14 500.00 usec
P24 2000.00 usec
PLW2 192.00000000 W
SPNAM[3] Crp60,0.5,20.1
SPOAL3 0.500
SPOFFS3 0 Hz
SPW3 49.57699966 W
SPNAM[7] Crp60comp.4
SPOAL7 0.500
SPOFFS7 0 Hz
SPW7 49.57699966 W
GPNAM[1] SMSQ10.100
GPZ1 80.00 %
GPNAM[3] SMSQ10.100
GPZ3 14.00 %
P16 1000.00 usec

F1 - Acquisition parameters
TD 256
SFO1 150.9305 MHz
FIDRES 235.316269 Hz
SW 199.565 ppm
FnMODE Echo-Antiecho

F2 - Processing parameters
SI 1024
SF 600.1800295 MHz
WDW QSINE
SSB 2
LB 0 Hz
GB 0
PC 1.40

F1 - Processing parameters
SI 1024
MC2 echo-antiecho
SF 150.9154008 MHz
WDW QSINE
SSB 2
LB 0 Hz
GB 0

YYS-739-u-20240304-blue-600 MHz-HMBC

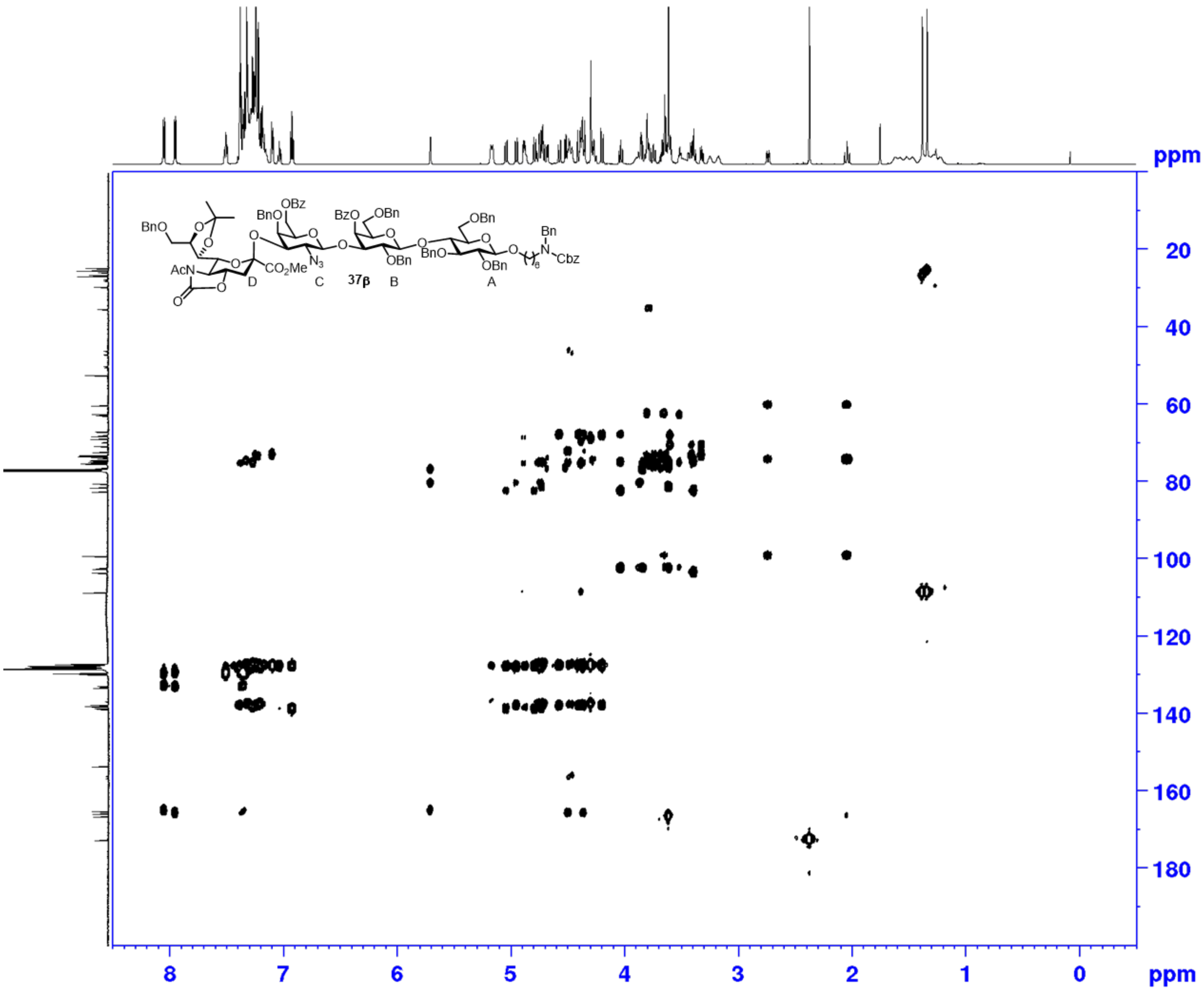


```
Current Data Parameters
NAME       YYS-739-u-20240304-blue-600 M
EXPNO                    3
PROCNO                   1

F2 - Acquisition Parameters
Date_             20240305
Time                 20.32 h
INSTRUM              spect
PROBHD     Z8323_0109 (TX
PULPROG   clhmbcetgpl3nd
TD                    2048
SOLVENT              CDCl3
NS                       8
DS                      16
SWH               6009.615 Hz
FIDRES            5.868765 Hz
AQ               0.1703936 sec
RG                  185.82
DW                  83.200 usec
DE                    6.50 usec
TE                   298.0 K
CNST6          125.0000000
CNST7          165.0000000
CNST13           8.0000000
D0              0.00000300 sec
D1              1.50000000 sec
D6              0.06250000 sec
D16             0.00020000 sec
D21                      0 sec
IN0             0.00001660 sec
L0                       0
TDav                     1
SFO1           600.1828208 MHz
NUC1                    1H
P1                    8.65 usec
P2                   17.30 usec
PLW1           15.70400047 W
SFO2           150.9304726 MHz
NUC2                   13C
P3                   13.00 usec
P14                 500.00 usec
P24                2000.00 usec
PLW2          192.00000000 W
SPNAM[3]  Crp60,0.5,20.1
SPOAL3               0.500
SPOFFS3                  0 Hz
SPW3           49.57699966 W
SPNAM[7]      Crp60comp.4
SPOAL7               0.500
SPOFFS7                  0 Hz
SPW7           49.57699966 W
GPNAM[1]       SMSQ10.100
GPZ1                 80.00 %
GPNAM[3]       SMSQ10.100
GPZ3                 14.00 %
P16                1000.00 usec

F1 - Acquisition parameters
TD                     256
SFO1              150.9305 MHz
FIDRES          235.316269 Hz
SW                 199.565 ppm
FnMODE       Echo-Antiecho

F2 - Processing parameters
SI                    1024
SF             600.1800295 MHz
WDW                  QSINE
SSB                      2
LB                       0 Hz
GB                       0
PC                    1.40

F1 - Processing parameters
SI                    1024
MC2          echo-antiecho
SF             150.9154008 MHz
WDW                  QSINE
SSB                      2
LB                       0 Hz
GB                       0
```

YYS-740-20240310-C-H-1

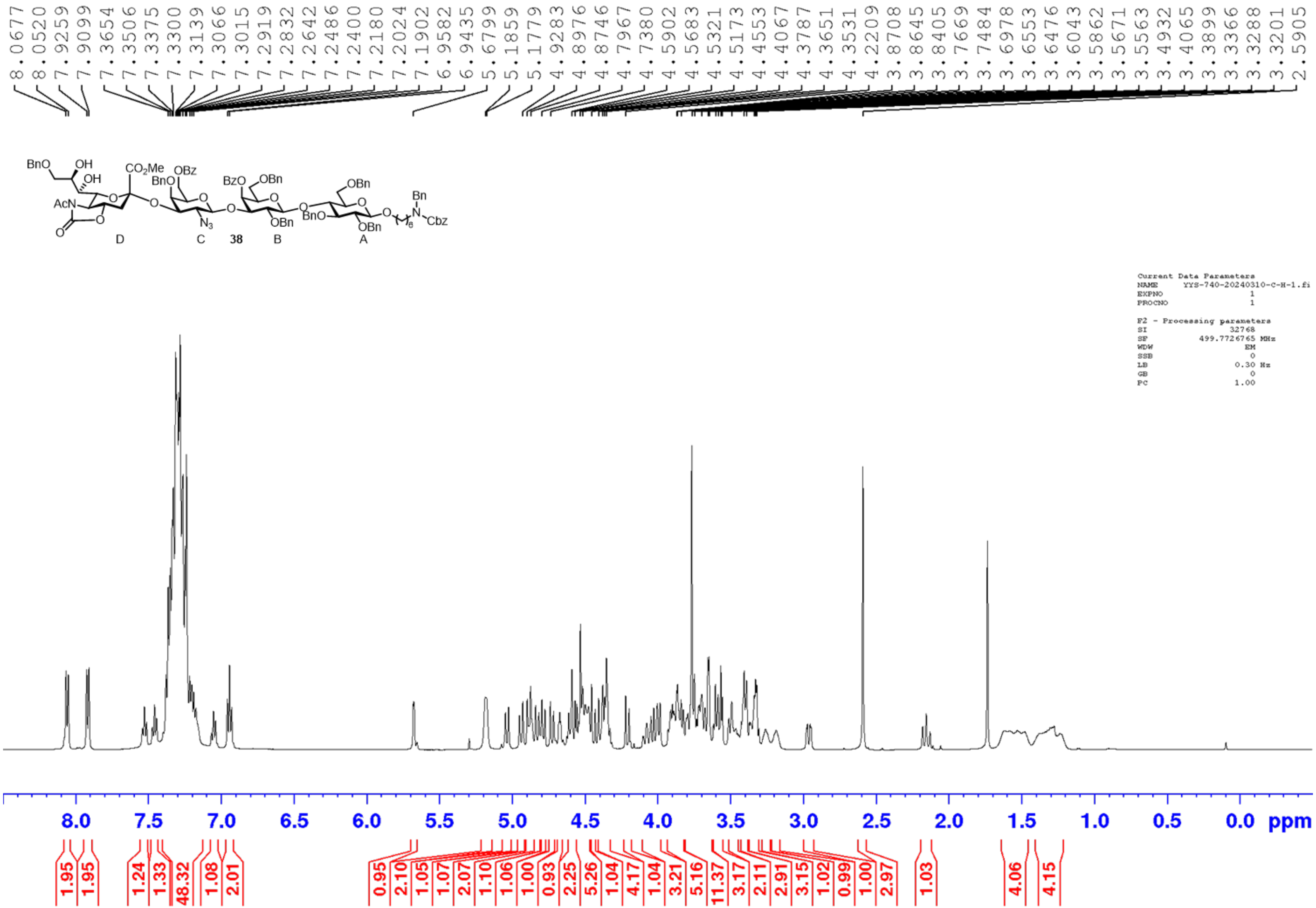

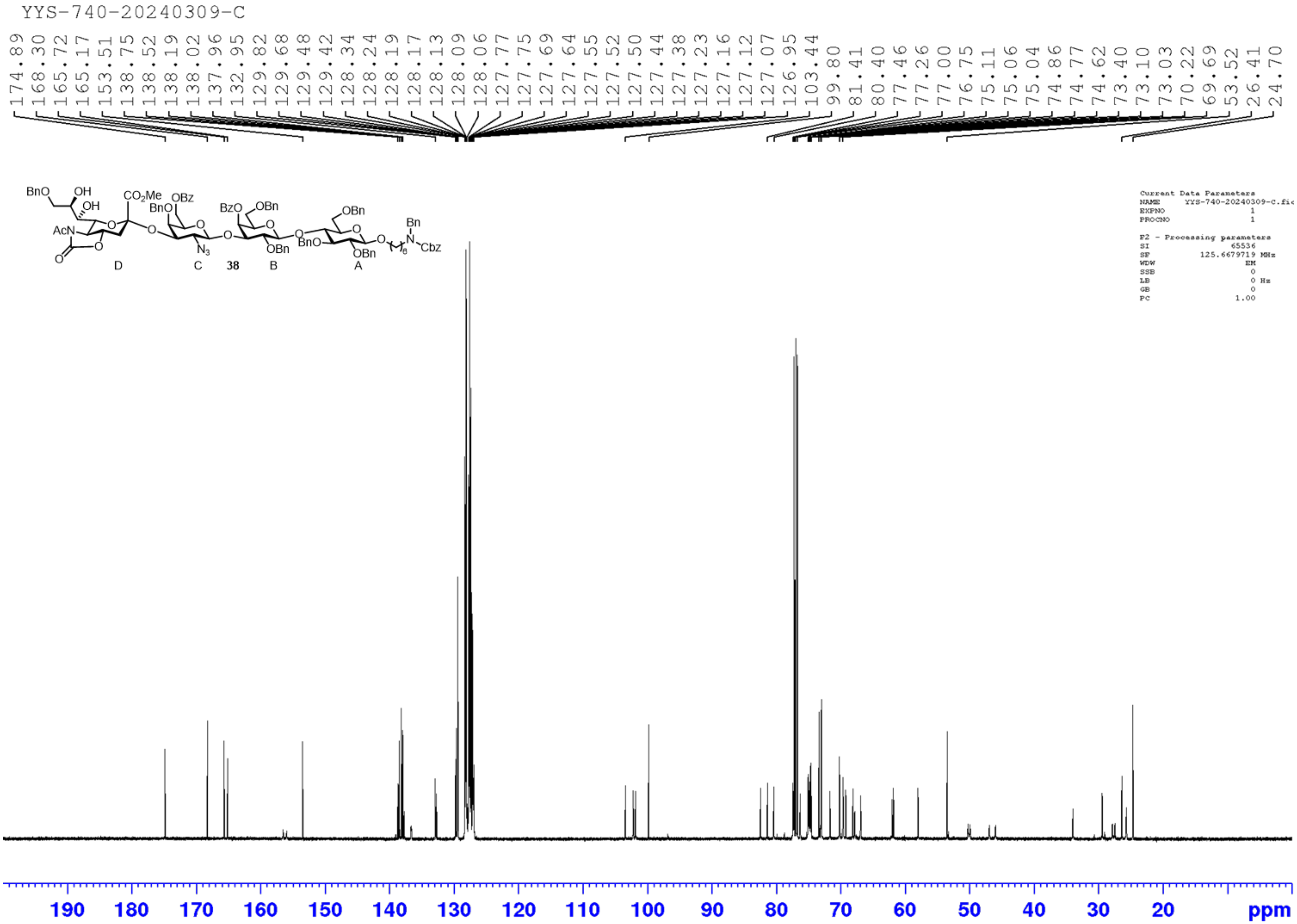
YYS-740-20240309-C
174.89
168.30
165.72
165.17
153.51
138.75
138.52
138.19
138.02
137.96
132.95
129.82
129.68
129.48
129.42
128.34
128.24
128.19
128.17
128.13
128.09
128.06
127.77
127.75
127.69
127.64
127.55
127.52
127.50
127.44
127.38
127.23
127.16
127.12
127.07
126.95
103.44
99.80
81.41
80.40
77.46
77.26
77.00
76.75
75.11
75.06
75.04
74.86
74.77
74.62
73.40
73.10
73.03
70.22
69.69
53.52
26.41
24.70
BnO
OH
OH
CO2Me
OBz
BnO
AcN
O
O
N3
BzO
OBn
OBn
BnO
OBn
OBn
Bn
N
Cbz
D
C
38
B
A
Current Data Parameters
NAME YYS-740-20240309-C.fid
EXPNO 1
PROCNO 1
F2 - Processing parameters
SI 65536
SF 125.6679719 MHz
WDW EM
SSB 0
LB 0 Hz
GB 0
PC 1.00
190
180
170
160
150
140
130
120
110
100
90
80
70
60
50
40
30
20
ppm

YYS-833-20250117-850 MHz

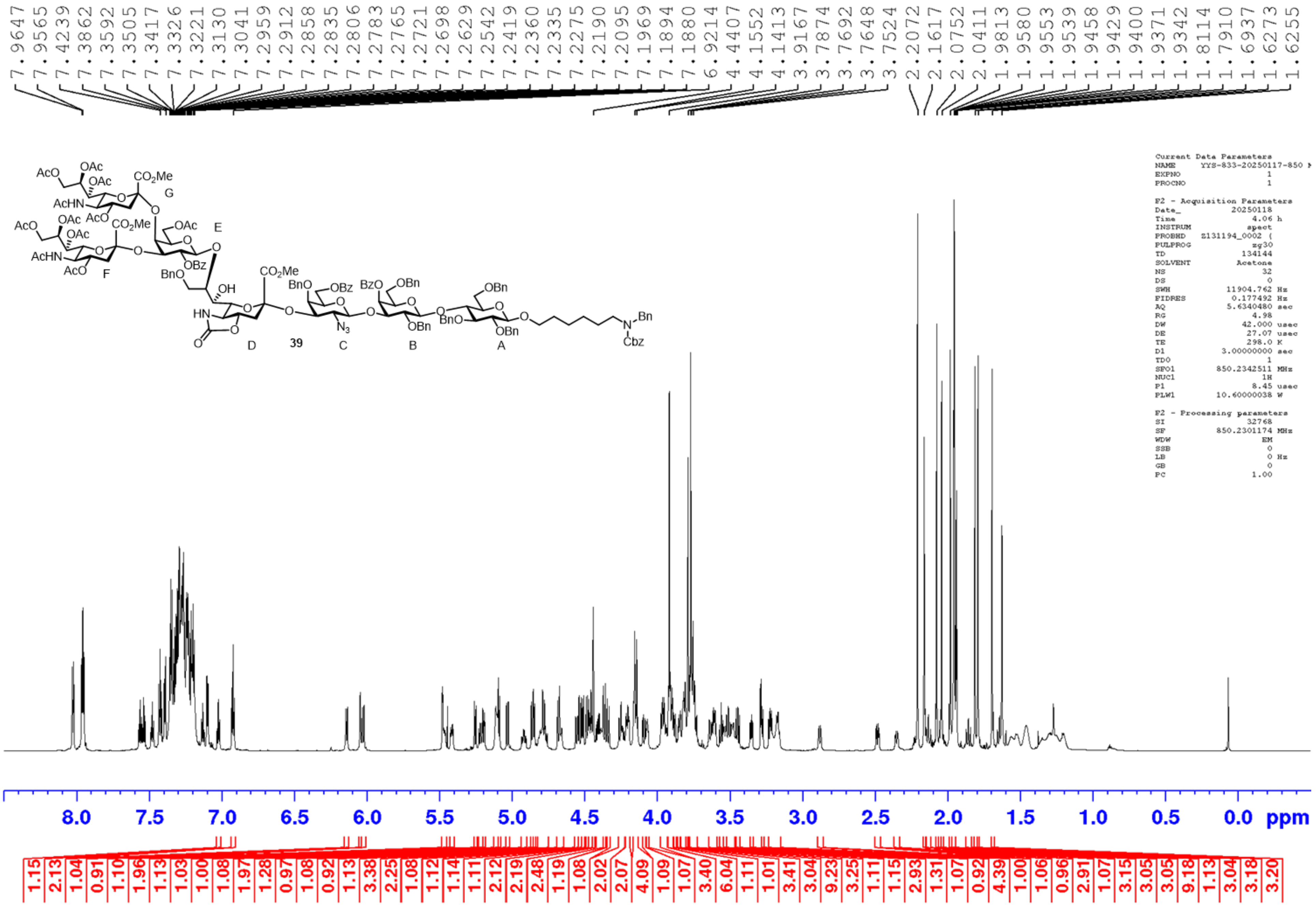

YYS-833-20250117-850 MHz

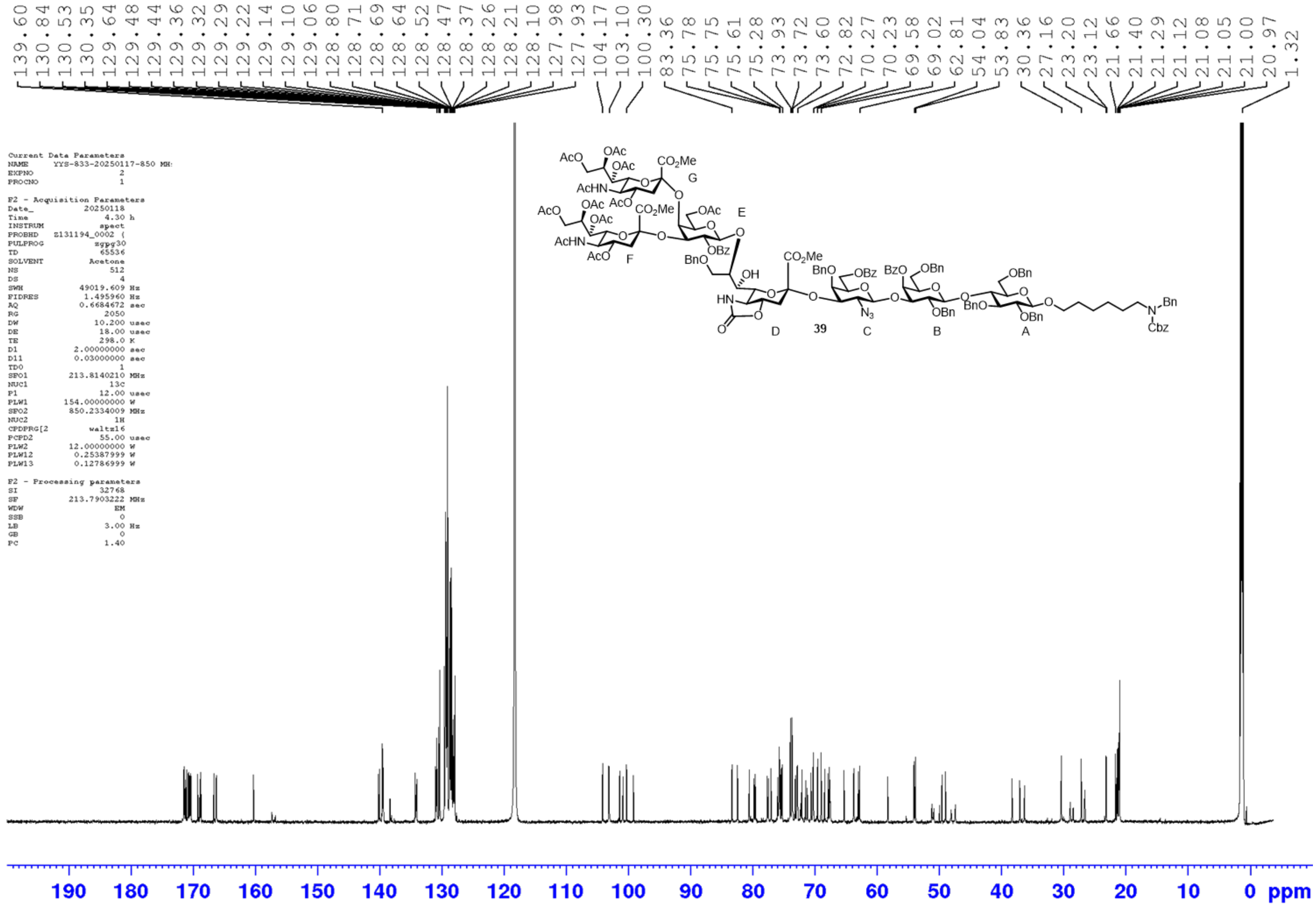

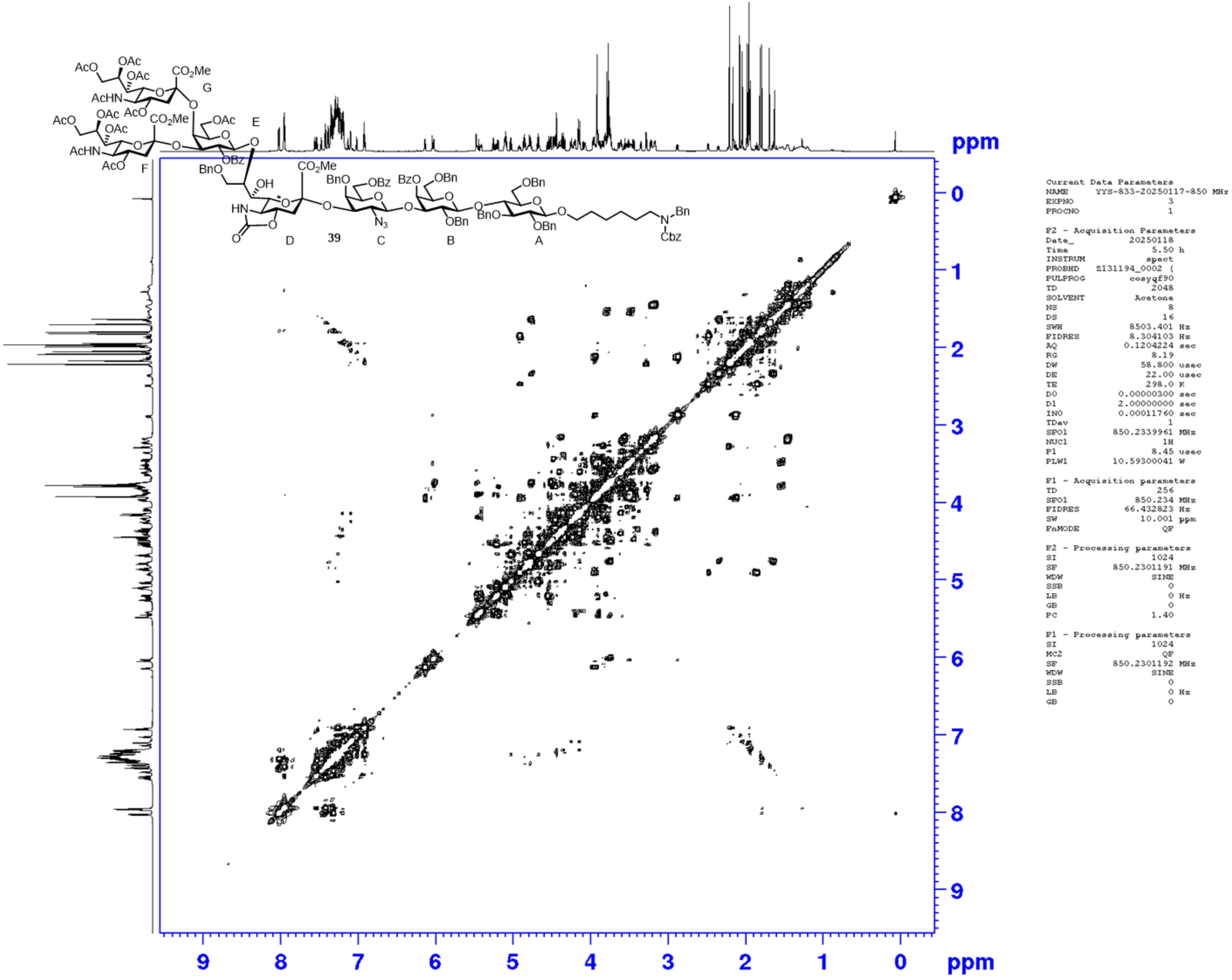
39
A
B
C
D
E
F
G
ppm
Current Data Parameters
NAME YYS-833-20250117-850 MHz
EXPNO 3
PROCNO 1
F2 - Acquisition Parameters
Date_ 20250118
Time 5.50 h
INSTRUM spect
PROBHD Z131194_0002 (
PULPROG cosygf90
TD 2048
SOLVENT Acetone
NS 8
DS 16
SWH 8503.401 Hz
FIDRES 8.304103 Hz
AQ 0.1204224 sec
RG 8.19
DW 58.800 usec
DE 22.00 usec
TE 298.0 K
D0 0.00000300 sec
D1 2.00000000 sec
IN0 0.00011760 sec
TDav 1
SFO1 850.2339961 MHz
NUC1 1H
P1 8.45 usec
PLW1 10.59300041 W
F1 - Acquisition parameters
TD 256
SFO1 850.234 MHz
FIDRES 66.432823 Hz
SW 10.001 ppm
FnMODE QF
F2 - Processing parameters
SI 1024
SF 850.2301191 MHz
WDW SINE
SSB 0
LB 0 Hz
GB 0
PC 1.40
F1 - Processing parameters
SI 1024
MC2 QF
SF 850.2301192 MHz
WDW SINE
SSB 0
LB 0 Hz
GB 0

HSQC

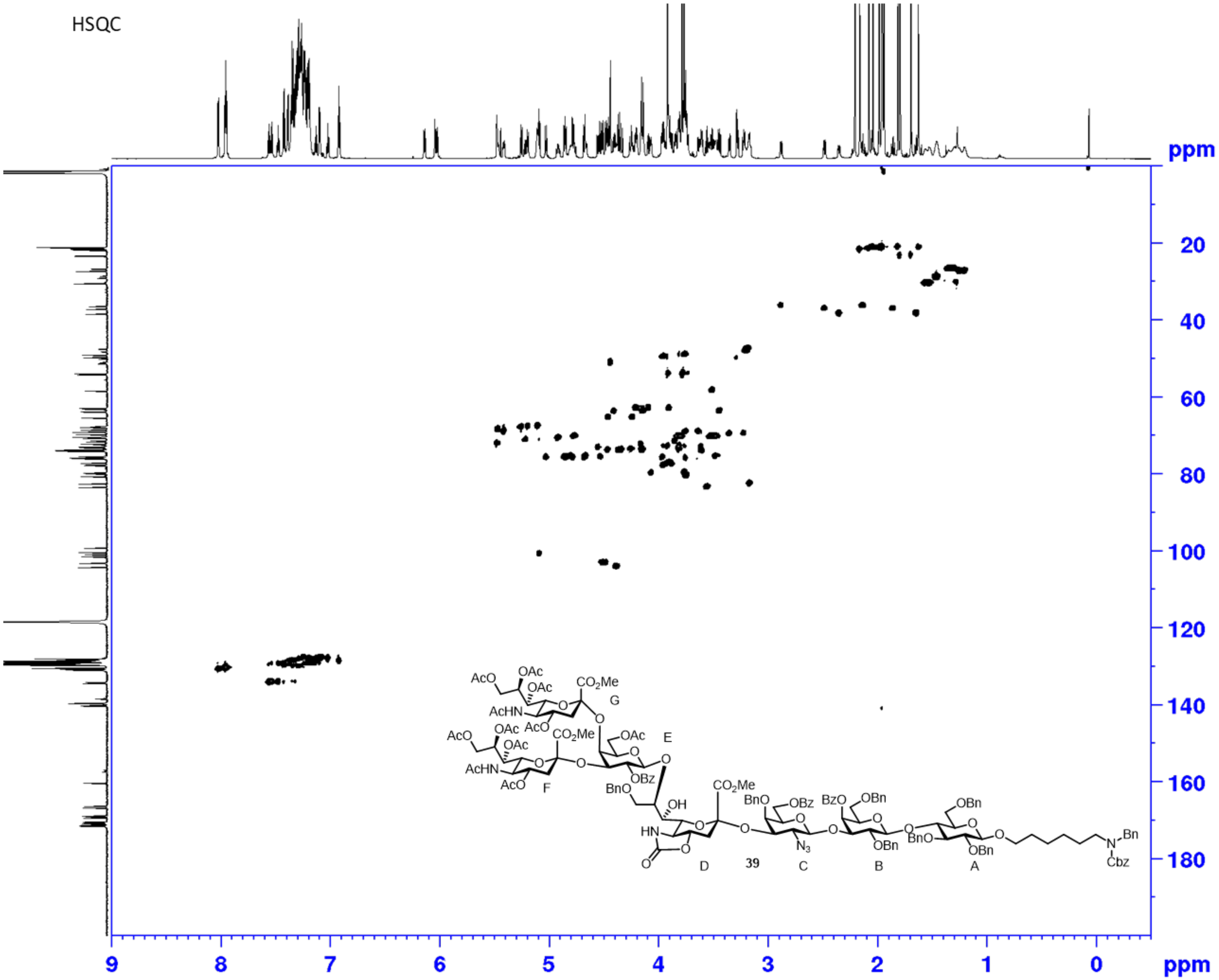


```
Current Data Parameters
NAME      YYS-833-20250117-850 MHz
EXPNO                    4
PROCNO                   1

F2 - Acquisition Parameters
Date_             20250118
Time                  4.31 h
INSTRUM              spect
PROBHD    Z131194_0002 (
PULPROG   hsqcedetgpsisp2.3
TD                    2048
SOLVENT            Acetone
NS                       8
DS                      16
SWH               8503.401 Hz
FIDRES            8.304103 Hz
AQ               0.1204224 sec
RG                   39.25
DW                  58.800 usec
DE                   22.00 usec
TE                   298.0 K
CNST2          145.0000000
CNST17          -0.5000000
D0              0.00000300 sec
D1              1.50000000 sec
D4              0.00172414 sec
D11             0.03000000 sec
D16             0.00020000 sec
D21             0.00344828 sec
D24             0.00086207 sec
IN0             0.00001560 sec
TDav                     1
SFO1           850.2339961 MHz
NUC1                    1H
P1                    8.45 usec
P2                   16.90 usec
P28                      0 usec
PLW1           10.59300041 W
SFO2           213.8054693 MHz
NUC2                   13C
CPDPRG[2  bi_p5m4sp_4sp.2
P3                   12.00 usec
P14                 500.00 usec
P24                2000.00 usec
P31                1453.00 usec
P63                1500.00 usec
PLW0                     0 W
PLW2          168.00000000 W
PLW12           9.67679977 W
SPNAM[3]  Crp80,0.5,20.1
SPOAL3               0.500
SPOFFS3                  0 Hz
SPW3           49.28400040 W
SPNAM[7]     Crp80comp.4
SPOAL7               0.500
SPOFFS7                  0 Hz
SPW7           49.28400040 W
SPNAM[14  Crp48,1.5,20.2
SPOAL14              0.500
SPOFFS14                 0 Hz
SPW14          23.65600014 W
SPNAM[18   Crp60_xfilt.2
SPOAL18              0.500
SPOFFS18                 0 Hz
SPW18          15.14400005 W
SPNAM[31  Crp48,1.5,20.2
SPOAL31              0.500
SPOFFS31                 0 Hz
SPW31           5.91400003 W
GPNAM[1]       SMSQ10.100
GPZ1                 80.00 %
GPNAM[2]       SMSQ10.100
GPZ2                 20.10 %
GPNAM[3]       SMSQ10.100
GPZ3                 11.00 %
GPNAM[4]       SMSQ10.100
GPZ4                 -5.00 %
P16                1000.00 usec
P19                 600.00 usec

F1 - Acquisition parameters
TD                     360
SFO1              213.8055 MHz
FIDRES          178.062683 Hz
SW                 149.909 ppm
FnMODE       Echo-Antiecho

F2 - Processing parameters
SI                    1024
SF             850.2301110 MHz
WDW                  QSINE
SSB                      2
LB                       0 Hz
GB                       0
PC                    1.40

F1 - Processing parameters
SI                    1024
MC2          echo-antiecho
[illegible]
```

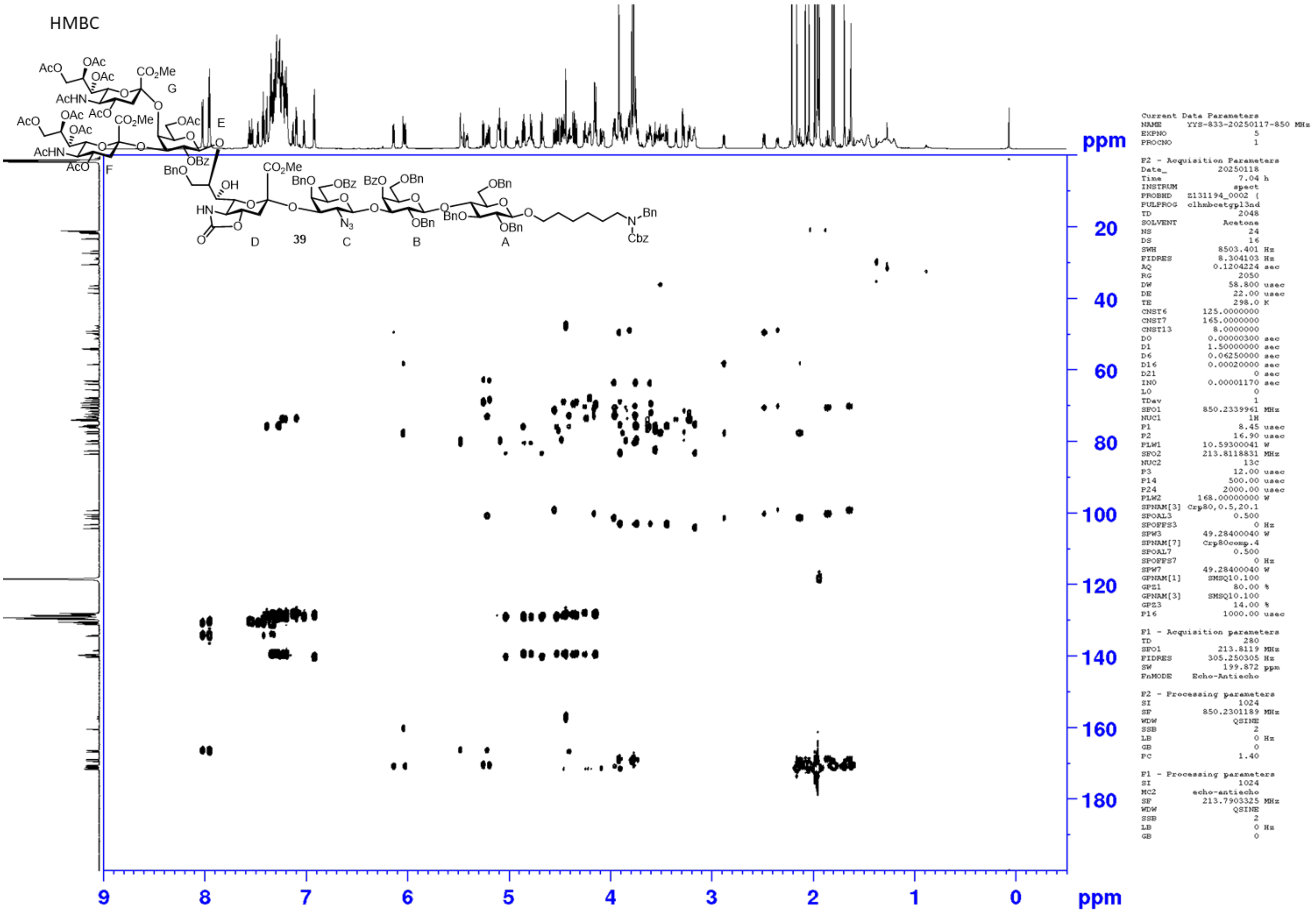

HMBC
39
A
B
C
D
E
F
G
ppm
Current Data Parameters
NAME YYS-833-20250117-850 MHz
EXPNO 5
PROCNO 1
F2 - Acquisition Parameters
Date_ 20250118
Time 7.04 h
INSTRUM spect
PULPROG clhmbcetgpl3nd
TD 2048
SOLVENT Acetone
NS 24
DS 16
SWH 8503.401 Hz
FIDRES 8.304103 Hz
AQ 0.1204224 sec
RG 2050
DW 58.800 usec
DE 22.00 usec
TE 298.0 K
SFO1 850.2339961 MHz
NUC1 1H
SFO2 213.8118831 MHz
NUC2 13C
F1 - Acquisition parameters
TD 280
SFO1 213.8119 MHz
FIDRES 305.250305 Hz
SW 199.872 ppm
FnMODE Echo-Antiecho
F2 - Processing parameters
SI 1024
SF 850.2301189 MHz
WDW QSINE
F1 - Processing parameters
SI 1024
MC2 echo-antiecho
SF 213.7903325 MHz
WDW QSINE

HSQC-TOCSY

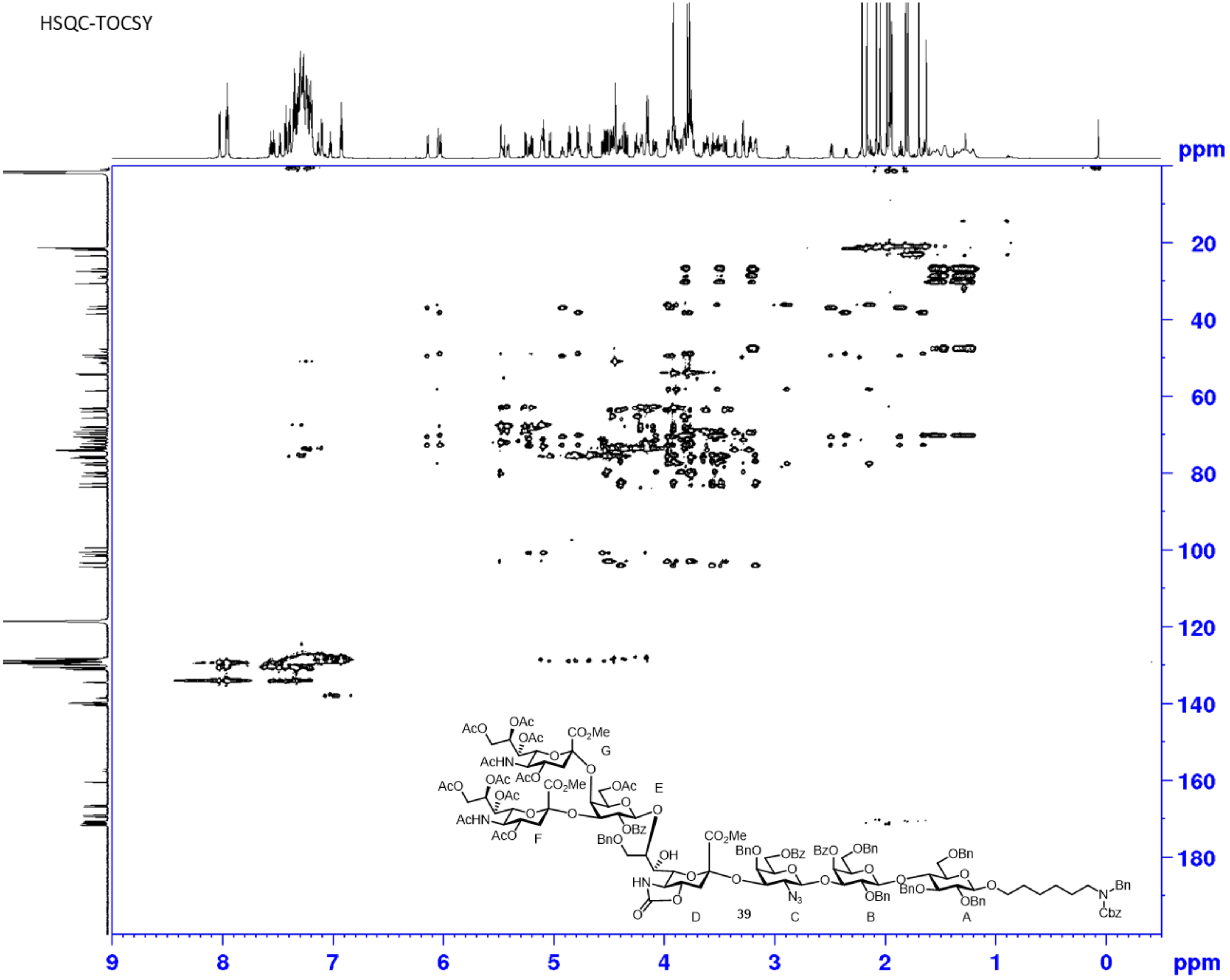


```
Current Data Parameters
NAME      YYS-833-20250117-850 MHz
EXPNO                    6
PROCNO                   1

F2 - Acquisition Parameters
Date_             20250118
Time                 10.16 h
INSTRUM              spect
PROBHD    Z131194_0002 (
PULPROG   hsqcdietgpsisp.2
TD                    2048
SOLVENT            Acetone
NS                      24
DS                      16
SWH               8503.401 Hz
FIDRES            8.304103 Hz
AQ               0.1204224 sec
RG                  164.29
DW                  58.800 usec
DE                   22.00 usec
TE                   298.0 K
CNST2          145.0000000
CNST17          -0.5000000
D0              0.00000300 sec
D1              1.20000005 sec
D4              0.00172414 sec
D9              0.06000000 sec
D11             0.03000000 sec
D16             0.00020000 sec
D24             0.00089000 sec
IN0             0.00001110 sec
L1                      26
TDav                     1
SFO1           850.2339961 MHz
NUC1                    1H
P1                    8.45 usec
P2                   16.90 usec
P6                   20.00 usec
P28                      0 usec
PLW1           10.59300041 W
PLW10           1.89080000 W
SFO2           213.8118831 MHz
NUC2                   13C
CPDPRG[2              garp
P3                   12.00 usec
P14                 500.00 usec
P24                2000.00 usec
PCPD2                50.00 usec
PLW0                     0 W
PLW2          168.00000000 W
PLW12           9.67679977 W
SPNAM[3]  Crp80,0.5,20.1
SPOAL3               0.500
SPOFFS3                  0 Hz
SPW3           49.28400040 W
SPNAM[7]     Crp80comp.4
SPOAL7               0.500
SPOFFS7                  0 Hz
SPW7           49.28400040 W
GPNAM[1]       SMSQ10.100
GPZ1                 80.00 %
GPNAM[2]       SMSQ10.100
GPZ2                 20.10 %
P16                1000.00 usec

F1 - Acquisition parameters
TD                     360
SFO1              213.8119 MHz
FIDRES          250.250244 Hz
SW                 210.676 ppm
FnMODE      Echo-Antiecho

F2 - Processing parameters
SI                    1024
SF             850.2301110 MHz
WDW                  QSINE
SSB                      2
LB                       0 Hz
GB                       0
PC                    1.40

F1 - Processing parameters
SI                    1024
MC2         echo-antiecho
SF             213.7903266 MHz
WDW                  QSINE
SSB                      2
LB                       0 Hz
GB                       0
```

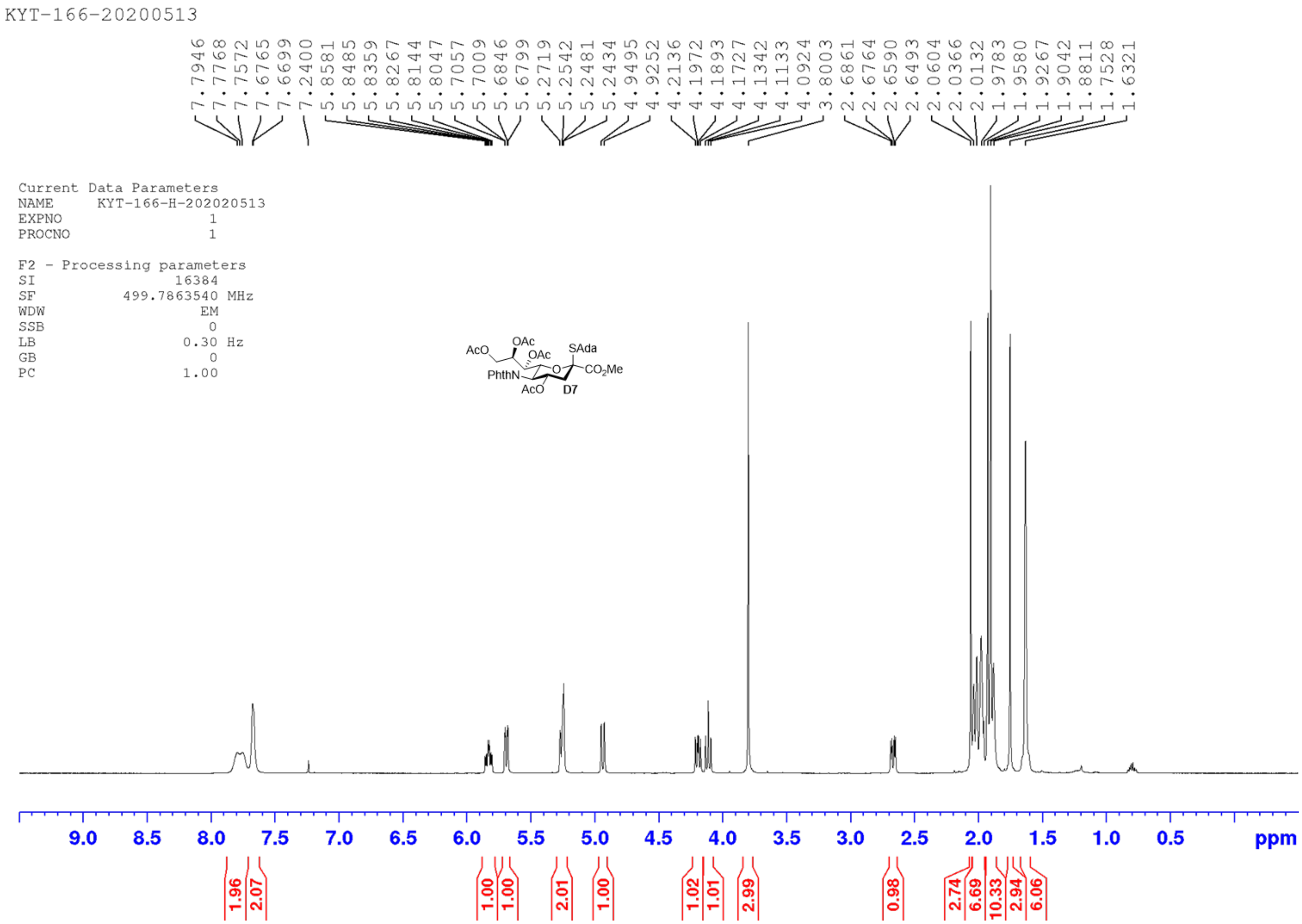
KYT-166-20200513
7.7946
7.7768
7.7572
7.6765
7.6699
7.2400
5.8581
5.8485
5.8359
5.8267
5.8144
5.8047
5.7057
5.7009
5.6846
5.6799
5.2719
5.2542
5.2481
5.2434
4.9495
4.9252
4.2136
4.1972
4.1893
4.1727
4.1342
4.1133
4.0924
3.8003
2.6861
2.6764
2.6590
2.6493
2.0604
2.0366
2.0132
1.9783
1.9580
1.9267
1.9042
1.8811
1.7528
1.6321
Current Data Parameters
NAME KYT-166-H-202020513
EXPNO 1
PROCNO 1
F2 - Processing parameters
SI 16384
SF 499.7863540 MHz
WDW EM
SSB 0
LB 0.30 Hz
GB 0
PC 1.00
AcO
OAc
OAc
SAda
PhthN
O
CO2Me
AcO
D7
9.0
8.5
8.0
7.5
7.0
6.5
6.0
5.5
5.0
4.5
4.0
3.5
3.0
2.5
2.0
1.5
1.0
0.5
ppm
1.96
2.07
1.00
1.00
2.01
1.00
1.02
1.01
2.99
0.98
2.74
6.69
10.33
2.94
6.06

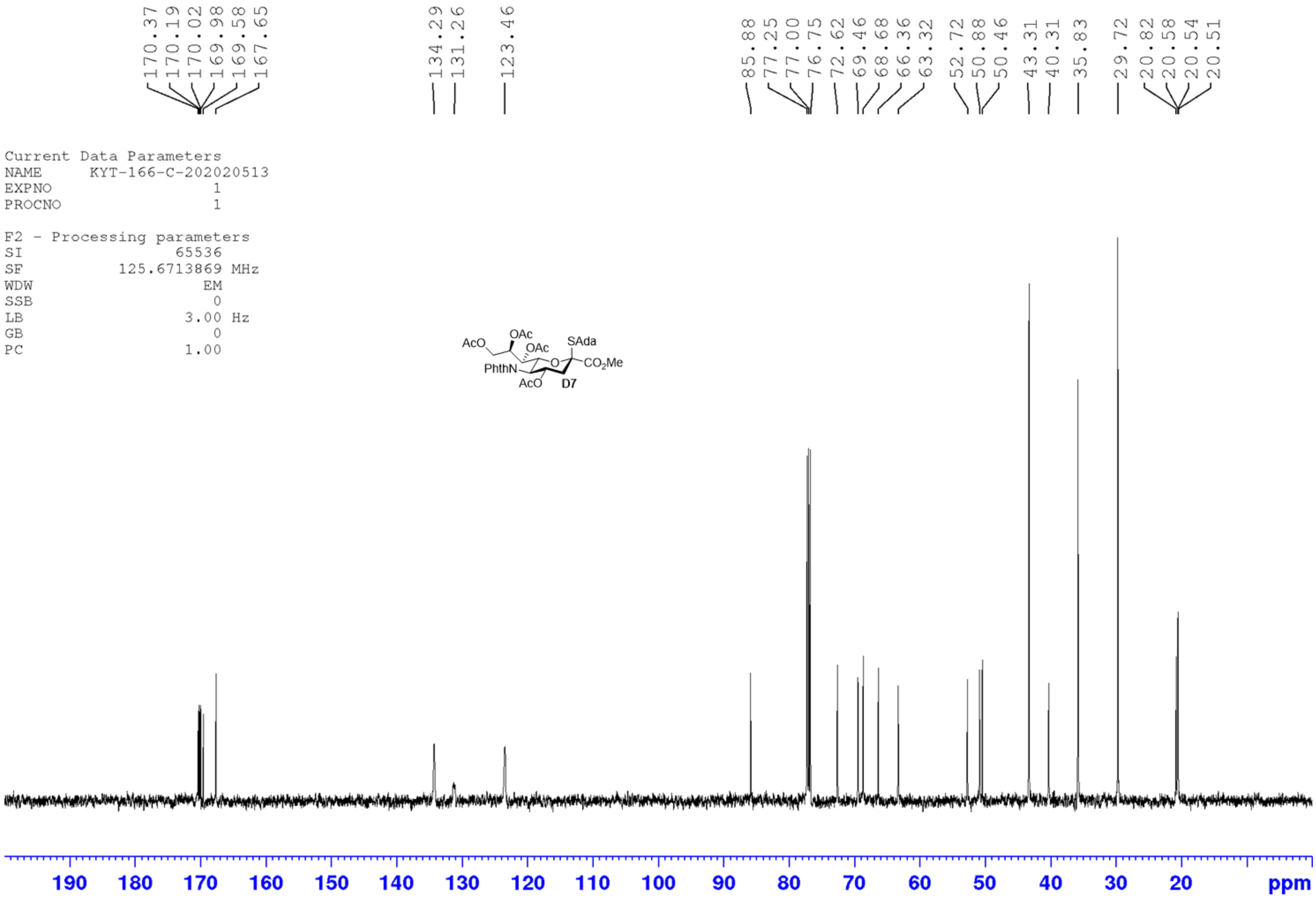
170.37
170.19
170.02
169.98
169.58
167.65
134.29
131.26
123.46
85.88
77.25
77.00
76.75
72.62
69.46
68.68
66.36
63.32
52.72
50.88
50.46
43.31
40.31
35.83
29.72
20.82
20.58
20.54
20.51
Current Data Parameters
NAME KYT-166-C-202020513
EXPNO 1
PROCNO 1
F2 - Processing parameters
SI 65536
SF 125.6713869 MHz
WDW EM
SSB 0
LB 3.00 Hz
GB 0
PC 1.00
AcO
OAc
OAc
SAda
PhthN
O
CO_2Me
AcO
D7
190
180
170
160
150
140
130
120
110
100
90
80
70
60
50
40
30
20
ppm

YYS-487-20220615

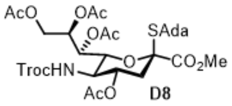

Current Data Parameters
NAME YYS-487-20220615.fid
EXPNO 1
PROCNO 1

F2 - Processing parameters
SI 32768
SF 499.7727655 MHz
WDW EM
SSB 0
LB 0.30 Hz
GB 0
PC 1.00

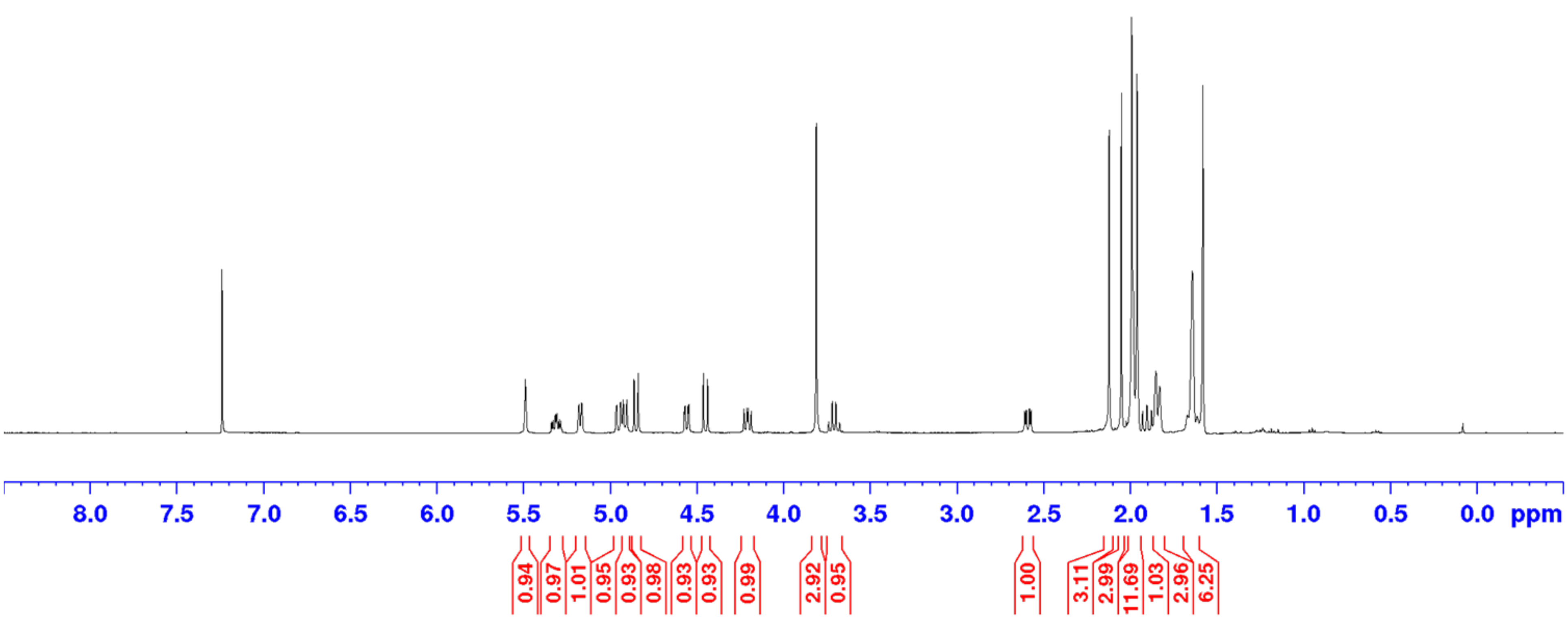

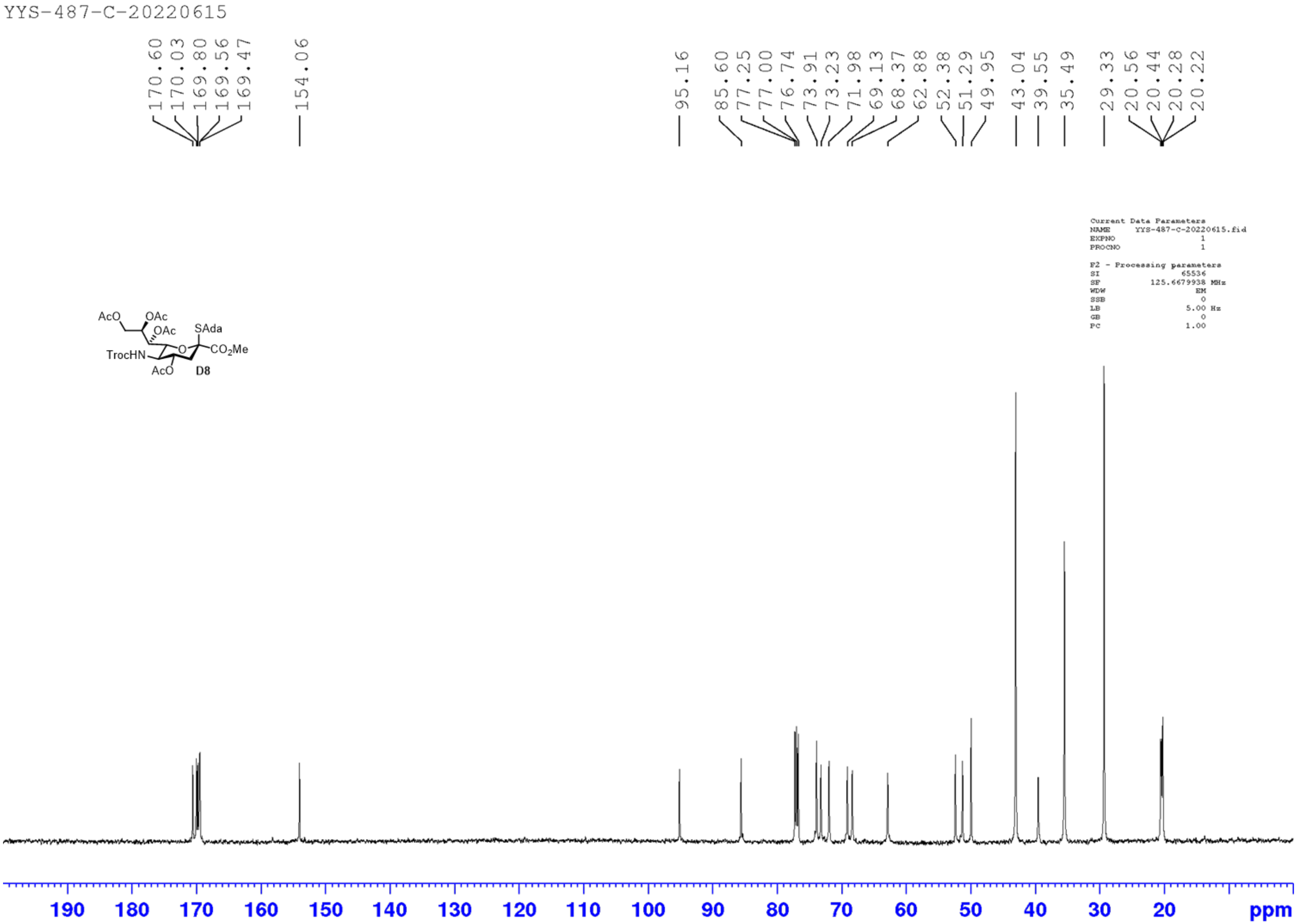
YYS-487-C-20220615
170.60
170.03
169.80
169.56
169.47
154.06
95.16
85.60
77.25
77.00
76.74
73.91
73.23
71.98
69.13
68.37
62.88
52.38
51.29
49.95
43.04
39.55
35.49
29.33
20.56
20.44
20.28
20.22
Current Data Parameters
NAME YYS-487-C-20220615.fid
EXPNO 1
PROCNO 1
F2 - Processing parameters
SI 65536
SF 125.6679938 MHz
WDW EM
SSB 0
LB 5.00 Hz
GB 0
PC 1.00
AcO
OAc
OAc
SAda
TrocHN
O
CO2Me
AcO
D8
190
180
170
160
150
140
130
120
110
100
90
80
70
60
50
40
30
20
ppm

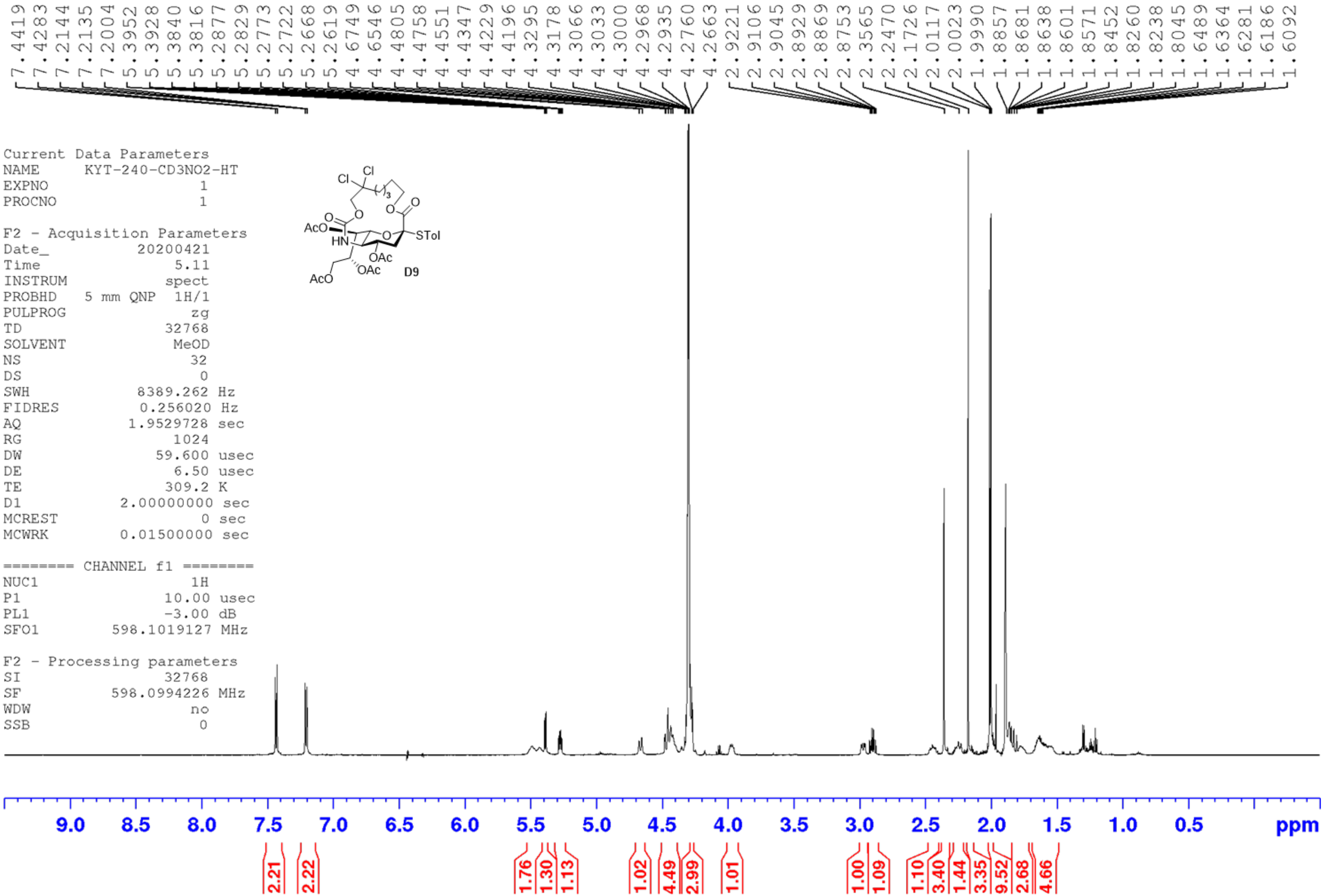
7.4419 7.4283 7.2144 7.2135 7.2004 5.3952 5.3928 5.3840 5.3816 5.2877 5.2829 5.2773 5.2722 5.2668 5.2619 4.6749 4.6546 4.4805 4.4758 4.4551 4.4347 4.4229 4.4196 4.3295 4.3178 4.3066 4.3033 4.3000 4.2968 4.2935 4.2760 4.2663 2.9221 2.9106 2.9045 2.8929 2.8869 2.8753 2.3565 2.2470 2.1726 2.0117 2.0023 1.9990 1.8857 1.8681 1.8638 1.8601 1.8571 1.8452 1.8260 1.8238 1.8045 1.6489 1.6364 1.6281 1.6186 1.6092
Current Data Parameters
NAME KYT-240-CD3NO2-HT
EXPNO 1
PROCNO 1
F2 - Acquisition Parameters
Date_ 20200421
Time 5.11
INSTRUM spect
PROBHD 5 mm QNP 1H/1
PULPROG zg
TD 32768
SOLVENT MeOD
NS 32
DS 0
SWH 8389.262 Hz
FIDRES 0.256020 Hz
AQ 1.9529728 sec
RG 1024
DW 59.600 usec
DE 6.50 usec
TE 309.2 K
D1 2.00000000 sec
MCREST 0 sec
MCWRK 0.01500000 sec
======== CHANNEL f1 ========
NUC1 1H
P1 10.00 usec
PL1 -3.00 dB
SFO1 598.1019127 MHz
F2 - Processing parameters
SI 32768
SF 598.0994226 MHz
WDW no
SSB 0
Cl
Cl
3
O
O
O
O
AcO
HN
O
STol
OAc
OAc
AcO
D9
9.0 8.5 8.0 7.5 7.0 6.5 6.0 5.5 5.0 4.5 4.0 3.5 3.0 2.5 2.0 1.5 1.0 0.5 ppm
2.21 2.22 1.76 1.30 1.13 1.02 4.49 2.99 1.01 1.00 1.09 1.10 3.40 1.44 3.35 9.52 2.68 4.66

```
Current Data Parameters
NAME      KYT-240-CD3NO2-HT
EXPNO                   2
PROCNO                  1

F2 - Acquisition Parameters
Date_          20200422
Time               5.11
INSTRUM           spect
PROBHD   5 mm QNP  1H/1
PULPROG            zgpg
TD                32768
SOLVENT           CDCl3
NS                 1518
DS                    0
SWH           45045.047 Hz
FIDRES         1.374666 Hz
AQ            0.3637248 sec
RG                 4096
DW               11.100 usec
DE                 6.50 usec
TE                311.4 K
D1           3.50000000 sec
d11          0.03000000 sec
DELTA        3.40000010 sec
MCREST                0 sec
MCWRK        0.01500000 sec

======== CHANNEL f1 ========
NUC1                13C
P1                 4.80 usec
PL1                   0 dB
SFO1        150.4092089 MHz

======== CHANNEL f2 ========
CPDPRG[2        waltz16
NUC2                 1H
PCPD2             92.00 usec
PL2              120.00 dB
PL12               4.00 dB
PL13               8.00 dB
SFO2        598.1029905 MHz

F2 - Processing parameters
SI                65536
SF          150.3929564 MHz
WDW                  EM
SSB                   0
LB                 5.00 Hz
GB                    0
PC                 1.00
```

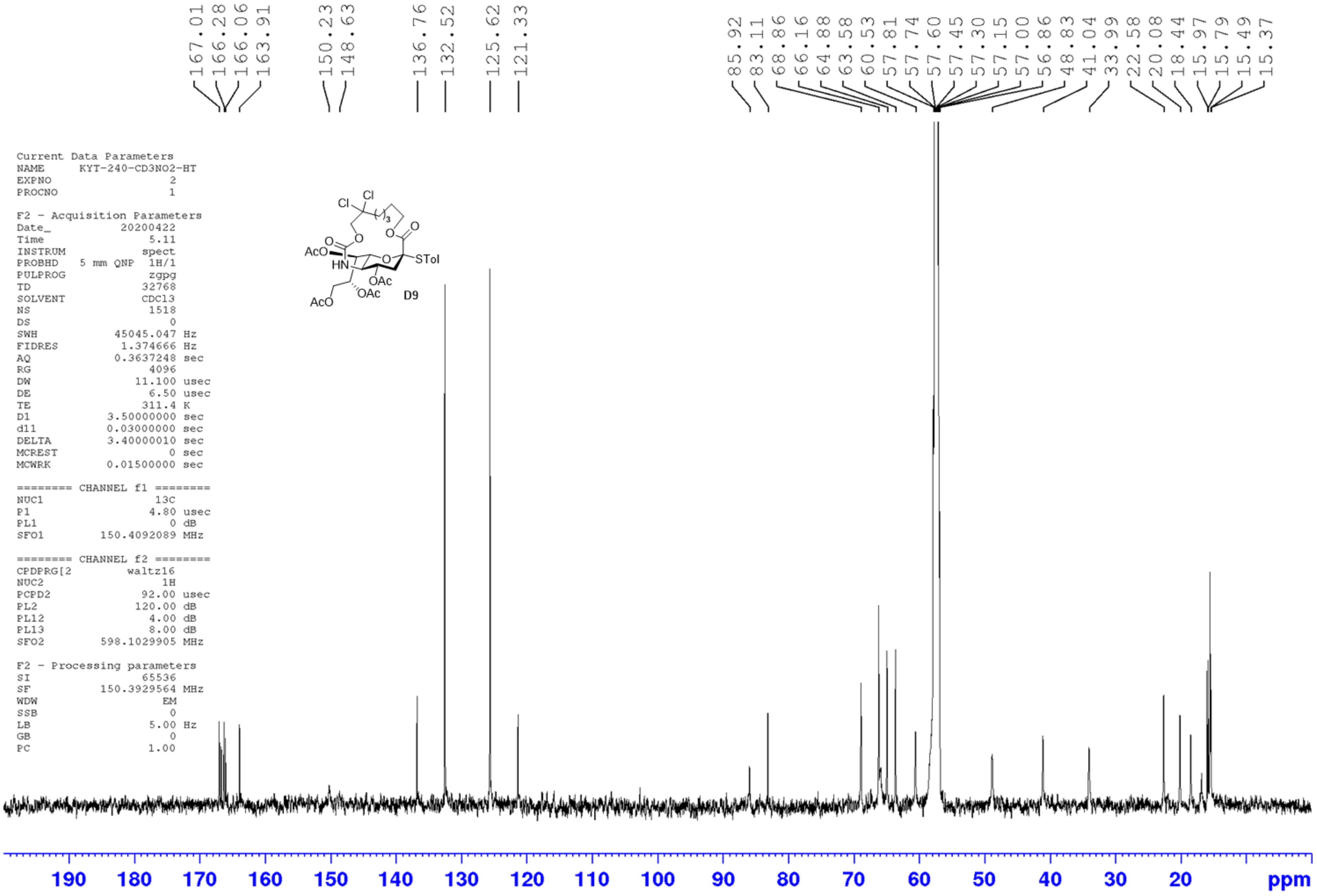

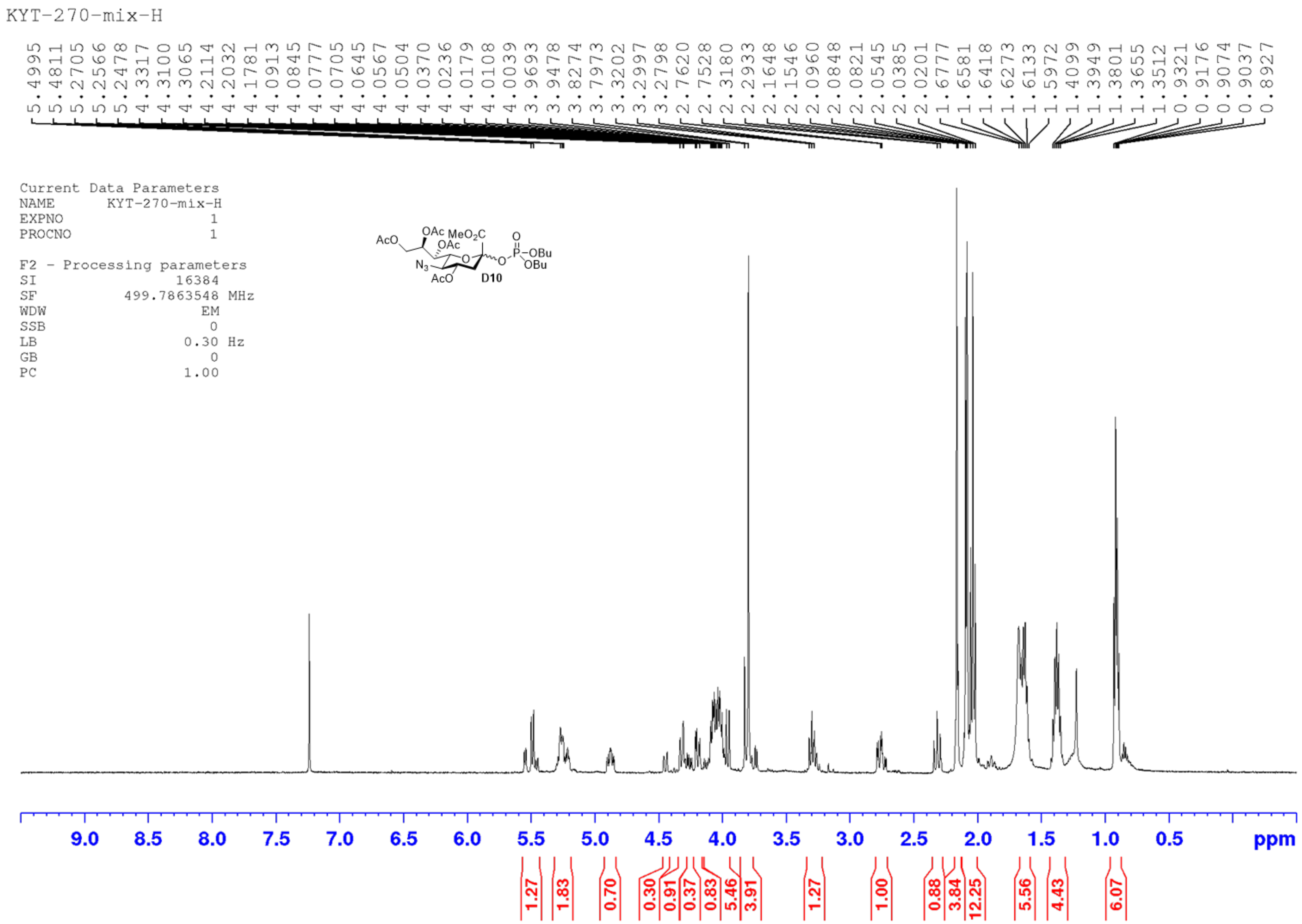
KYT-270-mix-H
5.4995 5.4811 5.2705 5.2566 5.2478 4.3317 4.3100 4.3065 4.2114 4.2032 4.1781 4.0913 4.0845 4.0777 4.0705 4.0645 4.0567 4.0504 4.0370 4.0236 4.0179 4.0108 4.0039 3.9693 3.9478 3.8274 3.7973 3.3202 3.2997 3.2798 2.7620 2.7528 2.3180 2.2933 2.1648 2.1546 2.0960 2.0848 2.0821 2.0545 2.0385 2.0201 1.6777 1.6581 1.6418 1.6273 1.6133 1.5972 1.4099 1.3949 1.3801 1.3655 1.3512 0.9321 0.9176 0.9074 0.9037 0.8927
Current Data Parameters
NAME KYT-270-mix-H
EXPNO 1
PROCNO 1
F2 - Processing parameters
SI 16384
SF 499.7863548 MHz
WDW EM
SSB 0
LB 0.30 Hz
GB 0
PC 1.00
AcO
OAc
MeO2C
OAc
O
OBu
OBu
N3
AcO
D10
9.0 8.5 8.0 7.5 7.0 6.5 6.0 5.5 5.0 4.5 4.0 3.5 3.0 2.5 2.0 1.5 1.0 0.5 ppm
1.27 1.83 0.70 0.30 0.91 0.37 0.83 5.46 3.91 1.27 1.00 0.88 3.84 12.25 5.56 4.43 6.07

KYT-270-mix-C

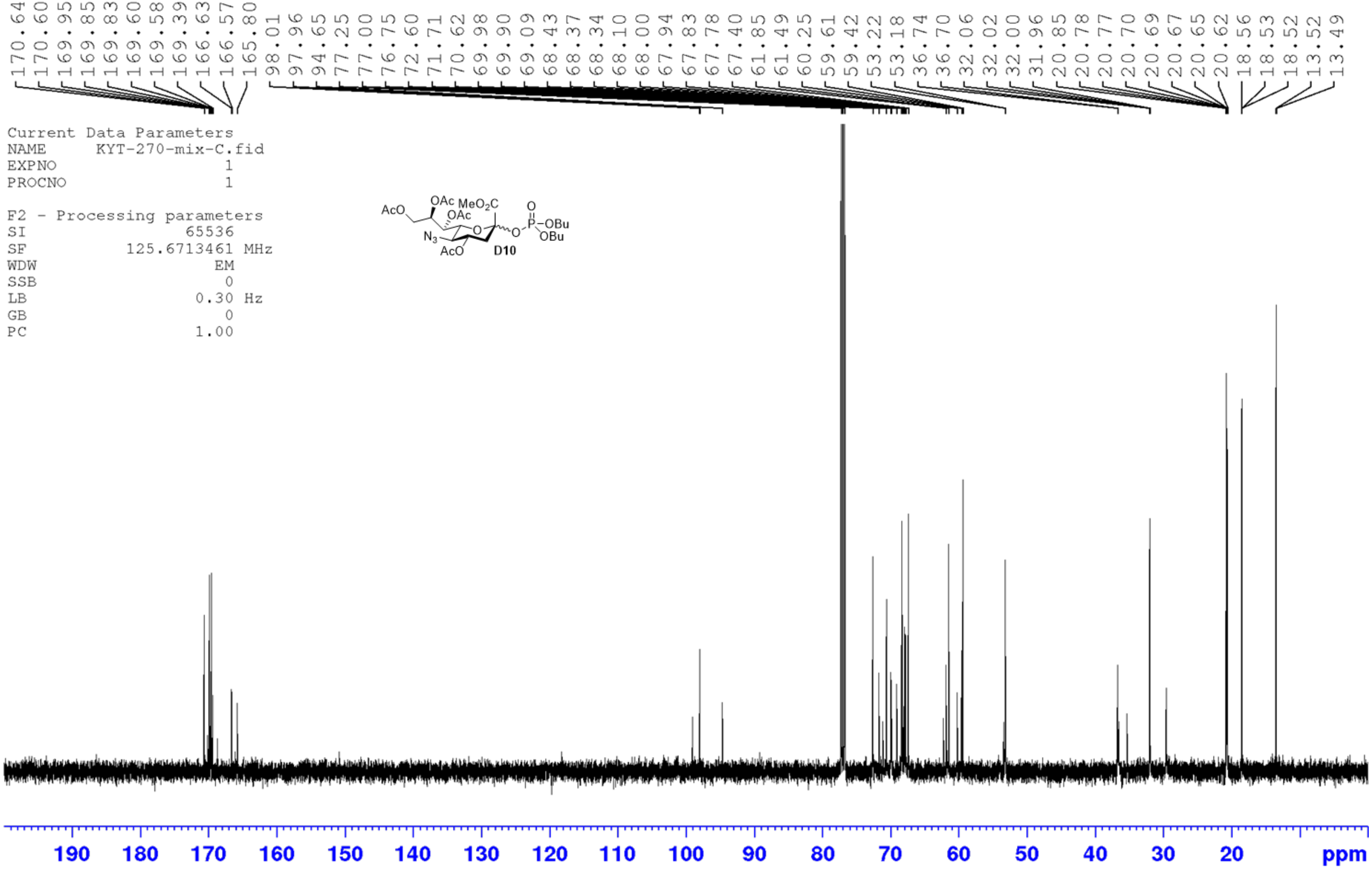
170.64
170.60
169.95
169.85
169.83
169.60
169.58
169.39
166.63
166.57
165.80
98.01
97.96
94.65
77.25
77.00
76.75
72.60
71.71
70.62
69.98
69.90
69.09
68.43
68.37
68.34
68.10
68.00
67.94
67.83
67.78
67.40
61.85
61.49
60.25
59.61
59.42
53.22
53.18
36.74
36.70
32.06
32.02
32.00
31.96
20.85
20.78
20.77
20.70
20.69
20.67
20.65
20.62
18.56
18.53
18.52
13.52
13.49
Current Data Parameters
NAME KYT-270-mix-C.fid
EXPNO 1
PROCNO 1
F2 - Processing parameters
SI 65536
SF 125.6713461 MHz
WDW EM
SSB 0
LB 0.30 Hz
GB 0
PC 1.00
AcO
OAc
MeO2C
OAc
N3
AcO
O
P
OBu
OBu
D10
190 180 170 160 150 140 130 120 110 100 90 80 70 60 50 40 30 20 ppm

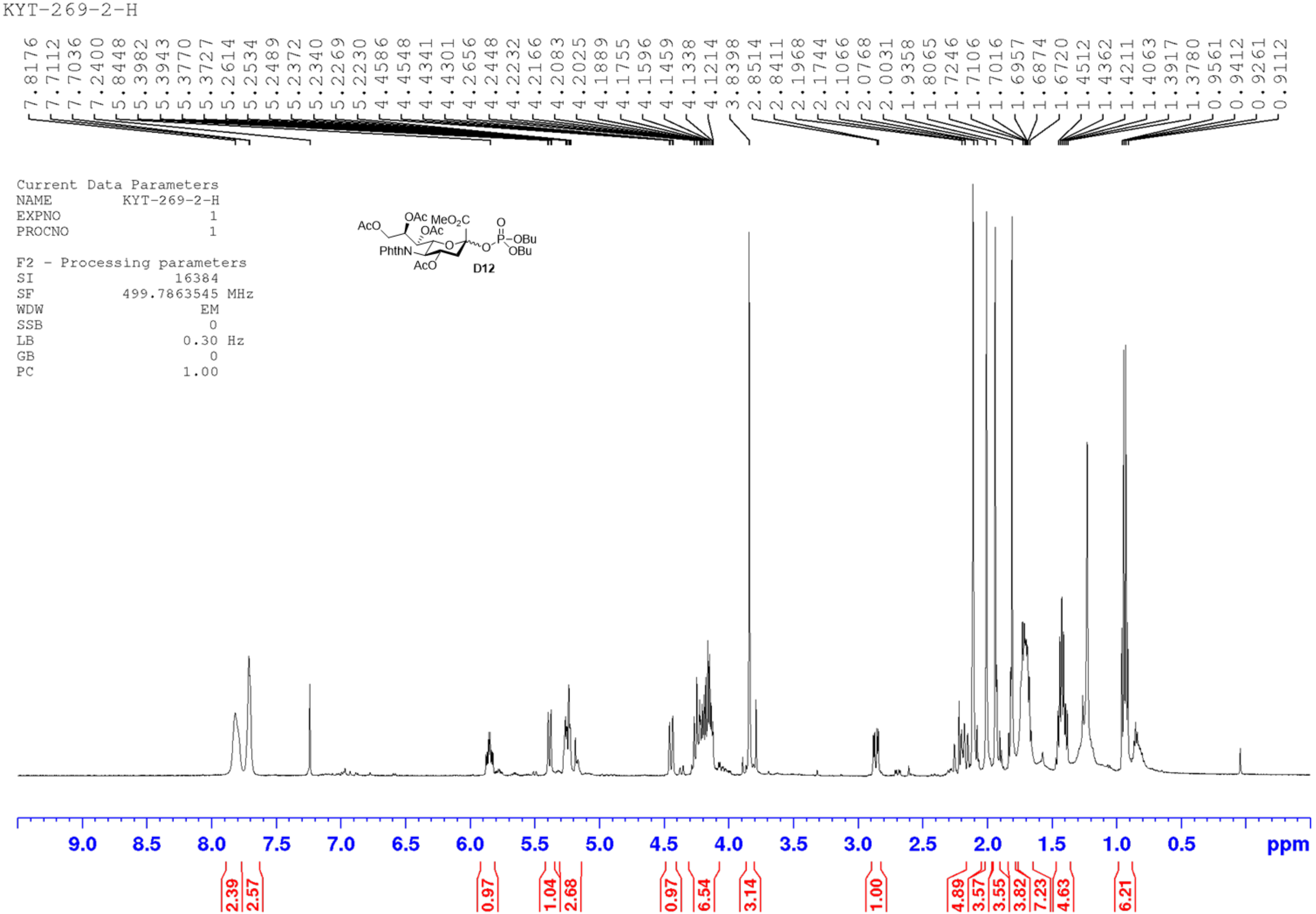
KYT-269-2-H
7.8176
7.7112
7.7036
7.2400
5.8448
5.3982
5.3943
5.3770
5.3727
5.2614
5.2534
5.2489
5.2372
5.2340
5.2269
5.2230
4.4586
4.4548
4.4341
4.4301
4.2656
4.2448
4.2232
4.2166
4.2083
4.2025
4.1889
4.1755
4.1596
4.1459
4.1338
4.1214
3.8398
2.8514
2.8411
2.1968
2.1744
2.1066
2.0768
2.0031
1.9358
1.8065
1.7246
1.7106
1.7016
1.6957
1.6874
1.6720
1.4512
1.4362
1.4211
1.4063
1.3917
1.3780
0.9561
0.9412
0.9261
0.9112
Current Data Parameters
NAME KYT-269-2-H
EXPNO 1
PROCNO 1
F2 - Processing parameters
SI 16384
SF 499.7863545 MHz
WDW EM
SSB 0
LB 0.30 Hz
GB 0
PC 1.00
AcO
OAc
MeO2C
OAc
O
P
OBu
OBu
PhthN
AcO
D12
9.0
8.5
8.0
7.5
7.0
6.5
6.0
5.5
5.0
4.5
4.0
3.5
3.0
2.5
2.0
1.5
1.0
0.5
ppm
2.39
2.57
0.97
1.04
2.68
0.97
6.54
3.14
1.00
4.89
3.57
3.55
3.82
7.23
4.63
6.21

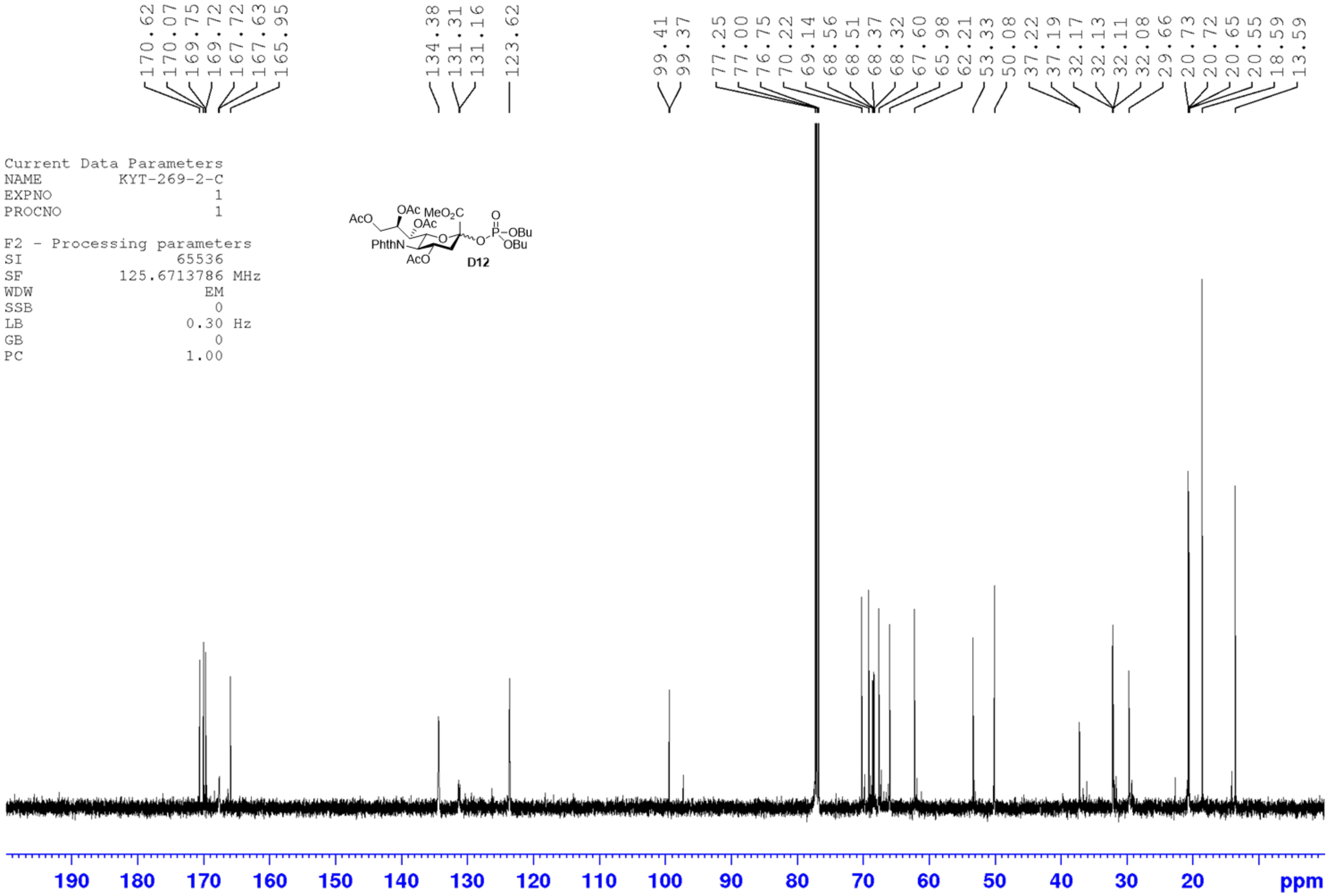
170.62
170.07
169.75
169.72
167.72
167.63
165.95
134.38
131.31
131.16
123.62
99.41
99.37
77.25
77.00
76.75
70.22
69.14
68.56
68.51
68.37
68.32
67.60
65.98
62.21
53.33
50.08
37.22
37.19
32.17
32.13
32.11
32.08
29.66
20.73
20.72
20.65
20.55
18.59
13.59
Current Data Parameters
NAME KYT-269-2-C
EXPNO 1
PROCNO 1
F2 - Processing parameters
SI 65536
SF 125.6713786 MHz
WDW EM
SSB 0
LB 0.30 Hz
GB 0
PC 1.00
AcO
OAc
MeO2C
OAc
O
P
OBu
OBu
PhthN
O
O
AcO
D12
190
180
170
160
150
140
130
120
110
100
90
80
70
60
50
40
30
20
ppm

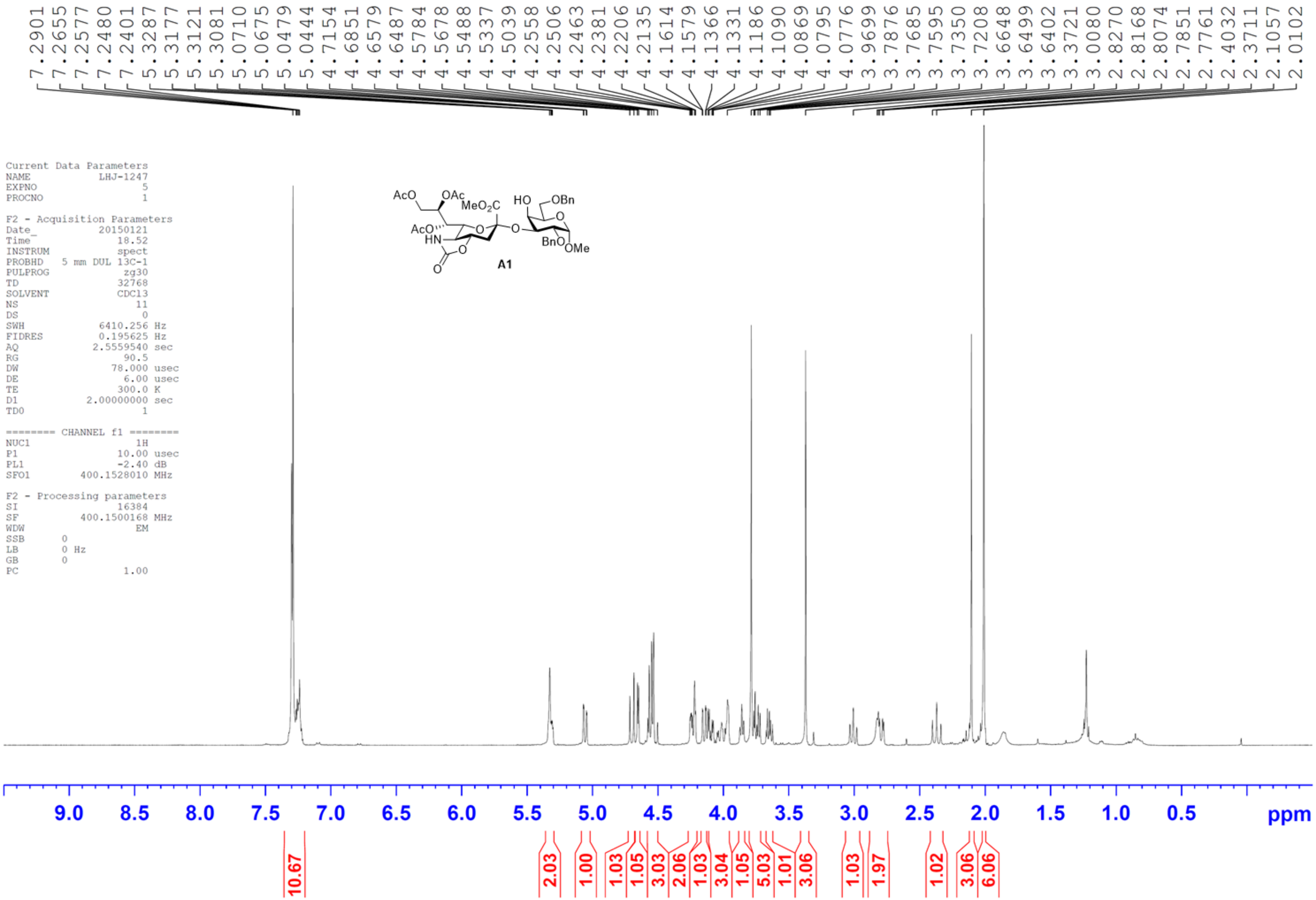
7.2901
7.2655
7.2577
7.2480
7.2401
5.3287
5.3177
5.3121
5.3081
5.0710
5.0675
5.0479
5.0444
4.7154
4.6851
4.6579
4.6487
4.5784
4.5678
4.5488
4.5337
4.5039
4.2558
4.2506
4.2463
4.2381
4.2206
4.2135
4.1614
4.1579
4.1366
4.1331
4.1186
4.1090
4.0869
4.0795
4.0776
3.9699
3.7876
3.7685
3.7595
3.7350
3.7208
3.6648
3.6499
3.6402
3.3721
3.0080
2.8270
2.8168
2.8074
2.7851
2.7761
2.4032
2.3711
2.1057
2.0102
Current Data Parameters
NAME LHJ-1247
EXPNO 5
PROCNO 1
F2 - Acquisition Parameters
Date_ 20150121
Time 18.52
INSTRUM spect
PROBHD 5 mm DUL 13C-1
PULPROG zg30
TD 32768
SOLVENT CDCl3
NS 11
DS 0
SWH 6410.256 Hz
FIDRES 0.195625 Hz
AQ 2.5559540 sec
RG 90.5
DW 78.000 usec
DE 6.00 usec
TE 300.0 K
D1 2.00000000 sec
TD0 1
======== CHANNEL f1 ========
NUC1 1H
P1 10.00 usec
PL1 -2.40 dB
SFO1 400.1528010 MHz
F2 - Processing parameters
SI 16384
SF 400.1500168 MHz
WDW EM
SSB 0
LB 0 Hz
GB 0
PC 1.00
AcO
OAc
MeO2C
HO
OBn
AcO
HN
O
O
O
O
BnO
OMe
O
A1
9.0
8.5
8.0
7.5
7.0
6.5
6.0
5.5
5.0
4.5
4.0
3.5
3.0
2.5
2.0
1.5
1.0
0.5
ppm
10.67
2.03
1.00
1.03
1.05
3.03
2.06
1.03
3.04
1.05
5.03
1.01
3.06
1.03
1.97
1.02
3.06
6.06

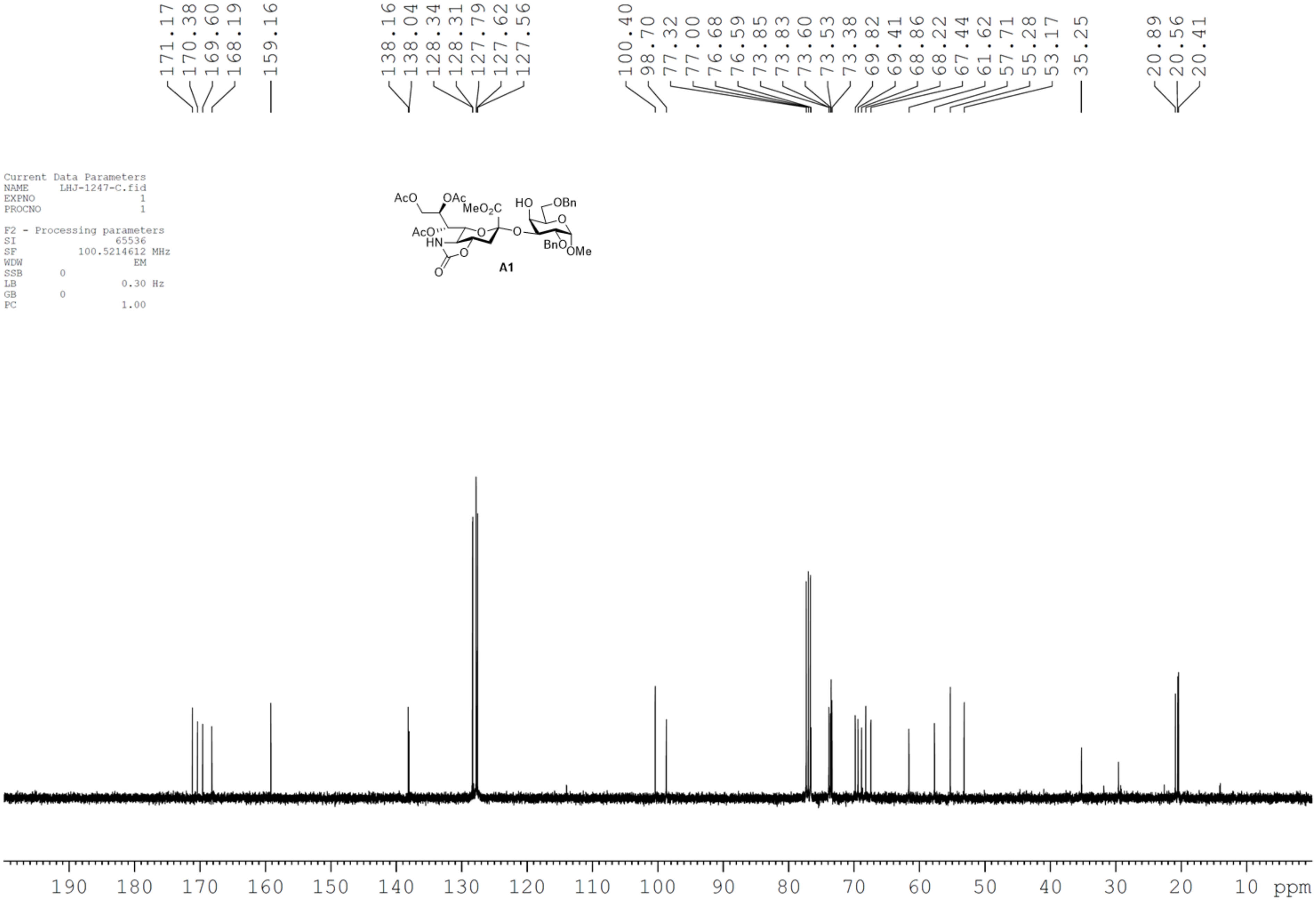
171.17
170.38
169.60
168.19
159.16
138.16
138.04
128.34
128.31
127.79
127.62
127.56
100.40
98.70
77.32
77.00
76.68
76.59
73.85
73.83
73.60
73.53
73.38
69.82
69.41
68.86
68.22
67.44
61.62
57.71
55.28
53.17
35.25
20.89
20.56
20.41
Current Data Parameters
NAME LHJ-1247-C.fid
EXPNO 1
PROCNO 1
F2 - Processing parameters
SI 65536
SF 100.5214612 MHz
WDW EM
SSB 0
LB 0.30 Hz
GB 0
PC 1.00
AcO
OAc
MeO2C
HO
OBn
AcO
HN
O
O
O
O
O
BnO
OMe
A1
190 180 170 160 150 140 130 120 110 100 90 80 70 60 50 40 30 20 10 ppm

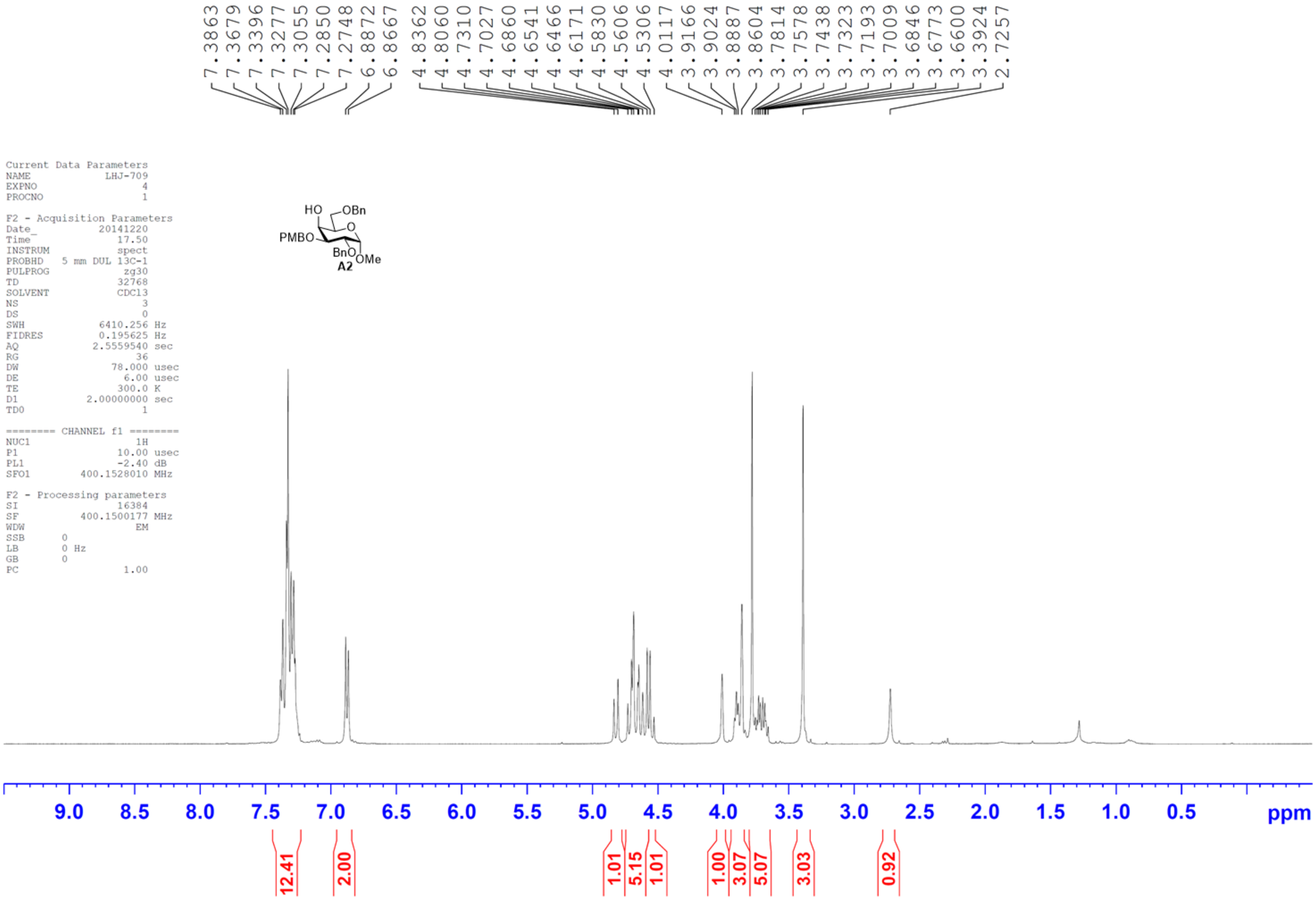


```
Current Data Parameters
NAME            LHJ-709
EXPNO                 4
PROCNO                1

F2 - Acquisition Parameters
Date_          20141220
Time              17.50
INSTRUM           spect
PROBHD   5 mm DUL 13C-1
PULPROG            zg30
TD                32768
SOLVENT           CDCl3
NS                    3
DS                    0
SWH            6410.256 Hz
FIDRES         0.195625 Hz
AQ            2.5559540 sec
RG                   36
DW               78.000 usec
DE                 6.00 usec
TE                300.0 K
D1           2.00000000 sec
TD0                   1

======== CHANNEL f1 ========
NUC1                 1H
P1                10.00 usec
PL1               -2.40 dB
SFO1        400.1528010 MHz

F2 - Processing parameters
SI                16384
SF          400.1500177 MHz
WDW                  EM
SSB      0
LB       0 Hz
GB       0
PC                 1.00
```

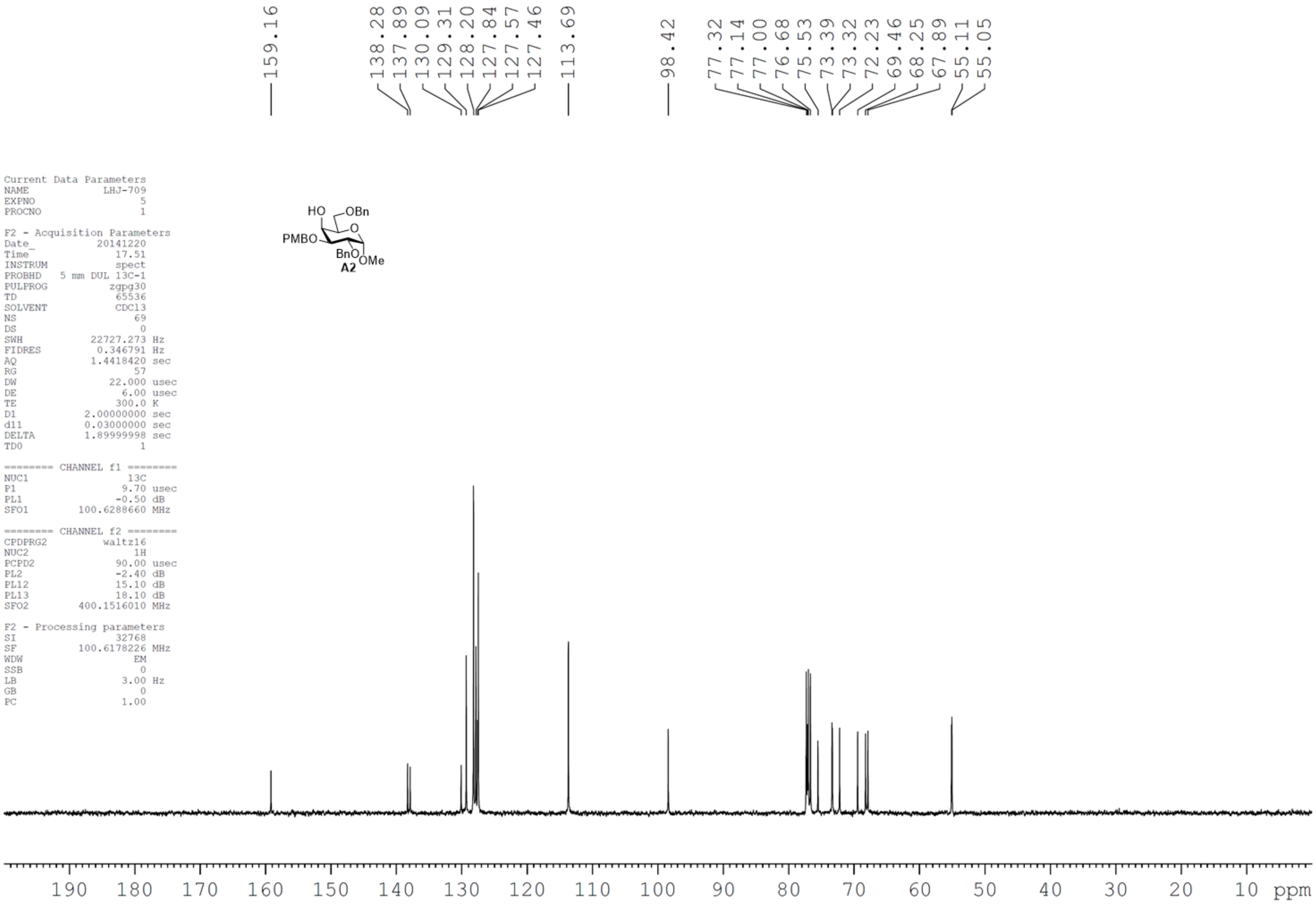
159.16
138.28
137.89
130.09
129.31
128.20
127.84
127.57
127.46
113.69
98.42
77.32
77.14
77.00
76.68
75.53
73.39
73.32
72.23
69.46
68.25
67.89
55.11
55.05
Current Data Parameters
NAME LHJ-709
EXPNO 5
PROCNO 1
F2 - Acquisition Parameters
Date_ 20141220
Time 17.51
INSTRUM spect
PROBHD 5 mm DUL 13C-1
PULPROG zgpg30
TD 65536
SOLVENT CDCl3
NS 69
DS 0
SWH 22727.273 Hz
FIDRES 0.346791 Hz
AQ 1.4418420 sec
RG 57
DW 22.000 usec
DE 6.00 usec
TE 300.0 K
D1 2.00000000 sec
d11 0.03000000 sec
DELTA 1.89999998 sec
TD0 1
======== CHANNEL f1 ========
NUC1 13C
P1 9.70 usec
PL1 -0.50 dB
SFO1 100.6288660 MHz
======== CHANNEL f2 ========
CPDPRG2 waltz16
NUC2 1H
PCPD2 90.00 usec
PL2 -2.40 dB
PL12 15.10 dB
PL13 18.10 dB
SFO2 400.1516010 MHz
F2 - Processing parameters
SI 32768
SF 100.6178226 MHz
WDW EM
SSB 0
LB 3.00 Hz
GB 0
PC 1.00
HO
OBn
PMBO
O
BnO
OMe
A2
190 180 170 160 150 140 130 120 110 100 90 80 70 60 50 40 30 20 10 ppm

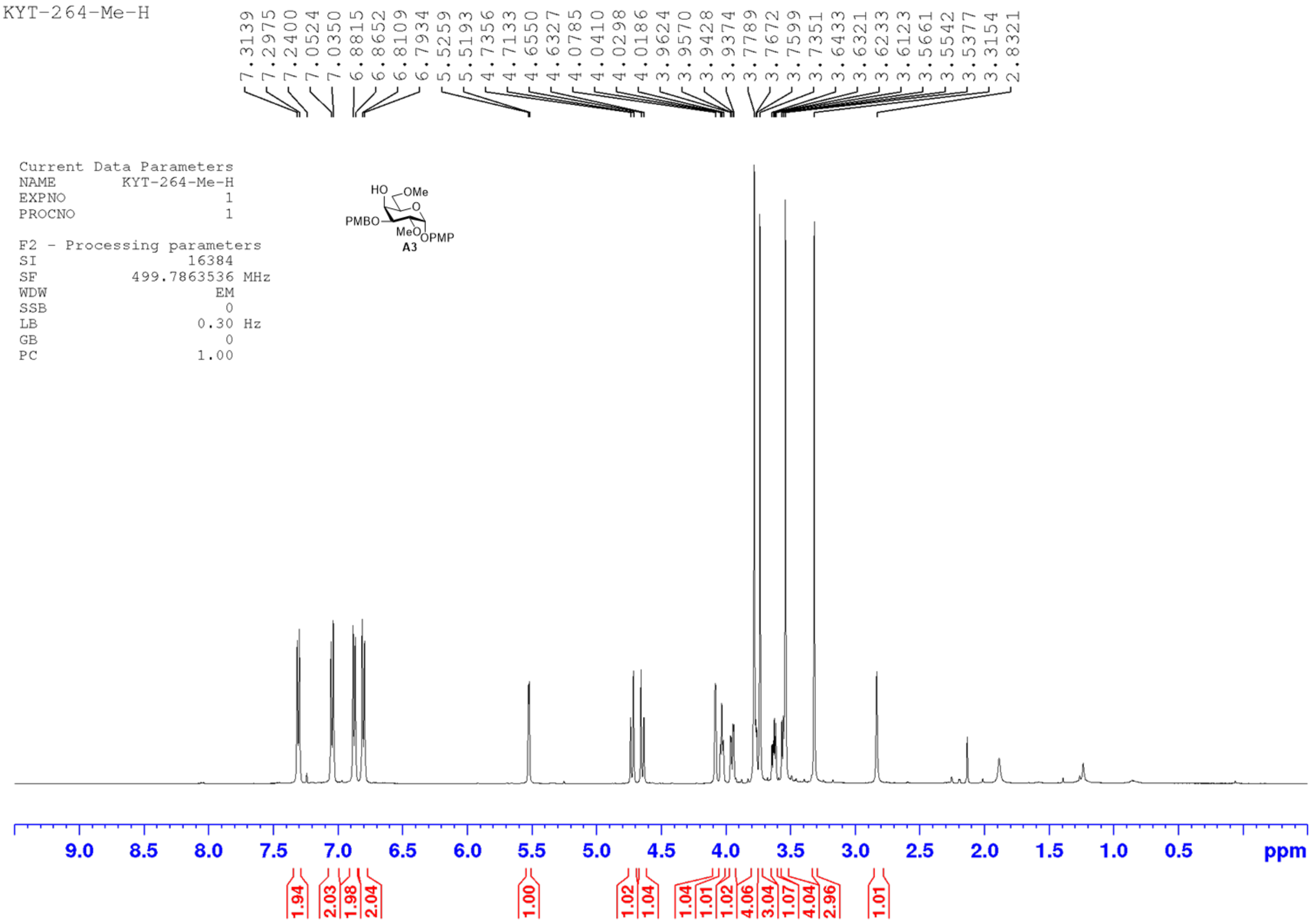
KYT-264-Me-H
7.3139
7.2975
7.2400
7.0524
7.0350
6.8815
6.8652
6.8109
6.7934
5.5259
5.5193
4.7356
4.7133
4.6550
4.6327
4.0785
4.0410
4.0298
4.0186
3.9624
3.9570
3.9428
3.9374
3.7789
3.7672
3.7599
3.7351
3.6433
3.6321
3.6233
3.6123
3.5661
3.5542
3.5377
3.3154
2.8321
Current Data Parameters
NAME KYT-264-Me-H
EXPNO 1
PROCNO 1
F2 - Processing parameters
SI 16384
SF 499.7863536 MHz
WDW EM
SSB 0
LB 0.30 Hz
GB 0
PC 1.00
HO
OMe
O
PMBO
MeO
OPMP
A3
9.0
8.5
8.0
7.5
7.0
6.5
6.0
5.5
5.0
4.5
4.0
3.5
3.0
2.5
2.0
1.5
1.0
0.5
ppm
1.94
2.03
1.98
2.04
1.00
1.02
1.04
1.04
1.01
1.02
4.06
3.04
1.07
4.04
2.96
1.01

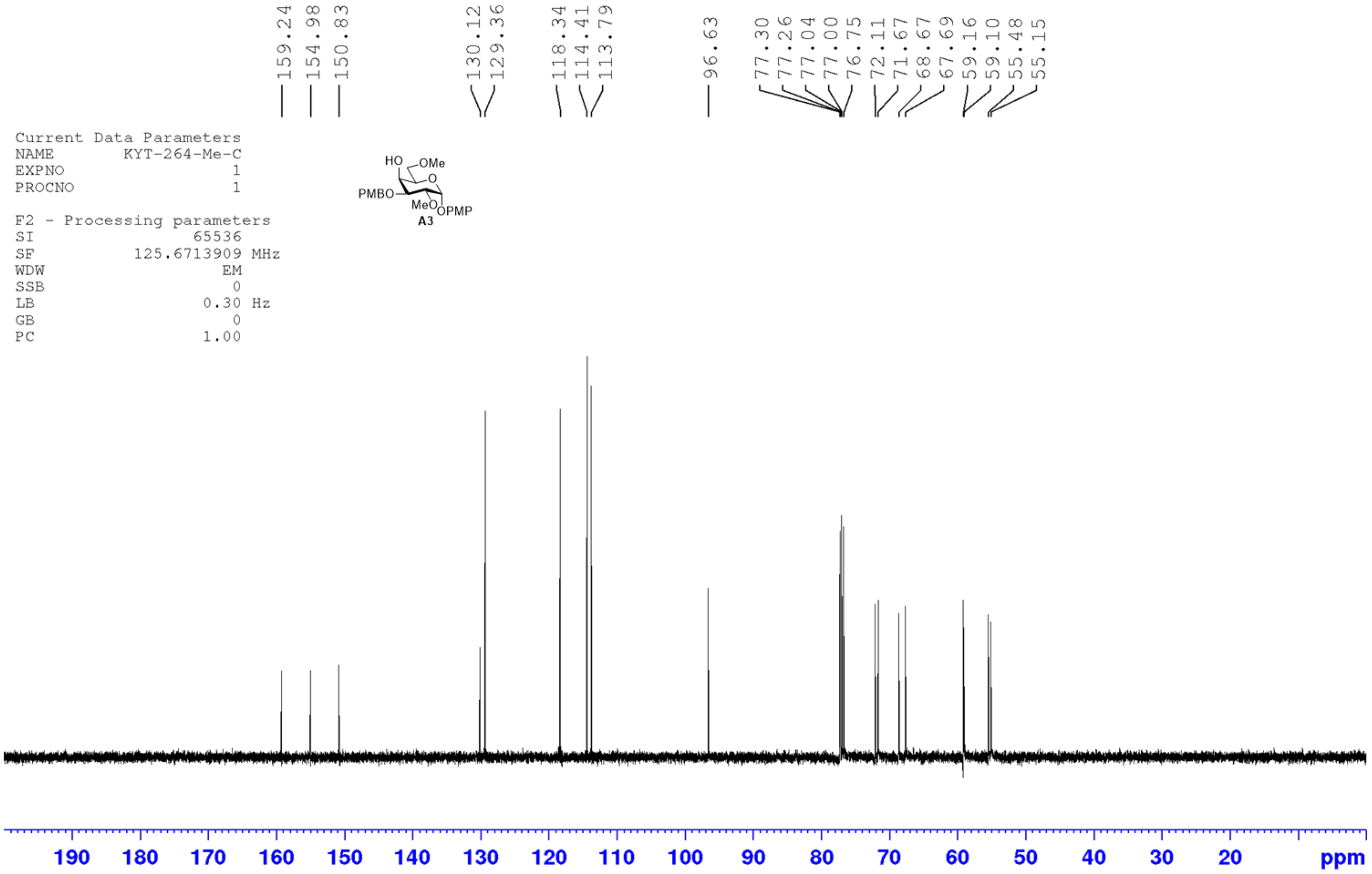
159.24
154.98
150.83
130.12
129.36
118.34
114.41
113.79
96.63
77.30
77.26
77.04
77.00
76.75
72.11
71.67
68.67
67.69
59.16
59.10
55.48
55.15
Current Data Parameters
NAME KYT-264-Me-C
EXPNO 1
PROCNO 1
F2 - Processing parameters
SI 65536
SF 125.6713909 MHz
WDW EM
SSB 0
LB 0.30 Hz
GB 0
PC 1.00
HO
OMe
O
PMBO
MeO
OPMP
A3
190
180
170
160
150
140
130
120
110
100
90
80
70
60
50
40
30
20
ppm

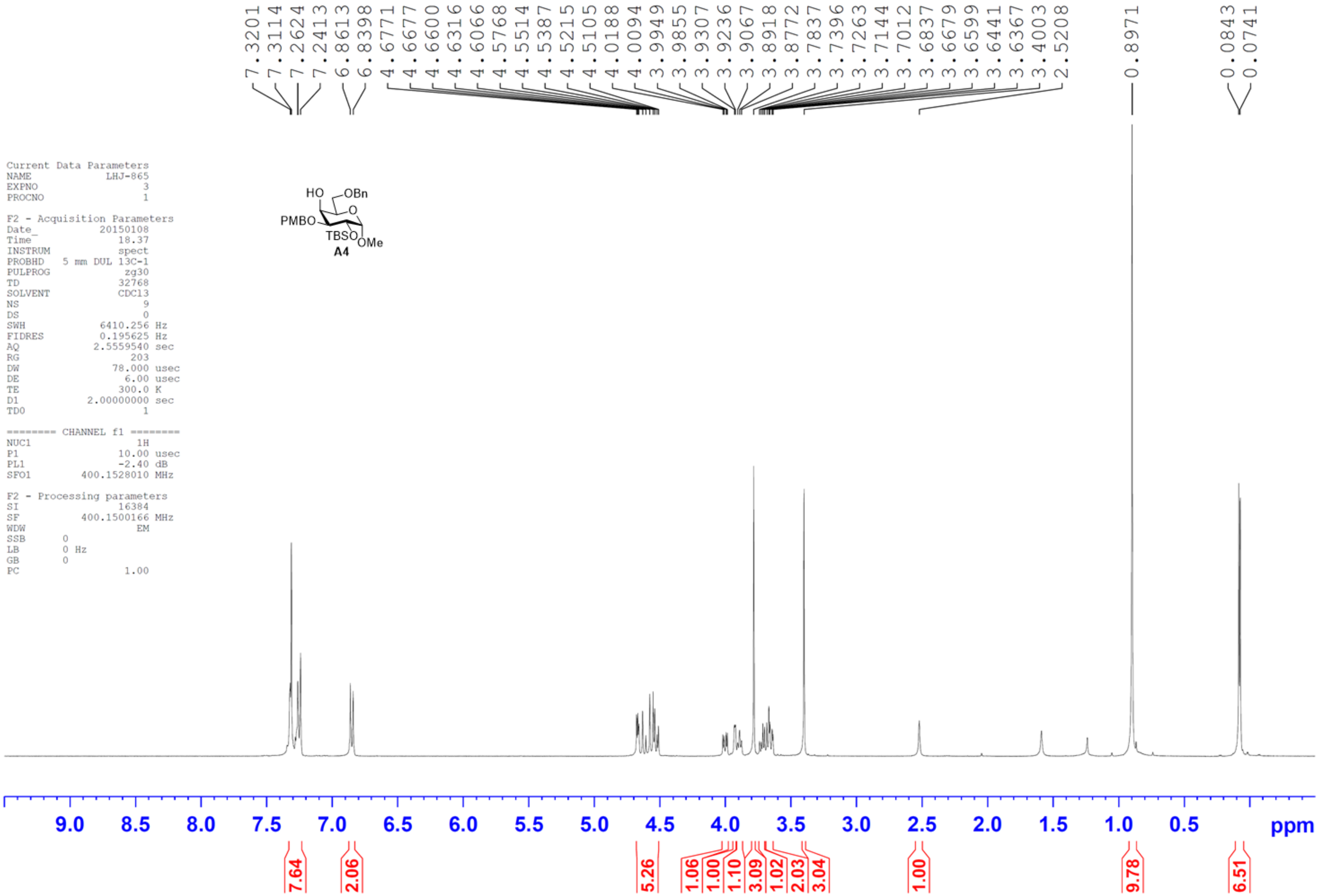
7.3201 7.3114 7.2624 7.2413 6.8613 6.8398 4.6771 4.6677 4.6600 4.6316 4.6066 4.5768 4.5514 4.5387 4.5215 4.5105 4.0188 4.0094 3.9949 3.9855 3.9307 3.9236 3.9067 3.8918 3.8772 3.7837 3.7396 3.7263 3.7144 3.7012 3.6837 3.6679 3.6599 3.6441 3.6367 3.4003 2.5208 0.8971 0.0843 0.0741
Current Data Parameters
NAME LHJ-865
EXPNO 3
PROCNO 1
F2 - Acquisition Parameters
Date_ 20150108
Time 18.37
INSTRUM spect
PROBHD 5 mm DUL 13C-1
PULPROG zg30
TD 32768
SOLVENT CDCl3
NS 9
DS 0
SWH 6410.256 Hz
FIDRES 0.195625 Hz
AQ 2.5559540 sec
RG 203
DW 78.000 usec
DE 6.00 usec
TE 300.0 K
D1 2.00000000 sec
TD0 1
======== CHANNEL f1 ========
NUC1 1H
P1 10.00 usec
PL1 -2.40 dB
SFO1 400.1528010 MHz
F2 - Processing parameters
SI 16384
SF 400.1500166 MHz
WDW EM
SSB 0
LB 0 Hz
GB 0
PC 1.00
HO
OBn
O
PMBO
TBSO
OMe
A4
9.0 8.5 8.0 7.5 7.0 6.5 6.0 5.5 5.0 4.5 4.0 3.5 3.0 2.5 2.0 1.5 1.0 0.5 ppm
7.64 2.06 5.26 1.06 1.00 1.10 3.09 1.02 2.03 3.04 1.00 9.78 6.51

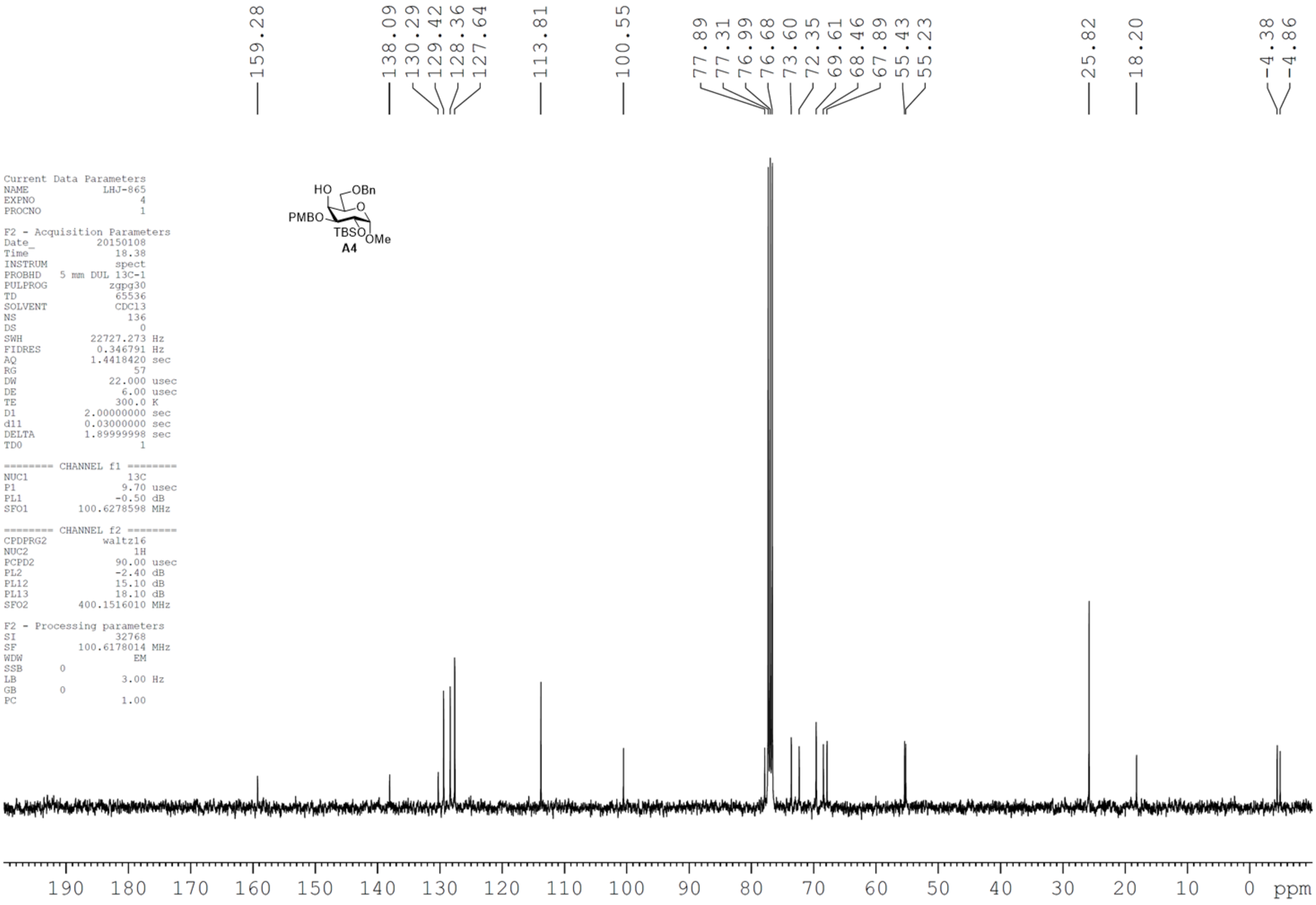
159.28
138.09
130.29
129.42
128.36
127.64
113.81
100.55
77.89
77.31
76.99
76.68
73.60
72.35
69.61
68.46
67.89
55.43
55.23
25.82
18.20
-4.38
-4.86
Current Data Parameters
NAME LHJ-865
EXPNO 4
PROCNO 1
F2 - Acquisition Parameters
Date_ 20150108
Time 18.38
INSTRUM spect
PROBHD 5 mm DUL 13C-1
PULPROG zgpg30
TD 65536
SOLVENT CDCl3
NS 136
DS 0
SWH 22727.273 Hz
FIDRES 0.346791 Hz
AQ 1.4418420 sec
RG 57
DW 22.000 usec
DE 6.00 usec
TE 300.0 K
D1 2.00000000 sec
d11 0.03000000 sec
DELTA 1.89999998 sec
TD0 1
======== CHANNEL f1 ========
NUC1 13C
P1 9.70 usec
PL1 -0.50 dB
SFO1 100.6278598 MHz
======== CHANNEL f2 ========
CPDPRG2 waltz16
NUC2 1H
PCPD2 90.00 usec
PL2 -2.40 dB
PL12 15.10 dB
PL13 18.10 dB
SFO2 400.1516010 MHz
F2 - Processing parameters
SI 32768
SF 100.6178014 MHz
WDW EM
SSB 0
LB 3.00 Hz
GB 0
PC 1.00
HO
OBn
O
PMBO
TBSO
OMe
A4
190 180 170 160 150 140 130 120 110 100 90 80 70 60 50 40 30 20 10 0 ppm

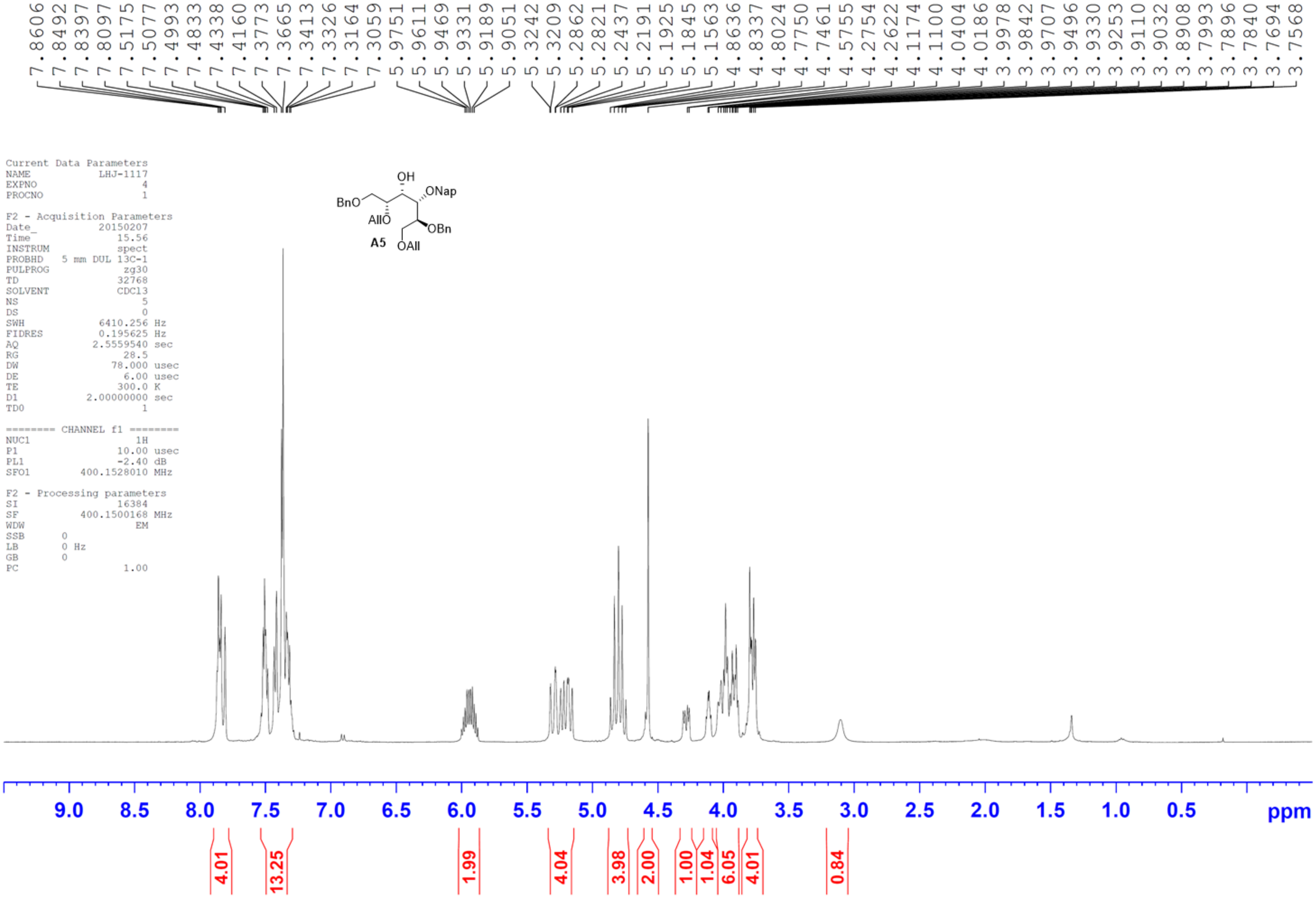

7.8606 7.8492 7.8397 7.8097 7.5175 7.5077 7.4993 7.4833 7.4338 7.4160 7.3773 7.3665 7.3413 7.3326 7.3164 7.3059 5.9751 5.9611 5.9469 5.9331 5.9189 5.9051 5.3242 5.3209 5.2862 5.2821 5.2437 5.2191 5.1925 5.1845 5.1563 4.8636 4.8337 4.8024 4.7750 4.7461 4.5755 4.2754 4.2622 4.1174 4.1100 4.0404 4.0186 3.9978 3.9842 3.9707 3.9496 3.9330 3.9253 3.9110 3.9032 3.8908 3.7993 3.7896 3.7840 3.7694 3.7568
Current Data Parameters
NAME LHJ-1117
EXPNO 4
PROCNO 1
F2 - Acquisition Parameters
Date_ 20150207
Time 15.56
INSTRUM spect
PROBHD 5 mm DUL 13C-1
PULPROG zg30
TD 32768
SOLVENT CDCl3
NS 5
DS 0
SWH 6410.256 Hz
FIDRES 0.195625 Hz
AQ 2.5559540 sec
RG 28.5
DW 78.000 usec
DE 6.00 usec
TE 300.0 K
D1 2.00000000 sec
TD0 1
======== CHANNEL f1 ========
NUC1 1H
P1 10.00 usec
PL1 -2.40 dB
SFO1 400.1528010 MHz
F2 - Processing parameters
SI 16384
SF 400.1500168 MHz
WDW EM
SSB 0
LB 0 Hz
GB 0
PC 1.00
OH
ONap
BnO
AllO
OBn
A5
OAll
9.0 8.5 8.0 7.5 7.0 6.5 6.0 5.5 5.0 4.5 4.0 3.5 3.0 2.5 2.0 1.5 1.0 0.5 ppm
4.01 13.25 1.99 4.04 3.98 2.00 1.00 1.04 6.05 4.01 0.84

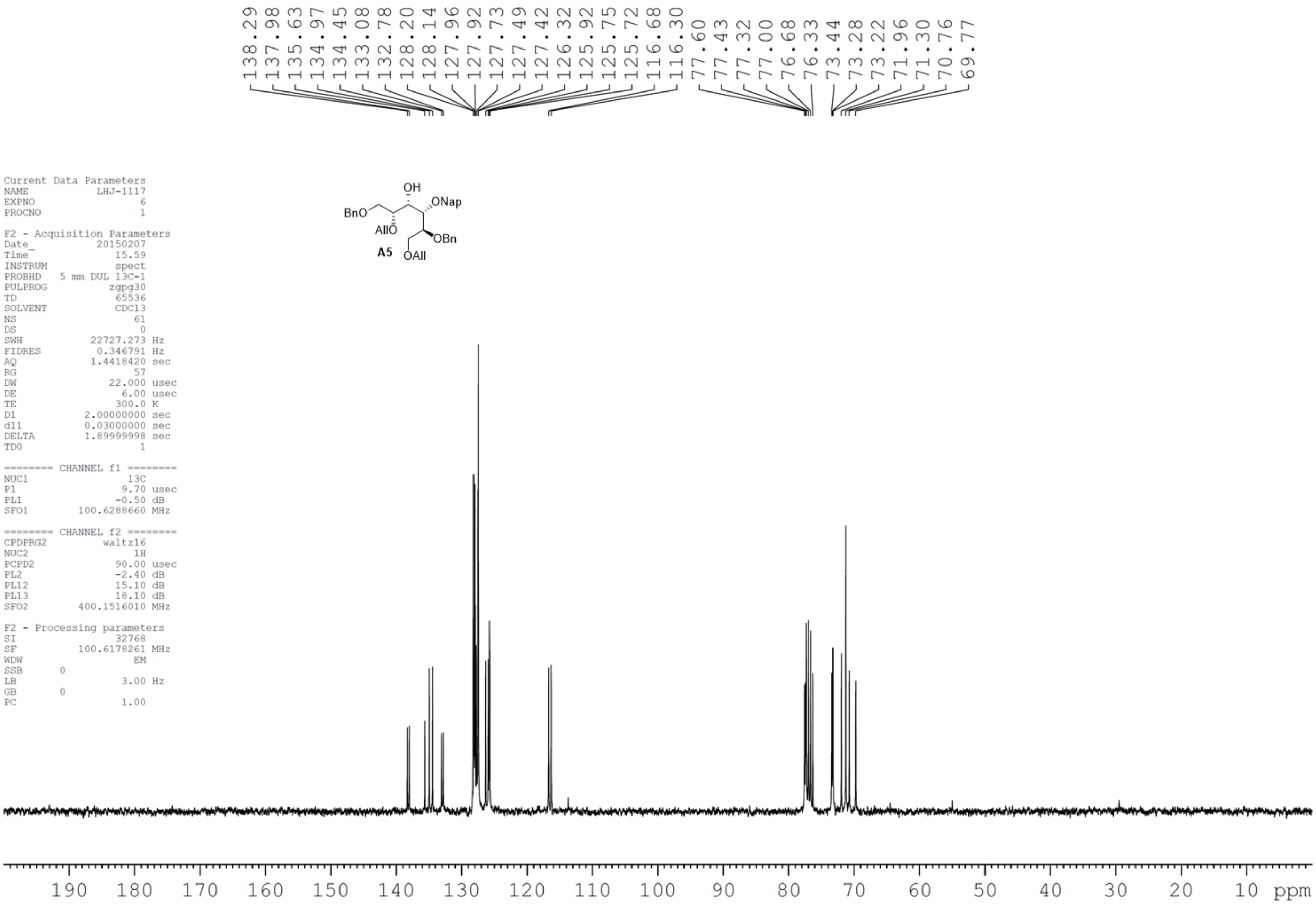
138.29
137.98
135.63
134.97
134.45
133.08
132.78
128.20
128.14
127.96
127.92
127.73
127.49
127.42
126.32
125.92
125.75
125.72
116.68
116.30
77.60
77.43
77.32
77.00
76.68
76.33
73.44
73.28
73.22
71.96
71.30
70.76
69.77
Current Data Parameters
NAME LHJ-1117
EXPNO 6
PROCNO 1
F2 - Acquisition Parameters
Date_ 20150207
Time 15.59
INSTRUM spect
PROBHD 5 mm DUL 13C-1
PULPROG zgpg30
TD 65536
SOLVENT CDCl3
NS 61
DS 0
SWH 22727.273 Hz
FIDRES 0.346791 Hz
AQ 1.4418420 sec
RG 57
DW 22.000 usec
DE 6.00 usec
TE 300.0 K
D1 2.00000000 sec
d11 0.03000000 sec
DELTA 1.89999998 sec
TD0 1
======== CHANNEL f1 ========
NUC1 13C
P1 9.70 usec
PL1 -0.50 dB
SFO1 100.6288660 MHz
======== CHANNEL f2 ========
CPDPRG2 waltz16
NUC2 1H
PCPD2 90.00 usec
PL2 -2.40 dB
PL12 15.10 dB
PL13 18.10 dB
SFO2 400.1516010 MHz
F2 - Processing parameters
SI 32768
SF 100.6178261 MHz
WDW EM
SSB 0
LB 3.00 Hz
GB 0
PC 1.00
OH
ONap
BnO
AllO
OBn
A5
OAll
190 180 170 160 150 140 130 120 110 100 90 80 70 60 50 40 30 20 10 ppm

KYT-152-20190109

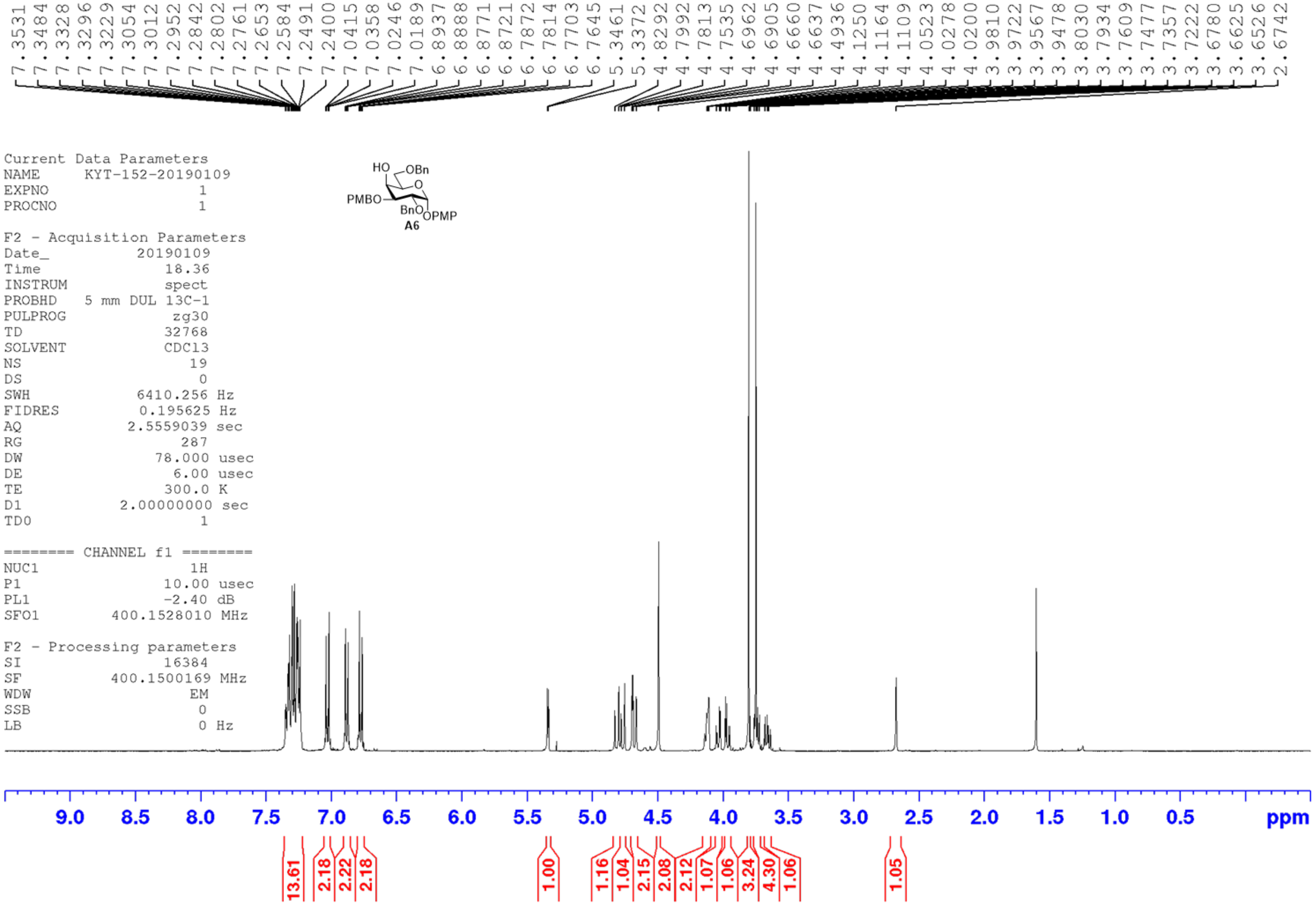

Current Data Parameters
NAME KYT-152-20190109
EXPNO 1
PROCNO 1

F2 - Acquisition Parameters
Date_ 20190109
Time 18.36
INSTRUM spect
PROBHD 5 mm DUL 13C-1
PULPROG zg30
TD 32768
SOLVENT CDCl3
NS 19
DS 0
SWH 6410.256 Hz
FIDRES 0.195625 Hz
AQ 2.5559039 sec
RG 287
DW 78.000 usec
DE 6.00 usec
TE 300.0 K
D1 2.00000000 sec
TD0 1

======== CHANNEL f1 ========
NUC1 1H
P1 10.00 usec
PL1 -2.40 dB
SFO1 400.1528010 MHz

F2 - Processing parameters
SI 16384
SF 400.1500169 MHz
WDW EM
SSB 0
LB 0 Hz

A6

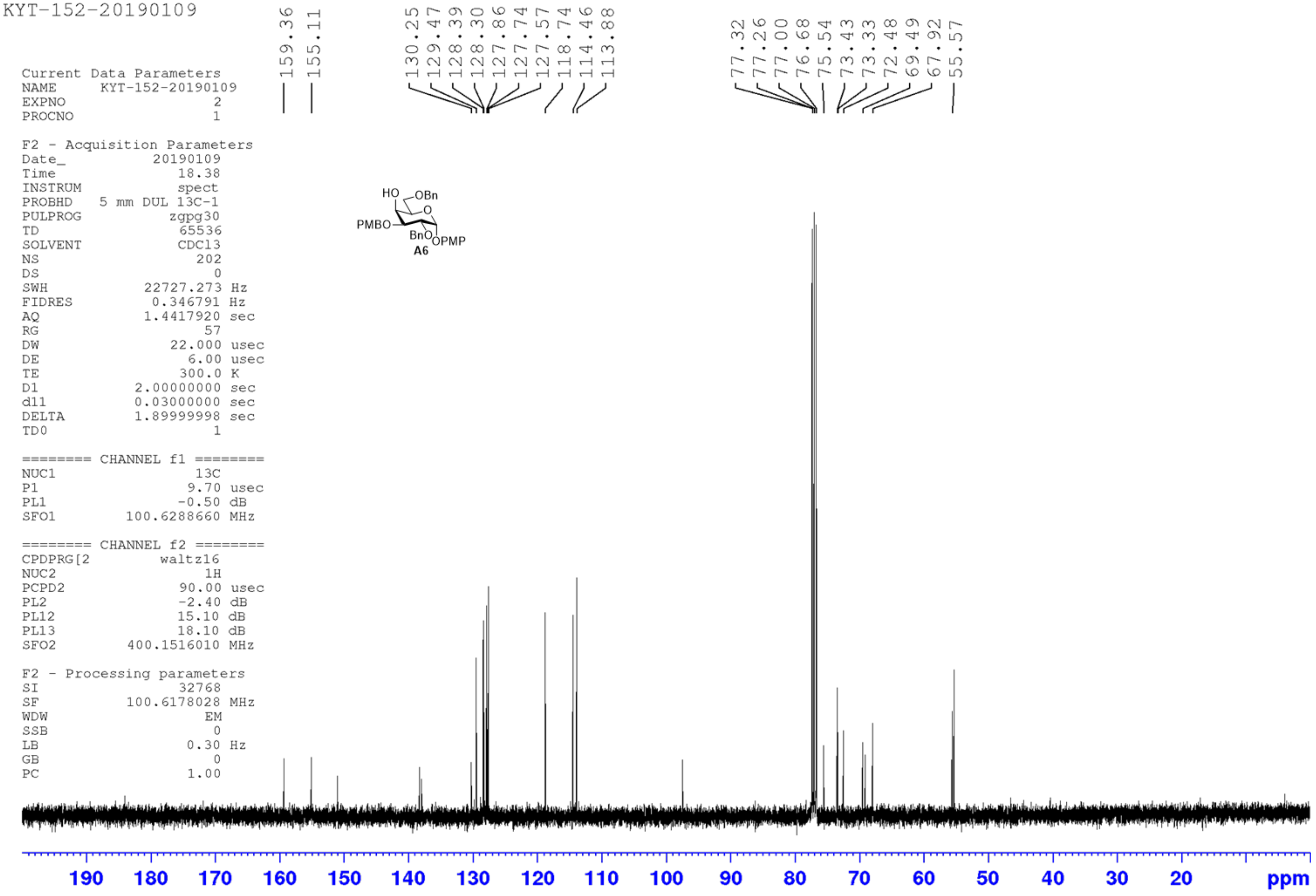
KYT-152-20190109
Current Data Parameters
NAME KYT-152-20190109
EXPNO 2
PROCNO 1
F2 - Acquisition Parameters
Date_ 20190109
Time 18.38
INSTRUM spect
PROBHD 5 mm DUL 13C-1
PULPROG zgpg30
TD 65536
SOLVENT CDCl3
NS 202
DS 0
SWH 22727.273 Hz
FIDRES 0.346791 Hz
AQ 1.4417920 sec
RG 57
DW 22.000 usec
DE 6.00 usec
TE 300.0 K
D1 2.00000000 sec
d11 0.03000000 sec
DELTA 1.89999998 sec
TD0 1
======== CHANNEL f1 ========
NUC1 13C
P1 9.70 usec
PL1 -0.50 dB
SFO1 100.6288660 MHz
======== CHANNEL f2 ========
CPDPRG[2 waltz16
NUC2 1H
PCPD2 90.00 usec
PL2 -2.40 dB
PL12 15.10 dB
PL13 18.10 dB
SFO2 400.1516010 MHz
F2 - Processing parameters
SI 32768
SF 100.6178028 MHz
WDW EM
SSB 0
LB 0.30 Hz
GB 0
PC 1.00
159.36
155.11
130.25
129.47
128.39
128.30
127.86
127.74
127.57
118.74
114.46
113.88
77.32
77.26
77.00
76.68
75.54
73.43
73.33
72.48
69.49
67.92
55.57
HO
OBn
O
PMBO
BnO
OPMP
A6
190
180
170
160
150
140
130
120
110
100
90
80
70
60
50
40
30
20
ppm

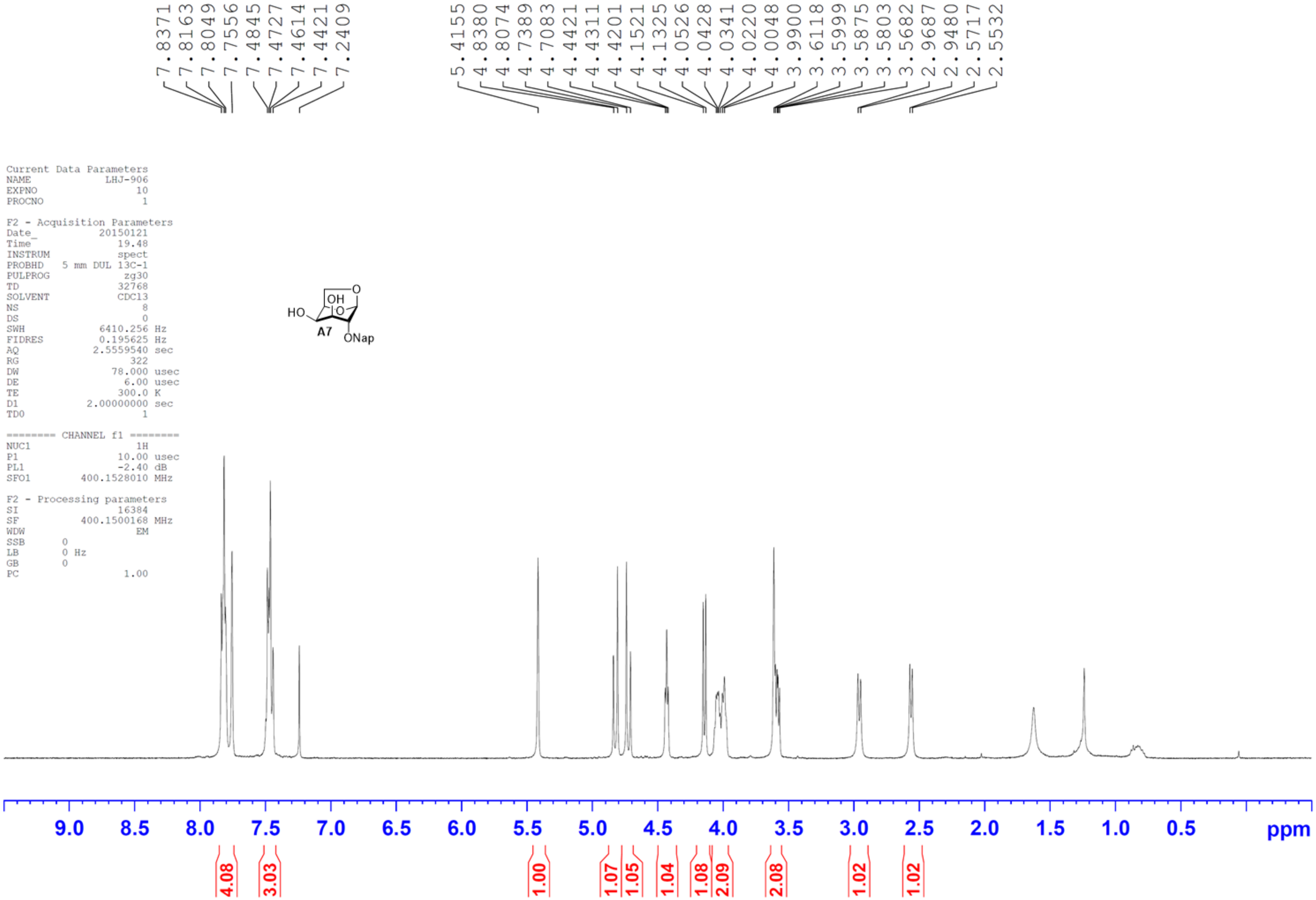

7.8371
7.8163
7.8049
7.7556
7.4845
7.4727
7.4614
7.4421
7.2409
5.4155
4.8380
4.8074
4.7389
4.7083
4.4421
4.4311
4.4201
4.1521
4.1325
4.0526
4.0428
4.0341
4.0220
4.0048
3.9900
3.6118
3.5999
3.5875
3.5803
3.5682
2.9687
2.9480
2.5717
2.5532
Current Data Parameters
NAME LHJ-906
EXPNO 10
PROCNO 1
F2 - Acquisition Parameters
Date_ 20150121
Time 19.48
INSTRUM spect
PROBHD 5 mm DUL 13C-1
PULPROG zg30
TD 32768
SOLVENT CDCl3
NS 8
DS 0
SWH 6410.256 Hz
FIDRES 0.195625 Hz
AQ 2.5559540 sec
RG 322
DW 78.000 usec
DE 6.00 usec
TE 300.0 K
D1 2.00000000 sec
TD0 1
======== CHANNEL f1 ========
NUC1 1H
P1 10.00 usec
PL1 -2.40 dB
SFO1 400.1528010 MHz
F2 - Processing parameters
SI 16384
SF 400.1500168 MHz
WDW EM
SSB 0
LB 0 Hz
GB 0
PC 1.00
9.0
8.5
8.0
7.5
7.0
6.5
6.0
5.5
5.0
4.5
4.0
3.5
3.0
2.5
2.0
1.5
1.0
0.5
ppm
4.08
3.03
1.00
1.07
1.05
1.04
1.08
2.09
2.08
1.02
1.02
HO
OH
O
O
ONap

A7

```
Current Data Parameters
NAME             LHJ-906
EXPNO                  7
PROCNO                 1

F2 - Acquisition Parameters
Date_           20150106
Time               19.31
INSTRUM            spect
PROBHD   5 mm DUL 13C-1
PULPROG          zgpg30
TD                 65536
SOLVENT            CDCl3
NS                   107
DS                     0
SWH            22727.273 Hz
FIDRES          0.346791 Hz
AQ             1.4418420 sec
RG                    57
DW                22.000 usec
DE                  6.00 usec
TE                 300.0 K
D1            2.00000000 sec
d11           0.03000000 sec
DELTA         1.89999998 sec
TD0                    1

======== CHANNEL f1 ========
NUC1                 13C
P1                  9.70 usec
PL1                -0.50 dB
SFO1         100.6288660 MHz

======== CHANNEL f2 ========
CPDPRG2          waltz16
NUC2                  1H
PCPD2              90.00 usec
PL2                -2.40 dB
PL12               15.10 dB
PL13               18.10 dB
SFO2         400.1516010 MHz

F1 - Acquisition parameters
TD                   256
SFO1             400.152 MHz
FIDRES         20.302755 Hz
SW                12.989 ppm
FnMODE                QF

F2 - Processing parameters
SI                 32768
SF           100.6182255 MHz
WDW                   EM
SSB       0
LB                  3.00 Hz
GB        0
PC                  1.00

F1 - Processing parameters
SI                  1024
MC2                   QF
SF           400.1500000 MHz
WDW                QSINE
SSB       0
LB        0 Hz
GB        0
```

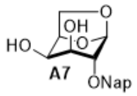

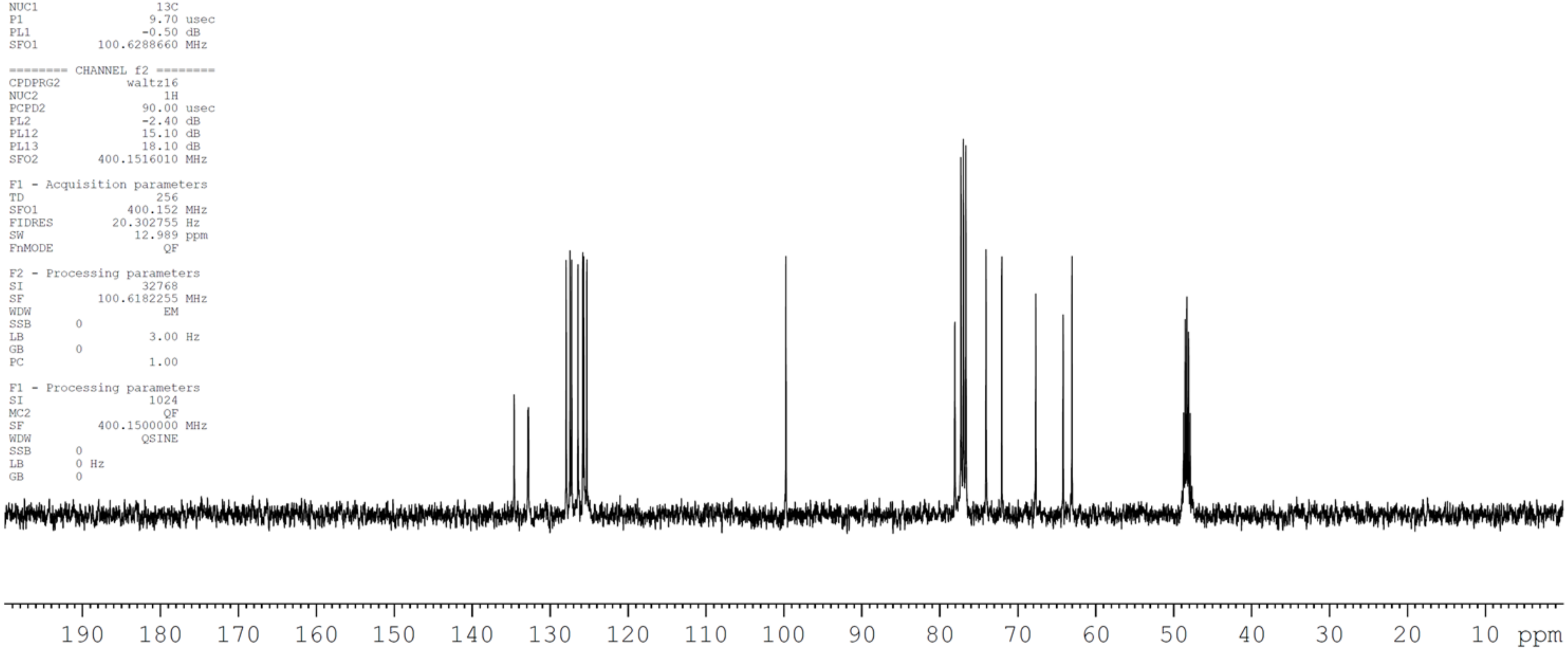

KYT-174-2-20190326

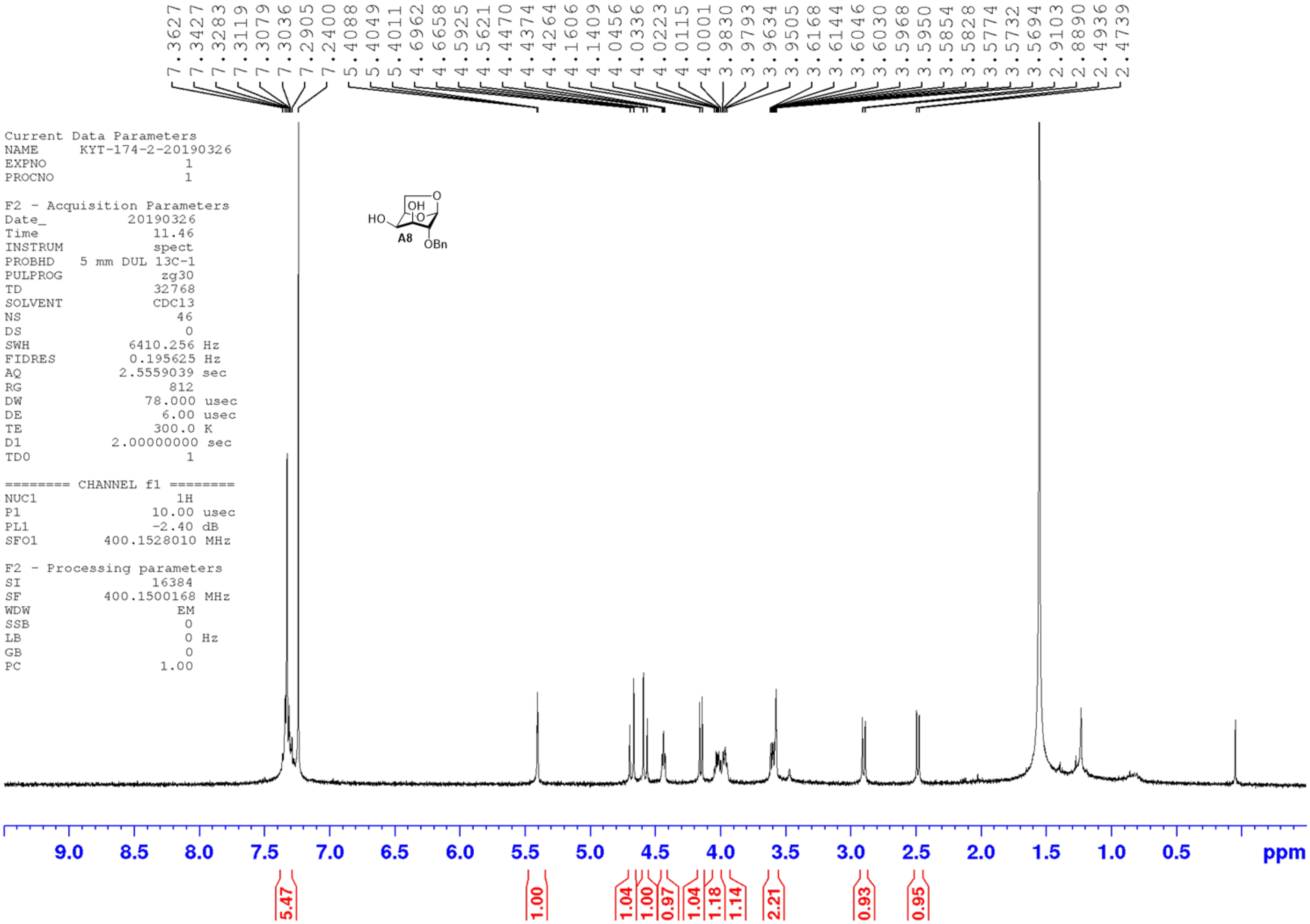

Current Data Parameters
NAME KYT-174-2-20190326
EXPNO 1
PROCNO 1

F2 - Acquisition Parameters
Date_ 20190326
Time 11.46
INSTRUM spect
PROBHD 5 mm DUL 13C-1
PULPROG zg30
TD 32768
SOLVENT CDCl3
NS 46
DS 0
SWH 6410.256 Hz
FIDRES 0.195625 Hz
AQ 2.5559039 sec
RG 812
DW 78.000 usec
DE 6.00 usec
TE 300.0 K
D1 2.00000000 sec
TD0 1

======== CHANNEL f1 ========
NUC1 1H
P1 10.00 usec
PL1 -2.40 dB
SFO1 400.1528010 MHz

F2 - Processing parameters
SI 16384
SF 400.1500168 MHz
WDW EM
SSB 0
LB 0 Hz
GB 0
PC 1.00

KYT-174-2-20190325

```
Current Data Parameters
NAME      KYT-174-2-20190325
EXPNO                      2
PROCNO                     1

F2 - Acquisition Parameters
Date_             20190325
Time                 19.10
INSTRUM              spect
PROBHD   5 mm DUL 13C-1
PULPROG            zgpg30
TD                  65536
SOLVENT             CDCl3
NS                    102
DS                      0
SWH             22727.273 Hz
FIDRES           0.346791 Hz
AQ              1.4417920 sec
RG                     57
DW                 22.000 usec
DE                   6.00 usec
TE                  300.0 K
D1             2.00000000 sec
d11            0.03000000 sec
DELTA          1.89999998 sec
TD0                     1

======== CHANNEL f1 ========
NUC1                  13C
P1                   9.70 usec
PL1                 -0.50 dB
SFO1          100.6288660 MHz

======== CHANNEL f2 ========
CPDPRG[2          waltz16
NUC2                   1H
PCPD2               90.00 usec
PL2                 -2.40 dB
PL12                15.10 dB
PL13                18.10 dB
SFO2          400.1516010 MHz

F2 - Processing parameters
SI                  32768
SF            100.6178072 MHz
WDW                    EM
SSB                     0
LB                   3.00 Hz
GB                      0
PC                   1.00
```

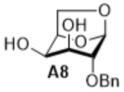

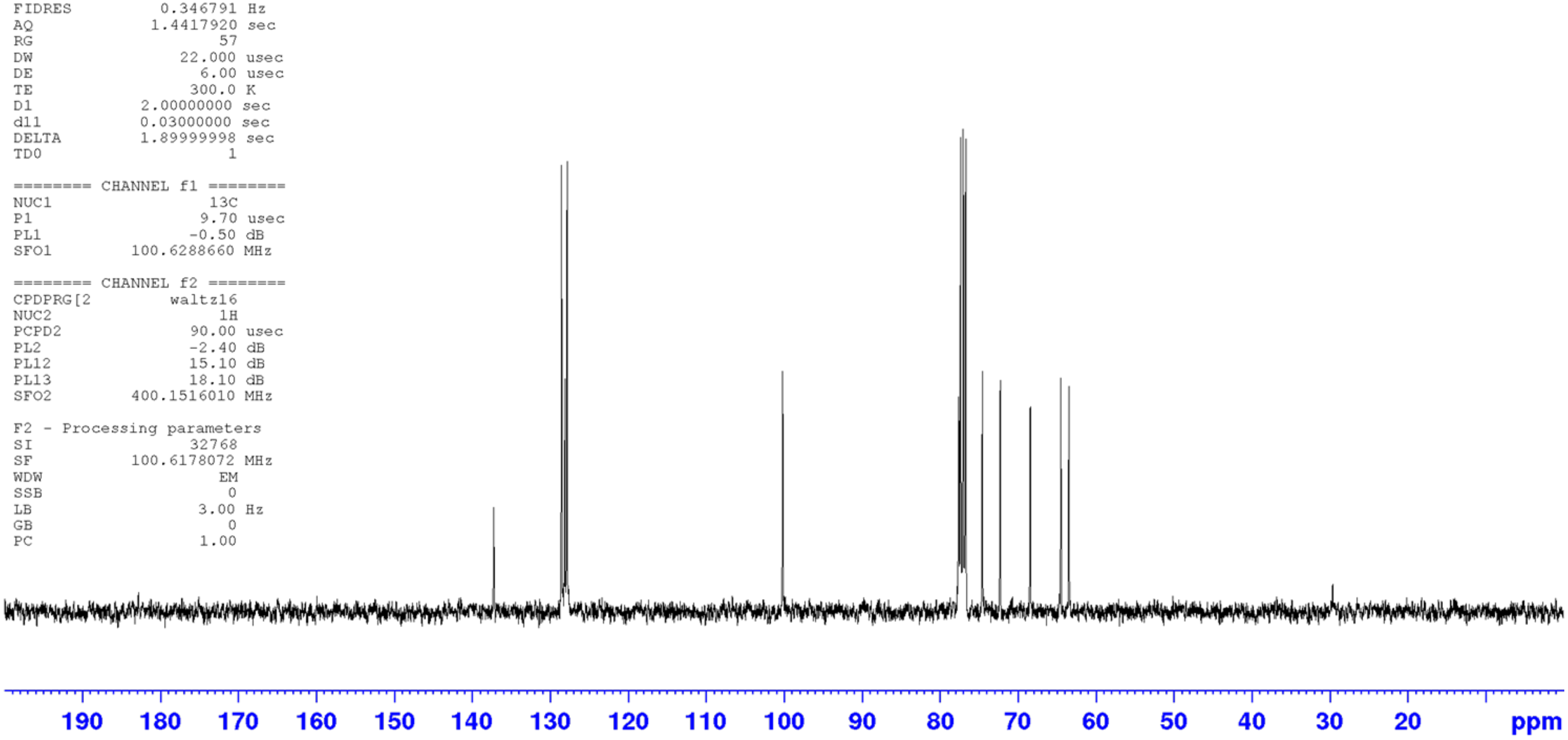

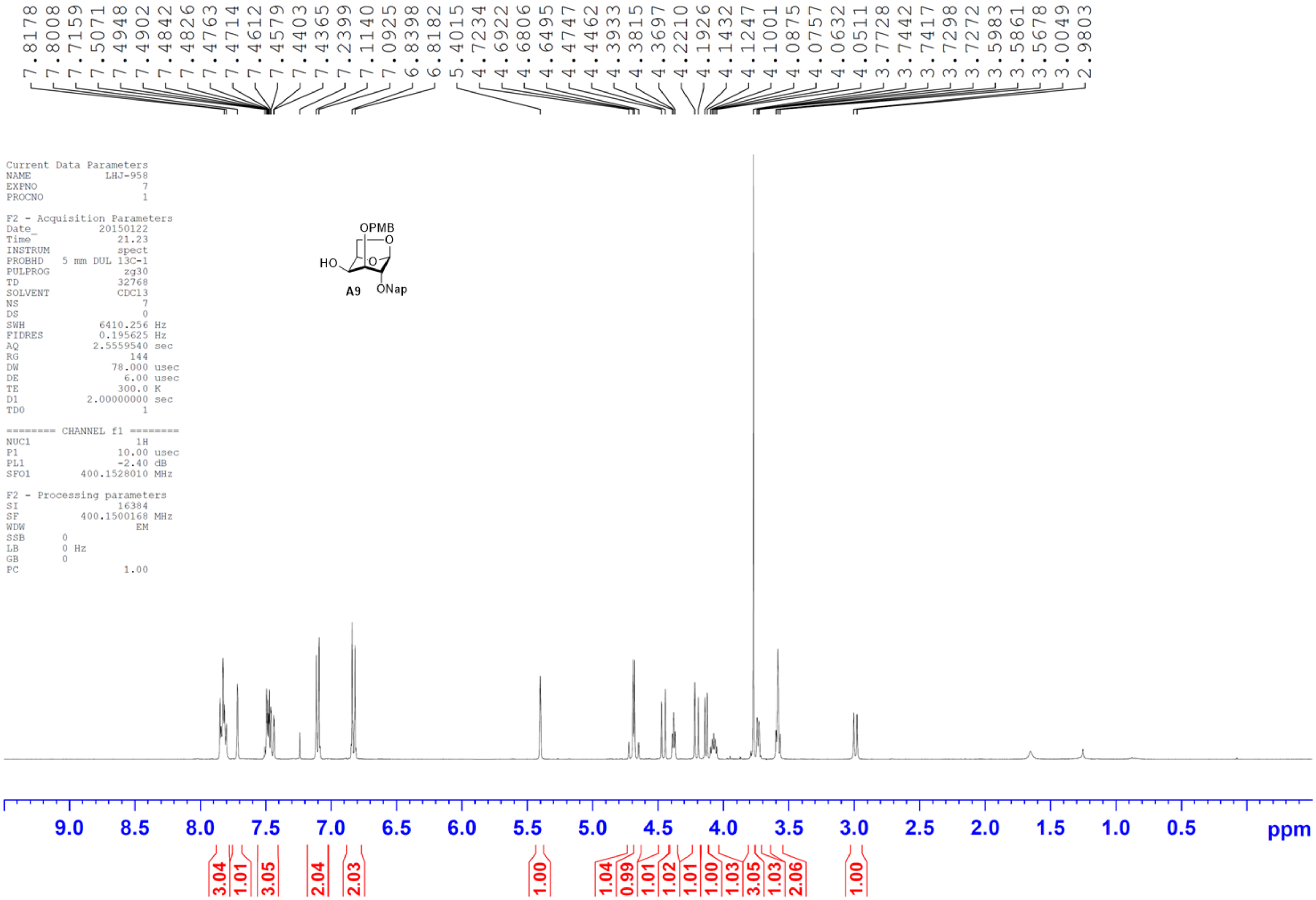
7.8178 7.8008 7.7159 7.5071 7.4948 7.4902 7.4842 7.4826 7.4763 7.4714 7.4612 7.4579 7.4403 7.4365 7.2399 7.1140 7.0925 6.8398 6.8182 5.4015 4.7234 4.6922 4.6806 4.6495 4.4747 4.4462 4.3933 4.3815 4.3697 4.2210 4.1926 4.1432 4.1247 4.1001 4.0875 4.0757 4.0632 4.0511 3.7728 3.7442 3.7417 3.7298 3.7272 3.5983 3.5861 3.5678 3.0049 2.9803
Current Data Parameters
NAME LHJ-958
EXPNO 7
PROCNO 1
F2 - Acquisition Parameters
Date_ 20150122
Time 21.23
INSTRUM spect
PROBHD 5 mm DUL 13C-1
PULPROG zg30
TD 32768
SOLVENT CDCl3
NS 7
DS 0
SWH 6410.256 Hz
FIDRES 0.195625 Hz
AQ 2.5559540 sec
RG 144
DW 78.000 usec
DE 6.00 usec
TE 300.0 K
D1 2.00000000 sec
TD0 1
======== CHANNEL f1 ========
NUC1 1H
P1 10.00 usec
PL1 -2.40 dB
SFO1 400.1528010 MHz
F2 - Processing parameters
SI 16384
SF 400.1500168 MHz
WDW EM
SSB 0
LB 0 Hz
GB 0
PC 1.00
OPMB
HO
O
O
A9
ONap
9.0 8.5 8.0 7.5 7.0 6.5 6.0 5.5 5.0 4.5 4.0 3.5 3.0 2.5 2.0 1.5 1.0 0.5 ppm
3.04 1.01 3.05 2.04 2.03 1.00 1.04 0.99 1.01 1.02 1.01 1.00 1.03 3.05 1.03 2.06 1.00

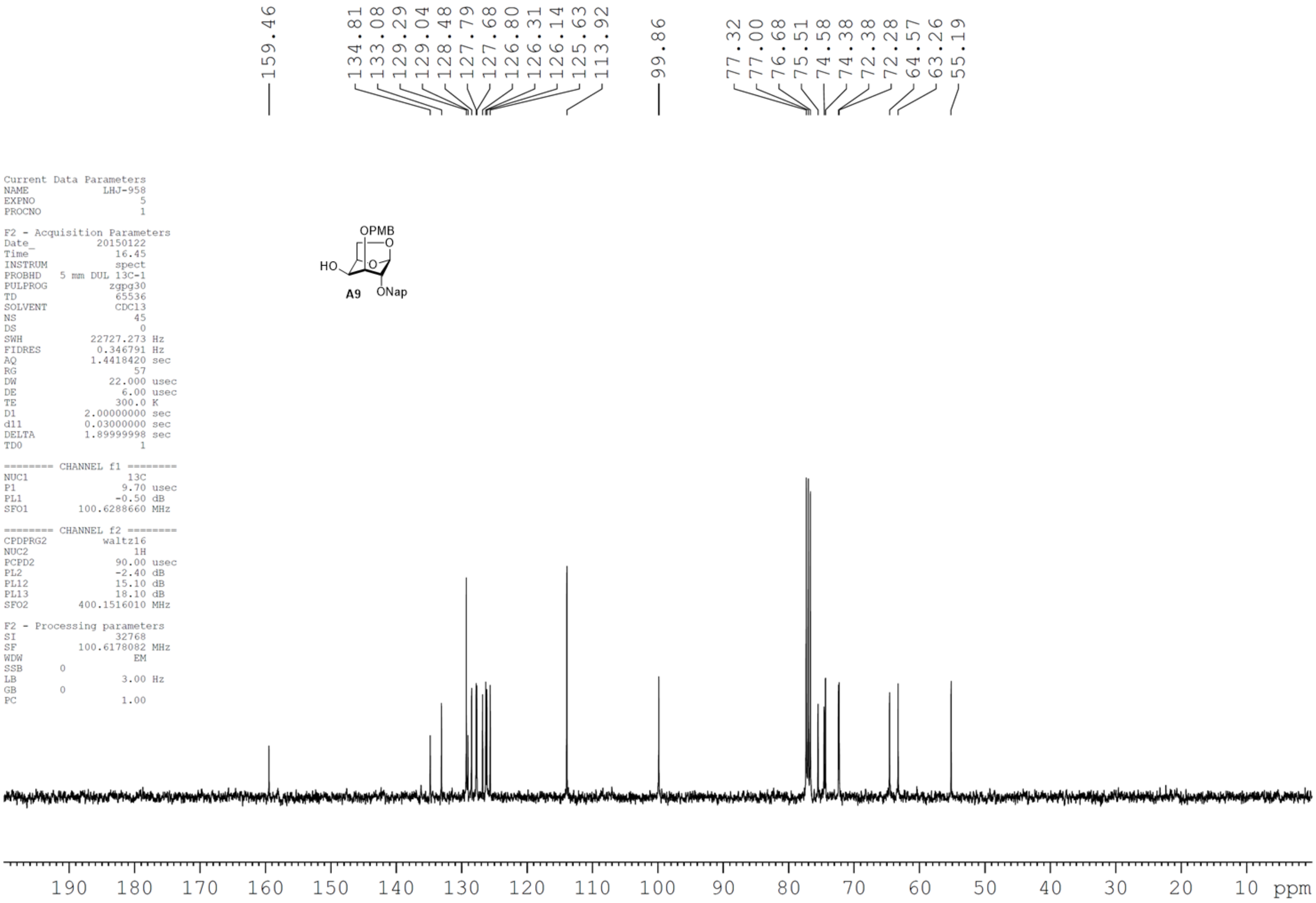

A9

```
Current Data Parameters
NAME              LHJ-958
EXPNO                   5
PROCNO                  1

F2 - Acquisition Parameters
Date_            20150122
Time                16.45
INSTRUM             spect
PROBHD   5 mm DUL 13C-1
PULPROG           zgpg30
TD                  65536
SOLVENT             CDCl3
NS                     45
DS                      0
SWH             22727.273 Hz
FIDRES           0.346791 Hz
AQ              1.4418420 sec
RG                     57
DW                 22.000 usec
DE                   6.00 usec
TE                  300.0 K
D1             2.00000000 sec
d11            0.03000000 sec
DELTA          1.89999998 sec
TD0                     1

======== CHANNEL f1 ========
NUC1                  13C
P1                   9.70 usec
PL1                 -0.50 dB
SFO1          100.6288660 MHz

======== CHANNEL f2 ========
CPDPRG2           waltz16
NUC2                   1H
PCPD2               90.00 usec
PL2                 -2.40 dB
PL12                15.10 dB
PL13                18.10 dB
SFO2          400.1516010 MHz

F2 - Processing parameters
SI                  32768
SF            100.6178082 MHz
WDW                    EM
SSB      0
LB                   3.00 Hz
GB       0
PC                   1.00
```

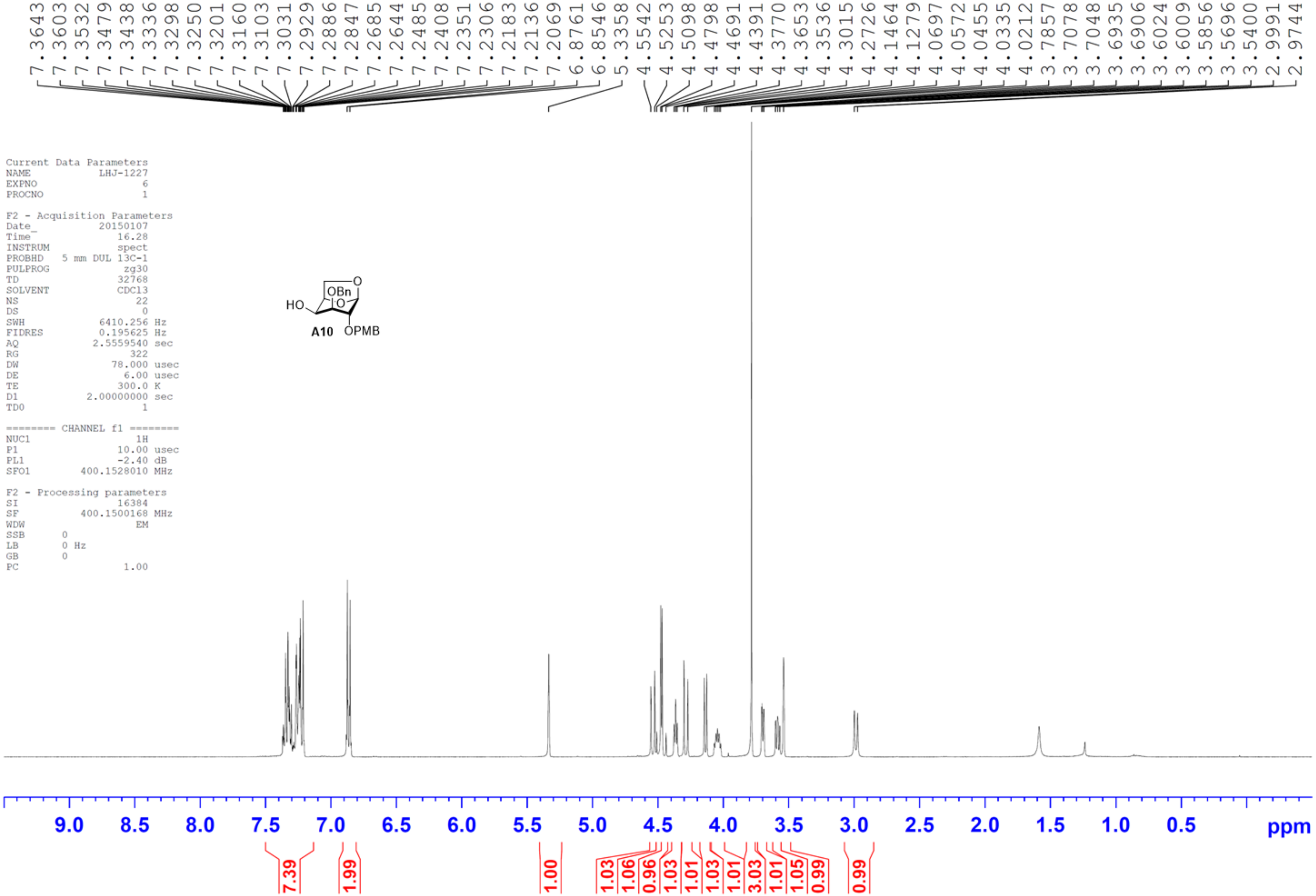
7.3643
7.3603
7.3532
7.3479
7.3438
7.3336
7.3298
7.3250
7.3201
7.3160
7.3103
7.3031
7.2929
7.2886
7.2847
7.2685
7.2644
7.2485
7.2408
7.2351
7.2306
7.2183
7.2136
7.2069
6.8761
6.8546
5.3358
4.5542
4.5253
4.5098
4.4798
4.4691
4.4391
4.3770
4.3653
4.3536
4.3015
4.2726
4.1464
4.1279
4.0697
4.0572
4.0455
4.0335
4.0212
3.7857
3.7078
3.7048
3.6935
3.6906
3.6024
3.6009
3.5856
3.5696
3.5400
2.9991
2.9744
Current Data Parameters
NAME LHJ-1227
EXPNO 6
PROCNO 1
F2 - Acquisition Parameters
Date_ 20150107
Time 16.28
INSTRUM spect
PROBHD 5 mm DUL 13C-1
PULPROG zg30
TD 32768
SOLVENT CDCl3
NS 22
DS 0
SWH 6410.256 Hz
FIDRES 0.195625 Hz
AQ 2.5559540 sec
RG 322
DW 78.000 usec
DE 6.00 usec
TE 300.0 K
D1 2.00000000 sec
TD0 1
======== CHANNEL f1 ========
NUC1 1H
P1 10.00 usec
PL1 -2.40 dB
SFO1 400.1528010 MHz
F2 - Processing parameters
SI 16384
SF 400.1500168 MHz
WDW EM
SSB 0
LB 0 Hz
GB 0
PC 1.00
HO
OBn
O
O
A10
OPMB
9.0
8.5
8.0
7.5
7.0
6.5
6.0
5.5
5.0
4.5
4.0
3.5
3.0
2.5
2.0
1.5
1.0
0.5
ppm
7.39
1.99
1.00
1.03
1.06
0.96
1.03
1.01
1.03
1.01
3.03
1.01
1.05
0.99
0.99

Current Data Parameters
NAME LHJ-1227
EXPNO 3
PROCNO 1

F2 - Acquisition Parameters
Date_ 20141118
Time 10.19
INSTRUM spect
PROBHD 5 mm DUL 13C-1
PULPROG zgpg30
TD 65536
SOLVENT CDCl3
NS 120
DS 0
SWH 22727.273 Hz
FIDRES 0.346791 Hz
AQ 1.4418420 sec
RG 57
DW 22.000 usec
DE 6.00 usec
TE 300.0 K
D1 2.00000000 sec
d11 0.03000000 sec
DELTA 1.89999998 sec
TD0 1

======== CHANNEL f1 ========
NUC1 13C
P1 9.70 usec
PL1 -0.50 dB
SFO1 100.6288660 MHz

======== CHANNEL f2 ========
CPDPRG2 waltz16
NUC2 1H
PCPD2 90.00 usec
PL2 -2.40 dB
PL12 15.10 dB
PL13 18.10 dB
SFO2 400.1516010 MHz

F2 - Processing parameters
SI 32768
SF 100.6178043 MHz
WDW EM
SSB 0
LB 3.00 Hz
GB 0
PC 1.00

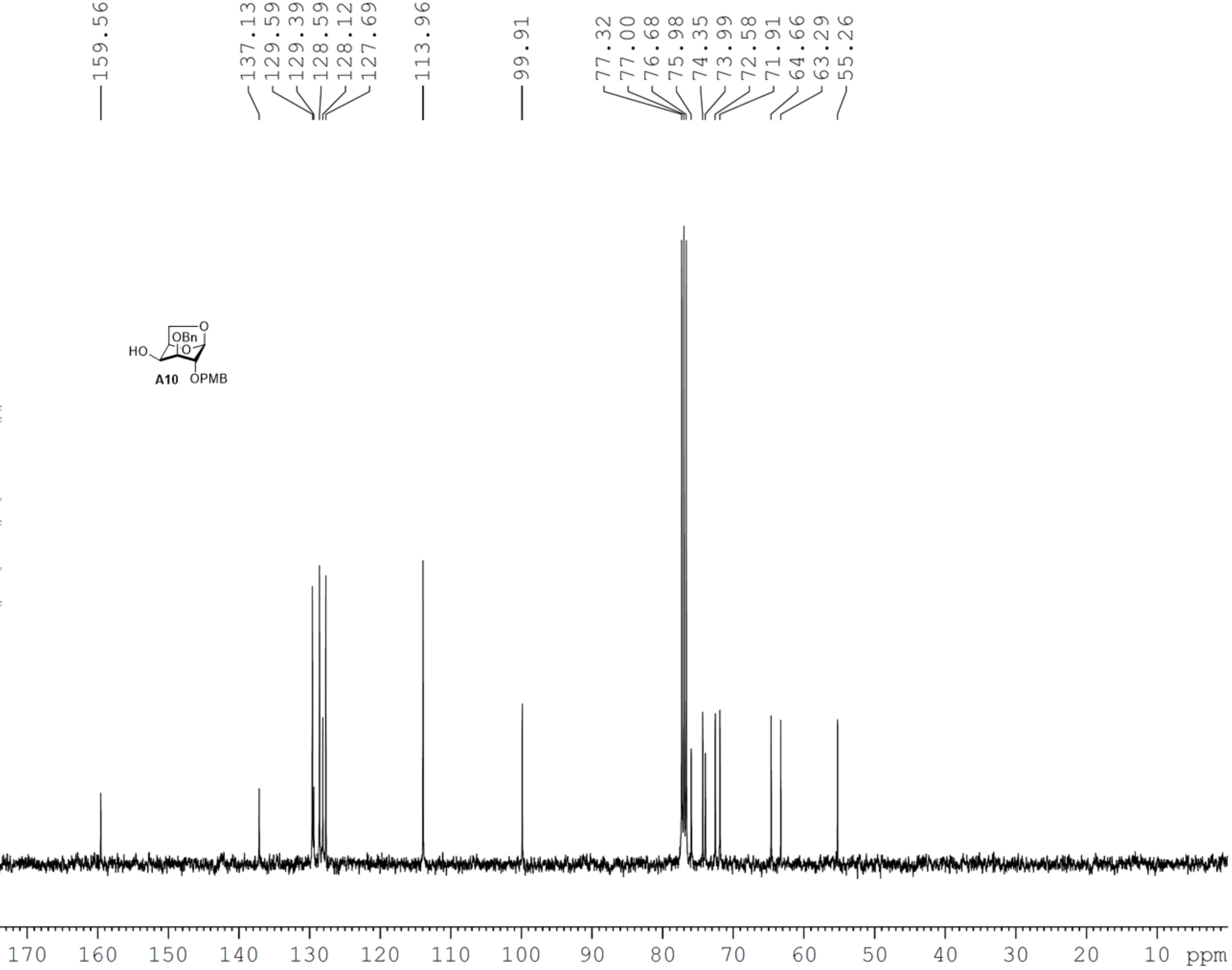

Current Data Parameters
NAME LHJ-1209
EXPNO 5
PROCNO 1

F2 - Acquisition Parameters
Date_ 20150318
Time 13.31
INSTRUM spect
PROBHD 5 mm DUL 13C-1
PULPROG zg30
TD 32768
SOLVENT CDCl3
NS 10
DS 0
SWH 6410.256 Hz
FIDRES 0.195625 Hz
AQ 2.5559540 sec
RG 322
DW 78.000 usec
DE 6.00 usec
TE 300.0 K
D1 2.00000000 sec
TD0 1

======== CHANNEL f1 ========
NUC1 1H
P1 10.00 usec
PL1 -2.40 dB
SFO1 400.1528010 MHz

F2 - Processing parameters
SI 16384
SF 400.1500168 MHz
WDW EM
SSB 0
LB 0 Hz
GB 0
PC 1.00

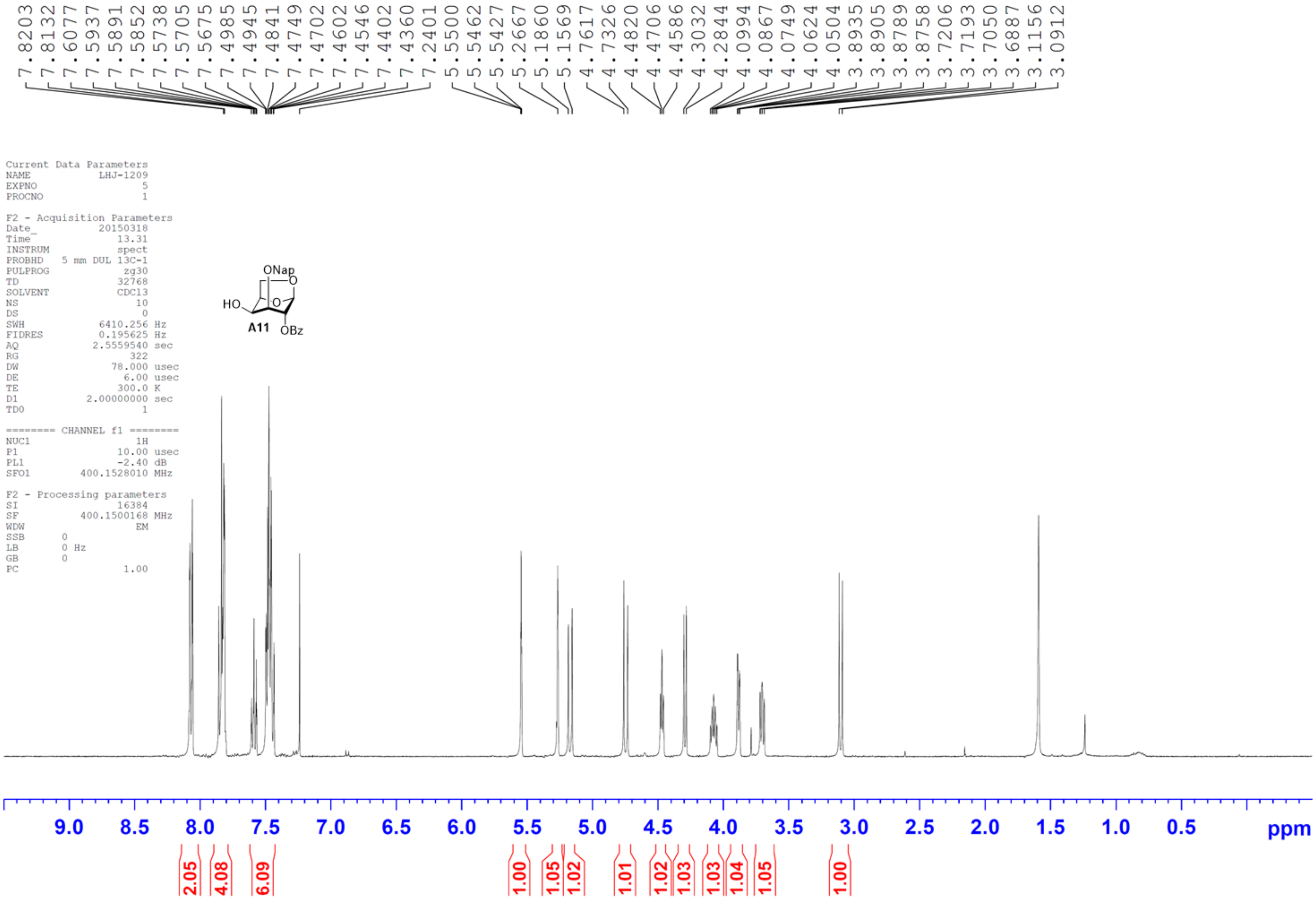

Current Data Parameters
NAME LHJ-1209
EXPNO 3
PROCNO 1

F2 - Acquisition Parameters
Date_ 20141126
Time 19.50
INSTRUM spect
PROBHD 5 mm DUL 13C-1
PULPROG zgpg30
TD 65536
SOLVENT CDCl3
NS 74
DS 0
SWH 22727.273 Hz
FIDRES 0.346791 Hz
AQ 1.4418420 sec
RG 57
DW 22.000 usec
DE 6.00 usec
TE 300.0 K
D1 2.00000000 sec
d11 0.03000000 sec
DELTA 1.89999998 sec
TD0 1

======== CHANNEL f1 ========
NUC1 13C
P1 9.70 usec
PL1 -0.50 dB
SFO1 100.6288660 MHz

======== CHANNEL f2 ========
CPDPRG2 waltz16
NUC2 1H
PCPD2 90.00 usec
PL2 -2.40 dB
PL12 15.10 dB
PL13 18.10 dB
SFO2 400.1516010 MHz

F2 - Processing parameters
SI 32768
SF 100.6178096 MHz
WDW EM
SSB 0
LB 3.00 Hz
GB 0
PC 1.00

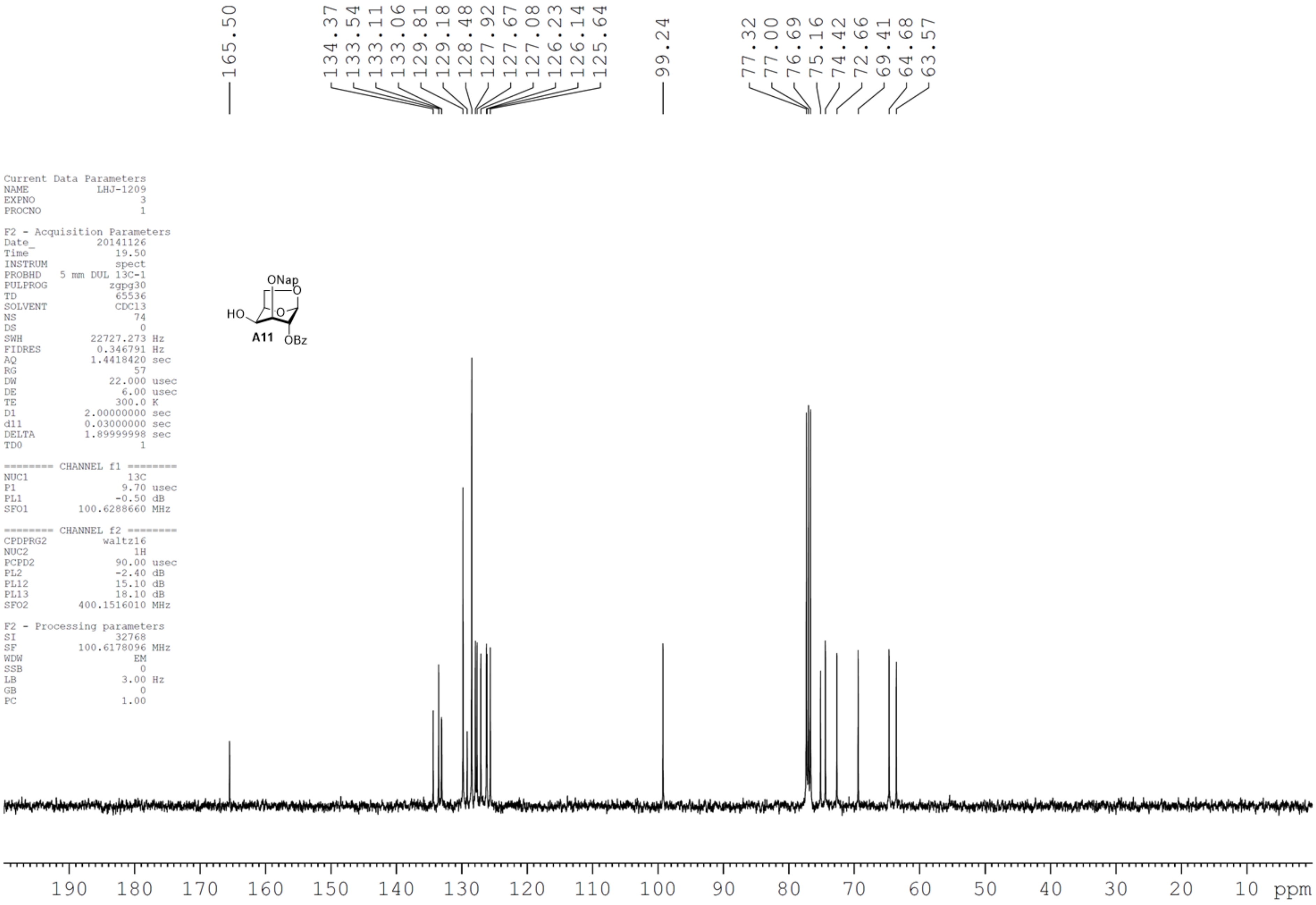

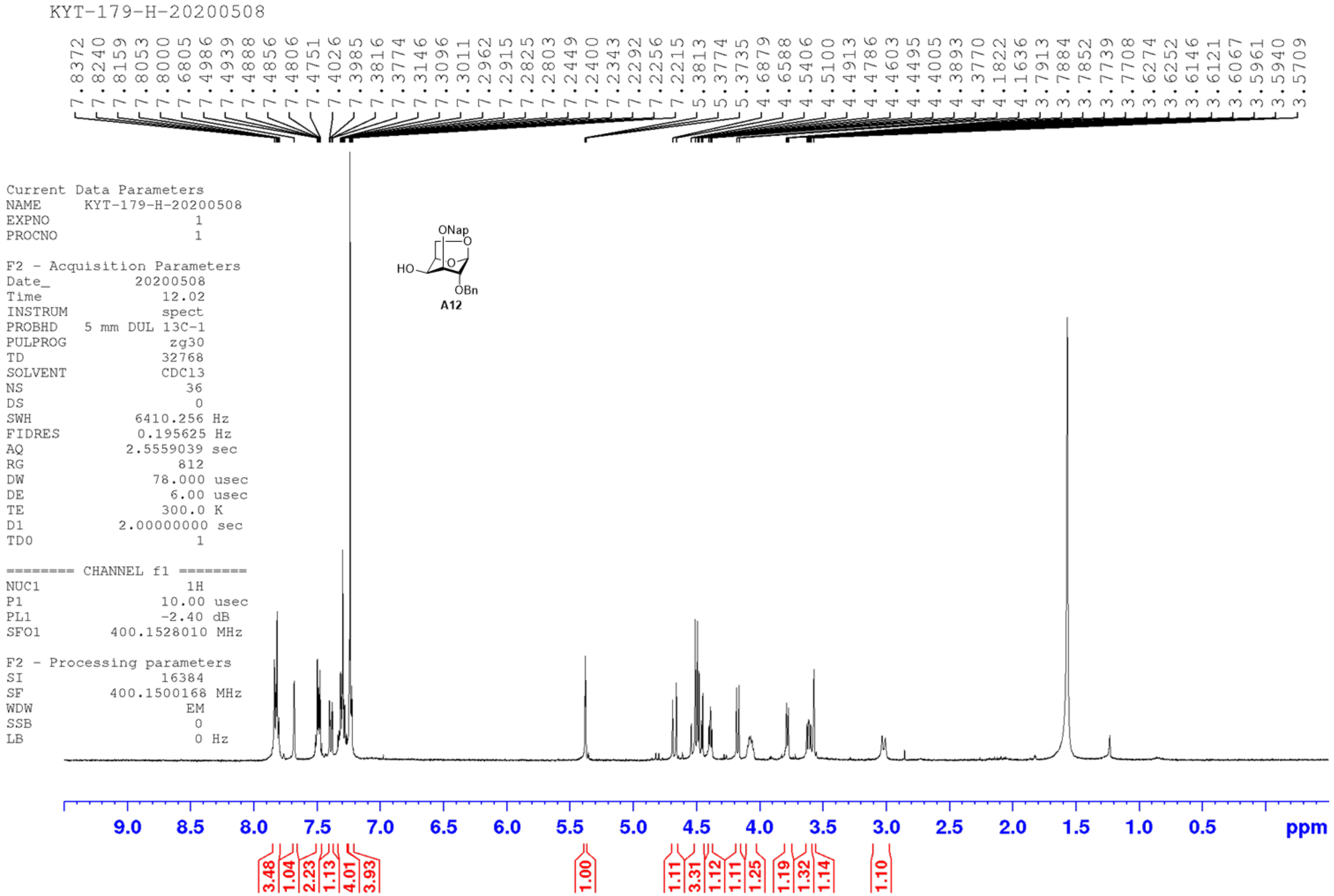
KYT-179-H-20200508
7.8372
7.8240
7.8159
7.8053
7.8000
7.6805
7.4986
7.4939
7.4888
7.4856
7.4806
7.4751
7.4026
7.3985
7.3816
7.3774
7.3146
7.3096
7.3011
7.2962
7.2915
7.2825
7.2803
7.2449
7.2400
7.2343
7.2292
7.2256
7.2215
5.3813
5.3774
5.3735
4.6879
4.6588
4.5406
4.5100
4.4913
4.4786
4.4603
4.4495
4.4005
4.3893
4.3770
4.1822
4.1636
3.7913
3.7884
3.7852
3.7739
3.7708
3.6274
3.6252
3.6146
3.6121
3.6067
3.5961
3.5940
3.5709
Current Data Parameters
NAME KYT-179-H-20200508
EXPNO 1
PROCNO 1
F2 - Acquisition Parameters
Date_ 20200508
Time 12.02
INSTRUM spect
PROBHD 5 mm DUL 13C-1
PULPROG zg30
TD 32768
SOLVENT CDCl3
NS 36
DS 0
SWH 6410.256 Hz
FIDRES 0.195625 Hz
AQ 2.5559039 sec
RG 812
DW 78.000 usec
DE 6.00 usec
TE 300.0 K
D1 2.00000000 sec
TD0 1
======== CHANNEL f1 ========
NUC1 1H
P1 10.00 usec
PL1 -2.40 dB
SFO1 400.1528010 MHz
F2 - Processing parameters
SI 16384
SF 400.1500168 MHz
WDW EM
SSB 0
LB 0 Hz
ONap
O
HO
O
OBn
A12
9.0
8.5
8.0
7.5
7.0
6.5
6.0
5.5
5.0
4.5
4.0
3.5
3.0
2.5
2.0
1.5
1.0
0.5
ppm
3.48
1.04
2.23
1.13
4.01
3.93
1.00
1.11
3.31
1.12
1.11
1.25
1.19
1.32
1.14
1.10

KYT-179-13C-20190610

Current Data Parameters
NAME KYT-179-13C-20190610
EXPNO 1
PROCNO 1

F2 - Processing parameters
SI 65536
SF 125.6720482 MHz
WDW EM
SSB 0
LB 0.30 Hz
GB 0
PC 1.00

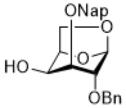

A12

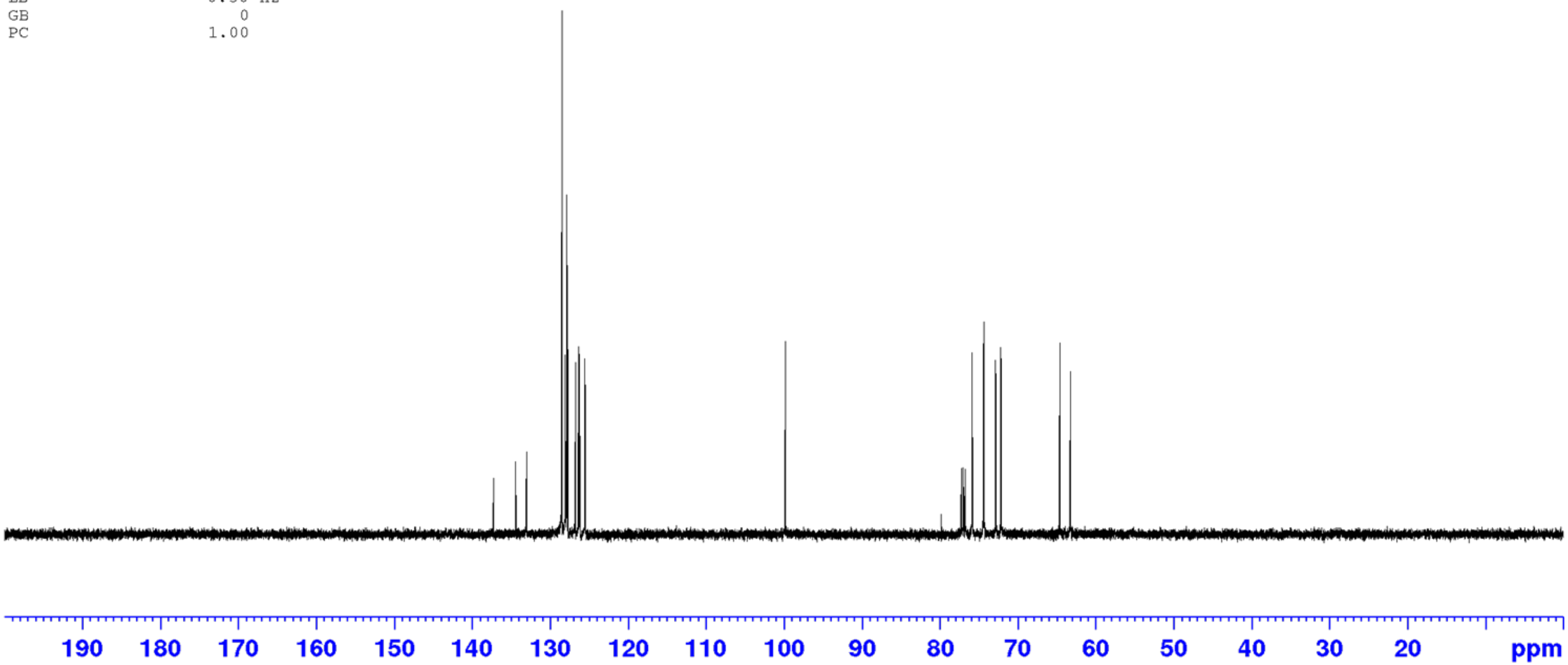

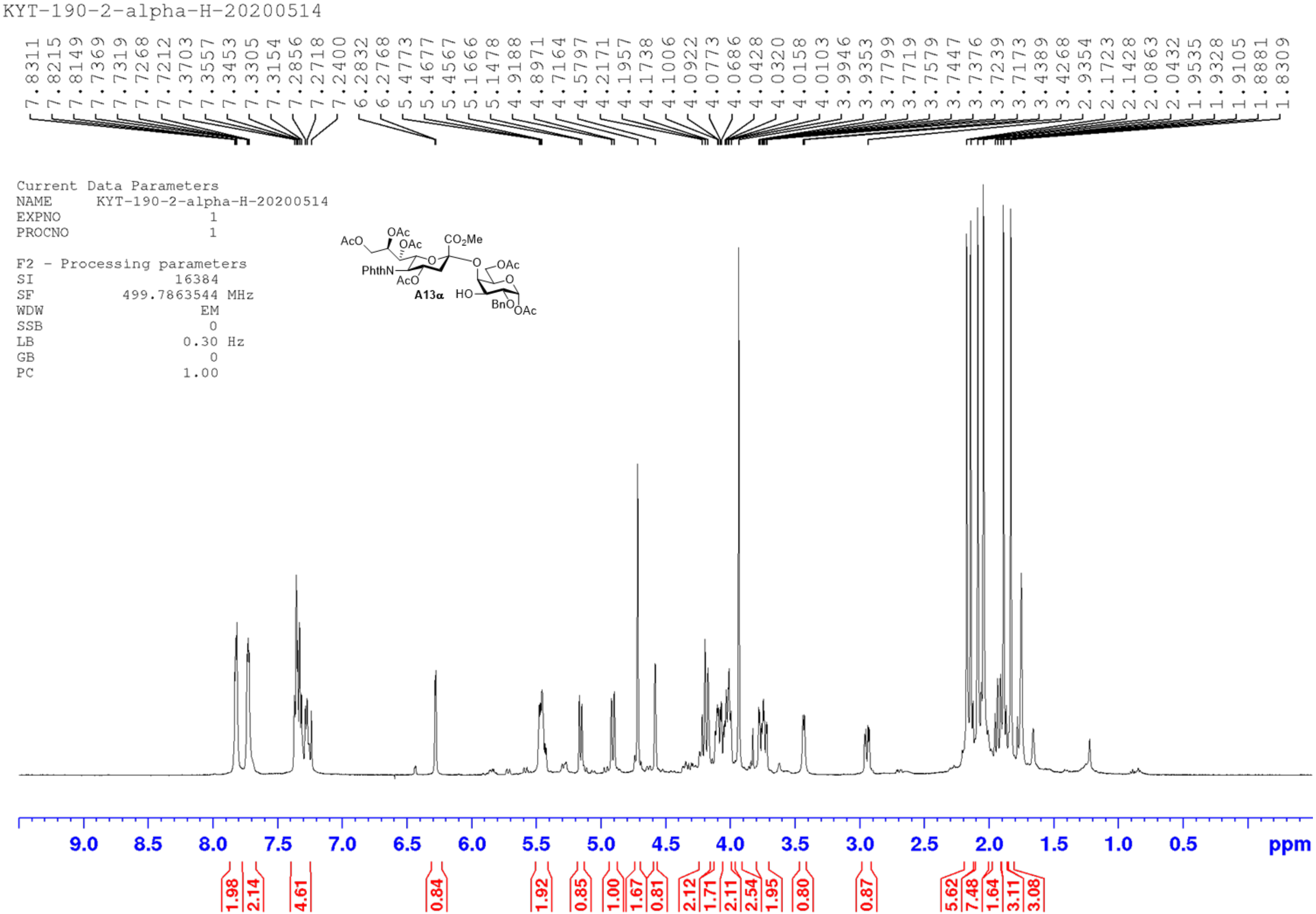

KYT-190-2-alpha-H-20200514
7.8311
7.8215
7.8149
7.7369
7.7319
7.7268
7.7212
7.3703
7.3557
7.3453
7.3305
7.3154
7.2856
7.2718
7.2400
6.2832
6.2768
5.4773
5.4677
5.4567
5.1666
5.1478
4.9188
4.8971
4.7164
4.5797
4.2171
4.1957
4.1738
4.1006
4.0922
4.0773
4.0686
4.0428
4.0320
4.0158
4.0103
3.9946
3.9353
3.7799
3.7719
3.7579
3.7447
3.7376
3.7239
3.7173
3.4389
3.4268
2.9354
2.1723
2.1428
2.0863
2.0432
1.9535
1.9328
1.9105
1.8881
1.8309
Current Data Parameters
NAME KYT-190-2-alpha-H-20200514
EXPNO 1
PROCNO 1
F2 - Processing parameters
SI 16384
SF 499.7863544 MHz
WDW EM
SSB 0
LB 0.30 Hz
GB 0
PC 1.00
AcO
OAc
OAc
CO2Me
O
O
OAc
PhthN
AcO
O
A13α
HO
BnO
OAc
9.0
8.5
8.0
7.5
7.0
6.5
6.0
5.5
5.0
4.5
4.0
3.5
3.0
2.5
2.0
1.5
1.0
0.5
ppm
1.98
2.14
4.61
0.84
1.92
0.85
1.00
1.67
0.81
2.12
1.71
2.11
2.54
1.95
0.80
0.87
5.62
7.48
1.64
3.11
3.08

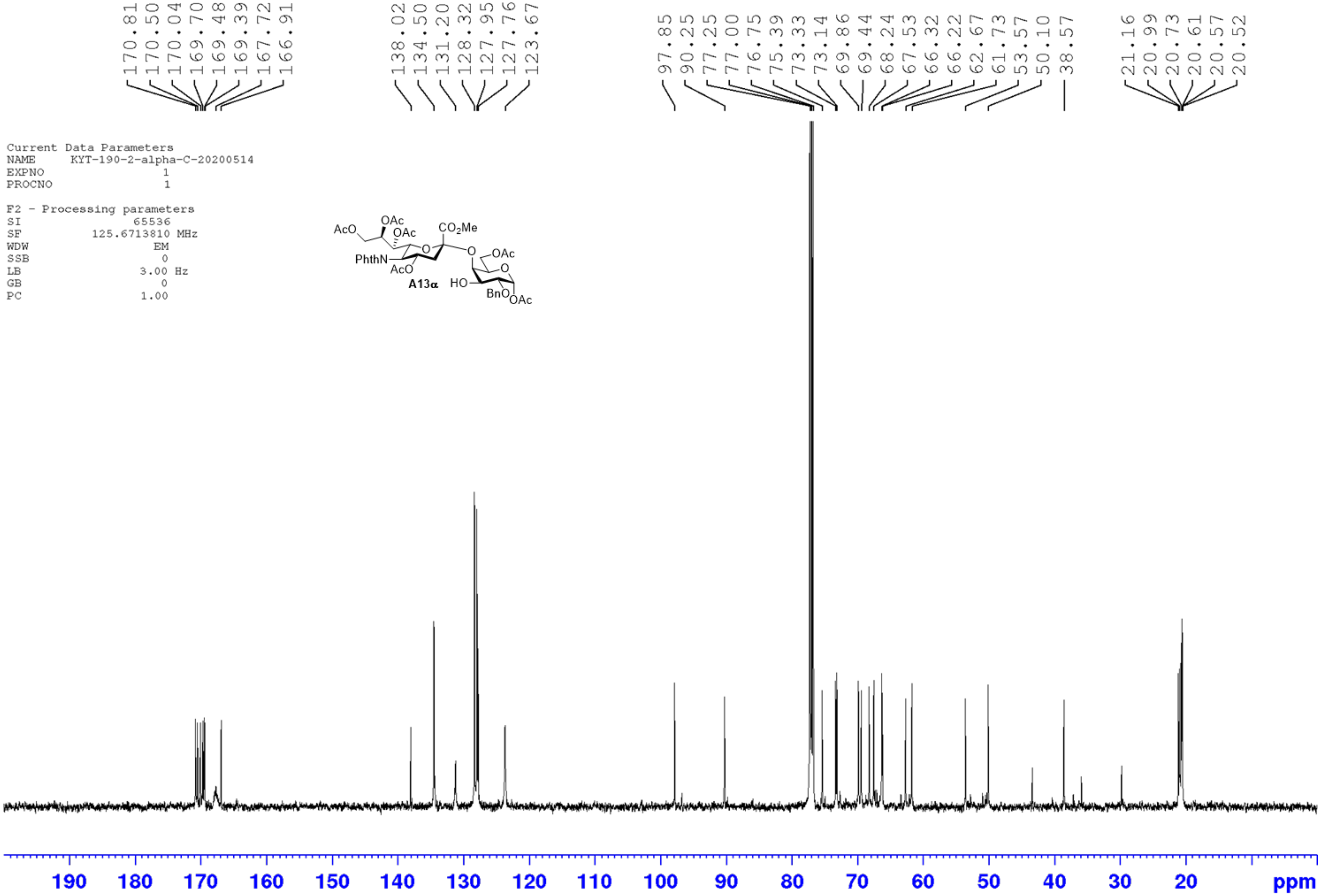
Current Data Parameters
NAME KYT-190-2-alpha-C-20200514
EXPNO 1
PROCNO 1
F2 - Processing parameters
SI 65536
SF 125.6713810 MHz
WDW EM
SSB 0
LB 3.00 Hz
GB 0
PC 1.00
170.81
170.50
170.04
169.70
169.48
169.39
167.72
166.91
138.02
134.50
131.20
128.32
127.95
127.76
123.67
97.85
90.25
77.25
77.00
76.75
75.39
73.33
73.14
69.86
69.44
68.24
67.53
66.32
66.22
62.67
61.73
53.57
50.10
38.57
21.16
20.99
20.73
20.61
20.57
20.52
AcO
OAc
OAc
CO2Me
O
O
OAc
PhthN
AcO
O
A13α
HO
BnO
OAc
190
180
170
160
150
140
130
120
110
100
90
80
70
60
50
40
30
20
ppm

KYT-191-2-beta-20200508

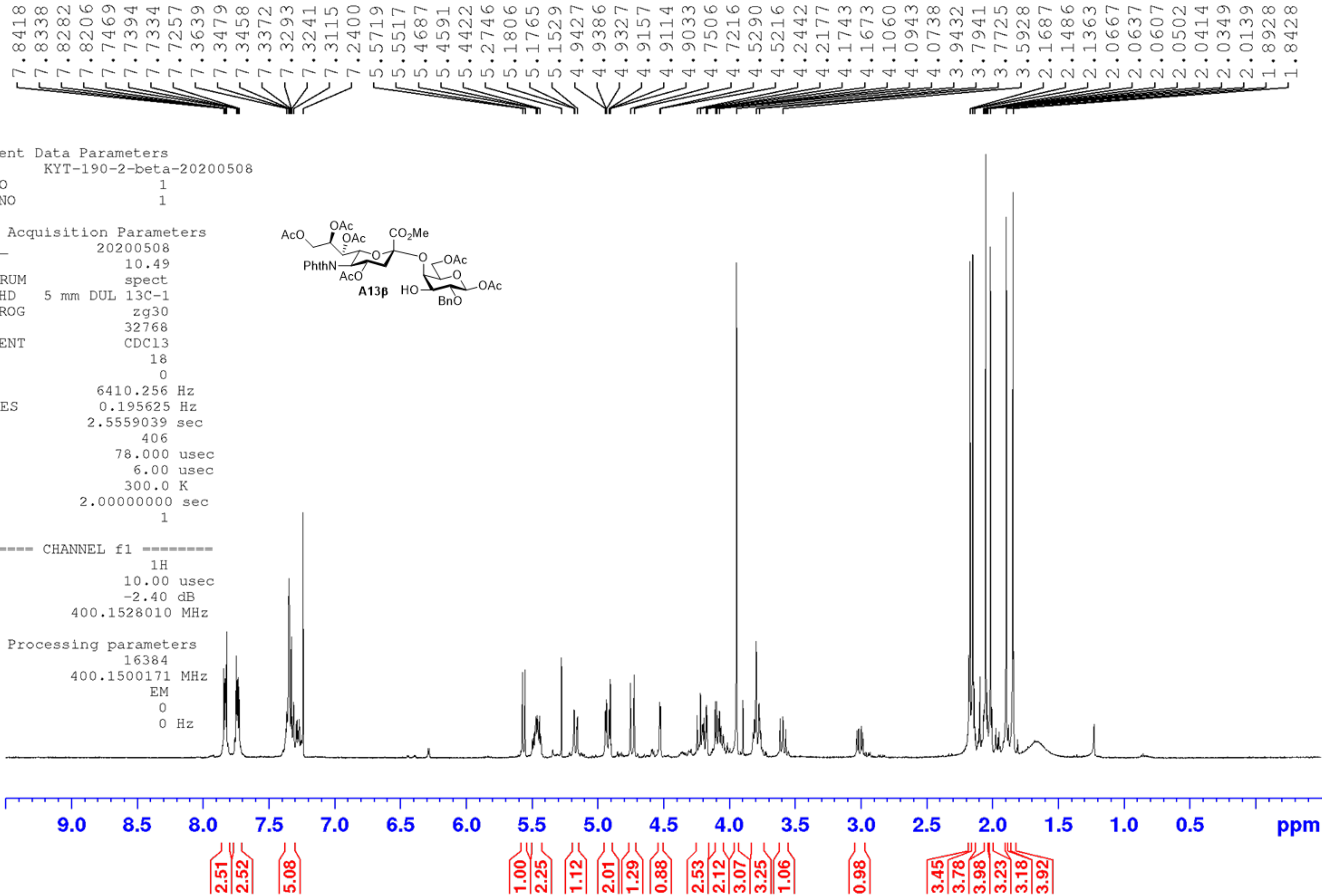

Current Data Parameters
NAME KYT-190-2-beta-20200508
EXPNO 1
PROCNO 1

F2 - Acquisition Parameters
Date_ 20200508
Time 10.49
INSTRUM spect
PROBHD 5 mm DUL 13C-1
PULPROG zg30
TD 32768
SOLVENT CDCl3
NS 18
DS 0
SWH 6410.256 Hz
FIDRES 0.195625 Hz
AQ 2.5559039 sec
RG 406
DW 78.000 usec
DE 6.00 usec
TE 300.0 K
D1 2.00000000 sec
TD0 1

======== CHANNEL f1 ========
NUC1 1H
P1 10.00 usec
PL1 -2.40 dB
SFO1 400.1528010 MHz

F2 - Processing parameters
SI 16384
SF 400.1500171 MHz
WDW EM
SSB 0
LB 0 Hz

A13β

KYT-190-2-beta-C

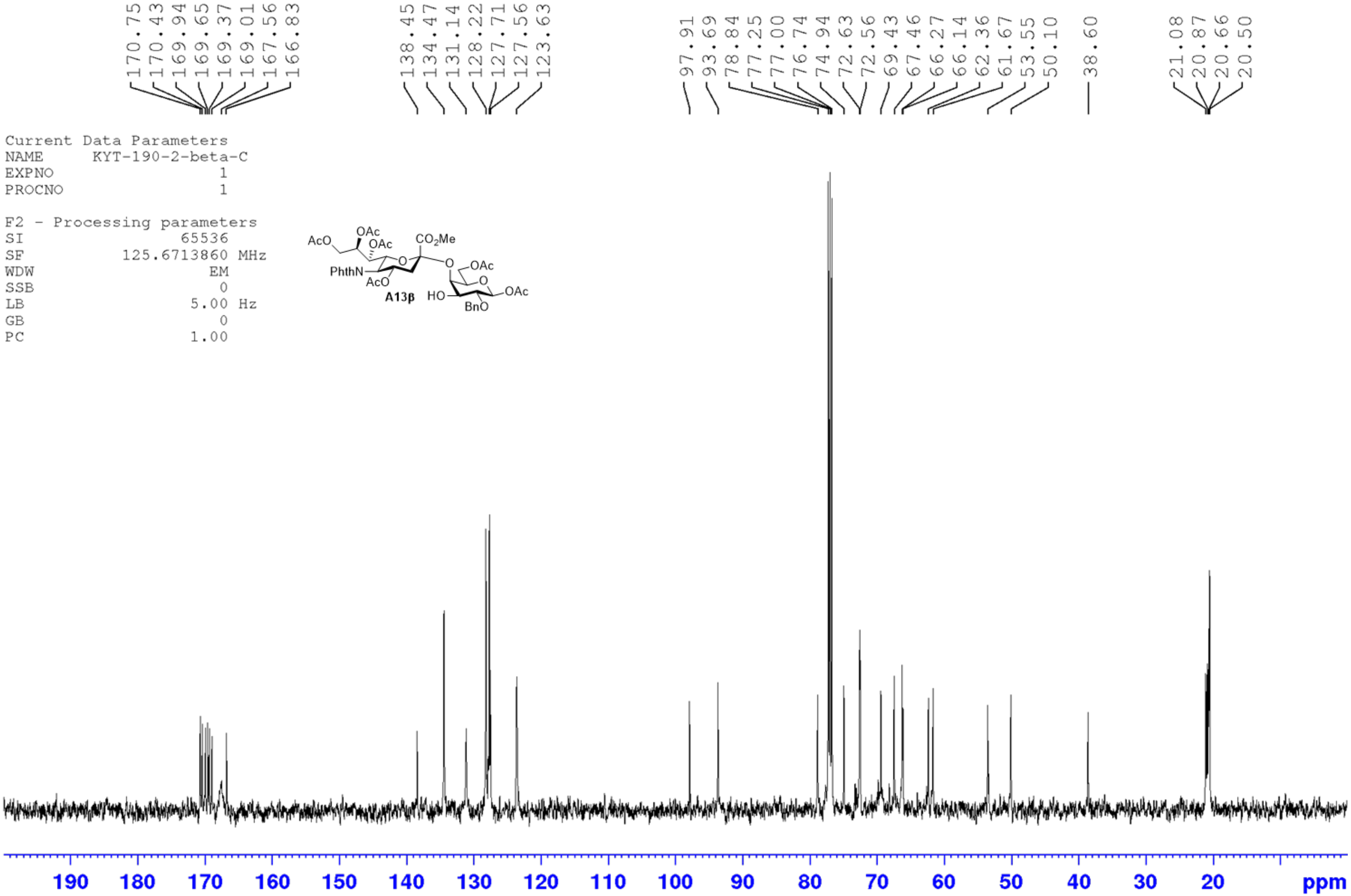

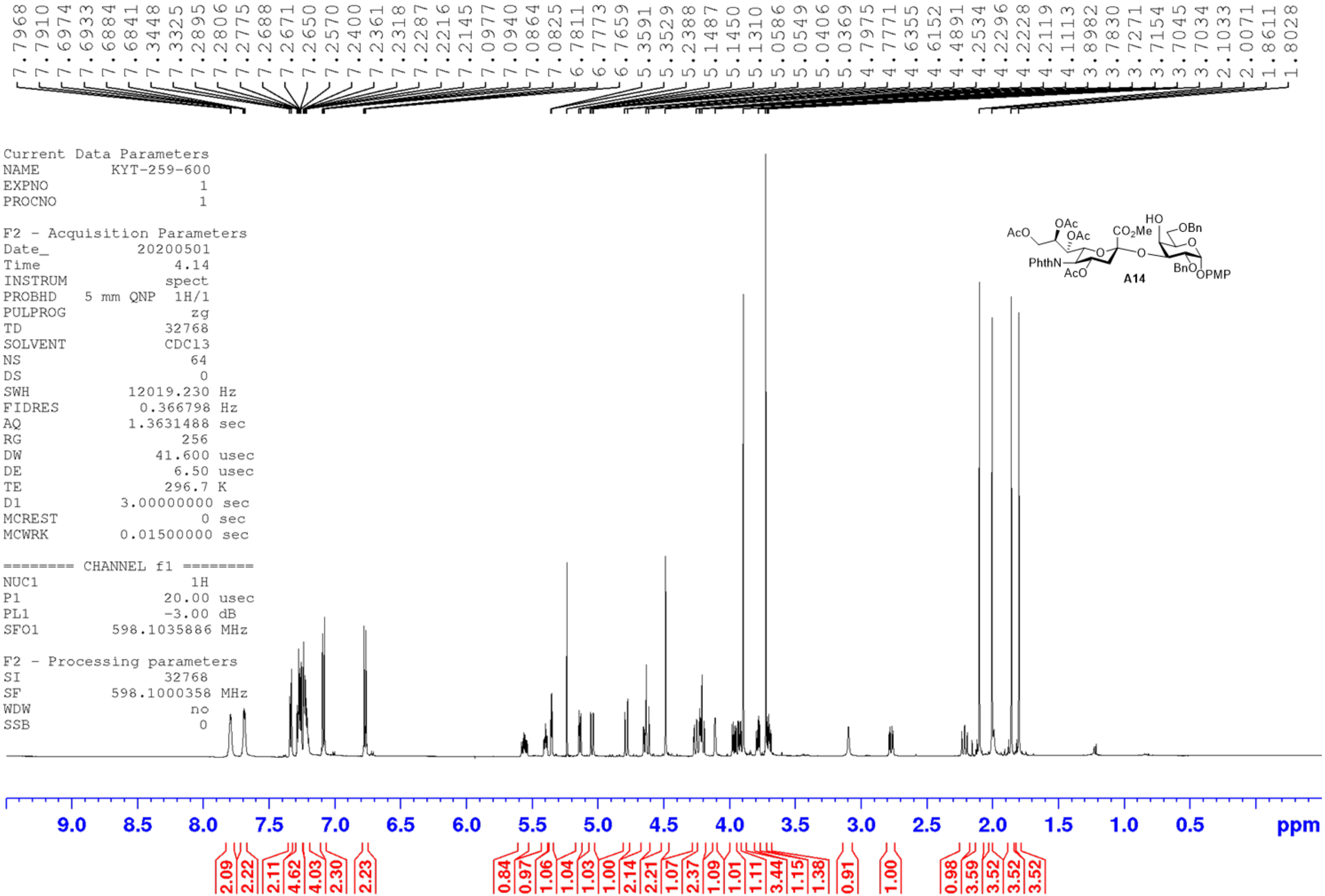
7.7968
7.7910
7.6974
7.6933
7.6884
7.6841
7.3448
7.3325
7.2895
7.2806
7.2775
7.2688
7.2671
7.2650
7.2570
7.2400
7.2361
7.2318
7.2287
7.2216
7.2145
7.0977
7.0940
7.0864
7.0825
6.7811
6.7773
6.7659
5.3591
5.3529
5.2388
5.1487
5.1450
5.1310
5.0586
5.0549
5.0406
5.0369
4.7975
4.7771
4.6355
4.6152
4.4891
4.2534
4.2296
4.2228
4.2119
4.1113
3.8982
3.7830
3.7271
3.7154
3.7045
3.7034
2.1033
2.0071
1.8611
1.8028
Current Data Parameters
NAME KYT-259-600
EXPNO 1
PROCNO 1
F2 - Acquisition Parameters
Date_ 20200501
Time 4.14
INSTRUM spect
PROBHD 5 mm QNP 1H/1
PULPROG zg
TD 32768
SOLVENT CDCl3
NS 64
DS 0
SWH 12019.230 Hz
FIDRES 0.366798 Hz
AQ 1.3631488 sec
RG 256
DW 41.600 usec
DE 6.50 usec
TE 296.7 K
D1 3.00000000 sec
MCREST 0 sec
MCWRK 0.01500000 sec
======== CHANNEL f1 ========
NUC1 1H
P1 20.00 usec
PL1 -3.00 dB
SFO1 598.1035886 MHz
F2 - Processing parameters
SI 32768
SF 598.1000358 MHz
WDW no
SSB 0
9.0 8.5 8.0 7.5 7.0 6.5 6.0 5.5 5.0 4.5 4.0 3.5 3.0 2.5 2.0 1.5 1.0 0.5 ppm
2.09 2.22 2.11 4.62 4.03 2.30 2.23 0.84 0.97 1.06 1.04 1.03 1.00 2.14 2.21 1.07 2.37 1.09 1.01 1.11 3.44 1.15 1.38 0.91 1.00 0.98 3.59 3.52 3.52 3.52
AcO
OAc
OAc
CO2Me
HO
OBn
O
O
O
PhthN
AcO
BnO
OPMP

A14

Current Data Parameters
NAME KYT-259-600
EXPNO 2
PROCNO 1

F2 - Acquisition Parameters
Date_ 20200501
Time 4.29
INSTRUM spect
PROBHD 5 mm QNP 1H/1
PULPROG zgpg
TD 32768
SOLVENT CDCl3
NS 169
DS 0
SWH 45045.047 Hz
FIDRES 1.374666 Hz
AQ 0.3637248 sec
RG 4096
DW 11.100 usec
DE 6.50 usec
TE 296.7 K
D1 3.50000000 sec
d11 0.03000000 sec
DELTA 3.40000010 sec
MCREST 0 sec
MCWRK 0.01500000 sec

======== CHANNEL f1 ========
NUC1 13C
P1 4.80 usec
PL1 0 dB
SFO1 150.4102296 MHz

======== CHANNEL f2 ========
CPDPRG[2 waltz16
NUC2 1H
PCPD2 92.00 usec
PL2 120.00 dB
PL12 6.00 dB
PL13 15.00 dB
SFO2 598.1020934 MHz

F2 - Processing parameters
SI 65536
SF 150.3923891 MHz
WDW EM
SSB 0
LB 3.00 Hz
GB 0
PC 1.00

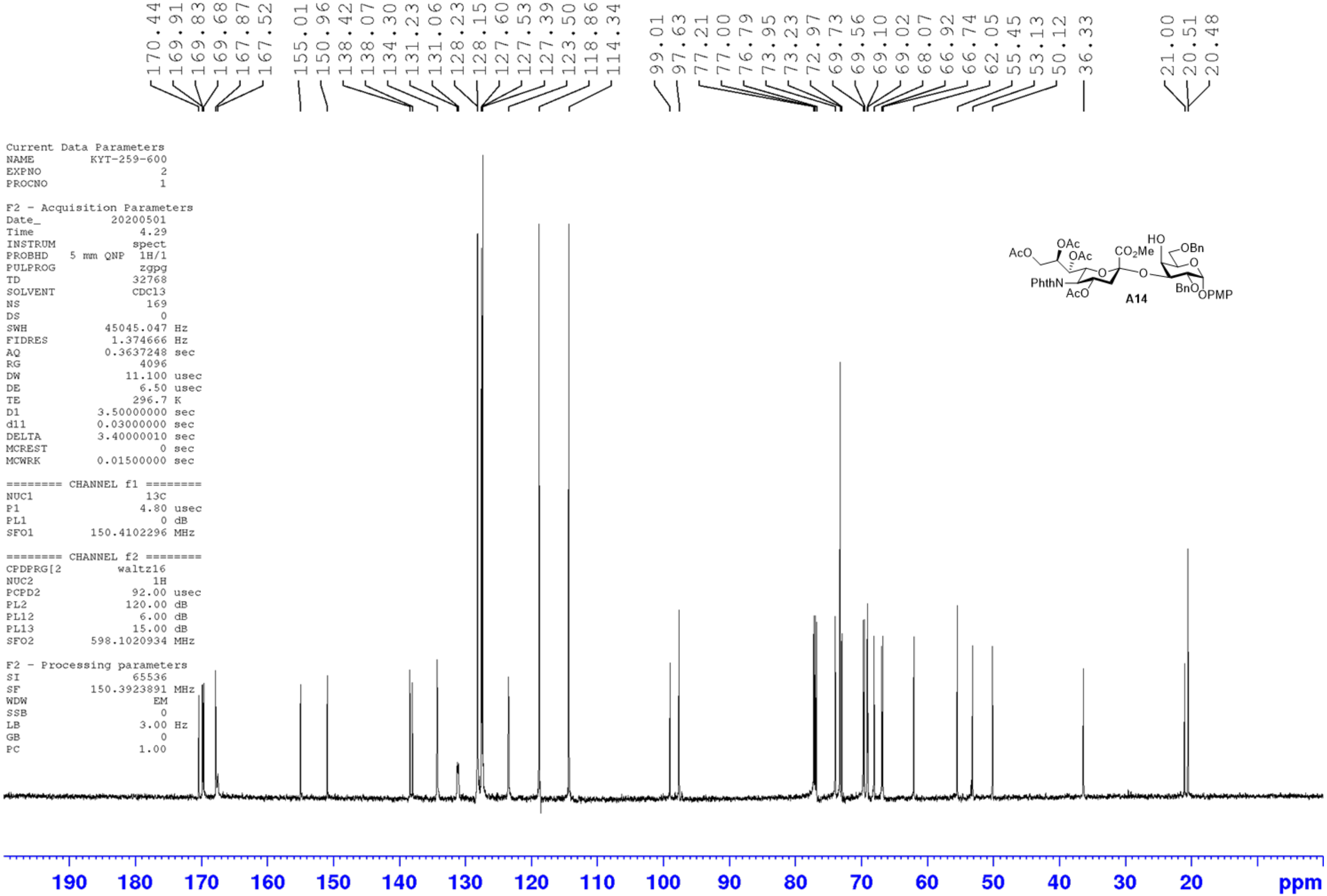

COSY

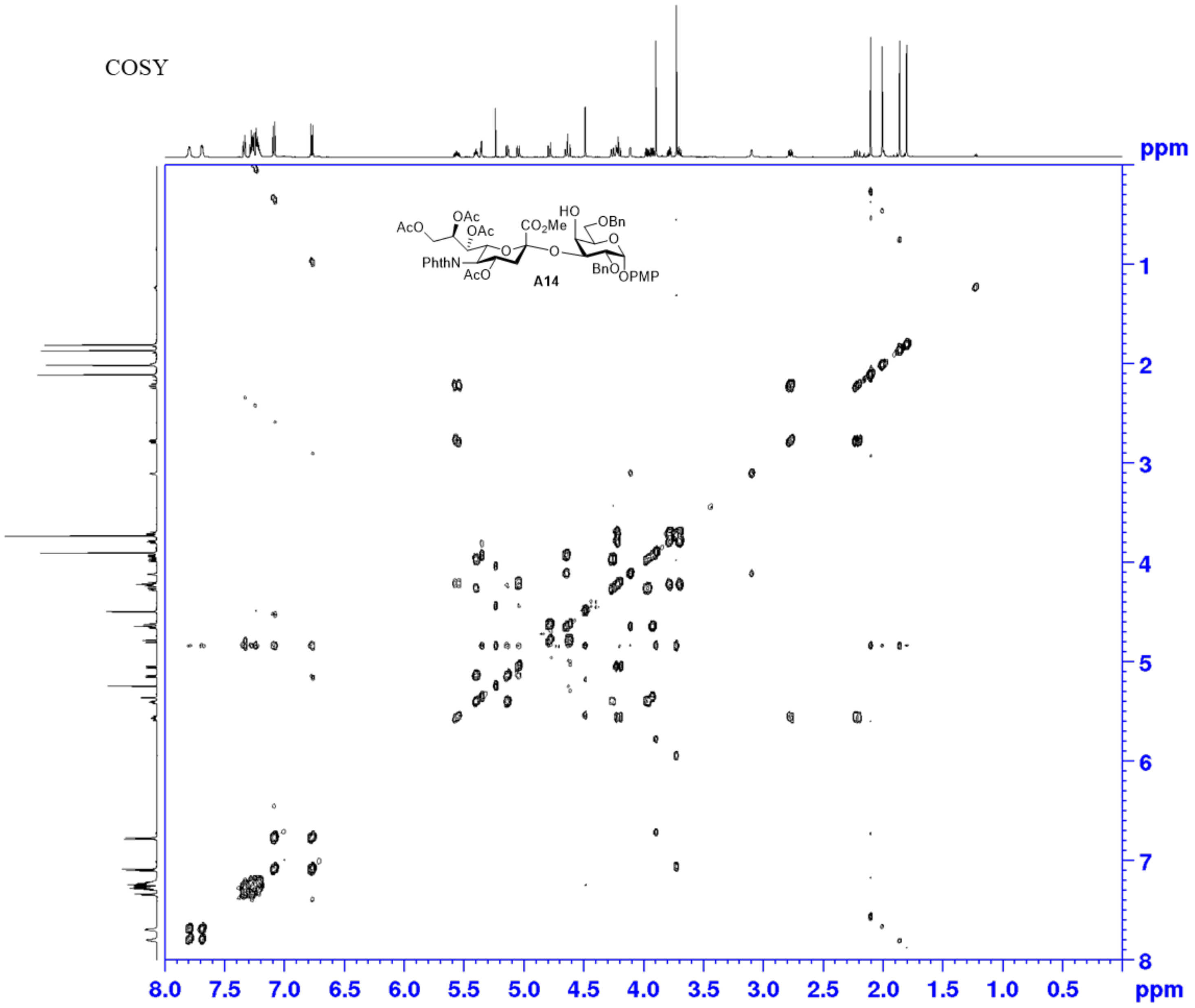

Current Data Parameters
NAME KYT-259-600
EXPNO 11
PROCNO 1

F2 - Acquisition Parameters
Date_ 20200501
Time 4.57
INSTRUM spect
PROBHD 5 mm QNP 1H/1
PULPROG cosygf
TD 2048
SOLVENT MeOD
NS 8
DS 0
SWH 6009.615 Hz
FIDRES 2.934382 Hz
AQ 0.1703936 sec
RG 16
DW 83.200 usec
DE 6.50 usec
TE 296.6 K
d0 0.00000300 sec
D1 1.50000000 sec
IN0 0.00016640 sec
MCREST 0 sec
MCWRK 1.50000000 sec

======== CHANNEL f1 ========
NUC1 1H
P0 14.00 usec
P1 30.00 usec
PL1 -3.00 dB
SFO1 598.1029307 MHz

F1 - Acquisition parameters
TD 396
SFO1 598.1029 MHz
FIDRES 30.351593 Hz
SW 10.048 ppm
FnMODE QF

F2 - Processing parameters
SI 2048
SF 598.1000358 MHz
WDW QSINE
SSB 0
LB 0 Hz
GB 0
PC 1.00

F1 - Processing parameters
SI 512
MC2 QF
SF 598.1000358 MHz
WDW QSINE
SSB 0
LB 0 Hz
GB 0

HSQC

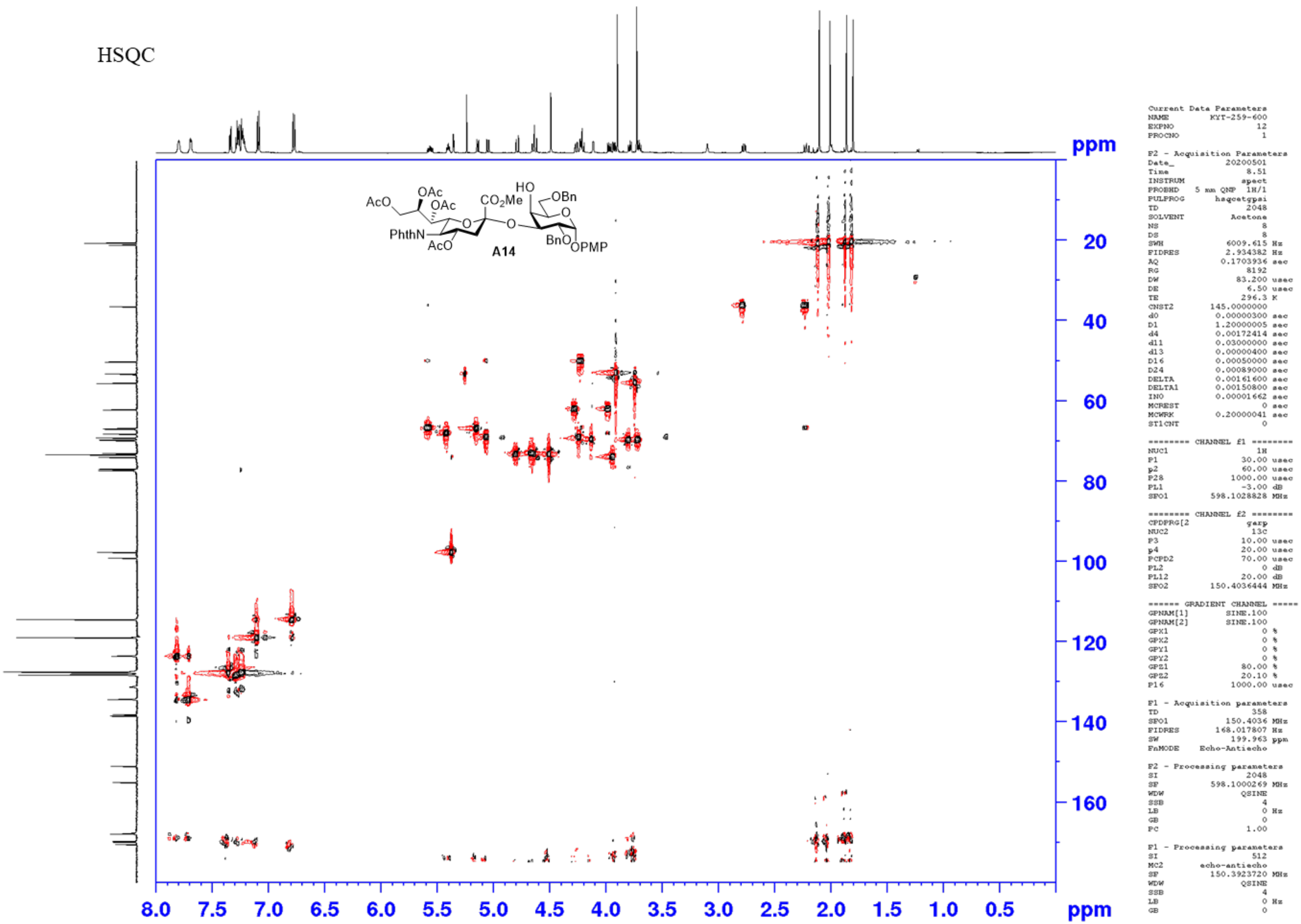
AcO
OAc
OAc
CO2Me
HO
OBn
O
O
O
PhthN
AcO
A14
BnO
OPMP
ppm
20
40
60
80
100
120
140
160
8.0
7.5
7.0
6.5
6.0
5.5
5.0
4.5
4.0
3.5
3.0
2.5
2.0
1.5
1.0
0.5
ppm
Current Data Parameters
NAME KYT-259-600
EXPNO 12
PROCNO 1
F2 - Acquisition Parameters
Date_ 20200501
Time 8.51
INSTRUM spect
PROBHD 5 mm QNP 1H/1
PULPROG hsqcetgpsi
TD 2048
SOLVENT Acetone
NS 8
DS 8
SWH 6009.615 Hz
FIDRES 2.934382 Hz
AQ 0.1703936 sec
RG 8192
DW 83.200 usec
DE 6.50 usec
TE 296.3 K
CNST2 145.0000000
d0 0.00000300 sec
D1 1.20000005 sec
d4 0.00172414 sec
d11 0.03000000 sec
d13 0.00000400 sec
D16 0.00050000 sec
D24 0.00089000 sec
DELTA 0.00161600 sec
DELTA1 0.00150800 sec
IN0 0.00001662 sec
MCREST 0 sec
MCWRK 0.20000041 sec
ST1CNT 0
======== CHANNEL f1 ========
NUC1 1H
P1 30.00 usec
p2 60.00 usec
P28 1000.00 usec
PL1 -3.00 dB
SFO1 598.1028828 MHz
======== CHANNEL f2 ========
CPDPRG[2 garp
NUC2 13C
P3 10.00 usec
p4 20.00 usec
PCPD2 70.00 usec
PL2 0 dB
PL12 20.00 dB
SFO2 150.4036444 MHz
====== GRADIENT CHANNEL =====
GPNAM[1] SINE.100
GPNAM[2] SINE.100
GPX1 0 %
GPX2 0 %
GPY1 0 %
GPY2 0 %
GPZ1 80.00 %
GPZ2 20.10 %
P16 1000.00 usec
F1 - Acquisition parameters
TD 358
SFO1 150.4036 MHz
FIDRES 168.017807 Hz
SW 199.963 ppm
FnMODE Echo-Antiecho
F2 - Processing parameters
SI 2048
SF 598.1000269 MHz
WDW QSINE
SSB 4
LB 0 Hz
GB 0
PC 1.00
F1 - Processing parameters
SI 512
MC2 echo-antiecho
SF 150.3923720 MHz
WDW QSINE
SSB 4
LB 0 Hz
GB 0

HMBC

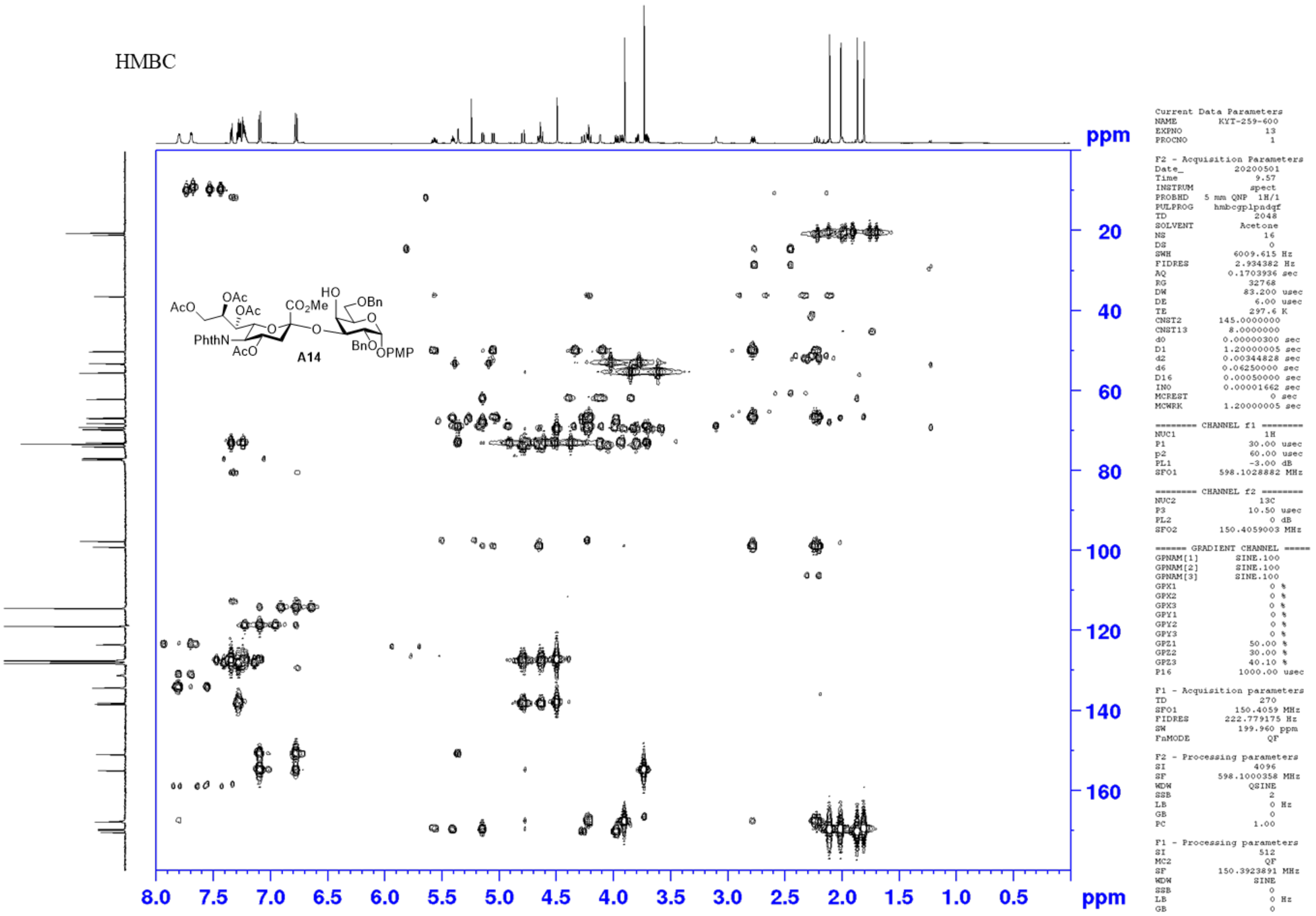
AcO
OAc
OAc
CO2Me
HO
OBn
O
PhthN
AcO
A14
BnO
OPMP
ppm
20
40
60
80
100
120
140
160
8.0
7.5
7.0
6.5
6.0
5.5
5.0
4.5
4.0
3.5
3.0
2.5
2.0
1.5
1.0
0.5
ppm
Current Data Parameters
NAME KYT-259-600
EXPNO 13
PROCNO 1
F2 - Acquisition Parameters
Date_ 20200501
Time 9.57
INSTRUM spect
PROBHD 5 mm QNP 1H/1
PULPROG hmbcgplpndqf
TD 2048
SOLVENT Acetone
NS 16
DS 0
SWH 6009.615 Hz
FIDRES 2.934382 Hz
AQ 0.1703936 sec
RG 32768
DW 83.200 usec
DE 6.00 usec
TE 297.6 K
CNST2 145.0000000
CNST13 8.0000000
d0 0.00000300 sec
D1 1.20000005 sec
d2 0.00344828 sec
d6 0.06250000 sec
D16 0.00050000 sec
IN0 0.00001662 sec
MCREST 0 sec
MCWRK 1.20000005 sec
======== CHANNEL f1 ========
NUC1 1H
P1 30.00 usec
p2 60.00 usec
PL1 -3.00 dB
SFO1 598.1028882 MHz
======== CHANNEL f2 ========
NUC2 13C
P3 10.50 usec
PL2 0 dB
SFO2 150.4059003 MHz
====== GRADIENT CHANNEL =====
GPNAM[1] SINE.100
GPNAM[2] SINE.100
GPNAM[3] SINE.100
GPX1 0 %
GPX2 0 %
GPX3 0 %
GPY1 0 %
GPY2 0 %
GPY3 0 %
GPZ1 50.00 %
GPZ2 30.00 %
GPZ3 40.10 %
P16 1000.00 usec
F1 - Acquisition parameters
TD 270
SFO1 150.4059 MHz
FIDRES 222.779175 Hz
SW 199.960 ppm
FnMODE QF
F2 - Processing parameters
SI 4096
SF 598.1000358 MHz
WDW QSINE
SSB 2
LB 0 Hz
GB 0
PC 1.00
F1 - Processing parameters
SI 512
MC2 QF
SF 150.3923891 MHz
WDW SINE
SSB 0
LB 0 Hz
GB 0

KYT-191-20190617

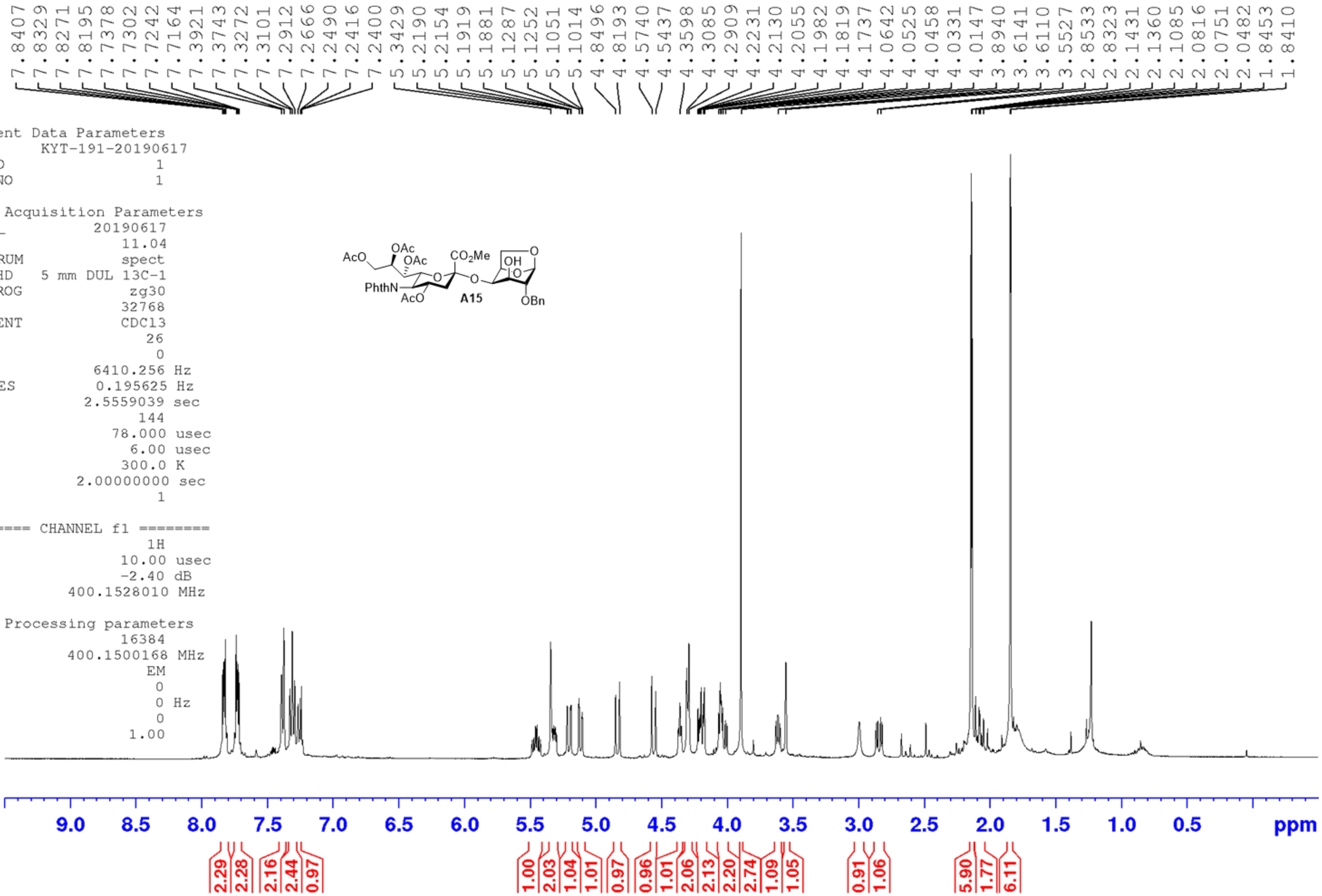

Current Data Parameters
NAME KYT-191-20190617
EXPNO 1
PROCNO 1

F2 - Acquisition Parameters
Date_ 20190617
Time 11.04
INSTRUM spect
PROBHD 5 mm DUL 13C-1
PULPROG zg30
TD 32768
SOLVENT CDCl3
NS 26
DS 0
SWH 6410.256 Hz
FIDRES 0.195625 Hz
AQ 2.5559039 sec
RG 144
DW 78.000 usec
DE 6.00 usec
TE 300.0 K
D1 2.00000000 sec
TD0 1

======== CHANNEL f1 ========
NUC1 1H
P1 10.00 usec
PL1 -2.40 dB
SFO1 400.1528010 MHz

F2 - Processing parameters
SI 16384
SF 400.1500168 MHz
WDW EM
SSB 0
LB 0 Hz
GB 0
PC 1.00

KYT-191-20190617

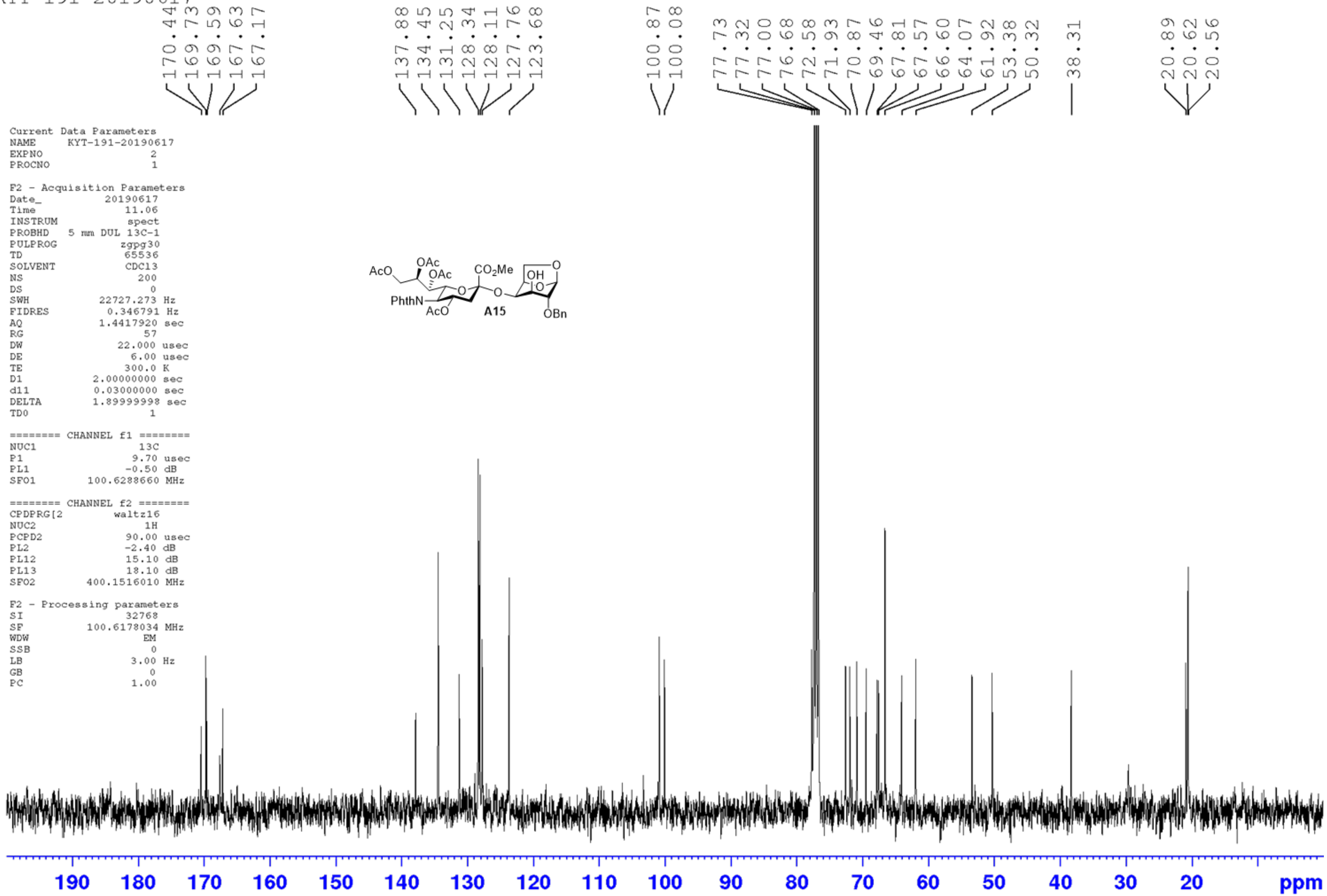


```
Current Data Parameters
NAME      KYT-191-20190617
EXPNO                    2
PROCNO                   1

F2 - Acquisition Parameters
Date_          20190617
Time              11.06
INSTRUM           spect
PROBHD   5 mm DUL 13C-1
PULPROG         zgpg30
TD               65536
SOLVENT          CDCl3
NS                 200
DS                   0
SWH          22727.273 Hz
FIDRES        0.346791 Hz
AQ           1.4417920 sec
RG                  57
DW              22.000 usec
DE                6.00 usec
TE               300.0 K
D1          2.00000000 sec
d11         0.03000000 sec
DELTA       1.89999998 sec
TD0                  1

======== CHANNEL f1 ========
NUC1               13C
P1                9.70 usec
PL1              -0.50 dB
SFO1       100.6288660 MHz

======== CHANNEL f2 ========
CPDPRG[2        waltz16
NUC2                1H
PCPD2            90.00 usec
PL2              -2.40 dB
PL12             15.10 dB
PL13             18.10 dB
SFO2       400.1516010 MHz

F2 - Processing parameters
SI               32768
SF         100.6178034 MHz
WDW                 EM
SSB                  0
LB                3.00 Hz
GB                   0
PC                1.00
```

YYS-546-20221104-C-H

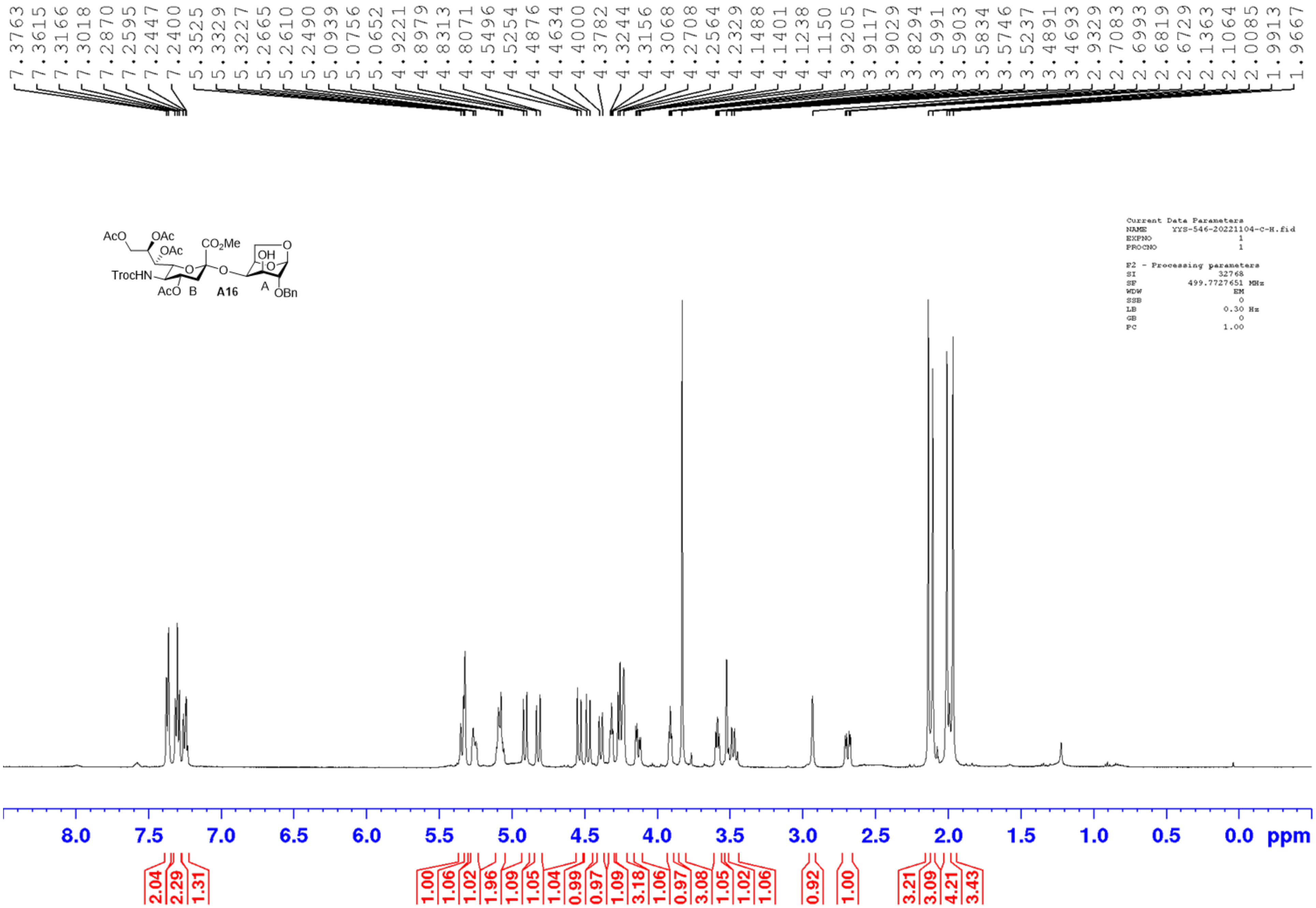

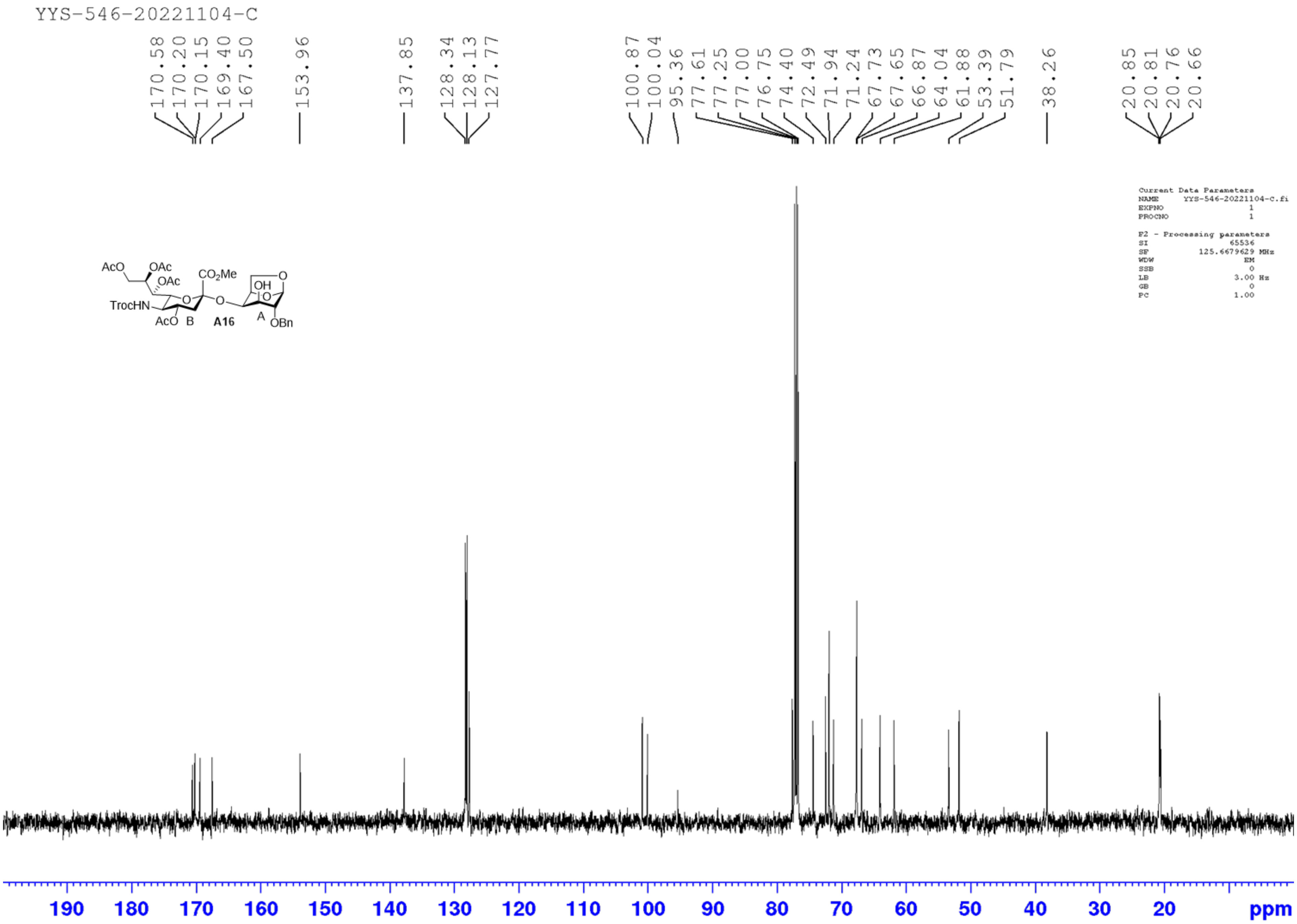
YYS-546-20221104-C
170.58
170.20
170.15
169.40
167.50
153.96
137.85
128.34
128.13
127.77
100.87
100.04
95.36
77.61
77.25
77.00
76.75
74.40
72.49
71.94
71.24
67.73
67.65
66.87
64.04
61.88
53.39
51.79
38.26
20.85
20.81
20.76
20.66
AcO
OAc
OAc
CO_2Me
TrocHN
AcO
B
A16
A
OH
OBn
Current Data Parameters
NAME YYS-546-20221104-C.fi
EXPNO 1
PROCNO 1
F2 - Processing parameters
SI 65536
SF 125.6679629 MHz
WDW EM
SSB 0
LB 3.00 Hz
GB 0
PC 1.00
190
180
170
160
150
140
130
120
110
100
90
80
70
60
50
40
30
20
ppm

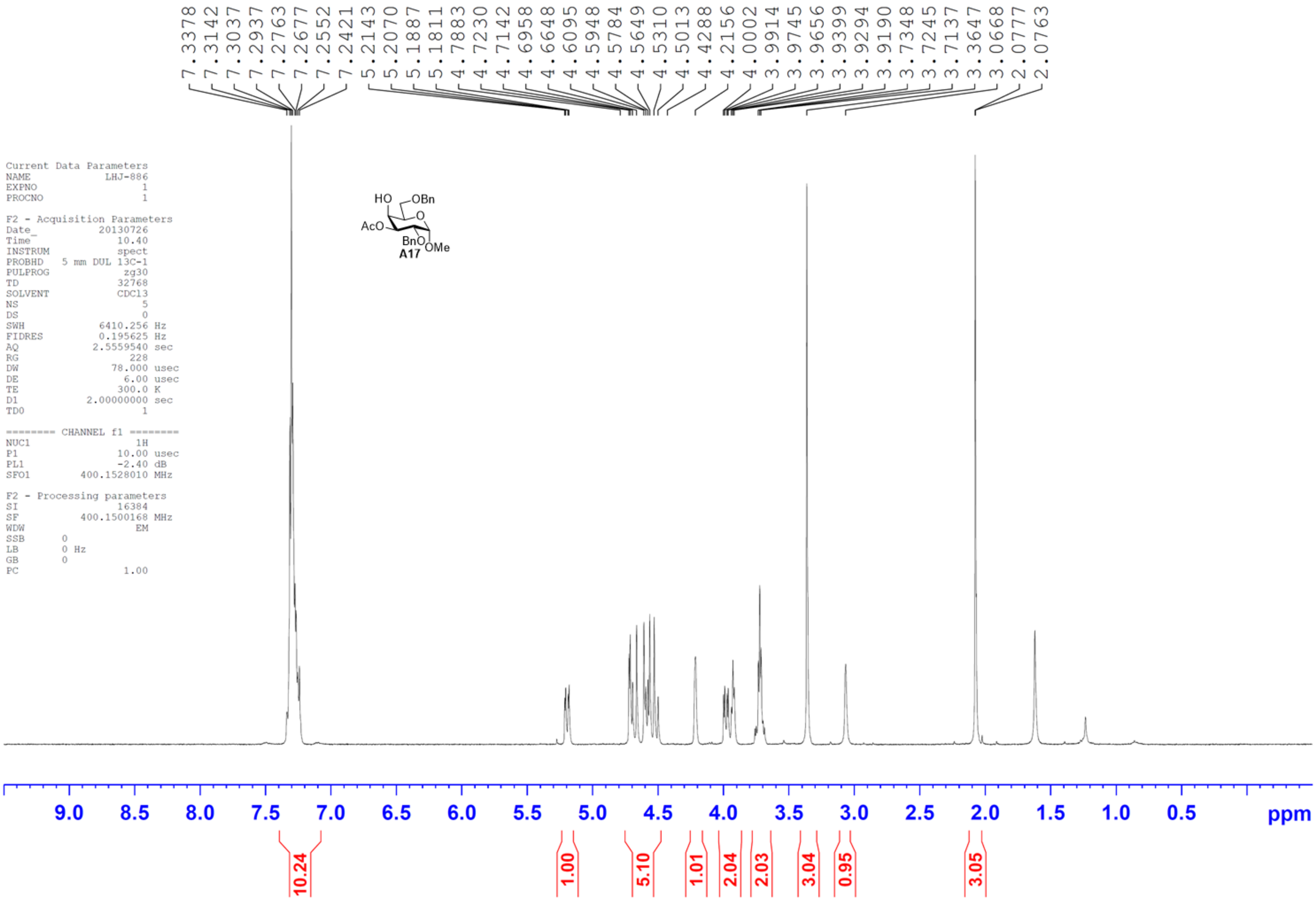

7.3378
7.3142
7.3037
7.2937
7.2763
7.2677
7.2552
7.2421
5.2143
5.2070
5.1887
5.1811
4.7883
4.7230
4.7142
4.6958
4.6648
4.6095
4.5948
4.5784
4.5649
4.5310
4.5013
4.4288
4.2156
4.0002
3.9914
3.9745
3.9656
3.9399
3.9294
3.9190
3.7348
3.7245
3.7137
3.3647
3.0668
2.0777
2.0763
Current Data Parameters
NAME LHJ-886
EXPNO 1
PROCNO 1
F2 - Acquisition Parameters
Date_ 20130726
Time 10.40
INSTRUM spect
PROBHD 5 mm DUL 13C-1
PULPROG zg30
TD 32768
SOLVENT CDCl3
NS 5
DS 0
SWH 6410.256 Hz
FIDRES 0.195625 Hz
AQ 2.5559540 sec
RG 228
DW 78.000 usec
DE 6.00 usec
TE 300.0 K
D1 2.00000000 sec
TD0 1
======== CHANNEL f1 ========
NUC1 1H
P1 10.00 usec
PL1 -2.40 dB
SFO1 400.1528010 MHz
F2 - Processing parameters
SI 16384
SF 400.1500168 MHz
WDW EM
SSB 0
LB 0 Hz
GB 0
PC 1.00
HO
OBn
O
AcO
BnO
OMe
A17
9.0
8.5
8.0
7.5
7.0
6.5
6.0
5.5
5.0
4.5
4.0
3.5
3.0
2.5
2.0
1.5
1.0
0.5
ppm
10.24
1.00
5.10
1.01
2.04
2.03
3.04
0.95
3.05

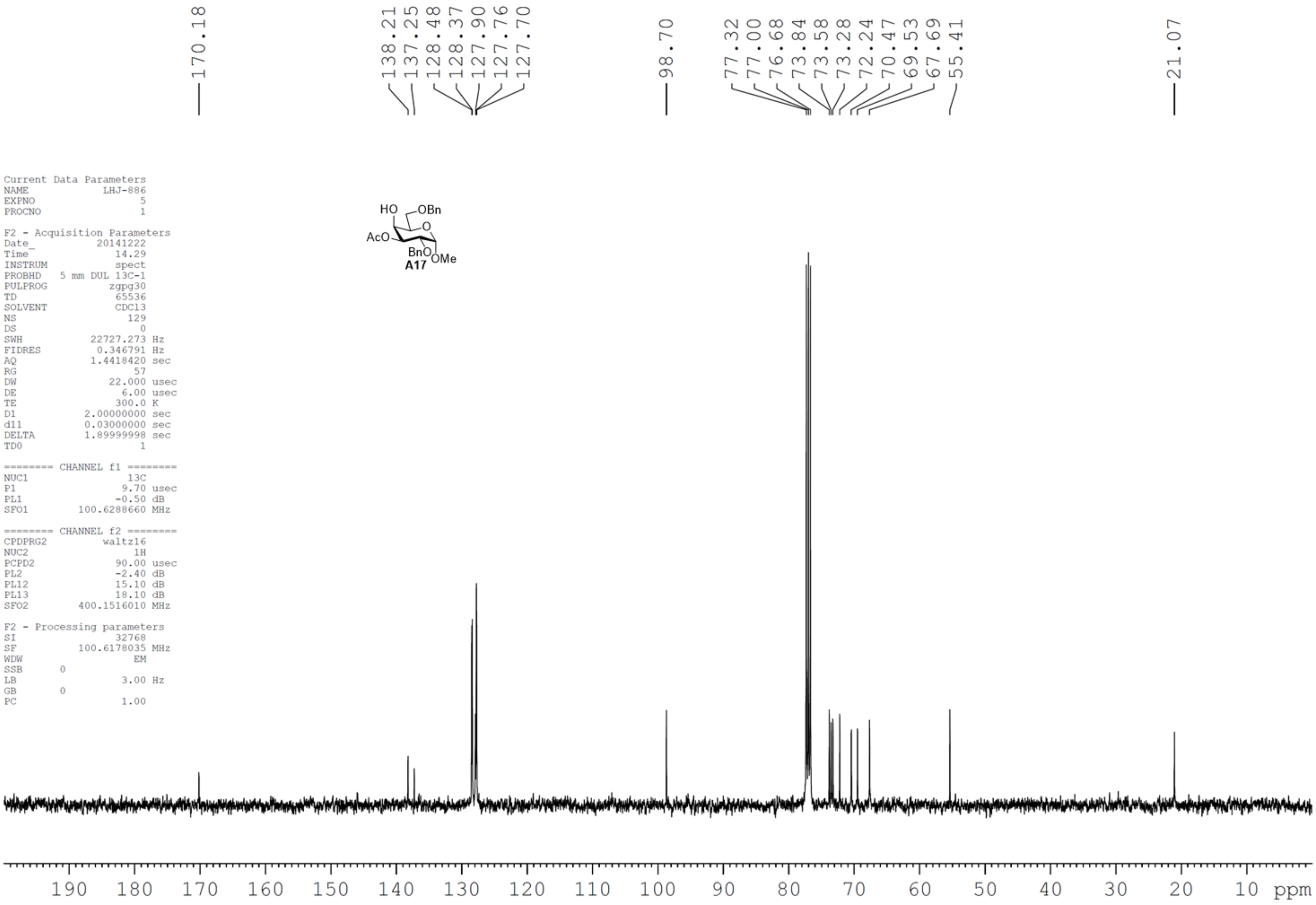

Current Data Parameters
NAME LHJ-886
EXPNO 5
PROCNO 1

F2 - Acquisition Parameters
Date_ 20141222
Time 14.29
INSTRUM spect
PROBHD 5 mm DUL 13C-1
PULPROG zgpg30
TD 65536
SOLVENT CDCl3
NS 129
DS 0
SWH 22727.273 Hz
FIDRES 0.346791 Hz
AQ 1.4418420 sec
RG 57
DW 22.000 usec
DE 6.00 usec
TE 300.0 K
D1 2.00000000 sec
d11 0.03000000 sec
DELTA 1.89999998 sec
TD0 1

======== CHANNEL f1 ========
NUC1 13C
P1 9.70 usec
PL1 -0.50 dB
SFO1 100.6288660 MHz

======== CHANNEL f2 ========
CPDPRG2 waltz16
NUC2 1H
PCPD2 90.00 usec
PL2 -2.40 dB
PL12 15.10 dB
PL13 18.10 dB
SFO2 400.1516010 MHz

F2 - Processing parameters
SI 32768
SF 100.6178035 MHz
WDW EM
SSB 0
LB 3.00 Hz
GB 0
PC 1.00

A17

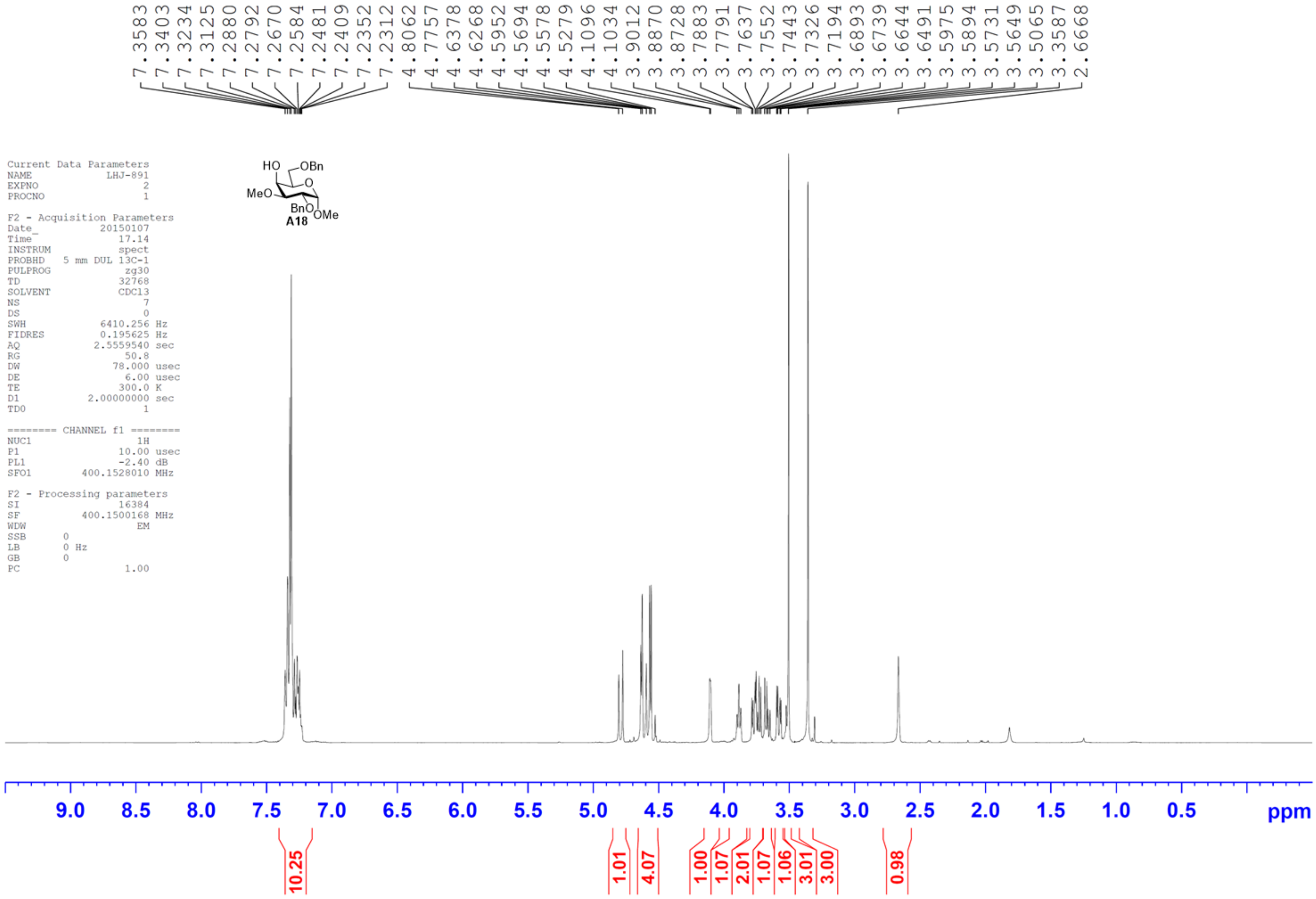
7.3583
7.3403
7.3234
7.3125
7.2880
7.2792
7.2670
7.2584
7.2481
7.2409
7.2352
7.2312
4.8062
4.7757
4.6378
4.6268
4.5952
4.5694
4.5578
4.5279
4.1096
4.1034
3.9012
3.8870
3.8728
3.7883
3.7791
3.7637
3.7552
3.7443
3.7326
3.7194
3.6893
3.6739
3.6644
3.6491
3.5975
3.5894
3.5731
3.5649
3.5065
3.3587
2.6668
Current Data Parameters
NAME LHJ-891
EXPNO 2
PROCNO 1
F2 - Acquisition Parameters
Date_ 20150107
Time 17.14
INSTRUM spect
PROBHD 5 mm DUL 13C-1
PULPROG zg30
TD 32768
SOLVENT CDCl3
NS 7
DS 0
SWH 6410.256 Hz
FIDRES 0.195625 Hz
AQ 2.5559540 sec
RG 50.8
DW 78.000 usec
DE 6.00 usec
TE 300.0 K
D1 2.00000000 sec
TD0 1
======== CHANNEL f1 ========
NUC1 1H
P1 10.00 usec
PL1 -2.40 dB
SFO1 400.1528010 MHz
F2 - Processing parameters
SI 16384
SF 400.1500168 MHz
WDW EM
SSB 0
LB 0 Hz
GB 0
PC 1.00
HO
OBn
O
MeO
BnO
OMe
A18
9.0
8.5
8.0
7.5
7.0
6.5
6.0
5.5
5.0
4.5
4.0
3.5
3.0
2.5
2.0
1.5
1.0
0.5
ppm
10.25
1.01
4.07
1.00
1.07
2.01
1.07
1.06
3.01
3.00
0.98

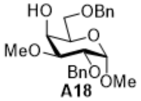


```
Current Data Parameters
NAME              LHJ-891
EXPNO                   3
PROCNO                  1

F2 - Acquisition Parameters
Date_            20150107
Time                17.15
INSTRUM             spect
PROBHD   5 mm DUL 13C-1
PULPROG           zgpg30
TD                 65536
SOLVENT            CDCl3
NS                    70
DS                     0
SWH            22727.273 Hz
FIDRES          0.346791 Hz
AQ             1.4418420 sec
RG                    57
DW                22.000 usec
DE                  6.00 usec
TE                 300.0 K
D1            2.00000000 sec
d11           0.03000000 sec
DELTA         1.89999998 sec
TD0                    1

======== CHANNEL f1 ========
NUC1                 13C
P1                  9.70 usec
PL1                -0.50 dB
SFO1         100.6288660 MHz

======== CHANNEL f2 ========
CPDPRG2          waltz16
NUC2                  1H
PCPD2              90.00 usec
PL2                -2.40 dB
PL12               15.10 dB
PL13               18.10 dB
SFO2         400.1516010 MHz

F2 - Processing parameters
SI                 32768
SF           100.6178133 MHz
WDW                   EM
SSB      0
LB                  3.00 Hz
GB       0
PC                  1.00
```

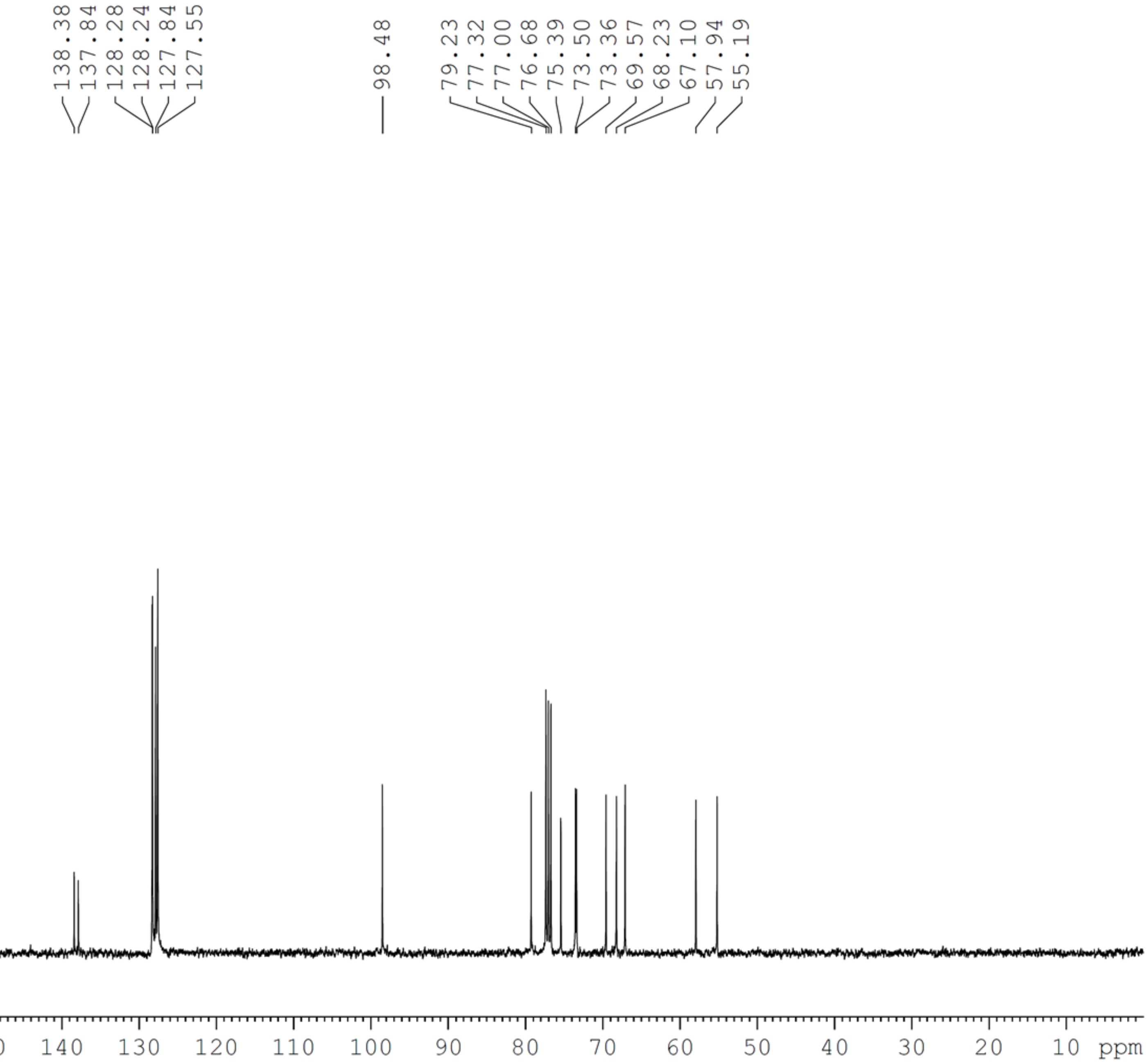

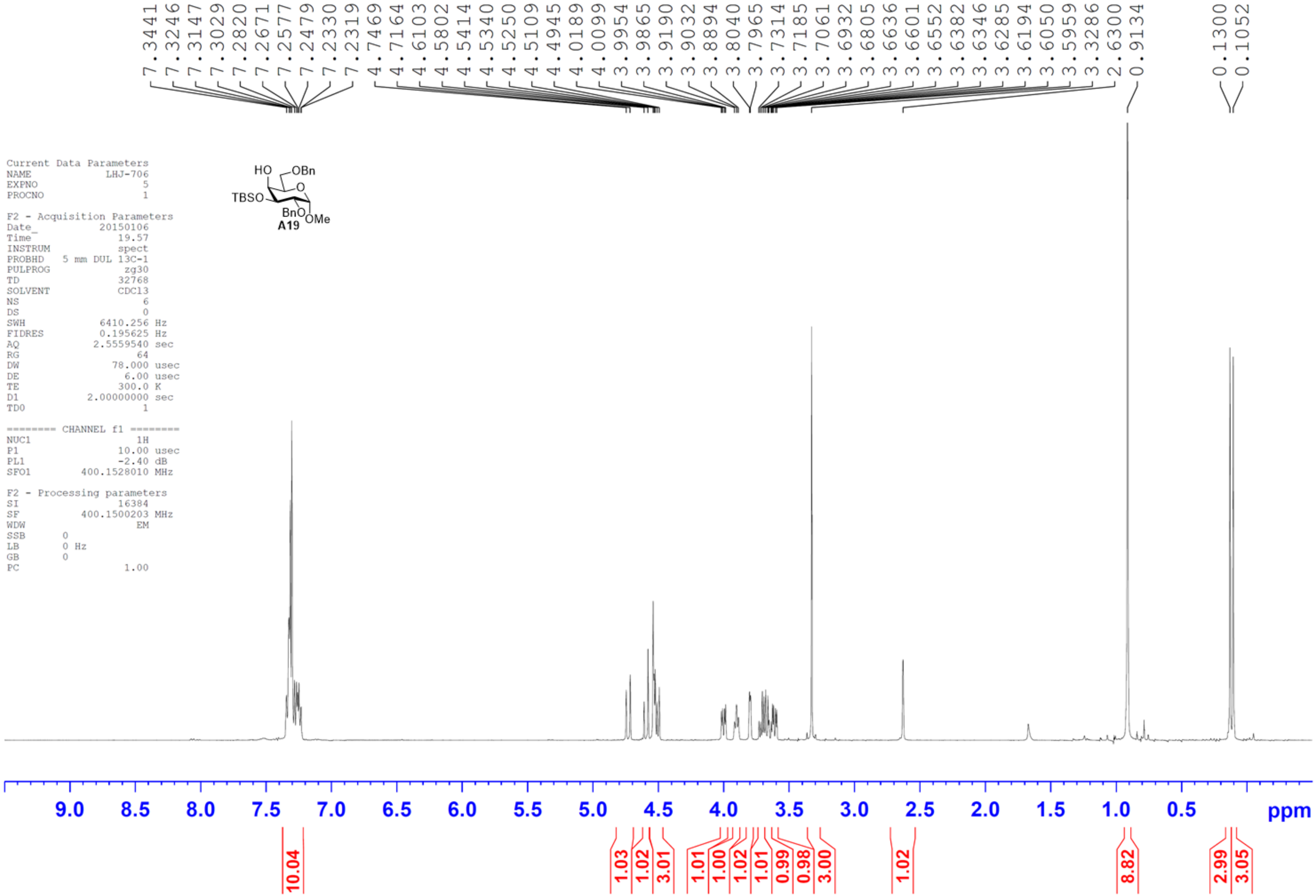
7.3441
7.3246
7.3147
7.3029
7.2820
7.2671
7.2577
7.2479
7.2330
7.2319
4.7469
4.7164
4.6103
4.5802
4.5414
4.5340
4.5250
4.5109
4.4945
4.0189
4.0099
3.9954
3.9865
3.9190
3.9032
3.8894
3.8040
3.7965
3.7314
3.7185
3.7061
3.6932
3.6805
3.6636
3.6601
3.6552
3.6382
3.6346
3.6285
3.6194
3.6050
3.5959
3.3286
2.6300
0.9134
0.1300
0.1052
Current Data Parameters
NAME LHJ-706
EXPNO 5
PROCNO 1
F2 - Acquisition Parameters
Date_ 20150106
Time 19.57
INSTRUM spect
PROBHD 5 mm DUL 13C-1
PULPROG zg30
TD 32768
SOLVENT CDCl3
NS 6
DS 0
SWH 6410.256 Hz
FIDRES 0.195625 Hz
AQ 2.5559540 sec
RG 64
DW 78.000 usec
DE 6.00 usec
TE 300.0 K
D1 2.00000000 sec
TD0 1
======== CHANNEL f1 ========
NUC1 1H
P1 10.00 usec
PL1 -2.40 dB
SFO1 400.1528010 MHz
F2 - Processing parameters
SI 16384
SF 400.1500203 MHz
WDW EM
SSB 0
LB 0 Hz
GB 0
PC 1.00
HO
OBn
O
TBSO
BnO
OMe
A19
9.0
8.5
8.0
7.5
7.0
6.5
6.0
5.5
5.0
4.5
4.0
3.5
3.0
2.5
2.0
1.5
1.0
0.5
ppm
10.04
1.03
1.02
3.01
1.01
1.00
1.02
1.01
0.99
0.98
3.00
1.02
8.82
2.99
3.05

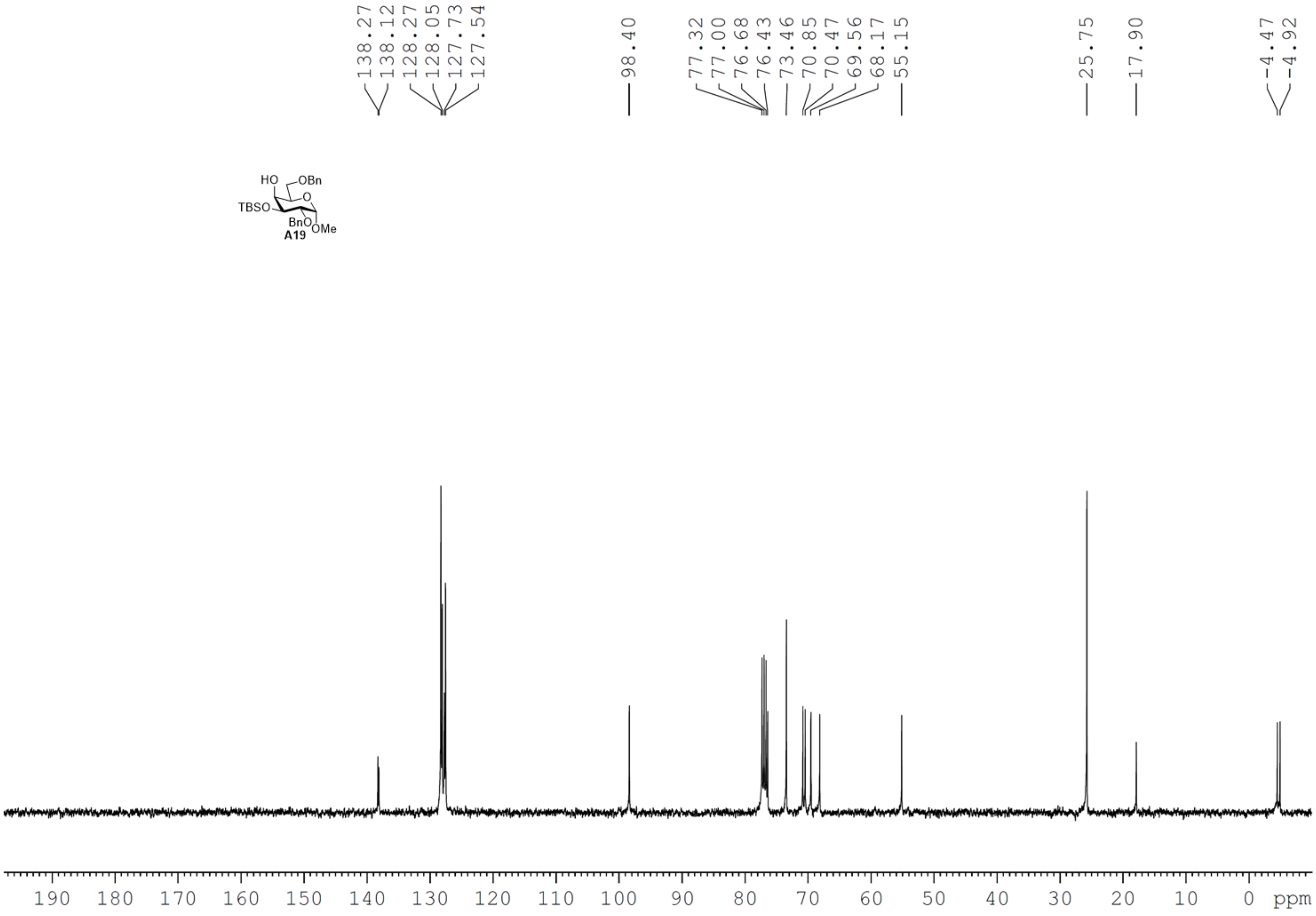

138.27
138.12
128.27
128.05
127.73
127.54
98.40
77.32
77.00
76.68
76.43
73.46
70.85
70.47
69.56
68.17
55.15
25.75
17.90
-4.47
-4.92
HO
OBn
TBSO
O
BnO
OMe
A19
190 180 170 160 150 140 130 120 110 100 90 80 70 60 50 40 30 20 10 0 ppm

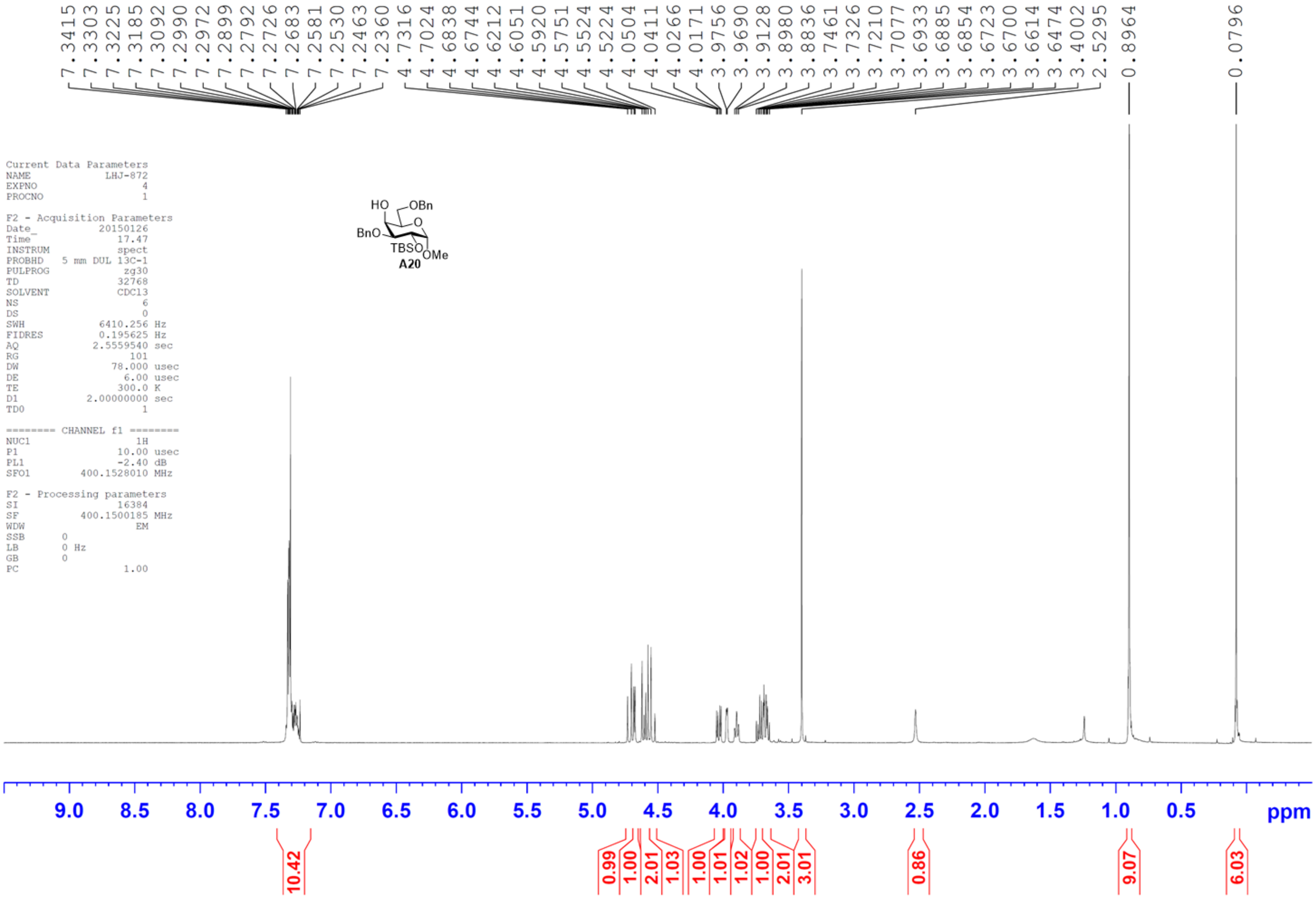
7.3415
7.3303
7.3225
7.3185
7.3092
7.2990
7.2972
7.2899
7.2792
7.2726
7.2683
7.2581
7.2530
7.2463
7.2360
4.7316
4.7024
4.6838
4.6744
4.6212
4.6051
4.5920
4.5751
4.5524
4.5224
4.0504
4.0411
4.0266
4.0171
3.9756
3.9690
3.9128
3.8980
3.8836
3.7461
3.7326
3.7210
3.7077
3.6933
3.6885
3.6854
3.6723
3.6700
3.6614
3.6474
3.4002
2.5295
0.8964
0.0796
Current Data Parameters
NAME LHJ-872
EXPNO 4
PROCNO 1
F2 - Acquisition Parameters
Date_ 20150126
Time 17.47
INSTRUM spect
PROBHD 5 mm DUL 13C-1
PULPROG zg30
TD 32768
SOLVENT CDCl3
NS 6
DS 0
SWH 6410.256 Hz
FIDRES 0.195625 Hz
AQ 2.5559540 sec
RG 101
DW 78.000 usec
DE 6.00 usec
TE 300.0 K
D1 2.00000000 sec
TD0 1
======== CHANNEL f1 ========
NUC1 1H
P1 10.00 usec
PL1 -2.40 dB
SFO1 400.1528010 MHz
F2 - Processing parameters
SI 16384
SF 400.1500185 MHz
WDW EM
SSB 0
LB 0 Hz
GB 0
PC 1.00
HO
OBn
O
BnO
TBSO
OMe
A20
9.0
8.5
8.0
7.5
7.0
6.5
6.0
5.5
5.0
4.5
4.0
3.5
3.0
2.5
2.0
1.5
1.0
0.5
ppm
10.42
0.99
1.00
2.01
1.03
1.00
1.01
1.02
1.00
2.01
3.01
0.86
9.07
6.03

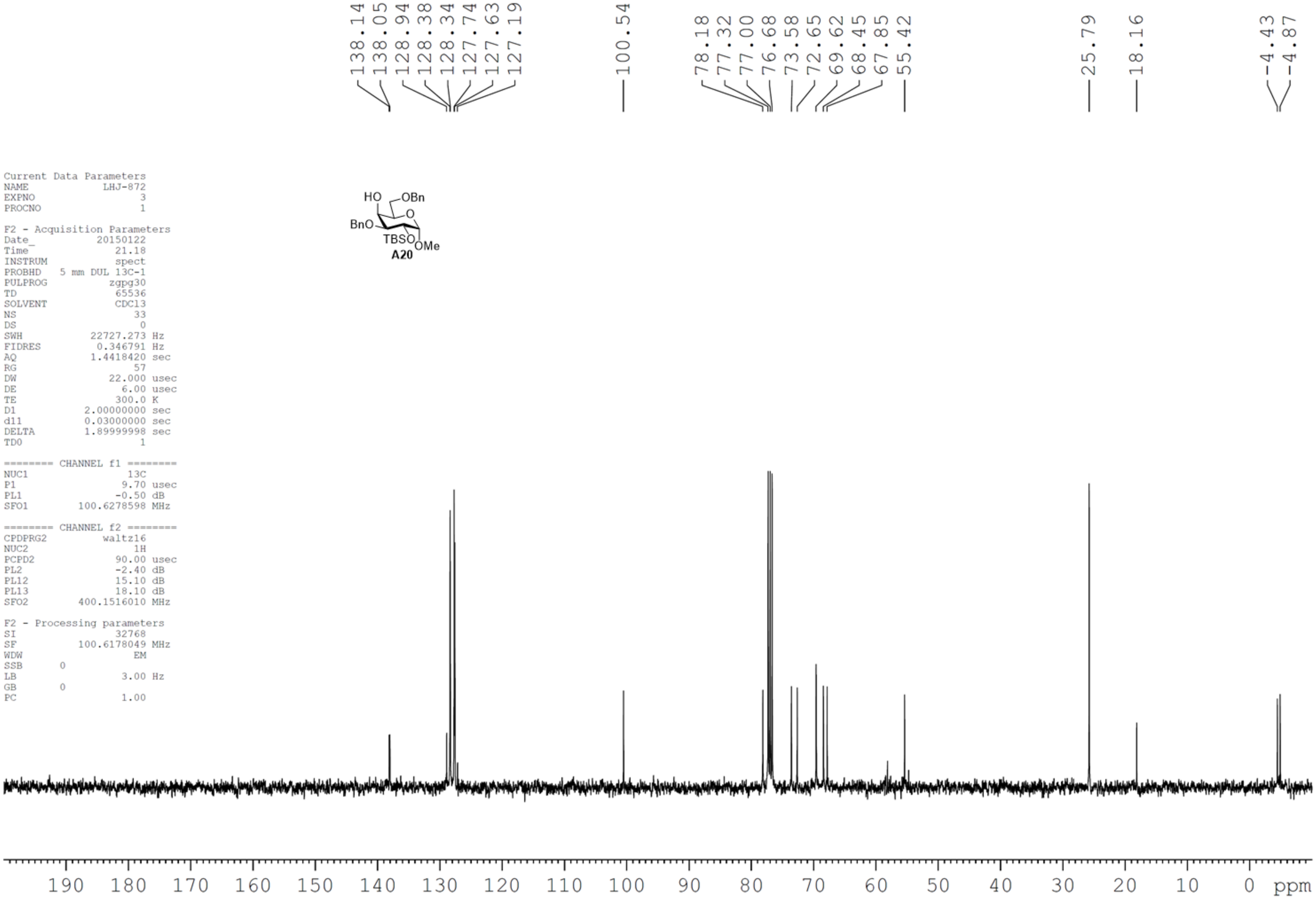


```
Current Data Parameters
NAME             LHJ-872
EXPNO                  3
PROCNO                 1

F2 - Acquisition Parameters
Date_           20150122
Time               21.18
INSTRUM            spect
PROBHD   5 mm DUL 13C-1
PULPROG          zgpg30
TD                 65536
SOLVENT            CDCl3
NS                    33
DS                     0
SWH          22727.273 Hz
FIDRES        0.346791 Hz
AQ           1.4418420 sec
RG                    57
DW              22.000 usec
DE                6.00 usec
TE               300.0 K
D1          2.00000000 sec
d11         0.03000000 sec
DELTA       1.89999998 sec
TD0                    1

======== CHANNEL f1 ========
NUC1                 13C
P1                  9.70 usec
PL1                -0.50 dB
SFO1         100.6278598 MHz

======== CHANNEL f2 ========
CPDPRG2          waltz16
NUC2                  1H
PCPD2              90.00 usec
PL2                -2.40 dB
PL12               15.10 dB
PL13               18.10 dB
SFO2         400.1516010 MHz

F2 - Processing parameters
SI                 32768
SF           100.6178049 MHz
WDW                   EM
SSB      0
LB                  3.00 Hz
GB       0
PC                  1.00
```


A20

KYT-177-OAc

```
Current Data Parameters
NAME          KYT-177-OAc
EXPNO                   1
PROCNO                  1

F2 - Acquisition Parameters
Date_            20190604
Time                13.42
INSTRUM             spect
PROBHD   5 mm DUL 13C-1
PULPROG              zg30
TD                  32768
SOLVENT             CDCl3
NS                     23
DS                      0
SWH              6410.256 Hz
FIDRES           0.195625 Hz
AQ              2.5559039 sec
RG                    114
DW                 78.000 usec
DE                   6.00 usec
TE                  300.0 K
D1             2.00000000 sec
TD0                     1

======== CHANNEL f1 ========
NUC1                   1H
P1                  10.00 usec
PL1                 -2.40 dB
SFO1          400.1528010 MHz

F2 - Processing parameters
SI                  16384
SF            400.1500168 MHz
WDW                    EM
SSB                     0
LB                      0 Hz
GB                      0
PC                   1.00
```

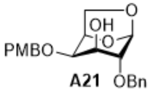

A21

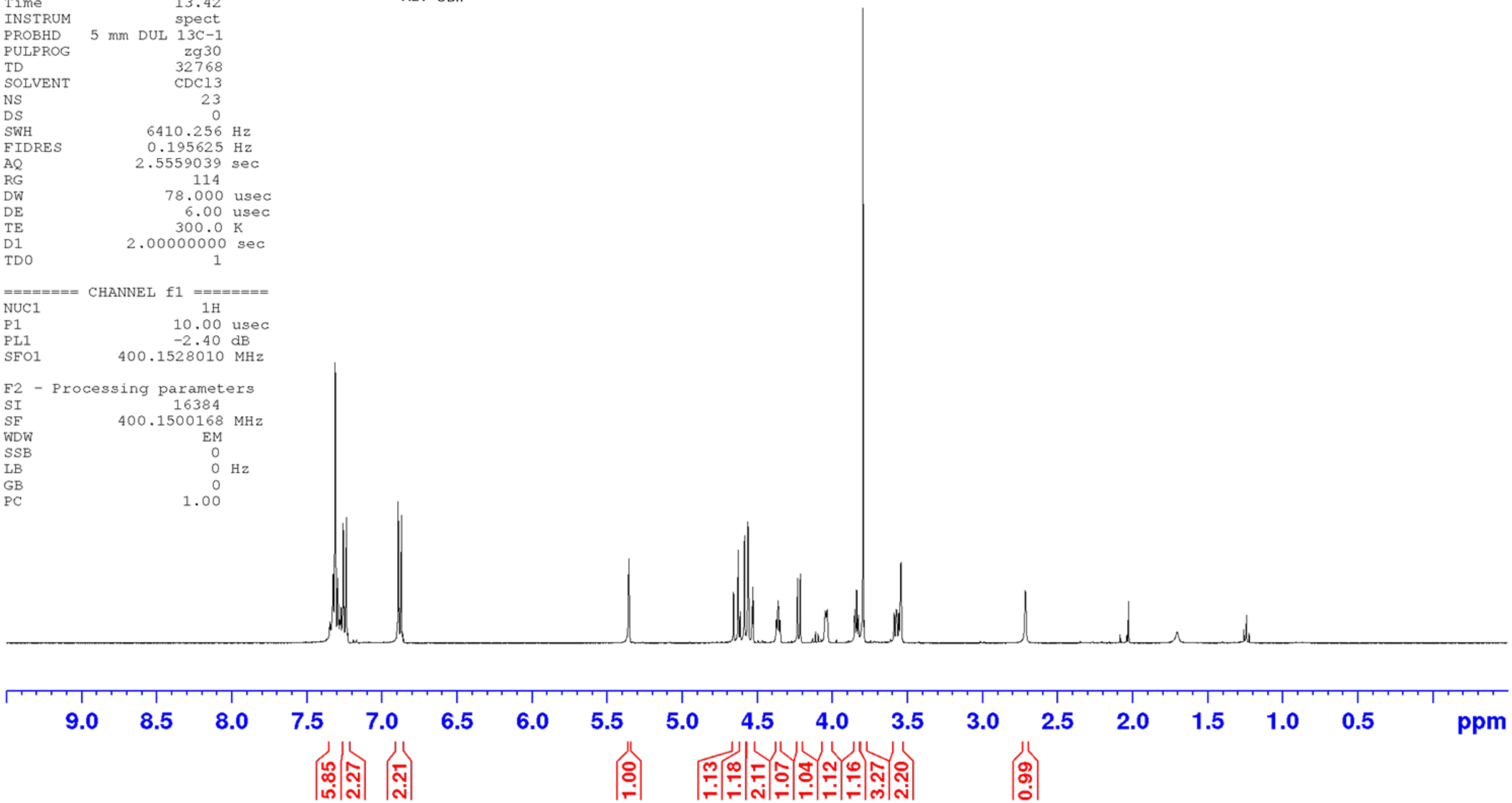

KYT-177-OAc

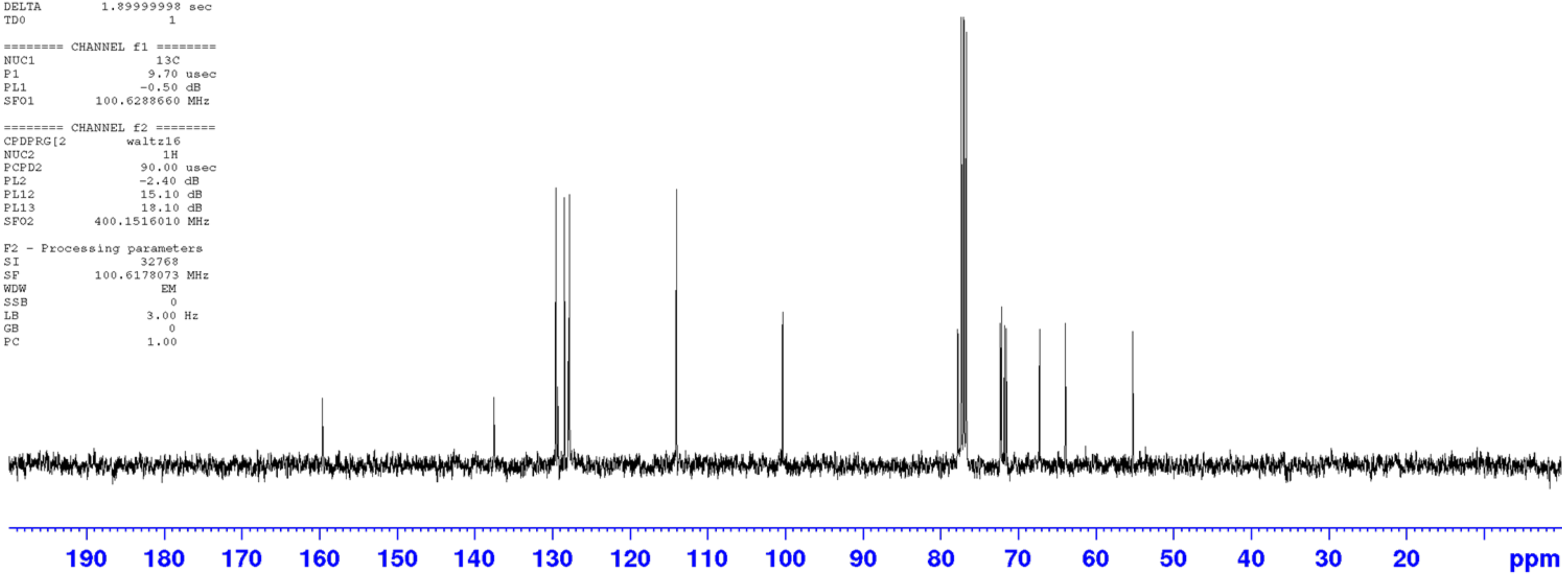

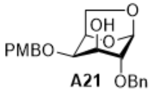

A21

```
Current Data Parameters
NAME          KYT-177-OAc
EXPNO                   2
PROCNO                  1

F2 - Acquisition Parameters
Date_            20190604
Time                13.57
INSTRUM             spect
PROBHD   5 mm DUL 13C-1
PULPROG           zgpg30
TD                 65536
SOLVENT            CDCl3
NS                    29
DS                     0
SWH            22727.273 Hz
FIDRES          0.346791 Hz
AQ             1.4417920 sec
RG                    57
DW                22.000 usec
DE                  6.00 usec
TE                 300.0 K
D1            2.00000000 sec
d11           0.03000000 sec
DELTA         1.89999998 sec
TD0                    1

======== CHANNEL f1 ========
NUC1                 13C
P1                  9.70 usec
PL1                -0.50 dB
SFO1         100.6288660 MHz

======== CHANNEL f2 ========
CPDPRG[2         waltz16
NUC2                  1H
PCPD2              90.00 usec
PL2                -2.40 dB
PL12               15.10 dB
PL13               18.10 dB
SFO2         400.1516010 MHz

F2 - Processing parameters
SI                 32768
SF           100.6178073 MHz
WDW                   EM
SSB                    0
LB                  3.00 Hz
GB                     0
PC                  1.00
```

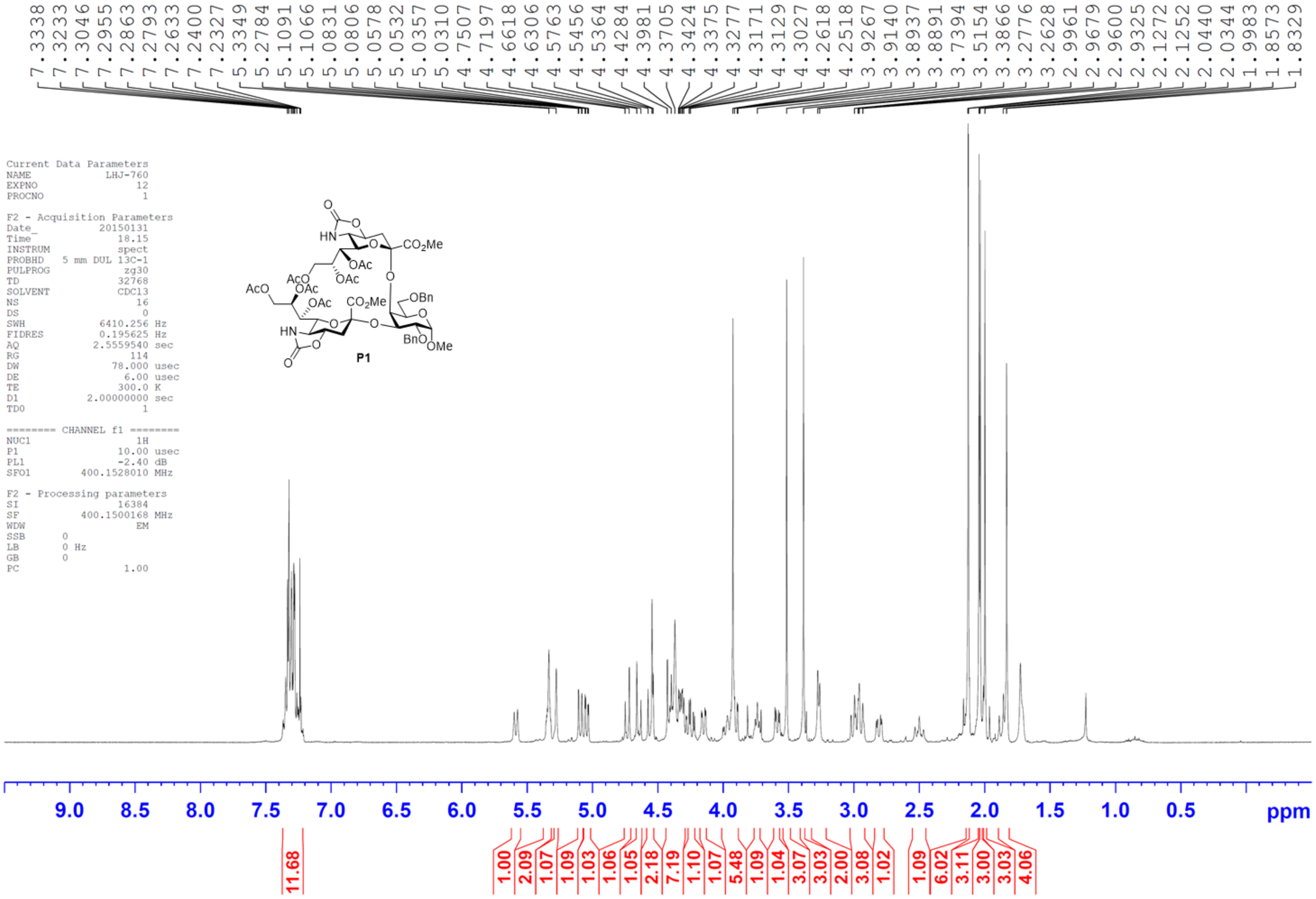

7.3338
7.3233
7.3046
7.2955
7.2863
7.2793
7.2633
7.2400
7.2327
5.3349
5.2784
5.1091
5.1066
5.0831
5.0806
5.0578
5.0532
5.0357
5.0310
4.7507
4.7197
4.6618
4.6306
4.5763
4.5456
4.5364
4.4284
4.3981
4.3705
4.3424
4.3375
4.3277
4.3171
4.3129
4.3027
4.2618
4.2518
3.9267
3.9140
3.8937
3.8891
3.7394
3.5154
3.3866
3.2776
3.2628
2.9961
2.9679
2.9600
2.9325
2.1272
2.1252
2.0440
2.0344
1.9983
1.8573
1.8329
Current Data Parameters
NAME LHJ-760
EXPNO 12
PROCNO 1
F2 - Acquisition Parameters
Date_ 20150131
Time 18.15
INSTRUM spect
PROBHD 5 mm DUL 13C-1
PULPROG zg30
TD 32768
SOLVENT CDCl3
NS 16
DS 0
SWH 6410.256 Hz
FIDRES 0.195625 Hz
AQ 2.5559540 sec
RG 114
DW 78.000 usec
DE 6.00 usec
TE 300.0 K
D1 2.00000000 sec
TD0 1
======== CHANNEL f1 ========
NUC1 1H
P1 10.00 usec
PL1 -2.40 dB
SFO1 400.1528010 MHz
F2 - Processing parameters
SI 16384
SF 400.1500168 MHz
WDW EM
SSB 0
LB 0 Hz
GB 0
PC 1.00
HN
O
O
O
CO2Me
OAc
OAc
AcO
OAc
AcO
OAc
CO2Me
OBn
HN
BnO
OMe
P1
9.0
8.5
8.0
7.5
7.0
6.5
6.0
5.5
5.0
4.5
4.0
3.5
3.0
2.5
2.0
1.5
1.0
0.5
ppm
11.68
1.00
2.09
1.07
1.09
1.03
1.06
1.05
2.18
7.19
1.10
1.07
5.48
1.09
1.04
3.07
3.03
2.00
3.08
1.02
1.09
6.02
3.11
3.00
3.03
4.06

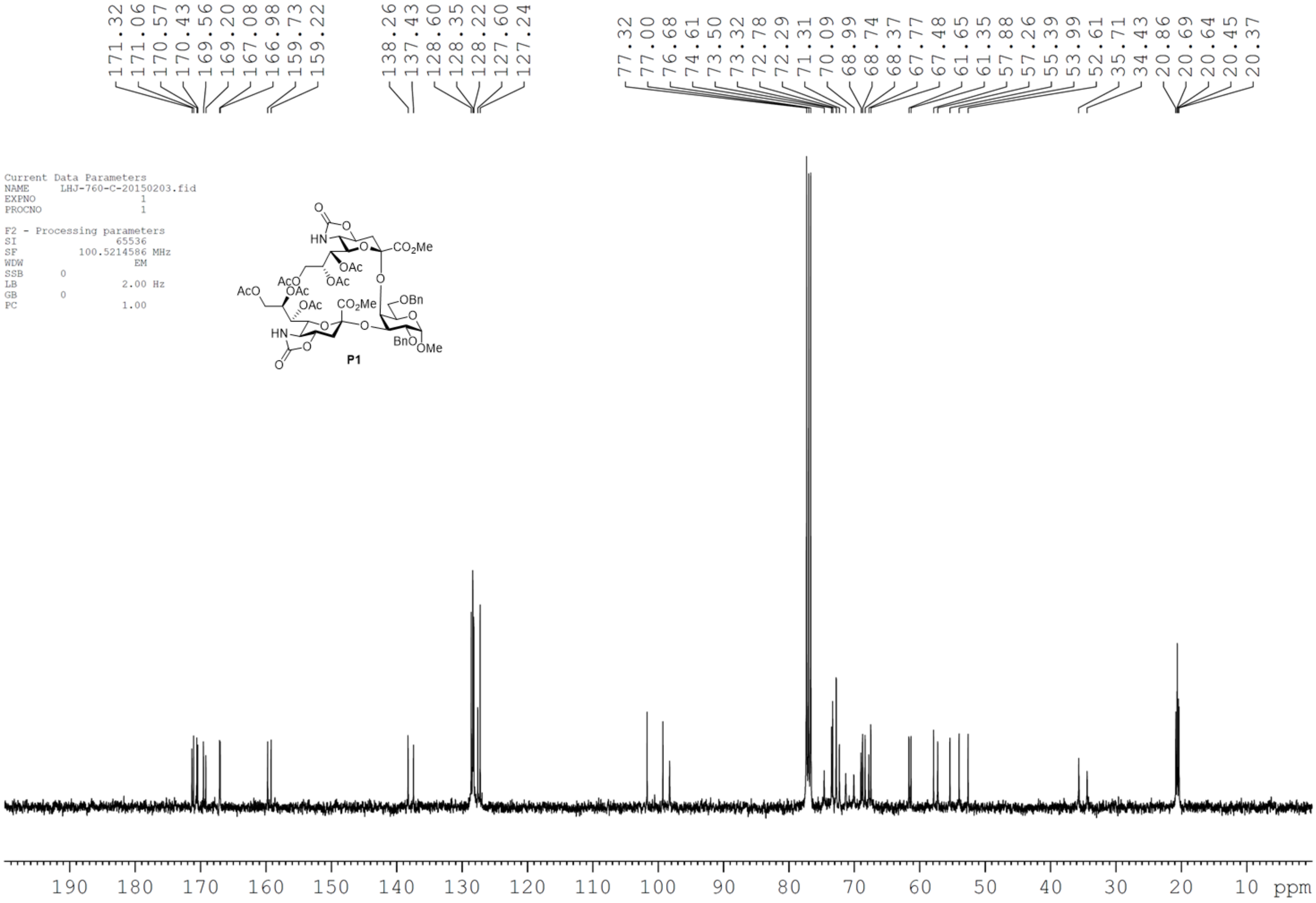
171.32
171.06
170.57
170.43
169.56
169.20
167.08
166.98
159.73
159.22
138.26
137.43
128.60
128.35
128.22
127.60
127.24
77.32
77.00
76.68
74.61
73.50
73.32
72.78
72.29
71.31
70.09
68.99
68.74
68.37
67.77
67.48
61.65
61.35
57.88
57.26
55.39
53.99
52.61
35.71
34.43
20.86
20.69
20.64
20.45
20.37
Current Data Parameters
NAME LHJ-760-C-20150203.fid
EXPNO 1
PROCNO 1
F2 - Processing parameters
SI 65536
SF 100.5214586 MHz
WDW EM
SSB 0
LB 2.00 Hz
GB 0
PC 1.00
HN
CO2Me
OAc
AcO
OBn
BnO
OMe
P1
190 180 170 160 150 140 130 120 110 100 90 80 70 60 50 40 30 20 10 ppm

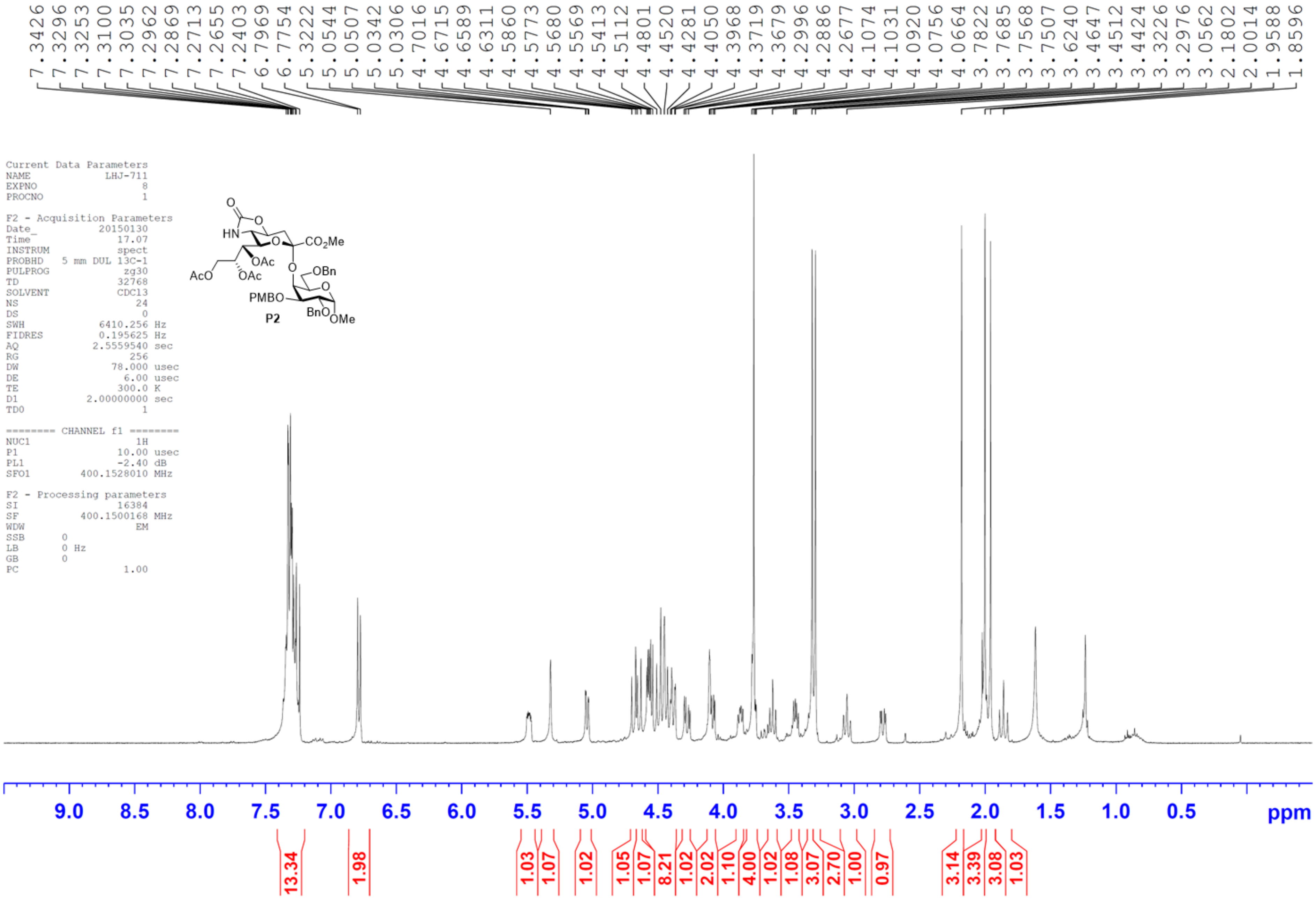
Current Data Parameters
NAME LHJ-711
EXPNO 8
PROCNO 1
F2 - Acquisition Parameters
Date_ 20150130
Time 17.07
INSTRUM spect
PROBHD 5 mm DUL 13C-1
PULPROG zg30
TD 32768
SOLVENT CDCl3
NS 24
DS 0
SWH 6410.256 Hz
FIDRES 0.195625 Hz
AQ 2.5559540 sec
RG 256
DW 78.000 usec
DE 6.00 usec
TE 300.0 K
D1 2.00000000 sec
TD0 1
======== CHANNEL f1 ========
NUC1 1H
P1 10.00 usec
PL1 -2.40 dB
SFO1 400.1528010 MHz
F2 - Processing parameters
SI 16384
SF 400.1500168 MHz
WDW EM
SSB 0
LB 0 Hz
GB 0
PC 1.00
HN
CO2Me
OAc
AcO
OBn
PMBO
BnO
OMe
P2
ppm

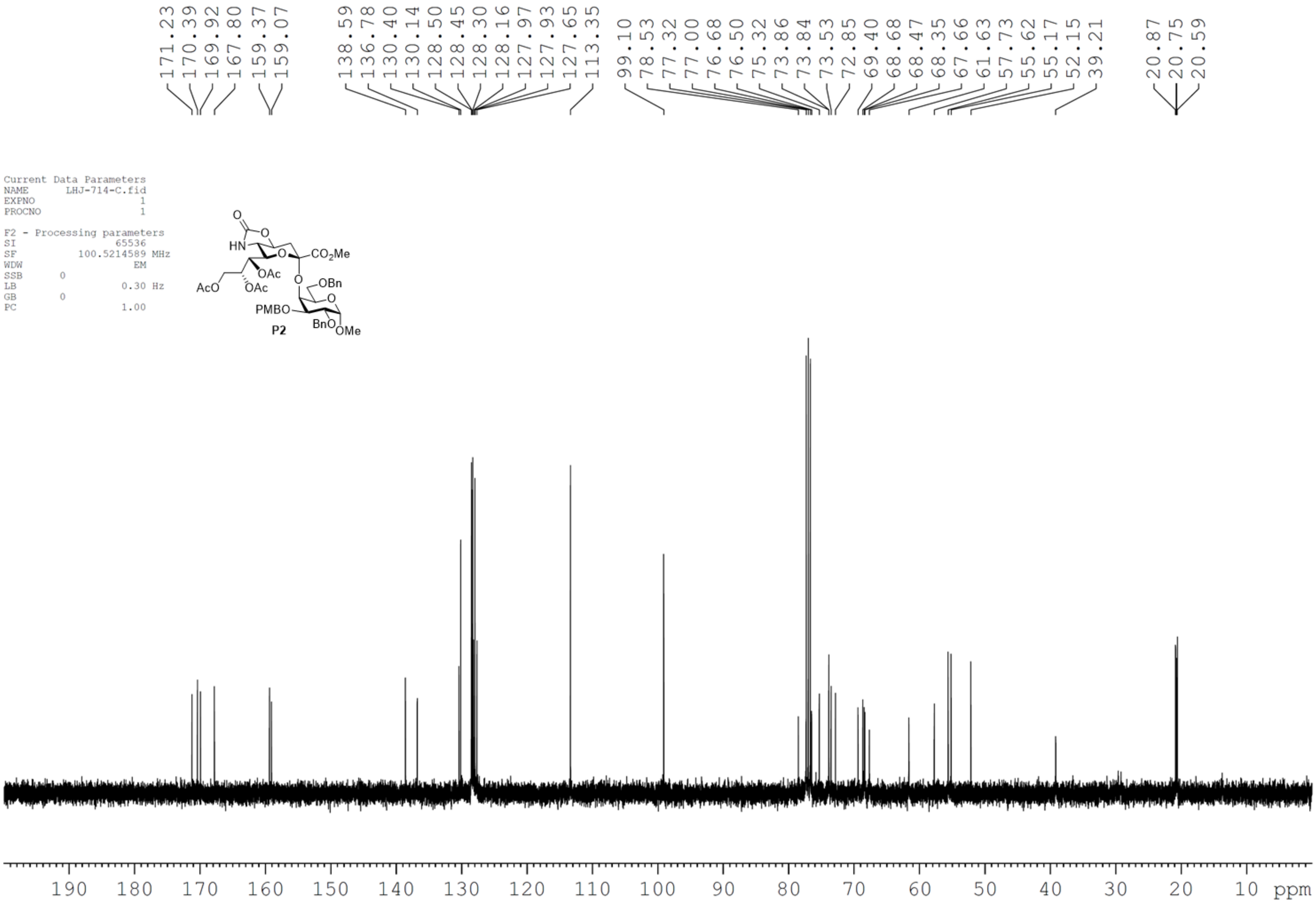

171.23
170.39
169.92
167.80
159.37
159.07
138.59
136.78
130.40
130.14
128.50
128.45
128.30
128.16
127.97
127.93
127.65
113.35
99.10
78.53
77.32
77.00
76.68
76.50
75.32
73.86
73.84
73.53
72.85
69.40
68.68
68.47
68.35
67.66
61.63
57.73
55.62
55.17
52.15
39.21
20.87
20.75
20.59
Current Data Parameters
NAME LHJ-714-C.fid
EXPNO 1
PROCNO 1
F2 - Processing parameters
SI 65536
SF 100.5214589 MHz
WDW EM
SSB 0
LB 0.30 Hz
GB 0
PC 1.00
P2
190 180 170 160 150 140 130 120 110 100 90 80 70 60 50 40 30 20 10 ppm

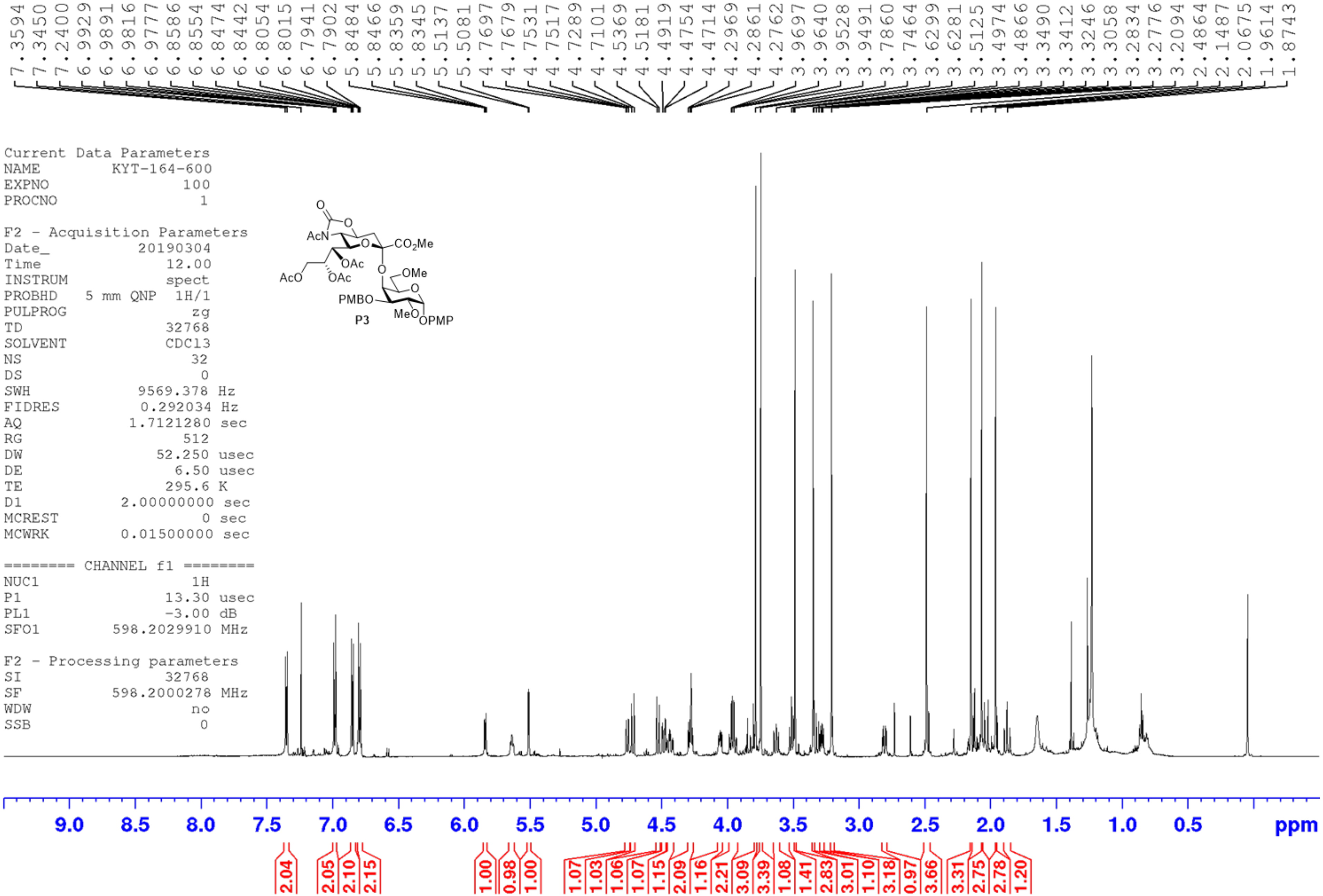
7.3594
7.3450
7.2400
6.9929
6.9891
6.9816
6.9777
6.8586
6.8554
6.8474
6.8442
6.8054
6.8015
6.7941
6.7902
5.8484
5.8466
5.8359
5.8345
5.5137
5.5081
4.7697
4.7679
4.7531
4.7517
4.7289
4.7101
4.5369
4.5181
4.4919
4.4754
4.4714
4.2969
4.2861
4.2762
3.9697
3.9640
3.9528
3.9491
3.7860
3.7464
3.6299
3.6281
3.5125
3.4974
3.4866
3.3490
3.3412
3.3246
3.3058
3.2834
3.2776
3.2094
2.4864
2.1487
2.0675
1.9614
1.8743
Current Data Parameters
NAME KYT-164-600
EXPNO 100
PROCNO 1
F2 - Acquisition Parameters
Date_ 20190304
Time 12.00
INSTRUM spect
PROBHD 5 mm QNP 1H/1
PULPROG zg
TD 32768
SOLVENT CDCl3
NS 32
DS 0
SWH 9569.378 Hz
FIDRES 0.292034 Hz
AQ 1.7121280 sec
RG 512
DW 52.250 usec
DE 6.50 usec
TE 295.6 K
D1 2.00000000 sec
MCREST 0 sec
MCWRK 0.01500000 sec
======== CHANNEL f1 ========
NUC1 1H
P1 13.30 usec
PL1 -3.00 dB
SFO1 598.2029910 MHz
F2 - Processing parameters
SI 32768
SF 598.2000278 MHz
WDW no
SSB 0
O
O
AcN
O
CO2Me
OAc
O
OMe
AcO
OAc
O
PMBO
MeO
OPMP
P3
9.0
8.5
8.0
7.5
7.0
6.5
6.0
5.5
5.0
4.5
4.0
3.5
3.0
2.5
2.0
1.5
1.0
0.5
ppm
2.04
2.05
2.10
2.15
1.00
0.98
1.00
1.07
1.03
1.06
1.07
1.15
2.09
1.16
2.21
3.09
3.39
1.08
1.41
2.83
3.01
1.10
3.18
0.97
3.66
3.31
2.75
2.78
1.20

Current Data Parameters
NAME KYT-164-600
EXPNO 2
PROCNO 1

F2 - Acquisition Parameters
Date_ 20190304
Time 9.50
INSTRUM spect
PROBHD 5 mm QNP 1H/1
PULPROG zgpg
TD 32768
SOLVENT CDCl3
NS 537
DS 0
SWH 45045.047 Hz
FIDRES 1.374666 Hz
AQ 0.3637248 sec
RG 4096
DW 11.100 usec
DE 6.50 usec
TE 296.1 K
D1 3.50000000 sec
d11 0.03000000 sec
DELTA 3.40000010 sec
MCREST 0 sec
MCWRK 0.01500000 sec

======== CHANNEL f1 ========
NUC1 13C
P1 4.80 usec
PL1 0 dB
SFO1 150.4340559 MHz

======== CHANNEL f2 ========
CPDPRG[2 waltz16
NUC2 1H
PCPD2 92.00 usec
PL2 120.00 dB
PL12 -1.00 dB
PL13 5.50 dB
SFO2 598.2029910 MHz

F2 - Processing parameters
SI 65536
SF 150.4175167 MHz
WDW EM
SSB 0
LB 5.00 Hz
GB 0
PC 1.50

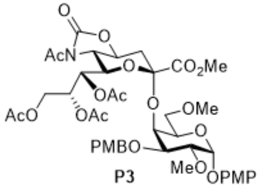

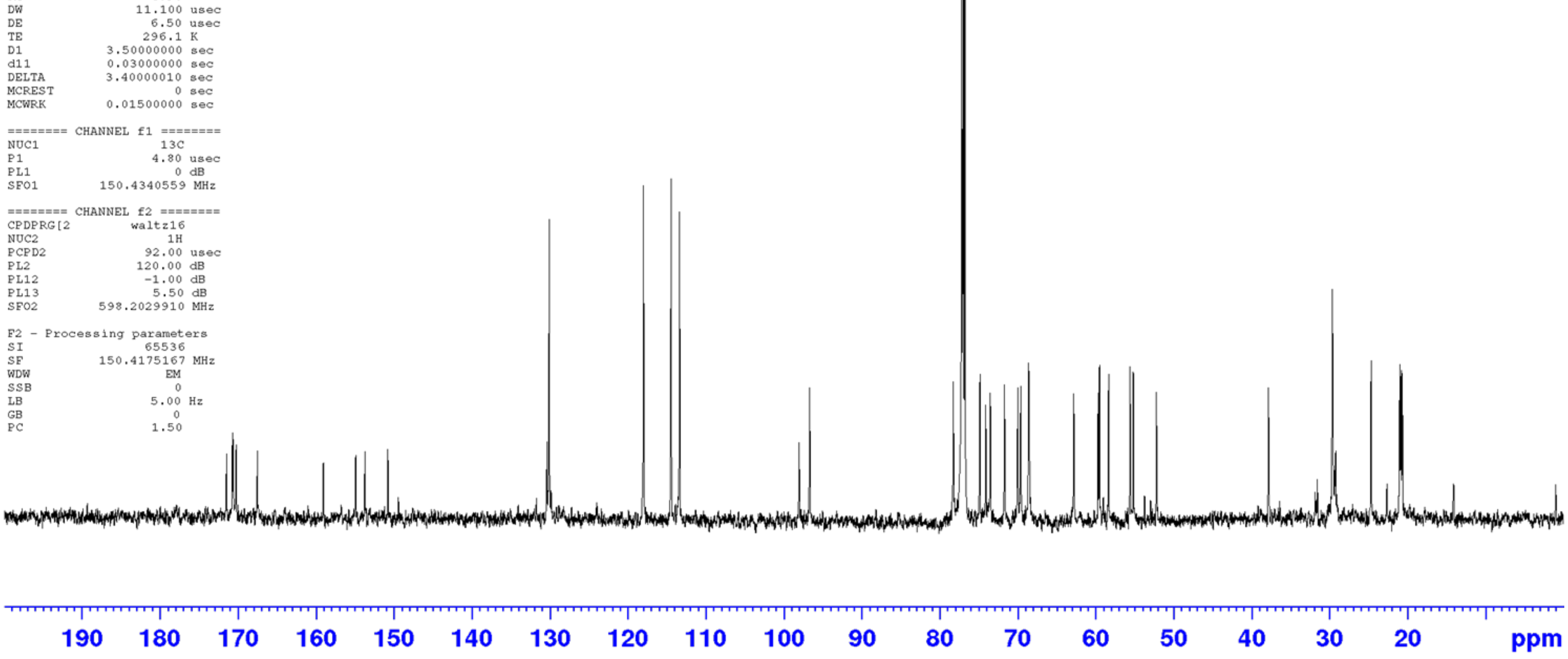

HMBC

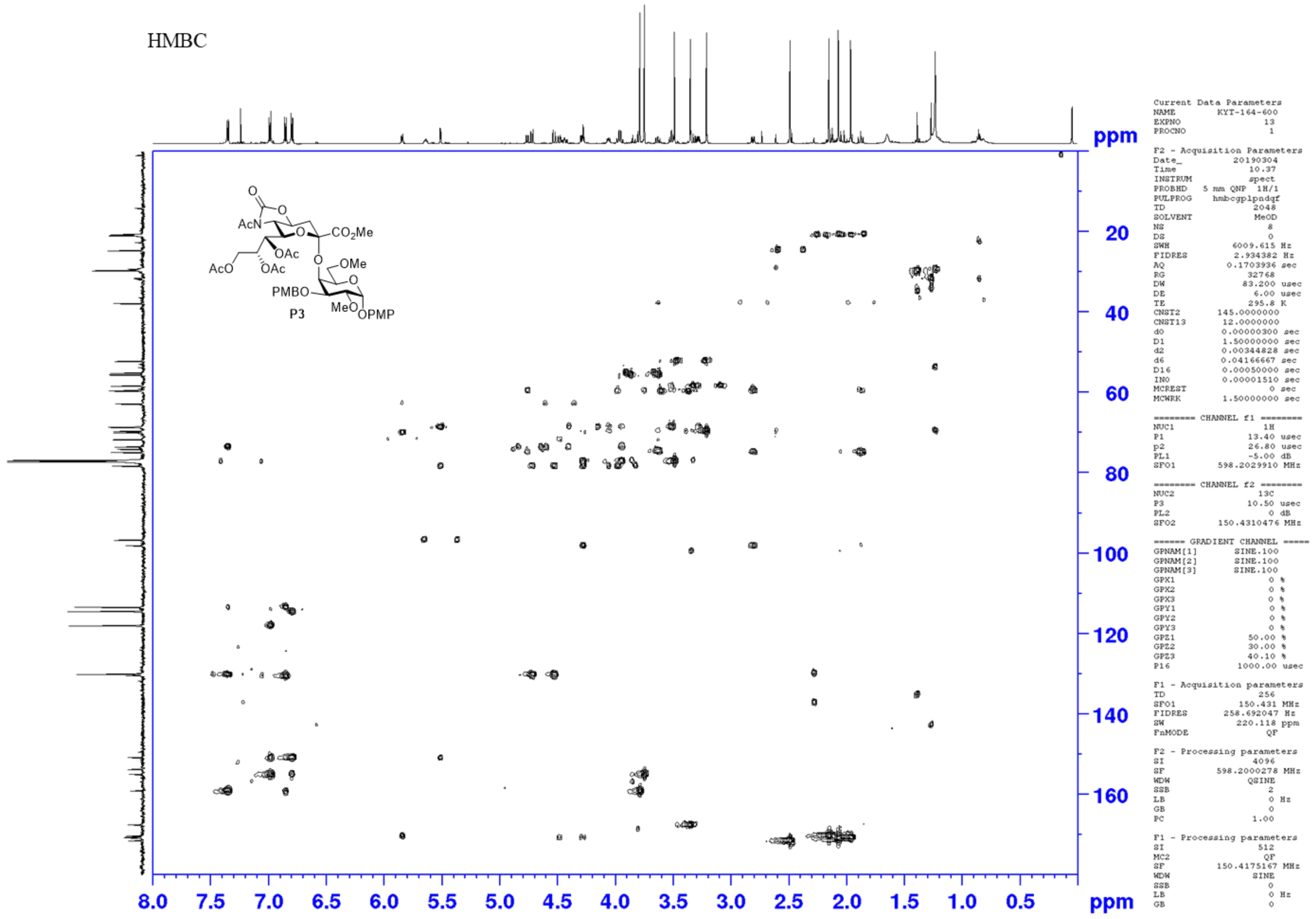
AcN
CO2Me
OAc
AcO
OMe
PMBO
P3
MeO
OPMP
ppm
20
40
60
80
100
120
140
160
8.0
7.5
7.0
6.5
6.0
5.5
5.0
4.5
4.0
3.5
3.0
2.5
2.0
1.5
1.0
0.5
ppm
Current Data Parameters
NAME KYT-164-600
EXPNO 13
PROCNO 1
F2 - Acquisition Parameters
Date_ 20190304
Time 10.37
INSTRUM spect
PROBHD 5 mm QNP 1H/1
PULPROG hmbcgplpndqf
TD 2048
SOLVENT MeOD
NS 8
DS 0
SWH 6009.615 Hz
FIDRES 2.934382 Hz
AQ 0.1703936 sec
RG 32768
DW 83.200 usec
DE 6.00 usec
TE 295.8 K
CNST2 145.0000000
CNST13 12.0000000
d0 0.00000300 sec
D1 1.50000000 sec
d2 0.00344828 sec
d6 0.04166667 sec
D16 0.00050000 sec
IN0 0.00001510 sec
MCREST 0 sec
MCWRK 1.50000000 sec
======== CHANNEL f1 ========
NUC1 1H
P1 13.40 usec
p2 26.80 usec
PL1 -5.00 dB
SFO1 598.2029910 MHz
======== CHANNEL f2 ========
NUC2 13C
P3 10.50 usec
PL2 0 dB
SFO2 150.4310476 MHz
====== GRADIENT CHANNEL =====
GPNAM[1] SINE.100
GPNAM[2] SINE.100
GPNAM[3] SINE.100
GPX1 0 %
GPX2 0 %
GPX3 0 %
GPY1 0 %
GPY2 0 %
GPY3 0 %
GPZ1 50.00 %
GPZ2 30.00 %
GPZ3 40.10 %
P16 1000.00 usec
F1 - Acquisition parameters
TD 256
SFO1 150.431 MHz
FIDRES 258.692047 Hz
SW 220.118 ppm
FnMODE QF
F2 - Processing parameters
SI 4096
SF 598.2000278 MHz
WDW QSINE
SSB 2
LB 0 Hz
GB 0
PC 1.00
F1 - Processing parameters
SI 512
MC2 QF
SF 150.4175167 MHz
WDW SINE
SSB 0
LB 0 Hz
GB 0

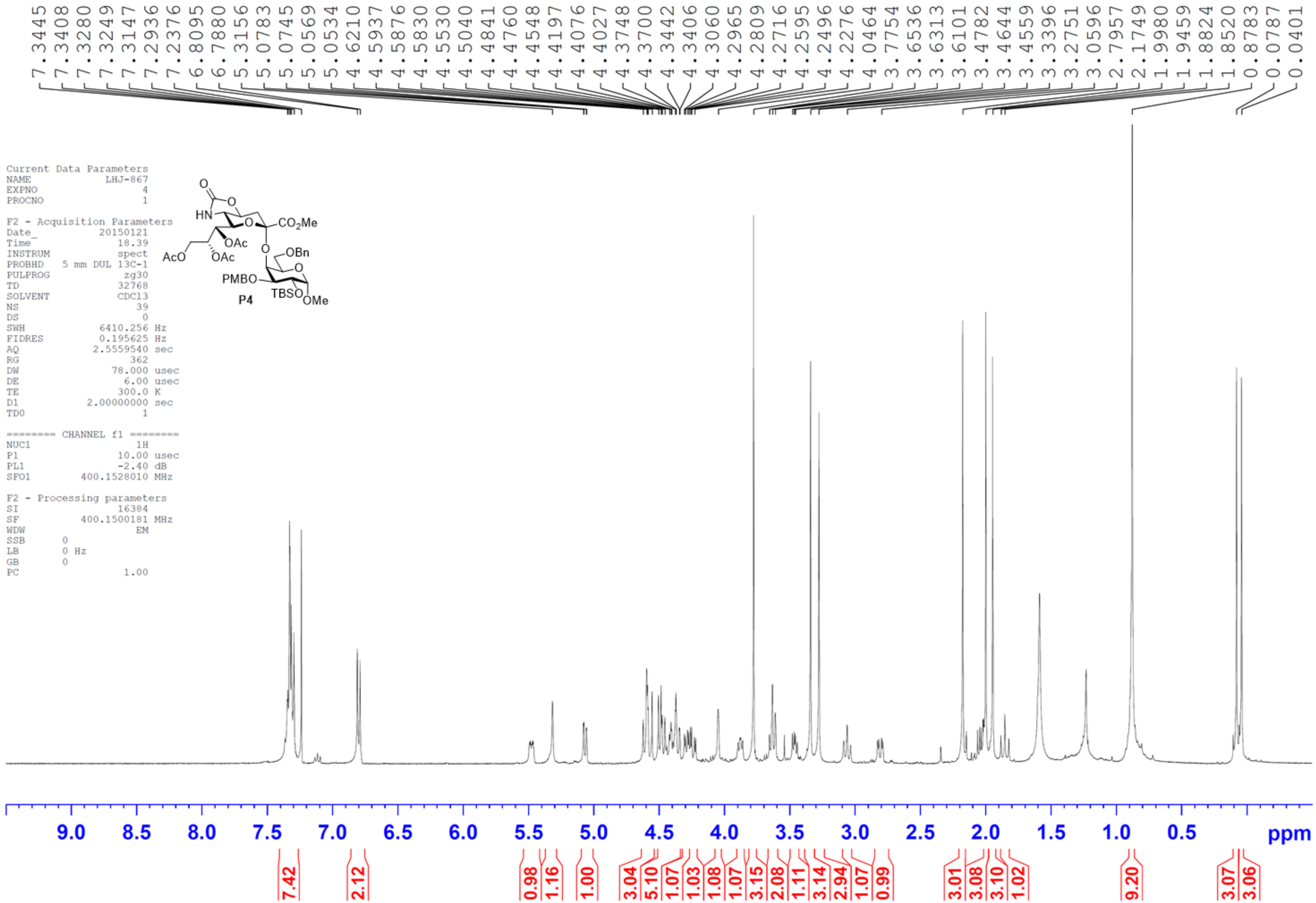

7.3445 7.3408 7.3280 7.3249 7.3147 7.2936 7.2376 6.8095 6.7880 5.3156 5.0783 5.0745 5.0569 5.0534 4.6210 4.5937 4.5876 4.5830 4.5530 4.5040 4.4841 4.4760 4.4548 4.4197 4.4076 4.4027 4.3748 4.3700 4.3442 4.3406 4.3060 4.2965 4.2809 4.2716 4.2595 4.2496 4.2276 4.0464 3.7754 3.6536 3.6313 3.6101 3.4782 3.4644 3.4559 3.3396 3.2751 3.0596 2.7957 2.1749 1.9980 1.9459 1.8824 1.8520 0.8783 0.0787 0.0401
Current Data Parameters
NAME LHJ-867
EXPNO 4
PROCNO 1
F2 - Acquisition Parameters
Date_ 20150121
Time 18.39
INSTRUM spect
PROBHD 5 mm DUL 13C-1
PULPROG zg30
TD 32768
SOLVENT CDCl3
NS 39
DS 0
SWH 6410.256 Hz
FIDRES 0.195625 Hz
AQ 2.5559540 sec
RG 362
DW 78.000 usec
DE 6.00 usec
TE 300.0 K
D1 2.00000000 sec
TD0 1
======== CHANNEL f1 ========
NUC1 1H
P1 10.00 usec
PL1 -2.40 dB
SFO1 400.1528010 MHz
F2 - Processing parameters
SI 16384
SF 400.1500181 MHz
WDW EM
SSB 0
LB 0 Hz
GB 0
PC 1.00
HN
O
CO2Me
OAc
AcO
OAc
OBn
PMBO
TBSO
OMe
P4
9.0 8.5 8.0 7.5 7.0 6.5 6.0 5.5 5.0 4.5 4.0 3.5 3.0 2.5 2.0 1.5 1.0 0.5 ppm
7.42 2.12 0.98 1.16 1.00 3.04 5.10 1.07 1.03 1.08 1.07 3.15 2.08 1.11 3.14 2.94 1.07 0.99 3.01 3.08 3.10 1.02 9.20 3.07 3.06

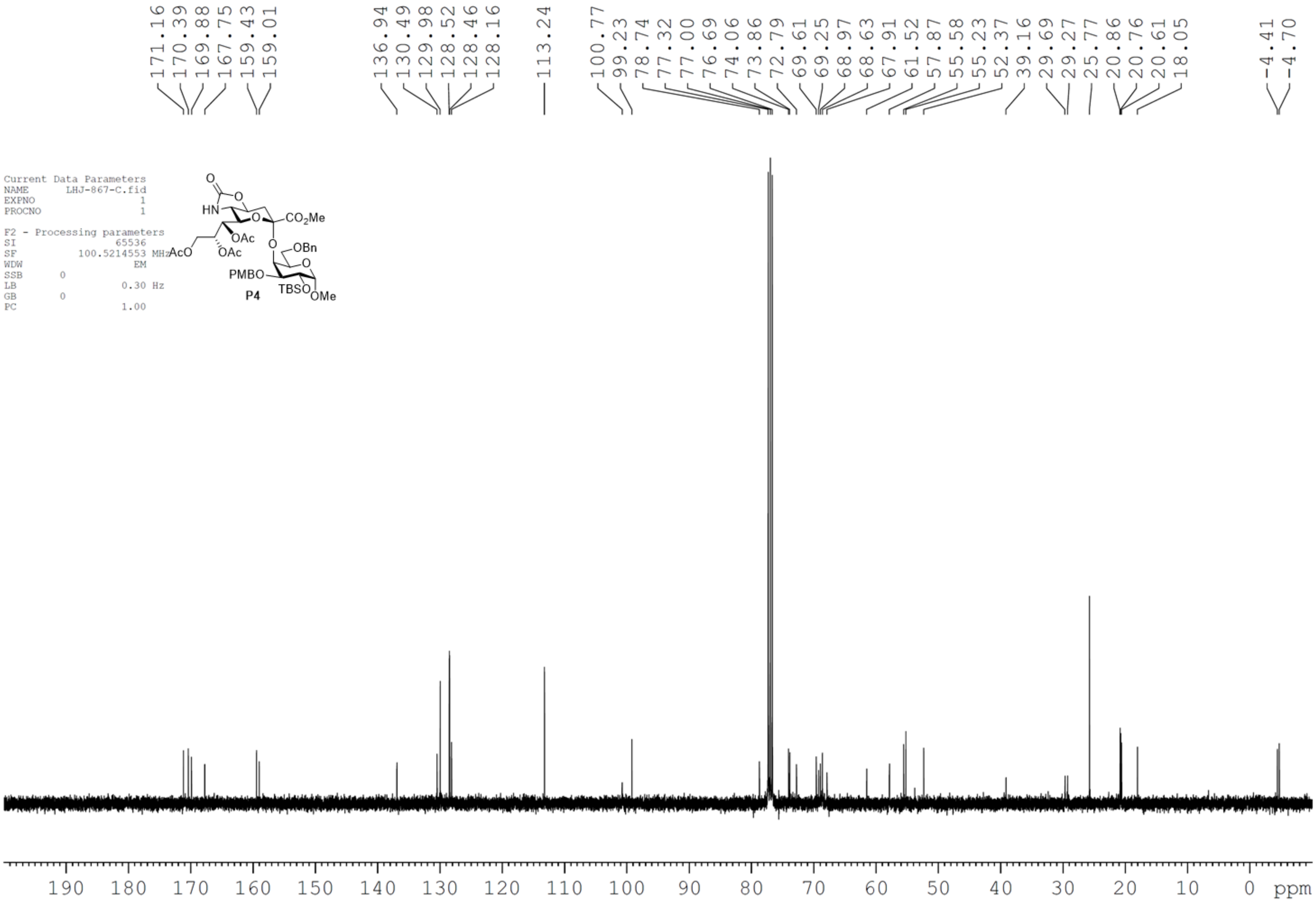
171.16
170.39
169.88
167.75
159.43
159.01
136.94
130.49
129.98
128.52
128.46
128.16
113.24
100.77
99.23
78.74
77.32
77.00
76.69
74.06
73.86
72.79
69.61
69.25
68.97
68.63
67.91
61.52
57.87
55.58
55.23
52.37
39.16
29.69
29.27
25.77
20.86
20.76
20.61
18.05
-4.41
-4.70
Current Data Parameters
NAME LHJ-867-C.fid
EXPNO 1
PROCNO 1
F2 - Processing parameters
SI 65536
SF 100.5214553 MHz
WDW EM
SSB 0
LB 0.30 Hz
GB 0
PC 1.00
HN
CO2Me
OAc
OAc
AcO
OBn
PMBO
TBSO
OMe
P4
190 180 170 160 150 140 130 120 110 100 90 80 70 60 50 40 30 20 10 0 ppm

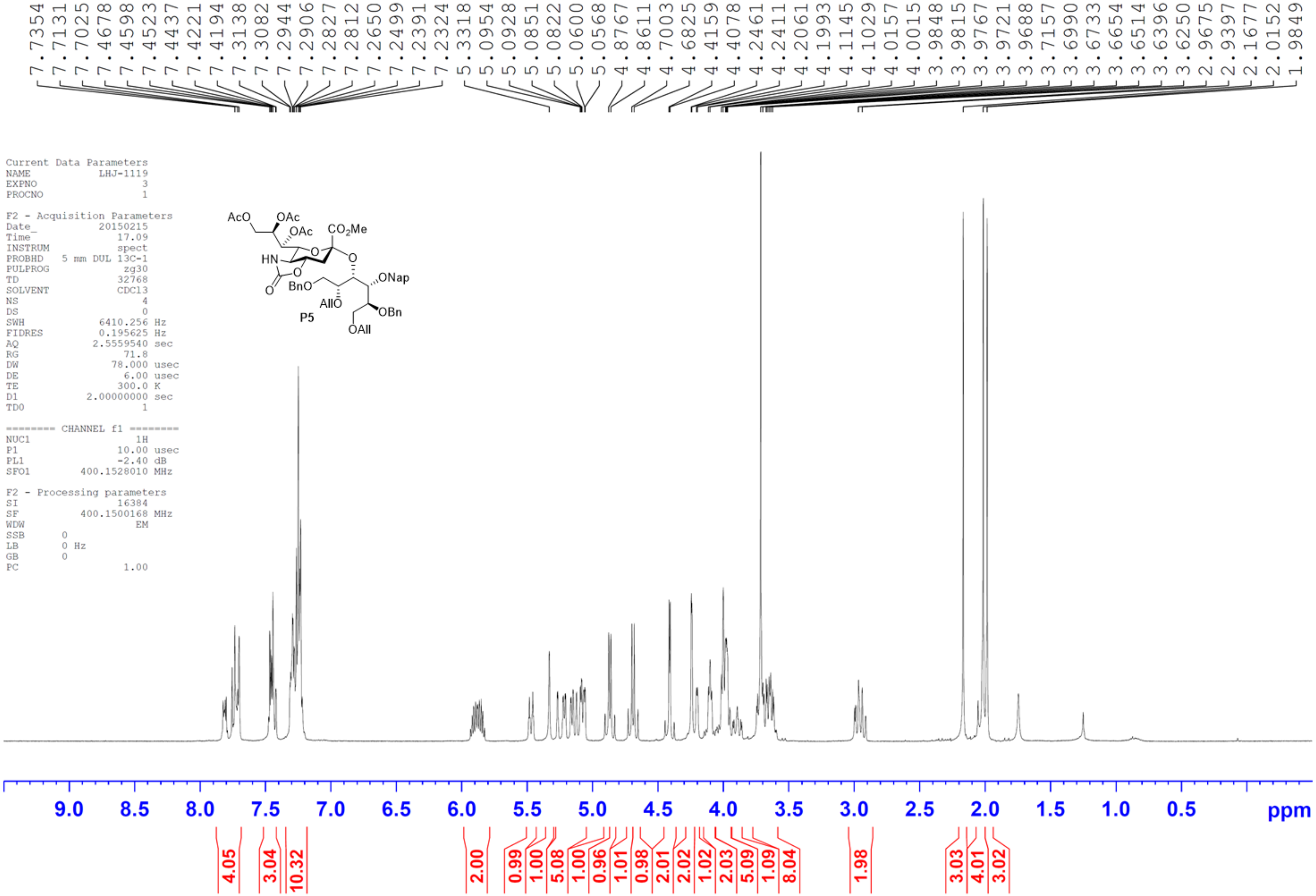
7.7354
7.7131
7.7025
7.4678
7.4598
7.4523
7.4437
7.4221
7.4194
7.3138
7.3082
7.2944
7.2906
7.2827
7.2812
7.2650
7.2499
7.2391
7.2324
5.3318
5.0954
5.0928
5.0851
5.0822
5.0600
5.0568
4.8767
4.8611
4.7003
4.6825
4.4159
4.4078
4.2461
4.2411
4.2061
4.1993
4.1145
4.1029
4.0157
4.0015
3.9848
3.9815
3.9767
3.9721
3.9688
3.7157
3.6990
3.6733
3.6654
3.6514
3.6396
3.6250
2.9675
2.9397
2.1677
2.0152
1.9849
Current Data Parameters
NAME LHJ-1119
EXPNO 3
PROCNO 1
F2 - Acquisition Parameters
Date_ 20150215
Time 17.09
INSTRUM spect
PROBHD 5 mm DUL 13C-1
PULPROG zg30
TD 32768
SOLVENT CDCl3
NS 4
DS 0
SWH 6410.256 Hz
FIDRES 0.195625 Hz
AQ 2.5559540 sec
RG 71.8
DW 78.000 usec
DE 6.00 usec
TE 300.0 K
D1 2.00000000 sec
TD0 1
======== CHANNEL f1 ========
NUC1 1H
P1 10.00 usec
PL1 -2.40 dB
SFO1 400.1528010 MHz
F2 - Processing parameters
SI 16384
SF 400.1500168 MHz
WDW EM
SSB 0
LB 0 Hz
GB 0
PC 1.00
AcO
OAc
OAc
CO2Me
HN
O
O
O
BnO
ONap
AllO
OBn
OAll
P5
9.0
8.5
8.0
7.5
7.0
6.5
6.0
5.5
5.0
4.5
4.0
3.5
3.0
2.5
2.0
1.5
1.0
0.5
ppm
4.05
3.04
10.32
2.00
0.99
1.00
5.08
1.00
0.96
1.01
0.98
2.01
2.02
1.02
2.03
5.09
1.09
8.04
1.98
3.03
4.01
3.02

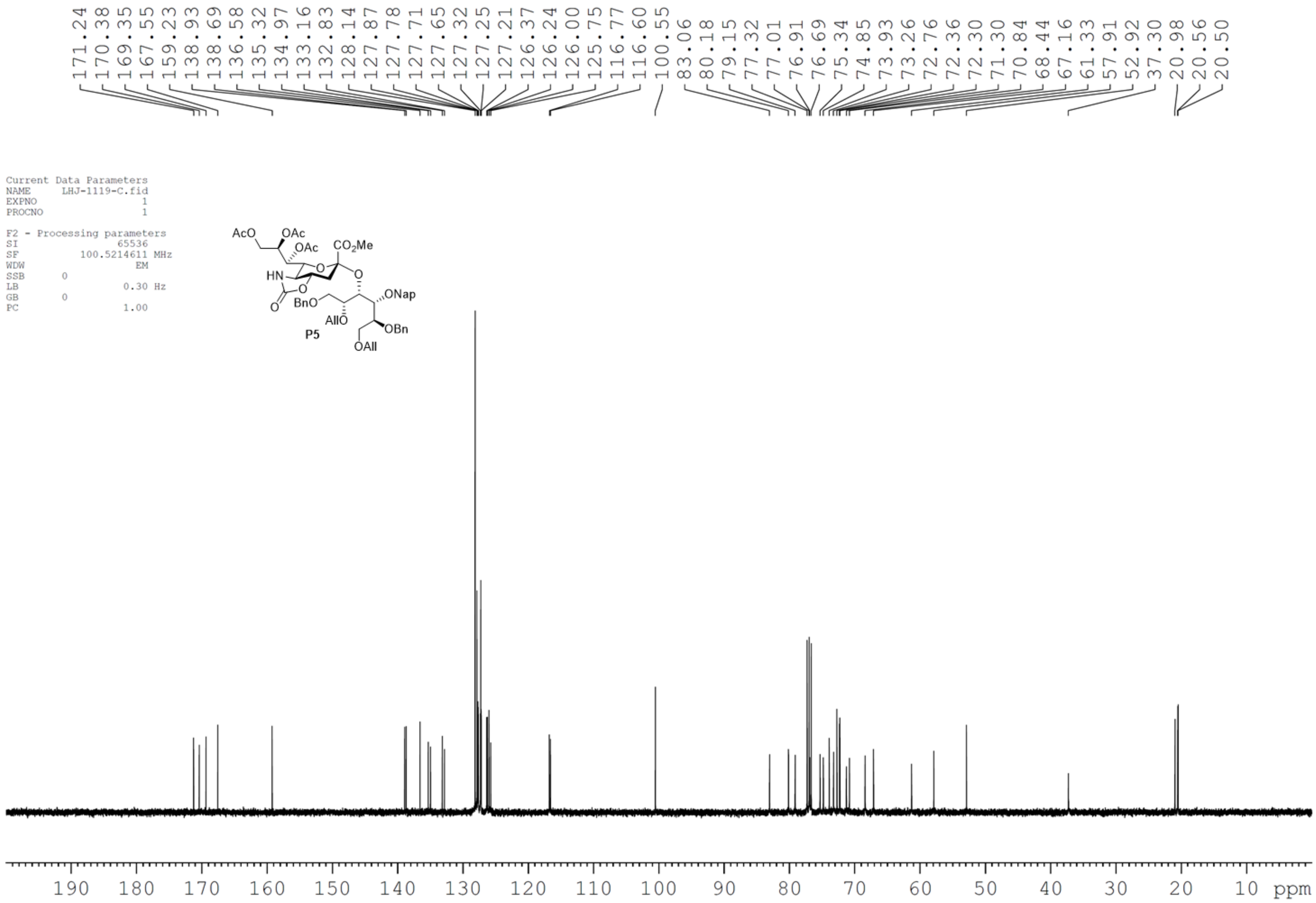
171.24
170.38
169.35
167.55
159.23
138.93
138.69
136.58
135.32
134.97
133.16
132.83
128.14
127.87
127.78
127.71
127.65
127.32
127.25
127.21
126.37
126.24
126.00
125.75
116.77
116.60
100.55
83.06
80.18
79.15
77.32
77.01
76.91
76.69
75.34
74.85
73.93
73.26
72.76
72.36
72.30
71.30
70.84
68.44
67.16
61.33
57.91
52.92
37.30
20.98
20.56
20.50
Current Data Parameters
NAME LHJ-1119-C.fid
EXPNO 1
PROCNO 1
F2 - Processing parameters
SI 65536
SF 100.5214611 MHz
WDW EM
SSB 0
LB 0.30 Hz
GB 0
PC 1.00
AcO
OAc
OAc
CO2Me
HN
O
O
O
BnO
ONap
AllO
OBn
OAll
P5
190
180
170
160
150
140
130
120
110
100
90
80
70
60
50
40
30
20
10 ppm

Current Data Parameters
NAME LHJ-1140
EXPNO 3
PROCNO 1

F2 - Acquisition Parameters
Date_ 20150227
Time_ 16.56
INSTRUM spect
PROBHD 5 mm DUL 13C-1
PULPROG zg30
TD 32768
SOLVENT CDCl3
NS 20
DS 0
SWH 6410.256 Hz
FIDRES 0.195625 Hz
AQ 2.5559540 sec
RG 287
DW 78.000 usec
DE 6.00 usec
TE 300.0 K
D1 2.00000000 sec
TD0 1

======== CHANNEL f1 ========
NUC1 1H
P1 10.00 usec
PL1 -2.40 dB
SFO1 400.1528010 MHz

F2 - Processing parameters
SI 16384
SF 400.1500168 MHz
WDW EM
SSB 0
LB 0 Hz
GB 0
PC 1.00

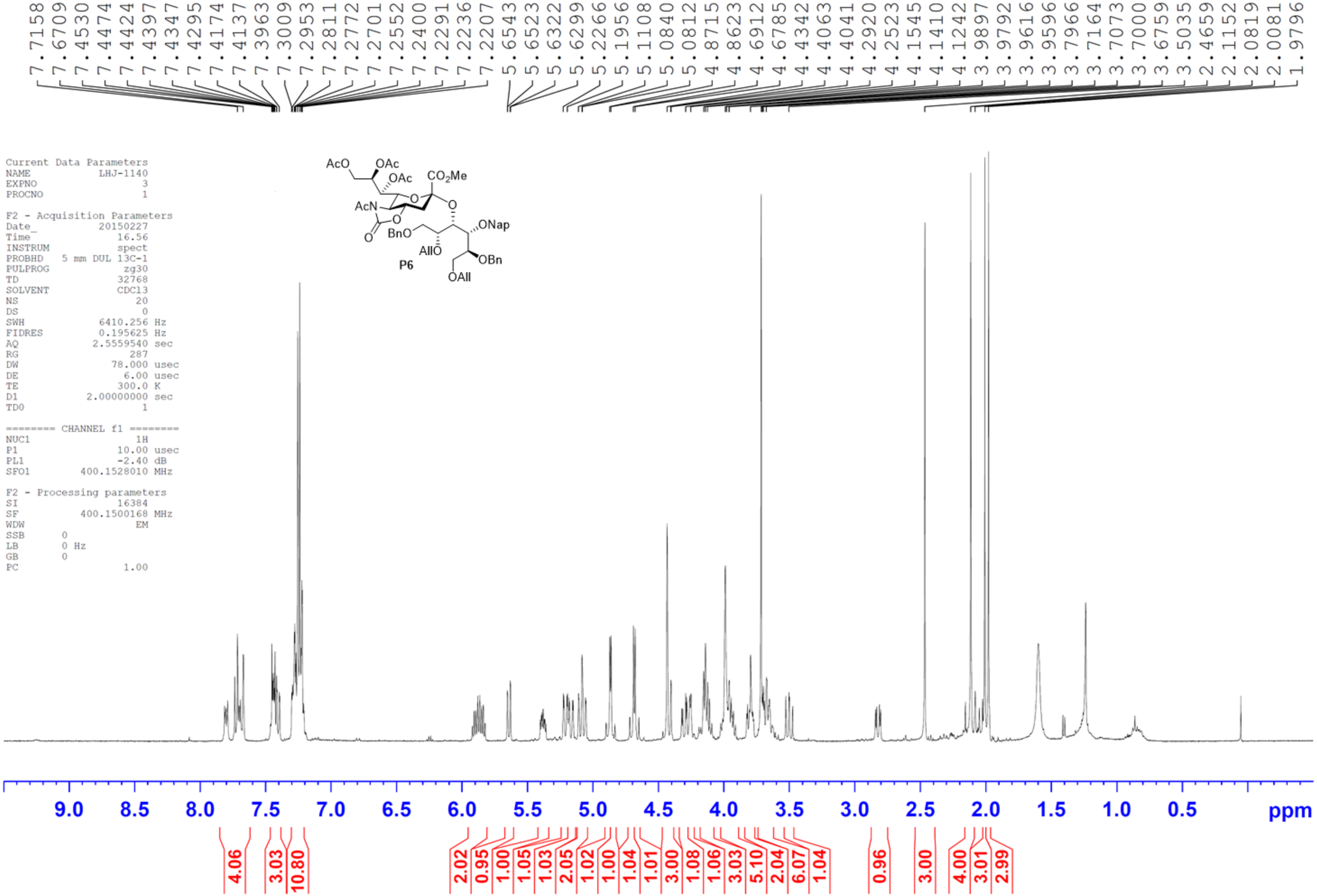

Current Data Parameters
NAME LHJ-1140-C.fid
EXPNO 1
PROCNO 1

F2 - Processing parameters
SI 65536
SF 100.5214578 MHz
WDW EM
SSB 0
LB 0.30 Hz
GB 0
PC 1.00

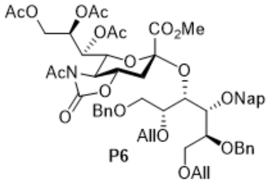

P6

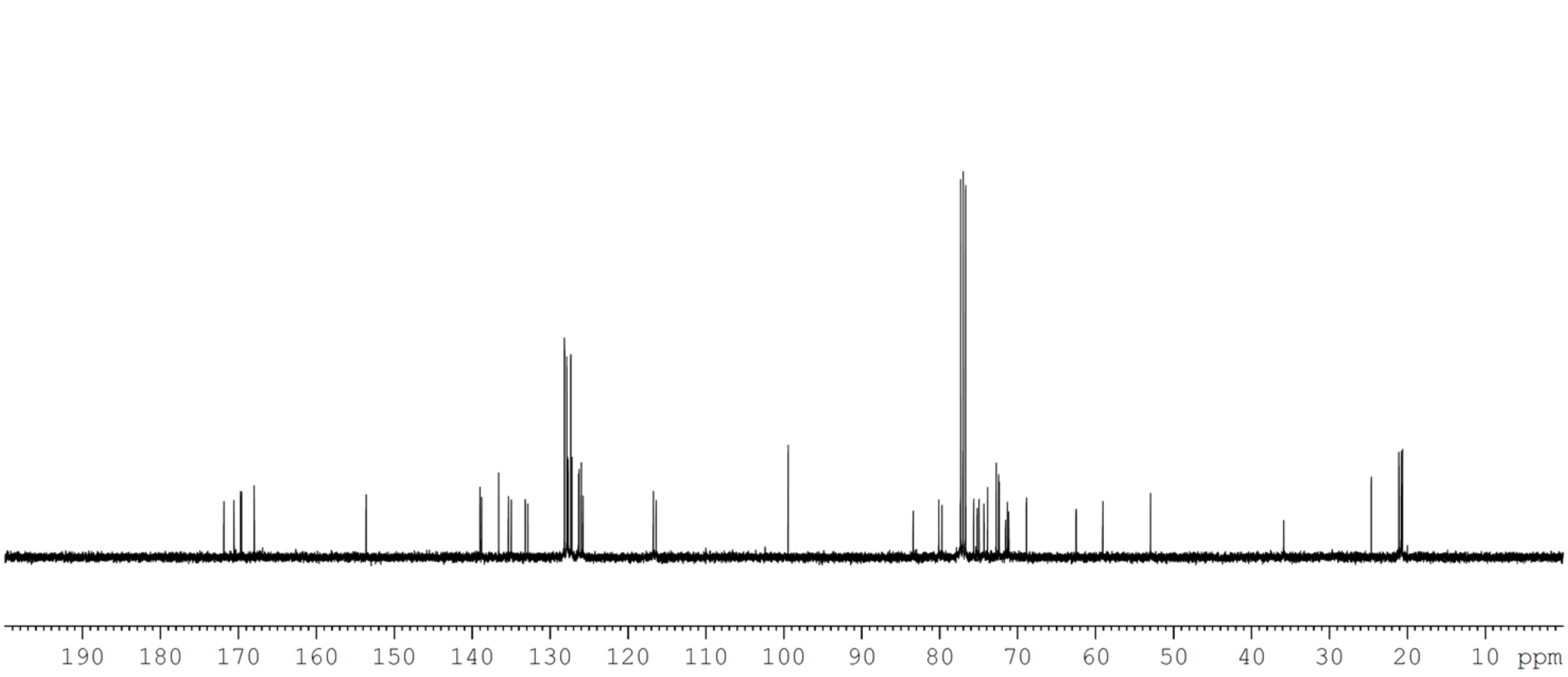

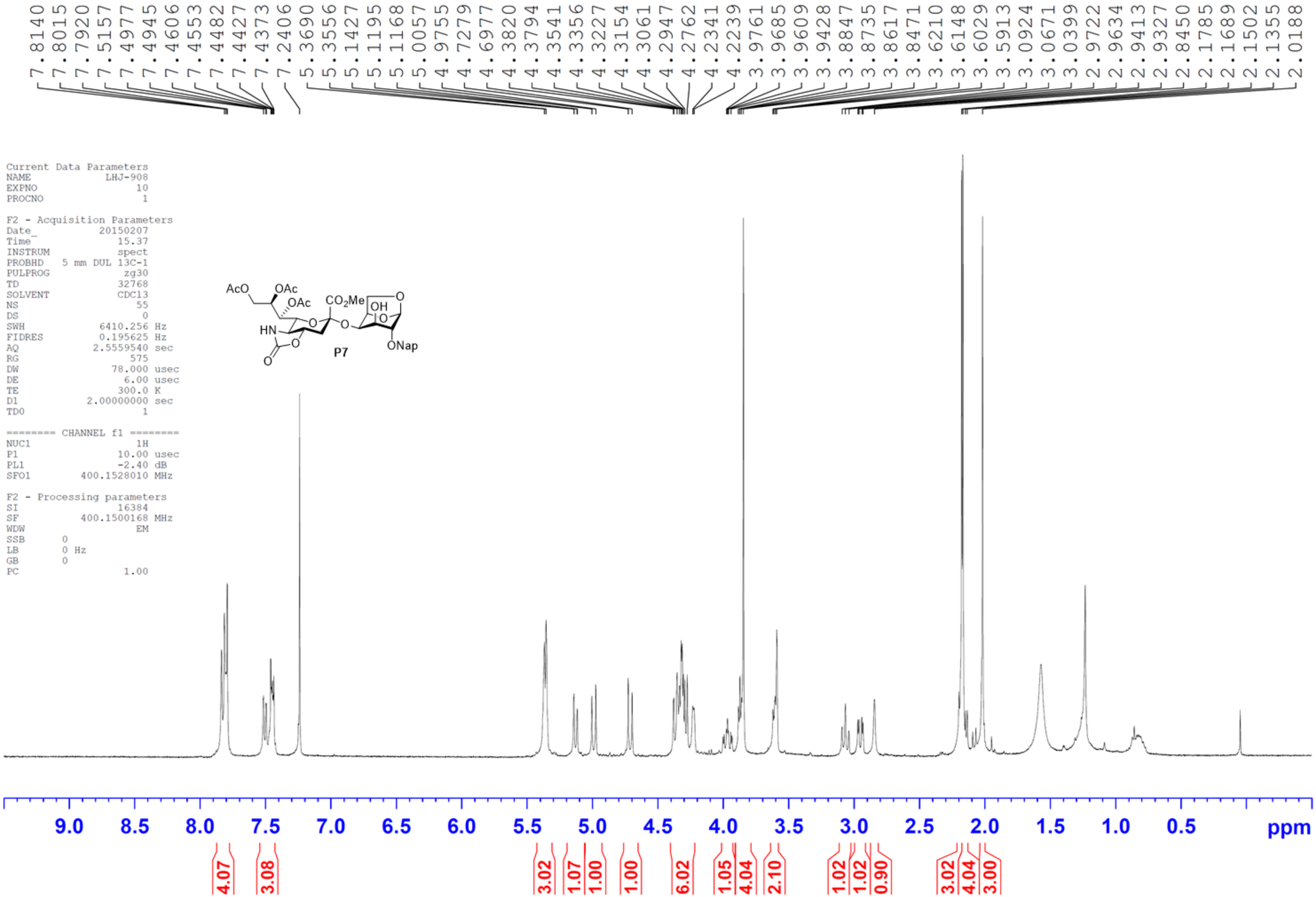
7.8140 7.8015 7.7920 7.5157 7.4977 7.4945 7.4606 7.4553 7.4482 7.4427 7.4373 7.2406 5.3690 5.3556 5.1427 5.1195 5.1168 5.0057 4.9755 4.7279 4.6977 4.3820 4.3794 4.3541 4.3356 4.3227 4.3154 4.3061 4.2947 4.2762 4.2341 4.2239 3.9761 3.9685 3.9609 3.9428 3.8847 3.8735 3.8617 3.8471 3.6210 3.6148 3.6029 3.5913 3.0924 3.0671 3.0399 2.9722 2.9634 2.9413 2.9327 2.8450 2.1785 2.1689 2.1502 2.1355 2.0188
Current Data Parameters
NAME LHJ-908
EXPNO 10
PROCNO 1
F2 - Acquisition Parameters
Date_ 20150207
Time 15.37
INSTRUM spect
PROBHD 5 mm DUL 13C-1
PULPROG zg30
TD 32768
SOLVENT CDCl3
NS 55
DS 0
SWH 6410.256 Hz
FIDRES 0.195625 Hz
AQ 2.5559540 sec
RG 575
DW 78.000 usec
DE 6.00 usec
TE 300.0 K
D1 2.00000000 sec
TD0 1
======== CHANNEL f1 ========
NUC1 1H
P1 10.00 usec
PL1 -2.40 dB
SFO1 400.1528010 MHz
F2 - Processing parameters
SI 16384
SF 400.1500168 MHz
WDW EM
SSB 0
LB 0 Hz
GB 0
PC 1.00
AcO
OAc
OAc
CO2Me
OH
O
HN
O
O
ONap
P7
9.0 8.5 8.0 7.5 7.0 6.5 6.0 5.5 5.0 4.5 4.0 3.5 3.0 2.5 2.0 1.5 1.0 0.5 ppm
4.07 3.08 3.02 1.07 1.00 1.00 6.02 1.05 4.04 2.10 1.02 1.02 0.90 3.02 4.04 3.00

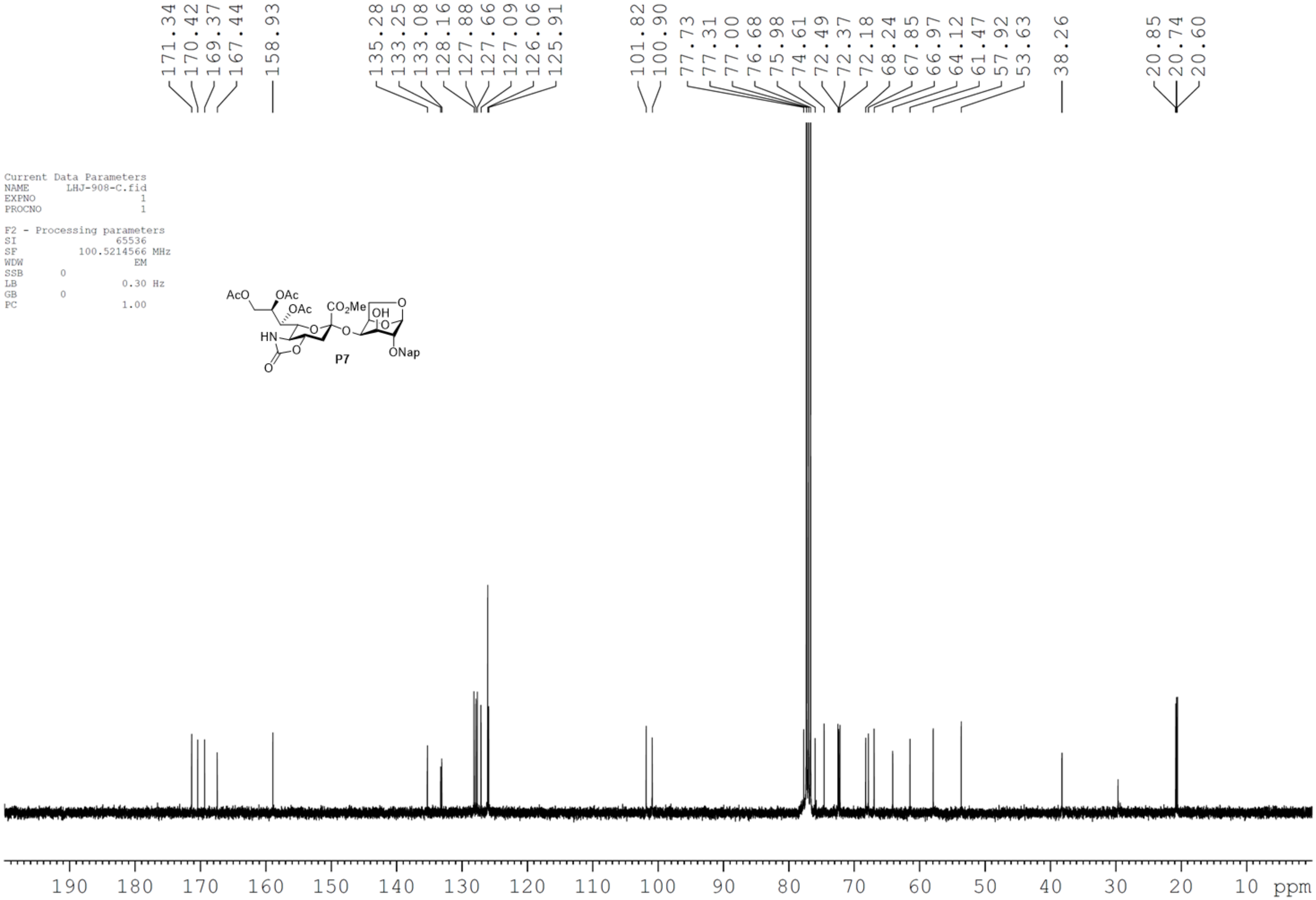
171.34
170.42
169.37
167.44
158.93
135.28
133.25
133.08
128.16
127.88
127.66
127.09
126.06
125.91
101.82
100.90
77.73
77.31
77.00
76.68
75.98
74.61
72.49
72.37
72.18
68.24
67.85
66.97
64.12
61.47
57.92
53.63
38.26
20.85
20.74
20.60
Current Data Parameters
NAME LHJ-908-C.fid
EXPNO 1
PROCNO 1
F2 - Processing parameters
SI 65536
SF 100.5214566 MHz
WDW EM
SSB 0
LB 0.30 Hz
GB 0
PC 1.00
AcO
OAc
OAc
CO_2Me
OH
O
HN
O
O
O
O
O
ONap
P7
190 180 170 160 150 140 130 120 110 100 90 80 70 60 50 40 30 20 10 ppm

Current Data Parameters
NAME KYT-194-new
EXPNO 1
PROCNO 1

F2 - Acquisition Parameters
Date_ 20190703
Time 12.38
INSTRUM spect
PROBHD 5 mm QNP 1H/1
PULPROG zg
TD 32768
SOLVENT H2O+D2O
NS 32
DS 0
SWH 9541.984 Hz
FIDRES 0.291198 Hz
AQ 1.7170432 sec
RG 512
DW 52.400 usec
DE 6.50 usec
TE 299.4 K
D1 2.00000000 sec
MCREST 0 sec
MCWRK 0.01500000 sec

======== CHANNEL f1 ========
NUC1 1H
P1 10.00 usec
PL1 -4.00 dB
SFO1 598.2028119 MHz

F2 - Processing parameters
SI 32768
SF 598.2000270 MHz
WDW no
SSB 0
LB 0 Hz
GB 0
PC 2.00

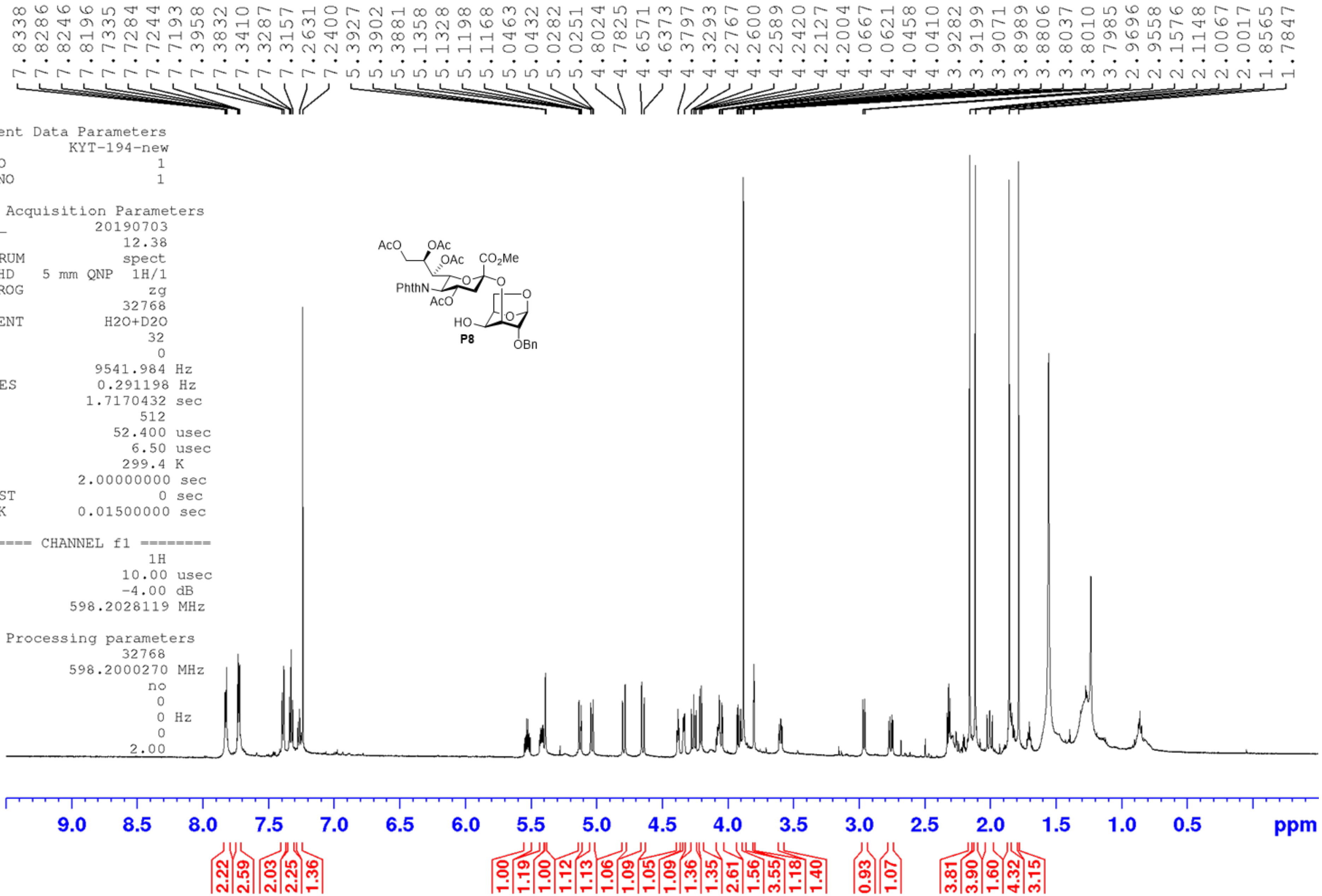

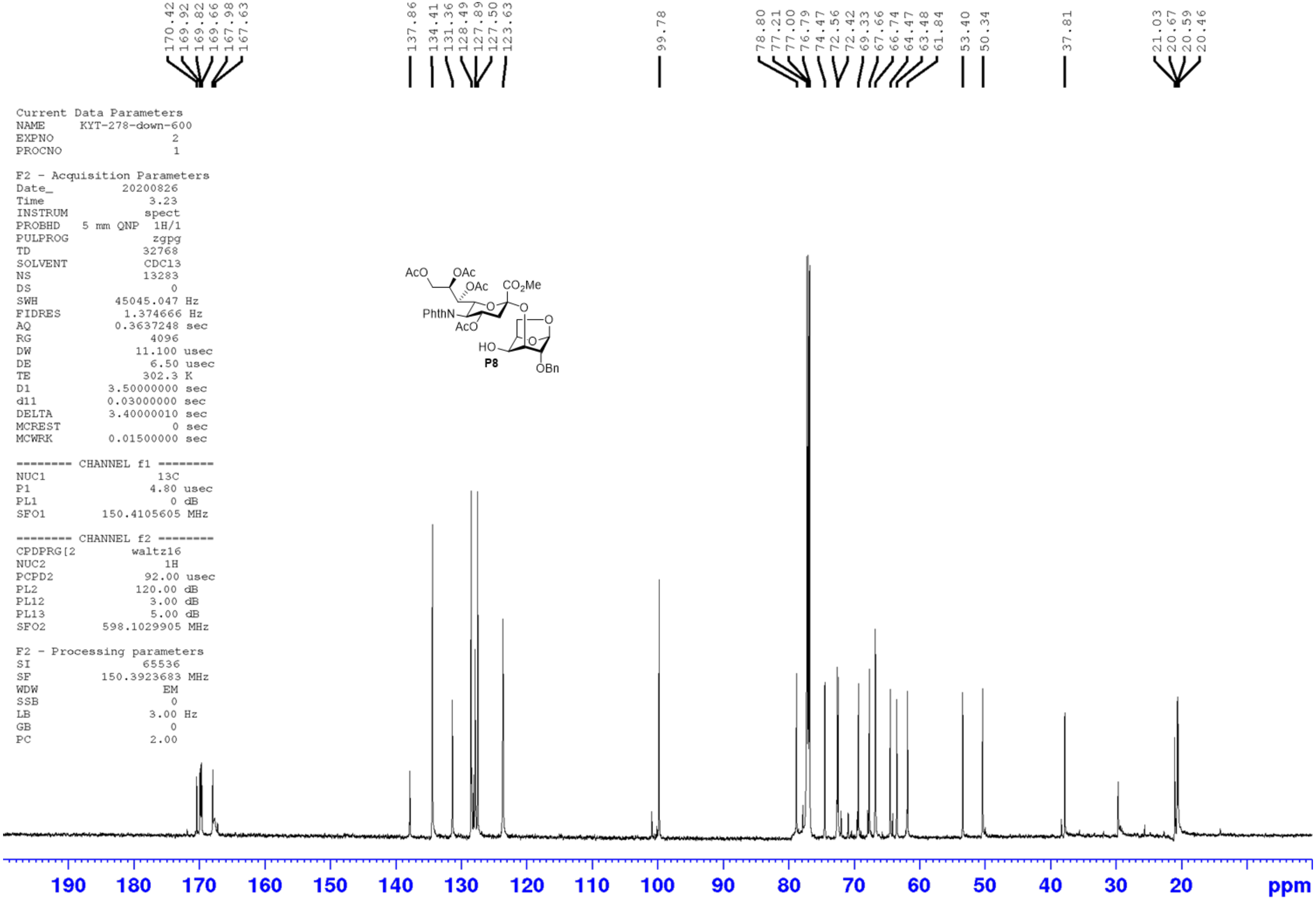
170.42
169.92
169.82
169.66
167.98
167.63
137.86
134.41
131.36
128.49
127.89
127.50
123.63
99.78
78.80
77.21
77.00
76.79
74.47
72.56
72.42
69.33
67.66
66.74
64.47
63.48
61.84
53.40
50.34
37.81
21.03
20.67
20.59
20.46
Current Data Parameters
NAME KYT-278-down-600
EXPNO 2
PROCNO 1
F2 - Acquisition Parameters
Date_ 20200826
Time 3.23
INSTRUM spect
PROBHD 5 mm QNP 1H/1
PULPROG zgpg
TD 32768
SOLVENT CDCl3
NS 13283
DS 0
SWH 45045.047 Hz
FIDRES 1.374666 Hz
AQ 0.3637248 sec
RG 4096
DW 11.100 usec
DE 6.50 usec
TE 302.3 K
D1 3.50000000 sec
d11 0.03000000 sec
DELTA 3.40000010 sec
MCREST 0 sec
MCWRK 0.01500000 sec
======== CHANNEL f1 ========
NUC1 13C
P1 4.80 usec
PL1 0 dB
SFO1 150.4105605 MHz
======== CHANNEL f2 ========
CPDPRG[2 waltz16
NUC2 1H
PCPD2 92.00 usec
PL2 120.00 dB
PL12 3.00 dB
PL13 5.00 dB
SFO2 598.1029905 MHz
F2 - Processing parameters
SI 65536
SF 150.3923683 MHz
WDW EM
SSB 0
LB 3.00 Hz
GB 0
PC 2.00
AcO
OAc
OAc
CO2Me
PhthN
AcO
HO
P8
OBn
190 180 170 160 150 140 130 120 110 100 90 80 70 60 50 40 30 20 ppm

COSY

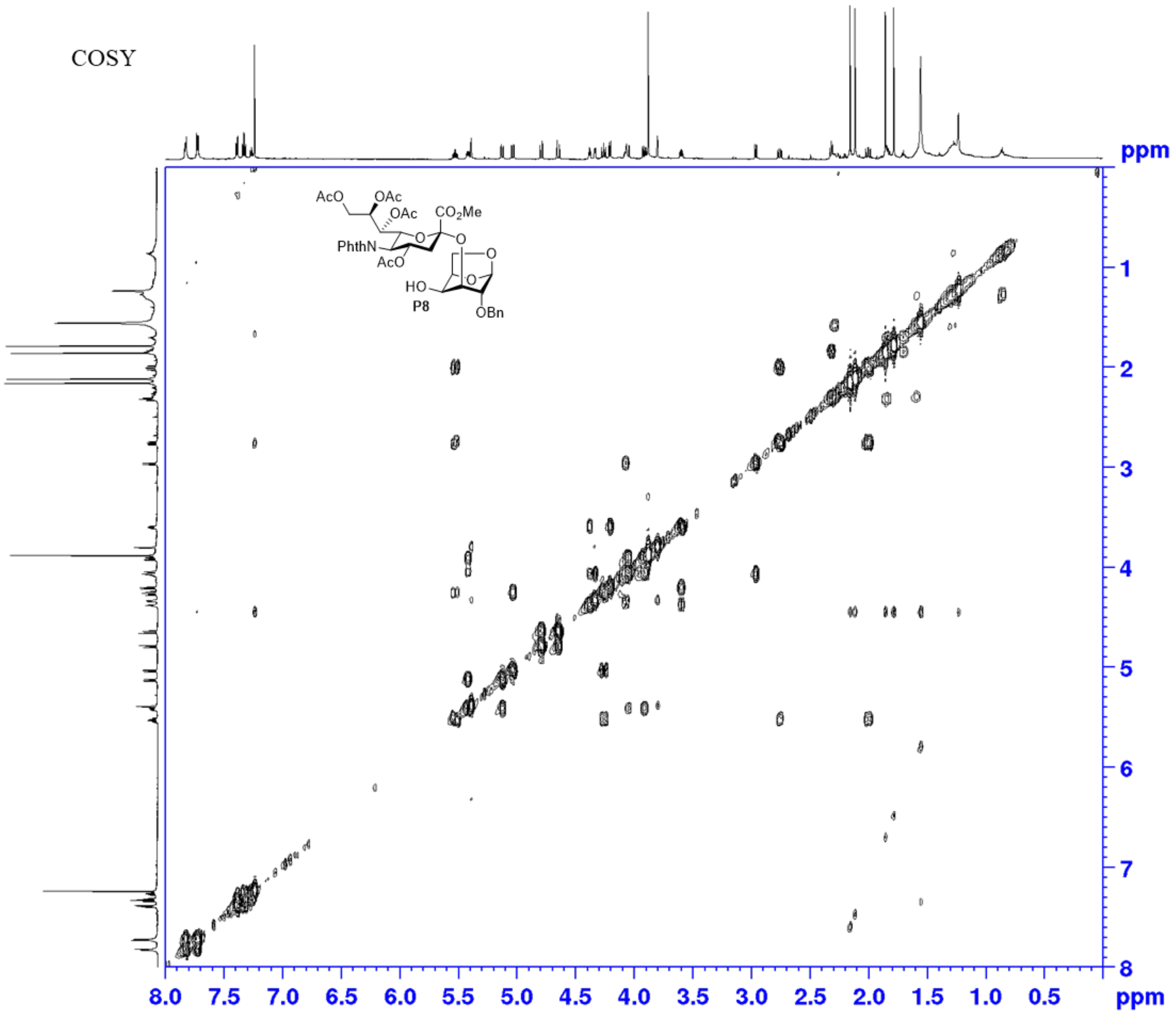


```
Current Data Parameters
NAME          KYT-194-new
EXPNO                  11
PROCNO                  1

F2 - Acquisition Parameters
Date_          20190703
Time              12.56
INSTRUM           spect
PROBHD   5 mm QNP  1H/1
PULPROG          cosyqf
TD                 2048
SOLVENT             MeOD
NS                   16
DS                    0
SWH            6009.615 Hz
FIDRES         2.934382 Hz
AQ            0.1703936 sec
RG                  256
DW               83.200 usec
DE                 6.50 usec
TE                298.9 K
d0           0.00000300 sec
D1           1.50000000 sec
IN0          0.00016640 sec
MCREST                0 sec
MCWRK        1.50000000 sec

======== CHANNEL f1 ========
NUC1                 1H
P0                14.00 usec
P1                10.20 usec
PL1               -1.00 dB
SFO1        598.2026919 MHz

F1 - Acquisition parameters
TD                  256
SFO1           598.2027 MHz
FIDRES        46.950119 Hz
SW               10.046 ppm
FnMODE               QF

F2 - Processing parameters
SI                 2048
SF          598.2000270 MHz
WDW               QSINE
SSB                   0
LB                    0 Hz
GB                    0
PC                 1.00

F1 - Processing parameters
SI                  512
MC2                  QF
SF          598.2000270 MHz
WDW               QSINE
SSB                   0
LB                    0 Hz
GB                    0
```

HSQC

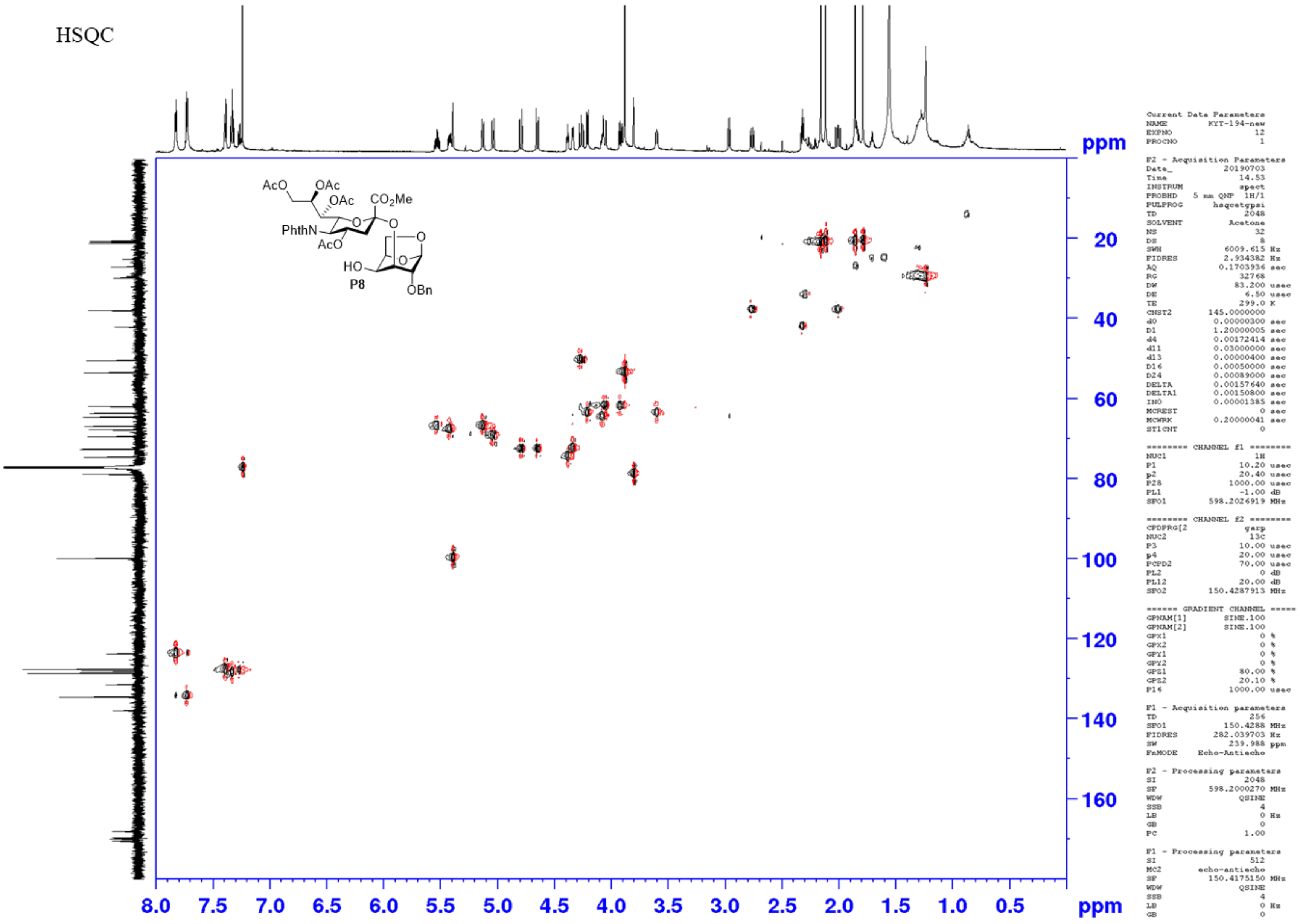

AcO
OAc
OAc
CO2Me
PhthN
AcO
O
O
O
O
HO
P8
OBn
ppm
20
40
60
80
100
120
140
160
ppm
8.0
7.5
7.0
6.5
6.0
5.5
5.0
4.5
4.0
3.5
3.0
2.5
2.0
1.5
1.0
0.5
Current Data Parameters
NAME KYT-194-new
EXPNO 12
PROCNO 1
F2 - Acquisition Parameters
Date_ 20190703
Time 14.53
INSTRUM spect
PROBHD 5 mm QNP 1H/1
PULPROG hsqcetgpsi
TD 2048
SOLVENT Acetone
NS 32
DS 8
SWH 6009.615 Hz
FIDRES 2.934382 Hz
AQ 0.1703936 sec
RG 32768
DW 83.200 usec
DE 6.50 usec
TE 299.0 K
CNST2 145.0000000
d0 0.00000300 sec
D1 1.20000005 sec
d4 0.00172414 sec
d11 0.03000000 sec
d13 0.00000400 sec
D16 0.00050000 sec
D24 0.00089000 sec
DELTA 0.00157640 sec
DELTA1 0.00150800 sec
IN0 0.00001385 sec
MCREST 0 sec
MCWRK 0.20000041 sec
ST1CNT 0
======== CHANNEL f1 ========
NUC1 1H
P1 10.20 usec
p2 20.40 usec
P28 1000.00 usec
PL1 -1.00 dB
SFO1 598.2026919 MHz
======== CHANNEL f2 ========
CPDPRG[2 garp
NUC2 13C
P3 10.00 usec
p4 20.00 usec
PCPD2 70.00 usec
PL2 0 dB
PL12 20.00 dB
SFO2 150.4287913 MHz
====== GRADIENT CHANNEL =====
GPNAM[1] SINE.100
GPNAM[2] SINE.100
GPX1 0 %
GPX2 0 %
GPY1 0 %
GPY2 0 %
GPZ1 80.00 %
GPZ2 20.10 %
P16 1000.00 usec
F1 - Acquisition parameters
TD 256
SFO1 150.4288 MHz
FIDRES 282.039703 Hz
SW 239.988 ppm
FnMODE Echo-Antiecho
F2 - Processing parameters
SI 2048
SF 598.2000270 MHz
WDW QSINE
SSB 4
LB 0 Hz
GB 0
PC 1.00
F1 - Processing parameters
SI 512
MC2 echo-antiecho
SF 150.4175150 MHz
WDW QSINE
SSB 4
LB 0 Hz
GB 0

HMBC

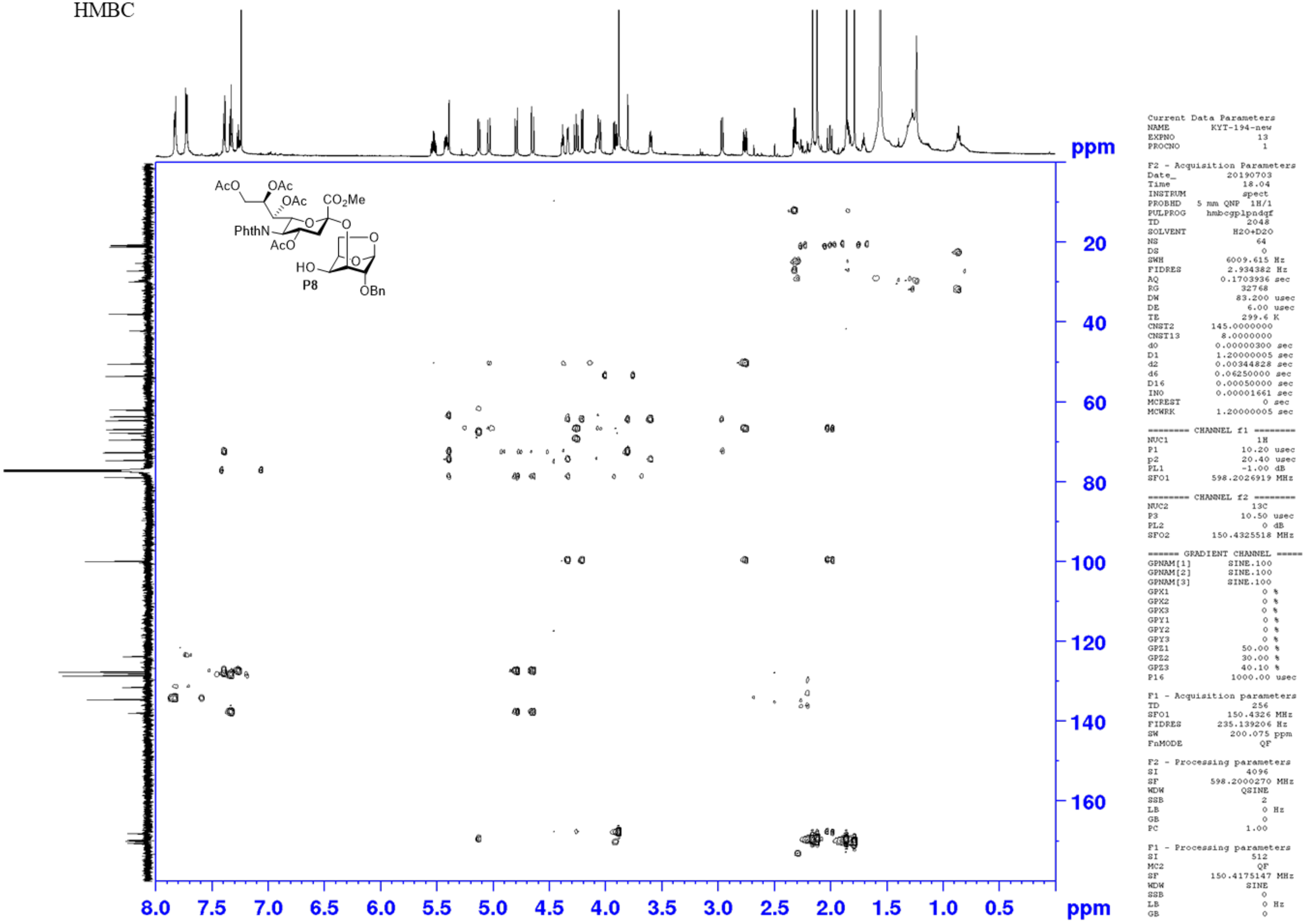

AcO
OAc
OAc
CO2Me
PhthN
AcO
O
HO
P8
OBn
ppm
20
40
60
80
100
120
140
160
8.0
7.5
7.0
6.5
6.0
5.5
5.0
4.5
4.0
3.5
3.0
2.5
2.0
1.5
1.0
0.5
ppm
Current Data Parameters
NAME KYT-194-new
EXPNO 13
PROCNO 1
F2 - Acquisition Parameters
Date_ 20190703
Time 18.04
INSTRUM spect
PROBHD 5 mm QNP 1H/1
PULPROG hmbcgplpndqf
TD 2048
SOLVENT H2O+D2O
NS 64
DS 0
SWH 6009.615 Hz
FIDRES 2.934382 Hz
AQ 0.1703936 sec
RG 32768
DW 83.200 usec
DE 6.00 usec
TE 299.6 K
CNST2 145.0000000
CNST13 8.0000000
d0 0.00000300 sec
D1 1.20000005 sec
d2 0.00344828 sec
d6 0.06250000 sec
D16 0.00050000 sec
IN0 0.00001661 sec
MCREST 0 sec
MCWRK 1.20000005 sec
======== CHANNEL f1 ========
NUC1 1H
P1 10.20 usec
p2 20.40 usec
PL1 -1.00 dB
SFO1 598.2026919 MHz
======== CHANNEL f2 ========
NUC2 13C
P3 10.50 usec
PL2 0 dB
SFO2 150.4325518 MHz
====== GRADIENT CHANNEL =====
GPNAM[1] SINE.100
GPNAM[2] SINE.100
GPNAM[3] SINE.100
GPX1 0 %
GPX2 0 %
GPX3 0 %
GPY1 0 %
GPY2 0 %
GPY3 0 %
GPZ1 50.00 %
GPZ2 30.00 %
GPZ3 40.10 %
P16 1000.00 usec
F1 - Acquisition parameters
TD 256
SFO1 150.4326 MHz
FIDRES 235.139206 Hz
SW 200.075 ppm
FnMODE QF
F2 - Processing parameters
SI 4096
SF 598.2000270 MHz
WDW QSINE
SSB 2
LB 0 Hz
GB 0
PC 1.00
F1 - Processing parameters
SI 512
MC2 QF
SF 150.4175147 MHz
WDW SINE
SSB 0
LB 0 Hz
GB 0

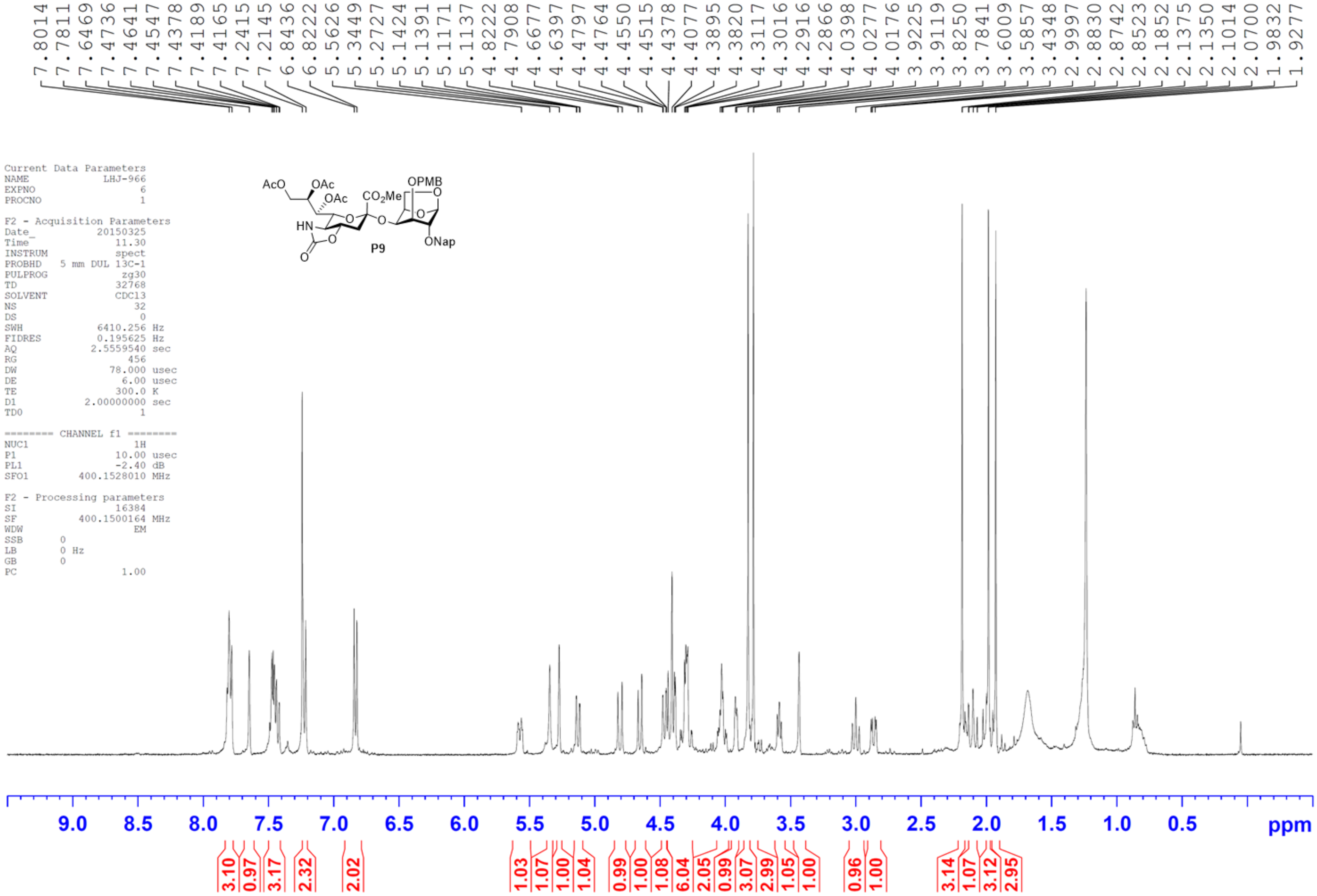

7.8014
7.7811
7.6469
7.4736
7.4641
7.4547
7.4378
7.4189
7.4165
7.2415
7.2145
6.8436
6.8222
5.5626
5.3449
5.2727
5.1424
5.1391
5.1171
5.1137
4.8222
4.7908
4.6677
4.6397
4.4797
4.4764
4.4550
4.4515
4.4378
4.4077
4.3895
4.3820
4.3117
4.3016
4.2916
4.2866
4.0398
4.0277
4.0176
3.9225
3.9119
3.8250
3.7841
3.6009
3.5857
3.4348
2.9997
2.8830
2.8742
2.8523
2.1852
2.1375
2.1350
2.1014
2.0700
1.9832
1.9277
Current Data Parameters
NAME LHJ-966
EXPNO 6
PROCNO 1
F2 - Acquisition Parameters
Date_ 20150325
Time 11.30
INSTRUM spect
PROBHD 5 mm DUL 13C-1
PULPROG zg30
TD 32768
SOLVENT CDCl3
NS 32
DS 0
SWH 6410.256 Hz
FIDRES 0.195625 Hz
AQ 2.5559540 sec
RG 456
DW 78.000 usec
DE 6.00 usec
TE 300.0 K
D1 2.00000000 sec
TD0 1
======== CHANNEL f1 ========
NUC1 1H
P1 10.00 usec
PL1 -2.40 dB
SFO1 400.1528010 MHz
F2 - Processing parameters
SI 16384
SF 400.1500164 MHz
WDW EM
SSB 0
LB 0 Hz
GB 0
PC 1.00
AcO
OAc
OAc
CO2Me
OPMB
HN
O
ONap
P9
9.0
8.5
8.0
7.5
7.0
6.5
6.0
5.5
5.0
4.5
4.0
3.5
3.0
2.5
2.0
1.5
1.0
0.5
ppm
3.10
0.97
3.17
2.32
2.02
1.03
1.07
1.00
1.04
0.99
1.00
1.08
6.04
2.05
0.99
3.07
2.99
1.05
1.00
0.96
1.00
3.14
1.07
3.12
2.95

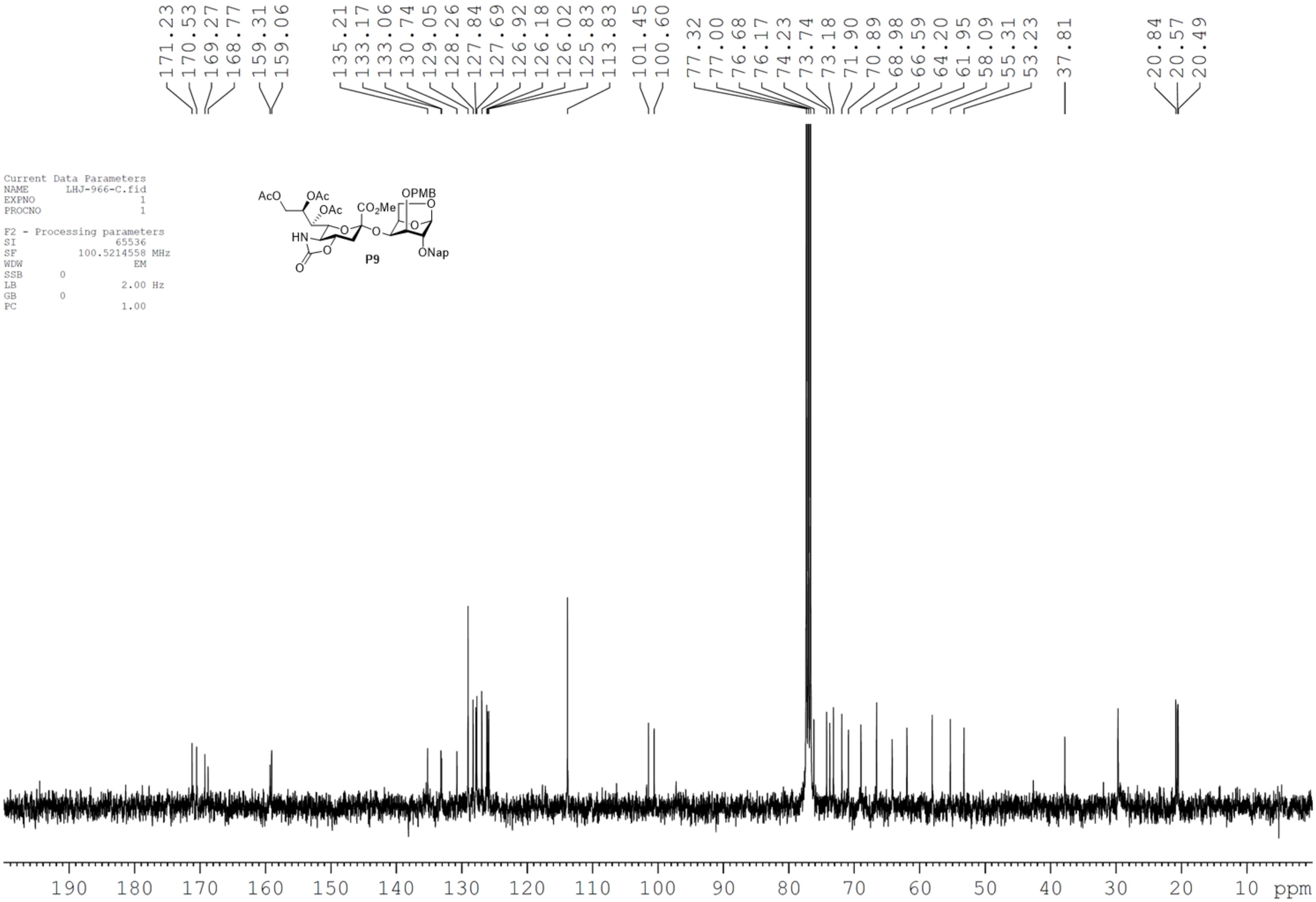
171.23
170.53
169.27
168.77
159.31
159.06
135.21
133.17
133.06
130.74
129.05
128.26
127.84
127.69
126.92
126.18
126.02
125.83
113.83
101.45
100.60
77.32
77.00
76.68
76.17
74.23
73.74
73.18
71.90
70.89
68.98
66.59
64.20
61.95
58.09
55.31
53.23
37.81
20.84
20.57
20.49
Current Data Parameters
NAME LHJ-966-C.fid
EXPNO 1
PROCNO 1
F2 - Processing parameters
SI 65536
SF 100.5214558 MHz
WDW EM
SSB 0
LB 2.00 Hz
GB 0
PC 1.00
AcO
OAc
OAc
CO2Me
OPMB
HN
O
O
O
O
O
ONap
P9
190 180 170 160 150 140 130 120 110 100 90 80 70 60 50 40 30 20 10 ppm

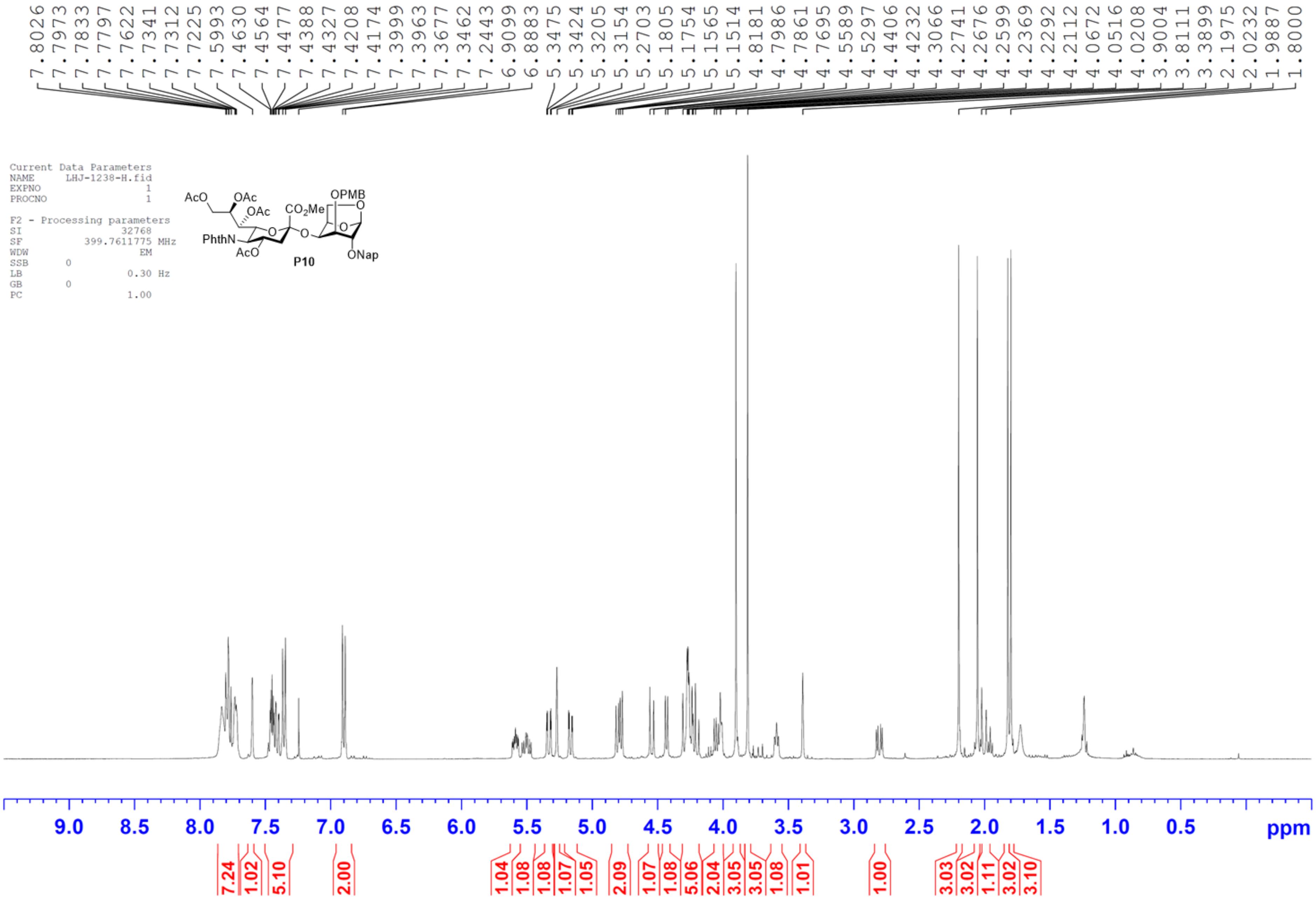
7.8026 7.7973 7.7833 7.7797 7.7622 7.7341 7.7312 7.7225 7.5993 7.4630 7.4564 7.4477 7.4388 7.4327 7.4208 7.4174 7.3999 7.3963 7.3677 7.3462 7.2443 6.9099 6.8883 5.3475 5.3424 5.3205 5.3154 5.2703 5.1805 5.1754 5.1565 5.1514 4.8181 4.7986 4.7861 4.7695 4.5589 4.5297 4.4406 4.4232 4.3066 4.2741 4.2676 4.2599 4.2369 4.2292 4.2112 4.0672 4.0516 4.0208 3.9004 3.8111 3.3899 2.1975 2.0232 1.9887 1.8000
Current Data Parameters
NAME LHJ-1238-H.fid
EXPNO 1
PROCNO 1
F2 - Processing parameters
SI 32768
SF 399.7611775 MHz
WDW EM
SSB 0
LB 0.30 Hz
GB 0
PC 1.00
AcO
OAc
OAc
CO2Me
OPMB
PhthN
AcO
O
ONap
P10
9.0 8.5 8.0 7.5 7.0 6.5 6.0 5.5 5.0 4.5 4.0 3.5 3.0 2.5 2.0 1.5 1.0 0.5 ppm
7.24 1.02 5.10 2.00 1.04 1.08 1.08 1.07 1.05 2.09 1.07 1.08 5.06 2.04 3.05 3.05 1.08 1.01 1.00 3.03 3.02 1.11 3.02 3.10

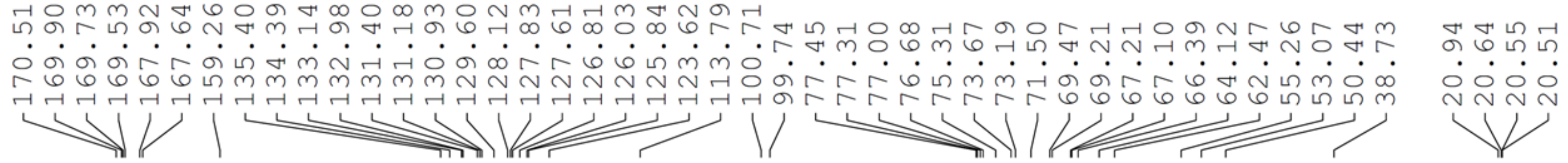

Current Data Parameters
NAME LHJ-1238-C.fid
EXPNO 1
PROCNO 1

F2 - Processing parameters
SI 65536
SF 100.5214599 MHz
WDW EM
SSB 0
LB 1.00 Hz
GB 0
PC 1.00

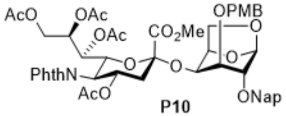

P10

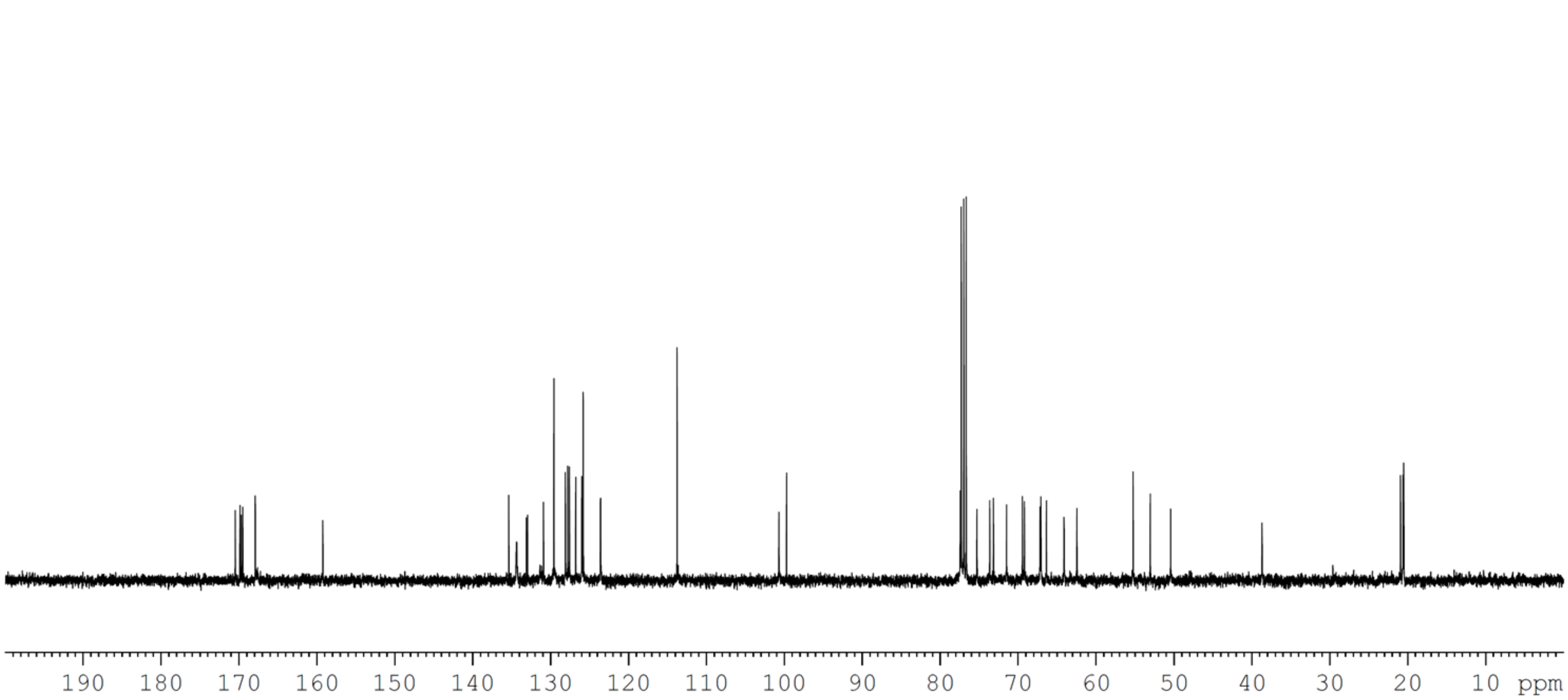

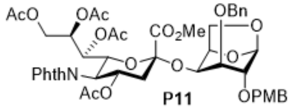


```
Current Data Parameters
NAME            LHJ-1228
EXPNO                  7
PROCNO                 1

F2 - Acquisition Parameters
Date_           20141119
Time               17.17
INSTRUM            spect
PROBHD   5 mm DUL 13C-1
PULPROG             zg30
TD                 32768
SOLVENT            CDCl3
NS                    12
DS                     0
SWH             6410.256 Hz
FIDRES          0.195625 Hz
AQ             2.5559540 sec
RG                   114
DW                78.000 usec
DE                  6.00 usec
TE                 300.0 K
D1            2.00000000 sec
TD0                    1

======== CHANNEL f1 ========
NUC1                  1H
P1                 10.00 usec
PL1                -2.40 dB
SFO1         400.1528010 MHz

F2 - Processing parameters
SI                 16384
SF           400.1500174 MHz
WDW                   EM
SSB       0
LB        0 Hz
GB        0
PC                  1.00
```

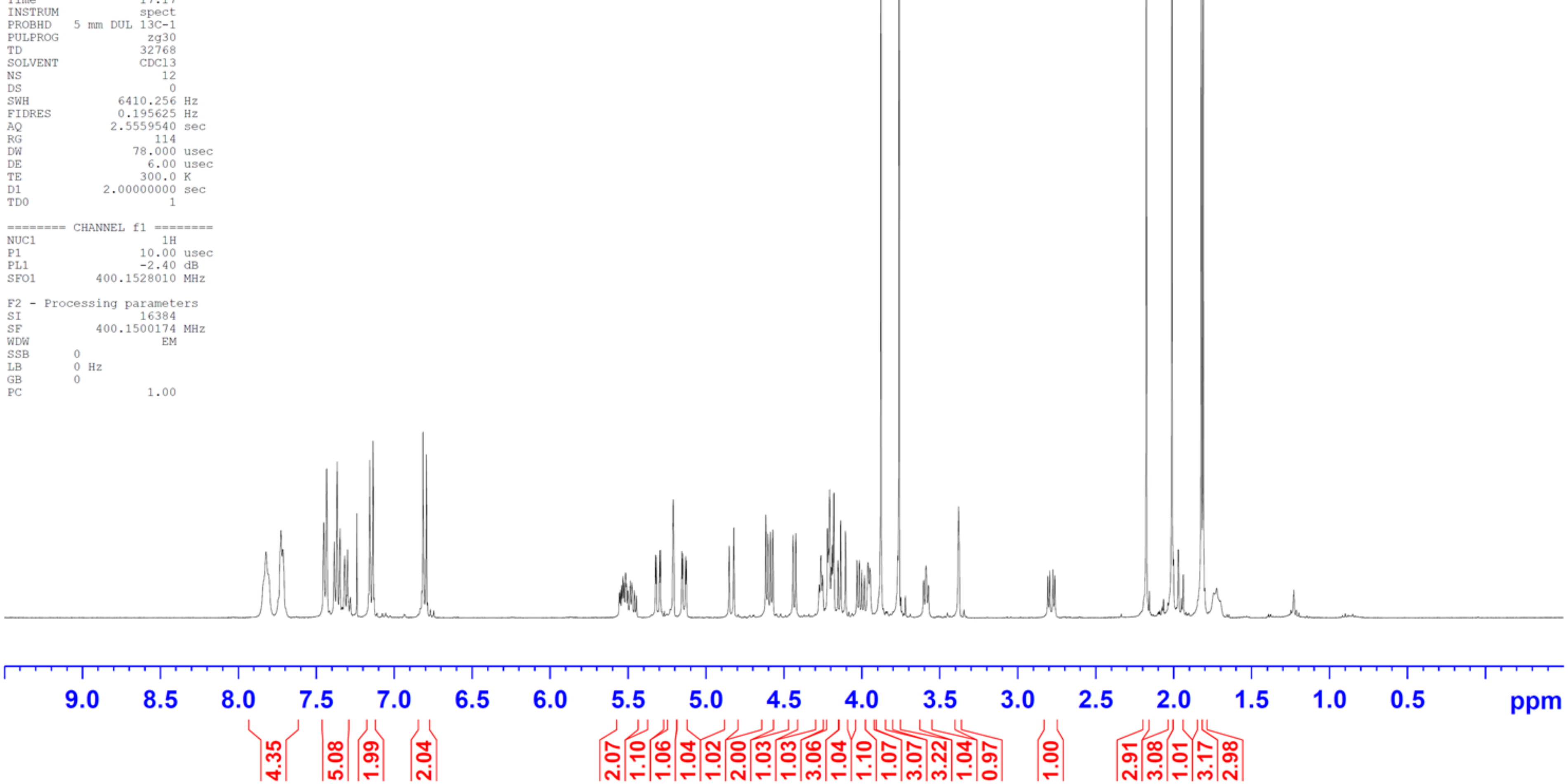

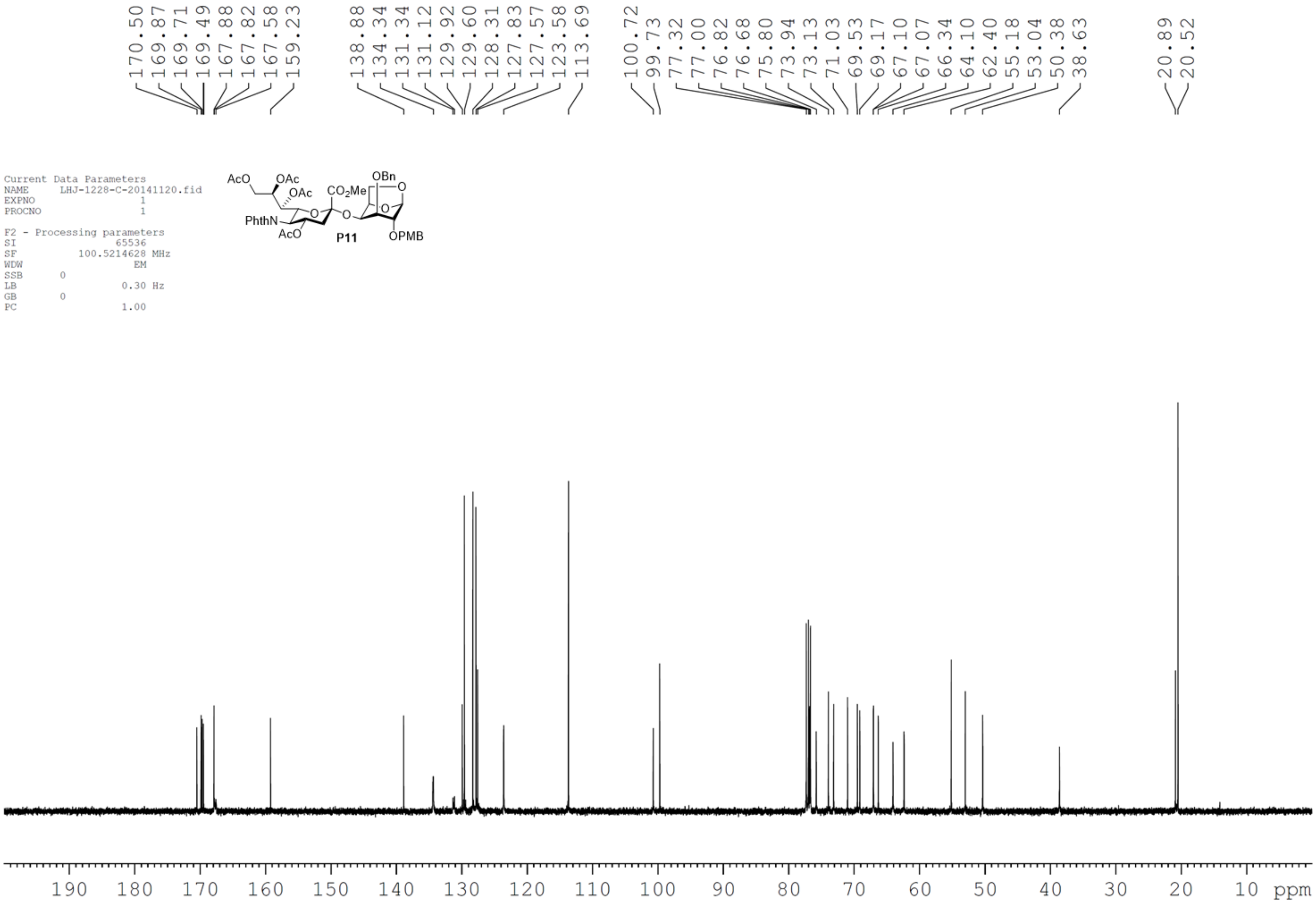
170.50
169.87
169.71
169.49
167.88
167.82
167.58
159.23
138.88
134.34
131.34
131.12
129.92
129.60
128.31
127.83
127.57
123.58
113.69
100.72
99.73
77.32
77.00
76.82
76.68
75.80
73.94
73.13
71.03
69.53
69.17
67.10
67.07
66.34
64.10
62.40
55.18
53.04
50.38
38.63
20.89
20.52
Current Data Parameters
NAME LHJ-1228-C-20141120.fid
EXPNO 1
PROCNO 1
F2 - Processing parameters
SI 65536
SF 100.5214628 MHz
WDW EM
SSB 0
LB 0.30 Hz
GB 0
PC 1.00
AcO
OAc
OAc
CO2Me
OBn
O
PhthN
O
O
O
AcO
P11
OPMB
190
180
170
160
150
140
130
120
110
100
90
80
70
60
50
40
30
20
10
ppm

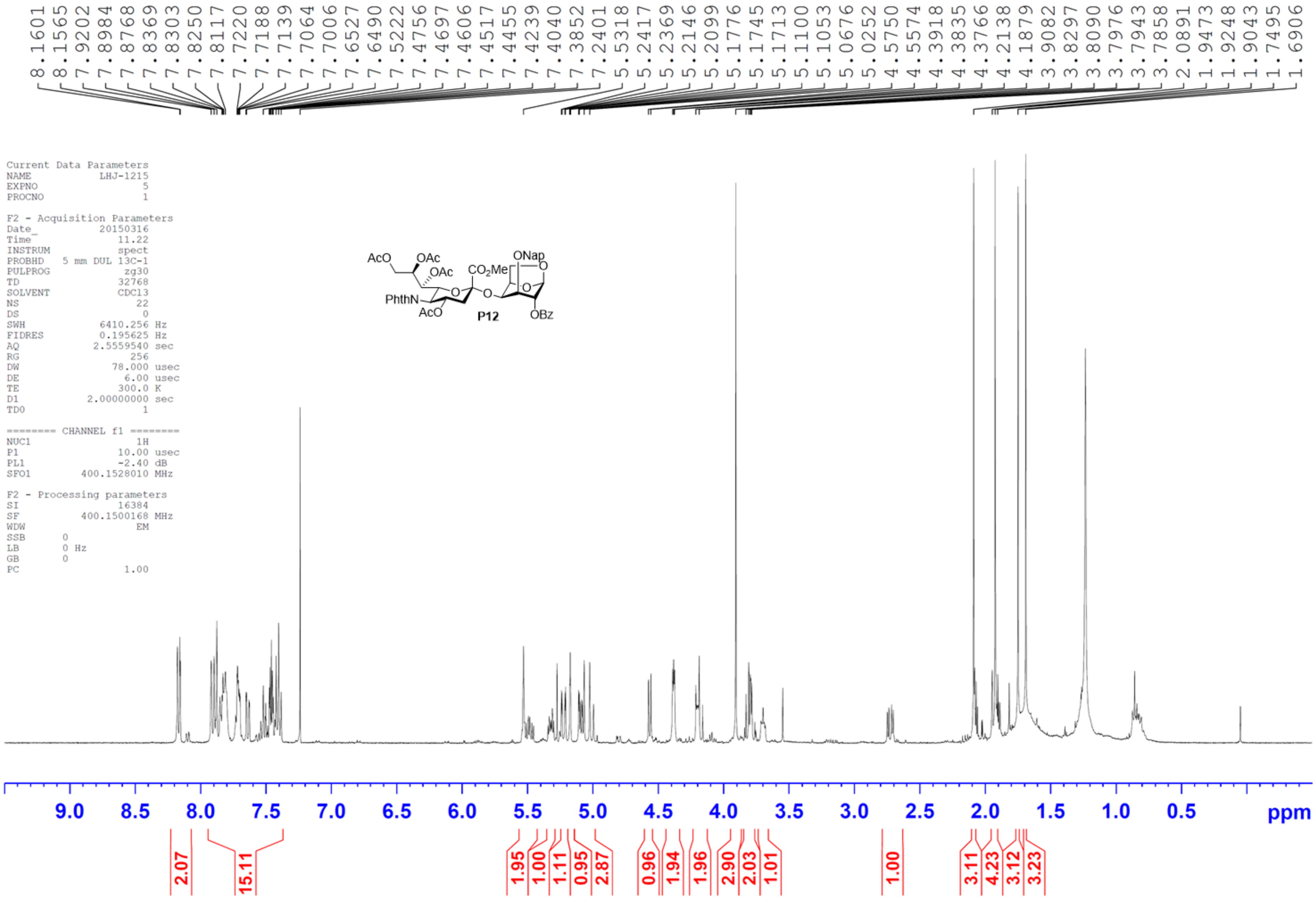
8.1601 8.1565 7.9202 7.8984 7.8768 7.8369 7.8303 7.8250 7.8117 7.7220 7.7188 7.7139 7.7064 7.7006 7.6527 7.6490 7.5222 7.4756 7.4697 7.4606 7.4517 7.4455 7.4239 7.4040 7.3852 7.2401 5.5318 5.2417 5.2369 5.2146 5.2099 5.1776 5.1745 5.1713 5.1100 5.1053 5.0676 5.0252 4.5750 4.5574 4.3918 4.3835 4.3766 4.2138 4.1879 3.9082 3.8297 3.8090 3.7976 3.7943 3.7858 2.0891 1.9473 1.9248 1.9043 1.7495 1.6906
Current Data Parameters
NAME LHJ-1215
EXPNO 5
PROCNO 1
F2 - Acquisition Parameters
Date_ 20150316
Time 11.22
INSTRUM spect
PROBHD 5 mm DUL 13C-1
PULPROG zg30
TD 32768
SOLVENT CDCl3
NS 22
DS 0
SWH 6410.256 Hz
FIDRES 0.195625 Hz
AQ 2.5559540 sec
RG 256
DW 78.000 usec
DE 6.00 usec
TE 300.0 K
D1 2.00000000 sec
TD0 1
======== CHANNEL f1 ========
NUC1 1H
P1 10.00 usec
PL1 -2.40 dB
SFO1 400.1528010 MHz
F2 - Processing parameters
SI 16384
SF 400.1500168 MHz
WDW EM
SSB 0
LB 0 Hz
GB 0
PC 1.00
AcO
OAc
OAc
CO2Me
ONap
O
O
O
O
PhthN
AcO
P12
OBz
9.0 8.5 8.0 7.5 7.0 6.5 6.0 5.5 5.0 4.5 4.0 3.5 3.0 2.5 2.0 1.5 1.0 0.5 ppm
2.07 15.11 1.95 1.00 1.11 0.95 2.87 0.96 1.94 1.96 2.90 2.03 1.01 1.00 3.11 4.23 3.12 3.23

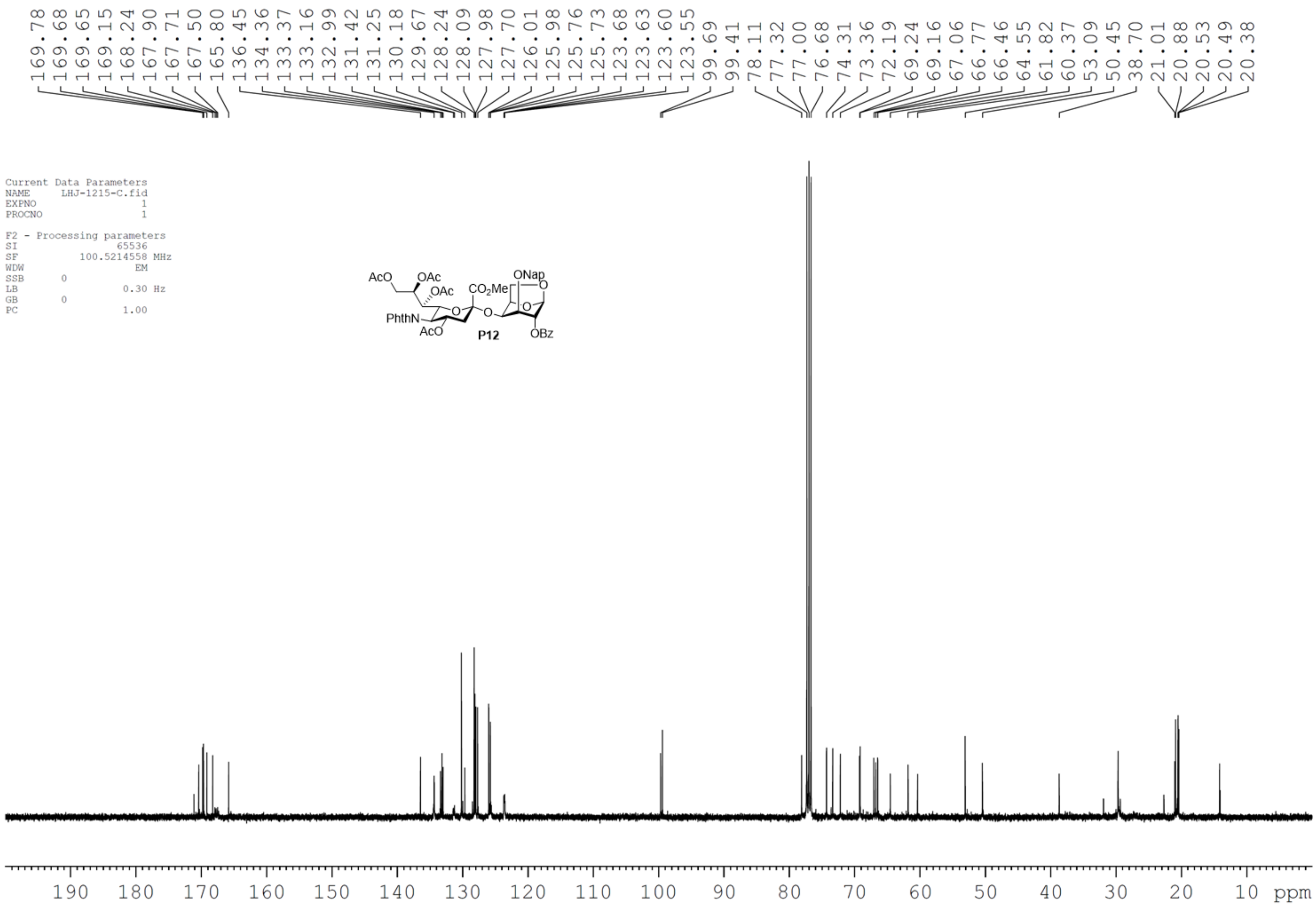
169.78
169.68
169.65
169.15
168.24
167.90
167.71
167.50
165.80
136.45
134.36
133.37
133.16
132.99
131.42
131.25
130.18
129.67
128.24
128.09
127.98
127.70
126.01
125.98
125.76
125.73
123.68
123.63
123.60
123.55
99.69
99.41
78.11
77.32
77.00
76.68
74.31
73.36
72.19
69.24
69.16
67.06
66.77
66.46
64.55
61.82
60.37
53.09
50.45
38.70
21.01
20.88
20.53
20.49
20.38
Current Data Parameters
NAME LHJ-1215-C.fid
EXPNO 1
PROCNO 1
F2 - Processing parameters
SI 65536
SF 100.5214558 MHz
WDW EM
SSB 0
LB 0.30 Hz
GB 0
PC 1.00
AcO
OAc
OAc
CO2Me
ONap
PhthN
AcO
P12
OBz
190 180 170 160 150 140 130 120 110 100 90 80 70 60 50 40 30 20 10 ppm

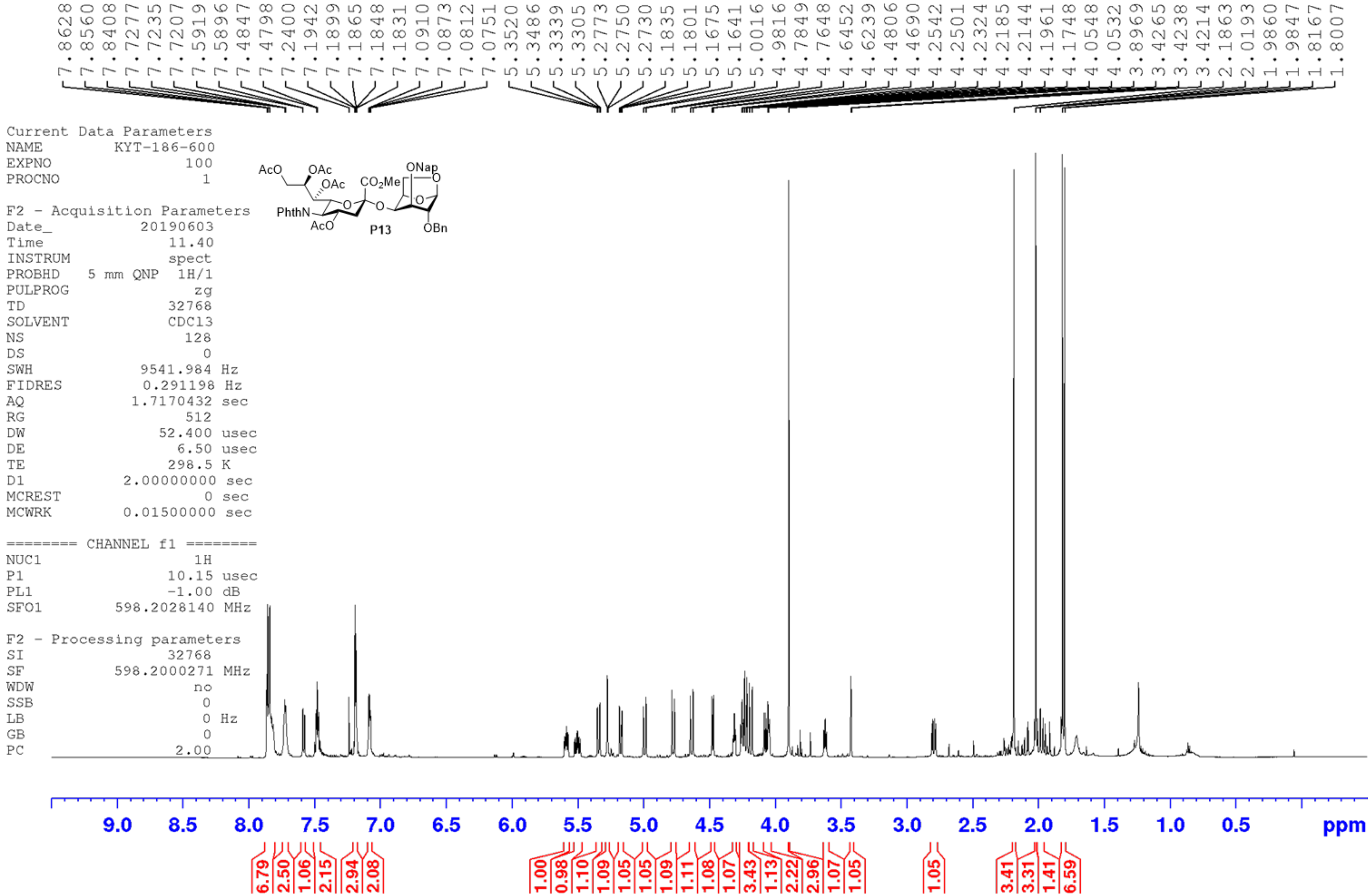
7.8628
7.8560
7.8408
7.7277
7.7235
7.7207
7.5919
7.5896
7.4847
7.4798
7.2400
7.1942
7.1899
7.1865
7.1848
7.1831
7.0910
7.0873
7.0812
7.0751
5.3520
5.3486
5.3339
5.3305
5.2773
5.2750
5.2730
5.1835
5.1801
5.1675
5.1641
5.0016
4.9816
4.7849
4.7648
4.6452
4.6239
4.4806
4.4690
4.2542
4.2501
4.2324
4.2185
4.2144
4.1961
4.1748
4.0548
4.0532
3.8969
3.4265
3.4238
3.4214
2.1863
2.0193
1.9860
1.9847
1.8167
1.8007
Current Data Parameters
NAME KYT-186-600
EXPNO 100
PROCNO 1
F2 - Acquisition Parameters
Date_ 20190603
Time 11.40
INSTRUM spect
PROBHD 5 mm QNP 1H/1
PULPROG zg
TD 32768
SOLVENT CDCl3
NS 128
DS 0
SWH 9541.984 Hz
FIDRES 0.291198 Hz
AQ 1.7170432 sec
RG 512
DW 52.400 usec
DE 6.50 usec
TE 298.5 K
D1 2.00000000 sec
MCREST 0 sec
MCWRK 0.01500000 sec
======== CHANNEL f1 ========
NUC1 1H
P1 10.15 usec
PL1 -1.00 dB
SFO1 598.2028140 MHz
F2 - Processing parameters
SI 32768
SF 598.2000271 MHz
WDW no
SSB 0
LB 0 Hz
GB 0
PC 2.00
AcO
OAc
OAc
CO2Me
ONap
O
PhthN
O
O
O
AcO
P13
OBn
9.0
8.5
8.0
7.5
7.0
6.5
6.0
5.5
5.0
4.5
4.0
3.5
3.0
2.5
2.0
1.5
1.0
0.5
ppm
6.79
2.50
1.06
2.15
2.94
2.08
1.00
0.98
1.10
1.09
1.05
1.05
1.09
1.11
1.08
1.07
3.43
1.13
2.22
2.96
1.07
1.05
1.05
3.41
3.31
1.41
6.59

```
Current Data Parameters
NAME            KYT-186-600
EXPNO                     2
PROCNO                    1

F2 - Acquisition Parameters
Date_              20190603
Time                   9.04
INSTRUM               spect
PROBHD   5 mm QNP   1H/1
PULPROG                zgpg
TD                    32768
SOLVENT               CDCl3
NS                      940
DS                        0
SWH               45045.047 Hz
FIDRES             1.374666 Hz
AQ                0.3637248 sec
RG                     4096
DW                   11.100 usec
DE                     6.50 usec
TE                    298.8 K
D1               3.50000000 sec
d11              0.03000000 sec
DELTA            3.40000010 sec
MCREST                    0 sec
MCWRK            0.01500000 sec

======== CHANNEL f1 ========
NUC1                    13C
P1                     6.20 usec
PL1                       0 dB
SFO1            150.4340559 MHz

======== CHANNEL f2 ========
CPDPRG[2            waltz16
NUC2                     1H
PCPD2                 92.00 usec
PL2                  120.00 dB
PL12                  12.00 dB
PL13                  15.00 dB
SFO2            598.2029910 MHz

F2 - Processing parameters
SI                    65536
SF              150.4175184 MHz
WDW                      EM
SSB                       0
LB                     3.00 Hz
GB                        0
PC                     1.00
```

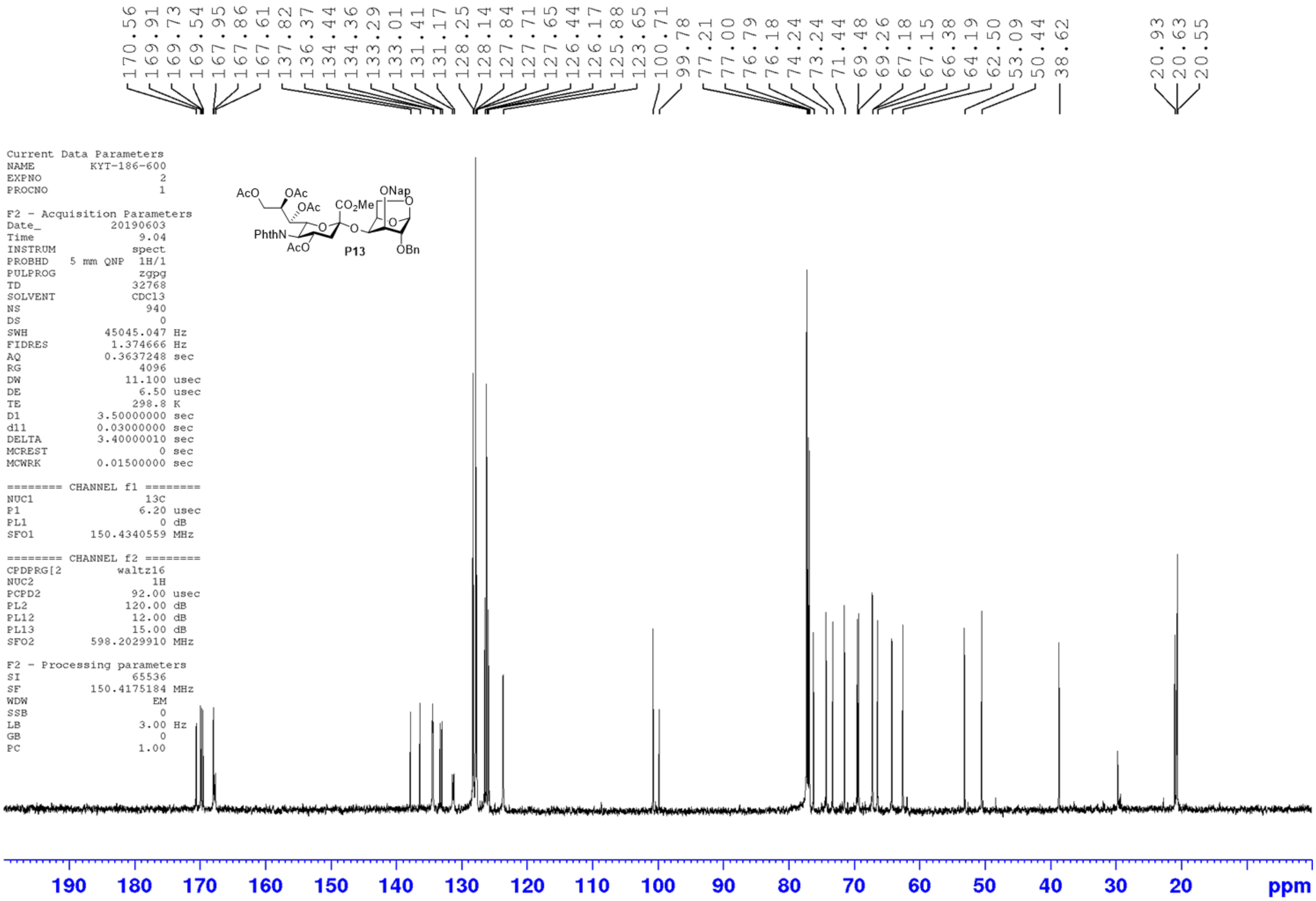

HMBC

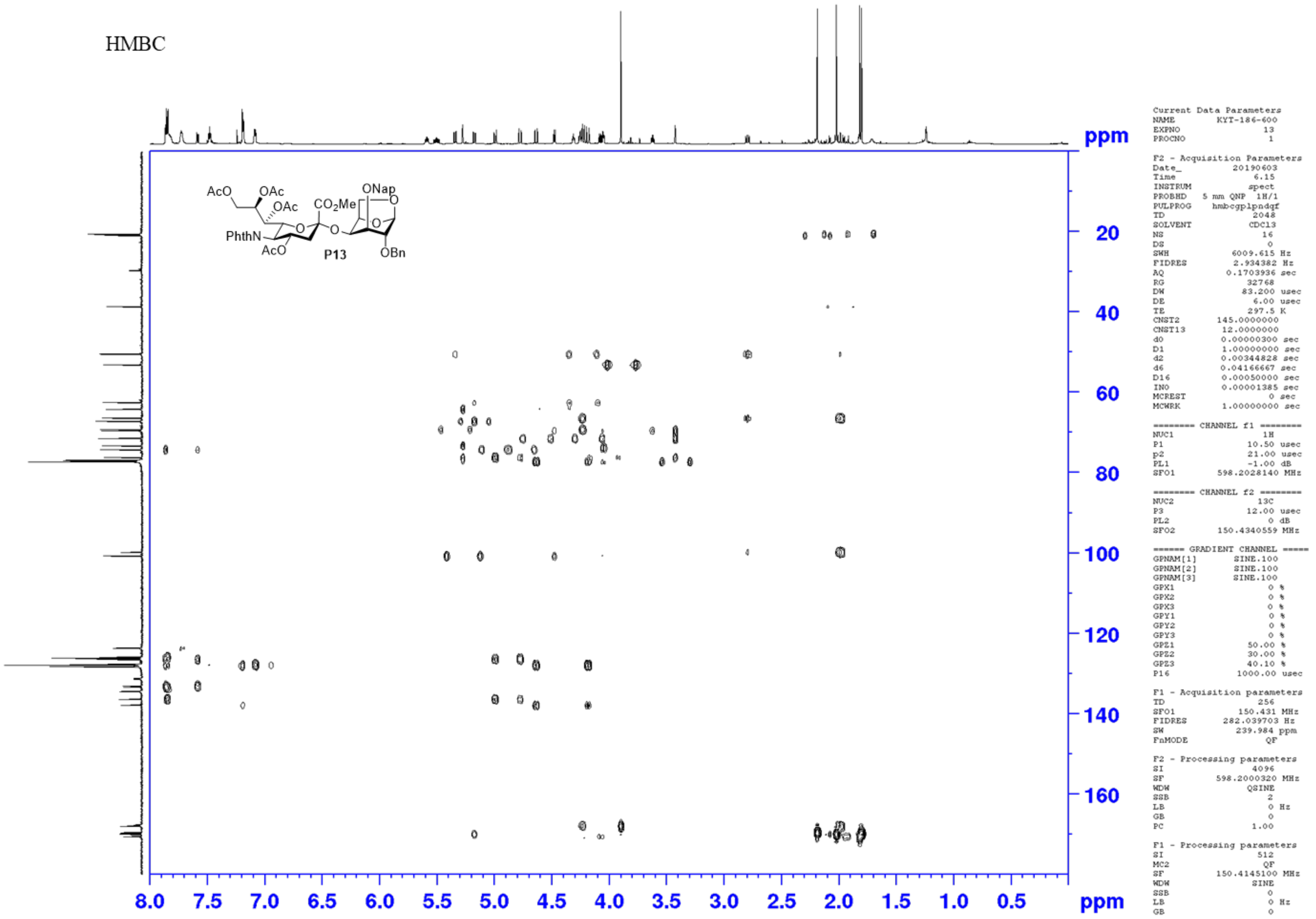
AcO
OAc
OAc
CO2Me
ONap
PhthN
AcO
O
O
O
O
P13
OBn
ppm
20
40
60
80
100
120
140
160
8.0
7.5
7.0
6.5
6.0
5.5
5.0
4.5
4.0
3.5
3.0
2.5
2.0
1.5
1.0
0.5
ppm
Current Data Parameters
NAME KYT-186-600
EXPNO 13
PROCNO 1
F2 - Acquisition Parameters
Date_ 20190603
Time 6.15
INSTRUM spect
PROBHD 5 mm QNP 1H/1
PULPROG hmbcgplpndqf
TD 2048
SOLVENT CDCl3
NS 16
DS 0
SWH 6009.615 Hz
FIDRES 2.934382 Hz
AQ 0.1703936 sec
RG 32768
DW 83.200 usec
DE 6.00 usec
TE 297.5 K
CNST2 145.0000000
CNST13 12.0000000
d0 0.00000300 sec
D1 1.00000000 sec
d2 0.00344828 sec
d6 0.04166667 sec
D16 0.00050000 sec
IN0 0.00001385 sec
MCREST 0 sec
MCWRK 1.00000000 sec
======== CHANNEL f1 ========
NUC1 1H
P1 10.50 usec
p2 21.00 usec
PL1 -1.00 dB
SFO1 598.2028140 MHz
======== CHANNEL f2 ========
NUC2 13C
P3 12.00 usec
PL2 0 dB
SFO2 150.4340559 MHz
====== GRADIENT CHANNEL =====
GPNAM[1] SINE.100
GPNAM[2] SINE.100
GPNAM[3] SINE.100
GPX1 0 %
GPX2 0 %
GPX3 0 %
GPY1 0 %
GPY2 0 %
GPY3 0 %
GPZ1 50.00 %
GPZ2 30.00 %
GPZ3 40.10 %
P16 1000.00 usec
F1 - Acquisition parameters
TD 256
SFO1 150.431 MHz
FIDRES 282.039703 Hz
SW 239.984 ppm
FnMODE QF
F2 - Processing parameters
SI 4096
SF 598.2000320 MHz
WDW QSINE
SSB 2
LB 0 Hz
GB 0
PC 1.00
F1 - Processing parameters
SI 512
MC2 QF
SF 150.4145100 MHz
WDW SINE
SSB 0
LB 0 Hz
GB 0

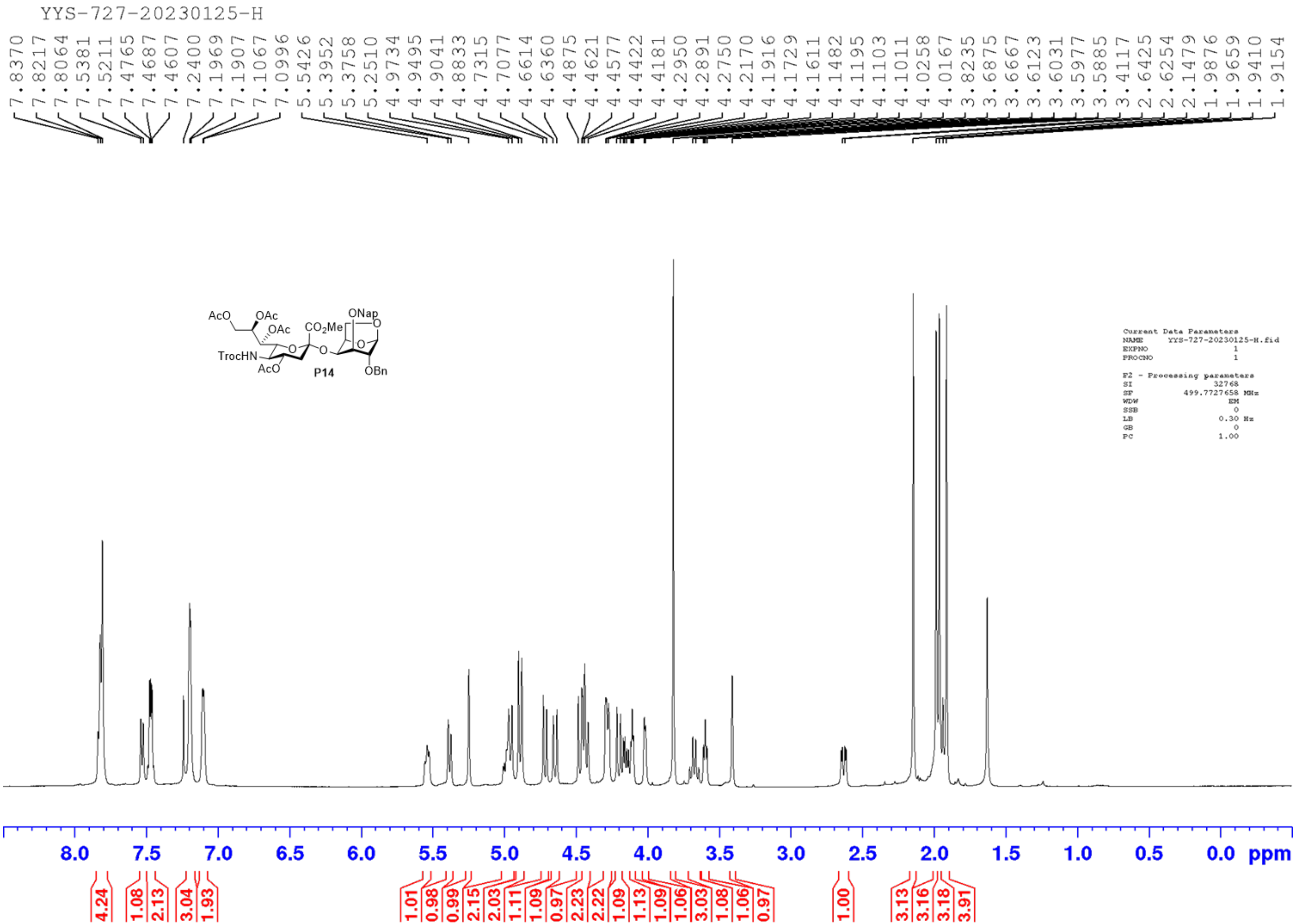

YYS-727-20230125-H
7.8370
7.8217
7.8064
7.5381
7.5211
7.4765
7.4687
7.4607
7.2400
7.1969
7.1907
7.1067
7.0996
5.5426
5.3952
5.3758
5.2510
4.9734
4.9495
4.9041
4.8833
4.7315
4.7077
4.6614
4.6360
4.4875
4.4621
4.4577
4.4422
4.4181
4.2950
4.2891
4.2750
4.2170
4.1916
4.1729
4.1611
4.1482
4.1195
4.1103
4.1011
4.0258
4.0167
3.8235
3.6875
3.6667
3.6123
3.6031
3.5977
3.5885
3.4117
2.6425
2.6254
2.1479
1.9876
1.9659
1.9410
1.9154
AcO
OAc
OAc
CO2Me
ONap
O
TrocHN
AcO
O
O
O
P14
OBn
Current Data Parameters
NAME YYS-727-20230125-H.fid
EXPNO 1
PROCNO 1
F2 - Processing parameters
SI 32768
SF 499.7727658 MHz
WDW EM
SSB 0
LB 0.30 Hz
GB 0
PC 1.00
8.0
7.5
7.0
6.5
6.0
5.5
5.0
4.5
4.0
3.5
3.0
2.5
2.0
1.5
1.0
0.5
0.0
ppm
4.24
1.08
2.13
3.04
1.93
1.01
0.98
0.99
2.15
2.03
1.11
1.09
0.97
2.23
2.22
1.09
1.13
1.09
1.06
3.03
1.08
1.06
0.97
1.00
3.13
3.16
3.18
3.91

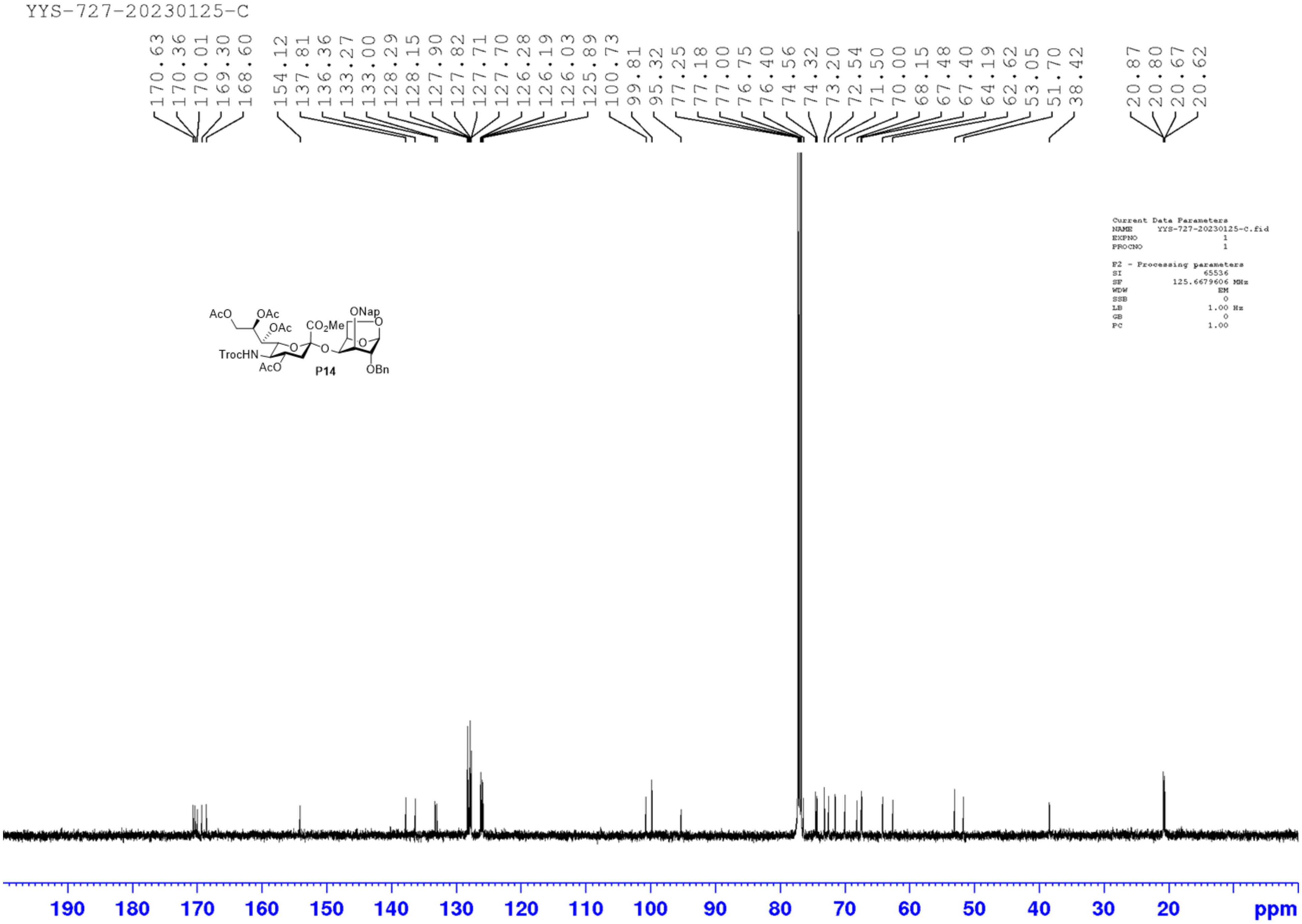
YYS-727-20230125-C
170.63
170.36
170.01
169.30
168.60
154.12
137.81
136.36
133.27
133.00
128.29
128.15
127.90
127.82
127.71
127.70
126.28
126.19
126.03
125.89
100.73
99.81
95.32
77.25
77.18
77.00
76.75
76.40
74.56
74.32
73.20
72.54
71.50
70.00
68.15
67.48
67.40
64.19
62.62
53.05
51.70
38.42
20.87
20.80
20.67
20.62
Current Data Parameters
NAME YYS-727-20230125-C.fid
EXPNO 1
PROCNO 1
F2 - Processing parameters
SI 65536
SF 125.6679606 MHz
WDW EM
SSB 0
LB 1.00 Hz
GB 0
PC 1.00
AcO
OAc
OAc
CO2Me
ONap
TrocHN
AcO
P14
OBn
190
180
170
160
150
140
130
120
110
100
90
80
70
60
50
40
30
20
ppm

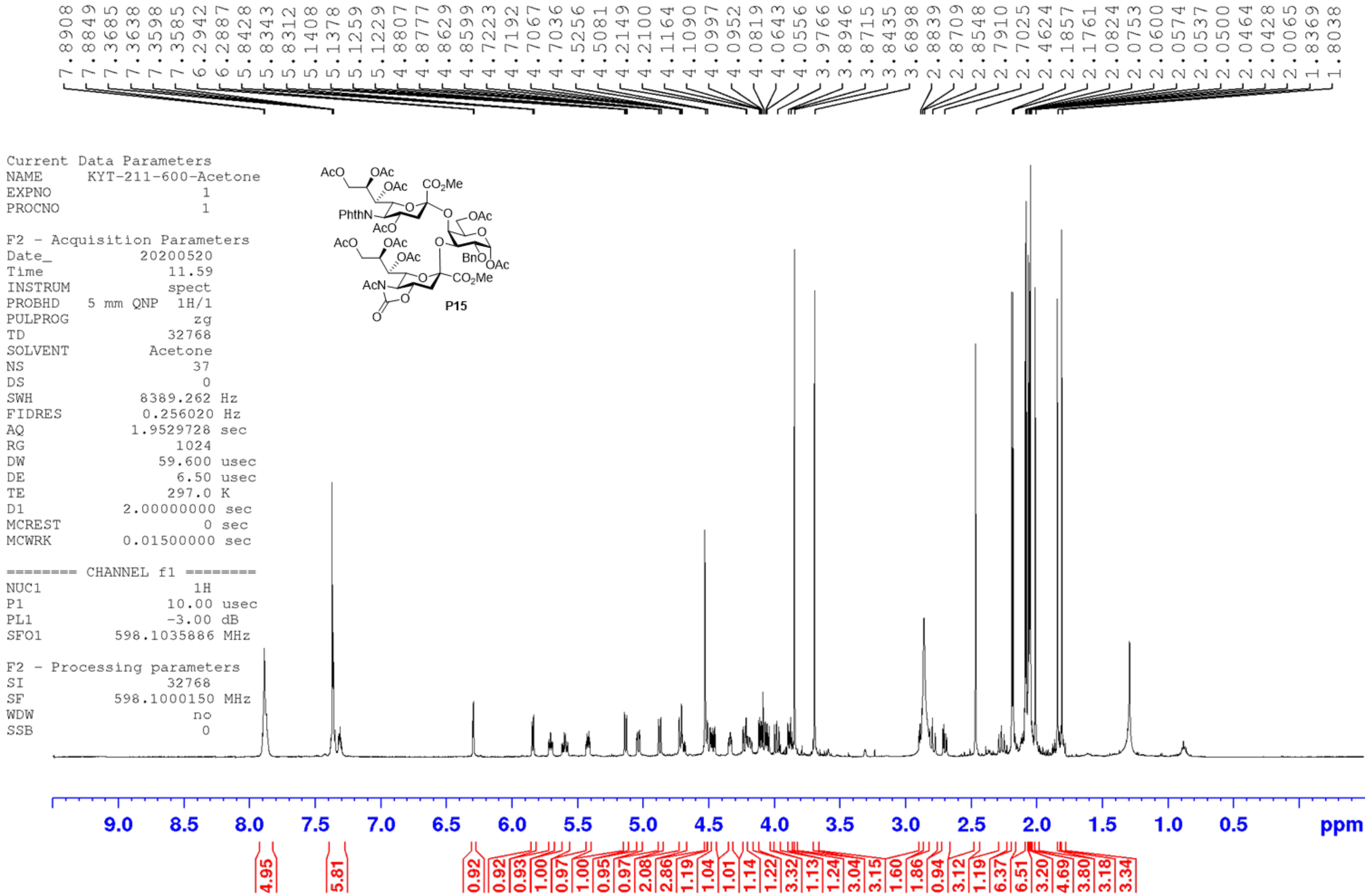
7.8908
7.8849
7.3685
7.3638
7.3598
7.3585
6.2942
6.2887
5.8428
5.8343
5.8312
5.1408
5.1378
5.1259
5.1229
4.8807
4.8777
4.8629
4.8599
4.7223
4.7192
4.7067
4.7036
4.5256
4.5081
4.2149
4.2100
4.1164
4.1090
4.0997
4.0952
4.0819
4.0643
4.0556
3.9766
3.8946
3.8715
3.8435
3.6898
2.8839
2.8709
2.8548
2.7910
2.7025
2.4624
2.1857
2.1761
2.0824
2.0753
2.0600
2.0574
2.0537
2.0500
2.0464
2.0428
2.0065
1.8369
1.8038
Current Data Parameters
NAME KYT-211-600-Acetone
EXPNO 1
PROCNO 1
F2 - Acquisition Parameters
Date_ 20200520
Time 11.59
INSTRUM spect
PROBHD 5 mm QNP 1H/1
PULPROG zg
TD 32768
SOLVENT Acetone
NS 37
DS 0
SWH 8389.262 Hz
FIDRES 0.256020 Hz
AQ 1.9529728 sec
RG 1024
DW 59.600 usec
DE 6.50 usec
TE 297.0 K
D1 2.00000000 sec
MCREST 0 sec
MCWRK 0.01500000 sec
======== CHANNEL f1 ========
NUC1 1H
P1 10.00 usec
PL1 -3.00 dB
SFO1 598.1035886 MHz
F2 - Processing parameters
SI 32768
SF 598.1000150 MHz
WDW no
SSB 0
AcO
OAc
OAc
CO2Me
PhthN
AcO
O
OAc
AcO
OAc
OAc
BnO
OAc
AcN
CO2Me
P15
9.0
8.5
8.0
7.5
7.0
6.5
6.0
5.5
5.0
4.5
4.0
3.5
3.0
2.5
2.0
1.5
1.0
0.5
ppm
4.95
5.81
0.92
0.92
0.93
1.00
0.97
1.00
0.95
0.97
2.08
2.86
1.19
1.04
1.01
1.14
1.22
3.32
1.13
1.24
3.04
3.15
1.60
1.86
0.94
3.12
1.19
6.37
6.51
3.20
4.69
3.80
3.18
3.34

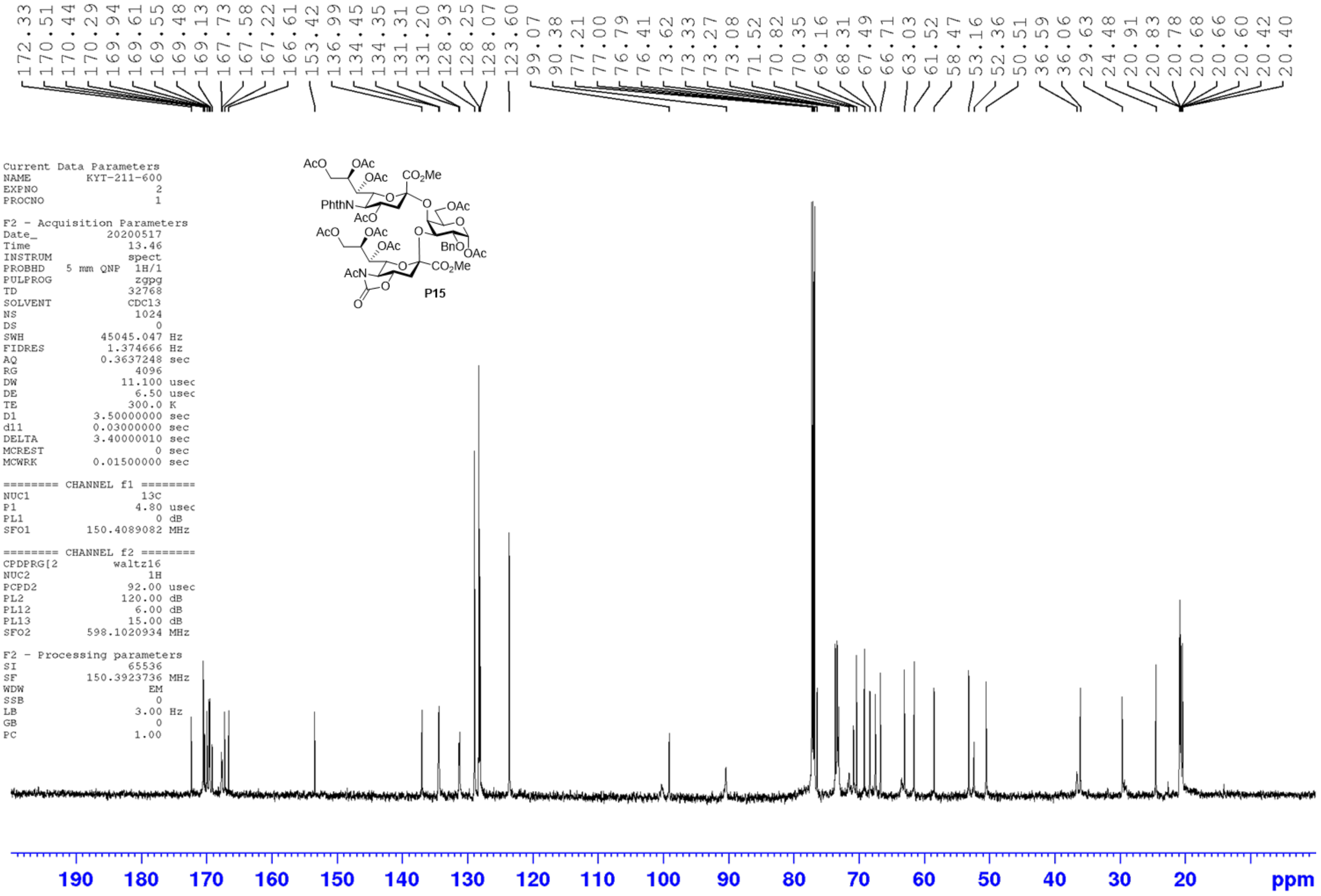
172.33
170.51
170.44
170.29
169.94
169.61
169.55
169.48
169.13
167.73
167.58
167.22
166.61
153.42
136.99
134.45
134.35
131.31
131.20
128.93
128.25
128.07
123.60
99.07
90.38
77.21
77.00
76.79
76.41
73.62
73.33
73.27
73.08
71.52
70.82
70.35
69.16
68.31
67.49
66.71
63.03
61.52
58.47
53.16
52.36
50.51
36.59
36.06
29.63
24.48
20.91
20.83
20.78
20.68
20.66
20.60
20.42
20.40
Current Data Parameters
NAME KYT-211-600
EXPNO 2
PROCNO 1
F2 - Acquisition Parameters
Date_ 20200517
Time 13.46
INSTRUM spect
PROBHD 5 mm QNP 1H/1
PULPROG zgpg
TD 32768
SOLVENT CDCl3
NS 1024
DS 0
SWH 45045.047 Hz
FIDRES 1.374666 Hz
AQ 0.3637248 sec
RG 4096
DW 11.100 usec
DE 6.50 usec
TE 300.0 K
D1 3.50000000 sec
d11 0.03000000 sec
DELTA 3.40000010 sec
MCREST 0 sec
MCWRK 0.01500000 sec
======== CHANNEL f1 ========
NUC1 13C
P1 4.80 usec
PL1 0 dB
SFO1 150.4089082 MHz
======== CHANNEL f2 ========
CPDPRG[2 waltz16
NUC2 1H
PCPD2 92.00 usec
PL2 120.00 dB
PL12 6.00 dB
PL13 15.00 dB
SFO2 598.1020934 MHz
F2 - Processing parameters
SI 65536
SF 150.3923736 MHz
WDW EM
SSB 0
LB 3.00 Hz
GB 0
PC 1.00
AcO
OAc
OAc
CO_2Me
PhthN
AcO
O
O
OAc
O
AcO
OAc
OAc
O
BnO
OAc
CO_2Me
AcN
O
O
P15
190
180
170
160
150
140
130
120
110
100
90
80
70
60
50
40
30
20
ppm

HMBC

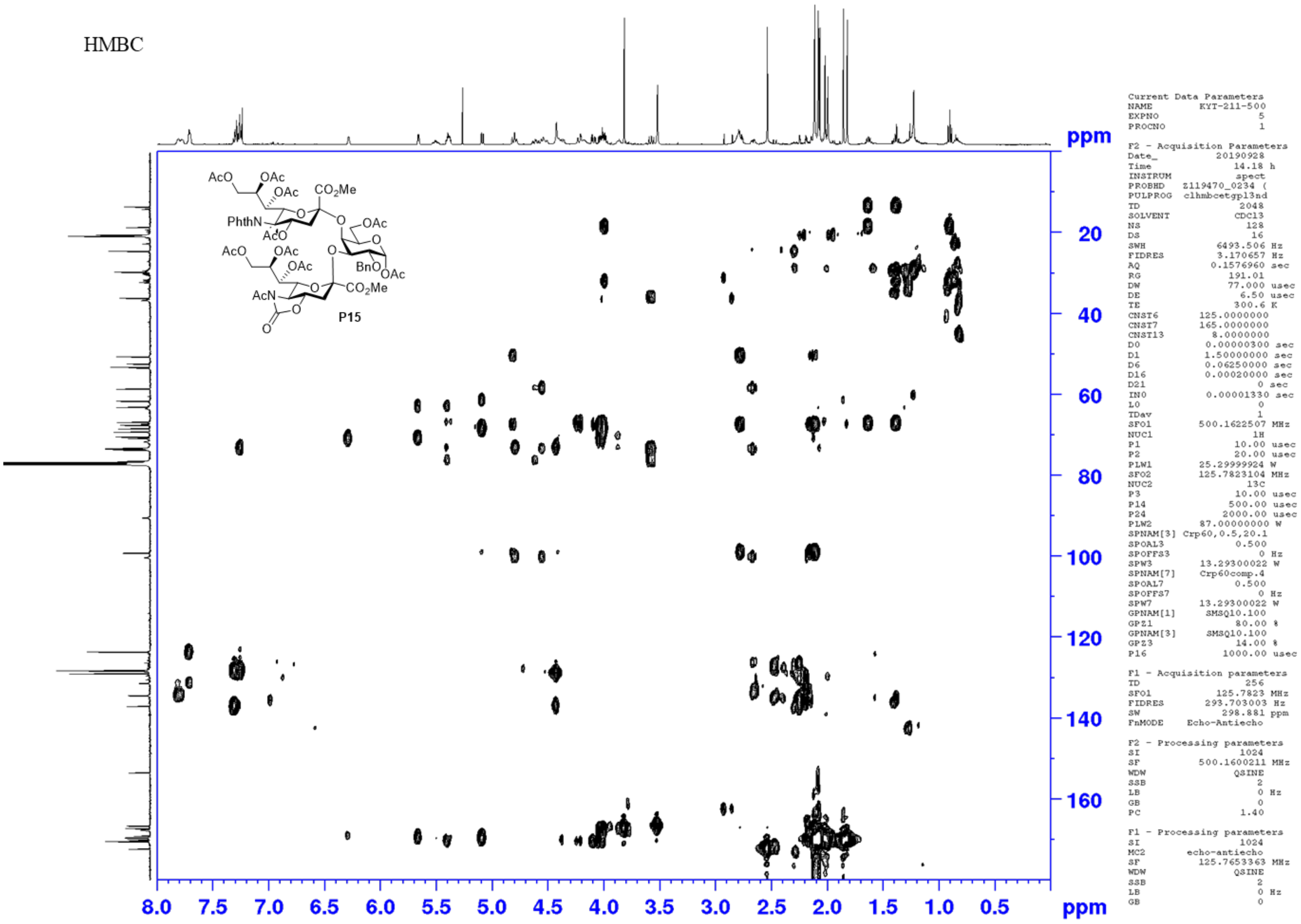
AcO
OAc
OAc
CO2Me
PhthN
AcO
OAc
O
AcO
OAc
OAc
BnO
OAc
AcN
CO2Me
O
P15
ppm
20
40
60
80
100
120
140
160
8.0
7.5
7.0
6.5
6.0
5.5
5.0
4.5
4.0
3.5
3.0
2.5
2.0
1.5
1.0
0.5
ppm
Current Data Parameters
NAME KYT-211-500
EXPNO 5
PROCNO 1
F2 - Acquisition Parameters
Date_ 20190928
Time 14.18 h
INSTRUM spect
PROBHD Z119470_0234 (
PULPROG clhmbcetgpl3nd
TD 2048
SOLVENT CDCl3
NS 128
DS 16
SWH 6493.506 Hz
FIDRES 3.170657 Hz
AQ 0.1576960 sec
RG 191.01
DW 77.000 usec
DE 6.50 usec
TE 300.6 K
CNST6 125.0000000
CNST7 165.0000000
CNST13 8.0000000
D0 0.00000300 sec
D1 1.50000000 sec
D6 0.06250000 sec
D16 0.00020000 sec
D21 0 sec
IN0 0.00001330 sec
L0 0
TDav 1
SFO1 500.1622507 MHz
NUC1 1H
P1 10.00 usec
P2 20.00 usec
PLW1 25.29999924 W
SFO2 125.7823104 MHz
NUC2 13C
P3 10.00 usec
P14 500.00 usec
P24 2000.00 usec
PLW2 87.00000000 W
SPNAM[3] Crp60,0.5,20.1
SPOAL3 0.500
SPOFFS3 0 Hz
SPW3 13.29300022 W
SPNAM[7] Crp60comp.4
SPOAL7 0.500
SPOFFS7 0 Hz
SPW7 13.29300022 W
GPNAM[1] SMSQ10.100
GPZ1 80.00 %
GPNAM[3] SMSQ10.100
GPZ3 14.00 %
P16 1000.00 usec
F1 - Acquisition parameters
TD 256
SFO1 125.7823 MHz
FIDRES 293.703003 Hz
SW 298.881 ppm
FnMODE Echo-Antiecho
F2 - Processing parameters
SI 1024
SF 500.1600211 MHz
WDW QSINE
SSB 2
LB 0 Hz
GB 0
PC 1.40
F1 - Processing parameters
SI 1024
MC2 echo-antiecho
SF 125.7653363 MHz
WDW QSINE
SSB 2
LB 0 Hz
GB 0

Current Data Parameters	
NAME	KYT-304-600
EXPNO	21
PROCNO	1
**F2 - Acquisition Parameters**	
Date_	20190902
Time	5.28
INSTRUM	spect
PROBHD	5 mm QNP 1H/1
PULPROG	zg
TD	32768
SOLVENT	Acetone
NS	64
DS	0
SWH	9541.984 Hz
FIDRES	0.291198 Hz
AQ	1.7170432 sec
RG	512
DW	52.400 usec
DE	6.50 usec
TE	299.9 K
D1	3.00000000 sec
MCREST	0 sec
MCWRK	0.01500000 sec
======== CHANNEL f1 ========	
NUC1	1H
P1	10.00 usec
PL1	-4.00 dB
SFO1	598.2032901 MHz
**F2 - Processing parameters**	
SI	32768
SF	598.2000107 MHz
WDW	no
SSB	0

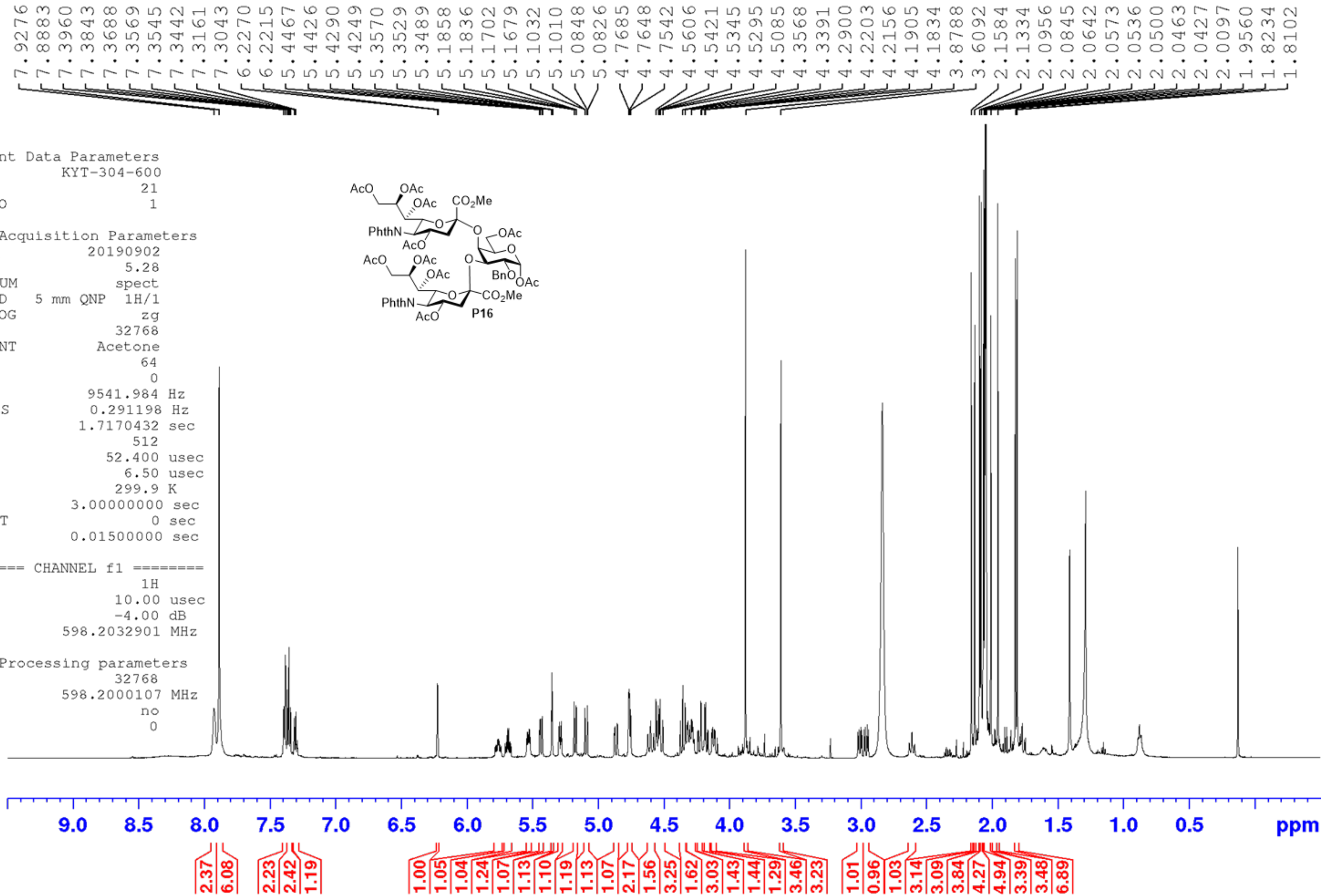

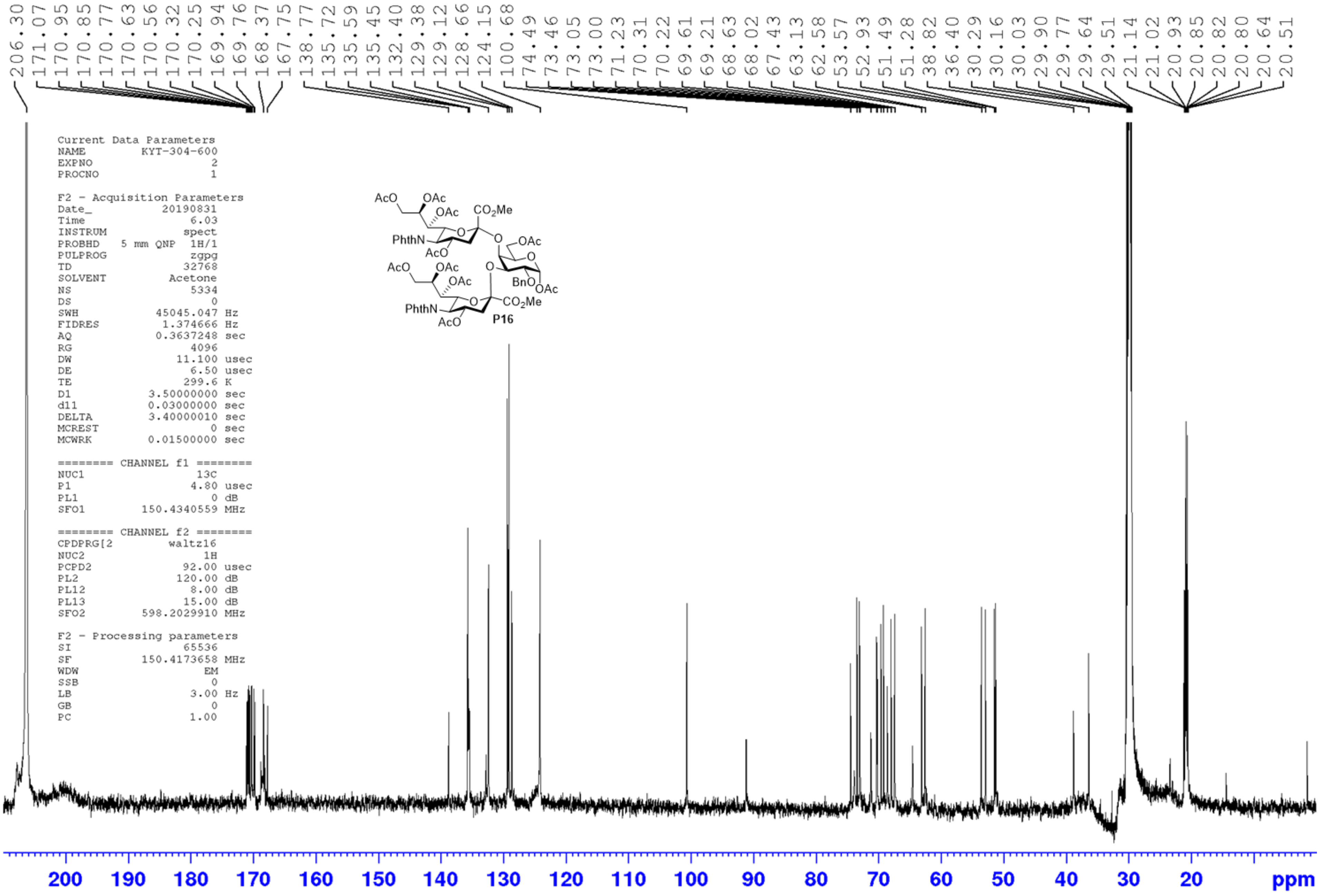
206.30
171.07
170.95
170.85
170.77
170.63
170.56
170.32
170.25
169.94
169.76
168.37
167.75
138.77
135.72
135.59
135.45
132.40
129.38
129.12
128.66
124.15
100.68
74.49
73.46
73.05
73.00
71.23
70.31
70.22
69.61
69.21
68.63
68.02
67.43
63.13
62.58
53.57
52.93
51.49
51.28
38.82
36.40
30.29
30.16
30.03
29.90
29.77
29.64
29.51
21.14
21.02
20.93
20.85
20.82
20.80
20.64
20.51
Current Data Parameters
NAME KYT-304-600
EXPNO 2
PROCNO 1
F2 - Acquisition Parameters
Date_ 20190831
Time 6.03
INSTRUM spect
PROBHD 5 mm QNP 1H/1
PULPROG zgpg
TD 32768
SOLVENT Acetone
NS 5334
DS 0
SWH 45045.047 Hz
FIDRES 1.374666 Hz
AQ 0.3637248 sec
RG 4096
DW 11.100 usec
DE 6.50 usec
TE 299.6 K
D1 3.50000000 sec
d11 0.03000000 sec
DELTA 3.40000010 sec
MCREST 0 sec
MCWRK 0.01500000 sec
======== CHANNEL f1 ========
NUC1 13C
P1 4.80 usec
PL1 0 dB
SFO1 150.4340559 MHz
======== CHANNEL f2 ========
CPDPRG[2 waltz16
NUC2 1H
PCPD2 92.00 usec
PL2 120.00 dB
PL12 8.00 dB
PL13 15.00 dB
SFO2 598.2029910 MHz
F2 - Processing parameters
SI 65536
SF 150.4173658 MHz
WDW EM
SSB 0
LB 3.00 Hz
GB 0
PC 1.00
AcO
OAc
OAc
CO2Me
PhthN
O
O
OAc
AcO
O
O
AcO
OAc
OAc
BnO
OAc
O
CO2Me
PhthN
AcO
P16
200
190
180
170
160
150
140
130
120
110
100
90
80
70
60
50
40
30
20
ppm

HMBC

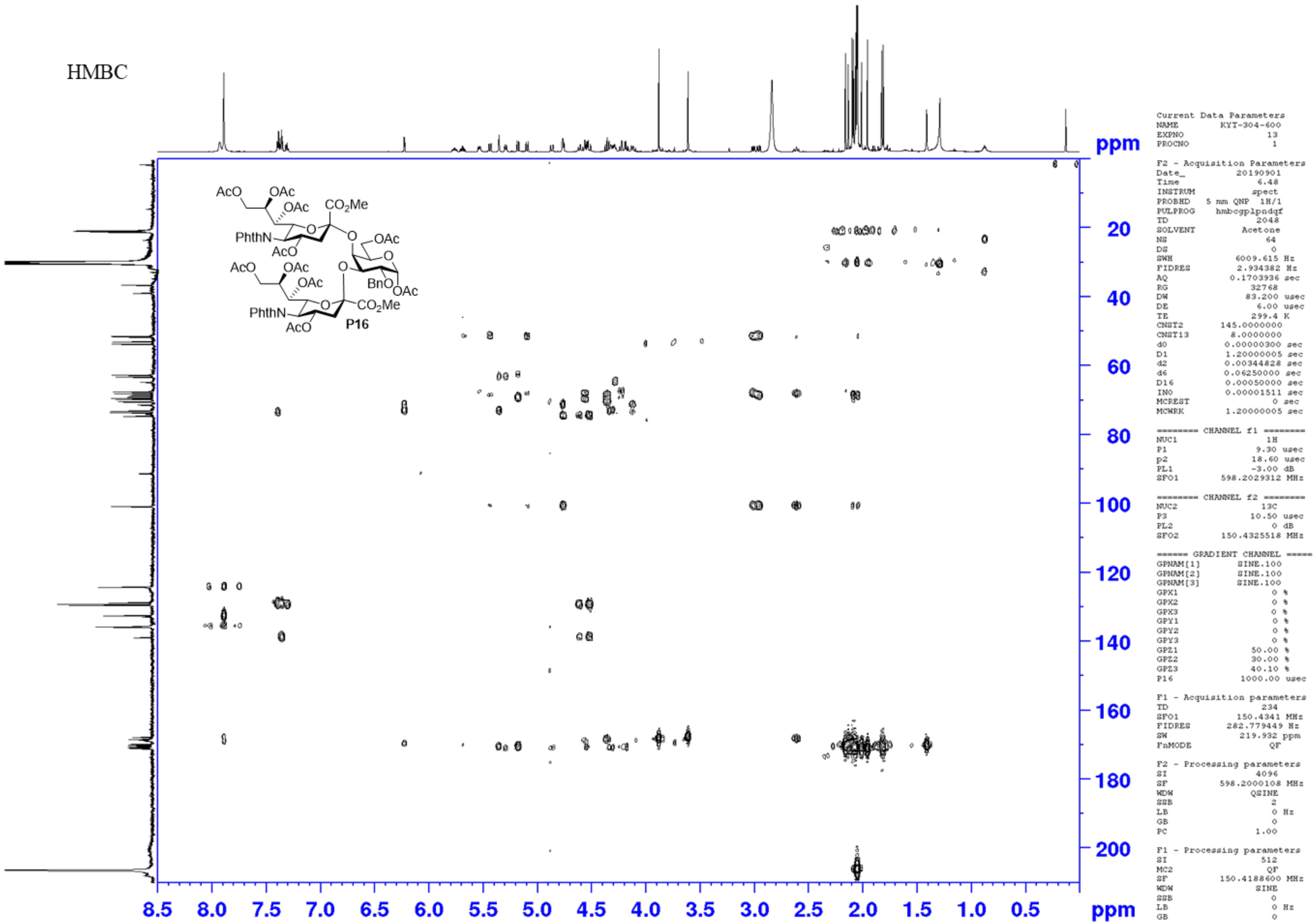

AcO
OAc
OAc
CO2Me
PhthN
AcO
O
OAc
O
O
BnO
OAc
CO2Me
P16
ppm
20
40
60
80
100
120
140
160
180
200
8.5
8.0
7.5
7.0
6.5
6.0
5.5
5.0
4.5
4.0
3.5
3.0
2.5
2.0
1.5
1.0
0.5
ppm
Current Data Parameters
NAME KYT-304-600
EXPNO 13
PROCNO 1
F2 - Acquisition Parameters
Date_ 20190901
Time 6.48
INSTRUM spect
PROBHD 5 mm QNP 1H/1
PULPROG hmbcgplpndqf
TD 2048
SOLVENT Acetone
NS 64
DS 0
SWH 6009.615 Hz
FIDRES 2.934382 Hz
AQ 0.1703936 sec
RG 32768
DW 83.200 usec
DE 6.00 usec
TE 299.4 K
CNST2 145.0000000
CNST13 8.0000000
d0 0.00000300 sec
D1 1.20000005 sec
d2 0.00344828 sec
d6 0.06250000 sec
D16 0.00050000 sec
IN0 0.00001511 sec
MCREST 0 sec
MCWRK 1.20000005 sec
======== CHANNEL f1 ========
NUC1 1H
P1 9.30 usec
p2 18.60 usec
PL1 -3.00 dB
SFO1 598.2029312 MHz
======== CHANNEL f2 ========
NUC2 13C
P3 10.50 usec
PL2 0 dB
SFO2 150.4325518 MHz
====== GRADIENT CHANNEL =====
GPNAM[1] SINE.100
GPNAM[2] SINE.100
GPNAM[3] SINE.100
GPX1 0 %
GPX2 0 %
GPX3 0 %
GPY1 0 %
GPY2 0 %
GPY3 0 %
GPZ1 50.00 %
GPZ2 30.00 %
GPZ3 40.10 %
P16 1000.00 usec
F1 - Acquisition parameters
TD 234
SFO1 150.4341 MHz
FIDRES 282.779449 Hz
SW 219.932 ppm
FnMODE QF
F2 - Processing parameters
SI 4096
SF 598.2000108 MHz
WDW QSINE
SSB 2
LB 0 Hz
GB 0
PC 1.00
F1 - Processing parameters
SI 512
MC2 QF
SF 150.4188600 MHz
WDW SINE
SSB 0
LB 0 Hz
GB 0

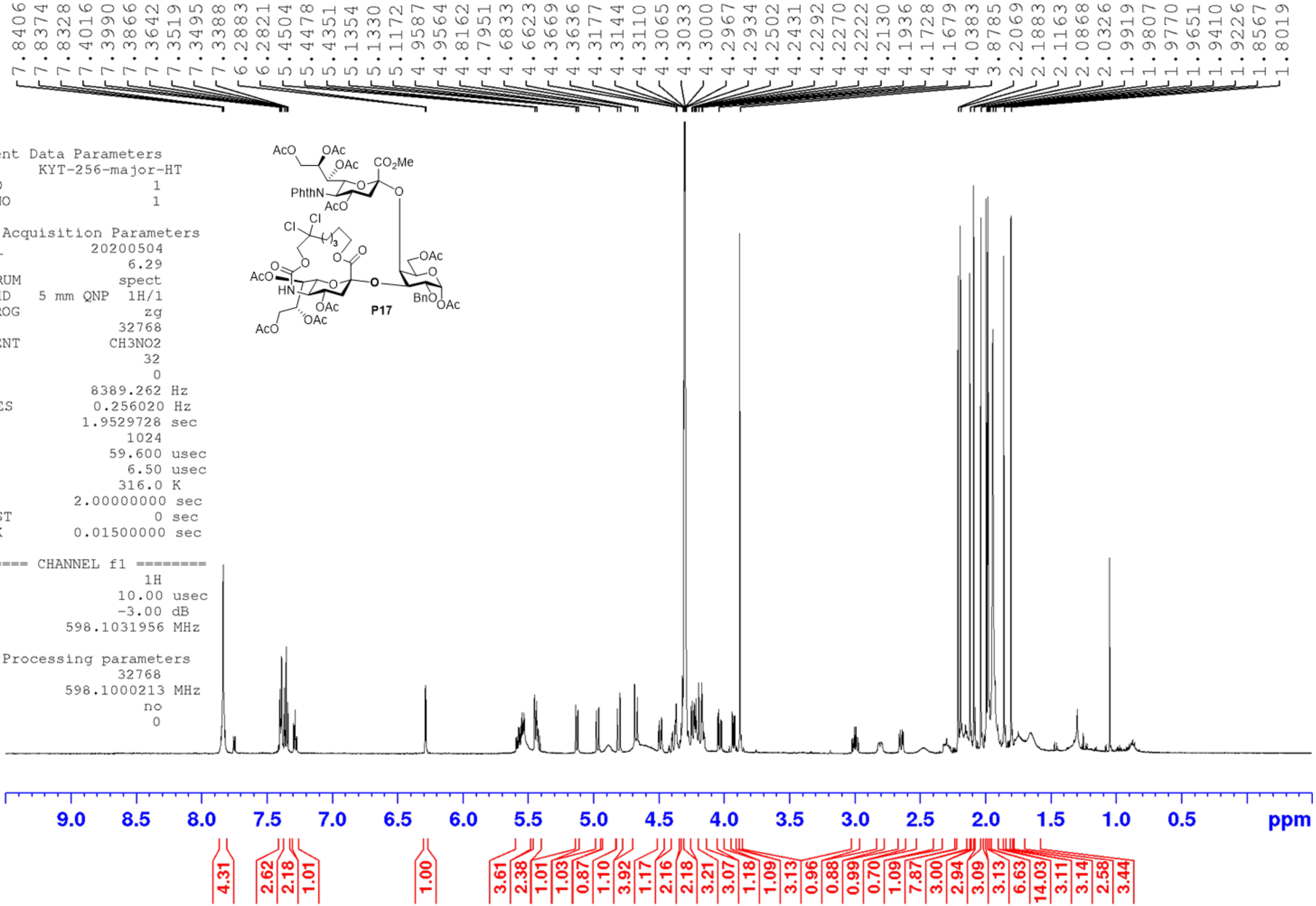


```
Current Data Parameters
NAME          KYT-256-major-HT
EXPNO                    1
PROCNO                   1

F2 - Acquisition Parameters
Date_             20200504
Time                  6.29
INSTRUM              spect
PROBHD   5 mm QNP   1H/1
PULPROG                 zg
TD                   32768
SOLVENT             CH3NO2
NS                      32
DS                       0
SWH               8389.262 Hz
FIDRES            0.256020 Hz
AQ               1.9529728 sec
RG                    1024
DW                  59.600 usec
DE                    6.50 usec
TE                   316.0 K
D1              2.00000000 sec
MCREST                   0 sec
MCWRK           0.01500000 sec

======== CHANNEL f1 ========
NUC1                    1H
P1                   10.00 usec
PL1                  -3.00 dB
SFO1           598.1031956 MHz

F2 - Processing parameters
SI                   32768
SF             598.1000213 MHz
WDW                     no
SSB                      0
```

P17

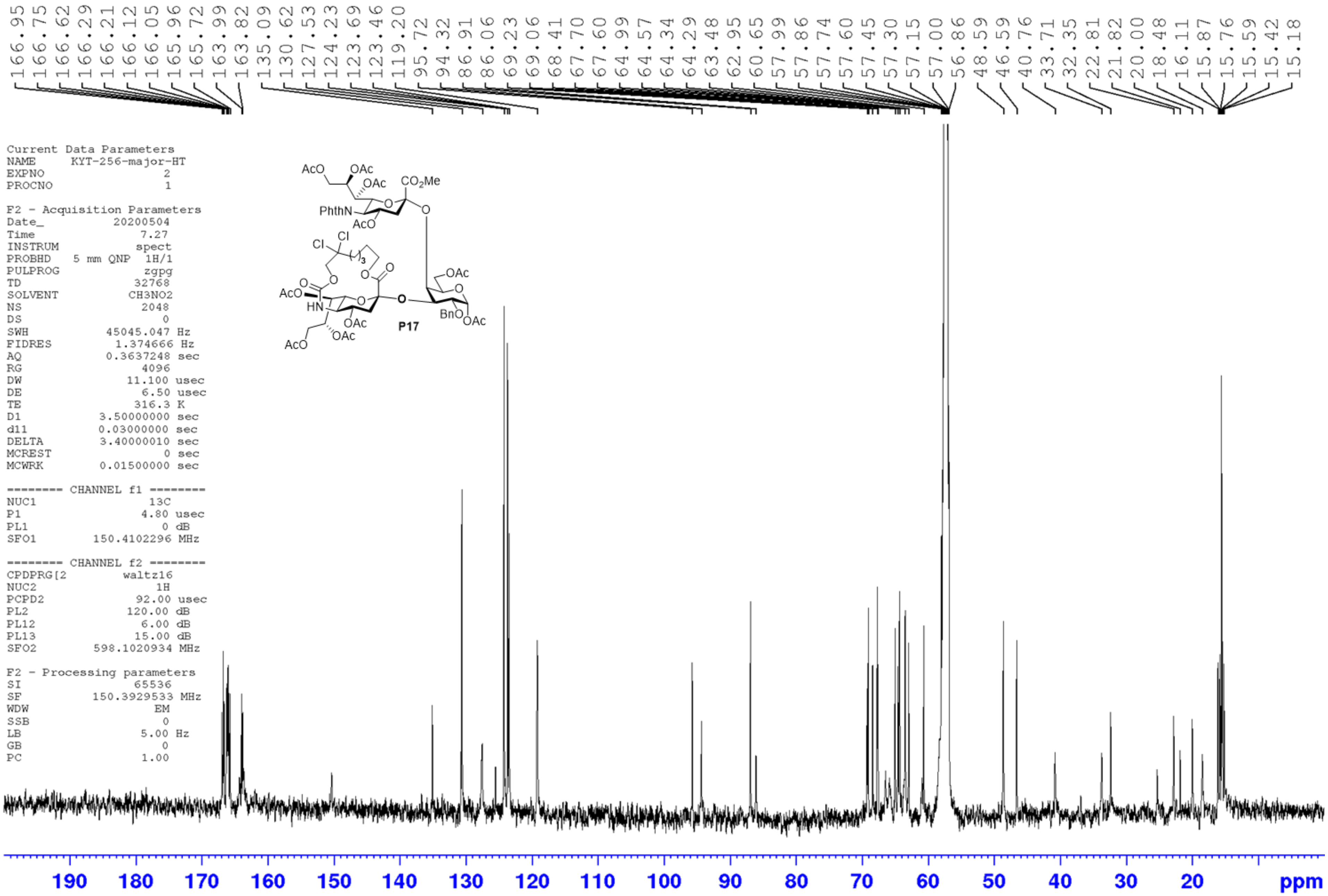
166.95
166.75
166.62
166.29
166.21
166.12
166.05
165.96
165.72
163.99
163.82
135.09
130.62
127.53
124.23
123.69
123.46
119.20
95.72
94.32
86.91
86.06
69.23
69.06
68.41
67.70
67.60
64.99
64.57
64.34
64.29
63.48
62.95
60.65
57.99
57.86
57.74
57.60
57.45
57.30
57.15
57.00
56.86
48.59
46.59
40.76
33.71
32.35
22.81
21.82
20.00
18.48
16.11
15.87
15.76
15.59
15.42
15.18
Current Data Parameters
NAME KYT-256-major-HT
EXPNO 2
PROCNO 1
F2 - Acquisition Parameters
Date_ 20200504
Time 7.27
INSTRUM spect
PROBHD 5 mm QNP 1H/1
PULPROG zgpg
TD 32768
SOLVENT CH3NO2
NS 2048
DS 0
SWH 45045.047 Hz
FIDRES 1.374666 Hz
AQ 0.3637248 sec
RG 4096
DW 11.100 usec
DE 6.50 usec
TE 316.3 K
D1 3.50000000 sec
d11 0.03000000 sec
DELTA 3.40000010 sec
MCREST 0 sec
MCWRK 0.01500000 sec
======== CHANNEL f1 ========
NUC1 13C
P1 4.80 usec
PL1 0 dB
SFO1 150.4102296 MHz
======== CHANNEL f2 ========
CPDPRG[2 waltz16
NUC2 1H
PCPD2 92.00 usec
PL2 120.00 dB
PL12 6.00 dB
PL13 15.00 dB
SFO2 598.1020934 MHz
F2 - Processing parameters
SI 65536
SF 150.3929533 MHz
WDW EM
SSB 0
LB 5.00 Hz
GB 0
PC 1.00
190
180
170
160
150
140
130
120
110
100
90
80
70
60
50
40
30
20
ppm
AcO
OAc
OAc
CO2Me
PhthN
AcO
O
Cl
Cl
3
O
AcO
HN
OAc
OAc
AcO
BnO
OAc
OAc

P17

COSY

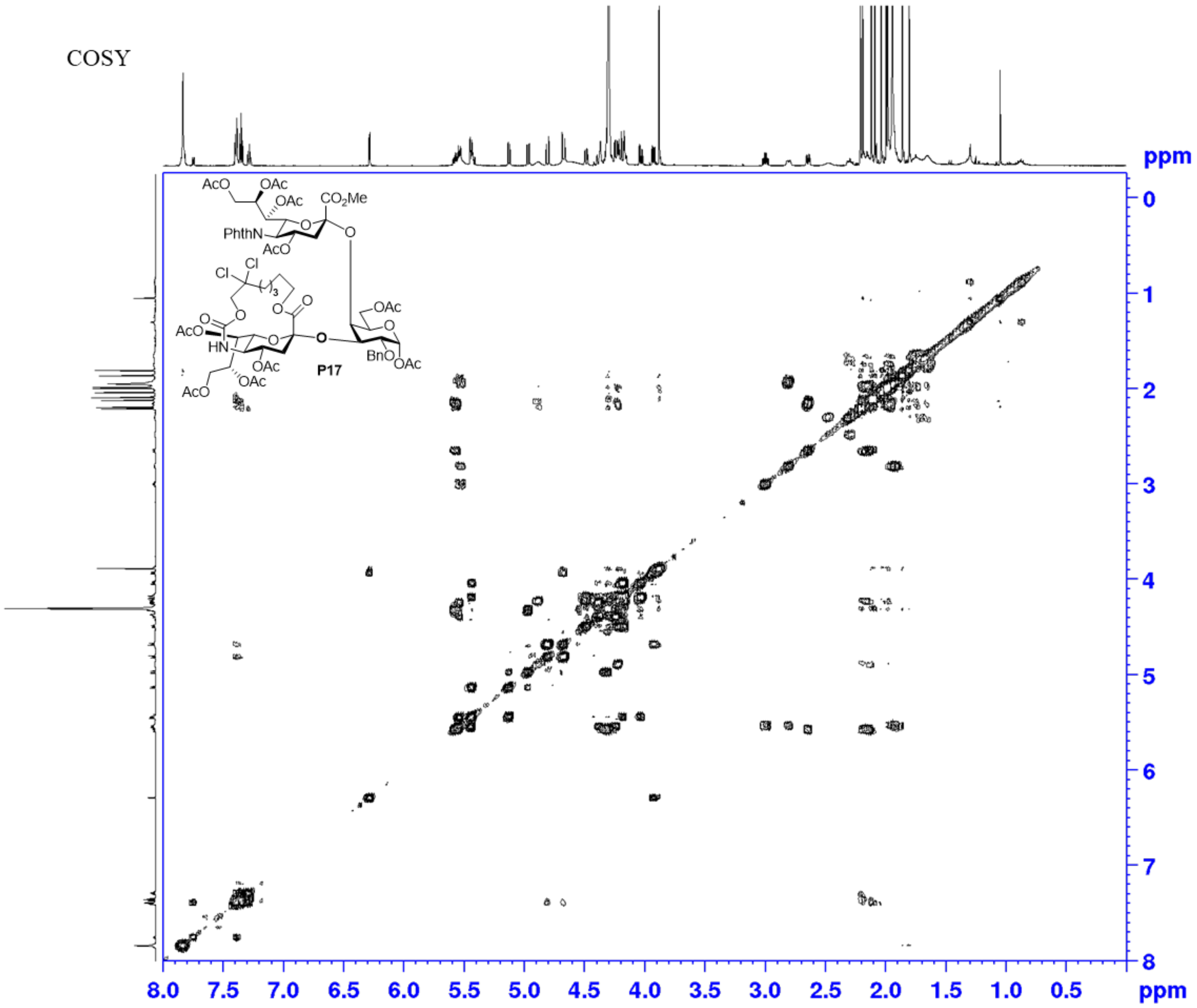


```
Current Data Parameters
NAME      KYT-256-major-HT
EXPNO                   11
PROCNO                   1

F2 - Acquisition Parameters
Date_             20200505
Time                  6.10
INSTRUM              spect
PROBHD    5 mm QNP   1H/1
PULPROG             cosygf
TD                    2048
SOLVENT             CH3NO2
NS                       8
DS                       0
SWH               6009.615 Hz
FIDRES            2.934382 Hz
AQ               0.1703936 sec
RG                      64
DW                  83.200 usec
DE                    6.50 usec
TE                   314.4 K
d0              0.00000300 sec
D1              1.50000000 sec
IN0             0.00016640 sec
MCREST                   0 sec
MCWRK           1.50000000 sec

======== CHANNEL f1 ========
NUC1                    1H
P0                   14.00 usec
P1                   29.00 usec
PL1                  -3.00 dB
SFO1           598.1028685 MHz

F1 - Acquisition parameters
TD                     300
SFO1              598.1029 MHz
FIDRES           40.064102 Hz
SW                  10.048 ppm
FnMODE                  QF

F2 - Processing parameters
SI                    2048
SF             598.1000213 MHz
WDW                  QSINE
SSB                      0
LB                       0 Hz
GB                       0
PC                    1.00

F1 - Processing parameters
SI                     512
MC2                     QF
SF             598.1000213 MHz
WDW                  QSINE
SSB                      0
LB                       0 Hz
GB                       0
```

HSQC

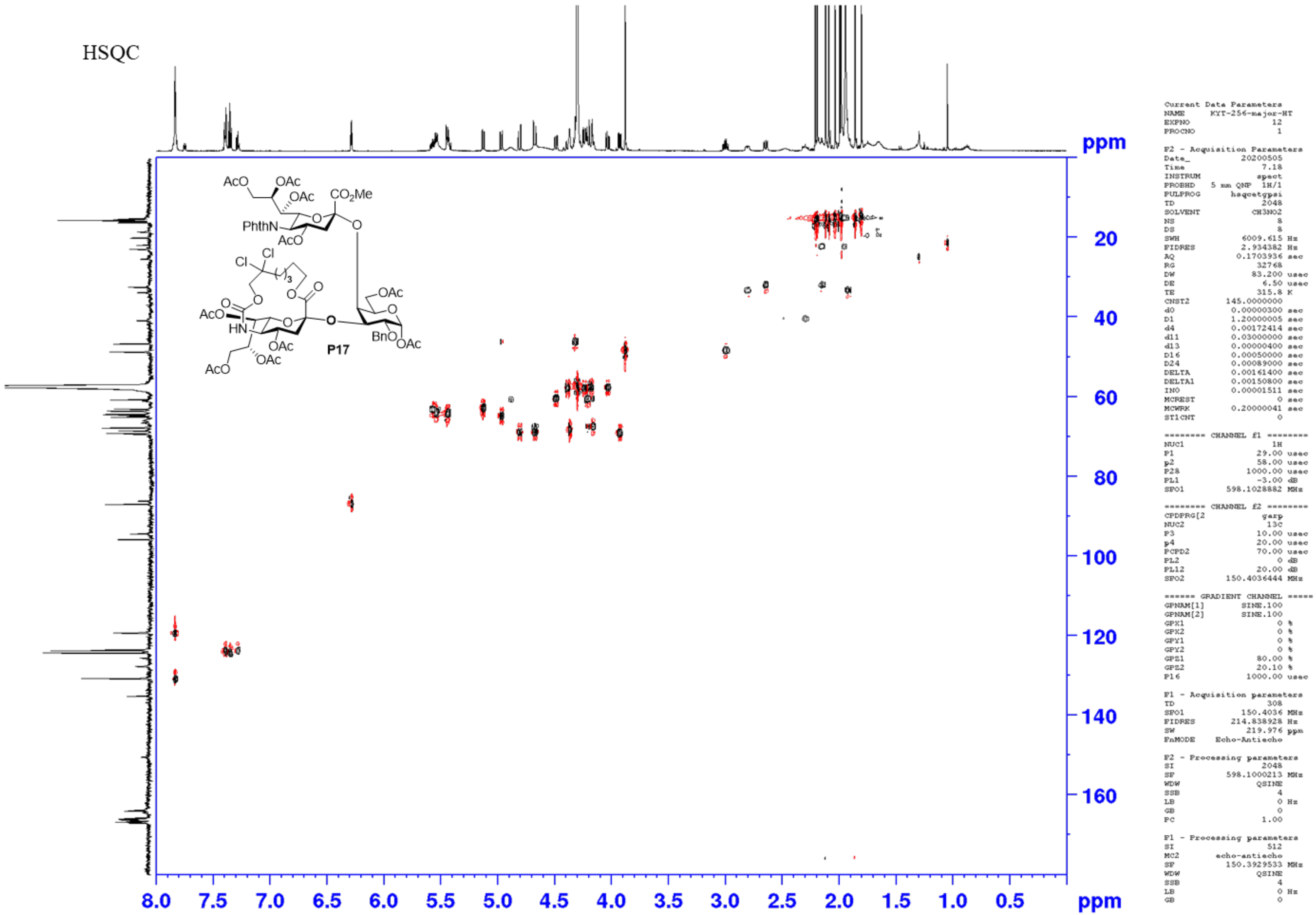
AcO
OAc
OAc
CO2Me
PhthN
O
O
AcO
Cl
Cl
3
O
O
O
OAc
AcO
O
HN
O
O
O
BnO
OAc
OAc
P17
OAc
AcO
ppm
20
40
60
80
100
120
140
160
8.0
7.5
7.0
6.5
6.0
5.5
5.0
4.5
4.0
3.5
3.0
2.5
2.0
1.5
1.0
0.5
ppm
Current Data Parameters
NAME KYT-256-major-HT
EXPNO 12
PROCNO 1
F2 - Acquisition Parameters
Date_ 20200505
Time 7.18
INSTRUM spect
PROBHD 5 mm QNP 1H/1
PULPROG hsqcetgpsi
TD 2048
SOLVENT CH3NO2
NS 8
DS 8
SWH 6009.615 Hz
FIDRES 2.934382 Hz
AQ 0.1703936 sec
RG 32768
DW 83.200 usec
DE 6.50 usec
TE 315.8 K
CNST2 145.0000000
d0 0.00000300 sec
D1 1.20000005 sec
d4 0.00172414 sec
d11 0.03000000 sec
d13 0.00000400 sec
D16 0.00050000 sec
D24 0.00089000 sec
DELTA 0.00161400 sec
DELTA1 0.00150800 sec
IN0 0.00001511 sec
MCREST 0 sec
MCWRK 0.20000041 sec
ST1CNT 0
======== CHANNEL f1 ========
NUC1 1H
P1 29.00 usec
p2 58.00 usec
P28 1000.00 usec
PL1 -3.00 dB
SFO1 598.1028882 MHz
======== CHANNEL f2 ========
CPDPRG[2 garp
NUC2 13C
P3 10.00 usec
p4 20.00 usec
PCPD2 70.00 usec
PL2 0 dB
PL12 20.00 dB
SFO2 150.4036444 MHz
====== GRADIENT CHANNEL =====
GPNAM[1] SINE.100
GPNAM[2] SINE.100
GPX1 0 %
GPX2 0 %
GPY1 0 %
GPY2 0 %
GPZ1 80.00 %
GPZ2 20.10 %
P16 1000.00 usec
F1 - Acquisition parameters
TD 308
SFO1 150.4036 MHz
FIDRES 214.838928 Hz
SW 219.976 ppm
FnMODE Echo-Antiecho
F2 - Processing parameters
SI 2048
SF 598.1000213 MHz
WDW QSINE
SSB 4
LB 0 Hz
GB 0
PC 1.00
F1 - Processing parameters
SI 512
MC2 echo-antiecho
SF 150.3929533 MHz
WDW QSINE
SSB 4
LB 0 Hz
GB 0

HMBC

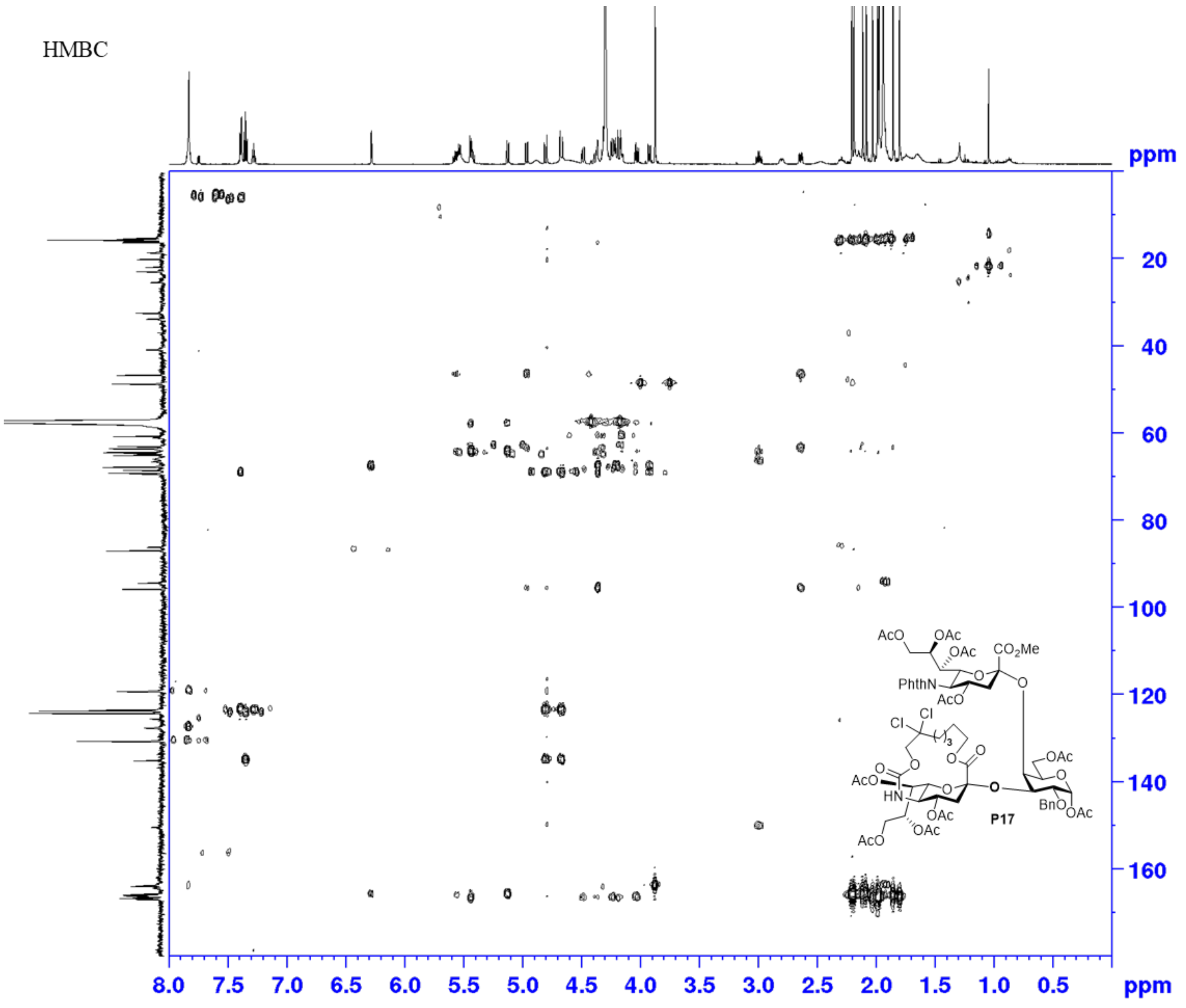


```
Current Data Parameters
NAME      KYT-256-major-HT
EXPNO                   13
PROCNO                   1

F2 - Acquisition Parameters
Date_             20200505
Time                  8.16
INSTRUM              spect
PROBHD    5 mm QNP  1H/1
PULPROG       hmbcgplpndqf
TD                    2048
SOLVENT             CH3NO2
NS                      16
DS                       0
SWH               6009.615 Hz
FIDRES            2.934382 Hz
AQ               0.1703936 sec
RG                   32768
DW                  83.200 usec
DE                    6.00 usec
TE                   314.7 K
CNST2          145.0000000
CNST13           8.0000000
d0              0.00000300 sec
D1              1.20000005 sec
d2              0.00344828 sec
d6              0.06250000 sec
D16             0.00050000 sec
IN0             0.00001583 sec
MCREST                   0 sec
MCWRK           1.20000005 sec

======== CHANNEL f1 ========
NUC1                    1H
P1                   29.00 usec
p2                   58.00 usec
PL1                  -3.00 dB
SFO1           598.1028882 MHz

======== CHANNEL f2 ========
NUC2                   13C
P3                   10.50 usec
PL2                      0 dB
SFO2           150.4059003 MHz

====== GRADIENT CHANNEL =====
GPNAM[1]          SINE.100
GPNAM[2]          SINE.100
GPNAM[3]          SINE.100
GPX1                     0 %
GPX2                     0 %
GPX3                     0 %
GPY1                     0 %
GPY2                     0 %
GPY3                     0 %
GPZ1                 50.00 %
GPZ2                 30.00 %
GPZ3                 40.10 %
P16                1000.00 usec

F1 - Acquisition parameters
TD                     262
SFO1              150.4059 MHz
FIDRES          241.187607 Hz
SW                 210.069 ppm
FnMODE                  QF

F2 - Processing parameters
SI                    4096
SF             598.1000213 MHz
WDW                  QSINE
SSB                      2
LB                       0 Hz
GB                       0
PC                    1.00

F1 - Processing parameters
SI                     512
MC2                     QF
SF             150.3929533 MHz
WDW                   SINE
SSB                      0
LB                       0 Hz
GB                       0
```

Current Data Parameters
NAME KYT-261-HT
EXPNO 1
PROCNO 1

F2 - Acquisition Parameters
Date_ 20200514
Time 5.00
INSTRUM spect
PROBHD 5 mm QNP 1H/1
PULPROG zg
TD 32768
SOLVENT CH3NO2
NS 128
DS 0
SWH 9541.984 Hz
FIDRES 0.291198 Hz
AQ 1.7170432 sec
RG 1024
DW 52.400 usec
DE 6.50 usec
TE 315.5 K
D1 2.00000000 sec
MCREST 0 sec
MCWRK 0.01500000 sec

======== CHANNEL f1 ========
NUC1 1H
P1 10.15 usec
PL1 -1.00 dB
SFO1 598.1036162 MHz

F2 - Processing parameters
SI 32768
SF 598.1000212 MHz
WDW no
SSB 0

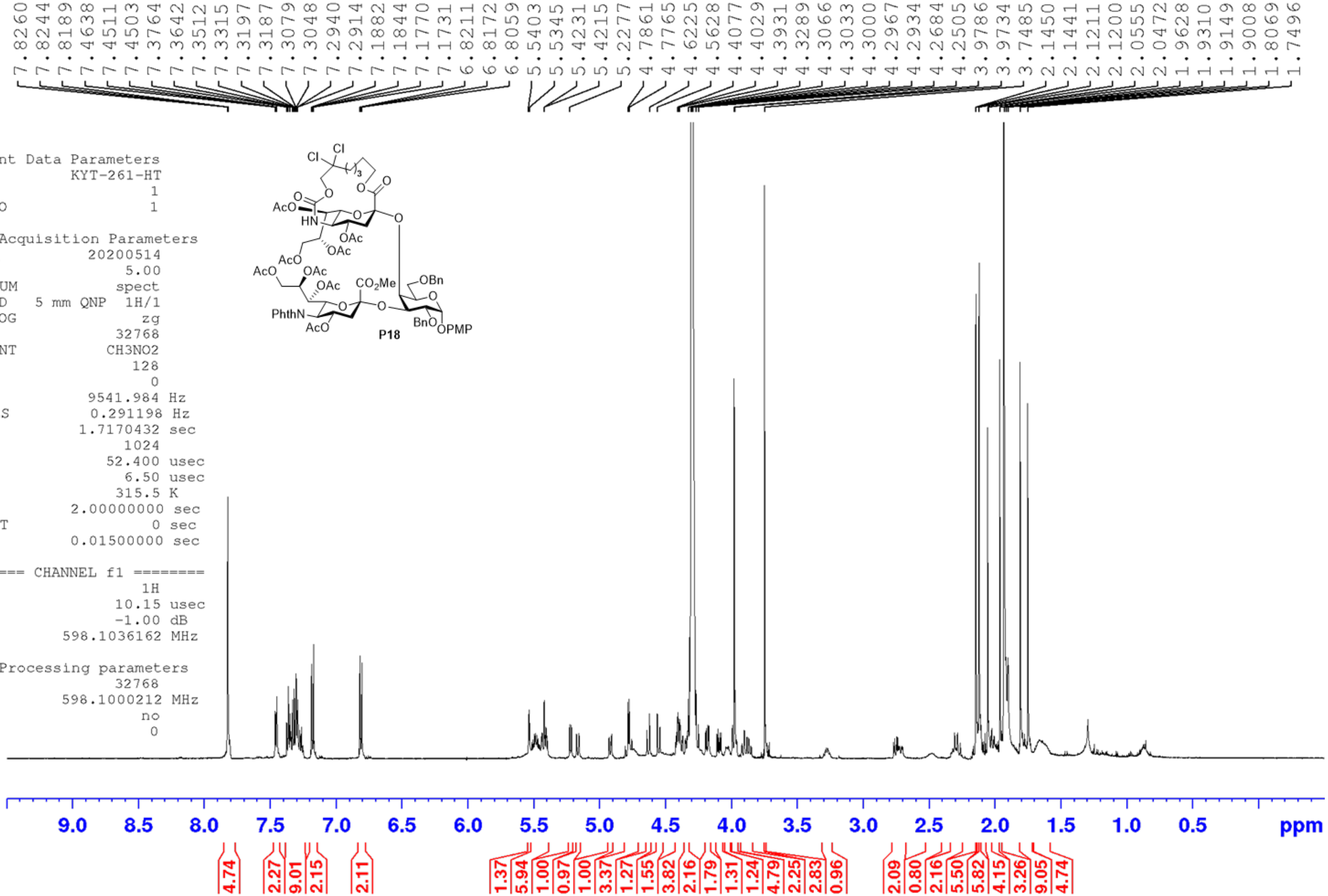

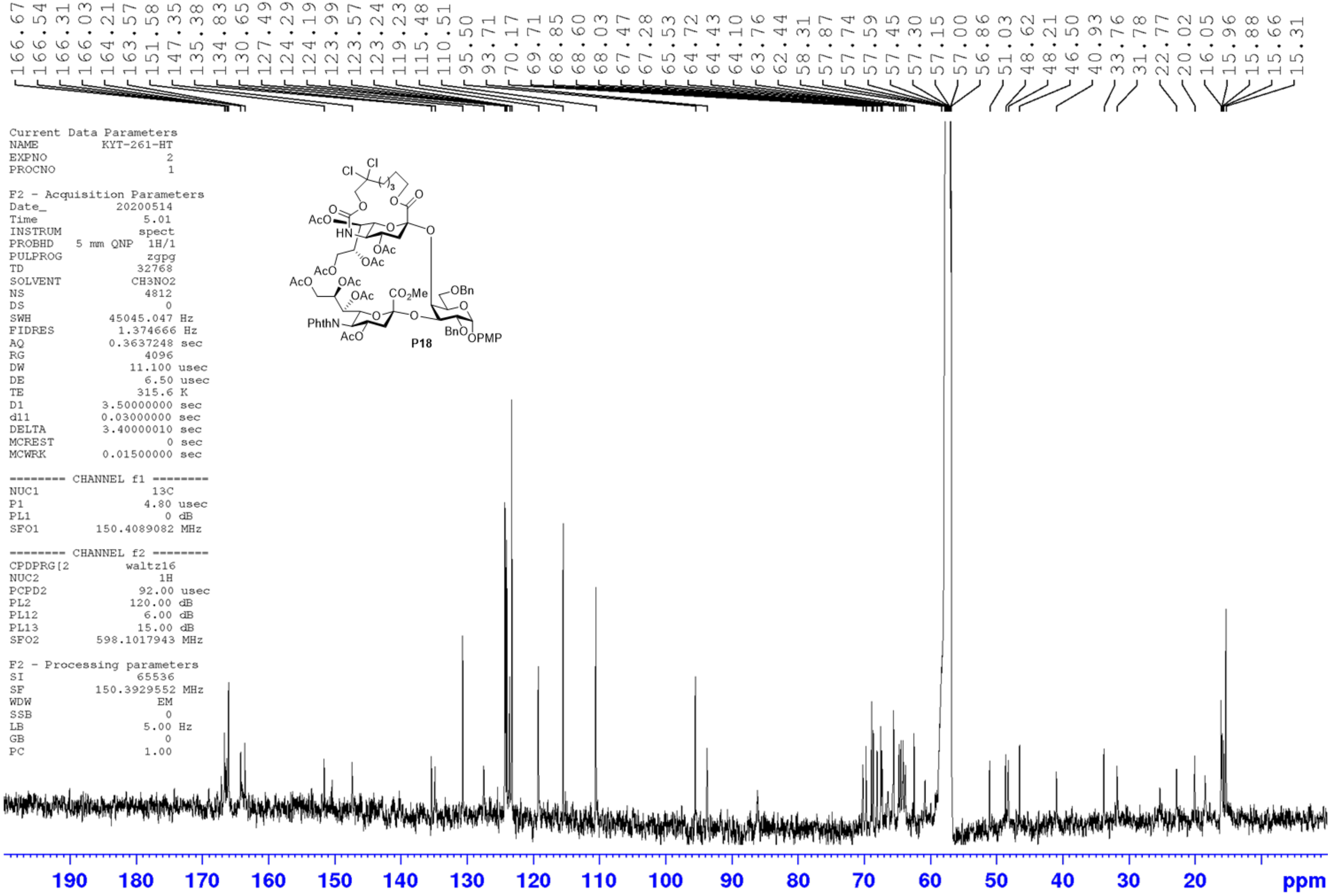
166.67
166.54
166.31
166.03
164.21
163.57
151.58
147.35
135.38
134.83
130.65
127.49
124.29
124.19
123.99
123.57
123.24
119.23
115.48
110.51
95.50
93.71
70.17
69.71
68.85
68.60
68.03
67.47
67.28
65.53
64.72
64.43
64.10
63.76
62.44
58.31
57.87
57.74
57.59
57.45
57.30
57.15
57.00
56.86
51.03
48.62
48.21
46.50
40.93
33.76
31.78
22.77
20.02
16.05
15.96
15.88
15.66
15.31
Current Data Parameters
NAME KYT-261-HT
EXPNO 2
PROCNO 1
F2 - Acquisition Parameters
Date_ 20200514
Time 5.01
INSTRUM spect
PROBHD 5 mm QNP 1H/1
PULPROG zgpg
TD 32768
SOLVENT CH3NO2
NS 4812
DS 0
SWH 45045.047 Hz
FIDRES 1.374666 Hz
AQ 0.3637248 sec
RG 4096
DW 11.100 usec
DE 6.50 usec
TE 315.6 K
D1 3.50000000 sec
d11 0.03000000 sec
DELTA 3.40000010 sec
MCREST 0 sec
MCWRK 0.01500000 sec
======== CHANNEL f1 ========
NUC1 13C
P1 4.80 usec
PL1 0 dB
SFO1 150.4089082 MHz
======== CHANNEL f2 ========
CPDPRG[2 waltz16
NUC2 1H
PCPD2 92.00 usec
PL2 120.00 dB
PL12 6.00 dB
PL13 15.00 dB
SFO2 598.1017943 MHz
F2 - Processing parameters
SI 65536
SF 150.3929552 MHz
WDW EM
SSB 0
LB 5.00 Hz
GB 0
PC 1.00
Cl
Cl
3
O
O
O
O
AcO
HN
O
O
OAc
OAc
AcO
AcO
OAc
OAc
CO2Me
OBn
O
O
O
PhthN
AcO
BnO
OPMP
P18
190
180
170
160
150
140
130
120
110
100
90
80
70
60
50
40
30
20
ppm

COSY

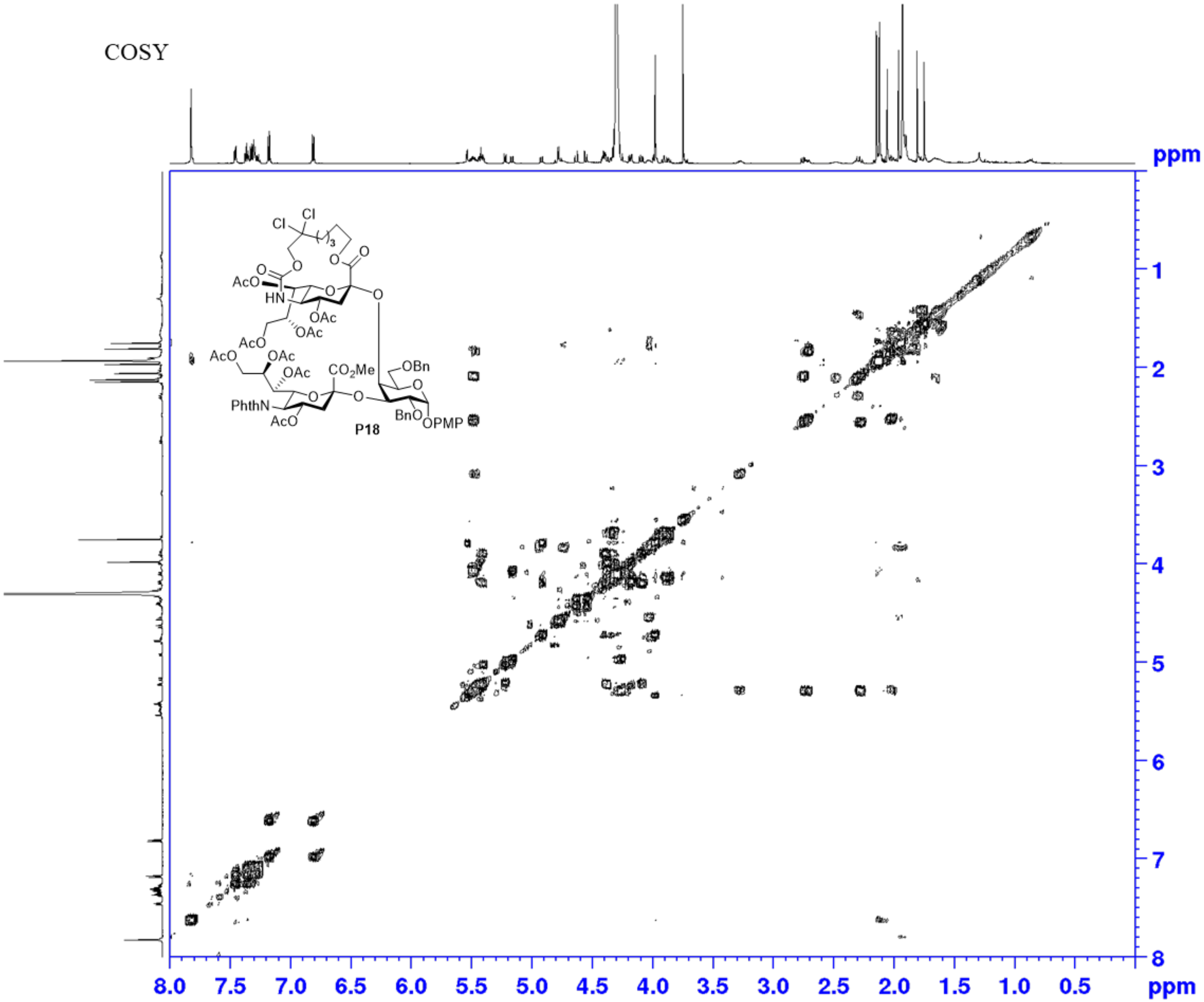


```
Current Data Parameters
NAME          KYT-261-HT
EXPNO                 11
PROCNO                 1

F2 - Acquisition Parameters
Date_           20200518
Time                5.39
INSTRUM            spect
PROBHD     5 mm QNP  1H/1
PULPROG           cosygf
TD                  2048
SOLVENT           CH3NO2
NS                     8
DS                     0
SWH             6009.615 Hz
FIDRES          2.934382 Hz
AQ             0.1703936 sec
RG                   128
DW                83.200 usec
DE                  6.50 usec
TE                 318.4 K
d0            0.00000300 sec
D1            1.50000000 sec
IN0           0.00016640 sec
MCREST                 0 sec
MCWRK         1.50000000 sec

======== CHANNEL f1 ========
NUC1                  1H
P0                 14.00 usec
P1                 29.00 usec
PL1                -3.00 dB
SFO1         598.1029905 MHz

F1 - Acquisition parameters
TD                   256
SFO1            598.1029 MHz
FIDRES         46.950119 Hz
SW                10.048 ppm
FnMODE                QF

F2 - Processing parameters
SI                  2048
SF           598.1000212 MHz
WDW                QSINE
SSB                    0
LB                     0 Hz
GB                     0
PC                  1.00

F1 - Processing parameters
SI                   512
MC2                   QF
SF           598.1000212 MHz
WDW                QSINE
SSB                    0
LB                     0 Hz
GB                     0
```

HSQC

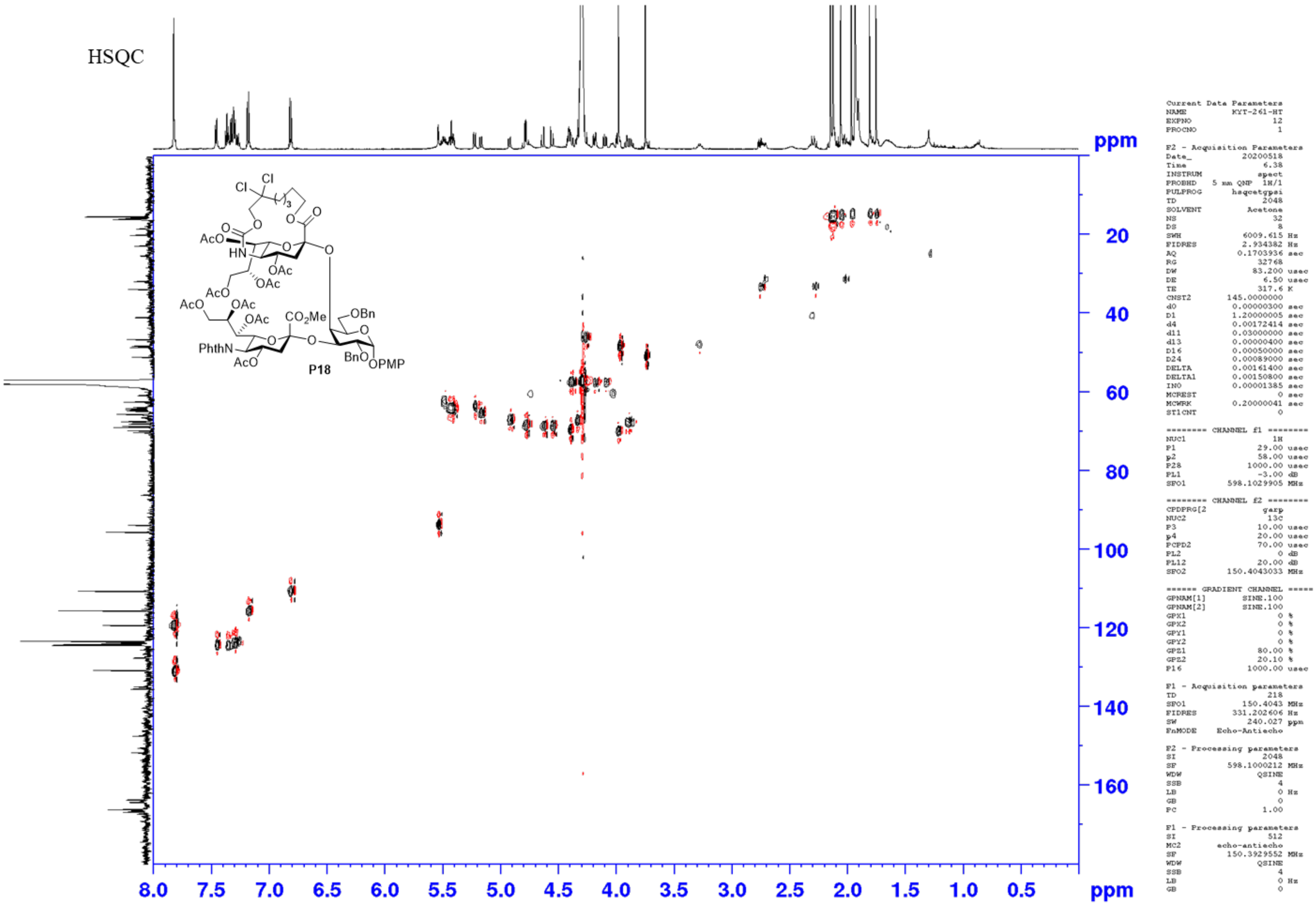

P18
ppm
20
40
60
80
100
120
140
160
8.0
7.5
7.0
6.5
6.0
5.5
5.0
4.5
4.0
3.5
3.0
2.5
2.0
1.5
1.0
0.5
ppm
Current Data Parameters
NAME KYT-261-HT
EXPNO 12
PROCNO 1
F2 - Acquisition Parameters
Date_ 20200518
Time 6.38
INSTRUM spect
PROBHD 5 mm QNP 1H/1
PULPROG hsqcetgpsi
TD 2048
SOLVENT Acetone
NS 32
DS 8
SWH 6009.615 Hz
FIDRES 2.934382 Hz
AQ 0.1703936 sec
RG 32768
DW 83.200 usec
DE 6.50 usec
TE 317.6 K
CNST2 145.0000000
d0 0.00000300 sec
D1 1.20000005 sec
d4 0.00172414 sec
d11 0.03000000 sec
d13 0.00000400 sec
D16 0.00050000 sec
D24 0.00089000 sec
DELTA 0.00161400 sec
DELTA1 0.00150800 sec
IN0 0.00001385 sec
MCREST 0 sec
MCWRK 0.20000041 sec
ST1CNT 0
======== CHANNEL f1 ========
NUC1 1H
P1 29.00 usec
p2 58.00 usec
P28 1000.00 usec
PL1 -3.00 dB
SFO1 598.1029905 MHz
======== CHANNEL f2 ========
CPDPRG[2 garp
NUC2 13C
P3 10.00 usec
p4 20.00 usec
PCPD2 70.00 usec
PL2 0 dB
PL12 20.00 dB
SFO2 150.4043033 MHz
====== GRADIENT CHANNEL =====
GPNAM[1] SINE.100
GPNAM[2] SINE.100
GPX1 0 %
GPX2 0 %
GPY1 0 %
GPY2 0 %
GPZ1 80.00 %
GPZ2 20.10 %
P16 1000.00 usec
F1 - Acquisition parameters
TD 218
SFO1 150.4043 MHz
FIDRES 331.202606 Hz
SW 240.027 ppm
FnMODE Echo-Antiecho
F2 - Processing parameters
SI 2048
SF 598.1000212 MHz
WDW QSINE
SSB 4
LB 0 Hz
GB 0
PC 1.00
F1 - Processing parameters
SI 512
MC2 echo-antiecho
SF 150.3929552 MHz
WDW QSINE
SSB 4
LB 0 Hz
GB 0

HMBC

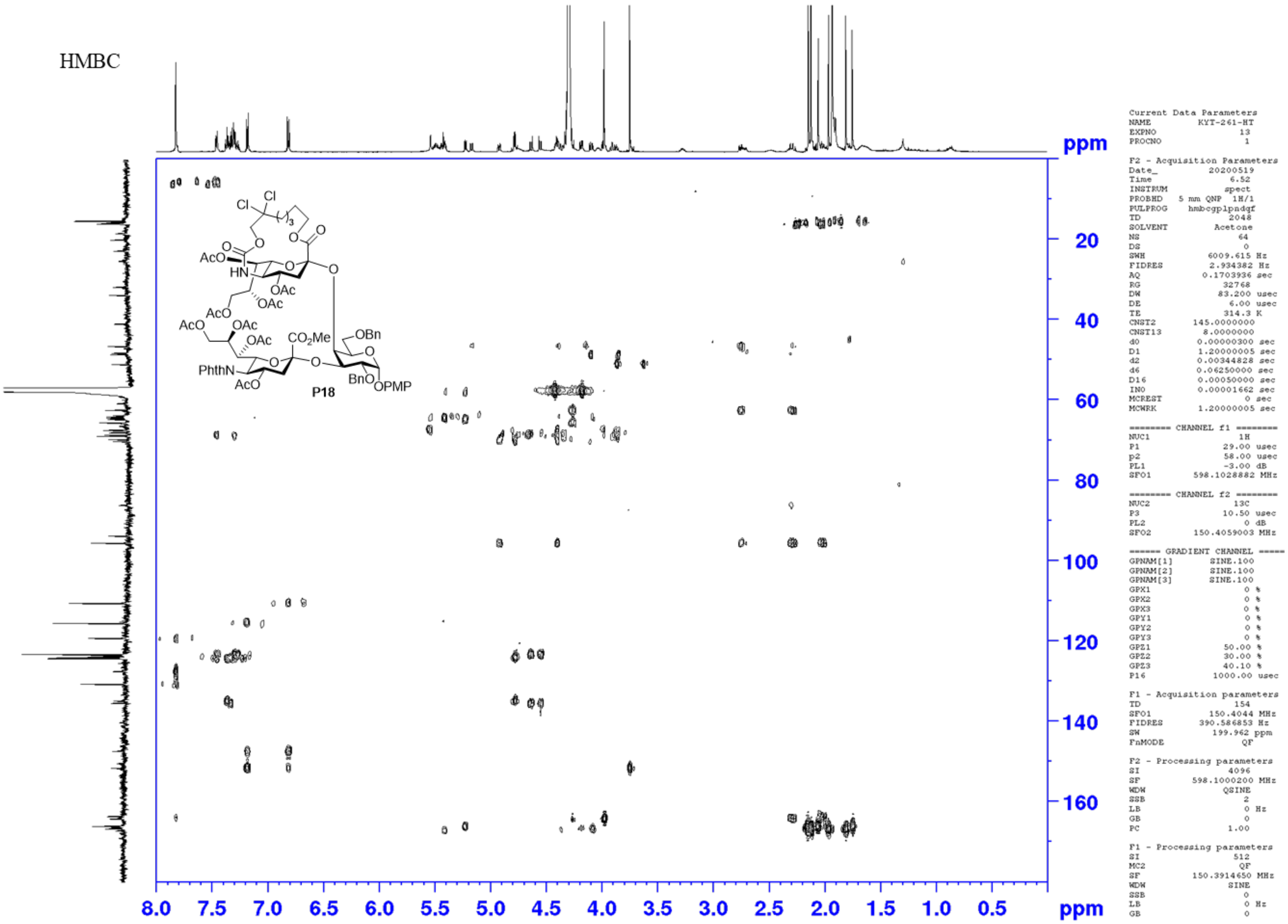
P18
ppm
8.0 7.5 7.0 6.5 6.0 5.5 5.0 4.5 4.0 3.5 3.0 2.5 2.0 1.5 1.0 0.5
20 40 60 80 100 120 140 160
Current Data Parameters
NAME KYT-261-HT
EXPNO 13
PROCNO 1
F2 - Acquisition Parameters
Date_ 20200519
Time 6.52
INSTRUM spect
PROBHD 5 mm QNP 1H/1
PULPROG hmbcgplpndqf
TD 2048
SOLVENT Acetone
NS 64
DS 0
SWH 6009.615 Hz
FIDRES 2.934382 Hz
AQ 0.1703936 sec
RG 32768
DW 83.200 usec
DE 6.00 usec
TE 314.3 K
CNST2 145.0000000
CNST13 8.0000000
d0 0.00000300 sec
D1 1.20000005 sec
d2 0.00344828 sec
d6 0.06250000 sec
D16 0.00050000 sec
IN0 0.00001662 sec
MCREST 0 sec
MCWRK 1.20000005 sec
======== CHANNEL f1 ========
NUC1 1H
P1 29.00 usec
p2 58.00 usec
PL1 -3.00 dB
SFO1 598.1028882 MHz
======== CHANNEL f2 ========
NUC2 13C
P3 10.50 usec
PL2 0 dB
SFO2 150.4059003 MHz
====== GRADIENT CHANNEL =====
GPNAM[1] SINE.100
GPNAM[2] SINE.100
GPNAM[3] SINE.100
GPX1 0 %
GPX2 0 %
GPX3 0 %
GPY1 0 %
GPY2 0 %
GPY3 0 %
GPZ1 50.00 %
GPZ2 30.00 %
GPZ3 40.10 %
P16 1000.00 usec
F1 - Acquisition parameters
TD 154
SFO1 150.4044 MHz
FIDRES 390.586853 Hz
SW 199.962 ppm
FnMODE QF
F2 - Processing parameters
SI 4096
SF 598.1000200 MHz
WDW QSINE
SSB 2
LB 0 Hz
GB 0
PC 1.00
F1 - Processing parameters
SI 512
MC2 QF
SF 150.3914650 MHz
WDW SINE
SSB 0
LB 0 Hz
GB 0

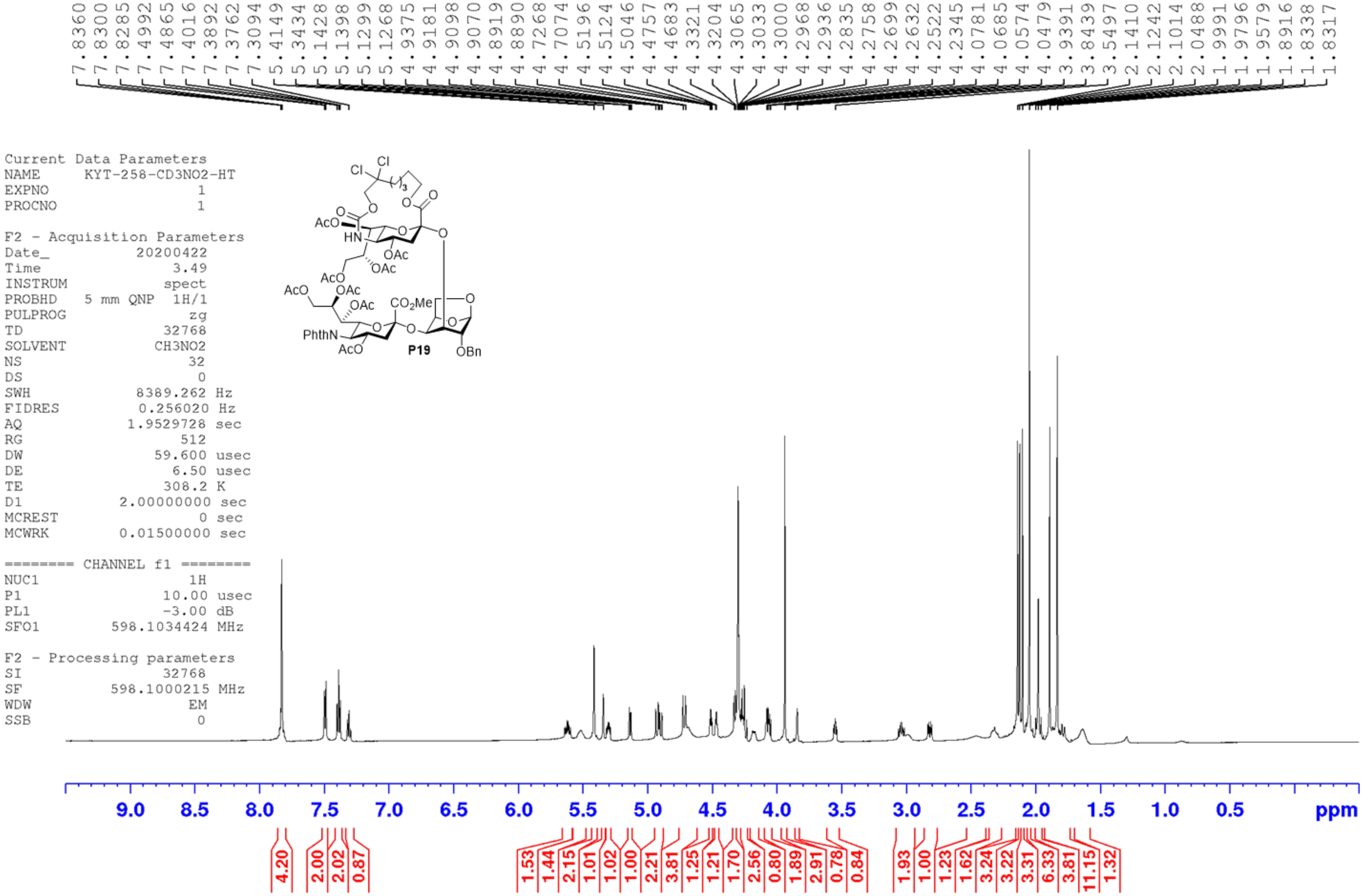

Current Data Parameters
NAME KYT-258-CD3NO2-HT
EXPNO 1
PROCNO 1
F2 - Acquisition Parameters
Date_ 20200422
Time 3.49
INSTRUM spect
PROBHD 5 mm QNP 1H/1
PULPROG zg
TD 32768
SOLVENT CH3NO2
NS 32
DS 0
SWH 8389.262 Hz
FIDRES 0.256020 Hz
AQ 1.9529728 sec
RG 512
DW 59.600 usec
DE 6.50 usec
TE 308.2 K
D1 2.00000000 sec
MCREST 0 sec
MCWRK 0.01500000 sec
======== CHANNEL f1 ========
NUC1 1H
P1 10.00 usec
PL1 -3.00 dB
SFO1 598.1034424 MHz
F2 - Processing parameters
SI 32768
SF 598.1000215 MHz
WDW EM
SSB 0
Cl
Cl
O
O
O
AcO
HN
O
O
OAc
OAc
AcO
AcO
OAc
OAc
CO2Me
O
O
PhthN
O
O
AcO
P19
OBn
7.8360
7.8300
7.8285
7.4992
7.4865
7.4016
7.3892
7.3762
7.3094
5.4149
5.3434
5.1428
5.1398
5.1299
5.1268
4.9375
4.9181
4.9098
4.9070
4.8919
4.8890
4.7268
4.7074
4.5196
4.5124
4.5046
4.4757
4.4683
4.3321
4.3204
4.3065
4.3033
4.3000
4.2968
4.2936
4.2835
4.2758
4.2699
4.2632
4.2522
4.2345
4.0781
4.0685
4.0574
4.0479
3.9391
3.8439
3.5497
2.1410
2.1242
2.1014
2.0488
1.9991
1.9796
1.9579
1.8916
1.8338
1.8317
9.0
8.5
8.0
7.5
7.0
6.5
6.0
5.5
5.0
4.5
4.0
3.5
3.0
2.5
2.0
1.5
1.0
0.5
ppm
4.20
2.00
2.02
0.87
1.53
1.44
2.15
1.01
1.02
1.00
2.21
3.81
1.25
1.21
1.70
2.56
0.80
1.89
2.91
0.78
0.84
1.93
1.00
1.23
1.62
3.24
3.22
3.31
6.33
3.81
11.15
1.32

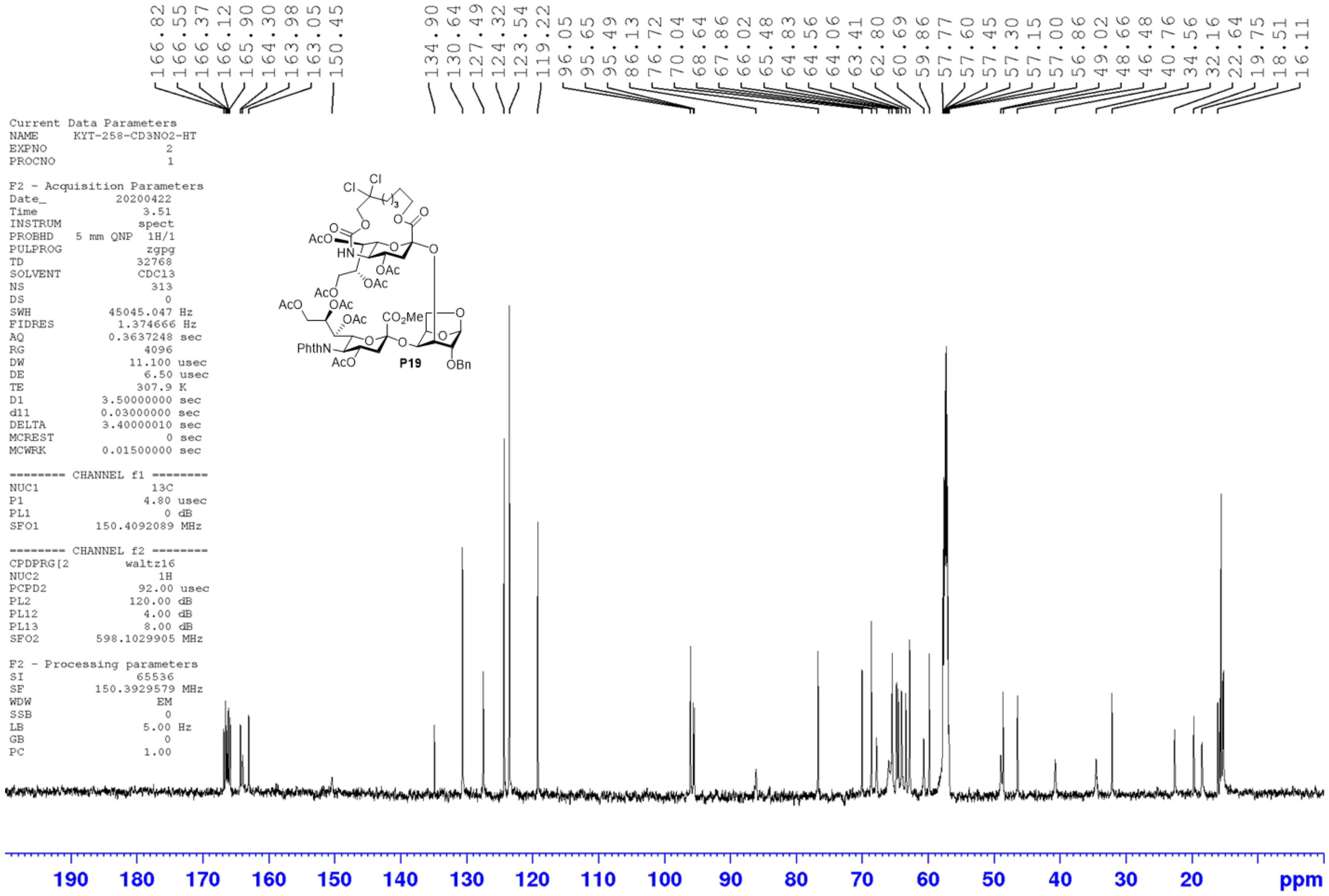
166.82
166.55
166.37
166.12
165.90
164.30
163.98
163.05
150.45
134.90
130.64
127.49
124.32
123.54
119.22
96.05
95.65
95.49
86.13
76.72
70.04
68.64
67.86
66.02
65.48
64.83
64.56
64.06
63.41
62.80
60.69
59.86
57.77
57.60
57.45
57.30
57.15
57.00
56.86
49.02
48.66
46.48
40.76
34.56
32.16
22.64
19.75
18.51
16.11
Current Data Parameters
NAME KYT-258-CD3NO2-HT
EXPNO 2
PROCNO 1
F2 - Acquisition Parameters
Date_ 20200422
Time 3.51
INSTRUM spect
PROBHD 5 mm QNP 1H/1
PULPROG zgpg
TD 32768
SOLVENT CDCl3
NS 313
DS 0
SWH 45045.047 Hz
FIDRES 1.374666 Hz
AQ 0.3637248 sec
RG 4096
DW 11.100 usec
DE 6.50 usec
TE 307.9 K
D1 3.50000000 sec
d11 0.03000000 sec
DELTA 3.40000010 sec
MCREST 0 sec
MCWRK 0.01500000 sec
======== CHANNEL f1 ========
NUC1 13C
P1 4.80 usec
PL1 0 dB
SFO1 150.4092089 MHz
======== CHANNEL f2 ========
CPDPRG[2 waltz16
NUC2 1H
PCPD2 92.00 usec
PL2 120.00 dB
PL12 4.00 dB
PL13 8.00 dB
SFO2 598.1029905 MHz
F2 - Processing parameters
SI 65536
SF 150.3929579 MHz
WDW EM
SSB 0
LB 5.00 Hz
GB 0
PC 1.00
Cl
Cl
AcO
HN
OAc
OAc
AcO
AcO
OAc
OAc
CO_2Me
PhthN
AcO
P19
OBn
190 180 170 160 150 140 130 120 110 100 90 80 70 60 50 40 30 20 ppm

COSY

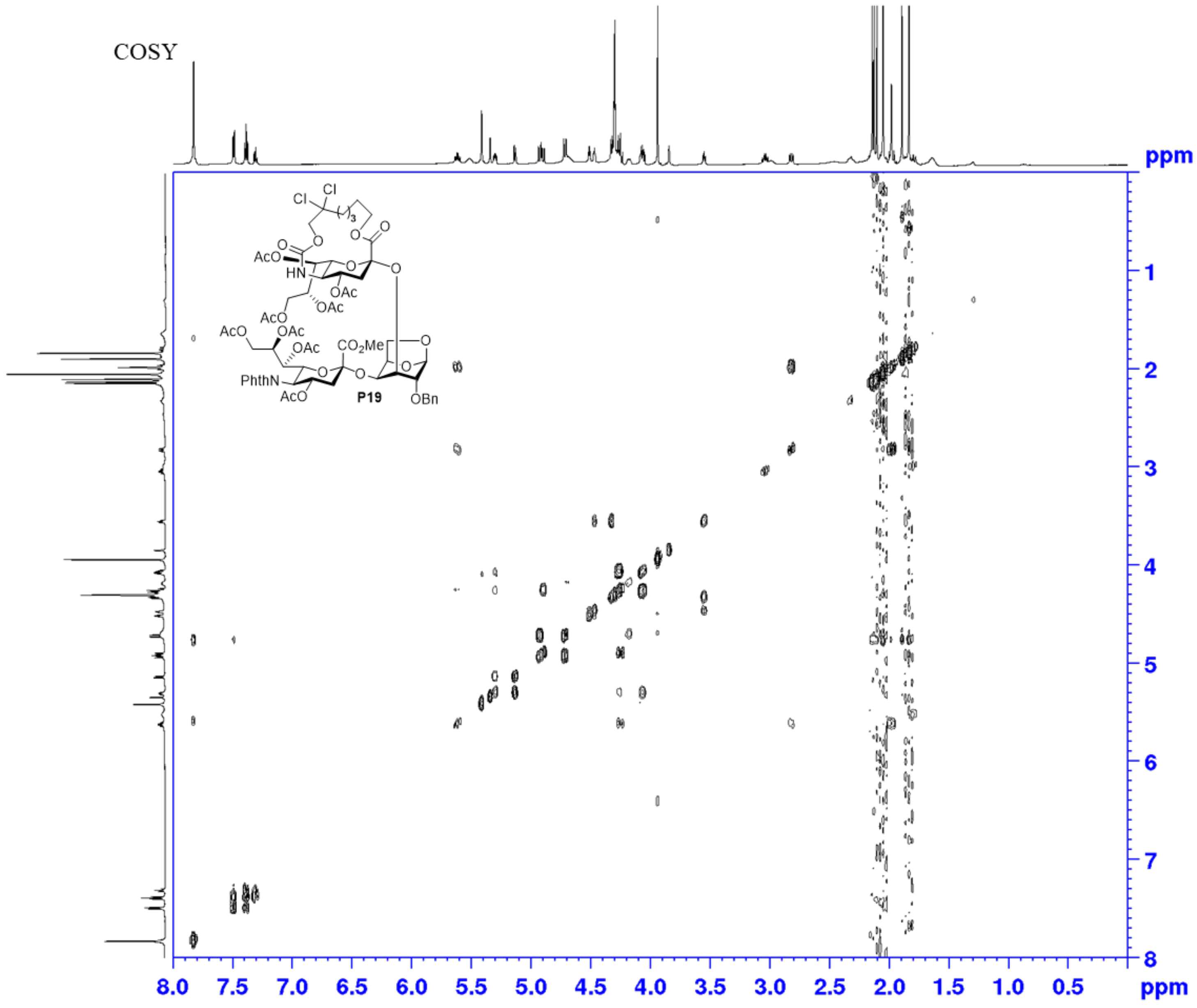


```
Current Data Parameters
NAME       KYT-258-CD3NO2-HT
EXPNO                     11
PROCNO                     1

F2 - Acquisition Parameters
Date_               20200422
Time                    7.05
INSTRUM                spect
PROBHD     5 mm QNP  1H/1
PULPROG               cosyqf
TD                      2048
SOLVENT               CH3NO2
NS                        16
DS                         0
SWH                 6009.615 Hz
FIDRES              2.934382 Hz
AQ                 0.1703936 sec
RG                        32
DW                    83.200 usec
DE                      6.50 usec
TE                     309.2 K
d0                0.00000300 sec
D1                1.50000000 sec
IN0               0.00016640 sec
MCREST                     0 sec
MCWRK             1.50000000 sec

======== CHANNEL f1 ========
NUC1                      1H
P0                     14.00 usec
P1                     40.00 usec
PL1                    -3.00 dB
SFO1             598.1028685 MHz

F1 - Acquisition parameters
TD                       256
SFO1                598.1029 MHz
FIDRES             46.950119 Hz
SW                    10.048 ppm
FnMODE                    QF

F2 - Processing parameters
SI                      2048
SF               598.1000215 MHz
WDW                    QSINE
SSB                        0
LB                         0 Hz
GB                         0
PC                      1.00

F1 - Processing parameters
SI                       512
MC2                       QF
SF               598.1000215 MHz
WDW                    QSINE
SSB                        0
LB                         0 Hz
GB                         0
```

HSQC

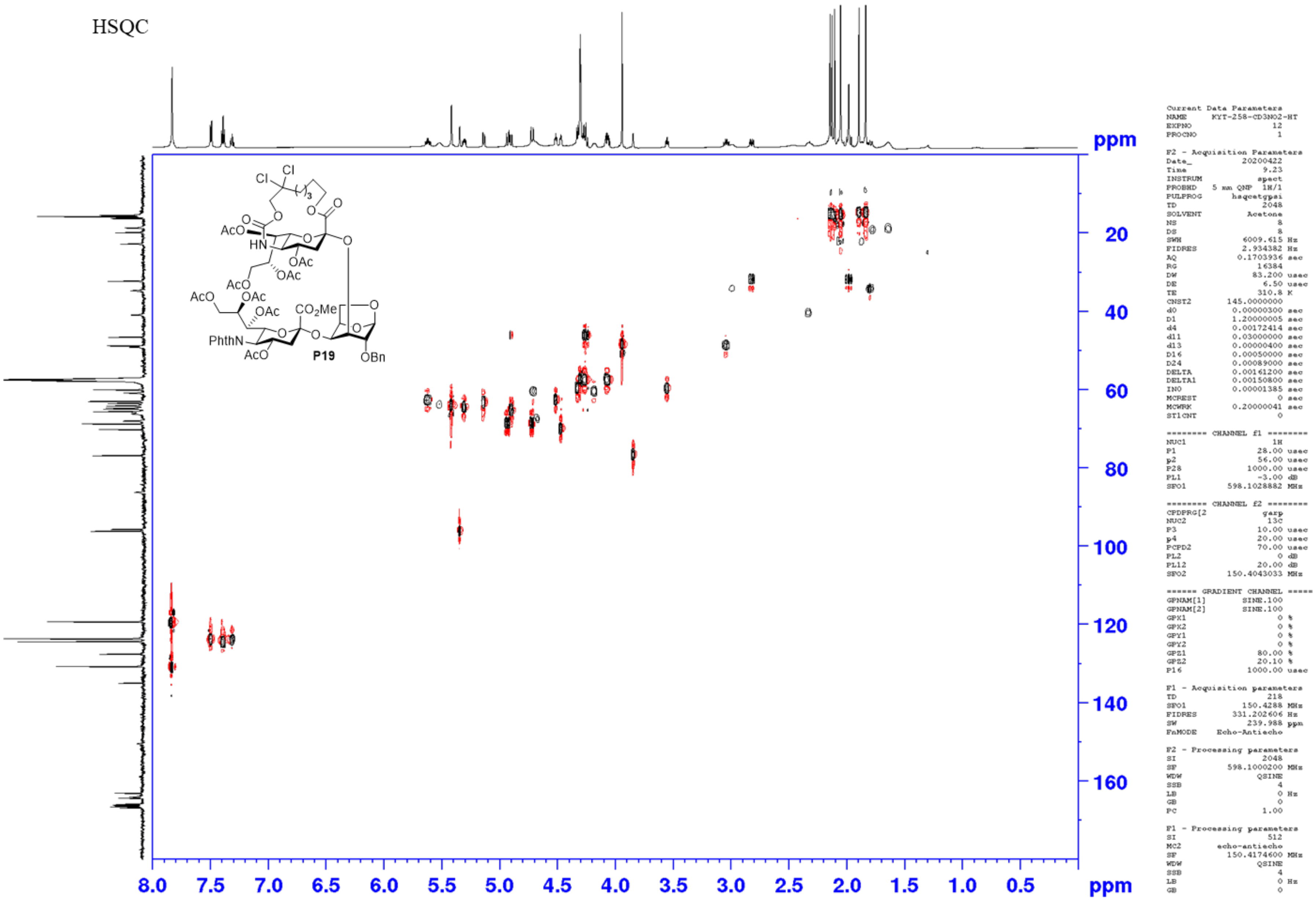

Cl
Cl
3
O
O
O
AcO
HN
O
O
OAc
OAc
AcO
AcO
OAc
OAc
CO2Me
O
PhthN
O
O
O
AcO
P19
OBn
ppm
20
40
60
80
100
120
140
160
8.0
7.5
7.0
6.5
6.0
5.5
5.0
4.5
4.0
3.5
3.0
2.5
2.0
1.5
1.0
0.5
ppm
Current Data Parameters
NAME KYT-258-CD3NO2-HT
EXPNO 12
PROCNO 1
F2 - Acquisition Parameters
Date_ 20200422
Time 9.23
INSTRUM spect
PROBHD 5 mm QNP 1H/1
PULPROG hsqcetgpsi
TD 2048
SOLVENT Acetone
NS 8
DS 8
SWH 6009.615 Hz
FIDRES 2.934382 Hz
AQ 0.1703936 sec
RG 16384
DW 83.200 usec
DE 6.50 usec
TE 310.8 K
CNST2 145.0000000
d0 0.00000300 sec
D1 1.20000005 sec
d4 0.00172414 sec
d11 0.03000000 sec
d13 0.00000400 sec
D16 0.00050000 sec
D24 0.00089000 sec
DELTA 0.00161200 sec
DELTA1 0.00150800 sec
IN0 0.00001385 sec
MCREST 0 sec
MCWRK 0.20000041 sec
ST1CNT 0
======== CHANNEL f1 ========
NUC1 1H
P1 28.00 usec
p2 56.00 usec
P28 1000.00 usec
PL1 -3.00 dB
SFO1 598.1028882 MHz
======== CHANNEL f2 ========
CPDPRG[2 garp
NUC2 13C
P3 10.00 usec
p4 20.00 usec
PCPD2 70.00 usec
PL2 0 dB
PL12 20.00 dB
SFO2 150.4043033 MHz
====== GRADIENT CHANNEL =====
GPNAM[1] SINE.100
GPNAM[2] SINE.100
GPX1 0 %
GPX2 0 %
GPY1 0 %
GPY2 0 %
GPZ1 80.00 %
GPZ2 20.10 %
P16 1000.00 usec
F1 - Acquisition parameters
TD 218
SFO1 150.4288 MHz
FIDRES 331.202606 Hz
SW 239.988 ppm
FnMODE Echo-Antiecho
F2 - Processing parameters
SI 2048
SF 598.1000200 MHz
WDW QSINE
SSB 4
LB 0 Hz
GB 0
PC 1.00
F1 - Processing parameters
SI 512
MC2 echo-antiecho
SF 150.4174600 MHz
WDW QSINE
SSB 4
LB 0 Hz
GB 0

HMBC

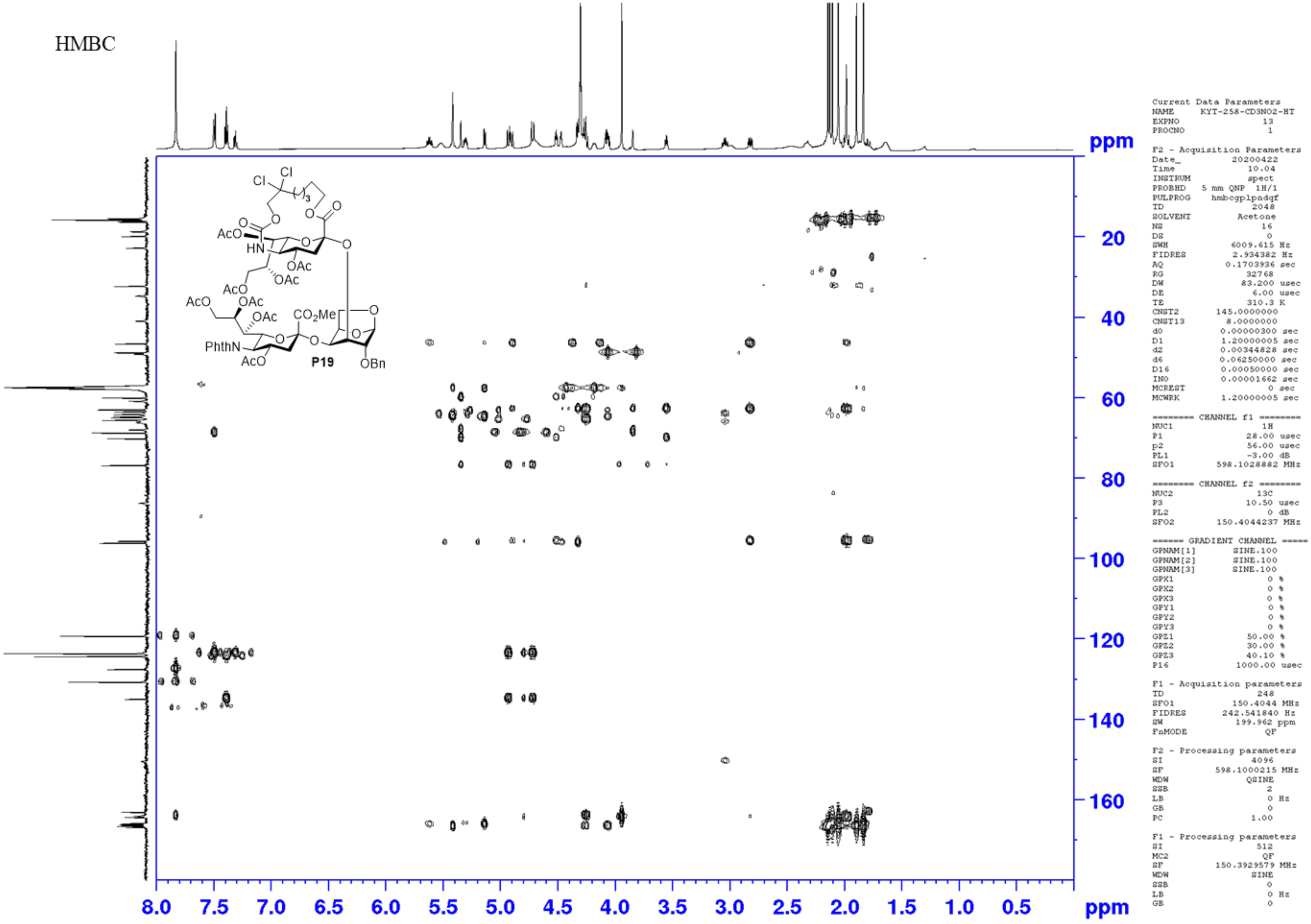
Cl
Cl
3
AcO
HN
O
OAc
OAc
AcO
AcO
OAc
OAc
CO2Me
PhthN
AcO
P19
OBn
ppm
20
40
60
80
100
120
140
160
8.0
7.5
7.0
6.5
6.0
5.5
5.0
4.5
4.0
3.5
3.0
2.5
2.0
1.5
1.0
0.5
ppm
Current Data Parameters
NAME KYT-258-CD3NO2-HT
EXPNO 13
PROCNO 1
F2 - Acquisition Parameters
Date_ 20200422
Time 10.04
INSTRUM spect
PROBHD 5 mm QNP 1H/1
PULPROG hmbcgplpndqf
TD 2048
SOLVENT Acetone
NS 16
DS 0
SWH 6009.615 Hz
FIDRES 2.934382 Hz
AQ 0.1703936 sec
RG 32768
DW 83.200 usec
DE 6.00 usec
TE 310.3 K
CNST2 145.0000000
CNST13 8.0000000
d0 0.00000300 sec
D1 1.20000005 sec
d2 0.00344828 sec
d6 0.06250000 sec
D16 0.00050000 sec
IN0 0.00001662 sec
MCREST 0 sec
MCWRK 1.20000005 sec
======== CHANNEL f1 ========
NUC1 1H
P1 28.00 usec
p2 56.00 usec
PL1 -3.00 dB
SFO1 598.1028882 MHz
======== CHANNEL f2 ========
NUC2 13C
P3 10.50 usec
PL2 0 dB
SFO2 150.4044237 MHz
====== GRADIENT CHANNEL =====
GPNAM[1] SINE.100
GPNAM[2] SINE.100
GPNAM[3] SINE.100
GPX1 0 %
GPX2 0 %
GPX3 0 %
GPY1 0 %
GPY2 0 %
GPY3 0 %
GPZ1 50.00 %
GPZ2 30.00 %
GPZ3 40.10 %
P16 1000.00 usec
F1 - Acquisition parameters
TD 248
SFO1 150.4044 MHz
FIDRES 242.541840 Hz
SW 199.962 ppm
FnMODE QF
F2 - Processing parameters
SI 4096
SF 598.1000215 MHz
WDW QSINE
SSB 2
LB 0 Hz
GB 0
PC 1.00
F1 - Processing parameters
SI 512
MC2 QF
SF 150.3929579 MHz
WDW SINE
SSB 0
LB 0 Hz
GB 0

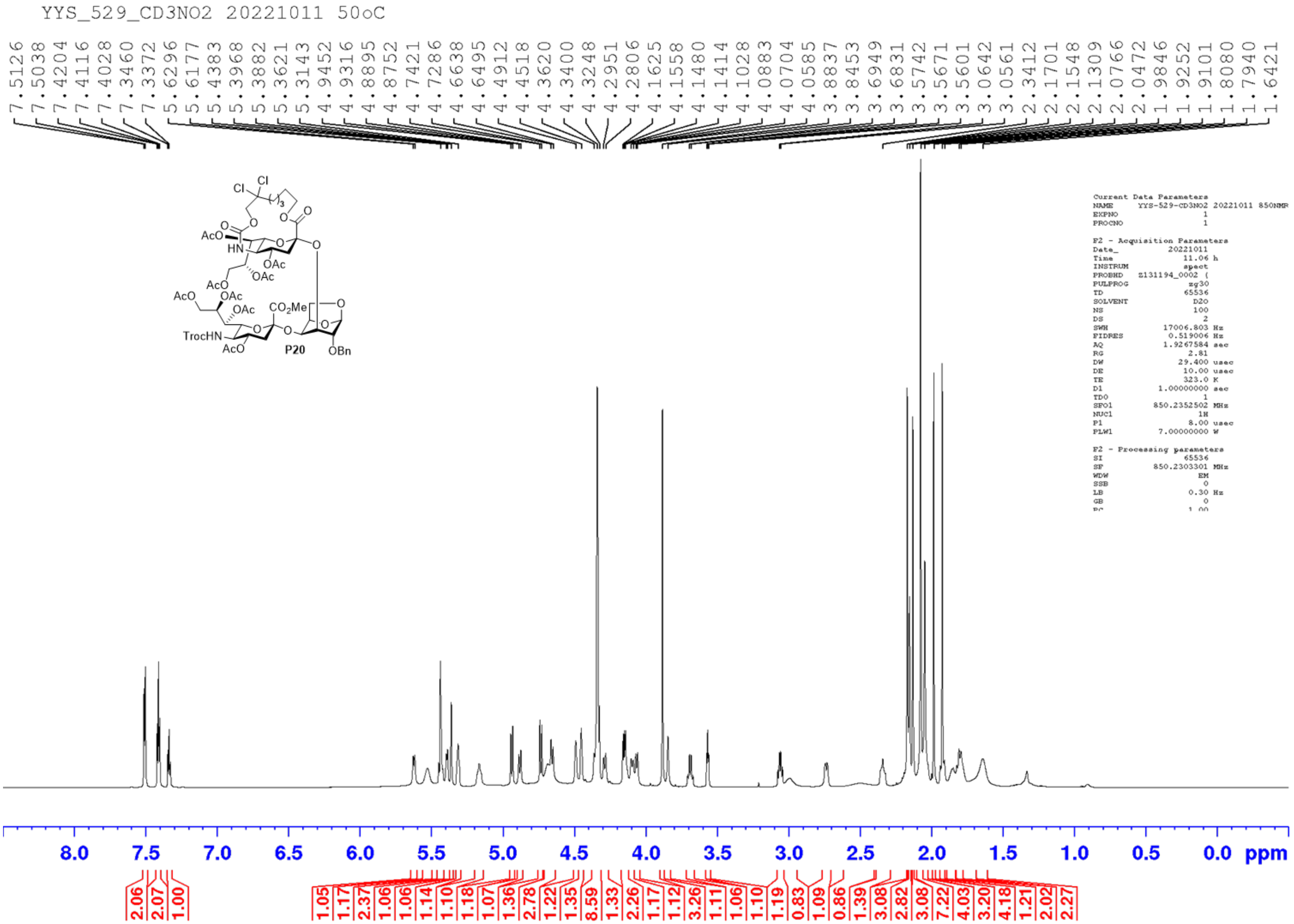
YYS_529_CD3NO2 20221011 50oC
7.5126
7.5038
7.4204
7.4116
7.4028
7.3460
7.3372
5.6296
5.6177
5.4383
5.3968
5.3882
5.3621
5.3143
4.9452
4.9316
4.8895
4.8752
4.7421
4.7286
4.6638
4.6495
4.4912
4.4518
4.3620
4.3400
4.3248
4.2951
4.2806
4.1625
4.1558
4.1480
4.1414
4.1028
4.0883
4.0704
4.0585
3.8837
3.8453
3.6949
3.6831
3.5742
3.5671
3.5601
3.0642
3.0561
2.3412
2.1701
2.1548
2.1309
2.0766
2.0472
1.9846
1.9252
1.9101
1.8080
1.7940
1.6421
Cl
Cl
O
O
O
O
AcO
HN
O
OAc
OAc
AcO
AcO
OAc
OAc
CO2Me
O
O
O
O
TrocHN
AcO
P20
OBn
Current Data Parameters
NAME YYS-529-CD3NO2 20221011 850NMR
EXPNO 1
PROCNO 1
F2 - Acquisition Parameters
Date_ 20221011
Time 11.06 h
INSTRUM spect
PROBHD Z131194_0002 (
PULPROG zg30
TD 65536
SOLVENT D2O
NS 100
DS 2
SWH 17006.803 Hz
FIDRES 0.519006 Hz
AQ 1.9267584 sec
RG 2.81
DW 29.400 usec
DE 10.00 usec
TE 323.0 K
D1 1.00000000 sec
TD0 1
SFO1 850.2352502 MHz
NUC1 1H
P1 8.00 usec
PLW1 7.00000000 W
F2 - Processing parameters
SI 65536
SF 850.2303301 MHz
WDW EM
SSB 0
LB 0.30 Hz
GB 0
8.0
7.5
7.0
6.5
6.0
5.5
5.0
4.5
4.0
3.5
3.0
2.5
2.0
1.5
1.0
0.5
0.0
ppm
2.06
2.07
1.00
1.05
1.17
2.37
1.06
1.06
1.14
1.10
1.18
1.07
1.36
2.78
1.22
1.35
8.59
1.33
2.26
1.17
1.12
3.26
1.11
1.06
1.10
1.19
0.83
1.09
0.86
1.39
3.08
2.82
3.08
7.22
4.03
3.20
4.18
1.21
2.02
2.27

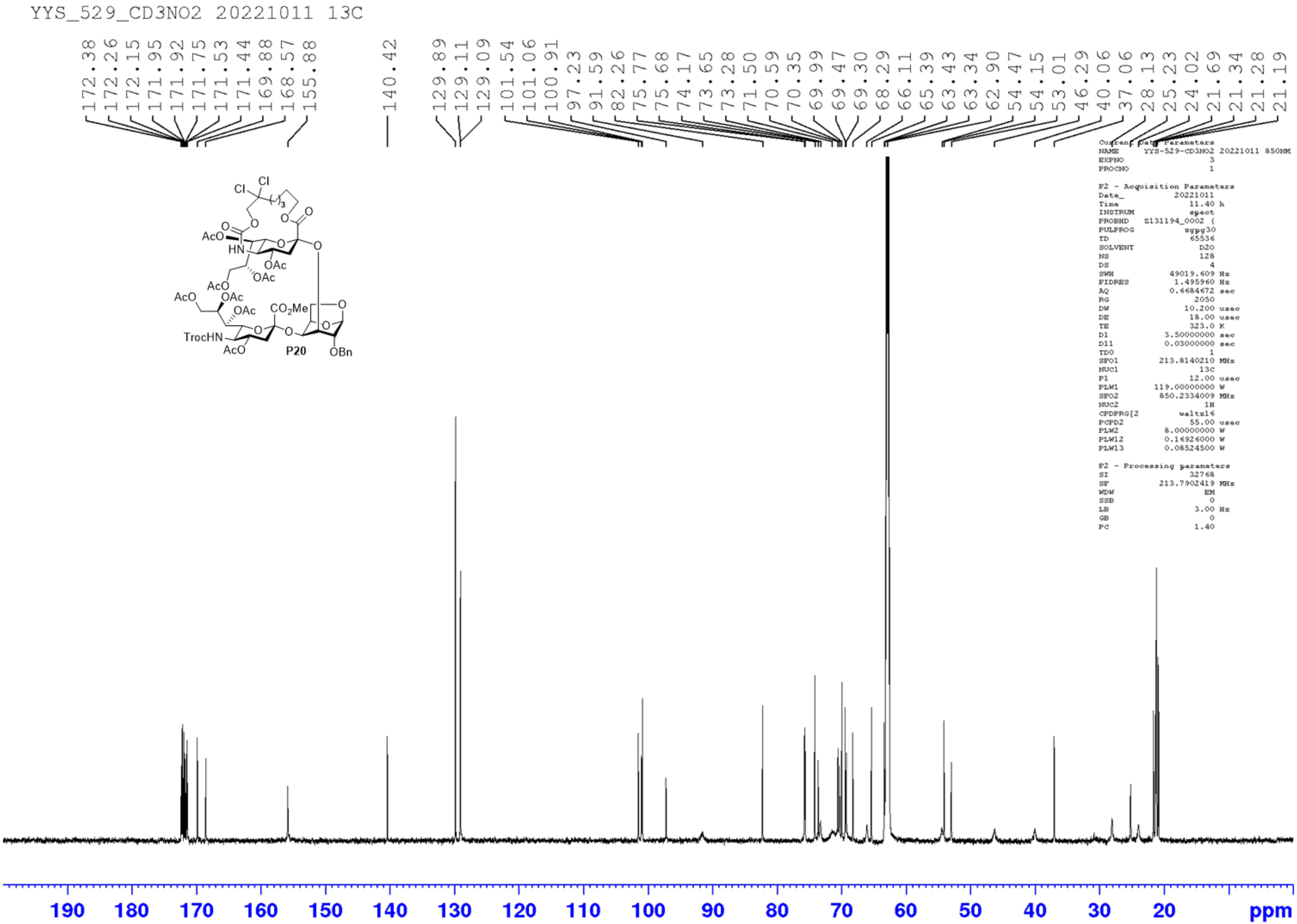
YYS_529_CD3NO2 20221011 13C
172.38
172.26
172.15
171.95
171.92
171.75
171.53
171.44
169.88
168.57
155.88
140.42
129.89
129.11
129.09
101.54
101.06
100.91
97.23
91.59
82.26
75.77
75.68
74.17
73.65
73.28
71.50
70.59
70.35
69.99
69.47
69.30
68.29
66.11
65.39
63.43
63.34
62.90
54.47
54.15
53.01
46.29
40.06
37.06
28.13
25.23
24.02
21.69
21.34
21.28
21.19
Cl
Cl
O
O
AcO
HN
O
O
O
OAc
OAc
AcO
AcO
OAc
OAc
CO2Me
O
TrocHN
AcO
O
O
O
O
P20
OBn
Current Data Parameters
NAME YYS-529-CD3NO2 20221011 850NM
EXPNO 3
PROCNO 1
F2 - Acquisition Parameters
Date_ 20221011
Time 11.40 h
INSTRUM spect
PROBHD Z131194_0002 (
PULPROG zgpg30
TD 65536
SOLVENT D2O
NS 128
DS 4
SWH 49019.609 Hz
FIDRES 1.495960 Hz
AQ 0.6684672 sec
RG 2050
DW 10.200 usec
DE 18.00 usec
TE 323.0 K
D1 3.50000000 sec
D11 0.03000000 sec
TD0 1
SFO1 213.8140210 MHz
NUC1 13C
P1 12.00 usec
PLW1 119.00000000 W
SFO2 850.2334009 MHz
NUC2 1H
CPDPRG[2 waltz16
PCPD2 55.00 usec
PLW2 8.00000000 W
PLW12 0.16926000 W
PLW13 0.08524500 W
F2 - Processing parameters
SI 32768
SF 213.7902419 MHz
WDW EM
SSB 0
LB 3.00 Hz
GB 0
PC 1.40
190
180
170
160
150
140
130
120
110
100
90
80
70
60
50
40
30
20
ppm

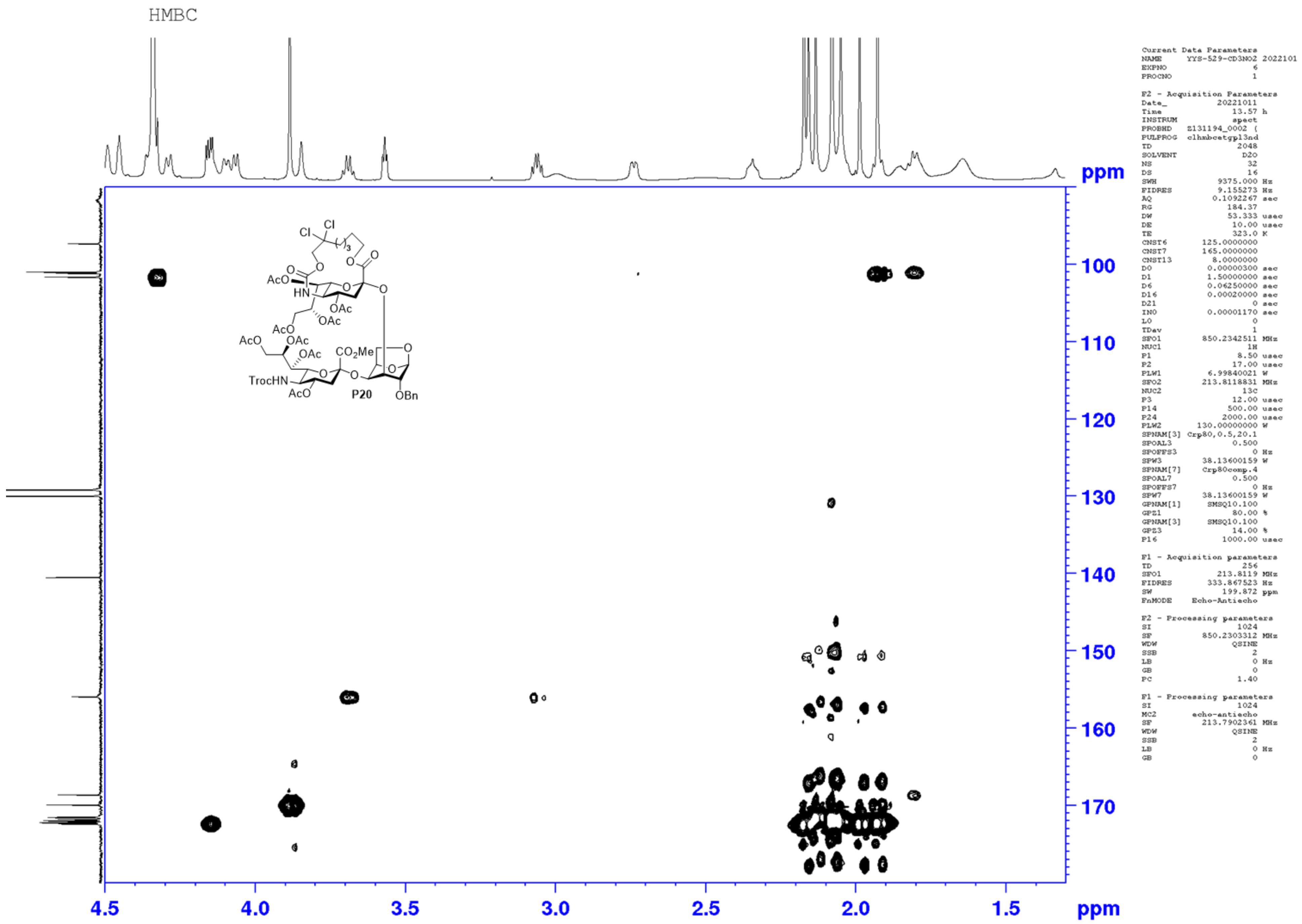
HMBC
Cl
Cl
3
O
O
O
O
AcO
HN
OAc
OAc
AcO
AcO
OAc
OAc
CO2Me
O
O
O
TrocHN
AcO
P20
OBn
ppm
100
110
120
130
140
150
160
170
4.5
4.0
3.5
3.0
2.5
2.0
1.5
ppm
Current Data Parameters
NAME YYS-529-CD3NO2 2022101
EXPNO 6
PROCNO 1
F2 - Acquisition Parameters
Date_ 20221011
Time 13.57 h
INSTRUM spect
PROBHD Z131194_0002 (
PULPROG clhmbcetgpl3nd
TD 2048
SOLVENT D2O
NS 32
DS 16
SWH 9375.000 Hz
FIDRES 9.155273 Hz
AQ 0.1092267 sec
RG 184.37
DW 53.333 usec
DE 10.00 usec
TE 323.0 K
CNST6 125.0000000
CNST7 165.0000000
CNST13 8.0000000
D0 0.00000300 sec
D1 1.50000000 sec
D6 0.06250000 sec
D16 0.00020000 sec
D21 0 sec
IN0 0.00001170 sec
L0 0
TDav 1
SFO1 850.2342511 MHz
NUC1 1H
P1 8.50 usec
P2 17.00 usec
PLW1 6.99840021 W
SFO2 213.8118831 MHz
NUC2 13C
P3 12.00 usec
P14 500.00 usec
P24 2000.00 usec
PLW2 130.00000000 W
SPNAM[3] Crp80,0.5,20.1
SPOAL3 0.500
SPOFFS3 0 Hz
SPW3 38.13600159 W
SPNAM[7] Crp80comp.4
SPOAL7 0.500
SPOFFS7 0 Hz
SPW7 38.13600159 W
GPNAM[1] SMSQ10.100
GPZ1 80.00 %
GPNAM[3] SMSQ10.100
GPZ3 14.00 %
P16 1000.00 usec
F1 - Acquisition parameters
TD 256
SFO1 213.8119 MHz
FIDRES 333.867523 Hz
SW 199.872 ppm
FnMODE Echo-Antiecho
F2 - Processing parameters
SI 1024
SF 850.2303312 MHz
WDW QSINE
SSB 2
LB 0 Hz
GB 0
PC 1.40
F1 - Processing parameters
SI 1024
MC2 echo-antiecho
SF 213.7902361 MHz
WDW QSINE
SSB 2
LB 0 Hz
GB 0

YYS_529_CD3NO2 20221011 H-H COSY

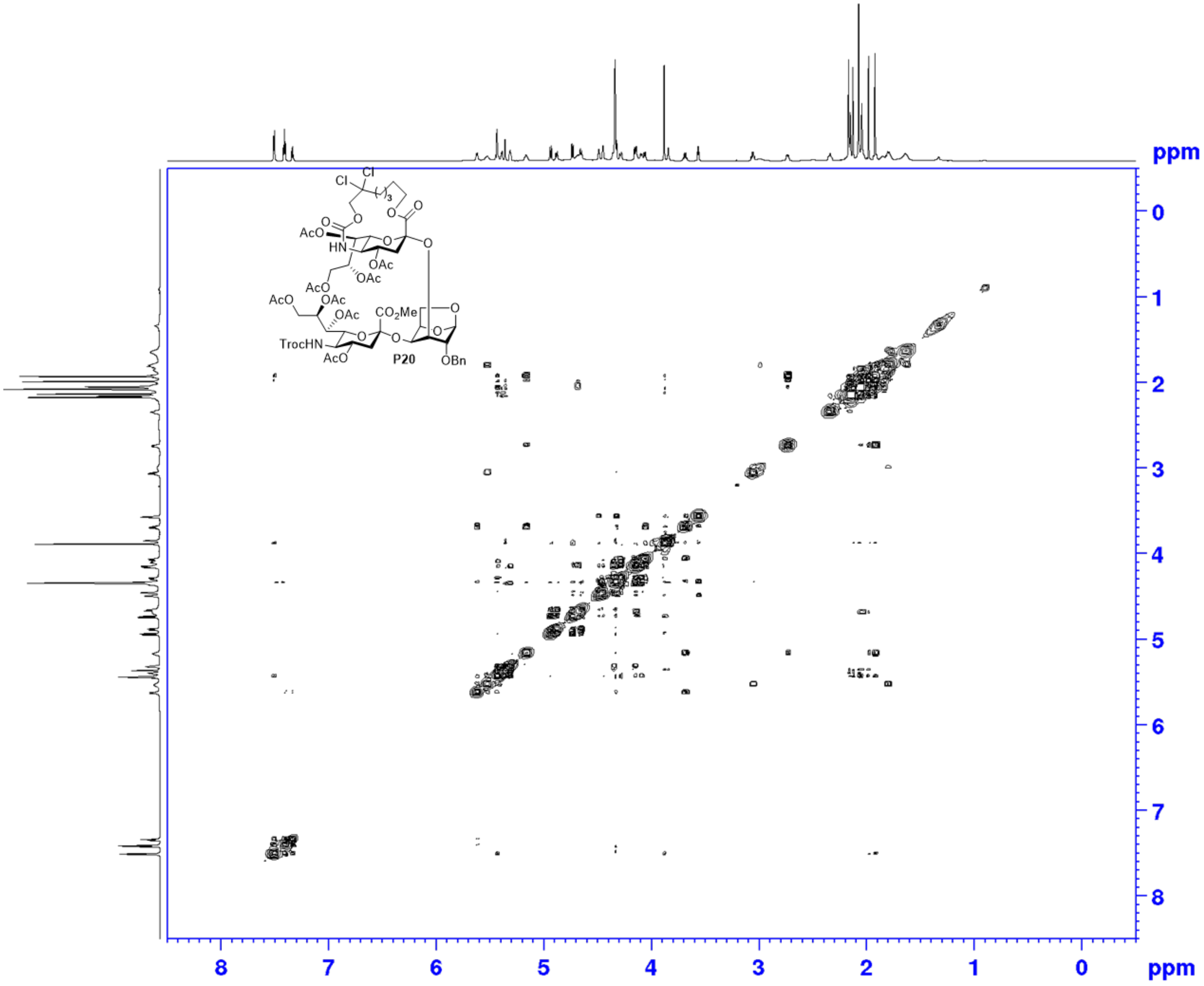


```
Current Data Parameters
NAME      YYS-529-CD3NO2 20221011 850N
EXPNO                    4
PROCNO                   1

F2 - Acquisition Parameters
Date_             20221011
Time                 11.41 h
INSTRUM              spect
PROBHD    Z131194_0002 (
PULPROG           cosygf90
TD                    2048
SOLVENT                D2O
NS                       8
DS                       0
SWH               8503.401 Hz
FIDRES            8.304103 Hz
AQ               0.1204224 sec
RG                    8.19
DW                  58.800 usec
DE                   10.00 usec
TE                   323.0 K
D0              0.00000300 sec
D1              1.50000000 sec
IN0             0.00011760 sec
TDav                     1
SFO1           850.2339961 MHz
NUC1                    1H
P1                    8.50 usec
PLW1            6.99840021 W

F1 - Acquisition parameters
TD                     256
SFO1               850.234 MHz
FIDRES           66.432823 Hz
SW                  10.001 ppm
FnMODE                  QF

F2 - Processing parameters
SI                    1024
SF             850.2303321 MHz
WDW                   SINE
SSB                      0
LB                       0 Hz
GB                       0
PC                    1.40

F1 - Processing parameters
SI                    1024
MC2                     QF
SF             850.2303333 MHz
WDW                   SINE
SSB                      0
LB                       0 Hz
GB                       0
```

HSQC

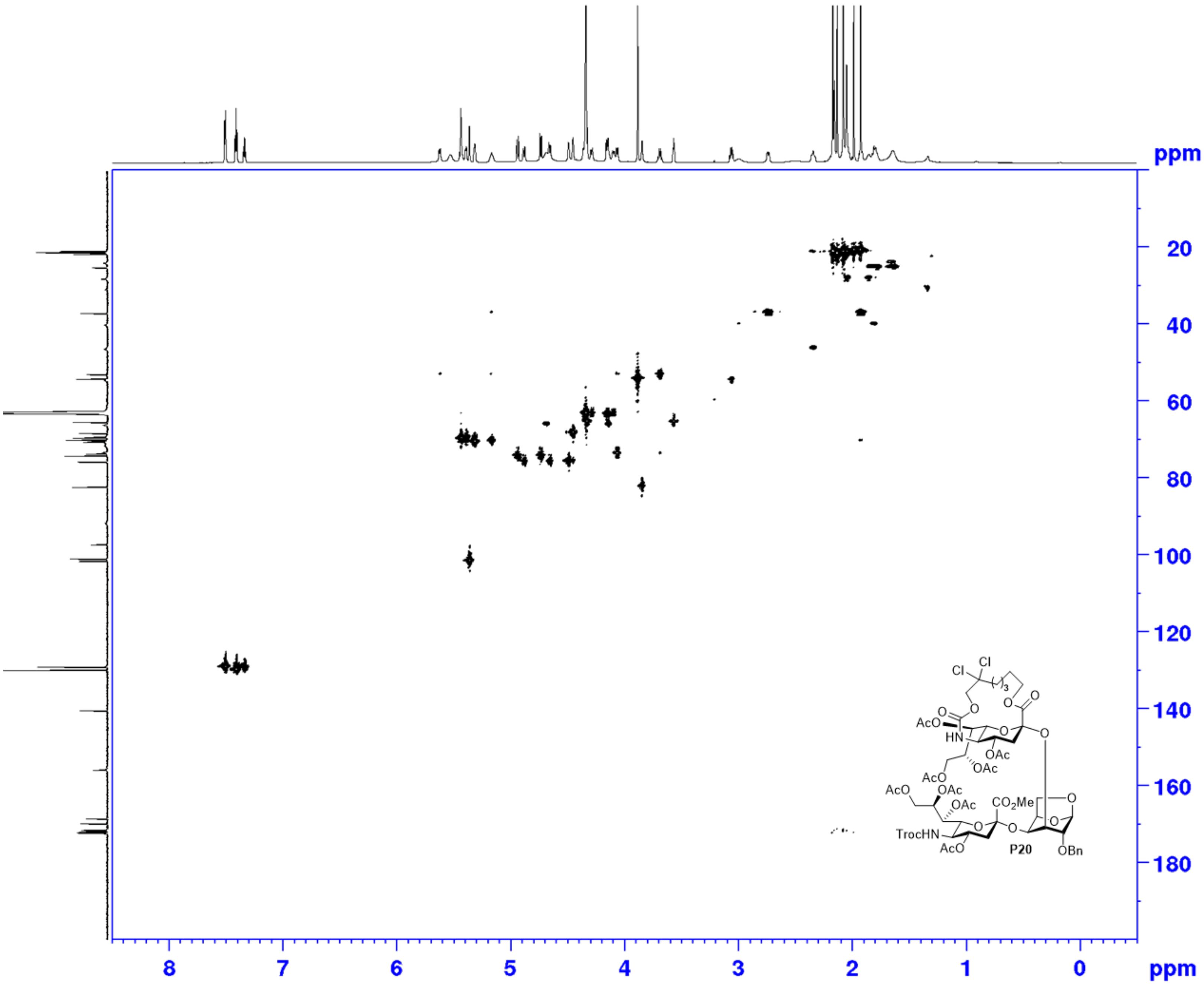


```
Current Data Parameters
NAME      YYS-529-CD3NO2 20221011 850N
EXPNO                  5
PROCNO                 1

F2 - Acquisition Parameters
Date_           20221011
Time               12.37 h
INSTRUM            spect
PROBHD   Z131194_0002 (
PULPROG  hsqcetgpsisp2.2
TD                  2048
SOLVENT              D2O
NS                     8
DS                     8
SWH             8503.401 Hz
FIDRES          8.304103 Hz
AQ             0.1204224 sec
RG                184.37
DW                58.800 usec
DE                 10.00 usec
TE                 323.0 K
CNST2        145.0000000
CNST17        -0.5000000
D0            0.00000300 sec
D1            1.50000000 sec
D4            0.00172414 sec
D11           0.03000000 sec
D16           0.00020000 sec
D24           0.00086207 sec
IN0           0.00001060 sec
TDav                   1
SFO1         850.2340054 MHz
NUC1                  1H
P1                  8.50 usec
P2                 17.00 usec
P28                    0 usec
PLW1          6.99840021 W
SFO2         213.8118831 MHz
NUC2                 13C
CPDPRG[2            garp
P3                 12.00 usec
P14               500.00 usec
P24              2000.00 usec
PCPD2              50.00 usec
PLW0                   0 W
PLW2        130.00000000 W
PLW12         7.48799992 W
SPNAM[3] Crp80,0.5,20.1
SPOAL3             0.500
SPOFFS3                0 Hz
SPW3         38.13600159 W
SPNAM[7]    Crp80comp.4
SPOAL7             0.500
SPOFFS7                0 Hz
SPW7         38.13600159 W
GPNAM[1]     SMSQ10.100
GPZ1               80.00 %
GPNAM[2]     SMSQ10.100
GPZ2               20.10 %
GPNAM[3]     SMSQ10.100
GPZ3               11.00 %
GPNAM[4]     SMSQ10.100
GPZ4               -5.00 %
P16              1000.00 usec
P19               600.00 usec

F1 - Acquisition parameters
TD                   360
SFO1            213.8119 MHz
FIDRES        262.054504 Hz
SW               220.614 ppm
FnMODE     Echo-Antiecho

F2 - Processing parameters
SI                  1024
SF           850.2303292 MHz
WDW                QSINE
SSB                    4
LB                     0 Hz
GB                     0
PC                  1.40

F1 - Processing parameters
SI                  1024
MC2        echo-antiecho
SF           213.7902426 MHz
WDW                QSINE
SSB                    4
LB                     0 Hz
GB                     0
```

HMBC

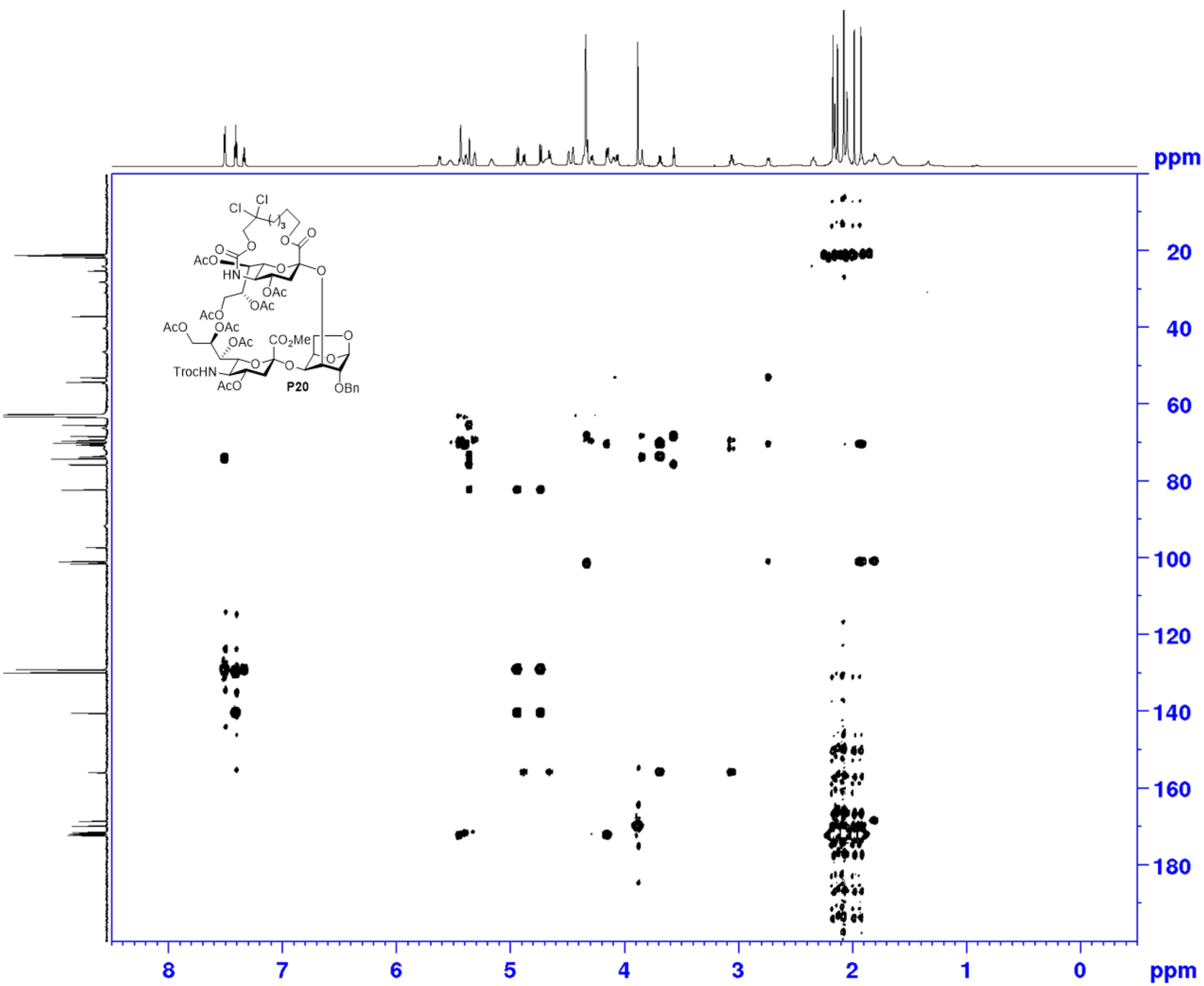

Current Data Parameters
NAME YYS-529-CD3NO2 20221011 850N[illegible]
EXPNO 6
PROCNO 1

F2 - Acquisition Parameters
Date_ 20221011
Time 13.57 h
INSTRUM spect
PROBHD Z131194_0002 (
PULPROG clhmbcetgpl3nd
TD 2048
SOLVENT D2O
NS 32
DS 16
SWH 9375.000 Hz
FIDRES 9.155273 Hz
AQ 0.1092267 sec
RG 184.37
DW 53.333 usec
DE 10.00 usec
TE 323.0 K
CNST6 125.0000000
CNST7 165.0000000
CNST13 8.0000000
D0 0.00000300 sec
D1 1.50000000 sec
D6 0.06250000 sec
D16 0.00020000 sec
D21 0 sec
IN0 0.00001170 sec
L0 0
TDav 1
SFO1 850.2342511 MHz
NUC1 1H
P1 8.50 usec
P2 17.00 usec
PLW1 6.99840021 W
SFO2 213.8118831 MHz
NUC2 13C
P3 12.00 usec
P14 500.00 usec
P24 2000.00 usec
PLW2 130.00000000 W
SPNAM[3] Crp80,0.5,20.1
SPOAL3 0.500
SPOFFS3 0 Hz
SPW3 38.13600159 W
SPNAM[7] Crp80comp.4
SPOAL7 0.500
SPOFFS7 0 Hz
SPW7 38.13600159 W
GPNAM[1] SMSQ10.100
GPZ1 80.00 %
GPNAM[3] SMSQ10.100
GPZ3 14.00 %
P16 1000.00 usec

F1 - Acquisition parameters
TD 256
SFO1 213.8119 MHz
FIDRES 333.867523 Hz
SW 199.872 ppm
FnMODE Echo-Antiecho

F2 - Processing parameters
SI 1024
SF 850.2303312 MHz
WDW QSINE
SSB 2
LB 0 Hz
GB 0
PC 1.40

F1 - Processing parameters
SI 1024
MC2 echo-antiecho
SF 213.7902361 MHz
WDW QSINE
SSB 2
LB 0 Hz
GB 0

YYS_529_CD3NO2 20221011 H-C HSQCTOCSY

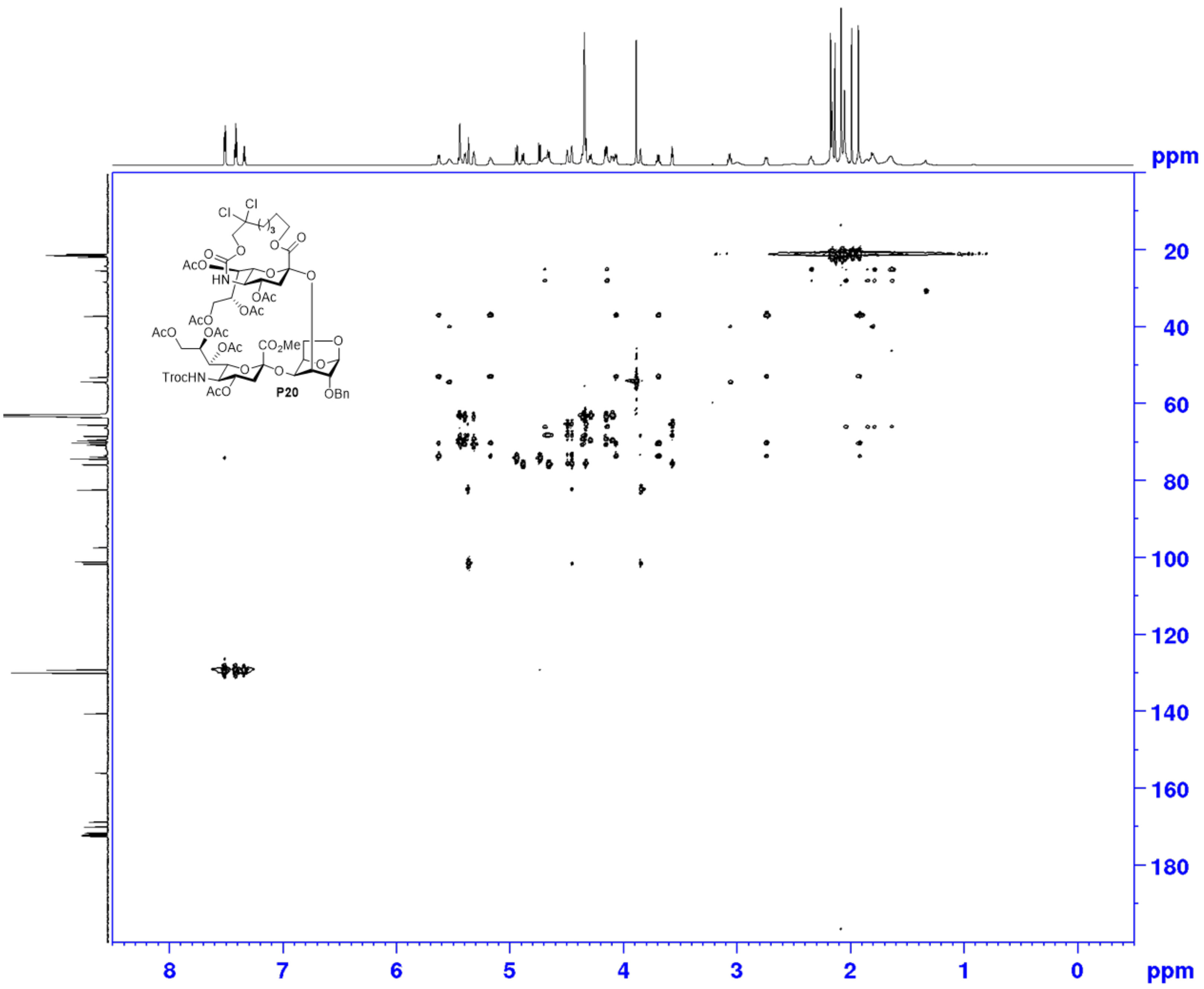


```
Current Data Parameters
NAME      YYS-529-CD3NO2 20221011 850NMR
EXPNO                    7
PROCNO                   1

F2 - Acquisition Parameters
Date_             20221011
Time                 17.48 h
INSTRUM              spect
PROBHD    Z131194_0002 (
PULPROG   hsqcdietgpsisp.2
TD                    2048
SOLVENT                D2O
NS                      32
DS                       8
SWH               9375.000 Hz
FIDRES            9.155273 Hz
AQ               0.1092267 sec
RG                    1030
DW                  53.333 usec
DE                   10.00 usec
TE                   323.0 K
CNST2          145.0000000
CNST17          -0.5000000
D0              0.00000300 sec
D1              1.20000005 sec
D4              0.00172414 sec
D9              0.07500000 sec
D11             0.03000000 sec
D16             0.00020000 sec
D24             0.00089000 sec
IN0             0.00000970 sec
L1                      32
TDav                     1
SFO1           850.2342511 MHz
NUC1                    1H
P1                    8.50 usec
P2                   17.00 usec
P6                   20.00 usec
P28                      0 usec
PLW1            6.99840021 W
PLW10           1.26409996 W
SFO2           213.8076072 MHz
NUC2                   13C
CPDPRG[2              garp
P3                   12.00 usec
P14                 500.00 usec
P24                2000.00 usec
PCPD2                50.00 usec
PLW0                     0 W
PLW2          130.00000000 W
PLW12           7.48799992 W
SPNAM[3]  Crp80,0.5,20.1
SPOAL3               0.500
SPOFFS3                  0 Hz
SPW3           38.13600159 W
SPNAM[7]     Crp80comp.4
SPOAL7               0.500
SPOFFS7                  0 Hz
SPW7           38.13600159 W
GPNAM[1]       SMSQ10.100
GPZ1                 80.00 %
GPNAM[2]       SMSQ10.100
GPZ2                 20.10 %
P16                1000.00 usec

F1 - Acquisition parameters
TD                     300
SFO1              213.8076 MHz
FIDRES          343.642609 Hz
SW                 241.088 ppm
FnMODE      Echo-Antiecho

F2 - Processing parameters
SI                    1024
SF             850.2303326 MHz
WDW                  QSINE
SSB                      3
LB                       0 Hz
GB                       0
PC                    1.40

F1 - Processing parameters
SI                    1024
MC2          echo-antiecho
SF             213.7902322 MHz
WDW                  QSINE
SSB                      3
LB                       0 Hz
GB                       0
```

```
Current Data Parameters
NAME          KYT-238-600
EXPNO                   1
PROCNO                  1

F2 - Acquisition Parameters
Date_            20200110
Time                 9.03
INSTRUM             spect
PROBHD   5 mm QNP  1H/1
PULPROG                zg
TD                  32768
SOLVENT             CDCl3
NS                     32
DS                      0
SWH              9541.984 Hz
FIDRES           0.291198 Hz
AQ              1.7170432 sec
RG                   1024
DW                 52.400 usec
DE                   6.50 usec
TE                  295.2 K
D1             2.00000000 sec
MCREST                  0 sec
MCWRK          0.01500000 sec

======== CHANNEL f1 ========
NUC1                   1H
P1                  10.15 usec
PL1                 -1.00 dB
SFO1          598.1034854 MHz

F2 - Processing parameters
SI                  32768
SF            598.1000311 MHz
WDW                    no
SSB                     0
LB                      0 Hz
GB                      0
PC                   2.00
```

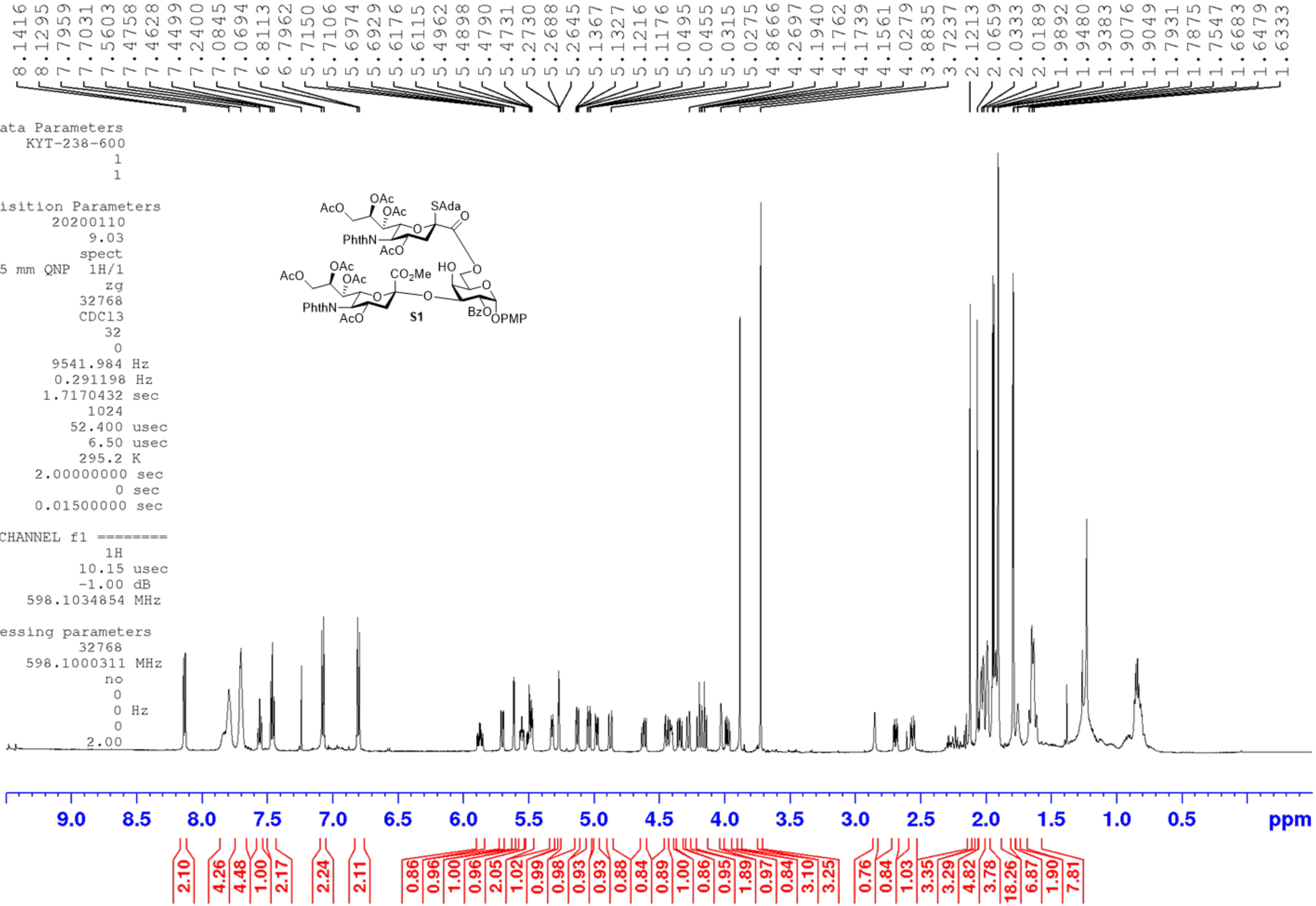

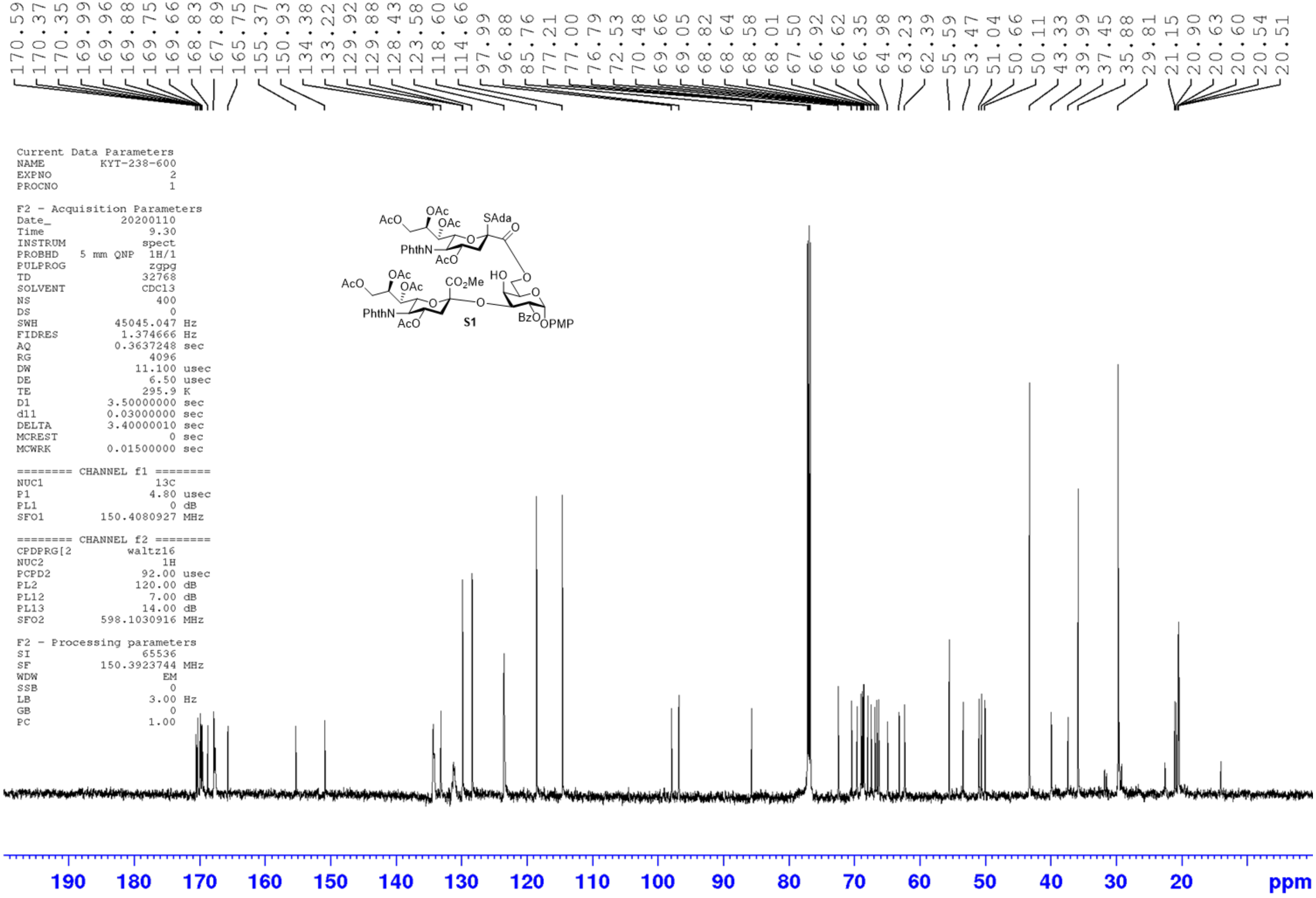

170.59
170.37
170.35
169.99
169.96
169.88
169.75
169.66
168.83
167.89
165.75
155.37
150.93
134.38
133.22
129.92
129.88
128.43
123.58
118.60
114.66
97.99
96.88
85.76
77.21
77.00
76.79
72.53
70.48
69.66
69.05
68.82
68.64
68.58
68.01
67.50
66.92
66.62
66.35
64.98
63.23
62.39
55.59
53.47
51.04
50.66
50.11
43.33
39.99
37.45
35.88
29.81
21.15
20.90
20.63
20.60
20.54
20.51
Current Data Parameters
NAME KYT-238-600
EXPNO 2
PROCNO 1
F2 - Acquisition Parameters
Date_ 20200110
Time 9.30
INSTRUM spect
PROBHD 5 mm QNP 1H/1
PULPROG zgpg
TD 32768
SOLVENT CDCl3
NS 400
DS 0
SWH 45045.047 Hz
FIDRES 1.374666 Hz
AQ 0.3637248 sec
RG 4096
DW 11.100 usec
DE 6.50 usec
TE 295.9 K
D1 3.50000000 sec
d11 0.03000000 sec
DELTA 3.40000010 sec
MCREST 0 sec
MCWRK 0.01500000 sec
======== CHANNEL f1 ========
NUC1 13C
P1 4.80 usec
PL1 0 dB
SFO1 150.4080927 MHz
======== CHANNEL f2 ========
CPDPRG[2 waltz16
NUC2 1H
PCPD2 92.00 usec
PL2 120.00 dB
PL12 7.00 dB
PL13 14.00 dB
SFO2 598.1030916 MHz
F2 - Processing parameters
SI 65536
SF 150.3923744 MHz
WDW EM
SSB 0
LB 3.00 Hz
GB 0
PC 1.00
AcO
OAc
OAc
SAda
O
PhthN
AcO
O
AcO
OAc
OAc
CO_2Me
HO
O
PhthN
AcO
S1
BzO
OPMP
190
180
170
160
150
140
130
120
110
100
90
80
70
60
50
40
30
20
ppm

COSY

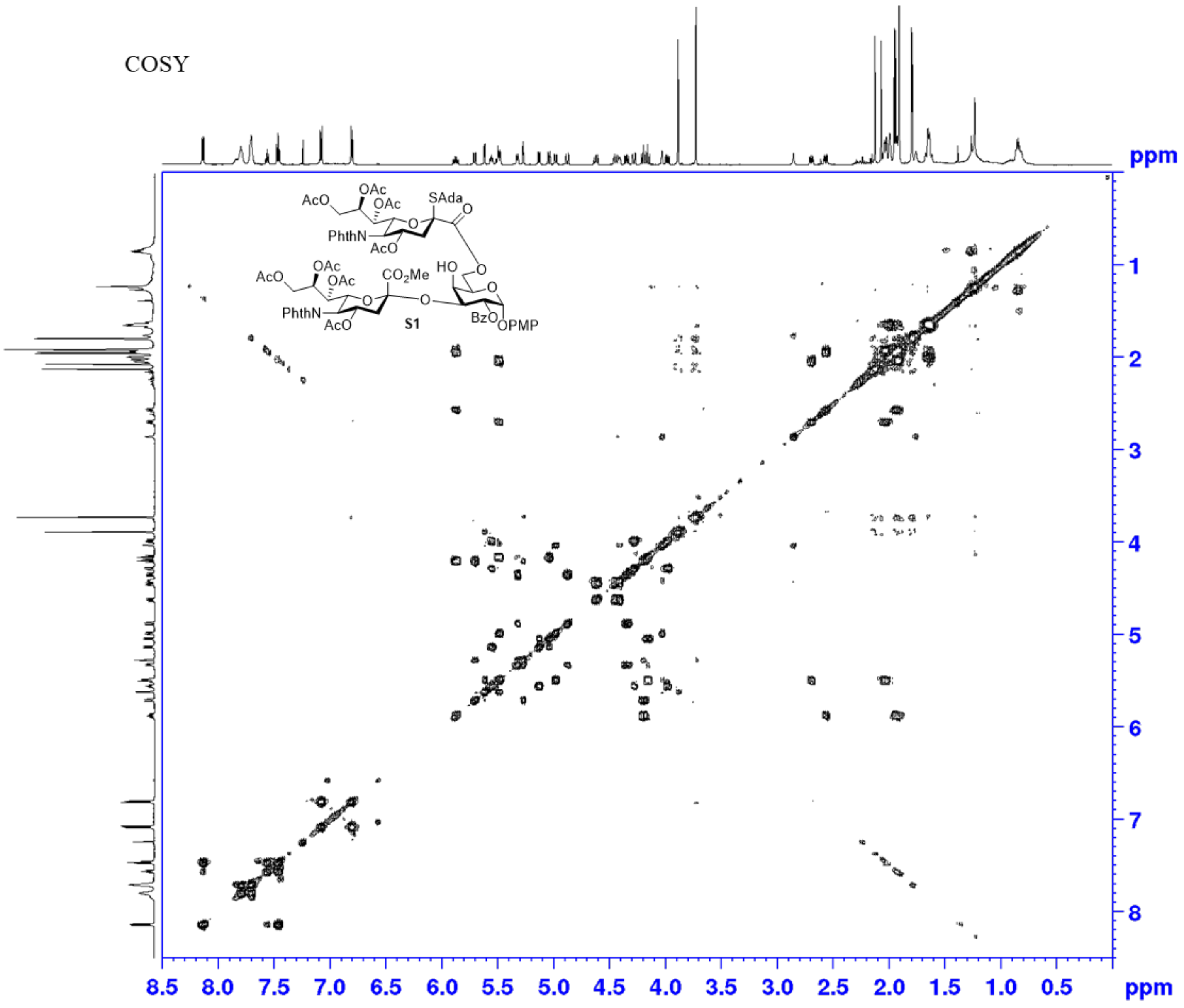


```
Current Data Parameters
NAME          KYT-238-600
EXPNO                  11
PROCNO                  1

F2 - Acquisition Parameters
Date_            20200110
Time                10.34
INSTRUM             spect
PROBHD    5 mm QNP  1H/1
PULPROG           cosygf
TD                   2048
SOLVENT              MeOD
NS                      8
DS                      0
SWH              6009.615 Hz
FIDRES           2.934382 Hz
AQ              0.1703936 sec
RG                     64
DW                 83.200 usec
DE                   6.50 usec
TE                  295.4 K
d0             0.00000300 sec
D1             1.50000000 sec
IN0            0.00016640 sec
MCREST                  0 sec
MCWRK          1.50000000 sec

======== CHANNEL f1 ========
NUC1                   1H
P0                  14.00 usec
P1                  10.20 usec
PL1                 -2.00 dB
SFO1          598.1028685 MHz

F1 - Acquisition parameters
TD                    400
SFO1          598.1029 MHz
FIDRES          30.048077 Hz
SW                 10.048 ppm
FnMODE                 QF

F2 - Processing parameters
SI                   2048
SF            598.1000311 MHz
WDW                 QSINE
SSB                     0
LB                      0 Hz
GB                      0
PC                   1.00

F1 - Processing parameters
SI                    512
MC2                    QF
SF            598.1000311 MHz
WDW                 QSINE
SSB                     0
LB                      0 Hz
GB                      0
```

HSQC

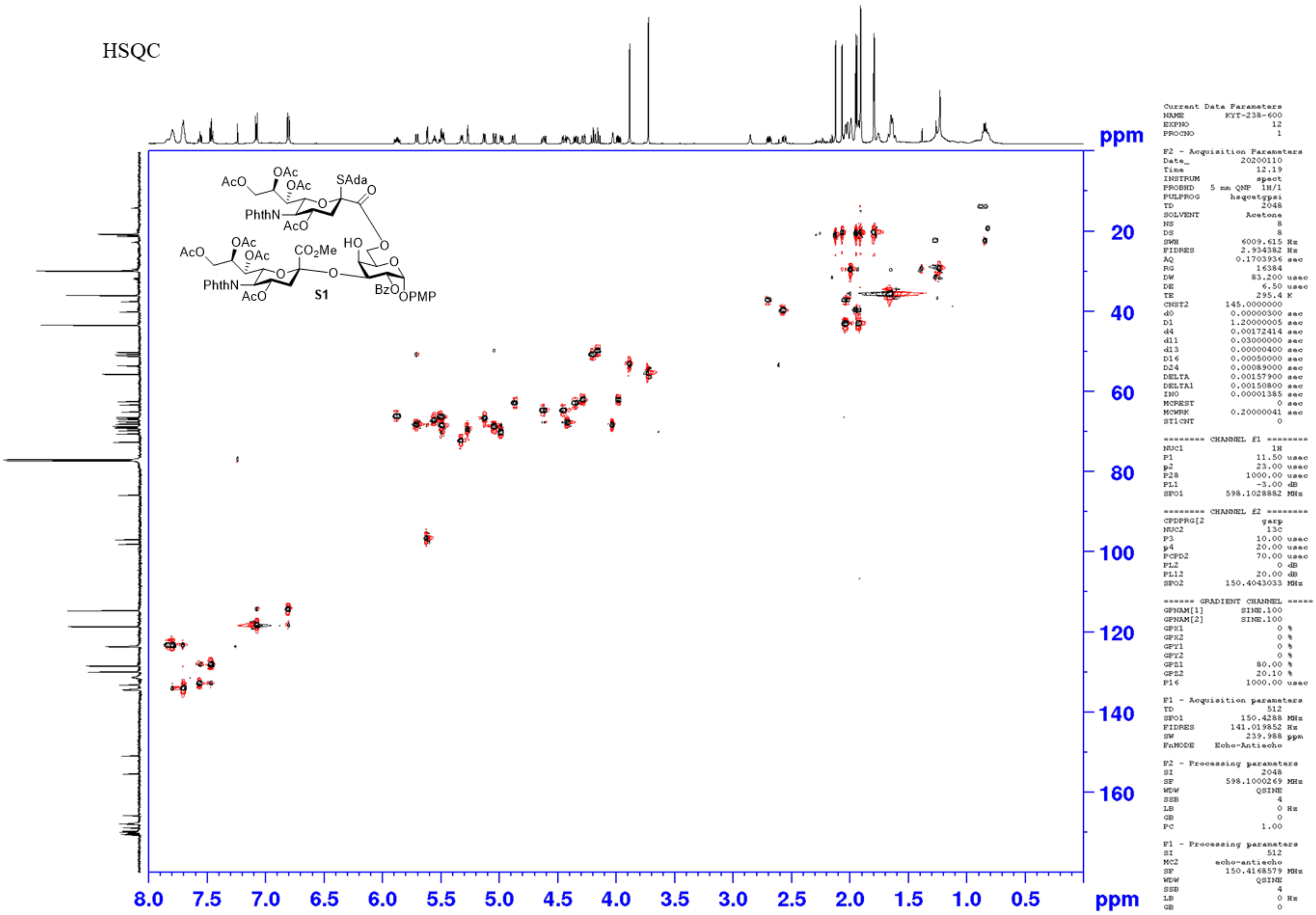

AcO
OAc
OAc
SAda
O
PhthN
AcO
O
AcO
OAc
OAc
CO2Me
HO
O
O
PhthN
AcO
S1
BzO
OPMP
ppm
20
40
60
80
100
120
140
160
ppm
8.0
7.5
7.0
6.5
6.0
5.5
5.0
4.5
4.0
3.5
3.0
2.5
2.0
1.5
1.0
0.5
ppm
Current Data Parameters
NAME KYT-238-600
EXPNO 12
PROCNO 1
F2 - Acquisition Parameters
Date_ 20200110
Time 12.19
INSTRUM spect
PROBHD 5 mm QNP 1H/1
PULPROG hsqcetgpsi
TD 2048
SOLVENT Acetone
NS 8
DS 8
SWH 6009.615 Hz
FIDRES 2.934382 Hz
AQ 0.1703936 sec
RG 16384
DW 83.200 usec
DE 6.50 usec
TE 295.4 K
CNST2 145.0000000
d0 0.00000300 sec
D1 1.20000005 sec
d4 0.00172414 sec
d11 0.03000000 sec
d13 0.00000400 sec
D16 0.00050000 sec
D24 0.00089000 sec
DELTA 0.00157900 sec
DELTA1 0.00150800 sec
IN0 0.00001385 sec
MCREST 0 sec
MCWRK 0.20000041 sec
ST1CNT 0
======== CHANNEL f1 ========
NUC1 1H
P1 11.50 usec
p2 23.00 usec
P28 1000.00 usec
PL1 -3.00 dB
SFO1 598.1028882 MHz
======== CHANNEL f2 ========
CPDPRG[2 garp
NUC2 13C
P3 10.00 usec
p4 20.00 usec
PCPD2 70.00 usec
PL2 0 dB
PL12 20.00 dB
SFO2 150.4043033 MHz
====== GRADIENT CHANNEL =====
GPNAM[1] SINE.100
GPNAM[2] SINE.100
GPX1 0 %
GPX2 0 %
GPY1 0 %
GPY2 0 %
GPZ1 80.00 %
GPZ2 20.10 %
P16 1000.00 usec
F1 - Acquisition parameters
TD 512
SFO1 150.4288 MHz
FIDRES 141.019852 Hz
SW 239.988 ppm
FnMODE Echo-Antiecho
F2 - Processing parameters
SI 2048
SF 598.1000269 MHz
WDW QSINE
SSB 4
LB 0 Hz
GB 0
PC 1.00
F1 - Processing parameters
SI 512
MC2 echo-antiecho
SF 150.4168579 MHz
WDW QSINE
SSB 4
LB 0 Hz
GB 0

HMBC

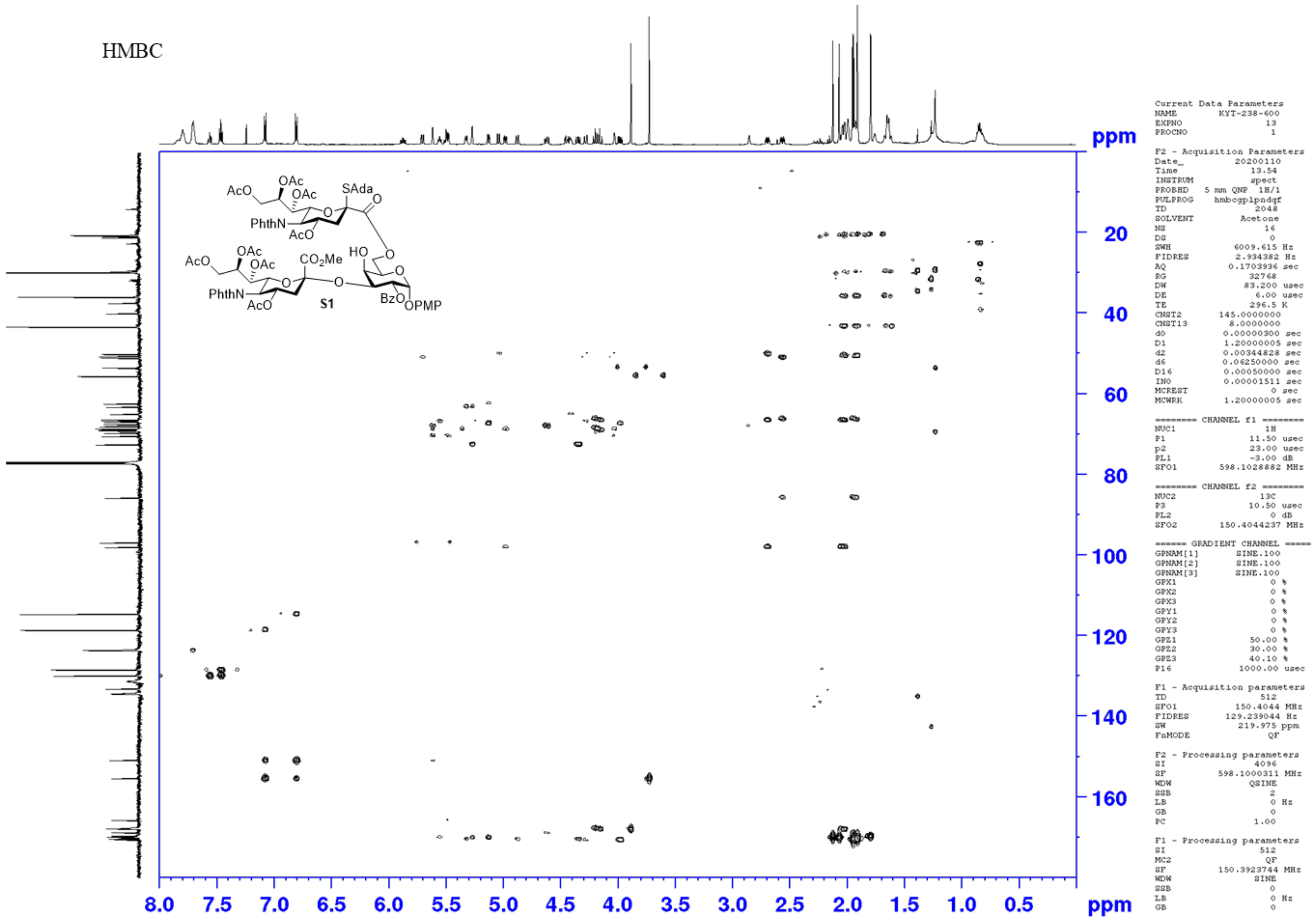

ppm
ppm
S1
Current Data Parameters
NAME KYT-238-600
EXPNO 13
PROCNO 1
F2 - Acquisition Parameters
Date_ 20200110
Time 13.54
INSTRUM spect
PROBHD 5 mm QNP 1H/1
PULPROG hmbcgplpndqf
TD 2048
SOLVENT Acetone
NS 16
DS 0
SWH 6009.615 Hz
FIDRES 2.934382 Hz
AQ 0.1703936 sec
RG 32768
DW 83.200 usec
DE 6.00 usec
TE 296.5 K
CNST2 145.0000000
CNST13 8.0000000
d0 0.00000300 sec
D1 1.20000005 sec
d2 0.00344828 sec
d6 0.06250000 sec
D16 0.00050000 sec
IN0 0.00001511 sec
MCREST 0 sec
MCWRK 1.20000005 sec
======== CHANNEL f1 ========
NUC1 1H
P1 11.50 usec
p2 23.00 usec
PL1 -3.00 dB
SFO1 598.1028882 MHz
======== CHANNEL f2 ========
NUC2 13C
P3 10.50 usec
PL2 0 dB
SFO2 150.4044237 MHz
====== GRADIENT CHANNEL =====
GPNAM[1] SINE.100
GPNAM[2] SINE.100
GPNAM[3] SINE.100
GPX1 0 %
GPX2 0 %
GPX3 0 %
GPY1 0 %
GPY2 0 %
GPY3 0 %
GPZ1 50.00 %
GPZ2 30.00 %
GPZ3 40.10 %
P16 1000.00 usec
F1 - Acquisition parameters
TD 512
SFO1 150.4044 MHz
FIDRES 129.239044 Hz
SW 219.975 ppm
FnMODE QF
F2 - Processing parameters
SI 4096
SF 598.1000311 MHz
WDW QSINE
SSB 2
LB 0 Hz
GB 0
PC 1.00
F1 - Processing parameters
SI 512
MC2 QF
SF 150.3923744 MHz
WDW SINE
SSB 0
LB 0 Hz
GB 0

HSQC-TOSCY

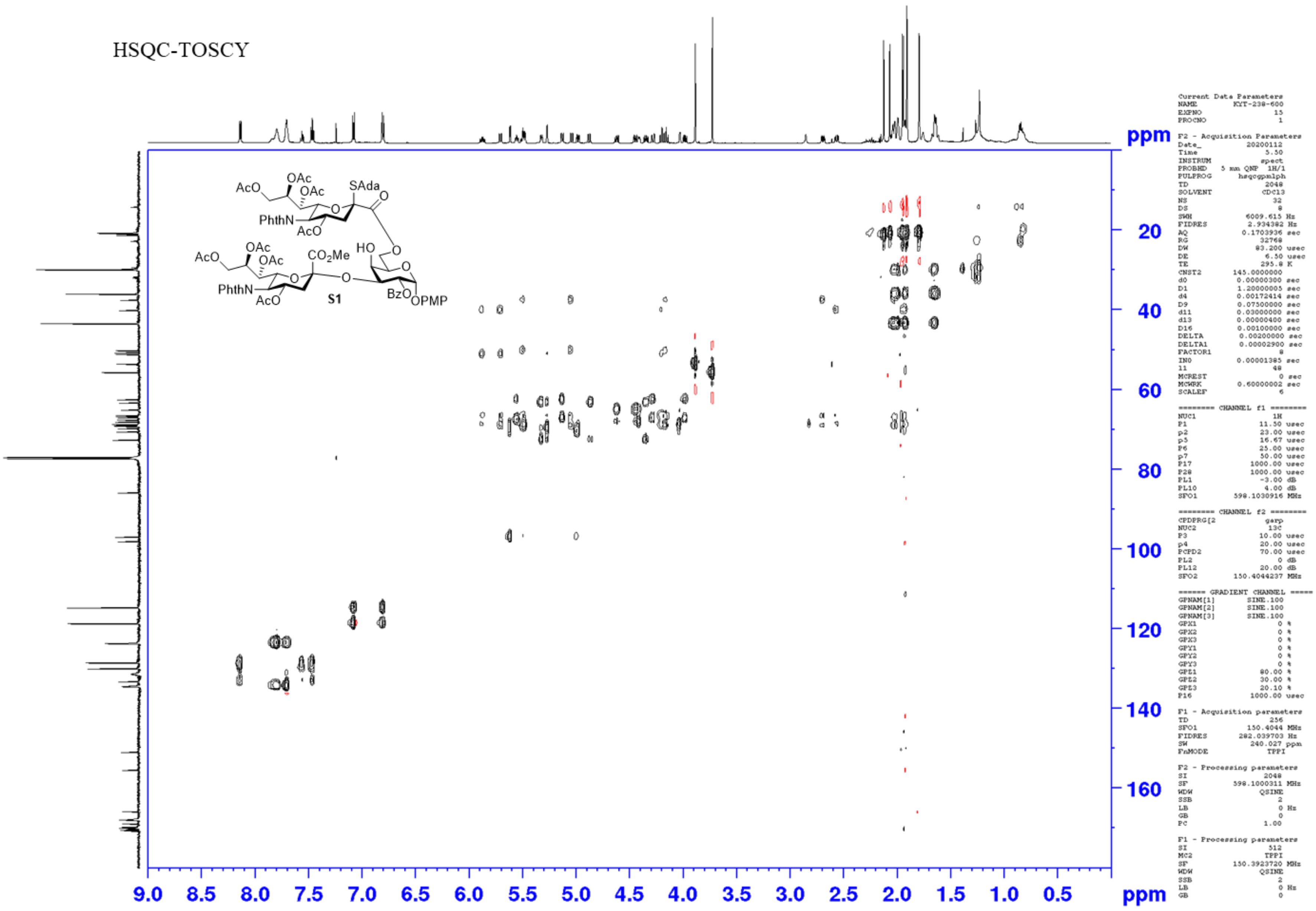

AcO
OAc
OAc
SAda
O
PhthN
AcO
O
AcO
OAc
OAc
CO_2Me
HO
PhthN
AcO
S1
BzO
OPMP
ppm

KYT-251-20200311-H

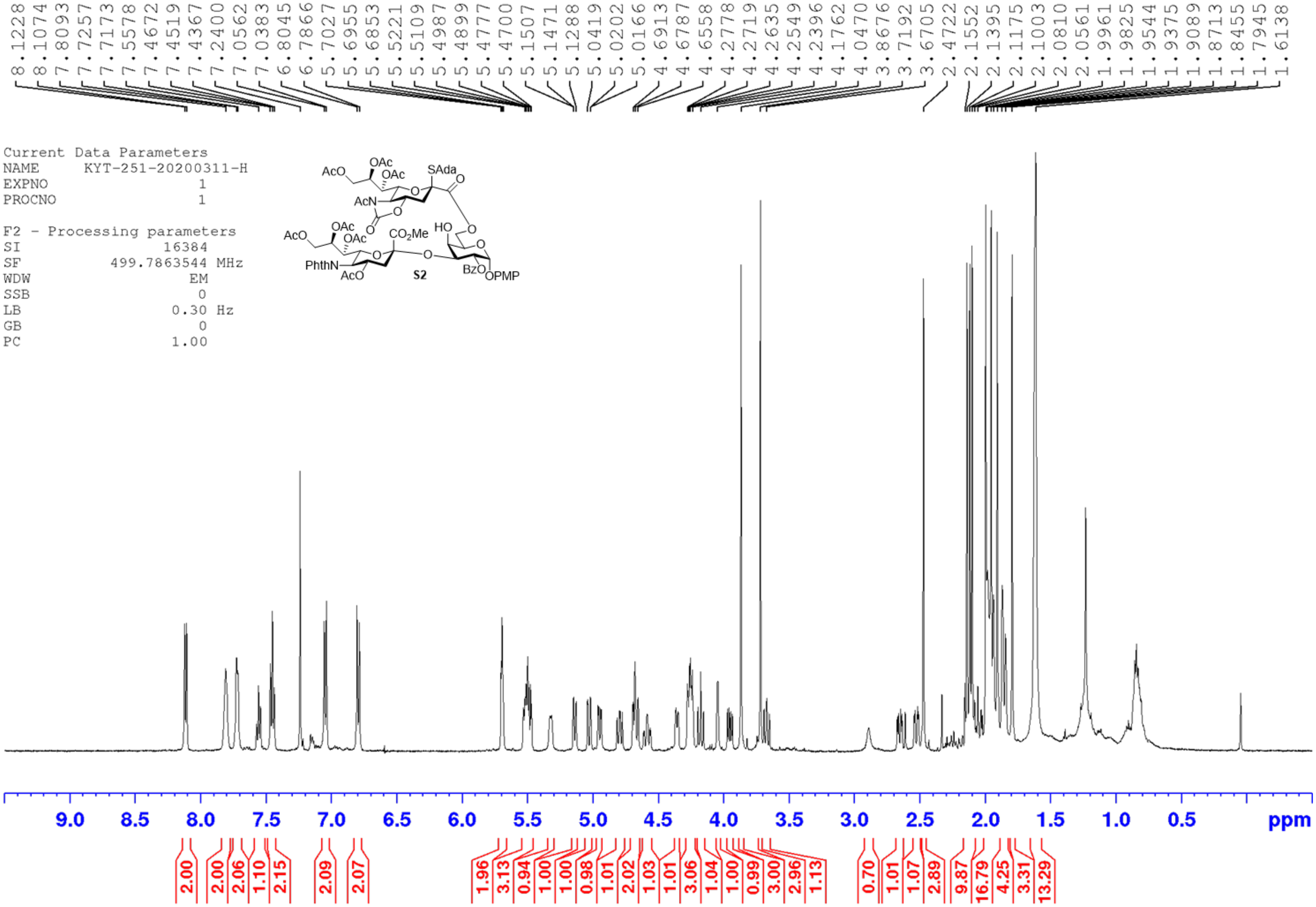

Current Data Parameters
NAME KYT-251-20200311-H
EXPNO 1
PROCNO 1

F2 - Processing parameters
SI 16384
SF 499.7863544 MHz
WDW EM
SSB 0
LB 0.30 Hz
GB 0
PC 1.00

Current Data Parameters
NAME KYT-251-20200311-C
EXPNO 1
PROCNO 1

F2 - Processing parameters
SI 65536
SF 125.6713834 MHz
WDW EM
SSB 0
LB 0.30 Hz
GB 0
PC 1.00

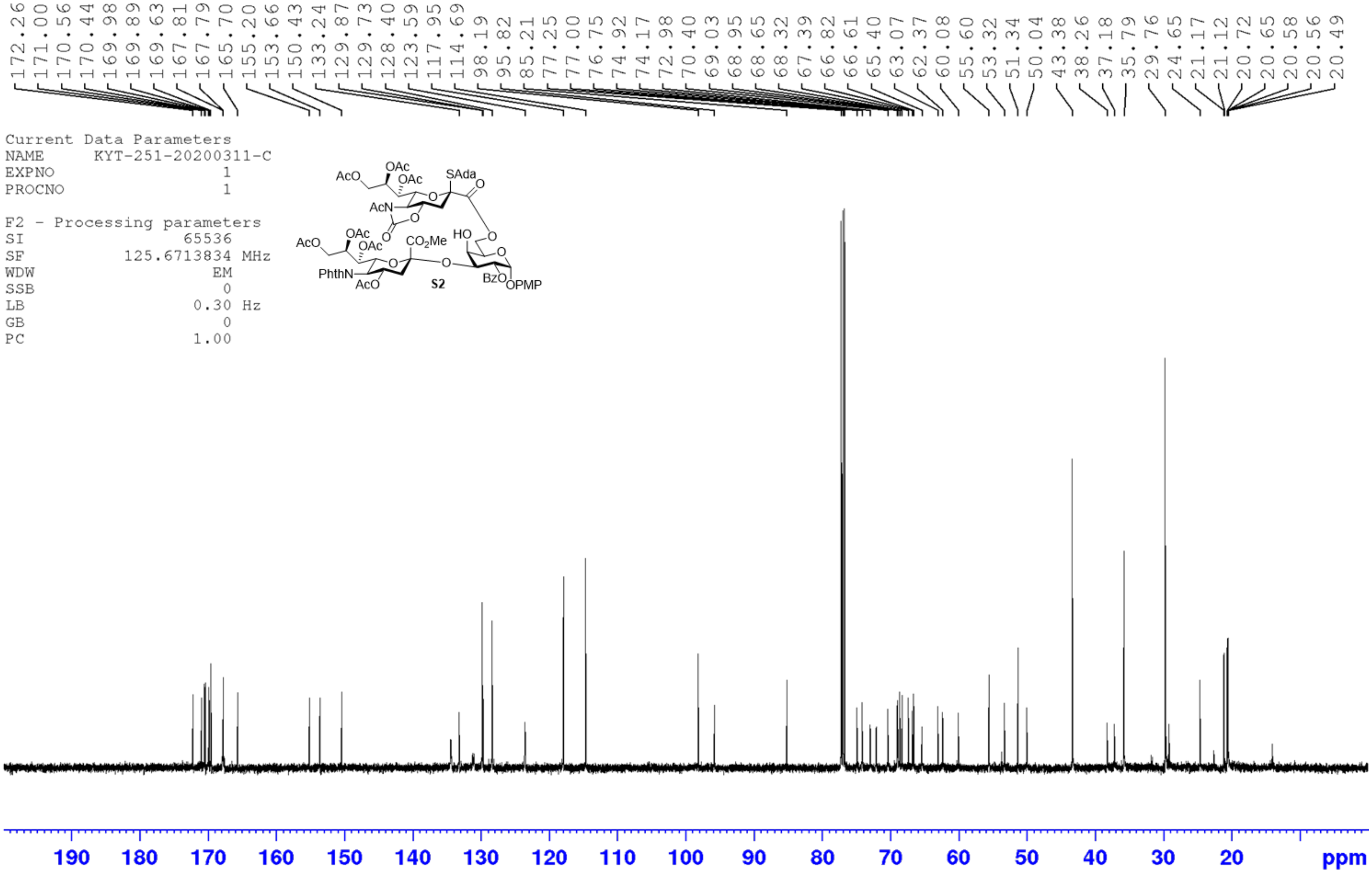

YYS-566-20221221-ACN

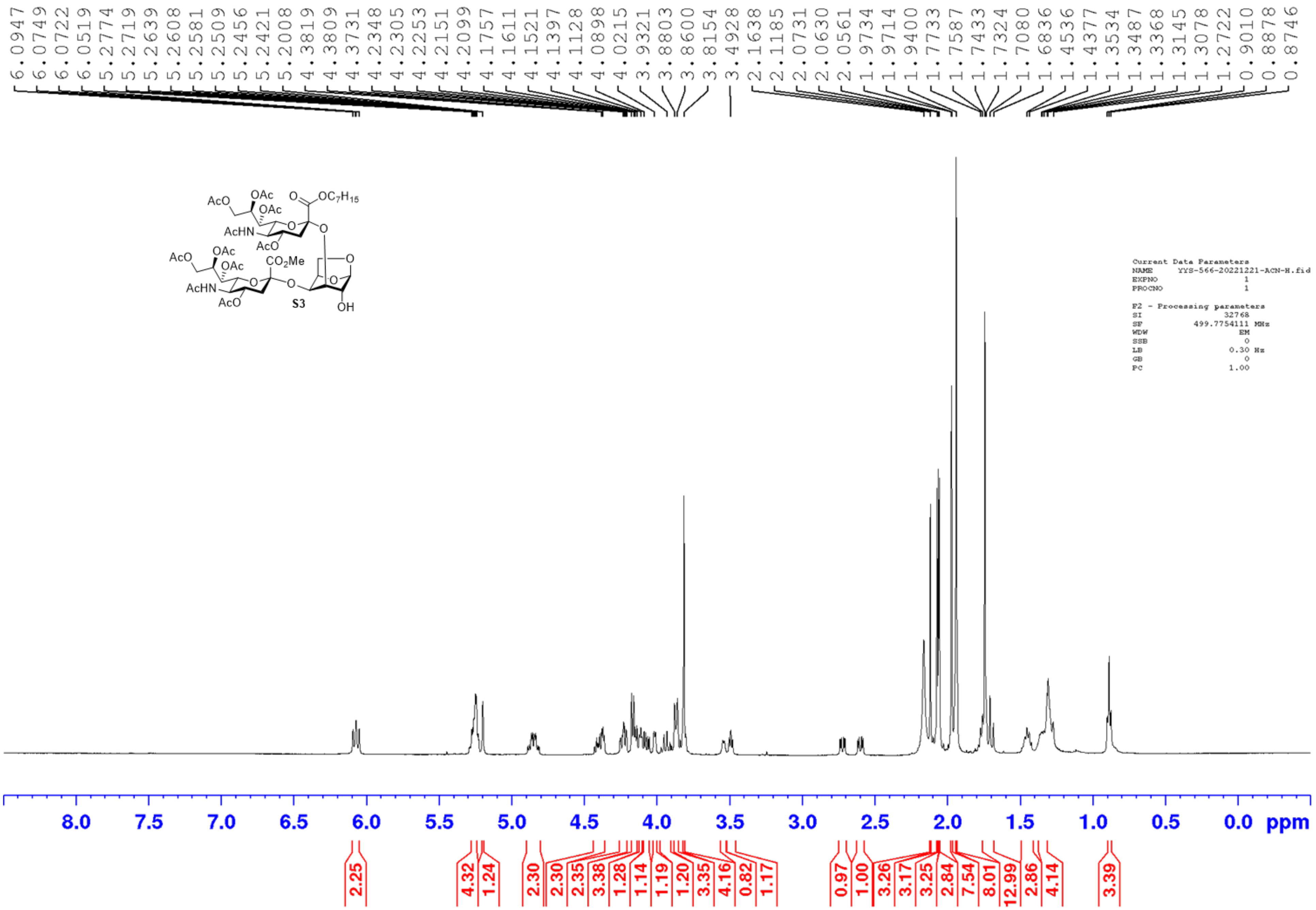

YYS-566-20221221-ACN-C

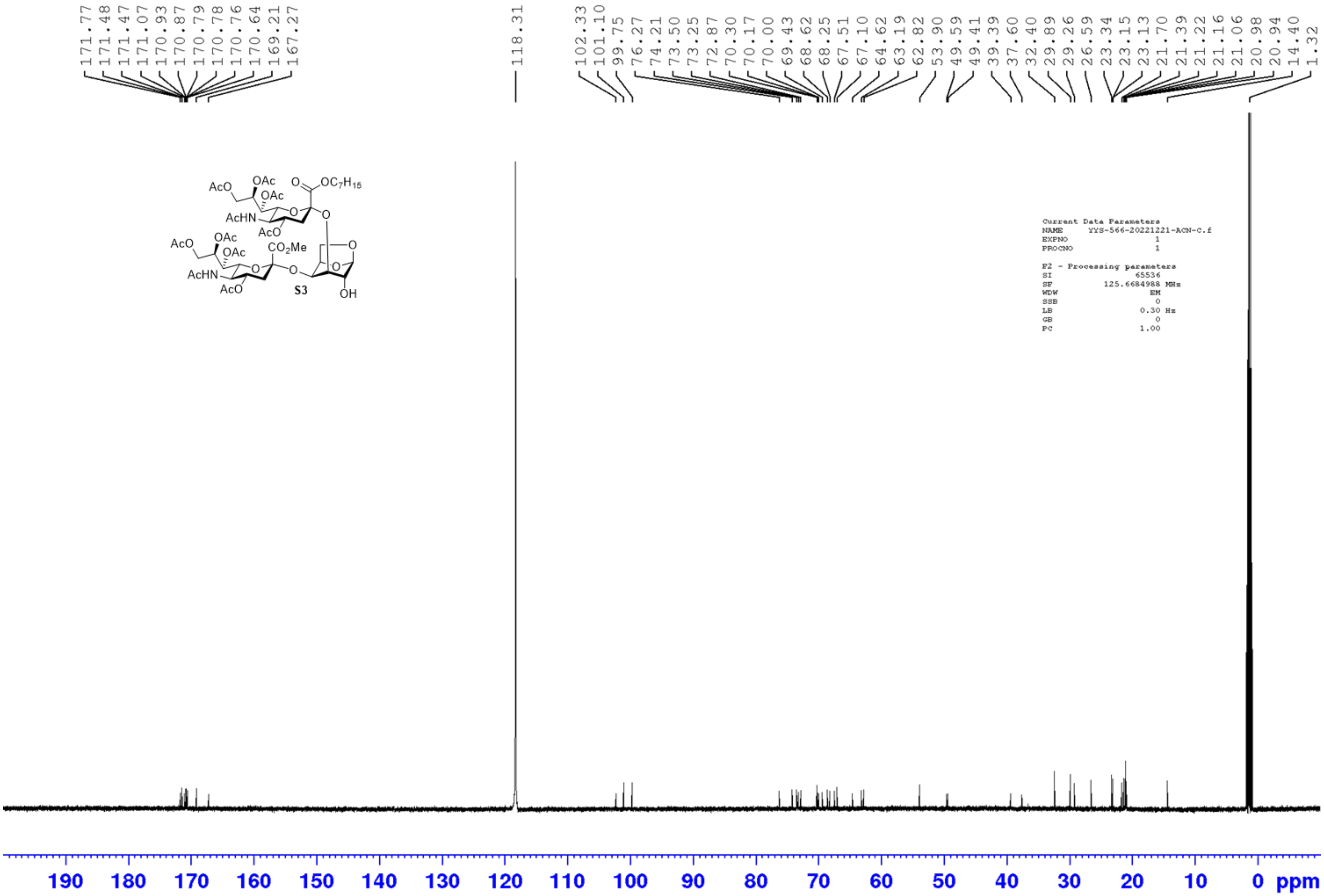

YYS-595-20230510-ACN-H

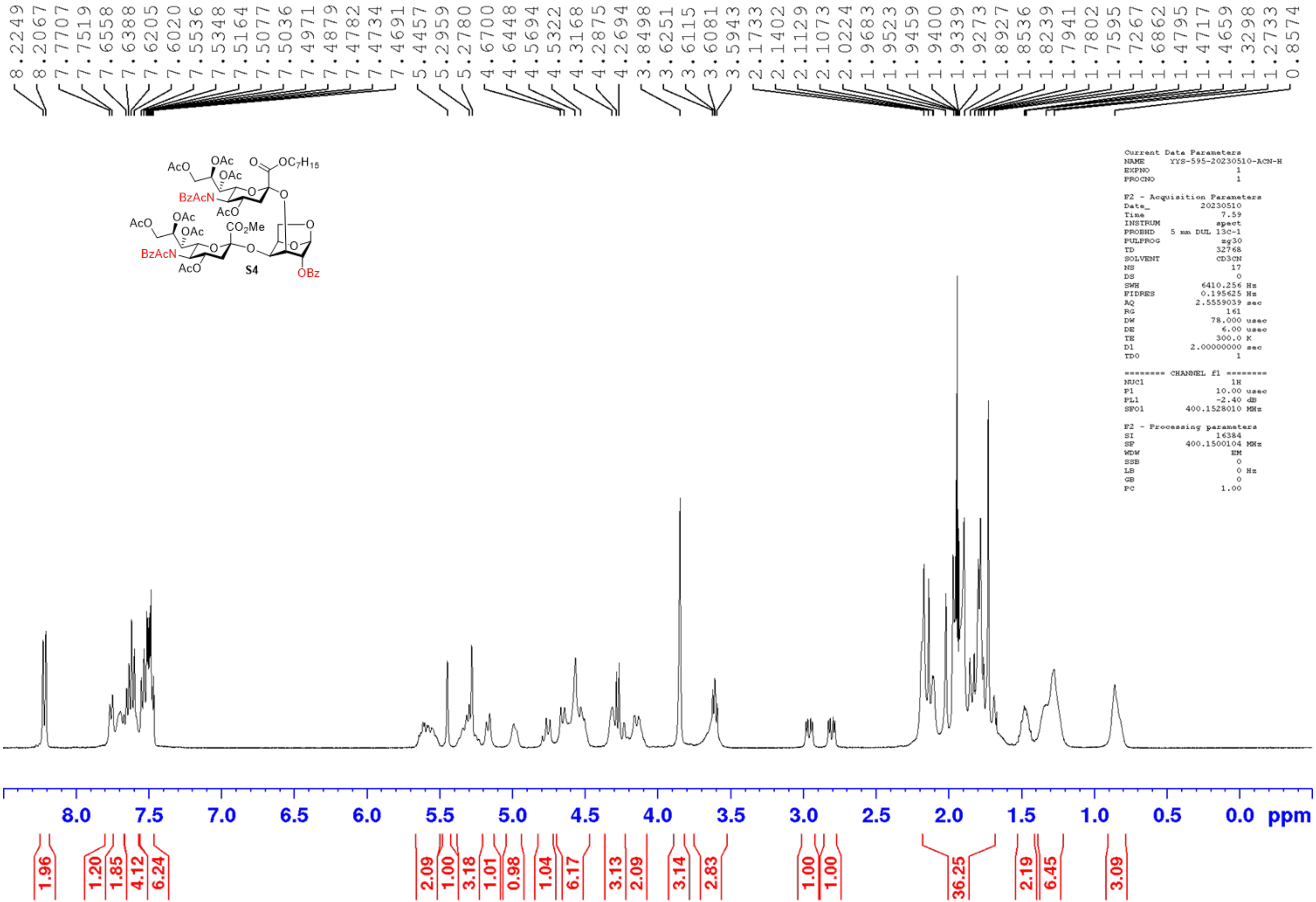

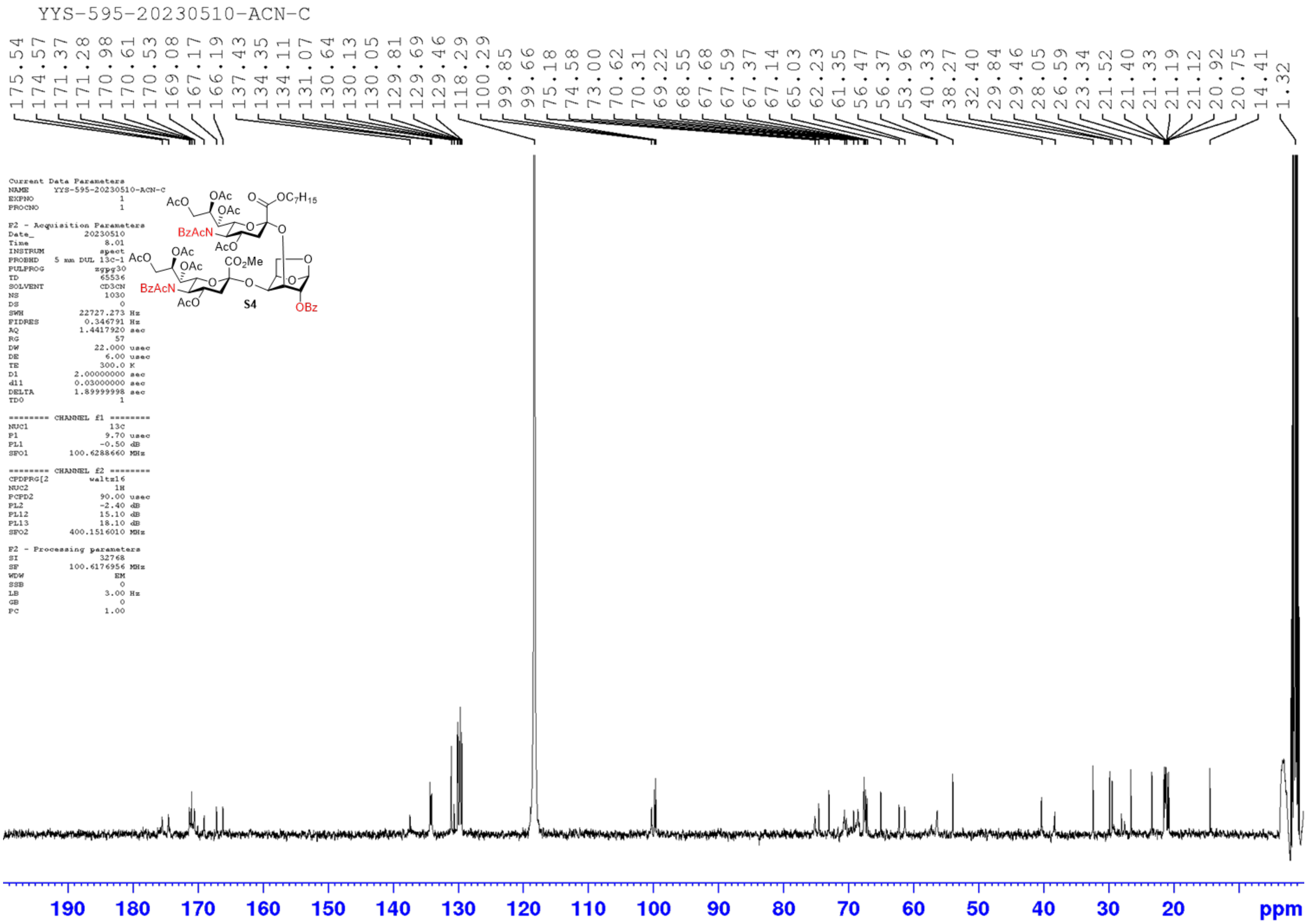

YYS-595-20230510-ACN-C
175.54
174.57
171.37
171.28
170.98
170.61
170.53
169.08
167.17
166.19
137.43
134.35
134.11
131.07
130.64
130.13
130.05
129.81
129.69
129.46
118.29
100.29
99.85
99.66
75.18
74.58
73.00
70.62
70.31
69.22
68.55
67.68
67.59
67.37
67.14
65.03
62.23
61.35
56.47
56.37
53.96
40.33
38.27
32.40
29.84
29.46
28.05
26.59
23.34
21.52
21.40
21.33
21.19
21.12
20.92
20.75
14.41
1.32
Current Data Parameters
NAME YYS-595-20230510-ACN-C
EXPNO 1
PROCNO 1
F2 - Acquisition Parameters
Date_ 20230510
Time 8.01
INSTRUM spect
PROBHD 5 mm DUL 13C-1
PULPROG zgpg30
TD 65536
SOLVENT CD3CN
NS 1030
DS 0
SWH 22727.273 Hz
FIDRES 0.346791 Hz
AQ 1.4417920 sec
RG 57
DW 22.000 usec
DE 6.00 usec
TE 300.0 K
D1 2.00000000 sec
d11 0.03000000 sec
DELTA 1.89999998 sec
TD0 1
======== CHANNEL f1 ========
NUC1 13C
P1 9.70 usec
PL1 -0.50 dB
SFO1 100.6288660 MHz
======== CHANNEL f2 ========
CPDPRG[2 waltz16
NUC2 1H
PCPD2 90.00 usec
PL2 -2.40 dB
PL12 15.10 dB
PL13 18.10 dB
SFO2 400.1516010 MHz
F2 - Processing parameters
SI 32768
SF 100.6176956 MHz
WDW EM
SSB 0
LB 3.00 Hz
GB 0
PC 1.00
AcO
OAc
OAc
O
OC7H15
BzAcN
AcO
CO2Me
OBz
S4
190
180
170
160
150
140
130
120
110
100
90
80
70
60
50
40
30
20
ppm

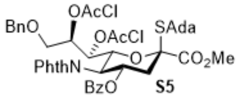
BnO
OAcCl
OAcCl
SAda
PhthN
O
CO_2Me
BzO
S5

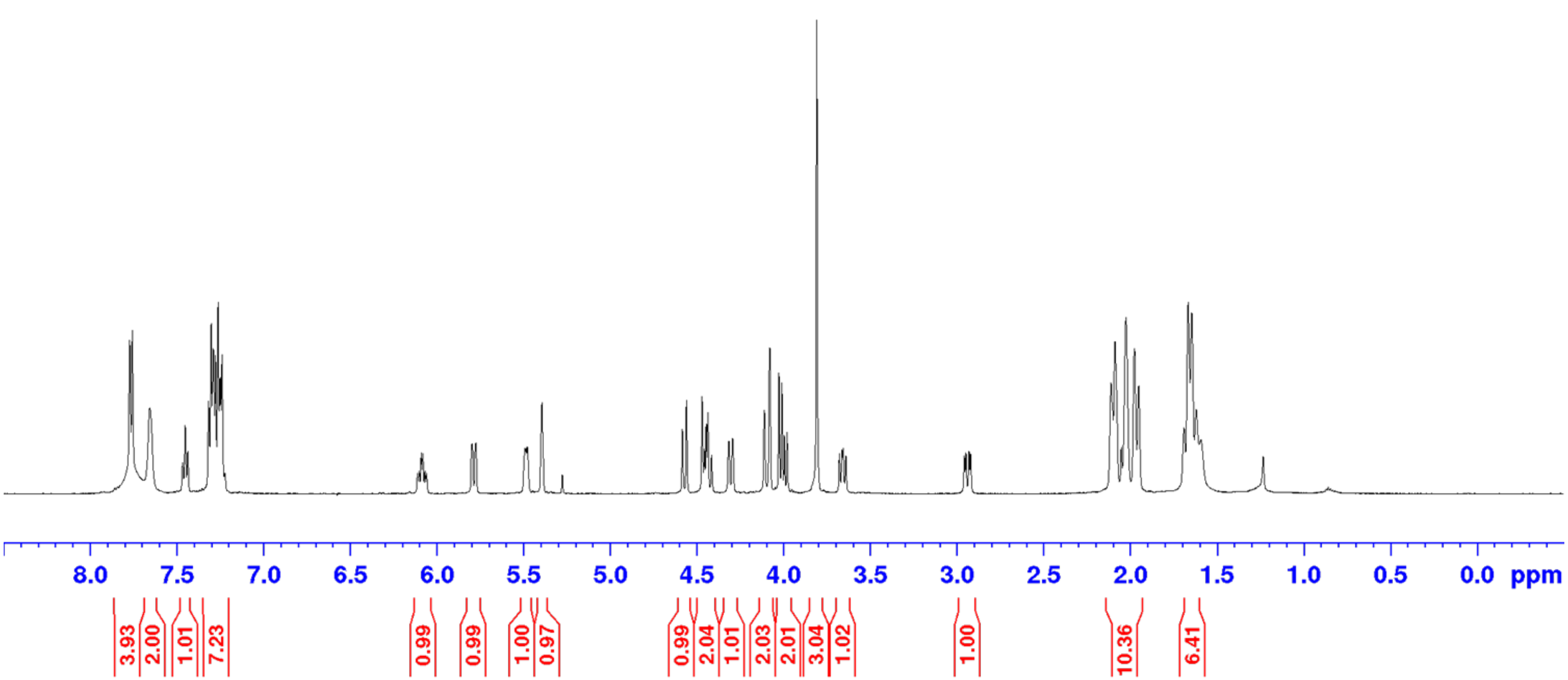
YYS-248-20201218-H
7.7734
7.7577
7.6570
7.4668
7.4522
7.4376
7.3176
7.3029
7.2904
7.2777
7.2630
7.2499
7.2400
6.1016
6.0900
6.0806
6.0690
5.7961
5.7750
5.4932
5.4867
5.4789
5.3944
4.5841
4.5605
4.4702
4.4572
4.4465
4.4363
4.4153
4.3154
4.2937
4.1101
4.0791
4.0269
4.0110
3.9960
3.9800
3.8085
3.6780
3.6628
3.6563
3.6411
2.9567
2.9474
2.9297
2.9204
2.1104
2.0873
2.0512
2.0239
1.9754
1.9520
1.6899
1.6654
1.6444
1.6184
1.5919
Current Data Parameters
NAME YYS-248-20201218-H.fi
EXPNO 1
PROCNO 1
F2 - Processing parameters
SI 16384
SF 499.7850506 MHz
WDW EM
SSB 0
LB 0.30 Hz
GB 0
PC 1.00
8.0
7.5
7.0
6.5
6.0
5.5
5.0
4.5
4.0
3.5
3.0
2.5
2.0
1.5
1.0
0.5
0.0
ppm
3.93
2.00
1.01
7.23
0.99
0.99
1.00
0.97
0.99
2.04
1.01
2.03
2.01
3.04
1.02
1.00
10.36
6.41

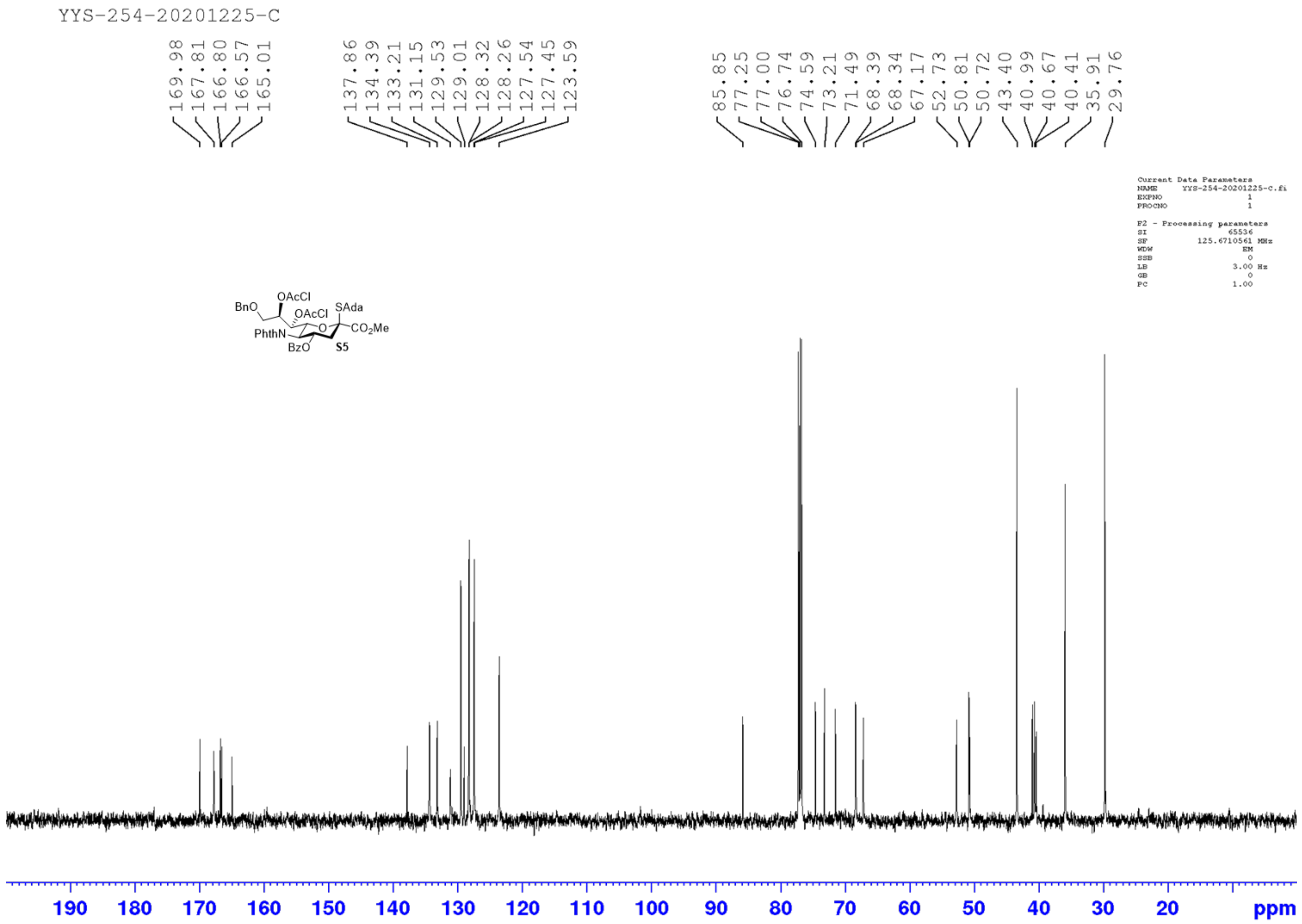
YYS-254-20201225-C
169.98
167.81
166.80
166.57
165.01
137.86
134.39
133.21
131.15
129.53
129.01
128.32
128.26
127.54
127.45
123.59
85.85
77.25
77.00
76.74
74.59
73.21
71.49
68.39
68.34
67.17
52.73
50.81
50.72
43.40
40.99
40.67
40.41
35.91
29.76
Current Data Parameters
NAME YYS-254-20201225-C.fi
EXPNO 1
PROCNO 1
F2 - Processing parameters
SI 65536
SF 125.6710561 MHz
WDW EM
SSB 0
LB 3.00 Hz
GB 0
PC 1.00
OAcCl
BnO
OAcCl
SAda
PhthN
O
CO2Me
BzO
S5
190
180
170
160
150
140
130
120
110
100
90
80
70
60
50
40
30
20
ppm

YYS-755-20240403-C-H

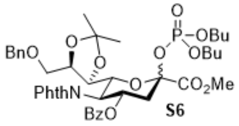

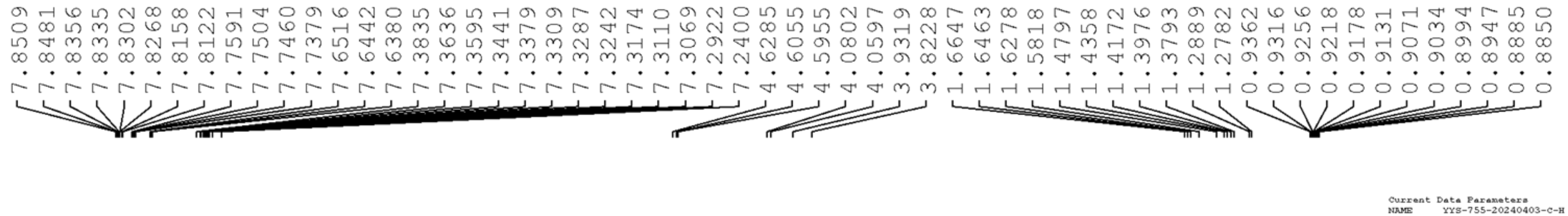

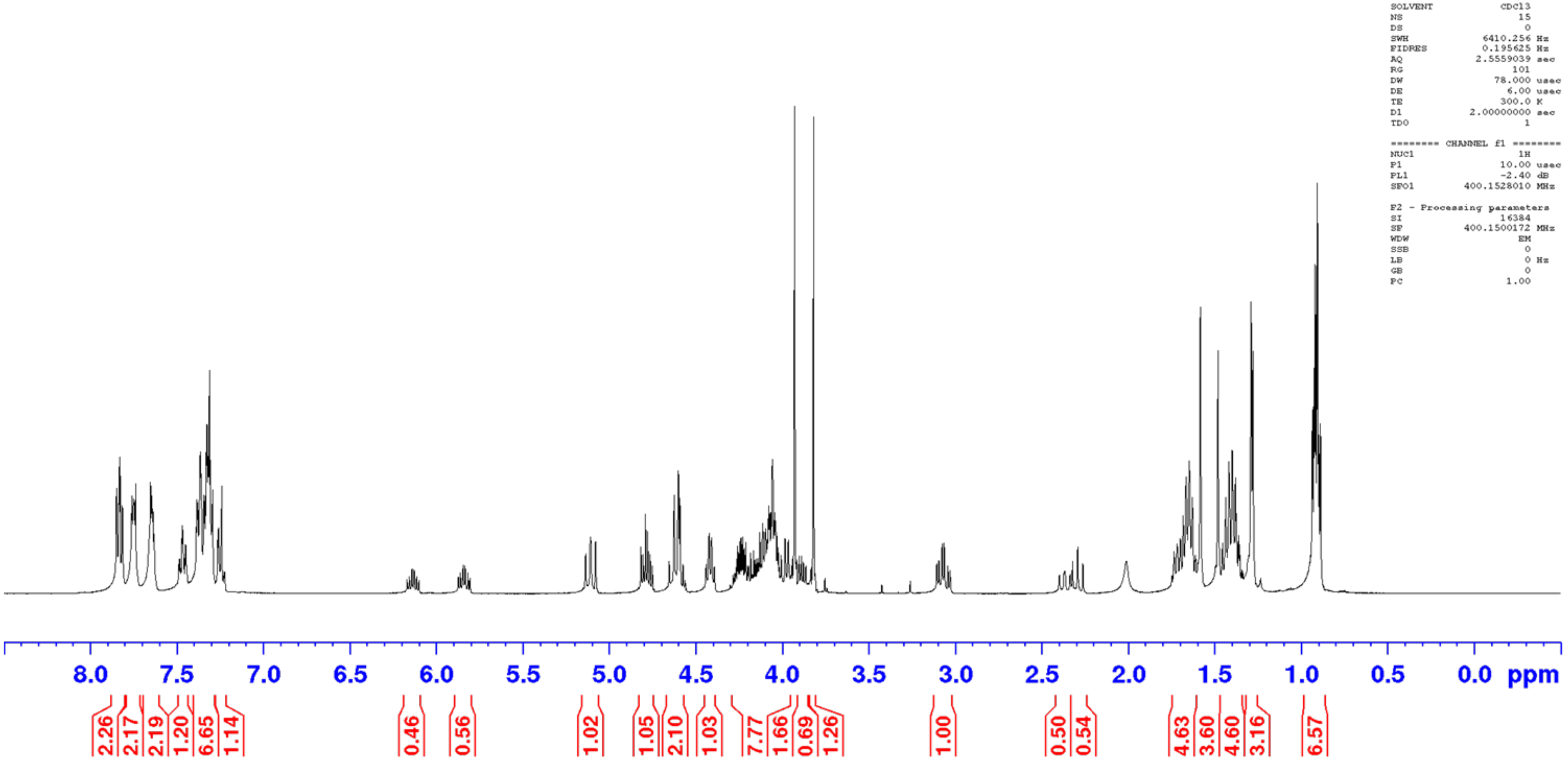

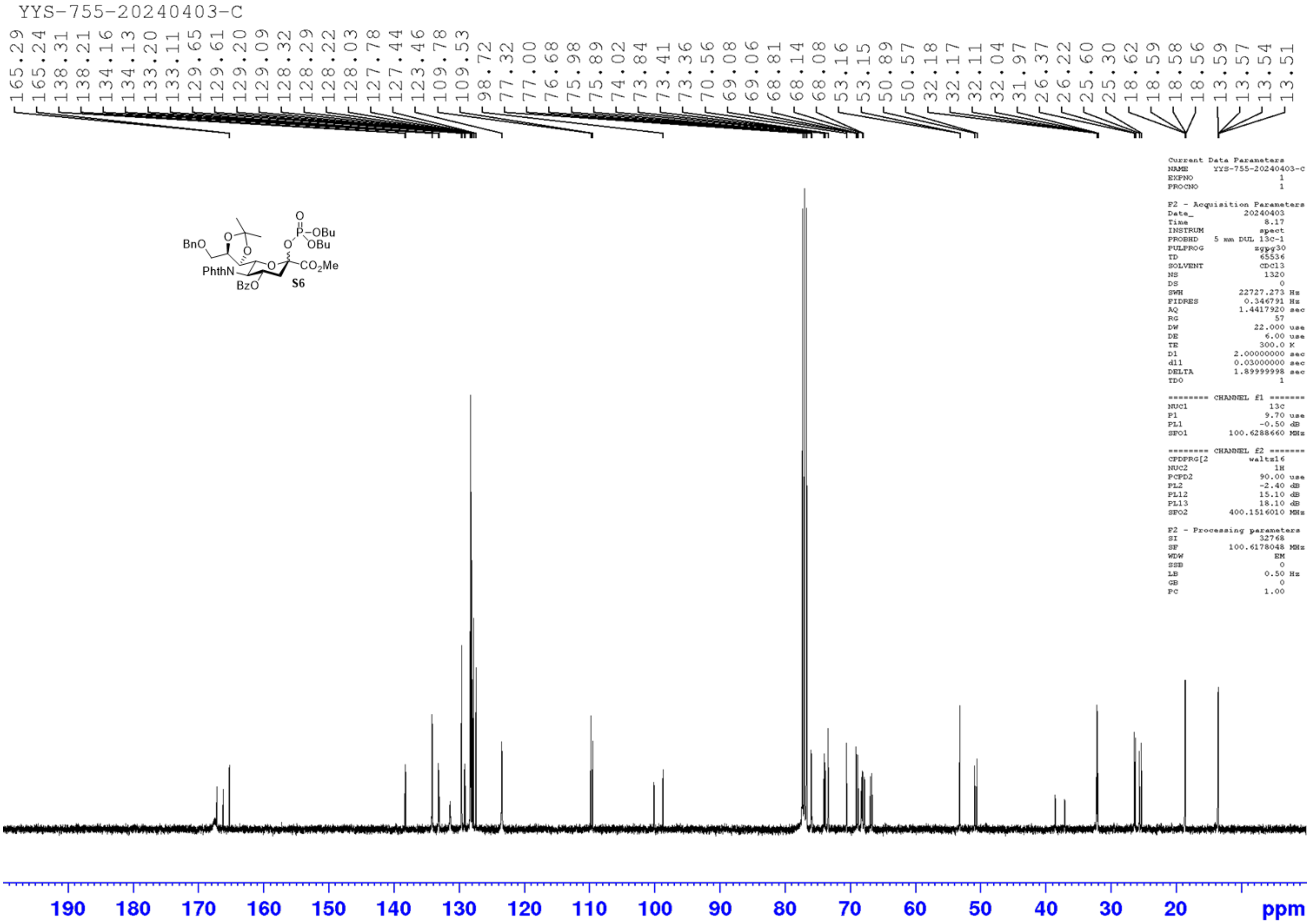
YYS-755-20240403-C
165.29
165.24
138.31
138.21
134.16
134.13
133.20
133.11
129.65
129.61
129.20
129.09
128.32
128.29
128.22
128.03
127.78
127.44
123.46
109.78
109.53
98.72
77.32
77.00
76.68
75.98
75.89
74.02
73.84
73.41
73.36
70.56
69.08
69.06
68.81
68.14
68.08
53.16
53.15
50.89
50.57
32.18
32.17
32.11
32.04
31.97
26.37
26.22
25.60
25.30
18.62
18.59
18.58
18.56
13.59
13.57
13.54
13.51
BnO
O
O
P
OBu
OBu
PhthN
BzO
CO2Me
S6
Current Data Parameters
NAME YYS-755-20240403-C
EXPNO 1
PROCNO 1
F2 - Acquisition Parameters
Date_ 20240403
Time 8.17
INSTRUM spect
PROBHD 5 mm DUL 13C-1
PULPROG zgpg30
TD 65536
SOLVENT CDCl3
NS 1320
DS 0
SWH 22727.273 Hz
FIDRES 0.346791 Hz
AQ 1.4417920 sec
RG 57
DW 22.000 usec
DE 6.00 usec
TE 300.0 K
D1 2.00000000 sec
d11 0.03000000 sec
DELTA 1.89999998 sec
TD0 1
======== CHANNEL f1 ========
NUC1 13C
P1 9.70 usec
PL1 -0.50 dB
SFO1 100.6288660 MHz
======== CHANNEL f2 ========
CPDPRG[2 waltz16
NUC2 1H
PCPD2 90.00 usec
PL2 -2.40 dB
PL12 15.10 dB
PL13 18.10 dB
SFO2 400.1516010 MHz
F2 - Processing parameters
SI 32768
SF 100.6178048 MHz
WDW EM
SSB 0
LB 0.50 Hz
GB 0
PC 1.00
190
180
170
160
150
140
130
120
110
100
90
80
70
60
50
40
30
20
ppm

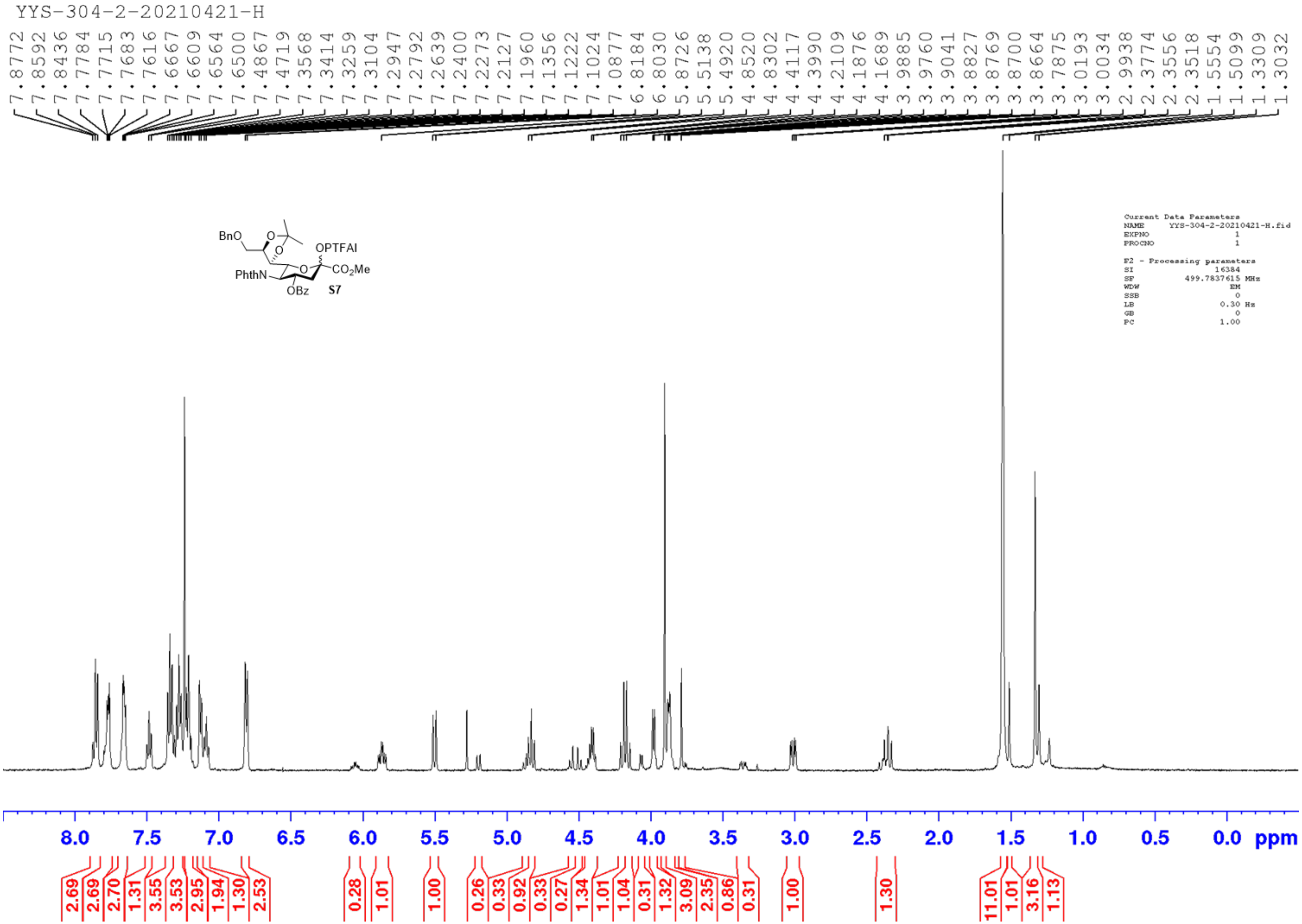
YYS-304-2-20210421-H
7.8772 7.8592 7.8436 7.7784 7.7715 7.7683 7.7616 7.6667 7.6609 7.6564 7.6500 7.4867 7.4719 7.3568 7.3414 7.3259 7.3104 7.2947 7.2792 7.2639 7.2400 7.2273 7.2127 7.1960 7.1356 7.1222 7.1024 7.0877 6.8184 6.8030 5.8726 5.5138 5.4920 4.8520 4.8302 4.4117 4.3990 4.2109 4.1876 4.1689 3.9885 3.9760 3.9041 3.8827 3.8769 3.8700 3.8664 3.7875 3.0193 3.0034 2.9938 2.3774 2.3556 2.3518 1.5554 1.5099 1.3309 1.3032
BnO
OPTFAI
CO_2Me
PhthN
OBz
S7
Current Data Parameters
NAME YYS-304-2-20210421-H.fid
EXPNO 1
PROCNO 1
F2 - Processing parameters
SI 16384
SF 499.7837615 MHz
WDW EM
SSB 0
LB 0.30 Hz
GB 0
PC 1.00
8.0 7.5 7.0 6.5 6.0 5.5 5.0 4.5 4.0 3.5 3.0 2.5 2.0 1.5 1.0 0.5 0.0 ppm
2.69 2.69 2.70 1.31 3.55 3.53 2.95 1.94 1.30 2.53 0.28 1.01 1.00 0.26 0.33 0.92 0.33 0.27 1.34 1.01 1.04 0.31 1.32 3.09 2.35 0.86 0.31 1.00 1.30 11.01 1.01 3.16 1.13

YYS-304-1-20210421-C

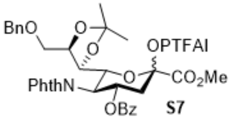

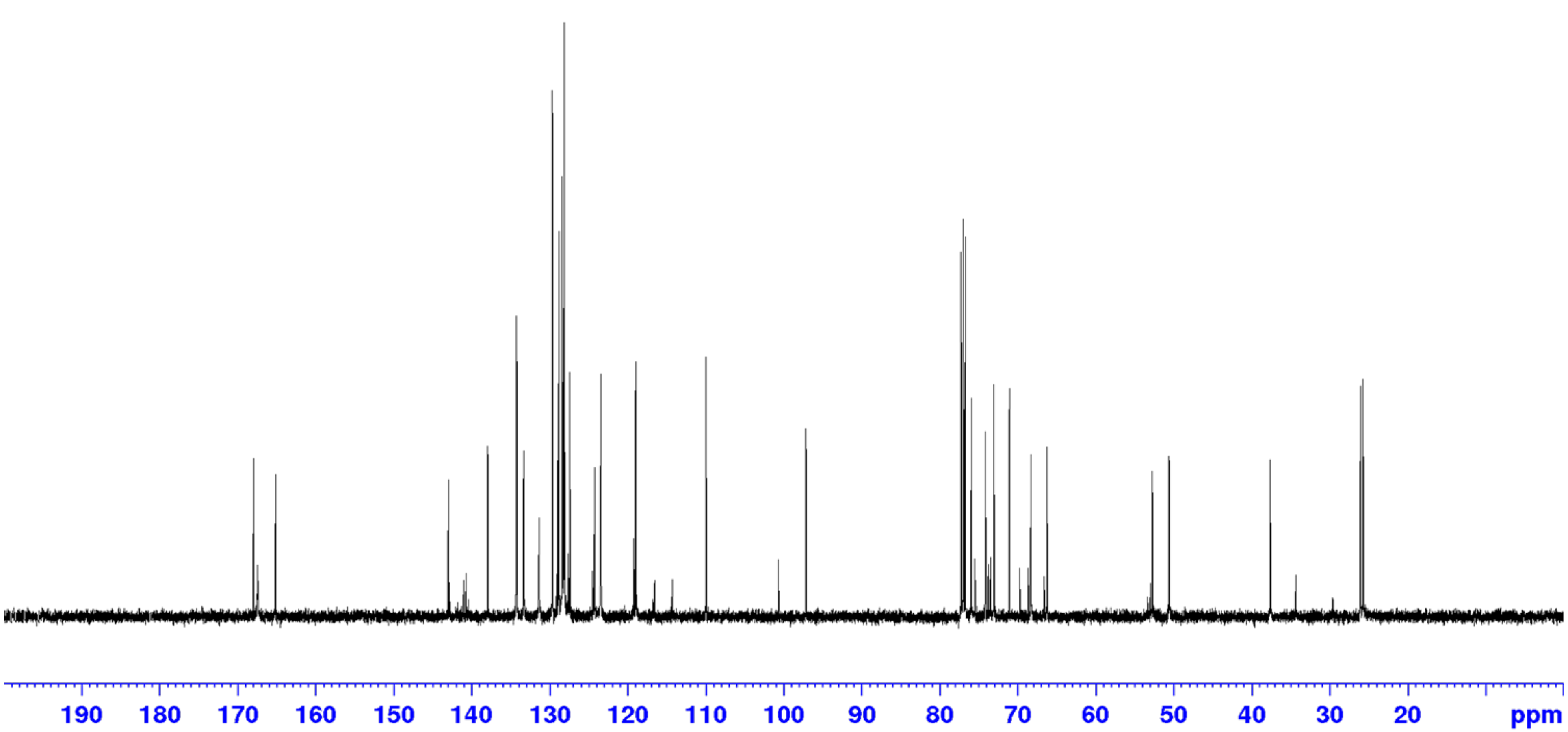

YYS-291-20210411-cosy-H

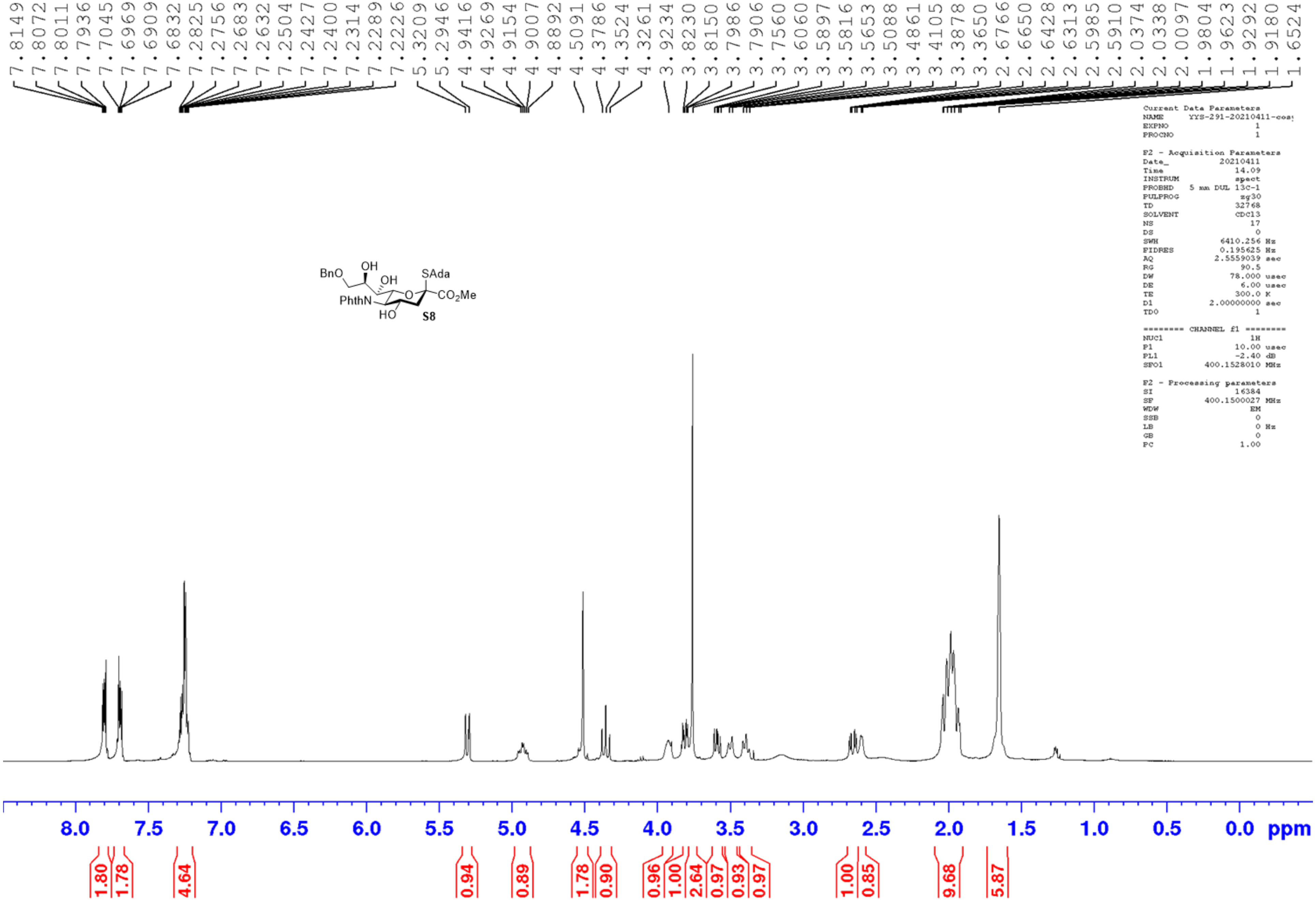

YYS-291-20210408-C

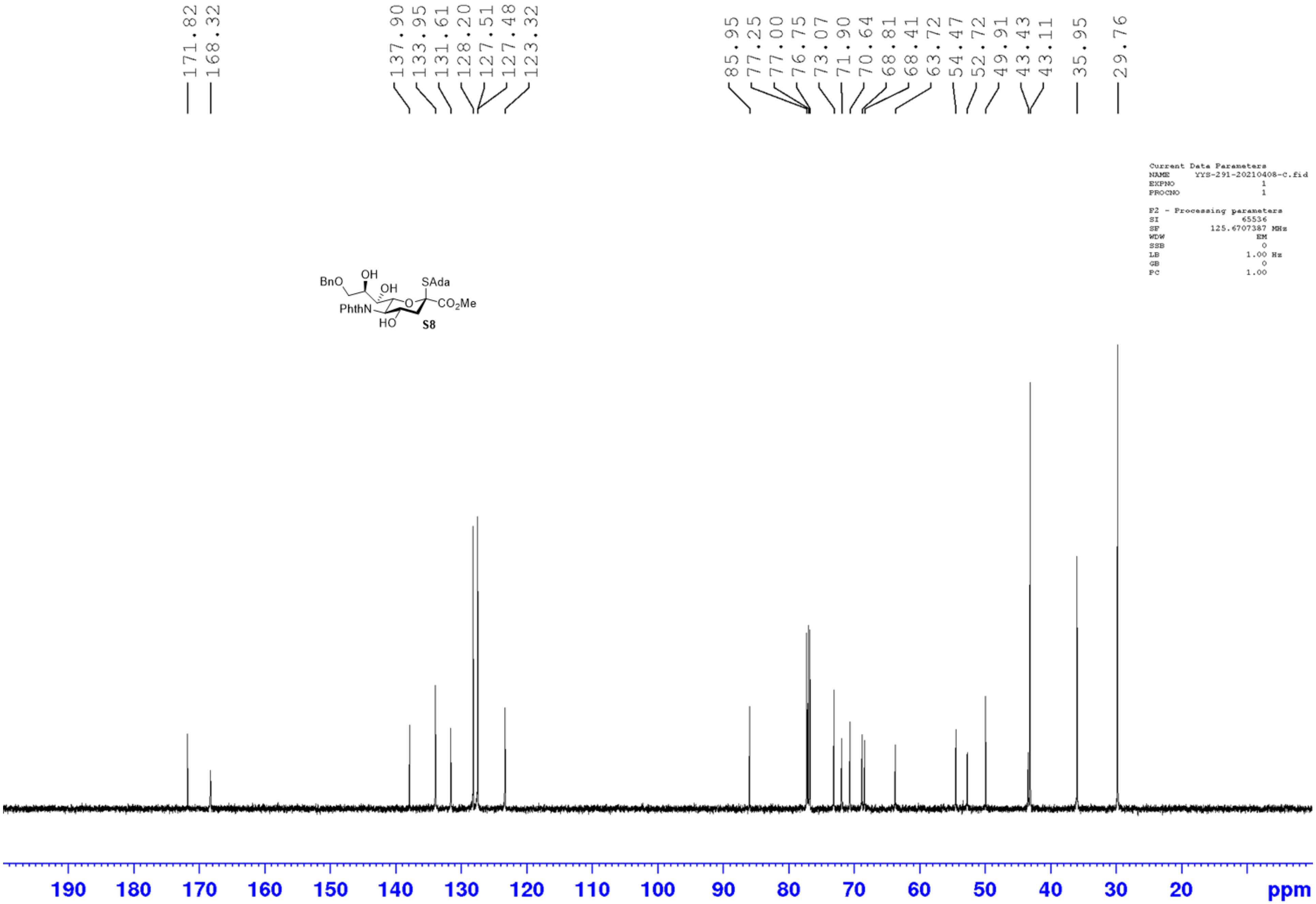

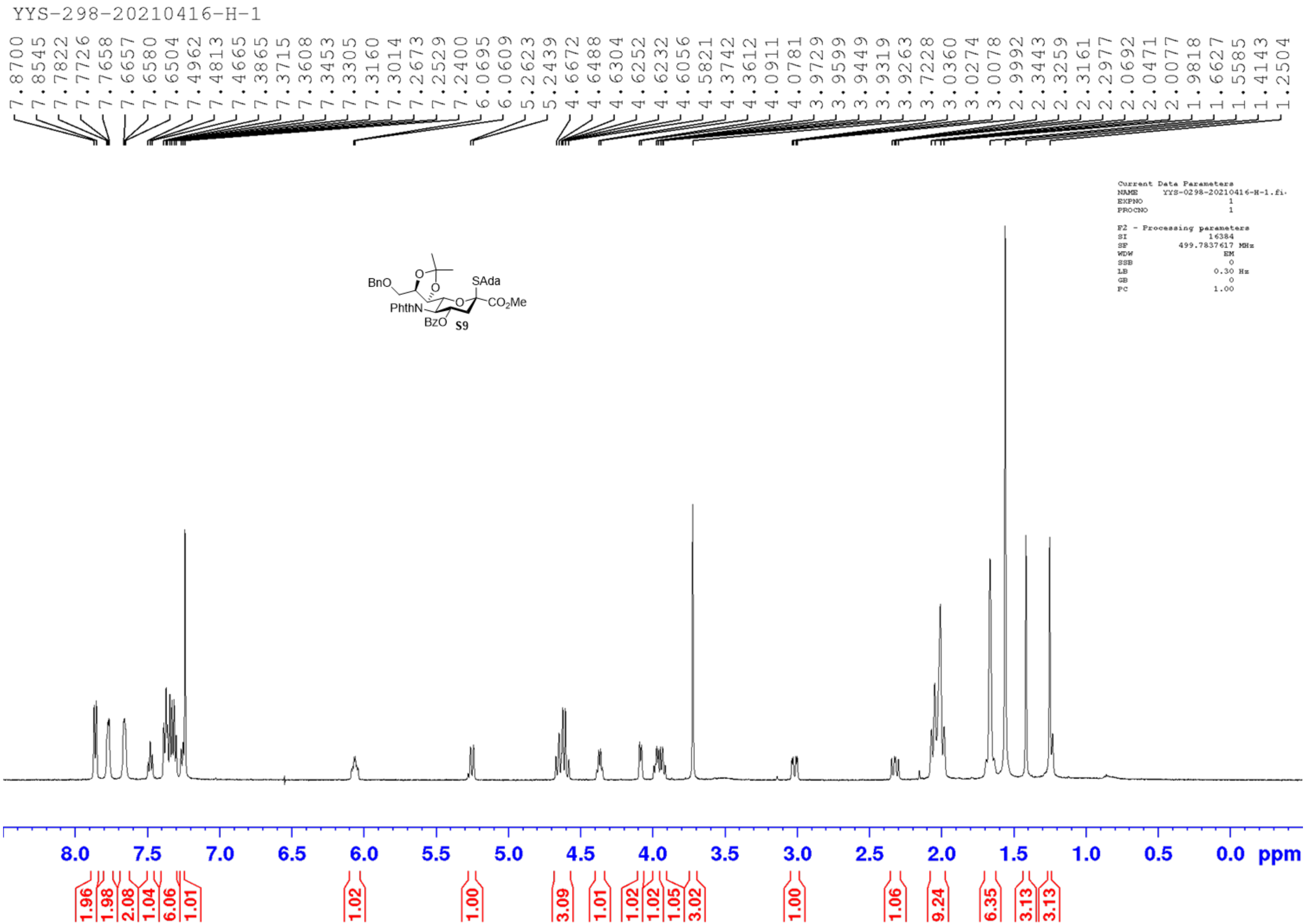
YYS-298-20210416-H-1
7.8700 7.8545 7.7822 7.7726 7.7658 7.6657 7.6580 7.6504 7.4962 7.4813 7.4665 7.3865 7.3715 7.3608 7.3453 7.3305 7.3160 7.3014 7.2673 7.2529 7.2400 6.0695 6.0609 5.2623 5.2439 4.6672 4.6488 4.6304 4.6252 4.6232 4.6056 4.5821 4.3742 4.3612 4.0911 4.0781 3.9729 3.9599 3.9449 3.9319 3.9263 3.7228 3.0360 3.0274 3.0078 2.9992 2.3443 2.3259 2.3161 2.2977 2.0692 2.0471 2.0077 1.9818 1.6627 1.5585 1.4143 1.2504
Current Data Parameters
NAME YYS-0298-20210416-H-1.fi
EXPNO 1
PROCNO 1
F2 - Processing parameters
SI 16384
SF 499.7837617 MHz
WDW EM
SSB 0
LB 0.30 Hz
GB 0
PC 1.00
BnO
SAda
PhthN
O
CO2Me
BzO
S9
8.0 7.5 7.0 6.5 6.0 5.5 5.0 4.5 4.0 3.5 3.0 2.5 2.0 1.5 1.0 0.5 0.0 ppm
1.96 1.98 2.08 1.04 6.06 1.01 1.02 1.00 3.09 1.01 1.02 1.02 1.05 3.02 1.00 1.06 9.24 6.35 3.13 3.13

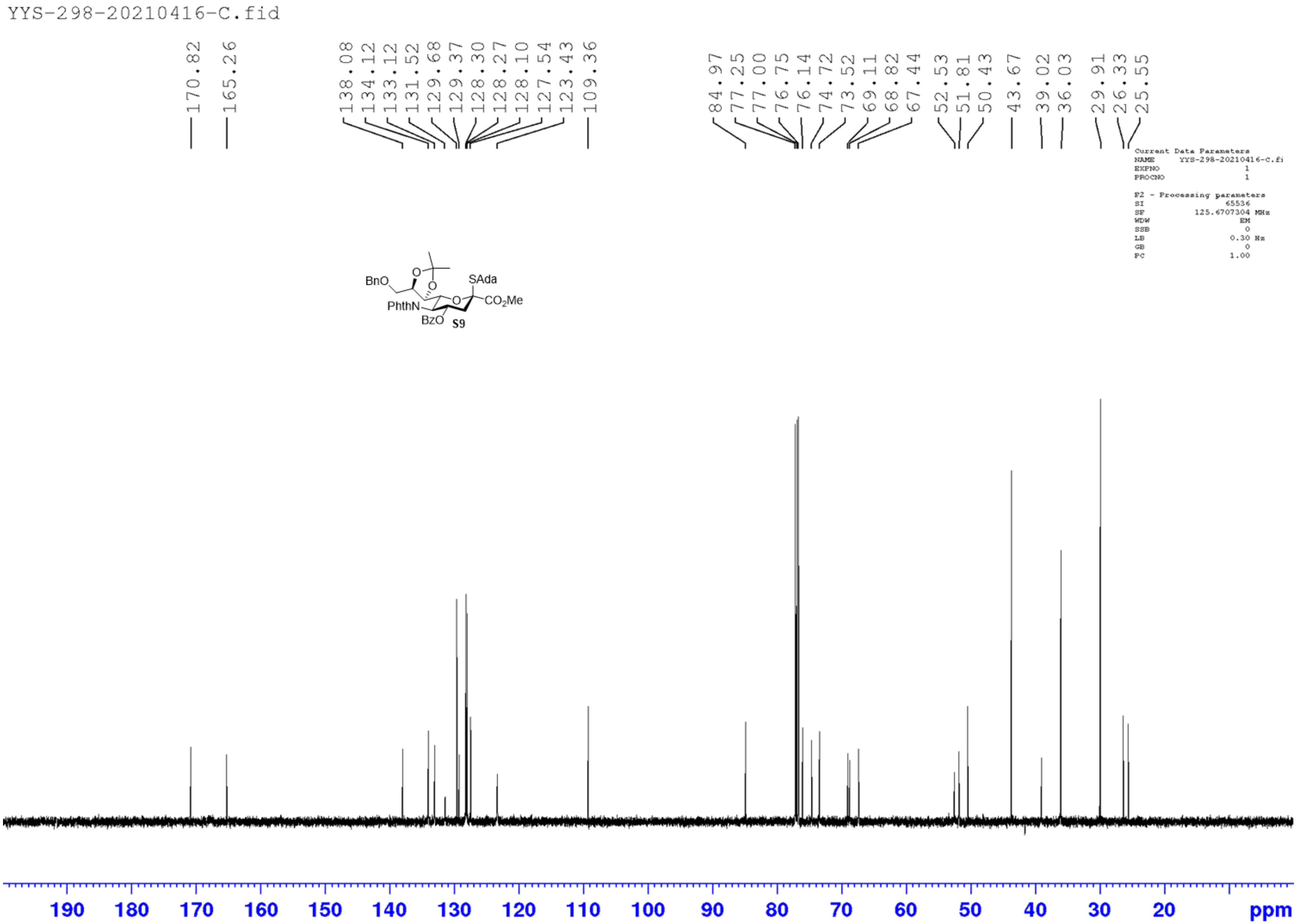
YYS-298-20210416-C.fid
170.82
165.26
138.08
134.12
133.12
131.52
129.68
129.37
128.30
128.27
128.10
127.54
123.43
109.36
84.97
77.25
77.00
76.75
76.14
74.72
73.52
69.11
68.82
67.44
52.53
51.81
50.43
43.67
39.02
36.03
29.91
26.33
25.55
Current Data Parameters
NAME YYS-298-20210416-C.fi
EXPNO 1
PROCNO 1
F2 - Processing parameters
SI 65536
SF 125.6707304 MHz
WDW EM
SSB 0
LB 0.30 Hz
GB 0
PC 1.00
BnO
SAda
PhthN
CO2Me
BzO
S9
190 180 170 160 150 140 130 120 110 100 90 80 70 60 50 40 30 20 ppm

YYS-605-20230420-H-1

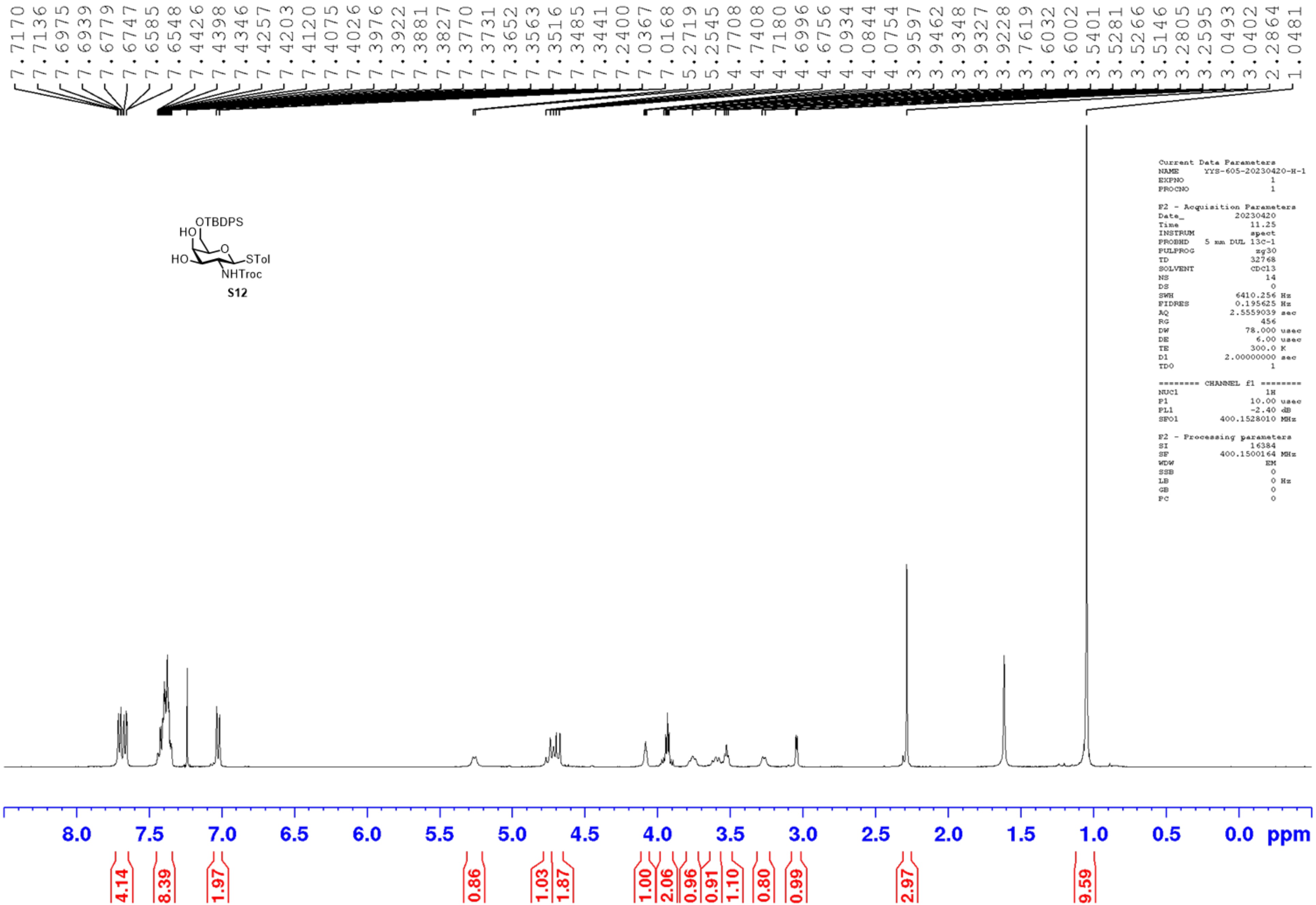

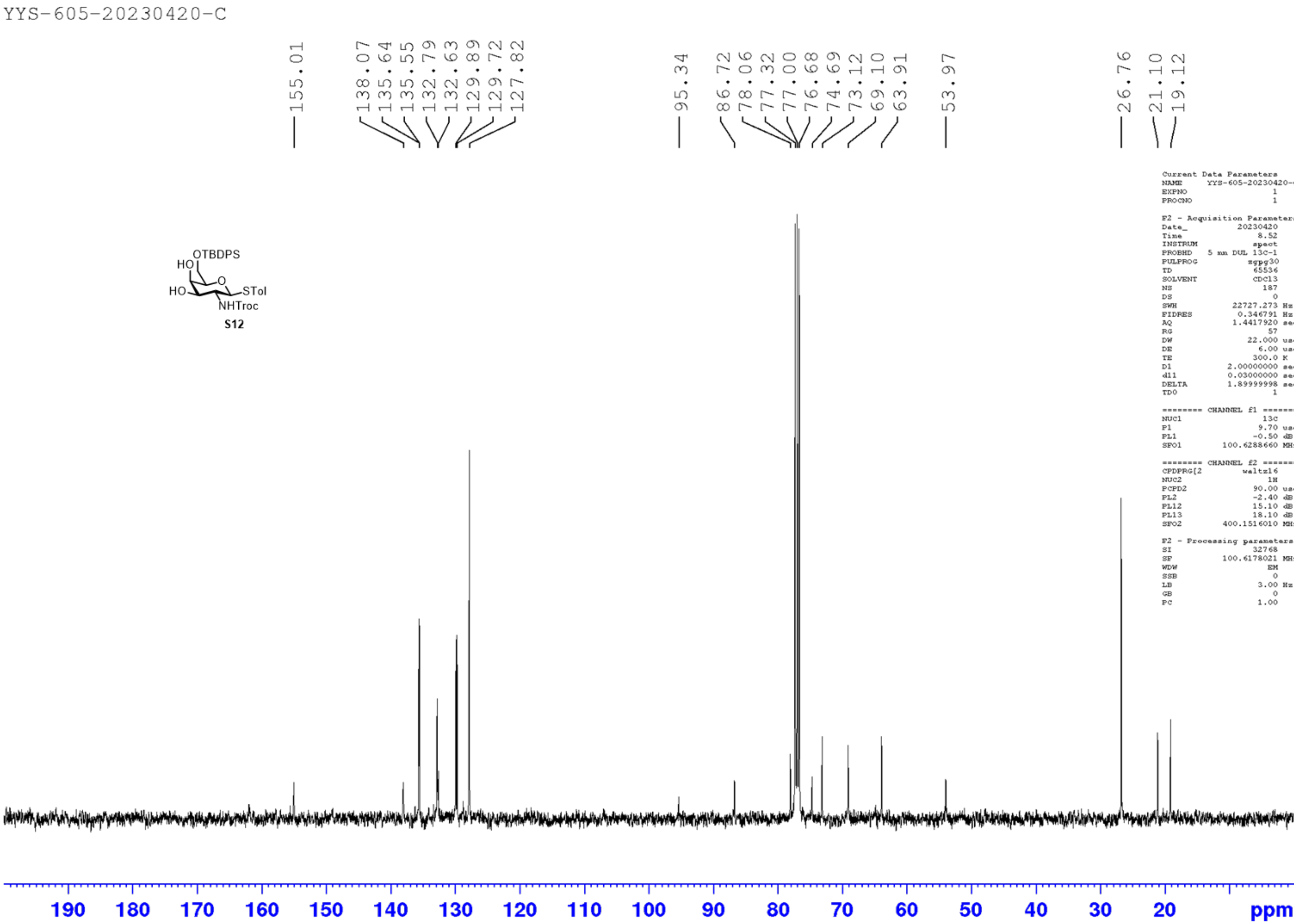
YYS-605-20230420-C
155.01
138.07
135.64
135.55
132.79
132.63
129.89
129.72
127.82
95.34
86.72
78.06
77.32
77.00
76.68
74.69
73.12
69.10
63.91
53.97
26.76
21.10
19.12
OTBDPS
HO
O
HO
STol
NHTroc
S12
Current Data Parameters
NAME YYS-605-20230420-
EXPNO 1
PROCNO 1
F2 - Acquisition Parameters
Date_ 20230420
Time 8.52
INSTRUM spect
PROBHD 5 mm DUL 13C-1
PULPROG zgpg30
TD 65536
SOLVENT CDCl3
NS 187
DS 0
SWH 22727.273 Hz
FIDRES 0.346791 Hz
AQ 1.4417920 sec
RG 57
DW 22.000 usec
DE 6.00 usec
TE 300.0 K
D1 2.00000000 sec
d11 0.03000000 sec
DELTA 1.89999998 sec
TD0 1
======== CHANNEL f1 ========
NUC1 13C
P1 9.70 usec
PL1 -0.50 dB
SFO1 100.6288660 MHz
======== CHANNEL f2 ========
CPDPRG[2 waltz16
NUC2 1H
PCPD2 90.00 usec
PL2 -2.40 dB
PL12 15.10 dB
PL13 18.10 dB
SFO2 400.1516010 MHz
F2 - Processing parameters
SI 32768
SF 100.6178021 MHz
WDW EM
SSB 0
LB 3.00 Hz
GB 0
PC 1.00
190
180
170
160
150
140
130
120
110
100
90
80
70
60
50
40
30
20
ppm

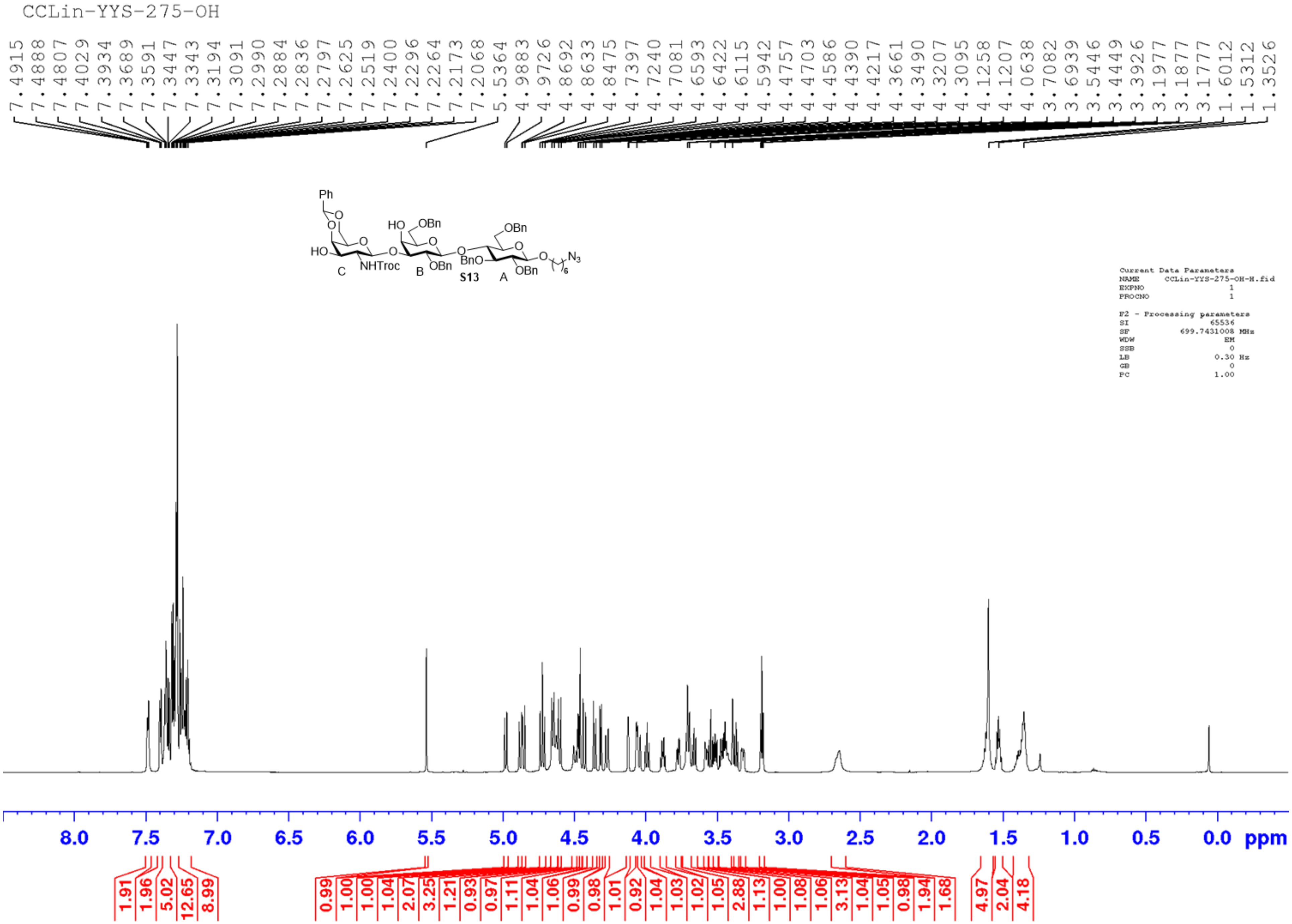

CCLin-YYS-275-OH
7.4915
7.4888
7.4807
7.4029
7.3934
7.3689
7.3591
7.3447
7.3343
7.3194
7.3091
7.2990
7.2884
7.2836
7.2797
7.2625
7.2519
7.2400
7.2296
7.2264
7.2173
7.2068
5.5364
4.9883
4.9726
4.8692
4.8633
4.8475
4.7397
4.7240
4.7081
4.6593
4.6422
4.6115
4.5942
4.4757
4.4703
4.4586
4.4390
4.4217
4.3661
4.3490
4.3207
4.3095
4.1258
4.1207
4.0638
3.7082
3.6939
3.5446
3.4449
3.3926
3.1977
3.1877
3.1777
1.6012
1.5312
1.3526
Ph
O
HO
OBn
HO
NHTroc
OBn
BnO
OBn
N3
C
B
S13
A
Current Data Parameters
NAME CCLin-YYS-275-OH-H.fid
EXPNO 1
PROCNO 1
F2 - Processing parameters
SI 65536
SF 699.7431008 MHz
WDW EM
SSB 0
LB 0.30 Hz
GB 0
PC 1.00
8.0 7.5 7.0 6.5 6.0 5.5 5.0 4.5 4.0 3.5 3.0 2.5 2.0 1.5 1.0 0.5 0.0 ppm
1.91
1.96
5.02
12.65
8.99
0.99
1.00
1.00
1.04
2.07
3.25
1.21
0.93
0.97
1.11
1.04
1.06
0.99
0.98
1.01
0.92
1.04
1.03
1.02
1.05
2.88
1.13
1.00
1.08
1.06
3.13
1.04
1.05
0.98
1.94
1.68
4.97
2.04
4.18

CCLIn-YYS-275-OH

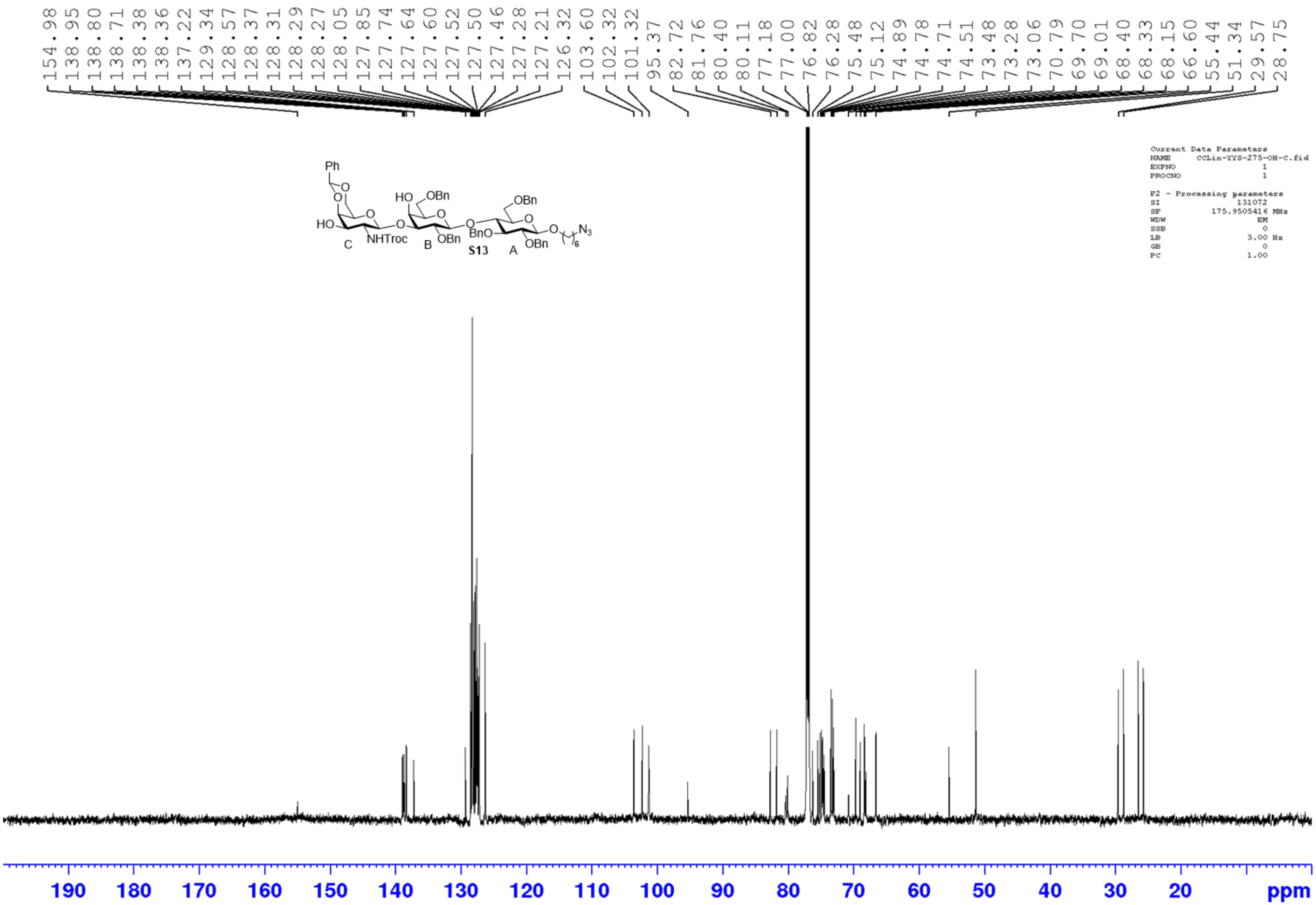

S13

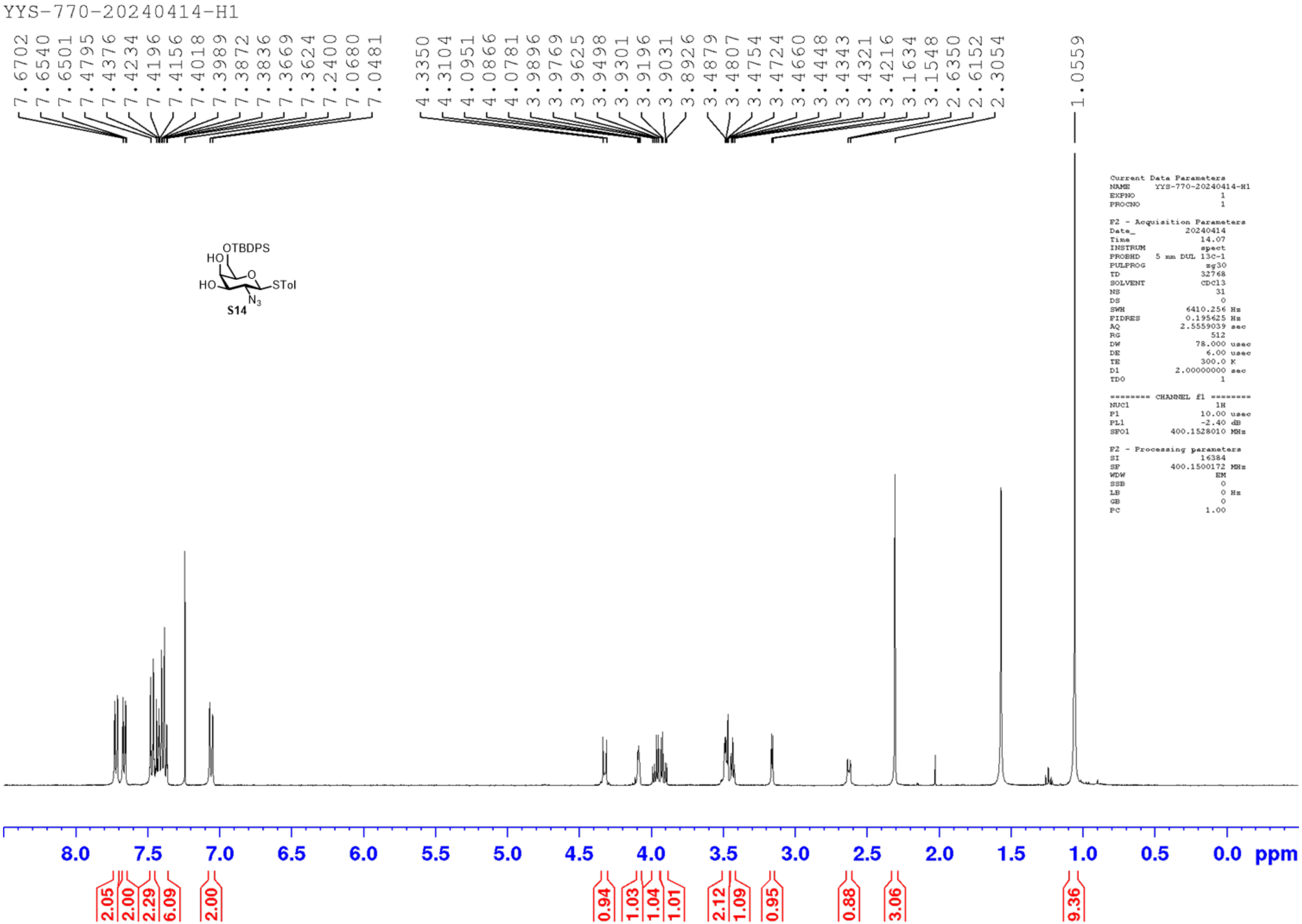

YYS-770-20240414-H1
7.6702 7.6540 7.6501 7.4795 7.4376 7.4234 7.4196 7.4156 7.4018 7.3989 7.3872 7.3836 7.3669 7.3624 7.2400 7.0680 7.0481
4.3350 4.3104 4.0951 4.0866 4.0781 3.9896 3.9769 3.9625 3.9498 3.9301 3.9196 3.9031 3.8926 3.4879 3.4807 3.4754 3.4724 3.4660 3.4448 3.4343 3.4321 3.4216 3.1634 3.1548 2.6350 2.6152 2.3054
1.0559
OTBDPS
HO
O
HO
STol
N3
S14
Current Data Parameters
NAME YYS-770-20240414-H1
EXPNO 1
PROCNO 1
F2 - Acquisition Parameters
Date_ 20240414
Time 14.07
INSTRUM spect
PROBHD 5 mm DUL 13C-1
PULPROG zg30
TD 32768
SOLVENT CDCl3
NS 31
DS 0
SWH 6410.256 Hz
FIDRES 0.195625 Hz
AQ 2.5559039 sec
RG 512
DW 78.000 usec
DE 6.00 usec
TE 300.0 K
D1 2.00000000 sec
TD0 1
======== CHANNEL f1 ========
NUC1 1H
P1 10.00 usec
PL1 -2.40 dB
SFO1 400.1528010 MHz
F2 - Processing parameters
SI 16384
SF 400.1500172 MHz
WDW EM
SSB 0
LB 0 Hz
GB 0
PC 1.00
8.0 7.5 7.0 6.5 6.0 5.5 5.0 4.5 4.0 3.5 3.0 2.5 2.0 1.5 1.0 0.5 0.0 ppm
2.05 2.00 2.29 6.09 2.00 0.94 1.03 1.04 1.01 2.12 1.09 0.95 0.88 3.06 9.36

YYS-770-20240414-C

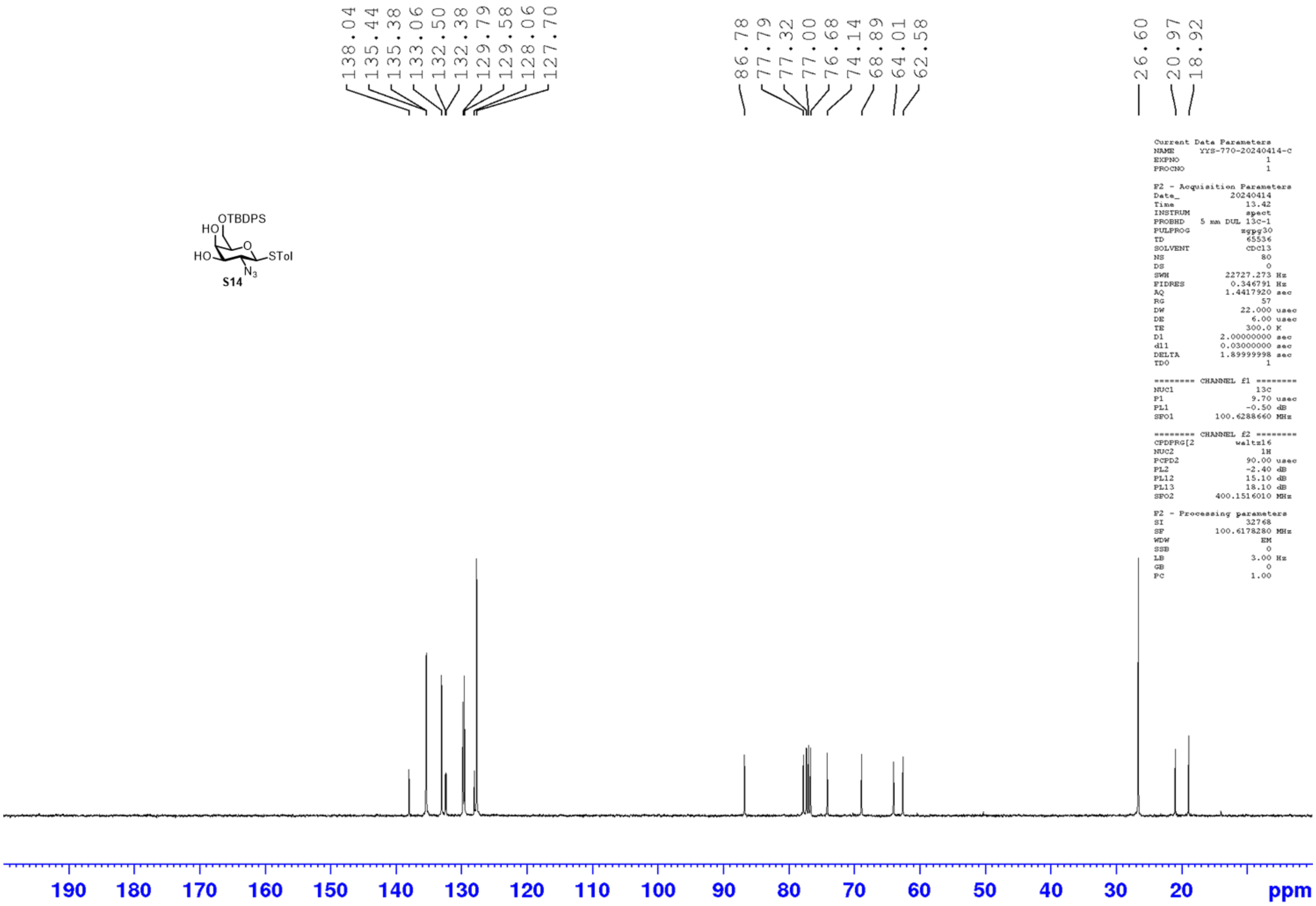

YYS-424-20211227-H

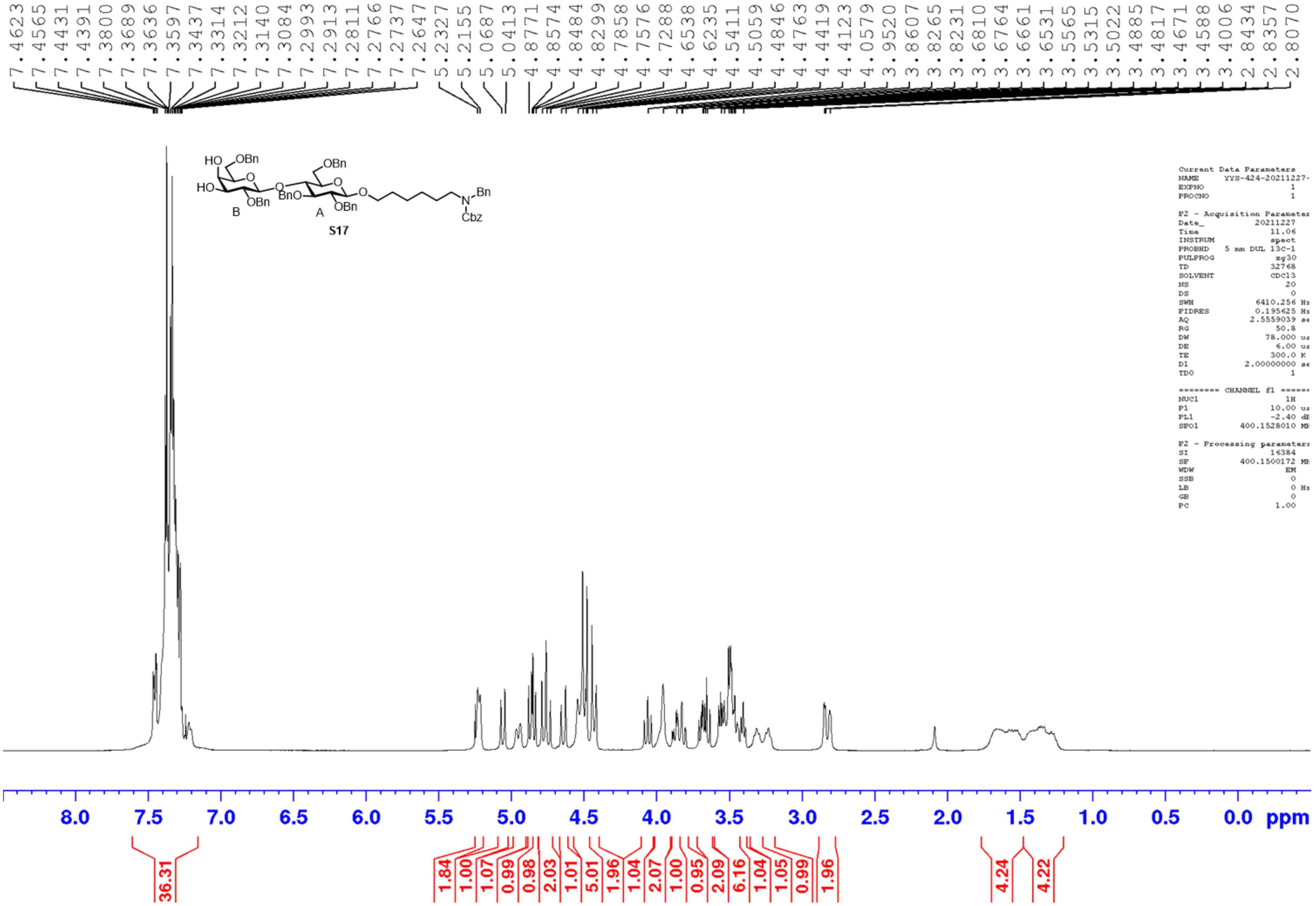

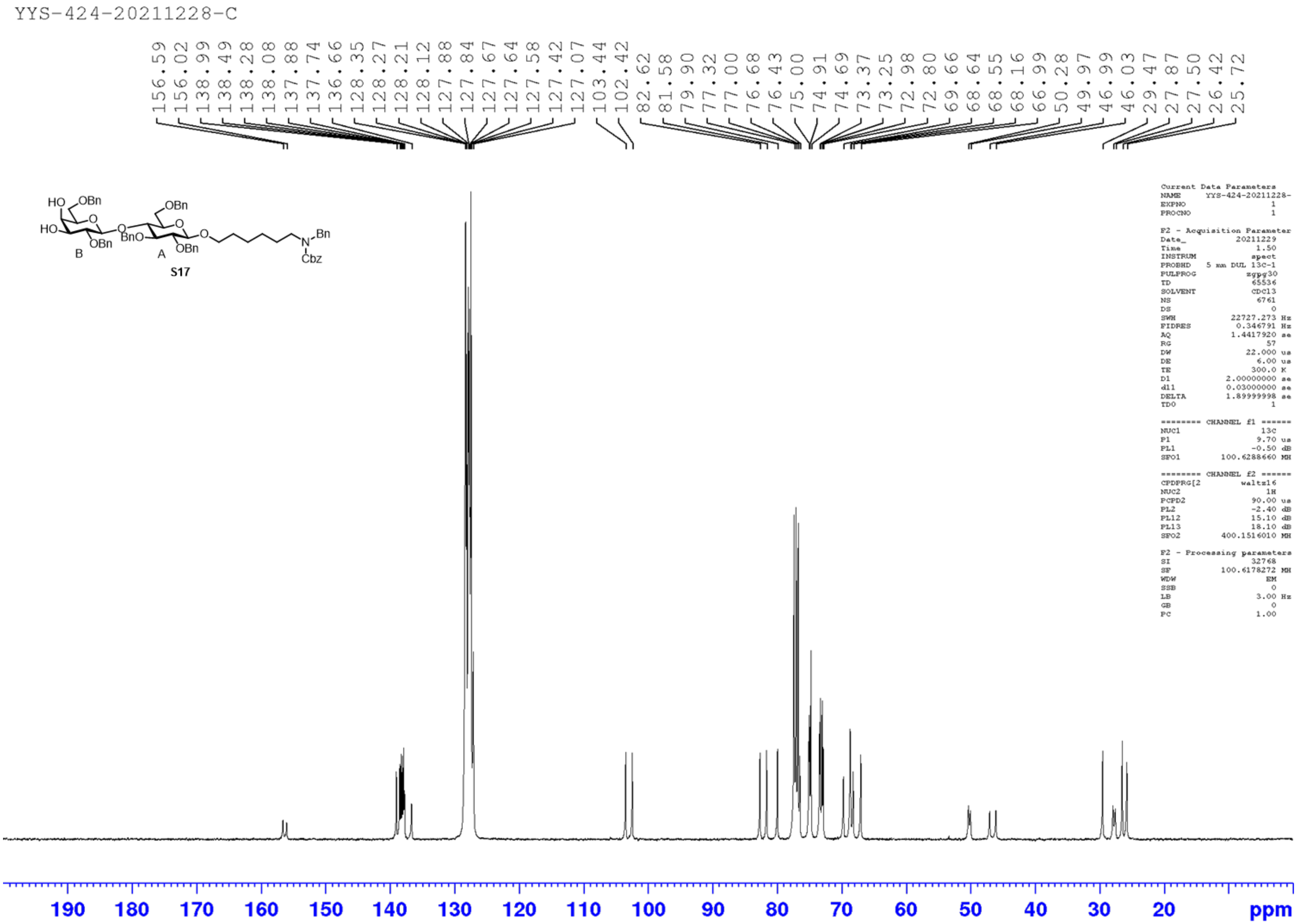
YYS-424-20211228-C
156.59
156.02
138.99
138.49
138.28
138.08
137.88
137.74
136.66
128.35
128.27
128.21
128.12
127.88
127.84
127.67
127.64
127.58
127.42
127.07
103.44
102.42
82.62
81.58
79.90
77.32
77.00
76.68
76.43
75.00
74.91
74.69
73.37
73.25
72.98
72.80
69.66
68.64
68.55
68.16
66.99
50.28
49.97
46.99
46.03
29.47
27.87
27.50
26.42
25.72
HO
OBn
O
HO
OBn
B
O
BnO
OBn
O
A
OBn
O
N
Bn
Cbz
S17
Current Data Parameters
NAME YYS-424-20211228-
EXPNO 1
PROCNO 1
F2 - Acquisition Parameter
Date_ 20211229
Time 1.50
INSTRUM spect
PROBHD 5 mm DUL 13C-1
PULPROG zgpg30
TD 65536
SOLVENT CDCl3
NS 6761
DS 0
SWH 22727.273 Hz
FIDRES 0.346791 Hz
AQ 1.4417920 se
RG 57
DW 22.000 us
DE 6.00 us
TE 300.0 K
D1 2.00000000 se
d11 0.03000000 se
DELTA 1.89999998 se
TD0 1
======== CHANNEL f1 ======
NUC1 13C
P1 9.70 us
PL1 -0.50 dB
SFO1 100.6288660 MH
======== CHANNEL f2 ======
CPDPRG[2 waltz16
NUC2 1H
PCPD2 90.00 us
PL2 -2.40 dB
PL12 15.10 dB
PL13 18.10 dB
SFO2 400.1516010 MH
F2 - Processing parameters
SI 32768
SF 100.6178272 MH
WDW EM
SSB 0
LB 3.00 Hz
GB 0
PC 1.00
190 180 170 160 150 140 130 120 110 100 90 80 70 60 50 40 30 20 ppm

YYS-413-20211202-H

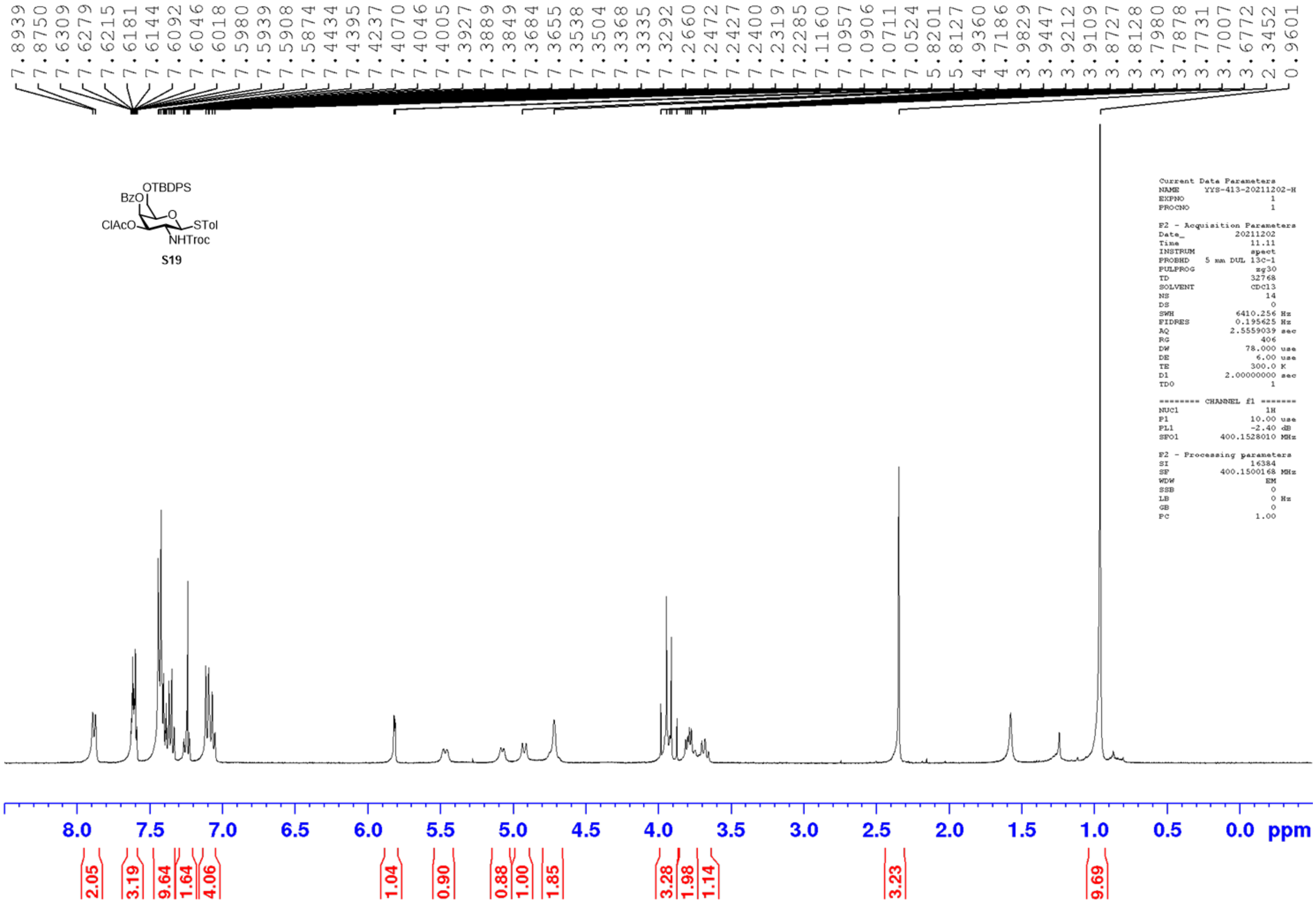

S19

YYS-413-20211204-C

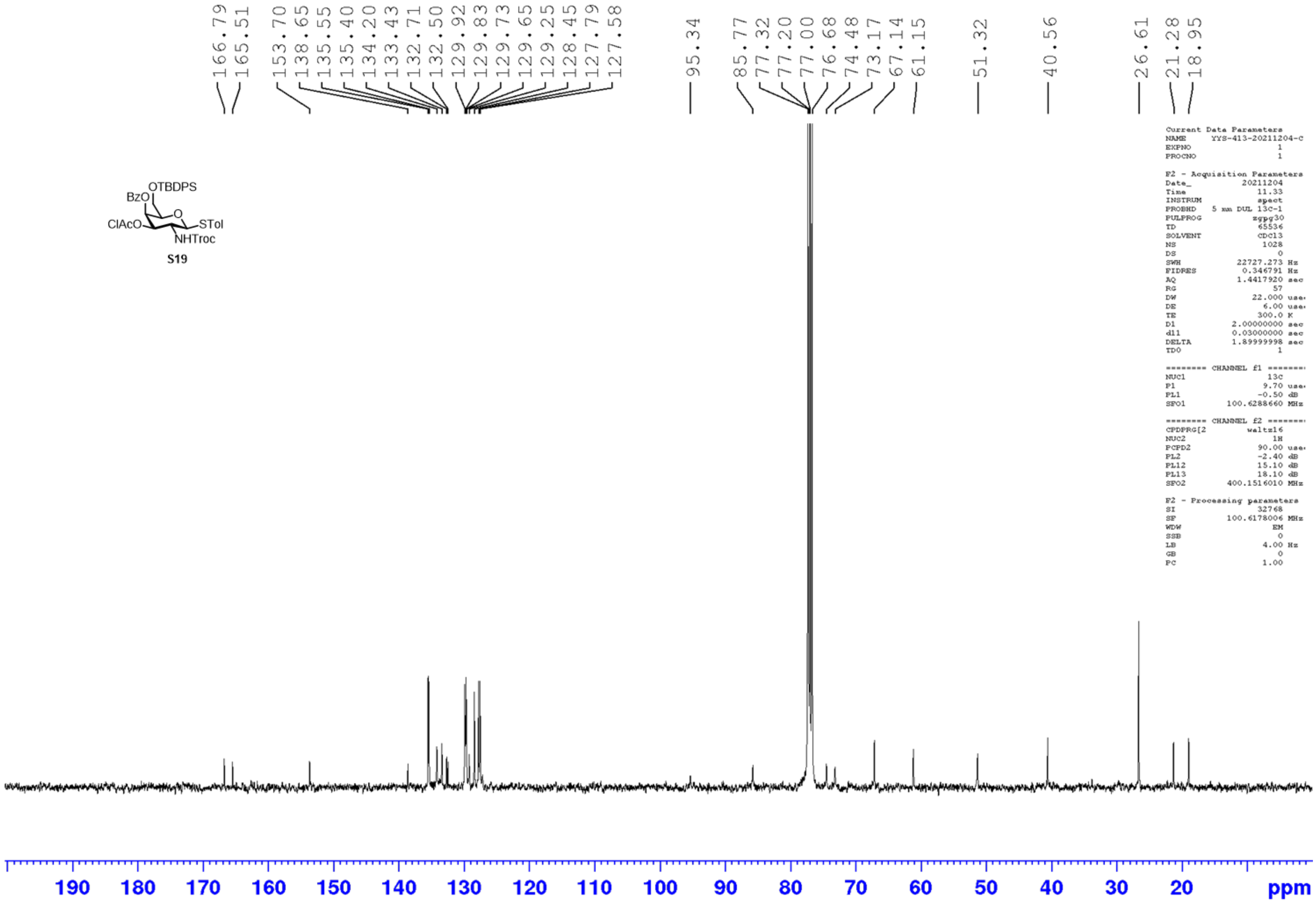

S19

YYS-452-20220311-H

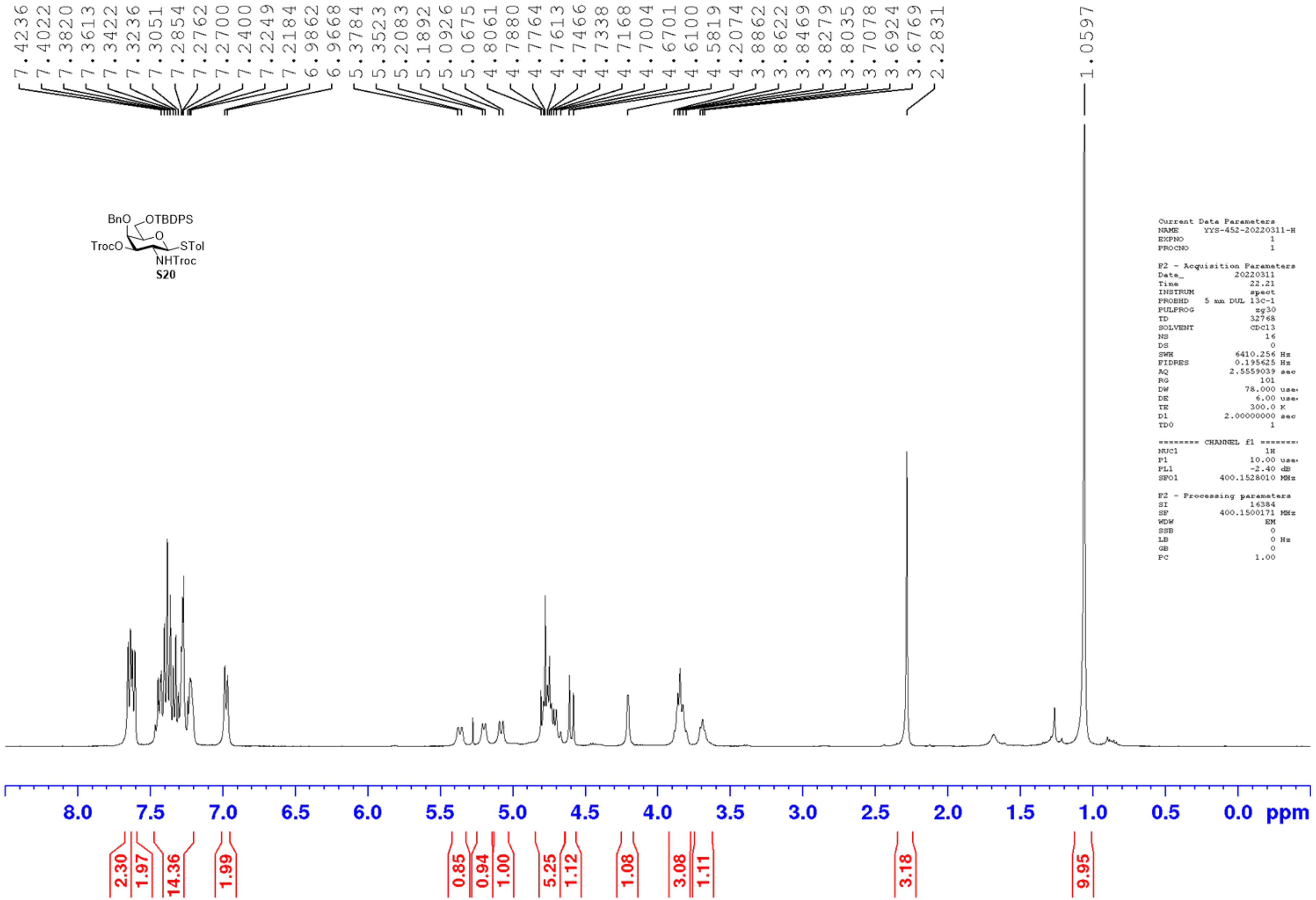

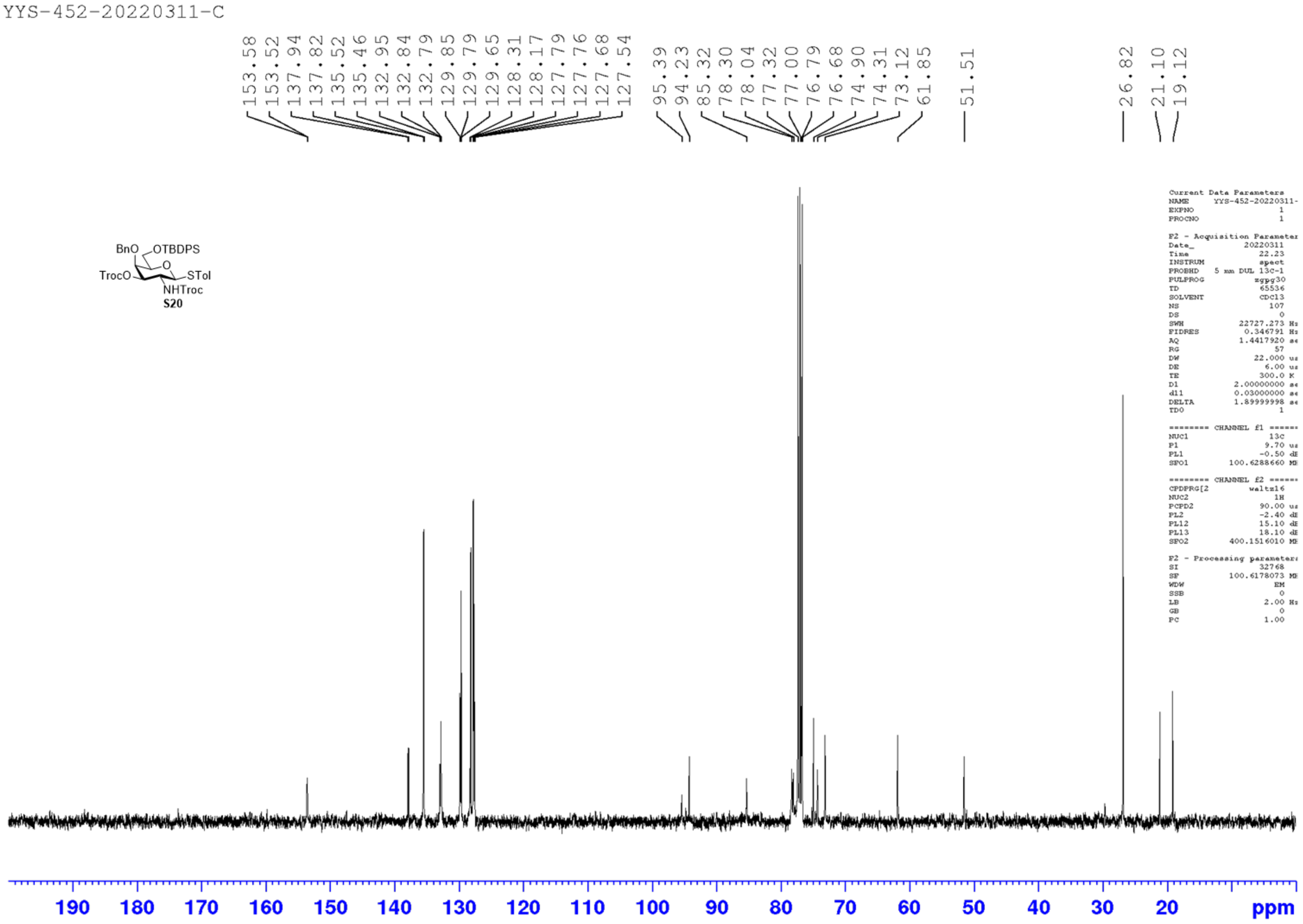
YYS-452-20220311-C
153.58
153.52
137.94
137.82
135.52
135.46
132.95
132.84
132.79
129.85
129.79
129.65
128.31
128.17
127.79
127.76
127.68
127.54
95.39
94.23
85.32
78.30
78.04
77.32
77.00
76.79
76.68
74.90
74.31
73.12
61.85
51.51
26.82
21.10
19.12
BnO OTBDPS
O
TrocO
STol
NHTroc
S20
Current Data Parameters
NAME YYS-452-20220311-
EXPNO 1
PROCNO 1
F2 - Acquisition Parameter
Date_ 20220311
Time 22.23
INSTRUM spect
PROBHD 5 mm DUL 13C-1
PULPROG zgpg30
TD 65536
SOLVENT CDCl3
NS 107
DS 0
SWH 22727.273 Hz
FIDRES 0.346791 Hz
AQ 1.4417920 se
RG 57
DW 22.000 us
DE 6.00 us
TE 300.0 K
D1 2.00000000 se
d11 0.03000000 se
DELTA 1.89999998 se
TD0 1
======== CHANNEL f1 ======
NUC1 13C
P1 9.70 us
PL1 -0.50 dB
SFO1 100.6288660 MH
======== CHANNEL f2 ======
CPDPRG[2 waltz16
NUC2 1H
PCPD2 90.00 us
PL2 -2.40 dB
PL12 15.10 dB
PL13 18.10 dB
SFO2 400.1516010 MH
F2 - Processing parameters
SI 32768
SF 100.6178073 MH
WDW EM
SSB 0
LB 2.00 Hz
GB 0
PC 1.00
190
180
170
160
150
140
130
120
110
100
90
80
70
60
50
40
30
20
ppm

YYS-556-20221130-2-H

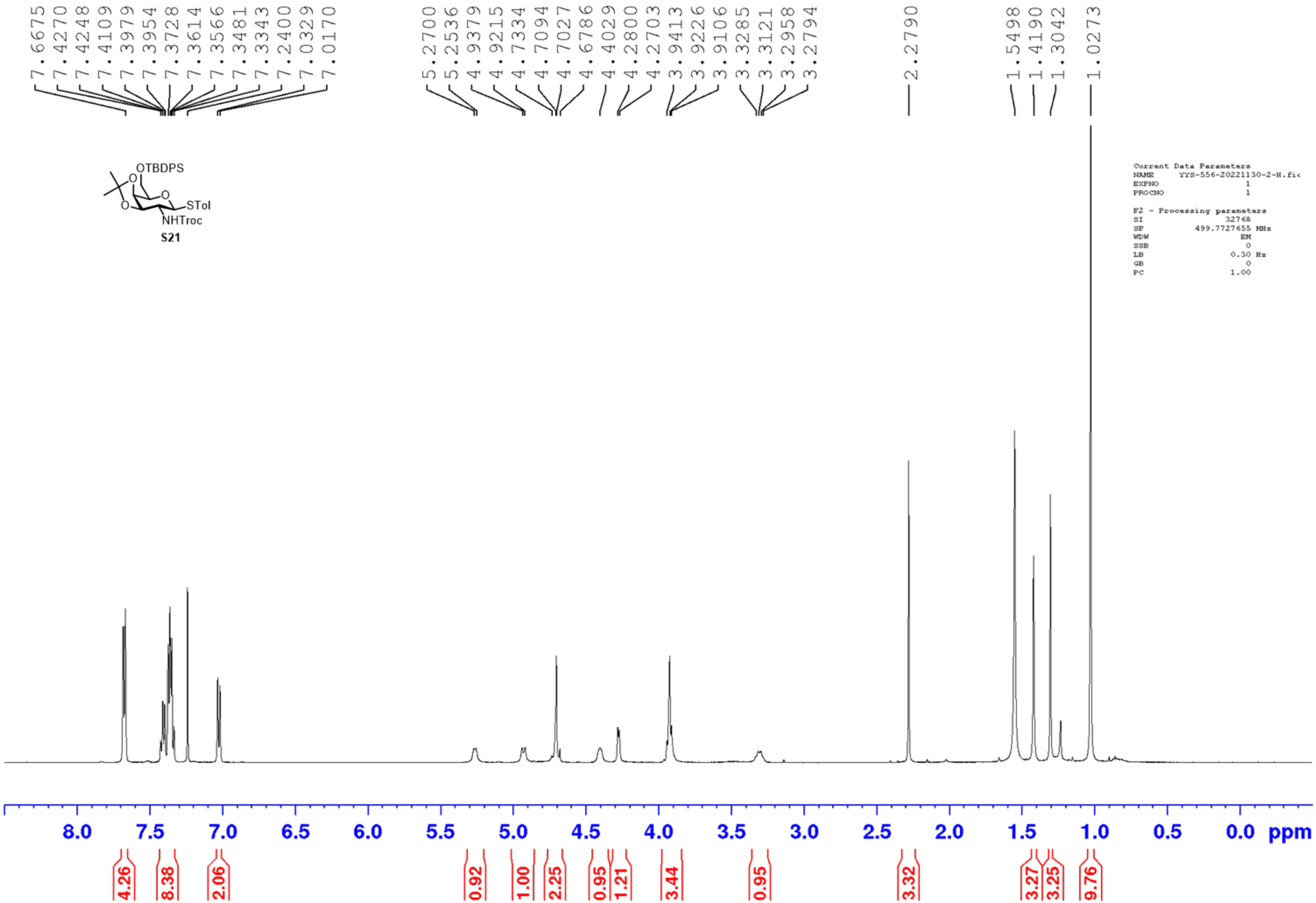

YYS-606-20230423-C

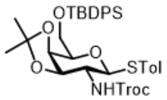

S21

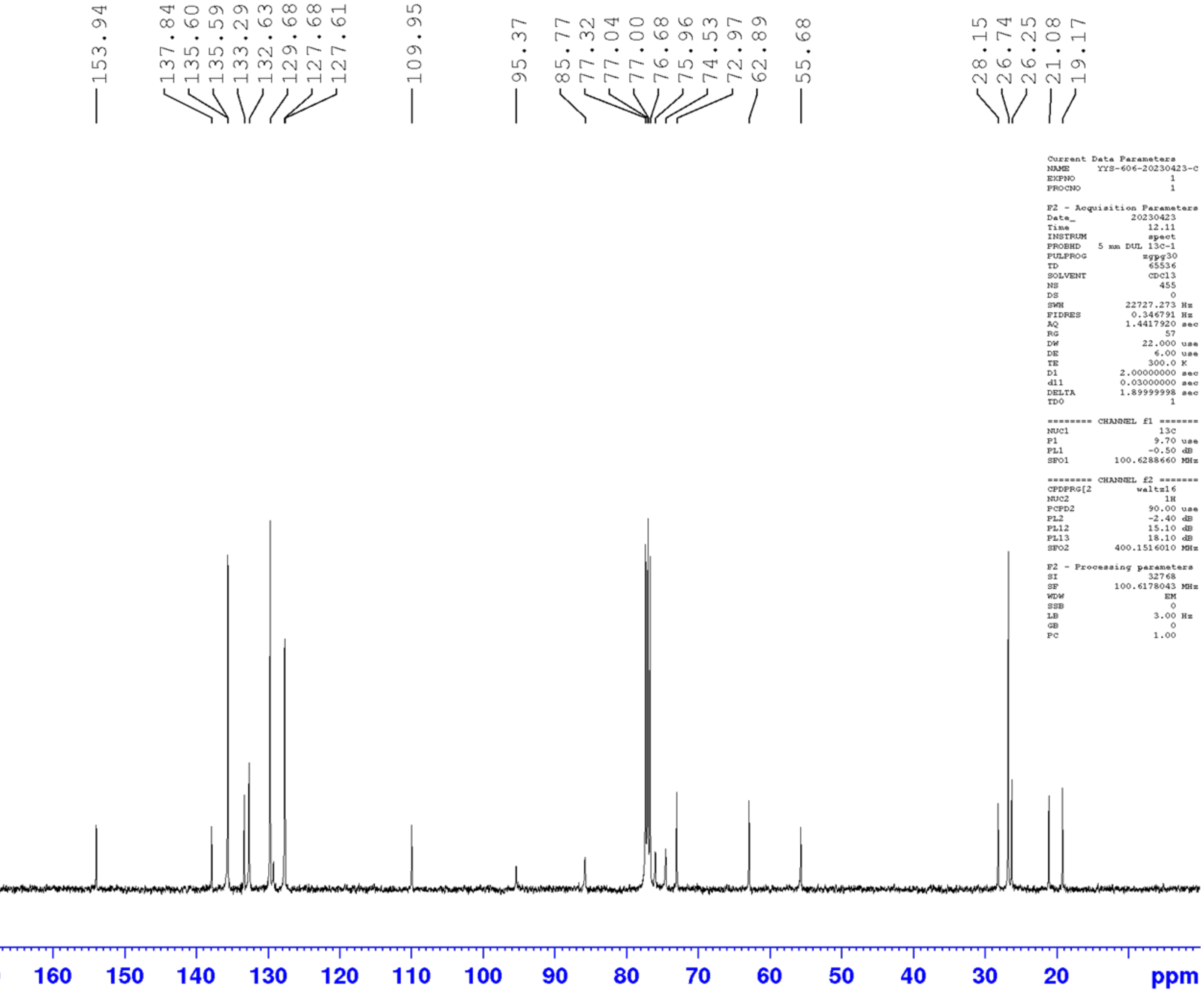

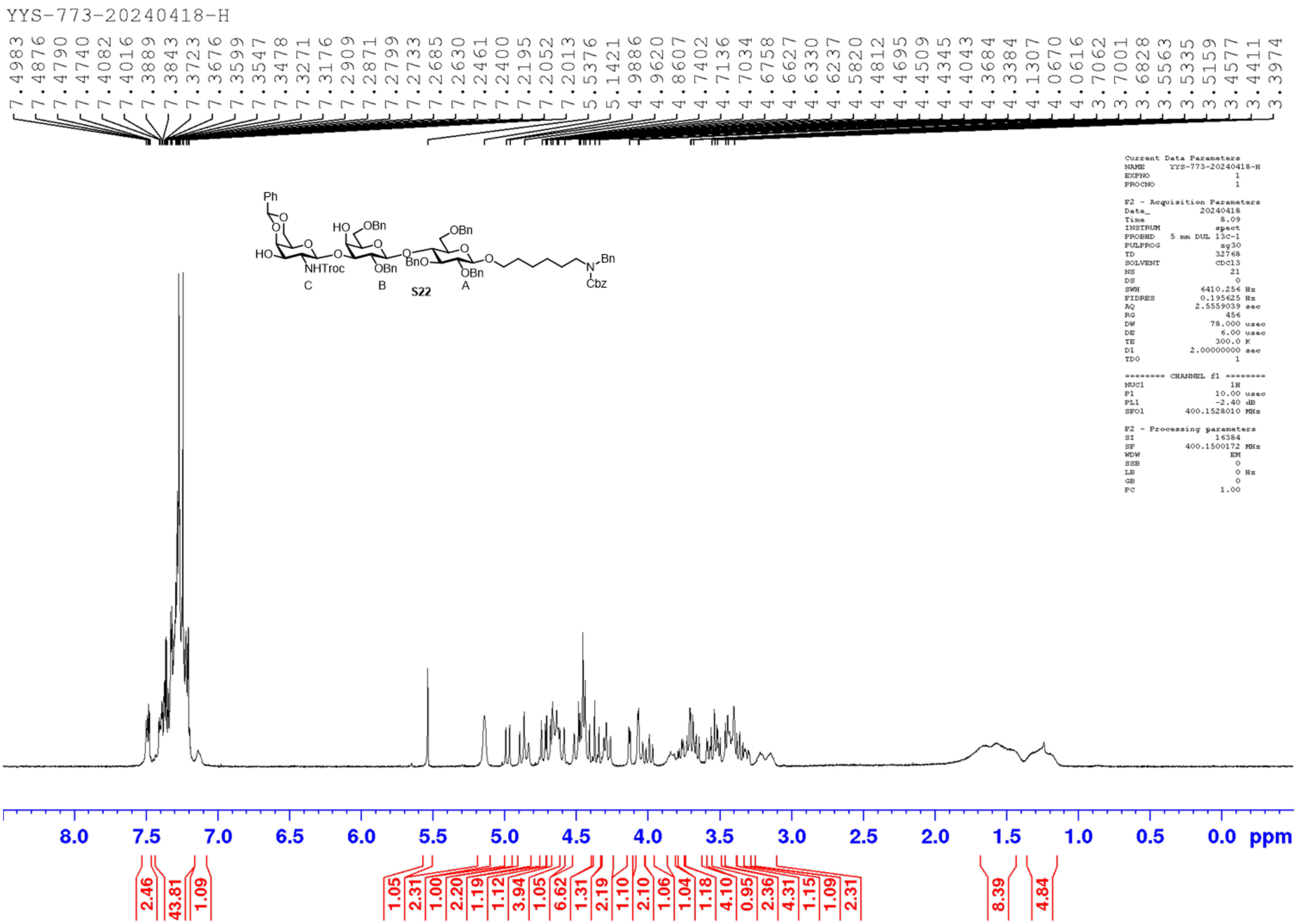

YYS-773-20240418-H
7.4983 7.4876 7.4790 7.4740 7.4082 7.4016 7.3889 7.3843 7.3723 7.3676 7.3599 7.3547 7.3478 7.3271 7.3176 7.2909 7.2871 7.2799 7.2733 7.2685 7.2630 7.2461 7.2400 7.2195 7.2052 7.2013 5.5376 5.1421 4.9886 4.9620 4.8607 4.7402 4.7136 4.7034 4.6758 4.6627 4.6330 4.6237 4.5820 4.4812 4.4695 4.4509 4.4345 4.4043 4.3684 4.3384 4.1307 4.0670 4.0616 3.7062 3.7001 3.6828 3.5563 3.5335 3.5159 3.4577 3.4411 3.3974
Ph
O
O
HO
O
HO
OBn
O
O
O
OBn
O
O
NHTroc
OBn
BnO
OBn
N
Bn
Cbz
C
B
S22
A
Current Data Parameters
NAME YYS-773-20240418-H
EXPNO 1
PROCNO 1
F2 - Acquisition Parameters
Date_ 20240418
Time 8.09
INSTRUM spect
PROBHD 5 mm DUL 13C-1
PULPROG zg30
TD 32768
SOLVENT CDCl3
NS 21
DS 0
SWH 6410.256 Hz
FIDRES 0.195625 Hz
AQ 2.5559039 sec
RG 456
DW 78.000 usec
DE 6.00 usec
TE 300.0 K
D1 2.00000000 sec
TD0 1
======== CHANNEL f1 ========
NUC1 1H
P1 10.00 usec
PL1 -2.40 dB
SFO1 400.1528010 MHz
F2 - Processing parameters
SI 16384
SF 400.1500172 MHz
WDW EM
SSB 0
LB 0 Hz
GB 0
PC 1.00
8.0 7.5 7.0 6.5 6.0 5.5 5.0 4.5 4.0 3.5 3.0 2.5 2.0 1.5 1.0 0.5 0.0 ppm
2.46 43.81 1.09 1.05 2.31 1.00 2.20 1.19 1.12 3.94 1.05 6.62 1.31 2.19 1.10 2.10 1.06 1.04 1.18 4.10 0.95 2.36 4.31 1.15 1.09 2.31 8.39 4.84

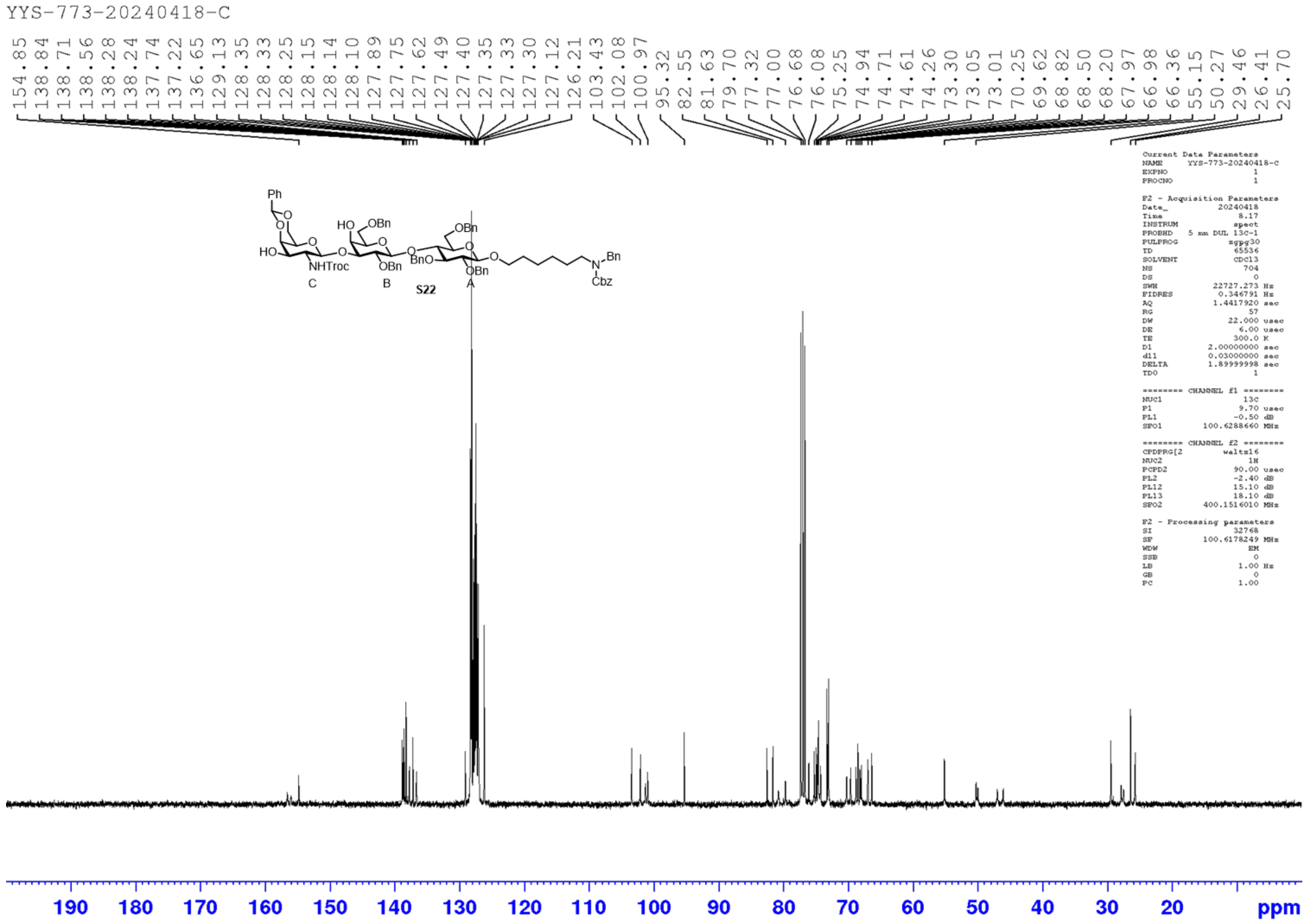
YYS-773-20240418-C
154.85
138.84
138.71
138.56
138.28
138.24
137.74
137.22
136.65
129.13
128.35
128.33
128.25
128.15
128.14
128.10
127.89
127.75
127.62
127.49
127.40
127.35
127.33
127.30
127.12
126.21
103.43
102.08
100.97
95.32
82.55
81.63
79.70
77.32
77.00
76.68
76.08
75.25
74.94
74.71
74.61
74.26
73.30
73.05
73.01
70.25
69.62
68.82
68.50
68.20
67.97
66.98
66.36
55.15
50.27
29.46
26.41
25.70
Ph
O
O
HO
O
NHTroc
C
HO
OBn
O
O
OBn
B
O
OBn
O
BnO
OBn
A
O
N
Bn
Cbz
S22
Current Data Parameters
NAME YYS-773-20240418-C
EXPNO 1
PROCNO 1
F2 - Acquisition Parameters
Date_ 20240418
Time 8.17
INSTRUM spect
PROBHD 5 mm DUL 13C-1
PULPROG zgpg30
TD 65536
SOLVENT CDCl3
NS 704
DS 0
SWH 22727.273 Hz
FIDRES 0.346791 Hz
AQ 1.4417920 sec
RG 57
DW 22.000 usec
DE 6.00 usec
TE 300.0 K
D1 2.00000000 sec
d11 0.03000000 sec
DELTA 1.89999998 sec
TD0 1
======== CHANNEL f1 ========
NUC1 13C
P1 9.70 usec
PL1 -0.50 dB
SFO1 100.6288660 MHz
======== CHANNEL f2 ========
CPDPRG[2 waltz16
NUC2 1H
PCPD2 90.00 usec
PL2 -2.40 dB
PL12 15.10 dB
PL13 18.10 dB
SFO2 400.1516010 MHz
F2 - Processing parameters
SI 32768
SF 100.6178249 MHz
WDW EM
SSB 0
LB 1.00 Hz
GB 0
PC 1.00
190
180
170
160
150
140
130
120
110
100
90
80
70
60
50
40
30
20
ppm

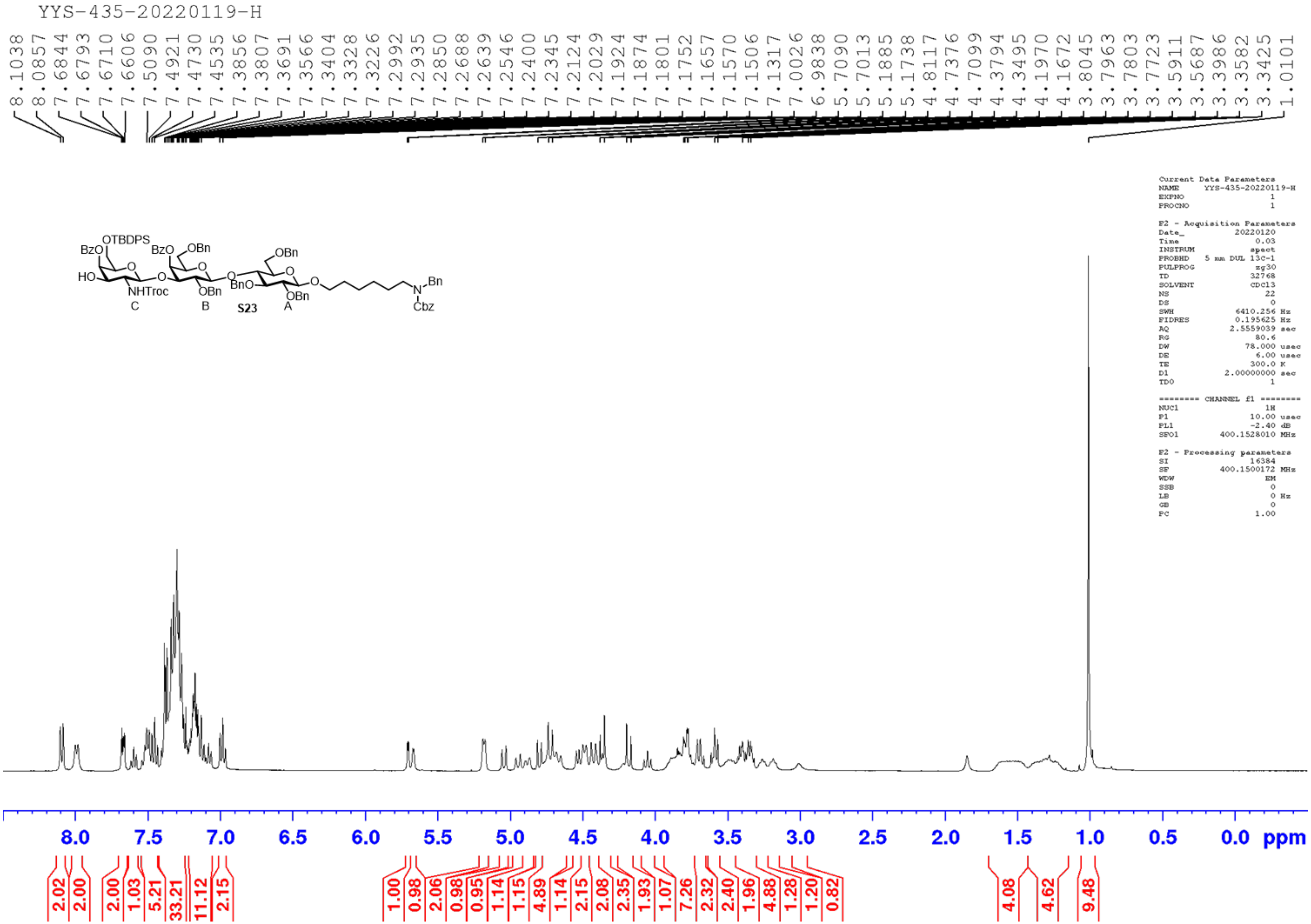
YYS-435-20220119-H
8.1038
8.0857
7.6844
7.6793
7.6710
7.6606
7.5090
7.4921
7.4730
7.4535
7.3856
7.3807
7.3691
7.3566
7.3404
7.3328
7.3226
7.2992
7.2935
7.2850
7.2688
7.2639
7.2546
7.2400
7.2345
7.2124
7.2029
7.1924
7.1874
7.1801
7.1752
7.1657
7.1570
7.1506
7.1317
7.0026
6.9838
5.7090
5.7013
5.1885
5.1738
4.8117
4.7376
4.7099
4.3794
4.3495
4.1970
4.1672
3.8045
3.7963
3.7803
3.7723
3.5911
3.5687
3.3986
3.3582
3.3425
1.0101
Current Data Parameters
NAME YYS-435-20220119-H
EXPNO 1
PROCNO 1
F2 - Acquisition Parameters
Date_ 20220120
Time 0.03
INSTRUM spect
PROBHD 5 mm DUL 13C-1
PULPROG zg30
TD 32768
SOLVENT CDCl3
NS 22
DS 0
SWH 6410.256 Hz
FIDRES 0.195625 Hz
AQ 2.5559039 sec
RG 80.6
DW 78.000 usec
DE 6.00 usec
TE 300.0 K
D1 2.00000000 sec
TD0 1
======== CHANNEL f1 ========
NUC1 1H
P1 10.00 usec
PL1 -2.40 dB
SFO1 400.1528010 MHz
F2 - Processing parameters
SI 16384
SF 400.1500172 MHz
WDW EM
SSB 0
LB 0 Hz
GB 0
PC 1.00
OTBDPS
BzO
HO
NHTroc
C
BzO
OBn
OBn
B
S23
OBn
BnO
OBn
A
N
Bn
Cbz
8.0 7.5 7.0 6.5 6.0 5.5 5.0 4.5 4.0 3.5 3.0 2.5 2.0 1.5 1.0 0.5 0.0 ppm
2.02 2.00 2.00 1.03 5.21 33.21 11.12 2.15 1.00 0.98 2.06 0.98 0.95 1.14 1.15 4.89 1.14 2.15 2.08 2.35 1.93 1.07 7.26 2.32 2.40 1.96 4.88 1.28 1.20 0.82 4.08 4.62 9.48

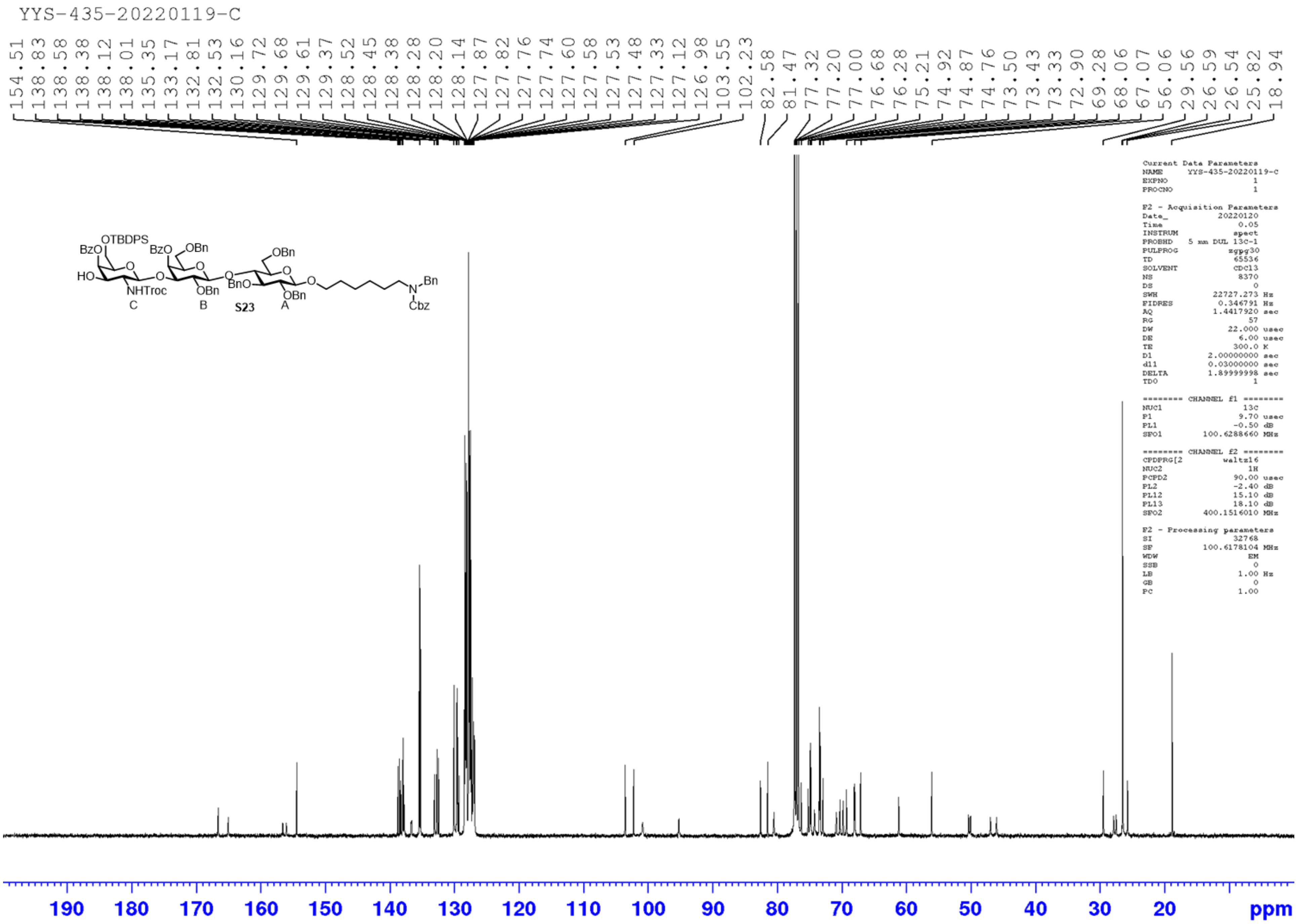

YYS-435-20220119-C
154.51
138.83
138.58
138.38
138.12
138.01
135.35
133.17
132.81
132.53
130.16
129.72
129.68
129.61
129.37
128.52
128.45
128.38
128.28
128.20
128.14
127.87
127.82
127.76
127.74
127.60
127.58
127.53
127.48
127.33
127.12
126.98
103.55
102.23
82.58
81.47
77.32
77.20
77.00
76.68
76.28
75.21
74.92
74.87
74.76
73.50
73.43
73.33
72.90
69.28
68.06
67.07
56.06
29.56
26.59
26.54
25.82
18.94
Current Data Parameters
NAME YYS-435-20220119-C
EXPNO 1
PROCNO 1
F2 - Acquisition Parameters
Date_ 20220120
Time 0.05
INSTRUM spect
PROBHD 5 mm DUL 13C-1
PULPROG zgpg30
TD 65536
SOLVENT CDCl3
NS 8370
DS 0
SWH 22727.273 Hz
FIDRES 0.346791 Hz
AQ 1.4417920 sec
RG 57
DW 22.000 usec
DE 6.00 usec
TE 300.0 K
D1 2.00000000 sec
d11 0.03000000 sec
DELTA 1.89999998 sec
TD0 1
======== CHANNEL f1 ========
NUC1 13C
P1 9.70 usec
PL1 -0.50 dB
SFO1 100.6288660 MHz
======== CHANNEL f2 ========
CPDPRG[2 waltz16
NUC2 1H
PCPD2 90.00 usec
PL2 -2.40 dB
PL12 15.10 dB
PL13 18.10 dB
SFO2 400.1516010 MHz
F2 - Processing parameters
SI 32768
SF 100.6178104 MHz
WDW EM
SSB 0
LB 1.00 Hz
GB 0
PC 1.00
BzO
OTBDPS
HO
O
NHTroc
C
BzO
OBn
O
OBn
B
S23
OBn
BnO
O
OBn
A
N
Bn
Cbz
190
180
170
160
150
140
130
120
110
100
90
80
70
60
50
40
30
20
ppm

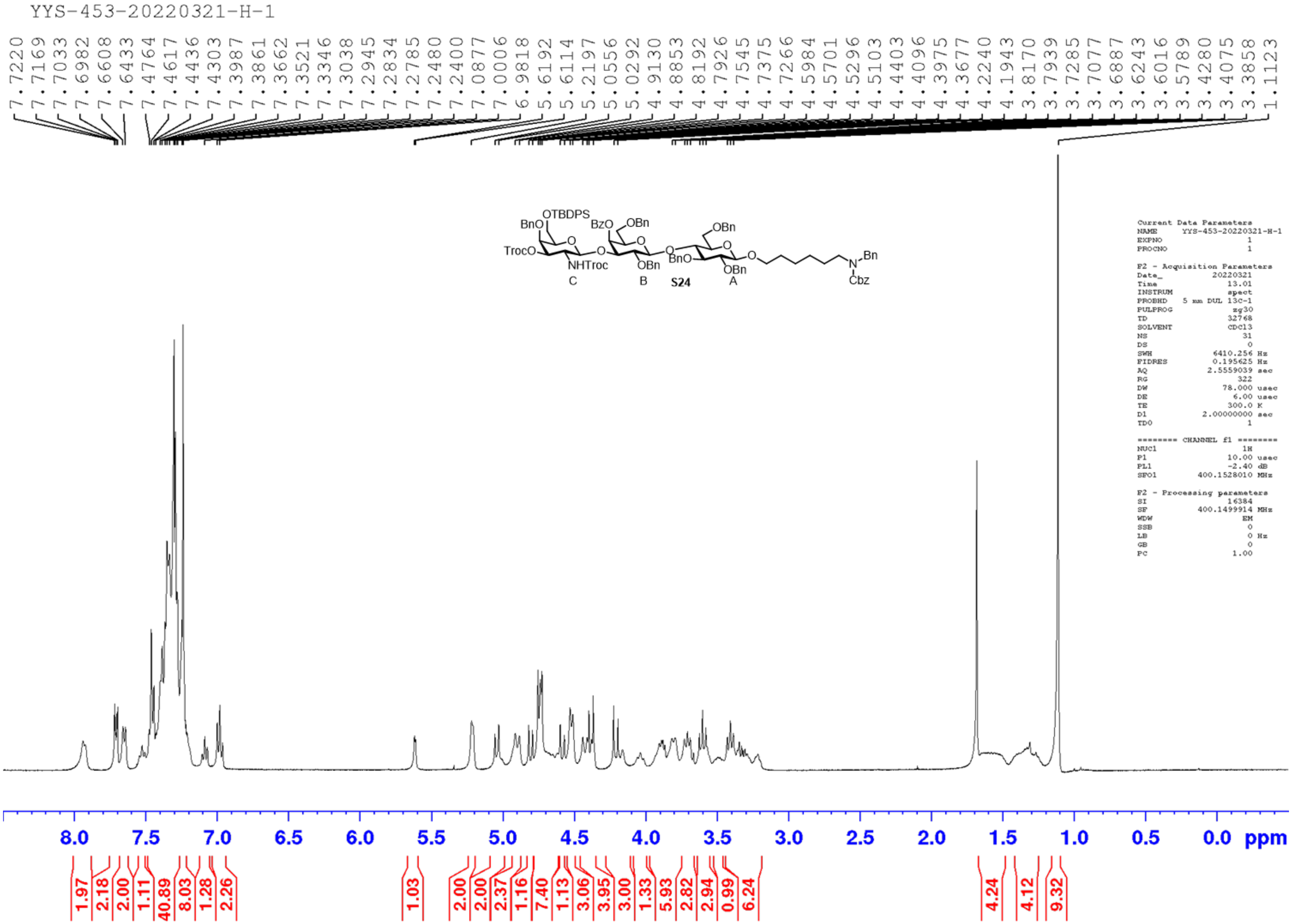
YYS-453-20220321-H-1
7.7220 7.7169 7.7033 7.6982 7.6608 7.6433 7.4764 7.4617 7.4436 7.4303 7.3987 7.3861 7.3662 7.3521 7.3346 7.3038 7.2945 7.2834 7.2785 7.2480 7.2400 7.0877 7.0006 6.9818 5.6192 5.6114 5.2197 5.0556 5.0292 4.9130 4.8853 4.8192 4.7926 4.7545 4.7375 4.7266 4.5984 4.5701 4.5296 4.5103 4.4403 4.4096 4.3975 4.3677 4.2240 4.1943 3.8170 3.7939 3.7285 3.7077 3.6887 3.6243 3.6016 3.5789 3.4280 3.4075 3.3858 1.1123
OTBDPS
BnO
BzO
OBn
TrocO
NHTroc
OBn
BnO
OBn
OBn
N
Bn
Cbz
C
B
S24
A
Current Data Parameters
NAME YYS-453-20220321-H-1
EXPNO 1
PROCNO 1
F2 - Acquisition Parameters
Date_ 20220321
Time 13.01
INSTRUM spect
PROBHD 5 mm DUL 13C-1
PULPROG zg30
TD 32768
SOLVENT CDCl3
NS 31
DS 0
SWH 6410.256 Hz
FIDRES 0.195625 Hz
AQ 2.5559039 sec
RG 322
DW 78.000 usec
DE 6.00 usec
TE 300.0 K
D1 2.00000000 sec
TD0 1
======== CHANNEL f1 ========
NUC1 1H
P1 10.00 usec
PL1 -2.40 dB
SFO1 400.1528010 MHz
F2 - Processing parameters
SI 16384
SF 400.1499914 MHz
WDW EM
SSB 0
LB 0 Hz
GB 0
PC 1.00
8.0 7.5 7.0 6.5 6.0 5.5 5.0 4.5 4.0 3.5 3.0 2.5 2.0 1.5 1.0 0.5 0.0 ppm
1.97 2.18 2.00 1.11 40.89 8.03 1.28 2.26 1.03 2.00 2.00 2.37 1.16 7.40 1.13 3.06 3.95 3.00 1.33 5.93 2.82 2.94 0.99 6.24 4.24 4.12 9.32

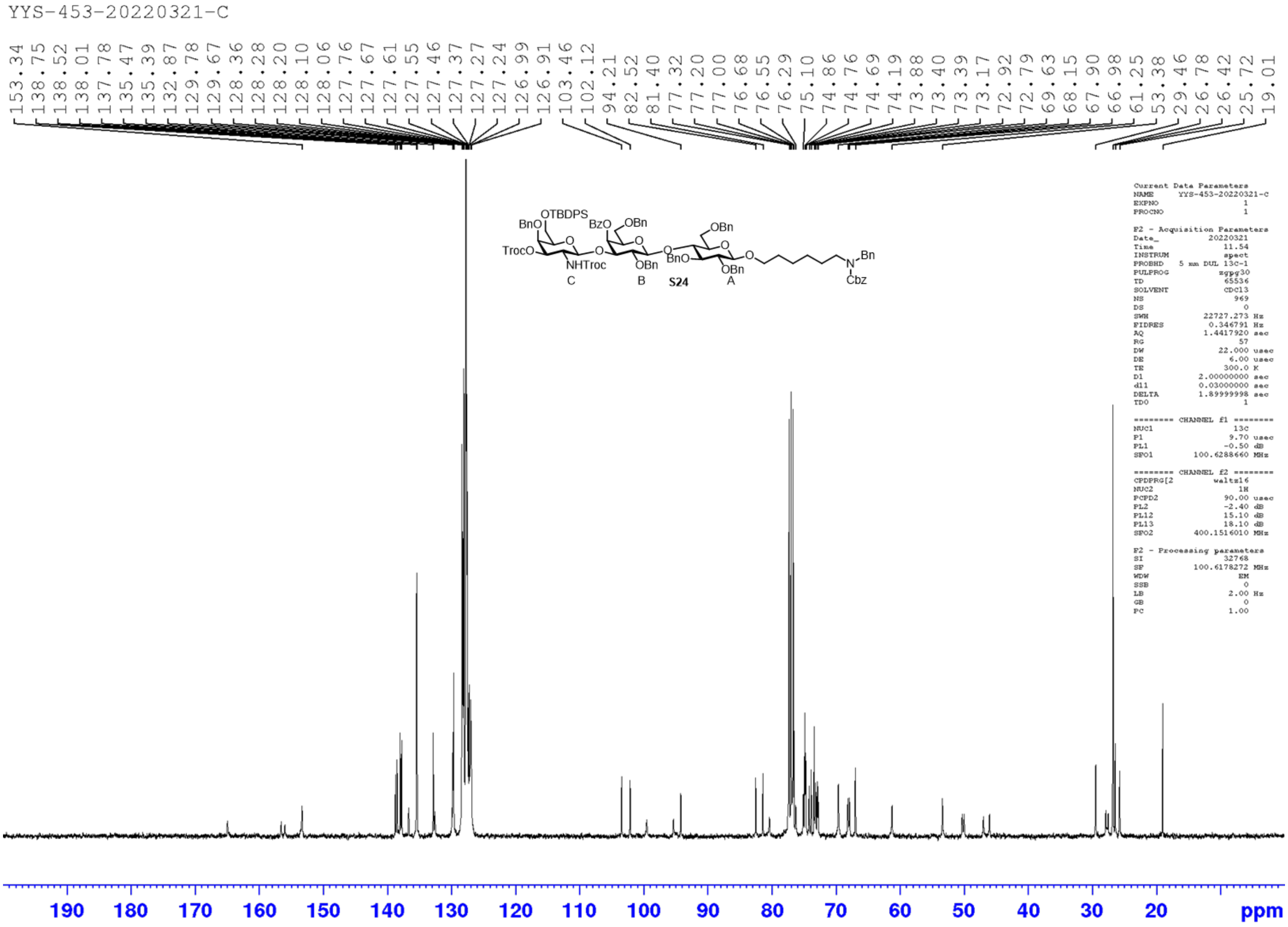
YYS-453-20220321-C
153.34
138.75
138.52
138.01
137.78
135.47
135.39
132.87
129.78
129.67
128.36
128.28
128.20
128.10
128.06
127.76
127.67
127.61
127.55
127.46
127.37
127.27
127.24
126.99
126.91
103.46
102.12
94.21
82.52
81.40
77.32
77.20
77.00
76.68
76.55
76.29
75.10
74.86
74.76
74.69
74.19
73.88
73.40
73.39
73.17
72.92
72.79
69.63
68.15
67.90
66.98
61.25
53.38
29.46
26.78
26.42
25.72
19.01
OTBDPS
BnO
BzO
OBn
OBn
TrocO
O
O
O
O
O
O
N
Bn
NHTroc
OBn
BnO
OBn
Cbz
C
B
S24
A
Current Data Parameters
NAME YYS-453-20220321-C
EXPNO 1
PROCNO 1
F2 - Acquisition Parameters
Date_ 20220321
Time 11.54
INSTRUM spect
PROBHD 5 mm DUL 13C-1
PULPROG zgpg30
TD 65536
SOLVENT CDCl3
NS 969
DS 0
SWH 22727.273 Hz
FIDRES 0.346791 Hz
AQ 1.4417920 sec
RG 57
DW 22.000 usec
DE 6.00 usec
TE 300.0 K
D1 2.00000000 sec
d11 0.03000000 sec
DELTA 1.89999998 sec
TD0 1
======== CHANNEL f1 ========
NUC1 13C
P1 9.70 usec
PL1 -0.50 dB
SFO1 100.6288660 MHz
======== CHANNEL f2 ========
CPDPRG[2 waltz16
NUC2 1H
PCPD2 90.00 usec
PL2 -2.40 dB
PL12 15.10 dB
PL13 18.10 dB
SFO2 400.1516010 MHz
F2 - Processing parameters
SI 32768
SF 100.6178272 MHz
WDW EM
SSB 0
LB 2.00 Hz
GB 0
PC 1.00
190
180
170
160
150
140
130
120
110
100
90
80
70
60
50
40
30
20
ppm

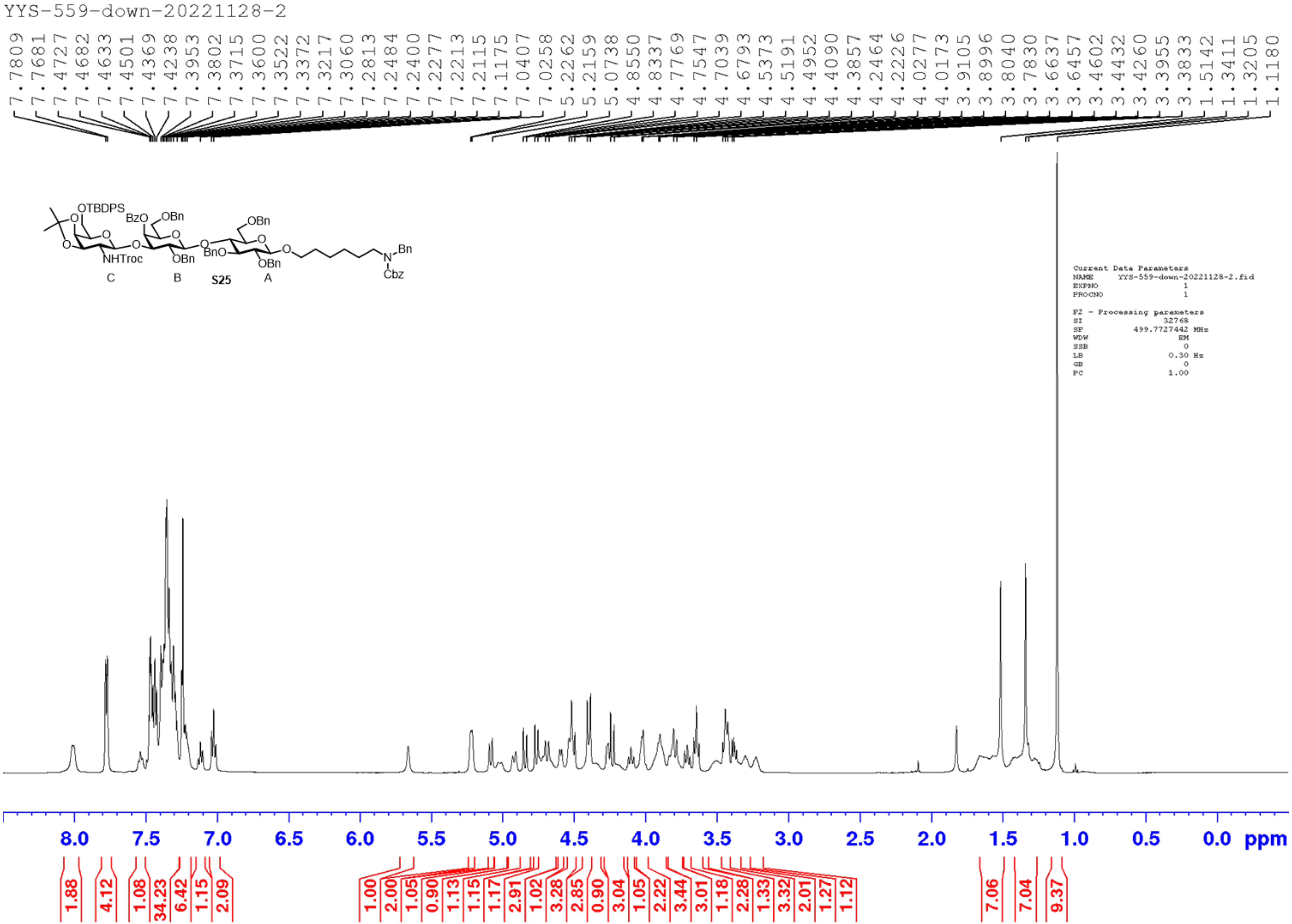

YYS-559-down-20221128-2
7.7809 7.7681 7.4727 7.4682 7.4633 7.4501 7.4369 7.4238 7.3953 7.3802 7.3715 7.3600 7.3522 7.3372 7.3217 7.3060 7.2813 7.2484 7.2400 7.2277 7.2213 7.2115 7.1175 7.0407 7.0258 5.2262 5.2159 5.0738 4.8550 4.8337 4.7769 4.7547 4.7039 4.6793 4.5373 4.5191 4.4952 4.4090 4.3857 4.2464 4.2226 4.0277 4.0173 3.9105 3.8996 3.8040 3.7830 3.6637 3.6457 3.4602 3.4432 3.4260 3.3955 3.3833 1.5142 1.3411 1.3205 1.1180
OTBDPS
BzO
OBn
O
NHTroc
OBn
BnO
OBn
N
Bn
Cbz
C
B
S25
A
Current Data Parameters
NAME YYS-559-down-20221128-2.fid
EXPNO 1
PROCNO 1
F2 - Processing parameters
SI 32768
SF 499.7727442 MHz
WDW EM
SSB 0
LB 0.30 Hz
GB 0
PC 1.00
8.0 7.5 7.0 6.5 6.0 5.5 5.0 4.5 4.0 3.5 3.0 2.5 2.0 1.5 1.0 0.5 0.0 ppm
1.88 4.12 1.08 34.23 6.42 1.15 2.09 1.00 2.00 1.05 0.90 1.13 1.15 1.17 2.91 1.02 3.28 2.85 0.90 3.04 1.05 2.22 3.44 3.01 1.18 2.28 1.33 3.32 2.01 1.27 1.12 7.06 7.04 9.37

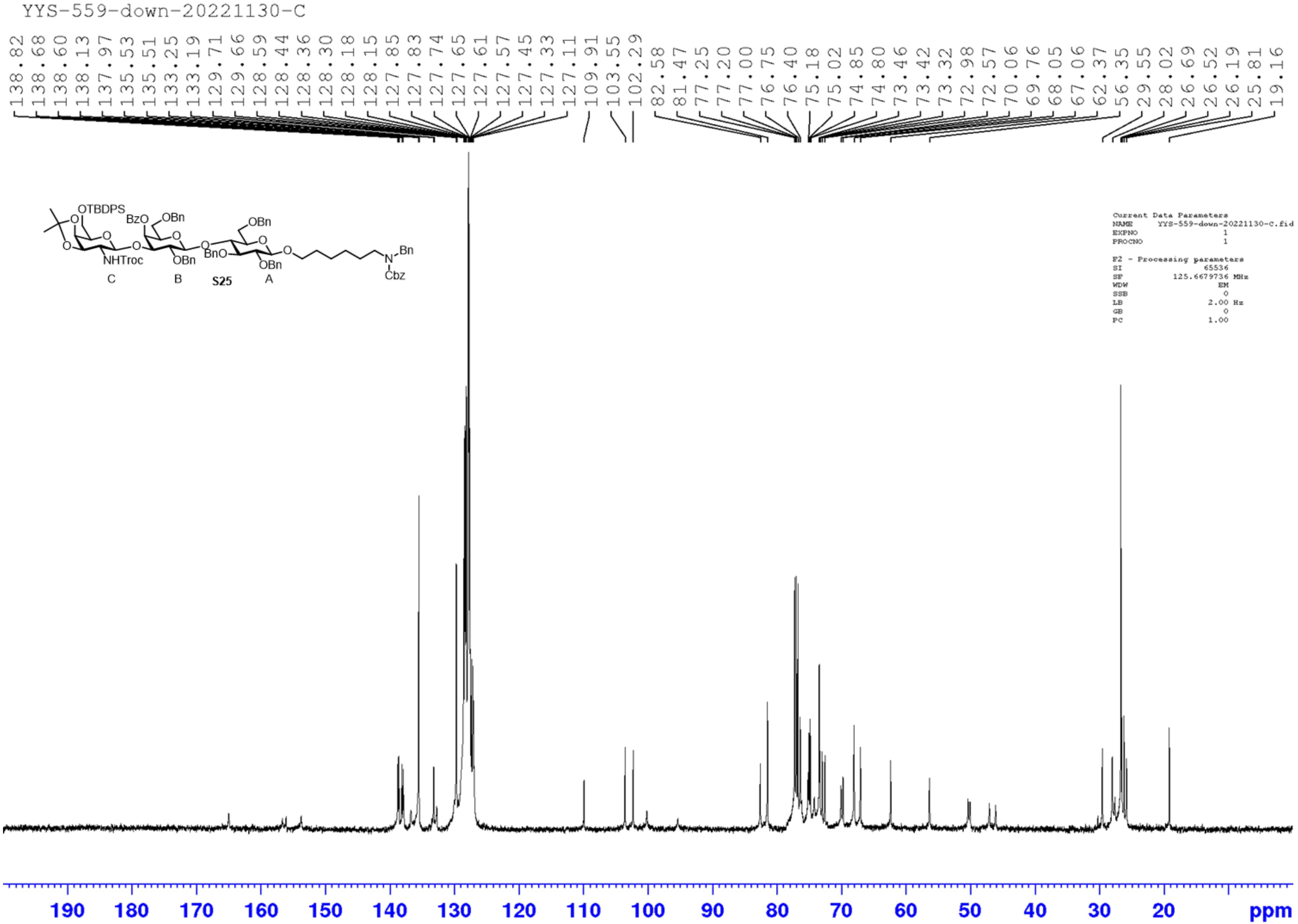
YYS-559-down-20221130-C
138.82 138.68 138.60 138.13 137.97 135.53 135.51 133.25 133.19 129.71 129.66 128.59 128.44 128.36 128.30 128.18 128.15 127.85 127.83 127.74 127.65 127.61 127.57 127.45 127.33 127.11 109.91 103.55 102.29 82.58 81.47 77.25 77.20 77.00 76.75 76.40 75.18 75.02 74.85 74.80 73.46 73.42 73.32 72.98 72.57 70.06 69.76 68.05 67.06 62.37 56.35 29.55 28.02 26.69 26.52 26.19 25.81 19.16
OTBDPS
BzO
OBn
O
NHTroc
OBn
BnO
OBn
OBn
N
Bn
Cbz
C
B
S25
A
Current Data Parameters
NAME YYS-559-down-20221130-C.fid
EXPNO 1
PROCNO 1
F2 - Processing parameters
SI 65536
SF 125.6679736 MHz
WDW EM
SSB 0
LB 2.00 Hz
GB 0
PC 1.00
190 180 170 160 150 140 130 120 110 100 90 80 70 60 50 40 30 20 ppm

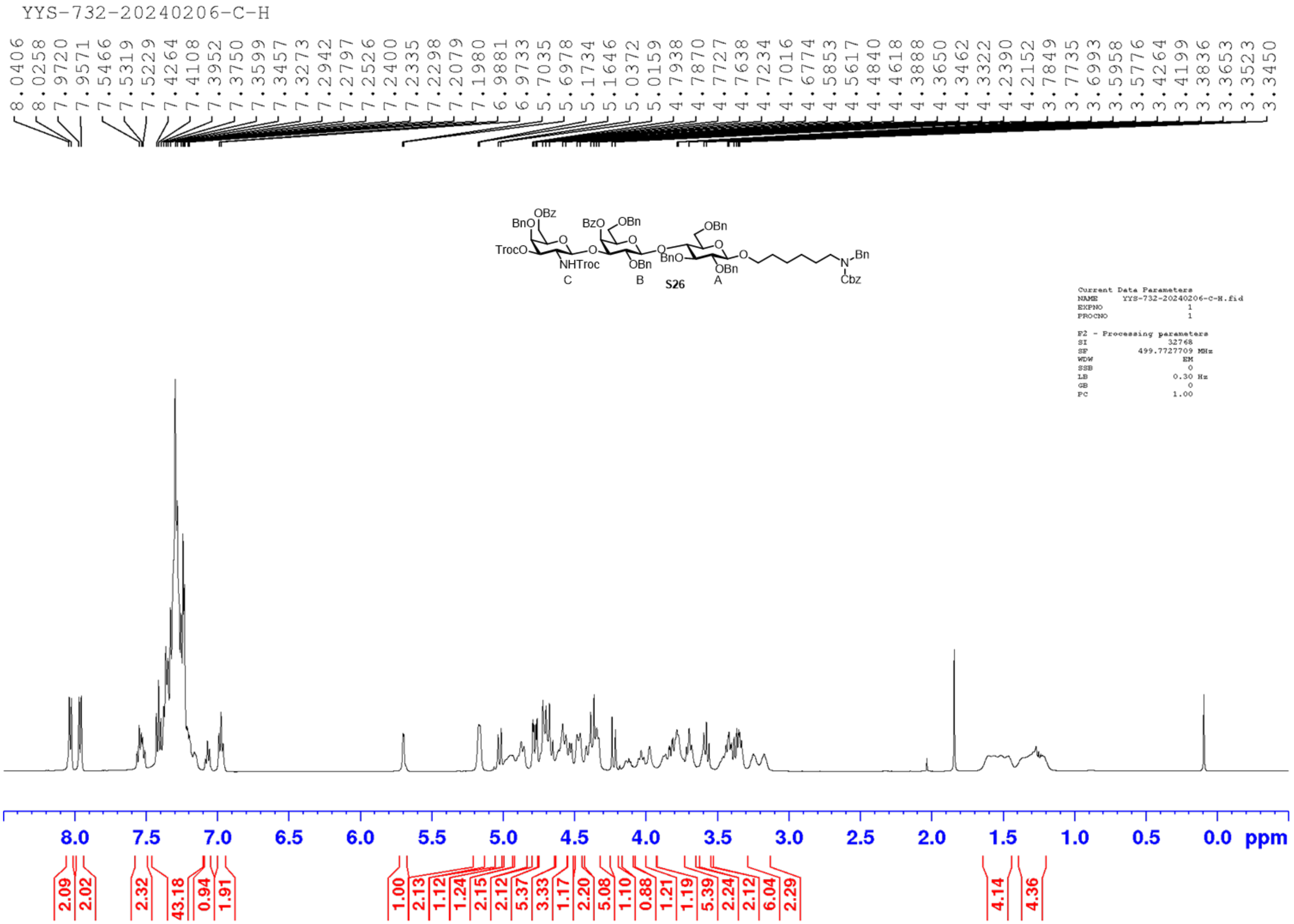
YYS-732-20240206-C-H
8.0406 8.0258 7.9720 7.9571 7.5466 7.5319 7.5229 7.4264 7.4108 7.3952 7.3750 7.3599 7.3457 7.3273 7.2942 7.2797 7.2526 7.2400 7.2335 7.2298 7.2079 7.1980 6.9881 6.9733 5.7035 5.6978 5.1734 5.1646 5.0372 5.0159 4.7938 4.7870 4.7727 4.7638 4.7234 4.7016 4.6774 4.5853 4.5617 4.4840 4.4618 4.3888 4.3650 4.3462 4.3322 4.2390 4.2152 3.7849 3.7735 3.6993 3.5958 3.5776 3.4264 3.4199 3.3836 3.3653 3.3523 3.3450
BnO
OBz
BzO
OBn
OBn
TrocO
NHTroc
OBn
BnO
OBn
N
Bn
Cbz
C
B
S26
A
Current Data Parameters
NAME YYS-732-20240206-C-H.fid
EXPNO 1
PROCNO 1
F2 - Processing parameters
SI 32768
SF 499.7727709 MHz
WDW EM
SSB 0
LB 0.30 Hz
GB 0
PC 1.00
8.0 7.5 7.0 6.5 6.0 5.5 5.0 4.5 4.0 3.5 3.0 2.5 2.0 1.5 1.0 0.5 0.0 ppm
2.09 2.02 2.32 43.18 0.94 1.91 1.00 2.13 1.12 1.24 2.15 2.12 5.37 3.33 1.17 2.20 5.08 1.10 0.88 1.21 1.19 5.39 2.24 2.12 6.04 2.29 4.14 4.36

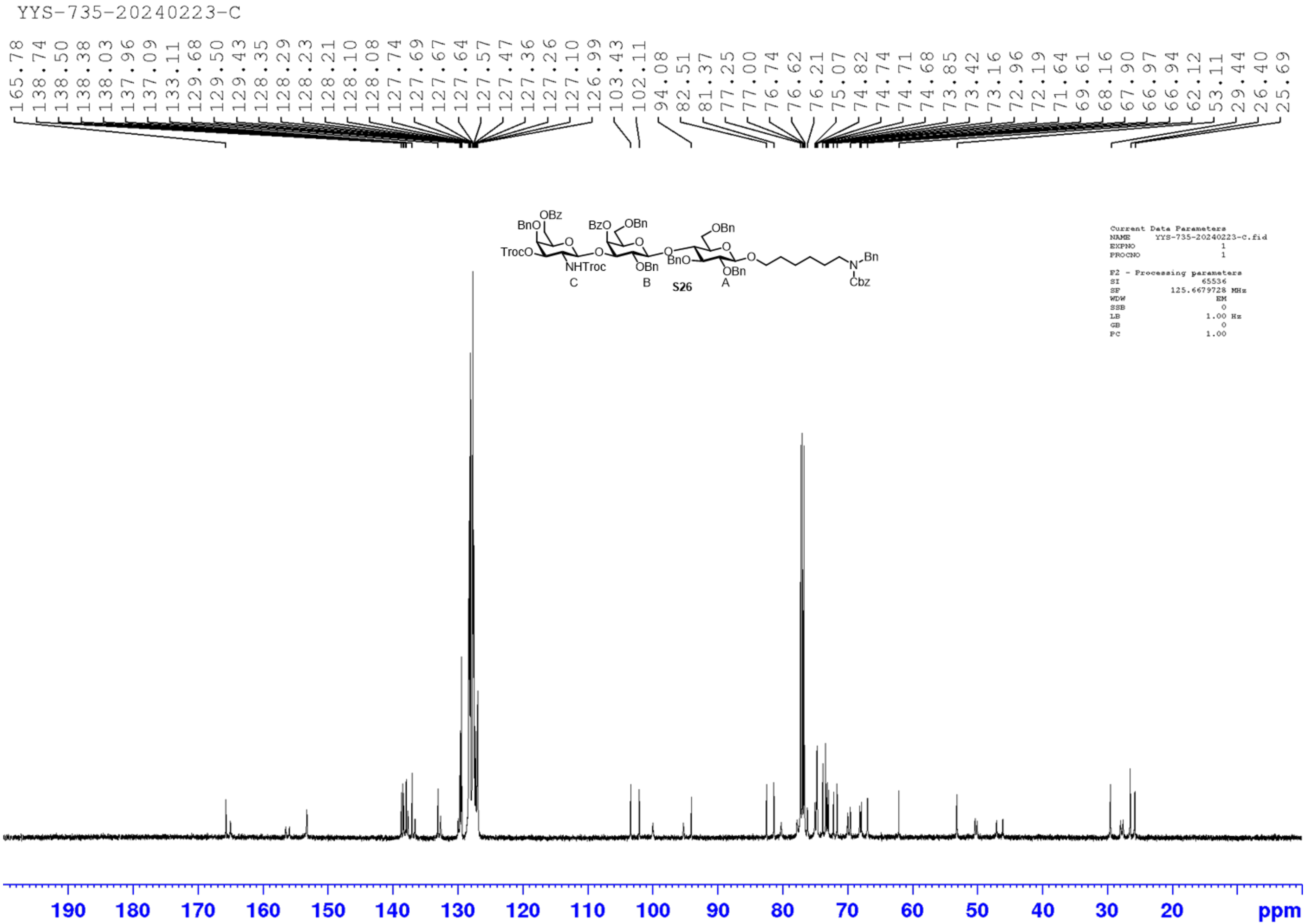

YYS-735-20240223-C
165.78
138.74
138.50
138.38
138.03
137.96
137.09
133.11
129.68
129.50
129.43
128.35
128.29
128.23
128.21
128.10
128.08
127.74
127.69
127.67
127.64
127.57
127.47
127.36
127.26
127.10
126.99
103.43
102.11
94.08
82.51
81.37
77.25
77.00
76.74
76.62
76.21
75.07
74.82
74.74
74.71
74.68
73.85
73.42
73.16
72.96
72.19
71.64
69.61
68.16
67.90
66.97
66.94
62.12
53.11
29.44
26.40
25.69
BnO
OBz
TrocO
NHTroc
C
BzO
OBn
OBn
B
S26
OBn
BnO
OBn
A
N
Bn
Cbz
Current Data Parameters
NAME YYS-735-20240223-C.fid
EXPNO 1
PROCNO 1
F2 - Processing parameters
SI 65536
SF 125.6679728 MHz
WDW EM
SSB 0
LB 1.00 Hz
GB 0
PC 1.00
190
180
170
160
150
140
130
120
110
100
90
80
70
60
50
40
30
20
ppm

YYS-496-20220723-H

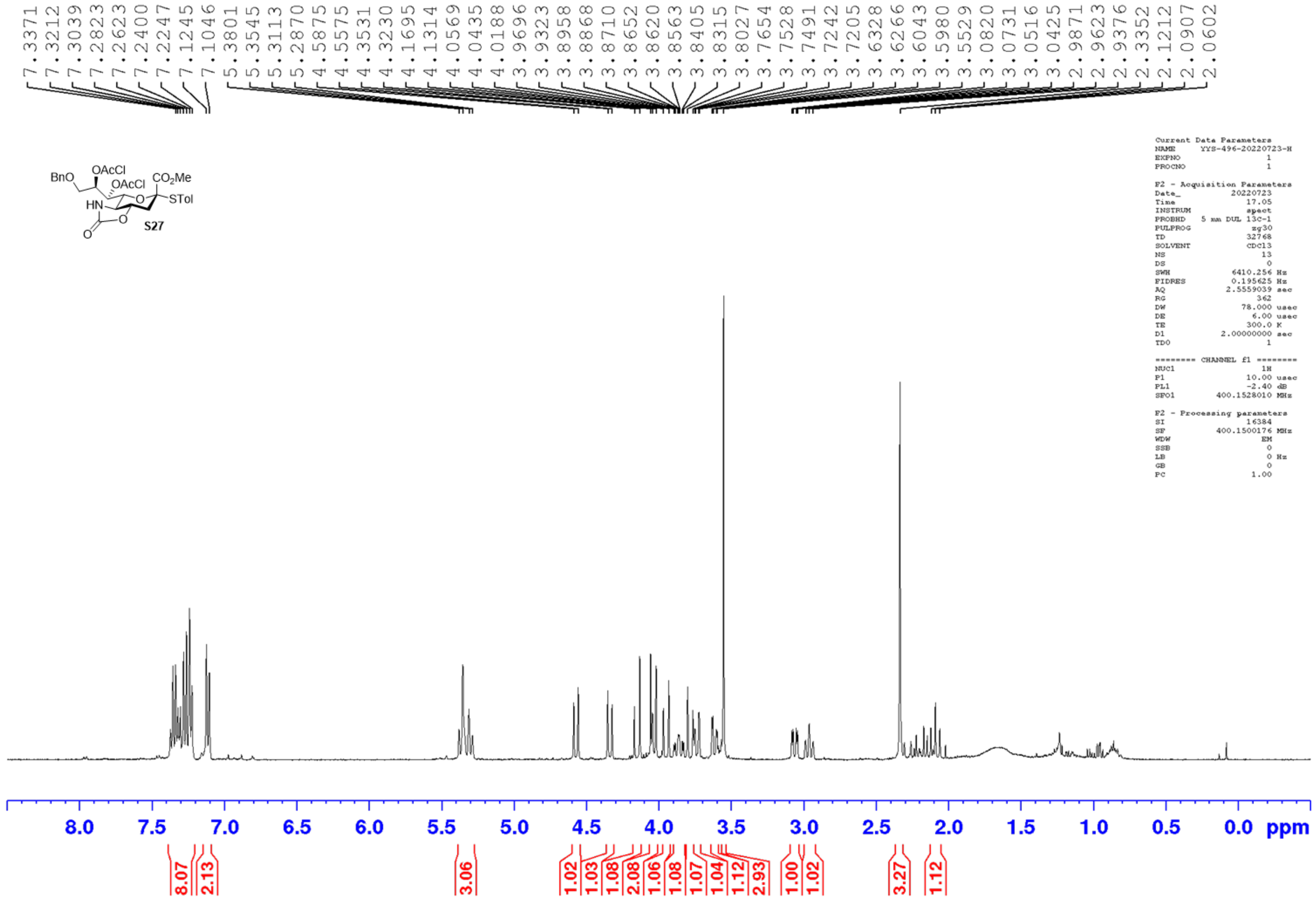

YYS-496-20220719-C

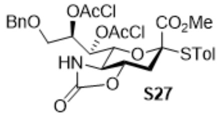

S27

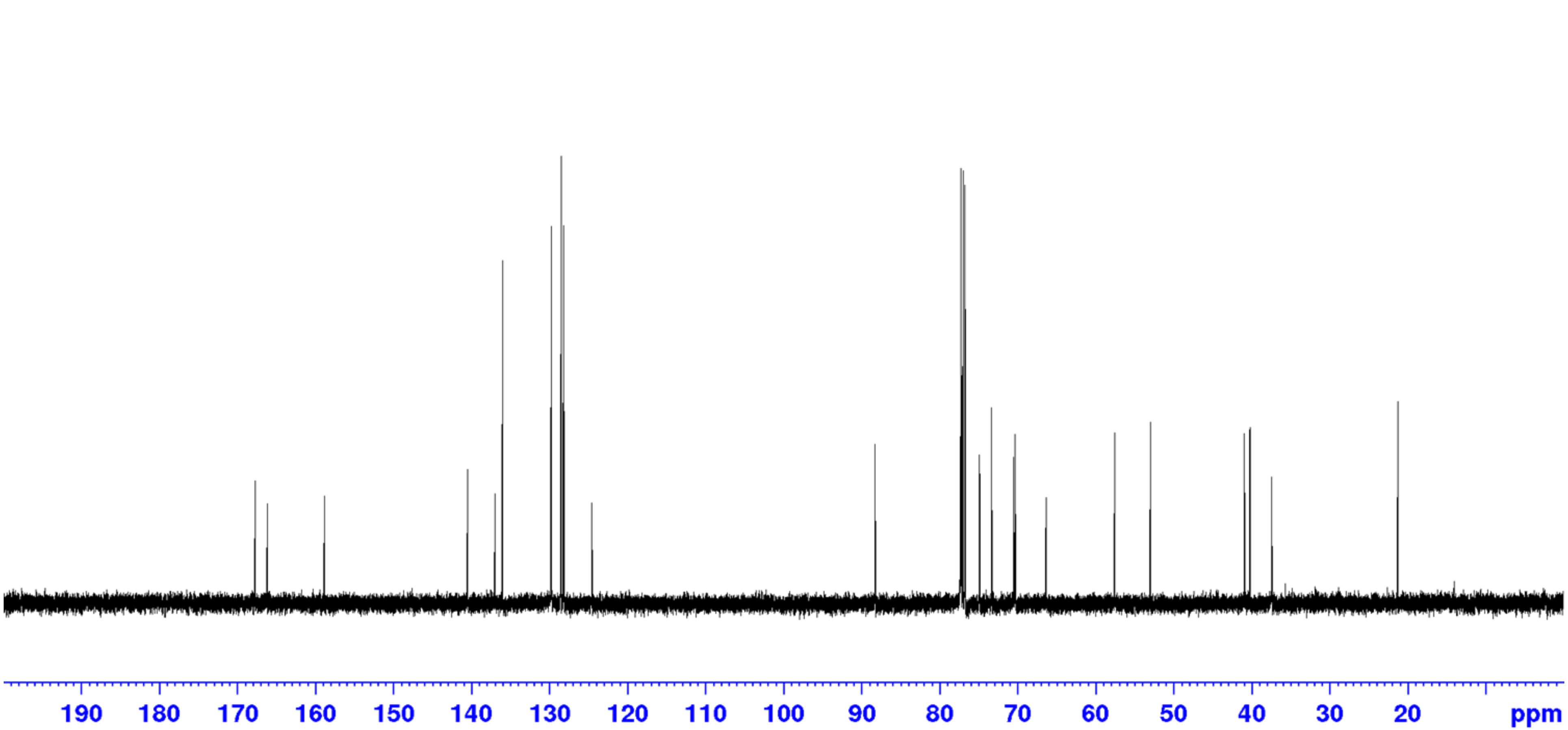

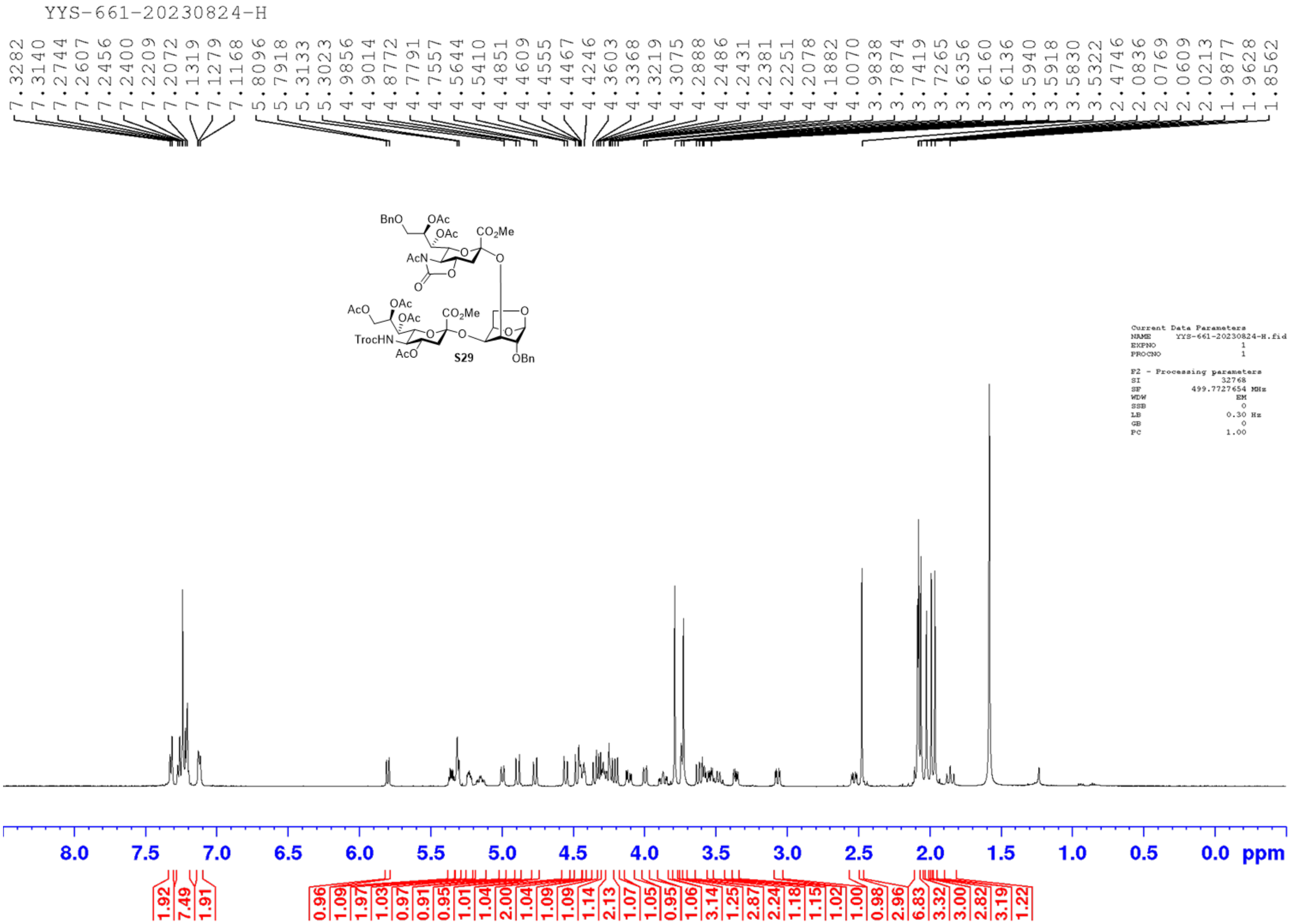
YYS-661-20230824-H
7.3282
7.3140
7.2744
7.2607
7.2456
7.2400
7.2209
7.2072
7.1319
7.1279
7.1168
5.8096
5.7918
5.3133
5.3023
4.9856
4.9014
4.8772
4.7791
4.7557
4.5644
4.5410
4.4851
4.4609
4.4555
4.4467
4.4246
4.3603
4.3368
4.3219
4.3075
4.2888
4.2486
4.2431
4.2381
4.2251
4.2078
4.1882
4.0070
3.9838
3.7874
3.7419
3.7265
3.6356
3.6160
3.6136
3.5940
3.5918
3.5830
3.5322
2.4746
2.0836
2.0769
2.0609
2.0213
1.9877
1.9628
1.8562
BnO
OAc
OAc
CO2Me
AcN
O
O
O
O
AcO
OAc
OAc
CO2Me
TrocHN
O
O
O
O
AcO
S29
OBn
Current Data Parameters
NAME YYS-661-20230824-H.fid
EXPNO 1
PROCNO 1
F2 - Processing parameters
SI 32768
SF 499.7727654 MHz
WDW EM
SSB 0
LB 0.30 Hz
GB 0
PC 1.00
8.0
7.5
7.0
6.5
6.0
5.5
5.0
4.5
4.0
3.5
3.0
2.5
2.0
1.5
1.0
0.5
0.0 ppm
1.92
7.49
1.91
0.96
1.09
1.97
1.03
0.97
0.91
0.95
1.01
1.04
2.00
1.04
1.09
1.09
1.14
2.13
1.07
1.05
0.95
1.06
3.14
1.25
2.87
2.24
1.18
1.15
1.02
1.00
0.98
2.96
6.83
3.32
3.00
2.82
3.19
1.22

YYS-661-20230824-C

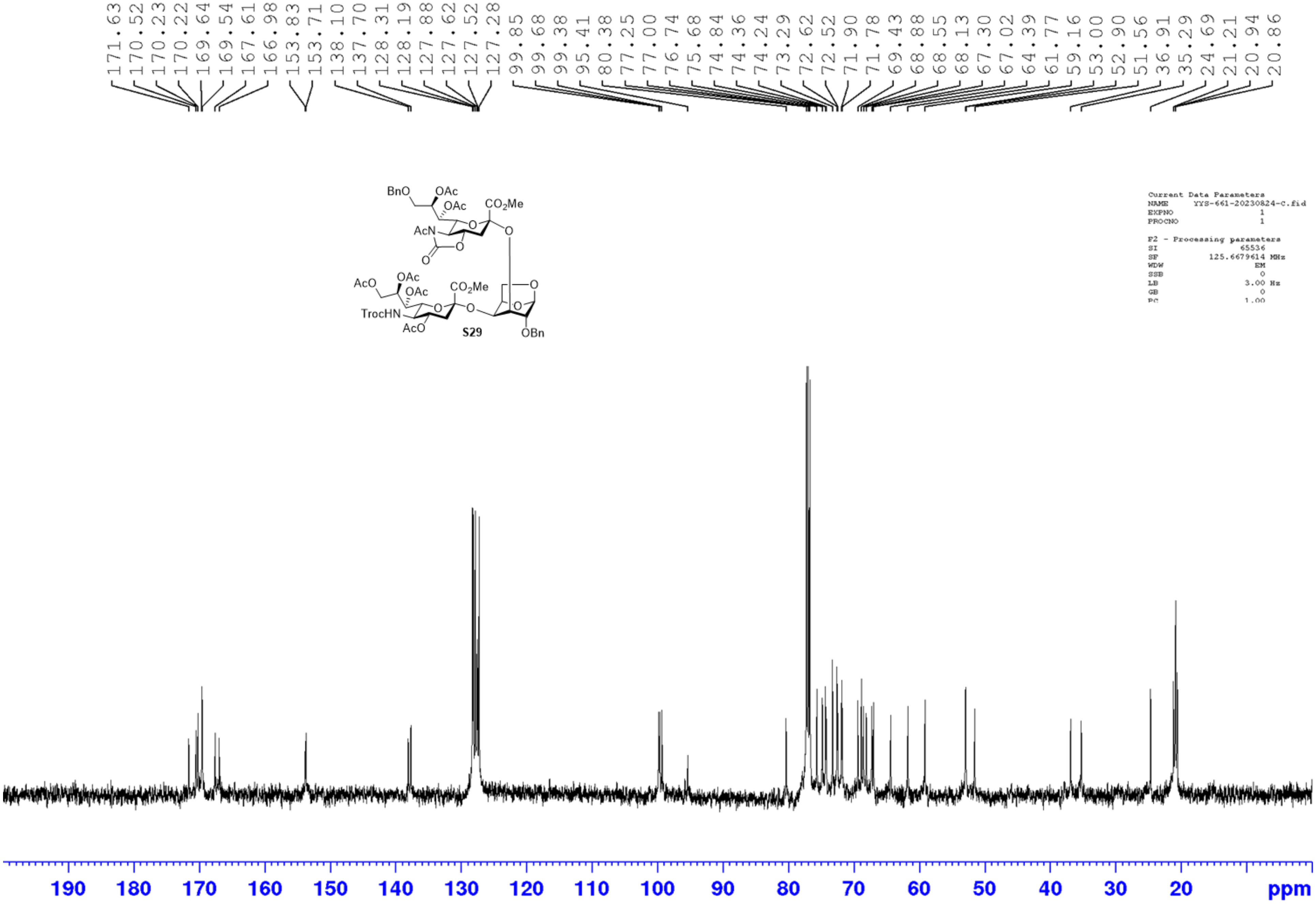

Current Data Parameters
NAME YYS-661-20230824-C.fid
EXPNO 1
PROCNO 1

F2 - Processing parameters
SI 65536
SF 125.6679614 MHz
WDW EM
SSB 0
LB 3.00 Hz
GB 0
PC 1.00

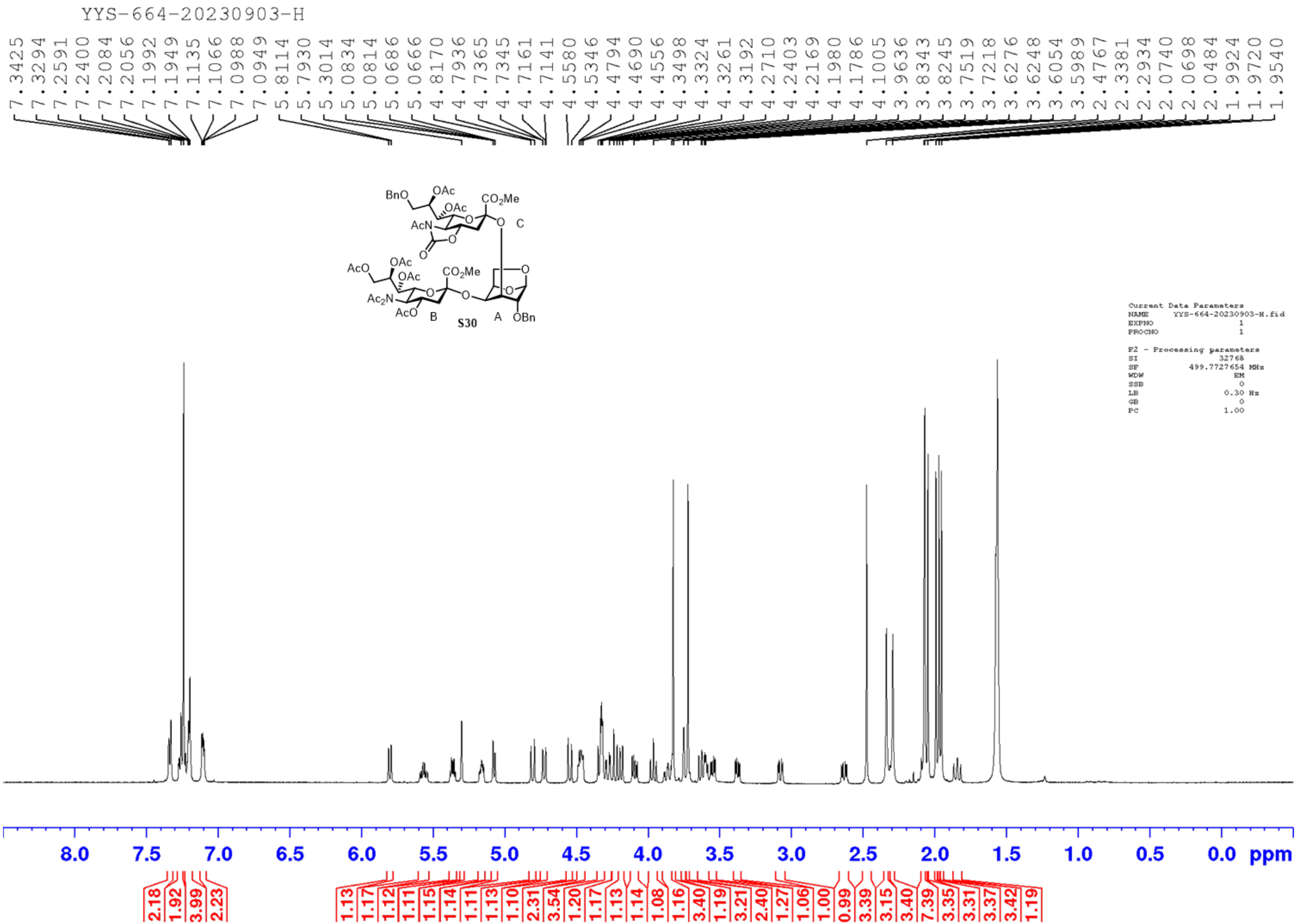

YYS-664-20230903-H
7.3425 7.3294 7.2591 7.2400 7.2084 7.2056 7.1992 7.1949 7.1135 7.1066 7.0988 7.0949 5.8114 5.7930 5.3014 5.0834 5.0814 5.0686 5.0666 4.8170 4.7936 4.7365 4.7345 4.7161 4.7141 4.5580 4.5346 4.4794 4.4690 4.4556 4.3498 4.3324 4.3261 4.3192 4.2710 4.2403 4.2169 4.1980 4.1786 4.1005 3.9636 3.8343 3.8245 3.7519 3.7218 3.6276 3.6248 3.6054 3.5989 2.4767 2.3381 2.2934 2.0740 2.0698 2.0484 1.9924 1.9720 1.9540
BnO
OAc
OAc
CO_2Me
AcN
O
C
AcO
OAc
OAc
CO_2Me
Ac_2N
AcO
B
S30
A
OBn
Current Data Parameters
NAME YYS-664-20230903-H.fid
EXPNO 1
PROCNO 1
F2 - Processing parameters
SI 32768
SF 499.7727654 MHz
WDW EM
SSB 0
LB 0.30 Hz
GB 0
PC 1.00
8.0 7.5 7.0 6.5 6.0 5.5 5.0 4.5 4.0 3.5 3.0 2.5 2.0 1.5 1.0 0.5 0.0 ppm
2.18 1.92 3.99 2.23 1.13 1.17 1.12 1.11 1.15 1.14 1.11 1.13 1.10 2.31 3.54 1.20 1.17 1.13 1.14 1.08 1.16 3.40 1.19 3.21 2.40 1.27 1.06 1.00 0.99 3.39 3.15 3.40 7.39 3.35 3.31 3.37 3.42 1.19

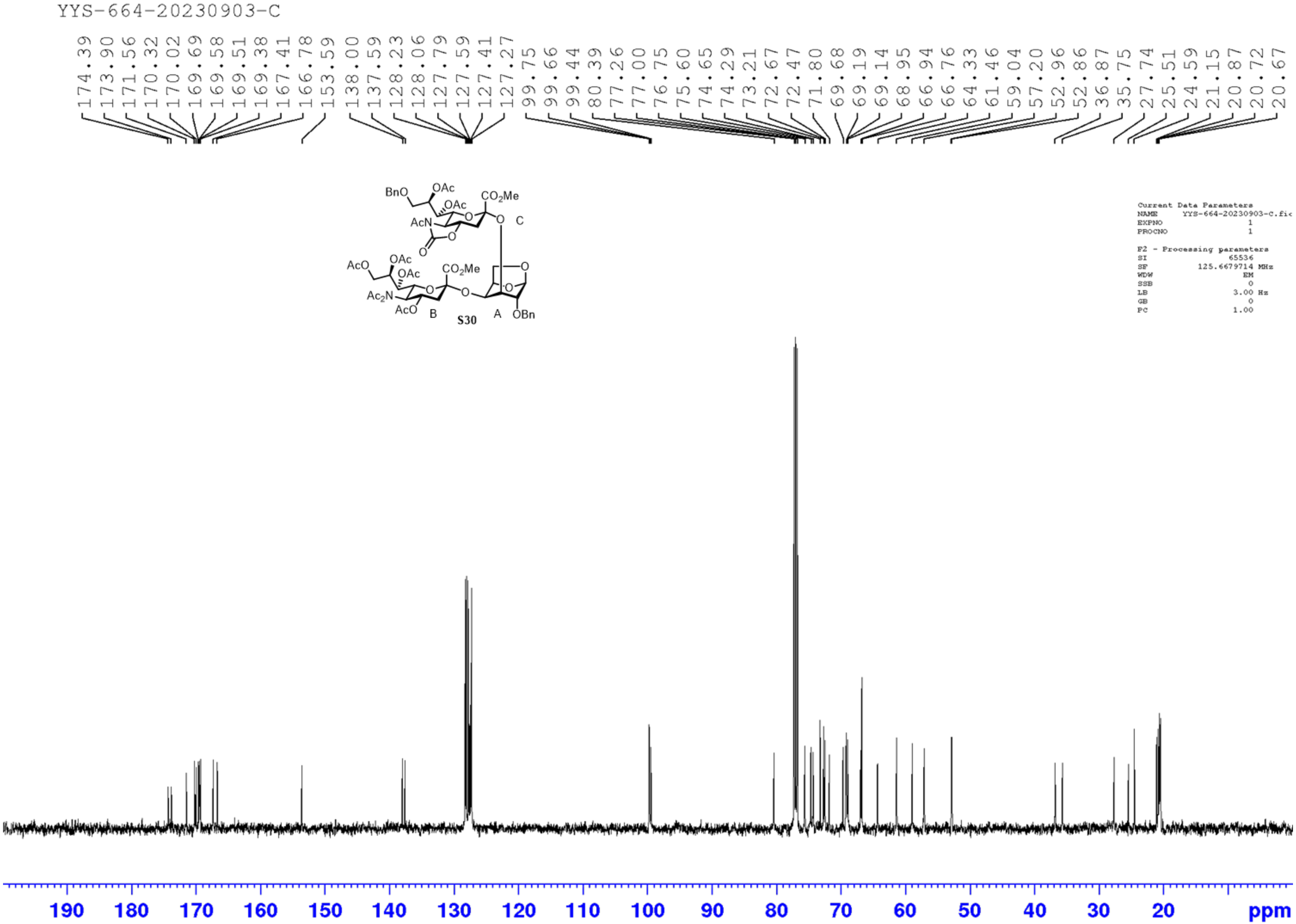
YYS-664-20230903-C
174.39
173.90
171.56
170.32
170.02
169.69
169.58
169.51
169.38
167.41
166.78
153.59
138.00
137.59
128.23
128.06
127.79
127.59
127.41
127.27
99.75
99.66
99.44
80.39
77.26
77.00
76.75
75.60
74.65
74.29
73.21
72.67
72.47
71.80
69.68
69.19
69.14
68.95
66.94
66.76
64.33
61.46
59.04
57.20
52.96
52.86
36.87
35.75
27.74
25.51
24.59
21.15
20.87
20.72
20.67
BnO
OAc
OAc
CO2Me
AcN
O
C
AcO
OAc
OAc
CO2Me
Ac2N
AcO
B
S30
A
OBn
Current Data Parameters
NAME YYS-664-20230903-C.fic
EXPNO 1
PROCNO 1
F2 - Processing parameters
SI 65536
SF 125.6679714 MHz
WDW EM
SSB 0
LB 3.00 Hz
GB 0
PC 1.00
190
180
170
160
150
140
130
120
110
100
90
80
70
60
50
40
30
20
ppm

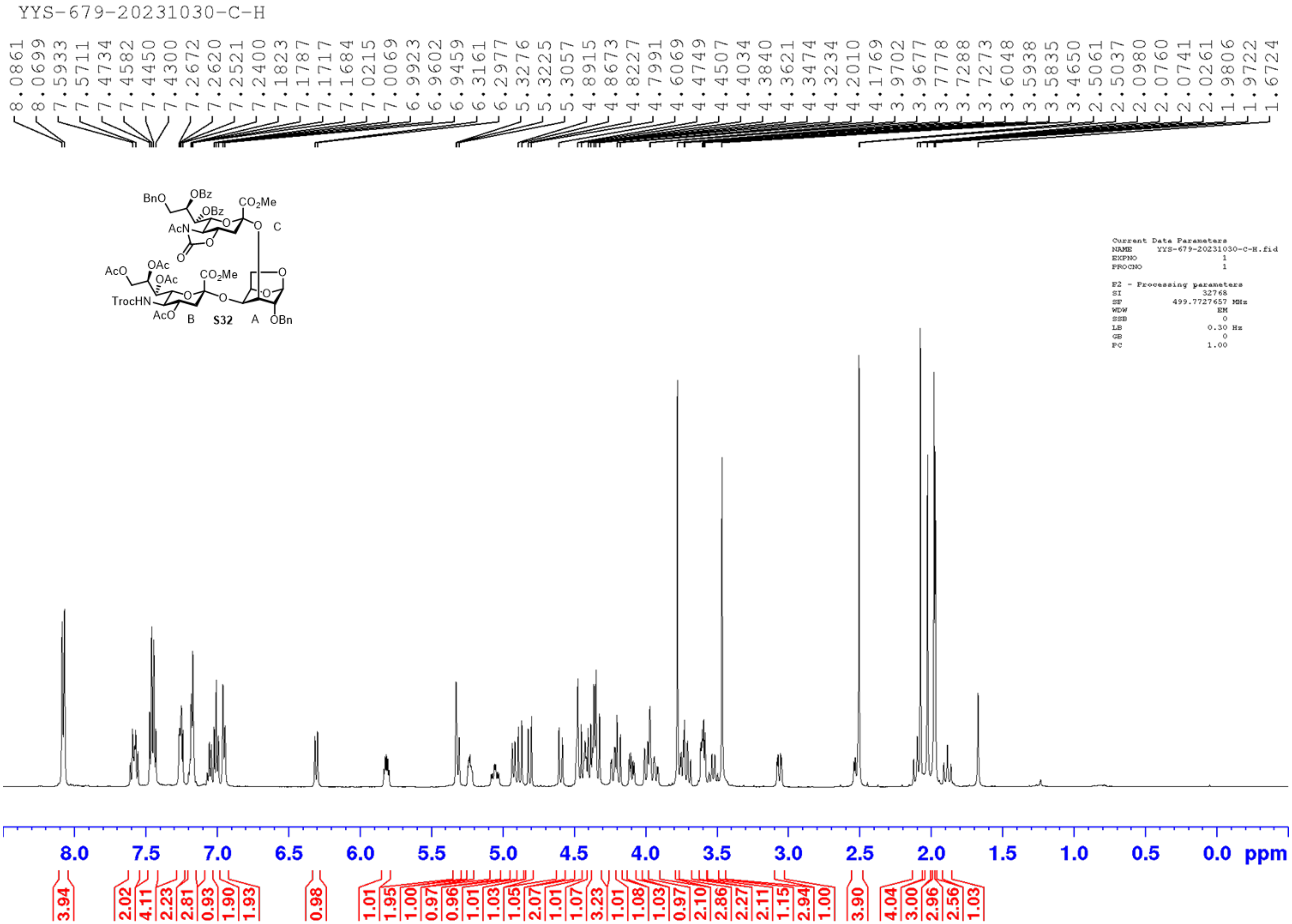
YYS-679-20231030-C-H
S32
Current Data Parameters
NAME YYS-679-20231030-C-H.fid
EXPNO 1
PROCNO 1
F2 - Processing parameters
SI 32768
SF 499.7727657 MHz
WDW EM
SSB 0
LB 0.30 Hz
GB 0
PC 1.00
ppm

YYS-679-20231030-C

170.55, 170.05, 169.57, 167.19, 165.41, 165.12, 153.66, 138.14, 137.56, 133.48, 133.19, 130.14, 129.99, 129.84, 129.58, 128.68, 128.51, 128.24, 128.00, 127.54, 127.49, 127.28, 127.22, 100.66, 99.93, 99.10, 80.10, 77.25, 77.00, 76.75, 75.61, 75.00, 74.43, 73.91, 73.26, 72.92, 72.50, 72.22, 72.01, 70.47, 69.01, 68.47, 68.25, 67.29, 67.17, 64.41, 61.78, 59.48, 53.04, 52.83, 51.61, 36.93, 35.68, 24.77, 20.91, 20.82, 20.67, 20.60

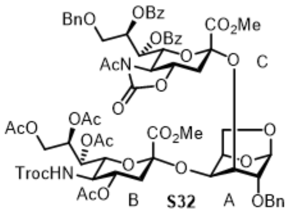

Current Data Parameters
NAME YYS-679-20231030-C.fid
EXPNO 1
PROCNO 1

F2 - Processing parameters
SI 65536
SF 125.6679604 MHz
WDW EM
SSB 0
LB 3.00 Hz
GB 0
PC 1.00

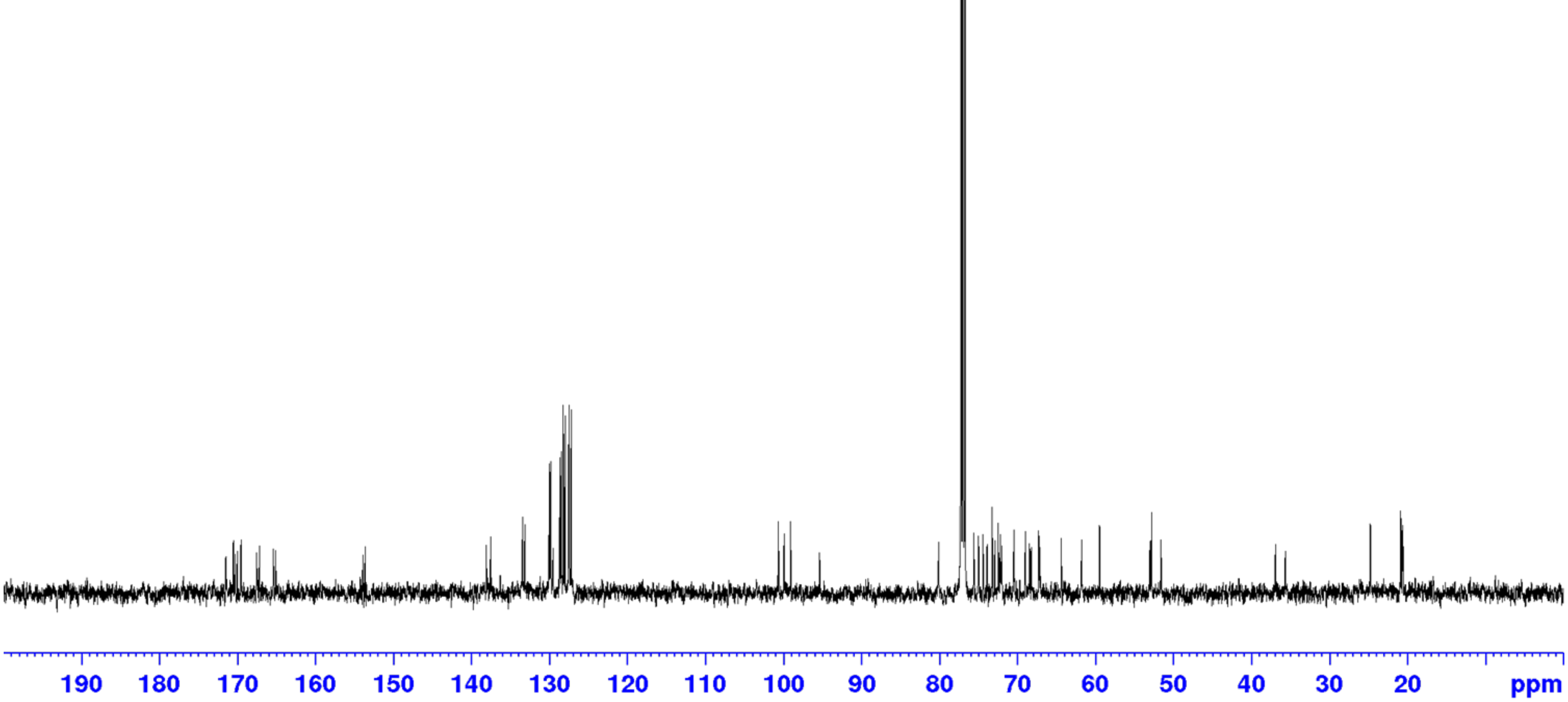

YYS-684-20231101-C-H

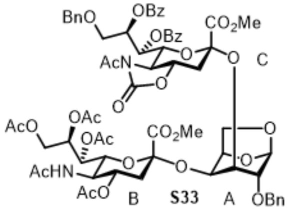

Current Data Parameters
NAME YYS-684-20231101-C-H.fi
EXPNO 1
PROCNO 1

F2 - Processing parameters
SI 32768
SF 499.7727654 MHz
WDW EM
SSB 0
LB 0.30 Hz
GB 0
PC 1.00

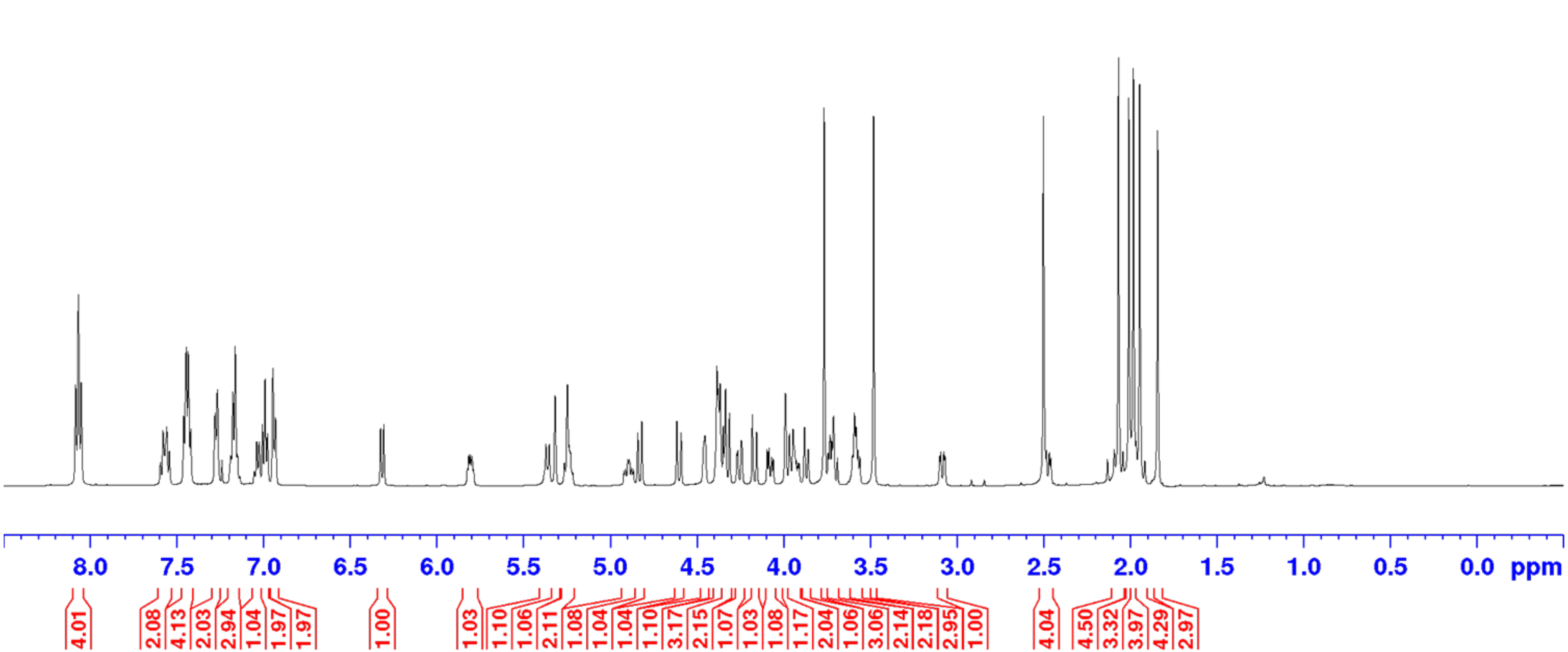

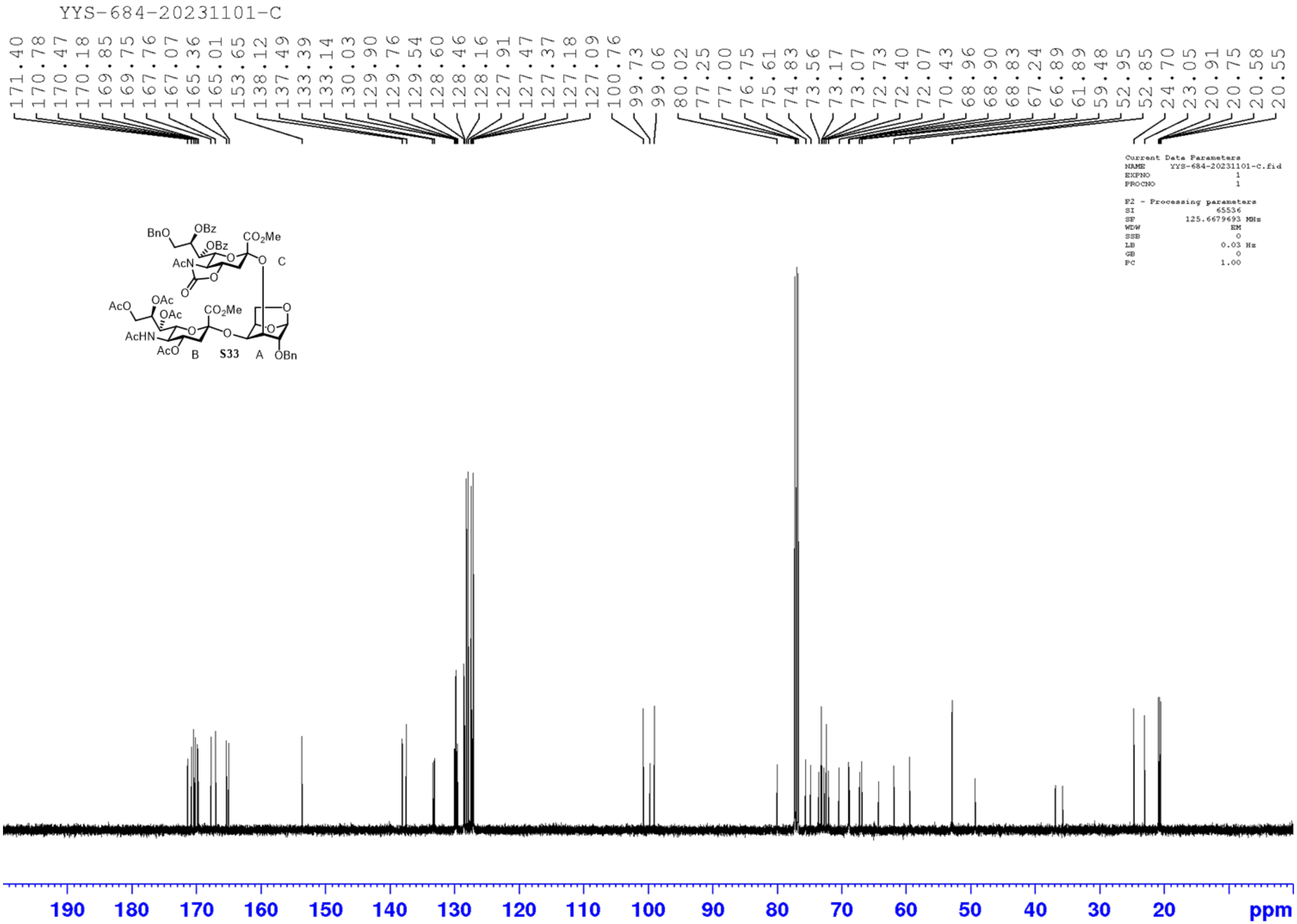
YYS-684-20231101-C
171.40
170.78
170.47
170.18
169.85
169.75
167.76
167.07
165.36
165.01
153.65
138.12
137.49
133.39
133.14
130.03
129.90
129.76
129.54
128.60
128.46
128.16
127.91
127.47
127.37
127.18
127.09
100.76
99.73
99.06
80.02
77.25
77.00
76.75
75.61
74.83
73.56
73.17
73.07
72.73
72.40
72.07
70.43
68.96
68.90
68.83
67.24
66.89
61.89
59.48
52.95
52.85
24.70
23.05
20.91
20.75
20.58
20.55
Current Data Parameters
NAME YYS-684-20231101-C.fid
EXPNO 1
PROCNO 1
F2 - Processing parameters
SI 65536
SF 125.6679693 MHz
WDW EM
SSB 0
LB 0.03 Hz
GB 0
PC 1.00
BnO
OBz
OBz
CO2Me
AcN
O
O
O
C
AcO
OAc
OAc
CO2Me
AcHN
AcO
B
S33
A
OBn
190
180
170
160
150
140
130
120
110
100
90
80
70
60
50
40
30
20
ppm

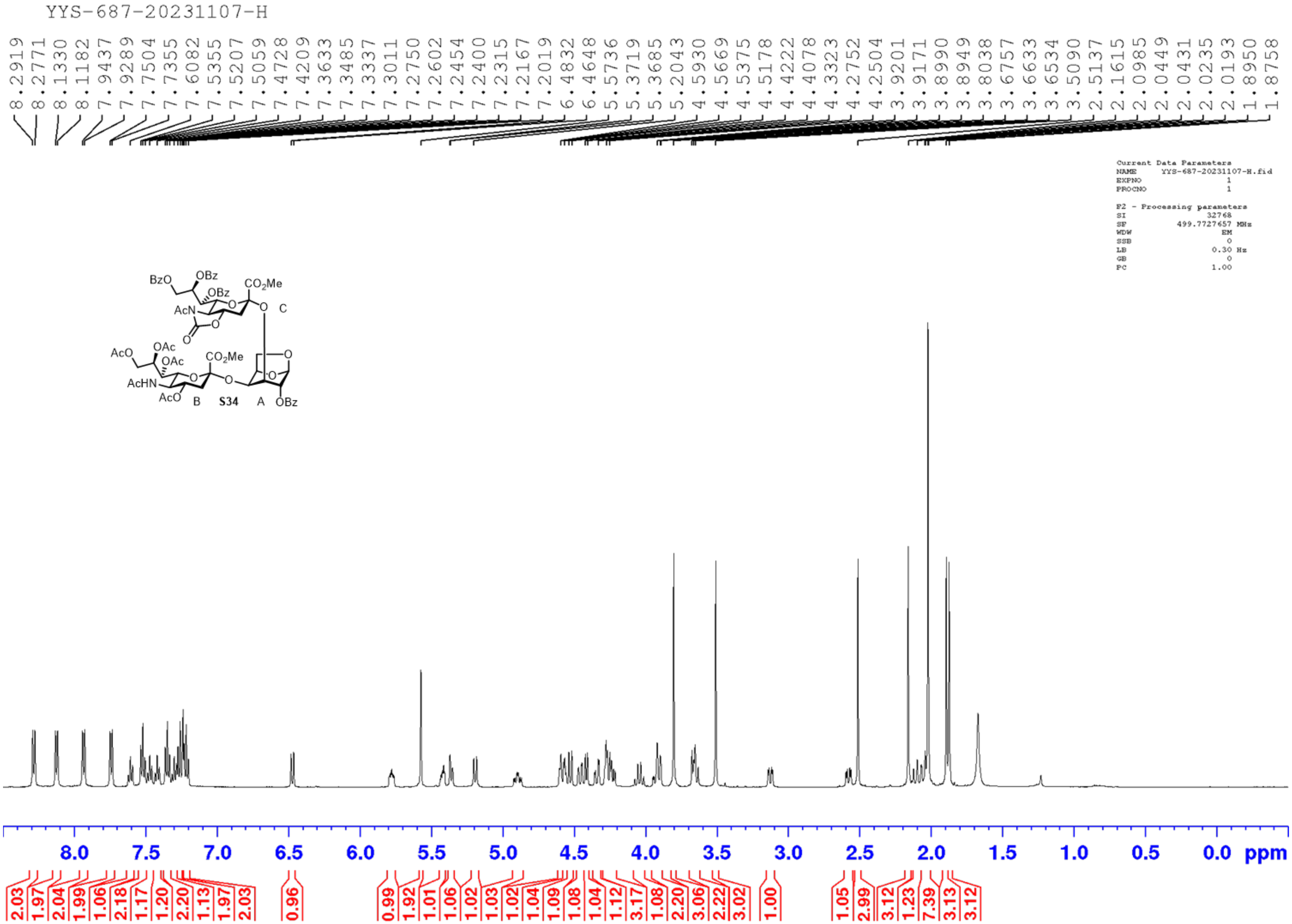
YYS-687-20231107-H
8.2919 8.2771 8.1330 8.1182 7.9437 7.9289 7.7504 7.7355 7.6082 7.5355 7.5207 7.5059 7.4728 7.4209 7.3633 7.3485 7.3337 7.3011 7.2750 7.2602 7.2454 7.2400 7.2315 7.2167 7.2019 6.4832 6.4648 5.5736 5.3719 5.3685 5.2043 4.5930 4.5669 4.5375 4.5178 4.4222 4.4078 4.3323 4.2752 4.2504 3.9201 3.9171 3.8990 3.8949 3.8038 3.6757 3.6633 3.6534 3.5090 2.5137 2.1615 2.0985 2.0449 2.0431 2.0235 2.0193 1.8950 1.8758
Current Data Parameters
NAME YYS-687-20231107-H.fid
EXPNO 1
PROCNO 1
F2 - Processing parameters
SI 32768
SF 499.7727657 MHz
WDW EM
SSB 0
LB 0.30 Hz
GB 0
PC 1.00
BzO
OBz
OBz
CO2Me
AcN
O
C
AcO
OAc
OAc
CO2Me
AcHN
AcO
B
S34
A
OBz
8.0 7.5 7.0 6.5 6.0 5.5 5.0 4.5 4.0 3.5 3.0 2.5 2.0 1.5 1.0 0.5 0.0 ppm
2.03 1.97 2.04 1.99 1.06 2.18 1.17 1.20 2.20 1.13 1.97 2.03 0.96 0.99 1.92 1.01 1.06 1.02 1.03 1.02 1.04 1.09 1.08 1.04 1.12 3.17 1.08 2.20 3.06 2.22 3.02 1.00 1.05 2.99 3.12 1.23 7.39 3.13 3.12

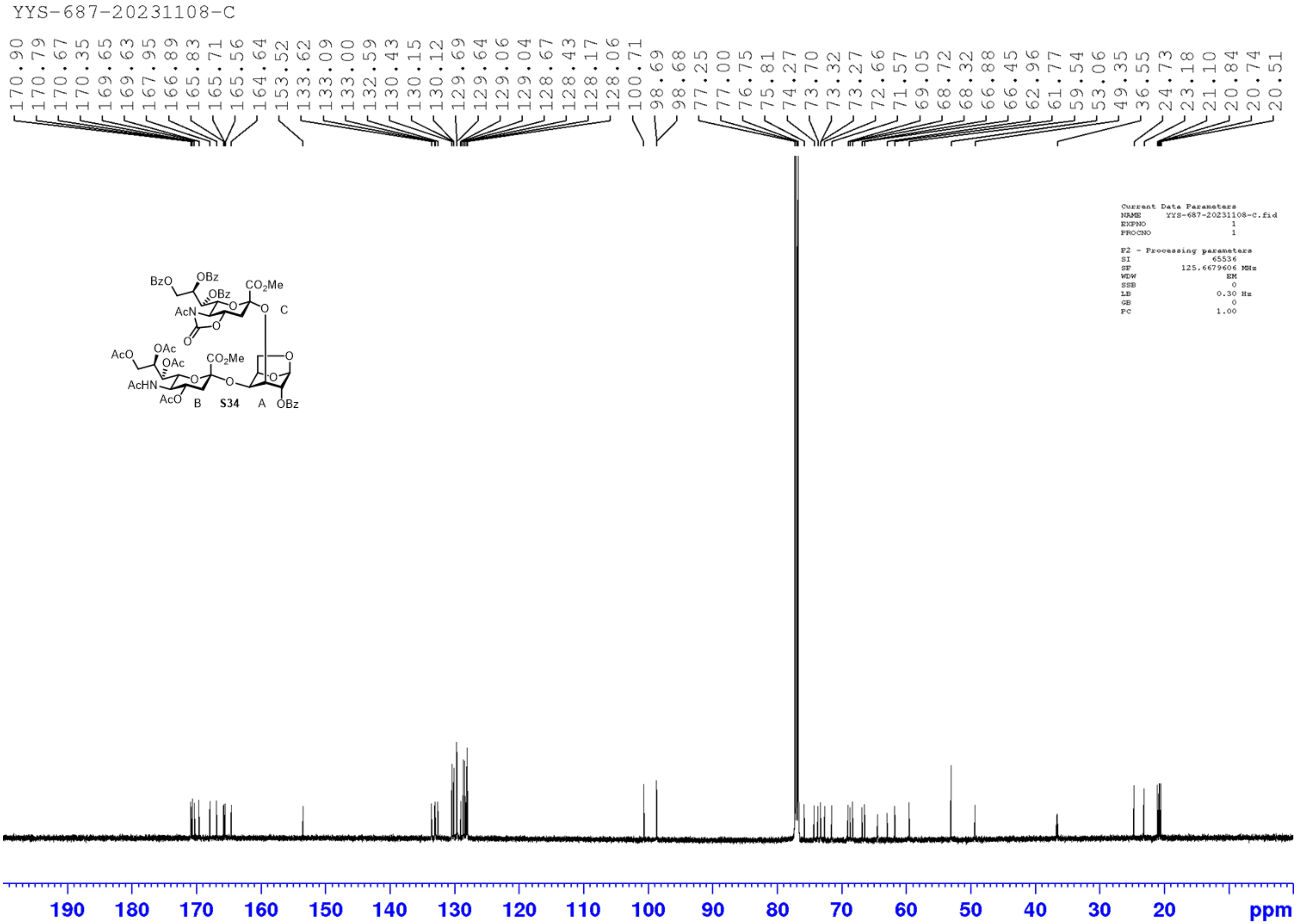
YYS-687-20231108-C
170.90
170.79
170.67
170.35
169.65
169.63
167.95
166.89
165.83
165.71
165.56
164.64
153.52
133.62
133.09
133.00
132.59
130.43
130.15
130.12
129.69
129.64
129.06
129.04
128.67
128.43
128.17
128.06
100.71
98.69
98.68
77.25
77.00
76.75
75.81
74.27
73.70
73.32
73.27
72.66
71.57
69.05
68.72
68.32
66.88
66.45
62.96
61.77
59.54
53.06
49.35
36.55
24.73
23.18
21.10
20.84
20.74
20.51
Current Data Parameters
NAME YYS-687-20231108-C.fid
EXPNO 1
PROCNO 1
F2 - Processing parameters
SI 65536
SF 125.6679606 MHz
WDW EM
SSB 0
LB 0.30 Hz
GB 0
PC 1.00
BzO
OBz
OBz
CO2Me
AcN
O
C
AcO
OAc
OAc
CO2Me
AcHN
AcO
B
S34
A
OBz
190
180
170
160
150
140
130
120
110
100
90
80
70
60
50
40
30
20
ppm

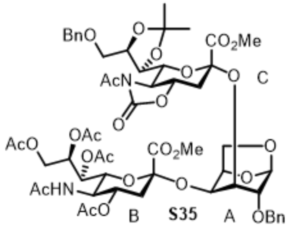
BnO
CO2Me
AcN
O
C
AcO
OAc
OAc
CO2Me
AcHN
AcO
B
S35
A
OBn

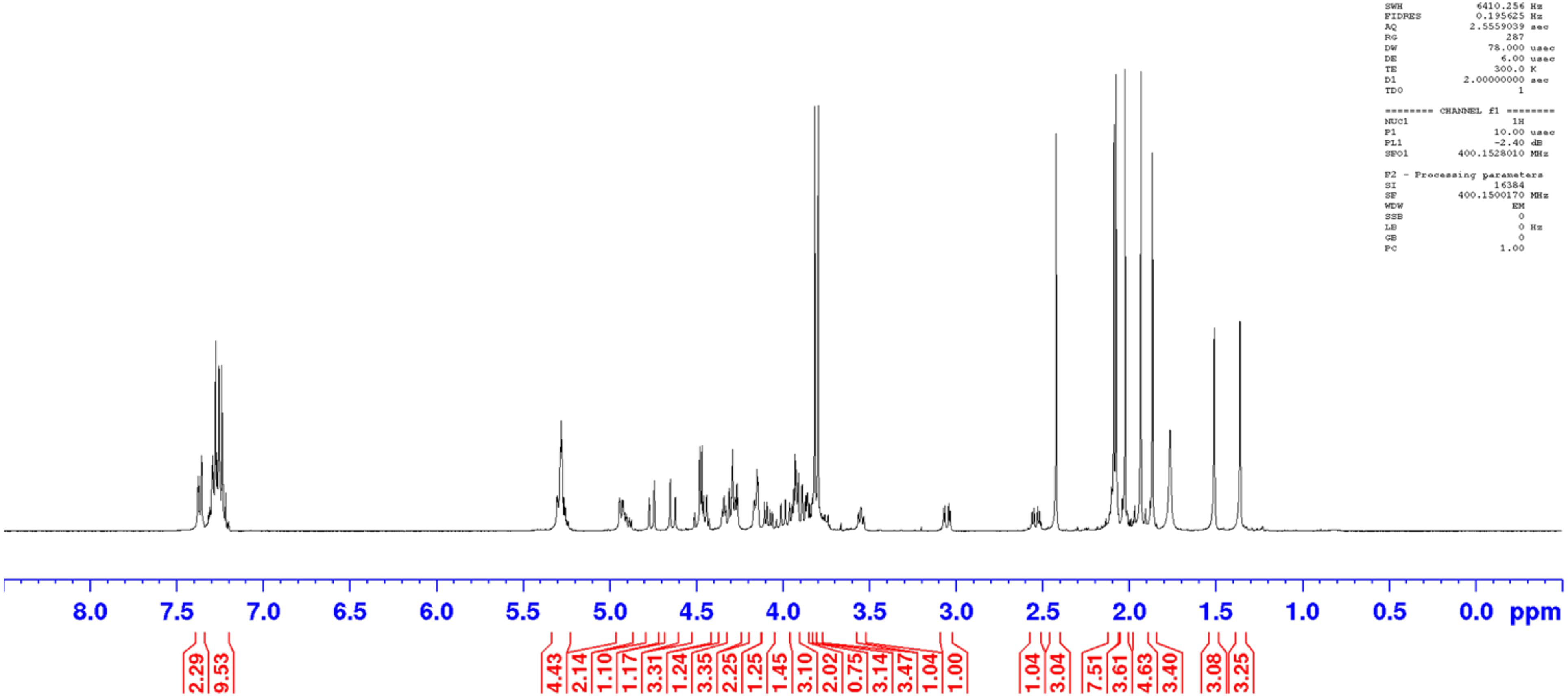
YYS-683-20231107
7.3793 7.3755 7.3585 7.2959 7.2924 7.2888 7.2848 7.2750 7.2709 7.2674 7.2640 7.2550 7.2400 7.2361 7.2183 5.3036 5.2843 5.2800 5.2753 5.2688 4.7412 4.6504 4.4787 4.4667 4.4572 4.4404 4.3392 4.3079 4.2955 4.2894 4.2760 4.2646 4.1478 4.1434 3.9360 3.9284 3.9227 3.9207 3.9138 3.9088 3.8894 3.8852 3.8694 3.8625 3.8577 3.8547 3.8144 3.7950 2.4201 2.1021 2.0996 2.0862 2.0739 2.0214 1.9302 1.8631 1.5069 1.3579
Current Data Parameters
NAME YYS-683-20231107
EXPNO 1
PROCNO 1
F2 - Acquisition Parameters
Date_ 20231107
Time 6.52
INSTRUM spect
PROBHD 5 mm DUL 13C-1
PULPROG zg30
TD 32768
SOLVENT CDCl3
NS 22
DS 0
SWH 6410.256 Hz
FIDRES 0.195625 Hz
AQ 2.5559039 sec
RG 287
DW 78.000 usec
DE 6.00 usec
TE 300.0 K
D1 2.00000000 sec
TD0 1
======== CHANNEL f1 ========
NUC1 1H
P1 10.00 usec
PL1 -2.40 dB
SFO1 400.1528010 MHz
F2 - Processing parameters
SI 16384
SF 400.1500170 MHz
WDW EM
SSB 0
LB 0 Hz
GB 0
PC 1.00
8.0 7.5 7.0 6.5 6.0 5.5 5.0 4.5 4.0 3.5 3.0 2.5 2.0 1.5 1.0 0.5 0.0 ppm
2.29 9.53 4.43 2.14 1.10 1.17 3.31 1.24 3.35 2.25 1.25 1.45 3.10 2.02 0.75 3.14 3.47 1.04 1.00 1.04 3.04 7.51 3.61 4.63 3.40 3.08 3.25

YYS-683-20231031-C

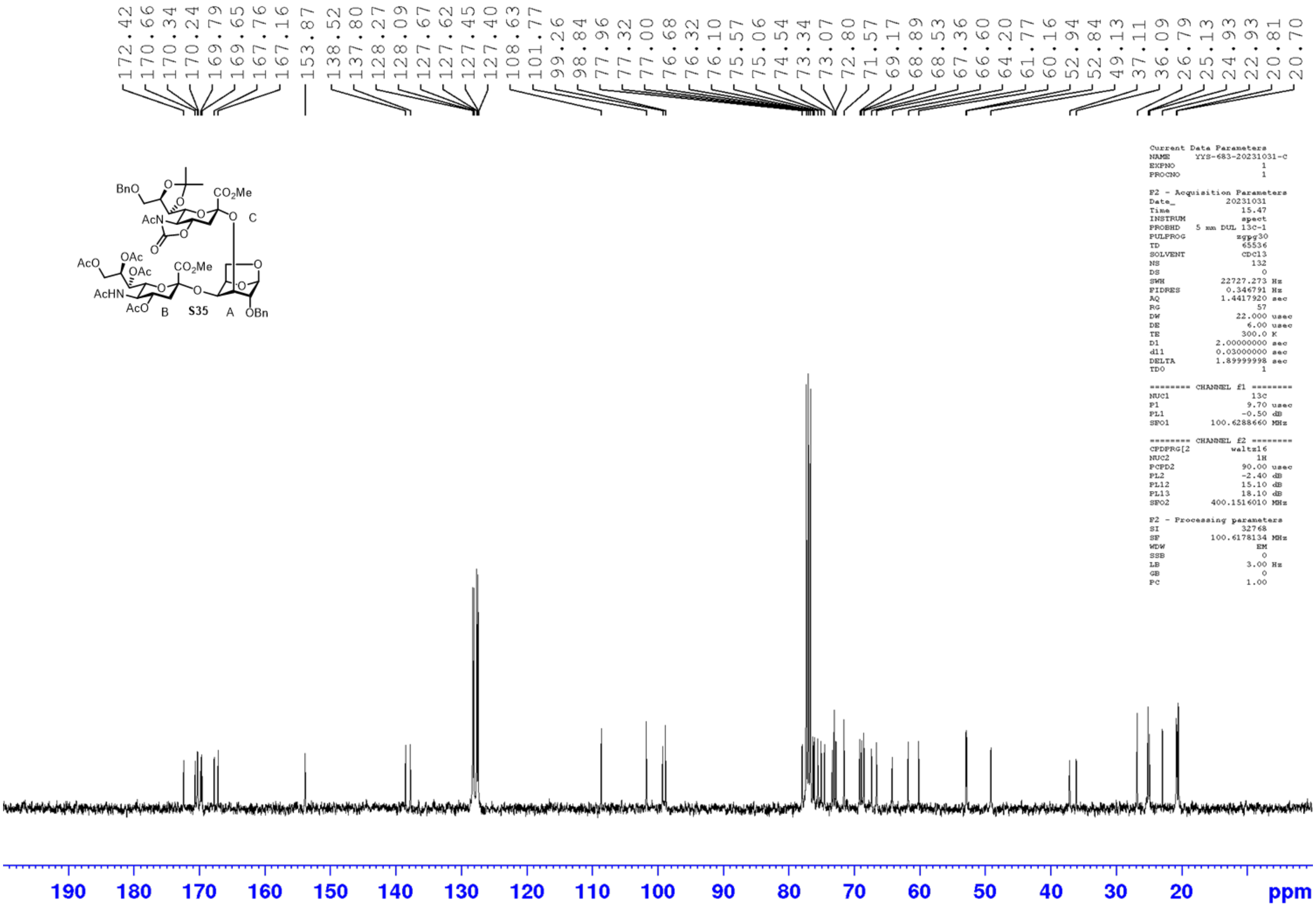

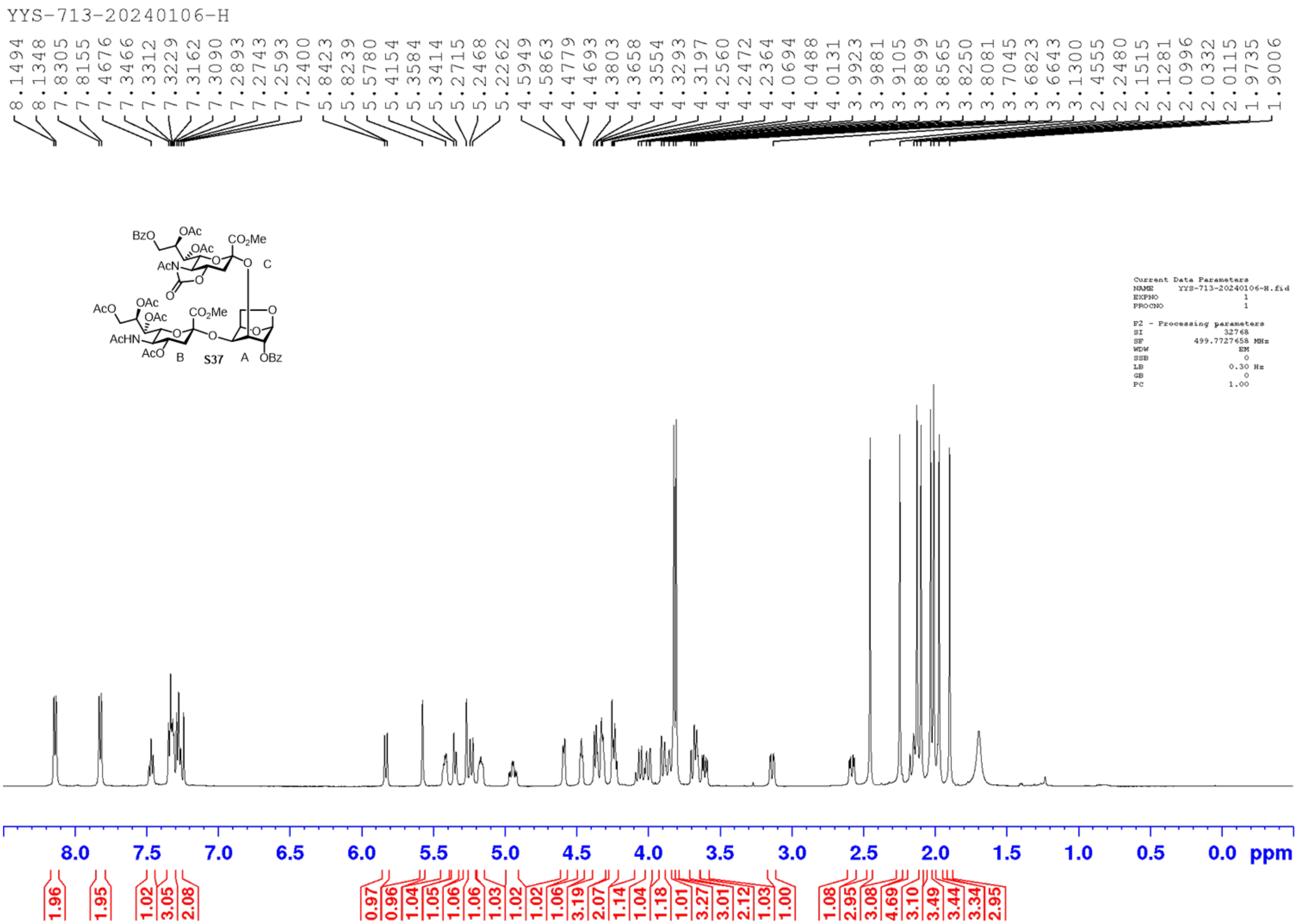
YYS-713-20240106-H
8.1494
8.1348
7.8305
7.8155
7.4676
7.3466
7.3312
7.3229
7.3162
7.3090
7.2893
7.2743
7.2593
7.2400
5.8423
5.8239
5.5780
5.4154
5.3584
5.3414
5.2715
5.2468
5.2262
4.5949
4.5863
4.4779
4.4693
4.3803
4.3658
4.3554
4.3293
4.3197
4.2560
4.2472
4.2364
4.0694
4.0488
4.0131
3.9923
3.9881
3.9105
3.8899
3.8565
3.8250
3.8081
3.7045
3.6823
3.6643
3.1300
2.4555
2.2480
2.1515
2.1281
2.0996
2.0332
2.0115
1.9735
1.9006
BzO
OAc
OAc
CO_2Me
AcN
O
O
C
O
O
AcO
OAc
OAc
CO_2Me
O
AcHN
O
O
AcO
B
S37
A
OBz
Current Data Parameters
NAME YYS-713-20240106-H.fid
EXPNO 1
PROCNO 1
F2 - Processing parameters
SI 32768
SF 499.7727658 MHz
WDW EM
SSB 0
LB 0.30 Hz
GB 0
PC 1.00
8.0
7.5
7.0
6.5
6.0
5.5
5.0
4.5
4.0
3.5
3.0
2.5
2.0
1.5
1.0
0.5
0.0
ppm
1.96
1.95
1.02
3.05
2.08
0.97
0.96
1.04
1.05
1.06
1.06
1.03
1.02
1.02
1.06
3.19
2.07
1.14
1.04
1.18
1.01
3.27
3.01
2.12
1.03
1.00
1.08
2.95
3.08
4.69
3.10
3.49
3.44
3.34
2.95

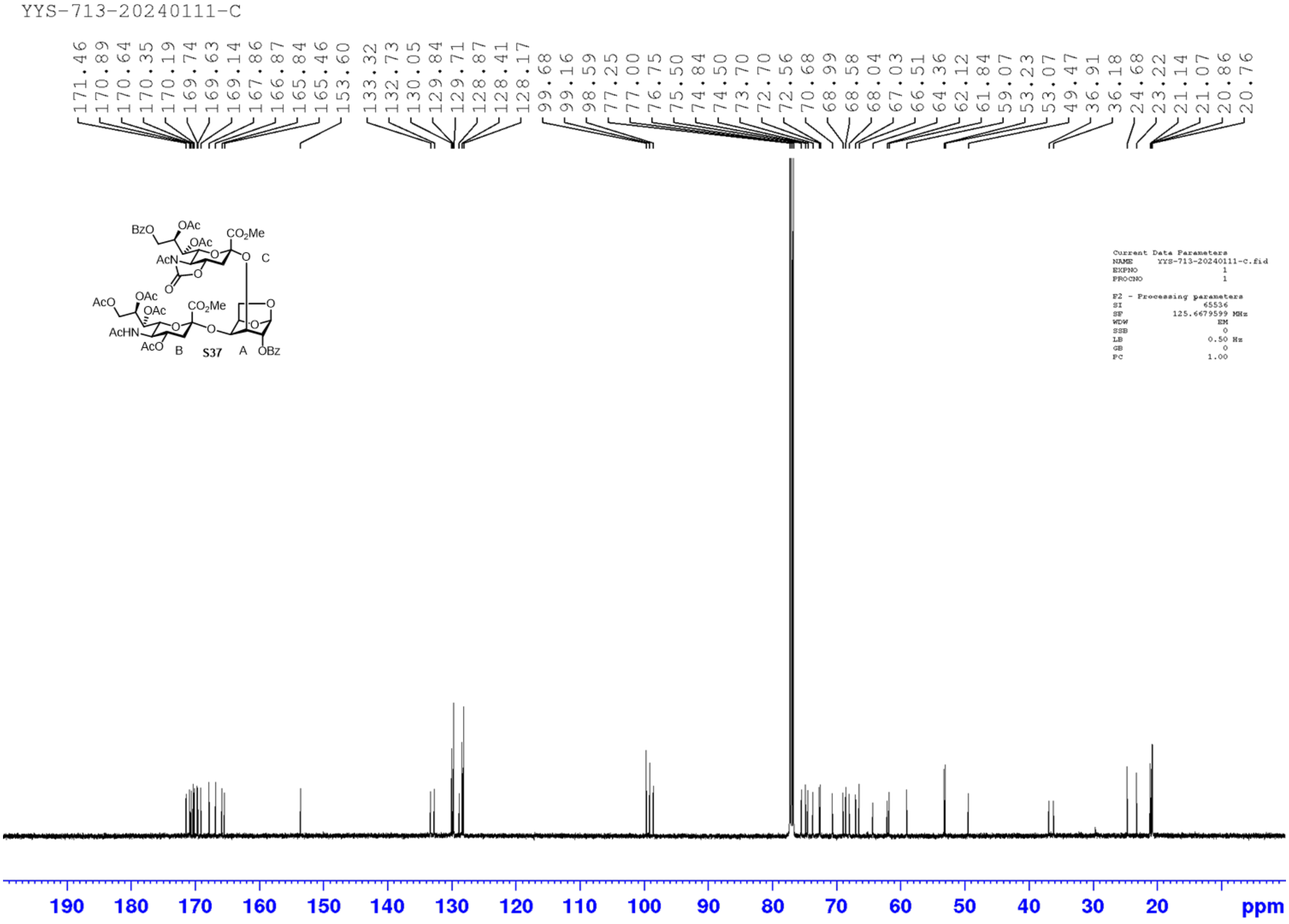
YYS-713-20240111-C
171.46 170.89 170.64 170.35 170.19 169.74 169.63 169.14 167.86 166.87 165.84 165.46 153.60 133.32 132.73 130.05 129.84 129.71 128.87 128.41 128.17 99.68 99.16 98.59 77.25 77.00 76.75 75.50 74.84 74.50 73.70 72.70 72.56 70.68 68.99 68.58 68.04 67.03 66.51 64.36 62.12 61.84 59.07 53.23 53.07 49.47 36.91 36.18 24.68 23.22 21.14 21.07 20.86 20.76
BzO
OAc
OAc
CO2Me
AcN
O
C
AcO
OAc
OAc
CO2Me
AcHN
AcO
B
S37
A
OBz
Current Data Parameters
NAME YYS-713-20240111-C.fid
EXPNO 1
PROCNO 1
F2 - Processing parameters
SI 65536
SF 125.6679599 MHz
WDW EM
SSB 0
LB 0.50 Hz
GB 0
PC 1.00
190 180 170 160 150 140 130 120 110 100 90 80 70 60 50 40 30 20 ppm

KYT-263-Bn-H

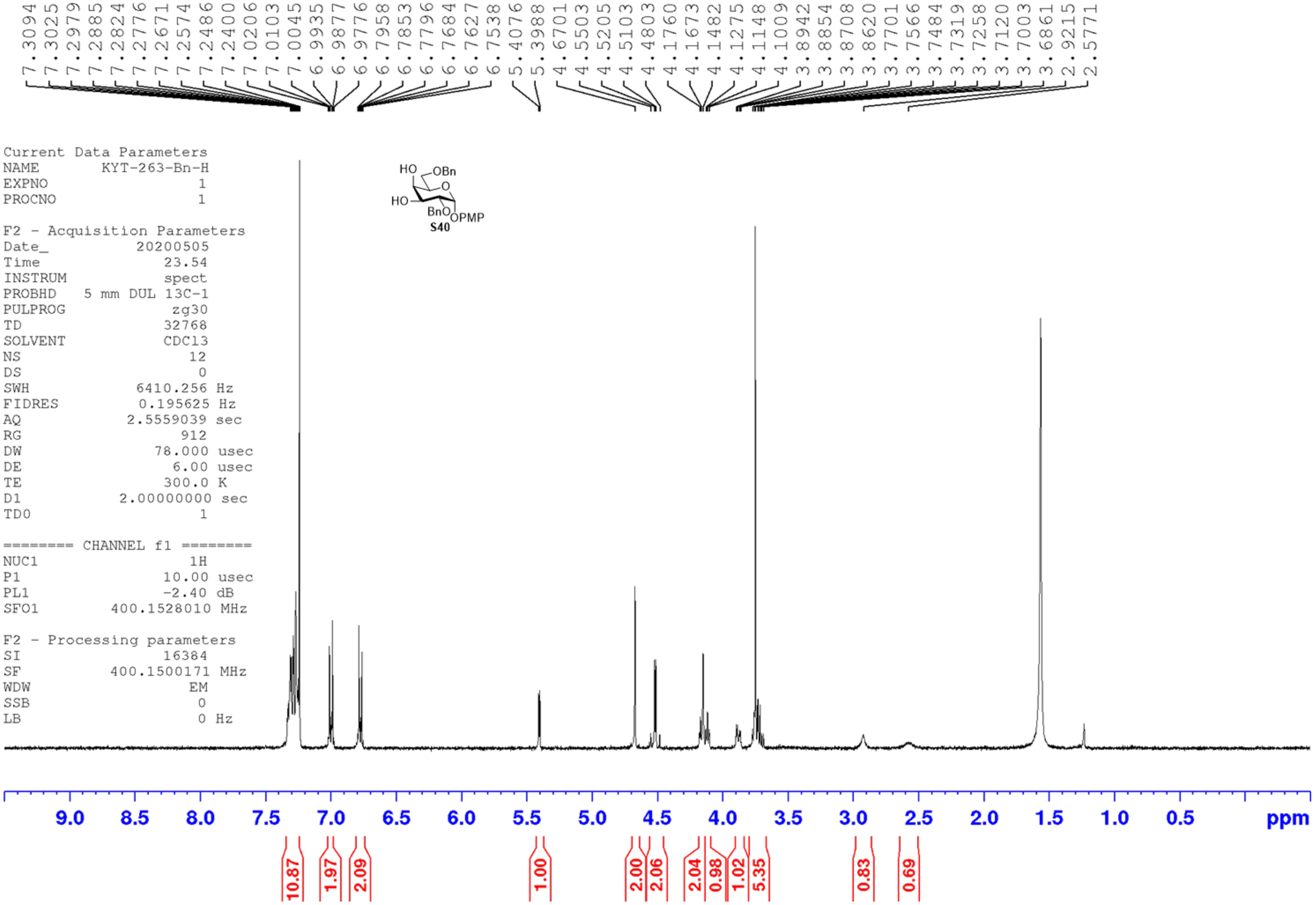

S40

Current Data Parameters
NAME KYT-263-Bn-H
EXPNO 1
PROCNO 1

F2 - Acquisition Parameters
Date_ 20200505
Time 23.54
INSTRUM spect
PROBHD 5 mm DUL 13C-1
PULPROG zg30
TD 32768
SOLVENT CDCl3
NS 12
DS 0
SWH 6410.256 Hz
FIDRES 0.195625 Hz
AQ 2.5559039 sec
RG 912
DW 78.000 usec
DE 6.00 usec
TE 300.0 K
D1 2.00000000 sec
TD0 1

======== CHANNEL f1 ========
NUC1 1H
P1 10.00 usec
PL1 -2.40 dB
SFO1 400.1528010 MHz

F2 - Processing parameters
SI 16384
SF 400.1500171 MHz
WDW EM
SSB 0
LB 0 Hz

KYT-263-Bn-C

155.05
150.98
137.73
137.69
128.50
128.32
127.99
127.66
127.56
118.41
114.46
96.58
77.25
77.00
76.75
76.33
73.45
72.68
69.80
69.66
69.23
69.05
55.54

Current Data Parameters
NAME KYT-263-Bn-C
EXPNO 1
PROCNO 1

F2 - Processing parameters
SI 65536
SF 125.6713878 MHz
WDW EM
SSB 0
LB 0.30 Hz
GB 0
PC 1.00

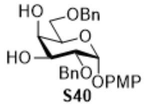

S40

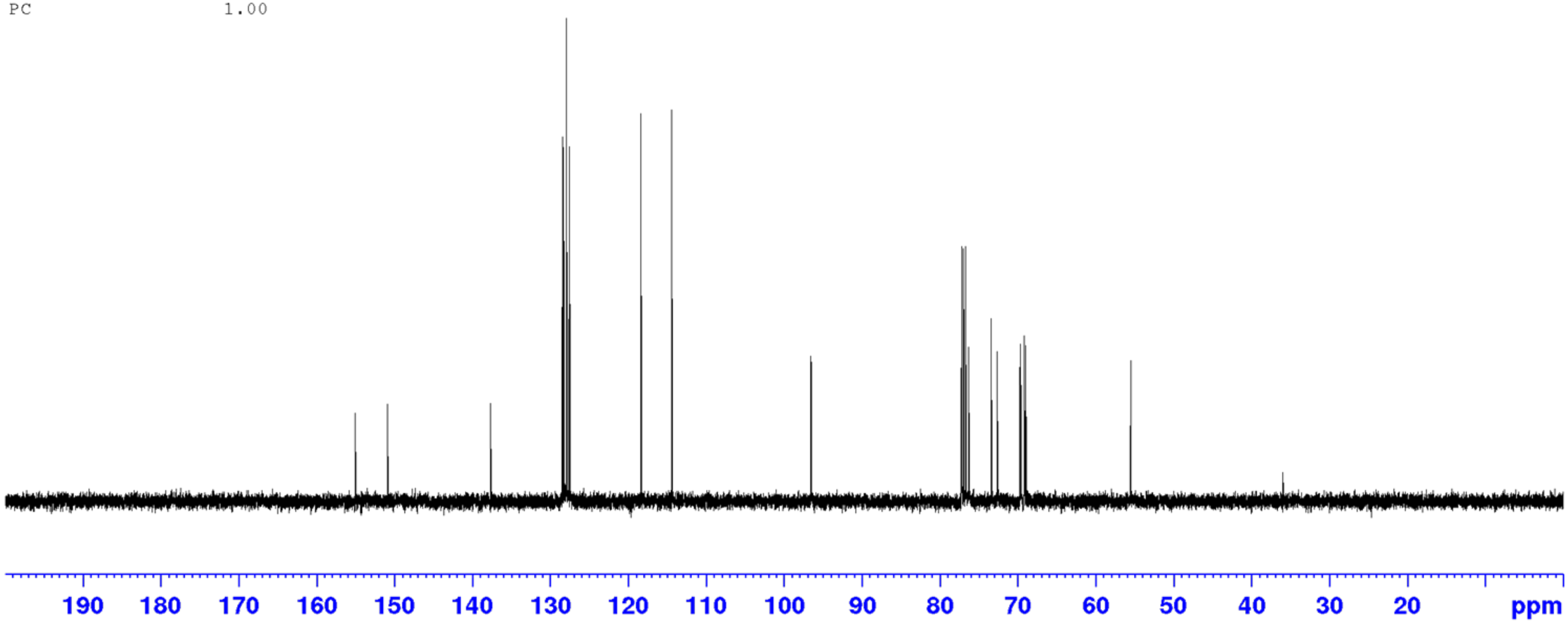

YYS-572-NHXX-20230103-H

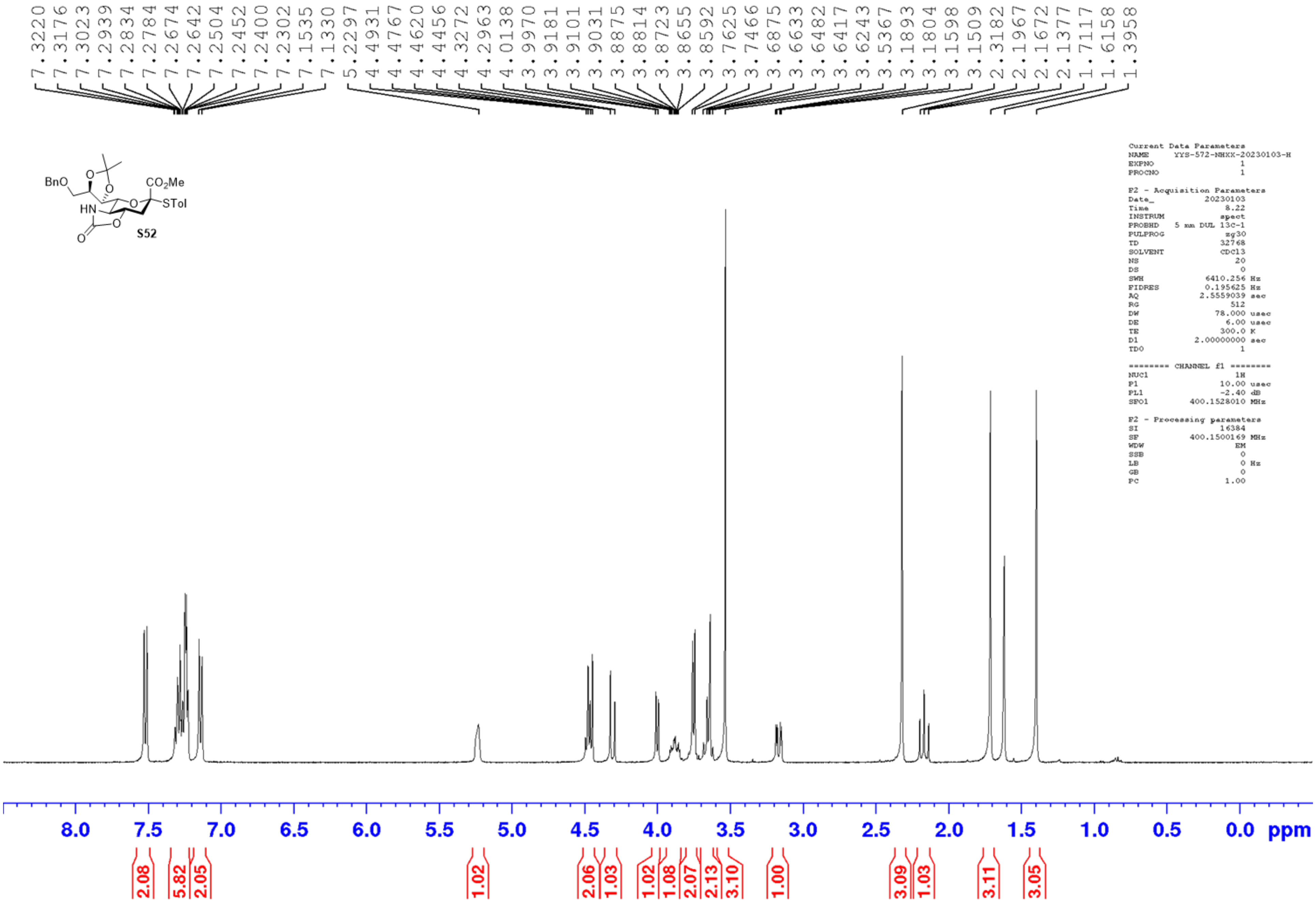

YYS-572-NHXX-20230103-C

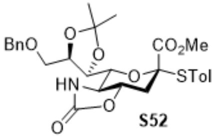

S52

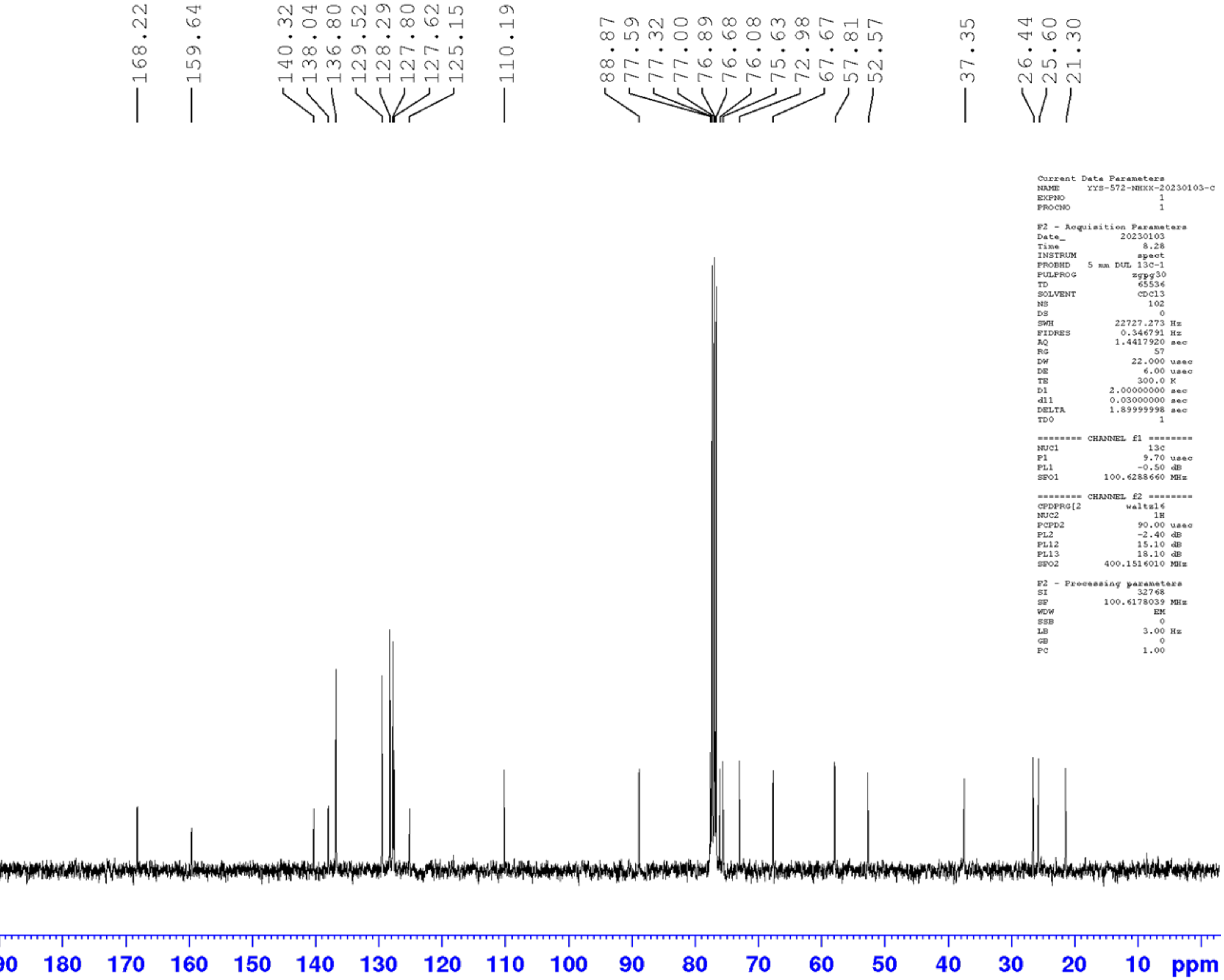

KYT-252-2-20200514-H

Current Data Parameters
NAME KYT-252-2-20200514-H
EXPNO 1
PROCNO 1

F2 - Processing parameters
SI 16384
SF 499.7863547 MHz
WDW EM
SSB 0
LB 0.30 Hz
GB 0
PC 1.00

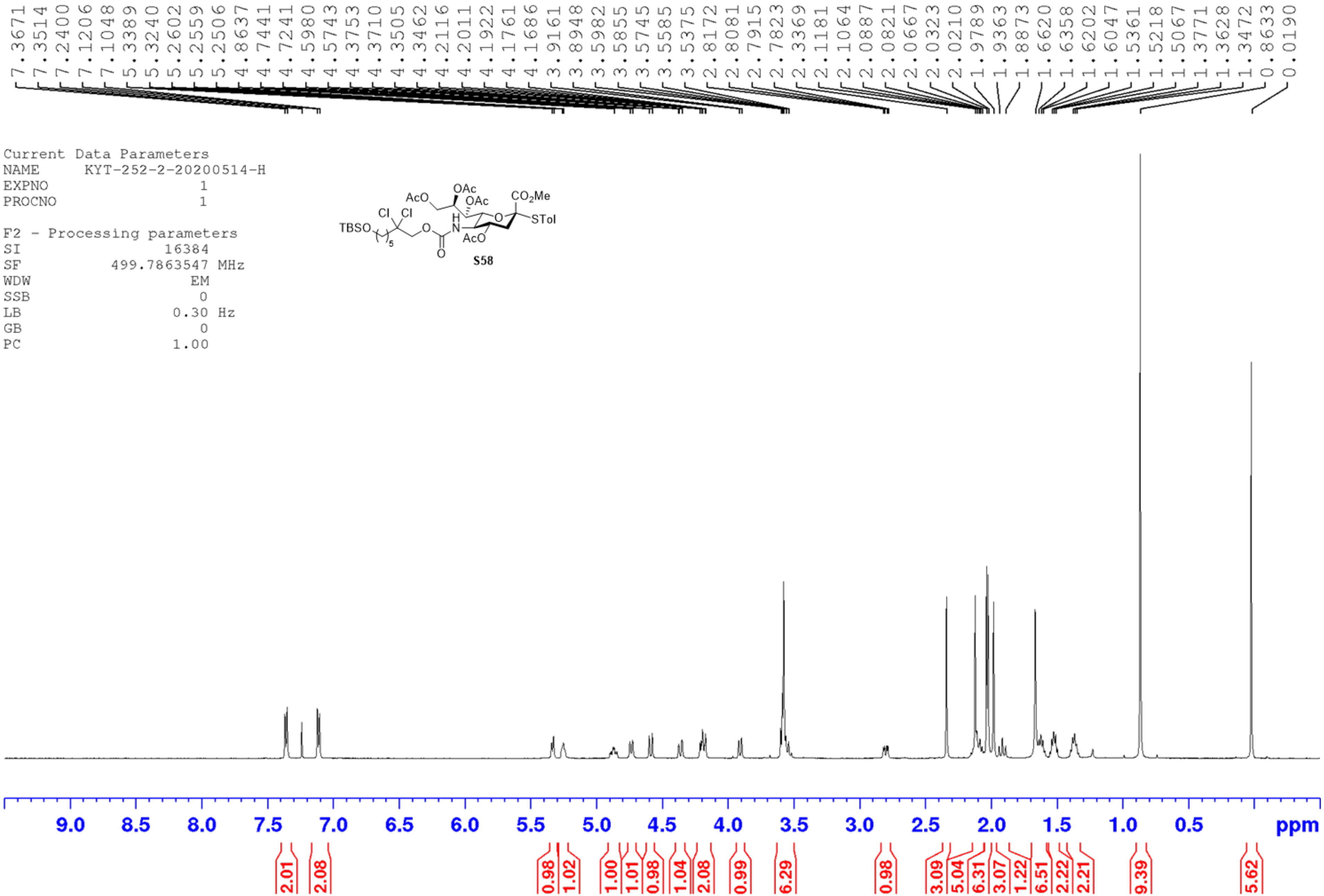

S58

KYT-252-2-20200514-C

Current Data Parameters
NAME KYT-252-2-20200514-C
EXPNO 1
PROCNO 1

F2 - Processing parameters
SI 65536
SF 125.6713833 MHz
WDW EM
SSB 0
LB 3.00 Hz
GB 0
PC 1.00

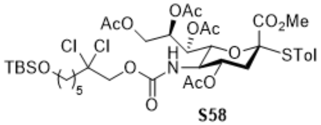

S58

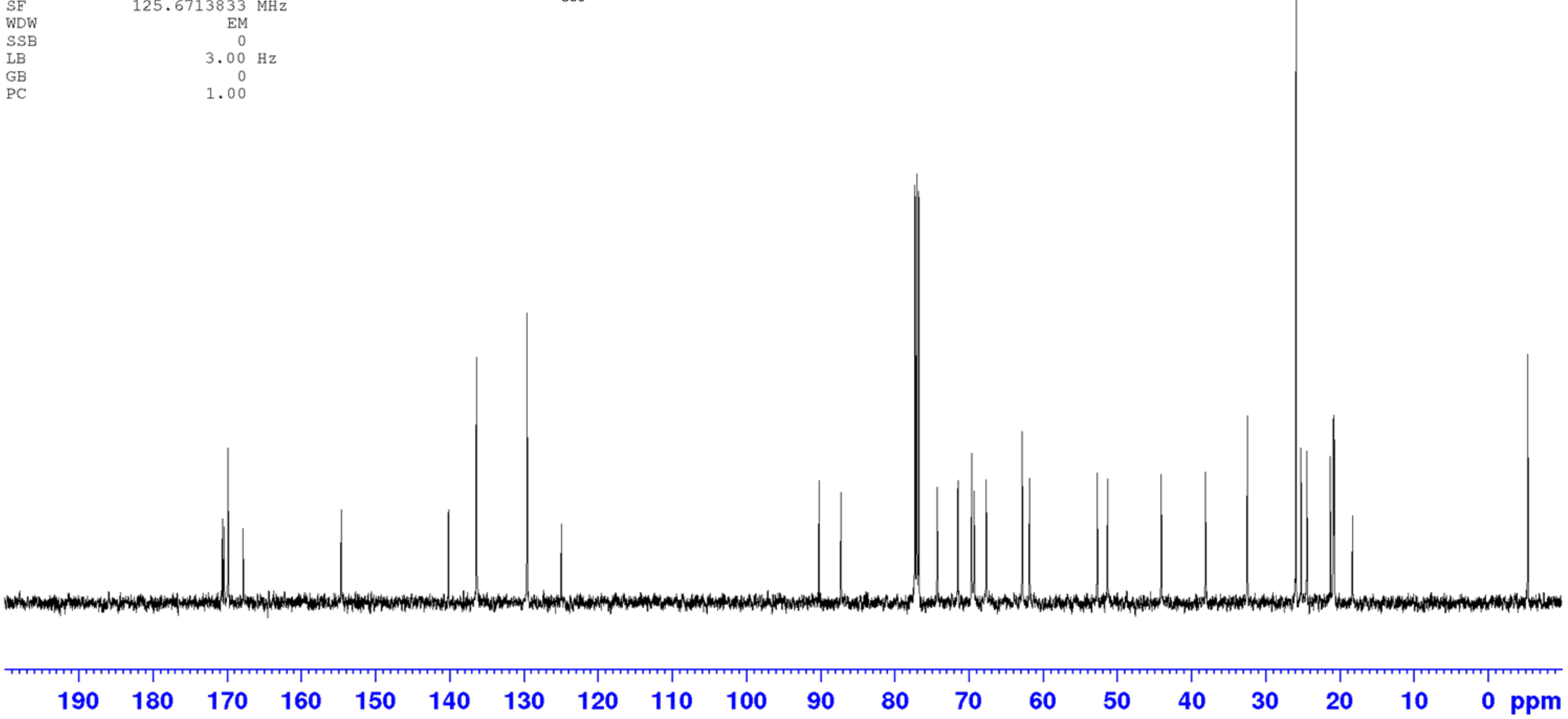

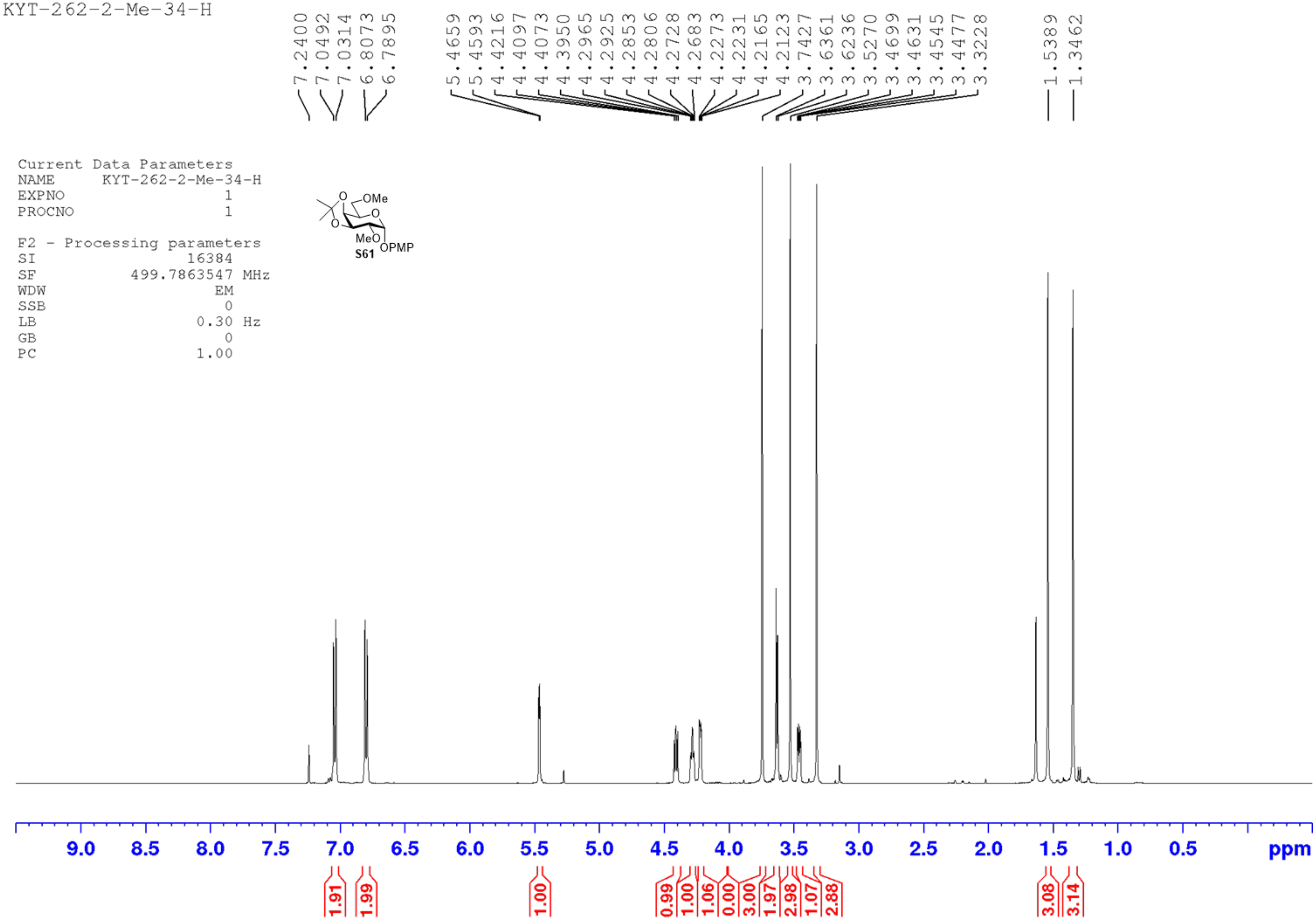
KYT-262-2-Me-34-H
7.2400
7.0492
7.0314
6.8073
6.7895
5.4659
5.4593
4.4216
4.4097
4.4073
4.3950
4.2965
4.2925
4.2853
4.2806
4.2728
4.2683
4.2273
4.2231
4.2165
4.2123
3.7427
3.6361
3.6236
3.5270
3.4699
3.4631
3.4545
3.4477
3.3228
1.5389
1.3462
Current Data Parameters
NAME KYT-262-2-Me-34-H
EXPNO 1
PROCNO 1
F2 - Processing parameters
SI 16384
SF 499.7863547 MHz
WDW EM
SSB 0
LB 0.30 Hz
GB 0
PC 1.00
OMe
O
O
O
MeO
OPMP
S61
9.0
8.5
8.0
7.5
7.0
6.5
6.0
5.5
5.0
4.5
4.0
3.5
3.0
2.5
2.0
1.5
1.0
0.5
ppm
1.91
1.99
1.00
0.99
1.00
1.06
0.00
3.00
1.97
2.98
1.07
2.88
3.08
3.14

KYT-262-2-Me-34-C

Current Data Parameters
NAME KYT-262-2-Me-34-C
EXPNO 1
PROCNO 1

F2 - Processing parameters
SI 65536
SF 125.6713866 MHz
WDW EM
SSB 0
LB 0.30 Hz
GB 0
PC 1.00

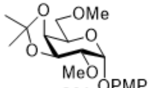

S61

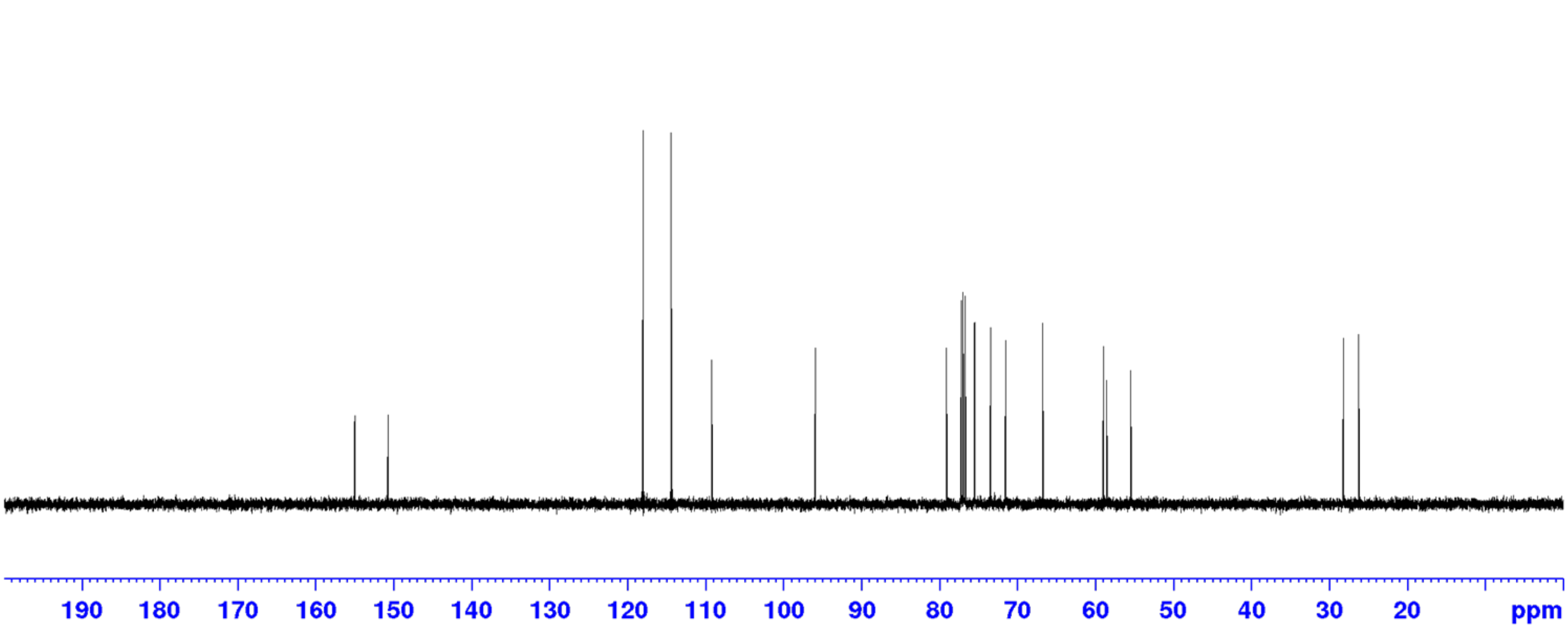

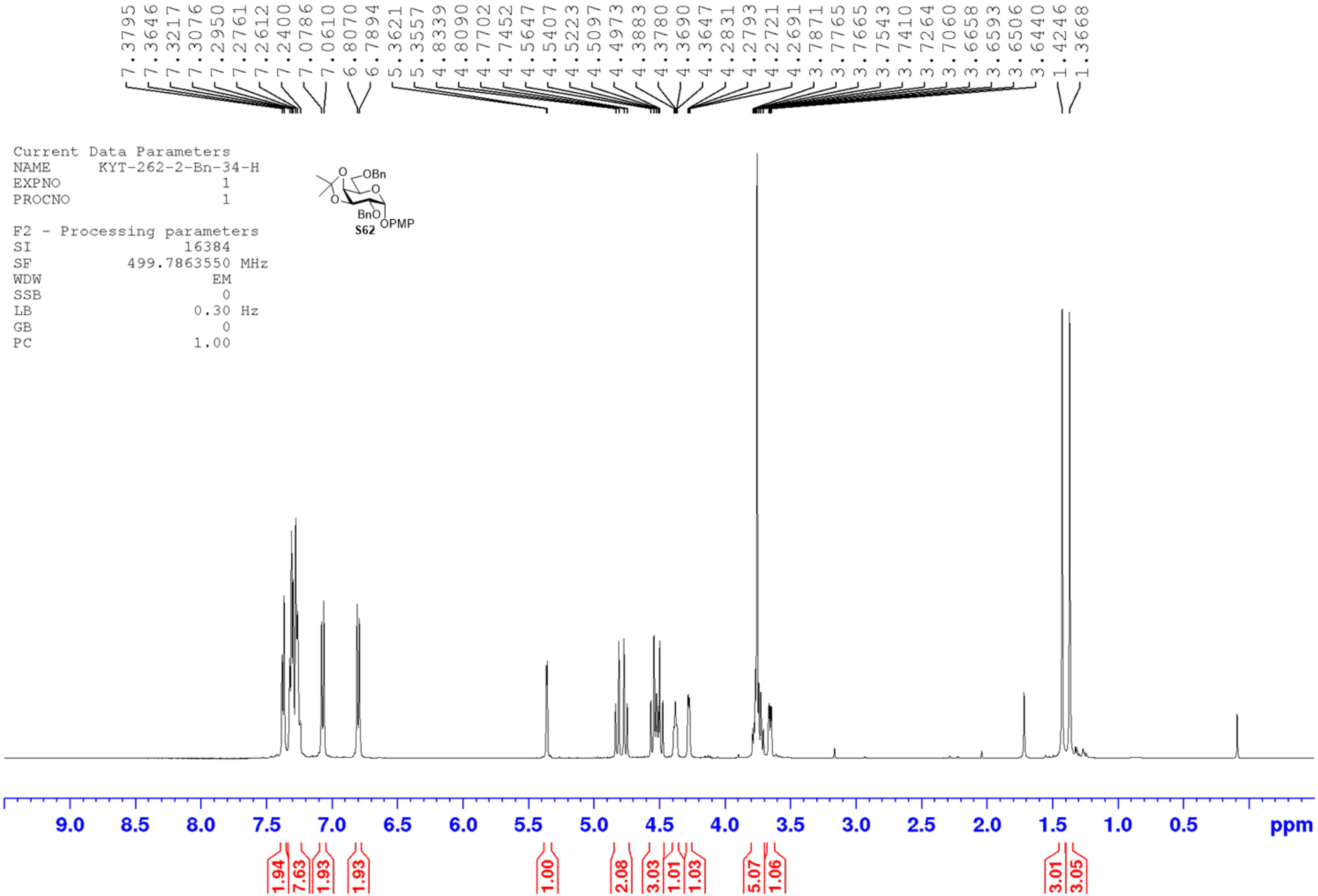
7.3795
7.3646
7.3217
7.3076
7.2950
7.2761
7.2612
7.2400
7.0786
7.0610
6.8070
6.7894
5.3621
5.3557
4.8339
4.8090
4.7702
4.7452
4.5647
4.5407
4.5223
4.5097
4.4973
4.3883
4.3780
4.3690
4.3647
4.2831
4.2793
4.2721
4.2691
3.7871
3.7765
3.7665
3.7543
3.7410
3.7264
3.7060
3.6658
3.6593
3.6506
3.6440
1.4246
1.3668
Current Data Parameters
NAME KYT-262-2-Bn-34-H
EXPNO 1
PROCNO 1
F2 - Processing parameters
SI 16384
SF 499.7863550 MHz
WDW EM
SSB 0
LB 0.30 Hz
GB 0
PC 1.00
OBn
O
O
O
BnO
OPMP
S62
9.0
8.5
8.0
7.5
7.0
6.5
6.0
5.5
5.0
4.5
4.0
3.5
3.0
2.5
2.0
1.5
1.0
0.5
ppm
1.94
7.63
1.93
1.93
1.00
2.08
3.03
1.01
1.03
5.07
1.06
3.01
3.05

155.03
150.83
138.16
137.98
128.29
128.18
127.80
127.67
127.42
127.36
118.21
114.43
109.23
96.53
77.25
77.00
76.75
76.16
75.78
73.57
73.07
72.22
69.31
67.06
55.50
28.04
26.31

Current Data Parameters
NAME KYT-262-2-Bn-34-C.fid
EXPNO 1
PROCNO 1

F2 - Processing parameters
SI 65536
SF 125.6713916 MHz
WDW EM
SSB 0
LB 0.30 Hz
GB 0
PC 1.00

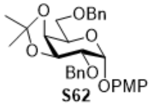

S62

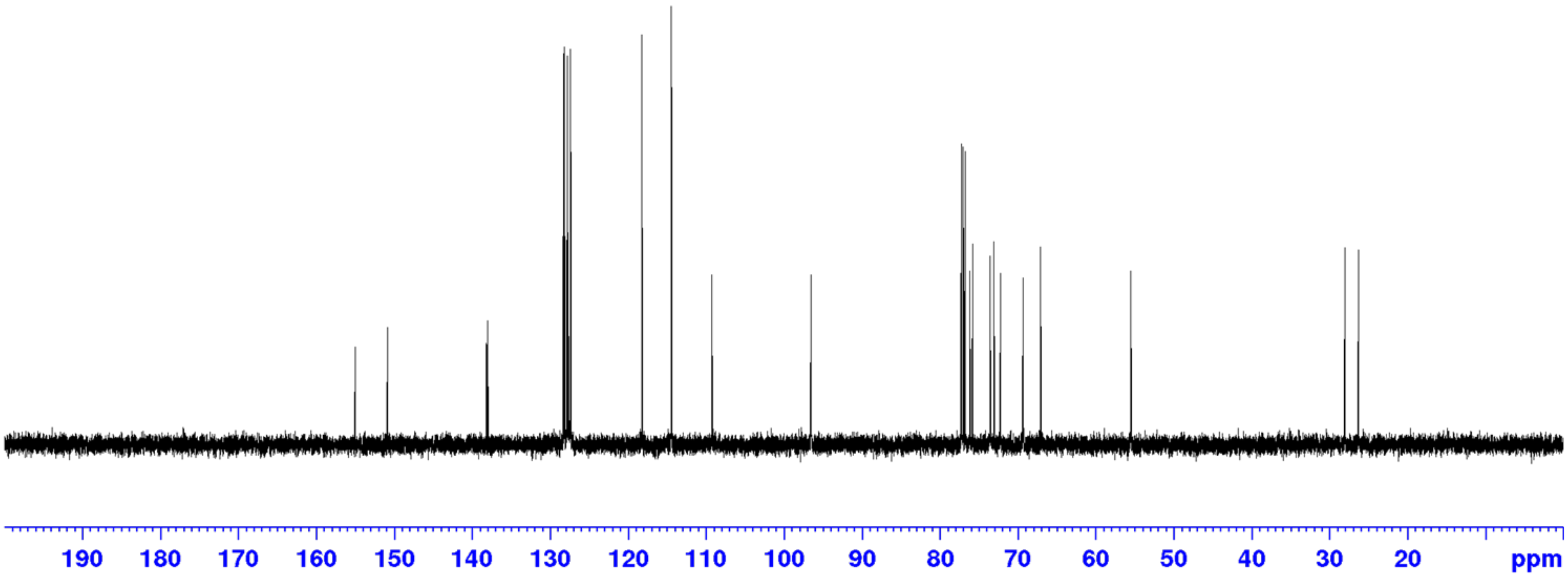

KYT-263-Me-H

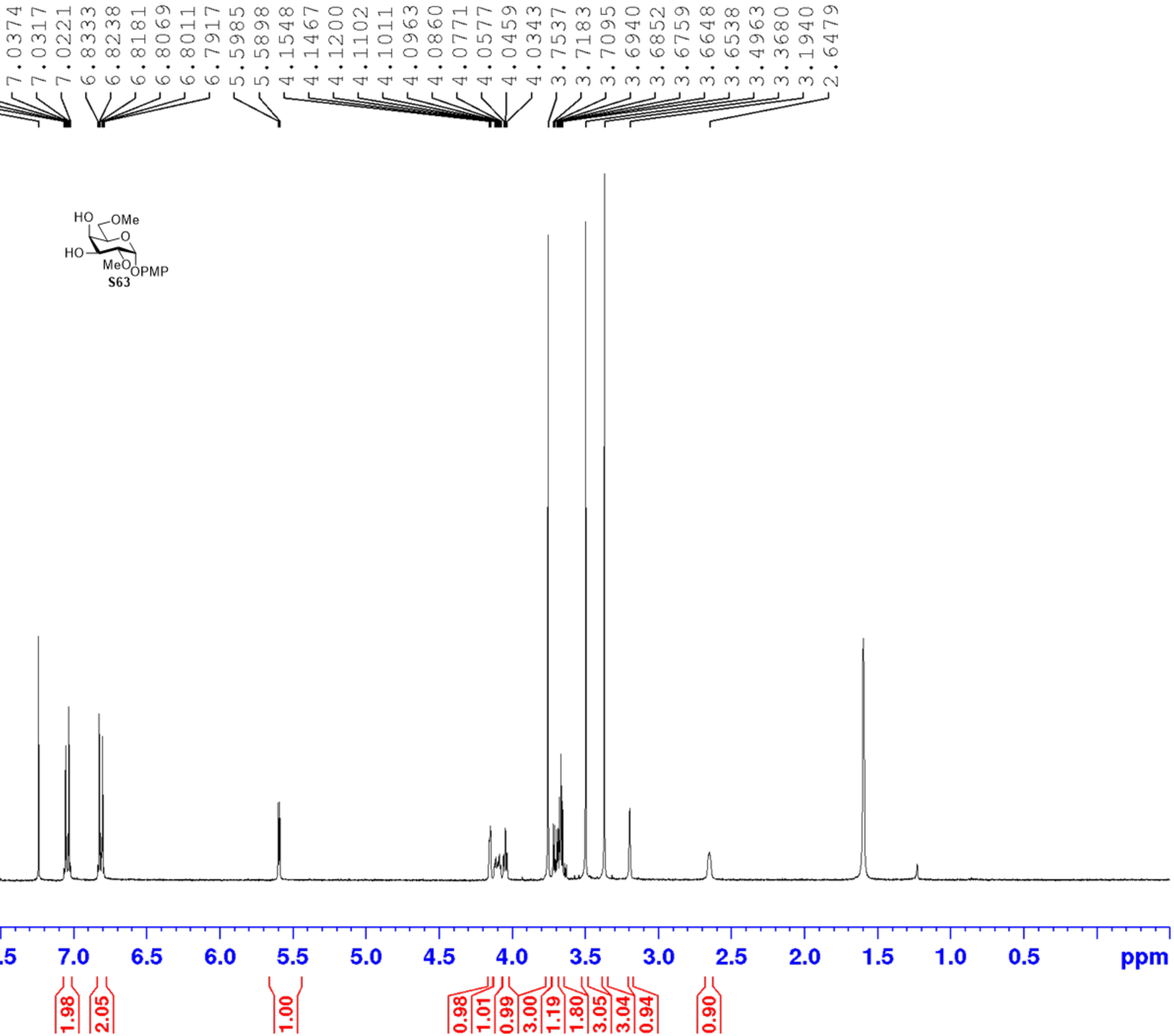

Current Data Parameters
NAME KYT-263-Me-H
EXPNO 1
PROCNO 1

F2 - Acquisition Parameters
Date_ 20200505
Time 23.57
INSTRUM spect
PROBHD 5 mm DUL 13C-1
PULPROG zg30
TD 32768
SOLVENT CDCl3
NS 9
DS 0
SWH 6410.256 Hz
FIDRES 0.195625 Hz
AQ 2.5559039 sec
RG 724
DW 78.000 usec
DE 6.00 usec
TE 300.0 K
D1 2.00000000 sec
TD0 1

======== CHANNEL f1 ========
NUC1 1H
P1 10.00 usec
PL1 -2.40 dB
SFO1 400.1528010 MHz

F2 - Processing parameters
SI 16384
SF 400.1500171 MHz
WDW EM
SSB 0
LB 0 Hz
GB 0
PC 1.00

S63

KYT-263-Me-C

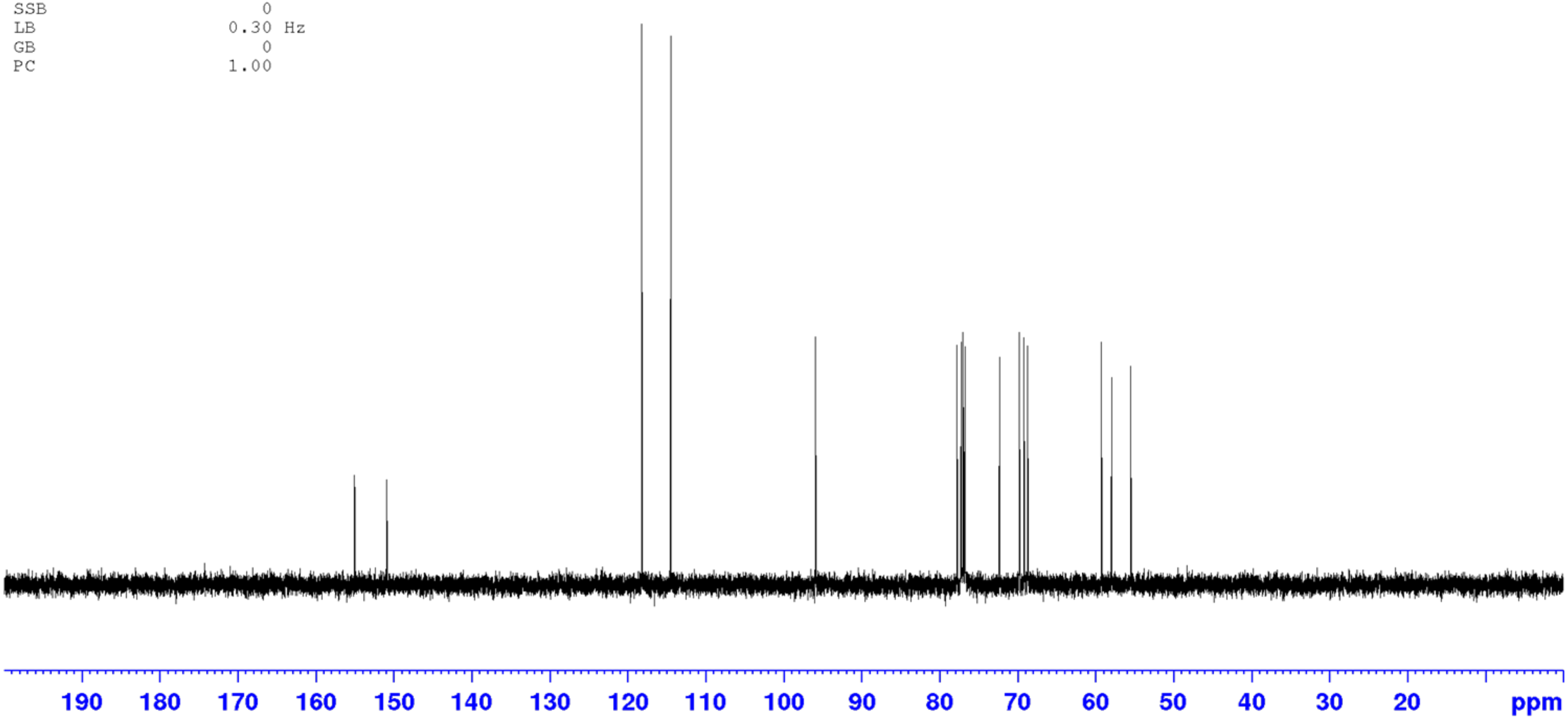

Current Data Parameters
NAME KYT-263-Me-C
EXPNO 1
PROCNO 1

F2 - Processing parameters
SI 65536
SF 125.6713873 MHz
WDW EM
SSB 0
LB 0.30 Hz
GB 0
PC 1.00

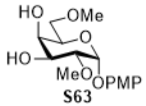

S63

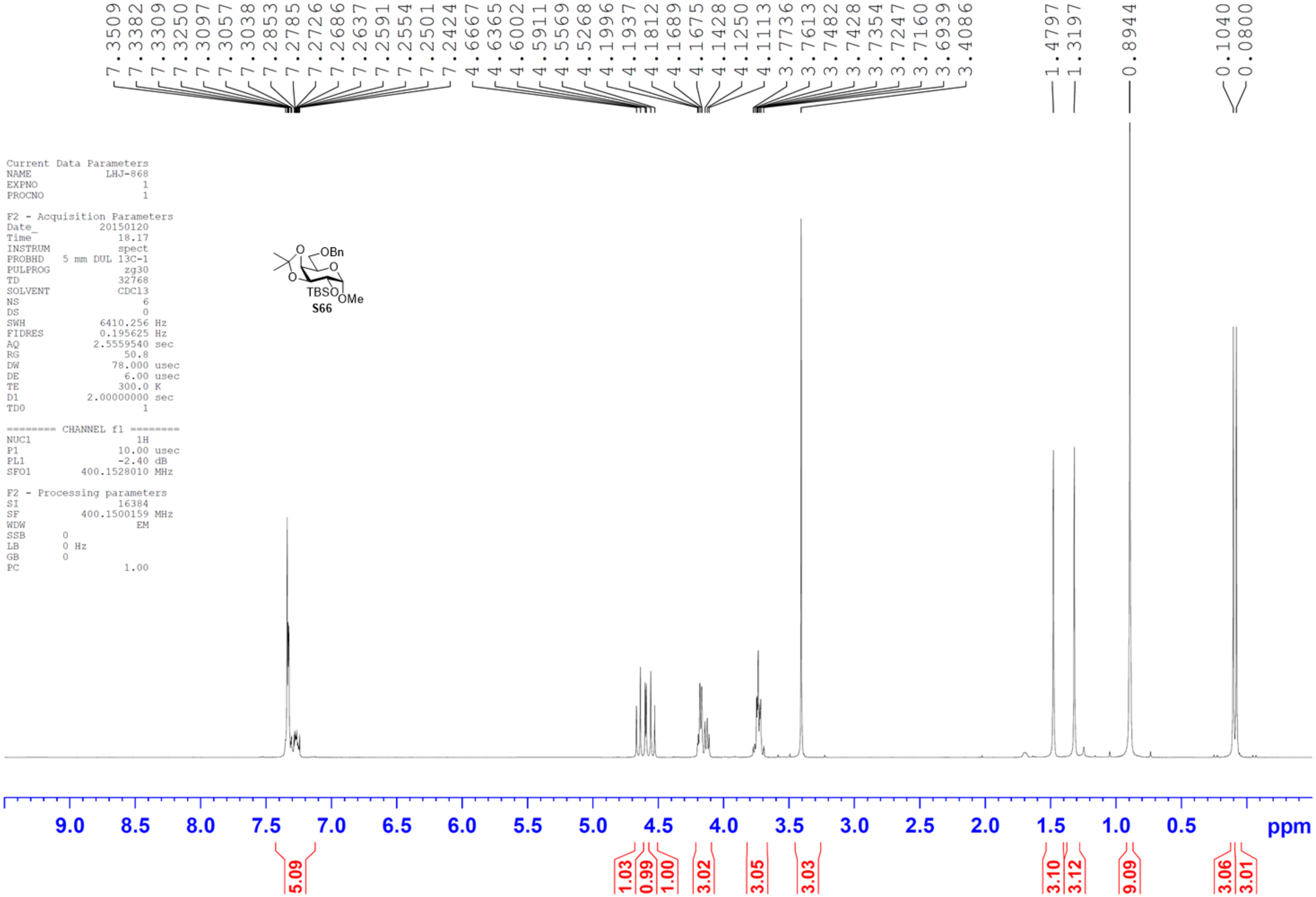
7.3509
7.3382
7.3309
7.3250
7.3097
7.3057
7.3038
7.2853
7.2785
7.2726
7.2686
7.2637
7.2591
7.2554
7.2501
7.2424
4.6667
4.6365
4.6002
4.5911
4.5569
4.5268
4.1996
4.1937
4.1812
4.1689
4.1675
4.1428
4.1250
4.1113
3.7736
3.7613
3.7482
3.7428
3.7354
3.7247
3.7160
3.6939
3.4086
1.4797
1.3197
0.8944
0.1040
0.0800
Current Data Parameters
NAME LHJ-868
EXPNO 1
PROCNO 1
F2 - Acquisition Parameters
Date_ 20150120
Time 18.17
INSTRUM spect
PROBHD 5 mm DUL 13C-1
PULPROG zg30
TD 32768
SOLVENT CDCl3
NS 6
DS 0
SWH 6410.256 Hz
FIDRES 0.195625 Hz
AQ 2.5559540 sec
RG 50.8
DW 78.000 usec
DE 6.00 usec
TE 300.0 K
D1 2.00000000 sec
TD0 1
======== CHANNEL f1 ========
NUC1 1H
P1 10.00 usec
PL1 -2.40 dB
SFO1 400.1528010 MHz
F2 - Processing parameters
SI 16384
SF 400.1500159 MHz
WDW EM
SSB 0
LB 0 Hz
GB 0
PC 1.00
OBn
O
O
O
TBSO
OMe
S66
9.0
8.5
8.0
7.5
7.0
6.5
6.0
5.5
5.0
4.5
4.0
3.5
3.0
2.5
2.0
1.5
1.0
0.5
ppm
5.09
1.03
0.99
1.00
3.02
3.05
3.03
3.10
3.12
9.09
3.06
3.01

```
Current Data Parameters
NAME             LHJ-868
EXPNO                  3
PROCNO                 1

F2 - Acquisition Parameters
Date_           20150120
Time               17.05
INSTRUM            spect
PROBHD   5 mm DUL 13C-1
PULPROG          zgpg30
TD                 65536
SOLVENT            CDCl3
NS                    43
DS                     0
SWH            22727.273 Hz
FIDRES          0.346791 Hz
AQ             1.4418420 sec
RG                    57
DW                22.000 usec
DE                  6.00 usec
TE                 300.0 K
D1            2.00000000 sec
d11           0.03000000 sec
DELTA         1.89999998 sec
TD0                    1

======== CHANNEL f1 ========
NUC1                 13C
P1                  9.70 usec
PL1                -0.50 dB
SFO1         100.6278598 MHz

======== CHANNEL f2 ========
CPDPRG2          waltz16
NUC2                  1H
PCPD2              90.00 usec
PL2                -2.40 dB
PL12               15.10 dB
PL13               18.10 dB
SFO2         400.1516010 MHz

F2 - Processing parameters
SI                 32768
SF           100.6178069 MHz
WDW                   EM
SSB      0
LB                  3.00 Hz
GB       0
PC                  1.00
```

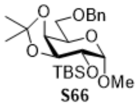

S66

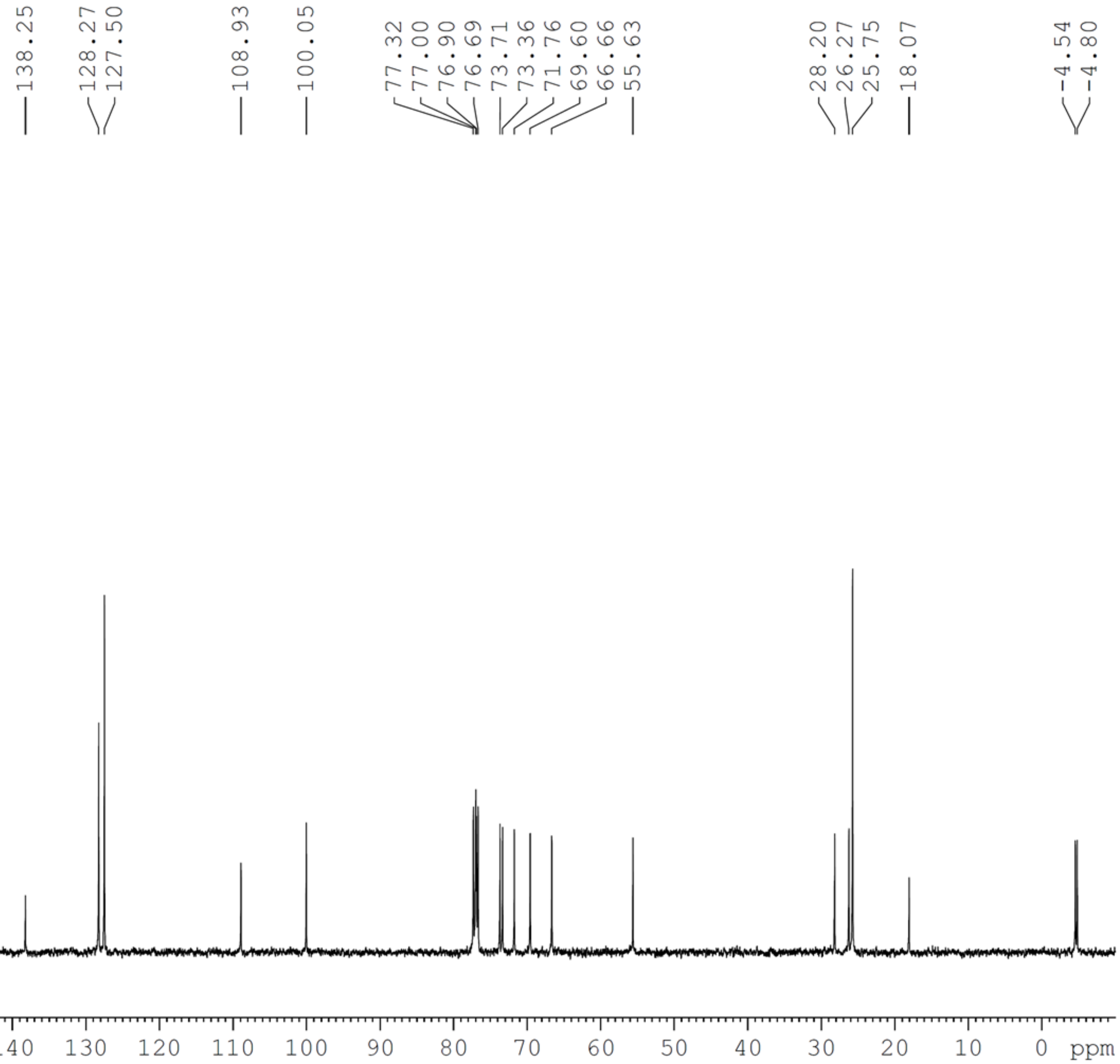

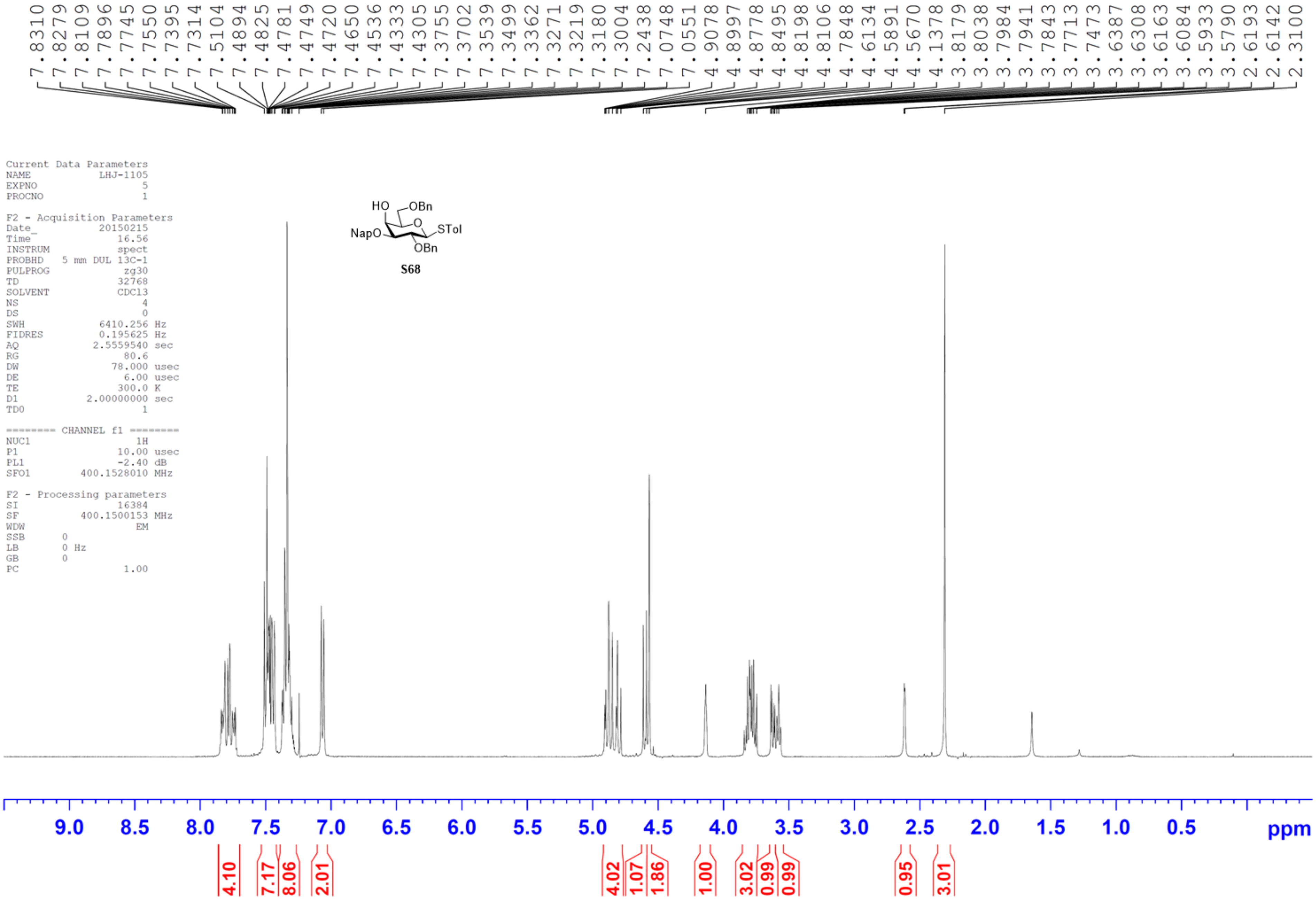
7.8310 7.8279 7.8109 7.7896 7.7745 7.7550 7.7395 7.7314 7.5104 7.4894 7.4825 7.4781 7.4749 7.4720 7.4650 7.4536 7.4333 7.4305 7.3755 7.3702 7.3539 7.3499 7.3362 7.3271 7.3219 7.3180 7.3004 7.2438 7.0748 7.0551 4.9078 4.8997 4.8778 4.8495 4.8198 4.8106 4.7848 4.6134 4.5891 4.5670 4.1378 3.8179 3.8038 3.7984 3.7941 3.7843 3.7713 3.7473 3.6387 3.6308 3.6163 3.6084 3.5933 3.5790 2.6193 2.6142 2.3100
Current Data Parameters
NAME LHJ-1105
EXPNO 5
PROCNO 1
F2 - Acquisition Parameters
Date_ 20150215
Time 16.56
INSTRUM spect
PROBHD 5 mm DUL 13C-1
PULPROG zg30
TD 32768
SOLVENT CDCl3
NS 4
DS 0
SWH 6410.256 Hz
FIDRES 0.195625 Hz
AQ 2.5559540 sec
RG 80.6
DW 78.000 usec
DE 6.00 usec
TE 300.0 K
D1 2.00000000 sec
TD0 1
======== CHANNEL f1 ========
NUC1 1H
P1 10.00 usec
PL1 -2.40 dB
SFO1 400.1528010 MHz
F2 - Processing parameters
SI 16384
SF 400.1500153 MHz
WDW EM
SSB 0
LB 0 Hz
GB 0
PC 1.00
HO
OBn
O
NapO
STol
OBn
S68
9.0 8.5 8.0 7.5 7.0 6.5 6.0 5.5 5.0 4.5 4.0 3.5 3.0 2.5 2.0 1.5 1.0 0.5 ppm
4.10 7.17 8.06 2.01 4.02 1.07 1.86 1.00 3.02 0.99 0.99 0.95 3.01

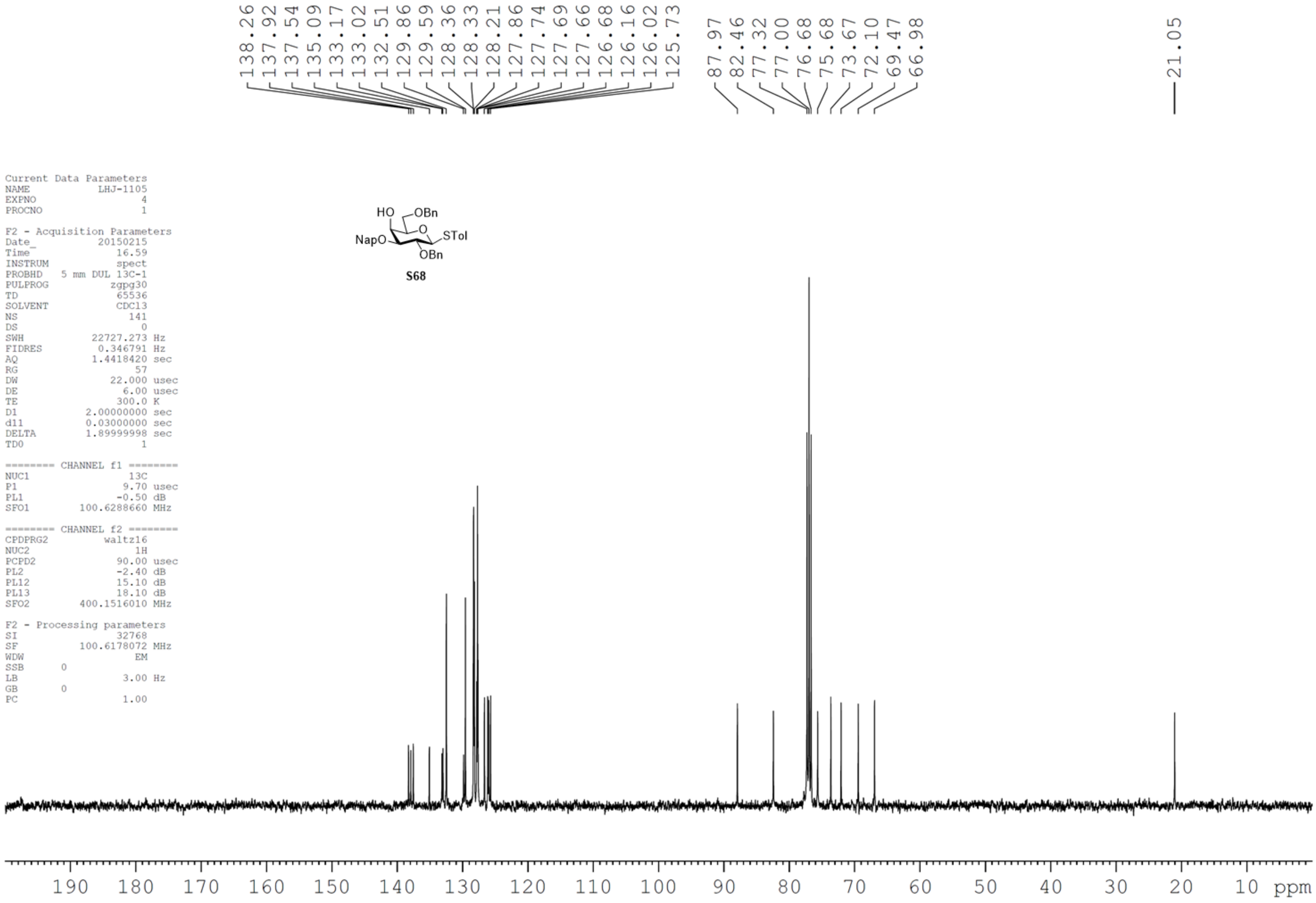
138.26
137.92
137.54
135.09
133.17
133.02
132.51
129.86
129.59
128.36
128.33
128.21
127.86
127.74
127.69
127.66
126.68
126.16
126.02
125.73
87.97
82.46
77.32
77.00
76.68
75.68
73.67
72.10
69.47
66.98
21.05
Current Data Parameters
NAME LHJ-1105
EXPNO 4
PROCNO 1
F2 - Acquisition Parameters
Date_ 20150215
Time 16.59
INSTRUM spect
PROBHD 5 mm DUL 13C-1
PULPROG zgpg30
TD 65536
SOLVENT CDCl3
NS 141
DS 0
SWH 22727.273 Hz
FIDRES 0.346791 Hz
AQ 1.4418420 sec
RG 57
DW 22.000 usec
DE 6.00 usec
TE 300.0 K
D1 2.00000000 sec
d11 0.03000000 sec
DELTA 1.89999998 sec
TD0 1
======== CHANNEL f1 ========
NUC1 13C
P1 9.70 usec
PL1 -0.50 dB
SFO1 100.6288660 MHz
======== CHANNEL f2 ========
CPDPRG2 waltz16
NUC2 1H
PCPD2 90.00 usec
PL2 -2.40 dB
PL12 15.10 dB
PL13 18.10 dB
SFO2 400.1516010 MHz
F2 - Processing parameters
SI 32768
SF 100.6178072 MHz
WDW EM
SSB 0
LB 3.00 Hz
GB 0
PC 1.00
HO
OBn
NapO
O
STol
OBn
S68
190 180 170 160 150 140 130 120 110 100 90 80 70 60 50 40 30 20 10 ppm

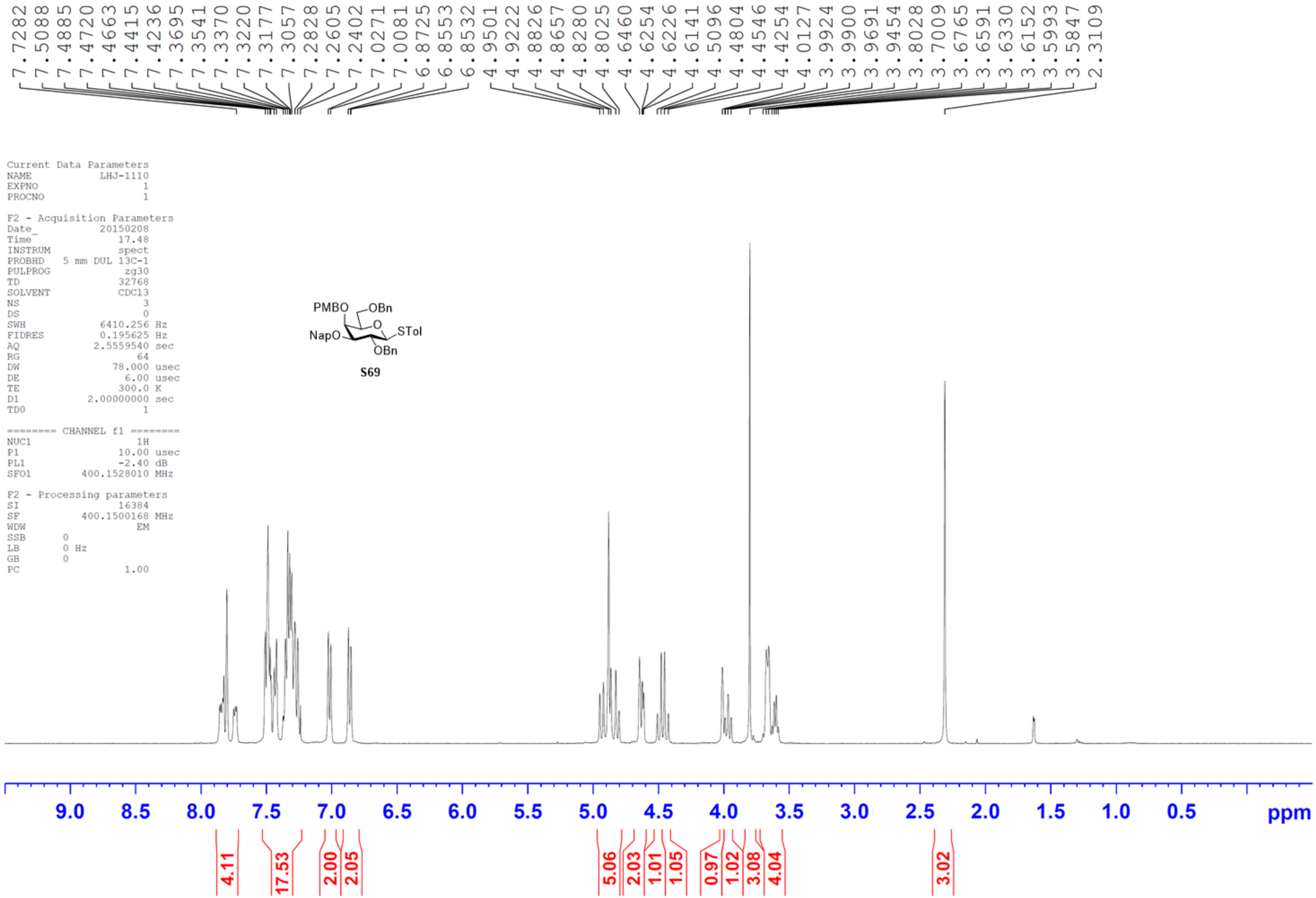
7.7282
7.5088
7.4885
7.4720
7.4663
7.4415
7.4236
7.3695
7.3541
7.3370
7.3220
7.3177
7.3057
7.2828
7.2605
7.2402
7.0271
7.0081
6.8725
6.8553
6.8532
4.9501
4.9222
4.8826
4.8657
4.8280
4.8025
4.6460
4.6254
4.6226
4.6141
4.5096
4.4804
4.4546
4.4254
4.0127
3.9924
3.9900
3.9691
3.9454
3.8028
3.7009
3.6765
3.6591
3.6330
3.6152
3.5993
3.5847
2.3109
Current Data Parameters
NAME LHJ-1110
EXPNO 1
PROCNO 1
F2 - Acquisition Parameters
Date_ 20150208
Time 17.48
INSTRUM spect
PROBHD 5 mm DUL 13C-1
PULPROG zg30
TD 32768
SOLVENT CDCl3
NS 3
DS 0
SWH 6410.256 Hz
FIDRES 0.195625 Hz
AQ 2.5559540 sec
RG 64
DW 78.000 usec
DE 6.00 usec
TE 300.0 K
D1 2.00000000 sec
TD0 1
======== CHANNEL f1 ========
NUC1 1H
P1 10.00 usec
PL1 -2.40 dB
SFO1 400.1528010 MHz
F2 - Processing parameters
SI 16384
SF 400.1500168 MHz
WDW EM
SSB 0
LB 0 Hz
GB 0
PC 1.00
PMBO
OBn
NapO
O
STol
OBn
S69
9.0
8.5
8.0
7.5
7.0
6.5
6.0
5.5
5.0
4.5
4.0
3.5
3.0
2.5
2.0
1.5
1.0
0.5
ppm
4.11
17.53
2.00
2.05
5.06
2.03
1.01
1.05
0.97
1.02
3.08
4.04
3.02

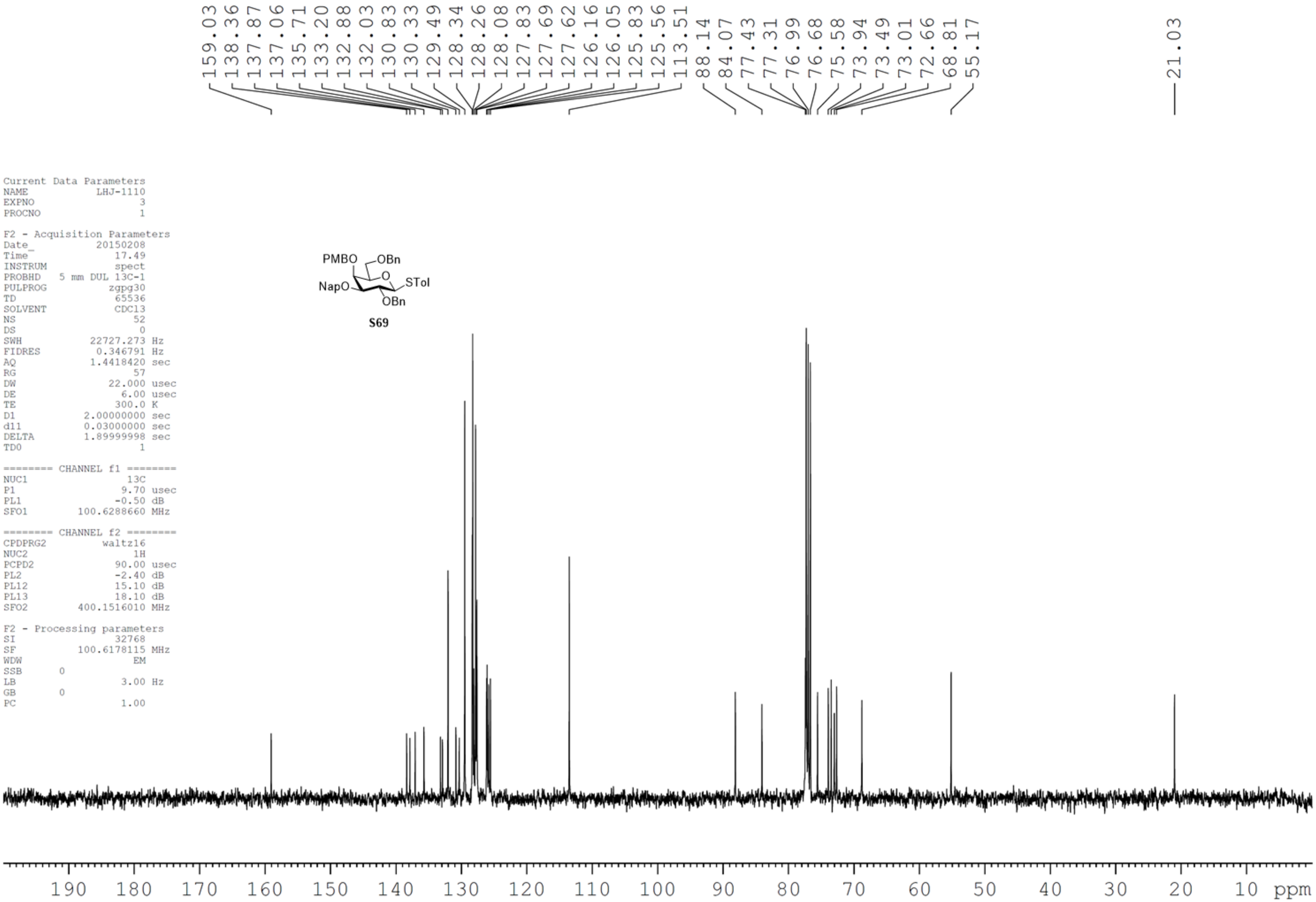

159.03
138.36
137.87
137.06
135.71
133.20
132.88
132.03
130.83
130.33
129.49
128.34
128.26
128.08
127.83
127.69
127.62
126.16
126.05
125.83
125.56
113.51
88.14
84.07
77.43
77.31
76.99
76.68
75.58
73.94
73.49
73.01
72.66
68.81
55.17
21.03
Current Data Parameters
NAME LHJ-1110
EXPNO 3
PROCNO 1
F2 - Acquisition Parameters
Date_ 20150208
Time 17.49
INSTRUM spect
PROBHD 5 mm DUL 13C-1
PULPROG zgpg30
TD 65536
SOLVENT CDCl3
NS 52
DS 0
SWH 22727.273 Hz
FIDRES 0.346791 Hz
AQ 1.4418420 sec
RG 57
DW 22.000 usec
DE 6.00 usec
TE 300.0 K
D1 2.00000000 sec
d11 0.03000000 sec
DELTA 1.89999998 sec
TD0 1
======== CHANNEL f1 ========
NUC1 13C
P1 9.70 usec
PL1 -0.50 dB
SFO1 100.6288660 MHz
======== CHANNEL f2 ========
CPDPRG2 waltz16
NUC2 1H
PCPD2 90.00 usec
PL2 -2.40 dB
PL12 15.10 dB
PL13 18.10 dB
SFO2 400.1516010 MHz
F2 - Processing parameters
SI 32768
SF 100.6178115 MHz
WDW EM
SSB 0
LB 3.00 Hz
GB 0
PC 1.00
PMBO
OBn
O
NapO
STol
OBn
S69
190
180
170
160
150
140
130
120
110
100
90
80
70
60
50
40
30
20
10
ppm

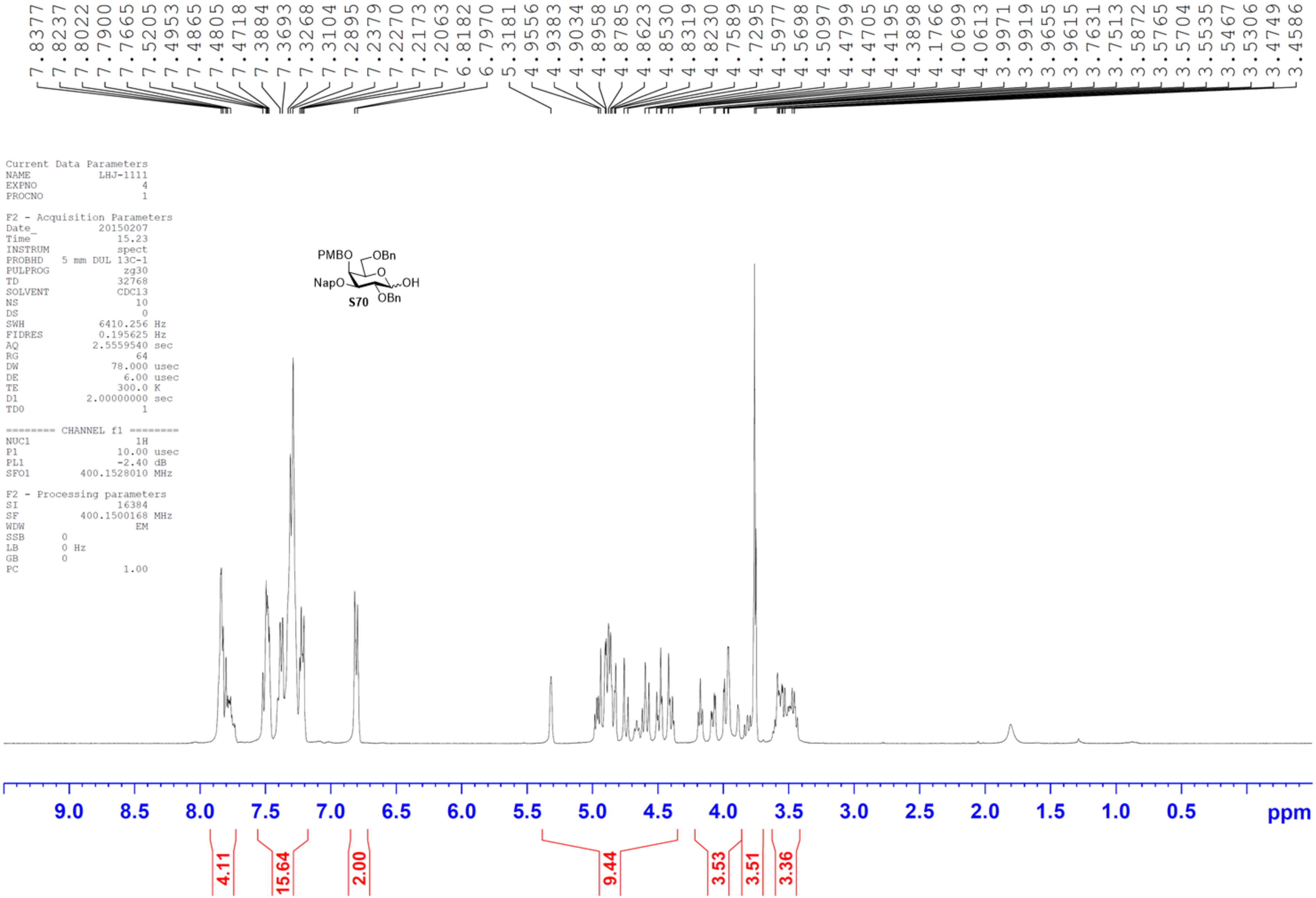
7.8377
7.8237
7.8022
7.7900
7.7665
7.5205
7.4953
7.4865
7.4805
7.4718
7.3884
7.3693
7.3268
7.3104
7.2895
7.2379
7.2270
7.2173
7.2063
6.8182
6.7970
5.3181
4.9556
4.9383
4.9034
4.8958
4.8785
4.8623
4.8530
4.8319
4.8230
4.7589
4.7295
4.5977
4.5698
4.5097
4.4799
4.4705
4.4195
4.3898
4.1766
4.0699
4.0613
3.9971
3.9919
3.9655
3.9615
3.7631
3.7513
3.5872
3.5765
3.5704
3.5535
3.5467
3.5306
3.4749
3.4586
Current Data Parameters
NAME LHJ-1111
EXPNO 4
PROCNO 1
F2 - Acquisition Parameters
Date_ 20150207
Time 15.23
INSTRUM spect
PROBHD 5 mm DUL 13C-1
PULPROG zg30
TD 32768
SOLVENT CDCl3
NS 10
DS 0
SWH 6410.256 Hz
FIDRES 0.195625 Hz
AQ 2.5559540 sec
RG 64
DW 78.000 usec
DE 6.00 usec
TE 300.0 K
D1 2.00000000 sec
TD0 1
======== CHANNEL f1 ========
NUC1 1H
P1 10.00 usec
PL1 -2.40 dB
SFO1 400.1528010 MHz
F2 - Processing parameters
SI 16384
SF 400.1500168 MHz
WDW EM
SSB 0
LB 0 Hz
GB 0
PC 1.00
PMBO
OBn
O
NapO
OH
OBn
S70
9.0
8.5
8.0
7.5
7.0
6.5
6.0
5.5
5.0
4.5
4.0
3.5
3.0
2.5
2.0
1.5
1.0
0.5
ppm
4.11
15.64
2.00
9.44
3.53
3.51
3.36

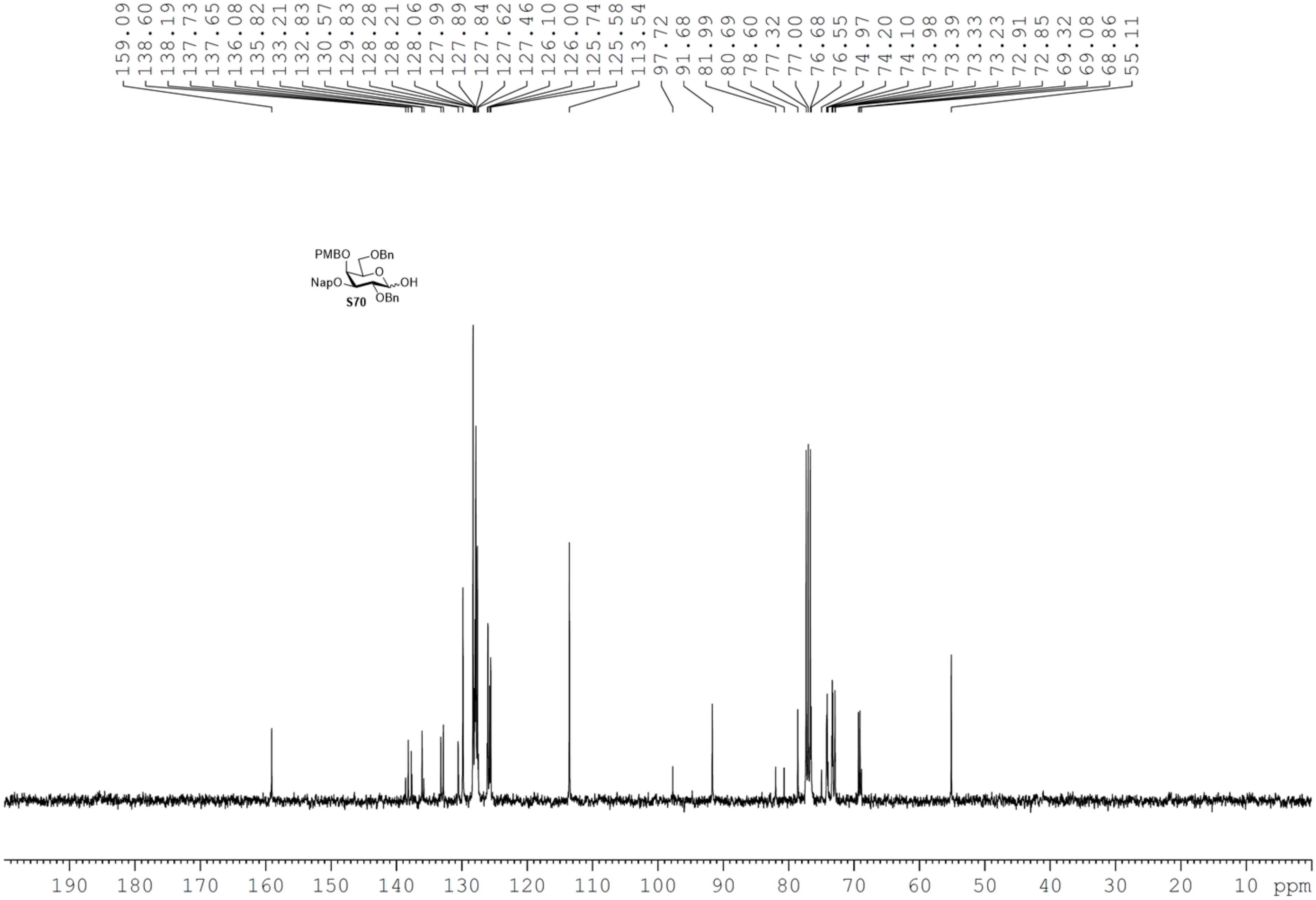
159.09
138.60
138.19
137.73
137.65
136.08
135.82
133.21
132.83
130.57
129.83
128.28
128.21
128.06
127.99
127.89
127.84
127.62
127.46
126.10
126.00
125.74
125.58
113.54
97.72
91.68
81.99
80.69
78.60
77.32
77.00
76.68
76.55
74.97
74.20
74.10
73.98
73.39
73.33
73.23
72.91
72.85
69.32
69.08
68.86
55.11
PMBO
OBn
O
NapO
OH
OBn
S70
190 180 170 160 150 140 130 120 110 100 90 80 70 60 50 40 30 20 10 ppm

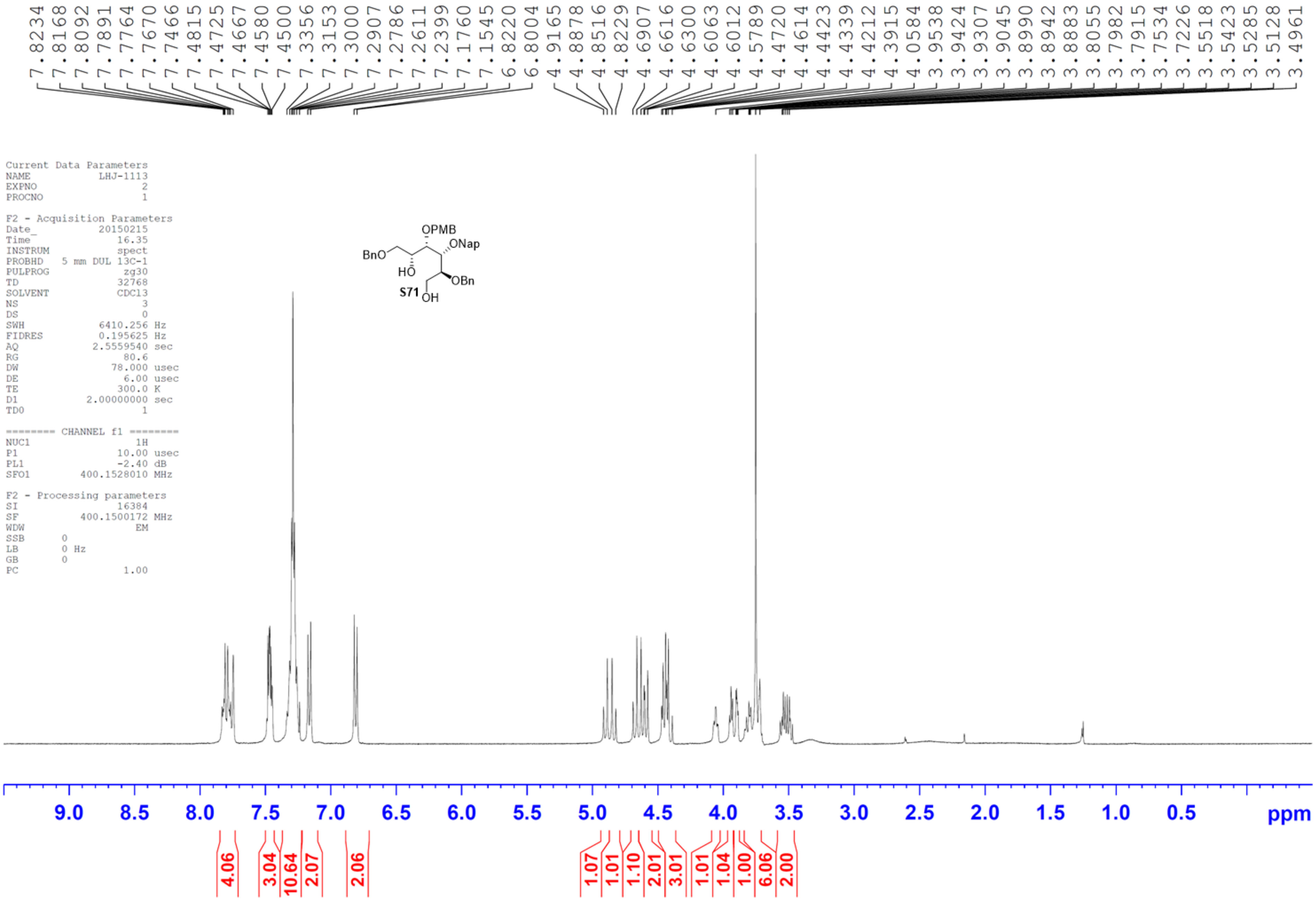
7.8234
7.8168
7.8092
7.7891
7.7764
7.7670
7.7466
7.4815
7.4725
7.4667
7.4580
7.4500
7.3356
7.3153
7.3000
7.2907
7.2786
7.2611
7.2399
7.1760
7.1545
6.8220
6.8004
4.9165
4.8878
4.8516
4.8229
4.6907
4.6616
4.6300
4.6063
4.6012
4.5789
4.4720
4.4614
4.4423
4.4339
4.4212
4.3915
4.0584
3.9538
3.9424
3.9307
3.9045
3.8990
3.8942
3.8883
3.8055
3.7982
3.7915
3.7534
3.7226
3.5518
3.5423
3.5285
3.5128
3.4961
Current Data Parameters
NAME LHJ-1113
EXPNO 2
PROCNO 1
F2 - Acquisition Parameters
Date_ 20150215
Time 16.35
INSTRUM spect
PROBHD 5 mm DUL 13C-1
PULPROG zg30
TD 32768
SOLVENT CDCl3
NS 3
DS 0
SWH 6410.256 Hz
FIDRES 0.195625 Hz
AQ 2.5559540 sec
RG 80.6
DW 78.000 usec
DE 6.00 usec
TE 300.0 K
D1 2.00000000 sec
TD0 1
======== CHANNEL f1 ========
NUC1 1H
P1 10.00 usec
PL1 -2.40 dB
SFO1 400.1528010 MHz
F2 - Processing parameters
SI 16384
SF 400.1500172 MHz
WDW EM
SSB 0
LB 0 Hz
GB 0
PC 1.00
OPMB
ONap
BnO
HO
OBn
S71
OH
9.0
8.5
8.0
7.5
7.0
6.5
6.0
5.5
5.0
4.5
4.0
3.5
3.0
2.5
2.0
1.5
1.0
0.5
ppm
4.06
3.04
10.64
2.07
2.06
1.07
1.01
1.10
2.01
3.01
1.01
1.04
1.00
6.06
2.00

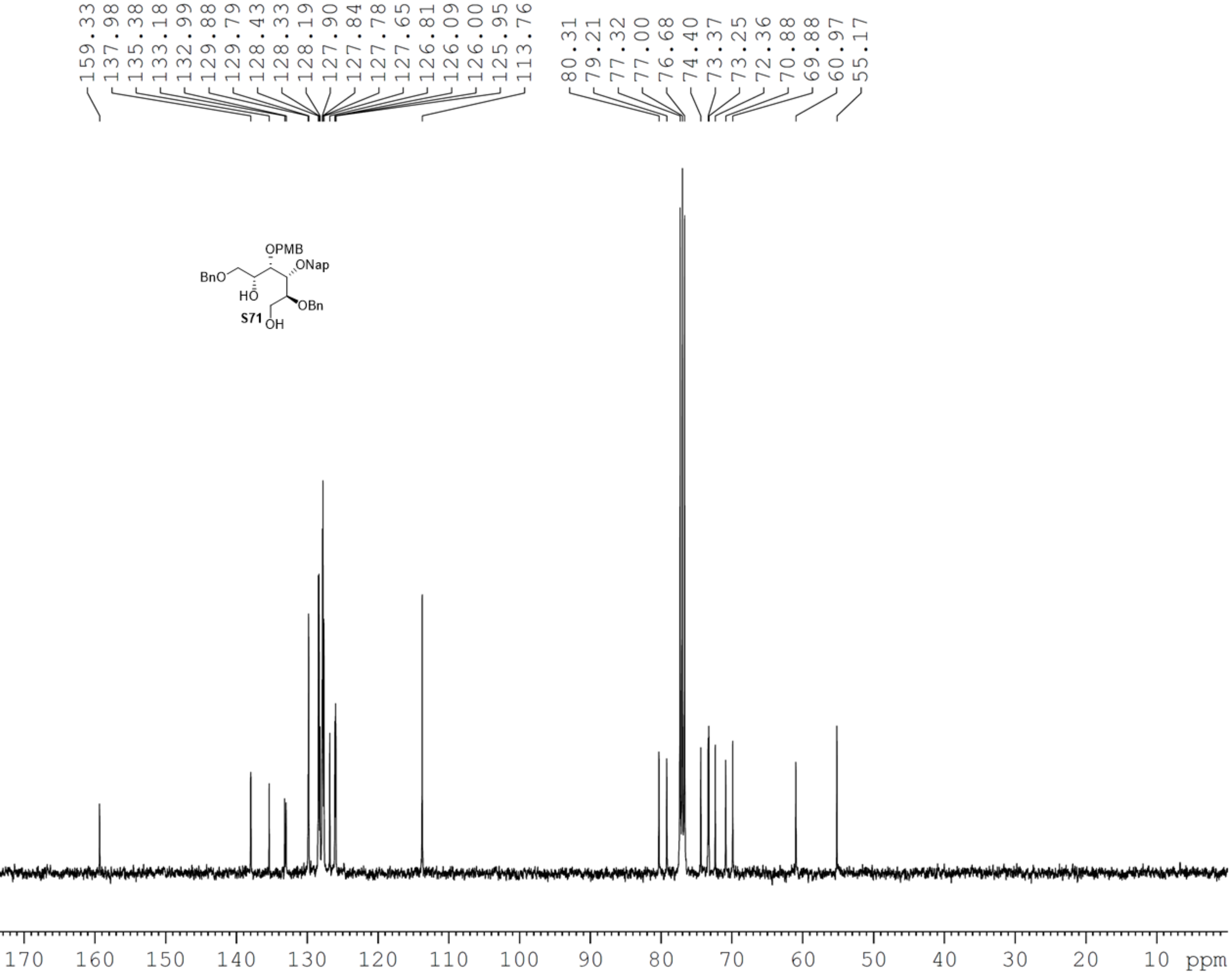


```
Current Data Parameters
NAME          LHJ-1113
EXPNO                3
PROCNO               1

F2 - Acquisition Parameters
Date_         20150215
Time             16.36
INSTRUM          spect
PROBHD   5 mm DUL 13C-1
PULPROG         zgpg30
TD               65536
SOLVENT          CDCl3
NS                 226
DS                   0
SWH          22727.273 Hz
FIDRES        0.346791 Hz
AQ           1.4418420 sec
RG                  57
DW              22.000 usec
DE                6.00 usec
TE               300.0 K
D1          2.00000000 sec
d11         0.03000000 sec
DELTA       1.89999998 sec
TD0                  1

======== CHANNEL f1 ========
NUC1               13C
P1                9.70 usec
PL1              -0.50 dB
SFO1       100.6288660 MHz

======== CHANNEL f2 ========
CPDPRG2        waltz16
NUC2                1H
PCPD2            90.00 usec
PL2              -2.40 dB
PL12             15.10 dB
PL13             18.10 dB
SFO2       400.1516010 MHz

F2 - Processing parameters
SI               32768
SF         100.6178061 MHz
WDW                 EM
SSB      0
LB                3.00 Hz
GB       0
PC                1.00
```

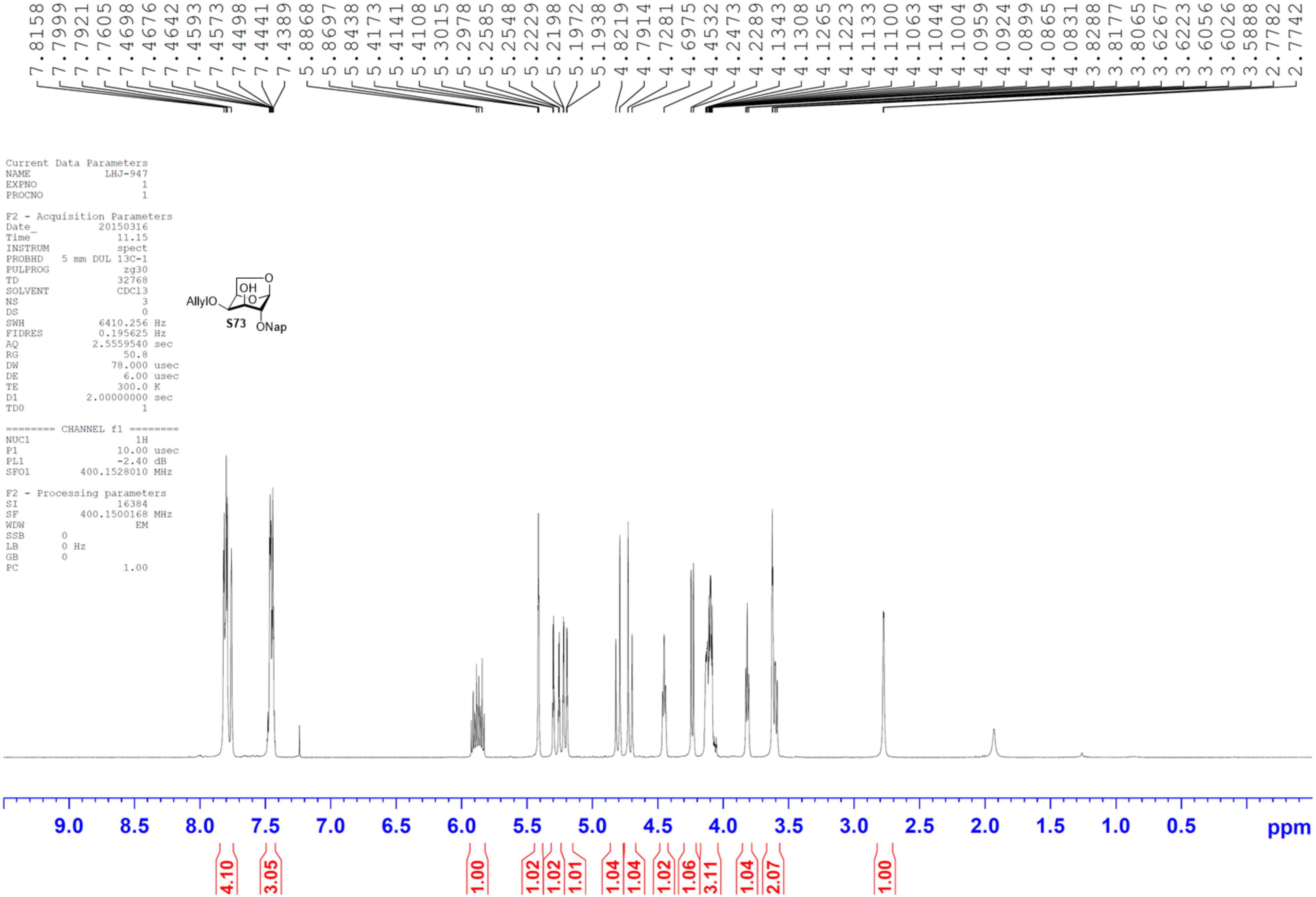
7.8158
7.7999
7.7921
7.7605
7.4698
7.4676
7.4642
7.4593
7.4573
7.4498
7.4441
7.4389
5.8868
5.8697
5.8438
5.4173
5.4141
5.4108
5.3015
5.2978
5.2585
5.2548
5.2229
5.2198
5.1972
5.1938
4.8219
4.7914
4.7281
4.6975
4.4532
4.2473
4.2289
4.1343
4.1308
4.1265
4.1223
4.1133
4.1100
4.1063
4.1044
4.1004
4.0959
4.0924
4.0899
4.0865
4.0831
3.8288
3.8177
3.8065
3.6267
3.6223
3.6056
3.6026
3.5888
2.7782
2.7742
Current Data Parameters
NAME LHJ-947
EXPNO 1
PROCNO 1
F2 - Acquisition Parameters
Date_ 20150316
Time 11.15
INSTRUM spect
PROBHD 5 mm DUL 13C-1
PULPROG zg30
TD 32768
SOLVENT CDCl3
NS 3
DS 0
SWH 6410.256 Hz
FIDRES 0.195625 Hz
AQ 2.5559540 sec
RG 50.8
DW 78.000 usec
DE 6.00 usec
TE 300.0 K
D1 2.00000000 sec
TD0 1
======== CHANNEL f1 ========
NUC1 1H
P1 10.00 usec
PL1 -2.40 dB
SFO1 400.1528010 MHz
F2 - Processing parameters
SI 16384
SF 400.1500168 MHz
WDW EM
SSB 0
LB 0 Hz
GB 0
PC 1.00
AllylO
OH
O
O
ONap
S73
9.0
8.5
8.0
7.5
7.0
6.5
6.0
5.5
5.0
4.5
4.0
3.5
3.0
2.5
2.0
1.5
1.0
0.5
ppm
4.10
3.05
1.00
1.02
1.02
1.01
1.04
1.04
1.02
1.06
3.11
1.04
2.07
1.00

```
Current Data Parameters
NAME             LHJ-947
EXPNO                  2
PROCNO                 1

F2 - Acquisition Parameters
Date_           20150316
Time               11.16
INSTRUM            spect
PROBHD   5 mm DUL 13C-1
PULPROG          zgpg30
TD                65536
SOLVENT            CDCl3
NS                    20
DS                     0
SWH            22727.273 Hz
FIDRES          0.346791 Hz
AQ             1.4418420 sec
RG                    57
DW                22.000 usec
DE                  6.00 usec
TE                 300.0 K
D1            2.00000000 sec
d11           0.03000000 sec
DELTA         1.89999998 sec
TD0                    1

======== CHANNEL f1 ========
NUC1                 13C
P1                  9.70 usec
PL1                -0.50 dB
SFO1         100.6288660 MHz

======== CHANNEL f2 ========
CPDPRG2          waltz16
NUC2                  1H
PCPD2              90.00 usec
PL2                -2.40 dB
PL12               15.10 dB
PL13               18.10 dB
SFO2         400.1516010 MHz

F2 - Processing parameters
SI                 32768
SF           100.6178189 MHz
WDW                   EM
SSB        0
LB                  3.00 Hz
GB         0
PC                  1.00
```

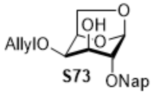

S73

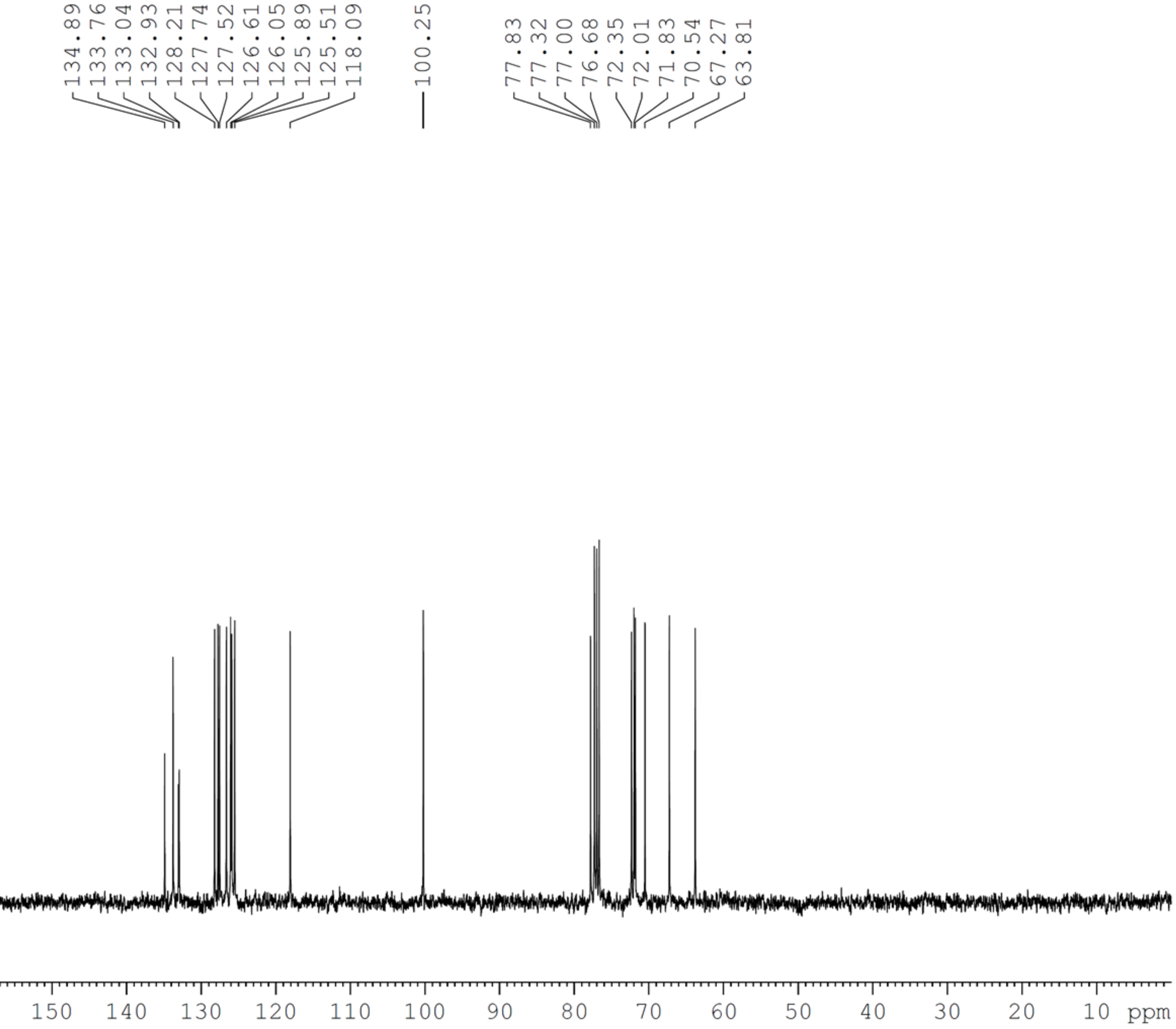

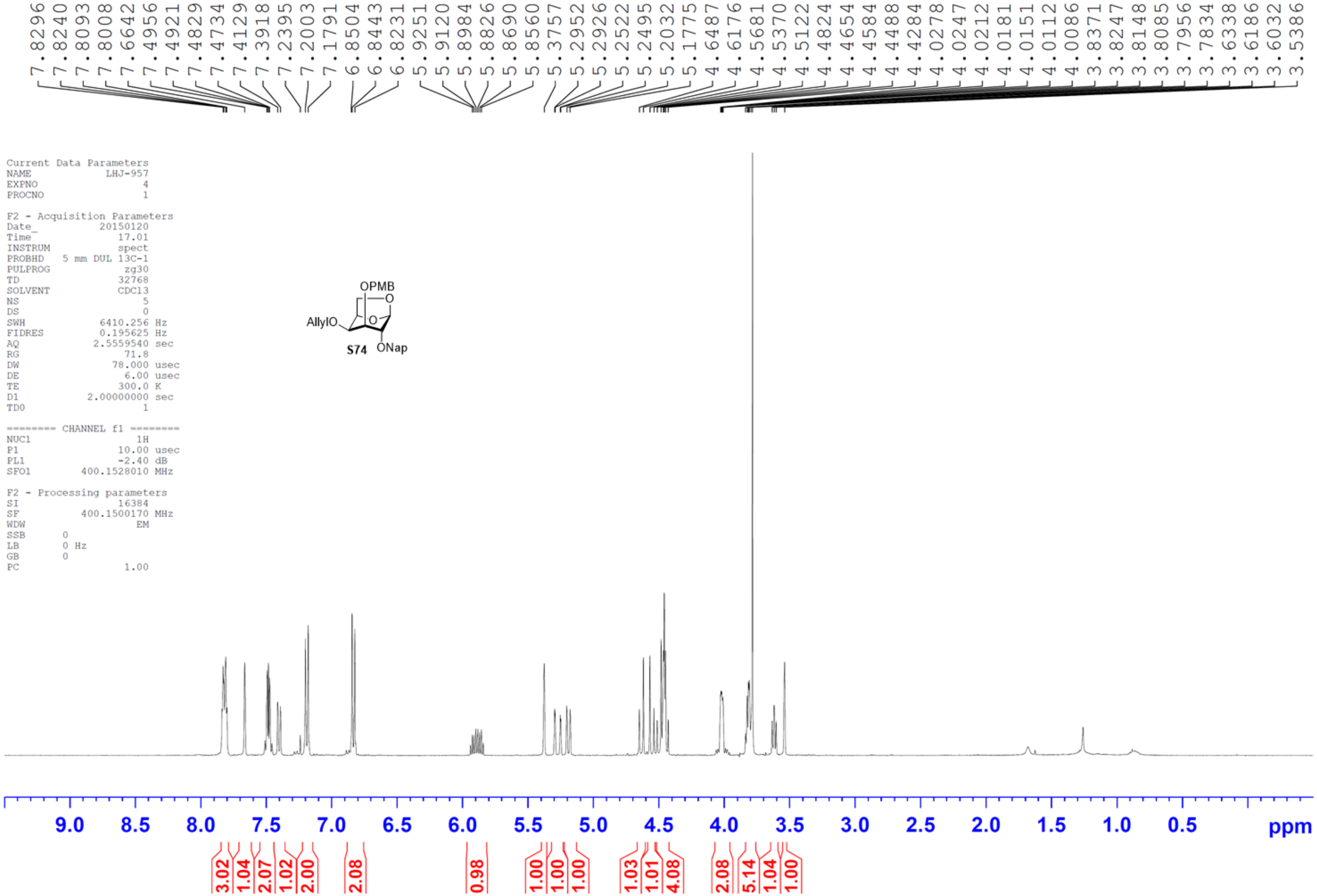
7.8296
7.8240
7.8093
7.8008
7.6642
7.4956
7.4921
7.4829
7.4734
7.4129
7.3918
7.2395
7.2003
7.1791
6.8504
6.8443
6.8231
5.9251
5.9120
5.8984
5.8826
5.8690
5.8560
5.3757
5.2952
5.2926
5.2522
5.2495
5.2032
5.1775
4.6487
4.6176
4.5681
4.5370
4.5122
4.4824
4.4654
4.4584
4.4488
4.4284
4.0278
4.0247
4.0212
4.0181
4.0151
4.0112
4.0086
3.8371
3.8247
3.8148
3.8085
3.7956
3.7834
3.6338
3.6186
3.6032
3.5386
Current Data Parameters
NAME LHJ-957
EXPNO 4
PROCNO 1
F2 - Acquisition Parameters
Date_ 20150120
Time 17.01
INSTRUM spect
PROBHD 5 mm DUL 13C-1
PULPROG zg30
TD 32768
SOLVENT CDCl3
NS 5
DS 0
SWH 6410.256 Hz
FIDRES 0.195625 Hz
AQ 2.5559540 sec
RG 71.8
DW 78.000 usec
DE 6.00 usec
TE 300.0 K
D1 2.00000000 sec
TD0 1
======== CHANNEL f1 ========
NUC1 1H
P1 10.00 usec
PL1 -2.40 dB
SFO1 400.1528010 MHz
F2 - Processing parameters
SI 16384
SF 400.1500170 MHz
WDW EM
SSB 0
LB 0 Hz
GB 0
PC 1.00
OPMB
O
AllylO
O
ONap
S74
9.0
8.5
8.0
7.5
7.0
6.5
6.0
5.5
5.0
4.5
4.0
3.5
3.0
2.5
2.0
1.5
1.0
0.5
ppm
3.02
1.04
2.07
1.02
2.00
2.08
0.98
1.00
1.00
1.00
1.03
1.01
4.08
2.08
5.14
1.04
1.00

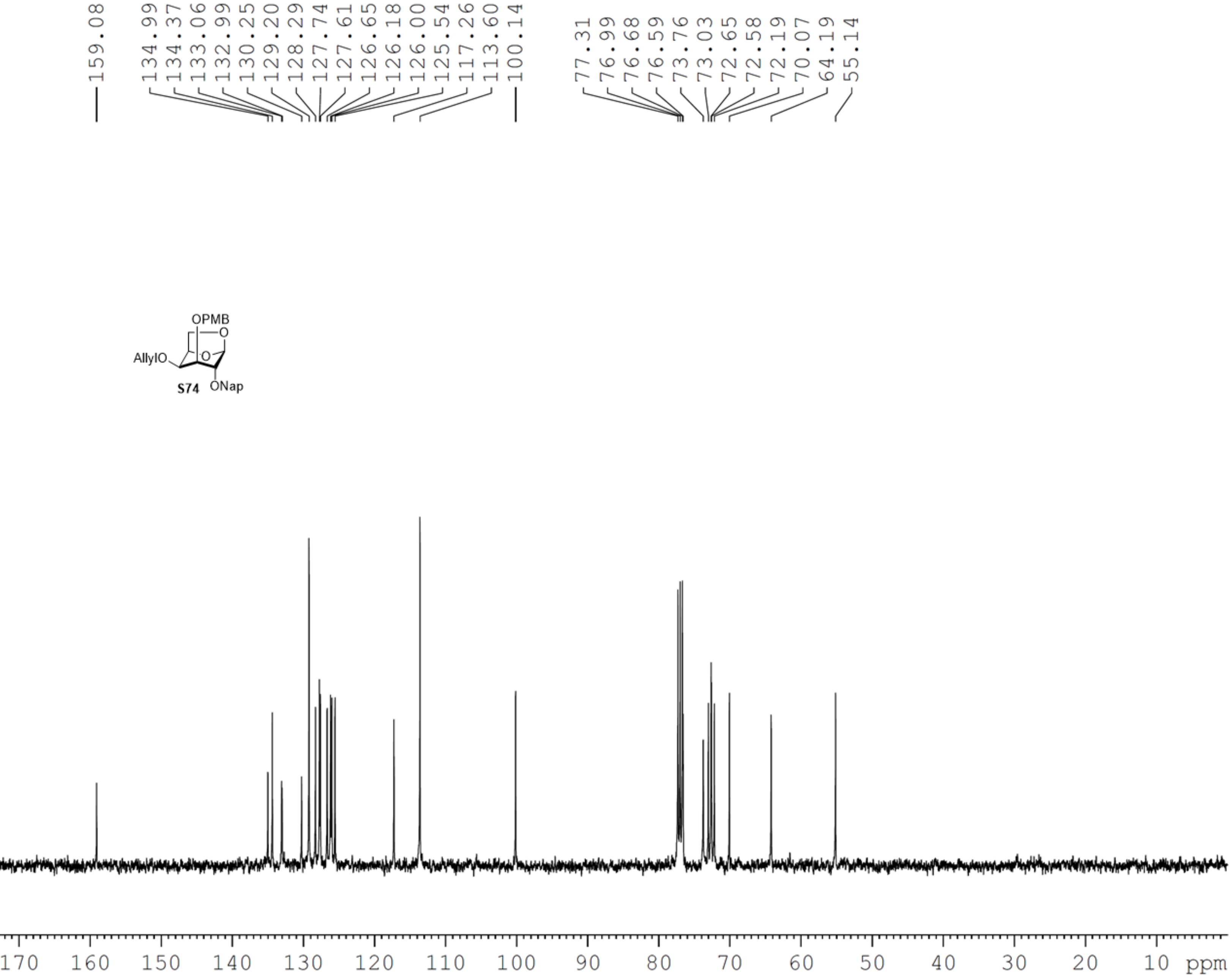

Current Data Parameters
NAME LHJ-957
EXPNO 3
PROCNO 1

F2 - Acquisition Parameters
Date_ 20150120
Time 15.50
INSTRUM spect
PROBHD 5 mm DUL 13C-1
PULPROG zgpg30
TD 65536
SOLVENT CDCl3
NS 55
DS 0
SWH 22727.273 Hz
FIDRES 0.346791 Hz
AQ 1.4418420 sec
RG 57
DW 22.000 usec
DE 6.00 usec
TE 300.0 K
D1 2.00000000 sec
d11 0.03000000 sec
DELTA 1.89999998 sec
TD0 1

======== CHANNEL f1 ========
NUC1 13C
P1 9.70 usec
PL1 -0.50 dB
SFO1 100.6288660 MHz

======== CHANNEL f2 ========
CPDPRG2 waltz16
NUC2 1H
PCPD2 90.00 usec
PL2 -2.40 dB
PL12 15.10 dB
PL13 18.10 dB
SFO2 400.1516010 MHz

F2 - Processing parameters
SI 32768
SF 100.6178142 MHz
WDW EM
SSB 0
LB 3.00 Hz
GB 0
PC 1.00

S74

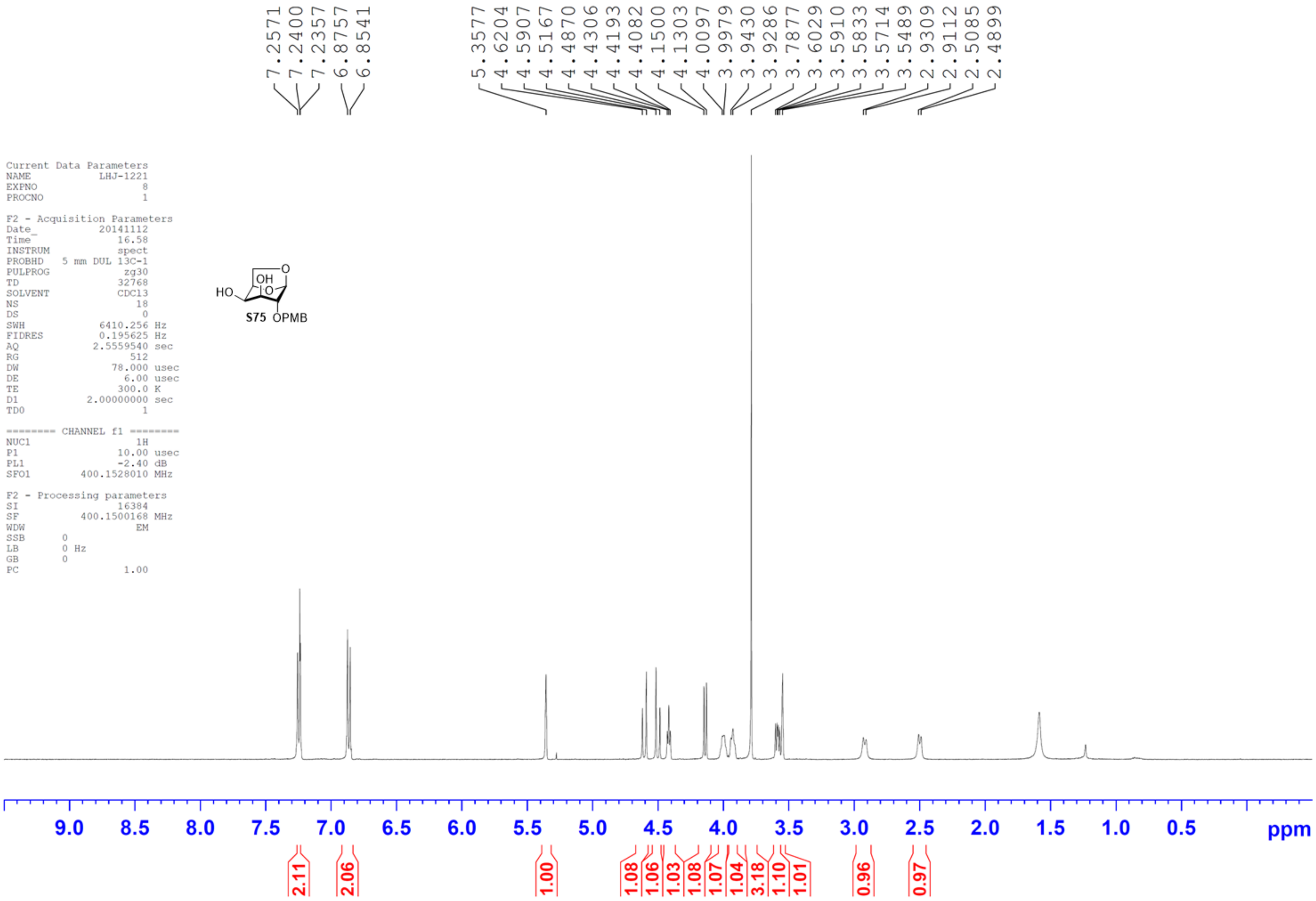
7.2571
7.2400
7.2357
6.8757
6.8541
5.3577
4.6204
4.5907
4.5167
4.4870
4.4306
4.4193
4.4082
4.1500
4.1303
4.0097
3.9979
3.9430
3.9286
3.7877
3.6029
3.5910
3.5833
3.5714
3.5489
2.9309
2.9112
2.5085
2.4899
Current Data Parameters
NAME LHJ-1221
EXPNO 8
PROCNO 1
F2 - Acquisition Parameters
Date_ 20141112
Time 16.58
INSTRUM spect
PROBHD 5 mm DUL 13C-1
PULPROG zg30
TD 32768
SOLVENT CDCl3
NS 18
DS 0
SWH 6410.256 Hz
FIDRES 0.195625 Hz
AQ 2.5559540 sec
RG 512
DW 78.000 usec
DE 6.00 usec
TE 300.0 K
D1 2.00000000 sec
TD0 1
======== CHANNEL f1 ========
NUC1 1H
P1 10.00 usec
PL1 -2.40 dB
SFO1 400.1528010 MHz
F2 - Processing parameters
SI 16384
SF 400.1500168 MHz
WDW EM
SSB 0
LB 0 Hz
GB 0
PC 1.00
HO
OH
O
O
S75
OPMB
9.0
8.5
8.0
7.5
7.0
6.5
6.0
5.5
5.0
4.5
4.0
3.5
3.0
2.5
2.0
1.5
1.0
0.5
ppm
2.11
2.06
1.00
1.08
1.06
1.03
1.08
1.07
1.04
3.18
1.10
1.01
0.96
0.97

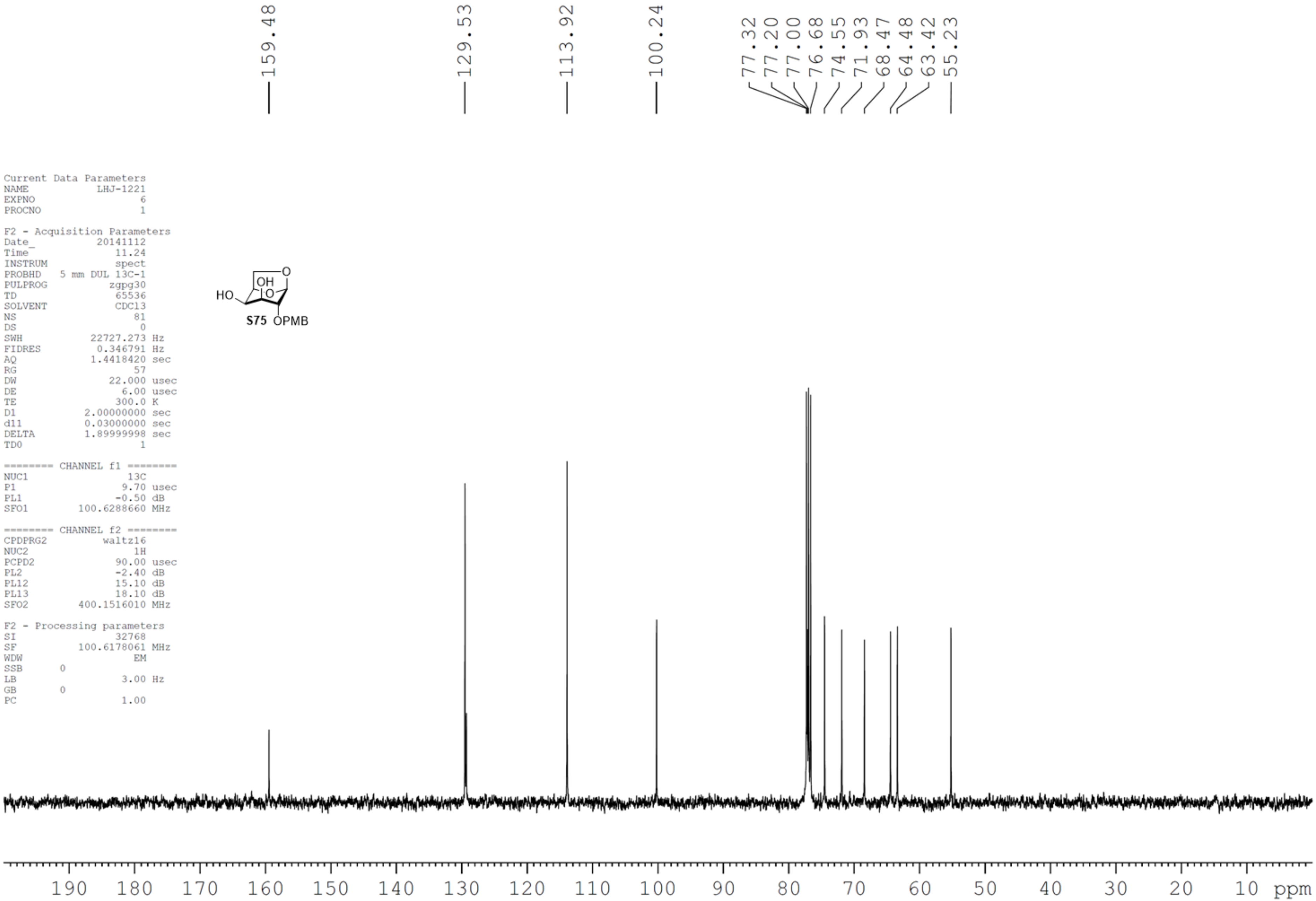


```
Current Data Parameters
NAME            LHJ-1221
EXPNO                  6
PROCNO                 1

F2 - Acquisition Parameters
Date_           20141112
Time               11.24
INSTRUM            spect
PROBHD   5 mm DUL 13C-1
PULPROG          zgpg30
TD                 65536
SOLVENT            CDCl3
NS                    81
DS                     0
SWH            22727.273 Hz
FIDRES          0.346791 Hz
AQ             1.4418420 sec
RG                    57
DW                22.000 usec
DE                  6.00 usec
TE                 300.0 K
D1            2.00000000 sec
d11           0.03000000 sec
DELTA         1.89999998 sec
TD0                    1

======== CHANNEL f1 ========
NUC1                 13C
P1                  9.70 usec
PL1                -0.50 dB
SFO1         100.6288660 MHz

======== CHANNEL f2 ========
CPDPRG2          waltz16
NUC2                  1H
PCPD2              90.00 usec
PL2                -2.40 dB
PL12               15.10 dB
PL13               18.10 dB
SFO2         400.1516010 MHz

F2 - Processing parameters
SI                 32768
SF           100.6178061 MHz
WDW                   EM
SSB      0
LB                  3.00 Hz
GB       0
PC                  1.00
```

S75

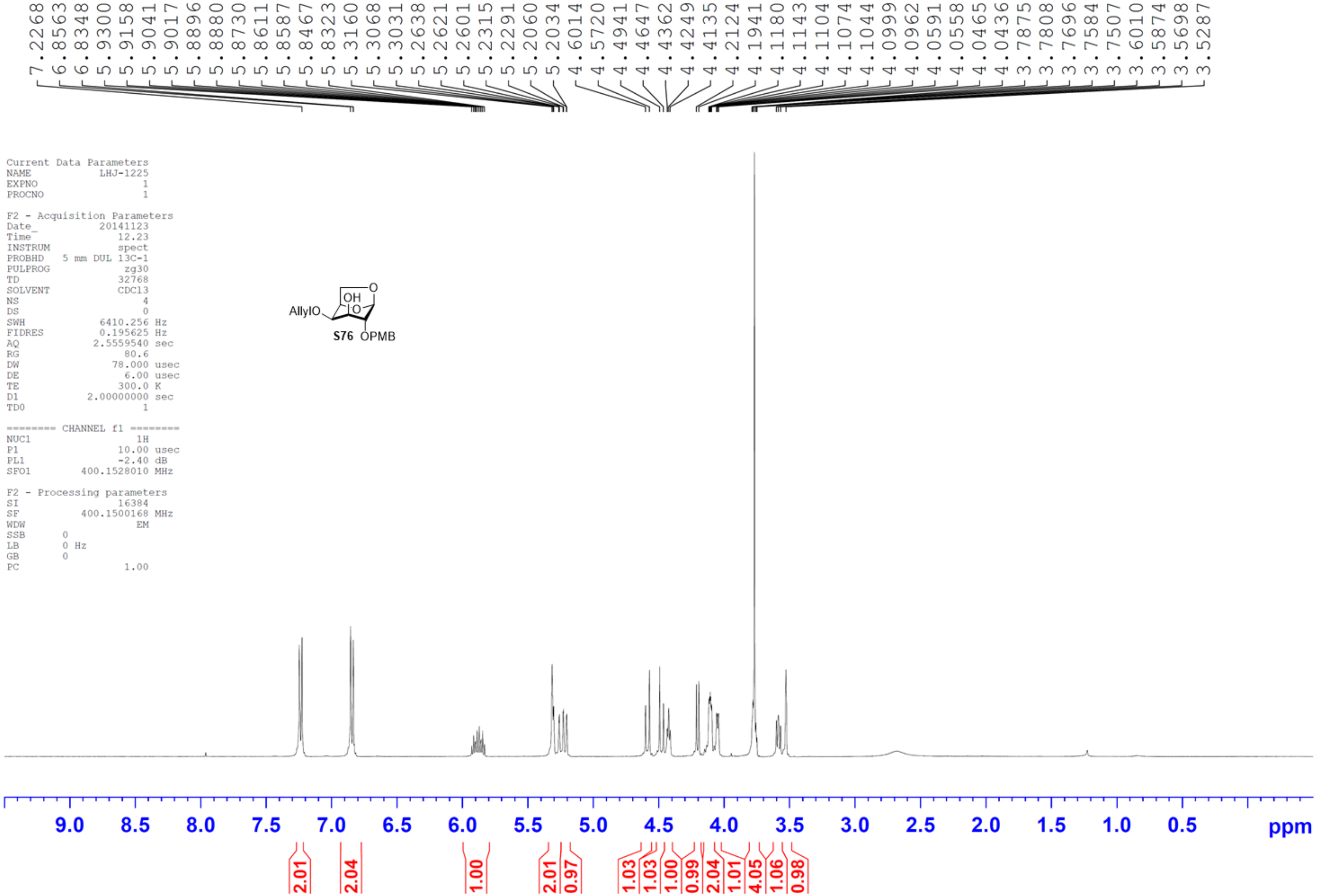
7.2268 6.8563 6.8348 5.9300 5.9158 5.9041 5.9017 5.8896 5.8880 5.8730 5.8611 5.8587 5.8467 5.8323 5.3160 5.3068 5.3031 5.2638 5.2621 5.2601 5.2315 5.2291 5.2060 5.2034 4.6014 4.5720 4.4941 4.4647 4.4362 4.4249 4.4135 4.2124 4.1941 4.1180 4.1143 4.1104 4.1074 4.1044 4.0999 4.0962 4.0591 4.0558 4.0465 4.0436 3.7875 3.7808 3.7696 3.7584 3.7507 3.6010 3.5874 3.5698 3.5287
Current Data Parameters
NAME LHJ-1225
EXPNO 1
PROCNO 1
F2 - Acquisition Parameters
Date_ 20141123
Time 12.23
INSTRUM spect
PROBHD 5 mm DUL 13C-1
PULPROG zg30
TD 32768
SOLVENT CDCl3
NS 4
DS 0
SWH 6410.256 Hz
FIDRES 0.195625 Hz
AQ 2.5559540 sec
RG 80.6
DW 78.000 usec
DE 6.00 usec
TE 300.0 K
D1 2.00000000 sec
TD0 1
======== CHANNEL f1 ========
NUC1 1H
P1 10.00 usec
PL1 -2.40 dB
SFO1 400.1528010 MHz
F2 - Processing parameters
SI 16384
SF 400.1500168 MHz
WDW EM
SSB 0
LB 0 Hz
GB 0
PC 1.00
AllylO
OH
O
O
S76
OPMB
9.0 8.5 8.0 7.5 7.0 6.5 6.0 5.5 5.0 4.5 4.0 3.5 3.0 2.5 2.0 1.5 1.0 0.5 ppm
2.01 2.04 1.00 2.01 0.97 1.03 1.03 1.00 0.99 2.04 1.01 4.05 1.06 0.98

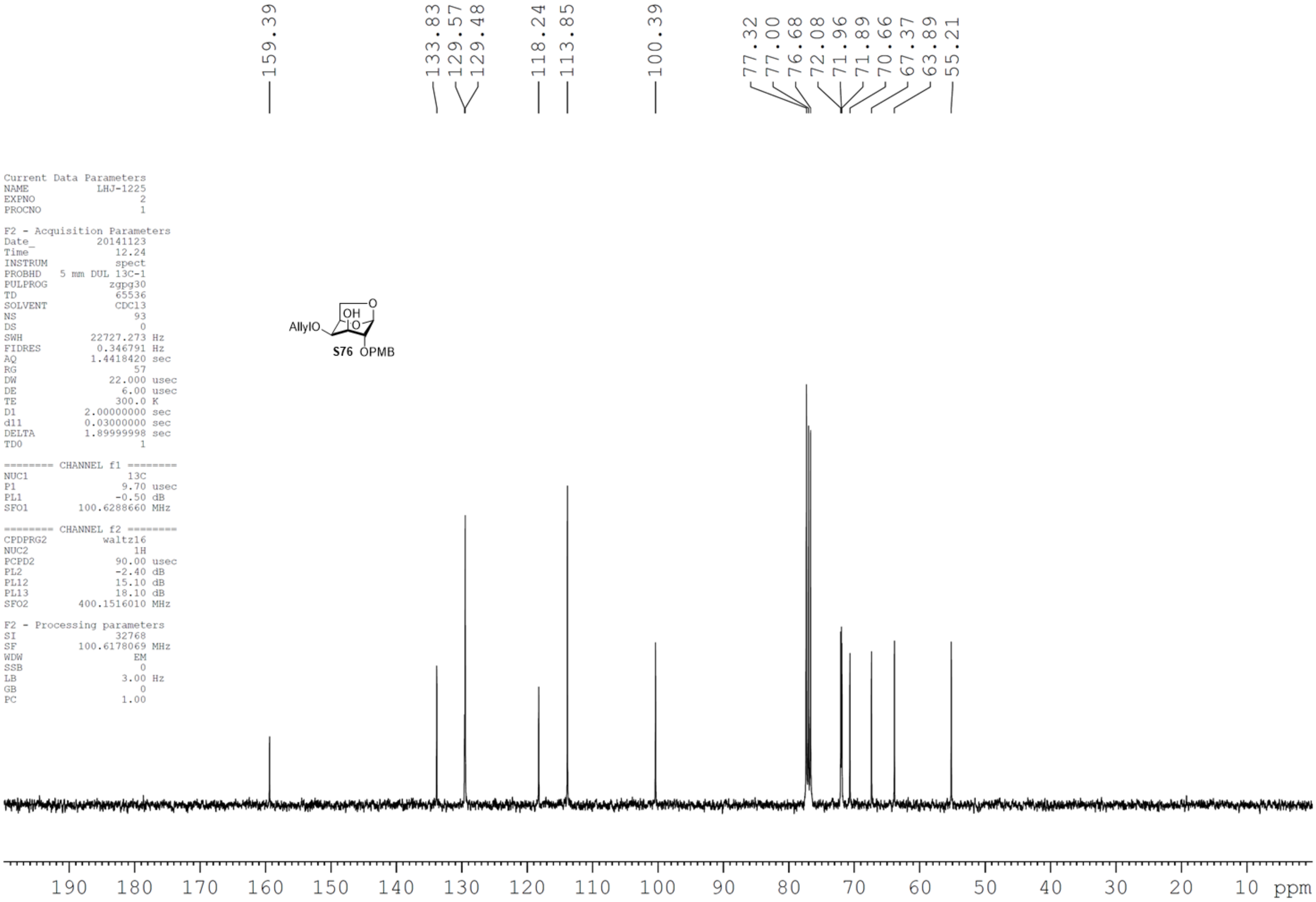

159.39
133.83
129.57
129.48
118.24
113.85
100.39
77.32
77.00
76.68
72.08
71.96
71.89
70.66
67.37
63.89
55.21
Current Data Parameters
NAME LHJ-1225
EXPNO 2
PROCNO 1
F2 - Acquisition Parameters
Date_ 20141123
Time 12.24
INSTRUM spect
PROBHD 5 mm DUL 13C-1
PULPROG zgpg30
TD 65536
SOLVENT CDCl3
NS 93
DS 0
SWH 22727.273 Hz
FIDRES 0.346791 Hz
AQ 1.4418420 sec
RG 57
DW 22.000 usec
DE 6.00 usec
TE 300.0 K
D1 2.00000000 sec
d11 0.03000000 sec
DELTA 1.89999998 sec
TD0 1
======== CHANNEL f1 ========
NUC1 13C
P1 9.70 usec
PL1 -0.50 dB
SFO1 100.6288660 MHz
======== CHANNEL f2 ========
CPDPRG2 waltz16
NUC2 1H
PCPD2 90.00 usec
PL2 -2.40 dB
PL12 15.10 dB
PL13 18.10 dB
SFO2 400.1516010 MHz
F2 - Processing parameters
SI 32768
SF 100.6178069 MHz
WDW EM
SSB 0
LB 3.00 Hz
GB 0
PC 1.00
190 180 170 160 150 140 130 120 110 100 90 80 70 60 50 40 30 20 10 ppm
AllylO
OH
O
O
OPMB

S76

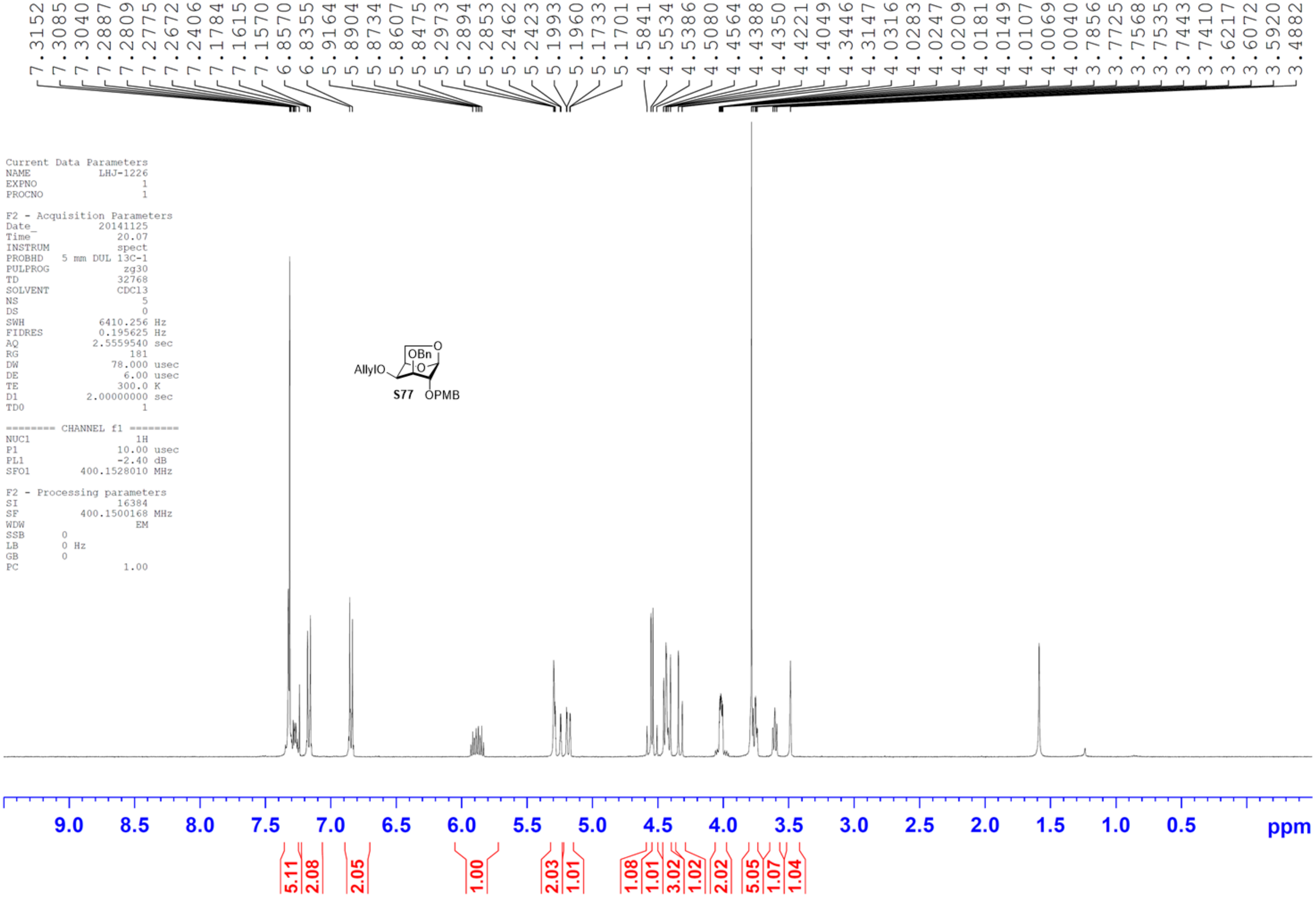

7.3152
7.3085
7.3040
7.2887
7.2809
7.2775
7.2672
7.2406
7.1784
7.1615
7.1570
6.8570
6.8355
5.9164
5.8904
5.8734
5.8607
5.8475
5.2973
5.2894
5.2853
5.2462
5.2423
5.1993
5.1960
5.1733
5.1701
4.5841
4.5534
4.5386
4.5080
4.4564
4.4388
4.4350
4.4221
4.4049
4.3446
4.3147
4.0316
4.0283
4.0247
4.0209
4.0181
4.0149
4.0107
4.0069
4.0040
3.7856
3.7725
3.7568
3.7535
3.7443
3.7410
3.6217
3.6072
3.5920
3.4882
Current Data Parameters
NAME LHJ-1226
EXPNO 1
PROCNO 1
F2 - Acquisition Parameters
Date_ 20141125
Time 20.07
INSTRUM spect
PROBHD 5 mm DUL 13C-1
PULPROG zg30
TD 32768
SOLVENT CDCl3
NS 5
DS 0
SWH 6410.256 Hz
FIDRES 0.195625 Hz
AQ 2.5559540 sec
RG 181
DW 78.000 usec
DE 6.00 usec
TE 300.0 K
D1 2.00000000 sec
TD0 1
======== CHANNEL f1 ========
NUC1 1H
P1 10.00 usec
PL1 -2.40 dB
SFO1 400.1528010 MHz
F2 - Processing parameters
SI 16384
SF 400.1500168 MHz
WDW EM
SSB 0
LB 0 Hz
GB 0
PC 1.00
AllylO
OBn
O
O
OPMB
S77
9.0
8.5
8.0
7.5
7.0
6.5
6.0
5.5
5.0
4.5
4.0
3.5
3.0
2.5
2.0
1.5
1.0
0.5
ppm
5.11
2.08
2.05
1.00
2.03
1.01
1.08
1.01
3.02
1.02
2.02
5.05
1.07
1.04

```
Current Data Parameters
NAME            LHJ-1226
EXPNO                  2
PROCNO                 1

F2 - Acquisition Parameters
Date_           20141125
Time               20.08
INSTRUM            spect
PROBHD   5 mm DUL 13C-1
PULPROG          zgpg30
TD                 65536
SOLVENT            CDCl3
NS                   602
DS                     0
SWH           22727.273 Hz
FIDRES         0.346791 Hz
AQ            1.4418420 sec
RG                    57
DW                22.000 usec
DE                  6.00 usec
TE                 300.0 K
D1            2.00000000 sec
d11           0.03000000 sec
DELTA         1.89999998 sec
TD0                    1

======== CHANNEL f1 ========
NUC1                 13C
P1                  9.70 usec
PL1                -0.50 dB
SFO1         100.6288660 MHz

======== CHANNEL f2 ========
CPDPRG2          waltz16
NUC2                  1H
PCPD2              90.00 usec
PL2                -2.40 dB
PL12               15.10 dB
PL13               18.10 dB
SFO2         400.1516010 MHz

F2 - Processing parameters
SI                 32768
SF           100.6178004 MHz
WDW                   EM
SSB      0
LB                  3.00 Hz
GB       0
PC                  1.00
```

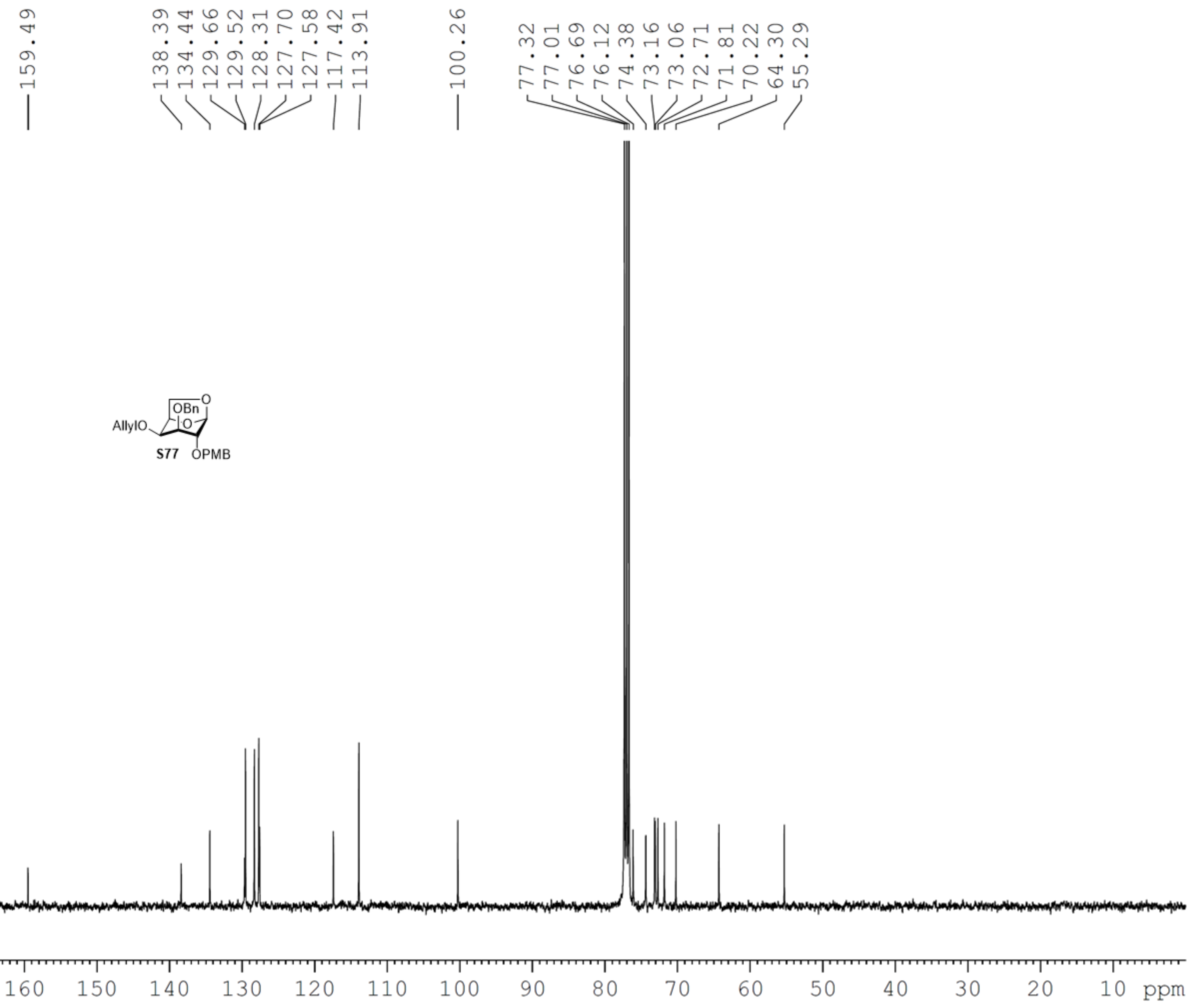

Current Data Parameters
NAME LHJ-1248
EXPNO 3
PROCNO 1

F2 - Acquisition Parameters
Date_ 20150119
Time 16.49
INSTRUM spect
PROBHD 5 mm DUL 13C-1
PULPROG zg30
TD 32768
SOLVENT CDCl3
NS 7
DS 0
SWH 6410.256 Hz
FIDRES 0.195625 Hz
AQ 2.5559540 sec
RG 57
DW 78.000 usec
DE 6.00 usec
TE 300.0 K
D1 2.00000000 sec
TD0 1

======== CHANNEL f1 ========
NUC1 1H
P1 10.00 usec
PL1 -2.40 dB
SFO1 400.1528010 MHz

F2 - Processing parameters
SI 16384
SF 400.1500168 MHz
WDW EM
SSB 0
LB 0 Hz
GB 0
PC 1.00

S78

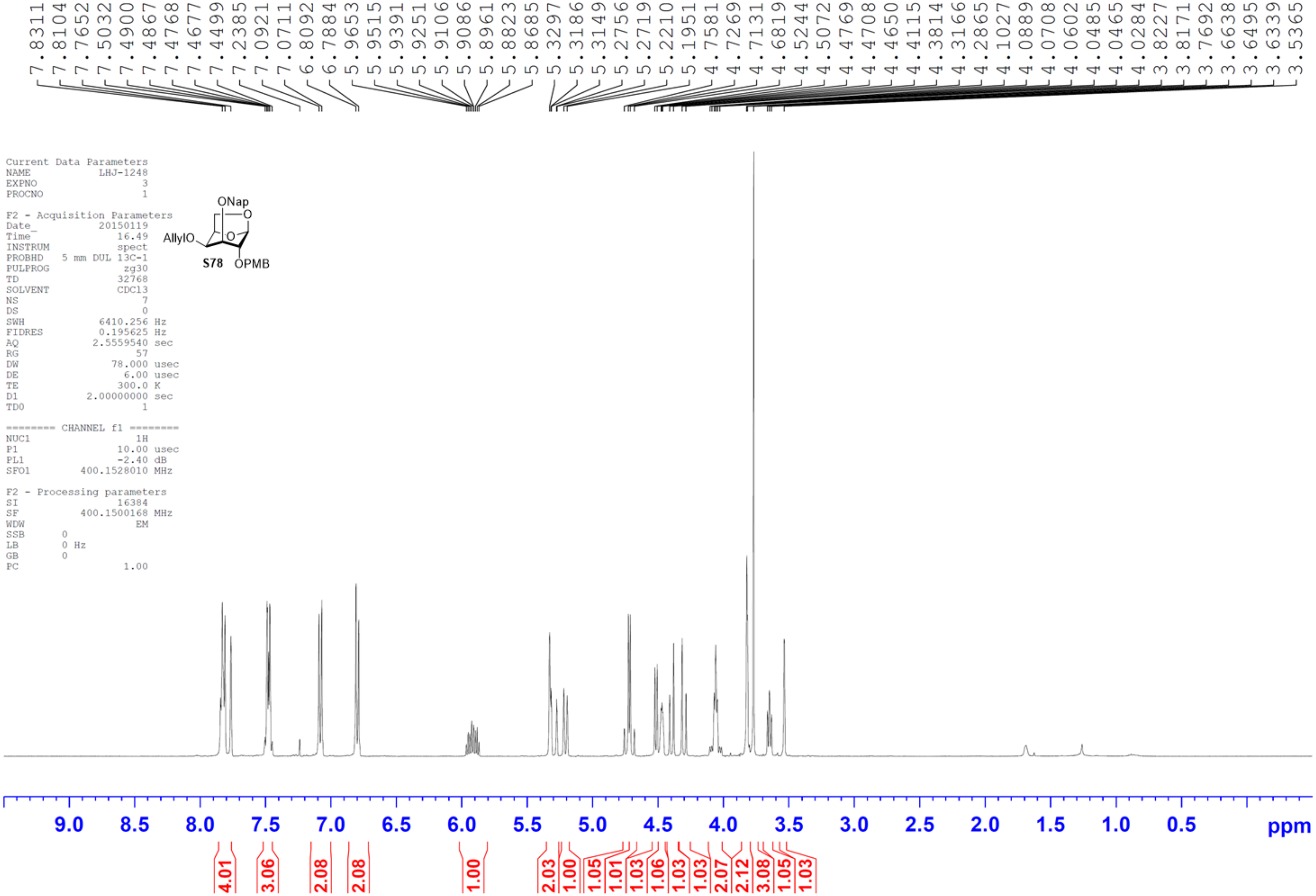

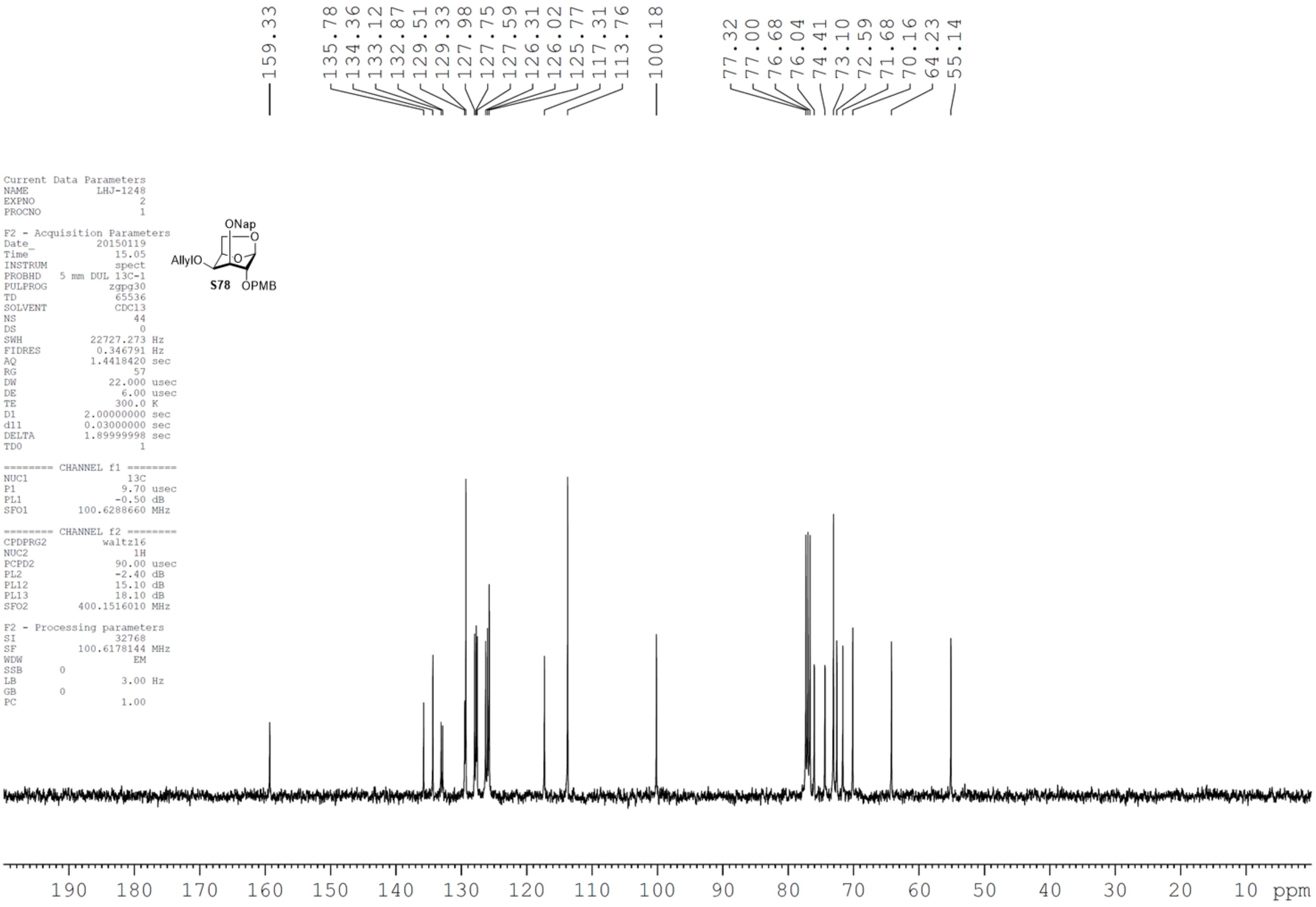
159.33
135.78
134.36
133.12
132.87
129.51
129.33
127.98
127.75
127.59
126.31
126.02
125.77
117.31
113.76
100.18
77.32
77.00
76.68
76.04
74.41
73.10
72.59
71.68
70.16
64.23
55.14
Current Data Parameters
NAME LHJ-1248
EXPNO 2
PROCNO 1
F2 - Acquisition Parameters
Date_ 20150119
Time 15.05
INSTRUM spect
PROBHD 5 mm DUL 13C-1
PULPROG zgpg30
TD 65536
SOLVENT CDCl3
NS 44
DS 0
SWH 22727.273 Hz
FIDRES 0.346791 Hz
AQ 1.4418420 sec
RG 57
DW 22.000 usec
DE 6.00 usec
TE 300.0 K
D1 2.00000000 sec
d11 0.03000000 sec
DELTA 1.89999998 sec
TD0 1
======== CHANNEL f1 ========
NUC1 13C
P1 9.70 usec
PL1 -0.50 dB
SFO1 100.6288660 MHz
======== CHANNEL f2 ========
CPDPRG2 waltz16
NUC2 1H
PCPD2 90.00 usec
PL2 -2.40 dB
PL12 15.10 dB
PL13 18.10 dB
SFO2 400.1516010 MHz
F2 - Processing parameters
SI 32768
SF 100.6178144 MHz
WDW EM
SSB 0
LB 3.00 Hz
GB 0
PC 1.00
ONap
O
AllylO
O
OPMB
190 180 170 160 150 140 130 120 110 100 90 80 70 60 50 40 30 20 10 ppm
S78

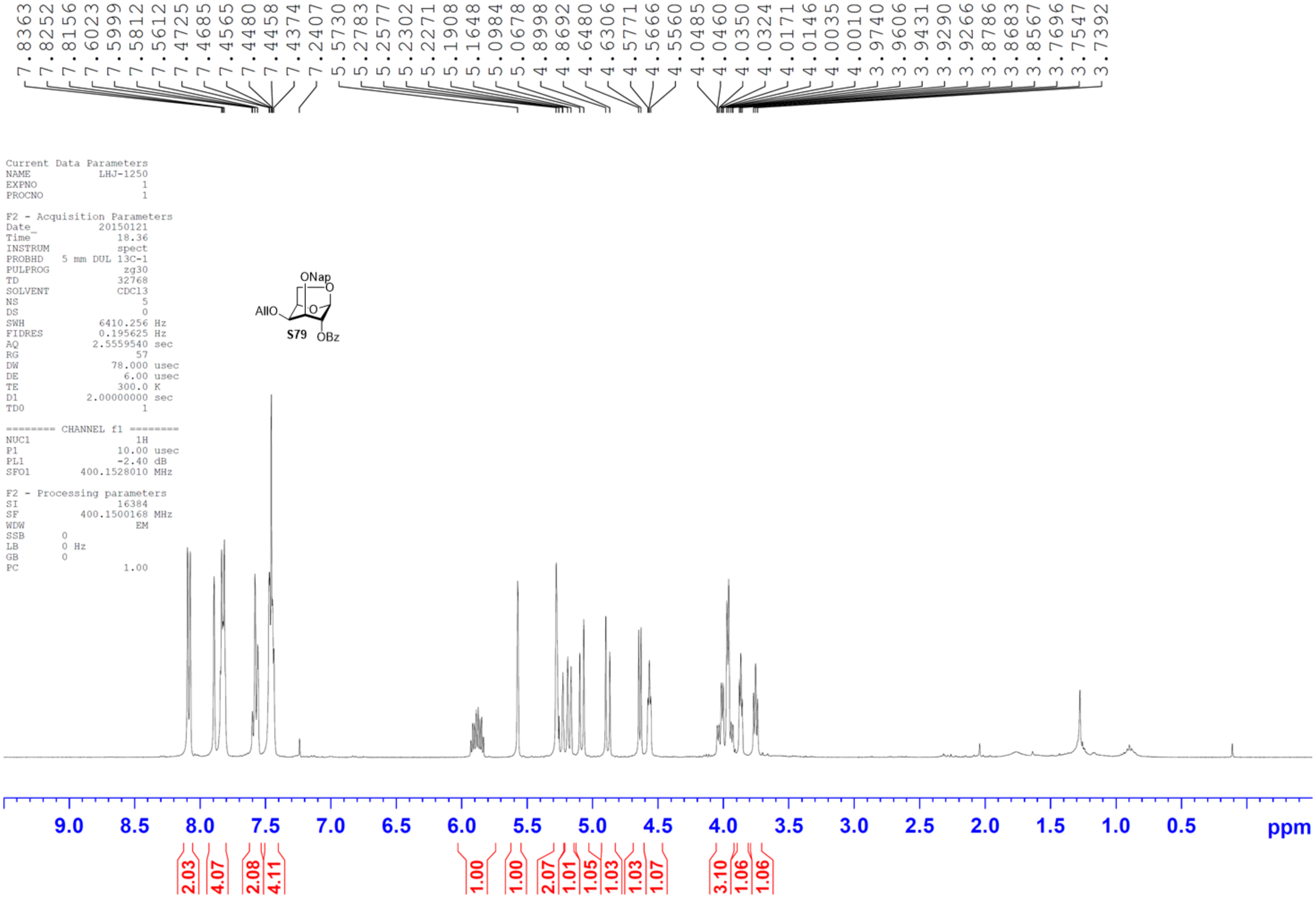
7.8363 7.8252 7.8156 7.6023 7.5999 7.5812 7.5612 7.4725 7.4685 7.4565 7.4480 7.4458 7.4374 7.2407 5.5730 5.2783 5.2577 5.2302 5.2271 5.1908 5.1648 5.0984 5.0678 4.8998 4.8692 4.6480 4.6306 4.5771 4.5666 4.5560 4.0485 4.0460 4.0350 4.0324 4.0171 4.0146 4.0035 4.0010 3.9740 3.9606 3.9431 3.9290 3.9266 3.8786 3.8683 3.8567 3.7696 3.7547 3.7392
Current Data Parameters
NAME LHJ-1250
EXPNO 1
PROCNO 1
F2 - Acquisition Parameters
Date_ 20150121
Time 18.36
INSTRUM spect
PROBHD 5 mm DUL 13C-1
PULPROG zg30
TD 32768
SOLVENT CDCl3
NS 5
DS 0
SWH 6410.256 Hz
FIDRES 0.195625 Hz
AQ 2.5559540 sec
RG 57
DW 78.000 usec
DE 6.00 usec
TE 300.0 K
D1 2.00000000 sec
TD0 1
======== CHANNEL f1 ========
NUC1 1H
P1 10.00 usec
PL1 -2.40 dB
SFO1 400.1528010 MHz
F2 - Processing parameters
SI 16384
SF 400.1500168 MHz
WDW EM
SSB 0
LB 0 Hz
GB 0
PC 1.00
ONap
O
AllO
O
S79
OBz
9.0 8.5 8.0 7.5 7.0 6.5 6.0 5.5 5.0 4.5 4.0 3.5 3.0 2.5 2.0 1.5 1.0 0.5 ppm
2.03 4.07 2.08 4.11 1.00 1.00 2.07 1.01 1.05 1.03 1.03 1.07 3.10 1.06 1.06

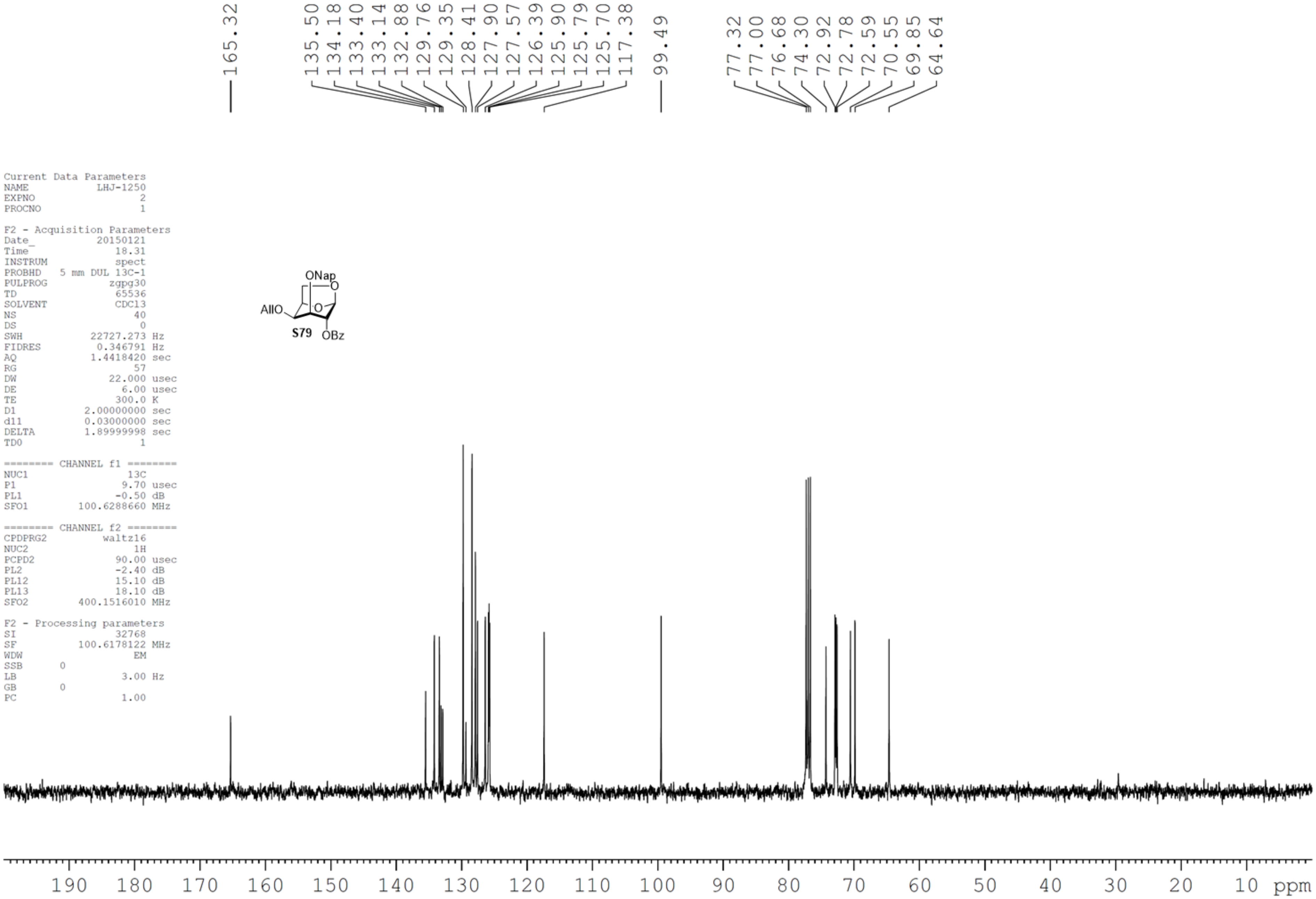


```
Current Data Parameters
NAME          LHJ-1250
EXPNO                2
PROCNO               1

F2 - Acquisition Parameters
Date_         20150121
Time             18.31
INSTRUM          spect
PROBHD   5 mm DUL 13C-1
PULPROG         zgpg30
TD               65536
SOLVENT          CDCl3
NS                  40
DS                   0
SWH          22727.273 Hz
FIDRES        0.346791 Hz
AQ           1.4418420 sec
RG                  57
DW              22.000 usec
DE                6.00 usec
TE               300.0 K
D1          2.00000000 sec
d11         0.03000000 sec
DELTA       1.89999998 sec
TD0                  1

======== CHANNEL f1 ========
NUC1               13C
P1                9.70 usec
PL1              -0.50 dB
SFO1       100.6288660 MHz

======== CHANNEL f2 ========
CPDPRG2        waltz16
NUC2                1H
PCPD2            90.00 usec
PL2              -2.40 dB
PL12             15.10 dB
PL13             18.10 dB
SFO2       400.1516010 MHz

F2 - Processing parameters
SI               32768
SF         100.6178122 MHz
WDW                 EM
SSB      0
LB                3.00 Hz
GB       0
PC                1.00
```


S79

KYT-233-2-2Bz-H

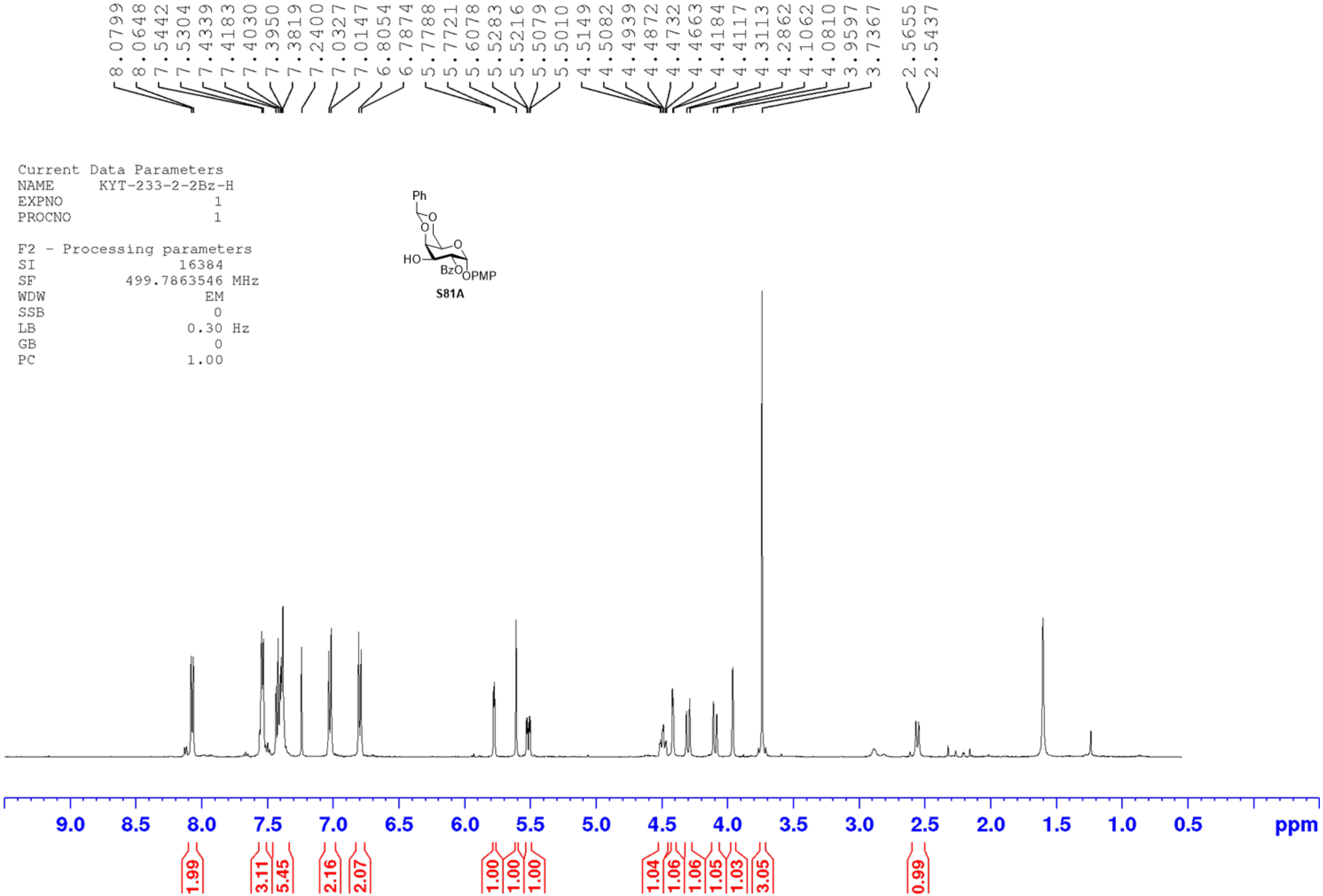

Current Data Parameters
NAME KYT-233-2-2Bz-H
EXPNO 1
PROCNO 1

F2 - Processing parameters
SI 16384
SF 499.7863546 MHz
WDW EM
SSB 0
LB 0.30 Hz
GB 0
PC 1.00

S81A

KYT-232-2-OBz-C

166.43
155.05
150.73
137.24
133.20
129.79
129.50
129.23
128.31
128.24
126.22
117.59
114.59
101.24
96.70
77.25
77.00
76.75
75.93
71.33
69.03
67.26
63.20
55.54

Current Data Parameters
NAME KYT-232-2OBz-C
EXPNO 1
PROCNO 1

F2 - Processing parameters
SI 65536
SF 125.6713920 MHz
WDW EM
SSB 0
LB 0.30 Hz
GB 0
PC 1.00

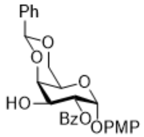

S81A

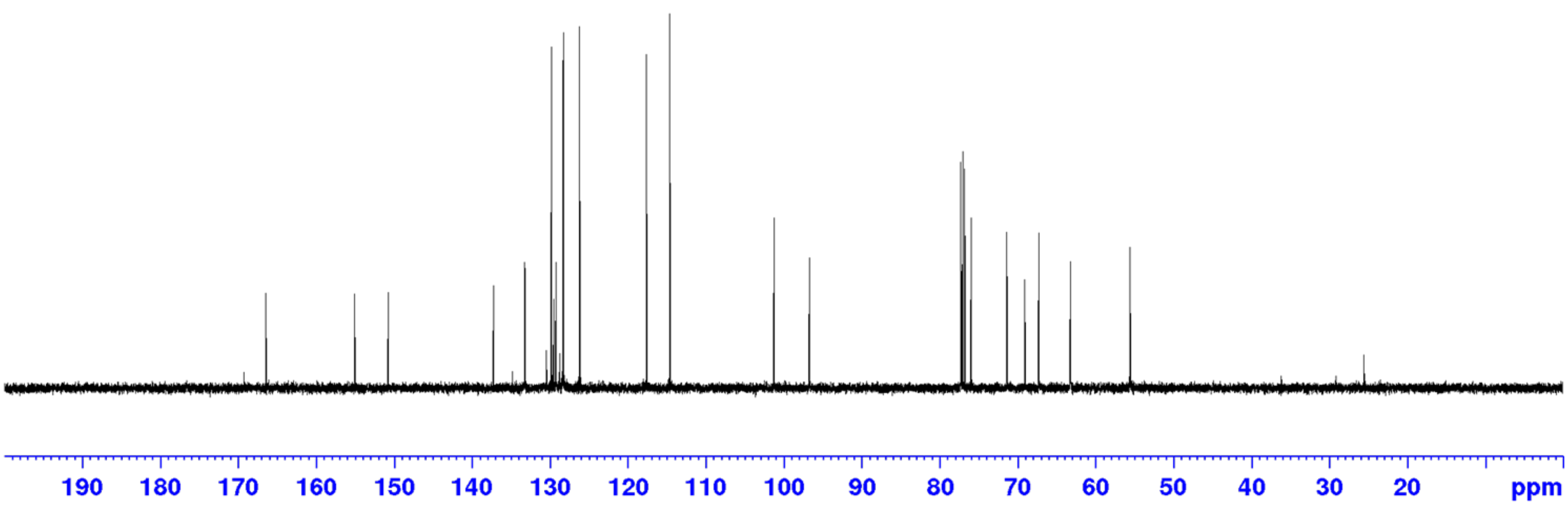

KYT-233-2-up-20191125

Current Data Parameters
NAME KYT-233-2-up
EXPNO 1
PROCNO 1

F2 - Processing parameters
SI 16384
SF 499.7883108 MHz
WDW EM
SSB 0
LB 0.30 Hz
GB 0
PC 1.00

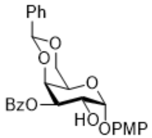

S81B

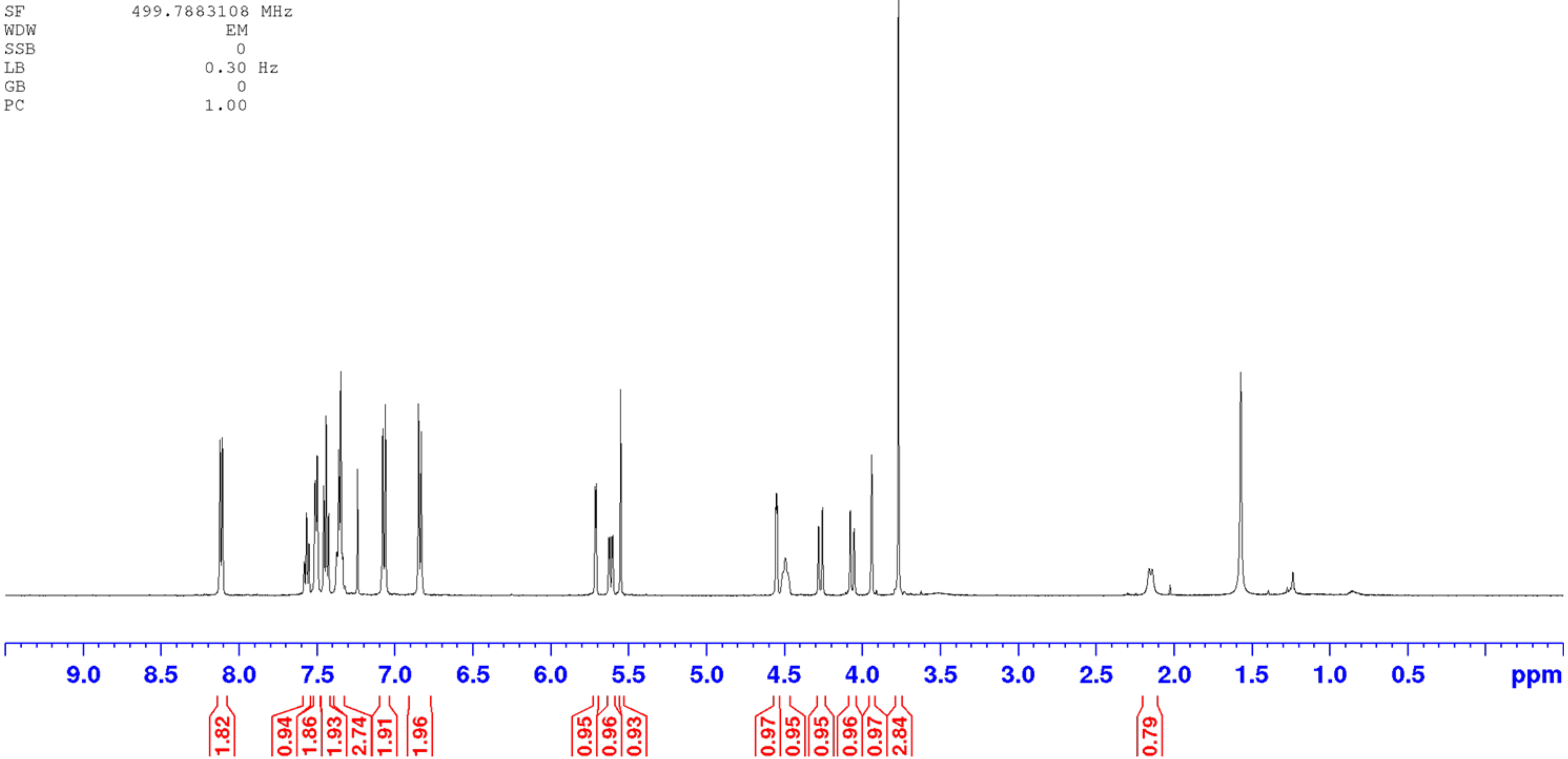

KYT-233-2-3OBz

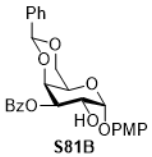

S81B

Current Data Parameters
NAME KYT-233-2-3OBz
EXPNO 2
PROCNO 1

F2 - Acquisition Parameters
Date_ 20200512
Time 16.24
INSTRUM spect
PROBHD 5 mm DUL 13C-1
PULPROG zgpg30
TD 65536
SOLVENT CDCl3
NS 387
DS 0
SWH 22727.273 Hz
FIDRES 0.346791 Hz
AQ 1.4417920 sec
RG 57
DW 22.000 usec
DE 6.00 usec
TE 300.0 K
D1 2.00000000 sec
d11 0.03000000 sec
DELTA 1.89999998 sec
TD0 1

======== CHANNEL f1 ========
NUC1 13C
P1 9.70 usec
PL1 -0.50 dB
SFO1 100.6288660 MHz

======== CHANNEL f2 ========
CPDPRG[2 waltz16
NUC2 1H
PCPD2 90.00 usec
PL2 -2.40 dB
PL12 15.10 dB
PL13 18.10 dB
SFO2 400.1516010 MHz

F2 - Processing parameters
SI 32768
SF 100.6178019 MHz
WDW EM
SSB 0
LB 3.00 Hz
GB 0
PC 1.00

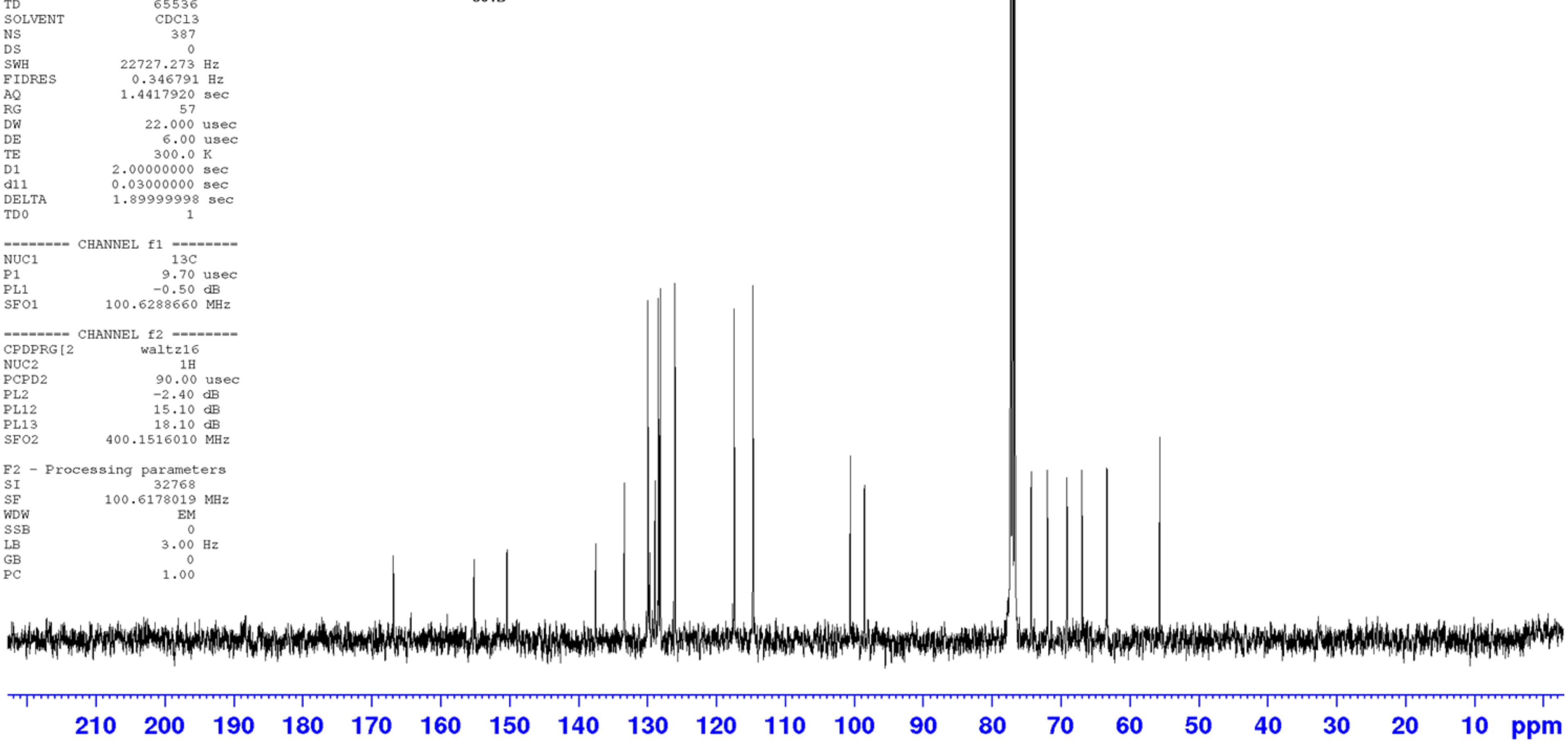

Current Data Parameters
NAME KYT-235-600-2
EXPNO 1
PROCNO 1

F2 - Acquisition Parameters
Date_ 20200827
Time 6.13
INSTRUM spect
PROBHD 5 mm QNP 1H/1
PULPROG zg
TD 32768
SOLVENT CDCl3
NS 128
DS 0
SWH 9541.984 Hz
FIDRES 0.291198 Hz
AQ 1.7170432 sec
RG 1024
DW 52.400 usec
DE 6.50 usec
TE 301.8 K
D1 2.00000000 sec
MCREST 0 sec
MCWRK 0.01500000 sec

======== CHANNEL f1 ========
NUC1 1H
P1 10.00 usec
PL1 -3.00 dB
SFO1 598.1035886 MHz

F2 - Processing parameters
SI 32768
SF 598.1000313 MHz
WDW no
SSB 0
LB 0 Hz
GB 0
PC 2.00

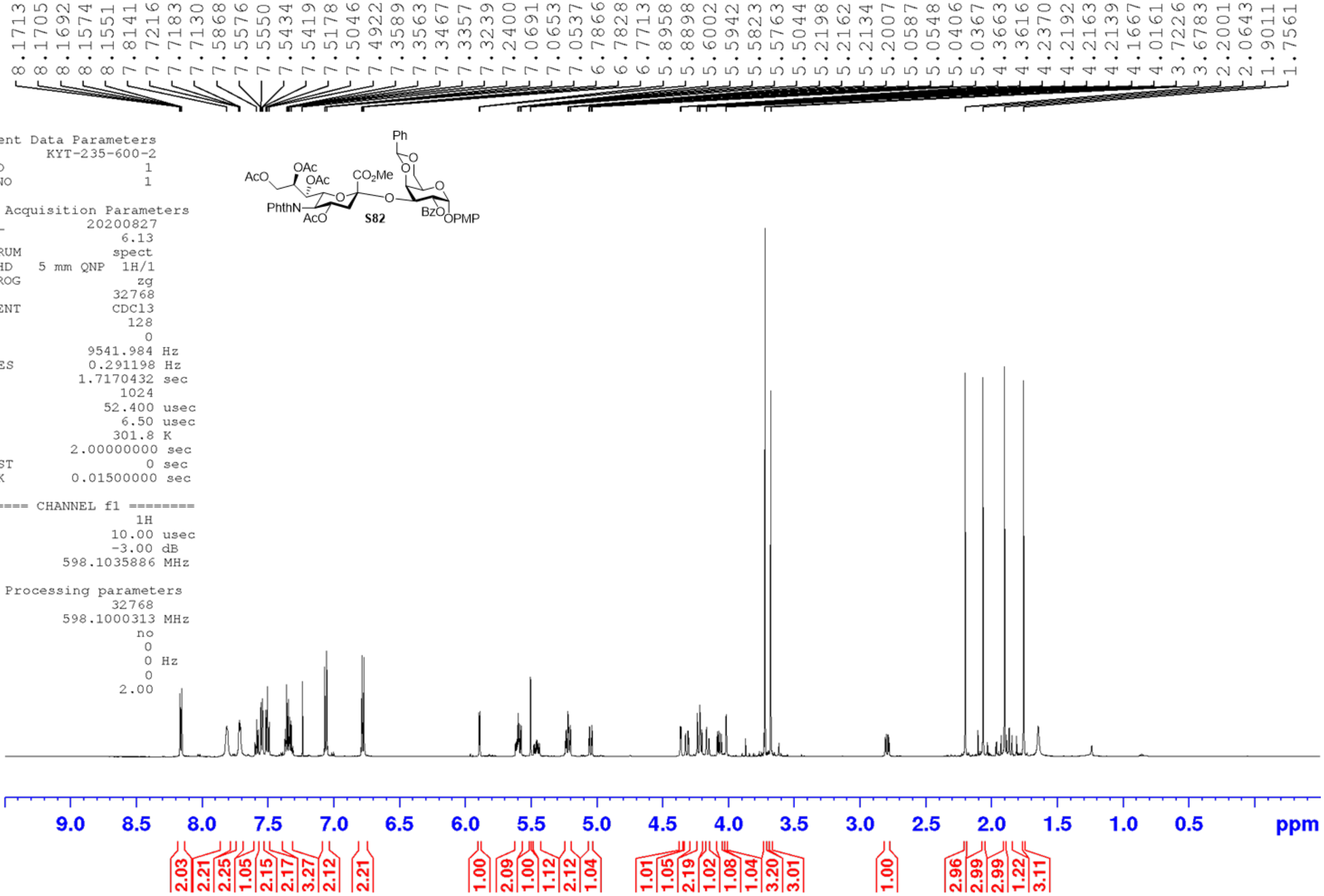

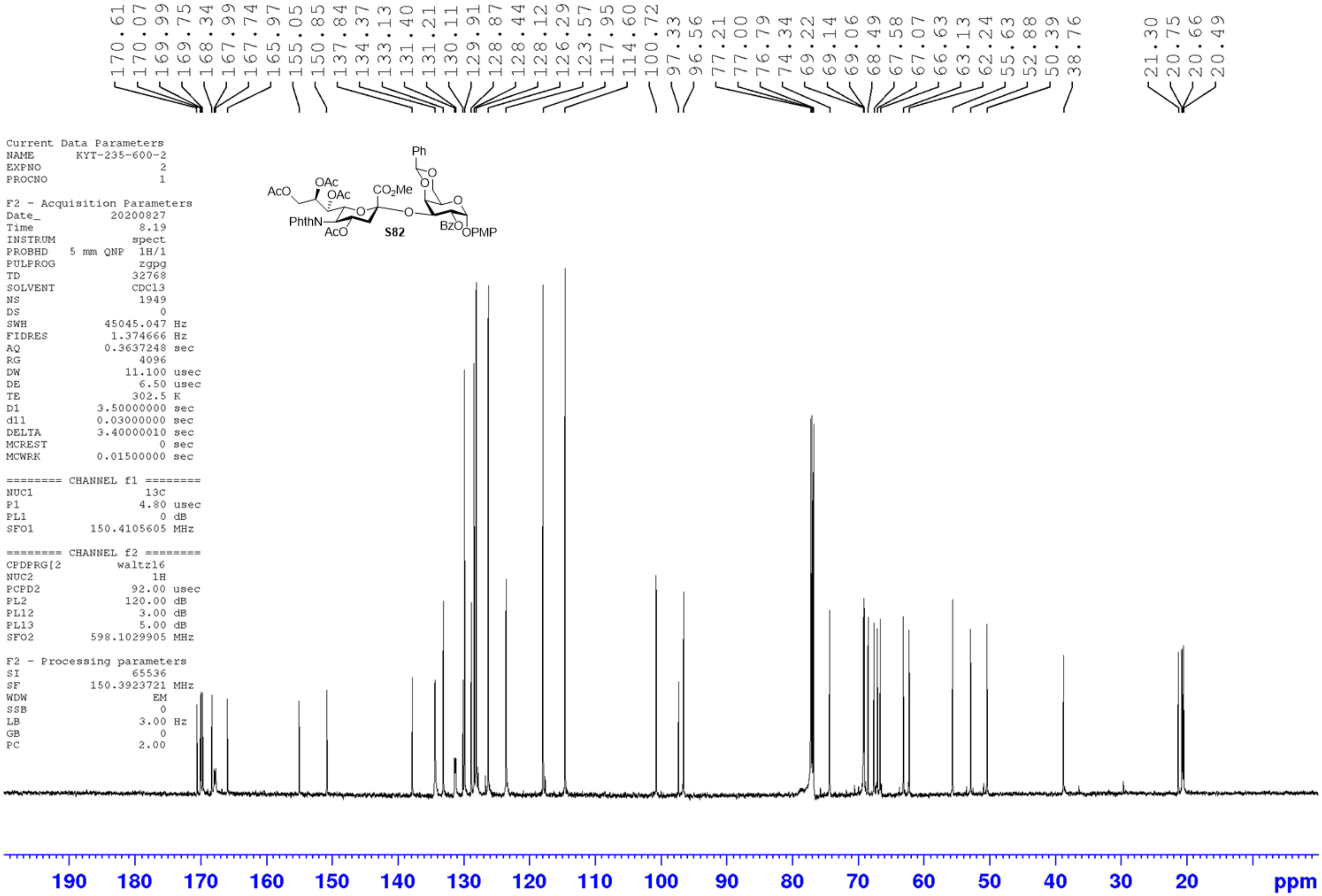
170.61
170.07
169.99
169.75
168.34
167.99
167.74
165.97
155.05
150.85
137.84
134.37
133.13
131.40
131.21
130.11
129.91
128.87
128.44
128.12
126.29
123.57
117.95
114.60
100.72
97.33
96.56
77.21
77.00
76.79
74.34
69.22
69.14
69.06
68.49
67.58
67.07
66.63
63.13
62.24
55.63
52.88
50.39
38.76
21.30
20.75
20.66
20.49
Current Data Parameters
NAME KYT-235-600-2
EXPNO 2
PROCNO 1
F2 - Acquisition Parameters
Date_ 20200827
Time 8.19
INSTRUM spect
PROBHD 5 mm QNP 1H/1
PULPROG zgpg
TD 32768
SOLVENT CDCl3
NS 1949
DS 0
SWH 45045.047 Hz
FIDRES 1.374666 Hz
AQ 0.3637248 sec
RG 4096
DW 11.100 usec
DE 6.50 usec
TE 302.5 K
D1 3.50000000 sec
d11 0.03000000 sec
DELTA 3.40000010 sec
MCREST 0 sec
MCWRK 0.01500000 sec
======== CHANNEL f1 ========
NUC1 13C
P1 4.80 usec
PL1 0 dB
SFO1 150.4105605 MHz
======== CHANNEL f2 ========
CPDPRG[2 waltz16
NUC2 1H
PCPD2 92.00 usec
PL2 120.00 dB
PL12 3.00 dB
PL13 5.00 dB
SFO2 598.1029905 MHz
F2 - Processing parameters
SI 65536
SF 150.3923721 MHz
WDW EM
SSB 0
LB 3.00 Hz
GB 0
PC 2.00
Ph
OAc
AcO
OAc
CO2Me
PhthN
AcO
S82
BzO
OPMP
190
180
170
160
150
140
130
120
110
100
90
80
70
60
50
40
30
20
ppm

HMBC

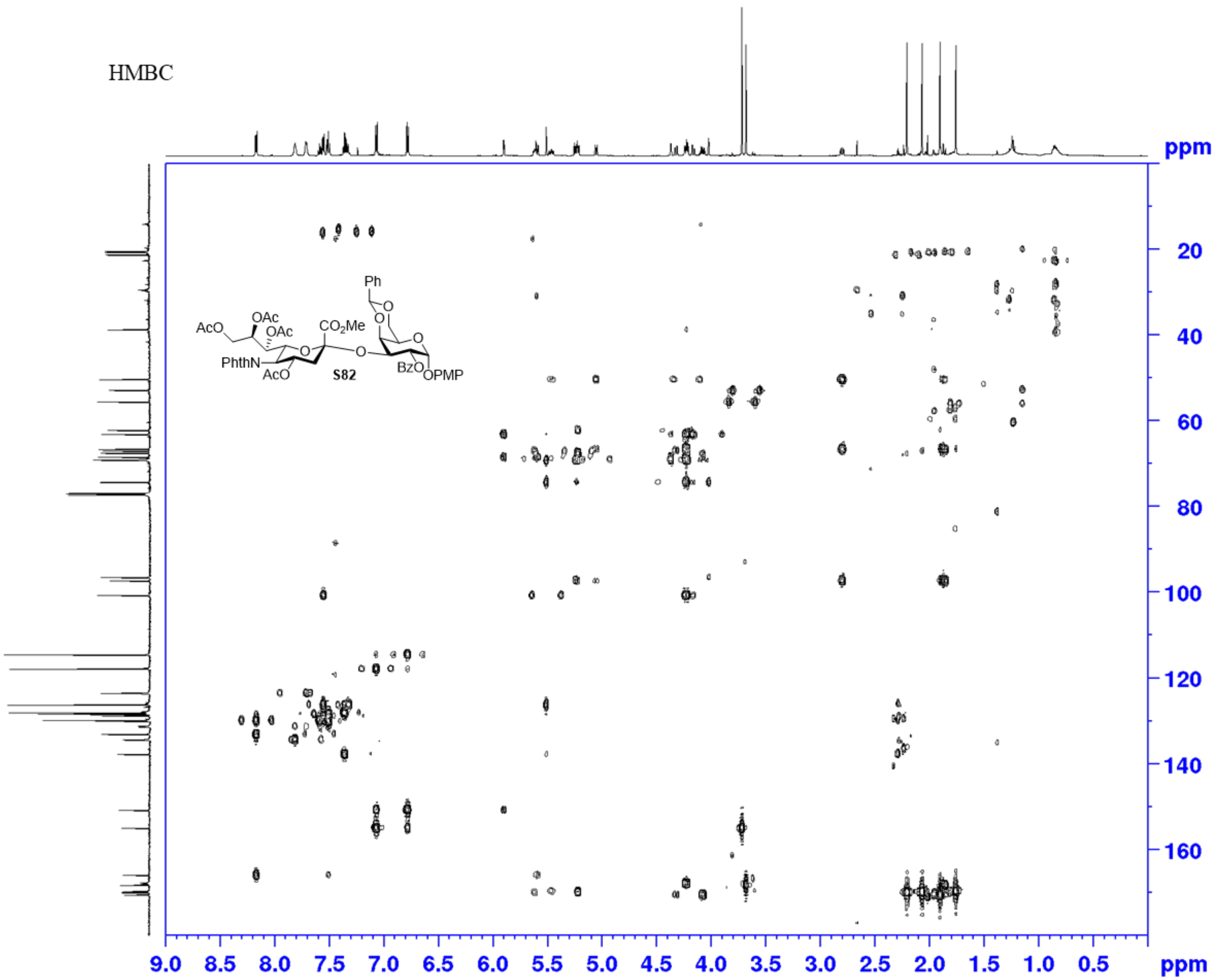


```
Current Data Parameters
NAME          KYT-235-600
EXPNO                  13
PROCNO                  1

F2 - Acquisition Parameters
Date_            20191204
Time                 9.13
INSTRUM             spect
PROBHD   5 mm QNP  1H/1
PULPROG      hmbcgplpndqf
TD                   2048
SOLVENT             CDCl3
NS                      8
DS                      0
SWH              6009.615 Hz
FIDRES           2.934382 Hz
AQ              0.1703936 sec
RG                  32768
DW                 83.200 usec
DE                   6.00 usec
TE                  298.7 K
CNST2         145.0000000
CNST13          8.0000000
d0             0.00000300 sec
D1             1.20000005 sec
d2             0.00344828 sec
d6             0.06250000 sec
D16            0.00050000 sec
IN0            0.00001661 sec
MCREST                  0 sec
MCWRK          1.20000005 sec

======== CHANNEL f1 ========
NUC1                   1H
P1                   9.80 usec
p2                  19.60 usec
PL1                 -3.00 dB
SFO1          598.2028130 MHz

======== CHANNEL f2 ========
NUC2                  13C
P3                  10.50 usec
PL2                     0 dB
SFO2          150.4314988 MHz

====== GRADIENT CHANNEL =====
GPNAM[1]        SINE.100
GPNAM[2]        SINE.100
GPNAM[3]        SINE.100
GPX1                    0 %
GPX2                    0 %
GPX3                    0 %
GPY1                    0 %
GPY2                    0 %
GPY3                    0 %
GPZ1                50.00 %
GPZ2                30.00 %
GPZ3                40.10 %
P16               1000.00 usec

F1 - Acquisition parameters
TD                    196
SFO1             150.4315 MHz
FIDRES         307.120605 Hz
SW                200.077 ppm
FnMODE                 QF

F2 - Processing parameters
SI                   4096
SF            598.2000258 MHz
WDW                 QSINE
SSB                     2
LB                      0 Hz
GB                      0
PC                   1.00

F1 - Processing parameters
SI                    512
MC2                    QF
SF            150.4175103 MHz
WDW                  SINE
SSB                     0
LB                      0 Hz
GB                      0
```

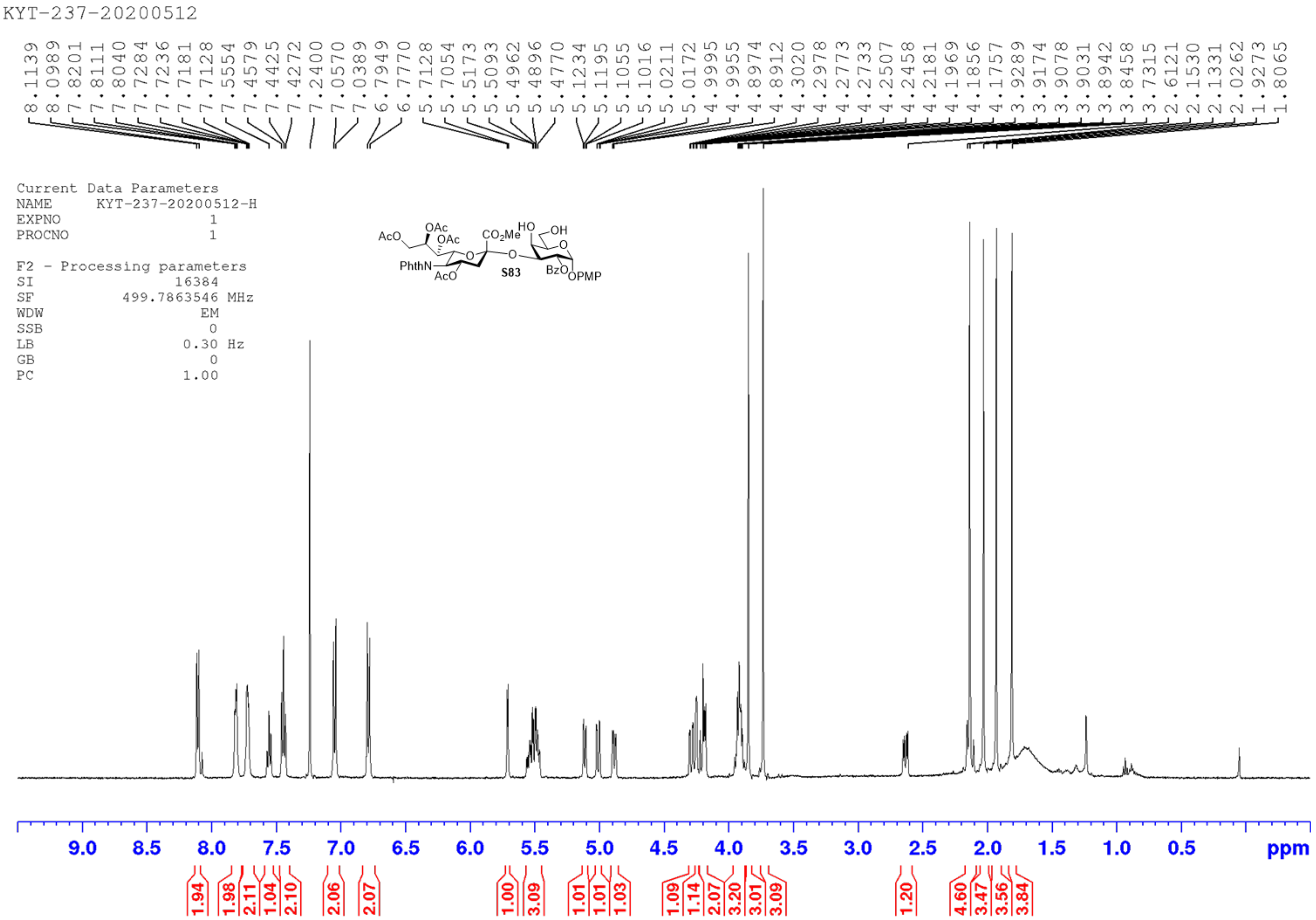
KYT-237-20200512
8.1139
8.0989
7.8201
7.8111
7.8040
7.7284
7.7236
7.7181
7.7128
7.5554
7.4579
7.4425
7.4272
7.2400
7.0570
7.0389
6.7949
6.7770
5.7128
5.7054
5.5173
5.5093
5.4962
5.4896
5.4770
5.1234
5.1195
5.1055
5.1016
5.0211
5.0172
4.9995
4.9955
4.8974
4.8912
4.3020
4.2978
4.2773
4.2733
4.2507
4.2458
4.2181
4.1969
4.1856
4.1757
3.9289
3.9174
3.9078
3.9031
3.8942
3.8458
3.7315
2.6121
2.1530
2.1331
2.0262
1.9273
1.8065
Current Data Parameters
NAME KYT-237-20200512-H
EXPNO 1
PROCNO 1
F2 - Processing parameters
SI 16384
SF 499.7863546 MHz
WDW EM
SSB 0
LB 0.30 Hz
GB 0
PC 1.00
AcO
OAc
OAc
CO_2Me
HO
OH
O
O
O
PhthN
AcO
S83
BzO
OPMP
9.0
8.5
8.0
7.5
7.0
6.5
6.0
5.5
5.0
4.5
4.0
3.5
3.0
2.5
2.0
1.5
1.0
0.5
ppm
1.94
1.98
2.11
1.04
2.10
2.06
2.07
1.00
3.09
1.01
1.01
1.03
1.09
1.14
2.07
3.20
3.01
3.09
1.20
4.60
3.47
3.56
3.84

Current Data Parameters
NAME KYT-237-20200512-C
EXPNO 1
PROCNO 1

F2 - Processing parameters
SI 65536
SF 125.6713864 MHz
WDW EM
SSB 0
LB 3.00 Hz
GB 0
PC 1.00

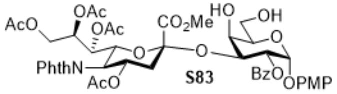

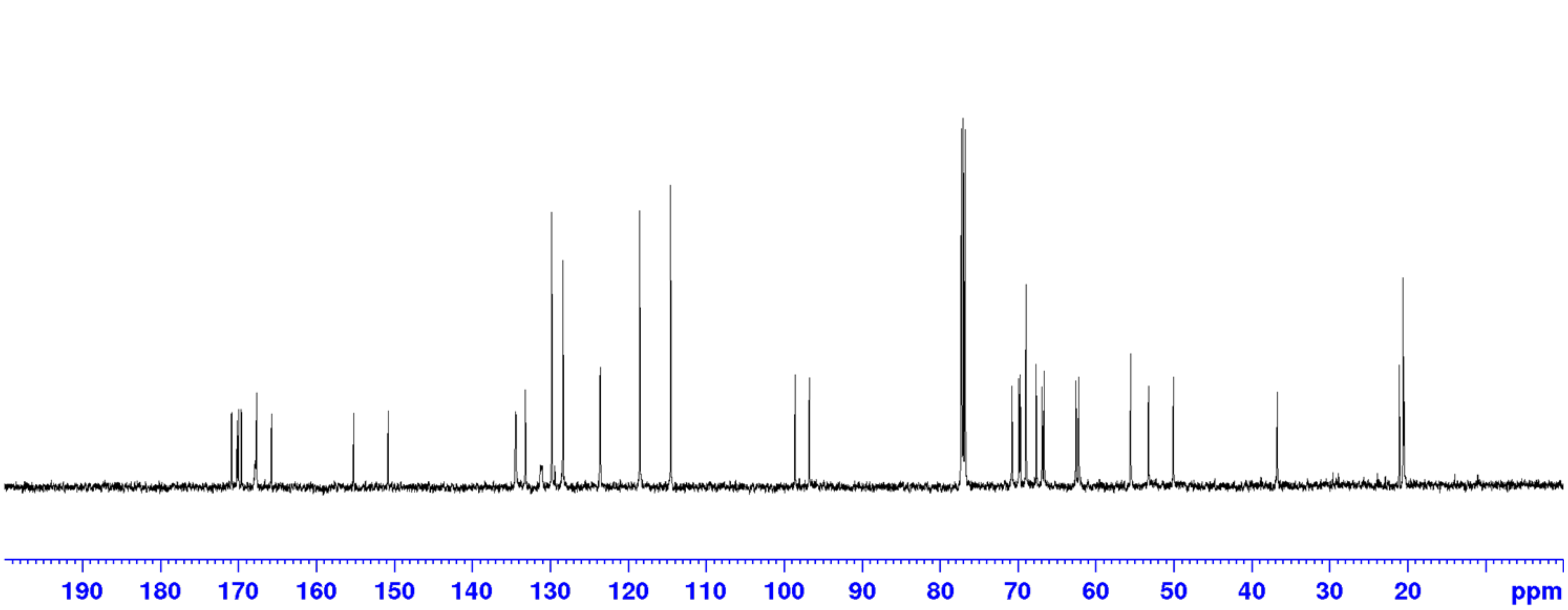

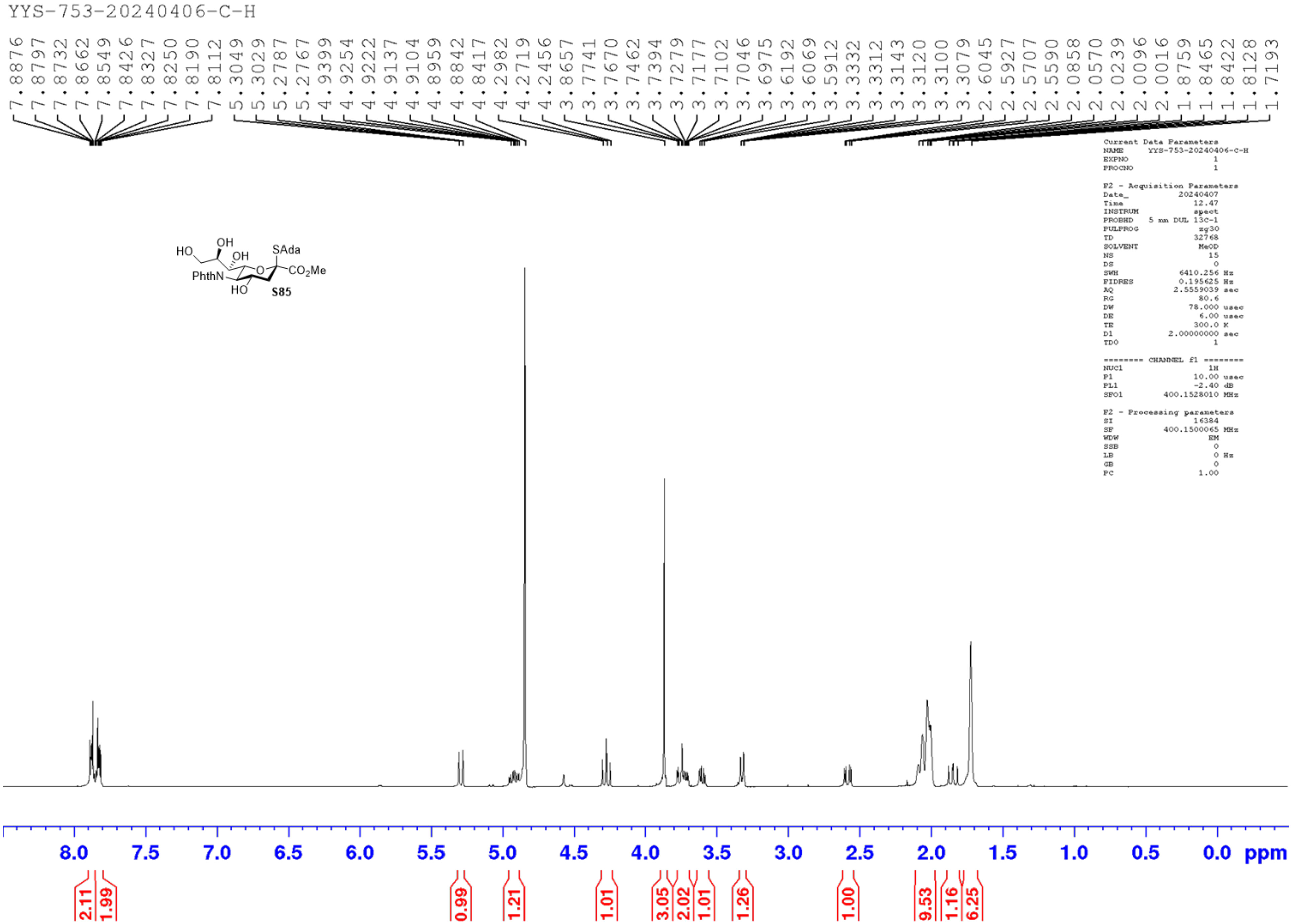
YYS-753-20240406-C-H
7.8876
7.8797
7.8732
7.8662
7.8549
7.8426
7.8327
7.8250
7.8190
7.8112
5.3049
5.3029
5.2787
5.2767
4.9399
4.9254
4.9222
4.9137
4.9104
4.8959
4.8842
4.8417
4.2982
4.2719
4.2456
3.8657
3.7741
3.7670
3.7462
3.7394
3.7279
3.7177
3.7102
3.7046
3.6975
3.6192
3.6069
3.5912
3.3332
3.3312
3.3143
3.3120
3.3100
3.3079
2.6045
2.5927
2.5707
2.5590
2.0858
2.0570
2.0239
2.0096
2.0016
1.8759
1.8465
1.8422
1.8128
1.7193
Current Data Parameters
NAME YYS-753-20240406-C-H
EXPNO 1
PROCNO 1
F2 - Acquisition Parameters
Date_ 20240407
Time 12.47
INSTRUM spect
PROBHD 5 mm DUL 13C-1
PULPROG zg30
TD 32768
SOLVENT MeOD
NS 15
DS 0
SWH 6410.256 Hz
FIDRES 0.195625 Hz
AQ 2.5559039 sec
RG 80.6
DW 78.000 usec
DE 6.00 usec
TE 300.0 K
D1 2.00000000 sec
TD0 1
======== CHANNEL f1 ========
NUC1 1H
P1 10.00 usec
PL1 -2.40 dB
SFO1 400.1528010 MHz
F2 - Processing parameters
SI 16384
SF 400.1500065 MHz
WDW EM
SSB 0
LB 0 Hz
GB 0
PC 1.00
HO
OH
OH
SAda
PhthN
O
CO2Me
HO
S85
8.0
7.5
7.0
6.5
6.0
5.5
5.0
4.5
4.0
3.5
3.0
2.5
2.0
1.5
1.0
0.5
0.0
ppm
2.11
1.99
0.99
1.21
1.01
3.05
2.02
1.01
1.26
1.00
9.53
1.16
6.25

YYS-753-20240406-C

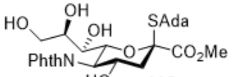

S85

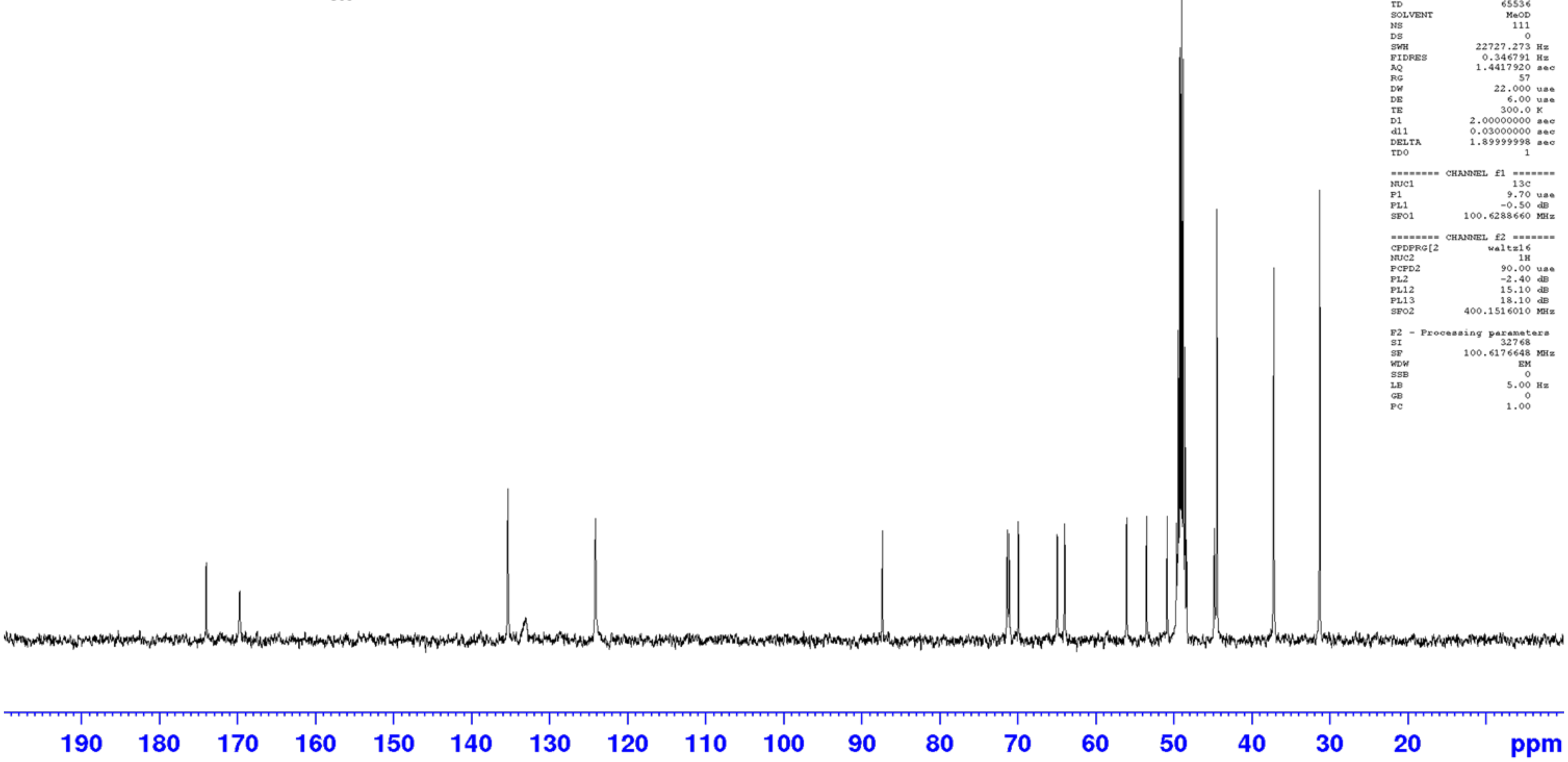

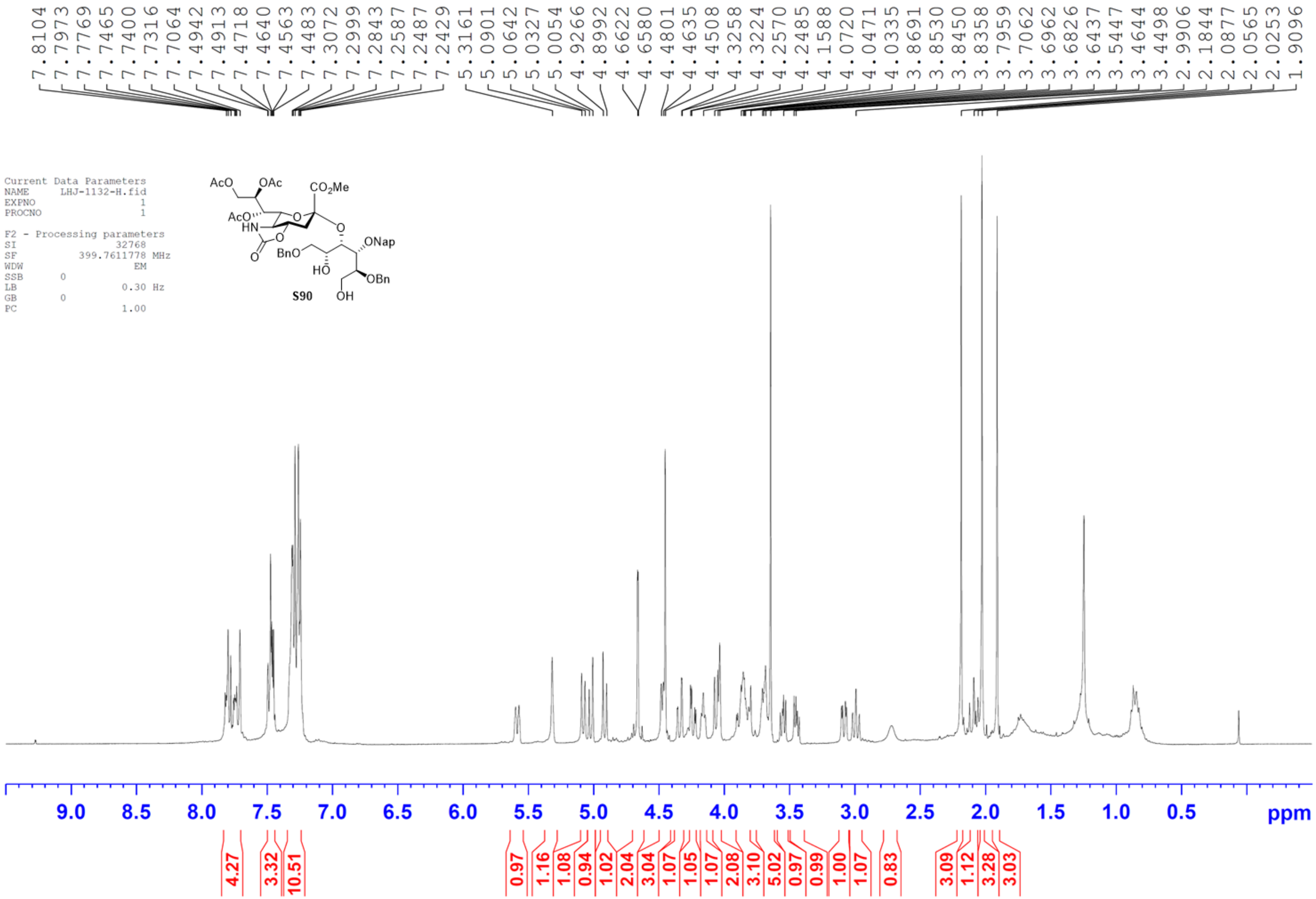
7.8104
7.7973
7.7769
7.7465
7.7400
7.7316
7.7064
7.4942
7.4913
7.4718
7.4640
7.4563
7.4483
7.3072
7.2999
7.2843
7.2587
7.2487
7.2429
5.3161
5.0901
5.0642
5.0327
5.0054
4.9266
4.8992
4.6622
4.6580
4.4801
4.4635
4.4508
4.3258
4.3224
4.2570
4.2485
4.1588
4.0720
4.0471
4.0335
3.8691
3.8530
3.8450
3.8358
3.7959
3.7062
3.6962
3.6826
3.6437
3.5447
3.4644
3.4498
2.9906
2.1844
2.0877
2.0565
2.0253
1.9096
Current Data Parameters
NAME LHJ-1132-H.fid
EXPNO 1
PROCNO 1
F2 - Processing parameters
SI 32768
SF 399.7611778 MHz
WDW EM
SSB 0
LB 0.30 Hz
GB 0
PC 1.00
AcO
OAc
CO2Me
AcO
HN
O
O
O
O
BnO
ONap
HO
OBn
OH
S90
9.0
8.5
8.0
7.5
7.0
6.5
6.0
5.5
5.0
4.5
4.0
3.5
3.0
2.5
2.0
1.5
1.0
0.5
ppm
4.27
3.32
10.51
0.97
1.16
1.08
0.94
1.02
2.04
3.04
1.07
1.05
1.07
2.08
3.10
5.02
0.97
0.99
1.00
1.07
0.83
3.09
1.12
3.28
3.03

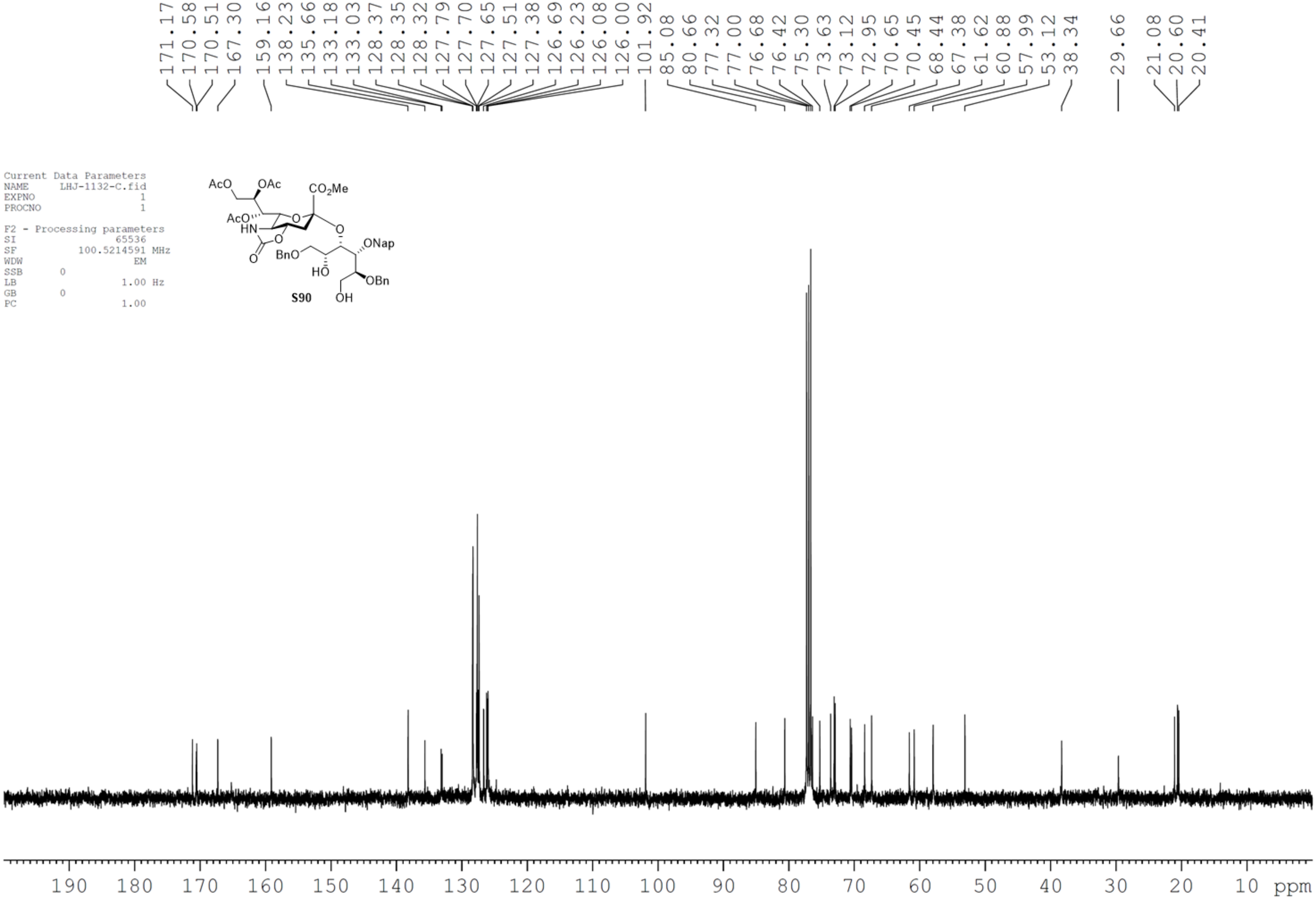
171.17
170.58
170.51
167.30
159.16
138.23
135.66
133.18
133.03
128.37
128.35
128.32
127.79
127.70
127.65
127.51
127.38
126.69
126.23
126.08
126.00
101.92
85.08
80.66
77.32
77.00
76.68
76.42
75.30
73.63
73.12
72.95
70.65
70.45
68.44
67.38
61.62
60.88
57.99
53.12
38.34
29.66
21.08
20.60
20.41
Current Data Parameters
NAME LHJ-1132-C.fid
EXPNO 1
PROCNO 1
F2 - Processing parameters
SI 65536
SF 100.5214591 MHz
WDW EM
SSB 0
LB 1.00 Hz
GB 0
PC 1.00
AcO
OAc
CO2Me
AcO
HN
O
O
O
O
BnO
HO
ONap
OBn
OH
S90
190 180 170 160 150 140 130 120 110 100 90 80 70 60 50 40 30 20 10 ppm
